# Gene expression profiling of oxidative stress response of *C. elegans *aging defective AMPK mutants using massively parallel transcriptome sequencing

**DOI:** 10.1186/1756-0500-4-34

**Published:** 2011-02-08

**Authors:** Heesun Shin, Hyojin Lee, Anthony P Fejes, David L Baillie, Hyeon-Sook Koo, Steven JM Jones

**Affiliations:** 1 Genome Sciences Centre, BC Cancer Agency, Suite 100 570 West 7th Avenue, Vancouver, British Columbia, Canada V5Z 4S6; 2 Department of Biochemistry, College of Life Science & Biotechnology, Yonsei University, Seoul 120-749, Republic of Korea; 3 Molecular Biology and Biochemistry, Simon Fraser University, 8888 University Drive, Burnaby, British Columbia, Canada V5A 1S6

## Abstract

**Background:**

A strong association between stress resistance and longevity in multicellular organisms has been established as many mutations that extend lifespan also show increased resistance to stress. AAK-2, the *C. elegans *homolog of an alpha subunit of AMP-activated protein kinase (AMPK) is an intracellular fuel sensor that regulates cellular energy homeostasis and functions in stress resistance and lifespan extension.

**Findings:**

Here, we investigated global transcriptional responses of *aak-2 *mutants to oxidative stress and in turn identified potential downstream targets of AAK-2 involved in stress resistance in *C. elegans*. We employed massively parallel Illumina sequencing technology and performed comprehensive comparative transcriptome analysis. Specifically, we compared the transcriptomes of *aak-2 *and wild type animals under normal conditions and conditions of induced oxidative stress. This research has presented a snapshot of genome-wide transcriptional activities that take place in *C. elegans *in response to oxidative stress both in the presence and absence of AAK-2.

**Conclusions:**

The analysis presented in this study has enabled us to identify potential genes involved in stress resistance that may be either directly or indirectly under the control of AAK-2. Furthermore, we have extended our current knowledge of general defense responses of *C. elegans *against oxidative stress supporting the function for AAK-2 in inhibition of biosynthetic processes, especially lipid synthesis, under oxidative stress and transcriptional regulation of genes involved in reproductive processes.

## Background

A growing body of research has revealed an association between stress resistance and longevity in multicellular organisms as many mutations that extend lifespan also increase stress resistance [1-3]. Understanding the cellular response to stress should therefore present significant insight into lifespan control.

The 5-AMP-activated protein kinase (AMPK), a ubiquitously expressed, multi-substrate serine/threonine protein kinase, is an intracellular fuel sensor. AMPK is activated when phosphorylated due to the depletion of ATP relative to AMP [4] that are resulted by various environmental or metabolic stresses caused by conditions such as heat shock, hypoxia, and low glucose [5], and AMPK mediates its effect by regulating the transcription of downstream genes by directly phosphorylating transcription factors and coactivators [6-8]. The main function of AMPK as a 'metabolic switch' is to maintain cellular energy homeostasis by up-regulating the pathways that produce ATP while down-regulating energy-consuming anabolic processes [4,5,9].

*C. elegans *AAK-1 and AAK-2 are the two homologs of the catalytic alpha subunit of mammalian AMPK. AAK-1 is identified to be required for negative regulation of germline proliferation during dauer development [10]; though no significant phenotype in relation to lifespan control has been reported. AAK-2 is however, known to mediate the long-term survival of dauer larvae. *aak-2 *dauer stage animals do not demonstrate the normal stress-resistant and long-lived phenotype in unfavorable conditions such as starvation or crowding [10-12]. AAK-2 is required for proper fat metabolism and osmoregulation for survival of dauer larvae [11] and is also involved in germ cell cycle arrest upon dauer entry [10].

Moreover, it has been shown that over-expressing *aak-2 *increases lifespan by 13% relative to wild type while disruption of *aak-2 *reduces the lifespan by 12% compared to wild type [13]. In addition to these findings we have previously demonstrated that in vivo induction of oxidative stress in AAK-2 deficient animals by treatment with paraquat (1,1'-dimethyl-4, 4'-bipyridinium dichloride) results in a significantly lower survival rate than wild type [14].

Oxidative stress induces a wide range of cellular responses by producing reactive oxygen species (ROS). Low doses of ROS are known to promote cell proliferation and extend lifespan and medium doses may cause senescence while high levels of oxidative stress result in cell death [15]. These outcomes are known to depend partly on intracellular stress signaling pathways that are activated in response to oxidative stress and as a consequence of direct damage to DNA, proteins, and lipids. These cellular injuries and signaling mechanisms directly or indirectly modulate transcription factor activities resulting in changes to gene expression profiles. Some of these signaling pathways are involved in survival, while others are linked with cell death [16,17].

Given that AMPK regulates transcriptional responses, the loss of transcriptional regulation of some gene targets of AAK-2 might contribute to the hypersensitivity to oxidative stress observed in *aak-2 *mutants in addition to indirect consequences of the absence of AAK-2 functions. Global searches for targets of the *aak-2 *pathway by transcriptome profiling should therefore provide new AMPK targets of which only a small number is known and insight into the regulatory functions of AAK-2 in stress resistance and lifespan control in *C. elegans*.

The advent of massively parallel next generation sequencing technologies has facilitated the production of high coverage sequence data enabling genome wide assays of transcriptional activities. The high-throughput sequencing, specifically Illumina sequencing technology, has been shown to be highly replicable and proven to be a superior method to study mRNA expression levels in its ability to identifying differentially expressed genes compared to existing array technologies [18]. Also, the strength of such method has been demonstrated in a recent study by Hillier et al. which used Illumina sequencing technology for *C. elegans *stage specific transcriptome analysis including stage specific differential gene expression analysis [19]. Additionally, the whole transcriptome sequencing allows detection of low-expressed genes and novel transcripts or alternative splice variants while the usage of the array technology is limited to identifying differential gene expression of previously identified genes [18]. Also, sequencing provides absolute abundance of mRNAs whereas the array technology measures relative abundance and there is potential for cross-hybridization.

Here, we have performed 'whole transcriptome shotgun sequencing (WTSS)' using Illumina sequencing technology [20], to identify *C. elegans *genes that were differentially expressed between *aak-2 *mutants and wild type, in the presence or absence of paraquat induced oxidative stress.

## Results

We undertook a global comparative analysis of transcriptomes from *aak-2 *and wild type animals under both normal conditions and conditions of oxidative stress, in order to identify differences between gene expression profiles. Specifically, we compared transcriptomes from stressed wild type and stressed *aak-2 *mutants and *aak-2 *mutants under normal conditions against an unstressed wild type (N2) transcriptome. We used gene expression levels in unstressed wild type transcriptome as the baseline/control and compared the gene expression levels in the other three transcriptomes against this baseline to identify genes with differential expression in each sample relative to wild type, and subsequently, compared the groups of differentially expressed genes relative to unstressed wild type between the transcriptomes (Unstressed wild type is referred simply as wild type from this point, and gene expression change measures are all relative to wild type).

In order to identify statistically significant changes in gene expression, we calculated Audic Claverie p-values [21] for each pair-wise comparison of the samples against wild type. Sequence read numbers were normalized for each gene, first by its length, given that the sequence reads showed a fairly even distribution, and subsequently by the total read numbers of each sample. We selected genes with p-values less than 0.01, and the minimum of ~1.5-fold difference (i.e. log_2 _(normalized *aak-2 *reads/normalized N2 reads) <-0.7 or >0.7).

The total number of sequence reads for each cDNA sequence library is indicated in Additional File 1. An average of 71% of the total sequence reads were mapped to the *C. elegans *transcriptome in WormBase (WS180). After we eliminated ambiguous sequence matches to multiple genes which included many pseudogenes, (data not shown), we were able to unambiguously assign approximately 65% of the total sequence reads from each sequence library to the *C. elegans *transcriptome. The number of sequence reads mapped per gene varies from one to over 100,000 reads. Approximately 85% of *C. elegans *transcripts were identified by the sequence reads in each library.

We then performed Q-PCR to confirm some of gene expression level changes measured by sequencing. We chose 20 genes that showed significant up or down-regulation in stressed *aak-2 *mutants compared to wild type but showed insignificant expression changes in stressed wild type animals relative to wild type. For this validation, we prepared new RNA samples which can serve as biological replicates as well. The Q-PCR result shows overall good agreement in gene expression profiles and changes with the WTSS data as shown in Additional File 2 which validates our approach.

### Oxidative stress response of wild type (N2) animals

**Figure 1:**
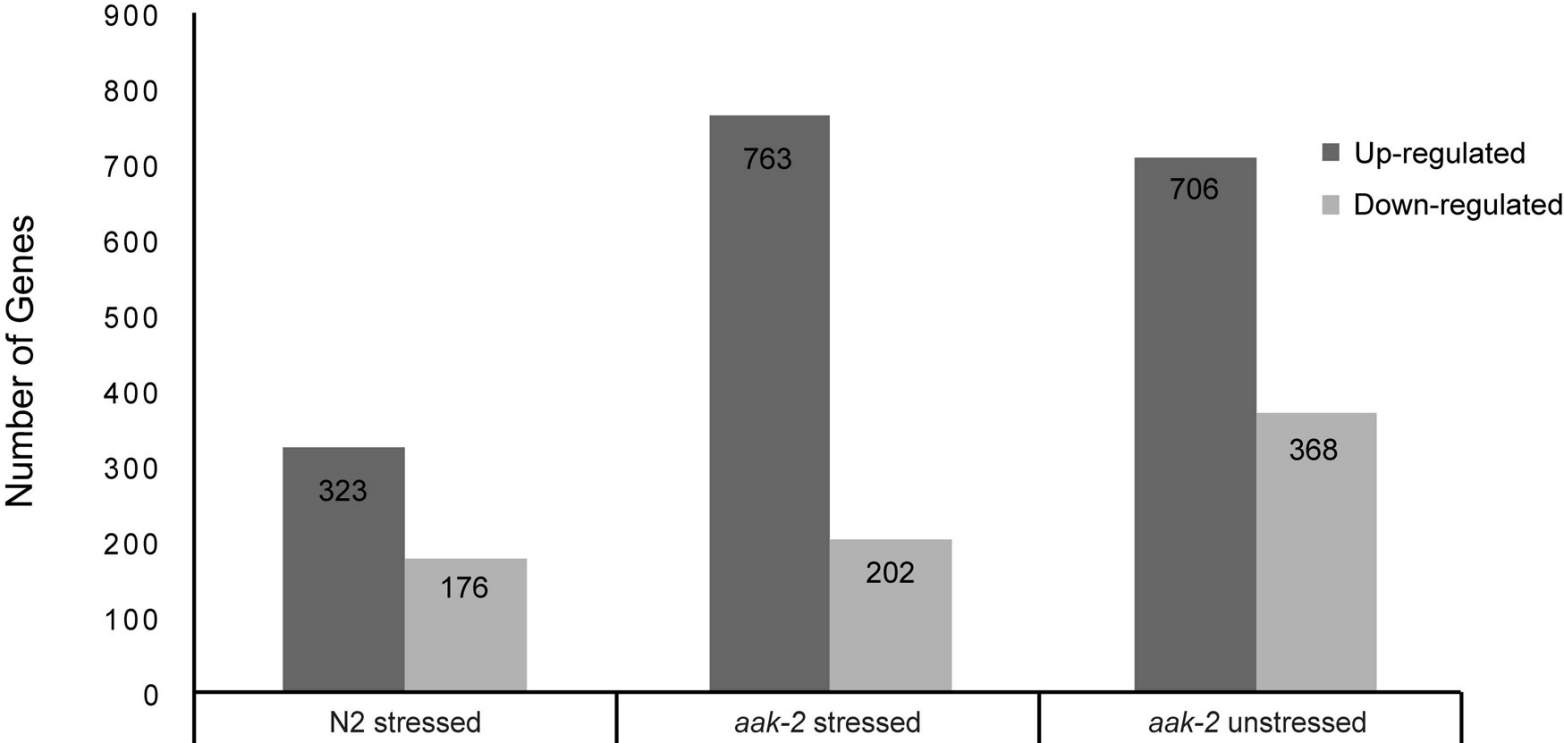
**Genes with statistically significant differential gene expression**. Number of genes significantly up or down-regulated in stressed wild type, stressed *aak-2 *mutants, and unstressed *aak-2 *mutants relative to unstressed wild type used as a control. There are considerably more genes up or down-regulated in stressed and unstressed *aak-2 *mutants than stressed wild type.

We first examined the transcriptional changes in wild type animals after exposure to paraquat induced oxidative stress. Using the statistical method discussed above, we identified 499 genes whose expression was significantly changed. Of the 499 differentially expressed genes, 323 were up-regulated and 176 down-regulated (Figure 1). In order to identify over represented functions associated with the differentially expressed genes, we used GOstat [22], a tool to categorize gene ontology annotations and generate the statistics of over represented terms.

#### Genes involved in stress response and aging are up-regulated in wild type animals under oxidative stress

As expected, stress response and aging related genes were up-regulated in stressed animals, including several heat shock protein genes (*hsp-16.1, hsp-16.11, hsp-16.2, hsp-16.41, hsp-16.48, hsp-16.49*), genes involved in oxidative phosphorylation and ATP synthesis (*atp-2*, H28O16.1, F58F12.1*, vha-1, vha-2, vha-4, vha-8, vha-11, vha-15 *), and genes involved in aging (*acdh-1, mca-3, ppn-1, cct-5*, T27F7.3, T27F7.1*, rab-1, cgh-1*, F59E10.3, T02H6.11, C30C11.4, *cpr-1, ifb-1, dod-6*). The most highly represented group of genes up-regulated in response to oxidative stress were however genes encoding collagens, approximately 50 of which were significantly up-regulated in these animals. Several hedgehog-like proteins (*grl-16, grl-4, grl-7, wrt-4, wrt-10*) were also up-regulated, as were the *daf-2 *and *daf-16 *regulated genes *dao-2, dao-4*, and *dao-5 *(Additional File 3). The most significantly over represented GO terms associated with up-regulated genes in wild type in response to oxidative stress are shown in Additional File 4: Supplementary Table S3a.

#### Biosynthetic and reproductive processes are down-regulated in wild type animals under oxidative stress

Genes that were significantly down-regulated in wild type animals under oxidative stress (Additional File 5) include histones (*his*) and ribosomal proteins (*rps, rpl*). Reduced histone and ribosomal protein gene transcription indicates that biosynthetic processes such as transcription and translation are down-regulated, as histone synthesis is dependent on ongoing DNA replication [23]. Other down-regulated genes involved in mRNA metabolic processes include *gut-2, lin-40, lsm-5*, *lsm-6, mxl-1, nhr-37*, and *rab-18*. Genes involved in reproduction were also down-regulated as were several genes known to function in the establishment of cellular localization (*aps-3, ddp-1, dyrb-1, elc-1, pfd-6*) and genes encoding prosaposins (*spp-3, spp-4, spp-5, spp-14, spp-17, spp-23*), lipid-binding proteins required for postembryonic development. The most significantly over represented GO terms (biological processes) associated with the down-regulated genes in wild type in response to oxidative stress are shown in Additional File 4: Supplementary Table S3b.

### Stress responses of *aak-2 *mutants in comparison to stress responses of wild type animals

We examined gene expression level changes in *aak-2 *mutants under conditions of oxidative stress relative to wild type. From this analysis we identified 763 genes that were significantly up-regulated and 202 genes that were significantly down-regulated relative to wild type (Figure 1, Additional File 6). We then compared these differentially expressed genes to transcriptional changes from wild type animals under stress, in order to identify similarities and differences in their responses to the oxidative stress.

**Figure 2:**
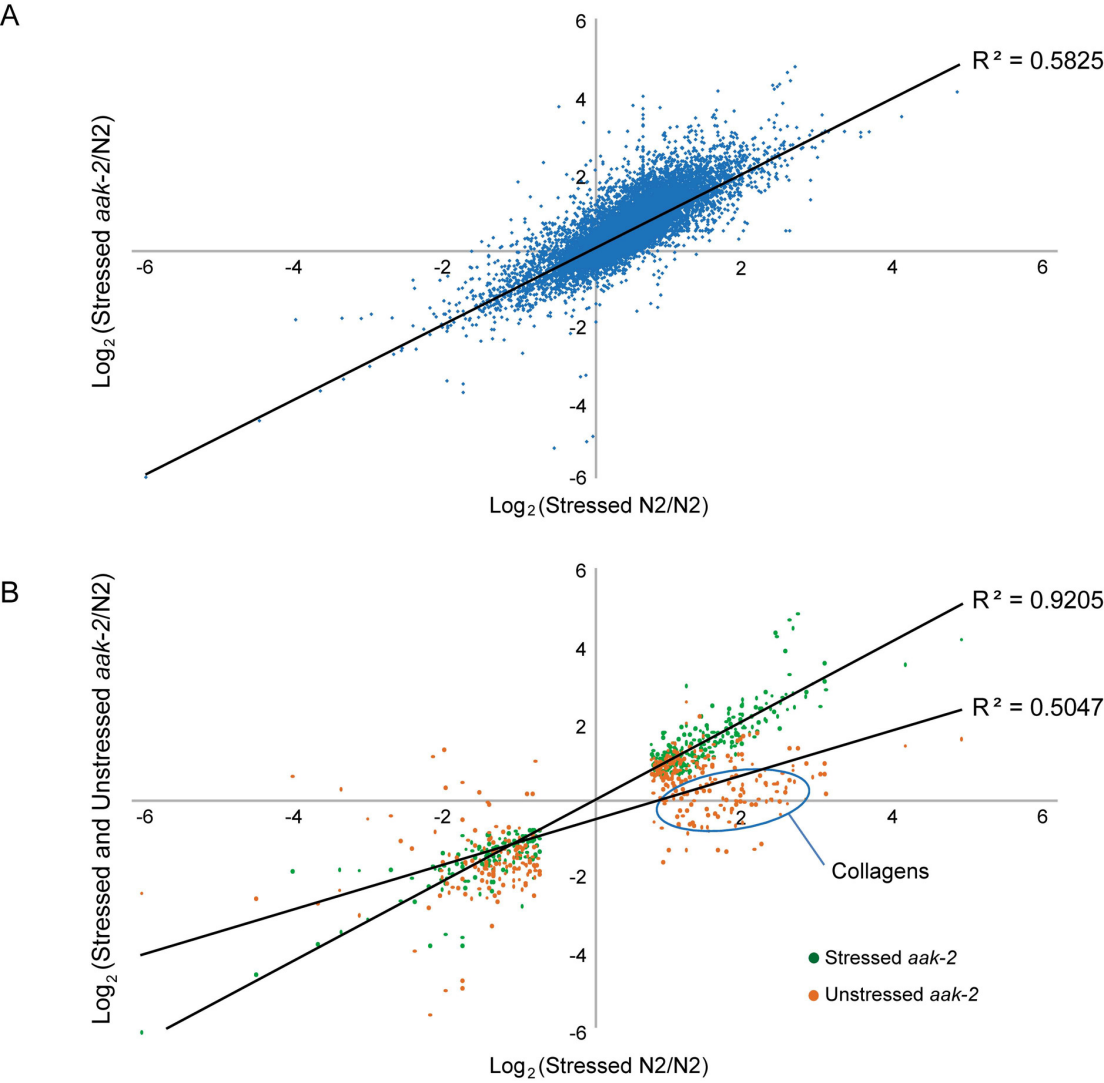
**Comparison of gene expression level changes against oxidative stress between wild type and *aak-2 *mutants**. **(A) **Correlation between gene expression level changes of all genes identified in stressed wild type and stressed *aak-2 *mutants relative to unstressed wild type. **(B) **Correlation between gene expression level changes of genes significantly up or down-regulated commonly in stressed wild type and stressed *aak-2 *mutants relative to unstressed wild type (green). The commonly up or down-regulated genes in stressed wild type and stressed *aak-2 *mutants show much higher correlation compared to all genes indicating these genes are not only commonly regulated in wild type and *aak-2 *mutants against oxidative stress, but also the degree of the expression level changes is overall quite similar. The commonly up or down-regulated genes in stressed wild type and stressed *aak-2 *mutants and their expression level changes in unstressed *aak-2 *mutants (orange). A large group of collagens are commonly up-regulated in stressed wild type and stressed *aak-2 *mutants, but insignificantly changed in unstressed *aak-2 *mutants as indicated.

**Figure 3:**
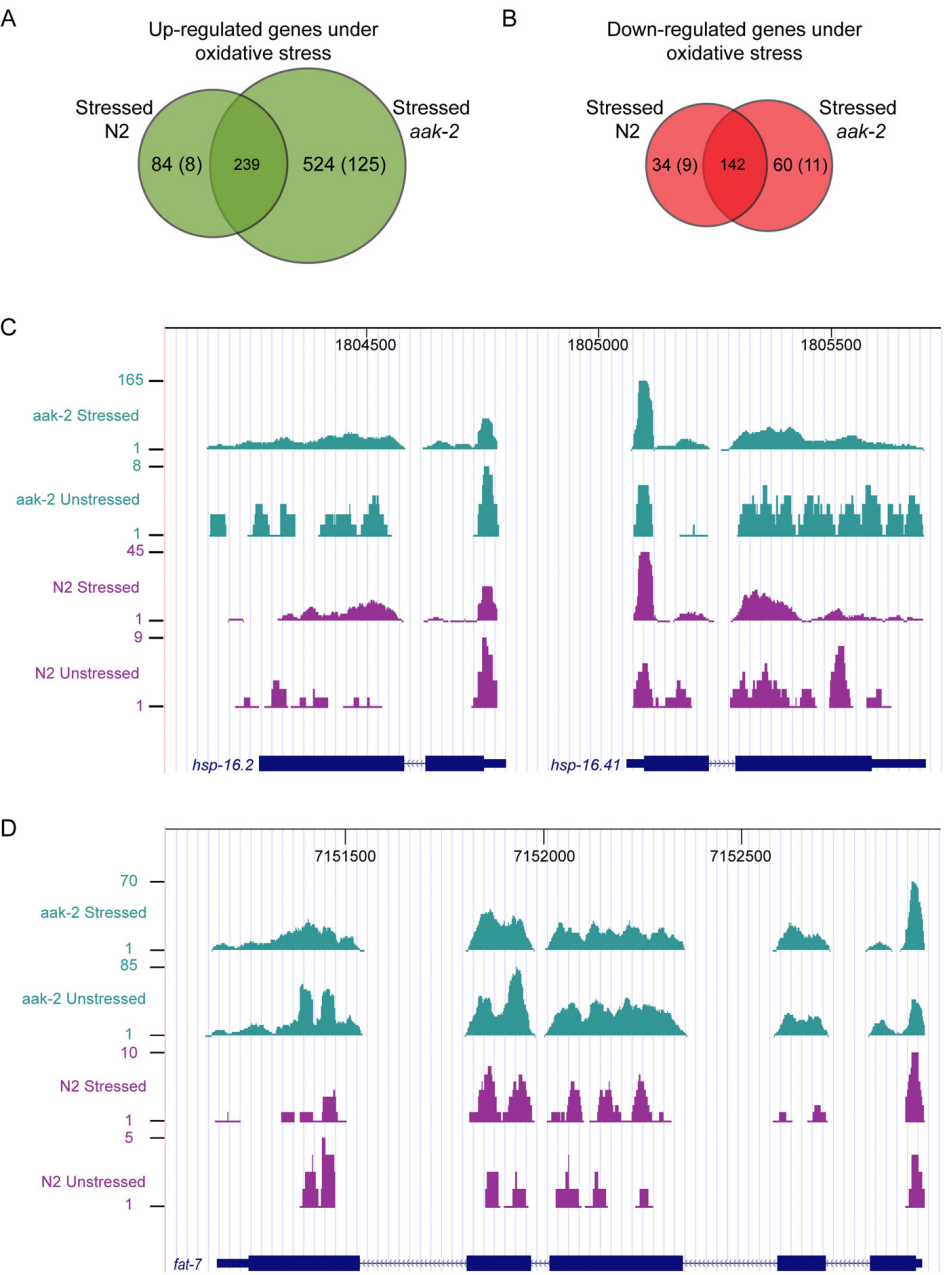
**Number of commonly or statistically differentially regulated genes in wild type and *aak-2 *mutants in response to oxidative stress and examples**. **(A) **Venn diagram showing numbers of commonly up or **(B) **down-regulated genes with statistically significant gene expression level changes in wild type and *aak-2 *mutants in response to oxidative stress relative to wild type (overlapping regions) as well as numbers of genes up or down-regulated exclusively in stressed wild type or stressed *aak-2 *mutants relative to wild type (non-overlapping regions). Numbers in brackets indicate genes that showed the most difference in gene expression relative to wild type between the two libraries, stressed wild type and stressed *aak-2 *(i.e. genes that showed significant changes in one library (i.e. Log_2 _ratio relative to wild type greater or less than |0.7|, p-val < 0.01), but showed negligible changes (i.e. Log2 ratio relative to wild type greater or less than ~|0.3|) or significantly changed in the opposite direction in the other library). For instance, there is a relatively large subset of genes in brackets (125) significantly up-regulated in *aak-2 *mutants in response to oxidative stress but either insignificantly regulated or significantly down-regulated in wild type under oxidative stress. **(C) **UCSC browser view of *hsp-16.2 *and *hsp-16.41 *with the sequence reads aligned to the gene models. Four samples (Stressed wild type (N2), Stressed *aak-2*, Unstressed wild type (N2), Unstressed *aak-2*) are shown. It is evident *hsp-16.2 *and *hsp-16.41 *are much more highly expressed in stressed wild type and stressed *aak-2 *mutants compared to unstressed wild type and unstressed *aak-2 *mutants, especially in stressed *aak-2 *mutants. Note: The scales for the minimum and maximum number of reads for the four samples are different. **(D) **UCSC browser view of *fat-7 *with the sequence reads aligned to the gene model. Four samples (Stressed wild type, Stressed *aak-2*, Unstressed wild type, Unstressed *aak-2*) are shown. As indicated by the number of sequence reads mapped, *fat-7*is much more highly expressed in *aak-2 *mutants (stressed and unstressed) compared to wild type (stressed and unstressed). Note: The scales for the minimum and maximum number of reads for the four samples are different.

The correlation between expression level changes of all genes in response to oxidative stress in wild type and *aak-2 *mutants is shown in Figure 2A (R^2 ^= 0.58). Figure 2B shows genes commonly up or down-regulated in both wild type and *aak-2 *mutants in response to oxidative stress relative to wild type (as indicated in green). This correlation is very high as indicated by the linear regression (R^2 ^= 0.92). 239 up-regulated genes and 142 down-regulated genes were common to both wild type and *aak-2 *mutants (Figure 3A, B), and mostly, the degree of expression level changes of these genes in response to oxidative stress were directly comparable between wild type and *aak-2 *mutants.

#### Commonly up or down-regulated genes in wild type and aak-2 mutants under oxidative stress - aak-2 mutants display a heightened stress response

The majority of commonly down-regulated genes in stressed wild type and *aak-2 *mutants are required for translation and transcription including ribosomal proteins and histones. Prosaposins are also highly down-regulated in both stressed wild type and *aak-2 *mutants (Additional File 7).

Most genes up-regulated in wild type animals in response to the oxidative stress were also up-regulated in stressed *aak-2 *mutants (Additional File 8). Some genes were notably more highly up-regulated in stressed *aak-2 *mutants, including heat shock proteins (*hsp-16.1, hsp-16.2, hsp-16.11, hsp-16.41, hsp-16.48*, and *hsp-16.49*) (Figure 3C), *acdh-1*, a short-chain acyl-CoA dehydrogenase involved in energy generation and lifespan extension [24], and *lgg-1*, a microtubule-associated anchor protein involved in autophagy and membrane trafficking [25]. Additionally *lys-4, lys-5, lys-6*, lysozymes known to function in innate immune system [26], were considerably more highly up-regulated in stressed *aak-2 *mutants. Finally, collagen genes that were significantly up-regulated in wild type animals were also up-regulated in *aak-2 *mutants in response to oxidative stress, showing slightly elevated overall expression in the stressed *aak-2 *population. From this result we conclude that stressed *aak-2 *mutants demonstrate a more heightened general stress response as transcription of genes involved in stress resistance are more highly up-regulated than in stressed wild type animals.

#### Differences in stress response between wild type and aak-2 mutants - AAK-2 functions in down-regulating biosynthetic processes in response to oxidative stress

As shown in Figure 3A, there is a subset of 125 genes that are specifically up-regulated in *aak-2 *mutants under oxidative stress, listed in Table 1. These are the most differentially expressed genes between stressed wild type and stressed *aak-2 *mutants as they show significant gene expression level changes in stressed *aak-2 *mutants relative wild type, but they are either negligible or significantly down-regulated in wild type animals under stress relative to wild type (Additional File 9 lists fold change comparisons for these genes in stressed wild type relative to wild type and stressed *aak-2 *mutants relative to wild type as well as the gene descriptions). Interestingly, examination of the functionally characterized genes in this group revealed an up-regulation of specific biosynthetic processes, particularly fatty acid synthesis (Table 2). Notable genes in this case comprise fatty acid desaturases (*fat*-), and several fatty acid binding proteins (*far-*) were also up-regulated in *aak-2 *mutants in response to oxidative stress relative to wild type. Although not statistically significant, it is interesting to note that some long chain fatty acid elongases (*elo-*), especially *elo-1*, were slightly up regulated in stressed *aak-2 *mutants relative to wild type. Additionally, genes involved in coenzyme and amino acid biosynthesis and genes involved in carbohydrate metabolic process are also up-regulated. These observations may imply that AAK-2 functions in down-regulating these energy consuming biosynthetic processes when animals are under oxidative stress (Additional File 10).

**Table 1:** 125 genes significantly up regulated exclusively in stressed *aak-2 *mutants sorted by the expression level changes in stressed *aak-2 *compared to wild type animals.

F22B7.9	Y38F1A.6	qdpr-1	R05F9.6	W10C8.5
Y38E10A.14	MTCE.33	F28A10.6	F32B5.1	lea-1
ZK970.7	scl-2	F54E2.1	tat-4	T15B7.2
F44A6.5	Y37A1B.5	ldh-1	ttr-36	C10G8.4
clec-52	R03D7.1	T01C8.2	Y43C5A.2	F08B12.4
C01B4.6	F44E7.2	let-721	F52A8.1	gdi-1
Y19D10A.16	nspc-17	ttr-47	mua-6	M02D8.1
C09H5.2	Y48G8AL.13	Y38E10A.13	nspc-10	pat-10
C17C3.1	F40F12.7	Y62E10A.13	C15C8.3	C31E10.7
pmp-5	T28F4.5	C42D4.1	T20G5.8	ZK637.2
clec-209	C45B2.2	fat-4	vha-14	F53A9.8
F56A4.2	F18E3.11	nspc-3	glt-1	sod-1
B0280.17	ccg-1	npa-1	pes-9	C16A3.10
F35E12.5	W05H9.1	F15B10.1	nspc-20	C06E7.1
C53A3.2	fat-5	M60.4	R107.5	rmd-2
nlp-29	K07C11.7	F13H8.7	nuo-1	dad-1
C33A12.19	C08F11.12	far-8	ncs-2	spp-10
K09H11.7	Y38C1AA.7	R11A5.4	baf-1	T25B9.9
F25E5.8	ech-6	C05C10.3	sqv-4	plp-1
F08G5.6	F10G2.1	mmaa-1	F52E1.14	rhr-1
F56A4.3	hsp-17	R05D11.5	F41C3.5	prdx-3
C01B4.9	F54D5.12	F54C9.3	F07H5.5	F56C9.7
Y19D10A.12	R02D3.1	far-6	gta-1	nspa-1
F55H12.2	hpd-1	Y51A2 D.14	C06A8.1	brp-1
C07E3.9	sel-9	lpl-1	tag-174	W03F11.1

Additional File 9 lists Log_2 _ratios of these genes in stressed wild type relative to wild type and stressed *aak-2 *mutants relative to wild type.

**Table 2:** Most highly represented biological processes the 125 genes listed in Table 1 are involved in as resulted from GO analysis

GO	Genes	Pvalue	GO as name
GO:0032787	fat-5; fat-4; r11a5.4; gta-1; c17c3.1; r03d7.1; fat-6	5.50E-06	monocarboxylic acid metabolic process;
GO:0019752	fat-4; r11a5.4; fat-6; y62e10a.13; lpl-1; fat-5; hpd-1; gta-1; c06a8.1; r03d7.1; c17c3.1	5.50E-06	carboxylic acid metabolic process;
GO:0006082	fat-4; r11a5.4; fat-6; y62e10a.13; lpl-1; fat-5; hpd-1; gta-1; c06a8.1; r03d7.1; c17c3.1	5.50E-06	organic acid metabolic process;
GO:0006631	fat-5; fat-4; gta-1; c17c3.1; fat-6	0.000325	fatty acid metabolic process;
GO:0044255	spp-10; fat-4; c07e3.9; fat-6; fat-5; let-721; gta-1; c17c3.1	0.00139	cellular lipid metabolic process;
GO:0006555	c06a8.1; r03d7.1	0.00139	methionine metabolic process;
GO:0042759	fat-5; fat-6	0.00139	long-chain fatty acid biosynthetic process;
GO:0006633	fat-5; fat-4; fat-6	0.00211	fatty acid biosynthetic process;
GO:0006629	spp-10; fat-4; c07e3.9; fat-6; fat-5; let-721; gta-1; c17c3.1	0.0123	lipid metabolic process;
GO:0009066	c06a8.1; r03d7.1	0.0123	aspartate family amino acid metabolic process;
GO:0006732	lpl-1; vha-14; t25b9.9; c17c3.1; r03d7.1	0.0123	coenzyme metabolic process;
GO:0000096	c06a8.1; r03d7.1	0.0152	sulfur amino acid metabolic process;
GO:0008610	fat-5; let-721; fat-4; fat-6	0.0236	lipid biosynthetic process;
GO:0006006	ldh-1; r11a5.4; t25b9.9	0.0248	glucose metabolic process;
GO:0006790	c06a8.1; r03d7.1	0.0422	sulfur metabolic process;
GO:0051186	lpl-1; vha-14; t25b9.9; c17c3.1; r03d7.1	0.0422	cofactor metabolic process;
GO:0006519	y62e10a.13; hpd-1; gta-1; c06a8.1; r03d7.1	0.0422	amino acid and derivative metabolic process;
GO:0005996	ldh-1; r11a5.4; t25b9.9	0.0474	monosaccharide metabolic process;
GO:0044249	fat-4; vha-14; r11a5.4; fat-6; y62e10a.13; lpl-1; fat-5; let-721; r03d7.1	0.0474	cellular biosynthetic process;

Eight genes significantly up-regulated in stressed wild type animals relative to wild type but insignificantly changed or significantly down-regulated in stressed *aak-2 *mutants relative to wild type are *tsn-1*, C17H12.8, C30G12.2, K07C5.4, K10C2.3, R09E12.3, Y57G11C.9, and Y113G7B.17. As shown in Figure 3B, only nine genes were significantly down-regulated exclusively in wild type animals in response to oxidative stress relative to wild type(*col-95*, C45B2.1, F53A9.1, F53A9.8, *lbp-6*, *MTCE.15*, *nspa-5, tin-9.1*, and Y60A3A.21) while they were either insignificantly changed or significantly up-regulated in stressed *aak-2 *mutants relative to wild type. A further 11 genes specifically down-regulated in stressed *aak-2 *mutants relative to wild type (*cct-8*, *rpl-25.2, rps-27*, F15E11.1, F15E11.14, F15E11.15, F45D11.14, F45D11.15, F45D11.16, K10C2.3, and Y48G8AL.12) were also noted.

These exclusively regulated genes are the most differentially regulated genes between stressed wild type and stressed *aak-2 *mutants, and therefore, these genes may be potential downstream targets that are potentially under either direct or indirect control of AAK-2. Many of these genes have no known functions and remain to be characterized. Further investigation of these genes may uncover novel relationships and functions for *aak-2 *and reveal specific targets of *aak-2 *that act in the biological processes that AAK-2 is known to be involved in.

### Differentially regulated genes in unstressed *aak-2 *mutants compared to wild type animals

A total of 1074 genes showed statistically significant differential gene expression in unstressed *aak-2 *mutants when compared to wild type. Among these genes, 706 genes were up-regulated and 368 genes were down-regulated (Figure 1).

#### Genes involved in stress response, ageing and the germline are up-regulated in unstressed aak-2 mutants relative to wild type

Additional File 11 lists genes that are significantly up-regulated in unstressed *aak-2 *mutants compared to wild type animals and Additional File 12 shows most highly represented biological processes these genes are involved in as analyzed by GOstat. Genes included are those known to be involved in aging and stress responses (*acdh-1, cgh-1, dao-5, daf-16, daf-18, fat-7, lin-41, mup-4, mdt-15*), heat shock proteins (*hsp-16.1, hsp-16.11, hsp-6, hsp25*), and vacuolar proton-translocating ATPases (*vha-4, vha-5, vha-8, vha-11, vha-13, vha-14, vha-15, vha-16*).

Another interesting group of genes that are highly up-regulated in unstressed *aak-2 *mutants relative to wild type are the vitellogenin genes (*vit-1 *to *vit-6*), all of which were statistically highly significant differentially expressed genes observed between wild type and unstressed *aak-2 *mutants (Average p-val = 4.05E-42). Finally, a large group of genes involved in reproduction was up-regulated in unstressed *aak-2 *mutants compared to wild type. These genes are involved in gamete generation, oviposition, and regulation of meiosis, oogenesis, sex differentiation, embryonic cleavage, and cell division.

#### Biosynthetic processes and energy generation are down-regulated in unstressed aak-2 mutants relative to wild type

Many ribosomal protein coding genes and histone genes were down-regulated in unstressed *aak-2 *mutants, indicating a general down regulation of biosynthetic processes. Genes involved in translational elongation (*rla-2*, *rla-1*, c37a2.7, zk512.4) and mRNA metabolic processes (*snr-7, snr-6, lsm-3, lsm-8, gut-2, snr-3, lsm-6, lsm-5, snr-5*, and y48g1c.9) were down-regulated. Additionally a large number of major sperm proteins (MSPs) are significantly down-regulated. Other interesting genes down-regulated include prosaposins (*spp-3, spp-4, spp-5, spp-14, spp-17, spp-23*), and *tin-13, tin-9.1*, and *ddp-1*which are involved in inner mitochondrial membrane organization and biogenesis and protein import into mitochondrial inner membrane (Additional File 13).

Genes involved in oxidative phosphorylation and generation of energy were also down-regulated in unstressed *aak-2 *mutants relative to wild type (*asg-1*, *atp-4*, *mtce.4*, *vha-3*, *cyc-2.1, tag-174*, F26E4.6, Y82E9BR.3, C33A12.1, T27E9.2, F45H10.2, B0035.18, R07E4.3, F29C4.2, Y71H2AM.5, F45H10.3, R04F11.2, and D2030.4) (Additional File 14). Finally, the most highly down-regulated genes in unstressed *aak-2 *mutants are F15E11.1, and the paralogs F15E11.12, F15E11.13, F15E11.15, and Y19D10B.7 which belong to a nematode-specific family. Transcription of these genes was almost completely suppressed showing a twelve to fourteen fold reduction of abundance in the unstressed *aak-2 *mutants compared to wild type.

### Comparison of unstressed *aak-2 *mutants with stressed *aak-2 *mutants

Finally, we compared significantly differentially expressed genes in unstressed *aak-2 *and stressed *aak-2 *mutants compared to wild type animals in order to identify *aak-2 *specific gene expression changes in the mutants before and after they were exposed to paraquat. Since most transcriptional changes in stressed *aak-2 *mutants were also observed in stressed wild type animals, we also included gene expression changes in stressed wild type animals in the comparison.

#### Commonly differentially regulated genes in unstressed aak-2 and stressed aak-2: unstressed aak-2 mutants display a stress response

**Figure 4:**
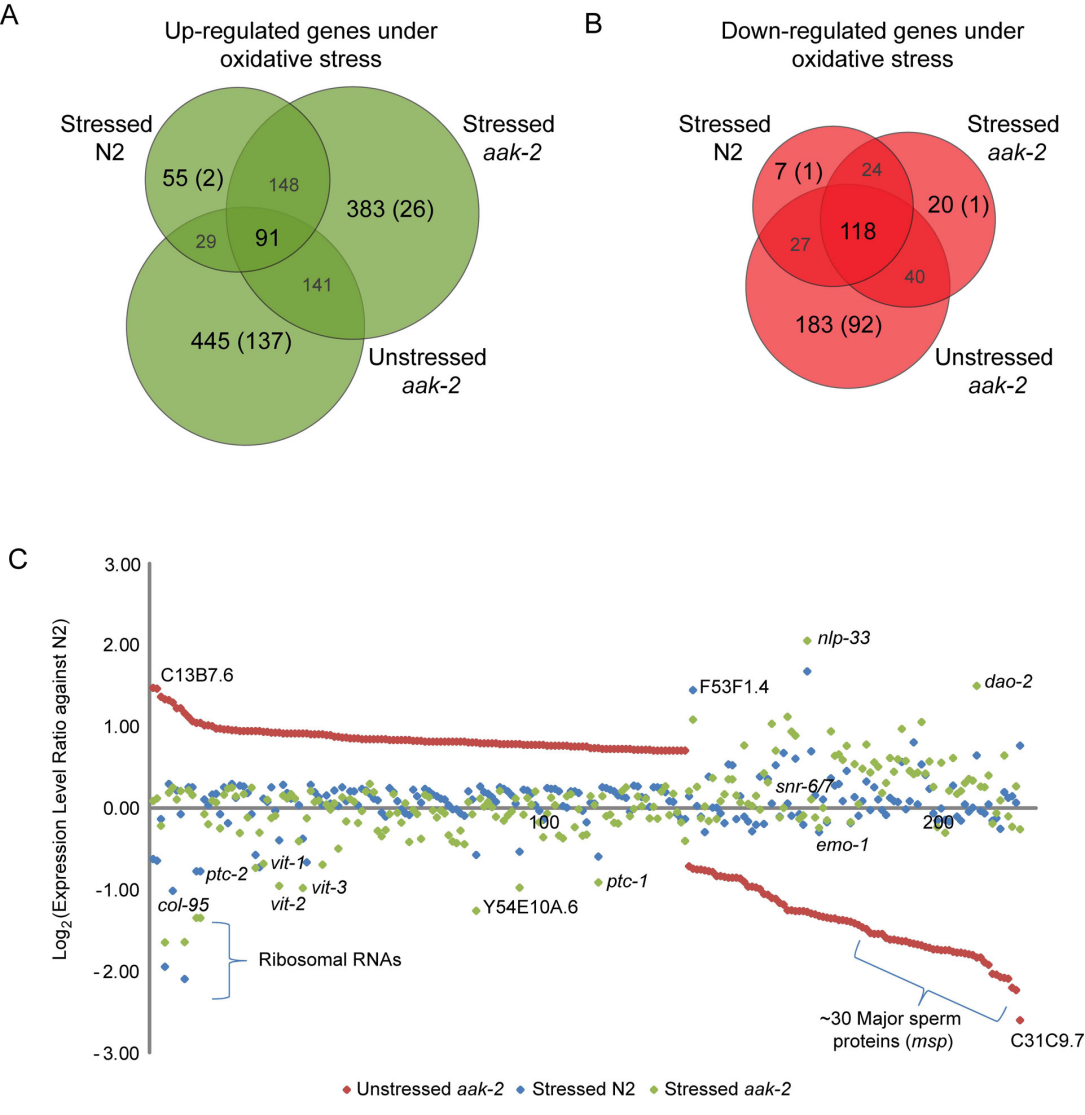
**Commonly differentially regulated genes in unstressed *aak-2 *and stressed *aak-2 *and transcriptional changes specific to unstressed *aak-2 *mutants**. **(A) **Venn diagram showing numbers of commonly up or **(B) **down-regulated genes with statistically significant gene expression level changes in stressed wild type, stressed *aak-2 *mutants, and unstressed *aak-2 *mutants (overlapping regions) as well as numbers of genes up or down-regulated exclusively in one library (non-overlapping regions). Notably, there is a relatively large subset of genes up (137) or down-regulated (92) significantly in unstressed *aak-2 *mutants, but either insignificantly regulated or significantly regulated in opposite direction in stressed wild type and stressed *aak-2 *mutants. **(C) **Genes significantly differentially expressed in unstressed *aak-2 *mutants relative to wild type, but insignificantly regulated or significantly changed in the opposite direction in stressed wild type or stressed *aak-2 *mutants. The genes are sorted by the expression level changes (log_2 _ratio) in unstressed *aak-2 *mutants, and some interesting genes are labelled such as vitellogenins (*vit*) and major sperm proteins (*msp*). For instance, the *msp *genes are highly down-regulated in unstressed *aak-2 *mutants as indicated by the red dots, but slightly up regulated on average in stressed wild type and stressed *aak-2 *mutants as shown by the blue and green dots respectively.

As shown in Figure 4A, there are 91 genes significantly up-regulated in unstressed *aak-2 *mutants relative to wild type that are in common with stressed *aak-2 *mutants and also with stressed wild type relative to wild type. These include genes involved in defense response, oxidative phosphorylation, and aging (Additional File 15). Also, there are 118 genes significantly down-regulated in unstressed *aak-2 *mutants that are also significantly down-regulated in stressed *aak-2 *and stressed wild type animals relative to wild type (Figure 4B, Additional File 16). These genes include a number of ribosomal proteins, histones, and prosaposins. This result may indicate that unstressed *aak-2 *mutants are responding to some background level of intrinsic oxidative stress, including metabolism and reproduction, presumably as a result of the absence of AAK-2.

#### Transcriptional changes specific to unstressed aak-2 mutants: unstressed aak-2 mutants show transcriptional changes in reproduction

There are however genes which show no significant change in expression, or are significantly down-regulated in unstressed *aak-2 *mutants relative to wild type that are commonly up-regulated in both stressed *aak-2 *and stressed wild type animals relative to wild type (as indicated in orange in Figure 2B). This group of genes includes collagens, genes involved in aging (*dod-6, dao-2, dao-4*, F59E10.3, T02H6.11, T27F7.3), defense response (*hsp-16.2, hsp-16.41*), and protein localization (*arf-3, dlc-1, trap-3, tag-170*, C28H8.4, F59E10.3, W10D9.5) (Additional File 17), suggesting that the stress response of unstressed *aak-2 *mutants is not as strong as that of paraquat treated animals either due to the different degree of severity of oxidative stress level or the absence of AAK-2 activities in stress resistance. Stressed *aak-2 *mutants did however show even more heightened stress response than stressed wild type animals as discussed earlier, potentially indicating the activity of alternative stress response pathways in these animals which compensated for the lack of AAK-2 functions in a more severe stress environment.

Conversely, there are 137 genes up-regulated and 92 genes down-regulated only in unstressed *aak-2 *mutants relative to wild type (Figure 4C). A large fraction of these genes are involved in reproductive processes such as gamete generation, sex differentiation, oocyte development, regulation of germline mitosis and meiosis, vulval development, and fertilization as are all vitellogenin genes (*vit-1*to *vit-6*), which are known to be involved in fat transport and storage in the germline [27] (Table 3). In addition, peculiarly, genes involved in stress responses such as the forkhead transcription factor, *daf-16*, and a lipid phosphatase, *daf-18*, are more highly up-regulated in unstressed *aak-2 *mutants relative to wild type than in stressed wild type or stressed *aak-2 *mutants relative to wild type. DAF-16 and DAF-18 are known to regulate a wide variety of genes involved in longevity and stress responses.

**Table 3:** Genes significantly up regulated exclusively in unstressed *aak-2 *mutants relative to wild type and the GO terms (biological process) associated with them

Function	Genes	Pvalue	GO as name
Reproduction	mtm-3, ptc-1, spd-5, puf-8, t21b10.3, egl-27, top-1, c23g10.8, npp-7, dab-1, prp-8, gld-3, smk-1, sur-6, mbk-2, atx-2, ptc-2, nmy-2, ruvb-1, pbrm-1, mep-1, zk858.1, mom-5, egl-45, c08b11.3, lin-35, scc-3, daz-1, f37c12.7, y71h2am.20, eif-3.b, csr-1, t09e8.1	2.14E-10	reproductive process,
	
	daz-1, f37c12.7, puf-8, ptp-2, top-1, gld-3, y71h2am.20, smk-1, mbk-2, eif-3.b, nmy-2, csr-1, ruvb-1, tag-319, mep-1, zk858.1, t09e8.1	4.14E-04	gamete generation,
	
	atx-2, ptc-2, mtm-3, ptc-1, egl-27, mom-5, egl-45, dab-1, lin-35, sur-6, smk-1	8.17E-04	oviposition,
	atx-2, puf-8, gld-3	8.17E-04	feminization of hermaphroditic germ-line,
	
	mep-1, ptp-2, let-92, lin-45, lin-35, sur-6	1.22E-02	vulval development,
	
	puf-8, gld-3, scc-3	2.78E-02	regulation of meiosis,
	
	egl-27, mom-5, lin-45, mbk-2	5.22E-02	cell fate commitment,
	
	top-1, f17c11.10, smk-1, scc-3	6.15E-02	chromosome segregation,
	
	
	npp-7, mbk-2, spd-5	8.21E-02	fertilization,
	
	ptp-2, top-1, smk-1	9.86E-02	oogenesis,
	
	top-1, smk-1	9.86E-02	oocyte development,
	
	vit-1, vit-2, vit-3, vit-4, vit-5, vit-6	7.68E-03	lipid transport,

Cell cycle	let-92, nmy-2, mat-1, spd-5, egl-27, mom-5, lig-1, mbk-2, sur-6	2.69E-03	cell division,
	
	atx-2, puf-8, mom-5, rpn-1, gld-3, scc-3, mbk-2	2.78E-02	cell cycle process,
	
	mom-5, let-92, lig-1, sur-6, mat-1, mbk-2, spd-5	1.53E-02	embryonic cleavage, cytokinesis,

Anatomical structure development	let-92, t21b10.3, ptp-2, unc-76, prp-8, c23g10.8, top-1, sur-6, smk-1, nmy-2, lin-45, ruvb-1, pbrm-1, zk858.1, mep-1, c08b11.3, lin-35, scc-3	2.99E-04	organ development,
	
	mtm-3, f37c12.7, t21b10.3, ptp-2, egl-27, ani-2, c23g10.8, top-1, smk-1, sur-6, lin-45, let-711, npl-4.1, zk858.1, mom-5, npl-4.2, c08b11.3, lin-35, scc-3, let-92, daf-18, unc-76, prp-8, pap-1, eif-3.b, mbk-2, nmy-2, ruvb-1, paa-1, pbrm-1, mep-1, ran-5	6.96E-10	anatomical structure development,
	
	nmy-2, let-711, paa-1, npl-4.1, f37c12.7, t21b10.3, egl-27, npl-4.2, daf-18, top-1, lin-35, sur-6, mbk-2	1.18E-05	morphogenesis of an epithelium,
	
	t14g10.5, nmy-2, pqn-51, spd-5, ran-5, mom-5, ptp-2, unc-76, daf-18, npp-7, dab-1, scc-3, eif-3.b, sur-6, mbk-2	7.10E-02	cellular component organization and biogenesis,

Response to stimulus	mtm-3, ptc-1, f37c12.7, t21b10.3, egl-27, ptp-2, dab-1, top-1, pap-1, smk-1, sur-6, eif-3.b, t12a2.2, t14g10.5, atx-2, ptc-2, let-711, pbrm-1, npl-4.1, arx-2, ran-5, mom-5, egl-45, npl-4.2, lin-35, scc-3	9.47E-05	locomotory behavior,
	
	daf-16, mtm-3, ptc-1, cdc-14, f37c12.7, t21b10.3, ptp-2, egl-27, daf-18, top-1, dab-1, lig-1, pap-1, smk-1, sur-6, eif-3.b, t12a2.2, col-179, atx-2, t14g10.5, ptc-2, let-711, pbrm-1, npl-4.1, arx-2, ran-5, mom-5, npl-4.2, egl-45, lin-35, scc-3	2.55E-05	response to stimulus,
	
	rpb-2, daf-16, let-711, daf-18, smk-1, eif-3.b	5.22E-02	determination of adult life span,

A large number of major sperm protein genes (~30) were highly down-regulated in unstressed *aak-2 *mutants relative to wild type, but were insignificantly altered in stressed wild type and stressed *aak-2 *mutants relative to wild type. Other interesting genes down-regulated only in unstressed *aak-2 *mutants include *dao-2 *and *emo-1*. DAO-2 is a secreted protein that is down-regulated in *daf-2 *mutant animals, and thus may be involved in dauer formation [28]. EMO-1 is an ortholog of *S. cerevisiae *Sec61p gamma subunit, which is required for translocation of secreted and membrane proteins into the endoplasmic reticulum. EMO-1 is required for oogenesis and ovulation [29].

Finally, lysozymes *lys-1 *and *lys-2*, small nuclear ribonucleoproteins *snr-3, snr-6, snr-7 *and F45H10.2, Y71H2AM.5, T27E9.2, D2030.4, genes involved in water homeostasis/osmoregulation and generation of precursor metabolites and energy are significantly down-regulated only in unstressed *aak-2 *mutants relative to wild type, but insignificantly changed in stressed wild type and stressed *aak-2 *mutants relative to wild type (Additional File 18).

## Discussion

In this study we have used deep sequencing to construct a global picture of transcriptional changes in response to perturbation of *aak-2 *in the presence or absence of paraquat induced oxidative stress. While this approach provides an indirect method for assessing specific gene functions and relationships, it has given insight into global stress responses as based on previously characterized functions of AAK-2. Many of the potential targets of AAK-2 identified in this study remain to be characterized at a developmental level. Further investigation of these genes is expected to increase our understanding of the complex biological network governing stress resistance and energy homeostasis in *C. elegans*.

### Collagens may be involved in stress resistance and longevity

When we grouped significantly up-regulated genes in both wild type and *aak-2 *mutants in response to oxidative stress by similar function we found significant enrichment for cuticle collagen genes. This may indicate that collagens may be involved in the necessary adaptive mechanism for survival against oxidative stress in *C. elegans*. Cuticle collagens are the major component of the cuticle, an exoskeleton that surrounds the body of *C. elegans *functioning as a barrier between the animal and its environment. The cuticle is essential for maintenance of body morphology and integrity, and has a critical role in locomotion via attachments to body-wall muscles [30-32]. Several cuticles are synthesized throughout development of *C. elegans *beginning at the end of embryogenesis prior to hatching and then prior to molting at the end of each larval stage [33]. As all samples were derived from developmentally synchronized populations of adult animals, the significant up-regulation of the cuticle collagens is not likely to be due to developmental synthesis of the cuticle, but possibly due to the response to oxidative stress. We have noted that these collagens were highly up-regulated in both stressed *aak-2 *and in stressed wild type animals, but insignificantly changed in unstressed *aak-2 *mutants. Considering unstressed *aak-2 *mutants also demonstrated some degree of stress response, this difference might be due to the degree of oxidative stress to which the worms were exposed.

The genes encoding cuticle collagens were also found to be differentially expressed in response to bacterial species [34,35]. Increased expression of *C. elegans *cuticle collagens was a common response to multiple pathogens (*S. marcescens, E. faecalis, E. carotovora*, and *P. luminescens*) [35]. In addition, it has been shown that collagen genes are over expressed under oxidative stress in rat hepatic stellate cells [36,37]. In aging research, a recent study identified a large number of collagens as age regulated genes [38,39], and it has been shown that collagen gene expression decreases with age in long-lived *C. elegans daf-2 *mutants [39]. Overall these data suggest that cuticle collagens may be differentially regulated indirectly in response to oxidative stress or possibly involved in defense against environmental perturbations and potentially in longevity.

### Comparison to previously identified genes involved in aging

We also examined previously identified age-regulated genes [38]. Budovskaya *et al. *compared transcriptomes from young and old adult *C. elegans *using DNA microarrays and identified 1254 age-regulated genes. Of those, we found 1029 genes in our data and identified 80 genes significantly up or down-regulated in stressed wild type animals. The majority of these genes were also significantly up or down-regulated in stressed *aak-2 *mutants. We observed the trend that most genes which were up-regulated in wild type in response to oxidative stress (average log_2 _ratio = ~1.3) were also up-regulated in *aak-2 *mutants (average log_2 _ratio = ~1.5), but in unstressed *aak-2 *mutants, these genes were only slightly up-regulated (average log_2 _ratio = ~0.3) which include many collagens. Interestingly, down-regulated genes under oxidative stress showed a similar degree of expression level changes in all three groups (average log_2 _ratio = ~-1.4). Furthermore, we identified 22 genes significantly up-regulated and 3 genes down-regulated in stressed *aak-2 *mutants only (Table 4). These genes are included in the 125 and 11 genes identified to be up or down regulated, respectively, in stressed *aak-2 *mutants only and we speculate that these age-regulated genes may be specifically under the control of AAK-2.

**Table 4:** Age-regulated genes (Budovskaya et al. 2008) which are differentially regulated in stressed *aak-2 *only

Gene	Log_2 _(Stressed *aak-2*/N2)	Audic & Claverie p-val
clec-52	3.12	9.77E-04
F35E12.5	2.25	5.13E-27
nlp-29	2.15	8.68E-07
F08G5.6	2.00	5.72E-07
Y19D10A.12	1.83	5.35E-08
scl-2	1.67	6.91E-03
F44E7.2	1.61	5.35E-05
T28F4.5	1.41	8.19E-07
ech-6	1.32	4.32E-11
K07C11.7	1.30	8.85E-03
ttr-47	1.18	1.98E-04
C42D4.1	1.14	1.68E-05
far-6	1.03	1.12E-03
R11A5.4	1.03	4.27E-07
T20G5.8	0.94	1.08E-03
glt-1	0.94	6.49E-03
gta-1	0.89	2.48E-04
T15B7.2	0.85	8.84E-04
F08B12.4	0.82	4.75E-03
W10C8.5	0.77	4.14E-03
spp-10	0.76	1.84E-04
F56C9.7	0.73	4.28E-03
F45D11.16	-3.24	2.04E-03
F15E11.1	-4.84	2.84E-173
F15E11.15	-6.96	3.24E-40

### Comparison to previously identified DAF-16 targets

The forkhead transcription factor DAF-16 regulates a wide variety of genes involved in longevity and stress responses. DAF-16 is negatively regulated by the insulin-like growth factor receptor DAF-2 [40,41], and DAF-16 mediates longevity Induced by dietary restriction in *C. elegans *[42]. Recent studies suggest that genes regulated by DAF-16 are involved in cellular stress response, metabolism, and energy generation. It has also been shown that AAK-2 activates DAF-16 dependent gene expression. Additionally, AAK-2 directly phosphorylates DAF-16 [42]. AAK-2 therefore is likely to contribute to DAF-16 function in stress response.

We observed a slight increase (log_2 _ratio = ~0.3) in transcription of *daf-16 *in our stressed wild type data. We examined previously identified DAF-16 targets [24] and found many of these genes differentially expressed in stressed wild type animals. We then compared the known DAF-16 targets [24,39,43] with genes that are significantly changed in response to oxidative stress in this study and observed increases in transcriptional levels of several previously identified positive targets of DAF-16 including *col-157, dao-4, dao-6*, *dod-6*, *hsp-16.2*, *hsp-16.49*, *hsp-70*, *odc-1*, and *sod-2*, and reduced transcription of genes known to be inhibited by DAF-16 including *fip-5*, and *mtl-2 *[24,43] in stressed wild type as well as stressed *aak-2 *mutants. These DAF-16 target genes were however, not as significantly changed in unstressed *aak-2 *mutants even though expression of *daf-16 *itself was most highly up-regulated in unstressed *aak-2 *mutants (log_2 _ratio = ~1).

This result is not conclusive as the expression level changes of the known DAF-16 targets were not highly statistically significant in our data, but we speculate that it might suggest that AAK-2 works together with DAF-16 and that in the absence of AAK-2 (as in unstressed *aak-2 *mutants), DAF-16 targets were not regulated as efficiently as in wild type. For example, the vitellogenin genes which have been shown to be up-regulated in the absence of DAF-16 [24], were significantly up-regulated only in unstressed *aak-2 *mutants.

The fact that DAF-16 downstream targets in stressed *aak-2 *mutants were regulated in the same way as in stressed wild type may indicate that under the severe oxidative stress, other alternative compensatory defense mechanisms were able to regulate DAF-16 downstream genes more efficiently even in the absence of AAK-2.

### AAK-2 may regulate genes involved in fat synthesis, transport, and storage

It has been previously shown that mammalian AMPK is involved in down-regulating fatty acid synthesis in liver and adipose cell, and up-regulating fatty acid oxidation in muscle [5,9]. Also, in another study, Narbonne and Roy found that in *C. elegans *dauer, AAK-2 acts in adipose-like tissues to down-regulate triglyceride hydrolysis so that these lipid reserves are rationed to last the entire duration of the arrest [11].

As discussed earlier, based on our data, we speculate that AAK-2 may act to inhibit fat synthesis under oxidative stress by down-regulating genes involved in lipid synthesis, such as Δ9 fatty acid desaturases (*fat-5 *to *fat-7*) which will require further experiments to confirm and reveal specific association with AAK-2. The regulation of fat synthesis is essential for survival of animals in unfavorable environmental conditions [44,45]. Δ9 fatty acid desaturases are involved in lipid synthesis, specifically in production of monounsaturated fatty acids which are components of triacylglycerides (TAGs) and phospholipids [46]. TAGs are stored in lipid droplets and yolk which make up important energy stores. The increased transcription levels of the Δ9 fatty acid desaturases in *aak-2 *mutants signify increased fat synthesis in these mutants. This energy-consuming fat synthesis is however, down-regulated in wild type animals under conditions of oxidative stress.

Vitellogenins were one of the most highly expressed groups of genes in *aak-2 *mutants. Vitellogenins function in fat storage and are used as protein components for intercellular transport of lipid particles, including yolk [47]. Speculatively, this might be explained as a consequence of the increased fat synthesis in *aak-2 *mutants. Additionally, *ptc-1*and *ptc-2 *were also significantly up-regulated in *aak-2 *mutants. *ptc-1 *and *ptc-2*, orthologs of Drosophila PATCHED (PTC) and human PTCH, define one of seven paralogous families of sterol sensing domain (SSD) proteins that are required for lipid transport [48,49].

Taken together, our data suggests that AAK-2 may be involved in negatively regulating lipid synthesis, transport, and storage in response to oxidative stress in *C. elegans *and provides potential genes which may be involved in these processes. This result supports the characterized complex role of AAK-2 in regulation of lipid metabolism.

### AAK-2 may regulate genes involved in reproduction

We have previously shown AAK-2 expression in the distal tip cells, spermatheca, and sheath cells [14]. Also, AAK-2 is known to be involved in germ cell cycle arrest upon dauer entry [10]. In *Drosophila melanogaster*, it has been shown that a deletion of the single AMPK α gene results in lethality, with severe abnormalities in cell polarity and mitosis [50].

Our data suggests that AAK-2 may have a general role in the regulation of genes involved in germline proliferation and reproduction in *C. elegans *as in unstressed *aak-2 *mutants, genes involved in reproductive processes such as gamete generation, oviposition, sex differentiation, regulation of meiosis, cell division, vulval development, and fertilization were up-regulated relative to wild type animals.

For instance, PTC-1 and PTC-2 are significantly up-regulated in unstressed *aak-2 *mutants relative to wild type but significantly down-regulated in stressed wild type and are involved in lipid transport as mentioned above. Interestingly, PTC-1, the activity and expression of which is essentially confined to the germ line [49], is required for cytokinesis in the germline, but not in somatic cells, and also essential to isolate meiotic germline nuclei from one another so that their nuclear divisions are asynchronous [48,49]. PTC-1 is enriched in the plasma membrane of mitotic germ cells undergoing proliferation, or membranes of oocytes undergoing rapid expansions [51]. Similarly, PTC-2 is also necessary for normal egg osmotic integrity, locomotion, egg laying, and viability [49]. Also, the significantly up-regulated transcription of vitellogenin genes which function in lipid transport and storage in the germline indicates energy-intensive production of vitellogenin-rich oocytes in unstressed *aak-2 *mutants.

In contrast, most major sperm protein genes were highly down-regulated in unstressed *aak-2 *mutants relative to wild type. Major sperm proteins (MSPs) are involved in both extracellular signaling and cytoskeletal functions during reproduction [52]. MSP antagonizes Eph/ephrin signaling, in part, by binding VAB-1 Eph receptor tyrosine kinase on oocytes and sheath cells to promote oocyte maturation and MAPK activation [53,54]. MSP proteins assemble into fibrous networks that drive movement of the *C. elegans *sperm [55]. Also, EMO-1 which is required for oogenesis and ovulation is highly down-regulated in unstressed *aak-2 *mutants relative to wild type [29].

These gene expression changes seen in unstressed *aak-2 *mutants relative to wild type were not shown in stressed *aak-2 *mutants. This suggests that the misregulation of germline specific genes shown in unstressed *aak-2 *mutants presumably caused by the absence of AAK-2 activities were recovered when the *aak-2 *mutants were under more severe oxidative stress, and this may indicate presence of alternative defense mechanisms that compensated for the lost AAK-2 activities.

Additionally, the most highly down-regulated genes in *aak-2 *mutants (stressed and unstressed) are F15E11.1 (i.e. log_2 _= -7, p-val = 1.2E-213) and the paralogs F15E11.12, F15E11.13, F15E11.15, and Y19D10B.7 which belong to a nematode-specific family. F15E11.1 encodes a 17.4 kDa protein with unknown function that is seven-fold more abundant in *glp-1 *mutant hermaphrodites (which lack a germline) than in normal hermaphrodites [56]. This also suggests that AAK-2 may function in the germline as it regulates transcription of F15E11.1 and the paralogs which are known to be involved in the germline.

## Conclusions

We have identified potential downstream target genes of AAK-2 which may be involved in oxidative stress response, specifically with regards to lipid metabolism and reproduction from this comparative transcriptome analysis, however further experiments would be necessary in order to confirm or identify the functions of these candidate genes, and also to elucidate precise relationships with AAK-2. A link between fat metabolism, germline stem cells, and longevity in *C. elegans *has recently been reported as germline stem cell arrest promotes systemic lipolysis, and consequently, life span is prolonged [57]. Our data suggests AAK-2 may play an important role in regulation of these interrelated biological processes. Therefore, the deregulation of lipid metabolism and reproductive processes caused by the absence of AAK-2 might account for the hyper-sensitivity to oxidative stress and potentially shortened life span of *aak-2 *mutants.

## Materials and methods

### Strains and Culture Conditions

Bristol N2 *C. elegans *strains were obtained from the *Caenorhabditis *Genetics Center (Minneapolis, MN). The *aak-2 *(*gt33*) deletion mutant used for this study was kindly provided by Dr. Anton Gartner, The University of Dundee, United Kingdom, and out-crossed three times. All worm strains propagated at 20°C on solid nematode growth media (NGM) seeded with the *E. coli *strain OP50. To prepare paraquat treated samples, adult worms were washed with phosphate-buffered saline and resuspended in the same buffer containing 200 mM paraquat (methyl viologen, Sigma-Aldrich), followed by incubation at 25°C for 3 h. Control pairs were made in the same way, using PBS buffer without adding paraquat. After checking all of the worms alive, total RNA was extracted from four samples using easy-BLUE RNA Extraction Kit (iNtRON, Korea), according to the manufacturer's instructions.

### Construction of whole transcriptome libraries from *C. elegans *RNA

Four libraries were constructed from strain N2, paraquat treated N2, *aak-2 *(*gt33*) and paraquat treated *aak-2 *(gt33). 14-20 ug Dnase I-treated RNA was used to purify poly A + RNA fraction using the MACSTM mRNA Isolation Kit (Cat# 130-075-102, Miltenyi Biotec). Double-stranded cDNAs were made from poly A + RNA (200 ng) using Superscript™Double-Stranded cDNA Synthesis kit (Cat# 11917-010, Invitrogen) and random hexamer primers at 5 μM.

The cDNAs were sonicated for 5 minutes using the cup horn Sonic Dismembrator 550 (Fisher Scientific). Fragmented cDNAs from all four libraries were each size-fractionated on 8% polyacrylamide gels, and the 100 to 300 base pair fractions were excised. The gel-purified cDNA products were modified for sequencing using the genomic DNA prep kit (FC-102-1002, Illumina) as follows: Size-selected cDNAs were subject to end-repair, 3' A overhangs generation, and phosphorylation by T4 DNA polymerase, Klenow DNA Polymerase, and T4 polynucleotide kinase respectively in a single reaction, and then ligated to Illumina adapters, which contain 5' T overhangs. The adapter-ligated products were purified on Qiaquick spin columns (Qiagen), then PCR-amplified with Phusion DNA Polymerase in 10 cycles using Illumina's genomic DNA primer set (Illumina). PCR products were purified on Qiaquick MinElute columns (Qiagen) and the DNA quality assessed and quantified using an Agilent DNA 1000 series II assay and Nanodrop 7500 spectrophotometer (Nanodrop) and diluted to 10 nM. Cluster generation and sequencing was performed on the Illumina cluster station and 1G analyzer (Illumina) following manufacturer's instructions. Sequences were extracted from the resulting image files using the open source Firecrest and Bustard applications (Illumina) on a 32 CPU cluster.

### Molecular mapping methods

Sequences were obtained from the Solexa (Illumina GA) sequencing machine in the seq.txt format, from lanes. The individual files were processed and converted to fasta format, while removing sequences with uncalled bases, or containing runs of more than 19 adenines. The FASTA files containing the sequences for each lane were then aligned against a collection of all annotated transcripts available from Biomart.org (WS180) using Exonerate 1.0, (--model ungapped--bestn 1--showalignment 0--showvulgar 1).

### Statistical analysis

In order to determine statistically significant differential gene expression from pair wise comparisons of the four libraries, i.e. wild type, stressed wild type, *aak-2 *and stressed *aak-2*, we applied Audic-Claverie test of statistical significance [21] using normalized read frequencies (i.e. Normalized number of reads per gene = number of reads mapped to a gene * the read length (36 bases)/the gene length/total number of reads in the library) of commonly identified genes. Additional File 19 lists number of reads before and after the normalization mapped to transcripts. We then selected genes that had p-values less than 0.01 and log_2 _expression level ratios greater than 0.7 (Minimum of ~1.5 difference) for every comparison.

## List of abbreviations

AMPK: AMP activated protein kinase; ROS: reactive oxygen species; MSPs: major sperm proteins; TAGs: triacylglycerides

## Competing interests

The authors declare that they have no competing interests.

## Authors' contributions

HS carried out the data analyses and wrote the manuscript. HL prepared the mRNA samples, contributed to analyzing the data and writing the manuscript, and performed Q-PCR validation experiments. AF designed the sequence mapping program. SJMJ, HK, and DLB conceived of the study. All authors read and approved the final manuscript.

## Authors' information

SJMJ is a senior scholar of the Michael Smith Foundation for Health Research. DLB holds a Canada Research Chair in Genomics.

## Supplementary Material

Additional file 1 **Supplementary Table S1**. Summary of the sequencing and mapping the data to the C. elegans transcriptome Click here for file

Additional file 2 **Supplementary Figure S1**. Q-PCR validation results using a biological replicate Click here for file

Additional file 3 **Supplementary Table S2**. Significantly up or down regulated in stressed wild type relative to unstressed wild type Click here for file

Additional file 4 **Supplementary Table S3**. GO analysis for up or down regualted genes in stressed wild type relative to unstressed wild type Click here for file

Additional file 5 **Supplementary Table S4**. Genes that were significantly down-regulated in wild type animals under oxidative stress Click here for file

Additional file 6 **Supplementary Table S5**. Significantly up-regulated genes in stressed aak-2 mutants relative to wild type Click here for file

Additional file 7 **Supplementary Table S6**. Commonly down-regulated genes in stressed wild type and stressed aak-2 mutants relative to wild type Click here for file

Additional file 8 **Supplementary Table S7**. Commonly up-regulated genes in stressed wild type and stressed aak-2 mutants relative to wild type Click here for file

Additional file 9 **Supplementary Table S8**. 125 genes up regulated in stressed aak-2, but insignificantly changed in stressed wild type and their fold changes of gene expression levels in stressed wild type and stressed aak-2 relative to wild type Click here for file

Additional file 10 **Supplementary Table S9**. 125 gene up-regulated in stressed aak-2 relative to wild type but insignificantly changed in stressed wild type relative to wild type and most highly represented biological processes these genes are involved in Click here for file

Additional file 11 **Supplementary Table S10**. Genes that are significantly up-regulated in unstressed aak-2 mutants compared to wild type animals Click here for file

Additional file 12 **Supplementary Table S11**. Genes that are significantly up-regulated in unstressed aak-2 mutants compared to wild type animals and most highly represented biological processes these genes are involved in Click here for file

Additional file 13 **Supplementary Table S12**. Significantly down-regulated genes in unstressed aak-2 relative to wild type Click here for file

Additional file 14 **Supplementary Table S13**. Significantly down-regulated genes in unstressed aak-2 relative to wild type and most highly represented biological processes these genes are involved in Click here for file

Additional file 15 **Supplementary Table S14**. Commonly up-regulated genes in unstressed aak-2, stressed aak-2, and stressed wild type and most highly represented biological processes these genes are involved in Click here for file

Additional file 16 **Supplementary Table S15**. Commonly down-regulated genes in unstressed aak-2, stressed aak-2, and stressed wild type and most highly represented biological processes these genes are involved in Click here for file

Additional file 17 **Supplementary Table S16**. Genes which show no significant change in expression, or are significantly down-regulated in unstressed aak-2 mutants that are commonly up-regulated in both stressed aak-2 and stressed wild type Click here for file

Additional file 18 **Supplementary Table S17**. Genes significantly up or down-regulated only in unstressed aak-2 mutants relative to wild type, but insignificantly changed in stressed wild type and stressed aak-2 mutants relative to wild type Click here for file

Additional file 19 **Supplementary Table S18**. Number of reads mapped to transcripts before and after normalization Click here for file

## References

[B1] Munoz MJ Riddle DL Positive selection of Caenorhabditis elegans mutants with increased stress resistance and longevity Genetics 2003 163 171 180 12586705 10.1093/genetics/163.1.171 PMC1462431

[B2] Johnson TE de Castro E Hegi de Castro S Cypser J Henderson S Relationship between increased longevity and stress resistance as assessed through gerontogene mutations in Caenorhabditis elegans Exp Gerontol 2001 36 1609 1617 10.1016/S0531-5565(01)00144-9 11672983

[B3] Murakami S Johnson TE The OLD-1 positive regulator of longevity and stress resistance is under DAF-16 regulation in Caenorhabditis elegans Curr Biol 2001 11 1517 1523 10.1016/S0960-9822(01)00453-5 11591319

[B4] Hardie DG Scott JW Pan DA Hudson ER Management of cellular energy by the AMP-activated protein kinase system FEBS Lett 2003 546 113 120 10.1016/S0014-5793(03)00560-X 12829246

[B5] Hardie DG The AMP-activated protein kinase pathway--new players upstream and downstream J Cell Sci 2004 117 5479 5487 10.1242/jcs.01540 15509864

[B6] Imamura K Ogura T Kishimoto A Kaminishi M Esumi H Cell cycle regulation via p53 phosphorylation by a 5'-AMP activated protein kinase activator, 5-aminoimidazole-4-carboxamide-1-beta-D-ribofuranoside, in a human hepatocellular carcinoma cell line Biochem Biophys Res Commun 2001 287 562 567 10.1006/bbrc.2001.5627 11554766

[B7] Shaw RJ Lamia KA Vasquez D Koo SH Bardeesy N The kinase LKB1 mediates glucose homeostasis in liver and therapeutic effects of metformin Science 2005 310 1642 1646 10.1126/science.1120781 16308421 PMC3074427

[B8] Solaz-Fuster MC Gimeno-Alcaniz JV Casado M Sanz P TRIP6 transcriptional co-activator is a novel substrate of AMP-activated protein kinase Cell Signal 2006 18 1702 1712 10.1016/j.cellsig.2006.01.021 16624523

[B9] Kahn BB Alquier T Carling D Hardie DG AMP-activated protein kinase: ancient energy gauge provides clues to modern understanding of metabolism Cell Metab 2005 1 15 25 10.1016/j.cmet.2004.12.003 16054041

[B10] Narbonne P Roy R Inhibition of germline proliferation during C. elegans dauer development requires PTEN, LKB1 and AMPK signalling Development 2006 133 611 619 10.1242/dev.02232 16407400

[B11] Narbonne P Roy R Caenorhabditis elegans dauers need LKB1/AMPK to ration lipid reserves and ensure long-term survival Nature 2009 457 210 214 10.1038/nature07536 19052547

[B12] Klass M Hirsh D Non-ageing developmental variant of Caenorhabditis elegans Nature 1976 260 523 525 10.1038/260523a0 1264206

[B13] Apfeld J O'Connor G McDonagh T DiStefano PS Curtis R The AMP-activated protein kinase AAK-2 links energy levels and insulin-like signals to lifespan in C. elegans Genes Dev 2004 18 3004 3009 10.1101/gad.1255404 15574588 PMC535911

[B14] Lee H Cho JS Lambacher N Lee J Lee SJ The Caenorhabditis elegans AMP-activated protein kinase AAK-2 is phosphorylated by LKB1 and is required for resistance to oxidative stress and for normal motility and foraging behavior J Biol Chem 2008 283 14988 14993 10.1074/jbc.M709115200 18408008 PMC3258889

[B15] Kim Y Sun H Functional genomic approach to identify novel genes involved in the regulation of oxidative stress resistance and animal lifespan Aging Cell 2007 6 489 503 10.1111/j.1474-9726.2007.00302.x 17608836

[B16] Allen RG Tresini M Oxidative stress and gene regulation Free Radic Biol Med 2000 28 463 499 10.1016/S0891-5849(99)00242-7 10699758

[B17] Curtis R O'Connor G DiStefano PS Aging networks in Caenorhabditis elegans: AMP-activated protein kinase (aak-2) links multiple aging and metabolism pathways Aging Cell 2006 5 119 126 10.1111/j.1474-9726.2006.00205.x 16626391

[B18] Marioni JC Mason CE Mane SM Stephens M Gilad Y RNA-seq: an assessment of technical reproducibility and comparison with gene expression arrays Genome Res 2008 18 1509 1517 10.1101/gr.079558.108 18550803 PMC2527709

[B19] Hillier LW Reinke V Green P Hirst M Marra MA Massively parallel sequencing of the polyadenylated transcriptome of C. elegans Genome Res 2009 19 657 666 10.1101/gr.088112.108 19181841 PMC2665784

[B20] Bennett S Solexa Ltd Pharmacogenomics 2004 5 433 438 10.1517/14622416.5.4.433 15165179

[B21] Audic S Claverie JM The significance of digital gene expression profiles Genome Res 1997 7 986 995 9331369 10.1101/gr.7.10.986

[B22] Beissbarth T Speed TP GOstat: find statistically overrepresented Gene Ontologies within a group of genes Bioinformatics 2004 20 1464 1465 10.1093/bioinformatics/bth088 14962934

[B23] Baumbach LL Stein GS Stein JL Regulation of human histone gene expression: transcriptional and posttranscriptional control in the coupling of histone messenger RNA stability with DNA replication Biochemistry 1987 26 6178 6187 10.1021/bi00393a034 3689769

[B24] Murphy CT McCarroll SA Bargmann CI Fraser A Kamath RS Genes that act downstream of DAF-16 to influence the lifespan of Caenorhabditis elegans Nature 2003 424 277 283 10.1038/nature01789 12845331

[B25] Melendez A Talloczy Z Seaman M Eskelinen EL Hall DH Autophagy genes are essential for dauer development and life-span extension in C. elegans Science 2003 301 1387 1391 10.1126/science.1087782 12958363

[B26] Alper S McBride SJ Lackford B Freedman JH Schwartz DA Specificity and complexity of the Caenorhabditis elegans innate immune response Mol Cell Biol 2007 27 5544 5553 10.1128/MCB.02070-06 17526726 PMC1952075

[B27] Matyash V Geier C Henske A Mukherjee S Hirsh D Distribution and transport of cholesterol in Caenorhabditis elegans Mol Biol Cell 2001 12 1725 1736 11408580 10.1091/mbc.12.6.1725 PMC37336

[B28] Yu H Larsen PL DAF-16-dependent and independent expression targets of DAF-2 insulin receptor-like pathway in Caenorhabditis elegans include FKBPs J Mol Biol 2001 314 1017 1028 10.1006/jmbi.2000.5210 11743719

[B29] Iwasaki K McCarter J Francis R Schedl T emo-1, a Caenorhabditis elegans Sec61p gamma homologue, is required for oocyte development and ovulation J Cell Biol 1996 134 699 714 10.1083/jcb.134.3.699 8707849 PMC2120936

[B30] Kramer JM Johnson JJ Edgar RS Basch C Roberts S The sqt-1 gene of C. elegans encodes a collagen critical for organismal morphogenesis Cell 1988 55 555 565 10.1016/0092-8674(88)90214-0 3180220

[B31] Johnstone IL Shafi Y Barry JD Molecular analysis of mutations in the Caenorhabditis elegans collagen gene dpy-7 EMBO J 1992 11 3857 3863 1396579 10.1002/j.1460-2075.1992.tb05478.x PMC556895

[B32] von Mende N Bird DM Albert PS Riddle DL dpy-13: a nematode collagen gene that affects body shape Cell 1988 55 567 576 10.1016/0092-8674(88)90215-2 2846184

[B33] Cox GN Hirsh D Stage-specific patterns of collagen gene expression during development of Caenorhabditis elegans Mol Cell Biol 1985 5 363 372 2983191 10.1128/mcb.5.2.363-372.1985 PMC366719

[B34] Coolon JD Jones KL Todd TC Carr BC Herman MA Caenorhabditis elegans genomic response to soil bacteria predicts environment-specific genetic effects on life history traits PLoS Genet 2009 5 e1000503 10.1371/journal.pgen.1000503 19503598 PMC2684633

[B35] Wong D Bazopoulou D Pujol N Tavernarakis N Ewbank JJ Genome-wide investigation reveals pathogen-specific and shared signatures in the response of Caenorhabditis elegans to infection Genome Biol 2007 8 R194 10.1186/gb-2007-8-9-r194 17875205 PMC2375032

[B36] Aragno M Mastrocola R Alloatti G Vercellinatto I Bardini P Oxidative stress triggers cardiac fibrosis in the heart of diabetic rats Endocrinology 2008 149 380 388 10.1210/en.2007-0877 17901230

[B37] del Carmen EM Souza V Bucio L Hernandez E Damian-Matsumura P Cadmium induces alpha(1)collagen (I) and metallothionein II gene and alters the antioxidant system in rat hepatic stellate cells Toxicology 2002 170 63 73 10.1016/S0300-483X(01)00531-5 11750084

[B38] Budovskaya YV Wu K Southworth LK Jiang M Tedesco P An elt-3/elt-5/elt-6 GATA transcription circuit guides aging in C. elegans Cell 2008 134 291 303 10.1016/j.cell.2008.05.044 18662544 PMC4719053

[B39] Halaschek-Wiener J Khattra JS McKay S Pouzyrev A Stott JM Analysis of long-lived C. elegans daf-2 mutants using serial analysis of gene expression Genome Res 2005 15 603 615 10.1101/gr.3274805 15837805 PMC1088289

[B40] Kenyon C Chang J Gensch E Rudner A Tabtiang R A C. elegans mutant that lives twice as long as wild type Nature 1993 366 461 464 10.1038/366461a0 8247153

[B41] Schaffitzel E Hertweck M Recent aging research in Caenorhabditis elegans Exp Gerontol 2006 41 557 563 10.1016/j.exger.2006.02.008 16584861

[B42] Greer EL Dowlatshahi D Banko MR Villen J Hoang K An AMPK-FOXO pathway mediates longevity induced by a novel method of dietary restriction in C. elegans Curr Biol 2007 17 1646 1656 10.1016/j.cub.2007.08.047 17900900 PMC2185793

[B43] McElwee J Bubb K Thomas JH Transcriptional outputs of the Caenorhabditis elegans forkhead protein DAF-16 Aging Cell 2003 2 111 121 10.1046/j.1474-9728.2003.00043.x 12882324

[B44] Murray P Hayward SA Govan GG Gracey AY Cossins AR An explicit test of the phospholipid saturation hypothesis of acquired cold tolerance in Caenorhabditis elegans Proc Natl Acad Sci USA 2007 104 5489 5494 10.1073/pnas.0609590104 17369360 PMC1838478

[B45] Van Gilst MR Hadjivassiliou H Yamamoto KR A Caenorhabditis elegans nutrient response system partially dependent on nuclear receptor NHR-49 Proc Natl Acad Sci USA 2005 102 13496 13501 10.1073/pnas.0506234102 16157872 PMC1201344

[B46] Watts JL Fat synthesis and adiposity regulation in Caenorhabditis elegans Trends Endocrinol Metab 2009 20 58 65 10.1016/j.tem.2008.11.002 19181539 PMC2665873

[B47] Mullaney BC Ashrafi K C. elegans fat storage and metabolic regulation Biochim Biophys Acta 2009 1791 474 478 19168149 10.1016/j.bbalip.2008.12.013 PMC2772880

[B48] Zugasti O Rajan J Kuwabara PE The function and expansion of the Patched- and Hedgehog-related homologs in C. elegans Genome Res 2005 15 1402 1410 10.1101/gr.3935405 16204193 PMC1240083

[B49] Kuwabara PE Lee MH Schedl T Jefferis GS A C. elegans patched gene, ptc-1, functions in germ-line cytokinesis Genes Dev 2000 14 1933 1944 10921907 PMC316821

[B50] Lee JH Koh H Kim M Kim Y Lee SY Energy-dependent regulation of cell structure by AMP-activated protein kinase Nature 2007 447 1017 1020 10.1038/nature05828 17486097

[B51] Burglin TR Kuwabara PE Homologs of the Hh signalling network in C. elegans WormBook 2006 1 14 18050469 10.1895/wormbook.1.76.1 PMC4781598

[B52] Miller MA Nguyen VQ Lee MH Kosinski M Schedl T A sperm cytoskeletal protein that signals oocyte meiotic maturation and ovulation Science 2001 291 2144 2147 10.1126/science.1057586 11251118

[B53] McCarter J Bartlett B Dang T Schedl T On the control of oocyte meiotic maturation and ovulation in Caenorhabditis elegans Dev Biol 1999 205 111 128 10.1006/dbio.1998.9109 9882501

[B54] Kuwabara PE The multifaceted C. elegans major sperm protein: an ephrin signaling antagonist in oocyte maturation Genes Dev 2003 17 155 161 10.1101/gad.1061103 12533505

[B55] Miller MA Ruest PJ Kosinski M Hanks SK Greenstein D An Eph receptor sperm-sensing control mechanism for oocyte meiotic maturation in Caenorhabditis elegans Genes Dev 2003 17 187 200 10.1101/gad.1028303 12533508 PMC195972

[B56] Krijgsveld J Ketting RF Mahmoudi T Johansen J Artal-Sanz M Metabolic labeling of C. elegans and D. melanogaster for quantitative proteomics Nat Biotechnol 2003 21 927 931 10.1038/nbt848 12858183

[B57] Wang MC O'Rourke EJ Ruvkun G Fat metabolism links germline stem cells and longevity in C. elegans Science 2008 322 957 960 10.1126/science.1162011 18988854 PMC2760269

