# Supplementary material for: Gene expression profiling of oxidative stress response of C. elegans aging defective AMPK mutants using massively parallel transcriptome sequencing
Source: BMC Res Notes. 2011 Feb 8;4:34. doi: 10.1186/1756-0500-4-34 (PMC3045954; doi:10.1186/1756-0500-4-34)
Supplement: Additional file 1 — Supplementary Table S1. Summary of the sequencing and mapping the data to the C. elegans transcriptome [file 1756-0500-4-34-S1.PDF]

Table 1. Summary of the sequencing and mapping the data to the C. elegans transcriptome

	N2 (wildtype)	Stressed N2	Unstressed *aak-2*	Stressed *aak-2*
**Total sequence reads**	12,446,501	8,419,728	11,860,313	9,086,838
**Unambiguously mapped to *C. elegans* transcriptome (%)**	7,486,131<br>60.15%	5,408,283<br>64.23%	8,418,505<br>70.98%	5,857,401<br>64.46%
***Genes identified from mapping (%)**	20,267<br>85.55%	20,258<br>85.51%	19,892<br>83.96%	20,441<br>86.28%

*WormBase version WS180 was used for this analysis. Total number of genes in WS180 is 23,691.
