# Supplementary material for: Gene expression profiling of oxidative stress response of C. elegans aging defective AMPK mutants using massively parallel transcriptome sequencing
Source: BMC Res Notes. 2011 Feb 8;4:34. doi: 10.1186/1756-0500-4-34 (PMC3045954; doi:10.1186/1756-0500-4-34)
Supplement: Additional file 2 — Supplementary Figure S1. Q-PCR validation results using a biological replicate [file 1756-0500-4-34-S2.PDF]

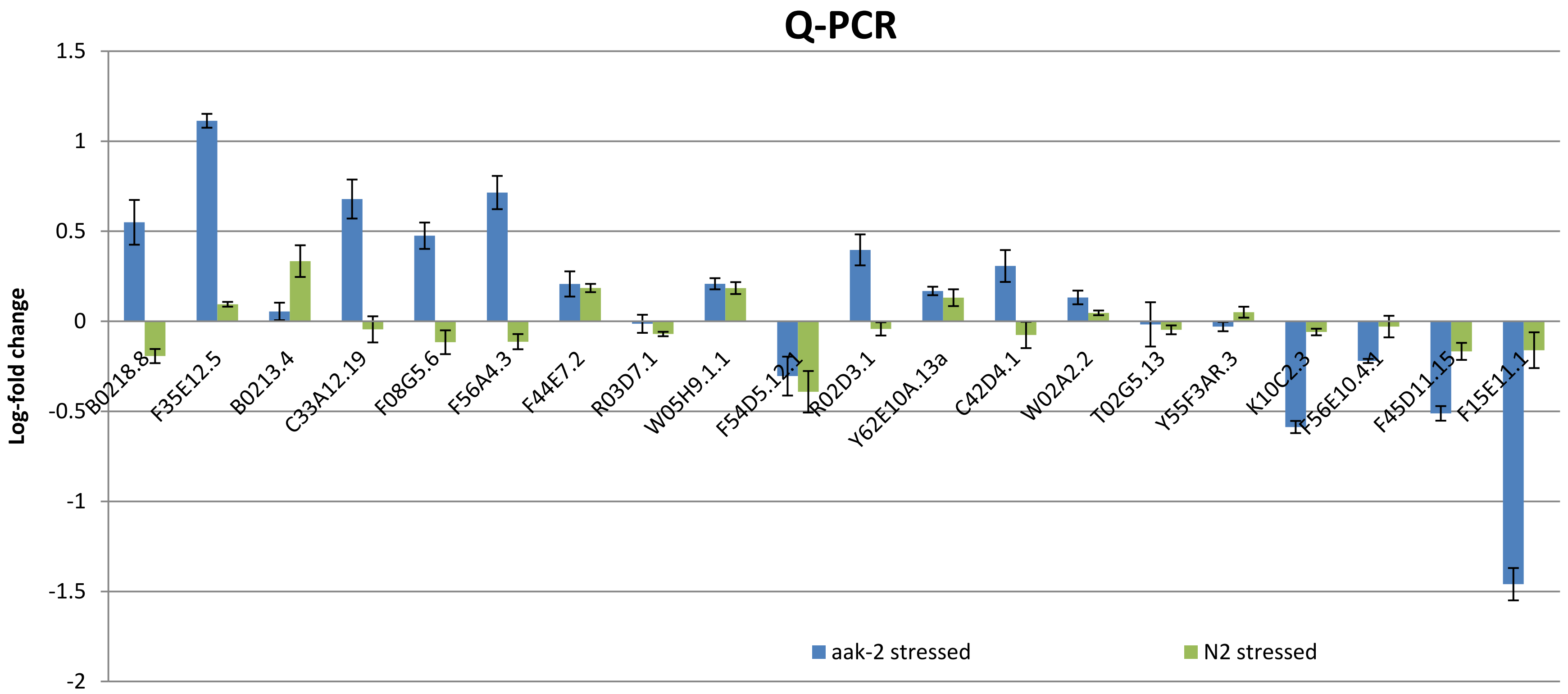

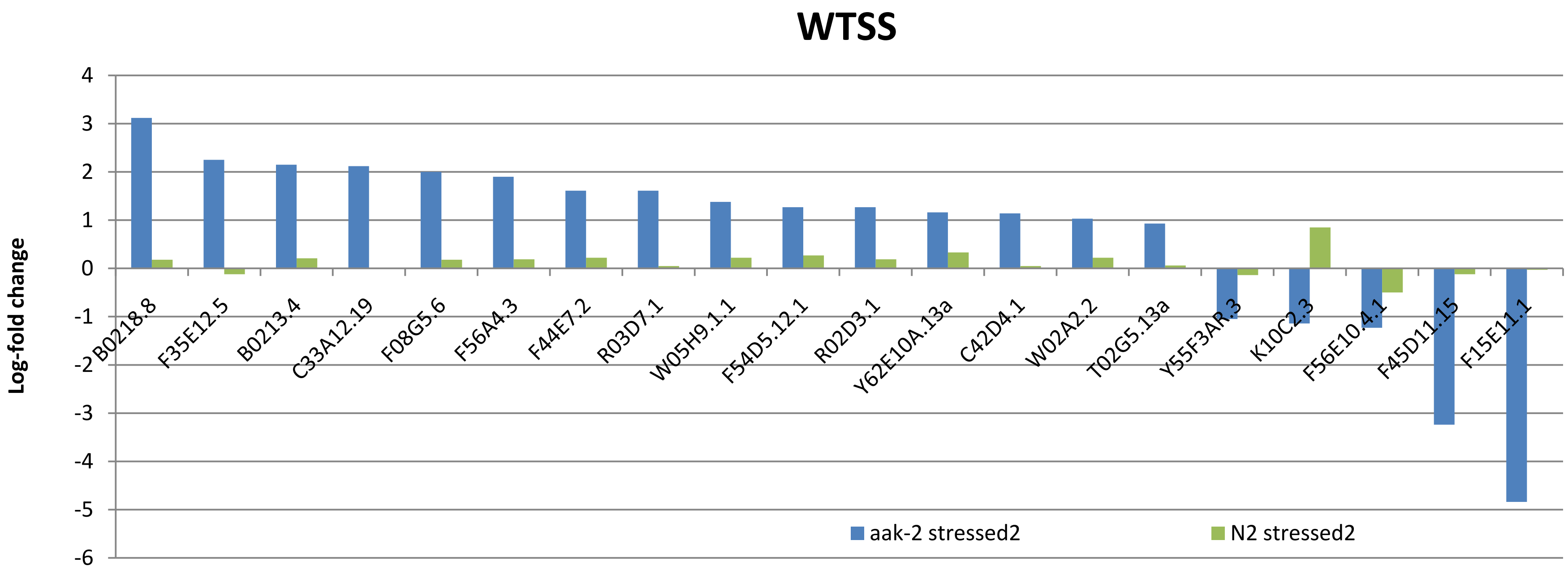

Supplemental Figure 1. Q-PCR validation results using a biological replicate

Comparison of Q-PCR and WTSS results for some of the most significantly up or down-regulated in stressed *aak-2* mutants but insignificantly changed in stressed wild type relative to wild type (unstressed). The results present a good correlation between Q-PCR and WTSS. Q-PCR experiment was performed using new RNA samples that represent a biological replicate.
