# Supplementary material for: Gene expression profiling of oxidative stress response of C. elegans aging defective AMPK mutants using massively parallel transcriptome sequencing
Source: BMC Res Notes. 2011 Feb 8;4:34. doi: 10.1186/1756-0500-4-34 (PMC3045954; doi:10.1186/1756-0500-4-34)
Supplement: Additional file 3 — Supplementary Table S2. Significantly up or down regulated in stressed wild type relative to unstressed wild type [file 1756-0500-4-34-S3.PDF]

**Supplementary Table 2. Significantly up or down regulated in stressed wild type relative to unstressed wild type**

| gene | log2(stressedN2/N2) | pval | log2(unstressed aak-2/N2) | pval2 | log2(stressed aak-2/N2) | pval3 |
|---|---|---|---|---|---|---|
| ttn-1 | 3.97 | 4.88E-04 | 1.44 | 1.83E-01 | 3.52 | 2.36E-03 |
| abu-6 | 3.55 | 1.28E-03 | 0.68 | 2.48E-01 | 3.12 | 1.10E-02 |
| pqn-5 | 3.44 | 4.58E-03 | 0.53 | 2.59E-01 | 3.11 | 1.08E-02 |
| abu-7 | 3.44 | 4.51E-03 | 0.55 | 2.58E-01 | 2.99 | 2.90E-02 |
| dao-4 | 2.95 | 6.09E-04 | 0.22 | 2.01E-01 | 2.88 | 8.66E-04 |
| qua-1 | 2.94 | 9.08E-03 | 0.72 | 1.82E-01 | 3.56 | 1.61E-03 |
| pqn-95 | 2.94 | 1.55E-04 | 1.00 | 8.11E-02 | 3.09 | 7.46E-05 |
| rol-1 | 2.88 | 1.31E-09 | 0.72 | 5.14E-02 | 2.45 | 9.39E-07 |
| F57H12.6 | 2.79 | 3.66E-04 | -0.76 | 1.39E-01 | 1.85 | 2.72E-02 |
| mlt-8 | 2.78 | 4.39E-06 | 0.64 | 8.49E-02 | 3.03 | 2.82E-08 |
| R02F11.1 | 2.70 | 4.66E-03 | 0.98 | 1.47E-01 | 2.83 | 3.20E-03 |
| C27D6.3 | 2.69 | 2.27E-03 | 0.07 | 2.14E-01 | 2.71 | 4.23E-03 |
| hsp-16.11 | 2.59 | 2.99E-08 | 1.39 | 3.08E-03 | 4.82 | 6.85E-59 |
| col-109 | 2.55 | 2.38E-05 | 0.52 | 1.08E-01 | 2.43 | 1.02E-04 |
| hsp-16.48 | 2.53 | 7.26E-12 | 0.93 | 1.31E-02 | 4.46 | 1.30E-67 |
| hsp-16.49 | 2.53 | 7.26E-12 | 0.93 | 1.31E-02 | 4.46 | 1.30E-67 |
| ZK180.6 | 2.53 | 4.97E-04 | 0.82 | 1.10E-01 | 2.77 | 3.47E-05 |
| hsp-16.1 | 2.49 | 8.83E-10 | 1.38 | 8.94E-04 | 4.67 | 6.41E-71 |
| F15B9.8 | 2.49 | 7.35E-03 | 0.56 | 1.58E-01 | 3.27 | 3.83E-05 |
| bli-1 | 2.46 | 1.67E-03 | -0.04 | 1.60E-01 | 2.37 | 2.16E-03 |
| T19C3.2 | 2.44 | 3.29E-03 | 0.36 | 1.65E-01 | 2.77 | 1.66E-03 |
| R02E4.3 | 2.43 | 2.32E-03 | 0.24 | 1.65E-01 | 3.87 | 5.68E-10 |
| col-138 | 2.39 | 6.61E-07 | 0.73 | 4.65E-02 | 2.68 | 1.14E-08 |
| bli-2 | 2.37 | 2.29E-04 | -0.31 | 1.21E-01 | 2.55 | 1.91E-05 |
| Y47D7A.15 | 2.36 | 5.61E-04 | 0.02 | 1.40E-01 | 1.88 | 4.86E-03 |
| wrt-4 | 2.33 | 9.24E-03 | 0.49 | 1.65E-01 | 2.20 | 9.96E-03 |
| hsp-16.41 | 2.32 | 9.07E-06 | 0.27 | 1.02E-01 | 4.24 | 1.37E-30 |
| hsp-16.2 | 2.30 | 8.95E-04 | 0.46 | 1.34E-01 | 4.34 | 1.20E-17 |
| F53B1.4 | 2.29 | 1.93E-04 | 0.46 | 1.08E-01 | 2.32 | 6.80E-05 |
| F33D4.6 | 2.26 | 8.38E-04 | 0.06 | 1.52E-01 | 2.73 | 7.35E-06 |
| K02E11.10 | 2.24 | 6.64E-04 | 0.07 | 1.53E-01 | 2.40 | 3.24E-04 |
| col-175 | 2.19 | 1.11E-06 | 0.01 | 1.03E-01 | 2.52 | 6.23E-09 |
| grl-16 | 2.19 | 8.66E-05 | 0.42 | 1.02E-01 | 2.08 | 3.51E-04 |
| E01G4.6 | 2.18 | 3.08E-04 | 0.47 | 1.00E-01 | 2.83 | 4.71E-08 |
| ZK180.5 | 2.17 | 1.45E-04 | 0.83 | 6.35E-02 | 2.13 | 2.86E-04 |
| H23N18.5 | 2.12 | 2.56E-05 | -1.08 | 5.88E-02 | 2.40 | 1.40E-06 |
| F49H12.5 | 2.11 | 1.46E-04 | 0.68 | 7.25E-02 | 2.39 | 4.24E-06 |
| F56D3.1 | 2.09 | 9.66E-04 | 0.08 | 1.38E-01 | 2.04 | 1.55E-03 |
| H06I04.3 | 2.07 | 6.44E-04 | 1.78 | 2.95E-03 | 1.81 | 3.19E-03 |
| Y94H6A.10 | 2.06 | 1.05E-06 | 0.55 | 4.68E-02 | 2.65 | 1.68E-12 |
| grl-7 | 2.05 | 2.17E-03 | -0.07 | 1.43E-01 | 2.42 | 2.36E-04 |
| K01D12.9 | 2.03 | 3.29E-04 | -1.21 | 1.07E-01 | 1.67 | 2.56E-03 |
| T06D8.1 | 2.01 | 8.14E-04 | 0.97 | 3.97E-02 | 1.92 | 1.12E-03 |

| | | | | | | |
|---|---|---|---|---|---|---|
| col-73 | 2.00 | 9.17E-16 | -0.06 | 4.98E-02 | 2.19 | 8.27E-20 |
| ZK546.14 | 1.97 | 3.25E-04 | 1.73 | 4.89E-04 | 1.77 | 7.87E-04 |
| col-49 | 1.91 | 2.50E-04 | -0.01 | 1.12E-01 | 1.90 | 2.41E-04 |
| col-60 | 1.90 | 4.08E-03 | 0.24 | 1.33E-01 | 1.92 | 2.85E-03 |
| frm-1 | 1.89 | 1.53E-04 | 1.70 | 4.50E-04 | 1.90 | 7.28E-05 |
| lon-8 | 1.88 | 9.26E-03 | -0.54 | 1.69E-01 | 2.62 | 2.98E-04 |
| ttr-14 | 1.88 | 9.14E-03 | 1.08 | 6.84E-02 | 2.52 | 1.27E-04 |
| col-14 | 1.88 | 4.94E-05 | 0.44 | 6.37E-02 | 2.23 | 2.09E-07 |
| col-63 | 1.87 | 6.80E-04 | 0.32 | 9.92E-02 | 2.48 | 1.23E-06 |
| Y22D7AL.5 | 1.87 | 4.37E-09 | 1.23 | 1.18E-04 | 1.50 | 6.25E-06 |
| pqn-32 | 1.86 | 8.25E-03 | 0.03 | 1.36E-01 | 2.04 | 3.12E-03 |
| D1005.t1 | 1.86 | 1.39E-03 | -1.50 | 6.84E-02 | 0.32 | 1.23E-01 |
| col-71 | 1.85 | 2.14E-05 | 0.18 | 8.59E-02 | 2.26 | 6.31E-08 |
| H42K12.3 | 1.85 | 5.17E-03 | 0.01 | 1.32E-01 | 2.11 | 4.89E-04 |
| F54C9.9 | 1.85 | 3.61E-03 | 1.60 | 1.12E-02 | 1.65 | 8.53E-03 |
| F49E2.5 | 1.83 | 5.38E-03 | 1.22 | 3.10E-02 | 1.75 | 7.13E-03 |
| K07H8.10 | 1.82 | 2.45E-08 | 1.58 | 6.60E-07 | 1.72 | 1.78E-07 |
| col-157 | 1.81 | 8.86E-07 | 0.02 | 7.44E-02 | 1.54 | 3.11E-05 |
| col-162 | 1.80 | 2.08E-06 | -0.05 | 7.15E-02 | 1.79 | 1.77E-06 |
| grd-5 | 1.80 | 1.17E-09 | 0.67 | 9.07E-03 | 1.73 | 6.01E-09 |
| F42A10.7 | 1.79 | 7.76E-03 | 0.98 | 6.42E-02 | 1.20 | 4.92E-02 |
| fkb-5 | 1.79 | 2.48E-03 | 0.03 | 1.29E-01 | 1.86 | 2.24E-03 |
| F27D4.4 | 1.79 | 2.71E-03 | 0.72 | 7.79E-02 | 0.86 | 6.30E-02 |
| col-145 | 1.79 | 1.86E-18 | -0.60 | 8.84E-03 | 1.21 | 2.99E-08 |
| C39D10.8 | 1.78 | 8.01E-04 | 0.88 | 3.40E-02 | 1.44 | 5.43E-03 |
| col-38 | 1.77 | 2.04E-08 | 0.33 | 4.35E-02 | 2.18 | 1.21E-13 |
| ZK380.t2 | 1.77 | 7.34E-03 | 0.72 | 7.82E-02 | 0.83 | 8.99E-02 |
| col-120 | 1.75 | 8.27E-03 | -0.10 | 1.49E-01 | 1.38 | 2.82E-02 |
| wrt-10 | 1.75 | 3.77E-03 | -0.19 | 1.31E-01 | 1.66 | 4.88E-03 |
| W02D3.1 | 1.75 | 2.40E-07 | 0.63 | 1.76E-02 | 1.53 | 5.54E-06 |
| dpy-5 | 1.74 | 1.92E-08 | -0.26 | 4.75E-02 | 1.94 | 5.92E-11 |
| col-17 | 1.74 | 4.68E-12 | -0.21 | 3.96E-02 | 1.91 | 3.49E-15 |
| cey-1 | 1.74 | 7.99E-08 | 1.12 | 3.44E-04 | 1.43 | 1.48E-05 |
| tts-2 | 1.73 | 2.10E-03 | 1.05 | 3.81E-02 | 1.99 | 2.75E-04 |
| sqt-1 | 1.71 | 3.54E-05 | -0.51 | 5.93E-02 | 2.39 | 3.52E-10 |
| col-48 | 1.71 | 4.25E-03 | 0.50 | 8.81E-02 | 2.12 | 1.93E-04 |
| F32A5.4 | 1.70 | 2.37E-03 | 0.46 | 8.21E-02 | 2.28 | 8.10E-06 |
| C37A2.8 | 1.69 | 8.22E-03 | 1.35 | 1.75E-02 | 1.68 | 1.14E-02 |
| F20D1.1 | 1.69 | 8.53E-03 | 1.18 | 2.67E-02 | 1.77 | 6.67E-03 |
| C30B5.4 | 1.68 | 4.41E-03 | 0.96 | 3.72E-02 | 1.36 | 1.53E-02 |
| nlp-33 | 1.68 | 1.00E-06 | -1.27 | 5.30E-03 | 2.05 | 2.39E-10 |
| ram-2 | 1.68 | 5.58E-07 | 0.05 | 6.64E-02 | 1.89 | 8.40E-09 |
| col-161 | 1.65 | 1.65E-06 | -0.18 | 5.99E-02 | 1.54 | 3.92E-06 |
| eel-1 | 1.64 | 4.45E-03 | 1.74 | 1.10E-03 | 1.36 | 1.58E-02 |
| Y55F3AM.3 | 1.64 | 9.05E-03 | 1.16 | 2.95E-02 | 0.99 | 6.75E-02 |
| col-77 | 1.63 | 3.05E-06 | 0.09 | 6.53E-02 | 1.69 | 1.08E-06 |
| col-91 | 1.62 | 8.53E-03 | -0.21 | 1.53E-01 | 1.51 | 1.96E-02 |

| | | | | | | |
|---|---|---|---|---|---|---|
| col-130 | 1.60 | 1.61E-03 | -0.08 | 1.14E-01 | 1.57 | 1.45E-03 |
| dpy-13 | 1.57 | 2.99E-08 | -0.38 | 4.13E-02 | 1.76 | 1.05E-10 |
| ZK1248.17 | 1.57 | 6.77E-04 | -1.23 | 4.28E-02 | 1.51 | 1.13E-03 |
| col-176 | 1.54 | 9.12E-03 | -0.64 | 9.77E-02 | 0.79 | 6.68E-02 |
| hsp-2 | 1.54 | 5.39E-06 | 0.59 | 2.19E-02 | 0.75 | 1.22E-02 |
| col-112 | 1.53 | 5.26E-05 | -0.31 | 6.13E-02 | 1.68 | 5.54E-06 |
| iff-2 | 1.53 | 5.26E-17 | 0.58 | 6.58E-04 | 1.53 | 2.33E-17 |
| dpy-4 | 1.53 | 2.72E-09 | -0.29 | 3.57E-02 | 1.47 | 8.96E-09 |
| T23G7.3 | 1.51 | 8.58E-03 | 1.48 | 6.40E-03 | 1.79 | 1.44E-03 |
| W04C9.4 | 1.51 | 4.09E-03 | 0.90 | 3.58E-02 | 1.05 | 2.94E-02 |
| hrp-2 | 1.51 | 6.64E-04 | 1.12 | 5.31E-03 | 1.17 | 6.19E-03 |
| C04F12.7 | 1.50 | 5.67E-03 | 0.47 | 8.38E-02 | 2.48 | 3.64E-07 |
| vha-10 | 1.50 | 1.21E-09 | 0.95 | 7.17E-05 | 1.79 | 1.92E-14 |
| Y71F9AM.6 | 1.49 | 2.13E-11 | 0.92 | 2.08E-05 | 1.28 | 1.14E-08 |
| C30G12.2 | 1.48 | 2.06E-04 | 0.78 | 1.82E-02 | 0.28 | 7.53E-02 |
| tag-210 | 1.47 | 2.50E-07 | 0.61 | 8.01E-03 | 1.28 | 9.09E-06 |
| T02H6.11 | 1.46 | 3.20E-10 | 0.30 | 2.43E-02 | 1.39 | 1.48E-09 |
| F53F1.5 | 1.46 | 4.81E-06 | -0.73 | 3.03E-02 | 0.70 | 1.09E-02 |
| F53F1.4 | 1.45 | 2.82E-18 | -0.74 | 2.21E-04 | 1.09 | 2.28E-10 |
| col-104 | 1.44 | 2.11E-03 | -0.34 | 1.06E-01 | 1.68 | 4.66E-04 |
| Y97E10C.1 | 1.44 | 5.10E-03 | 1.19 | 1.04E-02 | 1.58 | 1.61E-03 |
| rol-8 | 1.43 | 9.32E-04 | -0.64 | 5.60E-02 | 1.87 | 4.49E-06 |
| Y51H7C.13 | 1.42 | 7.67E-03 | 0.46 | 8.47E-02 | 1.95 | 2.78E-04 |
| col-90 | 1.42 | 8.27E-04 | -0.19 | 9.69E-02 | 1.71 | 3.72E-05 |
| fib-1 | 1.41 | 7.61E-07 | 0.93 | 6.09E-04 | 1.35 | 1.64E-06 |
| uaf-1 | 1.40 | 2.16E-03 | 1.45 | 6.41E-04 | 1.05 | 1.64E-02 |
| C36B1.7 | 1.40 | 9.00E-04 | 1.12 | 5.36E-03 | 1.14 | 5.39E-03 |
| sym-1 | 1.39 | 8.69E-03 | 0.28 | 9.85E-02 | 1.48 | 4.37E-03 |
| H03A11.2 | 1.38 | 3.30E-04 | 0.78 | 1.28E-02 | 1.76 | 1.84E-06 |
| prg-2 | 1.38 | 9.10E-03 | 1.68 | 1.84E-03 | 1.66 | 2.77E-03 |
| Y54G2A.23 | 1.38 | 2.26E-04 | 0.88 | 5.50E-03 | 1.25 | 6.70E-04 |
| C18B2.5 | 1.38 | 1.15E-03 | 0.92 | 1.11E-02 | 1.18 | 4.29E-03 |
| trap-1 | 1.37 | 9.84E-10 | 0.76 | 2.70E-04 | 1.18 | 1.41E-07 |
| cpg-7 | 1.36 | 8.23E-03 | -0.64 | 9.89E-02 | 2.20 | 6.03E-06 |
| F39H11.1 | 1.36 | 3.66E-03 | 0.65 | 5.36E-02 | 1.08 | 1.77E-02 |
| col-41 | 1.36 | 1.96E-03 | -1.23 | 4.22E-02 | 1.00 | 1.42E-02 |
| vha-11 | 1.35 | 3.64E-05 | 1.32 | 2.77E-05 | 1.50 | 3.75E-06 |
| F54A3.5 | 1.35 | 2.14E-04 | 0.36 | 4.73E-02 | 1.39 | 1.12E-04 |
| grl-4 | 1.34 | 7.09E-03 | 0.43 | 7.46E-02 | 1.82 | 1.91E-04 |
| ran-4 | 1.34 | 1.70E-05 | 0.85 | 3.03E-03 | 1.47 | 1.79E-06 |
| D1086.11 | 1.33 | 4.56E-04 | 2.20 | 4.43E-11 | 1.57 | 3.30E-05 |
| dim-1 | 1.31 | 8.39E-07 | 0.89 | 3.73E-04 | 1.16 | 1.61E-05 |
| taf-13 | 1.30 | 1.00E-03 | 0.98 | 5.97E-03 | 1.04 | 5.44E-03 |
| col-155 | 1.30 | 1.33E-06 | 0.25 | 3.64E-02 | 0.92 | 4.30E-04 |
| ZK105.1 | 1.28 | 1.40E-03 | 1.17 | 1.62E-03 | 1.88 | 1.08E-06 |
| C15C7.5 | 1.27 | 5.55E-03 | 0.66 | 4.04E-02 | 1.87 | 3.12E-05 |
| aqp-2 | 1.27 | 4.43E-03 | 1.31 | 2.46E-03 | 1.20 | 6.30E-03 |

| | | | | | | |
|---|---|---|---|---|---|---|
| sqt-2 | 1.26 | 3.96E-04 | -0.61 | 3.62E-02 | 1.59 | 4.14E-06 |
| col-154 | 1.26 | 5.54E-06 | 0.03 | 4.88E-02 | 1.03 | 1.69E-04 |
| cpi-2 | 1.24 | 1.71E-05 | -0.54 | 2.95E-02 | 1.25 | 9.20E-06 |
| F43G9.10 | 1.24 | 7.86E-03 | 1.05 | 1.15E-02 | 1.18 | 7.12E-03 |
| F49C12.11 | 1.23 | 2.35E-05 | -0.07 | 5.18E-02 | 1.49 | 1.25E-07 |
| T26E3.4 | 1.22 | 9.91E-03 | 0.84 | 3.38E-02 | 1.07 | 1.93E-02 |
| E01A2.4 | 1.22 | 9.97E-03 | 1.10 | 9.14E-03 | 0.73 | 4.83E-02 |
| Y39A1A.7 | 1.21 | 7.16E-03 | 0.64 | 4.61E-02 | 1.74 | 8.73E-05 |
| ppn-1 | 1.21 | 4.77E-04 | 1.43 | 1.71E-05 | 1.20 | 3.91E-04 |
| col-144 | 1.21 | 2.59E-16 | -0.57 | 3.49E-04 | 0.78 | 1.58E-07 |
| cct-7 | 1.21 | 1.94E-05 | 0.87 | 6.19E-04 | 0.69 | 5.76E-03 |
| Y59A8A.3 | 1.20 | 7.06E-05 | 0.93 | 9.53E-04 | 0.74 | 6.83E-03 |
| F10E9.4 | 1.19 | 7.69E-03 | 0.46 | 6.16E-02 | 0.99 | 2.13E-02 |
| col-107 | 1.18 | 1.53E-04 | -0.79 | 2.01E-02 | 0.95 | 1.46E-03 |
| dlc-1 | 1.17 | 1.67E-12 | 0.20 | 1.72E-02 | 1.25 | 1.88E-14 |
| E02H1.8 | 1.17 | 1.24E-03 | -0.22 | 7.99E-02 | 0.93 | 6.27E-03 |
| acdh-1 | 1.16 | 2.33E-06 | 2.56 | 9.38E-42 | 2.98 | 4.50E-60 |
| cpr-1 | 1.16 | 1.54E-03 | 2.02 | 3.71E-10 | 1.97 | 5.86E-09 |
| nspc-9 | 1.16 | 6.98E-03 | -1.22 | 4.22E-02 | 1.53 | 4.77E-04 |
| dod-6 | 1.16 | 8.24E-04 | 0.23 | 5.80E-02 | 1.50 | 7.22E-06 |
| K08D12.3 | 1.16 | 2.40E-09 | 0.85 | 5.22E-06 | 0.82 | 2.03E-05 |
| snr-4 | 1.15 | 3.56E-06 | 0.57 | 5.31E-03 | 0.94 | 1.47E-04 |
| ttr-18 | 1.14 | 1.06E-04 | 0.19 | 4.87E-02 | 1.73 | 9.41E-10 |
| aex-5 | 1.13 | 2.16E-06 | 1.10 | 9.15E-07 | 0.94 | 6.72E-05 |
| trap-3 | 1.12 | 1.88E-07 | 0.32 | 1.65E-02 | 0.99 | 3.09E-06 |
| col-169 | 1.12 | 1.98E-08 | -0.36 | 1.28E-02 | 0.79 | 5.28E-05 |
| trap-2 | 1.11 | 1.40E-05 | 0.54 | 7.17E-03 | 1.38 | 1.67E-08 |
| cgh-1 | 1.11 | 4.51E-11 | 1.26 | 1.43E-15 | 0.76 | 6.39E-06 |
| zip-2 | 1.10 | 6.73E-03 | 1.06 | 5.93E-03 | 1.92 | 2.17E-06 |
| F09G8.7 | 1.10 | 2.68E-03 | 0.09 | 7.22E-02 | 1.22 | 5.77E-04 |
| sdhd-1 | 1.10 | 5.71E-04 | 0.52 | 2.14E-02 | 0.91 | 3.04E-03 |
| ftn-2 | 1.09 | 5.41E-06 | 0.46 | 9.49E-03 | 1.32 | 1.41E-08 |
| F58F12.1 | 1.09 | 2.12E-09 | 0.54 | 6.76E-04 | 0.70 | 7.06E-05 |
| T21C9.4 | 1.09 | 1.07E-04 | 0.19 | 4.50E-02 | 0.57 | 1.32E-02 |
| arf-1.2 | 1.08 | 2.61E-06 | 1.00 | 3.46E-06 | 1.17 | 2.51E-07 |
| rab-11.1 | 1.08 | 2.37E-07 | 0.81 | 3.75E-05 | 1.03 | 6.43E-07 |
| col-166 | 1.08 | 2.77E-09 | -0.23 | 1.76E-02 | 0.77 | 1.72E-05 |
| col-10 | 1.07 | 3.36E-14 | -0.63 | 7.50E-05 | 0.71 | 5.22E-07 |
| msp-32 | 1.06 | 2.51E-04 | -0.58 | 2.25E-02 | 1.61 | 3.81E-09 |
| F09E5.3 | 1.06 | 5.08E-03 | 0.61 | 2.96E-02 | 1.19 | 2.31E-03 |
| dnj-12 | 1.06 | 2.37E-03 | 1.10 | 1.05E-03 | 1.14 | 8.26E-04 |
| rsp-3 | 1.06 | 1.71E-04 | 0.91 | 4.10E-04 | 0.46 | 2.20E-02 |
| glh-1 | 1.05 | 2.03E-03 | 1.27 | 7.27E-05 | 1.03 | 1.71E-03 |
| col-167 | 1.05 | 5.77E-09 | -0.37 | 8.32E-03 | 0.73 | 3.36E-05 |
| ZK1307.8 | 1.04 | 7.79E-03 | 0.80 | 1.36E-02 | 1.26 | 1.66E-03 |
| tag-170 | 1.03 | 8.55E-03 | 0.29 | 6.95E-02 | 1.34 | 1.01E-03 |
| F59E10.3 | 1.03 | 2.22E-03 | 0.38 | 4.44E-02 | 1.30 | 1.41E-04 |

| | | | | | | |
|---|---|---|---|---|---|---|
| rpl-24.2 | 1.03 | 4.59E-03 | 0.49 | 4.08E-02 | 0.83 | 1.32E-02 |
| Y66H1B.2 | 1.03 | 9.51E-05 | 1.15 | 3.90E-06 | 0.94 | 2.43E-04 |
| pfd-3 | 1.03 | 2.53E-03 | 0.75 | 1.23E-02 | 0.46 | 4.17E-02 |
| MTCE.35 | 1.02 | 1.79E-06 | 1.50 | 8.90E-15 | 1.49 | 1.43E-13 |
| ost-1 | 1.02 | 4.68E-06 | 0.84 | 7.56E-05 | 1.47 | 1.00E-11 |
| F47G9.1 | 1.02 | 5.03E-04 | 0.75 | 4.09E-03 | 1.15 | 1.09E-04 |
| arx-6 | 1.02 | 7.65E-04 | 0.87 | 1.83E-03 | 0.88 | 2.15E-03 |
| F26B1.2 | 1.02 | 3.15E-03 | 1.13 | 9.31E-04 | 0.88 | 6.89E-03 |
| ifb-1 | 1.01 | 1.39E-03 | 1.30 | 1.22E-05 | 1.40 | 5.87E-06 |
| R10H10.3 | 1.01 | 6.45E-03 | 0.41 | 4.95E-02 | 1.22 | 1.39E-03 |
| unc-15 | 1.01 | 3.33E-08 | 1.05 | 8.77E-10 | 1.00 | 2.50E-08 |
| H36L18.2 | 1.00 | 1.56E-03 | 0.10 | 5.66E-02 | 1.52 | 1.55E-06 |
| clic-1 | 1.00 | 3.91E-04 | 0.82 | 1.42E-03 | 1.07 | 1.03E-04 |
| tram-1 | 1.00 | 3.78E-04 | 1.01 | 1.60E-04 | 1.03 | 2.01E-04 |
| lev-11 | 1.00 | 1.98E-07 | 0.98 | 9.25E-08 | 0.96 | 4.46E-07 |
| col-168 | 1.00 | 4.04E-08 | -0.43 | 8.06E-03 | 0.67 | 1.24E-04 |
| dpy-11 | 0.99 | 4.64E-03 | 0.74 | 1.37E-02 | 1.23 | 7.08E-04 |
| ZK418.5 | 0.99 | 5.92E-03 | 0.48 | 3.97E-02 | 1.18 | 1.26E-03 |
| F52A8.5 | 0.99 | 6.30E-03 | 0.17 | 6.80E-02 | 1.16 | 1.44E-03 |
| col-170 | 0.99 | 6.66E-07 | -0.33 | 1.98E-02 | 0.57 | 1.60E-03 |
| rpl-20 | 0.99 | 1.71E-31 | 0.37 | 3.57E-06 | 0.42 | 9.03E-07 |
| alp-1 | 0.98 | 8.90E-03 | 0.64 | 3.20E-02 | 1.15 | 2.68E-03 |
| K12H4.4 | 0.98 | 4.61E-03 | 0.69 | 1.45E-02 | 1.03 | 2.52E-03 |
| F53H4.2 | 0.98 | 8.15E-03 | 1.52 | 3.19E-05 | 0.98 | 7.81E-03 |
| B0334.4 | 0.97 | 1.94E-03 | 0.61 | 1.72E-02 | 0.77 | 8.27E-03 |
| kin-2 | 0.96 | 2.26E-03 | 1.02 | 5.60E-04 | 0.97 | 1.51E-03 |
| unc-54 | 0.96 | 2.23E-07 | 1.08 | 6.78E-10 | 0.92 | 5.51E-07 |
| pas-3 | 0.96 | 4.57E-05 | 0.52 | 5.93E-03 | 0.68 | 1.82E-03 |
| ZC247.1 | 0.95 | 1.67E-03 | 0.99 | 7.24E-04 | 1.43 | 1.92E-06 |
| apl-1 | 0.95 | 7.79E-03 | 1.17 | 9.95E-04 | 1.29 | 7.28E-04 |
| ZC395.10 | 0.95 | 4.71E-05 | 0.97 | 1.32E-05 | 1.16 | 6.47E-07 |
| F44D12.7 | 0.94 | 5.43E-03 | -1.23 | 1.54E-02 | 1.45 | 2.82E-05 |
| T13F2.2 | 0.94 | 9.96E-04 | 0.58 | 1.04E-02 | 0.86 | 1.85E-03 |
| mdh-1 | 0.94 | 1.55E-06 | 0.43 | 4.63E-03 | 0.67 | 3.25E-04 |
| cpl-1 | 0.93 | 1.33E-10 | 1.32 | 6.99E-24 | 1.02 | 7.72E-13 |
| T05H4.6a | 0.93 | 2.82E-03 | 0.89 | 2.21E-03 | 0.89 | 3.36E-03 |
| T09E8.3 | 0.93 | 4.70E-03 | 0.41 | 3.67E-02 | 0.83 | 7.55E-03 |
| vha-4 | 0.92 | 2.69E-04 | 1.19 | 5.07E-07 | 1.23 | 1.00E-06 |
| R155.1 | 0.92 | 8.45E-03 | 0.64 | 2.24E-02 | 0.85 | 9.60E-03 |
| F56A8.3 | 0.92 | 8.07E-03 | 0.64 | 2.05E-02 | 0.72 | 2.04E-02 |
| R09E12.3 | 0.92 | 1.71E-03 | 0.44 | 2.50E-02 | 0.23 | 5.33E-02 |
| clec-1 | 0.91 | 2.88E-03 | 0.67 | 9.68E-03 | 1.27 | 5.71E-05 |
| ccdc-47 | 0.91 | 6.97E-03 | 0.93 | 3.82E-03 | 1.03 | 2.67E-03 |
| Y73B3A.18 | 0.91 | 7.22E-12 | 0.24 | 6.67E-03 | 0.47 | 1.72E-04 |
| vha-8 | 0.90 | 7.58E-06 | 1.10 | 6.67E-09 | 1.22 | 3.28E-10 |
| mlc-4 | 0.90 | 6.67E-04 | 0.65 | 4.84E-03 | 1.16 | 1.38E-05 |
| col-12 | 0.90 | 7.94E-06 | 0.03 | 4.02E-02 | 1.05 | 1.52E-07 |

| | | | | | | |
|---|---|---|---|---|---|---|
| arf-3 | 0.90 | 5.88E-06 | 0.67 | 2.37E-04 | 0.92 | 2.30E-06 |
| tag-18 | 0.90 | 3.16E-03 | 0.71 | 7.29E-03 | 0.83 | 5.01E-03 |
| C28H8.4 | 0.90 | 2.58E-03 | 0.45 | 2.58E-02 | 0.83 | 4.23E-03 |
| kin-19 | 0.90 | 6.51E-05 | 0.86 | 6.17E-05 | 0.74 | 6.35E-04 |
| mca-3 | 0.90 | 8.91E-03 | 1.01 | 2.01E-03 | 0.73 | 1.77E-02 |
| F57B9.3 | 0.90 | 3.34E-03 | 0.72 | 8.05E-03 | 0.43 | 3.68E-02 |
| ctb-1 | 0.89 | 1.28E-25 | 0.44 | 5.07E-08 | 0.69 | 5.57E-16 |
| vig-1 | 0.89 | 1.09E-12 | 0.96 | 2.23E-16 | 0.57 | 2.56E-06 |
| cpi-1 | 0.88 | 2.91E-03 | 0.63 | 1.08E-02 | 1.79 | 3.13E-10 |
| T08B2.7 | 0.88 | 7.46E-03 | 0.99 | 2.93E-03 | 1.28 | 2.35E-04 |
| col-13 | 0.88 | 5.28E-06 | -0.04 | 3.42E-02 | 1.02 | 9.06E-08 |
| erm-1 | 0.88 | 8.15E-04 | 0.93 | 2.35E-04 | 0.81 | 1.26E-03 |
| dao-2 | 0.87 | 7.49E-03 | -1.53 | 1.91E-03 | 1.76 | 7.03E-08 |
| lgg-1 | 0.87 | 1.41E-04 | 0.98 | 9.39E-06 | 1.66 | 1.09E-14 |
| iftb-1 | 0.87 | 2.87E-03 | 0.78 | 3.46E-03 | 1.21 | 5.85E-05 |
| T27F7.3 | 0.87 | 7.98E-05 | 0.57 | 2.75E-03 | 1.06 | 2.10E-06 |
| T07A9.9 | 0.87 | 6.54E-03 | 0.97 | 2.30E-03 | 0.94 | 3.73E-03 |
| K09G1.1 | 0.86 | 5.60E-03 | 0.93 | 2.45E-03 | 1.79 | 9.57E-09 |
| F57B10.5 | 0.86 | 8.29E-03 | 0.33 | 4.97E-02 | 1.10 | 1.45E-03 |
| W10D9.5 | 0.86 | 9.04E-03 | 0.03 | 6.53E-02 | 0.98 | 3.41E-03 |
| Y71F9AL.9 | 0.86 | 1.85E-04 | 0.88 | 6.81E-05 | 0.53 | 6.47E-03 |
| trs-1 | 0.86 | 4.32E-03 | 1.00 | 6.13E-04 | 0.51 | 2.64E-02 |
| col-180 | 0.85 | 4.70E-03 | -0.67 | 2.95E-02 | 0.80 | 5.60E-03 |
| T27F7.1 | 0.85 | 9.72E-03 | 0.67 | 1.48E-02 | 0.79 | 1.06E-02 |
| hsp-6 | 0.85 | 3.82E-04 | 0.94 | 3.47E-05 | 0.73 | 1.44E-03 |
| pabp-2 | 0.85 | 5.06E-03 | 0.48 | 2.88E-02 | 0.52 | 2.83E-02 |
| cey-4 | 0.85 | 2.29E-06 | 0.47 | 1.55E-03 | 0.47 | 2.42E-03 |
| K10C2.3 | 0.85 | 2.71E-04 | -0.96 | 1.47E-03 | -1.14 | 6.43E-04 |
| vha-2 | 0.84 | 4.21E-08 | 0.94 | 1.05E-10 | 1.10 | 2.03E-13 |
| rpt-4 | 0.84 | 5.02E-03 | 0.74 | 6.88E-03 | 1.05 | 7.85E-04 |
| hsp-25 | 0.84 | 5.45E-03 | 0.87 | 2.78E-03 | 1.02 | 1.52E-03 |
| cct-5 | 0.84 | 2.70E-04 | 1.01 | 4.93E-06 | 0.94 | 5.35E-05 |
| F25B5.3 | 0.84 | 4.56E-03 | 0.63 | 1.27E-02 | 0.88 | 2.80E-03 |
| unc-60 | 0.84 | 2.12E-03 | 0.09 | 5.39E-02 | 0.78 | 3.51E-03 |
| C18A3.5 | 0.84 | 5.01E-03 | 0.91 | 1.64E-03 | 0.58 | 2.05E-02 |
| let-2 | 0.83 | 3.83E-03 | 1.03 | 2.78E-04 | 1.00 | 7.01E-04 |
| LLC1.3 | 0.83 | 4.02E-03 | 0.73 | 4.63E-03 | 0.87 | 2.53E-03 |
| ubc-13 | 0.83 | 4.81E-03 | 0.85 | 2.12E-03 | 0.64 | 1.49E-02 |
| F25H2.5 | 0.83 | 6.17E-27 | 0.17 | 1.83E-03 | 0.60 | 2.57E-14 |
| dao-5 | 0.83 | 1.53E-03 | 1.14 | 7.29E-06 | 0.38 | 2.80E-02 |
| C17H12.8 | 0.83 | 5.81E-03 | -1.60 | 1.15E-03 | -0.69 | 2.15E-02 |
| pas-5 | 0.82 | 3.01E-03 | 0.11 | 5.30E-02 | 1.11 | 9.39E-05 |
| elo-5 | 0.82 | 2.14E-04 | 0.77 | 1.97E-04 | 0.72 | 6.36E-04 |
| Y105E8B.11 | 0.82 | 9.89E-41 | 0.75 | 7.33E-38 | 0.72 | 7.04E-32 |
| col-133 | 0.82 | 3.65E-04 | -0.23 | 3.94E-02 | 0.64 | 2.82E-03 |
| praf-3 | 0.82 | 2.30E-03 | 0.51 | 1.47E-02 | 0.56 | 1.33E-02 |
| mdt-28 | 0.81 | 1.51E-03 | 0.99 | 6.42E-05 | 0.89 | 6.17E-04 |

| | | | | | | |
|---|---|---|---|---|---|---|
| C30C11.4 | 0.81 | 3.26E-04 | 0.84 | 7.61E-05 | 0.74 | 6.48E-04 |
| rab-1 | 0.81 | 3.53E-04 | 0.79 | 1.79E-04 | 0.71 | 9.57E-04 |
| gpb-1 | 0.81 | 8.39E-03 | 0.66 | 1.47E-02 | 0.55 | 2.78E-02 |
| C04C3.3 | 0.81 | 2.25E-03 | 0.85 | 7.88E-04 | 0.54 | 1.43E-02 |
| nlp-36 | 0.81 | 1.54E-05 | -1.66 | 2.17E-08 | 0.42 | 5.90E-03 |
| Y57G11C.9 | 0.80 | 6.26E-03 | 0.71 | 8.42E-03 | 0.18 | 5.91E-02 |
| C14C6.5 | 0.79 | 8.50E-03 | 0.80 | 6.19E-03 | 1.23 | 1.28E-04 |
| eif-3.F | 0.79 | 7.03E-03 | 0.72 | 7.61E-03 | 0.95 | 1.80E-03 |
| phb-2 | 0.79 | 1.18E-03 | 0.84 | 3.44E-04 | 0.67 | 3.56E-03 |
| mev-1 | 0.79 | 2.31E-03 | 0.25 | 3.79E-02 | 0.66 | 6.45E-03 |
| tnt-2 | 0.78 | 9.83E-05 | 1.00 | 2.04E-07 | 0.69 | 3.80E-04 |
| Y54G2A.18 | 0.78 | 5.00E-03 | 0.37 | 3.11E-02 | 0.69 | 8.25E-03 |
| nduf-7 | 0.78 | 3.19E-03 | 0.71 | 3.42E-03 | 0.68 | 6.76E-03 |
| nol-5 | 0.78 | 7.18E-04 | 0.74 | 6.44E-04 | 0.57 | 5.20E-03 |
| K01G5.5 | 0.78 | 5.60E-04 | 0.56 | 3.47E-03 | 0.48 | 9.79E-03 |
| ubc-20 | 0.78 | 3.53E-03 | 0.52 | 1.34E-02 | 0.35 | 3.41E-02 |
| vha-15 | 0.77 | 3.71E-03 | 1.05 | 6.55E-05 | 1.13 | 6.01E-05 |
| Y113G7B.17 | 0.77 | 1.45E-05 | 0.72 | 1.49E-05 | 0.28 | 1.49E-02 |
| skr-1 | 0.76 | 2.83E-03 | 0.69 | 3.59E-03 | 0.98 | 2.47E-04 |
| W01A8.1b | 0.76 | 1.95E-03 | 0.95 | 1.25E-04 | 0.88 | 4.89E-04 |
| rab-5 | 0.76 | 5.41E-03 | 0.88 | 1.13E-03 | 0.84 | 2.75E-03 |
| hsp-3 | 0.76 | 1.29E-04 | 0.56 | 1.31E-03 | 0.54 | 2.58E-03 |
| atp-2 | 0.76 | 1.58E-13 | 0.76 | 3.87E-15 | 0.53 | 1.36E-07 |
| ile-1 | 0.76 | 5.35E-03 | 0.66 | 7.45E-03 | 0.46 | 2.65E-02 |
| tsn-1 | 0.76 | 1.84E-04 | 0.76 | 8.50E-05 | 0.22 | 2.87E-02 |
| tct-1 | 0.75 | 6.23E-12 | 0.58 | 1.85E-08 | 0.96 | 7.25E-20 |
| F58E10.3 | 0.75 | 6.69E-03 | 1.12 | 9.69E-05 | 0.90 | 1.86E-03 |
| R06C1.4 | 0.75 | 3.98E-05 | -0.51 | 4.96E-03 | 0.74 | 3.47E-05 |
| F59C6.5 | 0.74 | 1.58E-03 | 0.23 | 3.21E-02 | 1.32 | 5.00E-08 |
| nuo-4 | 0.74 | 7.16E-03 | 0.73 | 5.56E-03 | 1.15 | 1.16E-04 |
| vha-1 | 0.74 | 5.73E-03 | 0.54 | 1.49E-02 | 0.94 | 9.10E-04 |
| eft-4 | 0.74 | 1.40E-13 | 0.55 | 5.15E-09 | 0.66 | 1.72E-11 |
| rpl-7A | 0.74 | 1.03E-16 | 0.35 | 1.14E-05 | 0.35 | 1.92E-05 |
| K07C5.4 | 0.74 | 4.04E-05 | 0.80 | 2.72E-06 | 0.16 | 2.92E-02 |
| unc-116 | 0.73 | 8.63E-03 | 0.73 | 6.56E-03 | 0.63 | 1.41E-02 |
| W02F12.5 | 0.73 | 2.84E-03 | 0.76 | 1.31E-03 | 0.62 | 5.77E-03 |
| W06A7.4 | 0.73 | 3.27E-03 | 1.09 | 1.40E-05 | 0.56 | 1.11E-02 |
| C44B7.10 | 0.72 | 4.65E-03 | 0.72 | 3.22E-03 | 1.23 | 6.91E-06 |
| cmd-1 | 0.72 | 4.41E-04 | 0.98 | 1.15E-06 | 0.57 | 2.61E-03 |
| F10E7.5 | 0.72 | 6.11E-03 | 0.74 | 3.48E-03 | 0.44 | 2.56E-02 |
| col-81 | 0.72 | 4.88E-05 | 0.30 | 9.81E-03 | 0.42 | 3.81E-03 |
| pmt-1 | 0.71 | 8.74E-03 | 1.09 | 1.23E-04 | 1.48 | 4.77E-07 |
| H28O16.1 | 0.71 | 1.31E-08 | 0.57 | 9.56E-07 | 0.50 | 2.17E-05 |
| sgt-1 | 0.71 | 3.31E-03 | 0.67 | 3.43E-03 | 0.32 | 3.45E-02 |
| C18E9.4 | -0.71 | 6.43E-04 | -1.40 | 3.43E-08 | -0.43 | 9.82E-03 |
| Y82E9BR.3 | -0.71 | 3.08E-19 | -1.30 | 5.14E-55 | -0.84 | 6.27E-26 |
| rpl-33 | -0.71 | 2.77E-25 | -1.85 | 1.97E-118 | -1.26 | 4.73E-62 |

| | | | | | | |
|---|---|---|---|---|---|---|
| rps-5 | -0.72 | 5.40E-17 | -0.70 | 2.68E-18 | -0.72 | 1.84E-17 |
| tomm-7 | -0.72 | 3.59E-04 | -1.78 | 3.18E-11 | -0.94 | 2.43E-05 |
| Y37E3.8 | -0.74 | 1.20E-19 | -1.10 | 1.45E-40 | -0.70 | 1.77E-18 |
| rps-26 | -0.75 | 1.26E-28 | -1.62 | 2.90E-105 | -1.11 | 6.93E-55 |
| atp-4 | -0.76 | 1.54E-06 | -1.13 | 1.24E-11 | -0.86 | 1.23E-07 |
| rrn-1.1 | -0.77 | 1.24E-38 | 1.05 | 2.42E-149 | -1.34 | 1.38E-93 |
| rrn-1.2 | -0.77 | 1.24E-38 | 1.05 | 2.42E-149 | -1.34 | 1.38E-93 |
| rpb-12 | -0.77 | 1.90E-04 | -1.76 | 4.81E-11 | -1.38 | 1.03E-07 |
| K07F5.9 | -0.78 | 6.38E-03 | -2.41 | 2.22E-07 | -0.33 | 4.27E-02 |
| F25H2.4 | -0.78 | 9.45E-03 | -0.84 | 4.28E-03 | -0.51 | 3.30E-02 |
| rpl-25.1 | -0.78 | 4.27E-08 | -1.66 | 4.55E-24 | -0.95 | 1.73E-10 |
| rpl-43 | -0.78 | 3.26E-25 | -1.92 | 5.01E-108 | -0.96 | 1.90E-36 |
| Y69A2AR.3 | -0.79 | 7.49E-03 | -1.45 | 7.98E-05 | -0.62 | 1.84E-02 |
| rps-19 | -0.79 | 5.64E-26 | -1.15 | 8.96E-53 | -0.71 | 7.15E-23 |
| K12H4.5 | -0.79 | 1.16E-04 | -1.63 | 1.22E-11 | -0.75 | 8.99E-05 |
| Y119D3B.21 | -0.79 | 8.72E-18 | -2.39 | 2.28E-93 | -1.96 | 2.88E-66 |
| rpl-35 | -0.80 | 6.49E-20 | -1.40 | 1.05E-51 | -0.98 | 8.35E-28 |
| mlc-2 | -0.81 | 1.64E-08 | -0.20 | 5.40E-03 | -0.48 | 5.21E-05 |
| C28C12.2 | -0.81 | 1.33E-04 | -0.85 | 7.04E-05 | -0.99 | 2.63E-05 |
| ife-3 | -0.81 | 6.85E-04 | 0.20 | 2.21E-02 | -1.47 | 8.01E-07 |
| nspa-5 | -0.82 | 2.98E-03 | -1.28 | 1.94E-04 | 0.34 | 2.16E-02 |
| tin-9.1 | -0.82 | 4.58E-03 | -1.79 | 1.16E-05 | -0.20 | 3.78E-02 |
| T23F2.5 | -0.82 | 3.77E-04 | -1.22 | 1.22E-06 | -0.36 | 8.96E-03 |
| C45B2.1 | -0.83 | 9.19E-05 | -1.53 | 2.96E-10 | -0.26 | 1.31E-02 |
| C33A12.1 | -0.83 | 2.06E-03 | -0.96 | 1.08E-03 | -0.48 | 1.35E-02 |
| R07E5.13 | -0.83 | 3.50E-03 | -0.90 | 1.10E-03 | -0.61 | 8.85E-03 |
| ilys-5 | -0.84 | 1.01E-05 | -1.51 | 7.90E-13 | -0.73 | 2.26E-05 |
| rpl-22 | -0.84 | 6.68E-24 | -1.28 | 1.25E-50 | -0.86 | 2.30E-25 |
| rps-12 | -0.84 | 1.16E-30 | -1.27 | 4.48E-66 | -0.86 | 4.56E-33 |
| iff-1 | -0.84 | 5.16E-22 | -1.77 | 5.41E-73 | -1.44 | 1.51E-49 |
| F44E5.1 | -0.86 | 6.77E-12 | -1.89 | 8.42E-39 | -1.38 | 3.26E-23 |
| K10B2.4 | -0.87 | 1.97E-03 | -1.39 | 1.54E-05 | -1.02 | 6.63E-04 |
| rps-29 | -0.87 | 1.08E-30 | -1.86 | 3.15E-105 | -1.14 | 2.17E-47 |
| C08F8.9 | -0.88 | 9.30E-04 | -1.30 | 7.23E-06 | -0.69 | 2.01E-03 |
| C48B6.3 | -0.88 | 6.21E-03 | 0.21 | 3.65E-02 | -1.28 | 1.06E-03 |
| ZK813.2 | -0.89 | 1.77E-06 | -0.75 | 3.50E-06 | -0.58 | 2.96E-04 |
| rps-28 | -0.89 | 5.68E-53 | -1.96 | 2.14E-194 | -1.06 | 4.40E-72 |
| C49F5.7.2 | -0.89 | 2.57E-03 | -1.61 | 2.01E-05 | -1.11 | 4.47E-04 |
| C49F5.7.1 | -0.90 | 3.68E-03 | -1.60 | 2.71E-05 | -1.11 | 5.78E-04 |
| K10D2.4 | -0.90 | 2.45E-03 | -2.07 | 2.46E-07 | -1.40 | 1.04E-04 |
| F29B9.11 | -0.91 | 1.34E-12 | -2.07 | 2.70E-43 | -1.20 | 3.90E-19 |
| lec-6 | -0.92 | 2.57E-07 | -1.26 | 2.23E-12 | -0.37 | 2.71E-03 |
| rps-30 | -0.93 | 5.11E-27 | -1.26 | 4.52E-48 | -0.99 | 6.11E-30 |
| Y55B1AL.2 | -0.93 | 9.42E-04 | -1.18 | 3.43E-05 | -1.00 | 3.55E-04 |
| MTCE.4 | -0.94 | 4.36E-53 | -1.24 | 1.78E-91 | -2.66 | 3.34E-227 |
| mai-2 | -0.96 | 2.49E-05 | -1.03 | 2.92E-06 | -0.43 | 5.41E-03 |
| rpl-36 | -0.96 | 7.73E-49 | -1.74 | 4.56E-135 | -1.22 | 4.91E-73 |

| | | | | | | |
|---|---|---|---|---|---|---|
| his-48 | -0.97 | 6.29E-03 | -1.14 | 1.15E-03 | -1.43 | 7.39E-04 |
| F44E2.9 | -0.98 | 8.94E-03 | -1.24 | 3.95E-03 | -0.90 | 2.20E-02 |
| F53A3.3 | -0.98 | 1.76E-36 | -1.75 | 6.96E-97 | -0.97 | 1.36E-36 |
| Y59A8B.12 | -0.98 | 2.19E-03 | -1.69 | 3.21E-06 | -1.29 | 2.15E-04 |
| F58A4.2 | -0.98 | 7.86E-03 | -0.67 | 1.57E-02 | -1.34 | 2.05E-03 |
| T14B4.2 | -0.98 | 4.53E-03 | -0.93 | 3.93E-03 | -1.36 | 1.24E-03 |
| rpl-41 | -0.98 | 1.61E-68 | -1.84 | 1.17E-199 | -1.40 | 4.50E-123 |
| F53F4.16 | -1.00 | 9.49E-04 | -1.35 | 5.61E-05 | -0.82 | 3.07E-03 |
| elb-1 | -1.00 | 8.64E-03 | -1.19 | 2.01E-03 | -1.09 | 5.78E-03 |
| rpl-34 | -1.00 | 7.59E-54 | -2.28 | 1.19E-197 | -1.09 | 2.14E-64 |
| col-95 | -1.01 | 5.39E-04 | 1.30 | 9.64E-11 | 0.26 | 2.55E-02 |
| his-58 | -1.01 | 2.79E-03 | -1.14 | 8.25E-04 | -1.39 | 8.31E-04 |
| cpg-9 | -1.01 | 7.56E-10 | -1.72 | 9.58E-22 | -1.42 | 1.62E-15 |
| oig-2 | -1.02 | 8.06E-04 | -1.22 | 1.62E-04 | -0.75 | 3.36E-03 |
| Y39A3CL.3 | -1.04 | 3.22E-03 | -1.35 | 2.26E-04 | -0.48 | 1.80E-02 |
| W01D2.1 | -1.04 | 2.77E-69 | -2.02 | 1.36E-208 | -1.53 | 2.91E-129 |
| rpb-10 | -1.05 | 5.53E-05 | -1.80 | 3.90E-09 | -1.00 | 9.75E-05 |
| lsm-6 | -1.05 | 1.16E-03 | -1.42 | 1.83E-05 | -1.01 | 5.23E-04 |
| Y44E3A.3 | -1.08 | 5.68E-03 | -1.11 | 7.93E-03 | -0.57 | 2.99E-02 |
| F53A9.8 | -1.09 | 7.76E-03 | -1.82 | 4.09E-04 | 0.82 | 3.57E-03 |
| F53A9.1 | -1.09 | 5.49E-03 | -2.38 | 4.89E-05 | 0.14 | 5.36E-02 |
| ttr-4 | -1.09 | 5.35E-03 | -0.82 | 1.27E-02 | -0.76 | 2.01E-02 |
| B0205.12 | -1.09 | 2.99E-03 | -1.63 | 6.10E-05 | -1.07 | 2.54E-03 |
| rpl-38 | -1.10 | 1.97E-79 | -1.99 | 5.25E-212 | -0.90 | 2.38E-59 |
| lbp-6 | -1.11 | 1.39E-10 | -1.19 | 5.40E-13 | -0.25 | 5.97E-03 |
| ttr-45 | -1.11 | 2.33E-04 | -0.72 | 2.45E-03 | -0.39 | 1.56E-02 |
| ZC373.2 | -1.11 | 1.05E-10 | -0.65 | 2.69E-06 | -0.80 | 2.20E-07 |
| F22D6.14 | -1.11 | 4.83E-03 | -1.00 | 7.20E-03 | -1.27 | 4.57E-03 |
| Y45F10C.4 | -1.12 | 1.06E-05 | -0.87 | 6.71E-05 | -0.62 | 1.17E-03 |
| Y69A2AR.28 | -1.12 | 4.52E-03 | -1.31 | 1.02E-03 | -1.27 | 3.72E-03 |
| rps-11 | -1.13 | 2.36E-74 | -1.41 | 7.94E-119 | -1.15 | 1.17E-79 |
| rpb-11 | -1.14 | 6.96E-05 | -1.65 | 7.24E-08 | -1.78 | 2.14E-08 |
| C35B1.4 | -1.16 | 1.58E-06 | -0.98 | 4.81E-06 | -1.49 | 5.04E-09 |
| F29C4.2 | -1.17 | 6.48E-07 | -1.56 | 8.49E-11 | -1.03 | 1.83E-06 |
| F23F1.10 | -1.17 | 1.71E-03 | -1.18 | 9.40E-04 | -1.20 | 2.43E-03 |
| Y37D8A.19 | -1.18 | 6.80E-10 | -0.72 | 2.42E-06 | -0.50 | 3.75E-04 |
| pfd-6 | -1.19 | 5.88E-05 | -0.76 | 9.69E-04 | -1.05 | 2.08E-04 |
| gst-27 | -1.21 | 2.42E-04 | -1.07 | 3.45E-04 | -0.41 | 1.90E-02 |
| mxl-1 | -1.21 | 7.61E-04 | -1.26 | 2.19E-04 | -1.26 | 3.08E-04 |
| rpl-26 | -1.22 | 1.75E-63 | -1.57 | 3.76E-105 | -1.44 | 2.18E-84 |
| mtl-2 | -1.23 | 1.07E-03 | -1.45 | 1.16E-04 | -0.94 | 3.69E-03 |
| spp-3 | -1.23 | 3.07E-18 | -1.65 | 2.18E-30 | -1.07 | 1.73E-15 |
| spp-23 | -1.24 | 2.75E-08 | -2.31 | 3.61E-18 | -1.39 | 2.91E-09 |
| cpg-8 | -1.25 | 1.11E-05 | -1.01 | 4.51E-05 | -1.32 | 1.17E-05 |
| R102.2 | -1.26 | 6.52E-03 | -2.55 | 2.65E-05 | -1.17 | 7.18E-03 |
| vha-3 | -1.27 | 2.46E-05 | -0.63 | 4.12E-03 | -1.25 | 4.15E-05 |
| F23D12.7 | -1.28 | 1.85E-05 | -1.59 | 1.72E-07 | -1.16 | 3.45E-05 |

| | | | | | | |
|---|---|---|---|---|---|---|
| K01H12.1 | -1.28 | 9.68E-04 | -1.58 | 5.89E-05 | -1.29 | 1.16E-03 |
| phf-5 | -1.28 | 8.21E-03 | -1.27 | 8.34E-03 | -1.53 | 9.45E-03 |
| F23D12.1 | -1.29 | 1.73E-03 | -2.41 | 5.40E-06 | -1.50 | 4.40E-04 |
| K11H3.6 | -1.30 | 1.03E-06 | -1.70 | 2.52E-10 | -1.86 | 4.64E-10 |
| spp-14 | -1.31 | 2.57E-19 | -2.02 | 8.68E-39 | -0.97 | 2.86E-13 |
| W03G9.8 | -1.31 | 8.86E-04 | -1.04 | 3.82E-03 | -1.18 | 2.15E-03 |
| spp-5 | -1.31 | 1.51E-46 | -1.90 | 8.53E-90 | -1.26 | 8.45E-46 |
| C53H9.3 | -1.31 | 1.54E-04 | -1.84 | 9.05E-07 | -1.39 | 6.47E-05 |
| Y105E8A.11 | -1.31 | 2.37E-03 | -1.77 | 1.04E-04 | -1.72 | 3.34E-04 |
| Y110A2AM.4 | -1.31 | 5.33E-03 | -1.26 | 4.60E-03 | -2.05 | 9.25E-04 |
| MTCE.15 | -1.32 | 1.18E-03 | -0.34 | 3.41E-02 | 0.35 | 3.44E-02 |
| M02H5.8 | -1.33 | 2.10E-03 | -3.19 | 1.77E-06 | -2.29 | 1.92E-04 |
| C17E7.12 | -1.34 | 7.25E-03 | -0.93 | 9.35E-03 | -2.04 | 2.79E-04 |
| aps-3 | -1.36 | 9.63E-04 | -0.05 | 5.37E-02 | -0.85 | 8.76E-03 |
| W02D9.7 | -1.36 | 1.45E-07 | -1.85 | 2.33E-12 | -1.07 | 3.98E-06 |
| Y63D3A.7 | -1.36 | 1.46E-04 | -1.58 | 9.87E-06 | -1.17 | 4.18E-04 |
| rps-24 | -1.36 | 6.06E-110 | -1.58 | 5.57E-158 | -1.34 | 8.70E-112 |
| spp-17 | -1.36 | 3.41E-29 | -2.21 | 5.76E-63 | -1.61 | 2.31E-38 |
| clec-85 | -1.37 | 2.85E-03 | -0.78 | 8.44E-03 | -0.84 | 8.74E-03 |
| cyc-2.1 | -1.37 | 3.18E-31 | -1.56 | 1.90E-42 | -1.02 | 4.06E-21 |
| C50H11.8 | -1.38 | 5.06E-03 | 0.00 | 6.04E-02 | -1.31 | 6.31E-03 |
| msp-74 | -1.39 | 3.19E-05 | -2.46 | 5.13E-10 | -0.83 | 1.71E-03 |
| lsm-5 | -1.39 | 1.01E-04 | -1.72 | 3.67E-06 | -1.22 | 4.01E-04 |
| Y53F4B.14 | -1.40 | 3.97E-03 | -1.54 | 1.71E-03 | -1.29 | 5.29E-03 |
| C14C11.7 | -1.42 | 7.38E-04 | -1.70 | 3.68E-05 | -1.67 | 2.01E-04 |
| ZK686.1 | -1.43 | 2.01E-07 | -1.20 | 3.20E-07 | -1.48 | 2.32E-08 |
| C37A2.7 | -1.45 | 5.00E-82 | -2.15 | 1.27E-161 | -1.56 | 5.35E-96 |
| W02D9.6 | -1.48 | 8.22E-04 | -1.50 | 2.97E-04 | -2.35 | 9.56E-06 |
| Y65B4A.6 | -1.49 | 1.72E-03 | 0.26 | 4.33E-02 | -1.17 | 9.78E-03 |
| ttr-24 | -1.53 | 4.30E-05 | -1.04 | 3.39E-04 | -0.42 | 2.68E-02 |
| dyrb-1 | -1.54 | 1.06E-05 | -1.11 | 1.49E-04 | -1.41 | 5.41E-05 |
| gut-2 | -1.54 | 2.41E-07 | -1.67 | 3.76E-09 | -1.92 | 4.03E-10 |
| elc-1 | -1.55 | 2.60E-03 | -0.72 | 2.70E-02 | -1.43 | 4.24E-03 |
| F33G12.7 | -1.58 | 7.68E-04 | -1.51 | 1.69E-04 | -1.25 | 2.95E-03 |
| W08D2.9 | -1.59 | 5.36E-14 | -2.47 | 2.92E-26 | -1.03 | 2.19E-08 |
| rps-21 | -1.59 | 9.39E-91 | -2.14 | 1.66E-154 | -1.93 | 1.48E-121 |
| Y60A3A.21 | -1.60 | 7.78E-03 | -1.33 | 8.03E-03 | 0.00 | 7.49E-02 |
| ncbp-2 | -1.61 | 7.16E-03 | -0.09 | 6.50E-02 | -0.73 | 2.62E-02 |
| F09E10.1 | -1.61 | 8.28E-03 | -1.41 | 1.01E-02 | -1.07 | 1.80E-02 |
| rab-18 | -1.64 | 1.02E-04 | -0.98 | 1.36E-03 | -2.02 | 7.86E-06 |
| nduf-5 | -1.66 | 2.76E-15 | -1.58 | 4.45E-16 | -1.22 | 7.21E-11 |
| his-68 | -1.67 | 9.54E-04 | -1.53 | 3.38E-04 | -2.21 | 7.90E-05 |
| acbp-1 | -1.70 | 1.31E-28 | -2.21 | 4.81E-45 | -1.66 | 2.60E-28 |
| lin-40 | -1.71 | 3.40E-07 | 0.50 | 2.47E-03 | -0.86 | 9.20E-04 |
| F20C5.3 | -1.71 | 4.08E-03 | -0.46 | 3.82E-02 | -1.56 | 6.30E-03 |
| B0495.6 | -1.71 | 2.85E-08 | -1.97 | 1.59E-10 | -1.66 | 3.97E-08 |
| F26E4.6 | -1.71 | 2.31E-28 | -1.70 | 8.66E-32 | -2.00 | 9.81E-36 |

| | | | | | | |
|---|---|---|---|---|---|---|
| Y105C5A.12 | -1.71 | 1.07E-03 | -4.57 | 1.06E-07 | -3.47 | 6.33E-06 |
| Y105C5A.13 | -1.71 | 1.07E-03 | -4.57 | 1.06E-07 | -3.47 | 6.33E-06 |
| C08E3.1 | -1.71 | 1.07E-05 | -4.77 | 5.26E-13 | -3.69 | 1.50E-10 |
| R05H10.5 | -1.72 | 2.39E-03 | -0.70 | 2.99E-02 | -0.96 | 2.63E-02 |
| msp-37 | -1.76 | 2.86E-08 | -2.21 | 3.93E-12 | -1.35 | 8.35E-07 |
| kbp-4 | -1.78 | 3.31E-07 | -1.42 | 1.96E-06 | -1.57 | 8.70E-07 |
| ddp-1 | -1.83 | 1.60E-15 | -2.18 | 9.22E-22 | -1.86 | 1.39E-16 |
| his-47 | -1.88 | 4.09E-04 | -1.36 | 1.96E-03 | -1.73 | 5.22E-04 |
| F43G6.7 | -1.91 | 9.40E-04 | -2.09 | 5.78E-04 | -1.95 | 5.54E-04 |
| Y67D8C.12 | -1.92 | 5.79E-04 | 0.37 | 3.39E-02 | -2.09 | 4.28E-04 |
| C08E3.13 | -1.92 | 1.05E-06 | -4.83 | 7.48E-15 | -3.39 | 2.67E-11 |
| rrn-3.56 | -1.94 | 4.27E-82 | 1.34 | 2.27E-146 | -1.64 | 2.47E-68 |
| Y22D7AR.10 | -1.96 | 1.69E-21 | -1.54 | 1.47E-18 | -1.30 | 7.28E-14 |
| F56H9.2 | -1.96 | 3.93E-24 | -2.34 | 7.82E-35 | -1.64 | 1.66E-20 |
| F35H10.5 | -1.98 | 5.88E-05 | -1.76 | 6.96E-05 | -1.99 | 1.28E-04 |
| Y92H12BL.5 | -2.06 | 4.15E-03 | -0.77 | 3.53E-02 | -1.71 | 8.91E-03 |
| rrn-3.1 | -2.09 | 1.25E-173 | 1.17 | 5.61E-200 | -1.64 | 1.89E-131 |
| ins-27 | -2.14 | 3.04E-04 | -2.72 | 6.80E-06 | -2.18 | 1.48E-04 |
| T24B8.5 | -2.32 | 1.54E-29 | -3.82 | 3.86E-54 | -2.56 | 3.52E-34 |
| T26H5.9 | -2.36 | 8.08E-05 | -1.39 | 1.42E-03 | -0.75 | 1.72E-02 |
| his-67 | -2.36 | 1.50E-03 | -1.18 | 1.08E-02 | -1.95 | 2.31E-03 |
| Y110A2AL.13 | -2.50 | 7.93E-04 | -0.99 | 1.87E-02 | -2.53 | 4.99E-04 |
| nhr-93 | -2.63 | 2.73E-08 | -0.36 | 2.26E-02 | -1.74 | 6.49E-06 |
| F14H3.12 | -2.90 | 9.49E-03 | -1.99 | 8.30E-03 | -1.64 | 5.50E-02 |
| Y48G1BM.7 | -2.92 | 8.72E-03 | -0.43 | 8.39E-02 | -3.01 | 6.76E-03 |
| his-5 | -3.03 | 2.77E-04 | -2.91 | 3.63E-04 | -1.76 | 9.12E-03 |
| F49B2.3 | -3.26 | 2.37E-03 | 0.32 | 6.66E-02 | -3.34 | 1.71E-03 |
| his-18 | -3.28 | 2.58E-05 | -2.26 | 1.14E-04 | -1.75 | 1.31E-03 |
| nhr-37 | -3.56 | 5.10E-04 | -2.59 | 3.27E-04 | -3.65 | 3.41E-04 |
| ZK218.13 | -3.88 | 7.45E-29 | 0.66 | 6.89E-07 | -1.79 | 1.46E-14 |
| F53C11.2 | -4.35 | 1.17E-06 | -2.47 | 4.41E-05 | -4.43 | 5.81E-07 |
| srz-70 | -5.82 | 2.56E-18 | -2.34 | 1.56E-11 | -5.90 | 3.56E-19 |
