# Supplementary material for: Gene expression profiling of oxidative stress response of C. elegans aging defective AMPK mutants using massively parallel transcriptome sequencing
Source: BMC Res Notes. 2011 Feb 8;4:34. doi: 10.1186/1756-0500-4-34 (PMC3045954; doi:10.1186/1756-0500-4-34)
Supplement: Additional file 4 — Supplementary Table S3. GO analysis for up or down regualted genes in stressed wild type relative to unstressed wild type [file 1756-0500-4-34-S4.PDF]

Supplementary Table 3a. GO analysis for up-regualted genes in stressed wild type relative to unstressed wild type

GO	Genes	Pvalue	GO as name
GO:0006811	col-73; col-130; h28o16.1; col-161; mca-3; col-112; col-109; col-155; vha-2; col-14; col-91; col-12; dpy-13; dpy-5; col-77; col-38; bli-1; col-81; col-154; col-145; sqt-1; atp-2; vha-15; vha-8; col-166; ftn-2; vha-11; rol-8; sqt-2; col-176; col-168; col-175; ram-2; col-48; col-49; vha-1; col-180; col-104; col-71; rol-1; f58f12.1; col-63; col-138; col-107; col-169; col-133; col-13; col-170; dpy-4; col-60; col-90; col-167; bli-2; col-10; let-2; col-144; col-120; col-162; col-41; vha-4; col-157	5.55E-41	ion transport;
GO:0010171	mlc-4; apl-1; mlt-8; col-155; unc-54; unc-15; col-180; y66h1b.2; w10d9.5; dlc-1; dpy-13; col-169; f49c12.11; col-170; kin-2; arf-3; col-154; col-167; rpt-4; lev-11; col-166; eif-3.f; t02h6.11; col-168; c30c11.4; ifb-1; col-157; e01a2.4	6.24E-13	body morphogenesis;
GO:0008340	h28o16.1; acdh-1; mca-3; dao-5; ppn-1; cct-5; t27f7.3; hsp-16.49; t27f7.1; rab-1; hsp-16.11; hsp-16.48; hsp-16.2; cgh-1; f59e10.3; t02h6.11; c30c11.4; cpr-1; ifb-1; dod-6; hsp-16.1	5.32E-09	determination of adult life span; multicellular organismal aging;
GO:0006119	h28o16.1; mtce.35; vha-1; atp-2; vha-15; vha-8; vha-11; vha-2; t02h6.11; f58f12.1; vha-4; mev-1	1.19E-07	oxidative phosphorylation;
GO:0009201	h28o16.1; vha-1; atp-2; vha-15; vha-8; vha-11; f58f12.1; vha-2; vha-4; f25h2.5	3.75E-07	ribonucleoside triphosphate biosynthetic process;
GO:0051789	hsp-3; hsp-16.49; hsp-16.11; hsp-16.41; hsp-16.2; hsp-16.1	5.57E-07	response to protein stimulus;
GO:0015985	h28o16.1; vha-1; atp-2; vha-15; vha-8; vha-11; f58f12.1; vha-2; vha-4	1.33E-06	energy coupled proton transport, down electrochemical gradient;
GO:0006754	h28o16.1; vha-1; atp-2; vha-15; vha-8; vha-11; f58f12.1; vha-2; vha-4	1.56E-06	ATP biosynthetic process;
GO:0006732	h28o16.1; vha-1; atp-2; vha-15; vha-8; sdhd-1; vha-11; w02f12.5; f58f12.1; vha-2; vha-4; c44b7.10; mev-1	2.25E-06	coenzyme metabolic process;

Supplementary Table 3b. GO analysis for down-regualted genes in stressed wild type relative to unstressed wild type

GO	Genes	Pvalue	GO as name
GO:0006412	rps-26; rpl-25.1; c37a2.7; rpl-33; rpl-38; rps-30; rpl-34; rps-29; rps-19; t14b4.2; rps-24; rpl-36; k11h3.6; y37e3.8; rpl-41; w01d2.1; rpl-26; rps-12; iff-1; rps-21; rpl-22; rpl-43; ife-3; rps-5; rps-11; rpl-35; rps-28; rps-22	1.92E-22	translation;
GO:0010467	rpl-33; rpl-38; rpl-34; rps-29; rps-19; w01d2.1; rpl-41; y37e3.8; rps-12; rpl-22; rpl-43; rps-5; ife-3; his-5; rpb-10; his-67; rps-22; rps-26; rpl-25.1; his-18; c37a2.7; rpb-11; rpb-12; rps-30; mxl-1; t14b4.2; rps-24; rpl-36; k11h3.6; rab-18; rpl-26; iff-1; rps-21; rps-11; lin-40; rpl-35; nhr-37; rps-28	4.28E-20	gene expression;
GO:0009059	rps-26; rpl-25.1; c37a2.7; rpl-33; rpl-38; rps-30; rpl-34; rps-29; rps-19; t14b4.2; rps-24; rpl-36; k11h3.6; y37e3.8; rpl-41; w01d2.1; rpl-26; rps-12; iff-1; rps-21; rpl-22; rpl-43; ife-3; rps-5; rps-11; rpl-35; rps-28; rps-22	7.96E-20	macromolecule biosynthetic process;
GO:0065003	his-18; iff-1; aps-3; ife-3; his-48; his-5; his-68; his-47; his-58; his-67	2.01E-05	macromolecular complex assembly;
