# Supplementary material for: Gene expression profiling of oxidative stress response of C. elegans aging defective AMPK mutants using massively parallel transcriptome sequencing
Source: BMC Res Notes. 2011 Feb 8;4:34. doi: 10.1186/1756-0500-4-34 (PMC3045954; doi:10.1186/1756-0500-4-34)
Supplement: Additional file 5 — Supplementary Table S4. Genes that were significantly down-regulated in wild type animals under oxidative stress [file 1756-0500-4-34-S5.PDF]

Supplementary Table 4. Genes that were significantly down-regulated in wild type animals under oxidative stress

Gene	Log2 (Stressed N2/Unstressed N2)	p-val
srz-70	-5.82	2.56E-18
F53C11.2	-4.35	1.17E-06
ZK218.13	-3.88	7.45E-29
nhr-37	-3.56	5.10E-04
his-18	-3.28	2.58E-05
F49B2.3	-3.26	2.37E-03
his-5	-3.03	2.77E-04
Y48G1BM.7	-2.92	8.72E-03
F14H3.12	-2.90	9.49E-03
nhr-93	-2.63	2.73E-08
Y110A2AL.13	-2.50	7.93E-04
his-67	-2.36	1.50E-03
T26H5.9	-2.36	8.08E-05
T24B8.5	-2.32	1.54E-29
ins-27	-2.14	3.04E-04
Y105C5A.13	-2.12	6.48E-07
rrn-3.1	-2.09	1.25E-173
Y92H12BL.5	-2.06	4.15E-03
F35H10.5	-1.98	5.88E-05
F56H9.2	-1.96	3.93E-24
Y22D7AR.10	-1.96	1.69E-21
rrn-3.56	-1.94	4.27E-82
C08E3.13	-1.92	1.05E-06
Y67D8C.12	-1.92	5.79E-04
F43G6.7	-1.91	9.40E-04
his-47	-1.88	4.09E-04
ddp-1	-1.83	1.60E-15
kbp-4	-1.78	3.31E-07
msp-37	-1.76	2.86E-08
R05H10.5	-1.72	2.39E-03
B0495.6	-1.71	2.85E-08
C08E3.1	-1.71	1.07E-05
F20C5.3	-1.71	4.08E-03
F26E4.6	-1.71	2.31E-28
lin-40	-1.71	3.40E-07
Y105C5A.12	-1.71	1.07E-03
acbp-1	-1.70	1.31E-28
his-68	-1.67	9.54E-04
nduf-5	-1.66	2.76E-15
rab-18	-1.64	1.02E-04
F09E10.1	-1.61	8.28E-03
ncbp-2	-1.61	7.16E-03
Y60A3A.21	-1.60	7.78E-03
rps-21	-1.59	9.39E-91
W08D2.9	-1.59	5.36E-14
F33G12.7	-1.58	7.68E-04
elc-1	-1.55	2.60E-03
dyrb-1	-1.54	1.06E-05
gut-2	-1.54	2.41E-07
ttr-24	-1.53	4.30E-05

Y65B4A.6	-1.49	1.72E-03
W02D9.6	-1.48	8.22E-04
C37A2.7	-1.45	5.00E-82
ZK686.1	-1.43	2.01E-07
C14C11.7	-1.42	7.38E-04
Y53F4B.14	-1.40	3.97E-03
lsm-5	-1.39	1.01E-04
msp-74	-1.39	3.19E-05
C50H11.8	-1.38	5.06E-03
clec-85	-1.37	2.85E-03
cyc-2.1	-1.37	3.18E-31
aps-3	-1.36	9.63E-04
rps-24	-1.36	6.06E-110
spp-17	-1.36	3.41E-29
W02D9.7	-1.36	1.45E-07
Y63D3A.7	-1.36	1.46E-04
C17E7.12	-1.34	7.25E-03
M02H5.8	-1.33	2.10E-03
MTCE.15	-1.32	1.18E-03
C53H9.3	-1.31	1.54E-04
spp-14	-1.31	2.57E-19
spp-5	-1.31	1.51E-46
W03G9.8	-1.31	8.86E-04
Y105E8A.11	-1.31	2.37E-03
Y110A2AM.4	-1.31	5.33E-03
K11H3.6	-1.30	1.03E-06
F23D12.1	-1.29	1.73E-03
F23D12.7	-1.28	1.85E-05
K01H12.1	-1.28	9.68E-04
phf-5	-1.28	8.21E-03
vha-3	-1.27	2.46E-05
R102.2	-1.26	6.52E-03
cpg-8	-1.25	1.11E-05
spp-23	-1.24	2.75E-08
mtl-2	-1.23	1.07E-03
spp-3	-1.23	3.07E-18
rpl-26	-1.22	1.75E-63
gst-27	-1.21	2.42E-04
mxl-1	-1.21	7.61E-04
F29C4.2	-1.20	1.26E-07
pfd-6	-1.19	5.88E-05
Y37D8A.19	-1.18	6.80E-10
F23F1.10	-1.17	1.71E-03
C35B1.4	-1.16	1.58E-06
rpb-11	-1.14	6.96E-05
rps-11	-1.13	2.36E-74
mai-2	-1.12	1.07E-07
Y45F10C.4	-1.12	1.06E-05
Y69A2AR.28	-1.12	4.52E-03
F22D6.14	-1.11	4.83E-03
lbp-6	-1.11	1.39E-10
ttr-45	-1.11	2.33E-04
ZC373.2	-1.11	1.05E-10
rpl-38	-1.10	1.97E-79
B0205.12	-1.09	2.99E-03

F53A9.1	-1.09	5.49E-03
F53A9.8	-1.09	7.76E-03
ttr-4	-1.09	5.35E-03
Y44E3A.3	-1.08	5.68E-03
lsm-6	-1.05	1.16E-03
rpb-10	-1.05	5.53E-05
W01D2.1	-1.04	2.77E-69
Y39A3CL.3	-1.04	3.22E-03
oig-2	-1.02	8.06E-04
col-95	-1.01	5.39E-04
cpg-9	-1.01	7.56E-10
his-58	-1.01	2.79E-03
elb-1	-1.00	8.64E-03
F53F4.16	-1.00	9.49E-04
rpl-34	-1.00	7.59E-54
F44E2.9	-0.98	8.94E-03
F53A3.3	-0.98	1.76E-36
F58A4.2	-0.98	7.86E-03
rpl-41	-0.98	1.61E-68
T14B4.2	-0.98	4.53E-03
Y59A8B.12	-0.98	2.19E-03
his-48	-0.97	6.29E-03
ife-3	-0.96	1.18E-04
rpl-36	-0.96	7.73E-49
MTCE.4	-0.94	4.36E-53
rps-30	-0.93	5.11E-27
Y55B1AL.2	-0.93	9.42E-04
lec-6	-0.92	2.57E-07
F29B9.11	-0.91	1.34E-12
C49F5.7.1	-0.90	3.68E-03
K10D2.4	-0.90	2.45E-03
C49F5.7.2	-0.89	2.57E-03
mlc-2	-0.89	2.00E-10
rps-28	-0.89	5.68E-53
ZK813.2	-0.89	1.77E-06
C08F8.9	-0.88	9.30E-04
C48B6.3	-0.88	6.21E-03
K10B2.4	-0.87	1.97E-03
rps-29	-0.87	1.08E-30
F44E5.1	-0.86	6.77E-12
iff-1	-0.84	5.16E-22
ilys-5	-0.84	1.01E-05
rpl-22	-0.84	6.68E-24
rps-12	-0.84	1.16E-30
C33A12.1	-0.83	2.06E-03
C45B2.1	-0.83	9.19E-05
R07E5.13	-0.83	3.50E-03
nspa-5	-0.82	2.98E-03
T23F2.5	-0.82	3.77E-04
tin-9.1	-0.82	4.58E-03
C28C12.2	-0.81	1.33E-04
rpl-35	-0.80	6.49E-20
K12H4.5	-0.79	1.16E-04
rps-19	-0.79	5.64E-26
Y119D3B.21	-0.79	8.72E-18

Y69A2AR.3	-0.79	7.49E-03
F25H2.4	-0.78	9.45E-03
K07F5.9	-0.78	6.38E-03
rpl-25.1	-0.78	4.27E-08
rpl-43	-0.78	3.26E-25
rpb-12	-0.77	1.90E-04
rrn-1.1	-0.77	1.24E-38
rrn-1.2	-0.77	1.24E-38
atp-4	-0.76	1.54E-06
rps-26	-0.75	1.26E-28
Y37E3.8	-0.74	1.20E-19
rps-5	-0.72	5.40E-17
tomm-7	-0.72	3.59E-04
C18E9.4	-0.71	6.43E-04
rpl-33	-0.71	2.77E-25
Y82E9BR.3	-0.71	3.08E-19
