# Supplementary material for: Gene expression profiling of oxidative stress response of C. elegans aging defective AMPK mutants using massively parallel transcriptome sequencing
Source: BMC Res Notes. 2011 Feb 8;4:34. doi: 10.1186/1756-0500-4-34 (PMC3045954; doi:10.1186/1756-0500-4-34)
Supplement: Additional file 6 — Supplementary Table S5. Significantly up-regulated genes in stressed aak-2 mutants relative to wild type [file 1756-0500-4-34-S6.PDF]

Supplementary Table 5. Significantly up-regulated genes in stressed aak-2 mutants relative to wild type

Gene	Log2 (Stressed aak-2/Unstressed N2)	p-val
Y38E10A.15	6.09	4.55E-18
hsp-16.11	4.82	6.85E-59
hsp-16.1	4.67	6.41E-71
hsp-16.49	4.46	1.30E-67
hsp-16.48	4.46	1.30E-67
F35B3.4	4.37	2.31E-05
hsp-16.2	4.34	1.20E-17
hsp-70	4.30	2.82E-06
hsp-16.41	4.24	1.37E-30
fat-7	4.19	5.13E-11
ttn-1	4.16	1.52E-04
F26A1.8	4.05	3.50E-04
R02E4.3	3.87	5.68E-10
F22B7.9	3.81	4.50E-05
Y38E10A.14	3.78	1.27E-37
F17C11.11	3.75	3.50E-07
Y19D10A.7	3.73	1.27E-03
F18E3.7	3.69	3.77E-04
F41C3.2	3.66	3.55E-04
F53B2.8	3.57	2.81E-03
F56A4.9	3.57	2.76E-03
qua-1	3.56	1.61E-03
Y11D7A.9	3.56	2.65E-03
lips-10	3.50	9.64E-06
mxl-3	3.48	6.32E-10
F45D3.4a	3.48	1.99E-11
nspa-7	3.46	1.62E-03
nspa-6	3.46	1.62E-03
cyc-2.2	3.43	1.26E-04
lys-6	3.38	3.69E-03
ZK970.7	3.36	3.21E-04
lgc-21	3.36	5.53E-03
F26A1.6	3.30	4.42E-03
F56F10.3	3.29	8.68E-05
Y19D10A.4	3.29	4.09E-03
C01B4.7	3.29	4.09E-03
T28C12.4	3.28	1.17E-03
lys-5	3.27	2.60E-03
clec-190	3.27	3.89E-03
T28F4.6	3.27	1.14E-03
F15B9.8	3.27	3.83E-05
K07B1.8	3.26	6.23E-05

nspa-12	3.24	9.14E-04
DH11.2	3.23	6.66E-04
R11A5.7	3.17	7.55E-04
C10G11.8	3.16	1.92E-03
F32D8.12	3.15	3.40E-04
F45D3.4	3.14	3.46E-09
F44A6.5	3.14	3.84E-04
clec-52	3.12	9.77E-04
C33F10.11	3.11	4.70E-04
W09D6.4	3.11	9.10E-03
F10D2.10	3.09	1.17E-08
pqn-95	3.09	7.46E-05
T06D8.10	3.05	8.95E-03
B0218.2	3.05	9.97E-05
lys-4	3.04	1.13E-21
mlt-8	3.03	2.82E-08
C08F11.13	3.03	7.93E-03
C42D4.3	3.01	2.86E-07
nhr-105	3.01	7.94E-03
ZK593.3	2.98	9.36E-06
acdh-1	2.98	4.50E-60
tag-297	2.98	7.23E-05
F26A3.4	2.97	7.06E-07
ttr-33	2.96	6.16E-03
F46C8.8	2.94	3.71E-03
fip-6	2.92	3.80E-05
F19B2.5	2.92	5.94E-07
unc-23	2.90	1.42E-05
Y44A6C.1	2.90	5.00E-03
nspd-10	2.89	4.42E-05
dao-4	2.88	8.66E-04
T28H10.3	2.87	1.07E-05
R11F4.1	2.86	2.70E-03
nhr-114	2.85	4.11E-03
tyr-2	2.84	4.39E-03
nlp-31	2.84	1.60E-06
col-135	2.83	9.82E-03
cpn-2	2.83	9.59E-03
E01G4.6	2.83	4.71E-08
C45G9.4	2.83	6.35E-03
R02F11.1	2.83	3.20E-03
ZK1025.8	2.82	4.35E-03
ZK1025.2	2.82	4.35E-03
ZK154.1	2.79	1.88E-03
F53B6.4	2.77	3.56E-03
ZK180.6	2.77	3.47E-05
T19C3.2	2.77	1.66E-03

F29C6.1	2.76	3.41E-03
F10D11.6	2.76	3.01E-04
T17H7.7	2.75	8.15E-03
F26G1.5	2.75	2.70E-03
Y102A5C.6	2.73	2.24E-04
F33D4.6	2.73	7.35E-06
ZC395.5	2.72	6.12E-04
nhr-68	2.72	7.65E-05
wrt-1	2.71	5.00E-03
mlt-9	2.71	9.94E-03
C27D6.3	2.71	4.23E-03
ZK84.1	2.70	1.45E-05
H10E21.4	2.69	2.61E-03
F49C12.15	2.69	8.20E-03
col-138	2.68	1.14E-08
T24H10.7c	2.68	9.61E-04
F13H8.5	2.67	4.48E-03
R09E10.6	2.67	5.15E-06
ZK354.3	2.67	7.94E-03
C39E9.8	2.66	4.30E-07
Y94H6A.10	2.65	1.68E-12
C26B9.3	2.64	2.21E-03
C41G7.6	2.62	8.48E-03
lon-8	2.62	2.98E-04
fbxa-72	2.61	2.78E-05
F43H9.4	2.59	4.81E-03
pqn-46	2.58	6.99E-04
K08F8.1	2.56	1.14E-07
F45D3.3	2.56	4.83E-07
bli-2	2.55	1.91E-05
tag-196	2.54	1.10E-03
T21F4.1	2.54	1.59E-05
T19B10.3	2.54	9.75E-04
K05F1.9	2.53	9.58E-03
col-175	2.52	6.23E-09
ttr-14	2.52	1.27E-04
K08F8.1a	2.52	1.84E-06
nspa-3	2.51	7.54E-06
Y19D10A.16	2.49	4.39E-03
C01B4.6	2.49	4.39E-03
col-63	2.48	1.23E-06
C04F12.7	2.48	3.64E-07
C35D10.2	2.47	7.13E-04
C52G5.2	2.47	6.49E-04
W01B11.6	2.47	3.99E-05
spp-13	2.46	1.78E-04
rol-1	2.45	9.39E-07

K08F8.1e	2.45	3.80E-07
T07E3.4	2.44	2.52E-03
col-109	2.43	1.02E-04
grl-7	2.42	2.36E-04
C09H5.2	2.42	6.53E-03
Y47D3A.13	2.42	8.41E-05
C17C3.1	2.41	3.57E-03
T20F10.2	2.40	7.76E-03
H23N18.5	2.40	1.40E-06
Y43F8B.2	2.40	5.03E-04
K02E11.10	2.40	3.24E-04
C50F7.5	2.40	5.93E-03
sqt-1	2.39	3.52E-10
K07E1.1	2.39	6.20E-04
F49H12.5	2.39	4.24E-06
ZK484.5	2.38	2.22E-09
pqn-44	2.37	5.63E-06
bli-1	2.37	2.16E-03
ptr-4	2.37	6.99E-03
nspc-18	2.36	8.96E-04
F17H10.2	2.36	2.10E-03
nspa-4	2.35	6.50E-05
F17C11.4	2.35	3.93E-03
pmp-5	2.34	1.42E-06
Y11D7A.5	2.34	1.97E-03
C33F10.1	2.33	1.22E-04
F53B1.4	2.32	6.80E-05
clec-209	2.32	3.42E-130
F56A4.2	2.32	3.42E-130
F32A5.4	2.28	8.10E-06
M05B5.2	2.28	7.76E-03
B0280.17	2.28	8.32E-03
pmp-2	2.27	5.63E-03
rab-19	2.26	6.26E-03
col-71	2.26	6.31E-08
Y37D8A.3	2.25	4.75E-03
F35E12.5	2.25	5.13E-27
E03H12.5	2.25	3.70E-03
Y65B4BL.1	2.24	2.20E-03
nurf-1	2.23	3.67E-04
C49A1.10	2.23	7.52E-03
col-14	2.22	3.69E-08
phy-2	2.22	5.98E-03
F27C1.1	2.22	1.07E-04
T27D12.1	2.21	3.73E-03
C53A3.2	2.21	3.09E-06
wrt-4	2.20	9.96E-03

cpg-7	2.20	6.03E-06
B0410.3	2.19	1.58E-03
T22H6.2	2.19	9.21E-03
col-73	2.19	8.27E-20
col-38	2.18	1.21E-13
rol-6	2.18	5.75E-05
Y75B7AR.1	2.18	9.30E-03
C34E7.4	2.18	8.25E-04
C33G8.2	2.17	7.84E-03
nspa-2	2.16	2.09E-04
ttr-32	2.16	2.12E-03
maoc-1	2.15	6.94E-05
F28A12.4	2.15	1.94E-11
nlp-29	2.15	8.68E-07
T28B8.1	2.14	6.81E-03
rcn-1	2.13	5.01E-03
ZK180.5	2.13	2.86E-04
C33A12.19	2.12	1.94E-03
col-48	2.12	1.93E-04
H42K12.3	2.11	4.89E-04
C49F8.3	2.10	2.69E-03
K09H11.7	2.10	1.42E-06
dpy-10	2.09	6.44E-03
F07A5.2	2.09	2.50E-03
wrt-2	2.09	9.85E-03
grl-16	2.08	3.51E-04
grl-5	2.08	1.11E-03
ssq-4	2.08	5.42E-03
K06A5.2	2.08	6.98E-03
C54D10.3	2.07	2.99E-05
fkb-3	2.07	7.60E-04
C52B11.5	2.07	1.00E-02
F44D12.4	2.06	3.44E-04
F25E5.8	2.06	3.59E-03
nspc-19	2.05	4.61E-06
nlp-33	2.05	2.39E-10
cnc-10	2.05	8.80E-03
F56D3.1	2.04	1.55E-03
pqn-32	2.04	3.12E-03
nspc-8	2.03	1.48E-03
col-65	2.02	9.78E-03
C02F5.14	2.02	8.94E-03
C01B10.3	2.01	4.76E-03
sss-2	2.01	5.95E-03
dpy-7	2.01	8.58E-03
T23B3.5	2.00	5.20E-03
F08G5.6	2.00	5.72E-07

ZC410.5	2.00	2.48E-03
tts-2	1.99	2.75E-04
F08C6.2	1.98	7.82E-03
dhs-21	1.98	5.35E-03
tag-165	1.98	8.82E-05
F09F9.2	1.97	7.65E-03
cpr-1	1.97	5.86E-09
Y51H7C.13	1.95	2.78E-04
K01C8.1	1.95	7.57E-04
C05C12.4	1.95	8.63E-03
dpy-5	1.94	5.92E-11
C01B10.11	1.94	1.00E-03
W04G3.1	1.93	3.01E-03
T13F2.9	1.93	1.80E-04
C01G6.6a	1.92	1.04E-04
T06D8.1	1.92	1.12E-03
zip-2	1.92	2.17E-06
col-60	1.92	2.85E-03
lgc-22	1.92	9.08E-03
col-17	1.91	3.49E-15
T12G3.1	1.90	6.26E-03
F56A4.3	1.90	3.52E-11
R12C12.9	1.90	8.22E-03
col-49	1.90	2.41E-04
frm-1	1.90	7.04E-05
C17F3.1	1.89	1.60E-03
ram-2	1.89	8.40E-09
C31C9.2	1.89	7.94E-03
Y47D7A.15	1.88	4.86E-03
ZK105.1	1.88	1.08E-06
F32G8.4	1.88	4.45E-03
rol-8	1.87	4.49E-06
C15C7.5	1.87	3.12E-05
F22E10.5	1.87	7.63E-03
F26B1.1	1.86	1.67E-03
fkb-5	1.86	2.24E-03
H13N06.4	1.86	7.94E-03
C09G4.2	1.84	9.40E-04
K06A4.5	1.84	4.00E-03
aqp-8	1.84	1.08E-03
Y37D8A.16	1.83	5.73E-03
R07E5.4	1.83	2.03E-03
C01B4.9	1.83	2.21E-07
Y19D10A.12	1.83	5.35E-08
grl-4	1.82	1.91E-04
K11D12.7	1.82	4.88E-03
H06I04.3	1.81	3.19E-03

T23G11.1	1.79	1.99E-03
col-162	1.79	1.77E-06
T23G7.3	1.79	1.44E-03
K09G1.1	1.79	9.57E-09
vha-10	1.79	1.92E-14
cpi-1	1.79	3.13E-10
F20D1.1	1.77	6.67E-03
ZK546.14	1.77	7.87E-04
F18E2.1	1.77	7.02E-03
T14D7.2	1.77	7.18E-03
lon-3	1.76	9.49E-03
H03A11.2	1.76	1.84E-06
dpy-13	1.76	1.05E-10
dao-2	1.76	7.03E-08
lpr-4	1.75	7.10E-03
sss-1	1.75	1.36E-04
ras-2	1.75	2.40E-03
F49E2.5	1.75	7.13E-03
C04G2.8	1.75	3.22E-03
H19N07.3	1.75	9.60E-03
Y39A1A.7	1.74	8.73E-05
F18C5.10	1.74	2.66E-03
ttr-18	1.73	9.41E-10
C55A6.12	1.73	1.10E-03
C53B7.3	1.73	2.21E-03
cah-4	1.73	2.87E-08
grd-5	1.73	6.01E-09
F55H12.2	1.72	1.70E-03
grl-21	1.72	3.02E-03
F55C12.1	1.72	5.57E-03
C01G6.6b	1.72	4.24E-04
K07H8.10	1.72	1.78E-07
cyn-6	1.72	2.17E-03
col-90	1.71	3.72E-05
K01H12.2	1.71	9.63E-03
R07E3.1	1.70	5.29E-04
C07E3.9	1.69	6.36E-03
gly-8	1.69	3.79E-03
Y38F1A.6	1.69	1.21E-08
col-77	1.69	1.08E-06
col-104	1.68	4.66E-04
col-112	1.68	5.54E-06
MTCE.33	1.67	2.53E-04
K01D12.9	1.67	2.56E-03
T02C5.1	1.67	1.79E-04
scl-2	1.67	6.91E-03
wrt-10	1.66	4.88E-03

prg-2	1.66	2.77E-03
grl-15	1.66	1.29E-03
lgg-1	1.66	1.09E-14
ZK593.1	1.66	9.15E-03
sptl-3	1.65	6.31E-03
M03B6.2	1.65	3.43E-03
rnh-2	1.65	4.38E-03
M01F1.3	1.65	4.00E-03
lbp-3	1.65	1.64E-03
F54C9.9	1.65	8.53E-03
nspc-11	1.64	2.53E-03
T01B11.2	1.64	7.26E-03
prpf-4	1.64	3.36E-03
Y37A1B.5	1.64	3.95E-03
W07G4.5	1.64	9.62E-03
F42D1.2	1.63	2.25E-04
C46C2.5	1.63	3.08E-03
C48E7.1	1.62	2.35E-05
C48E7.7	1.62	2.32E-07
T01D1.4	1.61	1.79E-03
nspc-16	1.61	5.48E-07
R03D7.1	1.61	1.86E-06
F44E7.2	1.61	5.35E-05
msp-32	1.61	3.81E-09
T08B2.12	1.60	5.72E-07
F11A3.1	1.60	3.53E-03
C28H8.11	1.60	4.93E-04
Y69E1A.1	1.60	9.93E-03
Y45F10C.2	1.59	5.61E-09
clp-1	1.59	5.21E-03
sqt-2	1.59	4.14E-06
nspc-17	1.59	1.28E-04
Y97E10C.1	1.58	1.61E-03
K02D7.1	1.57	6.59E-04
Y48G8AL.13	1.57	7.74E-03
F13E6.1	1.57	2.41E-03
col-130	1.57	1.45E-03
K07B1.6b	1.57	2.38E-03
D1086.11	1.57	3.30E-05
F40F12.7	1.57	5.55E-03
vab-10	1.57	6.61E-03
C18A3.3	1.56	5.30E-03
nspc-4	1.55	2.52E-03
gpdh-2	1.54	2.24E-04
col-161	1.54	3.92E-06
col-157	1.54	3.11E-05
C24A3.2	1.53	2.46E-03

W02D3.1	1.53	5.54E-06
iff-2	1.53	2.33E-17
nspc-9	1.53	4.77E-04
nmy-1	1.52	2.49E-03
haf-9	1.52	1.00E-02
H36L18.2	1.52	1.55E-06
dur-1	1.52	5.69E-03
rfc-1	1.52	9.93E-03
phat-3	1.51	9.41E-03
ZK1248.17	1.51	1.13E-03
T12B3.3	1.51	2.42E-03
vha-11	1.50	3.75E-06
ttr-31	1.50	5.06E-03
Y22D7AL.5	1.50	6.25E-06
dod-6	1.50	7.22E-06
nspa-8	1.49	6.87E-06
F49C12.11	1.49	1.25E-07
MTCE.35	1.49	1.43E-13
nspc-5	1.49	1.31E-03
sym-1	1.48	4.37E-03
nspc-2	1.48	1.14E-03
nspc-6	1.48	1.14E-03
F46H5.7	1.48	5.13E-03
pmt-1	1.48	4.77E-07
ost-1	1.47	1.00E-11
unc-112	1.47	6.06E-03
ran-4	1.47	1.79E-06
C55B7.1	1.47	7.70E-03
dpy-4	1.47	8.96E-09
Y41C4A.13	1.46	6.56E-03
nspc-20	1.46	1.71E-06
dhs-28	1.46	2.52E-04
F44D12.7	1.45	2.82E-05
F55H12.4	1.45	5.37E-06
bcat-1	1.45	1.97E-05
E04F6.5	1.45	1.56E-03
M60.2	1.45	1.59E-03
C45B2.2	1.44	7.65E-06
C39D10.8	1.44	5.43E-03
F18E3.11	1.44	4.56E-04
ZK1307.1	1.44	3.16E-03
F29B9.8	1.43	6.32E-03
F15G9.1	1.43	4.71E-03
cey-1	1.43	1.48E-05
ccg-1	1.43	2.11E-03
ZC247.1	1.43	1.92E-06
W05H9.1	1.43	1.26E-03

tag-60	1.43	3.66E-04
B0303.3	1.42	6.52E-05
F21D5.7	1.42	1.63E-03
cpz-2	1.42	2.53E-03
ZK856.8	1.42	2.45E-04
K07C11.7	1.42	4.97E-03
mup-4	1.42	7.20E-03
T28F4.5	1.41	8.19E-07
ifb-1	1.40	5.87E-06
T02H6.11	1.39	1.48E-09
grd-14	1.39	7.27E-03
C08F11.12	1.39	6.71E-08
F54A3.5	1.39	1.12E-04
erd-2	1.39	8.05E-03
trap-2	1.38	1.67E-08
Y43D4A.2	1.37	7.86E-03
F42G8.10	1.37	1.47E-04
Y38C1AA.7	1.37	5.46E-04
F07H5.4	1.36	7.21E-03
C16A3.6	1.36	2.95E-03
F53A2.7	1.36	1.91E-04
E02D9.1	1.36	8.71E-03
sptl-1	1.35	8.12E-04
Y69E1A.2	1.35	1.00E-02
fib-1	1.35	1.64E-06
tag-170	1.34	1.01E-03
unc-43	1.33	6.73E-03
Y54E10A.17	1.33	3.97E-03
C44B7.5	1.33	1.54E-04
ads-1	1.33	6.63E-03
ech-6	1.33	8.83E-12
gst-36	1.32	4.98E-03
apl-1	1.32	1.91E-03
ftn-2	1.32	1.41E-08
H37A05.1	1.32	5.53E-04
T23F11.1	1.32	2.25E-03
F59C6.5	1.32	5.00E-08
C11E4.1	1.31	8.55E-03
F59E10.3	1.30	1.41E-04
F09F7.4	1.29	1.48E-04
C37E2.1	1.28	7.01E-03
F10G2.1	1.28	4.70E-03
hsp-17	1.28	4.15E-03
T08B2.7	1.28	2.35E-04
F57C2.4	1.28	4.47E-03
abcf-1	1.28	3.29E-03
Y71F9AM.6	1.28	1.14E-08

tag-210	1.28	9.09E-06
F35G2.2	1.28	7.86E-03
clec-1	1.27	5.71E-05
F20D1.4	1.27	1.47E-03
C10G11.9	1.27	6.29E-03
Y87G2A.10	1.27	7.08E-03
F54D5.12	1.27	9.34E-04
R02D3.1	1.27	2.87E-03
gst-7	1.26	1.65E-04
lbp-7	1.26	3.54E-03
R03E1.2	1.26	2.10E-05
ZK1307.8	1.26	1.66E-03
hpd-1	1.25	1.96E-04
pho-1	1.25	9.64E-03
cpi-2	1.25	9.20E-06
F07H5.3	1.25	1.36E-04
K09E2.3	1.25	2.97E-03
dlc-1	1.25	1.88E-14
Y54G2A.23	1.25	6.70E-04
sel-9	1.24	2.45E-05
Y54G2A.3	1.24	5.11E-03
C27B7.9	1.24	2.94E-03
qdpr-1	1.24	9.91E-04
asna-1	1.24	4.82E-03
C14C6.5	1.23	1.28E-04
C44B7.10	1.23	6.91E-06
dpy-11	1.23	7.08E-04
lmp-1	1.23	4.82E-05
F28A10.6	1.23	6.72E-03
F54E2.1	1.23	3.88E-03
vha-4	1.23	1.00E-06
R10H10.3	1.22	1.39E-03
F09G8.7	1.22	5.77E-04
cat-4	1.22	5.57E-03
F59B1.2	1.22	7.29E-05
ldh-1	1.22	4.61E-03
vha-8	1.22	3.28E-10
F17E9.5	1.22	1.75E-03
col-145	1.21	2.99E-08
iftb-1	1.21	5.85E-05
T01C8.2	1.21	6.34E-03
T08G2.3	1.21	6.43E-03
aqp-2	1.20	6.30E-03
ppn-1	1.20	3.91E-04
D1054.10	1.20	1.22E-11
M60.4	1.20	4.12E-03
F09E5.3	1.19	2.31E-03

R11A8.5	1.19	8.42E-03
let-721	1.19	4.60E-03
F02A9.4	1.19	2.60E-03
F57B10.3	1.19	2.43E-03
trap-1	1.18	1.41E-07
cpz-1	1.18	1.22E-05
ttr-47	1.18	1.98E-04
Y38E10A.13	1.18	6.12E-03
C18B2.5	1.18	4.29E-03
ZK418.5	1.18	1.26E-03
F43G9.10	1.18	7.12E-03
atn-1	1.17	3.70E-03
ugt-22	1.17	8.83E-04
arf-1.2	1.17	2.51E-07
hrp-2	1.17	6.19E-03
R12E2.7	1.16	2.10E-03
ZC395.10	1.16	6.47E-07
C34F11.3	1.16	8.50E-03
dim-1	1.16	1.61E-05
Y62E10A.13	1.16	7.92E-03
mlc-4	1.16	1.38E-05
F52A8.5	1.16	1.44E-03
K11H12.7	1.15	6.99E-03
Y110A7A.6	1.15	4.77E-03
nuo-4	1.15	1.16E-04
F42A9.6	1.15	5.46E-03
alp-1	1.15	2.68E-03
F47G9.1	1.15	1.09E-04
pqn-22	1.14	3.82E-03
dnj-12	1.14	8.26E-04
C42D4.1	1.14	1.68E-05
puf-12	1.14	3.63E-03
C36B1.7	1.14	5.39E-03
aqp-10	1.14	9.98E-03
C32D5.8	1.13	1.03E-03
fat-4	1.13	6.44E-05
him-3	1.13	7.67E-03
C54E4.2	1.13	4.02E-03
vha-15	1.13	6.01E-05
H28G03.1	1.13	9.88E-03
nspc-3	1.12	9.96E-04
npa-1	1.12	2.96E-04
F15B10.1	1.12	3.29E-03
apy-1	1.12	8.84E-03
F13H8.7	1.11	1.35E-03
W10D9.5	1.11	3.63E-03
ZK470.2	1.11	6.83E-03

pas-5	1.11	9.39E-05
F57B10.5	1.10	1.45E-03
F53F4.10	1.10	1.42E-05
vha-2	1.10	2.03E-13
uvt-5	1.10	3.41E-03
far-8	1.10	9.96E-03
T27A3.4	1.09	8.14E-03
R151.2	1.09	4.86E-04
F53F1.4	1.09	2.28E-10
F53F10.2	1.08	4.34E-03
mel-32	1.08	3.92E-07
R11A5.4	1.08	6.94E-08
T05E11.9	1.08	2.21E-03
pqn-70	1.08	1.49E-03
clic-1	1.07	1.03E-04
C36E8.1	1.07	6.57E-03
C05C10.3	1.06	6.65E-03
Y45F10D.4	1.06	2.95E-03
mmaa-1	1.06	4.55E-03
K07A1.10	1.06	8.12E-03
C04G2.9	1.06	9.03E-03
cpn-4	1.06	4.05E-03
nspc-13	1.06	3.60E-03
T27F7.3	1.06	2.10E-06
R107.5	1.06	2.87E-03
R05D11.5	1.06	9.45E-03
col-12	1.05	1.52E-07
ubh-4	1.05	6.08E-03
hif-1	1.05	7.23E-03
rpt-4	1.05	7.85E-04
taf-13	1.04	5.44E-03
cpt-2	1.04	9.60E-03
F54C9.3	1.04	2.38E-04
egl-30	1.03	7.49E-03
glh-1	1.03	1.71E-03
rpl-11.2	1.03	8.33E-12
ccdc-47	1.03	2.67E-03
K12H4.4	1.03	2.52E-03
col-154	1.03	1.69E-04
dnj-13	1.03	7.57E-05
tram-1	1.03	2.01E-04
B0416.5	1.03	9.62E-03
far-6	1.03	1.12E-03
rab-11.1	1.03	6.43E-07
col-13	1.02	9.06E-08
pqn-53	1.02	9.98E-03
cpl-1	1.02	7.72E-13

K02F2.2	1.01	2.34E-18
M03F4.6	1.01	3.24E-04
hsp-25	1.01	1.11E-03
Y51A2D.14	1.01	7.40E-04
anc-1	1.01	2.83E-03
lpl-1	1.01	2.29E-04
unc-32	1.01	9.74E-03
let-2	1.00	7.01E-04
MTCE.16	1.00	1.15E-08
R05F9.6	1.00	4.93E-04
sod-2	1.00	3.15E-03
sams-1	1.00	8.15E-06
unc-15	1.00	2.50E-08
F43E2.7	1.00	8.50E-04
pfn-2	0.99	3.36E-03
trap-3	0.99	3.09E-06
prs-1	0.99	2.07E-03
F41F3.3	0.99	1.97E-05
mag-1	0.99	1.91E-03
csn-2	0.98	9.83E-03
F32B5.1	0.98	2.43E-03
Y53G8AL.2	0.98	4.83E-03
F53H4.2	0.98	7.81E-03
skr-1	0.98	2.47E-04
tat-4	0.98	4.44E-03
ttr-36	0.98	6.66E-03
kin-2	0.97	1.51E-03
Y43C5A.2	0.97	9.46E-03
F52A8.1	0.96	3.67E-05
mua-6	0.96	4.81E-03
lev-11	0.96	4.46E-07
tct-1	0.96	7.25E-20
eif-3.F	0.95	1.80E-03
his-41	0.95	9.07E-03
col-107	0.95	1.46E-03
nspc-10	0.94	7.12E-03
C15C8.3	0.94	7.37E-03
aex-5	0.94	6.72E-05
vha-1	0.94	9.10E-04
cct-5	0.94	5.35E-05
T20G5.8	0.94	1.08E-03
Y66H1B.2	0.94	2.43E-04
F37C12.3	0.94	7.61E-04
glt-1	0.94	6.49E-03
T07A9.9	0.94	3.73E-03
snr-4	0.94	1.47E-04
pes-9	0.93	1.25E-03

E02H1.8	0.93	6.27E-03
unc-54	0.92	5.51E-07
MTCE.34	0.92	3.01E-17
arf-3	0.92	2.30E-06
col-155	0.92	4.30E-04
sqv-4	0.91	9.23E-03
F35G12.2	0.91	7.27E-03
F32D1.5	0.91	2.24E-04
sdhd-1	0.91	3.04E-03
R07H5.8	0.90	2.77E-06
lpd-5	0.90	2.72E-03
F52E1.14	0.90	2.72E-04
F58E10.3	0.90	1.86E-03
ftt-2	0.90	3.58E-06
T05H4.6a	0.89	3.36E-03
nspc-12	0.89	3.63E-03
vha-14	0.89	1.63E-04
ZC262.8	0.89	8.13E-03
F07H5.5	0.89	5.75E-08
gta-1	0.89	2.48E-04
mdt-28	0.89	6.17E-04
F41C3.5	0.88	4.68E-06
W01A8.1b	0.88	4.89E-04
arx-6	0.88	2.15E-03
F26B1.2	0.88	6.89E-03
F25B5.3	0.88	2.80E-03
C06A8.1	0.88	7.52E-04
LLC1.3	0.87	2.53E-03
W10C8.5	0.87	3.12E-03
T13F2.2	0.86	1.85E-03
C34B2.8	0.86	9.83E-04
C16A3.5	0.86	4.75E-04
K02D10.1	0.86	8.51E-03
tag-174	0.86	5.31E-07
C37C3.2	0.85	4.44E-03
nuo-1	0.85	3.39E-03
T15B7.2	0.85	8.84E-04
isp-1	0.85	4.30E-05
R155.1	0.85	9.60E-03
C10G8.4	0.85	5.83E-06
vha-16	0.84	2.91E-04
F08B12.4	0.84	1.49E-03
unc-87	0.84	2.81E-04
M02D8.1	0.84	2.17E-03
gdi-1	0.84	3.71E-04
rab-5	0.84	2.75E-03
pat-10	0.84	1.25E-12

M01H9.3	0.83	6.48E-03
H19N07.1	0.83	8.42E-03
lea-1	0.83	3.60E-03
C31E10.7	0.83	4.48E-03
tag-18	0.83	5.01E-03
ZK637.2	0.83	6.69E-03
T09E8.3	0.83	7.55E-03
C28H8.4	0.83	4.23E-03
F53A9.8	0.82	3.57E-03
ncs-2	0.82	4.27E-03
ubc-7	0.82	1.62E-04
K08D12.3	0.82	2.03E-05
sod-1	0.81	1.58E-03
erm-1	0.81	1.26E-03
baf-1	0.81	3.57E-03
tufm-1	0.80	9.25E-03
col-180	0.80	5.60E-03
T05H10.6	0.80	3.45E-04
MTCE.25	0.79	9.93E-06
col-169	0.79	5.28E-05
C16A3.10	0.79	3.58E-03
gpi-1	0.79	9.92E-03
col-144	0.78	1.58E-07
msp-38	0.78	2.70E-07
unc-60	0.78	3.51E-03
F32E10.6	0.78	7.83E-03
C06E7.1	0.78	3.83E-03
pdi-3	0.78	8.85E-07
rmd-2	0.78	6.19E-03
mup-2	0.78	2.52E-04
dad-1	0.77	6.24E-04
spp-10	0.77	2.01E-03
B0334.4	0.77	8.27E-03
col-166	0.77	1.25E-05
msp-52	0.77	4.61E-12
F57F5.1	0.77	1.88E-06
Y39G10AR.8	0.76	5.59E-03
cgh-1	0.76	6.39E-06
plp-1	0.75	7.83E-04
T25B9.9	0.75	3.81E-03
F21F3.6	0.75	2.50E-03
atp-3	0.75	4.45E-05
rhr-1	0.75	3.15E-03
Y59A8A.3	0.74	6.83E-03
R06C1.4	0.74	3.47E-05
kin-19	0.74	6.35E-04
C30C11.4	0.74	6.48E-04

hrp-1	0.73	6.50E-03
col-167	0.73	3.36E-05
prdx-3	0.73	1.67E-03
F56C9.7	0.73	3.67E-03
nspa-1	0.73	5.00E-05
hsp-6	0.73	1.44E-03
cpr-6	0.72	1.08E-05
elo-5	0.72	6.36E-04
brp-1	0.72	2.46E-03
Y105E8B.11	0.72	7.04E-32
col-10	0.71	5.22E-07
rab-1	0.71	9.57E-04
T27E9.2	0.71	8.71E-04
W03F11.1	0.70	2.41E-03
gst-1	0.70	2.37E-04
