# Supplementary material for: Gene expression profiling of oxidative stress response of C. elegans aging defective AMPK mutants using massively parallel transcriptome sequencing
Source: BMC Res Notes. 2011 Feb 8;4:34. doi: 10.1186/1756-0500-4-34 (PMC3045954; doi:10.1186/1756-0500-4-34)
Supplement: Additional file 7 — Supplementary Table S6. Commonly down-regulated genes in stressed wild type and stressed aak-2 mutants relative to wild type [file 1756-0500-4-34-S7.PDF]

Supplementary Table 6. Commonly down-regulated genes in stressed wild type and stressed aak-2 mutants relative to wild type

Gene	Stressed N2	pval	Stressed aak-2	pval2
srz-70	-5.82	2.56E-18	-5.90	3.56E-19
F53C11.2	-4.35	1.17E-06	-4.43	5.81E-07
ZK218.13	-3.88	7.45E-29	-1.79	1.46E-14
nhr-37	-3.56	5.10E-04	-3.65	3.41E-04
his-18	-3.28	2.58E-05	-1.75	1.31E-03
F49B2.3	-3.26	2.37E-03	-3.34	1.71E-03
his-5	-3.03	2.77E-04	-1.76	9.12E-03
Y48G1BM.7	-2.92	8.72E-03	-3.01	6.76E-03
nhr-93	-2.63	2.73E-08	-1.74	6.49E-06
Y110A2AL.13	-2.50	7.93E-04	-2.53	4.99E-04
his-67	-2.36	1.50E-03	-1.95	2.31E-03
T24B8.5	-2.32	1.54E-29	-2.56	3.52E-34
ins-27	-2.14	3.04E-04	-2.18	1.48E-04
Y105C5A.13	-2.12	6.48E-07	-3.68	2.05E-10
rrn-3.1	-2.09	1.25E-173	-1.64	1.89E-131
Y92H12BL.5	-2.06	4.15E-03	-1.71	8.91E-03
F56H9.2	-1.99	2.21E-31	-1.75	2.33E-27
F35H10.5	-1.98	5.88E-05	-1.99	1.28E-04
F26E4.6	-1.97	4.18E-48	-2.25	4.09E-58
Y22D7AR.10	-1.96	1.69E-21	-1.30	7.28E-14
rrn-3.56	-1.94	4.27E-82	-1.64	2.47E-68
C08E3.13	-1.92	1.05E-06	-3.39	2.67E-11
Y67D8C.12	-1.92	5.79E-04	-2.09	4.28E-04
F43G6.7	-1.91	9.40E-04	-1.95	5.54E-04
acbp-1	-1.88	6.50E-41	-1.84	1.54E-41
his-47	-1.88	4.09E-04	-1.73	5.22E-04
ddp-1	-1.83	1.60E-15	-1.86	1.39E-16
kbp-4	-1.78	3.31E-07	-1.57	8.70E-07
msp-37	-1.76	2.86E-08	-1.35	8.35E-07
B0495.6	-1.71	2.85E-08	-1.66	3.97E-08
C08E3.1	-1.71	1.07E-05	-3.69	1.50E-10
F20C5.3	-1.71	4.08E-03	-1.56	6.30E-03
lin-40	-1.71	3.40E-07	-0.86	9.20E-04
Y105C5A.12	-1.71	1.07E-03	-3.47	6.33E-06
his-68	-1.67	9.54E-04	-2.21	7.90E-05
rps-21	-1.67	3.21E-138	-2.02	3.98E-186
nduf-5	-1.66	2.76E-15	-1.22	7.21E-11
rab-18	-1.64	1.02E-04	-2.02	7.86E-06
W08D2.9	-1.59	5.36E-14	-1.03	2.19E-08
F33G12.7	-1.58	7.68E-04	-1.25	2.95E-03
elc-1	-1.55	2.60E-03	-1.43	4.24E-03
dyrb-1	-1.54	1.06E-05	-1.41	5.41E-05
gut-2	-1.54	2.41E-07	-1.92	4.03E-10
cyc-2.1	-1.50	1.15E-44	-1.19	1.59E-33

spp-14	-1.49	1.77E-29	-1.16	1.86E-21
Y65B4A.6	-1.49	1.72E-03	-1.17	9.78E-03
W02D9.6	-1.48	8.22E-04	-2.35	9.56E-06
C37A2.7	-1.45	9.14E-76	-1.58	9.08E-89
Y63D3A.7	-1.45	1.02E-05	-1.28	1.77E-05
ZK686.1	-1.43	2.01E-07	-1.48	2.32E-08
C14C11.7	-1.42	7.38E-04	-1.67	2.01E-04
rps-24	-1.40	2.64E-116	-1.40	5.35E-120
Y53F4B.14	-1.40	3.97E-03	-1.29	5.29E-03
lsm-5	-1.39	1.01E-04	-1.22	4.01E-04
msp-74	-1.39	3.19E-05	-0.83	1.71E-03
spp-5	-1.39	5.08E-61	-1.33	1.77E-59
C50H11.8	-1.38	5.06E-03	-1.31	6.31E-03
clec-85	-1.37	2.85E-03	-0.84	8.74E-03
aps-3	-1.36	9.63E-04	-0.85	8.76E-03
cpg-8	-1.36	1.26E-05	-1.50	6.70E-06
spp-17	-1.36	3.41E-29	-1.61	2.31E-38
W02D9.7	-1.36	1.45E-07	-1.07	3.98E-06
C17E7.12	-1.34	7.25E-03	-2.04	2.79E-04
rps-11	-1.34	2.13E-121	-1.34	6.84E-128
M02H5.8	-1.33	2.10E-03	-2.29	1.92E-04
C53H9.3	-1.31	1.54E-04	-1.39	6.47E-05
spp-3	-1.31	1.13E-25	-1.18	8.41E-23
W03G9.8	-1.31	8.86E-04	-1.18	2.15E-03
Y105E8A.11	-1.31	2.37E-03	-1.72	3.34E-04
Y110A2AM.4	-1.31	5.33E-03	-2.05	9.25E-04
K11H3.6	-1.30	1.03E-06	-1.86	4.64E-10
F23D12.1	-1.29	1.73E-03	-1.50	4.40E-04
F23D12.7	-1.28	1.85E-05	-1.16	3.45E-05
K01H12.1	-1.28	9.68E-04	-1.29	1.16E-03
phf-5	-1.28	8.21E-03	-1.53	9.45E-03
vha-3	-1.27	2.46E-05	-1.25	4.15E-05
R102.2	-1.26	6.52E-03	-1.17	7.18E-03
spp-23	-1.24	2.75E-08	-1.39	2.91E-09
mtl-2	-1.23	1.07E-03	-0.94	3.69E-03
rpl-26	-1.23	7.64E-61	-1.49	1.77E-82
rpl-36	-1.23	9.10E-91	-1.49	7.25E-123
mxl-1	-1.21	7.61E-04	-1.26	3.08E-04
F44E5.1	-1.19	5.12E-25	-1.74	9.20E-44
pfd-6	-1.19	5.88E-05	-1.05	2.08E-04
F23F1.10	-1.17	1.71E-03	-1.20	2.43E-03
F29C4.2	-1.17	6.48E-07	-1.03	1.83E-06
C35B1.4	-1.16	1.58E-06	-1.49	5.04E-09
rpl-38	-1.16	1.43E-61	-0.94	4.71E-47
rpb-11	-1.14	6.96E-05	-1.78	2.14E-08
Y69A2AR.28	-1.12	4.52E-03	-1.27	3.72E-03
F22D6.14	-1.11	4.83E-03	-1.27	4.57E-03

ZC373.2	-1.11	1.05E-10	-0.80	2.20E-07
B0205.12	-1.09	2.99E-03	-1.07	2.54E-03
lsm-6	-1.05	1.16E-03	-1.01	5.23E-04
rpb-10	-1.05	5.53E-05	-1.00	9.75E-05
rpl-34	-1.05	1.35E-49	-1.11	3.24E-55
W01D2.1	-1.05	1.49E-74	-1.55	4.75E-138
F53A3.3	-1.04	5.21E-36	-1.00	8.24E-36
oig-2	-1.02	8.06E-04	-0.75	3.36E-03
cpg-9	-1.01	7.56E-10	-1.42	1.62E-15
his-58	-1.01	2.79E-03	-1.39	8.31E-04
elb-1	-1.00	8.64E-03	-1.09	5.78E-03
F53F4.16	-1.00	9.49E-04	-0.82	3.07E-03
rps-30	-1.00	2.41E-34	-1.07	2.31E-39
F58A4.2	-0.98	7.86E-03	-1.34	2.05E-03
rpl-41	-0.98	2.03E-89	-1.43	2.67E-166
T14B4.2	-0.98	4.53E-03	-1.36	1.24E-03
Y59A8B.12	-0.98	2.19E-03	-1.29	2.15E-04
his-48	-0.97	6.29E-03	-1.43	7.39E-04
rpl-22	-0.96	7.75E-26	-0.96	8.41E-27
F29B9.11	-0.94	7.93E-17	-1.29	3.09E-27
MTCE.4	-0.94	4.36E-53	-2.66	3.34E-227
Y55B1AL.2	-0.93	9.42E-04	-1.00	3.55E-04
Y69A2AR.3	-0.93	1.40E-03	-0.86	1.00E-03
C49F5.7.1	-0.90	3.68E-03	-1.11	5.78E-04
K10D2.4	-0.90	2.45E-03	-1.40	1.04E-04
rps-12	-0.90	2.73E-31	-0.89	4.38E-32
rps-28	-0.90	2.47E-70	-1.16	7.84E-110
C49F5.7.2	-0.89	2.57E-03	-1.11	4.47E-04
C48B6.3	-0.88	6.21E-03	-1.28	1.06E-03
K10B2.4	-0.87	1.97E-03	-1.02	6.63E-04
rps-29	-0.87	1.08E-30	-1.14	2.17E-47
iff-1	-0.84	5.16E-22	-1.44	1.51E-49
ilys-5	-0.84	1.01E-05	-0.73	2.26E-05
rpl-35	-0.84	5.75E-27	-1.02	2.73E-37
C28C12.2	-0.81	1.33E-04	-0.99	2.63E-05
ife-3	-0.81	6.85E-04	-1.47	8.01E-07
Y37E3.8	-0.81	1.08E-33	-0.72	3.22E-29
K12H4.5	-0.79	1.16E-04	-0.75	8.99E-05
rps-19	-0.79	5.64E-26	-0.71	7.15E-23
Y119D3B.21	-0.79	8.72E-18	-1.96	2.88E-66
rpl-25.1	-0.78	4.27E-08	-0.95	1.73E-10
rpl-43	-0.78	3.26E-25	-0.96	1.90E-36
rpb-12	-0.77	1.90E-04	-1.38	1.03E-07
rrn-1.1	-0.77	1.24E-38	-1.34	1.38E-93
rrn-1.2	-0.77	1.24E-38	-1.34	1.38E-93
atp-4	-0.76	1.54E-06	-0.86	1.23E-07
rps-26	-0.75	1.26E-28	-1.11	6.93E-55

Y82E9BR.3	-0.73	3.75E-21	-0.90	9.48E-30
rpl-33	-0.72	4.35E-33	-1.26	2.05E-83
rps-5	-0.72	5.40E-17	-0.72	1.84E-17
tomm-7	-0.72	3.59E-04	-0.94	2.43E-05
	-1.44	1.09E-03	-1.54	1.06E-03
