# Supplementary material for: Gene expression profiling of oxidative stress response of C. elegans aging defective AMPK mutants using massively parallel transcriptome sequencing
Source: BMC Res Notes. 2011 Feb 8;4:34. doi: 10.1186/1756-0500-4-34 (PMC3045954; doi:10.1186/1756-0500-4-34)
Supplement: Additional file 8 — Supplementary Table S7. Commonly up-regulated genes in stressed wild type and stressed aak-2 mutants relative to wild type [file 1756-0500-4-34-S8.PDF]

Supplementary Table 7. Commonly up-regulated genes in stressed wild type and stressed aak-2 mutants relative to wild type

Gene	Stressed N2	pval	Stressed aak-2	pval2
ttn-1	4.69	6.62E-07	4.16	1.52E-04
dao-4	2.95	6.09E-04	2.88	8.66E-04
pqn-95	2.94	1.55E-04	3.09	7.46E-05
qua-1	2.94	9.08E-03	3.56	1.61E-03
rol-1	2.88	1.31E-09	2.45	9.39E-07
mlt-8	2.78	4.39E-06	3.03	2.82E-08
R02F11.1	2.70	4.66E-03	2.83	3.20E-03
C27D6.3	2.69	2.27E-03	2.71	4.23E-03
hsp-16.11	2.59	2.99E-08	4.82	6.85E-59
col-109	2.55	2.38E-05	2.43	1.02E-04
hsp-16.48	2.53	7.26E-12	4.46	1.30E-67
hsp-16.49	2.53	7.26E-12	4.46	1.30E-67
ZK180.6	2.53	4.97E-04	2.77	3.47E-05
hsp-16.1	2.49	8.83E-10	4.67	6.41E-71
F15B9.8	2.49	7.35E-03	3.27	3.83E-05
bli-1	2.46	1.67E-03	2.37	2.16E-03
T19C3.2	2.44	3.29E-03	2.77	1.66E-03
R02E4.3	2.43	2.32E-03	3.87	5.68E-10
col-138	2.39	6.61E-07	2.68	1.14E-08
bli-2	2.37	2.29E-04	2.55	1.91E-05
Y47D7A.15	2.36	5.61E-04	1.88	4.86E-03
wrt-4	2.33	9.24E-03	2.20	9.96E-03
hsp-16.41	2.32	9.07E-06	4.24	1.37E-30
hsp-16.2	2.30	8.95E-04	4.34	1.20E-17
F53B1.4	2.29	1.93E-04	2.32	6.80E-05
F33D4.6	2.26	8.38E-04	2.73	7.35E-06
K02E11.10	2.24	6.64E-04	2.40	3.24E-04
col-175	2.19	1.11E-06	2.52	6.23E-09
grl-16	2.19	8.66E-05	2.08	3.51E-04
E01G4.6	2.18	3.08E-04	2.83	4.71E-08
ZK180.5	2.17	1.45E-04	2.13	2.86E-04
H23N18.5	2.12	2.56E-05	2.40	1.40E-06
F49H12.5	2.11	1.46E-04	2.39	4.24E-06
F56D3.1	2.09	9.66E-04	2.04	1.55E-03
H06I04.3	2.07	6.44E-04	1.81	3.19E-03
Y94H6A.10	2.06	1.05E-06	2.65	1.68E-12
grl-7	2.05	2.17E-03	2.42	2.36E-04
K01D12.9	2.03	3.29E-04	1.67	2.56E-03
T06D8.1	2.01	8.14E-04	1.92	1.12E-03
col-73	2.00	9.17E-16	2.19	8.27E-20
ZK546.14	1.97	3.25E-04	1.77	7.87E-04
col-49	1.91	2.50E-04	1.90	2.41E-04

col-60	1.90	4.08E-03	1.92	2.85E-03
frm-1	1.89	1.53E-04	1.90	7.28E-05
col-14	1.88	4.94E-05	2.23	2.09E-07
ttr-14	1.88	9.14E-03	2.52	1.27E-04
lon-8	1.88	9.26E-03	2.62	2.98E-04
col-63	1.87	6.80E-04	2.48	1.23E-06
Y22D7AL.5	1.87	4.37E-09	1.50	6.25E-06
pqn-32	1.86	8.25E-03	2.04	3.12E-03
col-71	1.85	2.14E-05	2.26	6.31E-08
H42K12.3	1.85	5.17E-03	2.11	4.89E-04
F54C9.9	1.85	3.61E-03	1.65	8.53E-03
F49E2.5	1.83	5.38E-03	1.75	7.13E-03
K07H8.10	1.82	2.45E-08	1.72	1.78E-07
col-157	1.81	8.86E-07	1.54	3.11E-05
grd-5	1.80	1.17E-09	1.73	6.01E-09
col-162	1.80	2.08E-06	1.79	1.77E-06
col-145	1.79	1.86E-18	1.21	2.99E-08
fkb-5	1.79	2.48E-03	1.86	2.24E-03
C39D10.8	1.78	8.01E-04	1.44	5.43E-03
col-38	1.77	2.04E-08	2.18	1.21E-13
W02D3.1	1.75	2.40E-07	1.53	5.54E-06
wrt-10	1.75	3.77E-03	1.66	4.88E-03
col-17	1.74	4.68E-12	1.91	3.49E-15
dpy-5	1.74	1.92E-08	1.94	5.92E-11
cey-1	1.74	7.99E-08	1.43	1.48E-05
tts-2	1.73	2.10E-03	1.99	2.75E-04
sqt-1	1.71	3.54E-05	2.39	3.52E-10
col-48	1.71	4.25E-03	2.12	1.93E-04
F32A5.4	1.70	2.37E-03	2.28	8.10E-06
F20D1.1	1.69	8.53E-03	1.77	6.67E-03
nlp-33	1.68	1.00E-06	2.05	2.39E-10
ram-2	1.68	5.58E-07	1.89	8.40E-09
col-161	1.65	1.65E-06	1.54	3.92E-06
col-77	1.63	3.05E-06	1.69	1.08E-06
col-130	1.60	1.61E-03	1.57	1.45E-03
dpy-13	1.57	2.99E-08	1.76	1.05E-10
ZK1248.17	1.57	6.77E-04	1.51	1.13E-03
iff-2	1.53	5.26E-17	1.53	2.33E-17
dpy-4	1.53	2.72E-09	1.47	8.96E-09
col-112	1.53	5.26E-05	1.68	5.54E-06
T23G7.3	1.51	8.58E-03	1.79	1.44E-03
hrp-2	1.51	6.64E-04	1.17	6.19E-03
vha-10	1.50	1.21E-09	1.79	1.92E-14
C04F12.7	1.50	5.67E-03	2.48	3.64E-07
Y71F9AM.6	1.49	2.13E-11	1.28	1.14E-08
tag-210	1.47	2.50E-07	1.28	9.09E-06
T02H6.11	1.46	3.20E-10	1.39	1.48E-09

F53F1.4	1.45	2.82E-18	1.09	2.28E-10
col-104	1.44	2.11E-03	1.68	4.66E-04
Y97E10C.1	1.44	5.10E-03	1.58	1.61E-03
rol-8	1.43	9.32E-04	1.87	4.49E-06
col-90	1.42	8.27E-04	1.71	3.72E-05
Y51H7C.13	1.42	7.67E-03	1.95	2.78E-04
fib-1	1.41	7.61E-07	1.35	1.64E-06
C36B1.7	1.40	9.00E-04	1.14	5.39E-03
sym-1	1.39	8.69E-03	1.48	4.37E-03
H03A11.2	1.38	3.30E-04	1.76	1.84E-06
Y54G2A.23	1.38	2.26E-04	1.25	6.70E-04
prg-2	1.38	9.10E-03	1.66	2.77E-03
C18B2.5	1.38	1.15E-03	1.18	4.29E-03
trap-1	1.37	9.84E-10	1.18	1.41E-07
cpg-7	1.36	8.23E-03	2.20	6.03E-06
vha-11	1.35	3.64E-05	1.50	3.75E-06
F54A3.5	1.35	2.14E-04	1.39	1.12E-04
ran-4	1.34	1.70E-05	1.47	1.79E-06
grl-4	1.34	7.09E-03	1.82	1.91E-04
D1086.11	1.33	4.56E-04	1.57	3.30E-05
dim-1	1.31	8.39E-07	1.16	1.61E-05
col-155	1.30	1.33E-06	0.92	4.30E-04
taf-13	1.30	1.00E-03	1.04	5.44E-03
ZK105.1	1.28	1.40E-03	1.88	1.08E-06
C15C7.5	1.27	5.55E-03	1.87	3.12E-05
aqp-2	1.27	4.43E-03	1.20	6.30E-03
sqt-2	1.26	3.96E-04	1.59	4.14E-06
col-154	1.26	5.54E-06	1.03	1.69E-04
cpi-2	1.24	1.71E-05	1.25	9.20E-06
F43G9.10	1.24	7.86E-03	1.18	7.12E-03
F49C12.11	1.23	2.35E-05	1.49	1.25E-07
col-144	1.21	2.59E-16	0.78	1.58E-07
Y39A1A.7	1.21	7.16E-03	1.74	8.73E-05
ppn-1	1.21	4.77E-04	1.20	3.91E-04
Y59A8A.3	1.20	7.06E-05	0.74	6.83E-03
col-107	1.18	1.53E-04	0.95	1.46E-03
dlc-1	1.17	1.67E-12	1.25	1.88E-14
E02H1.8	1.17	1.24E-03	0.93	6.27E-03
acdh-1	1.16	2.33E-06	2.98	4.50E-60
cpr-1	1.16	1.54E-03	1.97	5.86E-09
dod-6	1.16	8.24E-04	1.50	7.22E-06
K08D12.3	1.16	2.40E-09	0.82	2.03E-05
nspc-9	1.16	6.98E-03	1.53	4.77E-04
snr-4	1.15	3.56E-06	0.94	1.47E-04
ttr-18	1.14	1.06E-04	1.73	9.41E-10
aex-5	1.13	2.16E-06	0.94	6.72E-05
trap-3	1.12	1.88E-07	0.99	3.09E-06

col-169	1.12	1.98E-08	0.79	5.28E-05
trap-2	1.11	1.40E-05	1.38	1.67E-08
cgh-1	1.11	4.51E-11	0.76	6.39E-06
zip-2	1.10	6.73E-03	1.92	2.17E-06
F09G8.7	1.10	2.68E-03	1.22	5.77E-04
sdhd-1	1.10	5.71E-04	0.91	3.04E-03
ftn-2	1.09	5.41E-06	1.32	1.41E-08
arf-1.2	1.08	2.61E-06	1.17	2.51E-07
rab-11.1	1.08	2.37E-07	1.03	6.43E-07
col-166	1.08	2.77E-09	0.77	1.72E-05
col-10	1.07	3.36E-14	0.71	5.22E-07
msp-32	1.06	2.51E-04	1.61	3.81E-09
dnj-12	1.06	2.37E-03	1.14	8.26E-04
F09E5.3	1.06	5.08E-03	1.19	2.31E-03
col-167	1.05	5.77E-09	0.73	3.36E-05
glh-1	1.05	2.03E-03	1.03	1.71E-03
ZK1307.8	1.04	7.79E-03	1.26	1.66E-03
F59E10.3	1.03	2.22E-03	1.30	1.41E-04
Y66H1B.2	1.03	9.51E-05	0.94	2.43E-04
tag-170	1.03	8.55E-03	1.34	1.01E-03
MTCE.35	1.02	1.79E-06	1.49	1.43E-13
ost-1	1.02	4.68E-06	1.47	1.00E-11
F47G9.1	1.02	5.03E-04	1.15	1.09E-04
arx-6	1.02	7.65E-04	0.88	2.15E-03
F26B1.2	1.02	3.15E-03	0.88	6.89E-03
unc-15	1.01	3.33E-08	1.00	2.50E-08
ifb-1	1.01	1.39E-03	1.40	5.87E-06
R10H10.3	1.01	6.45E-03	1.22	1.39E-03
lev-11	1.00	1.98E-07	0.96	4.46E-07
H36L18.2	1.00	1.56E-03	1.52	1.55E-06
clic-1	1.00	3.91E-04	1.07	1.03E-04
tram-1	1.00	3.78E-04	1.03	2.01E-04
dpy-11	0.99	4.64E-03	1.23	7.08E-04
ZK418.5	0.99	5.92E-03	1.18	1.26E-03
F52A8.5	0.99	6.30E-03	1.16	1.44E-03
K12H4.4	0.98	4.61E-03	1.03	2.52E-03
alp-1	0.98	8.90E-03	1.15	2.68E-03
F53H4.2	0.98	8.15E-03	0.98	7.81E-03
B0334.4	0.97	1.94E-03	0.77	8.27E-03
unc-54	0.96	2.23E-07	0.92	5.51E-07
kin-2	0.96	2.26E-03	0.97	1.51E-03
ZC395.10	0.95	4.71E-05	1.16	6.47E-07
ZC247.1	0.95	1.67E-03	1.43	1.92E-06
apl-1	0.95	7.79E-03	1.29	7.28E-04
F44D12.7	0.94	5.43E-03	1.45	2.82E-05
T13F2.2	0.94	9.96E-04	0.86	1.85E-03
cpl-1	0.93	1.33E-10	1.02	7.72E-13

T05H4.6a	0.93	2.82E-03	0.89	3.36E-03
T09E8.3	0.93	4.70E-03	0.83	7.55E-03
vha-4	0.92	2.69E-04	1.23	1.00E-06
R155.1	0.92	8.45E-03	0.85	9.60E-03
clec-1	0.91	2.88E-03	1.27	5.71E-05
ccdc-47	0.91	6.97E-03	1.03	2.67E-03
vha-8	0.90	7.58E-06	1.22	3.28E-10
col-12	0.90	7.94E-06	1.05	1.52E-07
arf-3	0.90	5.88E-06	0.92	2.30E-06
mlc-4	0.90	6.67E-04	1.16	1.38E-05
kin-19	0.90	6.51E-05	0.74	6.35E-04
C28H8.4	0.90	2.58E-03	0.83	4.23E-03
tag-18	0.90	3.16E-03	0.83	5.01E-03
cpi-1	0.88	2.91E-03	1.79	3.13E-10
col-13	0.88	5.28E-06	1.02	9.06E-08
T08B2.7	0.88	7.46E-03	1.28	2.35E-04
erm-1	0.88	8.15E-04	0.81	1.26E-03
lgg-1	0.87	1.41E-04	1.66	1.09E-14
dao-2	0.87	7.49E-03	1.76	7.03E-08
T27F7.3	0.87	7.98E-05	1.06	2.10E-06
iftb-1	0.87	2.87E-03	1.21	5.85E-05
T07A9.9	0.87	6.54E-03	0.94	3.73E-03
K09G1.1	0.86	5.60E-03	1.79	9.57E-09
F57B10.5	0.86	8.29E-03	1.10	1.45E-03
W10D9.5	0.86	9.04E-03	0.98	3.41E-03
hsp-6	0.85	3.82E-04	0.73	1.44E-03
col-180	0.85	4.70E-03	0.80	5.60E-03
vha-2	0.84	4.21E-08	1.10	2.03E-13
cct-5	0.84	2.70E-04	0.94	5.35E-05
rpt-4	0.84	5.02E-03	1.05	7.85E-04
hsp-25	0.84	5.45E-03	1.02	1.52E-03
F25B5.3	0.84	4.56E-03	0.88	2.80E-03
unc-60	0.84	2.12E-03	0.78	3.51E-03
let-2	0.83	3.83E-03	1.00	7.01E-04
LLC1.3	0.83	4.02E-03	0.87	2.53E-03
Y105E8B.11	0.82	9.89E-41	0.72	7.04E-32
pas-5	0.82	3.01E-03	1.11	9.39E-05
elo-5	0.82	2.14E-04	0.72	6.36E-04
mdt-28	0.81	1.51E-03	0.89	6.17E-04
C30C11.4	0.81	3.26E-04	0.74	6.48E-04
rab-1	0.81	3.53E-04	0.71	9.57E-04
C14C6.5	0.79	8.50E-03	1.23	1.28E-04
eif-3.F	0.79	7.03E-03	0.95	1.80E-03
vha-15	0.77	3.71E-03	1.13	6.01E-05
skr-1	0.76	2.83E-03	0.98	2.47E-04
W01A8.1b	0.76	1.95E-03	0.88	4.89E-04
rab-5	0.76	5.41E-03	0.84	2.75E-03

tct-1	0.75	6.23E-12	0.96	7.25E-20
R06C1.4	0.75	3.98E-05	0.74	3.47E-05
F58E10.3	0.75	6.69E-03	0.90	1.86E-03
F59C6.5	0.74	1.58E-03	1.32	5.00E-08
nuo-4	0.74	7.16E-03	1.15	1.16E-04
vha-1	0.74	5.73E-03	0.94	9.10E-04
C44B7.10	0.72	4.65E-03	1.23	6.91E-06
pmt-1	0.71	8.74E-03	1.48	4.77E-07
