# Supplementary material for: Gene expression profiling of oxidative stress response of C. elegans aging defective AMPK mutants using massively parallel transcriptome sequencing
Source: BMC Res Notes. 2011 Feb 8;4:34. doi: 10.1186/1756-0500-4-34 (PMC3045954; doi:10.1186/1756-0500-4-34)
Supplement: Additional file 9 — Supplementary Table S8. 125 genes up regulated in stressed aak-2, but insignificantly changed in stressed wild type and their fold changes of gene expression levels in stressed wild type and stressed aak-2 relative to wild type [file 1756-0500-4-34-S9.PDF]

Supplementary Table 8. 125 genes up regulated in stressed aak-2, but insignificantly changed in stressed wild type and their fold changes of gene expresssion levels in stressed wild type and stressed aak-2 relative to wild type

Gene	Log2(Stressed N2/N2)	Log2(Stressed aak-2/N2)	Gene Description (Concise)
B0280.17	0.43	2.28	
baf-1	0.50	0.81	baf-1 encodes a small, novel protein that is highly conserved amongst metazoa; BAF-1 binds double-stranded DNA nonspecifically in vitro and is essential for proper chromosome segregation, embryogenesis, and gonad development in vivo; biochemical and phenotypic analyses suggest that BAF-1 functions as an intramolecular bridge that mediates nuclear assembly and chromatin capture by the reforming nuclear envelope; by homology with mammalian BAF proteins, BAF-1 is also predicted to play a role in regulating gene expression and higher-order chromatin structure; during interphase, BAF-1 colocalizes with LMN-1/lamin to the nuclear envelope and nuclear interior; during mitosis, BAF-1 is present in a more punctate pattern, localizing near condensing chromatin and maintaining this localization through telophase; BAF-1 localization during nuclear assembly requires the activity of LMN-1/lamin, EMR-1/emerin, and LEM-2/MAN1.
brp-1	0.26	0.72	brp-1 encodes a glutamine-rich protein that is conserved in nematodes, but that has no known homologies or sequence motifs; BRP-1 is able to bypass the need for the S. cerevisiae mating pheromone MAP kinase cascade to activate the mating pheromone-responsive gene FUSl.
C01B4.6	0.07	2.49	
C01B4.9	-0.18	1.83	
C05C10.3	0.47	1.06	C05C10.3 is orthologous to the human gene 3-OXOACID COA TRANSFERASE (also called succinyl-CoA:3-ketoacid CoA transferase; OXCT; OMIM:245050), which when mutated leads to episodic ketoacidosis
C06A8.1	0.04	0.88	C06A8.1 is orthologous to human MSH HOMEO BOX HOMOLOG 1 (MTHFR; OMIM:142983, mutated in homocystinuria).
C06E7.1	0.33	0.78	
C07E3.9	0.47	1.69	
C08F11.12	0.36	1.39	
C09H5.2	0.38	2.42	
C10G8.4	0.17	0.85	C10G8.4 encodes a putative secreted TIL-domain protease inhibitor paralogous to SWM-1, ISL-1, and the products of 11 other C. elegans genes; C10G8.4 and its relatives are collectively similar to other TIL-domain protease inhibitors from nematodes, insects, and vertebrates; C10G8.4 has no obvious function in mass RNAi assays.
C15C8.3	0.45	0.94	
C16A3.10	0.45	0.79	The C16A3.10 gene encodes a homolog of the human gene OAT (OMIM:258870), which when mutated leads to ornithinemia and gyrate atrophy.
C17C3.1	0.46	2.41	
C31E10.7	0.21	0.83	C31E10.7 is orthologous to the human gene UNKNOWN (PROTEIN FOR MGC:10230) (CYB5; OMIM:250790), which when mutated leads to disease.
C33A12.19	0.00	2.12	
C42D4.1	0.05	1.14	
C45B2.2	-0.28	1.44	

C53A3.2	0.36	2.21	
ccg-1	0.38	1.43	
clec-52	0.18	3.12	
dad-1	0.39	0.77	dad-1 encodes an ortholog of human DAD1 (OMIM:600243) that inhibits cell death and can functionally complement the hamster protein in cultured cells.
ech-6	0.03	1.33	
F07H5.5	-0.05	0.89	
F08B12.4	-0.17	0.84	
F08G5.6	0.18	2.00	
F10G2.1	0.42	1.28	
F13H8.7	0.41	1.11	
F15B10.1	0.33	1.12	The F15B10.1 gene encodes an ortholog of the Kluyveromyces lactis UDP-N-acetylglucosamine (UPD-GlcNAc) transporter; when expressed in human cells deficient for GDP-fucose transport into the Golgi, F15B10.1 is able to weakly restore such transport (with ~10% efficiency).
F18E3.11	0.30	1.44	
F22B7.9	0.49	3.81	
F25E5.8	0.45	2.06	
F28A10.6	0.30	1.23	
F32B5.1	0.30	0.98	
F35E12.5	-0.12	2.25	
F40F12.7	0.44	1.57	
F41C3.5	0.38	0.90	
F44A6.5	0.42	3.14	
F44E7.2	0.22	1.61	
F52A8.1	0.47	0.96	
F52E1.14	0.47	0.90	
F53A9.8	-1.09	0.82	
F54C9.3	0.06	1.04	
F54D5.12	0.27	1.27	
F54E2.1	0.42	1.23	
F55H12.2	0.42	1.72	
F56A4.2	-0.67	2.32	
F56A4.3	0.19	1.90	
F56C9.7	0.21	0.73	
far-6	0.22	1.03	
far-8	0.35	1.10	
fat-4	0.49	1.14	fat-4 encodes a delta-5 fatty acid desaturase; when expressed heterologously in S. cerevisiae, FAT-4 converts di-homo-gamma-linolenic acid (20:3delta8,11,14) to arachidonic acid (20:4delta5,8,11,14), an important precursor in eicosanoid metabolism, as well as on other fatty acids (C18 to C20); FAT-4 is is required in vivo for delta-5 unsaturated fatty acid synthesis
fat-5	-0.84	1.42	fat-5 encodes a delta-9 fatty acid desaturase that is predicted to be mitochondrial; when expressed heterologously in S. cerevisiae, FAT-5 rescues the fatty acid auxotrophy of the yeast delta-9 desaturase mutant ole1
fat-6	-0.13	1.11	fat-6 encodes a delta-9 fatty acid desaturase that is predicted to be mitochondrial.
gdi-1	0.46	0.84	gdi-1 encodes a member of the Rab GDP dissociation inhibitor (GDI) family with high similarity to Drosophila Gdi; expressed around the pharyngeal, vulval, and tail regions.

glt-1	0.19	0.94	glt-1 encodes a predicted plasma membrane glutamate transporter that is functional when expressed in Xenopus oocytes; expressed in the M3 pharyngeal neuron, the male tail, some anterior hypodermal cells, and in cells in the terminal bulb of the pharynx.
gta-1	0.16	0.89	The gta-1 gene encodes an ortholog of the human gene GABAT, which when mutated leads to GABA-transaminase deficiency (OMIM:137150).
hpd-1	0.42	1.25	hpd-1 encodes a 4-hydroxyphenylpyruvate dioxygenase that is required for normally short lifespan and negative regulation of the dauer larval stage, with loss of hpd-1 function prolonging lifespan and promoting dauer formation; hpd-1 is a (perhaps evolutionarily conserved) target of transcriptional activation by DAF-16; HPD-1 is orthologous to human HPD (OMIM:276710, mutated in tyrosinemia type III).
hsp-17	0.37	1.28	hsp-17 encodes a heat shock protein that is a member of the hsp16/hsp20/alpha-crystallin family of heat shock proteins; by homology, HSP-17 is predicted to function as a molecular chaperone that protects cells from heat-induced protein aggregation and denaturation; at present, the precise developmental and/or behavioral role of HSP-17, as well as its expression pattern, are not yet known.
K07C11.7	0.41	1.42	
K09H11.7	0.41	2.10	
ldh-1	0.38	1.22	ldh-1 is orthologous to the human gene LACTATE DEHYDROGENASE B (LDHB; OMIM:150100), which when mutated leads to lactate dehydrogenase-B deficiency.
lea-1	0.00	0.85	The lea-1 gene encodes a protein that is predicted to be hydrophilic and heat-resistant, and that might participate in anhydrobiosis.
let-721	0.34	1.19	The let-721 gene encodes an ortholog of the human gene SIMILAR TO ELECTRON-TRANSFERRING-FLAVOPROTEIN DEHYDROGENASE (ETFDH), which when mutated leads to glutaricaciduria type IIC (OMIM:231675).
lpl-1	0.49	1.01	The lpl-1 gene encodes a lipoate ligase homolog; this gene resides in an operon that also contains the genes icl-1 and C01F6.9.
M02D8.1	0.49	0.84	
M60.4	0.50	1.20	
mmaa-1	0.19	1.06	
MTCE.33	0.39	1.67	
mua-6	0.40	0.96	mua-6 encodes an essential intermediate filament protein (MUA-6/IFA-2) that is coexpressed with the essential IF protein IFB-1; MUA-6 is required for hypodermal integrity and for lasting attachment of muscles to the body wall; MUA-6 is also required for normal positioning of excretory canals and muscles; MUA-6 forms heteropolymeric intermediate filaments in vitro with an equimolar mixture of IFB-1; mua-6 is transcribed from L1 larval to adult stages; MUA-6 resides in main body hypodermal desmosomes, but not in seam cells.
ncs-2	0.50	0.82	

nlp-29	0.21	2.15	nlp-29 encodes a neuropeptide-like protein; nlp-29 appears to play a role in innate immunity, as its expression is strongly induced following bacterial and fungal infection; nlp-29 is expressed in the hypodermis and in the intestine; nlp-29 expression is regulated by TIR-1, an ortholog of SARM, a Toll-interleukin 1 receptor (TIR) domain protein, and by the RAB-1 GTPase and the product of R53.4, a mitochondrial ATP synthase subunit f homolog.
npa-1	0.41	1.12	npa-1 encodes a large polyprotein precursor that is posttranslationally cleaved to multiple units of ~14.5kDa, each of which is a strong binding protein for fatty acids and retinol (Vitamin A); the lipid binding sites of NPA-1-derived peptides are unusually apolar; NPA-1-derived peptides probably are carrier proteins that enable distribution of these lipids within nematodes; NPA-1-derived peptides are also secreted by parasitic nematode species.
nspa-1	-0.55	0.73	
nspc-10	0.37	0.94	
nspc-17	0.41	1.59	
nspc-20	0.50	1.46	
nspc-3	0.06	1.12	
nuo-1	0.50	0.85	nuo-1 encodes a encodes a 51 kDa subunit of mitochondrial complex I that is required for oxidative phosphorylation, resistance to volatile anesthetics, and progression through development; nuo-1 is orthologous to human NDUFV1 (OMIM:161015, mutated in Leigh syndrome); nuo-1(ua1) induces a developmental arrest at the third larval (L3) stage that blocks reproduction, and thus is lethal to a population homozygous for nuo-1(ua1); yet arrested L3 nuo-1(ua1) animals have an individual lifespan significantly longer than normal.
pat-10	0.29	0.84	pat-10 encodes body wall muscle troponin C, the calcium-binding component of the troponin complex of actin thin filaments; PAT-10 is essential for muscle contraction and thus for completion of embryonic morphogenesis and elongation; by homology, PAT-10 likely functions to regulate body wall muscle contraction in response to changes in intracellular calcium; PAT-10 is expressed in body wall muscle but is also detected in vulval and anal muscles; expression in body wall muscle begins during early embryonic morphogenesis, concurrent with expression of other body wall muscle structural components.
pes-9	0.33	0.93	pes-9 encodes a zinc metallopeptidase; pes-9 was originally identified in a promoter trap screen for sequences that direct reporter gene expression in specific cell types during C. elegans development; pes-9 sequences direct expression in a limited number of cells in the early embryo and in putative head and tail hypodermal cells in adults; after some confusion, the pes-9 reporter gene fusion was mapped to [illegible]
plp-1	0.31	0.75	plp-1 encodes a protein containing three PUR repeats that are predicted to bind DNA/RNA and has similarity to the mammalian transcription factor pur alpha, and is required for embryonic development, development of the pharynx, and affects formation of the intestine; interacts with an Lef-1 site within the end-1 promoter and binding is [illegible]
pmp-5	0.42	2.37	
prdx-3	0.19	0.73	

qdpr-1	0.45	1.24	The T03F6.1 gene encodes an ortholog of the human gene QUINOID DIHYDROPTERIDINE REDUCTASE (QDPR; DHPR), which when mutated leads to phenylketonuria II (OMIM:261630).
R02D3.1	0.19	1.27	The R02D3.1 gene encodes an ortholog of the human gene ALPHA-AMINOADIPATE SEMIALDEHYDE SYNTHASE (AASS; OMIM:605113), which when mutated leads to hyperlysinemia (OMIM:238700).
R03D7.1	0.05	1.61	R03D7.1 is orthologous to the human gene METHIONINE SYNTHASE (MTR; OMIM:156570), which when mutated leads to disease.
R05D11.5	0.42	1.06	
R05F9.6	0.18	1.00	
R107.5	0.50	1.06	
R11A5.4	-0.03	1.08	
rhr-1	-0.09	0.75	rhr-1 encodes an ortholog of human Rhesus blood-group associated glycoprotein (RHAG; OMIM:180297, mutated in chronic hemolytic anemia), a member of the ammonium transporter family, and affects general levels of mRNA transcripts, and embryonic viability in a large-scale RNAi screen
sel-9	0.47	1.24	sel-9 encodes a member of the p24 family of proteins that affects growth and locomotion and likely functions to negatively regulate the transport of lin-12 and glp-1 to the cell surface; genetically interacts with mutations in lin-12 that affect the extracellular domain with respect to egg laying, AC/VU fate decision, and vulval formation and interacts with glp-1 mutations with respect to proximal proliferation of the germline and embryonic viability.
sod-1	0.34	0.81	sod-1 encodes the copper/zinc superoxide dismustase, an enzyme that is known to protect cells from oxidative damage; superoxide dismutase activity can be detected in worm extracts; sod-1 activity has been implicated in the increased life-span of dauer larvae where this enzyme demonstrates the highest activity compared to other life-stages as well as in the increased life span of age-1 mutants and their resistance to oxidative damage; sod-1 modulates the effect of let-60 ras on vulval and germline development via cytoplasmic reactive oxygen species; unlike other eukaryotic superoxide dismutases, sod-1 does not require the copper chaperone CCS for its activity and instead uses a glutathione pathway for acquiring copper; In humans, mutation of SOD1 (OMIM:147450) leads to amyotrophic lateral sclerosis (OMIM:105400).
spp-10	0.29	0.77	spp-10 encodes two protein isoforms that are orthologous to the human gene PROSAPOSIN (PSAP; OMIM:176801, mutated in Gaucher disease and metachromatic leukodystrophy); SPP-10A and -10B are predicted to have 3 and 4 embedded saposin sequences, that are likely to be split into individual saposin peptides by proteolysis; in mammals, saposins activate the enzymes sphingomyelinphosphodiesterase and beta-glucosylceramidase; spp-10 has no obvious function in mass RNAi assays.

sqv-4	0.49	0.91	sqv-4 encodes a UDP-glucose 6-dehydrogenase, biochemically active in vitro, that is required for cytokinesis of one-cell embryos and for vulval morphogenesis; SQV-4 is orthologous to Drosophila SUGARLESS, human UGDH (OMIM:603370), and zebrafish JEKYLL; SQV-4 is expressed in many cell types, notably oocytes and uterine, seam, pharyngeal, and spermathecal cells, with expression being prominent in 14 of the 22 vulval nuclei of late L4 larvae; the common requirement for SQV-4 in both cytokinesis and morphogenesis may be to promote filling an extracellular space with hygroscopic proteoglycans (either in the eggshell, or underneath the L4 cuticle), which in turn may cause the space to fill with fluid.
T01C8.2	0.44	1.21	
T15B7.2	0.16	0.85	
T20G5.8	-0.09	0.94	
T25B9.9	0.32	0.76	
T28F4.5	0.01	1.50	The T28F4.5 gene encodes a homolog of Death Associated Protein 1 (DAP-1) protein that may be involved in apoptosis.
tag-174	-0.03	0.87	
tat-4	0.38	0.98	
vha-14	0.39	0.94	vha-14 encodes an ortholog of subunit D of the cytoplasmic (V1) domain of vacuolar proton-translocating ATPase (V-ATPase); VHA-14 is a predicted cytosolic rotor (stalk) component.
W05H9.1	0.25	1.43	
W10C8.5	0.18	0.87	
Y19D10A.12	-0.18	1.83	
Y19D10A.16	0.07	2.49	
Y37A1B.5	-0.22	1.64	
Y38C1AA.7	0.18	1.37	
Y38E10A.13	-0.70	1.18	
Y38E10A.14	-0.47	3.78	
Y38F1A.6	0.39	1.69	
Y43C5A.2	0.39	0.97	
Y48G8AL.13	0.34	1.57	
Y51A2D.14	0.19	1.01	
Y62E10A.13	0.22	1.17	
ZK637.2	0.48	0.83	
ZK970.7	-0.15	3.36	
clec-209	-0.67	2.32	
rmd-2	0.19	0.78	
scl-2	0.18	1.67	
ttr-36	0.05	0.98	
ttr-47	0.08	1.18	
