# Supplementary material for: Gene expression profiling of oxidative stress response of C. elegans aging defective AMPK mutants using massively parallel transcriptome sequencing
Source: BMC Res Notes. 2011 Feb 8;4:34. doi: 10.1186/1756-0500-4-34 (PMC3045954; doi:10.1186/1756-0500-4-34)
Supplement: Additional file 10 — Supplementary Table S9. 125 gene up-regulated in stressed aak-2 relative to wild type but insignificantly changed in stressed wild type relative to wild type and most highly represented biological processes these genes are involved in [file 1756-0500-4-34-S10.PDF]

Supplementary Table 9. 125 gene up-regulated in stressed aak-2 relative to wild type but insignificantly changed in stressed wild type relative to wild type and most highly represented biological processes these genes are involved in

GO	Genes	Pvalue	GO as name
GO:0032787	fat-5; fat-4; r11a5.4; gta-1; c17c3.1; r03d7.1; fat-6	5.50E-06	monocarboxylic acid metabolic process;
GO:0019752	fat-4; r11a5.4; fat-6; y62e10a.13; lpl-1; fat-5; hpd-1; gta-1; c06a8.1; r03d7.1; c17c3.1	5.50E-06	carboxylic acid metabolic process;
GO:0006082	fat-4; r11a5.4; fat-6; y62e10a.13; lpl-1; fat-5; hpd-1; gta-1; c06a8.1; r03d7.1; c17c3.1	5.50E-06	organic acid metabolic process;
GO:0006631	fat-5; fat-4; gta-1; c17c3.1; fat-6	0.000325	fatty acid metabolic process;
GO:0044255	spp-10; fat-4; c07e3.9; fat-6; fat-5; let-721; gta-1; c17c3.1	0.00139	cellular lipid metabolic process;
GO:0001676	fat-5; fat-6	0.00139	long-chain fatty acid metabolic process;
GO:0006555	c06a8.1; r03d7.1	0.00139	methionine metabolic process;
GO:0042759	fat-5; fat-6	0.00139	long-chain fatty acid biosynthetic process;
GO:0016053	fat-5; fat-4; fat-6	0.00211	organic acid biosynthetic process;
GO:0046394	fat-5; fat-4; fat-6	0.00211	carboxylic acid biosynthetic process;
GO:0006633	fat-5; fat-4; fat-6	0.00211	fatty acid biosynthetic process;
GO:0006629	spp-10; fat-4; c07e3.9; fat-6; fat-5; let-721; gta-1; c17c3.1	0.0123	lipid metabolic process;
GO:0009066	c06a8.1; r03d7.1	0.0123	aspartate family amino acid metabolic process;
GO:0006732	lpl-1; vha-14; t25b9.9; c17c3.1; r03d7.1	0.0123	coenzyme metabolic process;
GO:0000096	c06a8.1; r03d7.1	0.0152	sulfur amino acid metabolic process;
GO:0008610	fat-5; let-721; fat-4; fat-6	0.0236	lipid biosynthetic process;
GO:0006006	ldh-1; r11a5.4; t25b9.9	0.0248	glucose metabolic process;
GO:0006790	c06a8.1; r03d7.1	0.0422	sulfur metabolic process;
GO:0051186	lpl-1; vha-14; t25b9.9; c17c3.1; r03d7.1	0.0422	cofactor metabolic process;
GO:0006519	y62e10a.13; hpd-1; gta-1; c06a8.1; r03d7.1	0.0422	amino acid and derivative metabolic process;
GO:0019318	ldh-1; r11a5.4; t25b9.9	0.0474	hexose metabolic process;
GO:0005996	ldh-1; r11a5.4; t25b9.9	0.0474	monosaccharide metabolic process;
GO:0044249	fat-4; vha-14; r11a5.4; fat-6; y62e10a.13; lpl-1; fat-5; let-721; r03d7.1	0.0474	cellular biosynthetic process;
GO:0019430	sod-1	0.0626	removal of superoxide radicals;
GO:0001306	sod-1	0.0626	age-dependent response to oxidative stress;
GO:0007571	sod-1	0.0626	aging#age-dependent general metabolic decline;
GO:0009086	r03d7.1	0.0626	methionine biosynthetic process;
GO:0019642	ldh-1	0.0626	anaerobic glycolysis;
GO:0005975	ldh-1; y19d10a.16; r05f9.6; r11a5.4; c01b4.6; t25b9.9	0.0804	carbohydrate metabolic process;

GO:0006118	let-721; nuo-1; r02d3.1; f28a10.6; sqv-4; tag-174	0.0804	electron transport;
GO:0008652	y62e10a.13; r03d7.1	0.0804	amino acid biosynthetic process;
GO:0006767	let-721; t25b9.9	0.0804	water-soluble vitamin metabolic process;
GO:0006766	let-721; t25b9.9	0.0804	vitamin metabolic process;
GO:0006520	y62e10a.13; hpd-1; c06a8.1; r03d7.1	0.0804	amino acid metabolic process;
GO:0009308	y62e10a.13; hpd-1; gta-1; c06a8.1; r03d7.1	0.0804	amine metabolic process;
GO:0009108	lpl-1; vha-14; r03d7.1	0.0804	coenzyme biosynthetic process;
GO:0009448	gta-1	0.0804	gamma-aminobutyric acid metabolic process;
GO:0000273	lpl-1	0.0804	lipoic acid metabolic process;
GO:0009105	lpl-1	0.0804	lipoic acid biosynthetic process;
GO:0046459	gta-1	0.0804	short-chain fatty acid metabolic process;
GO:0019605	gta-1	0.0804	butyrate metabolic process;
GO:0009107	lpl-1	0.0804	lipoate biosynthetic process;
GO:0006564	y62e10a.13	0.0804	L-serine biosynthetic process;
GO:0009106	lpl-1	0.0804	lipoate metabolic process;
