# Supplementary material for: Gene expression profiling of oxidative stress response of C. elegans aging defective AMPK mutants using massively parallel transcriptome sequencing
Source: BMC Res Notes. 2011 Feb 8;4:34. doi: 10.1186/1756-0500-4-34 (PMC3045954; doi:10.1186/1756-0500-4-34)
Supplement: Additional file 11 — Supplementary Table S10. Genes that are significantly up-regulated in unstressed aak-2 mutants compared to wild type animals [file 1756-0500-4-34-S11.PDF]

Supplementary Table 10. genes that are significantly up-regulated in unstressed aak-2 mutants compared to wild type animals

Gene	Log2 (Unstressed aak-2/Unstressed N2)	p-val
Y38E10A.15	5.02	1.06E-08
fat-7	4.23	2.89E-11
C01B4.7	3.52	3.01E-03
Y19D10A.4	3.52	3.01E-03
cnc-7	3.38	5.49E-04
lys-6	3.19	1.55E-03
lys-5	3.17	1.69E-03
F10D2.10	3.11	3.00E-09
Y19D10A.12	2.96	1.82E-27
C01B4.9	2.95	6.25E-26
nhr-114	2.62	8.19E-03
C01B4.6	2.59	3.01E-03
Y102A5C.6	2.59	3.52E-04
Y19D10A.16	2.59	3.00E-03
pmp-5	2.58	9.80E-09
acdh-1	2.56	9.38E-42
Y38E10A.14	2.49	7.49E-12
F32D8.12	2.46	4.85E-03
MTCE.22	2.42	9.11E-03
lys-4	2.41	1.84E-12
F28A12.4	2.39	1.67E-16
D1086.11	2.20	4.43E-11
dpy-17	2.15	4.64E-03
K07C11.7	2.11	4.78E-06
Y102A5C.5	2.07	4.08E-03
Y69H2.3	2.04	1.41E-06
cpr-1	2.02	3.71E-10
Y69H2.3a	2.01	1.51E-06
cah-4	1.97	1.09E-11
ZK632.2	1.95	2.85E-03
C33A12.19	1.91	4.97E-03
clec-209	1.89	9.13E-81
F56A4.2	1.89	5.81E-81
Y38F1A.6	1.89	5.10E-12
F09F7.4	1.79	1.40E-08
fbxa-72	1.75	4.37E-03
mxl-3	1.75	5.91E-03
eel-1	1.74	1.10E-03
F45D3.4a	1.74	5.19E-03
T09B4.5	1.73	8.01E-03
F18E2.1	1.72	5.00E-03
frm-1	1.70	4.45E-04

H06I04.3	1.70	2.72E-03
prg-2	1.68	1.84E-03
ZK546.14	1.68	9.00E-04
C01G6.6b	1.65	7.83E-04
C55B7.1	1.64	2.36E-03
F45D3.4	1.64	6.76E-03
C09D4.2	1.63	7.28E-03
prpf-4	1.63	2.04E-03
vab-10	1.63	3.13E-03
sptl-3	1.61	9.31E-03
K08F8.1e	1.60	1.60E-03
K08F8.1	1.59	1.02E-03
clh-1	1.58	7.97E-03
K07H8.10	1.58	6.60E-07
C09G4.2	1.56	3.49E-03
nas-20	1.56	3.35E-03
B0495.2	1.55	1.98E-03
lfi-1	1.53	7.79E-03
tag-310	1.53	9.12E-03
F53H4.2	1.52	3.19E-05
nmy-1	1.52	1.89E-03
clec-150	1.51	1.04E-06
K08F8.1a	1.51	3.32E-03
MTCE.35	1.50	8.90E-15
C01G6.6a	1.49	1.90E-03
C13B7.6	1.48	7.74E-05
T23G7.3	1.48	6.40E-03
tag-165	1.48	2.67E-03
Y45G12C.16	1.47	9.83E-05
rfc-1	1.46	8.71E-03
bcat-1	1.43	1.17E-05
pqn-44	1.43	9.80E-03
H37A05.1	1.42	1.56E-04
MTCE.33	1.41	1.03E-03
ppn-1	1.41	2.00E-04
vha-11	1.41	1.13E-06
hsp-16.11	1.39	3.08E-03
Y71H2AM.19	1.39	3.94E-03
hsp-16.1	1.38	8.94E-04
K07C5.6	1.38	5.23E-03
C18H9.3	1.37	5.43E-03
clp-1	1.37	6.30E-03
ced-7	1.35	2.66E-03
K08H10.2	1.35	1.10E-03
Y75B8A.13	1.35	3.34E-03
rrn-3.56	1.34	2.27E-146
aqp-8	1.33	8.76E-03

smc-4	1.33	3.04E-03
T20D3.3	1.33	8.41E-03
Y61A9LA.8	1.33	8.76E-03
aqp-2	1.31	2.46E-03
lea-1	1.31	5.56E-06
col-95	1.30	9.64E-11
ifb-1	1.30	1.22E-05
mup-4	1.30	6.73E-03
C14F11.5	1.29	2.08E-03
D1054.10	1.29	1.89E-14
vha-5	1.29	9.41E-04
ZK836.2	1.29	2.89E-03
ZK1127.9	1.28	2.61E-03
F42D1.2	1.27	2.19E-03
glh-1	1.27	7.27E-05
hex-1	1.26	6.47E-03
R10D12.14	1.26	1.23E-04
unc-52	1.26	2.87E-03
F29B9.8	1.25	8.05E-03
ZK1127.6	1.25	3.71E-03
cgh-1	1.24	7.70E-28
MTCE.25	1.24	3.08E-13
cpl-1	1.23	3.68E-21
eif-3.I	1.23	1.31E-03
F58G11.2	1.23	7.62E-03
K04E7.2	1.23	3.54E-03
vit-6	1.23	1.98E-91
Y22D7AL.5	1.23	1.18E-04
B0041.2	1.22	6.68E-03
F09F7.3	1.22	3.18E-03
F15D3.6	1.22	2.15E-03
F21D5.7	1.22	5.42E-03
T05H10.1	1.22	2.98E-03
T08G11.1	1.21	5.10E-03
T10F2.2	1.21	5.81E-03
T23F11.1	1.21	1.95E-03
ZC262.3	1.21	2.54E-03
C39E9.8	1.20	6.79E-03
D2096.11	1.20	9.46E-03
Y71H10B.1	1.20	6.27E-03
F28A10.6	1.19	4.94E-03
F44G4.1	1.19	7.06E-03
mdt-27	1.19	6.66E-03
pqn-45	1.19	6.24E-06
puf-12	1.19	1.62E-03
vha-4	1.19	5.07E-07
ama-1	1.18	7.99E-03

cki-2	1.18	3.20E-03
clec-222	1.18	5.99E-03
F46H5.7	1.18	7.19E-03
H28G03.1	1.18	7.99E-03
R02D3.1	1.18	4.24E-03
Y66H1B.5	1.18	2.57E-04
apl-1	1.17	9.95E-04
cey-1	1.17	1.61E-05
col-20	1.17	8.21E-23
egl-30	1.17	1.73E-03
gsk-3	1.17	3.00E-03
K09H11.7	1.17	5.65E-03
rrn-3.1	1.17	5.61E-200
vps-26	1.17	3.78E-03
ZK105.1	1.17	1.62E-03
abcf-1	1.16	4.04E-03
C53A3.2	1.16	8.87E-03
ergo-1	1.16	2.10E-03
F54D5.12	1.16	1.94E-03
R11A8.7	1.16	4.24E-03
T26C5.3	1.16	5.65E-03
C27A2.1	1.15	6.67E-03
rfp-1	1.15	4.60E-03
Y66H1B.2	1.15	3.90E-06
uaf-1	1.14	4.96E-03
Y66H1B.3	1.14	2.84E-03
B0416.5	1.13	4.65E-03
bath-43	1.13	6.37E-03
C05C10.3	1.13	4.57E-03
clec-48	1.13	6.86E-03
D2005.1	1.13	7.91E-03
F28B3.5	1.13	6.82E-03
unc-43	1.13	9.98E-03
C36B1.7	1.12	5.36E-03
C52E12.1	1.12	9.98E-03
hrp-2	1.12	5.31E-03
lin-35	1.12	6.41E-03
par-1	1.12	9.85E-03
sago-2	1.12	1.87E-03
Y105C5B.15	1.12	6.15E-03
C06E7.3	1.11	7.41E-05
col-143	1.11	3.52E-15
Y105C5B.12	1.11	2.60E-03
adr-2	1.10	6.27E-03
aex-5	1.10	9.15E-07
C27B7.9	1.10	6.19E-03
dnj-12	1.10	1.05E-03

E01A2.4	1.10	9.14E-03
F36A2.13	1.10	5.32E-03
mua-6	1.10	1.20E-03
npp-10	1.10	2.78E-03
ntl-4	1.10	1.57E-03
pqe-1	1.10	8.69E-03
vha-8	1.10	6.67E-09
Y41E3.1	1.10	9.37E-03
B0285.1	1.09	6.77E-03
csn-2	1.09	4.78E-03
D1054.11	1.09	1.41E-13
mel-32	1.09	9.99E-08
R04F11.3	1.09	1.03E-03
T15H9.1	1.09	1.04E-04
unc-57	1.09	1.23E-03
W06A7.4	1.09	1.40E-05
Y41D4A.5	1.09	1.43E-03
C53H9.2	1.08	8.38E-04
dao-5	1.08	5.96E-06
lst-3	1.08	2.81E-03
mdt-15	1.08	6.11E-03
R03D7.1	1.08	9.94E-04
sec-24.2	1.08	7.83E-03
tag-182	1.08	5.33E-03
unc-32	1.08	2.97E-03
unc-54	1.08	1.61E-10
Y45F10C.2	1.08	5.95E-05
C36B1.8	1.07	3.28E-03
smk-1	1.07	1.32E-03
Y51H7C.1	1.07	5.06E-03
C06E7.1	1.06	4.72E-12
C55C3.5	1.06	6.16E-03
F58E10.3	1.06	2.88E-04
pmt-1	1.06	2.25E-04
ppw-1	1.06	3.90E-03
ret-1	1.06	5.94E-14
zip-2	1.06	5.93E-03
dnj-11	1.05	1.50E-03
E04F6.5	1.05	9.35E-03
F08F8.9	1.05	9.92E-03
F30A10.10	1.05	7.21E-03
kin-19	1.05	5.77E-07
rrn-1.1	1.05	2.42E-149
rrn-1.2	1.05	2.42E-149
T23E7.2	1.05	2.52E-03
tram-1	1.05	3.00E-04
vha-15	1.05	6.55E-05

cpt-2	1.04	6.84E-03
cpz-1	1.04	6.40E-05
pqn-22	1.04	5.65E-03
tag-60	1.04	5.09E-03
ZK484.1	1.04	8.68E-11
apb-1	1.03	8.93E-03
cct-5	1.03	2.14E-06
F26B1.2	1.03	9.80E-05
let-721	1.03	8.46E-03
rhr-1	1.03	5.67E-05
B0035.12	1.02	8.03E-03
cid-1	1.02	6.42E-03
D1086.6	1.02	1.62E-03
daf-16	1.02	5.66E-03
kin-2	1.02	5.60E-04
mel-26	1.02	2.20E-04
npp-21	1.02	8.81E-03
Y37E11AL.3	1.02	5.98E-03
zfp-1	1.02	8.21E-03
dnj-18	1.01	9.82E-03
F21D5.2	1.01	5.90E-03
gld-2	1.01	4.25E-03
goa-1	1.01	4.24E-04
mca-3	1.01	2.01E-03
npp-8	1.01	9.36E-03
spk-1	1.01	1.83E-03
Y110A7A.6	1.01	6.31E-03
ZK858.1	1.01	5.72E-04
arf-1.2	1.00	3.46E-06
ldh-1	1.00	7.94E-03
lin-41	1.00	1.07E-03
R10D12.13	1.00	6.85E-03
trs-1	1.00	6.13E-04
vha-13	1.00	1.59E-07
vha-16	1.00	7.19E-06
E01A2.2	0.99	9.14E-03
gpi-1	0.99	1.82E-03
ifg-1	0.99	3.98E-04
ima-3	0.99	4.34E-06
mmaa-1	0.99	4.56E-03
sams-1	0.99	1.15E-05
Y71F9AL.17	0.99	4.11E-03
ZC247.1	0.99	7.24E-04
atn-1	0.98	7.44E-03
clu-1	0.98	9.72E-04
cmd-1	0.98	1.15E-06
cpr-6	0.98	4.02E-10

csnk-1	0.98	9.13E-03
csr-1	0.98	4.30E-05
F42G9.1	0.98	4.28E-03
glt-1	0.98	3.70E-03
let-2	0.98	6.14E-04
lgg-1	0.98	9.39E-06
nos-3	0.98	1.33E-03
nuo-1	0.98	6.44E-04
T24H7.2	0.98	8.38E-03
taf-13	0.98	5.97E-03
unc-94	0.98	8.75E-03
Y54G2A.2	0.98	6.40E-03
anc-1	0.97	2.29E-03
B0250.5	0.97	5.28E-03
col-106	0.97	3.85E-12
crs-1	0.97	9.87E-03
F20G4.3	0.97	8.11E-04
M01H9.3	0.97	3.36E-03
unc-15	0.97	1.20E-08
vha-14	0.97	4.41E-05
wnk-1	0.97	8.28E-03
B0511.6	0.96	3.59E-03
C25A8.4	0.96	1.33E-03
col-178	0.96	1.52E-26
egl-27	0.96	3.85E-03
F35G12.2	0.96	1.25E-03
F44E7.2	0.96	7.47E-03
F49E8.1	0.96	7.89E-04
F55C5.8	0.96	2.48E-03
inx-14	0.96	3.59E-03
pgl-1	0.96	4.09E-04
T01G1.3	0.96	6.95E-03
T05H4.6a	0.96	9.91E-04
T08B2.7	0.96	2.54E-03
vig-1	0.96	2.23E-16
asp-2	0.95	3.77E-11
C16A3.10	0.95	2.17E-04
col-179	0.95	9.47E-16
daz-1	0.95	1.75E-06
E01A2.6	0.95	5.64E-05
math-33	0.95	2.34E-03
pod-2	0.95	7.26E-03
pqn-53	0.95	8.44E-03
ptc-2	0.95	1.05E-03
rsks-1	0.95	5.66E-03
T20D3.11	0.95	4.74E-03
vha-10	0.95	7.17E-05

W01A8.1b	0.95	1.25E-04
xpo-3	0.95	7.20E-03
Y65B4BL.5	0.95	7.08E-05
Y71F9AL.9	0.95	8.38E-07
Y71H2AM.20	0.95	6.96E-03
C06A5.6	0.94	5.50E-03
C18B2.5	0.94	9.89E-03
C30H7.2	0.94	6.52E-03
C50F7.4	0.94	5.78E-03
ech-6	0.94	1.06E-06
emb-5	0.94	4.24E-03
hif-1	0.94	7.45E-03
hpd-1	0.94	2.50E-03
hsp-6	0.94	3.47E-05
mdt-28	0.94	1.61E-04
pde-2	0.94	3.66E-03
pme-1	0.94	8.01E-03
pyc-1	0.94	1.38E-03
T24G10.2	0.94	5.57E-03
ZK973.1	0.94	5.32E-03
aka-1	0.93	2.87E-03
ccdc-47	0.93	3.82E-03
F45F2.10	0.93	6.25E-03
F45H11.3	0.93	4.66E-03
fib-1	0.93	6.09E-04
iffb-1	0.93	8.81E-03
irs-1	0.93	6.74E-03
K02F2.2	0.93	3.23E-17
K09G1.1	0.93	2.45E-03
klc-1	0.93	7.92E-03
sup-17	0.93	5.23E-03
vit-4	0.93	3.38E-152
C23G10.7	0.92	8.59E-03
C23G10.8	0.92	9.52E-03
erm-1	0.92	2.41E-04
F52C6.12	0.92	6.12E-03
F52E1.13	0.92	1.62E-03
F53A2.7	0.92	6.45E-03
let-60	0.92	3.29E-03
mom-5	0.92	5.55E-03
pap-1	0.92	6.19E-03
R13F6.10	0.92	7.98E-03
rpb-2	0.92	9.52E-03
scpl-1	0.92	7.02E-03
snap-1	0.92	6.66E-03
tag-93	0.92	1.23E-03
top-1	0.92	2.67E-03

vit-1	0.92	1.62E-41
vit-3	0.92	2.42E-144
wwp-1	0.92	4.16E-03
Y71F9AM.6	0.92	2.08E-05
ZK484.1.5	0.92	3.60E-08
C08F11.12	0.91	2.23E-04
C18A3.5	0.91	1.42E-03
C34D4.14	0.91	9.87E-03
ccf-1	0.91	8.30E-04
dyci-1	0.91	6.94E-03
F58G1.1	0.91	5.28E-03
imb-3	0.91	4.12E-06
pqn-20	0.91	3.63E-03
prom-1	0.91	8.47E-03
sax-7	0.91	4.44E-03
T19A6.1	0.91	3.53E-03
tat-5	0.91	4.98E-03
tre-1	0.91	6.00E-03
ZK1320.9	0.91	7.56E-03
zyg-11	0.91	3.90E-03
zyx-1	0.91	7.23E-03
aco-2	0.90	5.59E-05
C36E8.1	0.90	9.29E-03
C37H5.6	0.90	5.38E-04
C42C1.8	0.90	6.12E-03
C49H3.9	0.90	9.78E-03
ceh-38	0.90	7.68E-03
col-119	0.90	2.84E-26
lig-1	0.90	8.69E-03
lrs-1	0.90	5.68E-03
M03F4.6	0.90	5.67E-04
mut-16	0.90	7.49E-03
nud-2	0.90	3.94E-03
Y59A8A.3	0.90	1.07E-03
C44B7.5	0.89	5.23E-03
cct-7	0.89	4.24E-04
F32E10.6	0.89	3.29E-03
F46E10.1	0.89	7.25E-03
K10C2.1	0.89	4.08E-03
tnt-2	0.89	2.30E-06
vbh-1	0.89	1.16E-03
Y45G5AL.1	0.89	5.99E-03
ZK484.1.3	0.89	1.13E-07
act-4	0.88	5.56E-25
brp-1	0.88	2.70E-04
C01G6.5	0.88	4.87E-03
C28H8.3	0.88	2.23E-03

D2030.7	0.88	9.82E-03
dim-1	0.88	4.16E-03
eif-3.C	0.88	1.90E-04
ent-2	0.88	7.01E-03
F37C12.7	0.88	3.25E-05
F59E12.11	0.88	9.49E-03
larp-2	0.88	4.17E-04
let-70	0.88	2.49E-10
let-711	0.88	9.17E-04
rab-5	0.88	1.13E-03
T22B11.5	0.88	5.21E-04
T23B12.4	0.88	9.70E-03
vrs-2	0.88	4.08E-03
W03F11.1	0.88	1.66E-04
Y54G2A.23	0.88	5.50E-03
alx-1	0.87	6.88E-03
arx-6	0.87	1.83E-03
F29G6.3	0.87	6.39E-03
F41C3.5	0.87	1.08E-06
hsr-9	0.87	2.04E-03
ptp-2	0.87	8.32E-03
T25B9.9	0.87	7.03E-04
C17G10.9	0.86	2.65E-03
C48A7.2	0.86	1.24E-03
cct-4	0.86	2.07E-04
cdc-48.2	0.86	1.82E-04
F57B10.1	0.86	3.73E-03
lin-45	0.86	7.82E-03
mep-1	0.86	5.02E-03
npl-4.2	0.86	6.10E-04
R08D7.4	0.86	8.91E-03
ubxn-6	0.86	6.94E-03
alh-8	0.85	6.20E-09
ani-2	0.85	1.87E-03
C04C3.3	0.85	7.88E-04
cdc-14	0.85	7.44E-03
F57F4.4	0.85	3.91E-07
gfi-1	0.85	5.86E-07
hmg-3	0.85	8.39E-03
K08D12.3	0.85	5.22E-06
K08F4.2	0.85	2.37E-05
lev-11	0.85	1.66E-08
mat-1	0.85	5.33E-03
npp-14	0.85	9.07E-03
R05F9.1	0.85	9.53E-03
ran-5	0.85	7.41E-03
rsp-3	0.85	1.19E-03

sur-6	0.85	1.51E-03
T28D9.4	0.85	2.90E-03
tat-4	0.85	7.02E-03
tufm-1	0.85	7.54E-03
ubc-13	0.85	2.12E-03
vha-2	0.85	6.02E-10
vps-35	0.85	8.67E-03
Y39G10AR.8	0.85	1.89E-03
C27H6.8	0.84	7.81E-03
cyld-1	0.84	7.54E-03
F33D11.10	0.84	3.59E-03
hsp-25	0.84	3.96E-03
M02B1.3	0.84	6.38E-04
mtx-1	0.84	5.88E-03
nhr-49	0.84	6.19E-03
ost-1	0.84	7.56E-05
phb-2	0.84	3.44E-04
ruvb-1	0.84	2.60E-03
sca-1	0.84	2.77E-05
T05F1.2	0.84	3.37E-03
T19B4.2	0.84	2.25E-03
unc-60	0.84	5.85E-06
C08H9.2	0.83	1.48E-06
cpb-3	0.83	2.50E-03
F13B12.6	0.83	8.38E-03
F33H1.3	0.83	6.01E-03
F53F10.2	0.83	3.17E-03
hda-1	0.83	1.03E-03
hrp-1	0.83	3.38E-03
K03B4.2	0.83	3.07E-03
prp-8	0.83	2.05E-03
sqd-1	0.83	3.80E-06
ZK418.9	0.83	9.12E-03
abcf-2	0.82	2.93E-04
atx-2	0.82	3.18E-03
C08B11.3	0.82	6.75E-03
C16C10.3	0.82	2.22E-04
C36B1.11	0.82	5.19E-03
cand-1	0.82	8.84E-03
ccr-4	0.82	7.37E-04
clic-1	0.82	1.42E-03
drr-2	0.82	7.92E-03
edc-3	0.82	5.23E-03
egl-45	0.82	8.06E-04
eif-3.B	0.82	4.12E-03
F25B5.3	0.82	1.15E-03
F57F5.1	0.82	1.06E-07

H19N07.1	0.82	5.48E-03
imb-1	0.82	8.21E-03
pbrm-1	0.82	6.89E-03
R07H5.8	0.82	1.97E-06
rnp-8	0.82	1.46E-03
scc-3	0.82	6.45E-03
set-1	0.82	3.22E-03
tag-153	0.82	4.46E-03
C30C11.4	0.81	1.01E-04
cct-1	0.81	8.84E-05
emb-9	0.81	4.24E-03
gfi-2	0.81	7.44E-03
K06B9.2	0.81	2.12E-03
pqn-59	0.81	1.05E-05
R31.2	0.81	4.96E-03
rab-11.1	0.81	3.75E-05
ran-4	0.81	2.89E-03
rme-1	0.81	5.57E-04
snb-1	0.81	6.89E-04
T21B10.3	0.81	7.74E-03
tag-319	0.81	7.80E-03
Y54E10A.6	0.81	6.30E-03
bath-41	0.80	6.72E-03
C14C6.5	0.80	6.19E-03
col-181	0.80	3.73E-15
daf-18	0.80	2.22E-03
dli-1	0.80	7.45E-03
F40F9.6	0.80	3.79E-03
F43E2.7	0.80	2.30E-03
F53C3.13	0.80	9.09E-03
K03E5.2c	0.80	7.90E-03
K07C5.4	0.80	2.72E-06
mtm-3	0.80	5.25E-03
npl-4.1	0.80	9.79E-04
srgp-1	0.80	1.94E-03
T04A8.7	0.80	9.06E-03
W07G4.4	0.80	2.06E-04
ZC395.10	0.80	5.30E-05
C01G5.6	0.79	7.87E-03
C14C10.5	0.79	4.34E-03
C56G2.1	0.79	4.24E-03
cgt-3	0.79	4.34E-03
cnx-1	0.79	5.55E-03
ctns-1	0.79	7.82E-03
egl-4	0.79	6.86E-03
F21H12.6	0.79	9.32E-03
F36D4.5	0.79	2.77E-03

him-1	0.79	9.90E-03
ivd-1	0.79	1.79E-03
R11A5.4	0.79	3.17E-05
rab-1	0.79	1.79E-04
set-25	0.79	6.06E-03
T25G3.3	0.79	8.80E-03
tlk-1	0.79	5.24E-03
ubxn-4	0.79	7.27E-03
Y104H12BR.1	0.79	4.59E-03
Y41D4A.4	0.79	9.58E-03
ZK863.4	0.79	3.89E-03
akt-1	0.78	9.54E-03
ani-1	0.78	6.94E-03
C36A4.4	0.78	3.27E-03
F17C11.10	0.78	8.67E-03
F21D5.1	0.78	5.74E-03
F21F3.6	0.78	6.15E-04
hmg-1.2	0.78	3.92E-03
iftb-1	0.78	3.46E-03
imp-1	0.78	4.46E-03
pgl-3	0.78	8.68E-03
rpn-10	0.78	2.89E-03
sec-24.1	0.78	9.92E-03
skn-1	0.78	5.63E-03
ubc-25	0.78	1.91E-03
zer-1	0.78	4.98E-03
elo-5	0.77	1.97E-04
F35E12.5	0.77	6.00E-04
gla-3	0.77	6.86E-03
K02D10.1	0.77	9.38E-03
K10C3.4	0.77	5.32E-03
let-92	0.77	7.02E-04
mpk-1	0.77	9.03E-03
npp-12	0.77	7.50E-03
R151.2	0.77	2.59E-03
sqv-5	0.77	5.79E-03
T07A9.9	0.77	2.97E-03
T09E8.1	0.77	8.71E-03
T22F3.3	0.77	1.32E-05
T23B5.1	0.77	7.40E-03
unc-76	0.77	6.32E-03
vha-12	0.77	2.55E-06
vps-32.1	0.77	6.60E-04
C06A8.1	0.76	2.06E-03
chc-1	0.76	8.49E-04
dnj-5	0.76	2.23E-03
F55A11.7	0.76	7.99E-03

ftt-2	0.76	2.44E-05
hmg-1.1	0.76	9.93E-11
lmn-1	0.76	1.83E-03
mbk-2	0.76	2.05E-03
mrs-1	0.76	6.42E-03
npp-9	0.76	6.49E-05
puf-8	0.76	3.76E-03
R03E1.2	0.76	4.01E-03
R74.8	0.76	4.94E-03
sec-23	0.76	9.87E-03
srs-2	0.76	2.62E-03
T04H1.2	0.76	8.26E-03
T12A2.2	0.76	8.57E-04
trap-1	0.76	2.70E-04
W02F12.5	0.76	1.31E-03
act-3	0.75	3.27E-34
atp-2	0.75	1.03E-12
C13B9.3	0.75	8.29E-03
F47G9.1	0.75	4.09E-03
lec-2	0.75	2.58E-04
pup-2	0.75	6.13E-04
retr-1	0.75	3.52E-06
sap-49	0.75	4.27E-03
T19B4.5	0.75	8.62E-03
W08E12.7	0.75	2.85E-07
Y105E8B.11	0.75	7.33E-38
ZK484.1.4	0.75	1.62E-05
ZK829.4	0.75	1.67E-10
F10E7.5	0.74	3.48E-03
F22F7.1	0.74	1.07E-03
far-6	0.74	6.59E-03
mlc-4	0.74	2.91E-03
nol-5	0.74	6.44E-04
pas-7	0.74	5.98E-03
pdi-1	0.74	9.68E-05
ptc-1	0.74	7.11E-04
spd-5	0.74	8.84E-03
tsn-1	0.74	1.08E-04
unc-61	0.74	7.31E-03
yop-1	0.74	1.37E-04
atf-7	0.73	2.25E-03
bath-40	0.73	1.97E-03
E04D5.1	0.73	3.77E-03
eat-6	0.73	7.34E-06
F14B4.2	0.73	6.82E-04
F44E7.4	0.73	2.72E-03
hcf-1	0.73	9.43E-03

LLC1.3	0.73	4.63E-03
nuo-4	0.73	5.56E-03
pqn-51	0.73	7.48E-03
rpt-4	0.73	9.22E-03
unc-116	0.73	6.56E-03
unc-87	0.73	1.38E-03
Y57G11C.9	0.73	5.76E-03
act-1	0.72	1.61E-35
C32F10.8	0.72	1.24E-03
C37C3.2	0.72	8.69E-03
C44B7.10	0.72	3.22E-03
cct-6	0.72	5.08E-04
cey-2	0.72	1.13E-13
EEED8.3	0.72	2.08E-03
eif-3.F	0.72	7.61E-03
F09G2.9	0.72	2.26E-03
F57B9.3	0.72	8.05E-03
fbxa-215	0.72	4.36E-03
gta-1	0.72	1.20E-03
K07H8.2	0.72	9.72E-03
K08E3.5	0.72	5.10E-04
ncs-2	0.72	6.86E-03
pes-9	0.72	4.97E-03
T13C2.6	0.72	3.81E-03
T13F2.2	0.72	7.13E-03
Y10G11A.1	0.72	5.24E-03
Y113G7B.17	0.72	1.49E-05
ztf-7	0.72	2.26E-05
arx-2	0.71	6.53E-03
cey-3	0.71	3.83E-06
dab-1	0.71	5.63E-04
F35G2.1	0.71	5.87E-03
F54E12.2	0.71	7.71E-03
gld-3	0.71	5.01E-03
grs-1	0.71	6.96E-03
inf-1	0.71	4.33E-17
larp-1	0.71	5.07E-04
lgg-2	0.71	5.24E-03
M88.5	0.71	5.22E-03
nduf-7	0.71	3.42E-03
npa-1	0.71	7.75E-03
npp-22	0.71	9.38E-03
paa-1	0.71	3.86E-04
rpn-1	0.71	7.29E-04
T05H4.5	0.71	9.99E-04
T09A5.11	0.71	1.89E-03
T14G10.5	0.71	7.98E-03

tag-18	0.71	7.29E-03
top-2	0.71	4.53E-03
tpxl-1	0.71	4.14E-03
W09C5.1	0.71	9.62E-03
Y39B6A.1	0.71	2.46E-03
ZK669.4	0.71	9.13E-03
