# Supplementary material for: Gene expression profiling of oxidative stress response of C. elegans aging defective AMPK mutants using massively parallel transcriptome sequencing
Source: BMC Res Notes. 2011 Feb 8;4:34. doi: 10.1186/1756-0500-4-34 (PMC3045954; doi:10.1186/1756-0500-4-34)
Supplement: Additional file 12 — Supplementary Table S11. Genes that are significantly up-regulated in unstressed aak-2 mutants compared to wild type animals and most highly represented biological processes these genes are involved in [file 1756-0500-4-34-S12.PDF]

Supplementary Table 11. genes that are significantly up-regulated in unstressed aak-2 mutants compared to wild type animals and most highly represented biological processes these genes are involved in

GO	Genes	Pvalue	GO as name
GO:0040008	erm-1; sams-1; phb-2; cdc-48.2; hmg-3; prpf-4; cmd-1; y71h2am.20; b0285.1; m03f4.6; kin-2; zk829.4; vha-8; mat-1; w02f12.5; mom-5; npl-4.2; c30c11.4; lin-35; mel-26; sec-23; h06i04.3; y105c5b.12; ppn-1; c06e7.3; f09f7.4; pmt-1; ccf-1; t09a5.11; nmy-1; c06e7.1; eif-3.b; vab-10; c30h7.2; ama-1; zk546.14; pbrm-1; opt-2; egl-45; larp-1; f29g6.3; b0416.5; arf-1.2; mtm-3; gld-2; t19b4.5; arx-6; c23g10.8; top-1; dab-1; t12a2.2; imb-3; pqn-20; gsk-3; k02f2.2; lev-11; unc-116; y71h10b.1; nuo-4; y39g10ar.8; f55c5.8; elo-5; spd-5; mrs-1; t05h4.5; y71f9al.17; ruvb-1; ech-6; vha-5; tsn-1; mep-1; f57b10.1; vit-4; rfc-1; ntl-4; crs-1; e01a2.4; ost-1; let-60; mca-3; dpy-17; t04a8.7; set-1; t07a9.9; b0035.12; ani-2; smk-1; tag-310; unc-32; rfp-1; gfi-2; atp-2; f43e2.7; anc-1; srs-2; cct-1; npl-4.1; nhr-49; c08h9.2; zk858.1; f44g4.1; k08e3.5; nol-5; vrs-2; fat-7; wnk-1; c37c3.2; nmy-2; k07h8.10; r07h5.8; eif-3.f; y113g7b.17; y102a5c.6; c36e8.1; vps-35; mdt-15; sec-24.2; tat-5; t21b10.3; egl-27; ifg-1; c37h5.6; t25g3.3; b0495.2; c06a8.1; lst-3; f09f7.3; h19n07.1; cpl-1; npp-21; hrp-1; emb-9; f15d3.6; arx-2; math-33; lrs-1; c53h9.2; vha-11; iftb-1; vit-3; k07c5.4; vha-13; e04f6.5; snap-1; f57f4.4; gfi-1; cdc-14; k08f4.2; pup-2; lin-41; let-721; fib-1; pap-1; mbk-2; t14g10.5; zc247.1; irs-1; nuo-1; rpt-4; mua-6; c04c3.3; unc-52; csn-2; aex-5; csnk-1; ran-4; [illegible]	2.83E-32	regulation of growth;
GO:0022414	spk-1; zyg-11; ptc-1; npp-9; dpy-17; cct-5; sap-49; unc-15; zk632.2; b0035.12; act-3; egl-30; npp-7; smk-1; sur-6; atx-2; m03f4.6; let-70; unc-32; rfp-1; atp-2; cct-1; uaf-1; c08h9.2; zk858.1; mom-5; c08b11.3; c30c11.4; lin-35; act-4; nol-5; smc-4; y105c5b.12; ppn-1; him-1; sqv-5; unc-54; c06e7.3; par-1; wnk-1; c48a7.2; scpl-1; ccf-1; prp-8; c06e7.1; gld-3; hrp-2; ptc-2; c30h7.2; vab-10; nmy-2; f52c6.12; pbrm-1; eif-3.f; egl-45; lmn-1; cct-6; arf-1.2; mtm-3; abcf-1; act-1; tat-5; t21b10.3; puf-8; egl-27; c37h5.6; top-1; dab-1; unc-60; c23g10.8; f35g12.2; lev-11; hda-1; c53h9.2; cgh-1; goa-1; e01a2.2; scc-3; w09c5.1; iftb-1; k07c5.4; f55c5.8; puf-12; spd-5; zfp-1; nos-3; npp-8; dyci-1; mbk-2; dli-1; aco-2; ent-2; ruvb-1; unc-61; mpk-1; vha-5; csn-2; mep-1; csnk-1; ran-4; f30a10.10	3.60E-22	reproductive process;
GO:0040035	spk-1; zyg-11; npp-9; dpy-17; cct-5; sap-49; zk632.2; act-3; smk-1; m03f4.6; let-70; rfp-1; unc-32; uaf-1; cct-1; c08h9.2; zk858.1; c08b11.3; c30c11.4; act-4; nol-5; smc-4; y105c5b.12; ppn-1; him-1; par-1; c06e7.3; c48a7.2; prp-8; ccf-1; c06e7.1; hrp-2; c30h7.2; vab-10; nmy-2; f52c6.12; pbrm-1; eif-3.f; cct-6; arf-1.2; abcf-1; act-1; tat-5; t21b10.3; c37h5.6; unc-60; c23g10.8; f35g12.2; lev-11; cgh-1; c53h9.2; hda-1; goa-1; scc-3; w09c5.1; iftb-1; k07c5.4; f55c5.8; zfp-1; npp-8; dli-1; aco-2; ruvb-1; ent-2; unc-61; mpk-1; vha-5; mep-1; csnk-1; f30a10.10	4.71E-22	hermaphrodite genitalia development;

GO:0048806	spk-1; zyg-11; npp-9; dpy-17; cct-5; sap-49; zk632.2; act-3; smk-1; m03f4.6; let-70; rfp-1; unc-32; uaf-1; cct-1; c08h9.2; zk858.1; c08b11.3; c30c11.4; act-4; nol-5; smc-4; y105c5b.12; ppn-1; him-1; par-1; c06e7.3; c48a7.2; prp-8; ccf-1; c06e7.1; hrp-2; c30h7.2; vab-10; nmy-2; f52c6.12; pbrm-1; eif-3.f; cct-6; arf-1.2; abcf-1; act-1; tat-5; t21b10.3; c37h5.6; unc-60; c23g10.8; f35g12.2; lev-11; cgh-1; c53h9.2; hda-1; goa-1; scc-3; w09c5.1; iftb-1; k07c5.4; f55c5.8; zfp-1; npp-8; dli-1; aco-2; ruvb-1; ent-2; unc-61; mpk-1; vha-5; mep-1; csnk-1; f30a10.10	2.11E-21	genitalia development;
GO:0019953	let-60; erm-1; sams-1; set-1; glh-2; t07a9.9; zk632.2; hmg-3; prpf-4; cmd-1; npp-7; y71h2am.20; smk-1; rfp-1; kin-2; csr-1; srs-2; vha-8; rpn-10; zk858.1; c55c3.5; r13f6.10; t09e8.1; nol-5; y105c5b.12; ppn-1; cct-4; par-1; c06e7.3; c48a7.2; c06e7.1; nmy-1; gld-3; eif-3.b; hrp-2; c30h7.2; vab-10; nmy-2; nud-2; tag-319; sptl-3; trs-1; glh-1; eif-3.f; vha-4; lmn-1; f29g6.3; y102a5c.6; b0416.5; arf-1.2; vps-35; daz-1; gld-2; t19b4.5; f37c12.7; tat-5; puf-8; ptp-2; pgl-1; c37h5.6; t25g3.3; top-1; hrp-1; f35g12.2; ima-3; hda-1; vha-11; cgh-1; f45f2.10; y71h10b.1; iftb-1; k07c5.4; cid-1; sqd-1; spd-5; lin-41; y66h1b.2; dyci-1; fib-1; y66h1b.3; mbk-2; zc247.1; irs-1; dli-1; hsp-6; ruvb-1; mep-1; ran-4; f30a10.10; f33h1.3	3.13E-21	sexual reproduction;
GO:0010259	acdh-1; mca-3; dao-5; mup-4; cct-5; mdt-15; k07c5.6; ifg-1; rab-1; smk-1; chc-1; let-70; emb-9; hsp-16.11; gfi-2; let-711; vha-12; cgh-1; cpr-1; c30c11.4; ifb-1; hsp-16.1; ppn-1; daf-16; fat-7; skn-1; rpb-2; lin-41; daf-18; eif-3.b; f28b3.5; f52c6.12; opt-2; unc-52; akt-1; inf-1; cct-6; sca-1	1.36E-20	multicellular organismal aging;
GO:0008340	acdh-1; mca-3; dao-5; mup-4; cct-5; mdt-15; k07c5.6; ifg-1; rab-1; smk-1; chc-1; let-70; emb-9; hsp-16.11; gfi-2; let-711; vha-12; cgh-1; cpr-1; c30c11.4; ifb-1; hsp-16.1; ppn-1; daf-16; fat-7; skn-1; rpb-2; lin-41; daf-18; eif-3.b; f28b3.5; f52c6.12; opt-2; unc-52; akt-1; inf-1; cct-6; sca-1	1.36E-20	determination of adult life span; multicellular organismal aging;
GO:0007626	let-60; erm-1; sams-1; ptc-1; cct-5; set-1; cdc-48.2; t07a9.9; snb-1; unc-15; b0035.12; zk632.2; f57f5.1; hmg-3; prpf-4; smk-1; sur-6; b0285.1; m03f4.6; let-70; tag-310; unc-32; kin-2; rfp-1; let-711; srs-2; npl-4.1; nhr-49; c08h9.2; mom-5; npl-4.2; c30c11.4; y105c5b.12; ppn-1; pod-2; him-1; fat-7; c06e7.3; pmt-1; t09a5.11; c06e7.1; nmy-1; eif-3.b; unc-87; ptc-2; vab-10; pqn-59; f52c6.12; pbrm-1; cah-4; let-2; inf-1; y113g7b.17; vha-4; cct-6; lmn-1; arf-1.2; vps-35; sec-24.2; mdt-15; apl-1; h37a05.1; f37c12.7; t21b10.3; egl-27; ptp-2; t25g3.3; c37h5.6; dab-1; unc-60; top-1; lst-3; t12a2.2; k08d12.3; h19n07.1; cpl-1; npp-21; emb-9; ima-3; lev-11; k02f2.2; arx-2; vha-12; goa-1; unc-116; t05h4.6a; f57b9.3; ifb-1; wwp-1; scc-3; y39g10ar.8; snap-1; lin-41; r05f9.1; pap-1; t14g10.5; y71f9al.17; dli-1; aco-2; r03e1.2; unc-52; mua-6; vha-5; ran-5; f57b10.1; csnk-1; ran-4; f30a10.10; e01a2.4	2.18E-20	locomotory behavior;

GO:0007276	let-60; erm-1; sams-1; set-1; glh-2; t07a9.9; zk632.2; hmg-3; prpf-4; cmd-1; y71h2am.20; smk-1; rfp-1; kin-2; csr-1; srs-2; vha-8; rpn-10; zk858.1; c55c3.5; r13f6.10; t09e8.1; nol-5; y105c5b.12; ppn-1; cct-4; par-1; c06e7.3; c48a7.2; nmy-1; c06e7.1; gld-3; eif-3.b; hrp-2; c30h7.2; vab-10; nmy-2; nud-2; tag-319; sptl-3; trs-1; glh-1; eif-3.f; vha-4; lmn-1; f29g6.3; y102a5c.6; b0416.5; arf-1.2; vps-35; daz-1; gld-2; t19b4.5; f37c12.7; tat-5; puf-8; ptp-2; pgl-1; c37h5.6; t25g3.3; top-1; hrp-1; f35g12.2; ima-3; hda-1; cgh-1; vha-11; f45f2.10; y71h10b.1; iftb-1; k07c5.4; cid-1; sqd-1; lin-41; y66h1b.2; fib-1; y66h1b.3; mbk-2; zc247.1; irs-1; hsp-6; ruvb-1; mep-1; f30a10.10; f33h1.3	2.24E-20	gamete generation;
GO:0065007	erm-1; sams-1; phb-2; cdc-48.2; snb-1; tag-182; hmg-3; prpf-4; cmd-1; rab-11.1; y71h2am.20; rab-1; sur-6; b0285.1; m03f4.6; d2096.11; kin-2; zk829.4; vha-8; mat-1; w02f12.5; mom-5; npl-4.2; c30c11.4; lin-35; mel-26; cki-2; sec-23; mxl-3; h06i04.3; y105c5b.12; let-92; ppn-1; pod-2; t13f2.2; tag-153; c06e7.3; f09f7.4; pmt-1; daf-18; t09a5.11; ccf-1; prp-8; c06e7.1; gld-3; nmy-1; eif-3.b; c30h7.2; vab-10; ama-1; zk546.14; pbrm-1; opt-2; egl-45; larp-1; f29g6.3; b0416.5; arf-1.2; mtm-3; gld-2; t19b4.5; arx-6; ptp-2; vha-2; dab-1; top-1; c23g10.8; t12a2.2; imb-3; pqn-20; gsk-3; lev-11; k02f2.2; vha-12; cgh-1; hda-1; unc-116; hmg-1.1; goa-1; vha-10; y71h10b.1; nuo-4; y39g10ar.8; f55c5.8; skn-1; elo-5; spd-5; zfp-1; cey-1; nos-3; mrs-1; unc-76; t05h4.5; y71f9al.17; ruvb-1; ech-6; vha-5; tsn-1; mep-1; f57b10.1; vit-4; rfc-1; ntl-4; crs-1; e01a2.4; ost-1; let-60; zyg-11; mca-3; dpy-17; t04a8.7; set-1; t07a9.9; b0035.12; egl-30; ani-2; smk-1; atx-2; cey-3; tag-310; rfp-1; unc-32; atf-7; gfi-2; atp-2; f43e2.7; npl-4.1; cct-1; anc-1; srs-2; nhr-49; c08h9.2; zk858.1; f44g4.1; k08e3.5; nol-5; vrs-2; nhr-114; lfi-1; fat-7; ceh-38; wnk-1; c37c3.2; c48a7.2; nmy-2; k07h8.10; r07h5.8; eif-3.f; y113g7b.17; vha-4; cct-6; sca-1; c36e8.1; y102a5c.6; vps-35; mdt-15; sec-24.2; mut-16; apl-1; tat-5; puf-8; t21b10.3; egl-27; ifg-1; b0495.2; t25g3.3; c37h5.6; zip-2; lst-3; [illegible]	2.91E-20	biological regulation;
GO:0048513	let-60; spk-1; zyg-11; npp-9; dpy-17; cct-5; sap-49; zk632.2; act-3; smk-1; sur-6; m03f4.6; let-70; rfp-1; unc-32; uaf-1; cct-1; c08h9.2; zk858.1; c08b11.3; c30c11.4; lin-35; act-4; nol-5; smc-4; y105c5b.12; ppn-1; let-92; him-1; sqv-5; unc-54; c06e7.3; par-1; c48a7.2; ccf-1; prp-8; c06e7.1; hrp-2; c30h7.2; vab-10; nmy-2; f52c6.12; pbrm-1; eif-3.f; cct-6; arf-1.2; abcf-1; act-1; sup-17; tat-5; t21b10.3; ptp-2; c37h5.6; c23g10.8; unc-60; top-1; f35g12.2; emb-9; lin-45; lev-11; cgh-1; c53h9.2; hda-1; goa-1; scc-3; w09c5.1; iftb-1; k07c5.4; f55c5.8; skn-1; zfp-1; npp-8; npa-1; lin-41; dli-1; aco-2; ent-2; ruvb-1; unc-61; mpk-1; unc-52; vha-5; mep-1; csnk-1; f30a10.10	3.16E-20	organ development;
GO:0007568	acdh-1; mca-3; dao-5; mup-4; cct-5; mdt-15; k07c5.6; ifg-1; rab-1; smk-1; chc-1; let-70; emb-9; hsp-16.11; gfi-2; let-711; vha-12; cgh-1; cpr-1; c30c11.4; ifb-1; hsp-16.1; ppn-1; daf-16; fat-7; skn-1; rpb-2; lin-41; daf-18; eif-3.b; f28b3.5; f52c6.12; opt-2; unc-52; akt-1; inf-1; cct-6; sca-1	3.42E-20	%biological_process%developmental process%aging;

GO:0050789	erm-1; sams-1; phb-2; cdc-48.2; snb-1; tag-182; hmg-3; prpf-4; cmd-1; rab-11.1; y71h2am.20; rab-1; sur-6; b0285.1; m03f4.6; d2096.11; kin-2; zk829.4; vha-8; mat-1; w02f12.5; mom-5; npl-4.2; c30c11.4; lin-35; mel-26; cki-2; sec-23; mxl-3; h06i04.3; y105c5b.12; let-92; ppn-1; t13f2.2; tag-153; c06e7.3; f09f7.4; pmt-1; daf-18; t09a5.11; ccf-1; c06e7.1; gld-3; nmy-1; eif-3.b; c30h7.2; vab-10; ama-1; zk546.14; pbrm-1; opt-2; egl-45; larp-1; f29g6.3; b0416.5; arf-1.2; mtm-3; gld-2; t19b4.5; arx-6; ptp-2; vha-2; dab-1; c23g10.8; top-1; t12a2.2; imb-3; pqn-20; gsk-3; lev-11; k02f2.2; vha-12; cgh-1; hda-1; hmg-1.1; unc-116; goa-1; vha-10; y71h10b.1; nuo-4; y39g10ar.8; f55c5.8; skn-1; elo-5; spd-5; zfp-1; cey-1; nos-3; mrs-1; unc-76; y71f9al.17; t05h4.5; ruvb-1; ech-6; vha-5; tsn-1; mep-1; f57b10.1; vit-4; rfc-1; ntl-4; crs-1; e01a2.4; ost-1; let-60; zyg-11; mca-3; dpy-17; t04a8.7; set-1; t07a9.9; b0035.12; egl-30; ani-2; smk-1; atx-2; cey-3; tag-310; rfp-1; unc-32; atf-7; gfi-2; atp-2; f43e2.7; anc-1; srs-2; cct-1; npl-4.1; nhr-49; c08h9.2; zk858.1; f44g4.1; k08e3.5; nol-5; vrs-2; nhr-114; lfi-1; fat-7; ceh-38; wnk-1; c37c3.2; c48a7.2; nmy-2; k07h8.10; r07h5.8; eif-3.f; y113g7b.17; c36e8.1; y102a5c.6; vps-35; mdt-15; sec-24.2; mut-16; apl-1; tat-5; puf-8; t21b10.3; egl-27; ifg-1; b0495.2; t25g3.3; c37h5.6; zip-2; lst-3; c06a8.1; f09f7.3; cpl-1; h19n07.1; npp-21; hrp-1; emb-9; f15d3.6; arx-2; hif-1; math-33; lrs-1;	5.33E-20	regulation of biological process;
GO:0007610	let-60; erm-1; sams-1; ptc-1; cct-5; set-1; cdc-48.2; t07a9.9; snb-1; unc-15; b0035.12; zk632.2; f57f5.1; hmg-3; egl-30; prpf-4; smk-1; sur-6; atx-2; b0285.1; m03f4.6; let-70; tag-310; unc-32; kin-2; rfp-1; let-711; atp-2; srs-2; npl-4.1; nhr-49; c08h9.2; mom-5; npl-4.2; c30c11.4; lin-35; y105c5b.12; ppn-1; pod-2; him-1; fat-7; sqv-5; unc-54; c06e7.3; wnk-1; pmt-1; scpl-1; t09a5.11; c06e7.1; nmy-1; eif-3.b; unc-87; ptc-2; vab-10; pqn-59; f52c6.12; pbrm-1; cah-4; let-2; inf-1; egl-45; vha-4; y113g7b.17; cct-6; lmn-1; arf-1.2; vps-35; mtm-3; mdt-15; sec-24.2; apl-1; h37a05.1; f37c12.7; t21b10.3; egl-27; ptp-2; t25g3.3; c37h5.6; dab-1; unc-60; top-1; lst-3; t12a2.2; k08d12.3; h19n07.1; cpl-1; npp-21; emb-9; ima-3; lev-11; k02f2.2; arx-2; vha-12; goa-1; unc-116; t05h4.6a; f57b9.3; ifb-1; wwp-1; e01a2.2; scc-3; y39g10ar.8; snap-1; puf-12; lin-41; r05f9.1; pap-1; t14g10.5; y71f9al.17; dli-1; aco-2; ent-2; r03e1.2; unc-61; unc-52; mua-6; vha-5; csn-2; ran-5; f57b10.1; csnk-1; ran-4; f30a10.10; e01a2.4	8.72E-20	%biological_process%response to stimulus%behavior;
GO:0003006	spk-1; zyg-11; npp-9; dpy-17; cct-5; sap-49; zk632.2; act-3; smk-1; atx-2; m03f4.6; let-70; rfp-1; unc-32; uaf-1; cct-1; c08h9.2; zk858.1; c08b11.3; c30c11.4; act-4; nol-5; smc-4; y105c5b.12; ppn-1; him-1; par-1; c06e7.3; c48a7.2; prp-8; ccf-1; c06e7.1; gld-3; hrp-2; c30h7.2; vab-10; nmy-2; f52c6.12; pbrm-1; eif-3.f; cct-6; arf-1.2; abcf-1; act-1; tat-5; puf-8; t21b10.3; c37h5.6; unc-60; top-1; c23g10.8; f35g12.2; lev-11; cgh-1; c53h9.2; hda-1; goa-1; scc-3; iftb-1; w09c5.1; k07c5.4; f55c5.8; zfp-1; nos-3; npp-8; dli-1; aco-2; ent-2; ruvb-1; unc-61; mpk-1; vha-5; mep-1; csnk-1; f30a10.10	1.65E-19	reproductive developmental process;

GO:0050896	erm-1; sams-1; ptc-1; cct-5; cdc-48.2; snb-1; zk632.2; hmg-3; prpf-4; sur-6; b0285.1; m03f4.6; kin-2; mom-5; c30c11.4; npl-4.2; lin-35; hsp-16.1; y105c5b.12; ppn-1; pod-2; him-1; c06e7.3; pmt-1; daf-18; t09a5.11; nmy-1; c06e7.1; eif-3.b; unc-87; vab-10; pbrm-1; pqn-59; cah-4; inf-1; egl-45; arf-1.2; mtm-3; h37a05.1; ptp-2; top-1; unc-60; dab-1; t12a2.2; ima-3; k02f2.2; lev-11; vha-12; hda-1; t05h4.6a; unc-116; goa-1; f57b9.3; scc-3; e01a2.2; y39g10ar.8; skn-1; r05f9.1; y71f9al.17; aco-2; r03e1.2; unc-61; vha-5; ran-5; f57b10.1; rfc-1; e01a2.4; let-60; set-1; t07a9.9; unc-15; b0035.12; f57f5.1; egl-30; smk-1; atx-2; col-179; let-70; tag-310; unc-32; rfp-1; let-711; atp-2; srs-2; npl-4.1; nhr-49; c08h9.2; fat-7; sqv-5; unc-54; wnk-1; scpl-1; ptc-2; f52c6.12; let-2; vha-4; y113g7b.17; cct-6; lmn-1; sca-1; acdh-1; vps-35; mdt-15; sec-24.2; apl-1; f37c12.7; pme-1; t21b10.3; egl-27; b0495.2; t25g3.3; c37h5.6; lst-3; k08d12.3; h19n07.1; cpl-1; npp-21; emb-9; hsp-16.11; hif-1; arx-2; ifb-1; wwp-1; lea-1; daf-16; snap-1; puf-12; cdc-14; lin-41; lig-1; pap-1; t14g10.5; set-25; dli-1; nuo-1; ent-2; mua-6; unc-52; csn-2; csnk-1; ran-4; f30a10.10	6.55E-19	response to stimulus;
GO:0048731	let-60; spk-1; zyg-11; npp-9; dpy-17; cct-5; sap-49; zk632.2; act-3; smk-1; sur-6; m03f4.6; let-70; rfp-1; unc-32; uaf-1; cct-1; c08h9.2; zk858.1; c08b11.3; c30c11.4; lin-35; act-4; nol-5; smc-4; y105c5b.12; ppn-1; let-92; him-1; sqv-5; unc-54; c06e7.3; par-1; c48a7.2; ccf-1; prp-8; c06e7.1; hrp-2; c30h7.2; vab-10; nmy-2; f52c6.12; pbrm-1; eif-3.f; cct-6; arf-1.2; abcf-1; act-1; sup-17; tat-5; t21b10.3; ptp-2; c37h5.6; c23g10.8; unc-60; top-1; f35g12.2; emb-9; lin-45; lev-11; cgh-1; c53h9.2; hda-1; goa-1; scc-3; w09c5.1; iftb-1; k07c5.4; f55c5.8; skn-1; zfp-1; npp-8; npa-1; lin-41; unc-76; dli-1; aco-2; ent-2; ruvb-1; unc-61; mpk-1; unc-52; vha-5; mep-1; csnk-1; f30a10.10	3.04E-18	system development;
GO:0007548	spk-1; zyg-11; npp-9; dpy-17; cct-5; sap-49; zk632.2; act-3; smk-1; m03f4.6; let-70; rfp-1; unc-32; uaf-1; cct-1; c08h9.2; zk858.1; c08b11.3; c30c11.4; act-4; nol-5; smc-4; y105c5b.12; ppn-1; him-1; par-1; c06e7.3; c48a7.2; prp-8; ccf-1; c06e7.1; hrp-2; c30h7.2; vab-10; nmy-2; f52c6.12; pbrm-1; eif-3.f; cct-6; arf-1.2; abcf-1; act-1; tat-5; t21b10.3; c37h5.6; unc-60; top-1; c23g10.8; f35g12.2; lev-11; cgh-1; c53h9.2; hda-1; goa-1; scc-3; w09c5.1; iftb-1; k07c5.4; f55c5.8; zfp-1; npp-8; dli-1; aco-2; ruvb-1; ent-2; unc-61; mpk-1; vha-5; mep-1; csnk-1; f30a10.10	3.48E-18	sex differentiation;
GO:0009653	let-60; erm-1; t04a8.7; snb-1; zk632.2; unc-15; ani-2; hmg-3; c01b4.6; sur-6; b0285.1; rfp-1; kin-2; gfi-2; let-711; npl-4.1; c08h9.2; mom-5; y19d10a.16; npl-4.2; c08b11.3; c30c11.4; lin-35; mel-26; cpz-1; ppn-1; cct-4; sqv-5; unc-54; par-1; ubc-25; daf-18; nmy-1; eif-3.b; vab-10; nmy-2; rsp-3; ama-1; pbrm-1; cah-4; eif-3.f; y113g7b.17; lmn-1; vps-32.1; arf-1.2; mtm-3; mlc-4; sec-24.2; apl-1; f37c12.7; t21b10.3; ptp-2; egl-27; ifg-1; top-1; imb-3; c18a3.5; k02f2.2; lev-11; cgh-1; lrs-1; unc-116; ifb-1; f33d11.10; lin-41; y66h1b.2; let-721; unc-76; pap-1; y66h1b.3; mbk-2; dli-1; ent-2; ruvb-1; paa-1; rpt-4; unc-61; mua-6; unc-52; vha-5; ran-5; csnk-1; f57b10.1; f33h1.3; e01a2.4	1.86E-17	anatomical structure morphogenesis;

GO:0016043	let-60; erm-1; zyg-11; mca-3; npp-9; f52e1.13; lys-5; snb-1; act-3; cmd-1; npp-7; rab-11.1; rab-1; sur-6; eif-3.c; pqn-51; anc-1; rme-1; mom-5; mel-26; act-4; f10e7.5; smc-4; sec-23; rab-5; kin-19; him-1; unc-54; lys-4; par-1; c37c3.2; c48a7.2; daf-18; dnj-12; nmy-1; eif-3.b; nmy-2; rsp-3; ama-1; tpxl-1; imb-1; lmn-1; arf-1.2; vps-26; mlc-4; sec-24.2; act-1; tat-5; taf-13; arx-6; ptp-2; pgl-1; unc-60; dab-1; chc-1; emb-9; imb-3; apb-1; gsk-3; ima-3; vha-12; c53h9.2; hda-1; goa-1; unc-116; t05h4.6a; c13b9.3; scc-3; iftb-1; snap-1; ced-7; spd-5; sec-24.1; npp-8; f21d5.7; unc-76; dyci-1; fib-1; mbk-2; t14g10.5; f32e10.6; y71f9al.17; dli-1; npp-10; lys-6; ran-5; csn-2; aex-5; f57b10.1; csnk-1; ran-4	1.88E-15	cellular component organization and biogenesis;
GO:0040016	rab-5; spk-1; zyg-11; let-92; mlc-4; kin-19; f33d11.10; spd-5; b0035.12; tat-5; npp-8; act-3; par-1; hmg-3; dnj-12; lig-1; mbk-2; sur-6; t22f3.3; npp-10; ama-1; gsk-3; tpxl-1; mat-1; csn-2; f57b10.1; csnk-1; goa-1; mom-5; imb-1; mel-26; act-4; smc-4	4.29E-15	embryonic cleavage;
GO:0051301	spk-1; zyg-11; mlc-4; tat-5; b0035.12; act-3; egl-27; pgl-1; hmg-3; rab-11.1; sur-6; t22f3.3; gsk-3; mat-1; mom-5; goa-1; mel-26; act-4; smc-4; rab-5; let-92; kin-19; f33d11.10; spd-5; npp-8; par-1; dnj-12; lig-1; mbk-2; npp-10; nmy-2; ama-1; tpxl-1; csn-2; f57b10.1; csnk-1; imb-1	4.91E-15	cell division;
GO:0010171	vps-32.1; let-60; mtm-3; mlc-4; t04a8.7; sec-24.2; apl-1; unc-15; egl-27; ifg-1; hmg-3; ani-2; c01b4.6; b0285.1; kin-2; lev-11; k02f2.2; lrs-1; c08h9.2; mom-5; y19d10a.16; c08b11.3; c30c11.4; ifb-1; mel-26; lin-35; cpz-1; cct-4; unc-54; y66h1b.2; let-721; nmy-1; pap-1; y66h1b.3; eif-3.b; vab-10; ruvb-1; pbrm-1; rpt-4; cah-4; mua-6; vha-5; ran-5; eif-3.f; csnk-1; f57b10.1; lmn-1; f33h1.3; e01a2.4	1.95E-13	body morphogenesis;
GO:0040032	vps-32.1; let-60; mtm-3; t04a8.7; sec-24.2; unc-15; ifg-1; egl-27; hmg-3; ani-2; c01b4.6; b0285.1; kin-2; lev-11; k02f2.2; lrs-1; c08h9.2; mom-5; y19d10a.16; c08b11.3; c30c11.4; ifb-1; mel-26; lin-35; cpz-1; cct-4; unc-54; y66h1b.2; let-721; nmy-1; pap-1; eif-3.b; y66h1b.3; vab-10; ruvb-1; pbrm-1; rpt-4; cah-4; mua-6; vha-5; ran-5; eif-3.f; csnk-1; f57b10.1; lmn-1; f33h1.3; e01a2.4	5.46E-13	post-embryonic body morphogenesis;
GO:0009886	vps-32.1; let-60; mtm-3; t04a8.7; sec-24.2; unc-15; ifg-1; egl-27; hmg-3; ani-2; c01b4.6; b0285.1; kin-2; lev-11; k02f2.2; lrs-1; c08h9.2; mom-5; y19d10a.16; c08b11.3; c30c11.4; ifb-1; mel-26; lin-35; cpz-1; cct-4; unc-54; y66h1b.2; let-721; nmy-1; pap-1; eif-3.b; y66h1b.3; vab-10; ruvb-1; pbrm-1; rpt-4; cah-4; mua-6; vha-5; ran-5; eif-3.f; csnk-1; f57b10.1; lmn-1; f33h1.3; e01a2.4	7.46E-13	post-embryonic morphogenesis;
GO:0051649	let-60; arf-1.2; zyg-11; npp-9; vps-26; sec-24.2; snb-1; tat-5; dab-1; rab-11.1; rab-1; chc-1; emb-9; imb-3; apb-1; gsk-3; ima-3; c53h9.2; unc-116; goa-1; mom-5; c13b9.3; mel-26; sec-23; rab-5; snap-1; spd-5; npp-8; sec-24.1; f21d5.7; par-1; c48a7.2; daf-18; dyci-1; mbk-2; t14g10.5; y71f9al.17; dli-1; csn-2; ran-5; aex-5; csnk-1; imb-1	2.68E-11	establishment of cellular localization;
GO:0051641	let-60; arf-1.2; zyg-11; npp-9; vps-26; sec-24.2; snb-1; tat-5; dab-1; rab-11.1; rab-1; chc-1; emb-9; imb-3; apb-1; gsk-3; ima-3; c53h9.2; unc-116; goa-1; mom-5; c13b9.3; mel-26; sec-23; rab-5; snap-1; spd-5; npp-8; sec-24.1; f21d5.7; par-1; c48a7.2; daf-18; dyci-1; mbk-2; t14g10.5; y71f9al.17; dli-1; csn-2; ran-5; aex-5; csnk-1; imb-1	6.78E-11	cellular localization;

GO:0002009	erm-1; arf-1.2; snb-1; zk632.2; f37c12.7; t21b10.3; egl-27; top-1; sur-6; rfp-1; c18a3.5; let-711; gfi-2; npl-4.1; cgh-1; unc-116; npl-4.2; c30c11.4; lin-35; ppn-1; sqv-5; cct-4; lin-41; ubc-25; daf-18; mbk-2; dli-1; vab-10; nmy-2; paa-1; ent-2; unc-61; y113g7b.17	6.83E-09	morphogenesis of an epithelium;
GO:0006732	b0250.5; vha-13; vha-14; vha-16; vha-2; c44b7.10; zk1320.9; f35g12.2; aco-2; atp-2; vha-8; t25b9.9; vha-15; vha-12; vha-11; w02f12.5; vha-4; r03d7.1	2.84E-07	coenzyme metabolic process;
GO:0033036	vps-32.1; let-60; arf-1.2; rab-5; snap-1; mlc-4; sec-24.2; sec-24.1; par-1; f21d5.7; eel-1; c48a7.2; daf-18; dab-1; rab-11.1; rab-1; nmy-1; mbk-2; chc-1; t14g10.5; y71f9al.17; imb-3; emb-9; apb-1; ima-3; c13b9.3; imb-1; t08g11.1; lmn-1; sec-23	3.67E-07	macromolecule localization;
GO:0048878	ost-1; mca-3; vps-35; pod-2; snap-1; mdt-15; apl-1; b0035.12; t21b10.3; eat-6; prp-8; y71h2am.20; m03f4.6; npp-21; opt-2; f43e2.7; cct-1; ech-6; vha-12; arx-2; vha-5; c50f7.4; f57b10.1; vha-4; cct-6; sca-1; f29g6.3; y71h10b.1	5.64E-07	chemical homeostasis;
GO:0035264	ost-1; erm-1; sams-1; vps-35; dpy-17; sec-24.2; phb-2; hmg-3; ani-2; t25g3.3; top-1; t12a2.2; b0285.1; h19n07.1; npp-21; hrp-1; kin-2; pqn-20; gfi-2; k02f2.2; lev-11; c08h9.2; vha-11; math-33; unc-116; lin-35; mel-26; iftb-1; k07c5.4; y39g10ar.8; vrs-2; f55c5.8; k08f4.2; lin-41; ccf-1; t09a5.11; nmy-1; mbk-2; zc247.1; nuo-1; opt-2; eif-3.f; f57b10.1; csnk-1; egl-45; f53a2.7; f33h1.3; crs-1	8.11E-07	multicellular organism growth;
GO:0040014	ost-1; erm-1; sams-1; vps-35; dpy-17; sec-24.2; phb-2; hmg-3; ani-2; t25g3.3; top-1; t12a2.2; b0285.1; h19n07.1; npp-21; hrp-1; kin-2; pqn-20; gfi-2; k02f2.2; lev-11; c08h9.2; vha-11; math-33; unc-116; lin-35; mel-26; iftb-1; k07c5.4; y39g10ar.8; vrs-2; f55c5.8; k08f4.2; lin-41; ccf-1; t09a5.11; nmy-1; mbk-2; zc247.1; nuo-1; opt-2; eif-3.f; f57b10.1; csnk-1; egl-45; f53a2.7; f33h1.3; crs-1	8.11E-07	regulation of multicellular organism growth;
GO:0008104	vps-32.1; let-60; arf-1.2; rab-5; snap-1; mlc-4; sec-24.2; sec-24.1; par-1; f21d5.7; c48a7.2; daf-18; dab-1; rab-11.1; rab-1; nmy-1; mbk-2; chc-1; t14g10.5; y71f9al.17; imb-3; emb-9; apb-1; ima-3; c13b9.3; imb-1; t08g11.1; lmn-1; sec-23	1.10E-06	protein localization;
GO:0022402	zyg-11; mlc-4; gld-2; rpn-1; him-1; tat-5; nos-3; act-3; par-1; puf-8; cmd-1; ccf-1; gld-3; mbk-2; atx-2; d2096.11; vab-10; ima-3; gsk-3; cgh-1; csn-2; csnk-1; goa-1; mom-5; mel-26; cki-2; scc-3	1.10E-06	cell cycle process;
GO:0030104	ost-1; vps-35; pod-2; snap-1; mdt-15; apl-1; b0035.12; t21b10.3; prp-8; y71h2am.20; m03f4.6; npp-21; f43e2.7; opt-2; ech-6; cct-1; arx-2; vha-5; c50f7.4; f57b10.1; vha-4; cct-6; sca-1; f29g6.3; y71h10b.1	1.65E-06	water homeostasis; osmoregulation;
GO:0007049	zyg-11; mlc-4; gld-2; rpn-1; him-1; tat-5; nos-3; act-3; par-1; puf-8; cmd-1; ccf-1; gld-3; mbk-2; atx-2; d2096.11; vab-10; ima-3; gsk-3; unc-61; cgh-1; csn-2; csnk-1; goa-1; mom-5; mel-26; lmn-1; cki-2; scc-3	1.80E-06	cell cycle;
GO:0018987	ost-1; vps-35; pod-2; snap-1; mdt-15; apl-1; b0035.12; t21b10.3; prp-8; y71h2am.20; m03f4.6; npp-21; f43e2.7; opt-2; ech-6; cct-1; arx-2; vha-5; c50f7.4; f57b10.1; vha-4; cct-6; sca-1; f29g6.3; y71h10b.1	1.86E-06	osmoregulation;
GO:0007010	erm-1; kin-19; act-1; spd-5; tat-5; unc-54; act-3; arx-6; par-1; dnj-12; unc-60; mbk-2; sur-6; npp-10; ama-1; gsk-3; tpxl-1; anc-1; csn-2; csnk-1; goa-1; unc-116; mom-5; imb-1; mel-26; act-4	2.13E-06	cytoskeleton organization and biogenesis;

GO:0050878	ost-1; vps-35; pod-2; snap-1; mdt-15; apl-1; b0035.12; t21b10.3; prp-8; y71h2am.20; m03f4.6; npp-21; f43e2.7; opt-2; ech-6; cct-1; arx-2; vha-5; c50f7.4; f57b10.1; vha-4; cct-6; sca-1; f29g6.3; y71h10b.1	2.13E-06	regulation of body fluid levels;
GO:0009987	sams-1; f52e1.13; act-3; pas-7; eel-1; f44e7.4; prpf-4; cmd-1; npp-7; b0285.1; t22f3.3; eif-3.c; kin-2; vha-8; mat-1; lin-35; act-4; mxl-3; b0250.5; y105c5b.12; rab-5; let-92; c25a8.4; vha-14; c06e7.3; prp-8; ccf-1; gld-3; eif-3.b; pyc-1; rsp-3; pbrm-1; cah-4; tufm-1; hpd-1; arf-1.2; gld-2; mlc-4; ptp-2; vha-2; top-1; f35g12.2; lin-45; apb-1; w07g4.4; ima-3; gsk-3; c09g4.2; c34d4.14; vha-12; hda-1; grs-1; t05h4.6a; goa-1; hmg-1.1; cpr-1; scc-3; nuo-4; skn-1; nos-3; cey-1; f53h4.2; f36a2.13; mrs-1; zk1320.9; lys-6; t25b9.9; tsn-1; f57b10.1; k08f8.1; crs-1; zyg-11; mca-3; r11a5.4; lys-5; b0035.12; atx-2; f42g9.1; cey-3; let-70; unc-32; anc-1; pqn-51; srs-2; cct-1; nhr-49; rme-1; tlk-1; r13f6.10; smc-4; f28a12.4; nhr-114; lfi-1; fat-7; cct-4; ceh-38; unc-54; nmy-2; t05h10.1; glh-1; vha-4; lgg-1; lmn-1; f13b12.6; vps-26; sec-24.2; tat-5; c27a2.1; puf-8; egl-27; zip-2; f35g2.1; cpl-1; hrp-1; hsp-16.11; vha-15; gpi-1; c53h9.2; pdi-1; c13b9.3; emb-5; vha-13; snap-1; cdc-14; rpn-1; ced-7; npp-8; dyci-1; llc1.3; pap-1; irs-1; set-25; nuo-1; npp-10; dli-1; pde-2; csn-2; adr-2; csnk-1; t22b11.5; ran-4; f30a10.10; hsp-60; t15h9.1; erm-1; spk-1; npp-9; cct-5; glh-2; cdc-48.2; f53f10.2; snb-1; hmg-3; tag-182; rab-11.1; rab-1; sur-6; zk836.2; d2096.11; zk829.4; uaf-1; w02f12.5; ldh-1; mom-5; mel-26; cki-2; hsp-16.1; f10e7.5; sec-23; t13f2.2; him-1; tag-153; lys-4;	3.25E-06	cellular process;
GO:0000910	spk-1; zyg-11; let-92; mlc-4; f33d11.10; spd-5; b0035.12; act-3; hmg-3; rab-11.1; lig-1; mbk-2; sur-6; t22f3.3; nmy-2; csn-2; act-4; smc-4	4.05E-06	cell division%cytokinesis;
GO:0042592	ost-1; mca-3; vps-35; pod-2; snap-1; mdt-15; apl-1; b0035.12; t21b10.3; eat-6; prp-8; f35g2.1; y71h2am.20; llc1.3; m03f4.6; npp-21; f43e2.7; opt-2; cct-1; ech-6; arx-2; vha-12; vha-5; c50f7.4; f57b10.1; pdi-1; vha-4; cct-6; sca-1; f29g6.3; y71h10b.1	5.42E-06	homeostatic process;
GO:0006886	let-60; arf-1.2; rab-5; snap-1; sec-24.2; sec-24.1; f21d5.7; c48a7.2; daf-18; rab-11.1; rab-1; chc-1; t14g10.5; y71f9al.17; imb-3; apb-1; ima-3; imb-1; c13b9.3; sec-23	6.06E-06	intracellular protein transport;
GO:0044238	spk-1; cct-5; glh-2; f53f10.2; hex-1; f44e7.4; eel-1; tag-182; hmg-3; pas-7; prpf-4; rab-11.1; rab-1; b0285.1; t22f3.3; zk836.2; kin-2; eif-3.c; zk829.4; vha-8; uaf-1; ldh-1; w02f12.5; lin-35; mxl-3; b0250.5; c25a8.4; vha-14; t13f2.2; him-1; tag-153; lys-4; par-1; daf-18; dnj-12; t09a5.11; prp-8; eif-3.b; pyc-1; rsp-3; ama-1; pbrm-1; opt-2; tufm-1; trs-1; imb-1; f21d5.1; hpd-1; unc-43; asp-2; zk973.1; mtm-3; ubc-13; sup-17; arx-6; vha-16; tag-93; ptp-2; vha-2; gta-1; top-1; unc-60; cpr-6; t12a2.2; imb-3; f35g12.2; lin-45; apb-1; gsk-3; w07g4.4; w03f11.1; vha-12; c34d4.14; c09g4.2; f41c3.5; cgh-1; hda-1; grs-1; hmg-1.1; alh-8; t05h4.6a; cpr-1; nuo-4; skn-1; zfp-1; cey-1; rpb-2; t08b2.7; t23f11.1; f53h4.2; f36a2.13; mrs-1; f32e10.6; egl-4; y71f9al.17; lys-6; aco-2; clp-1; t25b9.9; tsn-1; f57b10.1; k08f8.1; rfc-1; tre-1; crs-1; zyg-11; f14b4.2; t04a8.7; r11a5.4; lys-5; unc-15; b0035.12; f57f5.1; egl-30; c01b4.6; mel-32; smk-1; f42g9.1; cey-3; let-70; f21h12.6; atf-7; let-711; y110a7a.6; atp-2; pqn-51; srs-2; cct-1; nhr-49; rpn-10; y19d10a.16; tlk-1; f40f9.6; rsks-1; cpz-1; smc-4; vrs-2; nhr-114; f28a12.4; lfi-1; kin-19; fat-7; ceh-38; cct-4; unc-54; wnk-1; c37c3.2; c48a7.2; ubc-25; f42d1.2; t05h10.1; f52c6.12; r07h5.8; glh-1; vha-4; cct-6; dao-5; r151.2; mut-16; c27a2.1; puf-8; pme-1; taf-13; egl-27; ifg-1; k10c2.1; b0495.2; c37h5.6; pes-9; zip-2; c06a8.1; f09f7.3; chc-1; cpl-1; vha-15; hif-1; math-33; lrs-1; vha-11; gpi-1;	9.25E-06	primary metabolic process;
GO:0046907	let-60; arf-1.2; rab-5; snap-1; npp-9; vps-26; sec-24.2; sec-24.1; npp-8; f21d5.7; c48a7.2; daf-18; rab-11.1; rab-1; t14g10.5; chc-1; y71f9al.17; imb-3; apb-1; ima-3; ran-5; unc-116; c13b9.3; imb-1; sec-23	1.44E-05	intracellular transport;

GO:0044237	spk-1; sams-1; cct-5; f52e1.13; cdc-48.2; glh-2; f53f10.2; f44e7.4; eel-1; pas-7; tag-182; hmg-3; prpf-4; rab-11.1; rab-1; b0285.1; zk836.2; kin-2; eif-3.c; zk829.4; vha-8; uaf-1; ldh-1; w02f12.5; lin-35; mxl-3; b0250.5; y105c5b.12; c25a8.4; vha-14; t13f2.2; him-1; tag-153; c06e7.3; lys-4; par-1; daf-18; dnj-12; t09a5.11; prp-8; c06e7.1; eif-3.b; pyc-1; rsp-3; ama-1; pbrm-1; cah-4; tufm-1; trs-1; imb-1; hpd-1; unc-43; asp-2; zk973.1; mtm-3; ubc-13; sup-17; arx-6; vha-16; tag-93; ptp-2; vha-2; unc-60; gta-1; top-1; cpr-6; t12a2.2; f35g12.2; imb-3; lin-45; gsk-3; w07g4.4; k02f2.2; w03f11.1; vha-12; c34d4.14; c09g4.2; f41c3.5; cgh-1; hda-1; grs-1; hmg-1.1; alh-8; t05h4.6a; cpr-1; nuo-4; skn-1; zfp-1; cey-1; rpb-2; t08b2.7; t23f11.1; f53h4.2; f36a2.13; mrs-1; f32e10.6; egl-4; zk1320.9; lys-6; aco-2; clp-1; t25b9.9; tsn-1; f57b10.1; k08f8.1; rfc-1; crs-1; zyg-11; f14b4.2; t04a8.7; r11a5.4; lys-5; b0035.12; f57f5.1; egl-30; mel-32; smk-1; f42g9.1; cey-3; let-70; f21h12.6; atf-7; y110a7a.6; atp-2; pqn-51; srs-2; cct-1; nhr-49; rpn-10; tlk-1; rsks-1; cpz-1; smc-4; mtce.25; vrs-2; nhr-114; f28a12.4; lfi-1; kin-19; fat-7; ceh-38; cct-4; wnk-1; c37c3.2; ubc-25; f42d1.2; t05h10.1; f52c6.12; r07h5.8; glh-1; vha-4; cct-6; dao-5; r151.2; mut-16; c27a2.1; puf-8; pme-1; taf-13; egl-27; ifg-1; k10c2.1; b0495.2; c37h5.6; c44b7.10; pes-9; zip-2; c06a8.1; f09f7.3; cpl-1; vha-15; hif-1; math-33; lrs-1; vha-11; gpi-1; pas-20; c36b1.7; emb-5;	1.44E-05	cellular metabolic process;
GO:0016192	chc-1; t14g10.5; y71f9al.17; mca-3; apb-1; sec-24.2; nud-2; ced-7; snb-1; sec-24.1; rme-1; c48a7.2; c13b9.3; cmd-1; dab-1; rab-11.1; sec-23	1.52E-05	vesicle-mediated transport;
GO:0040018	ost-1; erm-1; sams-1; vps-35; dpy-17; sec-24.2; phb-2; hmg-3; t25g3.3; top-1; b0285.1; h19n07.1; pqn-20; kin-2; gfi-2; c08h9.2; math-33; vha-11; unc-116; mel-26; lin-35; iftb-1; k07c5.4; y39g10ar.8; vrs-2; f55c5.8; lin-41; ccf-1; nmy-1; mbk-2; zc247.1; nuo-1; opt-2; eif-3.f; f57b10.1; csnk-1; egl-45; f53a2.7; f33h1.3; crs-1	2.02E-05	positive regulation of multicellular organism growth;
GO:0045184	vps-32.1; let-60; arf-1.2; rab-5; snap-1; sec-24.2; sec-24.1; f21d5.7; c48a7.2; daf-18; dab-1; rab-11.1; rab-1; t14g10.5; chc-1; y71f9al.17; imb-3; emb-9; apb-1; ima-3; c13b9.3; imb-1; sec-23	7.57E-05	establishment of protein localization;
GO:0000226	npp-10; ama-1; kin-19; tpxl-1; gsk-3; spd-5; tat-5; csn-2; par-1; mom-5; goa-1; csnk-1; imb-1; dnj-12; mel-26; mbk-2; sur-6	8.73E-05	microtubule cytoskeleton organization and biogenesis; spindle organization and biogenesis;
GO:0007051	npp-10; ama-1; kin-19; gsk-3; tpxl-1; spd-5; mom-5; goa-1; imb-1; dnj-12; sur-6; mbk-2	1.16E-04	spindle organization and biogenesis;
GO:0030154	let-60; asp-2; glh-2; sup-17; egl-27; pgl-1; vha-2; cmd-1; top-1; unc-60; smk-1; hrp-1; lin-45; unc-32; vha-12; hda-1; cgh-1; c55c3.5; mom-5; vha-10; r13f6.10; rab-5; skn-1; ced-7; unc-54; par-1; lin-41; unc-76; mbk-2; hrp-2; clp-1; glh-1; lmn-1	1.43E-04	cell differentiation;
GO:0006119	mtce.35; mtce.25; vha-13; nuo-1; vha-14; atp-2; vha-8; vha-15; vha-12; vha-11; vha-16; vha-2; vha-4	1.46E-04	oxidative phosphorylation;
GO:0006818	vha-13; unc-32; vha-14; atp-2; vha-8; vha-15; vha-12; vha-5; vha-11; vha-16; vha-2; vha-4	1.59E-04	hydrogen transport; proton transport;
GO:0015992	vha-13; unc-32; vha-14; atp-2; vha-8; vha-15; vha-12; vha-5; vha-11; vha-16; vha-2; vha-4	1.59E-04	proton transport;
GO:0048869	let-60; asp-2; glh-2; sup-17; egl-27; pgl-1; vha-2; cmd-1; top-1; unc-60; smk-1; hrp-1; lin-45; unc-32; vha-12; hda-1; cgh-1; c55c3.5; mom-5; vha-10; r13f6.10; rab-5; skn-1; ced-7; unc-54; par-1; lin-41; unc-76; mbk-2; hrp-2; clp-1; glh-1; lmn-1	1.74E-04	cellular developmental process;

GO:0006082	b0250.5; vrs-2; r11a5.4; fat-7; t08b2.7; mrs-1; gta-1; c06a8.1; mel-32; f42d1.2; irs-1; pyc-1; zk829.4; srs-2; lrs-1; trs-1; gpi-1; grs-1; alh-8; hpd-1; c36b1.7; crs-1; r03d7.1	1.93E-04	organic acid metabolic process;
GO:0019752	b0250.5; vrs-2; r11a5.4; fat-7; t08b2.7; mrs-1; gta-1; c06a8.1; mel-32; f42d1.2; irs-1; pyc-1; zk829.4; srs-2; lrs-1; trs-1; gpi-1; grs-1; alh-8; hpd-1; c36b1.7; crs-1; r03d7.1	1.93E-04	carboxylic acid metabolic process;
GO:0040025	let-60; dli-1; let-92; lin-45; sqv-5; mpk-1; hda-1; npa-1; sup-17; mep-1; par-1; ptp-2; c48a7.2; c37h5.6; lin-35; nol-5; sur-6	1.93E-04	vulval development;
GO:0065008	ost-1; mca-3; vps-35; pod-2; snap-1; mdt-15; apl-1; b0035.12; t21b10.3; eat-6; prp-8; f35g2.1; y71h2am.20; llc1.3; m03f4.6; npp-21; f43e2.7; opt-2; cct-1; ech-6; arx-2; vha-12; vha-5; c50f7.4; f57b10.1; pdi-1; vha-4; cct-6; sca-1; f29g6.3; y71h10b.1	1.93E-04	regulation of biological quality;
GO:0051186	b0250.5; vha-13; vha-14; cdc-48.2; vha-16; vha-2; c44b7.10; zk1320.9; f35g12.2; aco-2; atp-2; vha-15; vha-8; t25b9.9; vha-12; vha-11; w02f12.5; vha-4; r03d7.1	2.11E-04	cofactor metabolic process; coenzyme metabolic process; amine metabolic process; nitrogen compound metabolic process;
GO:0015985	vha-13; vha-14; atp-2; vha-8; vha-15; vha-12; vha-11; vha-16; vha-2; vha-4	2.19E-04	energy coupled proton transport, down electrochemical gradient; ATP biosynthetic process;
GO:0015986	vha-13; vha-14; atp-2; vha-8; vha-15; vha-12; vha-11; vha-16; vha-2; vha-4	2.19E-04	ATP synthesis coupled proton transport;
GO:0006519	b0250.5; vrs-2; f53f10.2; mrs-1; gta-1; c06a8.1; f42d1.2; mel-32; irs-1; zk829.4; srs-2; trs-1; lrs-1; grs-1; alh-8; hpd-1; c36b1.7; crs-1; r03d7.1	2.33E-04	amino acid and derivative metabolic process;
GO:0006753	vha-13; vha-14; atp-2; vha-8; vha-15; vha-12; vha-11; vha-16; vha-2; vha-4	2.62E-04	nucleoside phosphate metabolic process;
GO:0006754	vha-13; vha-14; atp-2; vha-8; vha-15; vha-12; vha-11; vha-16; vha-2; vha-4	2.62E-04	ATP biosynthetic process;
GO:0006520	b0250.5; vrs-2; mrs-1; c06a8.1; f42d1.2; mel-32; irs-1; zk829.4; srs-2; trs-1; lrs-1; grs-1; alh-8; c36b1.7; hpd-1; crs-1; r03d7.1	2.71E-04	amino acid metabolic process;
GO:0006996	erm-1; zyg-11; act-1; tat-5; arx-6; act-3; pgl-1; npp-7; unc-60; sur-6; gsk-3; ima-3; anc-1; hda-1; unc-116; mom-5; goa-1; mel-26; act-4; f10e7.5; smc-4; scc-3; kin-19; him-1; spd-5; npp-8; unc-54; par-1; dnj-12; fib-1; mbk-2; f32e10.6; npp-10; ama-1; tpxl-1; csn-2; csnk-1; imb-1; ran-4; lmn-1	2.71E-04	organelle organization and biogenesis;
GO:0007017	kin-19; spd-5; tat-5; par-1; dnj-12; sur-6; mbk-2; npp-10; ama-1; gsk-3; tpxl-1; csn-2; csnk-1; goa-1; unc-116; mom-5; imb-1; mel-26	3.49E-04	microtubule-based process;
GO:0006006	gpi-1; zk836.2; b0250.5; ldh-1; pyc-1; t22b11.5; f14b4.2; r11a5.4; t25b9.9	3.53E-04	glucose metabolic process;
GO:0006752	vha-13; vha-14; atp-2; vha-8; vha-15; vha-12; vha-11; vha-16; vha-2; vha-4; r03d7.1	3.53E-04	group transfer coenzyme metabolic process;
GO:0046034	vha-13; vha-14; atp-2; vha-8; vha-15; vha-12; vha-11; vha-16; vha-2; vha-4	3.68E-04	ATP metabolic process;
GO:0048519	sams-1; k08f4.2; npa-1; lin-41; ani-2; c48a7.2; daf-18; c37h5.6; t09a5.11; t12a2.2; d2096.11; npp-21; vab-10; dli-1; hrp-1; k02f2.2; lev-11; hda-1; cgh-1; mep-1; goa-1; lin-35; cki-2; nol-5	3.77E-04	negative regulation of biological process;

GO:0043170	spk-1; cct-5; glh-2; hex-1; f44e7.4; tag-182; hmg-3; pas-7; eel-1; prpf-4; rab-11.1; rab-1; b0285.1; t22f3.3; zk836.2; eif-3.c; kin-2; uaf-1; ldh-1; w02f12.5; lin-35; b0250.5; c25a8.4; t13f2.2; him-1; lys-4; par-1; dnj-12; daf-18; prp-8; t09a5.11; eif-3.b; pyc-1; rsp-3; ama-1; pbrm-1; tufm-1; trs-1; imb-1; f21d5.1; unc-43; asp-2; mtm-3; ubc-13; sup-17; arx-6; tag-93; ptp-2; top-1; unc-60; cpr-6; t12a2.2; f35g12.2; imb-3; lin-45; apb-1; gsk-3; w07g4.4; w03f11.1; c09g4.2; c34d4.14; hda-1; f41c3.5; cgh-1; grs-1; hmg-1.1; t05h4.6a; cpr-1; skn-1; cey-1; t23f11.1; f53h4.2; f36a2.13; mrs-1; f32e10.6; egl-4; y71f9al.17; lys-6; aco-2; clp-1; t25b9.9; f57b10.1; k08f8.1; rfc-1; tre-1; crs-1; zyg-11; f14b4.2; t04a8.7; r11a5.4; lys-5; unc-15; b0035.12; f57f5.1; egl-30; c01b4.6; smk-1; f42g9.1; cey-3; let-70; f21h12.6; atf-7; let-711; y110a7a.6; srs-2; cct-1; pqn-51; nhr-49; rpn-10; y19d10a.16; rsks-1; f40f9.6; tlk-1; cpz-1; smc-4; f28a12.4; nhr-114; vrs-2; lfi-1; kin-19; cct-4; ceh-38; unc-54; wnk-1; c37c3.2; c48a7.2; ubc-25; t05h10.1; f52c6.12; glh-1; cct-6; dao-5; c27a2.1; pme-1; puf-8; taf-13; ifg-1; egl-27; k10c2.1; b0495.2; pes-9; zip-2; chc-1; cpl-1; hif-1; math-33; lrs-1; gpi-1; c13b9.3; nas-20; wwp-1; iftb-1; daf-16; cdc-14; cey-2; cnx-1; lin-41; retr-1; w08e12.7; d2005.1; fib-1; lig-1; llc1.3; pap-1; mbk-2; t14g10.5; sot-25; irs-1; hsp-6; rpt-4; mpk-1; akt-1; aex-5; cspk-1; [illegible]	3.77E-04	macromolecule metabolic process;
GO:0044262	zk836.2; b0250.5; pyc-1; f35g12.2; aco-2; c25a8.4; f14b4.2; t04a8.7; y110a7a.6; r11a5.4; t25b9.9; w03f11.1; gpi-1; w02f12.5; ldh-1; t22b11.5; tre-1	4.50E-04	cellular carbohydrate metabolic process;
GO:0015031	vps-32.1; let-60; arf-1.2; rab-5; snap-1; sec-24.2; sec-24.1; f21d5.7; c48a7.2; daf-18; rab-11.1; rab-1; chc-1; t14g10.5; y71f9al.17; imb-3; apb-1; ima-3; c13b9.3; imb-1; sec-23	4.54E-04	protein transport;
GO:0009201	vha-13; vha-14; atp-2; vha-8; vha-15; vha-12; vha-11; vha-16; vha-2; vha-4	5.03E-04	ribonucleoside triphosphate biosynthetic process;
GO:0009145	vha-13; vha-14; atp-2; vha-8; vha-15; vha-12; vha-11; vha-16; vha-2; vha-4	5.03E-04	purine nucleoside triphosphate biosynthetic process;
GO:0009206	vha-13; vha-14; atp-2; vha-8; vha-15; vha-12; vha-11; vha-16; vha-2; vha-4	5.03E-04	purine ribonucleoside triphosphate biosynthetic process;
GO:0018988	f55c5.8; mtm-3; ppn-1; npp-9; apl-1; vha-15; anc-1; mua-6; k07c5.6; ifb-1; top-1; dab-1; cpz-1; crs-1; sec-23	5.23E-04	molting cycle, protein-based cuticle;
GO:0042303	f55c5.8; mtm-3; ppn-1; npp-9; apl-1; vha-15; anc-1; mua-6; k07c5.6; ifb-1; top-1; dab-1; cpz-1; crs-1; sec-23	5.82E-04	molting cycle;
GO:0005975	b0250.5; c25a8.4; f14b4.2; t04a8.7; r11a5.4; lys-5; unc-15; lys-4; hex-1; c01b4.6; zk836.2; t22f3.3; lys-6; f35g12.2; pyc-1; aco-2; let-711; y110a7a.6; w03f11.1; t25b9.9; gpi-1; ldh-1; w02f12.5; y19d10a.16; t22b11.5; f40f9.6; f21d5.1; tre-1	5.99E-04	carbohydrate metabolic process;
GO:0009057	b0250.5; zyg-11; c25a8.4; f14b4.2; lys-5; lys-4; pas-7; mbk-2; zk836.2; lys-6; t05h10.1; rpt-4; t25b9.9; gpi-1; math-33; ldh-1; rpn-10; t22b11.5; f30a10.10	5.99E-04	macromolecule catabolic process; cellular catabolic process;
GO:0009142	vha-13; vha-14; atp-2; vha-8; vha-15; vha-12; vha-11; vha-16; vha-2; vha-4	6.62E-04	nucleoside triphosphate biosynthetic process;
GO:0009199	vha-13; vha-14; atp-2; vha-8; vha-15; vha-12; vha-11; vha-16; vha-2; vha-4	6.62E-04	ribonucleoside triphosphate metabolic process;
GO:0009205	vha-13; vha-14; atp-2; vha-8; vha-15; vha-12; vha-11; vha-16; vha-2; vha-4	6.62E-04	purine ribonucleoside triphosphate metabolic process;

GO:0009144	vha-13; vha-14; atp-2; vha-8; vha-15; vha-12; vha-11; vha-16; vha-2; vha-4	6.62E-04	purine nucleoside triphosphate metabolic process;
GO:0019318	zk836.2; b0250.5; pyc-1; f14b4.2; r11a5.4; y110a7a.6; t25b9.9; gpi-1; ldh-1; t22b11.5	6.62E-04	hexose metabolic process;
GO:0051726	atx-2; zyg-11; d2096.11; vab-10; gld-2; rpn-1; cgh-1; nos-3; puf-8; cmd-1; ccf-1; gld-3; cki-2	7.47E-04	regulation of cell cycle;
GO:0005996	zk836.2; b0250.5; pyc-1; f14b4.2; r11a5.4; y110a7a.6; t25b9.9; gpi-1; ldh-1; t22b11.5	7.75E-04	monosaccharide metabolic process;
GO:0001666	unc-32; hif-1; vha-12	7.99E-04	response to hypoxia;
GO:0009253	lys-4; lys-6; lys-5	7.99E-04	peptidoglycan catabolic process;
GO:0000270	lys-4; lys-6; lys-5	7.99E-04	peptidoglycan metabolic process;
GO:0019098	mtm-3; ptc-1; puf-12; sqv-5; b0035.12; unc-15; unc-54; wnk-1; egl-27; egl-30; scpl-1; dab-1; smk-1; sur-6; atx-2; m03f4.6; ptc-2; ent-2; atp-2; unc-61; lev-11; csn-2; csnk-1; goa-1; mom-5; egl-45; lin-35; e01a2.2	8.04E-04	reproductive behavior;
GO:0006164	vha-13; vha-14; atp-2; vha-8; vha-15; vha-12; vha-11; vha-16; vha-2; c37h5.6; vha-4	8.57E-04	purine nucleotide biosynthetic process;
GO:0009260	vha-13; r151.2; vha-14; atp-2; vha-8; vha-15; vha-12; vha-11; vha-16; vha-2; vha-4	8.57E-04	ribonucleotide biosynthetic process;
GO:0009141	vha-13; vha-14; atp-2; vha-8; vha-15; vha-12; vha-11; vha-16; vha-2; vha-4	1.02E-03	nucleoside triphosphate metabolic process;
GO:0009259	vha-13; r151.2; vha-14; atp-2; vha-8; vha-15; vha-12; vha-11; vha-16; vha-2; vha-4	1.30E-03	ribonucleotide metabolic process;
GO:0006163	vha-13; vha-14; atp-2; vha-8; vha-15; vha-12; vha-11; vha-16; vha-2; c37h5.6; vha-4	1.30E-03	purine nucleotide metabolic process;
GO:0018991	mtm-3; ptc-1; puf-12; sqv-5; b0035.12; unc-15; unc-54; wnk-1; egl-27; egl-30; scpl-1; dab-1; smk-1; sur-6; atx-2; m03f4.6; ptc-2; ent-2; unc-61; lev-11; csn-2; csnk-1; goa-1; mom-5; egl-45; lin-35; e01a2.2	1.58E-03	oviposition;
GO:0006869	tat-5; vit-3; vit-1; vit-6; vit-4; tat-4	1.58E-03	lipid transport;
GO:0033057	mtm-3; ptc-1; puf-12; sqv-5; b0035.12; unc-15; unc-54; wnk-1; egl-27; egl-30; scpl-1; dab-1; smk-1; sur-6; atx-2; m03f4.6; ptc-2; ent-2; unc-61; lev-11; csn-2; csnk-1; goa-1; mom-5; egl-45; lin-35; e01a2.2	1.58E-03	reproductive behavior in a multicellular organism;
GO:0007308	hrp-2; hrp-1; pgl-1; c55c3.5; r13f6.10; top-1; smk-1	1.62E-03	oocyte construction; oocyte differentiation;
GO:0051656	zyg-11; dli-1; gsk-3; spd-5; tat-5; c53h9.2; par-1; csn-2; goa-1; csnk-1; mom-5; mel-26; dyci-1; mbk-2	1.73E-03	establishment of organelle localization;
GO:0019538	spk-1; zyg-11; cct-5; f57f5.1; egl-30; pas-7; eel-1; f44e7.4; prpf-4; b0285.1; f42g9.1; let-70; f21h12.6; eif-3.c; kin-2; cct-1; srs-2; rpn-10; tlk-1; rsks-1; cpz-1; f28a12.4; vrs-2; kin-19; cct-4; unc-54; wnk-1; par-1; c37c3.2; c48a7.2; ubc-25; dnj-12; daf-18; t09a5.11; eif-3.b; f52c6.12; t05h10.1; tufm-1; trs-1; imb-1; cct-6; unc-43; asp-2; mtm-3; ubc-13; sup-17; arx-6; pme-1; puf-8; tag-93; ptp-2; k10c2.1; pes-9; b0495.2; unc-60; cpr-6; t12a2.2; cpl-1; chc-1; imb-3; lin-45; apb-1; gsk-3; w07g4.4; c34d4.14; c09g4.2; math-33; lrs-1; hda-1; f41c3.5; grs-1; t05h4.6a; c13b9.3; cpr-1; nas-20; wwp-1; iftb-1; cdc-14; cnx-1; t23f11.1; retr-1; lin-41; f53h4.2; w08e12.7; f36a2.13; mrs-1; mbk-2; egl-4; t14g10.5; irs-1; y71f9al.17; hsp-6; clp-1; rpt-4; akt-1; mpk-1; aex-5; csnk-1; y41d4a.5; k08f8.1; f30a10.10; hsp-60; t15h9.1; crs-1	1.73E-03	protein metabolic process;

GO:0007281	hrp-2; hrp-1; glh-2; glh-1; pgl-1; c55c3.5; top-1; lmn-1; r13f6.10; smk-1	1.76E-03	germ cell development;
GO:0050793	let-60; let-92; npa-1; lin-41; ptp-2; c48a7.2; unc-76; c37h5.6; fib-1; nmy-1; sur-6; dli-1; nmy-2; hda-1; mep-1; lin-35; nol-5	1.76E-03	regulation of developmental process;
GO:0043071	vha-2; unc-32; vha-10; vha-12	1.94E-03	positive regulation of non-apoptotic programmed cell death;
GO:0040017	y105c5b.12; mca-3; apl-1; lev-11; nhr-49; c08h9.2; c06e7.3; egl-30; pmt-1; dab-1; ntl-4; lst-3; tnt-2; c06e7.1	2.02E-03	positive regulation of locomotion;
GO:0051640	zyg-11; dli-1; gsk-3; spd-5; tat-5; c53h9.2; par-1; csn-2; goa-1; csnk-1; mom-5; mel-26; dyci-1; mbk-2	2.02E-03	organelle localization;
GO:0009152	vha-13; vha-14; atp-2; vha-8; vha-15; vha-12; vha-11; vha-16; vha-2; vha-4	2.62E-03	purine ribonucleotide biosynthetic process;
GO:0000132	tat-5; csn-2; mom-5; csnk-1; goa-1; mel-26; gsk-3; mbk-2	2.75E-03	establishment of mitotic spindle orientation;
GO:0048609	mtm-3; ptc-1; puf-12; sqv-5; b0035.12; unc-15; unc-54; wnk-1; egl-27; egl-30; scpl-1; dab-1; smk-1; sur-6; atx-2; m03f4.6; ptc-2; ent-2; unc-61; lev-11; csn-2; csnk-1; goa-1; mom-5; egl-45; lin-35; e01a2.2	2.75E-03	reproductive process in a multicellular organism;
GO:0032504	mtm-3; ptc-1; puf-12; sqv-5; b0035.12; unc-15; unc-54; wnk-1; egl-27; egl-30; scpl-1; dab-1; smk-1; sur-6; atx-2; m03f4.6; ptc-2; ent-2; unc-61; lev-11; csn-2; csnk-1; goa-1; mom-5; egl-45; lin-35; e01a2.2	2.75E-03	multicellular organism reproduction;
GO:0051294	tat-5; csn-2; mom-5; csnk-1; goa-1; mel-26; gsk-3; mbk-2	2.75E-03	establishment of spindle orientation;
GO:0006084	zk1320.9; w02f12.5; f35g12.2; aco-2; c44b7.10	2.81E-03	acetyl-CoA metabolic process;
GO:0009308	b0250.5; vrs-2; c25a8.4; f53f10.2; mrs-1; gta-1; c06a8.1; f42d1.2; mel-32; irs-1; zk829.4; srs-2; w03f11.1; trs-1; lrs-1; grs-1; alh-8; hpd-1; c36b1.7; crs-1; r03d7.1	2.83E-03	amine metabolic process;
GO:0006461	chc-1; t14g10.5; y71f9al.17; imb-3; apb-1; unc-54; c48a7.2; imb-1; c13b9.3	3.13E-03	protein complex assembly;
GO:0000074	zyg-11; d2096.11; vab-10; gld-2; nos-3; puf-8; gld-3; cki-2	3.16E-03	regulation of progression through cell cycle;
GO:0019320	gpi-1; zk836.2; b0250.5; ldh-1; t22b11.5; f14b4.2; t25b9.9	3.37E-03	hexose catabolic process;
GO:0006007	gpi-1; zk836.2; b0250.5; ldh-1; t22b11.5; f14b4.2; t25b9.9	3.37E-03	glucose catabolic process;
GO:0046365	gpi-1; zk836.2; b0250.5; ldh-1; t22b11.5; f14b4.2; t25b9.9	3.37E-03	monosaccharide catabolic process; hexose catabolic process;glucose catabolic process; alcohol catabolic process;
GO:0009150	vha-13; vha-14; atp-2; vha-8; vha-15; vha-12; vha-11; vha-16; vha-2; vha-4	3.58E-03	purine ribonucleotide metabolic process;
GO:0016052	zk836.2; b0250.5; lys-6; c25a8.4; f14b4.2; t25b9.9; lys-5; gpi-1; lys-4; ldh-1; t22b11.5	3.83E-03	carbohydrate catabolic process; hexose metabolic process; alcohol metabolic process;
GO:0009108	vha-13; vha-14; atp-2; vha-8; vha-15; vha-12; vha-11; vha-16; vha-2; vha-4; r03d7.1	4.27E-03	coenzyme biosynthetic process;
GO:0040020	nos-3; puf-8; gld-2; gld-3	4.70E-03	regulation of meiosis;

GO:0006807	b0250.5; vrs-2; c25a8.4; f53f10.2; mrs-1; gta-1; c06a8.1; f42d1.2; mel-32; irs-1; zk829.4; srs-2; w03f11.1; trs-1; lrs-1; grs-1; alh-8; hpd-1; c36b1.7; crs-1; r03d7.1	4.75E-03	nitrogen compound metabolic process;
GO:0051234	let-60; zyg-11; vit-6; mca-3; npp-9; dpy-17; snb-1; k07h8.2; col-143; eel-1; cmd-1; rab-11.1; rab-1; col-179; unc-32; atp-2; f43e2.7; vha-8; rme-1; mom-5; mel-26; sec-23; rab-5; vha-14; col-95; par-1; tat-4; c48a7.2; t10f2.2; daf-18; col-181; col-119; opt-2; nud-2; let-2; imb-1; col-20; vha-4; sca-1; vps-32.1; arf-1.2; vps-26; sec-24.2; y19d10a.12; clh-1; tat-5; y19d10a.4; vit-1; vha-16; c01b4.7; vha-2; dab-1; chc-1; glt-1; emb-9; imb-3; apb-1; gsk-3; ima-3; vha-15; vha-12; vha-11; c53h9.2; goa-1; unc-116; c13b9.3; vit-3; vha-13; snap-1; ced-7; k08f4.2; col-178; spd-5; sec-24.1; npp-8; col-106; f21d5.7; eat-6; aqp-8; pmp-5; dyci-1; c01b4.9; r05f9.1; mbk-2; t14g10.5; y71f9al.17; dli-1; npp-10; ent-2; vha-5; csn-2; ran-5; aex-5; aqp-2; csnk-1; ran-4; vit-4	4.75E-03	establishment of localization;
GO:0051293	gsk-3; tat-5; csn-2; par-1; goa-1; csnk-1; mom-5; mel-26; mbk-2	5.06E-03	establishment of spindle localization;
GO:0051653	gsk-3; tat-5; csn-2; par-1; goa-1; csnk-1; mom-5; mel-26; mbk-2	5.06E-03	spindle localization;
GO:0040001	gsk-3; tat-5; csn-2; par-1; goa-1; csnk-1; mom-5; mel-26; mbk-2	5.06E-03	establishment of mitotic spindle localization;
GO:0006094	gpi-1; pyc-1; r11a5.4	5.43E-03	gluconeogenesis; pyruvate metabolic process; monosaccharide biosynthetic process; hexose biosynthetic process;
GO:0046164	gpi-1; zk836.2; b0250.5; ldh-1; t22b11.5; f14b4.2; t25b9.9	5.45E-03	alcohol catabolic process;
GO:0048477	let-60; hrp-2; hrp-1; ima-3; ptp-2; pgl-1; c55c3.5; top-1; r13f6.10; smk-1	5.45E-03	oogenesis; female gamete generation;
GO:0006913	let-60; rab-5; imb-3; ima-3; npp-8; imb-1; daf-18; rab-1; rab-11.1	5.56E-03	nucleocytoplasmic transport; nuclear transport;
GO:0051169	let-60; rab-5; imb-3; ima-3; npp-8; imb-1; daf-18; rab-1; rab-11.1	5.56E-03	nuclear transport;
GO:0044248	b0250.5; zyg-11; c25a8.4; f14b4.2; f52e1.13; lys-5; lys-4; pas-7; zk836.2; lys-6; f35g12.2; aco-2; t05h10.1; t25b9.9; gpi-1; math-33; ldh-1; rpn-10; w02f12.5; t22b11.5; f30a10.10	6.97E-03	cellular catabolic process;
GO:0032940	rab-5; emb-9; sec-24.2; snb-1; sec-24.1; f21d5.7; aex-5; dab-1; sec-23	6.97E-03	secretion by cell;
GO:0043039	trs-1; lrs-1; irs-1; grs-1; vrs-2; mrs-1; srs-2; crs-1	6.97E-03	tRNA aminoacylation; amino acid activation; tRNA aminoacylation for protein translation;
GO:0043038	trs-1; lrs-1; irs-1; grs-1; vrs-2; mrs-1; srs-2; crs-1	6.97E-03	amino acid activation;
GO:0006418	trs-1; lrs-1; irs-1; grs-1; vrs-2; mrs-1; srs-2; crs-1	6.97E-03	tRNA aminoacylation for protein translation;
GO:0055086	b0250.5; vha-13; r151.2; vha-14; vha-16; vha-2; c37h5.6; atp-2; r07h5.8; vha-8; t25b9.9; vha-15; vha-12; vha-11; vha-4; c36b1.7	7.77E-03	nucleobase, nucleoside and nucleotide metabolic process;

GO:0009117	b0250.5; vha-13; r151.2; vha-14; atp-2; vha-8; t25b9.9; vha-15; vha-12; vha-11; vha-16; vha-2; c37h5.6; vha-4; c36b1.7	8.29E-03	nucleotide metabolic process;
GO:0009165	vha-13; r151.2; vha-14; atp-2; vha-8; vha-15; vha-12; vha-11; vha-16; vha-2; c37h5.6; vha-4; c36b1.7	8.95E-03	nucleotide biosynthetic process;
GO:0009948	zyg-11; par-1; pgl-1; nmy-2	9.01E-03	anterior/posterior axis specification; axis specification; anterior/posterior pattern formation;
GO:0006090	gpi-1; pyc-1; r11a5.4	9.51E-03	pyruvate metabolic process;
GO:0007050	d2096.11; vab-10; cki-2	9.51E-03	cell cycle arrest; negative regulation of progression through cell cycle;
GO:0009994	hrp-2; hrp-1; pgl-1; c55c3.5; r13f6.10; top-1; smk-1	9.65E-03	oocyte differentiation;
GO:0048599	hrp-2; hrp-1; pgl-1; c55c3.5; r13f6.10; top-1; smk-1	9.65E-03	oocyte development;
GO:0048468	asp-2; rab-5; ced-7; glh-2; unc-54; pgl-1; vha-2; cmd-1; unc-76; unc-60; top-1; smk-1; hrp-2; hrp-1; unc-32; clp-1; vha-12; cgh-1; glh-1; c55c3.5; vha-10; r13f6.10; lmn-1	1.13E-02	cell differentiation#cell development;
GO:0006555	c06a8.1; r03d7.1	1.13E-02	methionine metabolic process; aspartate family amino acid metabolic process;
GO:0007292	let-60; hrp-2; hrp-1; ima-3; ptp-2; pgl-1; c55c3.5; top-1; r13f6.10; smk-1	1.16E-02	female gamete generation;
GO:0043070	vha-2; unc-32; vha-10; vha-12	1.19E-02	regulation of non-apoptotic programmed cell death;
GO:0044267	spk-1; zyg-11; cct-5; f57f5.1; egl-30; pas-7; eel-1; f44e7.4; prpf-4; b0285.1; f42g9.1; let-70; f21h12.6; eif-3.c; kin-2; cct-1; srs-2; rpn-10; tlk-1; rsks-1; cpz-1; f28a12.4; vrs-2; kin-19; cct-4; wnk-1; par-1; c37c3.2; dnj-12; daf-18; ubc-25; t09a5.11; eif-3.b; t05h10.1; f52c6.12; tufm-1; trs-1; imb-1; cct-6; unc-43; asp-2; mtm-3; ubc-13; sup-17; arx-6; pme-1; tag-93; puf-8; ptp-2; k10c2.1; pes-9; b0495.2; unc-60; cpr-6; t12a2.2; cpl-1; imb-3; lin-45; gsk-3; w07g4.4; c09g4.2; c34d4.14; math-33; lrs-1; f41c3.5; hda-1; grs-1; t05h4.6a; cpr-1; nas-20; wwp-1; iftb-1; cdc-14; cnx-1; t23f11.1; lin-41; retr-1; f53h4.2; w08e12.7; f36a2.13; mrs-1; mbk-2; egl-4; irs-1; hsp-6; clp-1; akt-1; mpk-1; aex-5; csnk-1; y41d4a.5; k08f8.1; f30a10.10; t15h9.1; hsp-60; crs-1	1.25E-02	cellular protein metabolic process;
GO:0044260	spk-1; zyg-11; cct-5; t04a8.7; f57f5.1; f44e7.4; egl-30; pas-7; eel-1; prpf-4; b0285.1; f42g9.1; let-70; f21h12.6; eif-3.c; kin-2; srs-2; cct-1; rpn-10; tlk-1; rsks-1; cpz-1; f28a12.4; vrs-2; c25a8.4; kin-19; cct-4; wnk-1; par-1; c37c3.2; ubc-25; dnj-12; daf-18; t09a5.11; eif-3.b; f52c6.12; t05h10.1; tufm-1; trs-1; imb-1; cct-6; unc-43; asp-2; mtm-3; ubc-13; sup-17; arx-6; pme-1; tag-93; puf-8; ptp-2; k10c2.1; pes-9; b0495.2; unc-60; cpr-6; t12a2.2; cpl-1; imb-3; lin-45; gsk-3; w07g4.4; w03f11.1; c09g4.2; c34d4.14; math-33; lrs-1; hda-1; f41c3.5; grs-1; t05h4.6a; cpr-1; nas-20; wwp-1; iftb-1; cdc-14; cnx-1; t23f11.1; retr-1; lin-41; f53h4.2; w08e12.7; f36a2.13; mrs-1; mbk-2; egl-4; irs-1; hsp-6; clp-1; akt-1; mpk-1; aex-5; csnk-1; y41d4a.5; k08f8.1; f30a10.10; t15h9.1; hsp-60; crs-1	1.35E-02	cellular macromolecule metabolic process;

GO:0040028	hda-1; npa-1; mep-1; ptp-2; let-92; dli-1; c48a7.2; c37h5.6; lin-35; nol-5; sur-6	1.41E-02	regulation of vulval development;
GO:0007517	unc-54; let-60; emb-9; ptp-2; unc-60; act-1; unc-52	1.43E-02	muscle development;
GO:0000281	act-3; mlc-4; mbk-2	1.50E-02	cytokinesis after mitosis;
GO:0010382	lys-4; lys-6; f52e1.13; lys-5	1.53E-02	cell wall metabolic process; cell wall catabolic process; cell wall organization and biogenesis; peptidoglycan catabolic process;
GO:0016998	lys-4; lys-6; f52e1.13; lys-5	1.53E-02	cell wall catabolic process;
GO:0016244	vha-2; unc-32; vha-10; vha-12	1.53E-02	non-apoptotic programmed cell death;
GO:0040012	y105c5b.12; mca-3; apl-1; c06e7.3; egl-27; egl-30; pmt-1; dab-1; lst-3; c06e7.1; lev-11; nhr-49; c08h9.2; goa-1; ntl-4; tnt-2	1.54E-02	regulation of locomotion;
GO:0040027	hda-1; npa-1; mep-1; dli-1; c48a7.2; c37h5.6; lin-35; nol-5	1.70E-02	negative regulation of vulval development;
GO:0044265	zk836.2; b0250.5; zyg-11; c25a8.4; f14b4.2; t05h10.1; t25b9.9; gpi-1; math-33; rpn-10; ldh-1; pas-7; t22b11.5; f30a10.10	1.88E-02	cellular macromolecule catabolic process;
GO:0006606	imb-3; imb-1; daf-18; ima-3	1.91E-02	protein import into nucleus;
GO:0007047	lys-4; lys-6; f52e1.13; lys-5	1.91E-02	cell wall organization and biogenesis;
GO:0045229	lys-4; lys-6; f52e1.13; lys-5	1.91E-02	external encapsulating structure organization and biogenesis;
GO:0051170	imb-3; imb-1; daf-18; ima-3	1.91E-02	nucleocytoplasmic transport%nuclear import; protein import into nucleus;
GO:0055082	mca-3; eat-6; sca-1; vha-12	1.91E-02	cellular chemical homeostasis; cellular ion homeostasis;
GO:0006873	mca-3; eat-6; sca-1; vha-12	1.91E-02	cellular ion homeostasis;
GO:0044249	r151.2; r11a5.4; cdc-48.2; f53f10.2; puf-8; vha-16; vha-2; c37h5.6; eif-3.c; atp-2; vha-15; vha-8; srs-2; vha-12; vha-11; lrs-1; gpi-1; grs-1; t05h4.6a; c36b1.7; r03d7.1; iftb-1; vha-13; vrs-2; vha-14; fat-7; lin-41; c37c3.2; let-721; mrs-1; eif-3.b; irs-1; pyc-1; tufm-1; trs-1; vha-4; crs-1	1.92E-02	cellular biosynthetic process;
GO:0006730	c06e7.3; y105c5b.12; sams-1; k02f2.2; c06e7.1; cah-4	1.94E-02	one-carbon compound metabolic process;
GO:0051093	hda-1; npa-1; mep-1; dli-1; c48a7.2; c37h5.6; lin-35; nol-5	2.01E-02	negative regulation of developmental process;
GO:0000278	gsk-3; him-1; tat-5; csn-2; par-1; mom-5; goa-1; csnk-1; lmn-1; mel-26; mbk-2; scc-3	2.08E-02	mitotic cell cycle;
GO:0033205	act-3; mlc-4; mbk-2	2.10E-02	cytokinesis during cell cycle;
GO:0009056	b0250.5; zyg-11; c25a8.4; f14b4.2; f52e1.13; lys-5; lys-4; pas-7; mbk-2; zk836.2; lys-6; f35g12.2; aco-2; t05h10.1; rpt-4; t25b9.9; math-33; gpi-1; rpn-10; ldh-1; w02f12.5; t22b11.5; f30a10.10	2.28E-02	catabolic process;
GO:0006096	gpi-1; zk836.2; ldh-1; t22b11.5; f14b4.2	2.28E-02	glycolysis;

GO:0046903	rab-5; emb-9; sec-24.2; snb-1; sec-24.1; f21d5.7; aex-5; dab-1; sec-23	2.30E-02	secretion;
GO:0006512	f36a2.13; let-70; eel-1; ubc-25; f52c6.12; wwp-1; ubc-13; c34d4.14	2.40E-02	ubiquitin cycle;
GO:0030029	unc-54; erm-1; act-3; arx-6; unc-60; act-1; act-4	2.40E-02	actin filament-based process;
GO:0007169	dao-5; daf-16; ptp-2; unc-52; akt-1	2.65E-02	transmembrane receptor protein tyrosine kinase signaling pathway;
GO:0022618	iftb-1; c37c3.2; eif-3.c; rsp-3; eif-3.b	2.65E-02	protein-RNA complex assembly;
GO:0006573	b0250.5; alh-8	2.67E-02	valine metabolic process; branched chain family amino acid metabolic process;
GO:0007062	him-1; scc-3	2.67E-02	sister chromatid cohesion;
GO:0006999	ima-3; lmn-1	2.67E-02	nuclear pore organization and biogenesis; protein import into nucleus, docking;
GO:0016476	nmy-2; nmy-1	2.67E-02	shape changes of embryonic cells; regulation of cell shape;
GO:0022607	iftb-1; unc-54; taf-13; c37c3.2; pgl-1; c48a7.2; unc-60; eif-3.b; chc-1; t14g10.5; y71f9al.17; imb-3; rsp-3; eif-3.c; apb-1; pqn-51; imb-1; c13b9.3	2.72E-02	cellular component assembly;
GO:0040022	atx-2; puf-8; gld-3	2.80E-02	feminization of hermaphroditic germ-line;
GO:0009607	hsp-16.11; sca-1; hsp-16.1; smk-1	2.80E-02	response to biotic stimulus;
GO:0009306	rab-5; emb-9; dab-1	2.80E-02	protein secretion;
GO:0006888	sec-24.1; sec-24.2; sec-23	2.80E-02	ER to Golgi vesicle-mediated transport; Golgi vesicle transport;
GO:0022613	iftb-1; c37c3.2; eif-3.c; rsp-3; fib-1; f10e7.5; eif-3.b	2.85E-02	ribonucleoprotein complex biogenesis and assembly;
GO:0022404	f55c5.8; mtm-3; ppn-1; npp-9; ifb-1; apl-1; vha-15	3.15E-02	molting cycle process;
GO:0018996	f55c5.8; mtm-3; ppn-1; npp-9; ifb-1; apl-1; vha-15	3.15E-02	molting cycle, collagen and cuticulin-based cuticle;
GO:0040015	npp-21; sams-1; hrp-1; lev-11; k02f2.2; k08f4.2; ani-2; t09a5.11; t12a2.2	3.31E-02	negative regulation of multicellular organism growth;
GO:0051188	vha-13; vha-14; atp-2; vha-8; cdc-48.2; vha-15; vha-12; vha-11; vha-16; vha-2; vha-4; r03d7.1	3.36E-02	cofactor biosynthetic process;
GO:0065003	iftb-1; unc-54; taf-13; c37c3.2; c48a7.2; eif-3.b; chc-1; t14g10.5; y71f9al.17; imb-3; eif-3.c; rsp-3; apb-1; pqn-51; c13b9.3; imb-1	3.50E-02	macromolecular complex assembly;
GO:0045926	npp-21; sams-1; hrp-1; lev-11; k02f2.2; k08f4.2; ani-2; t09a5.11; t12a2.2	3.53E-02	negative regulation of growth;
GO:0016044	mca-3; ced-7; vha-12; rme-1; ran-4; cmd-1; npp-7; lmn-1; rab-11.1	3.53E-02	membrane organization and biogenesis;

GO:0046364	gpi-1; pyc-1; r11a5.4	3.61E-02	monosaccharide biosynthetic process;
GO:0019319	gpi-1; pyc-1; r11a5.4	3.61E-02	hexose biosynthetic process;
GO:0009408	daf-16; daf-18; skn-1	3.61E-02	response to stress%response to heat;
GO:0046165	gpi-1; pyc-1; r11a5.4	3.61E-02	alcohol biosynthetic process;
GO:0018985	ran-4; lmn-1; npp-7	3.61E-02	pronuclear envelope synthesis; nuclear membrane organization and biogenesis;
GO:0000377	rsp-3; prp-8; uaf-1	3.61E-02	RNA splicing, via transesterification reactions with bulged adenosine as nucleophile; nuclear mRNA splicing, via spliceosome;
GO:0000398	rsp-3; prp-8; uaf-1	3.61E-02	nuclear mRNA splicing, via spliceosome;
GO:0006457	hsp-6; cct-5; cct-1; cct-4; cnx-1; dnj-12; cct-6; t15h9.1; hsp-60	3.69E-02	protein folding;
GO:0050801	mca-3; eat-6; opt-2; sca-1; vha-12	3.76E-02	ion homeostasis;
GO:0015980	w02f12.5; f35g12.2; aco-2; t04a8.7; tre-1	3.76E-02	energy derivation by oxidation of organic compounds;
GO:0040021	nos-3; atx-2; puf-8; gld-3	3.83E-02	hermaphrodite germ-line sex determination; regulation of meiosis;
GO:0009798	zyg-11; par-1; pgl-1; nmy-2	3.83E-02	axis specification;
GO:0006399	irs-1; vrs-2; srs-2; trs-1; lrs-1; grs-1; mrs-1; llc1.3; crs-1	3.92E-02	tRNA metabolic process;
GO:0006139	glh-2; b0035.12; tag-182; hmg-3; rab-1; rab-11.1; smk-1; cey-3; atf-7; atp-2; pqn-51; uaf-1; vha-8; srs-2; nhr-49; lin-35; smc-4; mxl-3; b0250.5; nhr-114; vrs-2; lfi-1; vha-14; t13f2.2; him-1; ceh-38; tag-153; prp-8; rsp-3; ama-1; pbrm-1; r07h5.8; trs-1; glh-1; vha-4; zk973.1; dao-5; r151.2; mut-16; c27a2.1; taf-13; pme-1; vha-16; vha-2; egl-27; ifg-1; c37h5.6; b0495.2; zip-2; top-1; f09f7.3; vha-15; hif-1; vha-12; vha-11; cgh-1; hda-1; lrs-1; grs-1; hmg-1.1; c36b1.7; emb-5; nuo-4; vha-13; daf-16; cdc-14; skn-1; zfp-1; cey-2; cey-1; rpb-2; retr-1; mrs-1; d2005.1; fib-1; lig-1; llc1.3; pap-1; f32e10.6; set-25; irs-1; t25b9.9; tsn-1; f57b10.1; adr-2; rfc-1; crs-1	3.99E-02	nucleobase, nucleoside, nucleotide and nucleic acid metabolic process;
GO:0006091	mtce.35; mtce.25; vha-13; acdh-1; e04f6.5; vha-14; t04a8.7; f28a10.6; vha-16; vha-2; let-721; llc1.3; t05h4.5; f35g12.2; nuo-1; aco-2; atp-2; vha-8; vha-15; vha-12; vha-11; w02f12.5; vha-4; tag-165; tre-1; ivd-1; r02d3.1	4.13E-02	generation of precursor metabolites and energy;
GO:0045786	d2096.11; vab-10; cki-2	4.51E-02	negative regulation of progression through cell cycle;
GO:0000375	rsp-3; prp-8; uaf-1	4.51E-02	RNA splicing, via transesterification reactions;
GO:0048193	sec-24.1; sec-24.2; sec-23	4.51E-02	Golgi vesicle transport;

GO:0008286	dao-5; daf-16; akt-1	4.51E-02	insulin receptor signaling pathway;
GO:0006423	lrs-1; crs-1	4.51E-02	cysteinyl-tRNA aminoacylation;
GO:0006471	pme-1; egl-30	4.51E-02	protein amino acid ADP-ribosylation;
GO:0032787	gpi-1; t08b2.7; pyc-1; gta-1; r11a5.4; fat-7; r03d7.1	4.67E-02	monocarboxylic acid metabolic process;
GO:0007389	zyg-11; par-1; pgl-1; nmy-2; skn-1; mbk-2	4.89E-02	multicellular organismal development#pattern specification process;
GO:0006413	iftb-1; c37c3.2; eif-3.c; eif-3.b	5.23E-02	translational initiation;
GO:0030163	math-33; rpn-10; zyg-11; pas-7; f30a10.10; t05h10.1; rpt-4; mbk-2	5.38E-02	protein catabolic process;
GO:0030036	unc-54; erm-1; arx-6; unc-60; act-1; act-4	5.38E-02	actin cytoskeleton organization and biogenesis;
GO:0006984	hsp-16.11; sca-1; hsp-16.1	5.56E-02	ER-nuclear signaling pathway;
GO:0051789	hsp-16.11; sca-1; hsp-16.1	5.56E-02	response to protein stimulus;
GO:0030968	hsp-16.11; sca-1; hsp-16.1	5.56E-02	unfolded protein response;
GO:0006986	hsp-16.11; sca-1; hsp-16.1	5.56E-02	response to stress%response to unfolded protein; ER-nuclear signaling pathway;
GO:0030003	mca-3; sca-1; vha-12	5.56E-02	cellular cation homeostasis;
GO:0007028	rab-5; pgl-1; f57b10.1	5.56E-02	cytoplasm organization and biogenesis;
GO:0040006	f55c5.8; mtm-3; ppn-1; npp-9; ifb-1; vha-15	5.76E-02	molting cycle, protein-based cuticle#protein-based cuticle attachment to epithelium;
GO:0043067	cgh-1; vha-2; unc-32; vha-10; cmd-1; vha-12	5.76E-02	regulation of programmed cell death;
GO:0040004	f55c5.8; mtm-3; ppn-1; npp-9; ifb-1; vha-15	5.76E-02	collagen and cuticulin-based cuticle attachment to epithelium;
GO:0009952	zyg-11; par-1; pgl-1; nmy-2	5.87E-02	anterior/posterior pattern formation;
GO:0044275	gpi-1; zk836.2; b0250.5; ldh-1; t22b11.5; c25a8.4; f14b4.2; t25b9.9	6.01E-02	cellular carbohydrate catabolic process;
GO:0007338	dli-1; ran-4; dyci-1; lmn-1; npp-7; mbk-2; spd-5	6.26E-02	single fertilization;
GO:0009566	dli-1; ran-4; dyci-1; lmn-1; npp-7; mbk-2; spd-5	6.26E-02	reproductive process%fertilization; single fertilization;
GO:0009081	b0250.5; alh-8	6.36E-02	branched chain family amino acid metabolic process;
GO:0051728	cgh-1; atx-2; ccf-1	6.36E-02	cell cycle switching, mitotic to meiotic cell cycle;

GO:0000059	imb-3; imb-1	6.36E-02	protein import into nucleus, docking;
GO:0017038	imb-3; imb-1; daf-18; ima-3	6.36E-02	protein import;
GO:0006810	let-60; vit-6; mca-3; npp-9; dpy-17; snb-1; col-143; k07h8.2; eel-1; cmd-1; rab-11.1; rab-1; col-179; unc-32; atp-2; f43e2.7; vha-8; rme-1; sec-23; rab-5; vha-14; col-95; tat-4; c48a7.2; t10f2.2; daf-18; col-181; col-119; opt-2; nud-2; let-2; imb-1; vha-4; col-20; sca-1; vps-32.1; arf-1.2; vps-26; sec-24.2; y19d10a.12; clh-1; tat-5; y19d10a.4; vit-1; vha-16; c01b4.7; vha-2; dab-1; chc-1; glt-1; emb-9; imb-3; apb-1; ima-3; vha-15; vha-12; vha-11; unc-116; c13b9.3; vha-13; vit-3; snap-1; k08f4.2; ced-7; col-178; npp-8; sec-24.1; col-106; f21d5.7; eat-6; aqp-8; pmp-5; c01b4.9; r05f9.1; t14g10.5; y71f9al.17; npp-10; ent-2; vha-5; ran-5; aex-5; aqp-2; ran-4; vit-4	6.36E-02	transport;
GO:0055080	mca-3; sca-1; vha-12	6.36E-02	cation homeostasis;
GO:0051729	cgh-1; atx-2; ccf-1	6.36E-02	germline cell cycle switching, mitotic to meiotic cell cycle;
GO:0043068	vha-2; unc-32; vha-10; vha-12	6.36E-02	positive regulation of programmed cell death;
GO:0006950	lea-1; daf-16; cdc-14; skn-1; pme-1; b0495.2; daf-18; lig-1; smk-1; set-25; hsp-16.11; unc-32; hif-1; vha-12; hda-1; rfc-1; sca-1; hsp-16.1	6.36E-02	response to stress;
GO:0031344	let-60; ptp-2	6.36E-02	regulation of cell projection organization and biogenesis;
GO:0006997	npp-8; ran-4; ima-3; lmn-1; npp-7	6.36E-02	nuclear organization and biogenesis;
GO:0009949	par-1; nmy-2	6.36E-02	polarity specification of anterior/posterior axis;
GO:0046356	w02f12.5; f35g12.2; aco-2	6.36E-02	acetyl-CoA catabolic process;
GO:0007173	ptp-2; unc-52	6.36E-02	epidermal growth factor receptor signaling pathway;
GO:0014866	unc-54; unc-60	6.36E-02	skeletal myofibril assembly;
GO:0006099	w02f12.5; f35g12.2; aco-2	6.36E-02	tricarboxylic acid cycle; acetyl-CoA catabolic process; cellular respiration; aerobic respiration; coenzyme catabolic process; cofactor catabolic process;
GO:0009880	zyg-11; par-1; skn-1; mbk-2	6.36E-02	embryonic pattern specification;
GO:0045045	sec-24.1; aex-5; f21d5.7; sec-24.2; snb-1; sec-23	7.11E-02	secretory pathway;
GO:0018992	nos-3; atx-2; puf-8; gld-3	7.29E-02	germ-line sex determination;
GO:0006998	ran-4; lmn-1; npp-7	7.66E-02	nuclear membrane organization and biogenesis;
GO:0045333	w02f12.5; f35g12.2; aco-2	7.66E-02	cellular respiration;
GO:0009060	w02f12.5; f35g12.2; aco-2	7.66E-02	aerobic respiration;

GO:0040024	dao-5; daf-16; lgg-1; daf-18; sca-1; akt-1	7.66E-02	dauer larval development;
GO:0009058	r151.2; t04a8.7; r11a5.4; cdc-48.2; f53f10.2; puf-8; vha-16; vha-2; c37h5.6; t12a2.2; eif-3.c; atp-2; vha-15; vha-8; srs-2; c32f10.8; vha-12; vha-11; gpi-1; lrs-1; grs-1; t05h4.6a; c36b1.7; r03d7.1; iftb-1; vha-13; vrs-2; vha-14; fat-7; lin-41; let-721; c37c3.2; mrs-1; t09a5.11; f42d1.2; eif-3.b; irs-1; pyc-1; sptl-3; tufm-1; trs-1; vha-4; crs-1	8.15E-02	biosynthetic process;
GO:0008105	rab-5; par-1; mlc-4; mbk-2	8.20E-02	asymmetric protein localization;
GO:0006605	f21d5.7; imb-3; imb-1; daf-18; ima-3	8.22E-02	protein targeting;
GO:0006098	b0250.5; t25b9.9	8.64E-02	NADPH regeneration%pentose-phosphate shunt;
GO:0009066	c06a8.1; r03d7.1	8.64E-02	aspartate family amino acid metabolic process;
GO:0008360	nmy-2; nmy-1	8.64E-02	regulation of cell shape;
GO:0006740	b0250.5; t25b9.9	8.64E-02	NADP metabolic process%NADPH regeneration; %pentose-phosphate shunt;
GO:0015914	tat-5; tat-4	8.64E-02	phospholipid transport;
GO:0009109	w02f12.5; f35g12.2; aco-2	8.90E-02	coenzyme catabolic process;
GO:0051187	w02f12.5; f35g12.2; aco-2	8.90E-02	cofactor catabolic process;
GO:0000819	zyg-11; him-1; scc-3	8.90E-02	sister chromatid segregation;
GO:0006066	zk836.2; b0250.5; pyc-1; f14b4.2; r11a5.4; y110a7a.6; t25b9.9; gpi-1; ldh-1; t22b11.5	9.65E-02	alcohol metabolic process;
