# Supplementary material for: Gene expression profiling of oxidative stress response of C. elegans aging defective AMPK mutants using massively parallel transcriptome sequencing
Source: BMC Res Notes. 2011 Feb 8;4:34. doi: 10.1186/1756-0500-4-34 (PMC3045954; doi:10.1186/1756-0500-4-34)
Supplement: Additional file 13 — Supplementary Table S12. Significantly down-regulated genes in unstressed aak-2 relative to wild type [file 1756-0500-4-34-S13.PDF]

Supplementary Table 12. Significantly down-regulated genes in unstressed aak-2 relative to wild type

Gene	Log2 (Unstressed aak-2/Unstressed N2)	p-val
F15E11.15	-7.19	1.20E-45
F15E11.13	-6.81	3.03E-246
Y19D10B.7	-6.81	4.20E-196
F15E11.12	-6.38	7.42E-26
F15E11.14	-6.06	5.81E-212
F15E11.1	-5.99	1.21E-213
Y105C5A.13	-5.45	4.37E-14
C08E3.13	-4.83	7.48E-15
C08E3.1	-4.77	5.26E-13
Y105C5A.12	-4.57	1.06E-07
T24B8.5	-3.82	3.86E-54
F45D11.14	-3.61	5.07E-04
F45D11.15	-3.53	8.08E-04
F45D11.16	-3.53	8.08E-04
C02E7.7	-3.24	1.05E-10
Y39G8B.9	-3.21	3.70E-06
M02H5.8	-3.19	1.77E-06
Y48G8AL.12	-2.97	2.79E-08
D1086.3	-2.96	8.69E-03
his-5	-2.91	3.63E-04
ssp-19	-2.82	4.38E-03
ins-27	-2.72	6.80E-06
C31C9.7	-2.60	4.99E-03
nhr-37	-2.59	3.27E-04
F01D5.1	-2.55	3.97E-05
R102.2	-2.55	2.65E-05
Y41E3.5	-2.53	5.68E-03
F53C11.2	-2.47	4.41E-05
W08D2.9	-2.47	2.92E-26
msp-74	-2.46	5.13E-10
rps-27	-2.42	3.34E-260
F23D12.1	-2.41	5.40E-06
K07F5.9	-2.41	2.22E-07
Y119D3B.21	-2.39	2.28E-93
C50D2.6	-2.38	7.09E-03
F53A9.1	-2.38	4.89E-05
F56H9.2	-2.34	7.82E-35
srz-70	-2.34	1.56E-11
clec-47	-2.32	9.66E-03
R12E2.14	-2.32	4.14E-05
spp-23	-2.31	3.61E-18
T19C3.3	-2.31	2.98E-03

Y68A4A.13	-2.31	2.30E-03
his-11	-2.28	2.18E-03
his-18	-2.26	1.14E-04
F29B9.11	-2.23	3.22E-61
msp-63	-2.23	1.43E-05
msp-142	-2.22	3.15E-22
spp-14	-2.22	2.06E-56
msp-37	-2.21	3.93E-12
spp-17	-2.21	5.76E-63
BE10.6	-2.20	2.78E-08
acbp-1	-2.19	2.13E-46
ddp-1	-2.18	9.22E-22
msp-76	-2.18	1.93E-33
rpl-34	-2.17	3.16E-156
rps-21	-2.14	1.66E-154
rps-28	-2.13	1.12E-285
rpl-37	-2.11	2.61E-177
F20A1.1	-2.09	5.40E-05
F35B12.7	-2.09	1.60E-10
F43G6.7	-2.09	5.78E-04
nspc-14	-2.08	3.28E-04
K10D2.4	-2.07	2.46E-07
msp-42	-2.07	1.30E-06
msp-64	-2.04	4.21E-17
ZK1248.4	-2.03	3.89E-19
C37A2.7	-2.02	3.54E-137
F23A7.8	-2.00	3.71E-69
F14H3.12	-1.99	8.30E-03
fipr-1	-1.99	2.50E-05
rpl-41	-1.99	1.25E-290
W01D2.1	-1.99	6.84E-207
msp-81	-1.98	1.30E-32
B0495.6	-1.97	1.59E-10
rpl-36	-1.96	3.51E-199
Y47D3A.20	-1.95	6.75E-04
C35D10.11	-1.93	2.98E-06
R12E2.15	-1.93	3.81E-08
F44D12.3	-1.92	3.96E-06
rpl-43	-1.92	5.01E-108
spp-4	-1.91	2.19E-11
spp-5	-1.90	8.53E-90
F44E5.1	-1.89	8.42E-39
F57B10.14	-1.89	5.42E-32
msp-77	-1.89	2.58E-27
F43C11.3	-1.87	3.48E-03
F23H11.5	-1.86	7.04E-33
rpl-33	-1.86	3.78E-159

W02D9.7	-1.85	2.33E-12
C53H9.3	-1.84	9.05E-07
C14B9.10	-1.83	1.71E-18
dao-2	-1.83	4.12E-04
msp-56	-1.83	2.24E-29
rpl-38	-1.83	7.86E-137
F53A9.8	-1.82	4.09E-04
msp-55	-1.80	1.46E-27
nspc-1	-1.80	1.70E-05
rpb-10	-1.80	3.90E-09
ubq-2	-1.80	2.38E-156
F21C10.11	-1.79	1.00E-02
K10B3.1	-1.79	6.21E-03
T02B11.3	-1.79	9.93E-03
msp-50	-1.78	8.43E-09
tomm-7	-1.78	3.18E-11
iff-1	-1.77	5.41E-73
msp-10	-1.77	1.82E-27
msp-51	-1.77	6.68E-31
Y105E8A.11	-1.77	1.04E-04
F35H10.5	-1.76	6.96E-05
msp-36	-1.76	4.41E-18
rpb-12	-1.76	4.81E-11
F48D6.4	-1.75	1.02E-09
fipr-4	-1.75	7.36E-04
msp-19	-1.74	4.63E-30
msp-3	-1.74	1.96E-10
T07A5.5	-1.74	1.51E-06
Y17D7B.7	-1.74	1.21E-03
msp-65	-1.73	3.38E-24
Y43C5A.1	-1.73	6.32E-04
cpg-9	-1.72	9.58E-22
msp-49	-1.71	2.35E-06
C14C11.7	-1.70	3.68E-05
F26E4.6	-1.70	8.66E-32
fipr-7	-1.70	4.05E-03
K11H3.6	-1.70	2.52E-10
T23F2.3	-1.70	1.69E-03
C01F6.9	-1.69	1.18E-13
F53A3.3	-1.69	1.82E-84
rpl-30	-1.69	5.20E-140
Y59A8B.12	-1.69	3.21E-06
nspc-13	-1.68	5.66E-03
gut-2	-1.67	3.76E-09
msp-59	-1.67	5.58E-26
nspc-7	-1.67	6.84E-06
F18A11.3	-1.66	2.38E-03

nlp-36	-1.66	2.17E-08
rpl-25.1	-1.66	4.55E-24
msp-79	-1.65	7.28E-23
rpb-11	-1.65	7.24E-08
spp-3	-1.65	2.18E-30
F43G6.10	-1.64	5.46E-03
B0205.12	-1.63	6.10E-05
F41F3.3	-1.63	5.10E-07
Y59H11AM.1	-1.63	6.23E-07
msp-40	-1.62	1.61E-09
T27A1.1	-1.62	1.69E-34
tin-9.1	-1.62	1.12E-03
C04G2.3	-1.61	1.09E-10
C49F5.7.2	-1.61	2.01E-05
msp-57	-1.61	4.97E-20
W02D9.5	-1.61	9.71E-03
ZK546.3	-1.61	5.79E-07
C17H12.8	-1.60	1.15E-03
C49F5.7.1	-1.60	2.71E-05
F23D12.7	-1.59	1.72E-07
msp-53	-1.59	1.88E-23
K01H12.1	-1.58	5.89E-05
nduf-5	-1.58	4.45E-16
rpl-25.2	-1.58	1.06E-110
rps-24	-1.58	5.57E-158
Y63D3A.7	-1.58	9.87E-06
rpl-26	-1.57	3.76E-105
rpl-28	-1.57	1.55E-66
rpl-32	-1.57	1.41E-88
cyc-2.1	-1.56	4.35E-44
F15D4.3	-1.56	1.09E-05
F29C4.2	-1.56	8.49E-11
rpl-35	-1.56	3.18E-76
Y69A2AR.3	-1.56	1.50E-06
ZK512.4	-1.56	1.59E-04
rps-25	-1.55	5.58E-102
msp-58	-1.54	2.60E-18
msp-78	-1.54	5.14E-17
pcbd-1	-1.54	6.56E-03
rpl-14	-1.54	3.69E-66
Y22D7AR.10	-1.54	1.47E-18
Y53F4B.14	-1.54	1.71E-03
C45B2.1	-1.53	2.96E-10
his-68	-1.53	3.38E-04
msp-33	-1.53	1.77E-12
F33G12.7	-1.51	1.69E-04
T20D3.6	-1.51	9.47E-03

W02D9.6	-1.50	2.97E-04
C07H6.2	-1.49	9.15E-05
rps-29	-1.49	2.72E-41
msp-38	-1.48	1.59E-10
pqn-94	-1.47	5.10E-03
B0035.18	-1.46	4.22E-03
msp-31	-1.46	2.32E-13
mtl-2	-1.45	1.16E-04
F45H10.2	-1.44	1.88E-11
lec-9	-1.44	1.02E-09
lsm-5	-1.44	3.64E-03
R186.8	-1.44	2.70E-04
rps-30	-1.44	8.15E-68
C48B6.10	-1.43	2.00E-08
F13G3.10	-1.43	1.25E-04
K12H4.5	-1.43	2.08E-08
kbp-4	-1.42	1.96E-06
lsm-6	-1.42	1.83E-05
rps-26	-1.42	1.45E-71
ubl-5	-1.42	3.70E-05
C46G7.1	-1.41	5.05E-05
msp-152	-1.41	3.39E-12
R09B3.2	-1.41	1.01E-30
rps-11	-1.41	7.94E-119
snr-5	-1.41	1.09E-07
Y37E3.8	-1.41	9.50E-90
C18E9.4	-1.40	3.43E-08
ttr-41	-1.40	2.73E-06
K10B2.4	-1.39	1.54E-05
lys-2	-1.39	1.28E-04
T26H5.9	-1.39	1.42E-03
msp-52	-1.38	1.97E-18
msp-71	-1.37	1.73E-08
R01B10.3	-1.37	3.86E-03
rpl-22	-1.37	9.60E-50
Y59E9AR.1	-1.37	2.68E-15
F25H5.6	-1.36	9.35E-03
his-47	-1.36	1.96E-03
F53F4.16	-1.35	5.61E-05
nspc-10	-1.35	9.93E-03
Y39A3CL.3	-1.35	2.26E-04
Y59E9AR.7	-1.35	1.65E-15
dod-23	-1.34	8.28E-06
rps-17	-1.34	2.53E-62
lgg-3	-1.33	1.76E-04
snr-7	-1.33	5.92E-05
Y60A3A.21	-1.33	8.03E-03

rpl-23	-1.32	1.97E-42
Y39B6A.5	-1.32	4.43E-03
F29B9.10	-1.31	5.58E-04
F43G6.2	-1.31	4.16E-03
Y69A2AR.28	-1.31	1.02E-03
C08F8.9	-1.30	7.23E-06
rla-2	-1.30	1.71E-21
Y111B2A.2	-1.30	1.17E-05
snr-6	-1.29	2.25E-11
Y71H2AM.5	-1.29	3.47E-14
mai-2	-1.28	4.76E-10
nspa-5	-1.28	1.94E-04
Y82E9BR.3	-1.28	1.69E-46
nlp-33	-1.27	5.30E-03
phf-5	-1.27	8.34E-03
rpl-27	-1.27	6.41E-63
Y18D10A.16	-1.27	6.11E-03
emo-1	-1.26	7.71E-16
fipr-21	-1.26	4.09E-04
lec-6	-1.26	2.23E-12
mxl-1	-1.26	2.19E-04
nspc-12	-1.26	7.34E-03
Y110A2AM.4	-1.26	4.60E-03
C29E4.12	-1.25	1.18E-03
nspc-3	-1.25	5.51E-03
T20G5.8	-1.25	1.89E-03
C35D10.17	-1.24	6.93E-03
F42H10.2	-1.24	4.48E-03
F44E2.9	-1.24	3.95E-03
MTCE.4	-1.24	1.78E-91
rps-23	-1.24	4.36E-65
C17E4.4	-1.23	4.75E-03
Y38F2AR.9	-1.23	2.11E-11
oig-2	-1.22	1.62E-04
T23F2.5	-1.22	1.22E-06
ttr-16	-1.22	1.02E-09
cuc-1	-1.21	3.07E-05
F55B11.4	-1.21	4.35E-03
rpl-39	-1.21	2.97E-75
ZK686.1	-1.20	3.20E-07
elb-1	-1.19	2.01E-03
lbp-6	-1.19	5.40E-13
rps-14	-1.19	3.61E-53
dpm-3	-1.18	8.70E-04
F23F1.10	-1.18	9.40E-04
glb-1	-1.18	8.42E-03
ned-8	-1.18	1.18E-05

Y55B1AL.2	-1.18	3.43E-05
F36A2.8	-1.17	6.50E-03
rps-16	-1.16	4.09E-25
T02D1.8	-1.15	1.73E-03
his-48	-1.14	1.15E-03
his-58	-1.14	8.25E-04
rpl-31	-1.14	2.03E-48
Y105C5B.5	-1.14	1.31E-03
Y48G1C.9	-1.14	7.94E-03
rps-12	-1.12	2.52E-50
dyrb-1	-1.11	1.49E-04
T27E9.2	-1.11	2.00E-04
Y44E3A.3	-1.11	7.93E-03
F42A9.8	-1.10	6.02E-03
F54C9.3	-1.10	3.22E-03
rps-7	-1.10	4.16E-74
Y56A3A.19	-1.09	3.87E-05
gst-27	-1.07	3.45E-04
ilys-5	-1.07	2.15E-21
Y67H2A.5	-1.07	1.40E-12
Y97E10AL.3	-1.07	5.70E-03
zig-7	-1.06	9.15E-16
D2030.4	-1.05	6.73E-04
ttr-24	-1.04	3.39E-04
W03G9.8	-1.04	3.82E-03
glrx-21	-1.03	5.82E-03
rla-1	-1.03	9.91E-41
rpb-6	-1.03	8.90E-05
rps-18	-1.03	7.65E-39
ZK121.1b	-1.03	5.82E-03
nlp-27	-1.01	3.32E-04
F22D6.14	-1.00	7.20E-03
F54D5.4	-0.99	2.87E-04
lsm-3	-0.99	1.85E-03
Y71H2B.4	-0.99	5.08E-03
C35B1.4	-0.98	4.81E-06
rab-18	-0.98	1.36E-03
R07E4.3	-0.97	7.59E-03
C33A12.1	-0.96	1.08E-03
col-147	-0.96	7.78E-04
K10C2.3	-0.96	1.47E-03
ttr-48	-0.96	4.35E-03
cpg-8	-0.95	2.45E-04
K01G5.8	-0.95	4.13E-03
T24H7.4	-0.95	7.61E-03
asg-1	-0.94	1.42E-05
rps-19	-0.94	7.03E-31

tin-13	-0.94	1.55E-04
Y71H2AM.4	-0.94	4.73E-06
C17E7.12	-0.93	9.35E-03
F44E7.9	-0.93	2.87E-03
T14B4.2	-0.93	3.93E-03
atp-4	-0.92	1.24E-07
R04F11.2	-0.91	4.47E-06
rpl-11.1	-0.91	2.79E-23
R07E5.13	-0.90	1.10E-03
rps-13	-0.90	3.55E-23
tag-174	-0.90	4.07E-06
W04C9.2	-0.90	1.63E-03
F45H10.3	-0.88	6.84E-04
nlt-1	-0.87	6.77E-04
Y45F10C.4	-0.87	6.71E-05
F46F2.3	-0.86	4.54E-05
C28C12.2	-0.85	7.04E-05
col-117	-0.85	2.70E-08
col-3	-0.85	3.88E-08
nduf-6	-0.85	3.92E-03
F25H2.4	-0.84	4.28E-03
K07F5.15	-0.84	8.08E-03
lys-1	-0.83	1.04E-04
rps-15	-0.83	7.33E-21
rpl-24.1	-0.81	4.29E-31
F10E9.11	-0.80	3.81E-03
gmn-1	-0.80	7.24E-03
pfd-4	-0.79	5.23E-03
clec-85	-0.78	8.44E-03
lsm-8	-0.78	2.49E-03
MTCE.3	-0.78	7.04E-09
rps-10	-0.77	1.21E-20
pfd-6	-0.76	9.69E-04
tag-277	-0.76	6.43E-03
col-146	-0.75	4.06E-03
snr-3	-0.75	1.22E-03
Y51F10.7	-0.75	2.01E-03
ZK813.2	-0.75	3.50E-06
ced-10	-0.74	6.84E-03
F53F1.4	-0.74	2.21E-04
Y57E12AL.6	-0.73	8.94E-04
ttr-45	-0.72	2.45E-03
Y37D8A.19	-0.72	2.42E-06
rps-20	-0.71	2.07E-11
