# Supplementary material for: Gene expression profiling of oxidative stress response of C. elegans aging defective AMPK mutants using massively parallel transcriptome sequencing
Source: BMC Res Notes. 2011 Feb 8;4:34. doi: 10.1186/1756-0500-4-34 (PMC3045954; doi:10.1186/1756-0500-4-34)
Supplement: Additional file 14 — Supplementary Table S13. Significantly down-regulated genes in unstressed aak-2 relative to wild type and most highly represented biological processes these genes are involved in [file 1756-0500-4-34-S14.PDF]

Supplementary Table 13. Significantly down-regulated genes in unstressed aak-2 relative to wild type and most highly represented biological processes these genes are involved in

GO	Genes	Pvalue	GO as name
GO:0044249	y82e9br.3; rpl-33; rpl-38; zk512.4; rps-29; rpl-34; rps-19; rpl-25.2; rps-14; atp-4; w01d2.1; rpl-41; rpl-31; y37e3.8; f29b9.10; rps-12; rpl-22; rpl-43; rps-7; rla-1; rpl-37; rpl-14; rps-23; rps-22; rpl-28; rpl-25.1; rps-27; rps-26; rpl-32; rla-2; c37a2.7; rpl-39; rps-30; rps-16; rpl-27; rps-24; rpl-36; k11h3.6; y56a3a.19; rpl-26; iff-1; rps-21; rpl-30; rps-11; ubq-2; rpl-23; rpl-35; rps-17; rps-28; rps-18	3.07E-55	cellular biosynthetic process;
GO:0006412	rpl-33; rpl-38; zk512.4; rps-29; rpl-34; rps-19; rpl-25.2; rps-14; w01d2.1; rpl-41; rpl-31; y37e3.8; f29b9.10; rps-12; rpl-22; rpl-43; rps-7; rla-1; rpl-37; rpl-14; rps-23; rps-22; rpl-28; rpl-25.1; rps-27; rps-26; rpl-32; rla-2; c37a2.7; rpl-39; rps-30; rps-16; rpl-27; rps-24; rpl-36; k11h3.6; rpl-26; iff-1; rps-21; rpl-30; rps-11; ubq-2; rpl-23; rpl-35; rps-17; rps-28; rps-18	4.99E-38	translation; macromolecule biosynthetic process;
GO:0010467	rpl-33; rpl-38; zk512.4; rps-29; rpl-34; rps-19; rpl-25.2; rps-14; w01d2.1; rpl-41; rpl-31; y37e3.8; f29b9.10; rps-12; rpl-22; rpl-43; rps-7; rla-1; rpl-37; his-5; rpl-14; rpb-10; rps-23; rps-22; rpl-28; rpl-25.1; rps-27; rps-26; rpl-32; his-18; c37a2.7; rpb-6; rla-2; rpl-39; rpb-11; rps-30; rpb-12; rpl-27; rps-16; mxl-1; rps-24; rpl-36; k11h3.6; rpl-26; iff-1; rps-21; rpl-30; rps-11; ubq-2; rpl-23; rpl-35; rps-17; nhr-37; rps-28; rps-18	2.99E-20	gene expression;
GO:0009792	rpl-33; his-68; rps-19; lsm-5; rps-25; atp-4; rps-14; k01h12.1; f23h11.5; c17e4.4; k10d2.4; rps-12; rpl-22; rps-7; his-5; rpl-37; rpl-14; his-47; y47d3a.20; snr-5; y67h2a.5; rps-22; rps-27; rpb-11; rpl-39; msp-31; rps-16; rpl-27; rpl-36; r186.8; snr-7; snr-6; rps-21; rpl-30; emo-1; c04g2.3; rpl-23; cyc-2.1; c14b9.10; rps-17; d2030.4; f26e4.6; y82e9br.3; kbp-4; f57b10.14; rpl-34; rps-29; f29b9.11; rpl-25.2; f29c4.2; w01d2.1; rpl-41; rpl-31; y37e3.8; f29b9.10; rpl-43; rla-1; elb-1; rpb-10; c18e9.4; ilys-5; b0495.6; rpl-28; lec-9; rpl-25.1; rps-26; y63d3a.7; his-18; rla-2; c37a2.7; rpb-6; c29e4.12; his-48; rpb-12; k12h4.5; gut-2; his-11; lsm-6; phf-5; rpl-26; y71h2am.5; iff-1; tin-9.1; rps-11; ubq-2; dyrb-1; rpl-35; his-58; msp-152	3.31E-18	embryonic development ending in birth or egg hatching;
GO:0040010	f26e4.6; rpl-33; ubl-5; rpl-38; rps-29; rpl-34; y18d10a.16; f29b9.11; rpl-25.2; rps-25; rps-14; atp-4; f29c4.2; w01d2.1; f23h11.5; f29b9.10; f25h5.6; f44e5.1; rpl-22; rpl-43; c46g7.1; zk686.1; rpl-37; his-5; rpl-14; his-47; c18e9.4; rps-22; rps-27; rpl-25.1; his-18; c37a2.7; rla-2; rpb-11; rpl-39; fipr-1; rps-30; his-48; k12h4.5; t27e9.2; c07h6.2; gut-2; f45h10.2; k11h3.6; lsm-6; y56a3a.19; rpl-26; snr-7; y71h2am.5; ned-8; c01f6.9; cyc-2.1; rps-17; his-58; rps-28; d2030.4	4.16E-17	positive regulation of growth rate;

GO:0007275	rpl-33; ubl-5; dod-23; f35h10.5; his-68; rps-19; lsm-5; rps-25; rps-14; atp-4; k01h12.1; f23h11.5; c17e4.4; k10d2.4; rps-12; rpl-22; rps-7; rpl-37; his-5; rpl-14; his-47; y47d3a.20; snr-5; y67h2a.5; rps-22; rpl-32; rps-27; spp-5; mtl-2; fipr-1; rpb-11; rpl-39; msp-31; rpl-27; rps-16; rpl-36; r186.8; snr-7; rps-21; snr-6; rpl-30; emo-1; ned-8; c04g2.3; rpl-23; cyc-2.1; c14b9.10; rps-17; d2030.4; f26e4.6; y82e9br.3; kbp-4; f57b10.14; rpl-34; rps-29; f29b9.11; rpl-25.2; f29c4.2; w01d2.1; rpl-41; rpl-31; y37e3.8; f25h5.6; f29b9.10; rpl-43; c46g7.1; rla-1; zk686.1; elb-1; rpb-10; rps-23; ilys-5; c18e9.4; b0495.6; lec-9; rpl-28; rpl-25.1; rps-26; y63d3a.7; his-18; rla-2; c37a2.7; rpb-6; c29e4.12; rpb-12; rps-30; his-48; k12h4.5; gut-2; rps-24; his-11; lsm-6; phf-5; y56a3a.19; rpl-26; y71h2am.5; iff-1; tin-9.1; t20g5.8; rps-11; ubq-2; dyrb-1; rpl-35; his-58; msp-152	4.56E-17	multicellular organismal development;
GO:0044267	f53a9.1; rpl-33; ubl-5; rpl-38; zk512.4; rps-29; rpl-34; rps-19; rpl-25.2; rps-14; w01d2.1; rpl-41; rpl-31; y37e3.8; f14h3.12; f29b9.10; rps-12; rpl-22; rpl-43; rps-7; rla-1; rpl-37; rpl-14; rps-23; rps-22; rpl-28; rpl-25.1; rps-27; rps-26; rpl-32; rla-2; c37a2.7; rpl-39; rps-30; rpl-27; rps-16; rps-24; rpl-36; k11h3.6; rpl-26; iff-1; rps-21; rpl-30; rps-11; ned-8; ubq-2; rpl-23; rpl-35; rps-17; rps-28; rps-18	5.08E-09	cellular protein metabolic process;
GO:0016071	snr-7; gut-2; snr-6; lsm-5; lsm-6; snr-5; y48g1c.9	1.42E-04	mRNA metabolic process;
GO:0006414	c37a2.7; rla-2; rla-1; zk512.4	3.83E-04	translational elongation; biopolymer biosynthetic process;
GO:0006334	his-5; his-68; his-18; his-47; his-11; his-58; his-48	2.06E-03	nucleosome assembly; chromatin assembly; chromatin assembly or disassembly;
GO:0006605	emo-1; tin-9.1; zk512.4; ddp-1	1.44E-02	protein targeting;
GO:0007007	tin-9.1; ddp-1	1.44E-02	inner mitochondrial membrane organization and biogenesis; protein import into mitochondrial inner membrane;
GO:0019953	rps-27; y63d3a.7; rpl-39; rpb-11; rpl-33; ubl-5; rpl-38; gut-2; f45h10.2; rpl-25.2; rpl-31; rpl-26; y71h2am.5; iff-1; emo-1; ned-8; zk686.1; ssp-10; dyrb-1; rps-22	4.21E-02	sexual reproduction; gamete generation;
GO:0015677	cuc-1	7.66E-02	copper ion import;
GO:0016236	lgg-3	7.66E-02	macroautophagy; autophagic vacuole formation;
GO:0006119	mtce.4; t27e9.2; y82e9br.3; atp-4	8.65E-02	oxidative phosphorylation;
GO:0006091	mtce.4; f26e4.6; y82e9br.3; y71h2am.5; t27e9.2; f45h10.2; cyc-2.1; b0035.18; atp-4; f29c4.2; d2030.4	8.97E-02	generation of precursor metabolites and energy;
