# Supplementary material for: Gene expression profiling of oxidative stress response of C. elegans aging defective AMPK mutants using massively parallel transcriptome sequencing
Source: BMC Res Notes. 2011 Feb 8;4:34. doi: 10.1186/1756-0500-4-34 (PMC3045954; doi:10.1186/1756-0500-4-34)
Supplement: Additional file 15 — Supplementary Table S14. Commonly up-regulated genes in unstressed aak-2, stressed aak-2, and stressed wild type and most highly represented biological processes these genes are involved in [file 1756-0500-4-34-S15.PDF]

Supplementary Table 14. Commonly up-regulated genes in unstressed aak-2, stressed aak-2, and stressed wild type and most highly represented biological processes these genes are involved in

GO	Genes	Pvalue	GO as name
GO:0006119	vha-11; mtce.35; vha-2; vha-4; vha-8; vha-15	4.50E-05	oxidative phosphorylation;
GO:0007276	hrp-2; zc247.1; iftb-1; erm-1; arf-1.2; ppn-1; hsp-6; kin-2; vha-8; glh-1; vha-11; y66h1b.2; eif-3.f; vha-4; fib-1	6.67E-05	gamete generation;
GO:0015985	vha-11; vha-2; vha-4; vha-8; vha-15	6.67E-05	ATP biosynthetic process;
GO:0008340	ppn-1; acdh-1; hsp-16.11; cpr-1; ifb-1; cct-5; rab-1; hsp-16.1	9.40E-05	multicellular organismal aging#determination of adult life span;
GO:0006732	vha-11; vha-2; vha-4; c44b7.10; vha-8; vha-15	1.72E-04	coenzyme metabolic process;
GO:0016043	iftb-1; erm-1; arf-1.2; rab-5; mlc-4; unc-54; arx-6; taf-13; aex-5; t05h4.6a; ran-4; dnj-12; rab-11.1; fib-1; rab-1	2.95E-04	cellular component organization and biogenesis;
GO:0018996	ppn-1; ifb-1; apl-1; vha-15	8.45E-04	molting cycle, collagen and cuticulin-based cuticle;
GO:0007626	cpl-1; erm-1; arf-1.2; ppn-1; kin-2; cct-5; apl-1; let-2; unc-15; t05h4.6a; ran-4; vha-4; ifb-1; k08d12.3	1.48E-03	locomotory behavior;
GO:0050896	erm-1; arf-1.2; ppn-1; acdh-1; cct-5; apl-1; unc-15; unc-54; k08d12.3; cpl-1; hsp-16.11; kin-2; let-2; t05h4.6a; ran-4; ifb-1; vha-4; hsp-16.1	2.37E-03	response to stimulus;
GO:0006091	mtce.35; acdh-1; vha-8; vha-15; vha-11; vha-2; vha-4; llc1.3	3.14E-03	generation of precursor metabolites and energy;
GO:0006457	hsp-6; dnj-12; cct-5; hsp-60	3.82E-03	protein folding;
GO:0043071	vha-2; vha-10	3.82E-03	positive regulation of non-apoptotic programmed cell death;
GO:0040006	ppn-1; ifb-1; vha-15	7.51E-03	molting cycle, protein-based cuticle#protein-based cuticle attachment to epithelium;
GO:0048468	hrp-2; glh-1; unc-54; rab-5; vha-2; vha-10	1.07E-02	cell differentiation#cell development;
GO:0051169	rab-5; rab-11.1; rab-1	1.18E-02	nuclear transport;
GO:0009607	hsp-16.11; hsp-16.1	1.28E-02	response to biotic stimulus; ER-nuclear signaling pathway;
GO:0007242	unc-54; arf-1.2; rab-5; hsp-16.11; rab-11.1; rab-1; hsp-16.1	1.67E-02	intracellular signaling cascade;
GO:0044249	iftb-1; vha-8; vha-15; vha-11; t05h4.6a; vha-2; c36b1.7; vha-4	1.79E-02	cellular biosynthetic process;
GO:0006545	c36b1.7	1.81E-02	glycine biosynthetic process;
GO:0007110	mlc-4	1.81E-02	cytokinesis after meiosis I; establishment of cell polarity;cytokinesis during cell cycle;
GO:0008105	rab-5; mlc-4	2.49E-02	asymmetric protein localization;
GO:0008104	arf-1.2; rab-5; mlc-4; rab-11.1; rab-1	2.84E-02	protein localization;
GO:0035148	erm-1	2.93E-02	multicellular organismal development#tube development#tube morphogenesis#lumen formation;
GO:0043241	t05h4.6a	2.93E-02	protein complex disassembly; translational termination;
GO:0048017	unc-54	2.93E-02	inositol lipid-mediated signaling;

GO:0031033	unc-54	2.93E-02	myosin filament assembly or disassembly;
GO:0007264	arf-1.2; rab-5; rab-11.1; rab-1	3.21E-02	small GTPase mediated signal transduction; intracellular protein transport;
GO:0042395	apl-1	5.48E-02	molting cycle, collagen and cuticulin-based cuticle#ecdysis, collagen and cuticulin-based cuticle;
GO:0007010	unc-54; erm-1; arx-6; dnj-12	6.18E-02	cytoskeleton organization and biogenesis;
GO:0006952	acdh-1; hsp-16.11	7.16E-02	defense response;
GO:0051649	aex-5; arf-1.2; rab-5; rab-11.1; rab-1	7.70E-02	establishment of cellular localization;
GO:0008219	rab-5; vha-2; vha-10	7.70E-02	cell death;
GO:0051301	rab-5; mlc-4; dnj-12; rab-11.1	7.72E-02	cell division;
GO:0007281	hrp-2; glh-1	7.91E-02	gamete generation#germ cell development;
GO:0006914	lgg-1	7.91E-02	autophagy;
GO:0032940	aex-5; rab-5	8.10E-02	secretion by cell;
GO:0008154	arx-6	8.77E-02	actin polymerization and/or depolymerization;
GO:0009306	rab-5	9.70E-02	protein secretion;
GO:0022414	hrp-2; iftb-1; arf-1.2; ppn-1; cct-5; unc-54; unc-15; eif-3.f; ran-4	1.00E-01	reproductive process;
