# Supplementary material for: Gene expression profiling of oxidative stress response of C. elegans aging defective AMPK mutants using massively parallel transcriptome sequencing
Source: BMC Res Notes. 2011 Feb 8;4:34. doi: 10.1186/1756-0500-4-34 (PMC3045954; doi:10.1186/1756-0500-4-34)
Supplement: Additional file 16 — Supplementary Table S15. Commonly down-regulated genes in unstressed aak-2, stressed aak-2, and stressed wild type and most highly represented biological processes these genes are involved in [file 1756-0500-4-34-S16.PDF]

Supplementary Table 15. Commonly down-regulated genes in unstressed aak-2, stressed aak-2, and stressed wild type and most highly represented biological processes these genes are involved in

GO	Genes	Pvalue	GO as name
GO:0010467	rpl-25.1; his-18; c37a2.7; rpb-11; rpl-33; rpl-38; rps-30; rpb-12; rpl-34; mxl-1; t14b4.2; rps-24; k11h3.6; rpl-36; rab-18; y37e3.8; rpl-41; w01d2.1; rpl-26; rps-12; iff-1; rps-21; rpl-22; rpl-43; rps-11; his-5; rpl-35; rpb-10; nhr-37; rps-28; rps-22	2.31E-22	gene expression;
GO:0006412	rpl-25.1; c37a2.7; rpl-33; rpl-38; rps-30; rpl-34; t14b4.2; rps-24; k11h3.6; rpl-36; y37e3.8; rpl-41; w01d2.1; rpl-26; rps-12; iff-1; rps-21; rpl-22; rpl-43; rps-11; rpl-35; rps-28; rps-22	3.42E-21	translation;
GO:0006333	his-5; his-68; his-18; his-47; his-58; his-48	1.85E-04	chromatin assembly or disassembly;
GO:0007276	rpl-26; y63d3a.7; iff-1; rpb-11; rpl-33; pfd-6; rpl-38; zk686.1; gut-2; t14b4.2; rps-22	6.48E-03	gamete generation;
GO:0016043	his-18; iff-1; pfd-6; his-48; his-5; his-68; tomm-7; dyrb-1; his-47; his-58; rab-18; ddp-1	1.15E-02	cellular component organization and biogenesis;
GO:0006139	y82e9br.3; his-18; rpb-11; y55b1al.2; his-48; rpb-12; his-5; his-68; gut-2; mxl-1; his-47; his-58; rpb-10; lsm-6; nhr-37; rab-18	1.60E-02	nucleobase, nucleoside, nucleotide and nucleic acid metabolic process;
GO:0048806	his-18; rpb-11; pfd-6; his-48; zk686.1; his-5; his-58; rpl-41	2.75E-02	sex differentiation#genitalia development;
GO:0022414	his-18; rpb-11; pfd-6; his-48; zk686.1; his-5; his-68; his-47; dyrb-1; his-58; rpl-41	2.90E-02	reproductive process;
