# Supplementary material for: Gene expression profiling of oxidative stress response of C. elegans aging defective AMPK mutants using massively parallel transcriptome sequencing
Source: BMC Res Notes. 2011 Feb 8;4:34. doi: 10.1186/1756-0500-4-34 (PMC3045954; doi:10.1186/1756-0500-4-34)
Supplement: Additional file 17 — Supplementary Table S16. Genes which show no significant change in expression, or are significantly down-regulated in unstressed aak-2 mutants that are commonly up-regulated in both stressed aak-2 and stressed wild type [file 1756-0500-4-34-S17.PDF]

Supplementary Table 16. genes which show no significant change in expression, or are significantly down-regulated in unstressed aak-2 mutants that are commonly up-regulated in both stressed aak-2 and stressed wild type

GO	Commonly up regulated genes in stressed N2 and aak-2 mutants, but no significant changes in unstressed aak-2	Pvalue	GO as name
GO:0006817	col-73; col-130; col-161; col-112; col-109; col-155; col-14; dpy-13; col-12; dpy-5; col-38; col-77; col-154; bli-1; col-145; sqt-1; col-166; rol-8; sqt-2; col-175; ram-2; col-48; col-49; col-180; col-104; col-71; col-63; col-107; col-169; col-13; dpy-4; col-60; col-90; col-167; bli-2; col-10; col-144; col-162; col-157	1.96E-44	phosphate transport;
GO:0051234	col-73; col-130; col-161; col-112; col-109; col-155; w10d9.5; tag-170; col-14; col-12; dpy-13; dpy-5; trap-3; col-77; col-38; col-154; bli-1; sqt-1; col-145; col-166; ftn-2; rol-8; sqt-2; ram-2; col-175; col-48; col-49; vha-1; col-180; col-104; col-71; col-63; f57b10.5; col-107; dlc-1; col-169; col-13; dpy-4; col-90; col-60; arf-3; col-167; bli-2; col-10; f59e10.3; col-144; col-162; col-157	2.77E-28	establishment of localization;
GO:0007626	tct-1; c15c7.5; iff-2; t27f7.3; mlt-8; col-155; col-180; snr-4; tag-170; c27d6.3; dlc-1; unc-60; dpy-13; col-169; f49c12.11; dpy-4; pas-5; bli-1; col-154; arf-3; col-167; clec-1; col-166; bli-2; col-157	5.41E-09	locomotory behavior;
GO:0048856	iff-2; mlc-4; mlt-8; col-155; col-180; w10d9.5; tag-170; skr-1; col-14; dlc-1; dpy-13; unc-60; dpy-5; col-169; lon-8; f49c12.11; bli-1; col-154; col-167; arf-3; sqt-1; col-166; bli-2; t02h6.11; ram-2; col-157	1.41E-07	anatomical structure development;
GO:0010171	f49c12.11; mlc-4; arf-3; col-154; col-167; col-166; mlt-8; col-155; col-180; w10d9.5; t02h6.11; dlc-1; dpy-13; col-157; col-169	2.28E-06	body morphogenesis;
GO:0007610	tct-1; c15c7.5; iff-2; t27f7.3; mlt-8; col-155; col-180; snr-4; tag-170; c27d6.3; dlc-1; unc-60; dpy-13; col-169; f49c12.11; dpy-4; pas-5; bli-1; col-154; arf-3; col-167; clec-1; col-166; bli-2; col-157	2.28E-06	behavior;
GO:0050896	tct-1; c15c7.5; iff-2; t27f7.3; hsp-16.41; mlt-8; col-155; col-180; snr-4; tag-170; c27d6.3; dlc-1; unc-60; dpy-13; col-169; f49c12.11; pas-5; dpy-4; col-154; arf-3; bli-1; col-167; clec-1; hsp-16.2; col-166; bli-2; col-157	2.69E-05	response to stimulus;
GO:0018988	tag-170; k12h4.4; arf-3; bli-1; bli-2	1.13E-02	molting cycle, protein-based cuticle;
GO:0040002	bli-1; sqt-1; bli-2	1.79E-02	collagen and cuticulin-based cuticle development;
GO:0006986	hsp-16.2; hsp-16.41	3.03E-02	response to unfolded protein; response to protein stimulus;
GO:0018996	tag-170; bli-1; bli-2	4.59E-02	molting cycle, collagen and cuticulin-based cuticle;
GO:0009607	hsp-16.2; hsp-16.41	5.10E-02	response to biotic stimulus;
GO:0007423	ram-2	5.11E-02	sensory organ development;
GO:0007110	mlc-4	5.11E-02	cytokinesis after meiosis I;
GO:0009264	f09e5.3	5.11E-02	deoxyribonucleotide catabolic process;
GO:0008104	f59e10.3; trap-3; w10d9.5; mlc-4; c28h8.4; arf-3	6.08E-02	protein localization;
GO:0006413	iff-2; t27f7.3	6.64E-02	translational initiation;
GO:0051641	f59e10.3; trap-3; tag-170; w10d9.5; c28h8.4; arf-3; dlc-1	8.36E-02	cellular localization;

GO:0016485	k12h4.4; wrt-4	8.36E-02	protein processing;
GO:0030042	unc-60	8.36E-02	actin filament depolymerization;
GO:0032507	c28h8.4	8.36E-02	maintenance of cellular protein localization; protein retention in ER;
GO:0008340	f59e10.3; t02h6.11; t27f7.3; dod-6; hsp-16.2	8.51E-02	determination of adult life span; aging;
