# Supplementary material for: Gene expression profiling of oxidative stress response of C. elegans aging defective AMPK mutants using massively parallel transcriptome sequencing
Source: BMC Res Notes. 2011 Feb 8;4:34. doi: 10.1186/1756-0500-4-34 (PMC3045954; doi:10.1186/1756-0500-4-34)
Supplement: Additional file 18 — Supplementary Table S17. Genes significantly up or down-regulated only in unstressed aak-2 mutants relative to wild type, but insignificantly changed in stressed wild type and stressed aak-2 mutants relative to wild type [file 1756-0500-4-34-S18.PDF]

Supplementary Table 17. Genes significantly up or down-regulated only in unstressed aak-2 mutants relative to wild type, but insignificantly changed in stressed wild type and stressed aak-2 mutants relative to wild type

Gene	Unstressed aak-2	pval
C13B7.6	1.48	7.74E-05
Y45G12C.16	1.47	9.83E-05
C18H9.3	1.37	5.43E-03
rrn-3.56	1.34	2.27E-146
Y61A9LA.8	1.33	8.76E-03
col-95	1.30	9.64E-11
eif-3.I	1.23	1.31E-03
F58G11.2	1.23	7.62E-03
rrn-3.1	1.17	5.61E-200
lin-35	1.12	6.41E-03
smk-1	1.07	1.32E-03
rrn-1.1	1.05	2.42E-149
rrn-1.2	1.05	2.42E-149
daf-16	1.02	5.66E-03
Y37E11AL.3	1.02	5.98E-03
ZK858.1	1.01	5.72E-04
csr-1	0.98	4.30E-05
F42G9.1	0.98	4.28E-03
col-106	0.97	3.85E-12
F20G4.3	0.97	8.11E-04
inx-14	0.96	3.59E-03
egl-27	0.96	3.85E-03
col-179	0.95	9.47E-16
daz-1	0.95	1.75E-06
ptc-2	0.95	1.05E-03
rsks-1	0.95	5.66E-03
Y71H2AM.20	0.95	6.96E-03
xpo-3	0.95	7.20E-03
ZK973.1	0.94	5.32E-03
C06A5.6	0.94	5.50E-03
vit-4	0.93	3.38E-152
aka-1	0.93	2.87E-03
F45H11.3	0.93	4.66E-03
vit-3	0.92	2.42E-144
vit-1	0.92	1.62E-41
top-1	0.92	2.67E-03
mom-5	0.92	5.55E-03
pap-1	0.92	6.19E-03
C23G10.8	0.92	9.52E-03
rpb-2	0.92	9.52E-03
pqn-20	0.91	3.63E-03
sax-7	0.91	4.44E-03

F58G1.1	0.91	5.28E-03
prom-1	0.91	8.47E-03
ceh-38	0.90	7.68E-03
lig-1	0.90	8.69E-03
F37C12.7	0.88	3.25E-05
let-711	0.88	9.17E-04
hsr-9	0.87	2.04E-03
ptp-2	0.87	8.32E-03
npl-4.2	0.86	6.10E-04
mep-1	0.86	5.02E-03
lin-45	0.86	7.82E-03
alh-8	0.85	6.20E-09
sur-6	0.85	1.51E-03
ani-2	0.85	1.87E-03
mat-1	0.85	5.33E-03
ran-5	0.85	7.41E-03
cdc-14	0.85	7.44E-03
npp-14	0.85	9.07E-03
M02B1.3	0.84	6.38E-04
T19B4.2	0.84	2.25E-03
ruvb-1	0.84	2.60E-03
T05F1.2	0.84	3.37E-03
mtx-1	0.84	5.88E-03
cyld-1	0.84	7.54E-03
prp-8	0.83	2.05E-03
cpb-3	0.83	2.50E-03
F13B12.6	0.83	8.38E-03
C16C10.3	0.82	2.22E-04
ccr-4	0.82	7.37E-04
egl-45	0.82	8.06E-04
rnp-8	0.82	1.46E-03
atx-2	0.82	3.18E-03
eif-3.B	0.82	4.12E-03
tag-153	0.82	4.46E-03
scc-3	0.82	6.45E-03
C08B11.3	0.82	6.75E-03
pbrm-1	0.82	6.89E-03
cand-1	0.82	8.84E-03
Y54E10A.6	0.81	6.30E-03
T21B10.3	0.81	7.74E-03
tag-319	0.81	7.80E-03
npl-4.1	0.80	9.79E-04
srgp-1	0.80	1.94E-03
daf-18	0.80	2.22E-03
mtm-3	0.80	5.25E-03
F53C3.13	0.80	9.09E-03
F36D4.5	0.79	2.77E-03

ZK863.4	0.79	3.89E-03
C14C10.5	0.79	4.34E-03
Y104H12BR.1	0.79	4.59E-03
C01G5.6	0.79	7.87E-03
F21H12.6	0.79	9.32E-03
C36A4.4	0.78	3.27E-03
imp-1	0.78	4.46E-03
zer-1	0.78	4.98E-03
F21D5.1	0.78	5.74E-03
ani-1	0.78	6.94E-03
F17C11.10	0.78	8.67E-03
let-92	0.77	7.02E-04
K10C3.4	0.77	5.32E-03
unc-76	0.77	6.32E-03
gla-3	0.77	6.86E-03
T23B5.1	0.77	7.40E-03
T09E8.1	0.77	8.71E-03
T12A2.2	0.76	8.57E-04
mbk-2	0.76	2.05E-03
dnj-5	0.76	2.23E-03
puf-8	0.76	3.76E-03
F55A11.7	0.76	7.99E-03
ptc-1	0.74	7.11E-04
pas-7	0.74	5.98E-03
spd-5	0.74	8.84E-03
eat-6	0.73	7.34E-06
F14B4.2	0.73	6.82E-04
bath-40	0.73	1.97E-03
atf-7	0.73	2.25E-03
F44E7.4	0.73	2.72E-03
Y57G11C.9	0.73	5.76E-03
pqn-51	0.73	7.48E-03
hcf-1	0.73	9.43E-03
cey-2	0.72	1.13E-13
K08E3.5	0.72	5.10E-04
EEED8.3	0.72	2.08E-03
T13C2.6	0.72	3.81E-03
fbxa-215	0.72	4.36E-03
paa-1	0.71	3.86E-04
dab-1	0.71	5.63E-04
rpn-1	0.71	7.29E-04
top-2	0.71	4.53E-03
gld-3	0.71	5.01E-03
F35G2.1	0.71	5.87E-03
arx-2	0.71	6.53E-03
T14G10.5	0.71	7.98E-03
npp-22	0.71	9.38E-03

rps-20	-0.71	2.07E-11
F53F1.4	-0.74	2.21E-04
snr-3	-0.75	1.22E-03
col-146	-0.75	4.06E-03
tag-277	-0.76	6.43E-03
rps-10	-0.77	1.21E-20
MTCE.3	-0.78	7.04E-09
rps-15	-0.83	7.33E-21
lys-1	-0.83	1.04E-04
K07F5.15	-0.84	8.08E-03
col-117	-0.85	2.70E-08
col-3	-0.85	3.88E-08
nduf-6	-0.85	3.92E-03
F46F2.3	-0.86	4.54E-05
tag-174	-0.90	4.07E-06
col-147	-0.96	7.78E-04
ttr-48	-0.96	4.35E-03
R07E4.3	-0.97	7.59E-03
F54D5.4	-0.99	2.87E-04
D2030.4	-1.05	6.73E-04
zig-7	-1.06	9.15E-16
F54C9.3	-1.10	3.22E-03
T27E9.2	-1.11	2.00E-04
rps-16	-1.16	4.09E-25
ned-8	-1.18	1.18E-05
T20G5.8	-1.25	1.89E-03
nspc-3	-1.25	5.51E-03
emo-1	-1.26	7.71E-16
fipr-21	-1.26	4.09E-04
nspc-12	-1.26	7.34E-03
nlp-33	-1.27	5.30E-03
snr-6	-1.29	2.25E-11
rla-2	-1.30	1.71E-21
rpl-23	-1.32	1.97E-42
snr-7	-1.33	5.92E-05
rps-17	-1.34	2.53E-62
Y59E9AR.7	-1.35	1.65E-15
nspc-10	-1.35	9.93E-03
Y59E9AR.1	-1.37	2.68E-15
msp-71	-1.37	1.73E-08
msp-52	-1.38	1.97E-18
lys-2	-1.39	1.28E-04
msp-152	-1.41	3.39E-12
F13G3.10	-1.43	1.25E-04
msp-31	-1.46	2.32E-13
msp-38	-1.48	1.59E-10
msp-33	-1.53	1.77E-12

msp-58	-1.54	2.60E-18
msp-78	-1.54	5.14E-17
pcbd-1	-1.54	6.56E-03
msp-53	-1.59	1.88E-23
msp-57	-1.61	4.97E-20
C04G2.3	-1.61	1.09E-10
msp-40	-1.62	1.61E-09
F41F3.3	-1.63	5.10E-07
Y59H11AM.1	-1.63	6.23E-07
msp-79	-1.65	7.28E-23
nlp-36	-1.66	2.17E-08
msp-59	-1.67	5.58E-26
nspc-13	-1.68	5.66E-03
fipr-7	-1.70	4.05E-03
msp-49	-1.71	2.35E-06
msp-65	-1.73	3.38E-24
Y43C5A.1	-1.73	6.32E-04
msp-19	-1.74	4.63E-30
msp-3	-1.74	1.96E-10
T07A5.5	-1.74	1.51E-06
msp-36	-1.76	4.41E-18
msp-51	-1.77	6.68E-31
msp-10	-1.77	1.82E-27
msp-50	-1.78	8.43E-09
T02B11.3	-1.79	9.93E-03
msp-55	-1.80	1.46E-27
msp-56	-1.83	2.24E-29
dao-2	-1.83	4.12E-04
msp-77	-1.89	2.58E-27
F44D12.3	-1.92	3.96E-06
ZK1248.4	-2.03	3.89E-19
msp-64	-2.04	4.21E-17
msp-42	-2.07	1.30E-06
nspc-14	-2.08	3.28E-04
F35B12.7	-2.09	1.60E-10
BE10.6	-2.20	2.78E-08
msp-63	-2.23	1.43E-05
C31C9.7	-2.60	4.99E-03
