# Supplementary material for: Gene expression profiling of oxidative stress response of C. elegans aging defective AMPK mutants using massively parallel transcriptome sequencing
Source: BMC Res Notes. 2011 Feb 8;4:34. doi: 10.1186/1756-0500-4-34 (PMC3045954; doi:10.1186/1756-0500-4-34)
Supplement: Additional file 19 — Supplementary Table S18. Number of reads mapped to transcripts before and after normalization [file 1756-0500-4-34-S19.PDF]

Supplementary Table 18. Number of reads mapped to transcripts before and after normalization

Transcript	Stressed N2	Stressed aak-2	Unstressed N2	Unstressed aak-2	Stressed N2 after normalization	Stressed aak-2 after normalization	Unstressed N2 after normalization	Unstressed aak-2 after normalization
2L52.1	13	31	21	26	2.80E-06	2.65E-06	1.82E-06	2.25E-06
2RSSE.1	36	39	71	77	3.11E-06	3.17E-06	3.97E-06	5.33E-06
2RSSE.2	5	10	11	13	2.80E-06	2.65E-06	1.82E-06	2.25E-06
3R5.1	40	62	107	56	6.92E-06	1.01E-05	1.20E-05	7.78E-06
3R5.2	23	40	50	74	5.77E-06	9.47E-06	8.14E-06	1.49E-05
4R79.1a	16	21	10	3	2.80E-06	2.65E-06	1.82E-06	2.25E-06
4R79.1b	11	13	2	2	2.80E-06	2.65E-06	1.82E-06	2.25E-06
4R79.2	13	12	3	4	2.80E-06	2.65E-06	1.82E-06	2.25E-06
6R55.2	3	3	2	6	2.80E-06	2.65E-06	1.82E-06	2.25E-06
AC3.1	9	5	6	4	2.80E-06	2.65E-06	1.82E-06	2.25E-06
AC3.10	12	36	20	13	2.80E-06	3.60E-06	1.82E-06	2.25E-06
AC3.2	213	284	145	172	1.47E-05	1.85E-05	6.49E-06	9.51E-06
AC3.3	180	213	32	47	1.44E-05	1.61E-05	1.82E-06	3.01E-06
AC3.4	169	191	29	44	1.48E-05	1.58E-05	1.82E-06	3.10E-06
AC3.6	2	6	11	3	2.80E-06	2.65E-06	1.82E-06	2.25E-06
AC3.7	131	58	51	56	9.02E-06	3.78E-06	2.30E-06	3.10E-06
AC3.8	44	63	47	61	3.08E-06	4.18E-06	2.15E-06	3.44E-06
AC3.9	79	90	59	69	4.90E-06	5.29E-06	2.39E-06	3.44E-06
AC7.1a	8	13	11	16	2.80E-06	2.65E-06	1.82E-06	2.25E-06
AC7.1b	8	10	13	17	2.80E-06	2.65E-06	1.82E-06	2.25E-06
AC7.2a.1	206	296	230	347	1.20E-05	1.63E-05	8.73E-06	1.63E-05
AC7.2a.2	203	289	219	342	1.07E-05	1.44E-05	7.51E-06	1.45E-05
AC7.3	11	20	6	5	2.80E-06	2.96E-06	1.82E-06	2.25E-06
AC8.1	463	384	487	786	2.34E-05	1.84E-05	1.60E-05	3.19E-05
AC8.10	16	42	11	27	2.80E-06	3.36E-06	1.82E-06	2.25E-06
AC8.11	10	12	38	17	2.80E-06	2.65E-06	4.23E-06	2.34E-06
AC8.12	5	11	6	13	2.80E-06	2.65E-06	1.82E-06	2.25E-06
AC8.2	9	14	7	6	2.80E-06	2.65E-06	1.82E-06	2.25E-06
AC8.3	16	42	11	27	2.80E-06	3.36E-06	1.82E-06	2.25E-06
AC8.4	10	12	38	17	2.80E-06	2.65E-06	4.23E-06	2.34E-06
AC8.5	20	20	81	67	2.80E-06	2.65E-06	5.03E-06	5.13E-06
AC8.6	86	57	120	158	2.18E-05	1.37E-05	1.98E-05	3.22E-05
AC8.7	5	11	6	13	2.80E-06	2.65E-06	1.82E-06	2.25E-06
AC8.9	132	123	133	198	2.81E-05	2.47E-05	1.84E-05	3.38E-05
AH10.1	42	73	48	25	2.80E-06	4.55E-06	2.06E-06	2.25E-06
AH10.2	1	4	4	1	2.80E-06	2.65E-06	1.82E-06	2.25E-06
AH10.3	9	14	12	14	2.80E-06	2.65E-06	1.82E-06	2.25E-06
AH10.4	6	8	6	3	3.08E-06	3.86E-06	2.00E-06	2.25E-06
AH6.1	10	18	11	10	2.80E-06	2.65E-06	1.82E-06	2.25E-06
AH6.10	8	3	2	2	2.80E-06	2.65E-06	1.82E-06	2.25E-06
AH6.11	5	9	6	2	2.80E-06	2.65E-06	1.82E-06	2.25E-06
AH6.12	5	7	2	4	2.80E-06	2.65E-06	1.82E-06	2.25E-06
AH6.13	7	4	2	3	2.80E-06	2.65E-06	1.82E-06	2.25E-06
AH6.14	12	4	5	3	2.80E-06	2.65E-06	1.82E-06	2.25E-06
AH6.16	8	9	8	5	2.80E-06	2.65E-06	1.82E-06	2.25E-06
AH6.2	17	36	16	10	2.80E-06	3.84E-06	1.82E-06	2.25E-06
AH6.3	27	42	11	12	4.37E-06	6.40E-06	1.82E-06	2.25E-06
AH6.4	4	5	2	2	2.80E-06	2.65E-06	1.82E-06	2.25E-06
AH6.5	1902	1813	3350	3452	1.12E-04	1.01E-04	1.29E-04	1.64E-04
AH6.6	6	3	13	7	2.80E-06	2.65E-06	1.82E-06	2.25E-06
AH6.7	11	4	17	5	2.80E-06	2.65E-06	1.82E-06	2.25E-06
AH6.8	5	5	3	3	2.80E-06	2.65E-06	1.82E-06	2.25E-06
AH6.9	5	7	8	6	2.80E-06	2.65E-06	1.82E-06	2.25E-06

AH9.1	10	9	8	9	2.80E-06	2.65E-06	1.82E-06	2.25E-06
AH9.2	54	39	61	74	6.69E-06	4.58E-06	4.92E-06	7.35E-06
AH9.3	39	67	44	48	4.65E-06	7.54E-06	3.41E-06	4.59E-06
AH9.4	3	6	7	5	2.80E-06	2.65E-06	1.82E-06	2.25E-06
AH9.6	7	15	12	10	2.80E-06	2.65E-06	1.82E-06	2.25E-06
B0001.1	380	342	506	500	5.20E-05	4.42E-05	4.50E-05	5.49E-05
B0001.2	632	769	424	893	2.43E-05	2.80E-05	1.06E-05	2.76E-05
B0001.4	455	421	503	580	6.82E-05	5.96E-05	4.91E-05	6.99E-05
B0001.5	107	114	157	190	6.44E-06	6.48E-06	6.16E-06	9.20E-06
B0001.6.1	446	529	466	758	2.14E-05	2.40E-05	1.46E-05	2.93E-05
B0001.6.2	434	517	457	736	2.22E-05	2.50E-05	1.52E-05	3.02E-05
B0001.6.3	415	490	438	715	2.28E-05	2.55E-05	1.57E-05	3.16E-05
B0001.7	548	422	866	1031	2.65E-05	1.93E-05	2.73E-05	4.01E-05
B0001.8.1	216	274	329	452	1.47E-05	1.76E-05	1.46E-05	2.47E-05
B0001.8.2	191	237	286	402	1.44E-05	1.70E-05	1.41E-05	2.44E-05
B0001.8.3	199	245	292	405	1.50E-05	1.75E-05	1.44E-05	2.46E-05
B0019.1	125	188	93	219	5.85E-06	8.31E-06	2.82E-06	8.21E-06
B0019.2	236	250	395	442	1.19E-05	1.19E-05	1.30E-05	1.79E-05
B0024.1	679	531	757	325	7.36E-05	5.43E-05	5.34E-05	2.83E-05
B0024.10.1	434	466	440	615	2.65E-05	2.69E-05	1.75E-05	3.02E-05
B0024.10.2	391	428	395	573	2.54E-05	2.63E-05	1.67E-05	2.99E-05
B0024.11	368	416	389	527	2.30E-05	2.46E-05	1.59E-05	2.65E-05
B0024.12	30	57	25	21	5.46E-06	9.81E-06	2.97E-06	3.08E-06
B0024.13a	333	335	415	366	4.01E-05	3.81E-05	3.25E-05	3.54E-05
B0024.13b	319	322	390	356	3.47E-05	3.31E-05	2.76E-05	3.11E-05
B0024.14a	101	202	89	144	3.84E-06	7.22E-06	2.19E-06	4.39E-06
B0024.14b	103	197	84	146	3.92E-06	7.06E-06	2.08E-06	4.45E-06
B0024.14c.1	86	180	80	129	3.61E-06	7.14E-06	2.19E-06	4.36E-06
B0024.14c.2	36	92	39	80	2.80E-06	6.45E-06	1.88E-06	4.77E-06
B0024.14d	99	197	86	142	3.75E-06	7.06E-06	2.13E-06	4.34E-06
B0024.14e	79	158	70	121	3.64E-06	6.88E-06	2.10E-06	4.48E-06
B0024.15	7	6	13	9	2.80E-06	2.65E-06	1.82E-06	2.25E-06
B0024.2	85	133	73	56	1.06E-05	1.57E-05	5.96E-06	5.65E-06
B0024.3	8	12	13	12	2.80E-06	2.65E-06	1.82E-06	2.25E-06
B0024.4	97	39	60	23	8.82E-06	3.36E-06	3.55E-06	2.25E-06
B0024.6	13	18	24	6	2.80E-06	2.65E-06	1.82E-06	2.25E-06
B0024.8	12	28	25	16	2.80E-06	2.65E-06	1.82E-06	2.25E-06
B0025.1b.1	255	400	251	537	9.83E-06	1.45E-05	6.29E-06	1.66E-05
B0025.1b.2	269	431	271	553	1.08E-05	1.63E-05	7.05E-06	1.78E-05
B0025.1c.1	269	431	271	553	1.08E-05	1.63E-05	7.05E-06	1.78E-05
B0025.1c.2	248	400	241	521	1.03E-05	1.57E-05	6.52E-06	1.74E-05
B0025.1d.1	196	323	210	444	1.01E-05	1.58E-05	7.07E-06	1.84E-05
B0025.1d.2	189	321	198	427	9.83E-06	1.58E-05	6.71E-06	1.78E-05
B0025.1e	196	323	210	444	1.01E-05	1.58E-05	7.07E-06	1.84E-05
B0025.2.1	635	790	600	1035	4.40E-05	5.17E-05	2.71E-05	5.76E-05
B0025.2.2	583	735	538	956	4.34E-05	5.17E-05	2.61E-05	5.72E-05
B0025.2.3	599	753	577	996	4.44E-05	5.27E-05	2.78E-05	5.93E-05
B0025.4	128	160	170	149	2.41E-05	2.85E-05	2.08E-05	2.25E-05
B0034.1	21	40	10	13	3.08E-06	5.53E-06	1.82E-06	2.25E-06
B0034.2	1	1	0	0	2.80E-06	2.65E-06	1.82E-06	2.25E-06
B0034.3a	329	469	315	450	1.23E-05	1.66E-05	7.67E-06	1.35E-05
B0034.3b	67	100	55	73	1.49E-05	2.10E-05	7.96E-06	1.30E-05
B0034.3c.1	391	555	403	557	1.28E-05	1.71E-05	8.56E-06	1.46E-05
B0034.3c.2	128	187	133	174	1.40E-05	1.94E-05	9.49E-06	1.53E-05
B0034.4	6	8	5	3	2.80E-06	2.65E-06	1.82E-06	2.25E-06
B0034.5	7	4	3	3	2.80E-06	2.65E-06	1.82E-06	2.25E-06
B0034.7	7	7	7	4	3.30E-06	3.12E-06	2.15E-06	2.25E-06
B0035.10	39	51	82	69	8.18E-06	1.01E-05	1.12E-05	1.16E-05
B0035.11	358	495	488	597	2.80E-05	3.66E-05	2.48E-05	3.75E-05

B0035.12.1	1142	1007	1073	1769	4.48E-05	3.73E-05	2.74E-05	5.57E-05
B0035.12.2	1001	875	942	1547	4.43E-05	3.66E-05	2.71E-05	5.50E-05
B0035.13	248	397	380	172	2.76E-05	4.17E-05	2.75E-05	1.54E-05
B0035.15	211	241	140	189	2.54E-05	2.74E-05	1.10E-05	1.83E-05
B0035.16	42	54	50	52	4.14E-06	5.03E-06	3.21E-06	4.12E-06
B0035.18	89	73	176	52	3.65E-05	2.83E-05	4.70E-05	1.71E-05
B0035.1a	435	469	612	730	5.27E-05	5.36E-05	4.82E-05	7.10E-05
B0035.1b	588	593	779	964	5.42E-05	5.16E-05	4.67E-05	7.14E-05
B0035.2	355	450	333	491	3.16E-05	3.78E-05	1.93E-05	3.51E-05
B0035.3	317	307	350	346	4.46E-05	4.08E-05	3.21E-05	3.91E-05
B0035.4.1	318	355	646	303	6.48E-05	6.83E-05	8.56E-05	4.96E-05
B0035.4.2	267	305	487	264	6.32E-05	6.82E-05	7.50E-05	5.02E-05
B0035.5.1	500	709	548	697	3.24E-05	4.34E-05	2.31E-05	3.63E-05
B0035.5.2	451	642	471	635	3.21E-05	4.32E-05	2.18E-05	3.64E-05
B0035.6	700	604	1337	1618	3.17E-05	2.58E-05	3.94E-05	5.88E-05
B0035.7	57	67	323	102	1.66E-05	1.85E-05	6.13E-05	2.39E-05
B0035.8	134	103	404	148	3.65E-05	2.65E-05	7.16E-05	3.24E-05
B0035.9	23	26	34	16	8.26E-06	8.81E-06	7.94E-06	4.61E-06
B0041.1	6	2	3	3	2.80E-06	2.65E-06	1.82E-06	2.25E-06
B0041.2a.1	627	718	499	878	3.28E-05	3.54E-05	1.70E-05	3.68E-05
B0041.2a.2	625	713	496	876	3.27E-05	3.52E-05	1.69E-05	3.68E-05
B0041.2b	832	1009	635	1200	3.41E-05	3.91E-05	1.69E-05	3.95E-05
B0041.2c	393	423	293	530	3.59E-05	3.65E-05	1.74E-05	3.89E-05
B0041.3	30	29	31	35	7.06E-06	6.43E-06	4.74E-06	6.61E-06
B0041.4.1	14055	12791	19309	21735	1.45E-03	1.25E-03	1.30E-03	1.81E-03
B0041.4.2	13227	11822	18239	21184	1.30E-03	1.10E-03	1.16E-03	1.67E-03
B0041.5	405	455	468	663	3.00E-05	3.18E-05	2.25E-05	3.94E-05
B0041.6a	32	39	46	15	8.48E-06	9.76E-06	7.93E-06	3.19E-06
B0041.6b	32	39	46	15	8.48E-06	9.79E-06	7.94E-06	3.19E-06
B0041.7	1087	1041	832	1570	2.70E-05	2.44E-05	1.35E-05	3.14E-05
B0047.1a	4	15	14	10	2.80E-06	2.65E-06	1.82E-06	2.25E-06
B0047.1b	2	10	3	6	2.80E-06	2.65E-06	1.82E-06	2.25E-06
B0047.2	5	10	6	8	2.80E-06	2.65E-06	1.82E-06	2.25E-06
B0047.3	8	15	17	15	2.80E-06	2.65E-06	1.82E-06	2.25E-06
B0047.4	6	5	9	5	2.80E-06	2.65E-06	1.82E-06	2.25E-06
B0047.5	4	12	12	15	2.80E-06	2.65E-06	1.82E-06	2.25E-06
B0198.1	36	34	42	37	4.79E-06	4.29E-06	3.64E-06	3.96E-06
B0198.2	9	10	11	13	2.80E-06	2.65E-06	1.82E-06	2.25E-06
B0198.3	41	54	42	59	2.80E-06	2.65E-06	1.82E-06	2.25E-06
B0205.10.1	70	114	36	49	5.26E-06	8.09E-06	1.82E-06	2.95E-06
B0205.10.2	80	128	38	50	6.33E-06	9.55E-06	1.95E-06	3.17E-06
B0205.11	490	484	428	535	6.23E-05	5.81E-05	3.54E-05	5.46E-05
B0205.12	56	60	183	48	4.10E-05	4.15E-05	8.72E-05	2.82E-05
B0205.13	21	55	13	19	7.84E-06	1.94E-05	3.15E-06	5.69E-06
B0205.14	2	5	5	0	2.80E-06	2.65E-06	1.82E-06	2.25E-06
B0205.1a	334	326	464	655	1.67E-05	1.54E-05	1.51E-05	2.63E-05
B0205.1b	386	376	526	717	1.65E-05	1.51E-05	1.46E-05	2.45E-05
B0205.2a	5	8	3	4	2.80E-06	2.65E-06	1.82E-06	2.25E-06
B0205.2b	5	10	9	9	2.80E-06	2.65E-06	1.82E-06	2.25E-06
B0205.3.1	1078	930	1182	1537	9.43E-05	7.69E-05	6.73E-05	1.08E-04
B0205.3.2	889	770	889	1235	9.27E-05	7.59E-05	6.03E-05	1.03E-04
B0205.4	9	21	9	9	2.80E-06	2.65E-06	1.82E-06	2.25E-06
B0205.6	292	374	231	326	2.29E-05	2.77E-05	1.18E-05	2.05E-05
B0205.8	149	96	173	104	3.75E-05	2.28E-05	2.83E-05	2.10E-05
B0205.9	320	368	362	539	1.99E-05	2.16E-05	1.46E-05	2.69E-05
B0207.1	22	34	10	6	2.80E-06	2.65E-06	1.82E-06	2.25E-06
B0207.10	1	3	2	1	2.80E-06	2.65E-06	1.82E-06	2.25E-06
B0207.11	17	34	6	4	2.80E-06	4.36E-06	1.82E-06	2.25E-06
B0207.12a	45	66	25	43	2.80E-06	3.28E-06	1.82E-06	2.25E-06

B0207.12b	39	49	23	36	3.00E-06	3.54E-06	1.82E-06	2.25E-06
B0207.12c	21	23	8	19	2.80E-06	2.65E-06	1.82E-06	2.25E-06
B0207.2	8	12	6	2	2.80E-06	2.65E-06	1.82E-06	2.25E-06
B0207.3a	7	10	5	7	2.80E-06	2.65E-06	1.82E-06	2.25E-06
B0207.3b	9	9	5	8	2.80E-06	2.65E-06	1.82E-06	2.25E-06
B0207.4	252	307	511	519	2.53E-05	2.91E-05	3.33E-05	4.18E-05
B0207.5	82	82	88	44	2.80E-06	2.65E-06	1.82E-06	2.25E-06
B0207.6	145	190	210	194	2.01E-05	2.49E-05	1.90E-05	2.16E-05
B0207.7	12	28	8	6	2.80E-06	2.65E-06	1.82E-06	2.25E-06
B0207.8	5	7	21	10	2.80E-06	2.65E-06	3.59E-06	2.25E-06
B0207.9	13	15	7	6	3.42E-06	3.73E-06	1.82E-06	2.25E-06
B0212.1	13	24	14	11	2.80E-06	2.65E-06	1.82E-06	2.25E-06
B0212.2	2	6	5	3	2.80E-06	2.65E-06	1.82E-06	2.25E-06
B0212.3	66	132	69	138	3.64E-06	6.90E-06	2.48E-06	6.14E-06
B0212.4a	17	26	20	14	2.80E-06	2.65E-06	1.82E-06	2.25E-06
B0212.4b	16	23	19	14	2.80E-06	2.65E-06	1.82E-06	2.25E-06
B0212.4c	23	29	24	17	2.80E-06	2.65E-06	1.82E-06	2.25E-06
B0212.5	19	23	17	10	2.80E-06	2.65E-06	1.82E-06	2.25E-06
B0212.6	5	2	9	0	2.80E-06	2.65E-06	1.82E-06	2.25E-06
B0213.10	11	10	6	5	2.80E-06	2.65E-06	1.82E-06	2.25E-06
B0213.11	16	15	13	11	2.80E-06	2.65E-06	1.82E-06	2.25E-06
B0213.12	12	11	8	18	2.80E-06	2.65E-06	1.82E-06	2.25E-06
B0213.14	68	128	109	154	4.62E-06	8.20E-06	4.81E-06	8.39E-06
B0213.15a	69	87	42	48	4.76E-06	5.69E-06	1.90E-06	2.68E-06
B0213.15b	49	60	35	33	3.53E-06	4.10E-06	1.82E-06	2.25E-06
B0213.15c	10	6	5	7	3.11E-06	2.65E-06	1.82E-06	2.25E-06
B0213.16	7	11	8	2	2.80E-06	2.65E-06	1.82E-06	2.25E-06
B0213.18	11	6	6	8	1.16E-05	5.98E-06	4.12E-06	6.79E-06
B0213.2	402	274	515	207	1.51E-04	9.70E-05	1.26E-04	6.23E-05
B0213.3	62	111	104	64	3.51E-05	5.93E-05	3.83E-05	2.91E-05
B0213.4	73	295	97	107	2.32E-05	8.87E-05	2.01E-05	2.73E-05
B0213.5	37	115	40	22	5.77E-06	1.69E-05	4.06E-06	2.74E-06
B0213.6	67	218	44	64	2.05E-05	6.30E-05	8.76E-06	1.57E-05
B0213.7	5	5	5	5	2.80E-06	2.65E-06	1.82E-06	2.25E-06
B0213.8	5	5	3	2	2.80E-06	2.65E-06	1.82E-06	2.25E-06
B0213.9	3	5	6	2	2.80E-06	2.65E-06	1.82E-06	2.25E-06
B0218.1a.1	141	311	160	219	8.37E-06	1.75E-05	6.20E-06	1.05E-05
B0218.1a.2	132	301	133	208	8.43E-06	1.81E-05	5.52E-06	1.07E-05
B0218.1a.3	127	295	126	203	8.20E-06	1.80E-05	5.30E-06	1.05E-05
B0218.1a.4	123	289	123	201	7.98E-06	1.77E-05	5.19E-06	1.05E-05
B0218.1b.1	109	267	112	167	7.84E-06	1.81E-05	5.23E-06	9.63E-06
B0218.1b.2	104	261	105	162	7.56E-06	1.80E-05	4.97E-06	9.47E-06
B0218.1b.3	100	255	102	160	7.31E-06	1.76E-05	4.87E-06	9.40E-06
B0218.2.1	180	634	114	220	1.05E-05	3.51E-05	4.34E-06	1.03E-05
B0218.2.2	166	585	103	194	1.08E-05	3.59E-05	4.35E-06	1.01E-05
B0218.3	109	116	113	183	1.06E-05	1.07E-05	7.18E-06	1.44E-05
B0218.5	6	29	8	8	2.80E-06	2.78E-06	1.82E-06	2.25E-06
B0218.6	144	96	124	114	1.66E-05	1.05E-05	9.31E-06	1.06E-05
B0218.7	68	133	71	32	7.14E-06	1.32E-05	4.85E-06	2.70E-06
B0218.8	34	275	46	160	3.92E-06	3.00E-05	3.46E-06	1.48E-05
B0222.1	5	26	8	4	2.80E-06	2.96E-06	1.82E-06	2.25E-06
B0222.10	2	5	3	8	2.80E-06	2.65E-06	1.82E-06	2.25E-06
B0222.11	6	10	19	13	2.80E-06	2.65E-06	1.82E-06	2.25E-06
B0222.2	2	6	4	7	2.80E-06	2.65E-06	1.82E-06	2.25E-06
B0222.3	22	74	24	60	2.80E-06	4.84E-06	1.82E-06	3.35E-06
B0222.4	20	43	17	31	2.80E-06	2.65E-06	1.82E-06	2.25E-06
B0222.5	146	247	130	211	1.08E-05	1.72E-05	6.23E-06	1.25E-05
B0222.6	5493	4334	3652	1995	6.28E-04	4.68E-04	2.72E-04	1.83E-04
B0222.7	3192	2248	1414	754	3.97E-04	2.64E-04	1.14E-04	7.53E-05

B0222.8	5547	4571	4059	2119	6.46E-04	5.03E-04	3.08E-04	1.98E-04
B0222.9	130	142	98	192	3.95E-06	4.07E-06	1.93E-06	4.68E-06
B0228.1	39	57	13	25	6.69E-06	9.23E-06	1.82E-06	3.44E-06
B0228.4a	426	593	347	512	9.74E-06	1.28E-05	5.16E-06	9.40E-06
B0228.4b	330	482	346	389	3.05E-06	4.21E-06	2.08E-06	2.90E-06
B0228.4c	1291	1900	1038	1560	6.30E-06	8.76E-06	3.30E-06	6.12E-06
B0228.4d	256	393	249	345	6.41E-06	9.31E-06	4.06E-06	6.95E-06
B0228.4e	866	1235	610	1049	1.20E-05	1.61E-05	5.48E-06	1.16E-05
B0228.5a	7	12	8	10	2.80E-06	2.65E-06	1.82E-06	2.25E-06
B0228.5b	15	19	9	15	2.83E-06	3.39E-06	1.82E-06	2.27E-06
B0228.6	10	15	11	14	2.80E-06	3.44E-06	1.82E-06	2.72E-06
B0228.8	6	4	10	9	2.80E-06	2.65E-06	1.82E-06	2.25E-06
B0228.9	2	4	21	7	2.80E-06	2.65E-06	1.82E-06	2.25E-06
B0238.1	24	53	27	37	2.80E-06	3.17E-06	1.82E-06	2.25E-06
B0238.10	282	315	600	430	3.07E-05	3.24E-05	4.25E-05	3.76E-05
B0238.12	4	31	13	18	2.80E-06	4.89E-06	1.82E-06	2.41E-06
B0238.13	23	29	26	20	2.80E-06	2.65E-06	1.82E-06	2.25E-06
B0238.14	2	2	7	2	2.80E-06	2.65E-06	1.82E-06	2.25E-06
B0238.15	4	1	8	2	2.80E-06	2.65E-06	2.35E-06	2.25E-06
B0238.3	7	3	2	1	2.80E-06	2.65E-06	1.82E-06	2.25E-06
B0238.5	6	5	5	4	2.80E-06	2.65E-06	1.82E-06	2.25E-06
B0238.6	4	3	8	2	2.80E-06	2.65E-06	1.82E-06	2.25E-06
B0238.7	2	5	5	3	2.80E-06	2.65E-06	1.82E-06	2.25E-06
B0238.8	6	6	8	7	2.80E-06	2.65E-06	1.82E-06	2.25E-06
B0240.2	16	38	22	13	2.80E-06	2.65E-06	1.82E-06	2.25E-06
B0240.3	39	34	42	31	2.80E-06	2.65E-06	1.82E-06	2.25E-06
B0240.4.1	811	869	1304	1726	4.63E-05	4.68E-05	4.84E-05	7.91E-05
B0240.4.2	750	803	1200	1624	4.75E-05	4.80E-05	4.94E-05	8.26E-05
B0244.10	34	47	17	29	2.80E-06	2.65E-06	1.82E-06	2.25E-06
B0244.11	16	7	20	16	3.02E-06	2.65E-06	2.44E-06	2.43E-06
B0244.4	6	9	4	8	2.80E-06	2.65E-06	1.82E-06	2.25E-06
B0244.5	7	6	9	5	2.80E-06	2.65E-06	1.82E-06	2.25E-06
B0244.6	17	23	16	13	2.80E-06	2.65E-06	1.82E-06	2.25E-06
B0244.7	5	10	7	7	2.80E-06	2.65E-06	1.82E-06	2.25E-06
B0244.8	2400	2279	4412	4794	1.52E-04	1.36E-04	1.82E-04	2.44E-04
B0244.9	27	34	21	18	5.07E-06	6.00E-06	2.55E-06	2.70E-06
B0244.t1	0	1	0	1	2.80E-06	2.65E-06	1.82E-06	2.25E-06
B0244.t3	0	1	0	0	2.80E-06	2.65E-06	1.82E-06	2.25E-06
B0250.1.1	13491	13226	18156	16415	1.79E-03	1.66E-03	1.57E-03	1.75E-03
B0250.1.2	12105	12049	16190	15731	1.72E-03	1.62E-03	1.50E-03	1.79E-03
B0250.10	3	4	6	2	2.80E-06	2.65E-06	1.82E-06	2.25E-06
B0250.2	13	9	8	2	2.80E-06	2.65E-06	1.82E-06	2.25E-06
B0250.3	7554	7037	10816	10028	1.08E-03	9.52E-04	1.01E-03	1.15E-03
B0250.4	16	12	11	15	2.80E-06	2.65E-06	1.82E-06	2.25E-06
B0250.5	399	346	429	679	4.38E-05	3.59E-05	3.07E-05	5.99E-05
B0250.6	3	5	2	4	2.80E-06	2.65E-06	1.82E-06	2.25E-06
B0250.7	383	477	567	717	7.01E-05	8.25E-05	6.75E-05	1.05E-04
B0250.8	11	13	11	15	2.80E-06	2.65E-06	1.82E-06	2.25E-06
B0250.9	24	22	22	31	2.80E-06	2.65E-06	1.82E-06	2.25E-06
B0252.1	30	55	34	51	2.80E-06	2.65E-06	1.82E-06	2.25E-06
B0252.2	49	124	44	77	3.11E-06	7.41E-06	1.82E-06	3.91E-06
B0252.3a	48	106	66	129	3.78E-06	7.91E-06	3.39E-06	8.19E-06
B0252.3b	57	119	68	136	4.00E-06	7.88E-06	3.10E-06	7.65E-06
B0252.3c.1	50	113	67	133	3.95E-06	8.41E-06	3.44E-06	8.41E-06
B0252.3c.2	48	106	66	129	3.78E-06	7.91E-06	3.39E-06	8.19E-06
B0252.4a	130	125	231	108	3.28E-05	2.98E-05	3.79E-05	2.19E-05
B0252.4b	148	145	251	121	3.19E-05	2.96E-05	3.52E-05	2.10E-05
B0252.5	38	50	28	16	4.70E-06	5.85E-06	2.26E-06	2.25E-06
B0252.8	10	22	23	3	5.32E-06	1.11E-05	7.98E-06	2.25E-06

B0261.2a	1263	1238	1519	2623	1.74E-05	1.61E-05	1.36E-05	2.90E-05
B0261.2b	1351	1331	1587	2719	1.77E-05	1.65E-05	1.35E-05	2.86E-05
B0261.4a	194	231	307	278	2.67E-05	3.00E-05	2.75E-05	3.07E-05
B0261.4b	149	191	222	230	2.56E-05	3.11E-05	2.49E-05	3.18E-05
B0261.5	8	8	10	4	2.80E-06	2.65E-06	1.82E-06	2.25E-06
B0261.6a	27	68	19	7	6.55E-06	1.56E-05	3.01E-06	2.25E-06
B0261.6b	23	50	14	7	5.80E-06	1.19E-05	2.30E-06	2.25E-06
B0261.7	1073	756	1425	1782	1.04E-04	6.96E-05	9.03E-05	1.39E-04
B0261.8	62	107	40	46	1.16E-05	1.90E-05	4.88E-06	6.93E-06
B0272.1	36	44	38	38	2.80E-06	3.02E-06	1.82E-06	2.25E-06
B0272.2	33	30	41	40	5.77E-06	4.95E-06	4.65E-06	5.60E-06
B0272.3	295	457	313	338	3.25E-05	4.76E-05	2.24E-05	2.99E-05
B0272.4	53	77	29	53	7.73E-06	1.06E-05	2.75E-06	6.21E-06
B0273.1	23	33	24	10	3.61E-06	4.92E-06	2.46E-06	2.25E-06
B0273.2.1	2683	2171	4605	4843	1.61E-04	1.23E-04	1.79E-04	2.33E-04
B0273.2.2	1972	1581	3394	3606	1.48E-04	1.12E-04	1.66E-04	2.17E-04
B0273.3	74	108	109	125	6.50E-06	8.94E-06	6.21E-06	8.79E-06
B0273.4a	116	146	65	146	4.31E-06	5.13E-06	1.82E-06	4.36E-06
B0273.4b	71	87	58	71	3.70E-06	4.29E-06	1.97E-06	2.97E-06
B0280.10	87	101	70	89	9.38E-06	1.03E-05	4.92E-06	7.71E-06
B0280.11	22	48	13	15	2.80E-06	3.84E-06	1.82E-06	2.25E-06
B0280.12a	26	25	25	16	2.80E-06	2.65E-06	1.82E-06	2.25E-06
B0280.12b	26	30	25	16	2.80E-06	2.65E-06	1.82E-06	2.25E-06
B0280.13	90	75	63	101	5.29E-06	4.15E-06	2.41E-06	4.77E-06
B0280.14	0	1	6	11	2.80E-06	2.65E-06	4.08E-06	9.24E-06
B0280.15	0	1	5	8	2.80E-06	2.65E-06	3.33E-06	6.61E-06
B0280.16	0	2	7	8	2.80E-06	2.65E-06	4.77E-06	6.72E-06
B0280.17	36	137	41	23	5.68E-06	2.05E-05	4.23E-06	2.92E-06
B0280.1a	174	218	216	293	1.81E-05	2.14E-05	1.46E-05	2.44E-05
B0280.1b	144	180	171	249	1.65E-05	1.95E-05	1.27E-05	2.29E-05
B0280.2	25	39	25	43	2.80E-06	2.65E-06	1.82E-06	2.25E-06
B0280.3.1	573	575	679	840	6.78E-05	6.42E-05	5.23E-05	7.98E-05
B0280.3.2	411	429	465	607	5.66E-05	5.58E-05	4.17E-05	6.72E-05
B0280.4	9	24	9	5	2.80E-06	3.49E-06	1.82E-06	2.25E-06
B0280.5	10232	8233	17072	20461	6.66E-04	5.06E-04	7.23E-04	1.07E-03
B0280.6	12	36	28	49	2.80E-06	5.03E-06	2.70E-06	5.80E-06
B0280.7	37	47	14	21	3.64E-06	4.39E-06	1.82E-06	2.25E-06
B0280.8	128	192	86	118	9.30E-06	1.32E-05	4.06E-06	6.88E-06
B0280.9	341	354	389	541	2.73E-05	2.68E-05	2.03E-05	3.48E-05
B0281.1	2	4	2	0	2.80E-06	2.65E-06	1.82E-06	2.25E-06
B0281.3	30	61	44	69	3.36E-06	6.48E-06	3.23E-06	6.23E-06
B0281.4	7	8	6	11	2.80E-06	2.65E-06	1.82E-06	2.25E-06
B0281.5	23	22	18	35	4.12E-06	3.73E-06	2.10E-06	5.04E-06
B0281.6	5	6	3	2	2.80E-06	2.65E-06	1.82E-06	2.25E-06
B0281.7	1	2	4	2	2.80E-06	2.65E-06	1.82E-06	2.25E-06
B0281.8	6	7	5	4	2.80E-06	2.65E-06	1.82E-06	2.25E-06
B0284.1	19	18	74	12	2.80E-06	2.65E-06	4.32E-06	2.25E-06
B0284.2	9	9	14	9	2.80E-06	2.65E-06	1.82E-06	2.25E-06
B0284.3	19	10	15	4	3.47E-06	2.65E-06	1.82E-06	2.25E-06
B0284.4	7	3	3	5	2.80E-06	2.65E-06	1.82E-06	2.25E-06
B0285.1	708	505	819	1410	3.27E-05	2.20E-05	2.46E-05	5.23E-05
B0285.10	7	3	3	4	2.80E-06	2.65E-06	1.82E-06	2.25E-06
B0285.11	8	20	8	2	2.80E-06	2.65E-06	1.82E-06	2.25E-06
B0285.3	66	60	91	51	1.38E-05	1.19E-05	1.24E-05	8.57E-06
B0285.4	235	329	176	273	2.28E-05	3.02E-05	1.11E-05	2.13E-05
B0285.5	455	456	535	736	2.34E-05	2.22E-05	1.79E-05	3.04E-05
B0285.6	20	16	23	11	2.80E-06	2.65E-06	1.82E-06	2.25E-06
B0285.7	114	110	73	148	4.45E-06	4.05E-06	1.86E-06	4.63E-06
B0285.8	31	33	25	30	3.11E-06	3.12E-06	1.82E-06	2.43E-06

B0285.9	89	90	75	67	8.99E-06	8.57E-06	4.92E-06	5.42E-06
B0285.t1	0	1	0	0	2.80E-06	2.65E-06	1.82E-06	2.25E-06
B0286.1	48	59	24	36	6.94E-06	8.07E-06	2.26E-06	4.18E-06
B0286.2a	40	62	32	58	2.80E-06	2.65E-06	1.82E-06	2.25E-06
B0286.2b	15	29	8	31	2.80E-06	2.65E-06	1.82E-06	2.25E-06
B0286.3	173	367	152	258	1.37E-05	2.75E-05	7.85E-06	1.65E-05
B0286.4a.1	245	322	277	421	1.65E-05	2.05E-05	1.22E-05	2.28E-05
B0286.4a.2	234	304	265	414	1.88E-05	2.31E-05	1.39E-05	2.67E-05
B0286.4b	193	260	215	370	1.96E-05	2.49E-05	1.42E-05	3.02E-05
B0286.4c.1	187	250	210	359	1.66E-05	2.09E-05	1.21E-05	2.56E-05
B0286.4c.2	220	283	227	380	1.77E-05	2.15E-05	1.19E-05	2.45E-05
B0286.4c.3	234	304	265	414	1.85E-05	2.28E-05	1.37E-05	2.64E-05
B0286.4d.1	255	358	301	462	1.86E-05	2.47E-05	1.43E-05	2.71E-05
B0286.4d.2	278	387	320	477	1.65E-05	2.17E-05	1.24E-05	2.28E-05
B0286.4d.3	226	326	258	424	1.96E-05	2.67E-05	1.46E-05	2.95E-05
B0286.4d.4	230	331	266	428	1.94E-05	2.64E-05	1.46E-05	2.90E-05
B0286.4d.5	234	304	265	414	1.88E-05	2.31E-05	1.39E-05	2.67E-05
B0286.5	20	20	19	28	2.80E-06	2.65E-06	1.82E-06	2.59E-06
B0286.6	2	3	3	2	2.80E-06	2.65E-06	1.82E-06	2.25E-06
B0294.1	9	7	13	7	2.80E-06	2.65E-06	2.19E-06	2.25E-06
B0294.3	0	0	3	0	2.80E-06	2.65E-06	1.82E-06	2.25E-06
B0302.1a	271	314	171	414	7.53E-06	8.23E-06	3.08E-06	9.22E-06
B0302.1b	239	268	144	348	7.87E-06	8.33E-06	3.08E-06	9.20E-06
B0302.1c	81	86	34	99	7.48E-06	7.51E-06	2.04E-06	7.35E-06
B0302.2	3	1	2	2	2.80E-06	2.65E-06	1.82E-06	2.25E-06
B0302.4	0	0	1	0	2.80E-06	2.65E-06	1.82E-06	2.25E-06
B0302.5	97	120	70	88	2.18E-05	2.55E-05	1.02E-05	1.59E-05
B0303.11	102	165	114	201	3.92E-06	6.00E-06	2.86E-06	6.23E-06
B0303.14	41	53	38	55	2.88E-06	3.54E-06	1.82E-06	3.13E-06
B0303.15	341	272	374	282	6.14E-05	4.63E-05	4.38E-05	4.08E-05
B0303.16	6	12	7	3	2.80E-06	2.65E-06	1.82E-06	2.25E-06
B0303.2	63	123	74	61	8.06E-06	1.49E-05	6.16E-06	6.27E-06
B0303.3.1	762	1528	865	1056	4.95E-05	9.38E-05	3.66E-05	5.51E-05
B0303.3.2	743	1492	812	1037	4.83E-05	9.16E-05	3.43E-05	5.41E-05
B0303.3.3	628	1232	668	902	5.21E-05	9.65E-05	3.61E-05	6.01E-05
B0303.4.1	824	827	942	1207	4.09E-05	3.88E-05	3.04E-05	4.82E-05
B0303.4.2	694	686	815	1055	4.16E-05	3.88E-05	3.18E-05	5.08E-05
B0303.7	97	147	159	139	6.19E-06	8.83E-06	6.60E-06	7.11E-06
B0303.8	6	5	4	2	2.80E-06	2.65E-06	1.82E-06	2.25E-06
B0303.9	238	322	297	403	1.34E-05	1.71E-05	1.09E-05	1.82E-05
B0304.1a	22	17	16	13	2.80E-06	2.65E-06	1.82E-06	2.25E-06
B0304.1b	22	19	16	13	2.80E-06	2.65E-06	1.82E-06	2.25E-06
B0304.1c	23	21	17	12	2.80E-06	2.65E-06	1.82E-06	2.25E-06
B0304.2	207	139	281	274	3.18E-05	2.02E-05	2.81E-05	3.38E-05
B0304.3	127	135	157	87	8.37E-06	8.38E-06	6.72E-06	4.61E-06
B0304.4	71	67	58	72	2.06E-05	1.83E-05	1.09E-05	1.67E-05
B0304.5	10	9	9	8	2.80E-06	2.65E-06	1.82E-06	2.25E-06
B0304.6	9	8	11	1	2.80E-06	2.65E-06	1.82E-06	2.25E-06
B0304.7	12	4	7	10	2.80E-06	2.65E-06	1.82E-06	2.25E-06
B0304.8	2	2	2	2	2.80E-06	2.65E-06	1.82E-06	2.25E-06
B0310.1	27	46	34	30	4.40E-06	7.09E-06	3.61E-06	3.94E-06
B0310.2.1	30	44	34	51	2.80E-06	2.96E-06	1.82E-06	2.92E-06
B0310.2.2	23	39	15	39	2.80E-06	3.31E-06	1.82E-06	2.81E-06
B0310.3	8	20	15	10	2.80E-06	2.65E-06	1.82E-06	2.25E-06
B0310.4	5	5	6	14	2.80E-06	2.65E-06	1.82E-06	3.71E-06
B0310.5	328	296	381	359	2.08E-05	1.77E-05	1.57E-05	1.83E-05
B0310.6	9	6	6	3	3.50E-06	2.65E-06	1.82E-06	2.25E-06
B0331.1	8	22	11	10	2.80E-06	2.65E-06	1.82E-06	2.25E-06
B0331.2	23	23	15	11	2.80E-06	2.65E-06	1.82E-06	2.25E-06

B0334.1.1	623	1006	460	397	1.28E-04	1.95E-04	6.15E-05	6.55E-05
B0334.1.2	578	922	403	372	1.31E-04	1.97E-04	5.92E-05	6.75E-05
B0334.10	3	4	3	6	2.80E-06	2.65E-06	1.82E-06	2.25E-06
B0334.11a	374	441	499	592	2.65E-05	2.95E-05	2.30E-05	3.37E-05
B0334.11b	338	382	456	544	2.82E-05	3.01E-05	2.48E-05	3.65E-05
B0334.13	26	23	10	10	7.76E-06	6.48E-06	1.95E-06	2.41E-06
B0334.2a	26	68	28	69	3.36E-06	8.31E-06	2.35E-06	7.15E-06
B0334.2b	34	85	36	86	3.78E-06	8.94E-06	2.61E-06	7.69E-06
B0334.3a.1	705	870	910	1189	4.02E-05	4.69E-05	3.38E-05	5.45E-05
B0334.3b	685	876	932	1211	3.30E-05	3.98E-05	2.92E-05	4.68E-05
B0334.4	697	639	547	674	9.39E-05	8.14E-05	4.80E-05	7.30E-05
B0334.5	769	692	905	964	5.03E-05	4.27E-05	3.85E-05	5.06E-05
B0334.6	24	19	21	13	2.80E-06	2.65E-06	1.82E-06	2.25E-06
B0334.7	10	5	3	8	2.80E-06	2.65E-06	1.82E-06	2.25E-06
B0334.8	692	556	946	1133	2.06E-05	1.56E-05	1.83E-05	2.71E-05
B0334.9	11	5	10	7	2.80E-06	2.65E-06	1.82E-06	2.25E-06
B0336.10.1	5340	5842	10007	3246	1.18E-03	1.22E-03	1.44E-03	5.75E-04
B0336.10.2	4555	5017	7958	2975	9.98E-04	1.04E-03	1.14E-03	5.24E-04
B0336.11a	57	71	41	39	3.72E-06	4.36E-06	1.82E-06	2.25E-06
B0336.11b	23	30	16	16	3.08E-06	3.81E-06	1.82E-06	2.25E-06
B0336.12	5	1	5	3	2.80E-06	2.65E-06	1.82E-06	2.25E-06
B0336.13	248	185	464	298	5.24E-05	3.69E-05	6.38E-05	5.06E-05
B0336.2.1	2309	2688	1955	3004	2.13E-04	2.34E-04	1.17E-04	2.23E-04
B0336.2.2	1318	1484	958	1553	2.27E-04	2.42E-04	1.08E-04	2.15E-04
B0336.3	839	809	1812	1903	3.31E-05	3.02E-05	4.66E-05	6.03E-05
B0336.4	114	110	85	122	6.94E-06	6.32E-06	3.37E-06	5.98E-06
B0336.5a	212	139	310	261	3.61E-05	2.24E-05	3.43E-05	3.57E-05
B0336.5b	229	146	339	283	3.72E-05	2.24E-05	3.58E-05	3.69E-05
B0336.6.1	524	665	910	1001	3.77E-05	4.52E-05	4.26E-05	5.79E-05
B0336.6.2	519	656	914	966	4.03E-05	4.81E-05	4.61E-05	6.02E-05
B0336.7a	184	207	253	362	1.35E-05	1.44E-05	1.21E-05	2.14E-05
B0336.7b.1	191	212	256	363	1.36E-05	1.43E-05	1.19E-05	2.08E-05
B0336.7b.2	173	199	208	313	1.25E-05	1.36E-05	9.78E-06	1.82E-05
B0336.8	199	250	484	156	6.24E-05	7.41E-05	9.88E-05	3.93E-05
B0336.9a	272	280	385	608	1.36E-05	1.32E-05	1.25E-05	2.43E-05
B0336.9b.1	268	274	381	605	1.33E-05	1.29E-05	1.23E-05	2.41E-05
B0336.9b.2	276	292	391	613	1.37E-05	1.37E-05	1.26E-05	2.45E-05
B0336.9c	29	35	40	61	5.40E-06	6.16E-06	4.87E-06	9.15E-06
B0336.9d	280	293	391	615	1.39E-05	1.38E-05	1.26E-05	2.45E-05
B0336.9e	81	77	92	157	1.59E-05	1.43E-05	1.17E-05	2.47E-05
B0344.1	9	8	12	12	2.80E-06	2.65E-06	1.82E-06	2.25E-06
B0344.2	43	61	19	31	2.88E-06	3.89E-06	1.82E-06	2.25E-06
B0344.t1	0	0	1	0	2.80E-06	2.65E-06	1.82E-06	2.25E-06
B0344.t2	0	0	1	0	2.80E-06	2.65E-06	1.82E-06	2.25E-06
B0344.t3	0	0	1	0	2.80E-06	2.65E-06	1.82E-06	2.25E-06
B0348.1	2	1	2	0	2.80E-06	2.65E-06	1.82E-06	2.25E-06
B0348.2	4	0	4	0	2.80E-06	2.65E-06	1.82E-06	2.25E-06
B0348.4a	288	397	241	489	6.50E-06	8.46E-06	3.53E-06	8.86E-06
B0348.4b	290	396	239	489	6.50E-06	8.38E-06	3.48E-06	8.79E-06
B0348.4c	119	148	87	167	5.99E-06	7.04E-06	2.86E-06	6.75E-06
B0348.5	5	10	6	2	2.80E-06	2.65E-06	1.82E-06	2.25E-06
B0348.6a.1	929	625	2504	2335	9.53E-05	6.05E-05	1.67E-04	1.92E-04
B0348.6a.2	737	530	2053	1971	8.78E-05	5.96E-05	1.59E-04	1.89E-04
B0348.6b	610	484	1827	1825	8.99E-05	6.74E-05	1.75E-04	2.16E-04
B0348.6c	595	468	1774	1769	8.80E-05	6.54E-05	1.71E-04	2.10E-04
B0350.2a.1	1088	1446	1153	1575	1.92E-05	2.41E-05	1.32E-05	2.23E-05
B0350.2a.2	1060	1380	1108	1513	1.90E-05	2.34E-05	1.29E-05	2.18E-05
B0350.2b.1	536	732	589	829	2.01E-05	2.59E-05	1.44E-05	2.50E-05
B0350.2b.2	521	696	562	788	2.10E-05	2.65E-05	1.47E-05	2.55E-05

B0350.2c.1	962	1281	987	1450	1.85E-05	2.33E-05	1.24E-05	2.24E-05
B0350.2c.2	936	1224	947	1392	1.83E-05	2.26E-05	1.20E-05	2.19E-05
B0350.2d.1	597	812	648	929	1.91E-05	2.46E-05	1.35E-05	2.39E-05
B0350.2d.2	521	696	562	788	2.10E-05	2.65E-05	1.47E-05	2.55E-05
B0350.2d.3	475	630	510	720	2.19E-05	2.75E-05	1.53E-05	2.67E-05
B0350.2e.1	944	1262	968	1422	1.85E-05	2.34E-05	1.24E-05	2.24E-05
B0350.2e.2	921	1208	933	1374	1.83E-05	2.26E-05	1.20E-05	2.19E-05
B0350.2f.1	1374	1777	1284	1979	7.08E-06	8.65E-06	4.30E-06	8.19E-06
B0350.2f.2	1340	1724	1264	1926	7.11E-06	8.65E-06	4.35E-06	8.21E-06
B0350.2g.1	435	578	418	649	1.54E-05	1.93E-05	9.60E-06	1.84E-05
B0350.2g.2	404	523	380	607	1.44E-05	1.76E-05	8.82E-06	1.74E-05
B0350.3	2	1	2	1	2.80E-06	2.65E-06	1.82E-06	2.25E-06
B0350.t1	0	0	3	0	2.80E-06	2.65E-06	2.99E-06	2.25E-06
B0353.1	26	43	29	54	5.24E-06	8.17E-06	3.81E-06	8.73E-06
B0361.10	302	392	327	512	2.98E-05	3.66E-05	2.10E-05	4.07E-05
B0361.11	16	30	9	18	2.80E-06	2.65E-06	1.82E-06	2.25E-06
B0361.2a	175	281	173	247	1.33E-05	2.02E-05	8.55E-06	1.50E-05
B0361.2b	39	69	18	27	1.01E-05	1.69E-05	3.04E-06	5.65E-06
B0361.3.1	358	412	280	457	1.97E-05	2.15E-05	1.00E-05	2.02E-05
B0361.3.2	332	391	245	428	1.97E-05	2.19E-05	9.48E-06	2.04E-05
B0361.4	29	35	29	21	2.80E-06	2.94E-06	1.82E-06	2.25E-06
B0361.5a	193	300	297	388	1.86E-05	2.74E-05	1.87E-05	3.01E-05
B0361.5b	253	353	399	500	1.98E-05	2.61E-05	2.03E-05	3.14E-05
B0361.6	332	432	326	403	2.78E-05	3.42E-05	1.78E-05	2.71E-05
B0361.7	230	245	266	356	1.76E-05	1.77E-05	1.32E-05	2.18E-05
B0361.9	85	133	84	103	9.27E-06	1.37E-05	5.96E-06	9.04E-06
B0365.1	1770	1651	2989	3774	5.83E-05	5.14E-05	6.41E-05	9.99E-05
B0365.2	3	6	7	3	2.80E-06	2.65E-06	1.82E-06	2.25E-06
B0365.3.1	6365	7382	8895	11801	2.20E-04	2.41E-04	2.00E-04	3.28E-04
B0365.3.2	6191	7131	8669	11631	2.20E-04	2.39E-04	2.00E-04	3.32E-04
B0365.5a	89	116	24	31	8.20E-06	1.01E-05	1.82E-06	2.29E-06
B0365.5b	102	122	32	26	1.39E-05	1.58E-05	2.84E-06	2.86E-06
B0365.6.1	601	599	1395	1198	3.44E-05	3.24E-05	5.19E-05	5.51E-05
B0365.6.2	601	599	1393	1197	3.49E-05	3.29E-05	5.26E-05	5.58E-05
B0365.6.3	601	599	1400	1200	3.44E-05	3.24E-05	5.21E-05	5.51E-05
B0365.7	42	57	57	45	2.80E-06	2.65E-06	1.82E-06	2.25E-06
B0379.1	130	153	136	128	1.92E-05	2.13E-05	1.31E-05	1.52E-05
B0379.2	21	55	15	14	3.44E-06	8.54E-06	1.82E-06	2.25E-06
B0379.3a	1104	833	1296	2026	3.99E-05	2.85E-05	3.05E-05	5.89E-05
B0379.3b	1219	916	1452	2190	4.17E-05	2.96E-05	3.24E-05	6.03E-05
B0379.4a	446	442	516	793	4.45E-05	4.17E-05	3.35E-05	6.36E-05
B0379.4b	387	398	454	742	2.83E-05	2.75E-05	2.16E-05	4.35E-05
B0379.6	7	10	4	6	2.80E-06	2.65E-06	1.82E-06	2.25E-06
B0379.7.1	63	115	42	29	5.29E-06	9.13E-06	2.30E-06	2.25E-06
B0379.7.2	56	105	36	28	4.90E-06	8.68E-06	2.06E-06	2.25E-06
B0391.1	2	4	1	2	2.80E-06	2.65E-06	1.82E-06	2.25E-06
B0391.10	26	35	29	42	1.04E-05	1.33E-05	7.58E-06	1.35E-05
B0391.11	10	3	2	4	2.80E-06	2.65E-06	1.82E-06	2.25E-06
B0391.12	6	5	13	2	2.80E-06	2.65E-06	1.82E-06	2.25E-06
B0391.2	6	2	1	0	2.80E-06	2.65E-06	1.82E-06	2.25E-06
B0391.3	3	3	3	4	2.80E-06	2.65E-06	1.82E-06	2.25E-06
B0391.4	4	6	2	2	2.80E-06	2.65E-06	1.82E-06	2.25E-06
B0391.5	15	29	20	46	2.80E-06	2.70E-06	1.82E-06	3.64E-06
B0391.6	1	4	3	3	2.80E-06	2.65E-06	1.82E-06	2.25E-06
B0391.8	21	23	28	27	2.80E-06	2.65E-06	1.82E-06	2.25E-06
B0391.9	1	1	2	0	2.80E-06	2.65E-06	1.82E-06	2.25E-06
B0393.1.1	16005	16606	21826	18524	2.02E-03	1.98E-03	1.79E-03	1.88E-03
B0393.1.2	14370	14944	19688	17878	1.93E-03	1.89E-03	1.72E-03	1.92E-03
B0393.2.1	826	887	1471	1784	4.37E-05	4.44E-05	5.07E-05	7.59E-05

B0393.2.2	744	789	1324	1643	4.66E-05	4.67E-05	5.39E-05	8.26E-05
B0393.3	896	861	1649	1939	4.82E-05	4.37E-05	5.77E-05	8.37E-05
B0393.4	59	85	42	18	2.80E-06	3.54E-06	1.82E-06	2.25E-06
B0393.5	108	137	63	84	3.44E-06	4.13E-06	1.82E-06	2.25E-06
B0393.6	539	472	769	971	4.96E-05	4.10E-05	4.60E-05	7.17E-05
B0393.7	1	2	3	3	2.80E-06	2.65E-06	1.82E-06	2.25E-06
B0393.8	111	109	238	108	3.09E-05	2.87E-05	4.31E-05	2.42E-05
B0395.1	26	53	38	60	2.80E-06	3.73E-06	1.84E-06	3.58E-06
B0395.2	69	78	48	52	5.43E-06	5.82E-06	2.46E-06	3.28E-06
B0395.3.1	385	444	320	590	2.07E-05	2.26E-05	1.12E-05	2.55E-05
B0395.3.2	343	390	292	544	2.07E-05	2.23E-05	1.15E-05	2.64E-05
B0399.1a	23	35	38	34	2.80E-06	2.65E-06	1.82E-06	2.25E-06
B0399.1b	32	45	42	46	2.80E-06	2.65E-06	1.82E-06	2.25E-06
B0399.1c	20	27	34	30	2.80E-06	2.65E-06	1.82E-06	2.25E-06
B0399.1d	25	29	34	32	2.80E-06	2.65E-06	1.82E-06	2.25E-06
B0399.1e	22	29	36	32	2.80E-06	2.65E-06	1.82E-06	2.25E-06
B0399.2	18	39	18	17	2.80E-06	2.65E-06	1.82E-06	2.25E-06
B0399.t1	1	0	0	0	2.80E-06	2.65E-06	1.82E-06	2.25E-06
B0399.t11	0	0	1	0	2.80E-06	2.65E-06	1.82E-06	2.25E-06
B0399.t15	0	0	1	0	2.80E-06	2.65E-06	1.82E-06	2.25E-06
B0399.t2	0	0	1	0	2.80E-06	2.65E-06	1.82E-06	2.25E-06
B0399.t7	0	0	1	0	2.80E-06	2.65E-06	1.82E-06	2.25E-06
B0399.t8	1	0	0	0	2.80E-06	2.65E-06	1.82E-06	2.25E-06
B0399.t9	1	0	0	0	2.80E-06	2.65E-06	1.82E-06	2.25E-06
B0403.2	27	31	10	18	2.80E-06	2.65E-06	1.82E-06	2.25E-06
B0403.3	30	33	28	36	4.14E-06	4.29E-06	2.51E-06	3.98E-06
B0403.4	1225	1278	1491	1369	8.37E-05	8.24E-05	6.63E-05	7.51E-05
B0403.5	56	130	38	51	5.04E-06	1.11E-05	2.22E-06	3.69E-06
B0403.6	12	24	17	17	2.80E-06	3.81E-06	1.86E-06	2.29E-06
B0410.1	0	0	2	0	2.80E-06	2.65E-06	1.82E-06	2.25E-06
B0410.2a	45	49	84	109	3.14E-06	3.23E-06	3.81E-06	6.12E-06
B0410.2b	48	56	88	113	2.83E-06	3.12E-06	3.39E-06	5.38E-06
B0410.3	79	227	72	125	1.26E-05	3.42E-05	7.47E-06	1.60E-05
B0412.1a	14	30	15	8	2.80E-06	2.65E-06	1.82E-06	2.25E-06
B0412.1b	10	29	8	6	2.80E-06	2.65E-06	1.82E-06	2.25E-06
B0412.2	31	44	45	27	2.80E-06	2.83E-06	1.99E-06	2.25E-06
B0412.3.1	506	535	691	1030	1.66E-05	1.66E-05	1.48E-05	2.72E-05
B0412.4.1	3518	3099	9894	2206	1.23E-03	1.02E-03	2.25E-03	6.18E-04
B0412.4.2	1809	1533	4433	1280	7.34E-04	5.88E-04	1.17E-03	4.17E-04
B0412.6	1	0	0	0	2.80E-06	2.65E-06	1.82E-06	2.25E-06
B0414.1	7	7	5	5	2.80E-06	2.65E-06	1.82E-06	2.25E-06
B0414.2	10	18	18	13	2.80E-06	2.65E-06	1.82E-06	2.25E-06
B0414.3	1489	1220	2796	2285	1.93E-04	1.49E-04	2.36E-04	2.38E-04
B0414.5	1584	1413	2072	2991	6.35E-05	5.35E-05	5.40E-05	9.63E-05
B0414.6	326	297	481	671	1.46E-05	1.26E-05	1.41E-05	2.42E-05
B0414.7a	695	725	887	1416	1.76E-05	1.74E-05	1.46E-05	2.89E-05
B0414.7b	685	714	874	1404	1.76E-05	1.74E-05	1.46E-05	2.90E-05
B0414.8a	460	491	561	815	2.33E-05	2.35E-05	1.85E-05	3.32E-05
B0414.8b	475	499	583	833	2.33E-05	2.31E-05	1.86E-05	3.28E-05
B0416.1	71	95	52	99	2.80E-06	2.65E-06	1.82E-06	2.32E-06
B0416.2	4	2	14	4	2.80E-06	2.65E-06	2.02E-06	2.25E-06
B0416.3	8	19	6	8	2.80E-06	2.91E-06	1.82E-06	2.25E-06
B0416.4	22	44	26	14	3.75E-06	7.09E-06	2.88E-06	2.25E-06
B0416.5b	475	702	499	886	3.81E-05	5.32E-05	2.61E-05	5.71E-05
B0416.6	107	211	119	164	7.03E-06	1.31E-05	5.08E-06	8.66E-06
B0416.7a	26	30	16	8	7.31E-06	7.96E-06	2.92E-06	2.25E-06
B0416.7b	1	7	11	3	2.80E-06	3.86E-06	4.17E-06	2.25E-06
B0432.1	5	8	7	6	2.80E-06	2.65E-06	1.82E-06	2.25E-06
B0432.11	15	13	5	5	2.94E-06	2.65E-06	1.82E-06	2.25E-06

B0432.12	9	11	5	2	2.80E-06	2.65E-06	1.82E-06	2.25E-06
B0432.14	11	14	9	5	4.06E-06	4.89E-06	2.17E-06	2.25E-06
B0432.2	167	230	214	246	2.88E-05	3.74E-05	2.40E-05	3.41E-05
B0432.3	151	164	168	215	2.70E-05	2.77E-05	1.96E-05	3.09E-05
B0432.4	359	444	351	473	3.63E-05	4.23E-05	2.31E-05	3.84E-05
B0432.5a	7	8	1	6	2.80E-06	2.65E-06	1.82E-06	2.25E-06
B0432.5b	5	6	0	7	2.80E-06	2.65E-06	1.82E-06	2.25E-06
B0432.6	41	31	89	123	4.65E-06	3.31E-06	6.56E-06	1.12E-05
B0432.7	93	59	140	214	7.36E-06	4.42E-06	7.20E-06	1.36E-05
B0432.8	78	75	88	165	6.78E-06	6.16E-06	4.99E-06	1.15E-05
B0432.9	6	6	16	12	2.80E-06	2.65E-06	1.82E-06	2.25E-06
B0454.1	41	29	64	79	3.67E-06	2.65E-06	3.72E-06	5.67E-06
B0454.10	4	5	3	2	2.80E-06	2.65E-06	1.82E-06	2.25E-06
B0454.2	2	7	7	8	2.80E-06	2.65E-06	1.82E-06	2.25E-06
B0454.3	3	2	4	1	2.80E-06	2.65E-06	1.82E-06	2.25E-06
B0454.4	0	2	0	1	2.80E-06	2.65E-06	1.82E-06	2.25E-06
B0454.5	40	40	55	35	7.45E-06	7.04E-06	6.67E-06	5.24E-06
B0454.6	51	78	40	62	2.88E-06	4.18E-06	1.82E-06	2.83E-06
B0454.7	4	5	1	2	2.80E-06	2.65E-06	1.82E-06	2.25E-06
B0454.8	11	40	4	6	2.80E-06	2.65E-06	1.82E-06	2.25E-06
B0454.9	23	33	44	57	2.80E-06	3.31E-06	3.04E-06	4.88E-06
B0457.1a	1320	1261	2190	2797	4.08E-05	3.68E-05	4.41E-05	6.95E-05
B0457.1b	1274	1210	2117	2733	3.96E-05	3.56E-05	4.29E-05	6.83E-05
B0457.1c	131	124	176	233	3.06E-05	2.73E-05	2.67E-05	4.37E-05
B0457.2	63	101	52	60	6.19E-06	9.39E-06	3.33E-06	4.75E-06
B0457.3	7	14	9	9	2.80E-06	2.65E-06	1.82E-06	2.25E-06
B0457.4	5	6	5	4	2.80E-06	2.65E-06	1.82E-06	2.25E-06
B0457.6	42	74	76	27	9.44E-06	1.57E-05	1.11E-05	4.88E-06
B0462.1	4	9	7	4	2.80E-06	2.65E-06	1.82E-06	2.25E-06
B0462.2	0	6	4	1	2.80E-06	2.65E-06	1.82E-06	2.25E-06
B0462.3	6	12	3	6	2.80E-06	2.65E-06	1.82E-06	2.25E-06
B0462.4	1	5	2	2	2.80E-06	2.65E-06	1.82E-06	2.25E-06
B0464.1.1	1613	2018	2167	2414	1.02E-04	1.21E-04	8.93E-05	1.23E-04
B0464.1.2	1472	1878	1927	2248	1.03E-04	1.24E-04	8.77E-05	1.26E-04
B0464.2	986	1015	1076	1715	3.03E-05	2.95E-05	2.15E-05	4.23E-05
B0464.3.1	920	740	806	206	2.95E-04	2.24E-04	1.68E-04	5.31E-05
B0464.3.2	667	576	615	188	2.10E-04	1.71E-04	1.26E-04	4.75E-05
B0464.4.1	702	802	785	1099	3.72E-05	4.01E-05	2.71E-05	4.67E-05
B0464.4.2	448	500	475	686	3.64E-05	3.84E-05	2.51E-05	4.48E-05
B0464.5a	1011	1005	1018	1999	3.39E-05	3.18E-05	2.22E-05	5.38E-05
B0464.5b.1	1710	1506	1721	2758	6.60E-05	5.49E-05	4.32E-05	8.55E-05
B0464.5b.2	1104	1042	1051	2035	5.82E-05	5.19E-05	3.60E-05	8.62E-05
B0464.5c.1	1664	1470	1691	2754	6.08E-05	5.07E-05	4.02E-05	8.08E-05
B0464.5c.2	1207	1127	1174	2208	5.14E-05	4.53E-05	3.25E-05	7.55E-05
B0464.6.1	999	833	1227	1545	5.40E-05	4.25E-05	4.31E-05	6.70E-05
B0464.6.2	888	775	1126	1483	5.13E-05	4.23E-05	4.23E-05	6.88E-05
B0464.6.3	917	811	1206	1525	4.82E-05	4.02E-05	4.12E-05	6.43E-05
B0464.7.1	570	710	657	573	1.15E-04	1.35E-04	8.63E-05	9.29E-05
B0464.7.2	349	459	380	362	8.41E-05	1.04E-04	5.96E-05	7.00E-05
B0464.8	228	256	273	368	2.41E-05	2.56E-05	1.88E-05	3.13E-05
B0464.9	172	193	353	475	1.76E-05	1.86E-05	2.35E-05	3.90E-05
B0478.1a	18	28	20	23	2.80E-06	2.65E-06	1.82E-06	2.25E-06
B0478.1b.1	13	22	17	15	2.80E-06	2.65E-06	1.82E-06	2.25E-06
B0478.1b.2	15	22	17	17	2.80E-06	2.65E-06	1.82E-06	2.25E-06
B0478.1b.3	18	28	20	23	2.80E-06	2.65E-06	1.82E-06	2.25E-06
B0478.1b.4	15	22	16	17	2.80E-06	2.65E-06	1.82E-06	2.25E-06
B0478.3	6	6	5	3	2.80E-06	2.65E-06	1.82E-06	2.25E-06
B0491.1	235	191	329	382	2.10E-05	1.61E-05	1.91E-05	2.74E-05
B0491.2.1	889	1533	439	212	9.84E-05	1.60E-04	3.16E-05	1.88E-05

B0491.2.2	775	1318	365	207	8.63E-05	1.39E-04	2.64E-05	1.85E-05
B0491.3	88	134	53	27	1.24E-05	1.79E-05	4.87E-06	3.06E-06
B0491.4	8	14	18	14	2.80E-06	2.65E-06	1.82E-06	2.25E-06
B0491.5.1	1116	1116	1294	1585	1.10E-04	1.03E-04	8.27E-05	1.25E-04
B0491.5.2	1088	1098	1240	1509	1.08E-04	1.03E-04	8.03E-05	1.21E-04
B0491.5.3	1121	1060	1256	1436	1.27E-04	1.13E-04	9.26E-05	1.31E-04
B0491.7	67	52	86	73	9.10E-06	6.67E-06	7.60E-06	7.96E-06
B0491.8a	17	46	27	25	2.80E-06	2.65E-06	1.82E-06	2.25E-06
B0491.8b	11	38	21	25	2.80E-06	2.65E-06	1.82E-06	2.25E-06
B0491.8c	17	47	28	26	2.80E-06	2.65E-06	1.82E-06	2.25E-06
B0491.t1	0	0	1	0	2.80E-06	2.65E-06	1.82E-06	2.25E-06
B0491.t2	0	0	1	0	2.80E-06	2.65E-06	1.82E-06	2.25E-06
B0495.1	11	10	4	2	2.80E-06	2.65E-06	1.82E-06	2.25E-06
B0495.10a	70	133	62	79	3.28E-06	5.87E-06	1.88E-06	2.97E-06
B0495.10b	54	114	52	77	2.80E-06	5.34E-06	1.82E-06	3.06E-06
B0495.10c	68	129	62	77	3.11E-06	5.58E-06	1.86E-06	2.83E-06
B0495.2	546	622	471	1119	2.73E-05	2.94E-05	1.53E-05	4.49E-05
B0495.4	376	625	384	558	2.06E-05	3.23E-05	1.36E-05	2.45E-05
B0495.5.1	172	231	187	320	8.46E-06	1.07E-05	5.98E-06	1.26E-05
B0495.5.2	161	223	175	305	8.20E-06	1.07E-05	5.79E-06	1.25E-05
B0495.6	122	134	615	127	5.17E-05	5.37E-05	1.70E-04	4.33E-05
B0495.7.1	1276	1466	1441	2014	4.77E-05	5.18E-05	3.51E-05	6.05E-05
B0495.7.2	1256	1442	1420	1985	4.92E-05	5.34E-05	3.62E-05	6.25E-05
B0495.8a	207	250	262	300	2.19E-05	2.50E-05	1.81E-05	2.55E-05
B0495.8b	10	19	13	20	3.05E-06	5.48E-06	2.59E-06	4.90E-06
B0495.9	189	235	233	154	2.33E-05	2.73E-05	1.87E-05	1.52E-05
B0496.1	11	13	5	4	2.80E-06	2.65E-06	1.82E-06	2.25E-06
B0496.2	7	13	22	11	2.80E-06	2.65E-06	1.82E-06	2.25E-06
B0496.3a	244	361	149	357	5.88E-06	8.20E-06	2.33E-06	6.90E-06
B0496.3b	270	401	172	383	5.96E-06	8.38E-06	2.48E-06	6.79E-06
B0496.3c	82	136	44	109	6.83E-06	1.07E-05	2.39E-06	7.31E-06
B0496.4	2	6	3	2	2.80E-06	2.65E-06	1.82E-06	2.25E-06
B0496.5	10	7	9	1	2.80E-06	2.65E-06	1.82E-06	2.25E-06
B0496.6	11	14	16	12	2.80E-06	2.65E-06	1.82E-06	2.25E-06
B0496.7	35	38	45	15	1.54E-05	1.58E-05	1.29E-05	5.29E-06
B0496.8	53	110	49	75	3.58E-06	7.01E-06	2.15E-06	4.07E-06
B0507.1	42	55	23	32	2.80E-06	2.94E-06	1.82E-06	2.25E-06
B0507.10	14	26	14	22	2.80E-06	2.65E-06	1.82E-06	2.25E-06
B0507.11	4	1	0	1	2.80E-06	2.65E-06	1.82E-06	2.25E-06
B0507.2	65	144	50	124	5.15E-06	1.08E-05	2.59E-06	7.92E-06
B0507.3a	44	38	31	30	3.42E-06	2.78E-06	1.82E-06	2.25E-06
B0507.3b	39	35	28	26	4.56E-06	3.86E-06	2.13E-06	2.45E-06
B0507.4	2	3	3	3	2.80E-06	2.65E-06	1.82E-06	2.25E-06
B0507.5	7	5	7	4	2.80E-06	2.65E-06	1.82E-06	2.25E-06
B0507.6	33	20	41	44	2.80E-06	2.65E-06	1.82E-06	2.25E-06
B0507.7	14	10	26	12	2.80E-06	2.65E-06	1.82E-06	2.25E-06
B0507.8	9	12	12	10	2.80E-06	2.65E-06	1.82E-06	2.25E-06
B0507.9	6	9	10	5	2.80E-06	2.65E-06	1.82E-06	2.25E-06
B0511.1	29	43	14	19	3.39E-06	4.73E-06	1.82E-06	2.25E-06
B0511.10	396	474	610	852	2.77E-05	3.13E-05	2.78E-05	4.78E-05
B0511.11	32	53	30	29	3.14E-06	4.92E-06	1.91E-06	2.29E-06
B0511.12	1084	1034	1560	2312	2.41E-05	2.17E-05	2.25E-05	4.12E-05
B0511.13	228	223	240	261	1.78E-05	1.65E-05	1.22E-05	1.64E-05
B0511.14a	215	225	351	446	1.26E-05	1.25E-05	1.34E-05	2.10E-05
B0511.14b	168	176	269	339	1.33E-05	1.32E-05	1.39E-05	2.16E-05
B0511.2	44	37	105	73	3.58E-06	2.83E-06	5.56E-06	4.77E-06
B0511.3	11	21	3	3	2.80E-06	2.83E-06	1.82E-06	2.25E-06
B0511.4	11	36	8	7	2.80E-06	3.44E-06	1.82E-06	2.25E-06
B0511.5	18	28	8	6	2.97E-06	4.36E-06	1.82E-06	2.25E-06

B0511.6.1	967	1085	1127	1551	4.52E-05	4.80E-05	3.43E-05	5.83E-05
B0511.6.2	796	906	868	1365	5.43E-05	5.84E-05	3.85E-05	7.48E-05
B0511.7	203	267	343	426	2.49E-05	3.10E-05	2.74E-05	4.20E-05
B0511.8.1	596	619	579	570	3.97E-05	3.90E-05	2.51E-05	3.05E-05
B0511.8.2	501	540	485	506	3.55E-05	3.61E-05	2.23E-05	2.88E-05
B0511.9a	304	205	493	541	4.12E-05	2.63E-05	4.35E-05	5.89E-05
B0511.9b	225	153	340	399	4.07E-05	2.62E-05	4.00E-05	5.80E-05
B0513.1	573	639	524	821	1.77E-05	1.86E-05	1.05E-05	2.04E-05
B0513.4	298	248	703	458	7.93E-05	6.23E-05	1.22E-04	9.79E-05
B0513.5	159	272	147	196	8.57E-06	1.39E-05	5.16E-06	8.50E-06
B0513.6	6	20	15	12	2.80E-06	2.65E-06	1.82E-06	2.25E-06
B0513.7	81	94	46	63	7.67E-06	8.41E-06	2.82E-06	4.79E-06
B0513.8	7	6	12	5	2.80E-06	2.65E-06	1.82E-06	2.25E-06
B0513.9a	30	37	38	23	9.58E-06	1.12E-05	7.89E-06	5.89E-06
B0513.9b.1	34	49	44	29	8.26E-06	1.12E-05	6.96E-06	5.67E-06
B0513.9b.2	30	37	38	23	9.72E-06	1.13E-05	8.00E-06	5.98E-06
B0523.1	16	21	14	7	2.80E-06	2.65E-06	1.82E-06	2.25E-06
B0523.3	236	313	283	376	1.55E-05	1.94E-05	1.21E-05	1.98E-05
B0523.5	800	757	963	1395	1.94E-05	1.73E-05	1.52E-05	2.71E-05
B0524.1	57	60	48	130	3.98E-06	3.97E-06	2.19E-06	7.29E-06
B0524.2	1	11	3	2	2.80E-06	2.65E-06	1.82E-06	2.25E-06
B0524.3	1	4	4	3	2.80E-06	2.65E-06	1.82E-06	2.25E-06
B0524.4	11	27	14	7	2.80E-06	2.65E-06	1.82E-06	2.25E-06
B0524.5	9	14	7	7	2.80E-06	2.65E-06	1.82E-06	2.25E-06
B0524.6	147	112	232	313	2.00E-05	1.44E-05	2.05E-05	3.41E-05
B0524.7	73	85	110	160	4.73E-06	5.18E-06	4.63E-06	8.32E-06
B0545.1a	108	126	126	126	5.63E-06	6.19E-06	4.26E-06	5.26E-06
B0545.1b.1	133	163	170	174	6.16E-06	7.12E-06	5.12E-06	6.45E-06
B0545.1b.2	142	169	173	175	6.02E-06	6.74E-06	4.77E-06	5.96E-06
B0545.3	6	14	1	4	2.80E-06	2.65E-06	1.82E-06	2.25E-06
B0545.4	11	12	7	8	2.80E-06	2.65E-06	1.82E-06	2.25E-06
B0546.1.1	583	875	1952	652	1.39E-04	1.97E-04	3.03E-04	1.25E-04
B0546.1.2	499	763	1490	590	1.17E-04	1.69E-04	2.28E-04	1.11E-04
B0546.2.1	346	300	422	502	2.44E-05	1.99E-05	1.93E-05	2.84E-05
B0546.2.2	282	257	339	440	2.34E-05	2.01E-05	1.83E-05	2.93E-05
B0546.3	80	78	140	152	6.36E-06	5.87E-06	7.25E-06	9.72E-06
B0546.4a	85	76	69	115	1.29E-05	1.09E-05	6.81E-06	1.40E-05
B0546.4b	80	77	107	133	8.09E-06	7.35E-06	7.05E-06	1.08E-05
B0546.5	4	5	8	6	2.80E-06	2.65E-06	1.82E-06	2.25E-06
B0547.1.1	437	439	509	667	4.14E-05	3.93E-05	3.14E-05	5.08E-05
B0547.1.2	388	394	420	597	3.91E-05	3.76E-05	2.76E-05	4.84E-05
B0547.2	1	1	0	1	2.80E-06	2.65E-06	1.82E-06	2.25E-06
B0547.3	21	40	18	20	2.80E-06	4.36E-06	1.82E-06	2.25E-06
B0547.4	8	11	14	8	2.80E-06	2.65E-06	1.82E-06	2.25E-06
B0547.5	5	2	7	3	2.80E-06	2.65E-06	1.82E-06	2.25E-06
B0554.1	1	0	4	1	2.80E-06	2.65E-06	1.82E-06	2.25E-06
B0554.2	1	5	6	2	2.80E-06	2.65E-06	1.82E-06	2.25E-06
B0554.3	0	1	1	0	2.80E-06	2.65E-06	1.82E-06	2.25E-06
B0554.4	3	4	4	1	2.80E-06	2.65E-06	1.82E-06	2.25E-06
B0554.5	10	13	15	8	2.80E-06	2.65E-06	1.82E-06	2.25E-06
B0554.6	15	27	24	16	2.80E-06	2.65E-06	1.82E-06	2.25E-06
B0554.7	5	4	5	0	2.80E-06	2.65E-06	1.82E-06	2.25E-06
B0563.1	2	3	1	1	2.80E-06	2.65E-06	1.82E-06	2.25E-06
B0563.2	23	34	38	24	2.80E-06	3.78E-06	2.90E-06	2.27E-06
B0563.4	290	362	303	432	3.02E-05	3.56E-05	2.05E-05	3.61E-05
B0563.5	6	2	11	2	2.80E-06	2.65E-06	2.33E-06	2.25E-06
B0563.6a	25	43	41	37	2.80E-06	2.65E-06	1.82E-06	2.25E-06
B0563.6b.1	18	33	22	30	2.80E-06	2.65E-06	1.82E-06	2.25E-06
B0563.6b.2	15	28	13	18	2.80E-06	2.65E-06	1.82E-06	2.25E-06

B0563.7	9	11	5	9	2.80E-06	2.65E-06	1.82E-06	2.25E-06
B0563.8	0	1	3	1	2.80E-06	2.65E-06	1.82E-06	2.25E-06
B0563.9	0	3	0	1	2.80E-06	2.65E-06	1.82E-06	2.25E-06
B0564.10a	11	12	6	11	2.80E-06	2.65E-06	1.82E-06	2.25E-06
B0564.10b	6	11	7	9	2.80E-06	2.65E-06	1.82E-06	2.25E-06
B0564.11	188	175	184	339	1.73E-05	1.53E-05	1.10E-05	2.51E-05
B0564.1a	548	512	827	452	5.19E-05	4.58E-05	5.10E-05	3.44E-05
B0564.2	43	59	105	57	9.97E-06	1.29E-05	1.59E-05	1.06E-05
B0564.3.1	8	35	19	11	2.80E-06	2.65E-06	1.82E-06	2.25E-06
B0564.3.2	7	34	19	11	2.80E-06	2.65E-06	1.82E-06	2.25E-06
B0564.4	20	21	19	16	2.80E-06	2.65E-06	1.82E-06	2.25E-06
B0564.6a	44	57	43	58	3.14E-06	3.81E-06	1.99E-06	3.31E-06
B0564.6b	35	48	34	52	3.08E-06	3.99E-06	1.95E-06	3.69E-06
B0564.8	3	1	1	0	2.80E-06	2.65E-06	1.82E-06	2.25E-06
B0564.9	44	57	43	58	3.14E-06	3.81E-06	1.99E-06	3.31E-06
BE0003N10.1	117	100	245	273	5.91E-06	4.76E-06	8.05E-06	1.11E-05
BE0003N10.2	164	145	199	337	1.12E-05	9.39E-06	8.87E-06	1.85E-05
BE0003N10.3	89	32	34	22	2.86E-06	2.65E-06	1.82E-06	2.25E-06
BE10.1	22	36	24	7	2.80E-06	3.81E-06	1.82E-06	2.25E-06
BE10.2	89	171	46	39	6.13E-06	1.11E-05	2.06E-06	2.25E-06
BE10.3	7	8	15	13	2.80E-06	2.65E-06	1.82E-06	2.25E-06
BE10.4	6	6	6	5	2.80E-06	2.65E-06	1.82E-06	2.25E-06
BE10.5	4	2	1	0	2.80E-06	2.65E-06	1.82E-06	2.25E-06
BE10.6	192	157	266	47	1.32E-04	1.02E-04	1.19E-04	2.59E-05
C01A2.1	13	17	33	11	2.80E-06	2.65E-06	1.82E-06	2.25E-06
C01A2.2	21	42	20	23	3.56E-06	6.69E-06	2.20E-06	3.13E-06
C01A2.3	298	385	436	438	2.09E-05	2.55E-05	1.99E-05	2.47E-05
C01A2.4	93	192	131	179	1.50E-05	2.93E-05	1.38E-05	2.32E-05
C01A2.5	226	270	301	345	2.29E-05	2.58E-05	1.98E-05	2.80E-05
C01A2.6	3	6	9	1	2.80E-06	2.65E-06	1.82E-06	2.25E-06
C01A2.7.1	23	37	21	20	3.81E-06	5.82E-06	2.28E-06	2.68E-06
C01A2.7.2	22	36	20	17	4.00E-06	6.16E-06	2.37E-06	2.47E-06
C01B10.1	82	178	62	135	3.33E-06	6.82E-06	1.82E-06	4.41E-06
C01B10.10	49	86	51	67	2.83E-06	4.71E-06	1.91E-06	3.10E-06
C01B10.11	221	461	175	314	2.32E-05	4.57E-05	1.19E-05	2.64E-05
C01B10.3	186	336	121	243	1.69E-05	2.89E-05	7.16E-06	1.77E-05
C01B10.4	27	36	24	31	2.80E-06	2.65E-06	1.82E-06	2.25E-06
C01B10.5a	49	28	97	94	1.22E-05	6.56E-06	1.57E-05	1.88E-05
C01B10.5b.1	47	25	94	92	1.06E-05	5.32E-06	1.38E-05	1.66E-05
C01B10.5b.2	44	22	88	86	1.09E-05	5.13E-06	1.42E-05	1.71E-05
C01B10.5c	47	25	94	94	1.10E-05	5.55E-06	1.44E-05	1.77E-05
C01B10.5e	1	3	3	2	2.80E-06	2.65E-06	1.82E-06	2.25E-06
C01B10.6a.1	1133	1284	1944	2161	8.24E-05	8.82E-05	9.20E-05	1.26E-04
C01B10.6b	1094	1275	1865	2143	7.09E-05	7.81E-05	7.87E-05	1.12E-04
C01B10.7	8	6	9	3	2.80E-06	2.65E-06	1.82E-06	2.25E-06
C01B10.8.1	625	616	686	880	3.25E-05	3.02E-05	2.32E-05	3.67E-05
C01B10.8.2	570	565	626	838	3.23E-05	3.03E-05	2.31E-05	3.82E-05
C01B10.9	398	528	523	672	2.25E-05	2.82E-05	1.92E-05	3.05E-05
C01B12.1	801	1066	515	273	9.17E-05	1.15E-04	3.84E-05	2.51E-05
C01B12.2	138	175	96	120	1.07E-05	1.29E-05	4.85E-06	7.49E-06
C01B12.3	43	64	26	23	2.80E-06	3.41E-06	1.82E-06	2.25E-06
C01B12.4	16	22	17	17	2.80E-06	2.65E-06	1.82E-06	2.25E-06
C01B12.5	16	16	7	11	2.80E-06	2.65E-06	1.82E-06	2.25E-06
C01B12.8	382	476	329	217	3.16E-05	3.72E-05	1.77E-05	1.44E-05
C01B12.9	7	14	12	7	2.80E-06	4.18E-06	2.46E-06	2.25E-06
C01B4.1	4	0	6	3	2.80E-06	2.65E-06	1.82E-06	2.25E-06
C01B4.10	0	5	3	5	2.80E-06	2.65E-06	1.82E-06	2.25E-06
C01B4.2	0	0	1	1	2.80E-06	2.65E-06	1.82E-06	2.25E-06
C01B4.3	2	4	3	4	2.80E-06	2.65E-06	1.82E-06	2.25E-06

C01B4.4	5	5	2	3	2.80E-06	2.65E-06	1.82E-06	2.25E-06
C01B4.5	4	7	4	7	2.80E-06	2.65E-06	1.82E-06	2.25E-06
C01B4.6	39	220	57	278	4.40E-06	2.34E-05	4.19E-06	2.52E-05
C01B4.7	32	240	33	332	2.80E-06	1.78E-05	1.82E-06	2.09E-05
C01B4.8	12	37	11	40	2.80E-06	2.72E-06	1.82E-06	2.50E-06
C01B4.9	499	2123	869	5436	3.26E-05	1.31E-04	3.69E-05	2.85E-04
C01B7.1a.1	153	221	126	245	5.26E-06	7.17E-06	2.82E-06	6.77E-06
C01B7.1a.2	159	226	131	252	5.40E-06	7.27E-06	2.90E-06	6.88E-06
C01B7.1b	81	121	94	141	5.26E-06	7.41E-06	3.97E-06	7.35E-06
C01B7.3	4	5	4	1	2.80E-06	2.65E-06	1.82E-06	2.25E-06
C01B7.4	79	102	38	65	3.75E-06	4.58E-06	1.82E-06	2.47E-06
C01B7.5	1	2	0	2	2.80E-06	2.65E-06	1.82E-06	2.25E-06
C01B7.6	213	226	223	418	2.80E-06	2.65E-06	1.82E-06	3.26E-06
C01B7.7	7	10	4	5	2.80E-06	2.65E-06	1.82E-06	2.25E-06
C01B9.3	2	1	1	0	2.80E-06	2.65E-06	1.82E-06	2.25E-06
C01C10.1	24	34	26	22	2.80E-06	3.76E-06	1.99E-06	2.25E-06
C01C10.2a	17	37	16	35	3.11E-06	6.37E-06	1.90E-06	5.13E-06
C01C10.2b	31	75	25	56	3.53E-06	8.04E-06	1.84E-06	5.11E-06
C01C10.3	62	182	83	154	4.65E-06	1.29E-05	4.05E-06	9.27E-06
C01C10.4	40	65	41	40	3.98E-06	6.11E-06	2.66E-06	3.19E-06
C01C4.1	14	34	36	8	2.80E-06	5.13E-06	3.75E-06	2.25E-06
C01C4.2	5	3	4	4	2.80E-06	2.65E-06	1.82E-06	2.25E-06
C01C4.3a	38	87	36	51	2.88E-06	6.27E-06	1.82E-06	3.13E-06
C01C4.3b	26	72	29	43	2.80E-06	6.22E-06	1.82E-06	3.17E-06
C01C7.1	419	429	543	810	1.31E-05	1.27E-05	1.11E-05	2.04E-05
C01F1.1	335	398	199	444	2.39E-05	2.68E-05	9.24E-06	2.55E-05
C01F1.2	197	217	283	278	2.35E-05	2.44E-05	2.20E-05	2.66E-05
C01F1.3b	40	71	25	32	7.11E-06	1.19E-05	2.90E-06	4.57E-06
C01F1.4	3	16	4	11	2.80E-06	2.65E-06	1.82E-06	2.25E-06
C01F1.5	28	36	24	15	3.39E-06	4.10E-06	1.90E-06	2.25E-06
C01F1.6	499	409	718	846	3.96E-05	3.07E-05	3.71E-05	5.39E-05
C01F4.2a	26	33	32	24	2.80E-06	2.65E-06	1.82E-06	2.25E-06
C01F4.2b	19	24	16	18	2.80E-06	2.65E-06	1.82E-06	2.25E-06
C01F6.1	585	670	883	1143	2.51E-05	2.72E-05	2.47E-05	3.94E-05
C01F6.2	51	97	43	46	3.89E-06	7.01E-06	2.13E-06	2.83E-06
C01F6.3	85	94	110	120	6.86E-06	7.17E-06	5.78E-06	7.78E-06
C01F6.4	292	350	384	428	1.96E-05	2.22E-05	1.68E-05	2.31E-05
C01F6.5	84	127	107	75	1.22E-05	1.74E-05	1.01E-05	8.75E-06
C01F6.6a	942	1433	781	1321	5.69E-05	8.17E-05	3.07E-05	6.41E-05
C01F6.6b	650	949	542	926	3.79E-05	5.22E-05	2.05E-05	4.33E-05
C01F6.6c	815	1248	673	1123	5.30E-05	7.66E-05	2.85E-05	5.86E-05
C01F6.6d	799	1213	659	1110	5.31E-05	7.62E-05	2.85E-05	5.93E-05
C01F6.6e.1	624	916	520	902	6.15E-05	8.53E-05	3.33E-05	7.14E-05
C01F6.6e.2	676	1013	583	1002	5.95E-05	8.42E-05	3.34E-05	7.08E-05
C01F6.6e.3	723	1065	623	1043	5.48E-05	7.62E-05	3.07E-05	6.35E-05
C01F6.6e.4	726	1067	625	1050	5.78E-05	8.03E-05	3.24E-05	6.72E-05
C01F6.6e.5	725	1061	621	1045	5.78E-05	7.99E-05	3.22E-05	6.69E-05
C01F6.8a	313	308	318	373	4.58E-05	4.26E-05	3.03E-05	4.39E-05
C01F6.8b	331	329	397	403	4.58E-05	4.30E-05	3.57E-05	4.48E-05
C01F6.9	807	748	1432	359	2.42E-04	2.12E-04	2.80E-04	8.66E-05
C01G10.1	31	58	28	14	2.80E-06	2.65E-06	1.82E-06	2.25E-06
C01G10.10	63	86	79	105	5.49E-06	7.06E-06	4.48E-06	7.33E-06
C01G10.11a.	544	497	714	986	3.57E-05	3.08E-05	3.05E-05	5.20E-05
C01G10.11a.	551	510	718	995	5.39E-05	4.71E-05	4.57E-05	7.82E-05
C01G10.11b.	556	506	745	989	2.41E-05	2.07E-05	2.10E-05	3.44E-05
C01G10.11b.	348	345	428	625	3.49E-05	3.26E-05	2.79E-05	5.03E-05
C01G10.12	60	53	52	34	1.09E-05	9.13E-06	6.16E-06	4.97E-06
C01G10.13	0	0	0	1	2.80E-06	2.65E-06	1.82E-06	2.25E-06
C01G10.14	25	37	19	5	3.22E-06	4.50E-06	1.82E-06	2.25E-06

C01G10.15	20	18	39	7	7.39E-06	6.30E-06	9.38E-06	2.25E-06
C01G10.16	1	1	0	0	2.80E-06	2.65E-06	1.82E-06	2.25E-06
C01G10.17	6	13	7	6	2.94E-06	6.03E-06	2.24E-06	2.36E-06
C01G10.2	5	7	5	3	2.80E-06	2.65E-06	1.82E-06	2.25E-06
C01G10.3	6	8	5	10	2.80E-06	2.65E-06	1.82E-06	2.25E-06
C01G10.4	10	9	10	3	5.12E-06	4.34E-06	3.33E-06	2.25E-06
C01G10.5	9	4	13	4	2.80E-06	2.65E-06	2.28E-06	2.25E-06
C01G10.6	19	20	48	5	7.87E-06	7.83E-06	1.30E-05	2.25E-06
C01G10.7	150	194	247	228	1.39E-05	1.69E-05	1.49E-05	1.69E-05
C01G10.8.1	1050	1366	1252	1247	9.97E-05	1.22E-04	7.73E-05	9.51E-05
C01G10.8.2	940	1196	1083	1156	1.01E-04	1.22E-04	7.60E-05	1.00E-04
C01G10.9	43	82	100	97	4.20E-06	7.54E-06	6.34E-06	7.60E-06
C01G12.1	58	79	70	60	3.84E-06	4.92E-06	3.01E-06	3.19E-06
C01G12.10	8	16	13	3	3.72E-06	7.06E-06	3.95E-06	2.25E-06
C01G12.11	10	6	3	1	4.68E-06	2.65E-06	1.82E-06	2.25E-06
C01G12.12	65	91	116	95	5.54E-06	7.35E-06	6.45E-06	6.52E-06
C01G12.13	12	15	8	8	2.80E-06	2.65E-06	1.82E-06	2.25E-06
C01G12.2	15	7	6	5	7.00E-06	3.09E-06	1.82E-06	2.25E-06
C01G12.3	16	47	18	17	3.11E-06	8.62E-06	2.28E-06	2.65E-06
C01G12.4	30	20	68	22	2.80E-06	2.65E-06	2.44E-06	2.25E-06
C01G12.5	6	2	1	1	2.80E-06	2.65E-06	1.82E-06	2.25E-06
C01G12.6	15	8	7	3	7.00E-06	3.52E-06	2.13E-06	2.25E-06
C01G12.7	10	16	7	4	2.80E-06	2.65E-06	1.82E-06	2.25E-06
C01G12.8	158	237	140	89	5.60E-06	7.96E-06	3.24E-06	2.54E-06
C01G12.9	7	14	8	6	2.80E-06	2.65E-06	1.82E-06	2.25E-06
C01G5.2	803	1032	474	1230	3.50E-05	4.25E-05	1.34E-05	4.31E-05
C01G5.3	54	58	40	52	4.20E-06	4.26E-06	2.02E-06	3.24E-06
C01G5.4	37	53	21	18	2.80E-06	2.65E-06	1.82E-06	2.25E-06
C01G5.5	93	99	75	133	1.21E-05	1.21E-05	6.32E-06	1.39E-05
C01G5.6	477	528	703	988	4.16E-05	4.35E-05	3.99E-05	6.92E-05
C01G5.7	6	3	1	3	2.80E-06	2.65E-06	1.82E-06	2.25E-06
C01G5.8	288	309	340	556	1.21E-05	1.23E-05	9.33E-06	1.88E-05
C01G5.9	15	13	8	8	2.80E-06	2.65E-06	1.82E-06	2.25E-06
C01G6.10	19	20	33	13	2.80E-06	2.65E-06	1.82E-06	2.25E-06
C01G6.1a.1	561	569	358	720	5.28E-05	5.06E-05	2.19E-05	5.45E-05
C01G6.1a.2	563	566	358	721	5.28E-05	5.01E-05	2.18E-05	5.43E-05
C01G6.1b.1	602	621	395	733	5.45E-05	5.31E-05	2.33E-05	5.33E-05
C01G6.1b.2	405	416	268	553	5.25E-05	5.09E-05	2.26E-05	5.76E-05
C01G6.2	11	20	10	4	2.80E-06	3.15E-06	1.82E-06	2.25E-06
C01G6.3	378	469	633	703	3.31E-05	3.88E-05	3.61E-05	4.95E-05
C01G6.4	249	258	333	393	2.91E-05	2.85E-05	2.53E-05	3.69E-05
C01G6.5	1653	1784	1987	2955	4.96E-05	5.06E-05	3.88E-05	7.12E-05
C01G6.6a.1	519	1325	487	1104	2.65E-05	6.39E-05	1.62E-05	4.53E-05
C01G6.6a.3	470	1228	472	1072	2.56E-05	6.33E-05	1.68E-05	4.70E-05
C01G6.6b	138	324	143	363	2.61E-05	5.78E-05	1.76E-05	5.51E-05
C01G6.7	140	169	153	135	9.32E-06	1.06E-05	6.63E-06	7.22E-06
C01G6.8a	409	495	607	834	1.40E-05	1.60E-05	1.35E-05	2.29E-05
C01G6.8b	409	495	607	834	1.40E-05	1.60E-05	1.35E-05	2.29E-05
C01G6.8c	409	495	607	834	1.40E-05	1.60E-05	1.35E-05	2.29E-05
C01G6.9	14	35	10	20	2.80E-06	3.36E-06	1.82E-06	2.25E-06
C01G8.1	1499	1188	2588	2495	1.39E-04	1.04E-04	1.56E-04	1.85E-04
C01G8.2.1	52	74	77	72	4.20E-06	5.63E-06	4.03E-06	4.66E-06
C01G8.2.2	52	73	76	72	4.42E-06	5.87E-06	4.21E-06	4.93E-06
C01G8.3	241	336	291	426	2.25E-05	2.96E-05	1.77E-05	3.19E-05
C01G8.4	69	103	71	85	8.09E-06	1.14E-05	5.41E-06	8.01E-06
C01G8.5a.1	2869	2919	2433	3737	1.38E-04	1.33E-04	7.63E-05	1.45E-04
C01G8.5a.2	2840	2882	2378	3678	1.37E-04	1.31E-04	7.46E-05	1.42E-04
C01G8.5b	2806	2851	2376	3688	1.37E-04	1.32E-04	7.56E-05	1.45E-04
C01G8.6	381	435	313	472	3.40E-05	3.67E-05	1.82E-05	3.39E-05

C01G8.9a	1238	1286	2079	3050	2.33E-05	2.28E-05	2.54E-05	4.60E-05
C01G8.9b	415	507	925	1349	1.97E-05	2.27E-05	2.85E-05	5.13E-05
C01H6.1	14	15	5	6	2.80E-06	2.65E-06	1.82E-06	2.25E-06
C01H6.2	43	73	25	35	2.80E-06	3.60E-06	1.82E-06	2.25E-06
C01H6.3	4	10	2	2	2.80E-06	2.65E-06	1.82E-06	2.25E-06
C01H6.4	31	72	36	48	2.80E-06	5.98E-06	2.06E-06	3.40E-06
C01H6.5a	409	358	375	588	1.64E-05	1.35E-05	9.77E-06	1.89E-05
C01H6.5c	347	305	311	486	2.53E-05	2.10E-05	1.48E-05	2.85E-05
C01H6.5d	396	350	383	613	2.92E-05	2.44E-05	1.84E-05	3.63E-05
C01H6.6	24	31	44	65	2.80E-06	2.65E-06	2.37E-06	4.30E-06
C01H6.7	611	679	848	1203	3.14E-05	3.30E-05	2.84E-05	4.97E-05
C01H6.9	186	264	248	440	7.17E-06	9.63E-06	6.23E-06	1.36E-05
C02A12.1	2	6	7	5	2.80E-06	2.65E-06	1.82E-06	2.25E-06
C02A12.10	3	8	3	1	2.80E-06	2.65E-06	1.82E-06	2.25E-06
C02A12.2	3	2	4	0	2.80E-06	2.65E-06	1.82E-06	2.25E-06
C02A12.3	8	7	5	9	2.80E-06	2.65E-06	1.82E-06	2.25E-06
C02A12.5	2	3	3	2	2.80E-06	2.65E-06	1.82E-06	2.25E-06
C02A12.6	3	4	4	2	2.80E-06	2.65E-06	1.82E-06	2.25E-06
C02A12.8	4	4	13	4	2.80E-06	2.65E-06	1.82E-06	2.25E-06
C02A12.9	9	5	5	4	2.80E-06	2.65E-06	1.82E-06	2.25E-06
C02B10.1	1055	1245	1284	1802	8.14E-05	9.07E-05	6.44E-05	1.12E-04
C02B10.2	222	195	346	273	3.79E-05	3.14E-05	3.84E-05	3.74E-05
C02B10.3.1	85	105	108	124	6.58E-06	7.67E-06	5.45E-06	7.71E-06
C02B10.3.2	33	53	46	59	4.90E-06	7.46E-06	4.45E-06	7.04E-06
C02B10.4.1	473	568	779	682	6.05E-05	6.87E-05	6.49E-05	7.01E-05
C02B10.4.2	342	366	564	549	5.90E-05	5.97E-05	6.33E-05	7.61E-05
C02B10.5.1	658	592	802	1141	2.82E-05	2.40E-05	2.24E-05	3.93E-05
C02B10.5.2	536	462	646	943	2.27E-05	1.85E-05	1.78E-05	3.21E-05
C02B10.6	47	75	36	16	4.40E-06	6.64E-06	2.19E-06	2.25E-06
C02B4.1	39	85	34	53	2.80E-06	2.65E-06	1.82E-06	2.25E-06
C02B4.2	92	230	65	97	6.47E-06	1.53E-05	2.97E-06	5.49E-06
C02B4.3	1	2	2	3	2.80E-06	2.65E-06	1.82E-06	2.25E-06
C02B4.t2	0	0	2	0	2.80E-06	2.65E-06	2.00E-06	2.25E-06
C02B8.1.1	11	14	5	7	2.80E-06	2.65E-06	1.82E-06	2.25E-06
C02B8.1.2	11	15	6	10	2.80E-06	3.09E-06	1.82E-06	2.25E-06
C02B8.2	2	5	7	4	2.80E-06	2.65E-06	1.82E-06	2.25E-06
C02B8.3	8	9	21	5	2.80E-06	2.65E-06	2.35E-06	2.25E-06
C02B8.4	1	9	1	5	2.80E-06	2.65E-06	1.82E-06	2.25E-06
C02B8.5	5	9	3	7	2.80E-06	2.65E-06	1.82E-06	2.25E-06
C02B8.6	57	81	60	75	3.56E-06	4.79E-06	2.44E-06	3.78E-06
C02C2.3	60	96	74	96	4.82E-06	7.27E-06	3.86E-06	6.18E-06
C02C2.4	34	45	41	20	2.80E-06	2.78E-06	1.82E-06	2.25E-06
C02C2.5	6	24	19	20	2.80E-06	2.65E-06	1.82E-06	2.25E-06
C02C6.1a	464	465	424	531	1.55E-05	1.47E-05	9.24E-06	1.43E-05
C02C6.1b	464	463	417	531	1.59E-05	1.50E-05	9.31E-06	1.46E-05
C02C6.2a	42	84	54	94	2.80E-06	3.54E-06	1.82E-06	3.37E-06
C02C6.2b	20	37	23	31	2.80E-06	2.65E-06	1.82E-06	2.25E-06
C02C6.3a	151	260	173	210	1.45E-05	2.36E-05	1.08E-05	1.62E-05
C02C6.3b	73	137	62	101	1.21E-05	2.14E-05	6.67E-06	1.34E-05
C02D4.1	15	11	10	4	2.80E-06	2.65E-06	1.82E-06	2.25E-06
C02D4.2a	7	15	8	9	2.80E-06	2.65E-06	1.82E-06	2.25E-06
C02D4.2b	8	15	8	9	2.80E-06	2.65E-06	1.82E-06	2.25E-06
C02D4.2c	4	2	5	3	2.80E-06	2.65E-06	1.82E-06	2.25E-06
C02D4.2d	3	2	5	3	2.80E-06	2.65E-06	1.82E-06	2.25E-06
C02D4.2e	7	15	8	9	2.80E-06	2.65E-06	1.82E-06	2.25E-06
C02D4.2f	11	23	17	16	2.80E-06	2.65E-06	1.82E-06	2.25E-06
C02D4.t1	0	0	1	0	2.80E-06	2.65E-06	1.82E-06	2.25E-06
C02D5.1	32	61	8	5	2.91E-06	5.26E-06	1.82E-06	2.25E-06
C02D5.2a	50	69	33	26	4.73E-06	6.16E-06	2.02E-06	2.25E-06

C02D5.2b	32	39	18	18	4.54E-06	5.21E-06	1.82E-06	2.25E-06
C02D5.3a	162	200	203	129	1.12E-05	1.31E-05	9.17E-06	7.20E-06
C02D5.3b	158	196	188	124	2.23E-05	2.61E-05	1.72E-05	1.40E-05
C02D5.3c	9	11	12	9	2.80E-06	2.65E-06	1.82E-06	2.25E-06
C02E11.1a.1	1064	1331	1079	1558	3.28E-05	3.88E-05	2.16E-05	3.86E-05
C02E11.1a.2	962	1172	997	1455	3.20E-05	3.68E-05	2.16E-05	3.89E-05
C02E11.1b	958	1167	989	1449	3.18E-05	3.66E-05	2.14E-05	3.87E-05
C02E7.1	11	13	14	7	2.80E-06	2.65E-06	1.82E-06	2.25E-06
C02E7.10	6	9	24	8	2.80E-06	2.65E-06	1.82E-06	2.25E-06
C02E7.11	6	7	2	2	2.80E-06	2.65E-06	1.82E-06	2.25E-06
C02E7.12	7	8	3	2	2.80E-06	2.65E-06	1.82E-06	2.25E-06
C02E7.13	0	4	7	4	2.80E-06	2.65E-06	1.82E-06	2.25E-06
C02E7.2	8	11	12	2	2.80E-06	2.65E-06	1.82E-06	2.25E-06
C02E7.3	6	4	3	5	2.80E-06	2.65E-06	1.82E-06	2.25E-06
C02E7.4	2	5	8	4	2.80E-06	2.65E-06	1.82E-06	2.25E-06
C02E7.5	3	6	2	6	2.80E-06	2.65E-06	1.82E-06	2.25E-06
C02E7.7	307	241	433	37	1.14E-04	8.47E-05	1.05E-04	1.11E-05
C02E7.8	6	3	3	5	2.80E-06	2.65E-06	1.82E-06	2.25E-06
C02E7.9	2	1	3	4	2.80E-06	2.65E-06	1.82E-06	2.25E-06
C02F12.1	5	2	3	2	2.80E-06	2.65E-06	1.82E-06	2.25E-06
C02F12.3.1	31	54	60	31	5.21E-06	8.57E-06	6.56E-06	4.18E-06
C02F12.3.2	28	48	59	25	5.12E-06	8.31E-06	7.03E-06	3.69E-06
C02F12.4	124	146	189	240	6.30E-06	7.01E-06	6.27E-06	9.81E-06
C02F12.5	24	65	58	48	3.53E-06	9.07E-06	5.58E-06	5.69E-06
C02F12.7	142	247	88	131	4.51E-06	7.41E-06	1.82E-06	3.33E-06
C02F12.8	96	132	104	186	4.82E-06	6.27E-06	3.39E-06	7.49E-06
C02F12.9	6	4	2	1	2.80E-06	2.65E-06	1.82E-06	2.25E-06
C02F12.t1	1	0	0	0	2.80E-06	2.65E-06	1.82E-06	2.25E-06
C02F4.1	989	859	1187	1885	1.95E-05	1.60E-05	1.52E-05	2.98E-05
C02F4.2a	318	376	361	541	2.21E-05	2.47E-05	1.64E-05	3.03E-05
C02F4.2b	315	365	354	529	1.23E-05	1.34E-05	8.96E-06	1.66E-05
C02F4.4	56	93	27	14	1.02E-05	1.60E-05	3.21E-06	2.25E-06
C02F4.5	0	2	1	0	2.80E-06	2.65E-06	1.82E-06	2.25E-06
C02F5.1	832	865	937	1463	2.73E-05	2.68E-05	2.00E-05	3.86E-05
C02F5.10	24	33	23	17	6.27E-06	8.15E-06	3.90E-06	3.58E-06
C02F5.11	7	10	9	3	2.80E-06	2.65E-06	1.82E-06	2.25E-06
C02F5.12	32	35	33	47	3.14E-06	3.23E-06	2.10E-06	3.69E-06
C02F5.13	98	124	130	105	1.73E-05	2.07E-05	1.50E-05	1.49E-05
C02F5.14	55	100	36	21	1.36E-05	2.33E-05	5.78E-06	4.16E-06
C02F5.2	15	25	10	5	6.52E-06	1.03E-05	2.82E-06	2.25E-06
C02F5.3	396	485	462	582	3.66E-05	4.23E-05	2.78E-05	4.32E-05
C02F5.4	397	372	432	476	4.20E-05	3.72E-05	2.97E-05	4.04E-05
C02F5.5	46	61	15	16	9.86E-06	1.24E-05	2.10E-06	2.77E-06
C02F5.6a	205	228	179	311	1.82E-05	1.92E-05	1.03E-05	2.22E-05
C02F5.6b	232	258	196	327	1.83E-05	1.93E-05	1.01E-05	2.08E-05
C02F5.7a.1	147	238	107	219	7.81E-06	1.19E-05	3.70E-06	9.33E-06
C02F5.7a.2	146	230	106	218	7.50E-06	1.12E-05	3.55E-06	9.02E-06
C02F5.7a.3	120	190	86	182	9.16E-06	1.37E-05	4.26E-06	1.12E-05
C02F5.7a.4	120	190	86	183	9.16E-06	1.37E-05	4.26E-06	1.12E-05
C02F5.7b.1	169	257	156	249	8.40E-06	1.21E-05	5.05E-06	9.94E-06
C02F5.7b.2	122	193	86	183	9.21E-06	1.38E-05	4.23E-06	1.11E-05
C02F5.8	2	11	1	7	2.80E-06	2.65E-06	1.82E-06	2.25E-06
C02F5.9.1	1203	1017	1872	1376	1.53E-04	1.22E-04	1.55E-04	1.40E-04
C02G6.1	165	187	184	237	6.27E-06	6.72E-06	4.56E-06	7.24E-06
C02G6.2	63	76	80	95	2.88E-06	3.28E-06	2.39E-06	3.49E-06
C02G6.3	5	8	4	3	2.80E-06	2.65E-06	1.82E-06	2.25E-06
C02H6.1	5	1	9	5	2.80E-06	2.65E-06	1.82E-06	2.25E-06
C02H6.2	21	45	26	21	2.80E-06	4.68E-06	1.86E-06	2.25E-06
C02H7.1	16	29	19	23	2.80E-06	2.65E-06	1.82E-06	2.25E-06

C02H7.2	4	13	8	4	2.80E-06	2.65E-06	1.82E-06	2.25E-06
C02H7.3a	97	108	73	112	2.80E-06	2.65E-06	1.82E-06	2.25E-06
C02H7.3b	79	85	60	98	2.80E-06	2.65E-06	1.82E-06	2.25E-06
C03A3.1a	15	19	15	20	2.80E-06	2.65E-06	1.82E-06	2.25E-06
C03A3.1b	20	26	21	21	2.80E-06	2.65E-06	1.82E-06	2.25E-06
C03A3.2.1	371	426	353	519	2.11E-05	2.29E-05	1.31E-05	2.37E-05
C03A3.2.2	280	310	277	417	2.03E-05	2.13E-05	1.31E-05	2.43E-05
C03A3.3	7	6	0	9	2.80E-06	2.65E-06	1.82E-06	2.25E-06
C03A7.10	3	6	5	2	2.80E-06	2.65E-06	1.82E-06	2.25E-06
C03A7.11	21	55	31	27	2.80E-06	3.54E-06	1.82E-06	2.25E-06
C03A7.12	8	3	2	7	2.80E-06	2.65E-06	1.82E-06	2.25E-06
C03A7.13	11	8	10	4	2.80E-06	2.65E-06	1.82E-06	2.25E-06
C03A7.14	229	226	27	42	1.71E-05	1.59E-05	1.82E-06	2.52E-06
C03A7.2	0	2	3	2	2.80E-06	2.65E-06	1.82E-06	2.25E-06
C03A7.3	11	4	6	10	2.80E-06	2.65E-06	1.82E-06	2.25E-06
C03A7.4	217	182	16	36	1.98E-05	1.57E-05	1.82E-06	2.63E-06
C03A7.5	1	8	4	3	2.80E-06	2.65E-06	1.82E-06	2.25E-06
C03A7.6	8	8	11	4	2.80E-06	2.65E-06	1.82E-06	2.25E-06
C03A7.7	223	175	11	38	2.13E-05	1.58E-05	1.82E-06	2.90E-06
C03A7.8	232	179	21	39	1.97E-05	1.44E-05	1.82E-06	2.65E-06
C03A7.9	7	4	3	5	2.80E-06	2.65E-06	1.82E-06	2.25E-06
C03B1.1	5	1	2	4	2.80E-06	2.65E-06	1.82E-06	2.25E-06
C03B1.10	1	1	10	0	2.80E-06	2.65E-06	4.59E-06	2.25E-06
C03B1.12.1	815	1304	877	937	8.54E-05	1.29E-04	5.98E-05	7.89E-05
C03B1.12.2	551	890	548	634	8.29E-05	1.27E-04	5.37E-05	7.67E-05
C03B1.13	33	45	29	28	2.83E-06	3.62E-06	1.82E-06	2.25E-06
C03B1.14	24	25	23	17	3.47E-06	3.41E-06	2.17E-06	2.25E-06
C03B1.15	2	2	0	0	2.80E-06	2.65E-06	1.82E-06	2.25E-06
C03B1.2	15	10	32	14	2.80E-06	2.65E-06	2.77E-06	2.25E-06
C03B1.3	5	1	3	1	2.80E-06	2.65E-06	1.82E-06	2.25E-06
C03B1.4	2	4	1	1	2.80E-06	2.65E-06	1.82E-06	2.25E-06
C03B1.5	14	18	14	21	2.80E-06	2.65E-06	1.82E-06	2.25E-06
C03B1.6	14	16	18	14	3.39E-06	3.65E-06	2.82E-06	2.72E-06
C03B1.7	45	69	33	75	2.80E-06	2.96E-06	1.82E-06	2.74E-06
C03B1.9	2	2	2	0	2.80E-06	2.65E-06	1.82E-06	2.25E-06
C03B8.1	4	10	1	4	2.80E-06	3.33E-06	1.82E-06	2.25E-06
C03B8.2	5	9	0	7	3.11E-06	5.29E-06	1.82E-06	3.51E-06
C03B8.3	6	6	14	6	2.80E-06	2.65E-06	2.64E-06	2.25E-06
C03B8.4	2023	1694	2962	3649	3.22E-05	2.55E-05	3.07E-05	4.67E-05
C03C10.1.1	3681	3420	3147	5177	2.17E-04	1.91E-04	1.21E-04	2.45E-04
C03C10.1.2	3552	3289	3056	5112	2.11E-04	1.84E-04	1.18E-04	2.43E-04
C03C10.1.3	2028	1910	1666	2443	2.05E-04	1.82E-04	1.09E-04	1.98E-04
C03C10.2	10	24	9	3	2.80E-06	2.65E-06	1.82E-06	2.25E-06
C03C10.3.1	2261	2533	3004	3613	1.84E-04	1.95E-04	1.59E-04	2.36E-04
C03C10.3.2	1950	2171	2481	3259	1.70E-04	1.79E-04	1.41E-04	2.28E-04
C03C10.4	291	462	363	441	3.39E-05	5.08E-05	2.75E-05	4.12E-05
C03C10.5	4	3	0	2	2.80E-06	2.65E-06	1.82E-06	2.25E-06
C03C10.7	12	4	5	4	2.80E-06	2.65E-06	1.82E-06	2.25E-06
C03C11.1	17	42	16	12	3.16E-06	7.35E-06	1.93E-06	2.25E-06
C03C11.2	16	25	10	6	2.80E-06	2.94E-06	1.82E-06	2.25E-06
C03D6.1	15	36	14	24	2.80E-06	4.84E-06	1.82E-06	2.74E-06
C03D6.3a	319	372	372	544	1.66E-05	1.83E-05	1.26E-05	2.28E-05
C03D6.3b	251	288	288	468	1.63E-05	1.77E-05	1.22E-05	2.44E-05
C03D6.4	1607	1408	2086	3052	4.16E-05	3.44E-05	3.51E-05	6.34E-05
C03D6.5	188	238	314	287	2.14E-05	2.56E-05	2.32E-05	2.62E-05
C03D6.6	125	141	185	83	2.88E-05	3.07E-05	2.78E-05	1.54E-05
C03D6.8.1	424	395	338	337	7.99E-05	7.04E-05	4.15E-05	5.10E-05
C03D6.8.2	363	334	274	311	6.96E-05	6.05E-05	3.42E-05	4.79E-05
C03E10.1	7	6	1	2	2.80E-06	2.65E-06	1.82E-06	2.25E-06

C03E10.3	5	11	10	10	2.80E-06	2.65E-06	1.82E-06	2.25E-06
C03E10.4	572	614	880	1177	3.44E-05	3.49E-05	3.45E-05	5.69E-05
C03E10.5	76	98	80	230	4.40E-06	5.34E-06	3.01E-06	1.07E-05
C03E10.6	69	129	87	160	2.39E-05	4.21E-05	1.96E-05	4.44E-05
C03F11.1	6	10	3	8	2.80E-06	2.65E-06	1.82E-06	2.25E-06
C03F11.2	7	15	7	6	2.80E-06	2.65E-06	1.82E-06	2.25E-06
C03F11.3	299	357	285	357	1.87E-05	2.11E-05	1.16E-05	1.79E-05
C03F11.4.1	44	59	39	39	3.25E-06	4.10E-06	1.88E-06	2.32E-06
C03F11.4.2	39	55	38	39	3.14E-06	4.18E-06	1.99E-06	2.52E-06
C03F11.4.3	40	57	38	41	3.22E-06	4.34E-06	1.99E-06	2.65E-06
C03F11.t1	1	0	0	0	2.80E-06	2.65E-06	1.82E-06	2.25E-06
C03G5.1.1	957	1077	954	1237	4.68E-05	4.97E-05	3.04E-05	4.86E-05
C03G5.1.2	947	1064	924	1233	4.63E-05	4.92E-05	2.94E-05	4.84E-05
C03G5.10	113	251	167	57	4.14E-05	8.68E-05	3.98E-05	1.68E-05
C03G5.11	74	139	69	32	2.71E-05	4.81E-05	1.64E-05	9.40E-06
C03G5.12	91	170	88	49	3.33E-05	5.88E-05	2.10E-05	1.44E-05
C03G5.13	93	178	93	55	3.40E-05	6.16E-05	2.22E-05	1.62E-05
C03G5.2	192	457	444	113	6.36E-05	1.43E-04	9.57E-05	3.01E-05
C03G5.7	47	79	82	28	9.69E-06	1.54E-05	1.10E-05	4.63E-06
C03G5.8	192	468	480	112	5.53E-05	1.27E-04	8.99E-05	2.59E-05
C03G5.9	93	178	93	55	3.40E-05	6.16E-05	2.22E-05	1.62E-05
C03G6.1	4	3	4	4	2.80E-06	2.65E-06	1.82E-06	2.25E-06
C03G6.10	8	14	3	12	2.80E-06	2.65E-06	1.82E-06	2.25E-06
C03G6.11	9	6	5	5	2.80E-06	2.65E-06	1.82E-06	2.25E-06
C03G6.12	1	8	8	4	2.80E-06	2.65E-06	1.82E-06	2.25E-06
C03G6.13	6	18	7	13	2.80E-06	2.65E-06	1.82E-06	2.25E-06
C03G6.14	34	53	30	30	2.91E-06	4.29E-06	1.82E-06	2.25E-06
C03G6.15	606	805	646	790	4.33E-05	5.43E-05	3.00E-05	4.53E-05
C03G6.16	7	6	6	3	2.80E-06	2.65E-06	1.82E-06	2.25E-06
C03G6.17	11	15	14	8	2.80E-06	2.65E-06	1.82E-06	2.25E-06
C03G6.18	72	91	67	116	7.36E-06	8.78E-06	4.45E-06	9.51E-06
C03G6.19	141	191	145	232	1.35E-05	1.72E-05	9.02E-06	1.78E-05
C03G6.2	10	8	6	4	2.80E-06	2.65E-06	1.82E-06	2.25E-06
C03G6.20	1	11	5	2	2.80E-06	2.65E-06	1.82E-06	2.25E-06
C03G6.3	2	8	0	3	2.80E-06	2.65E-06	1.82E-06	2.25E-06
C03G6.5	23	58	28	12	4.37E-06	1.04E-05	3.46E-06	2.25E-06
C03G6.6	5	14	22	13	2.80E-06	2.65E-06	1.82E-06	2.25E-06
C03G6.7	2	3	3	2	2.80E-06	2.65E-06	1.82E-06	2.25E-06
C03G6.8	5	12	21	14	2.80E-06	2.65E-06	1.82E-06	2.25E-06
C03G6.9	4	3	8	3	2.80E-06	2.65E-06	1.82E-06	2.25E-06
C03H12.1	120	139	75	169	5.85E-06	6.40E-06	2.37E-06	6.61E-06
C03H5.2	370	495	513	415	3.42E-05	4.32E-05	3.08E-05	3.08E-05
C03H5.3	124	134	149	248	1.50E-05	1.53E-05	1.17E-05	2.40E-05
C03H5.4	1	0	4	1	2.80E-06	2.65E-06	1.82E-06	2.25E-06
C03H5.5	51	84	53	55	3.42E-06	5.29E-06	2.30E-06	2.95E-06
C03H5.6	32	28	48	34	2.80E-06	2.65E-06	2.20E-06	2.25E-06
C03H5.7	3	4	1	1	2.80E-06	2.65E-06	1.82E-06	2.25E-06
C04A11.1	19	21	12	15	2.80E-06	2.65E-06	1.82E-06	2.25E-06
C04A11.2	20	29	32	37	2.80E-06	2.65E-06	1.82E-06	2.25E-06
C04A11.3	239	265	170	400	7.25E-06	7.59E-06	3.35E-06	9.76E-06
C04A11.4	123	190	174	210	4.28E-06	6.27E-06	3.95E-06	5.89E-06
C04A11.5	0	8	4	3	2.80E-06	3.09E-06	1.82E-06	2.25E-06
C04A11.t1	0	1	0	0	2.80E-06	2.65E-06	1.82E-06	2.25E-06
C04A2.3a	1202	1257	1464	2206	3.33E-05	3.29E-05	2.64E-05	4.91E-05
C04A2.3b	907	949	1086	1687	3.01E-05	2.98E-05	2.35E-05	4.50E-05
C04A2.3c.1	823	850	1014	1588	4.50E-05	4.39E-05	3.61E-05	6.97E-05
C04A2.3c.2	760	779	946	1494	4.59E-05	4.45E-05	3.72E-05	7.25E-05
C04A2.7a	2027	1883	2615	3588	7.14E-05	6.27E-05	5.99E-05	1.02E-04
C04A2.7b.1	1967	1837	2643	3577	5.71E-05	5.03E-05	4.99E-05	8.33E-05

C04A2.7b.2	2189	2040	2825	3827	6.37E-05	5.60E-05	5.35E-05	8.94E-05
C04A2.7c	1998	1858	2570	3529	7.06E-05	6.20E-05	5.91E-05	1.00E-04
C04A2.7d	1806	1662	2430	3336	7.71E-05	6.71E-05	6.75E-05	1.14E-04
C04A2.t1	0	1	6	0	2.80E-06	2.65E-06	5.34E-06	2.25E-06
C04B4.1	6	4	4	4	2.80E-06	2.65E-06	1.82E-06	2.25E-06
C04B4.2	340	361	678	787	2.29E-05	2.30E-05	2.97E-05	4.26E-05
C04B4.3	4	3	5	2	2.80E-06	2.65E-06	1.82E-06	2.25E-06
C04B4.4	4	3	2	5	2.80E-06	2.65E-06	1.82E-06	2.25E-06
C04B4.5	6	8	16	21	2.80E-06	2.65E-06	1.82E-06	2.72E-06
C04B4.6	0	0	3	2	2.80E-06	2.65E-06	1.82E-06	2.25E-06
C04C11.1a	15	30	9	32	2.80E-06	3.65E-06	1.82E-06	3.31E-06
C04C11.2.1	177	313	189	341	8.88E-06	1.48E-05	6.16E-06	1.37E-05
C04C11.2.2	135	250	148	290	8.60E-06	1.50E-05	6.12E-06	1.48E-05
C04C3.1	3	9	4	2	2.80E-06	2.65E-06	1.82E-06	2.25E-06
C04C3.2	12	6	9	8	2.80E-06	2.65E-06	1.82E-06	2.25E-06
C04C3.3.1	1407	1236	1419	1886	1.22E-04	1.02E-04	8.03E-05	1.32E-04
C04C3.3.2	1144	1009	1004	1470	1.19E-04	9.93E-05	6.81E-05	1.23E-04
C04C3.4	5	10	3	8	3.11E-06	5.87E-06	1.82E-06	4.00E-06
C04C3.5a	13	15	10	10	2.80E-06	2.65E-06	1.82E-06	2.25E-06
C04C3.5b	18	21	17	22	2.80E-06	2.65E-06	1.82E-06	2.25E-06
C04C3.5c	17	17	12	15	2.80E-06	2.65E-06	1.82E-06	2.25E-06
C04C3.6	1	4	7	5	2.80E-06	2.65E-06	1.82E-06	2.25E-06
C04C3.7	2	2	1	2	2.80E-06	2.65E-06	1.82E-06	2.25E-06
C04D8.1	749	708	648	1155	1.75E-05	1.56E-05	9.84E-06	2.17E-05
C04E12.1	5	5	12	3	2.80E-06	2.65E-06	1.82E-06	2.25E-06
C04E12.10	6	6	22	3	2.80E-06	2.65E-06	1.82E-06	2.25E-06
C04E12.11	7	7	4	3	2.80E-06	2.65E-06	1.82E-06	2.25E-06
C04E12.12	8	13	9	5	2.80E-06	2.65E-06	1.82E-06	2.25E-06
C04E12.5	16	19	19	22	2.80E-06	2.65E-06	1.82E-06	2.25E-06
C04E12.6	0	2	4	0	2.80E-06	2.65E-06	1.82E-06	2.25E-06
C04E12.7	8	9	2	4	2.80E-06	2.65E-06	1.82E-06	2.25E-06
C04E12.8	15	7	6	5	2.80E-06	2.65E-06	1.82E-06	2.25E-06
C04E12.9	7	2	6	4	2.80E-06	2.65E-06	1.82E-06	2.25E-06
C04E6.10	5	8	26	7	2.80E-06	2.65E-06	1.88E-06	2.25E-06
C04E6.11	363	341	420	665	1.29E-05	1.15E-05	9.73E-06	1.90E-05
C04E6.12	27	32	8	8	5.24E-06	5.85E-06	1.82E-06	2.25E-06
C04E6.13	10	15	22	2	3.36E-06	4.76E-06	4.79E-06	2.25E-06
C04E6.2	6	3	7	4	2.80E-06	2.65E-06	1.82E-06	2.25E-06
C04E6.3	3	2	12	2	2.80E-06	2.65E-06	4.56E-06	2.25E-06
C04E6.4	12	15	12	7	2.80E-06	2.65E-06	1.82E-06	2.25E-06
C04E6.5	24	30	16	12	2.80E-06	2.65E-06	1.82E-06	2.25E-06
C04E6.6	13	14	12	10	2.80E-06	2.65E-06	1.82E-06	2.25E-06
C04E6.7	28	56	26	54	2.88E-06	5.48E-06	1.82E-06	4.48E-06
C04E6.8	2	2	2	1	2.80E-06	2.65E-06	1.82E-06	2.25E-06
C04E6.9	7	6	8	3	2.80E-06	2.65E-06	1.82E-06	2.25E-06
C04E7.1	1	0	1	2	2.80E-06	2.65E-06	1.82E-06	2.25E-06
C04E7.2	53	71	83	123	3.28E-06	4.15E-06	3.33E-06	6.12E-06
C04E7.3	22	23	15	30	2.80E-06	2.65E-06	1.82E-06	2.25E-06
C04E7.4	22	25	25	28	2.80E-06	2.65E-06	1.82E-06	2.25E-06
C04E7.t1	0	0	1	0	2.80E-06	2.65E-06	1.82E-06	2.25E-06
C04F1.1	26	36	30	7	3.02E-06	3.94E-06	2.26E-06	2.25E-06
C04F1.3	30	41	31	37	2.80E-06	3.20E-06	1.82E-06	2.45E-06
C04F12.1	292	392	497	706	1.07E-05	1.36E-05	1.19E-05	2.08E-05
C04F12.10	490	688	680	782	3.74E-05	4.96E-05	3.38E-05	4.79E-05
C04F12.3	39	106	23	52	2.80E-06	5.85E-06	1.82E-06	2.45E-06
C04F12.4.1	5055	6523	11413	3184	1.24E-03	1.51E-03	1.82E-03	6.27E-04
C04F12.4.2	4022	5286	8699	2893	9.69E-04	1.20E-03	1.36E-03	5.60E-04
C04F12.5	44	75	66	52	1.29E-05	2.08E-05	1.26E-05	1.23E-05
C04F12.6	21	30	5	5	3.05E-06	4.13E-06	1.82E-06	2.25E-06

C04F12.7	267	557	145	163	4.31E-05	8.49E-05	1.52E-05	2.11E-05
C04F12.8	35	58	43	71	4.79E-06	7.49E-06	3.83E-06	7.80E-06
C04F12.9	15	21	52	15	4.00E-06	5.29E-06	9.02E-06	3.22E-06
C04F2.1	2	2	0	2	2.80E-06	2.65E-06	1.82E-06	2.25E-06
C04F2.2	4	5	5	4	2.80E-06	2.65E-06	1.82E-06	2.25E-06
C04F2.3	3	10	4	2	2.80E-06	2.65E-06	1.82E-06	2.25E-06
C04F2.4	3	3	6	2	2.80E-06	2.65E-06	1.82E-06	2.25E-06
C04F2.5	2	4	2	2	2.80E-06	2.65E-06	1.82E-06	2.25E-06
C04F2.t1	0	0	1	1	2.80E-06	2.65E-06	1.82E-06	2.25E-06
C04F5.1	321	342	500	663	1.36E-05	1.36E-05	1.38E-05	2.25E-05
C04F5.2	0	3	4	3	2.80E-06	2.65E-06	1.82E-06	2.25E-06
C04F5.3	4	7	13	6	2.80E-06	2.65E-06	1.82E-06	2.25E-06
C04F5.4	1	5	6	2	2.80E-06	2.65E-06	1.82E-06	2.25E-06
C04F5.5	4	2	5	3	2.80E-06	2.65E-06	1.82E-06	2.25E-06
C04F5.6	5	4	0	2	2.80E-06	2.65E-06	1.82E-06	2.25E-06
C04F5.7	131	155	241	273	9.66E-06	1.08E-05	1.16E-05	1.61E-05
C04F5.8	293	260	303	296	3.12E-05	2.62E-05	2.10E-05	2.53E-05
C04F5.9	141	131	194	288	9.32E-06	8.17E-06	8.35E-06	1.53E-05
C04F6.2	5	5	4	0	2.80E-06	2.65E-06	1.82E-06	2.25E-06
C04F6.3.1	182	159	96	271	9.94E-06	8.20E-06	3.41E-06	1.19E-05
C04F6.3.2	158	137	89	256	9.55E-06	7.80E-06	3.50E-06	1.24E-05
C04F6.4a	265	321	281	365	1.50E-05	1.71E-05	1.03E-05	1.66E-05
C04F6.4b.1	272	323	296	358	1.47E-05	1.65E-05	1.04E-05	1.55E-05
C04F6.4b.2	207	255	232	293	1.49E-05	1.74E-05	1.09E-05	1.70E-05
C04F6.5	63	88	58	47	2.88E-06	3.81E-06	1.82E-06	2.25E-06
C04G2.1	20	30	23	15	4.23E-06	5.98E-06	3.15E-06	2.54E-06
C04G2.10	29	47	40	74	3.25E-06	4.97E-06	2.92E-06	6.66E-06
C04G2.11	12	12	29	8	2.80E-06	2.65E-06	1.82E-06	2.25E-06
C04G2.2	15	55	12	14	2.80E-06	5.18E-06	1.82E-06	2.25E-06
C04G2.3	1005	1489	1408	374	2.50E-04	3.49E-04	2.28E-04	7.46E-05
C04G2.4	1802	2420	2287	548	4.47E-04	5.66E-04	3.69E-04	1.09E-04
C04G2.5	19	34	15	12	3.14E-06	5.32E-06	1.82E-06	2.25E-06
C04G2.6	553	608	634	959	1.89E-05	1.97E-05	1.41E-05	2.63E-05
C04G2.7	12	10	4	4	2.80E-06	2.65E-06	1.82E-06	2.25E-06
C04G2.8	159	271	117	63	2.52E-05	4.06E-05	1.21E-05	8.03E-06
C04G2.9	430	603	421	296	4.04E-05	5.35E-05	2.57E-05	2.23E-05
C04G6.10	24	30	13	16	4.79E-06	5.66E-06	1.82E-06	2.56E-06
C04G6.11	21	17	17	22	5.94E-06	4.55E-06	3.13E-06	4.99E-06
C04G6.1a	32	95	43	43	2.80E-06	5.34E-06	1.82E-06	2.25E-06
C04G6.1b	26	72	38	29	2.80E-06	5.77E-06	2.10E-06	2.25E-06
C04G6.2	41	50	11	14	5.46E-06	6.27E-06	1.82E-06	2.25E-06
C04G6.4	195	227	269	256	1.48E-05	1.63E-05	1.33E-05	1.56E-05
C04G6.5	18	16	12	4	7.42E-06	6.22E-06	3.21E-06	2.25E-06
C04G6.6	3	5	13	6	2.80E-06	2.65E-06	3.04E-06	2.25E-06
C04G6.7	9	13	2	10	2.80E-06	2.65E-06	1.82E-06	2.25E-06
C04H4.1	2	0	1	1	2.80E-06	2.65E-06	1.82E-06	2.25E-06
C04H5.1	70	50	192	75	2.07E-05	1.40E-05	3.70E-05	1.79E-05
C04H5.2	20	33	53	64	2.80E-06	4.23E-06	4.68E-06	6.97E-06
C04H5.3	10	13	26	10	2.80E-06	2.65E-06	1.82E-06	2.25E-06
C04H5.6	417	424	478	720	1.46E-05	1.40E-05	1.09E-05	2.03E-05
C04H5.8	17	19	7	7	7.22E-06	7.62E-06	1.93E-06	2.38E-06
C04H5.9	2	3	1	1	2.80E-06	2.65E-06	1.82E-06	2.25E-06
C05A2.1	60	128	63	100	4.54E-06	9.13E-06	3.10E-06	6.07E-06
C05A9.1	258	145	143	147	7.39E-06	3.91E-06	2.66E-06	3.37E-06
C05A9.2	36	30	14	27	6.72E-06	5.29E-06	1.82E-06	4.05E-06
C05A9.3	5	7	6	1	2.80E-06	2.65E-06	1.82E-06	2.25E-06
C05B10.1	398	365	534	702	4.02E-05	3.48E-05	3.51E-05	5.69E-05
C05B5.1	1	7	2	0	2.80E-06	2.65E-06	1.82E-06	2.25E-06
C05B5.10	5	2	0	2	3.36E-06	2.65E-06	1.82E-06	2.25E-06

C05B5.2	43	136	39	23	4.17E-06	1.24E-05	2.46E-06	2.25E-06
C05B5.3	25	29	12	10	2.80E-06	2.65E-06	1.82E-06	2.25E-06
C05B5.4	9	14	15	23	2.80E-06	2.65E-06	1.82E-06	2.25E-06
C05B5.5	16	12	6	9	2.80E-06	2.65E-06	1.82E-06	2.25E-06
C05B5.6	16	33	7	11	2.80E-06	2.86E-06	1.82E-06	2.25E-06
C05B5.7	41	39	35	54	3.61E-06	3.25E-06	2.00E-06	3.82E-06
C05B5.8	61	137	60	45	1.26E-05	2.67E-05	8.07E-06	7.47E-06
C05B5.9	2	5	0	0	2.80E-06	2.65E-06	1.82E-06	2.25E-06
C05C10.1	7	6	9	2	2.80E-06	2.65E-06	1.82E-06	2.25E-06
C05C10.2a	895	758	1144	1757	2.01E-05	1.61E-05	1.67E-05	3.18E-05
C05C10.3.1	608	938	676	1149	3.97E-05	5.78E-05	2.87E-05	6.02E-05
C05C10.3.2	547	873	608	1075	3.60E-05	5.43E-05	2.61E-05	5.69E-05
C05C10.5a	1034	814	1464	1626	1.18E-04	8.77E-05	1.09E-04	1.49E-04
C05C10.5b	520	406	784	857	9.00E-05	6.64E-05	8.83E-05	1.19E-04
C05C10.5c.1	498	394	741	831	8.77E-05	6.55E-05	8.49E-05	1.18E-04
C05C10.5c.2	495	393	735	831	8.76E-05	6.57E-05	8.46E-05	1.18E-04
C05C10.5c.3	454	366	728	833	9.36E-05	7.13E-05	9.77E-05	1.38E-04
C05C10.6a	1296	1311	2000	2362	5.22E-05	4.99E-05	5.24E-05	7.64E-05
C05C10.6b	1241	1247	1883	2261	5.38E-05	5.11E-05	5.31E-05	7.87E-05
C05C10.7	72	74	81	102	1.14E-05	1.10E-05	8.33E-06	1.29E-05
C05C12.1	19	47	18	7	2.80E-06	4.05E-06	1.82E-06	2.25E-06
C05C12.3	306	358	207	443	6.61E-06	7.30E-06	2.92E-06	7.69E-06
C05C12.4	169	243	91	126	1.98E-05	2.69E-05	6.94E-06	1.19E-05
C05C12.5	37	82	30	16	8.57E-06	1.79E-05	4.52E-06	2.97E-06
C05C12.6	8	24	5	13	2.80E-06	5.58E-06	1.82E-06	2.56E-06
C05C8.2	373	366	285	439	3.51E-05	3.26E-05	1.75E-05	3.32E-05
C05C8.3	211	367	127	144	2.81E-05	4.62E-05	1.10E-05	1.54E-05
C05C8.4	768	599	738	1204	2.47E-05	1.82E-05	1.55E-05	3.11E-05
C05C8.5	284	275	377	535	1.43E-05	1.30E-05	1.23E-05	2.16E-05
C05C8.6	668	623	889	1199	4.08E-05	3.59E-05	3.53E-05	5.88E-05
C05C8.7.1	497	554	434	568	2.53E-05	2.66E-05	1.44E-05	2.32E-05
C05C8.7.2	357	377	310	409	2.78E-05	2.77E-05	1.57E-05	2.56E-05
C05C8.7.3	382	398	327	418	2.56E-05	2.52E-05	1.42E-05	2.25E-05
C05C8.7.4	379	395	324	418	2.75E-05	2.71E-05	1.53E-05	2.44E-05
C05C8.8	6	11	7	4	2.80E-06	2.65E-06	1.82E-06	2.25E-06
C05C8.9a	121	160	152	173	1.51E-05	1.89E-05	1.24E-05	1.74E-05
C05C9.1	13	20	12	9	2.80E-06	2.65E-06	1.82E-06	2.25E-06
C05C9.2	4	6	10	3	2.80E-06	2.65E-06	1.82E-06	2.25E-06
C05C9.3	62	59	64	81	2.80E-06	2.65E-06	1.82E-06	2.25E-06
C05C9.t1	0	0	1	0	2.80E-06	2.65E-06	1.82E-06	2.25E-06
C05C9.t2	0	0	1	0	2.80E-06	2.65E-06	1.82E-06	2.25E-06
C05D10.1b	97	145	92	146	7.78E-06	1.10E-05	4.81E-06	9.40E-06
C05D10.1c.1	99	130	92	161	7.98E-06	9.89E-06	4.83E-06	1.04E-05
C05D10.1c.2	123	157	107	178	9.24E-06	1.11E-05	5.23E-06	1.08E-05
C05D10.1c.3	113	144	98	169	7.90E-06	9.50E-06	4.46E-06	9.49E-06
C05D10.1c.4	99	130	92	160	8.01E-06	9.92E-06	4.85E-06	1.04E-05
C05D10.2a	18	35	25	19	2.80E-06	2.65E-06	1.82E-06	2.25E-06
C05D10.2b	5	4	3	5	2.80E-06	2.65E-06	1.82E-06	2.25E-06
C05D10.2c	18	35	23	18	2.80E-06	2.65E-06	1.82E-06	2.25E-06
C05D10.3	20	38	21	18	2.80E-06	2.65E-06	1.82E-06	2.25E-06
C05D10.4a	63	139	42	58	3.89E-06	8.09E-06	1.82E-06	2.88E-06
C05D10.4b	64	149	42	60	3.56E-06	7.83E-06	1.82E-06	2.68E-06
C05D10.4c	48	113	29	46	3.92E-06	8.73E-06	1.82E-06	3.01E-06
C05D11.1	1027	1339	1577	1938	3.71E-05	4.57E-05	3.71E-05	5.62E-05
C05D11.10	200	247	228	216	3.74E-05	4.36E-05	2.78E-05	3.24E-05
C05D11.11a.	2346	3672	2574	4328	1.80E-04	2.66E-04	1.28E-04	2.66E-04
C05D11.11a.[illegible]	2287	3583	2456	4239	1.75E-04	2.59E-04	1.23E-04	2.61E-04
C05D11.11a.[illegible]	2383	3718	2607	4363	1.74E-04	2.57E-04	1.24E-04	2.56E-04
C05D11.11b.	2601	3973	2835	4698	1.75E-04	2.53E-04	1.24E-04	2.54E-04

C05D11.11b.	2624	4000	2846	4710	1.92E-04	2.76E-04	1.35E-04	2.77E-04
C05D11.12.1	541	1027	679	1082	3.02E-05	5.42E-05	2.47E-05	4.85E-05
C05D11.12.2	493	938	599	989	3.08E-05	5.53E-05	2.43E-05	4.96E-05
C05D11.13	28	35	18	25	3.86E-06	4.55E-06	1.82E-06	2.77E-06
C05D11.2.1	329	449	480	658	1.36E-05	1.75E-05	1.29E-05	2.19E-05
C05D11.2.2	316	417	449	627	1.38E-05	1.72E-05	1.28E-05	2.20E-05
C05D11.3	310	406	233	230	5.54E-05	6.85E-05	2.71E-05	3.30E-05
C05D11.4	46	91	54	73	3.00E-06	5.63E-06	2.30E-06	3.85E-06
C05D11.5	25	30	23	13	3.42E-06	3.89E-06	2.06E-06	2.25E-06
C05D11.6	40	61	29	24	2.80E-06	2.99E-06	1.82E-06	2.25E-06
C05D11.7a.1	673	874	1068	1370	2.76E-05	3.38E-05	2.85E-05	4.51E-05
C05D11.7a.2	489	617	800	945	2.93E-05	3.49E-05	3.12E-05	4.55E-05
C05D11.7b	676	866	1058	1380	2.88E-05	3.49E-05	2.94E-05	4.73E-05
C05D11.8	222	181	448	756	9.49E-06	7.30E-06	1.24E-05	2.59E-05
C05D11.9	347	428	347	608	1.62E-05	1.88E-05	1.05E-05	2.27E-05
C05D12.1	74	66	51	50	4.68E-06	3.94E-06	2.10E-06	2.54E-06
C05D12.2	41	64	55	81	2.80E-06	2.65E-06	1.82E-06	2.29E-06
C05D12.3c	609	388	978	535	2.23E-05	1.34E-05	2.33E-05	1.57E-05
C05D12.4	37	27	28	19	3.64E-06	2.65E-06	1.82E-06	2.25E-06
C05D12.5	6	11	10	5	2.80E-06	2.65E-06	1.82E-06	2.25E-06
C05D12.7	3	8	3	0	2.80E-06	3.91E-06	1.82E-06	2.25E-06
C05D2.10a.1	297	409	470	646	2.06E-05	2.68E-05	2.12E-05	3.59E-05
C05D2.10a.2	271	382	413	588	2.07E-05	2.76E-05	2.06E-05	3.62E-05
C05D2.10b	268	373	412	582	2.04E-05	2.69E-05	2.05E-05	3.57E-05
C05D2.11	24	32	132	41	2.80E-06	2.67E-06	7.62E-06	2.92E-06
C05D2.1a	155	176	227	295	5.91E-06	6.35E-06	5.63E-06	9.04E-06
C05D2.1c	89	100	135	197	5.10E-06	5.40E-06	5.03E-06	9.04E-06
C05D2.1d	77	88	117	154	5.49E-06	5.92E-06	5.43E-06	8.84E-06
C05D2.3	30	44	24	12	2.80E-06	2.96E-06	1.82E-06	2.25E-06
C05D2.4a	101	141	138	137	7.03E-06	9.26E-06	6.25E-06	7.65E-06
C05D2.4b	97	137	134	124	6.83E-06	9.13E-06	6.16E-06	7.04E-06
C05D2.5	1101	801	1364	1737	5.34E-05	3.67E-05	4.31E-05	6.77E-05
C05D2.6b	239	180	346	460	1.73E-05	1.23E-05	1.63E-05	2.67E-05
C05D2.8	216	304	211	263	1.80E-05	2.40E-05	1.15E-05	1.76E-05
C05D9.1.1	151	231	140	185	9.74E-06	1.41E-05	5.87E-06	9.58E-06
C05D9.1.2	150	228	138	181	9.83E-06	1.41E-05	5.89E-06	9.54E-06
C05D9.3	68	135	137	162	4.70E-06	8.83E-06	6.16E-06	9.00E-06
C05D9.4	0	1	1	0	2.80E-06	2.65E-06	1.82E-06	2.25E-06
C05D9.5	32	37	29	28	5.60E-06	6.11E-06	3.30E-06	3.94E-06
C05D9.7	30	35	45	32	3.89E-06	4.29E-06	3.79E-06	3.33E-06
C05D9.9a	29	58	19	30	6.75E-06	1.27E-05	2.88E-06	5.60E-06
C05D9.9b	28	45	14	22	7.48E-06	1.13E-05	2.42E-06	4.72E-06
C05E11.1	108	114	78	123	9.41E-06	9.39E-06	4.43E-06	8.61E-06
C05E11.2	1	2	1	1	2.80E-06	2.65E-06	1.82E-06	2.25E-06
C05E11.3	34	47	61	37	3.14E-06	4.10E-06	3.66E-06	2.74E-06
C05E11.4	16	7	14	6	2.80E-06	2.65E-06	1.82E-06	2.25E-06
C05E11.5	114	243	145	279	6.24E-06	1.25E-05	5.16E-06	1.23E-05
C05E11.6	7	2	9	7	2.80E-06	2.65E-06	1.82E-06	2.25E-06
C05E11.7	15	22	10	12	2.80E-06	2.65E-06	1.82E-06	2.25E-06
C05E4.1	30	93	56	102	2.88E-06	8.44E-06	3.50E-06	7.87E-06
C05E4.10	6	0	13	3	2.80E-06	2.65E-06	1.82E-06	2.25E-06
C05E4.11	2	4	13	2	2.80E-06	2.65E-06	1.82E-06	2.25E-06
C05E4.12	3	8	16	11	2.80E-06	2.65E-06	1.82E-06	2.25E-06
C05E4.13	8	4	8	3	2.80E-06	2.65E-06	1.82E-06	2.25E-06
C05E4.14a	3	4	3	1	2.80E-06	2.65E-06	1.82E-06	2.25E-06
C05E4.14b	3	9	4	3	2.80E-06	2.65E-06	1.82E-06	2.25E-06
C05E4.2	11	3	13	20	2.80E-06	2.65E-06	1.82E-06	2.25E-06
C05E4.3	65	86	103	138	6.33E-06	7.91E-06	6.52E-06	1.08E-05
C05E4.4	5	6	6	2	2.80E-06	2.65E-06	1.82E-06	2.25E-06

C05E4.5	3	4	1	5	2.80E-06	2.65E-06	1.82E-06	2.25E-06
C05E4.6	6	4	0	1	2.80E-06	2.65E-06	1.82E-06	2.25E-06
C05E4.7	0	2	0	0	2.80E-06	2.65E-06	1.82E-06	2.25E-06
C05E7.1	77	102	22	28	9.16E-06	1.15E-05	1.82E-06	2.68E-06
C05E7.2	58	102	18	20	7.53E-06	1.25E-05	1.82E-06	2.25E-06
C05E7.3	5	6	5	3	2.80E-06	2.65E-06	1.82E-06	2.25E-06
C05E7.4	5	4	4	0	2.80E-06	2.65E-06	1.82E-06	2.25E-06
C05E7.t3	0	0	1	0	2.80E-06	2.65E-06	1.82E-06	2.25E-06
C05G5.1	79	51	48	75	5.24E-06	3.20E-06	2.08E-06	4.00E-06
C05G5.2	99	118	141	170	5.24E-06	5.90E-06	4.85E-06	7.22E-06
C05G5.3	17	31	14	16	2.80E-06	3.94E-06	1.82E-06	2.25E-06
C05G5.4	589	784	773	860	4.08E-05	5.13E-05	3.48E-05	4.78E-05
C05G5.5	8	7	9	8	2.80E-06	2.65E-06	1.82E-06	2.25E-06
C05G6.1	30	57	68	65	2.80E-06	3.89E-06	3.19E-06	3.76E-06
C05H8.1a	16	40	34	29	2.80E-06	2.65E-06	1.82E-06	2.25E-06
C05H8.1b	18	44	36	30	2.80E-06	2.65E-06	1.82E-06	2.25E-06
C06A1.2	42	55	48	20	5.82E-06	7.22E-06	4.34E-06	2.25E-06
C06A1.3	38	89	34	23	3.64E-06	8.07E-06	2.11E-06	2.25E-06
C06A1.4	1303	984	2657	2797	5.04E-05	3.59E-05	6.69E-05	8.69E-05
C06A1.5	458	515	1063	422	9.70E-05	1.03E-04	1.46E-04	7.18E-05
C06A1.6	6	13	2	3	2.80E-06	2.99E-06	1.82E-06	2.25E-06
C06A1.7	5	14	2	5	2.80E-06	3.28E-06	1.82E-06	2.25E-06
C06A12.3	34	66	51	31	4.98E-06	9.15E-06	4.88E-06	3.67E-06
C06A12.4	9	10	6	2	2.80E-06	2.65E-06	1.82E-06	2.25E-06
C06A12.5	12	12	20	9	2.80E-06	2.65E-06	1.82E-06	2.25E-06
C06A12.8	3	5	0	3	2.80E-06	2.65E-06	1.82E-06	2.25E-06
C06A5.1	548	474	1142	1676	1.06E-05	8.65E-06	1.44E-05	2.60E-05
C06A5.10	20	9	40	18	9.32E-06	3.97E-06	1.22E-05	6.75E-06
C06A5.11	75	69	55	49	9.32E-06	8.12E-06	4.45E-06	4.90E-06
C06A5.12	5	7	8	6	2.80E-06	3.44E-06	2.70E-06	2.50E-06
C06A5.2	9	16	8	6	2.80E-06	2.65E-06	1.82E-06	2.25E-06
C06A5.3a	420	534	649	873	1.82E-05	2.18E-05	1.83E-05	3.03E-05
C06A5.3b	389	501	595	814	1.85E-05	2.25E-05	1.84E-05	3.10E-05
C06A5.4	27	31	47	56	4.56E-06	4.95E-06	5.17E-06	7.60E-06
C06A5.5	27	28	21	33	7.31E-06	7.17E-06	3.70E-06	7.17E-06
C06A5.6	660	402	934	1452	3.37E-05	1.94E-05	3.10E-05	5.96E-05
C06A5.7a	684	763	767	1171	3.59E-05	3.78E-05	2.62E-05	4.93E-05
C06A5.7b	427	478	493	786	3.69E-05	3.90E-05	2.77E-05	5.46E-05
C06A5.8a	136	117	233	207	1.55E-05	1.26E-05	1.73E-05	1.89E-05
C06A5.8b	99	91	150	155	1.37E-05	1.18E-05	1.35E-05	1.72E-05
C06A5.9	181	225	267	306	1.60E-05	1.89E-05	1.54E-05	2.18E-05
C06A6.2a	119	142	139	137	6.30E-06	7.09E-06	4.79E-06	5.82E-06
C06A6.2b	124	147	141	140	6.52E-06	7.30E-06	4.83E-06	5.91E-06
C06A6.3	178	246	279	259	2.04E-05	2.66E-05	2.08E-05	2.38E-05
C06A6.4b	576	707	740	967	4.83E-05	5.60E-05	4.04E-05	6.51E-05
C06A6.5	249	421	241	296	2.00E-05	3.20E-05	1.26E-05	1.91E-05
C06A6.7	7	16	6	6	2.80E-06	2.99E-06	1.82E-06	2.25E-06
C06A8.1a.1	1488	2809	2319	3171	7.13E-05	1.27E-04	7.24E-05	1.22E-04
C06A8.1a.2	1347	2561	2020	2798	7.30E-05	1.31E-04	7.13E-05	1.22E-04
C06A8.1b	1353	2578	2068	2804	6.86E-05	1.23E-04	6.82E-05	1.14E-04
C06A8.2	285	312	391	522	2.13E-05	2.20E-05	1.90E-05	3.14E-05
C06A8.3	1923	2910	3298	2567	2.61E-04	3.74E-04	2.92E-04	2.80E-04
C06A8.4	252	191	368	297	3.80E-05	2.72E-05	3.61E-05	3.60E-05
C06A8.5	320	337	503	531	2.30E-05	2.29E-05	2.36E-05	3.07E-05
C06A8.6	66	127	31	23	6.50E-06	1.18E-05	1.99E-06	2.25E-06
C06A8.7	11	9	16	9	2.80E-06	2.65E-06	1.82E-06	2.25E-06
C06A8.8a	21	58	16	14	2.80E-06	5.50E-06	1.82E-06	2.25E-06
C06A8.9	33	62	31	34	2.80E-06	2.65E-06	1.82E-06	2.25E-06
C06B3.1	5	3	7	2	2.80E-06	2.65E-06	1.82E-06	2.25E-06

C06B3.10	7	5	13	2	2.80E-06	2.65E-06	1.82E-06	2.25E-06
C06B3.11	5	4	7	1	2.80E-06	2.65E-06	1.82E-06	2.25E-06
C06B3.12	3	5	6	1	2.80E-06	2.65E-06	1.82E-06	2.25E-06
C06B3.13	5	7	7	4	2.80E-06	2.65E-06	1.82E-06	2.25E-06
C06B3.14	3	6	19	8	2.80E-06	2.65E-06	1.82E-06	2.25E-06
C06B3.2	8	14	9	3	2.80E-06	2.65E-06	1.82E-06	2.25E-06
C06B3.3	375	429	437	435	2.79E-05	3.02E-05	2.12E-05	2.60E-05
C06B3.4	92	173	120	90	1.09E-05	1.94E-05	9.26E-06	8.57E-06
C06B3.5	7	7	7	6	2.80E-06	2.65E-06	1.82E-06	2.25E-06
C06B3.7	20	25	7	12	2.80E-06	2.67E-06	1.82E-06	2.25E-06
C06B3.8.1	9	15	6	8	2.80E-06	2.65E-06	1.82E-06	2.25E-06
C06B3.8.2	9	15	6	8	2.80E-06	2.65E-06	1.82E-06	2.25E-06
C06B3.9	10	6	7	3	2.80E-06	2.65E-06	1.82E-06	2.25E-06
C06B8.1	8	7	10	2	2.80E-06	2.65E-06	1.82E-06	2.25E-06
C06B8.10	5	2	7	5	2.80E-06	2.65E-06	1.82E-06	2.25E-06
C06B8.11	2	1	3	2	2.80E-06	2.65E-06	1.82E-06	2.25E-06
C06B8.2a	20	34	25	28	2.80E-06	3.39E-06	1.82E-06	2.38E-06
C06B8.2b	28	42	35	33	2.80E-06	3.68E-06	2.11E-06	2.45E-06
C06B8.3	4	8	5	0	2.80E-06	2.65E-06	1.82E-06	2.25E-06
C06B8.4	3	9	5	5	2.80E-06	2.65E-06	1.82E-06	2.25E-06
C06B8.6	4	8	7	5	2.80E-06	2.65E-06	1.82E-06	2.25E-06
C06B8.7	141	136	118	234	2.80E-06	2.65E-06	1.82E-06	2.25E-06
C06B8.8.1	5225	6381	17241	3527	1.96E-03	2.26E-03	4.20E-03	1.06E-03
C06B8.8.2	3873	4751	13281	3019	1.34E-03	1.56E-03	3.00E-03	8.41E-04
C06B8.9	3	4	10	2	2.80E-06	2.65E-06	1.82E-06	2.25E-06
C06B8.t2	0	0	2	0	2.80E-06	2.65E-06	2.00E-06	2.25E-06
C06B8.t3	0	1	0	1	2.80E-06	2.65E-06	1.82E-06	2.25E-06
C06C3.10	26	27	10	11	1.21E-05	1.19E-05	3.04E-06	4.12E-06
C06C3.1a	1082	1007	1020	1631	3.48E-05	3.06E-05	2.13E-05	4.21E-05
C06C3.1b	974	893	948	1465	3.56E-05	3.09E-05	2.26E-05	4.31E-05
C06C3.1c	952	885	936	1440	3.16E-05	2.77E-05	2.02E-05	3.84E-05
C06C3.1d	912	842	868	1372	3.51E-05	3.07E-05	2.18E-05	4.25E-05
C06C3.3	30	19	27	10	2.80E-06	2.65E-06	1.82E-06	2.25E-06
C06C3.4	20	55	23	34	2.80E-06	4.60E-06	1.82E-06	2.41E-06
C06C3.5	6	3	13	12	2.80E-06	2.65E-06	1.82E-06	2.25E-06
C06C3.6	8	8	10	18	2.80E-06	2.65E-06	1.82E-06	2.25E-06
C06C3.7	5	5	1	4	2.80E-06	2.65E-06	1.82E-06	2.25E-06
C06C3.8	5	13	21	4	2.80E-06	2.65E-06	1.82E-06	2.25E-06
C06C3.9	8	15	11	4	2.80E-06	2.65E-06	1.82E-06	2.25E-06
C06C6.1	1	3	0	1	2.80E-06	2.65E-06	1.82E-06	2.25E-06
C06C6.2	9	1	3	1	2.80E-06	2.65E-06	1.82E-06	2.25E-06
C06C6.3	2	2	5	1	2.80E-06	2.65E-06	1.82E-06	2.25E-06
C06C6.4	13	8	7	9	2.80E-06	2.65E-06	1.82E-06	2.25E-06
C06C6.5a	17	38	25	21	2.80E-06	3.54E-06	1.82E-06	2.25E-06
C06C6.6	17	24	36	14	2.80E-06	2.65E-06	1.82E-06	2.25E-06
C06C6.7	17	37	26	14	2.80E-06	2.65E-06	1.82E-06	2.25E-06
C06C6.8	5	5	9	5	2.80E-06	2.65E-06	1.82E-06	2.25E-06
C06C6.9	0	3	4	0	2.80E-06	2.65E-06	1.82E-06	2.25E-06
C06E1.1	31	23	9	24	6.02E-06	4.23E-06	1.82E-06	3.76E-06
C06E1.10	301	224	230	350	9.77E-06	6.88E-06	4.87E-06	9.13E-06
C06E1.11	11	10	13	10	2.80E-06	2.65E-06	1.82E-06	2.25E-06
C06E1.3	63	91	60	93	3.81E-06	5.21E-06	2.37E-06	4.52E-06
C06E1.4	21	24	18	19	2.80E-06	2.65E-06	1.82E-06	2.25E-06
C06E1.5	1	2	4	0	2.80E-06	2.65E-06	1.91E-06	2.25E-06
C06E1.6	0	3	2	0	2.80E-06	2.65E-06	1.82E-06	2.25E-06
C06E1.7	17	23	13	9	2.80E-06	2.65E-06	1.82E-06	2.25E-06
C06E1.8	10	25	16	14	2.80E-06	2.65E-06	1.82E-06	2.25E-06
C06E1.9	221	274	293	530	1.22E-05	1.42E-05	1.05E-05	2.34E-05
C06E2.1	8	24	5	19	2.80E-06	2.91E-06	1.82E-06	2.25E-06

C06E2.2	0	0	1	0	2.80E-06	2.65E-06	1.82E-06	2.25E-06
C06E2.3	1	0	6	1	2.80E-06	2.65E-06	1.82E-06	2.25E-06
C06E2.5.1	27	26	61	30	5.26E-06	4.81E-06	7.76E-06	4.70E-06
C06E2.5.2	29	29	65	40	5.10E-06	4.81E-06	7.43E-06	5.65E-06
C06E2.7	3	1	5	2	2.80E-06	2.65E-06	1.82E-06	2.25E-06
C06E2.8	6	3	1	4	2.80E-06	2.65E-06	1.82E-06	2.25E-06
C06E2.9	1	0	1	1	2.80E-06	2.65E-06	1.82E-06	2.25E-06
C06E4.1	4	0	1	2	2.80E-06	2.65E-06	1.82E-06	2.25E-06
C06E4.2	18	18	6	7	8.09E-06	7.64E-06	1.82E-06	2.52E-06
C06E4.3	1	9	8	6	2.80E-06	2.65E-06	1.82E-06	2.25E-06
C06E4.4	1	2	5	1	2.80E-06	2.65E-06	1.82E-06	2.25E-06
C06E4.5	5	1	0	1	2.80E-06	2.65E-06	1.82E-06	2.25E-06
C06E4.6	4	2	2	0	2.80E-06	2.65E-06	1.82E-06	2.25E-06
C06E4.7	5	1	6	2	2.80E-06	2.65E-06	1.82E-06	2.25E-06
C06E4.8	5	18	10	11	2.80E-06	2.65E-06	1.82E-06	2.25E-06
C06E7.1a	1324	1766	1748	2969	9.97E-05	1.26E-04	8.56E-05	1.79E-04
C06E7.1b	1219	1601	1646	2783	2.60E-04	3.22E-04	2.28E-04	4.76E-04
C06E7.1c	220	312	237	372	3.80E-05	5.09E-05	2.67E-05	5.16E-05
C06E7.1d	400	581	490	835	7.44E-05	1.02E-04	5.93E-05	1.25E-04
C06E7.2.1	83	121	49	67	5.40E-06	7.43E-06	2.08E-06	3.51E-06
C06E7.2.2	79	110	43	65	5.63E-06	7.41E-06	1.99E-06	3.71E-06
C06E7.3a	989	1352	1337	2357	8.87E-05	1.15E-04	7.80E-05	1.70E-04
C06E7.3b.1	1108	1514	1467	2539	6.21E-05	8.02E-05	5.35E-05	1.14E-04
C06E7.3b.2	1085	1490	1418	2514	8.08E-05	1.05E-04	6.87E-05	1.50E-04
C06E7.3b.3	999	1352	1350	2355	6.90E-05	8.82E-05	6.07E-05	1.31E-04
C06E7.3b.4	989	1352	1337	2357	8.87E-05	1.15E-04	7.80E-05	1.70E-04
C06E7.4	104	140	75	88	7.31E-06	9.31E-06	3.43E-06	4.97E-06
C06E7.6	5	9	6	3	2.80E-06	2.65E-06	1.82E-06	2.25E-06
C06E7.7	6	8	15	1	2.80E-06	2.65E-06	1.82E-06	2.25E-06
C06E8.3a	87	180	111	167	5.35E-06	1.04E-05	4.43E-06	8.23E-06
C06E8.3b	34	72	38	56	4.26E-06	8.49E-06	3.08E-06	5.62E-06
C06E8.3c	63	141	84	124	4.68E-06	9.87E-06	4.05E-06	7.38E-06
C06E8.5	63	78	25	42	4.23E-06	4.95E-06	1.82E-06	2.27E-06
C06G1.1	164	259	94	79	9.24E-06	1.38E-05	3.44E-06	3.58E-06
C06G1.2	51	36	30	29	1.04E-05	6.93E-06	3.99E-06	4.75E-06
C06G1.4.1	392	471	325	537	1.29E-05	1.46E-05	6.96E-06	1.42E-05
C06G1.4.2	228	276	169	371	1.30E-05	1.48E-05	6.25E-06	1.70E-05
C06G1.5	81	131	77	134	3.78E-06	5.79E-06	2.33E-06	5.04E-06
C06G3.10	373	498	558	714	1.90E-05	2.39E-05	1.85E-05	2.91E-05
C06G3.11a	102	147	201	53	4.19E-05	5.70E-05	5.37E-05	1.75E-05
C06G3.11b.1	145	236	393	92	4.89E-05	7.52E-05	8.63E-05	2.49E-05
C06G3.11b.2	117	185	241	63	4.24E-05	6.33E-05	5.69E-05	1.84E-05
C06G3.1a.1	41	192	17	39	3.25E-06	1.44E-05	1.82E-06	2.47E-06
C06G3.1a.2	38	183	12	36	3.08E-06	1.40E-05	1.82E-06	2.34E-06
C06G3.1b	38	174	11	33	3.42E-06	1.47E-05	1.82E-06	2.38E-06
C06G3.2	1352	1091	2049	2167	4.74E-05	3.61E-05	4.67E-05	6.10E-05
C06G3.3	8	10	16	13	3.39E-06	4.02E-06	4.41E-06	4.43E-06
C06G3.4	9	9	19	16	2.80E-06	2.65E-06	1.82E-06	2.25E-06
C06G3.5a	482	492	385	525	4.07E-05	3.92E-05	2.11E-05	3.56E-05
C06G3.5b.1	462	469	358	490	4.28E-05	4.11E-05	2.16E-05	3.65E-05
C06G3.5b.2	449	444	344	478	4.60E-05	4.30E-05	2.29E-05	3.93E-05
C06G3.6	127	182	85	146	7.59E-06	1.03E-05	3.32E-06	7.02E-06
C06G3.7	221	365	569	747	9.49E-06	1.48E-05	1.59E-05	2.58E-05
C06G3.8	111	105	134	111	2.54E-05	2.27E-05	1.99E-05	2.04E-05
C06G3.9.1	491	578	581	884	2.35E-05	2.62E-05	1.81E-05	3.41E-05
C06G4.1	364	396	470	772	1.48E-05	1.52E-05	1.24E-05	2.52E-05
C06G4.2a.1	670	940	483	997	3.07E-05	4.07E-05	1.44E-05	3.67E-05
C06G4.2a.2	667	932	472	994	3.06E-05	4.04E-05	1.41E-05	3.67E-05
C06G4.2b.1	726	1043	515	1040	3.12E-05	4.24E-05	1.44E-05	3.59E-05

C06G4.2b.2	685	995	481	1004	2.98E-05	4.10E-05	1.36E-05	3.52E-05
C06G4.2c	26	28	28	30	8.51E-06	8.65E-06	5.96E-06	7.89E-06
C06G4.2d	706	1016	500	1027	3.09E-05	4.20E-05	1.42E-05	3.61E-05
C06G4.4	10	18	31	13	2.88E-06	4.89E-06	5.79E-06	2.99E-06
C06G4.5	21	41	21	29	2.80E-06	3.07E-06	1.82E-06	2.25E-06
C06G4.6	10	18	14	11	2.80E-06	3.41E-06	1.82E-06	2.25E-06
C06G8.1	219	303	106	171	1.63E-05	2.13E-05	5.12E-06	1.02E-05
C06G8.2	232	274	254	441	9.88E-06	1.10E-05	7.05E-06	1.51E-05
C06G8.4	10	9	9	5	2.80E-06	2.65E-06	1.82E-06	2.25E-06
C06H2.1.1	1940	2681	2817	2737	2.49E-04	3.25E-04	2.35E-04	2.82E-04
C06H2.1.2	1868	2564	2576	2667	3.17E-04	4.11E-04	2.84E-04	3.64E-04
C06H2.2	94	142	134	169	7.06E-06	1.01E-05	6.54E-06	1.02E-05
C06H2.3	179	202	212	216	1.15E-05	1.23E-05	8.89E-06	1.12E-05
C06H2.4	23	31	30	43	2.80E-06	3.02E-06	2.02E-06	3.58E-06
C06H2.5	4	12	6	9	2.80E-06	2.65E-06	1.82E-06	2.25E-06
C06H2.7	73	106	165	166	8.37E-06	1.15E-05	1.23E-05	1.53E-05
C06H5.1	63	134	50	67	5.71E-06	1.15E-05	2.95E-06	4.88E-06
C06H5.2	56	109	41	49	5.26E-06	9.65E-06	2.50E-06	3.69E-06
C06H5.6	86	136	79	120	6.30E-06	9.39E-06	3.75E-06	7.04E-06
C06H5.7.1	153	292	133	216	1.27E-05	2.28E-05	7.16E-06	1.43E-05
C06H5.7.2	129	252	98	187	1.21E-05	2.23E-05	5.98E-06	1.41E-05
C06H5.8	8	15	13	22	2.80E-06	2.65E-06	1.82E-06	2.25E-06
C07A12.1a	35	38	18	54	3.33E-06	3.41E-06	1.82E-06	4.14E-06
C07A12.1b	19	27	12	38	2.80E-06	2.65E-06	1.82E-06	2.79E-06
C07A12.1c	17	17	11	32	2.80E-06	2.65E-06	1.82E-06	2.90E-06
C07A12.2	3	7	2	1	2.80E-06	3.62E-06	1.82E-06	2.25E-06
C07A12.4a.1	5706	5475	6274	6661	3.43E-04	3.11E-04	2.45E-04	3.21E-04
C07A12.4a.2	5398	5200	6113	6398	3.44E-04	3.13E-04	2.53E-04	3.27E-04
C07A12.4b	4889	4708	5323	5654	4.17E-04	3.79E-04	2.95E-04	3.87E-04
C07A12.5a	141	165	198	280	6.33E-06	6.98E-06	5.78E-06	1.01E-05
C07A12.5b	133	156	182	249	6.44E-06	7.14E-06	5.74E-06	9.69E-06
C07A12.7a.1	113	172	99	130	8.54E-06	1.23E-05	4.88E-06	7.89E-06
C07A12.7a.2	101	148	88	117	8.60E-06	1.19E-05	4.88E-06	8.01E-06
C07A12.7b	83	119	70	98	7.67E-06	1.04E-05	4.21E-06	7.26E-06
C07A12.7c.1	83	119	70	98	7.67E-06	1.04E-05	4.21E-06	7.26E-06
C07A12.7c.2	41	53	36	45	7.31E-06	8.94E-06	4.17E-06	6.45E-06
C07A4.1	32	42	29	38	4.40E-06	5.48E-06	2.61E-06	4.21E-06
C07A4.2	10	10	3	8	2.80E-06	2.65E-06	1.82E-06	2.25E-06
C07A4.3	16	15	15	14	2.80E-06	2.65E-06	1.82E-06	2.25E-06
C07A9.1	12	8	7	6	2.80E-06	2.65E-06	1.82E-06	2.25E-06
C07A9.10	4	4	1	1	2.80E-06	2.65E-06	1.82E-06	2.25E-06
C07A9.11	132	146	64	172	6.72E-06	7.04E-06	2.11E-06	7.04E-06
C07A9.12	13	13	17	15	2.80E-06	2.65E-06	1.82E-06	2.25E-06
C07A9.13	6	16	5	7	2.80E-06	2.65E-06	1.82E-06	2.25E-06
C07A9.2.1	241	332	444	346	4.58E-05	5.96E-05	5.49E-05	5.29E-05
C07A9.2.2	174	249	304	228	2.31E-05	3.12E-05	2.62E-05	2.43E-05
C07A9.3a	2304	1988	2697	3830	6.63E-05	5.40E-05	5.05E-05	8.85E-05
C07A9.3b	1838	1563	2129	2967	6.25E-05	5.02E-05	4.71E-05	8.10E-05
C07A9.3c	1514	1288	1828	2565	6.32E-05	5.08E-05	4.97E-05	8.60E-05
C07A9.4	65	53	32	47	4.12E-06	3.17E-06	1.82E-06	2.38E-06
C07A9.5	6	5	5	6	2.80E-06	2.65E-06	1.82E-06	2.25E-06
C07A9.7a	290	274	362	500	2.09E-05	1.86E-05	1.70E-05	2.89E-05
C07A9.7b	298	282	391	503	2.07E-05	1.85E-05	1.77E-05	2.80E-05
C07A9.8	32	55	15	15	2.80E-06	4.05E-06	1.82E-06	2.25E-06
C07A9.9	43	61	49	59	9.91E-06	1.33E-05	7.34E-06	1.09E-05
C07B5.2	3	5	5	0	2.80E-06	2.65E-06	1.82E-06	2.25E-06
C07B5.3	3	1	1	2	2.80E-06	2.65E-06	1.82E-06	2.25E-06
C07B5.4b.1	21	58	40	26	2.80E-06	3.62E-06	1.82E-06	2.25E-06
C07B5.4b.2	20	55	38	26	2.80E-06	3.60E-06	1.82E-06	2.25E-06

C07B5.5	479	531	621	535	4.61E-05	4.83E-05	3.89E-05	4.14E-05
C07B5.6	6	6	4	5	2.80E-06	2.65E-06	1.82E-06	2.25E-06
C07C7.1	10	7	12	13	2.80E-06	2.65E-06	1.82E-06	2.25E-06
C07D10.1	15	35	11	19	2.80E-06	3.33E-06	1.82E-06	2.25E-06
C07D10.2a	433	408	510	719	4.12E-05	3.67E-05	3.16E-05	5.50E-05
C07D10.2b.1	628	626	732	1027	4.09E-05	3.86E-05	3.10E-05	5.38E-05
C07D10.2b.2	431	407	511	723	4.03E-05	3.60E-05	3.11E-05	5.43E-05
C07D10.3	7	8	10	5	2.80E-06	2.65E-06	1.82E-06	2.25E-06
C07D10.4	65	118	31	47	6.33E-06	1.09E-05	1.97E-06	3.69E-06
C07D10.5	82	69	77	104	7.00E-06	5.58E-06	4.28E-06	7.15E-06
C07D10.6	2	2	1	1	2.80E-06	2.65E-06	1.82E-06	2.25E-06
C07D8.2	0	2	1	0	2.80E-06	2.65E-06	1.82E-06	2.25E-06
C07D8.3	1	0	0	1	2.80E-06	2.65E-06	1.82E-06	2.25E-06
C07D8.5	1	1	1	3	2.80E-06	2.65E-06	1.82E-06	2.25E-06
C07E3.10	44	63	57	17	1.05E-05	1.42E-05	8.84E-06	3.26E-06
C07E3.1a	395	389	433	633	1.74E-05	1.62E-05	1.24E-05	2.24E-05
C07E3.1b	204	204	215	359	1.64E-05	1.55E-05	1.12E-05	2.32E-05
C07E3.2.1	566	590	516	810	2.75E-05	2.71E-05	1.63E-05	3.16E-05
C07E3.2.2	528	562	472	765	2.75E-05	2.77E-05	1.60E-05	3.20E-05
C07E3.3	35	66	28	29	3.30E-06	5.90E-06	1.82E-06	2.25E-06
C07E3.4	70	76	30	33	4.87E-06	5.00E-06	1.82E-06	2.25E-06
C07E3.5	2	4	24	5	2.80E-06	2.65E-06	1.82E-06	2.25E-06
C07E3.6	91	43	106	102	1.02E-05	4.55E-06	7.73E-06	9.18E-06
C07E3.8	7	4	5	3	2.80E-06	2.65E-06	1.82E-06	2.25E-06
C07E3.9	55	136	61	63	1.33E-05	3.11E-05	9.62E-06	1.23E-05
C07F11.1	415	436	687	971	1.15E-05	1.13E-05	1.23E-05	2.15E-05
C07F11.2	13	13	12	7	2.80E-06	2.65E-06	1.82E-06	2.25E-06
C07G1.1	2	15	11	2	2.80E-06	2.65E-06	1.82E-06	2.25E-06
C07G1.2	22	29	10	9	2.80E-06	2.65E-06	1.82E-06	2.25E-06
C07G1.3a.1	653	746	866	1187	3.27E-05	3.53E-05	2.82E-05	4.77E-05
C07G1.3a.2	637	719	826	1157	3.26E-05	3.48E-05	2.75E-05	4.76E-05
C07G1.3b	497	575	655	890	2.24E-05	2.44E-05	1.92E-05	3.22E-05
C07G1.3c	432	499	582	784	2.40E-05	2.62E-05	2.10E-05	3.49E-05
C07G1.4a	508	473	658	990	2.49E-05	2.19E-05	2.10E-05	3.90E-05
C07G1.4b	262	272	351	581	1.25E-05	1.23E-05	1.09E-05	2.23E-05
C07G1.6	16	28	6	7	4.45E-06	7.38E-06	1.82E-06	2.25E-06
C07G1.7	4	3	3	5	2.80E-06	2.65E-06	1.82E-06	2.25E-06
C07G1.8	116	161	207	91	3.28E-05	4.30E-05	3.81E-05	2.07E-05
C07G2.1a.1	10445	8979	16996	19976	6.22E-04	5.05E-04	6.59E-04	9.56E-04
C07G2.1a.2	10183	8776	16529	19735	6.48E-04	5.28E-04	6.85E-04	1.01E-03
C07G2.2a.1	1611	1757	2237	2908	8.38E-05	8.64E-05	7.58E-05	1.22E-04
C07G2.2a.2	1523	1690	2116	2849	8.08E-05	8.47E-05	7.30E-05	1.21E-04
C07G2.2a.3	1039	1119	1401	1831	5.44E-05	5.54E-05	4.78E-05	7.71E-05
C07G2.2b	1066	1147	1431	1877	8.04E-05	8.17E-05	7.02E-05	1.14E-04
C07G2.2c.1	1518	1722	2068	2856	7.62E-05	8.16E-05	6.75E-05	1.15E-04
C07G2.2c.2	1006	1138	1405	1862	7.03E-05	7.51E-05	6.39E-05	1.05E-04
C07G2.3a	2682	3041	2306	3752	1.81E-04	1.94E-04	1.01E-04	2.03E-04
C07G2.3b.1	3168	3693	2755	4335	1.84E-04	2.02E-04	1.04E-04	2.02E-04
C07G2.3b.2	3079	3556	2661	4215	1.88E-04	2.05E-04	1.06E-04	2.07E-04
C07G2.3b.3	2624	3094	2295	3786	1.88E-04	2.09E-04	1.07E-04	2.17E-04
C07G2.3b.4	2215	2527	1918	3237	1.92E-04	2.06E-04	1.08E-04	2.25E-04
C07G2.3b.5	2221	2514	1903	3155	1.95E-04	2.09E-04	1.09E-04	2.23E-04
C07G2.3b.6	2682	3041	2306	3752	1.81E-04	1.94E-04	1.01E-04	2.03E-04
C07G3.10	2	2	3	1	2.80E-06	2.65E-06	1.82E-06	2.25E-06
C07G3.11	0	3	1	0	2.80E-06	2.65E-06	1.82E-06	2.25E-06
C07G3.2	24	9	15	5	4.20E-06	2.65E-06	1.82E-06	2.25E-06
C07G3.3	4	2	4	0	2.80E-06	2.65E-06	1.82E-06	2.25E-06
C07G3.4	2	2	5	4	2.80E-06	2.65E-06	1.82E-06	2.25E-06
C07G3.5	2	2	6	2	2.80E-06	2.65E-06	1.82E-06	2.25E-06

C07G3.6	5	2	4	0	2.80E-06	2.65E-06	1.82E-06	2.25E-06
C07G3.7	2	4	3	2	2.80E-06	2.65E-06	1.82E-06	2.25E-06
C07G3.8	6	5	12	4	2.80E-06	2.65E-06	1.82E-06	2.25E-06
C07H4.1	17	50	12	17	3.56E-06	9.84E-06	1.82E-06	2.86E-06
C07H6.1	188	210	137	280	9.02E-06	9.52E-06	4.28E-06	1.08E-05
C07H6.2	218	219	523	151	5.61E-05	5.33E-05	8.76E-05	3.12E-05
C07H6.4	484	560	561	876	1.88E-05	2.05E-05	1.41E-05	2.73E-05
C07H6.5.1	17368	13707	12826	24546	8.61E-04	6.42E-04	4.14E-04	9.77E-04
C07H6.5.2	9092	7511	6455	12481	4.51E-04	3.52E-04	2.08E-04	4.97E-04
C07H6.6	331	355	454	708	1.32E-05	1.34E-05	1.18E-05	2.27E-05
C07H6.7.1	82	85	61	94	7.00E-06	6.85E-06	3.39E-06	6.45E-06
C07H6.7.2	82	84	65	93	7.34E-06	7.09E-06	3.79E-06	6.68E-06
C07H6.9	10	17	2	8	2.80E-06	3.91E-06	1.82E-06	2.25E-06
C08A9.10	122	132	143	169	3.25E-05	3.32E-05	2.48E-05	3.62E-05
C08A9.2	0	4	0	0	2.80E-06	2.65E-06	1.82E-06	2.25E-06
C08A9.3	4	5	7	3	2.80E-06	2.65E-06	1.82E-06	2.25E-06
C08A9.6	35	31	44	51	3.22E-06	2.67E-06	2.62E-06	3.76E-06
C08A9.7	8	9	14	13	2.80E-06	2.65E-06	1.82E-06	2.25E-06
C08A9.8	24	15	25	24	2.80E-06	2.65E-06	1.82E-06	2.25E-06
C08A9.9	10	10	8	16	2.80E-06	2.65E-06	1.82E-06	2.25E-06
C08B11.1	1114	1325	1448	2199	4.46E-05	5.02E-05	3.78E-05	7.08E-05
C08B11.2	153	190	200	364	1.03E-05	1.21E-05	8.78E-06	1.97E-05
C08B11.3	1720	1615	2444	3501	4.40E-05	3.90E-05	4.07E-05	7.19E-05
C08B11.4	799	1097	973	1342	3.48E-05	4.51E-05	2.76E-05	4.69E-05
C08B11.5.1	516	460	527	793	4.40E-05	3.71E-05	2.93E-05	5.44E-05
C08B11.5.2	494	445	481	750	4.29E-05	3.65E-05	2.72E-05	5.23E-05
C08B11.5.3	802	793	867	1183	7.70E-05	7.19E-05	5.42E-05	9.12E-05
C08B11.6.1	802	793	867	1183	2.84E-05	2.65E-05	2.00E-05	3.36E-05
C08B11.7	462	602	422	483	4.64E-05	5.71E-05	2.76E-05	3.89E-05
C08B11.8	266	275	345	378	1.97E-05	1.93E-05	1.66E-05	2.25E-05
C08B11.9	871	876	1033	1248	2.85E-05	2.71E-05	2.20E-05	3.28E-05
C08B6.10	69	87	112	70	1.55E-05	1.84E-05	1.63E-05	1.26E-05
C08B6.11	43	73	23	42	3.22E-06	5.16E-06	1.82E-06	2.52E-06
C08B6.12	6	5	6	6	2.80E-06	2.65E-06	1.82E-06	2.25E-06
C08B6.13	8	6	6	3	2.80E-06	2.65E-06	1.82E-06	2.25E-06
C08B6.14	1	2	6	4	2.80E-06	2.65E-06	1.82E-06	2.25E-06
C08B6.2	13	33	41	29	2.80E-06	2.70E-06	2.31E-06	2.25E-06
C08B6.3	47	64	40	45	3.53E-06	4.52E-06	1.95E-06	2.70E-06
C08B6.4a	29	34	31	21	2.80E-06	2.65E-06	1.82E-06	2.25E-06
C08B6.4b	27	36	20	15	2.80E-06	2.65E-06	1.82E-06	2.25E-06
C08B6.5	5	11	12	7	2.80E-06	2.65E-06	1.82E-06	2.25E-06
C08B6.6	5	6	7	3	2.80E-06	2.65E-06	1.82E-06	2.25E-06
C08B6.7	1031	925	1445	1997	4.24E-05	3.60E-05	3.87E-05	6.60E-05
C08B6.8	214	231	207	176	3.87E-05	3.94E-05	2.43E-05	2.55E-05
C08B6.9	424	579	506	717	3.93E-05	5.07E-05	3.05E-05	5.33E-05
C08C3.1a	2	5	7	6	2.80E-06	2.65E-06	1.82E-06	2.25E-06
C08C3.1b	23	23	40	31	2.80E-06	2.65E-06	1.88E-06	2.25E-06
C08C3.1c	3	6	5	5	2.80E-06	2.65E-06	1.82E-06	2.25E-06
C08C3.2	94	128	169	187	1.13E-05	1.45E-05	1.32E-05	1.80E-05
C08C3.3	28	53	55	34	3.05E-06	5.42E-06	3.88E-06	2.97E-06
C08C3.4a	623	557	861	970	7.50E-05	6.34E-05	6.75E-05	9.38E-05
C08C3.4b	443	387	535	663	6.64E-05	5.48E-05	5.22E-05	7.98E-05
C08D8.1	10	8	17	6	2.80E-06	2.65E-06	1.82E-06	2.25E-06
C08D8.2a	44	80	42	50	4.82E-06	8.28E-06	2.99E-06	4.39E-06
C08D8.2b	91	135	81	110	4.03E-06	5.66E-06	2.35E-06	3.94E-06
C08E3.1	67	18	337	10	3.17E-05	8.04E-06	1.04E-04	3.80E-06
C08E3.10a	5	6	5	3	2.80E-06	2.65E-06	1.82E-06	2.25E-06
C08E3.10b	4	4	4	3	2.80E-06	2.65E-06	1.82E-06	2.25E-06
C08E3.11	6	5	8	2	2.80E-06	2.65E-06	1.82E-06	2.25E-06

C08E3.12	0	1	1	3	2.80E-06	2.65E-06	1.82E-06	2.25E-06
C08E3.13	79	30	458	13	3.11E-05	1.11E-05	1.17E-04	4.09E-06
C08E3.14	1	3	5	1	2.80E-06	2.65E-06	1.82E-06	2.25E-06
C08E3.15	0	0	0	1	2.80E-06	2.65E-06	1.82E-06	2.25E-06
C08E3.2	3	3	1	1	2.80E-06	2.65E-06	1.82E-06	2.25E-06
C08E3.3	0	5	2	1	2.80E-06	2.65E-06	1.82E-06	2.25E-06
C08E3.4	5	6	4	2	2.80E-06	2.65E-06	1.82E-06	2.25E-06
C08E3.5	6	9	5	3	2.80E-06	2.65E-06	1.82E-06	2.25E-06
C08E3.6	6	5	8	3	2.80E-06	2.65E-06	1.82E-06	2.25E-06
C08E3.7	3	5	10	4	2.80E-06	2.65E-06	1.82E-06	2.25E-06
C08E3.8	2	3	1	2	2.80E-06	2.65E-06	1.82E-06	2.25E-06
C08E3.9	7	12	7	2	2.80E-06	2.65E-06	1.82E-06	2.25E-06
C08E8.1	3	3	4	4	2.80E-06	2.65E-06	1.82E-06	2.25E-06
C08E8.2	1	3	5	4	2.80E-06	2.65E-06	1.82E-06	2.25E-06
C08E8.3	3	18	2	4	2.80E-06	2.65E-06	1.82E-06	2.25E-06
C08E8.4	14	17	6	6	3.44E-06	3.94E-06	1.82E-06	2.25E-06
C08E8.5	0	2	1	1	2.80E-06	2.65E-06	1.82E-06	2.25E-06
C08E8.6	0	0	1	0	2.80E-06	2.65E-06	1.82E-06	2.25E-06
C08E8.t1	1	0	0	0	2.80E-06	2.65E-06	1.82E-06	2.25E-06
C08E8.t3	1	0	0	0	2.80E-06	2.65E-06	1.82E-06	2.25E-06
C08F1.1	12	17	19	3	2.80E-06	2.65E-06	1.82E-06	2.25E-06
C08F1.10	18	16	25	14	2.80E-06	2.65E-06	1.95E-06	2.25E-06
C08F1.11	5	7	1	8	2.80E-06	2.65E-06	1.82E-06	2.25E-06
C08F1.2	2	4	2	2	2.80E-06	2.65E-06	1.82E-06	2.25E-06
C08F1.3	5	4	3	2	2.80E-06	2.65E-06	1.82E-06	2.25E-06
C08F1.4a	44	127	35	59	2.80E-06	7.30E-06	1.82E-06	2.88E-06
C08F1.4b	44	128	22	57	2.80E-06	7.43E-06	1.82E-06	2.81E-06
C08F1.5a	73	102	59	85	3.14E-06	4.13E-06	1.82E-06	2.92E-06
C08F1.5b	62	95	57	80	2.80E-06	3.70E-06	1.82E-06	2.65E-06
C08F1.6	10	13	11	8	2.80E-06	2.65E-06	1.82E-06	2.25E-06
C08F1.7	5	1	9	2	2.80E-06	2.65E-06	1.82E-06	2.25E-06
C08F1.8	6	6	6	8	2.80E-06	2.65E-06	1.82E-06	2.25E-06
C08F11.1	11	15	18	4	2.80E-06	2.65E-06	1.82E-06	2.25E-06
C08F11.10	22	51	16	7	3.14E-06	6.88E-06	1.82E-06	2.25E-06
C08F11.11	2742	4907	6690	5774	8.37E-04	1.41E-03	1.33E-03	1.42E-03
C08F11.12	329	712	394	600	9.99E-05	2.04E-04	7.78E-05	1.46E-04
C08F11.13.1	81	237	49	46	6.58E-06	1.82E-05	2.59E-06	3.01E-06
C08F11.13.2	78	225	40	37	6.94E-06	1.89E-05	2.31E-06	2.65E-06
C08F11.14	13	39	13	23	2.80E-06	2.65E-06	1.82E-06	2.25E-06
C08F11.2	9	12	14	4	2.80E-06	2.65E-06	1.82E-06	2.25E-06
C08F11.3	12	13	10	7	2.80E-06	2.65E-06	1.82E-06	2.25E-06
C08F11.4	7	7	17	4	2.80E-06	2.65E-06	1.82E-06	2.25E-06
C08F11.5	24	27	19	13	2.80E-06	2.78E-06	1.82E-06	2.25E-06
C08F11.6	23	21	30	34	6.19E-06	5.34E-06	5.25E-06	7.35E-06
C08F11.7	0	10	3	4	2.80E-06	2.65E-06	1.82E-06	2.25E-06
C08F11.8	1235	1692	1092	1358	6.81E-05	8.81E-05	3.92E-05	6.01E-05
C08F11.9	10	10	23	8	2.80E-06	2.65E-06	1.82E-06	2.25E-06
C08F8.1	419	494	517	277	1.01E-04	1.12E-04	8.10E-05	5.36E-05
C08F8.2a	312	371	322	497	1.42E-05	1.60E-05	9.57E-06	1.82E-05
C08F8.3	409	331	599	699	3.45E-05	2.64E-05	3.29E-05	4.73E-05
C08F8.4	15	37	16	11	2.80E-06	2.65E-06	1.82E-06	2.25E-06
C08F8.5	12	7	14	11	2.80E-06	2.65E-06	1.82E-06	2.25E-06
C08F8.6	48	94	25	21	4.70E-06	8.70E-06	1.82E-06	2.25E-06
C08F8.7	23	39	11	16	4.17E-06	6.67E-06	1.82E-06	2.34E-06
C08F8.8	19	29	12	20	2.80E-06	2.65E-06	1.82E-06	2.25E-06
C08F8.9	179	215	506	166	7.57E-05	8.58E-05	1.39E-04	5.64E-05
C08G5.1	13	17	19	6	2.80E-06	2.65E-06	1.82E-06	2.25E-06
C08G5.2	4	7	1	1	2.80E-06	2.65E-06	1.82E-06	2.25E-06
C08G5.3	6	8	5	3	2.80E-06	2.65E-06	1.82E-06	2.25E-06

C08G5.4	7	15	10	16	2.80E-06	2.65E-06	1.82E-06	2.25E-06
C08G5.5	5	13	15	5	2.80E-06	4.58E-06	3.64E-06	2.25E-06
C08G5.6	85	63	261	237	1.03E-05	7.25E-06	2.07E-05	2.31E-05
C08G5.7a	6	7	0	5	2.80E-06	2.65E-06	1.82E-06	2.25E-06
C08G5.7b	1	2	0	0	2.80E-06	2.65E-06	1.82E-06	2.25E-06
C08G9.1	34	132	43	65	4.45E-06	1.63E-05	3.66E-06	6.81E-06
C08G9.2	46	44	35	35	2.80E-06	2.65E-06	1.82E-06	2.25E-06
C08H9.1	13	13	12	9	2.80E-06	2.65E-06	1.82E-06	2.25E-06
C08H9.10	11	4	8	8	2.80E-06	2.65E-06	1.82E-06	2.25E-06
C08H9.11	7	11	9	3	2.80E-06	2.65E-06	1.82E-06	2.25E-06
C08H9.12	3	9	9	8	2.80E-06	2.65E-06	1.82E-06	2.25E-06
C08H9.13	11	13	6	2	2.80E-06	2.65E-06	1.82E-06	2.25E-06
C08H9.14	9	13	16	8	2.80E-06	2.65E-06	1.82E-06	2.25E-06
C08H9.15	56	69	20	37	1.58E-05	1.84E-05	3.68E-06	8.41E-06
C08H9.2	10290	9525	10691	15388	2.68E-04	2.34E-04	1.81E-04	3.22E-04
C08H9.3a	167	176	183	310	7.84E-06	7.80E-06	5.59E-06	1.17E-05
C08H9.3b	44	45	47	76	8.18E-06	7.91E-06	5.69E-06	1.13E-05
C08H9.4a	2	6	2	2	2.80E-06	2.65E-06	1.82E-06	2.25E-06
C08H9.4b	6	13	7	5	2.80E-06	2.65E-06	1.82E-06	2.25E-06
C08H9.5	17	21	23	10	2.80E-06	2.65E-06	1.82E-06	2.25E-06
C08H9.6	5	3	4	4	2.80E-06	2.65E-06	1.82E-06	2.25E-06
C08H9.7	18	30	38	30	2.80E-06	2.65E-06	1.93E-06	2.25E-06
C08H9.8	3	7	2	2	2.80E-06	2.65E-06	1.82E-06	2.25E-06
C08H9.9	7	16	9	10	2.80E-06	2.65E-06	1.82E-06	2.25E-06
C09B7.1a	16	12	21	15	2.80E-06	2.65E-06	1.82E-06	2.25E-06
C09B7.1b	15	12	21	15	2.80E-06	2.65E-06	1.82E-06	2.25E-06
C09B7.1c	27	21	32	27	2.80E-06	2.65E-06	1.82E-06	2.25E-06
C09B7.2	61	70	66	42	9.30E-06	1.01E-05	6.54E-06	5.15E-06
C09B7.3	5	4	4	4	2.80E-06	2.65E-06	1.82E-06	2.25E-06
C09B7.4	1	4	2	1	2.80E-06	2.65E-06	1.82E-06	2.25E-06
C09B8.1	94	122	117	156	6.24E-06	7.64E-06	5.05E-06	8.32E-06
C09B8.3	10	12	5	4	2.80E-06	2.65E-06	1.82E-06	2.25E-06
C09B8.4	19	15	16	32	2.80E-06	2.65E-06	1.82E-06	3.31E-06
C09B8.5	26	22	30	54	2.97E-06	2.65E-06	2.22E-06	4.95E-06
C09B8.6a	506	604	434	630	8.59E-05	9.68E-05	4.79E-05	8.59E-05
C09B8.6b	475	567	408	603	8.55E-05	9.64E-05	4.78E-05	8.72E-05
C09B8.6c.1	718	892	642	875	8.93E-05	1.05E-04	5.19E-05	8.74E-05
C09B8.6c.2	709	861	628	882	8.53E-05	9.78E-05	4.92E-05	8.52E-05
C09B8.6c.3	568	686	546	772	8.45E-05	9.64E-05	5.28E-05	9.22E-05
C09B8.6c.4	475	567	408	603	8.55E-05	9.64E-05	4.78E-05	8.72E-05
C09B8.7a.1	163	219	208	291	1.06E-05	1.34E-05	8.76E-06	1.51E-05
C09B8.7a.2	156	210	201	287	1.01E-05	1.29E-05	8.47E-06	1.49E-05
C09B8.7b	147	198	194	285	7.17E-06	9.10E-06	6.14E-06	1.12E-05
C09B8.7c.1	148	203	196	287	7.20E-06	9.34E-06	6.21E-06	1.12E-05
C09B8.7c.2	143	194	189	280	9.83E-06	1.26E-05	8.45E-06	1.55E-05
C09B8.7e.1	87	123	109	155	9.55E-06	1.27E-05	7.78E-06	1.37E-05
C09B8.7e.2	220	293	273	366	1.06E-05	1.34E-05	8.58E-06	1.42E-05
C09B8.8	9	10	10	10	2.80E-06	2.65E-06	1.82E-06	2.25E-06
C09B9.1	6	4	11	4	2.80E-06	2.65E-06	1.82E-06	2.25E-06
C09B9.2	32	57	13	24	3.64E-06	6.11E-06	1.82E-06	2.25E-06
C09B9.3	18	40	23	12	2.80E-06	2.65E-06	1.82E-06	2.25E-06
C09B9.4	35	60	25	32	3.39E-06	5.50E-06	1.82E-06	2.50E-06
C09B9.6	2053	2729	3127	728	5.79E-04	7.27E-04	5.74E-04	1.65E-04
C09B9.7	33	56	17	20	2.80E-06	2.65E-06	1.82E-06	2.25E-06
C09B9.8	6	13	4	5	2.80E-06	2.65E-06	1.82E-06	2.25E-06
C09B9.t1	1	0	3	0	2.80E-06	2.65E-06	2.99E-06	2.25E-06
C09C7.1	11	22	8	13	2.80E-06	2.65E-06	1.82E-06	2.25E-06
C09D1.1a	1458	1702	1218	1984	8.20E-06	9.05E-06	4.46E-06	8.97E-06
C09D1.1b	1928	2251	1494	2627	8.74E-06	9.65E-06	4.41E-06	9.58E-06

C09D1.1c	413	464	243	574	1.09E-05	1.15E-05	4.15E-06	1.21E-05
C09D1.1d	408	461	243	573	1.06E-05	1.13E-05	4.12E-06	1.20E-05
C09D1.1e	1547	1780	1270	2094	9.30E-06	1.01E-05	4.96E-06	1.01E-05
C09D1.1f	1819	2124	1422	2523	9.13E-06	1.01E-05	4.65E-06	1.02E-05
C09D1.1g	1566	1845	1298	2166	8.20E-06	9.13E-06	4.43E-06	9.11E-06
C09D1.2	7	11	1	3	2.80E-06	2.65E-06	1.82E-06	2.25E-06
C09D4.1a	77	162	69	150	3.92E-06	7.78E-06	2.28E-06	6.12E-06
C09D4.1b.1	54	122	38	106	3.53E-06	7.51E-06	1.82E-06	5.56E-06
C09D4.1b.2	54	122	38	106	3.53E-06	7.51E-06	1.82E-06	5.56E-06
C09D4.2	105	163	98	246	1.54E-05	2.25E-05	9.33E-06	2.89E-05
C09D4.3	45	99	32	29	3.56E-06	7.41E-06	1.82E-06	2.25E-06
C09D4.4a	517	553	638	1089	2.11E-05	2.13E-05	1.70E-05	3.57E-05
C09D4.4b	520	553	634	1097	2.12E-05	2.13E-05	1.68E-05	3.59E-05
C09D4.4c	567	633	786	1243	2.02E-05	2.13E-05	1.82E-05	3.56E-05
C09D4.5.1	9433	9908	15138	9304	1.54E-03	1.53E-03	1.61E-03	1.22E-03
C09D4.5.2	7282	7646	11116	8338	1.20E-03	1.19E-03	1.19E-03	1.11E-03
C09D4.5.3	7246	7613	11064	8312	1.29E-03	1.28E-03	1.29E-03	1.19E-03
C09D4.6	1	5	6	2	2.80E-06	2.65E-06	1.82E-06	2.25E-06
C09D8.1a	406	551	415	767	6.50E-06	8.33E-06	4.32E-06	9.85E-06
C09D8.1b	344	441	348	648	8.74E-06	1.06E-05	5.76E-06	1.32E-05
C09D8.1c	305	376	295	542	9.35E-06	1.09E-05	5.89E-06	1.33E-05
C09E10.2a	34	36	26	48	2.80E-06	2.65E-06	1.82E-06	2.25E-06
C09E10.2b	34	33	26	48	2.80E-06	2.65E-06	1.82E-06	2.25E-06
C09E10.2c	30	30	24	42	2.80E-06	2.65E-06	1.82E-06	2.25E-06
C09E10.2d	30	27	24	42	2.80E-06	2.65E-06	1.82E-06	2.25E-06
C09E10.2e	34	33	23	46	2.80E-06	2.65E-06	1.82E-06	2.25E-06
C09E7.1	5	3	3	3	2.80E-06	2.65E-06	1.82E-06	2.25E-06
C09E7.10	2	1	8	4	2.80E-06	2.65E-06	1.82E-06	2.25E-06
C09E7.2	7	12	12	5	2.80E-06	2.65E-06	1.82E-06	2.25E-06
C09E7.3	3	9	4	4	2.80E-06	2.65E-06	1.82E-06	2.25E-06
C09E7.4	84	115	111	131	4.76E-06	6.14E-06	4.08E-06	5.96E-06
C09E7.5	26	17	12	14	2.80E-06	2.65E-06	1.82E-06	2.25E-06
C09E7.6	14	20	10	27	2.88E-06	3.89E-06	1.82E-06	4.48E-06
C09E7.8a	301	345	426	642	9.72E-06	1.05E-05	8.95E-06	1.66E-05
C09E7.8b	290	334	406	628	9.94E-06	1.08E-05	9.04E-06	1.73E-05
C09E7.9	152	175	167	330	6.33E-06	6.88E-06	4.52E-06	1.10E-05
C09E8.1a	161	202	228	239	7.03E-06	8.33E-06	6.47E-06	8.37E-06
C09E8.1b	143	178	198	219	6.86E-06	8.07E-06	6.18E-06	8.43E-06
C09E8.2a	18	35	7	11	2.80E-06	4.05E-06	1.82E-06	2.25E-06
C09E8.2b	8	17	3	6	2.80E-06	2.65E-06	1.82E-06	2.25E-06
C09E9.1	46	73	122	46	8.65E-06	1.30E-05	1.49E-05	6.95E-06
C09E9.2.1	13	41	20	24	2.80E-06	5.74E-06	1.93E-06	2.86E-06
C09E9.2.2	16	50	24	30	2.80E-06	6.16E-06	2.04E-06	3.15E-06
C09F12.2	15	24	10	5	2.80E-06	2.65E-06	1.82E-06	2.25E-06
C09F12.3	6	7	7	2	2.80E-06	2.65E-06	1.82E-06	2.25E-06
C09F5.1	63	125	111	125	3.61E-06	6.80E-06	4.15E-06	5.78E-06
C09F5.2	76	70	77	67	9.04E-06	7.88E-06	5.98E-06	6.41E-06
C09F5.3	9	6	4	3	2.80E-06	2.65E-06	1.82E-06	2.25E-06
C09F9.1	6	12	7	2	2.80E-06	3.20E-06	1.82E-06	2.25E-06
C09F9.2	334	312	213	248	7.59E-06	6.69E-06	3.15E-06	4.52E-06
C09F9.3	130	148	104	211	3.70E-06	3.97E-06	1.93E-06	4.81E-06
C09F9.4	60	56	44	47	8.82E-06	7.78E-06	4.21E-06	5.56E-06
C09G1.1	8	10	11	5	2.80E-06	2.65E-06	1.82E-06	2.25E-06
C09G1.2	20	26	20	9	2.80E-06	2.65E-06	1.82E-06	2.25E-06
C09G1.3	8	5	11	5	2.80E-06	2.65E-06	1.82E-06	2.25E-06
C09G1.4	28	28	32	34	2.80E-06	2.65E-06	1.82E-06	2.25E-06
C09G1.5	15	21	39	18	2.80E-06	3.04E-06	3.90E-06	2.25E-06
C09G12.1	1	2	10	2	2.80E-06	2.65E-06	1.82E-06	2.25E-06
C09G12.10	3	1	10	5	2.80E-06	2.65E-06	1.82E-06	2.25E-06

C09G12.11	7	5	7	9	2.80E-06	2.65E-06	1.82E-06	2.25E-06
C09G12.12	4	1	5	2	2.80E-06	2.65E-06	1.82E-06	2.25E-06
C09G12.13	7	7	8	7	2.80E-06	2.65E-06	1.82E-06	2.25E-06
C09G12.14	0	1	0	0	2.80E-06	2.65E-06	1.82E-06	2.25E-06
C09G12.15	6	5	4	4	2.80E-06	2.65E-06	1.82E-06	2.25E-06
C09G12.16	2	3	7	3	2.80E-06	2.65E-06	1.82E-06	2.25E-06
C09G12.17	5	0	10	3	2.80E-06	2.65E-06	1.82E-06	2.25E-06
C09G12.2	2	4	7	2	2.80E-06	2.65E-06	1.82E-06	2.25E-06
C09G12.3	2	3	2	4	2.80E-06	2.65E-06	1.82E-06	2.25E-06
C09G12.4	8	3	1	2	2.80E-06	2.65E-06	1.82E-06	2.25E-06
C09G12.5	5	6	1	1	2.80E-06	2.65E-06	1.82E-06	2.25E-06
C09G12.6	5	6	1942	311	2.80E-06	2.65E-06	1.58E-04	3.13E-05
C09G12.8a	15	23	39	43	4.17E-06	6.03E-06	7.03E-06	9.58E-06
C09G12.8b	349	235	720	350	6.41E-05	4.08E-05	8.60E-05	5.16E-05
C09G12.9	162	145	329	477	1.42E-05	1.20E-05	1.88E-05	3.36E-05
C09G4.1.1	460	472	652	894	3.37E-05	3.27E-05	3.11E-05	5.26E-05
C09G4.1.2	358	351	474	707	3.43E-05	3.18E-05	2.96E-05	5.44E-05
C09G4.2a	254	527	213	449	1.57E-05	3.07E-05	8.56E-06	2.23E-05
C09G4.2b.1	243	474	196	407	2.24E-05	4.13E-05	1.18E-05	3.01E-05
C09G4.2b.2	229	453	183	400	2.57E-05	4.81E-05	1.34E-05	3.61E-05
C09G4.2c	347	687	280	637	1.70E-05	3.17E-05	8.91E-06	2.50E-05
C09G4.2d.1	142	326	127	304	2.21E-05	4.80E-05	1.29E-05	3.80E-05
C09G4.2d.2	159	365	148	335	2.32E-05	5.04E-05	1.41E-05	3.93E-05
C09G4.2d.3	252	513	209	442	2.60E-05	4.99E-05	1.40E-05	3.66E-05
C09G4.2d.4	229	453	183	400	2.57E-05	4.81E-05	1.34E-05	3.61E-05
C09G4.3	767	844	924	1059	1.39E-04	1.45E-04	1.09E-04	1.54E-04
C09G4.4	78	102	104	109	2.20E-05	2.71E-05	1.90E-05	2.46E-05
C09G5.1	22	20	33	26	2.80E-06	2.65E-06	1.82E-06	2.25E-06
C09G5.2	241	211	233	315	1.80E-05	1.49E-05	1.13E-05	1.89E-05
C09G5.3	79	127	37	30	1.04E-05	1.58E-05	3.17E-06	3.17E-06
C09G5.4	933	951	1123	679	1.00E-04	9.67E-05	7.86E-05	5.87E-05
C09G5.5	4450	5784	8803	8080	4.89E-04	6.00E-04	6.29E-04	7.13E-04
C09G5.6	808	805	226	178	3.04E-05	2.86E-05	5.52E-06	5.38E-06
C09G5.7	14	32	13	10	2.80E-06	2.86E-06	1.82E-06	2.25E-06
C09G5.8	31	36	39	29	2.80E-06	2.65E-06	1.82E-06	2.25E-06
C09G9.1.1	981	792	1176	1087	6.41E-05	4.89E-05	5.00E-05	5.71E-05
C09G9.1.2	860	719	999	1003	6.19E-05	4.89E-05	4.68E-05	5.80E-05
C09G9.2	295	283	394	441	2.77E-05	2.51E-05	2.41E-05	3.33E-05
C09G9.3	2	6	1	2	2.80E-06	2.65E-06	1.82E-06	2.25E-06
C09G9.4	30	33	15	17	2.80E-06	2.65E-06	1.82E-06	2.25E-06
C09G9.5	10	14	6	3	2.80E-06	2.65E-06	1.82E-06	2.25E-06
C09G9.6.1	1921	1700	3037	3371	1.19E-04	9.96E-05	1.23E-04	1.68E-04
C09G9.6.2	1388	1161	2106	2467	1.17E-04	9.21E-05	1.15E-04	1.66E-04
C09G9.7	14	23	8	22	4.03E-06	6.24E-06	1.82E-06	5.08E-06
C09G9.8	1	1	1	0	2.80E-06	2.65E-06	1.82E-06	2.25E-06
C09H10.1	11	15	11	0	2.88E-06	3.70E-06	1.88E-06	2.25E-06
C09H10.10	60	66	110	90	1.48E-05	1.54E-05	1.77E-05	1.79E-05
C09H10.2.1	10051	7766	30403	6215	2.93E-03	2.14E-03	5.77E-03	1.46E-03
C09H10.2.2	7919	6241	23970	5422	2.22E-03	1.65E-03	4.38E-03	1.22E-03
C09H10.3	1190	1609	1297	2070	7.74E-05	9.88E-05	5.49E-05	1.08E-04
C09H10.5	16	20	119	45	2.80E-06	3.17E-06	1.30E-05	6.07E-06
C09H10.6	604	747	800	874	5.24E-05	6.12E-05	4.52E-05	6.09E-05
C09H10.7	120	130	173	270	1.37E-05	1.41E-05	1.29E-05	2.48E-05
C09H10.8	3	11	5	2	2.80E-06	2.65E-06	1.82E-06	2.25E-06
C09H10.9	11	20	10	9	2.80E-06	2.65E-06	1.82E-06	2.25E-06
C09H5.1	1	4	17	4	2.80E-06	2.65E-06	3.63E-06	2.25E-06
C09H5.2a	180	775	214	216	6.16E-06	2.50E-05	4.76E-06	5.94E-06
C09H5.2b	175	758	207	213	5.99E-06	2.45E-05	4.61E-06	5.85E-06
C09H5.3	5	12	7	4	2.80E-06	2.65E-06	1.82E-06	2.25E-06

C09H5.4	9	10	8	7	2.80E-06	2.65E-06	1.82E-06	2.25E-06
C09H5.5	3	10	8	3	2.80E-06	2.65E-06	1.82E-06	2.25E-06
C09H5.6	6	9	8	4	2.80E-06	2.65E-06	1.82E-06	2.25E-06
C09H5.7	13	21	6	2	2.80E-06	2.65E-06	1.82E-06	2.25E-06
C09H5.8	4	1	9	7	2.80E-06	2.65E-06	1.82E-06	2.25E-06
C09H5.9	6	3	6	2	2.80E-06	2.65E-06	1.82E-06	2.25E-06
C09H6.1a	526	511	601	877	1.43E-05	1.31E-05	1.06E-05	1.92E-05
C09H6.1b	529	522	608	880	1.41E-05	1.31E-05	1.06E-05	1.88E-05
C09H6.2a	227	281	181	324	8.62E-06	1.01E-05	4.46E-06	9.87E-06
C09H6.2b	253	320	206	345	8.71E-06	1.04E-05	4.61E-06	9.54E-06
C09H6.3	512	457	647	930	2.85E-05	2.40E-05	2.34E-05	4.15E-05
C10A4.1	7	12	18	7	2.80E-06	2.65E-06	1.82E-06	2.25E-06
C10A4.2	1	2	3	3	2.80E-06	2.65E-06	1.82E-06	2.25E-06
C10A4.3	3	4	6	4	2.80E-06	2.65E-06	1.82E-06	2.25E-06
C10A4.4	16	17	25	31	3.11E-06	3.09E-06	3.15E-06	4.81E-06
C10A4.5	3	23	13	14	2.80E-06	2.65E-06	1.82E-06	2.25E-06
C10A4.6	3	8	10	3	2.80E-06	2.65E-06	1.82E-06	2.25E-06
C10A4.7	3	16	2	3	2.80E-06	2.94E-06	1.82E-06	2.25E-06
C10A4.8	15	21	11	19	2.80E-06	2.96E-06	1.82E-06	2.27E-06
C10B5.1	42	63	41	37	5.01E-06	7.12E-06	3.19E-06	3.55E-06
C10B5.3	45	97	64	78	1.13E-05	2.30E-05	1.04E-05	1.57E-05
C10C5.1	201	231	323	424	8.85E-06	9.63E-06	9.26E-06	1.50E-05
C10C5.2	24	27	17	28	2.80E-06	2.70E-06	1.82E-06	2.38E-06
C10C5.3	87	126	48	77	7.84E-06	1.07E-05	2.82E-06	5.58E-06
C10C5.4	89	144	91	117	8.12E-06	1.24E-05	5.39E-06	8.57E-06
C10C5.5	37	64	36	58	3.47E-06	5.66E-06	2.20E-06	4.36E-06
C10C5.6a	1240	1227	1623	2286	2.57E-05	2.40E-05	2.19E-05	3.81E-05
C10C5.6b	1305	1289	1706	2426	2.55E-05	2.38E-05	2.17E-05	3.80E-05
C10C5.7	1	9	11	1	2.80E-06	3.78E-06	3.19E-06	2.25E-06
C10C6.1	1875	1668	2650	3740	4.12E-05	3.46E-05	3.78E-05	6.59E-05
C10C6.2	4	16	4	5	2.80E-06	2.65E-06	1.82E-06	2.25E-06
C10C6.3	6	24	12	9	2.80E-06	2.65E-06	1.82E-06	2.25E-06
C10C6.5.1	331	362	423	622	1.69E-05	1.75E-05	1.41E-05	2.55E-05
C10C6.6	990	1197	1264	1738	2.90E-05	3.31E-05	2.41E-05	4.09E-05
C10E2.1	3	6	4	3	2.80E-06	2.65E-06	1.82E-06	2.25E-06
C10E2.2	19	20	19	17	2.80E-06	2.65E-06	1.82E-06	2.25E-06
C10E2.3	173	222	183	295	5.71E-06	6.93E-06	3.94E-06	7.83E-06
C10E2.4	0	4	2	2	2.80E-06	2.65E-06	1.82E-06	2.25E-06
C10E2.5	5	4	7	4	2.80E-06	2.65E-06	1.82E-06	2.25E-06
C10E2.6.1	599	726	789	1134	2.61E-05	2.99E-05	2.24E-05	3.97E-05
C10E2.6.2	463	528	638	964	2.90E-05	3.12E-05	2.60E-05	4.85E-05
C10F3.1	532	551	970	1109	2.44E-05	2.39E-05	2.89E-05	4.08E-05
C10F3.2	20	26	17	10	2.80E-06	2.65E-06	1.82E-06	2.25E-06
C10F3.3	33	29	61	28	2.80E-06	2.65E-06	1.82E-06	2.25E-06
C10F3.4a	24	42	34	31	2.80E-06	3.17E-06	1.82E-06	2.25E-06
C10F3.4b	28	50	38	37	2.80E-06	3.39E-06	1.82E-06	2.25E-06
C10F3.6	99	101	66	115	5.60E-06	5.40E-06	2.42E-06	5.22E-06
C10F3.7	11	9	6	6	3.44E-06	2.67E-06	1.82E-06	2.25E-06
C10G11.1	3	7	6	4	2.80E-06	2.65E-06	1.82E-06	2.25E-06
C10G11.10	6	7	8	2	2.80E-06	2.65E-06	1.82E-06	2.25E-06
C10G11.2	5	4	6	2	2.80E-06	2.65E-06	1.82E-06	2.25E-06
C10G11.3	6	10	6	3	2.80E-06	2.65E-06	1.82E-06	2.25E-06
C10G11.4	6	6	5	3	2.80E-06	2.65E-06	1.82E-06	2.25E-06
C10G11.5a	377	540	396	593	2.69E-05	3.64E-05	1.84E-05	3.41E-05
C10G11.5b	368	531	388	584	2.76E-05	3.76E-05	1.89E-05	3.51E-05
C10G11.5c.1	303	464	334	518	2.74E-05	3.96E-05	1.96E-05	3.76E-05
C10G11.5c.2	327	489	374	543	2.69E-05	3.80E-05	2.00E-05	3.59E-05
C10G11.6	119	71	146	242	1.04E-05	5.87E-06	8.33E-06	1.70E-05
C10G11.7.1	1282	1639	2107	2487	8.79E-05	1.06E-04	9.40E-05	1.37E-04

C10G11.7.2	1252	1604	2073	2438	8.59E-05	1.04E-04	9.25E-05	1.34E-04
C10G11.7.3	1257	1604	2078	2442	8.63E-05	1.04E-04	9.29E-05	1.35E-04
C10G11.8	79	313	51	71	6.33E-06	2.37E-05	2.66E-06	4.57E-06
C10G11.9	210	293	176	68	3.67E-05	4.84E-05	2.00E-05	9.54E-06
C10G6.1b.1	205	289	154	220	1.06E-05	1.41E-05	5.19E-06	9.15E-06
C10G6.1b.2	180	250	133	200	1.11E-05	1.46E-05	5.36E-06	9.96E-06
C10G8.1	8	4	7	8	2.80E-06	2.65E-06	1.82E-06	2.25E-06
C10G8.2	2	2	12	6	2.80E-06	2.65E-06	1.82E-06	2.25E-06
C10G8.3	4	4	3	2	2.80E-06	2.65E-06	1.82E-06	2.25E-06
C10G8.4	499	839	680	695	1.88E-04	2.99E-04	1.67E-04	2.11E-04
C10G8.5a	625	837	553	788	2.12E-05	2.68E-05	1.22E-05	2.14E-05
C10G8.5b	694	899	647	884	2.18E-05	2.67E-05	1.32E-05	2.24E-05
C10G8.5c.1	222	290	210	261	1.97E-05	2.43E-05	1.21E-05	1.86E-05
C10G8.5c.2	226	296	210	270	1.92E-05	2.38E-05	1.16E-05	1.84E-05
C10G8.5d	537	662	486	636	2.05E-05	2.39E-05	1.21E-05	1.95E-05
C10G8.6	15	18	14	19	2.80E-06	2.65E-06	1.82E-06	2.25E-06
C10G8.7	5	6	6	4	2.80E-06	2.65E-06	1.82E-06	2.25E-06
C10G8.8a	246	341	159	230	1.97E-05	2.59E-05	8.31E-06	1.48E-05
C10G8.8b.1	206	279	122	192	2.03E-05	2.60E-05	7.84E-06	1.52E-05
C10G8.8b.2	193	259	117	184	2.16E-05	2.74E-05	8.51E-06	1.65E-05
C10H11.1	326	409	467	642	1.21E-05	1.43E-05	1.12E-05	1.91E-05
C10H11.10	791	638	1294	1243	6.30E-05	4.80E-05	6.71E-05	7.95E-05
C10H11.3	77	153	46	56	5.26E-06	9.87E-06	2.04E-06	3.06E-06
C10H11.4	68	135	54	70	4.48E-06	8.41E-06	2.31E-06	3.71E-06
C10H11.5	14	11	16	8	2.80E-06	2.65E-06	1.82E-06	2.25E-06
C10H11.6	174	216	120	190	1.06E-05	1.25E-05	4.77E-06	9.31E-06
C10H11.7	12	29	30	7	2.80E-06	2.99E-06	2.11E-06	2.25E-06
C10H11.8	83	86	192	250	8.09E-06	7.91E-06	1.22E-05	1.96E-05
C10H11.9	1155	1192	1576	2330	2.97E-05	2.90E-05	2.64E-05	4.81E-05
C11D2.1	4	3	14	4	2.80E-06	2.65E-06	4.66E-06	2.25E-06
C11D2.2	8	9	10	7	2.80E-06	2.65E-06	1.82E-06	2.25E-06
C11D2.3	9	9	6	6	2.80E-06	2.65E-06	1.82E-06	2.25E-06
C11D2.4	198	287	266	253	1.90E-05	2.61E-05	1.67E-05	1.95E-05
C11D2.6a	41	54	62	70	2.80E-06	2.65E-06	1.82E-06	2.25E-06
C11D2.6b	28	35	34	48	2.80E-06	2.65E-06	1.82E-06	2.25E-06
C11D2.6c	54	77	75	84	2.80E-06	2.65E-06	1.82E-06	2.25E-06
C11D2.6d	53	72	76	81	2.80E-06	2.65E-06	1.82E-06	2.25E-06
C11D2.7.1	266	321	461	223	4.09E-05	4.67E-05	4.62E-05	2.76E-05
C11D2.7.2	228	276	318	172	3.77E-05	4.31E-05	3.42E-05	2.29E-05
C11D9.1	29	48	20	37	2.80E-06	2.70E-06	1.82E-06	2.25E-06
C11E4.1	241	384	225	307	2.60E-05	3.92E-05	1.58E-05	2.66E-05
C11E4.2	52	102	49	44	8.62E-06	1.60E-05	5.28E-06	5.87E-06
C11E4.3	13	19	15	14	2.80E-06	2.65E-06	1.82E-06	2.25E-06
C11E4.4	8	3	2	3	2.80E-06	2.65E-06	1.82E-06	2.25E-06
C11E4.6	75	117	73	123	2.80E-06	3.12E-06	1.82E-06	2.79E-06
C11E4.7	62	33	43	19	6.75E-06	3.39E-06	3.04E-06	2.25E-06
C11E4.8	5	3	4	3	2.80E-06	2.65E-06	1.82E-06	2.25E-06
C11E4.t1	1	0	3	0	2.80E-06	2.65E-06	3.04E-06	2.25E-06
C11G10.1	13	25	14	9	2.80E-06	4.95E-06	1.91E-06	2.25E-06
C11G10.2	2	10	7	1	2.80E-06	3.09E-06	1.82E-06	2.25E-06
C11G10.t1	0	0	0	1	2.80E-06	2.65E-06	1.82E-06	2.25E-06
C11G6.1	58	69	56	115	3.36E-06	3.78E-06	2.11E-06	5.35E-06
C11G6.2	4	4	2	1	2.80E-06	2.65E-06	1.82E-06	2.25E-06
C11G6.3	67	98	75	142	4.70E-06	6.48E-06	3.43E-06	7.98E-06
C11G6.4b.1	51	103	55	71	3.14E-06	5.95E-06	2.19E-06	3.49E-06
C11G6.4b.2	38	80	30	58	2.80E-06	5.34E-06	1.82E-06	3.28E-06
C11G6.4b.3	45	91	35	64	4.37E-06	8.36E-06	2.22E-06	4.99E-06
C11H1.2	34	59	27	29	2.80E-06	3.86E-06	1.82E-06	2.25E-06
C11H1.3	96	132	84	113	6.69E-06	8.70E-06	3.81E-06	6.34E-06

C11H1.5	72	107	25	19	4.51E-06	6.35E-06	1.82E-06	2.25E-06
C11H1.7	9	11	6	7	2.80E-06	2.65E-06	1.82E-06	2.25E-06
C11H1.8	30	35	19	46	5.82E-06	6.43E-06	2.41E-06	7.17E-06
C11H1.9a	122	138	121	94	8.88E-06	9.47E-06	5.72E-06	5.49E-06
C11H1.9b	119	131	115	90	9.07E-06	9.44E-06	5.70E-06	5.51E-06
C12C8.1	193	789	58	83	1.08E-05	4.15E-05	2.10E-06	3.71E-06
C12C8.3a	2799	2167	3032	4933	6.53E-05	4.77E-05	4.60E-05	9.24E-05
C12C8.3b	2775	2157	3020	4900	6.49E-05	4.77E-05	4.60E-05	9.21E-05
C12D12.1a	1073	1149	1314	1460	4.74E-05	4.80E-05	3.78E-05	5.18E-05
C12D12.1b	960	1079	1183	1337	5.26E-05	5.58E-05	4.21E-05	5.88E-05
C12D12.2b.2	641	1139	862	1378	3.86E-05	6.49E-05	3.38E-05	6.67E-05
C12D12.3	2	5	1	2	2.80E-06	2.65E-06	1.82E-06	2.25E-06
C12D12.4	11	11	3	5	3.33E-06	3.15E-06	1.82E-06	2.25E-06
C12D12.5	20	21	16	31	3.05E-06	3.04E-06	1.82E-06	3.80E-06
C12D12.7	5	14	20	7	2.80E-06	2.65E-06	1.82E-06	2.25E-06
C12D5.1	11	7	7	1	2.80E-06	2.65E-06	1.82E-06	2.25E-06
C12D5.10	16	10	24	9	2.80E-06	2.65E-06	1.82E-06	2.25E-06
C12D5.11	5	9	5	2	2.80E-06	2.65E-06	1.82E-06	2.25E-06
C12D5.2	11	19	22	16	2.80E-06	2.65E-06	1.82E-06	2.25E-06
C12D5.3	9	2	11	6	2.80E-06	2.65E-06	1.82E-06	2.25E-06
C12D5.4	5	5	1	0	2.80E-06	2.65E-06	1.82E-06	2.25E-06
C12D5.5	7	7	10	1	2.80E-06	2.65E-06	1.82E-06	2.25E-06
C12D5.7	50	142	64	66	3.64E-06	9.73E-06	3.02E-06	3.85E-06
C12D5.8a	9	30	18	12	2.80E-06	2.65E-06	1.82E-06	2.25E-06
C12D5.8b	1	13	3	1	2.80E-06	3.41E-06	1.82E-06	2.25E-06
C12D5.9	17	20	12	9	2.80E-06	2.65E-06	1.82E-06	2.25E-06
C12D8.10b.1	859	1024	1000	1364	4.51E-05	5.07E-05	3.41E-05	5.74E-05
C12D8.10b.2	833	989	951	1325	5.52E-05	6.19E-05	4.10E-05	7.05E-05
C12D8.10c	421	478	475	644	4.90E-05	5.26E-05	3.60E-05	6.02E-05
C12D8.11.1	608	816	754	1126	3.37E-05	4.27E-05	2.71E-05	5.00E-05
C12D8.11.2	591	801	719	1095	3.28E-05	4.20E-05	2.60E-05	4.88E-05
C12D8.12	7	7	12	10	2.80E-06	2.65E-06	1.82E-06	2.25E-06
C12D8.13	4	5	2	5	2.80E-06	2.65E-06	1.82E-06	2.25E-06
C12D8.14	83	91	158	38	4.19E-05	4.34E-05	5.19E-05	1.54E-05
C12D8.15	5	6	4	4	2.80E-06	2.65E-06	1.82E-06	2.25E-06
C12D8.16	83	89	96	24	4.19E-05	4.24E-05	3.15E-05	9.72E-06
C12D8.17	49	57	54	21	2.47E-05	2.72E-05	1.77E-05	8.50E-06
C12D8.18	46	67	46	21	1.53E-05	2.11E-05	9.99E-06	5.62E-06
C12D8.19	45	35	61	18	2.27E-05	1.67E-05	2.00E-05	7.29E-06
C12D8.1a	679	771	856	1127	4.07E-05	4.36E-05	3.34E-05	5.42E-05
C12D8.1b	702	813	860	1128	3.07E-05	3.36E-05	2.45E-05	3.97E-05
C12D8.1c.1	656	756	835	1101	4.05E-05	4.41E-05	3.35E-05	5.46E-05
C12D8.1c.2	680	780	858	1127	3.36E-05	3.64E-05	2.76E-05	4.47E-05
C12D8.1c.3	602	707	778	1059	3.97E-05	4.41E-05	3.34E-05	5.61E-05
C12D8.2	42	52	11	9	1.14E-05	1.33E-05	1.93E-06	2.25E-06
C12D8.3	3	9	1	0	2.80E-06	5.21E-06	1.82E-06	2.25E-06
C12D8.4	2	1	1	2	2.80E-06	2.65E-06	1.82E-06	2.25E-06
C12D8.5	80	150	79	133	6.64E-06	1.17E-05	4.26E-06	8.86E-06
C12D8.6	65	65	53	24	1.36E-05	1.28E-05	7.20E-06	4.03E-06
C12D8.7	1	0	1	1	2.80E-06	2.65E-06	1.82E-06	2.25E-06
C12D8.8	14	17	13	6	2.80E-06	2.65E-06	1.82E-06	2.25E-06
C12D8.9	7	7	23	2	2.80E-06	2.65E-06	6.01E-06	2.25E-06
C13A10.1	25	30	38	41	2.80E-06	2.65E-06	1.82E-06	2.34E-06
C13A10.2	5	17	10	10	2.80E-06	3.52E-06	1.82E-06	2.25E-06
C13A2.1	5	4	5	6	2.80E-06	2.65E-06	1.82E-06	2.25E-06
C13A2.10	3	5	6	4	2.80E-06	2.65E-06	1.82E-06	2.25E-06
C13A2.11	2	4	1	3	2.80E-06	2.65E-06	1.82E-06	2.25E-06
C13A2.12	5	3	2	3	2.80E-06	2.65E-06	1.82E-06	2.25E-06
C13A2.2	3	1	1	1	2.80E-06	2.65E-06	1.82E-06	2.25E-06

C13A2.3	3	4	3	2	2.80E-06	2.65E-06	1.82E-06	2.25E-06
C13A2.4	0	2	8	2	2.80E-06	2.65E-06	1.82E-06	2.25E-06
C13A2.5	6	6	10	7	2.80E-06	2.65E-06	1.82E-06	2.25E-06
C13A2.6	4	4	4	4	2.80E-06	2.65E-06	1.82E-06	2.25E-06
C13A2.7	2	2	0	1	2.80E-06	2.65E-06	1.82E-06	2.25E-06
C13A2.8	3	1	0	0	2.80E-06	2.65E-06	1.82E-06	2.25E-06
C13A2.9	5	3	17	4	2.80E-06	2.65E-06	1.82E-06	2.25E-06
C13B4.2.1	1146	1185	1715	1893	7.59E-05	7.41E-05	7.39E-05	1.01E-04
C13B4.2.2	919	972	1377	1618	6.10E-05	6.09E-05	5.95E-05	8.62E-05
C13B7.1	4	1	2	6	2.80E-06	2.65E-06	1.82E-06	2.25E-06
C13B7.2	10	3	7	7	2.80E-06	2.65E-06	1.82E-06	2.25E-06
C13B7.3	5	6	9	0	2.80E-06	2.65E-06	1.82E-06	2.25E-06
C13B7.4	1	5	8	6	2.80E-06	2.65E-06	1.82E-06	2.25E-06
C13B7.5	4	5	4	2	2.80E-06	2.65E-06	1.82E-06	2.25E-06
C13B7.6	107	186	253	573	2.06E-05	3.38E-05	3.17E-05	8.86E-05
C13B9.1.1	41	114	52	92	2.80E-06	5.24E-06	1.82E-06	3.60E-06
C13B9.1.2	35	100	36	81	2.80E-06	5.61E-06	1.82E-06	3.87E-06
C13B9.2	24	33	71	87	2.80E-06	2.94E-06	4.34E-06	6.54E-06
C13B9.3	1012	1038	1103	1502	6.19E-05	5.99E-05	4.39E-05	7.37E-05
C13B9.4a.1	69	116	62	79	2.80E-06	4.15E-06	1.82E-06	2.41E-06
C13B9.4a.2	46	92	39	56	3.00E-06	5.66E-06	1.82E-06	2.92E-06
C13B9.4b.1	70	117	61	80	2.80E-06	4.23E-06	1.82E-06	2.45E-06
C13B9.4b.2	43	86	34	55	2.94E-06	5.58E-06	1.82E-06	3.04E-06
C13B9.4c.1	69	116	63	79	2.80E-06	4.10E-06	1.82E-06	2.38E-06
C13B9.4c.2	45	92	38	56	2.97E-06	5.71E-06	1.82E-06	2.95E-06
C13C12.1	21	35	27	9	3.58E-06	5.63E-06	2.99E-06	2.25E-06
C13C12.2	62	110	48	32	5.77E-06	9.68E-06	2.92E-06	2.38E-06
C13C4.1	70	92	93	84	6.27E-06	7.80E-06	5.43E-06	6.05E-06
C13C4.2	45	61	52	63	4.06E-06	5.21E-06	3.06E-06	4.57E-06
C13C4.3	11	29	8	14	2.80E-06	2.65E-06	1.82E-06	2.25E-06
C13C4.4	21	24	16	23	2.80E-06	2.67E-06	1.82E-06	2.25E-06
C13C4.6	48	91	71	51	3.84E-06	6.85E-06	3.68E-06	3.26E-06
C13C4.7	9	11	13	3	2.80E-06	2.65E-06	1.82E-06	2.25E-06
C13D9.1	2	20	6	9	2.80E-06	2.65E-06	1.82E-06	2.25E-06
C13D9.2	3	10	6	4	2.80E-06	2.65E-06	1.82E-06	2.25E-06
C13D9.3	8	8	8	4	2.80E-06	2.65E-06	1.82E-06	2.25E-06
C13D9.4	5	7	3	5	2.80E-06	2.65E-06	1.82E-06	2.25E-06
C13D9.5	9	7	9	3	2.80E-06	2.65E-06	1.82E-06	2.25E-06
C13D9.6	7	5	6	5	2.80E-06	2.65E-06	1.82E-06	2.25E-06
C13D9.8	14	23	26	10	2.80E-06	2.65E-06	1.82E-06	2.25E-06
C13D9.9	63	79	74	114	4.42E-06	5.24E-06	3.39E-06	6.43E-06
C13E3.1	22	34	38	39	2.80E-06	2.65E-06	1.82E-06	2.25E-06
C13F10.1a	18	18	21	14	3.58E-06	3.39E-06	2.71E-06	2.25E-06
C13F10.1b	11	19	8	14	2.80E-06	2.65E-06	1.82E-06	2.25E-06
C13F10.2	54	73	78	72	1.43E-05	1.83E-05	1.34E-05	1.53E-05
C13F10.4	782	936	1276	1784	1.34E-05	1.51E-05	1.42E-05	2.44E-05
C13F10.5	112	116	106	127	2.18E-05	2.13E-05	1.34E-05	1.99E-05
C13F10.6	302	327	409	638	2.39E-05	2.45E-05	2.11E-05	4.06E-05
C13G3.1	46	75	41	66	1.25E-05	1.93E-05	7.27E-06	1.44E-05
C13G3.2	14	28	9	14	2.80E-06	4.68E-06	1.82E-06	2.25E-06
C13G3.3a.1	756	807	1184	1458	4.21E-05	4.25E-05	4.29E-05	6.52E-05
C13G3.3a.2	681	699	987	1283	4.13E-05	4.01E-05	3.90E-05	6.26E-05
C13G3.3b.1	673	691	988	1284	4.44E-05	4.30E-05	4.24E-05	6.80E-05
C13G3.3b.2	663	686	970	1271	4.39E-05	4.29E-05	4.18E-05	6.76E-05
C13G5.1	1	9	4	5	2.80E-06	2.65E-06	1.82E-06	2.25E-06
C13G5.2	220	208	303	317	2.28E-05	2.04E-05	2.04E-05	2.64E-05
C14A11.1	6	11	3	12	2.80E-06	2.65E-06	1.82E-06	2.25E-06
C14A11.2	7	12	6	10	2.80E-06	2.65E-06	1.82E-06	2.25E-06
C14A11.3a	107	116	145	199	8.18E-06	8.38E-06	7.22E-06	1.22E-05

C14A11.3b	184	190	213	283	1.07E-05	1.04E-05	8.07E-06	1.32E-05
C14A11.5	11	13	13	5	2.80E-06	2.65E-06	1.82E-06	2.25E-06
C14A11.6	70	96	100	93	3.89E-06	5.05E-06	3.63E-06	4.16E-06
C14A11.7	3	5	9	1	2.80E-06	2.65E-06	1.82E-06	2.25E-06
C14A4.1.1	217	287	271	258	2.44E-05	3.04E-05	1.98E-05	2.33E-05
C14A4.1.2	200	263	223	231	2.44E-05	3.02E-05	1.77E-05	2.26E-05
C14A4.10	303	267	189	301	7.63E-05	6.35E-05	3.10E-05	6.09E-05
C14A4.11	372	394	407	444	3.93E-05	3.93E-05	2.80E-05	3.76E-05
C14A4.12a	48	84	40	65	2.80E-06	3.84E-06	1.82E-06	2.52E-06
C14A4.12b	44	76	35	62	2.80E-06	3.84E-06	1.82E-06	2.65E-06
C14A4.13	15	33	17	11	2.80E-06	2.65E-06	1.82E-06	2.25E-06
C14A4.14	368	525	366	478	2.92E-05	3.94E-05	1.89E-05	3.05E-05
C14A4.15	5	5	7	2	2.80E-06	2.65E-06	1.82E-06	2.25E-06
C14A4.2	301	403	317	408	2.72E-05	3.44E-05	1.86E-05	2.96E-05
C14A4.3	367	413	504	745	2.05E-05	2.18E-05	1.83E-05	3.34E-05
C14A4.4a	760	652	867	1327	3.18E-05	2.57E-05	2.36E-05	4.46E-05
C14A4.4b	520	425	579	840	3.11E-05	2.40E-05	2.25E-05	4.03E-05
C14A4.5	90	125	200	113	1.48E-05	1.94E-05	2.14E-05	1.49E-05
C14A4.6.1	84	104	58	58	1.52E-05	1.78E-05	6.83E-06	8.43E-06
C14A4.6.2	66	82	48	54	1.27E-05	1.49E-05	5.99E-06	8.32E-06
C14A4.7a	36	35	26	26	6.24E-06	5.74E-06	2.93E-06	3.62E-06
C14A4.7b	20	17	12	14	5.18E-06	4.15E-06	2.02E-06	2.92E-06
C14A4.8	8	15	5	3	2.80E-06	2.65E-06	1.82E-06	2.25E-06
C14A4.9.1	85	140	59	27	1.68E-05	2.62E-05	7.60E-06	4.30E-06
C14A4.9.2	71	103	41	22	1.39E-05	1.90E-05	5.23E-06	3.46E-06
C14A6.1	191	437	305	539	2.21E-05	4.79E-05	2.30E-05	5.02E-05
C14A6.2	8	3	2	9	2.80E-06	2.65E-06	1.82E-06	2.25E-06
C14A6.3	7	10	13	5	2.80E-06	2.65E-06	1.82E-06	2.25E-06
C14A6.5	20	26	19	14	2.80E-06	2.65E-06	1.82E-06	2.25E-06
C14A6.6	16	27	3	8	2.80E-06	4.34E-06	1.82E-06	2.25E-06
C14A6.7	6	5	6	4	2.80E-06	2.65E-06	1.82E-06	2.25E-06
C14A6.8	5	10	4	4	2.80E-06	2.72E-06	1.82E-06	2.25E-06
C14B1.1.1	2701	3095	3086	4058	1.90E-04	2.06E-04	1.41E-04	2.30E-04
C14B1.1.2	2494	2826	2860	3859	1.91E-04	2.04E-04	1.42E-04	2.37E-04
C14B1.10.1	136	164	277	355	7.73E-06	8.81E-06	1.03E-05	1.62E-05
C14B1.10.2	136	161	263	350	7.98E-06	8.94E-06	1.00E-05	1.65E-05
C14B1.2	350	371	597	474	6.16E-05	6.17E-05	6.84E-05	6.70E-05
C14B1.3	80	126	71	125	7.25E-06	1.08E-05	4.19E-06	9.11E-06
C14B1.4	364	380	635	626	3.00E-05	2.96E-05	3.41E-05	4.14E-05
C14B1.6	225	351	260	423	1.00E-05	1.47E-05	7.53E-06	1.51E-05
C14B1.7	593	513	698	905	2.21E-05	1.80E-05	1.69E-05	2.70E-05
C14B1.8	203	215	135	155	3.27E-05	3.27E-05	1.41E-05	2.00E-05
C14B1.9	726	747	1097	1155	4.85E-05	4.71E-05	4.77E-05	6.20E-05
C14B4.2	106	115	305	187	2.80E-06	2.65E-06	4.12E-06	3.13E-06
C14B9.10.1	768	969	1761	402	2.46E-04	2.93E-04	3.67E-04	1.03E-04
C14B9.10.2	613	787	1232	339	1.94E-04	2.35E-04	2.54E-04	8.62E-05
C14B9.2.1	550	672	582	595	2.91E-05	3.36E-05	2.01E-05	2.53E-05
C14B9.2.2	540	661	573	591	3.24E-05	3.75E-05	2.24E-05	2.85E-05
C14B9.3	39	93	18	42	4.09E-06	9.23E-06	1.82E-06	3.55E-06
C14B9.4a.1	1478	1640	2772	3242	7.22E-05	7.57E-05	8.82E-05	1.27E-04
C14B9.4a.2	1427	1603	2671	3184	6.99E-05	7.41E-05	8.51E-05	1.25E-04
C14B9.4b	1400	1566	2600	3105	6.82E-05	7.21E-05	8.24E-05	1.21E-04
C14B9.6a	158	171	163	271	5.21E-06	5.32E-06	3.50E-06	7.17E-06
C14B9.6b	267	324	305	472	5.60E-06	6.43E-06	4.15E-06	7.94E-06
C14B9.6c	296	344	305	480	5.99E-06	6.59E-06	4.03E-06	7.80E-06
C14B9.7.1	9976	8547	15007	10627	2.07E-03	1.67E-03	2.02E-03	1.77E-03
C14B9.7.2	8917	7673	13261	10075	1.88E-03	1.53E-03	1.82E-03	1.71E-03
C14B9.8	527	585	612	1013	1.49E-05	1.56E-05	1.12E-05	2.30E-05
C14B9.t1	1	0	0	0	2.80E-06	2.65E-06	1.82E-06	2.25E-06

C14B9.t2	0	1	0	0	2.80E-06	2.65E-06	1.82E-06	2.25E-06
C14C10.1	39	94	22	26	4.34E-06	9.89E-06	1.82E-06	2.32E-06
C14C10.2a	50	65	54	85	5.38E-06	6.61E-06	3.77E-06	7.33E-06
C14C10.2b	48	58	52	82	5.52E-06	6.30E-06	3.90E-06	7.58E-06
C14C10.3a.1	658	746	767	1185	3.26E-05	3.50E-05	2.48E-05	4.72E-05
C14C10.3a.2	614	681	717	1118	3.26E-05	3.42E-05	2.48E-05	4.77E-05
C14C10.3b	625	697	711	1139	3.20E-05	3.37E-05	2.37E-05	4.68E-05
C14C10.4.1	970	908	1175	1806	3.22E-05	2.85E-05	2.54E-05	4.82E-05
C14C10.5	3146	2541	4106	5735	5.76E-05	4.39E-05	4.89E-05	8.43E-05
C14C10.6	58	65	49	47	1.33E-05	1.41E-05	7.31E-06	8.64E-06
C14C10.7	7	6	15	3	2.80E-06	2.65E-06	2.53E-06	2.25E-06
C14C11.1	37	116	22	25	3.98E-06	1.18E-05	1.82E-06	2.25E-06
C14C11.3.1	140	278	113	157	8.09E-06	1.52E-05	4.25E-06	7.29E-06
C14C11.3.2	130	265	99	149	8.01E-06	1.54E-05	3.97E-06	7.35E-06
C14C11.4	61	35	31	49	3.98E-06	2.65E-06	1.82E-06	2.56E-06
C14C11.5	4	5	2	4	2.80E-06	2.65E-06	1.82E-06	2.25E-06
C14C11.6	170	253	265	336	1.13E-05	1.59E-05	1.15E-05	1.79E-05
C14C11.7	67	60	276	69	3.01E-05	2.55E-05	8.08E-05	2.49E-05
C14C11.8	129	165	49	65	6.78E-06	8.20E-06	1.82E-06	2.74E-06
C14C6.1	2	3	6	0	2.80E-06	2.65E-06	1.82E-06	2.25E-06
C14C6.10	4	2	6	2	2.80E-06	2.65E-06	1.82E-06	2.25E-06
C14C6.11	8	5	5	2	2.80E-06	2.65E-06	1.82E-06	2.25E-06
C14C6.12	2	0	0	3	2.80E-06	2.65E-06	1.82E-06	2.25E-06
C14C6.13	4	3	4	4	2.80E-06	2.65E-06	1.82E-06	2.25E-06
C14C6.2	127	99	98	48	2.28E-05	1.68E-05	1.14E-05	6.90E-06
C14C6.3	3	2	22	2	2.80E-06	2.65E-06	1.82E-06	2.25E-06
C14C6.4	3	5	9	7	2.80E-06	2.65E-06	1.82E-06	2.25E-06
C14C6.5	397	571	353	497	8.14E-05	1.11E-04	4.71E-05	8.19E-05
C14C6.6	13	7	6	7	2.80E-06	2.65E-06	1.82E-06	2.25E-06
C14C6.7	8	12	14	8	2.80E-06	2.65E-06	1.82E-06	2.25E-06
C14C6.8	4	1	5	0	2.80E-06	2.65E-06	1.82E-06	2.25E-06
C14C6.9	8	5	7	5	2.80E-06	2.65E-06	1.82E-06	2.25E-06
C14E2.1	6	2	4	0	2.80E-06	2.65E-06	1.82E-06	2.25E-06
C14E2.2	2	5	1	1	2.80E-06	2.65E-06	1.82E-06	2.25E-06
C14E2.3	2	1	1	0	2.80E-06	2.65E-06	1.82E-06	2.25E-06
C14E2.4	4	5	7	3	2.80E-06	2.65E-06	1.82E-06	2.25E-06
C14E2.5	5	7	10	5	2.80E-06	2.65E-06	1.82E-06	2.25E-06
C14E2.6	1	1	1	2	2.80E-06	2.65E-06	1.82E-06	2.25E-06
C14E2.7	3	3	5	3	2.80E-06	2.65E-06	1.82E-06	2.25E-06
C14F11.1a	1158	1398	1873	1765	8.96E-05	1.02E-04	9.44E-05	1.10E-04
C14F11.1b.1	1062	1293	1737	1529	8.73E-05	1.00E-04	9.29E-05	1.01E-04
C14F11.1b.2	738	897	1258	1186	9.03E-05	1.04E-04	1.00E-04	1.17E-04
C14F11.2	64	71	62	76	3.56E-06	3.73E-06	2.24E-06	3.40E-06
C14F11.4a	247	316	291	434	2.67E-05	3.22E-05	2.04E-05	3.76E-05
C14F11.4b	209	299	237	391	2.28E-05	3.08E-05	1.68E-05	3.43E-05
C14F11.5	365	578	432	856	3.14E-05	4.70E-05	2.42E-05	5.92E-05
C14F11.6	77	121	93	27	1.22E-05	1.81E-05	9.60E-06	3.44E-06
C14F11.7	1	1	2	2	2.80E-06	2.65E-06	1.82E-06	2.25E-06
C14F5.1a	35	66	38	68	5.74E-06	1.02E-05	4.06E-06	8.97E-06
C14F5.1b	31	49	29	60	5.29E-06	7.91E-06	3.23E-06	8.23E-06
C14F5.2	13	27	12	27	2.80E-06	3.78E-06	1.82E-06	3.22E-06
C14F5.3a	110	177	64	106	9.30E-06	1.41E-05	3.52E-06	7.20E-06
C14F5.3b	280	396	144	321	8.54E-06	1.14E-05	2.86E-06	7.85E-06
C14F5.3c.1	113	198	75	116	9.21E-06	1.52E-05	3.97E-06	7.60E-06
C14F5.3c.2	116	203	80	117	1.49E-05	2.47E-05	6.69E-06	1.21E-05
C14F5.4	57	88	35	47	5.68E-06	8.31E-06	2.28E-06	3.78E-06
C14F5.5	236	312	286	431	1.81E-05	2.26E-05	1.43E-05	2.66E-05
C14H10.1	348	522	363	378	2.96E-05	4.19E-05	2.01E-05	2.58E-05
C14H10.3	244	373	202	287	9.07E-06	1.31E-05	4.88E-06	8.57E-06

C14H10.4	21	18	14	8	2.80E-06	2.65E-06	1.82E-06	2.25E-06
C15A11.1	34	42	15	17	4.17E-06	4.87E-06	1.82E-06	2.25E-06
C15A11.2	39	27	10	16	8.37E-06	5.48E-06	1.82E-06	2.77E-06
C15A11.4	16	26	13	28	2.80E-06	2.65E-06	1.82E-06	2.25E-06
C15A11.5	515	463	652	283	6.06E-05	5.15E-05	5.00E-05	2.68E-05
C15A11.6	483	416	544	262	5.69E-05	4.63E-05	4.17E-05	2.48E-05
C15A11.7	56	42	23	20	3.95E-06	2.80E-06	1.82E-06	2.25E-06
C15A7.1	7	10	11	6	2.80E-06	2.65E-06	1.82E-06	2.25E-06
C15A7.2	4	15	9	4	2.80E-06	2.65E-06	1.82E-06	2.25E-06
C15B12.1	34	40	18	35	3.19E-06	3.54E-06	1.82E-06	2.63E-06
C15B12.2	5	2	2	2	2.80E-06	2.65E-06	1.82E-06	2.25E-06
C15B12.3	0	0	1	0	2.80E-06	2.65E-06	1.82E-06	2.25E-06
C15B12.4	20	21	24	12	2.80E-06	2.65E-06	1.82E-06	2.25E-06
C15B12.5a	9	15	7	11	2.80E-06	2.65E-06	1.82E-06	2.25E-06
C15B12.5b	10	14	8	11	2.80E-06	2.65E-06	1.82E-06	2.25E-06
C15B12.5c	9	13	8	11	2.80E-06	2.65E-06	1.82E-06	2.25E-06
C15B12.6	21	12	17	15	2.80E-06	2.65E-06	1.82E-06	2.25E-06
C15B12.7a	133	156	106	152	9.10E-06	1.01E-05	4.72E-06	8.37E-06
C15B12.7b	164	202	134	193	8.71E-06	1.01E-05	4.63E-06	8.23E-06
C15B12.8	1	10	6	10	2.80E-06	2.65E-06	1.82E-06	2.25E-06
C15B12.9	4	4	6	1	2.80E-06	2.65E-06	1.82E-06	2.25E-06
C15C6.1	70	124	41	40	1.02E-05	1.70E-05	3.86E-06	4.66E-06
C15C6.2a	61	136	49	56	8.48E-06	1.79E-05	4.43E-06	6.25E-06
C15C6.2b	85	195	72	68	9.74E-06	2.11E-05	5.38E-06	6.25E-06
C15C6.3	111	77	200	282	7.64E-06	5.00E-06	8.96E-06	1.56E-05
C15C6.4	71	79	99	123	1.29E-05	1.35E-05	1.17E-05	1.79E-05
C15C6.t1	0	0	0	1	2.80E-06	2.65E-06	1.82E-06	2.25E-06
C15C7.2	65	111	74	84	3.56E-06	5.71E-06	2.62E-06	3.69E-06
C15C7.4	13	32	11	14	3.11E-06	7.25E-06	1.82E-06	2.70E-06
C15C7.5	338	541	215	275	5.07E-05	7.66E-05	2.10E-05	3.31E-05
C15C7.6	6	12	15	10	2.80E-06	2.65E-06	1.82E-06	2.25E-06
C15C7.7	60	95	63	79	5.29E-06	7.94E-06	3.63E-06	5.60E-06
C15C8.1	45	32	35	30	3.53E-06	2.65E-06	1.82E-06	2.25E-06
C15C8.2	22	28	12	12	2.80E-06	2.65E-06	1.82E-06	2.25E-06
C15C8.3	546	811	612	486	4.46E-05	6.26E-05	3.26E-05	3.19E-05
C15C8.4.1	280	275	188	312	2.42E-05	2.24E-05	1.06E-05	2.16E-05
C15C8.5	6	5	4	2	2.80E-06	2.65E-06	1.82E-06	2.25E-06
C15C8.6	10	17	5	10	2.80E-06	2.65E-06	1.82E-06	2.25E-06
C15C8.7	206	255	260	272	4.57E-05	5.34E-05	3.75E-05	4.84E-05
C15F1.1	105	165	78	105	1.09E-05	1.61E-05	5.25E-06	8.73E-06
C15F1.2	98	162	53	59	4.34E-06	6.80E-06	1.82E-06	2.25E-06
C15F1.3a.1	769	747	1181	1643	1.85E-05	1.70E-05	1.85E-05	3.17E-05
C15F1.3a.2	605	583	961	1215	1.45E-05	1.33E-05	1.50E-05	2.35E-05
C15F1.3b	378	373	590	764	1.20E-05	1.11E-05	1.21E-05	1.94E-05
C15F1.4	264	274	279	390	2.34E-05	2.30E-05	1.61E-05	2.78E-05
C15F1.5a	25	41	18	23	2.80E-06	3.78E-06	1.82E-06	2.25E-06
C15F1.5b	23	44	19	24	2.80E-06	4.15E-06	1.82E-06	2.25E-06
C15F1.7a	534	796	688	565	8.32E-05	1.17E-04	6.98E-05	7.07E-05
C15F1.7b.1	557	825	750	566	9.60E-05	1.34E-04	8.41E-05	7.83E-05
C15F1.7b.2	473	697	576	483	9.12E-05	1.27E-04	7.23E-05	7.48E-05
C15F1.8	25	29	21	36	5.60E-06	6.14E-06	3.06E-06	6.45E-06
C15F1.9	2	1	4	0	2.80E-06	2.65E-06	1.82E-06	2.25E-06
C15H11.1	15	34	14	7	2.80E-06	5.40E-06	1.82E-06	2.25E-06
C15H11.10	2	3	2	0	2.80E-06	2.65E-06	1.82E-06	2.25E-06
C15H11.11	3	2	3	4	2.80E-06	2.65E-06	1.82E-06	2.25E-06
C15H11.2	74	28	109	47	7.28E-06	2.65E-06	6.96E-06	3.71E-06
C15H11.3	343	323	743	972	2.04E-05	1.81E-05	2.87E-05	4.63E-05
C15H11.4	596	748	937	1083	5.85E-05	6.93E-05	5.98E-05	8.53E-05
C15H11.7.1	1384	1174	1653	1457	1.66E-04	1.33E-04	1.29E-04	1.41E-04

C15H11.8	123	115	341	137	2.93E-05	2.58E-05	5.28E-05	2.62E-05
C15H11.9.1	556	532	449	575	5.55E-05	5.02E-05	2.92E-05	4.61E-05
C15H11.9.2	470	451	381	537	5.23E-05	4.74E-05	2.76E-05	4.80E-05
C15H7.2	11	15	8	4	2.80E-06	2.65E-06	1.82E-06	2.25E-06
C15H7.3	43	82	26	29	4.03E-06	7.25E-06	1.82E-06	2.25E-06
C15H7.4	17	26	8	8	2.80E-06	2.80E-06	1.82E-06	2.25E-06
C15H9.1	36	70	36	60	2.80E-06	2.65E-06	1.82E-06	2.25E-06
C15H9.10	32	47	21	35	5.80E-06	8.04E-06	2.48E-06	5.11E-06
C15H9.2	3	0	2	4	2.80E-06	2.65E-06	1.82E-06	2.25E-06
C15H9.3	2	5	12	2	2.80E-06	2.65E-06	1.82E-06	2.25E-06
C15H9.4	48	101	125	143	3.84E-06	7.62E-06	6.49E-06	9.15E-06
C15H9.5.1	78	130	140	160	3.64E-06	5.74E-06	4.26E-06	6.00E-06
C15H9.5.2	52	96	94	129	3.30E-06	5.77E-06	3.90E-06	6.61E-06
C15H9.6.1	4901	4538	4705	5652	2.30E-04	2.02E-04	1.44E-04	2.13E-04
C15H9.6.2	5285	4772	4905	5742	2.23E-04	1.91E-04	1.35E-04	1.95E-04
C15H9.6.3	4294	3896	3895	4642	2.40E-04	2.05E-04	1.41E-04	2.08E-04
C15H9.7	313	337	412	373	2.31E-05	2.35E-05	1.98E-05	2.21E-05
C15H9.8	11	35	16	26	3.33E-06	1.00E-05	3.15E-06	6.34E-06
C15H9.9.1	342	483	578	283	6.08E-05	8.11E-05	6.69E-05	4.04E-05
C15H9.9.2	298	410	481	264	5.75E-05	7.47E-05	6.03E-05	4.09E-05
C16A11.1	6	13	15	8	2.80E-06	2.65E-06	1.82E-06	2.25E-06
C16A11.2b.1	170	269	234	357	1.04E-05	1.55E-05	9.29E-06	1.75E-05
C16A11.2b.2	172	271	237	372	1.12E-05	1.66E-05	1.00E-05	1.94E-05
C16A11.3	124	133	144	172	6.36E-06	6.45E-06	4.81E-06	7.08E-06
C16A11.5	78	107	40	102	4.51E-06	5.87E-06	1.82E-06	4.75E-06
C16A11.6	139	256	266	161	8.93E-06	1.55E-05	1.11E-05	8.30E-06
C16A11.7	17	34	12	11	2.80E-06	5.21E-06	1.82E-06	2.25E-06
C16A11.8	1	1	3	3	2.80E-06	2.65E-06	1.82E-06	2.25E-06
C16A3.10a.1	1040	1456	1281	2000	9.04E-05	1.20E-04	7.25E-05	1.40E-04
C16A3.10a.2	1012	1426	1241	1977	8.92E-05	1.19E-04	7.12E-05	1.40E-04
C16A3.10b	913	1219	1024	1680	8.41E-05	1.06E-04	6.14E-05	1.24E-04
C16A3.10c.1	922	1308	1118	1663	8.18E-05	1.10E-04	6.46E-05	1.19E-04
C16A3.10c.2	952	1354	1166	1693	8.42E-05	1.13E-04	6.71E-05	1.20E-04
C16A3.10c.3	520	724	631	917	8.54E-05	1.12E-04	6.74E-05	1.21E-04
C16A3.11	0	1	0	1	2.80E-06	2.65E-06	1.82E-06	2.25E-06
C16A3.1b	173	207	228	356	9.41E-06	1.06E-05	8.07E-06	1.55E-05
C16A3.1c	193	230	234	368	9.66E-06	1.09E-05	7.62E-06	1.48E-05
C16A3.2	86	119	176	136	1.45E-05	1.89E-05	1.93E-05	1.84E-05
C16A3.3	1322	1317	1229	2198	2.72E-05	2.56E-05	1.65E-05	3.63E-05
C16A3.5.1	731	967	911	892	1.17E-04	1.46E-04	9.50E-05	1.15E-04
C16A3.5.2	707	925	739	784	1.25E-04	1.54E-04	8.49E-05	1.11E-04
C16A3.6	389	552	313	386	3.94E-05	5.28E-05	2.06E-05	3.14E-05
C16A3.7	1133	1142	1159	1989	3.60E-05	3.43E-05	2.40E-05	5.08E-05
C16A3.8	1121	1176	998	1738	2.82E-05	2.80E-05	1.63E-05	3.52E-05
C16A3.9.1	4766	4903	9599	4165	1.05E-03	1.02E-03	1.37E-03	7.35E-04
C16A3.9.2	3728	3862	7057	3519	8.85E-04	8.66E-04	1.09E-03	6.71E-04
C16B8.1.1	55	74	75	88	3.19E-06	4.05E-06	2.82E-06	4.09E-06
C16B8.1.2	47	68	62	84	3.00E-06	4.10E-06	2.57E-06	4.30E-06
C16B8.2	20	17	21	6	2.80E-06	2.65E-06	1.82E-06	2.25E-06
C16B8.3.1	308	268	292	200	5.73E-05	4.71E-05	3.53E-05	2.99E-05
C16B8.3.2	306	267	292	200	5.72E-05	4.72E-05	3.55E-05	3.00E-05
C16B8.4	35	28	21	30	2.80E-06	2.65E-06	1.82E-06	2.25E-06
C16B8.5	1	1	9	3	2.80E-06	2.65E-06	1.82E-06	2.25E-06
C16C10.1.1	146	190	230	297	1.50E-05	1.84E-05	1.54E-05	2.45E-05
C16C10.1.2	143	184	197	283	1.47E-05	1.79E-05	1.32E-05	2.34E-05
C16C10.10	134	215	248	207	1.78E-05	2.69E-05	2.14E-05	2.20E-05
C16C10.11.1	963	991	1008	881	1.57E-04	1.53E-04	1.07E-04	1.16E-04
C16C10.11.2	482	428	511	431	1.15E-04	9.68E-05	7.96E-05	8.29E-05
C16C10.12	12	31	20	20	2.80E-06	2.65E-06	1.82E-06	2.25E-06

C16C10.13	2	14	4	3	2.80E-06	2.65E-06	1.82E-06	2.25E-06
C16C10.2.1	204	221	289	190	2.74E-05	2.80E-05	2.52E-05	2.05E-05
C16C10.2.2	180	197	236	166	2.54E-05	2.62E-05	2.16E-05	1.88E-05
C16C10.3	3206	2613	4777	6819	1.01E-04	7.81E-05	9.84E-05	1.73E-04
C16C10.5	214	281	203	323	1.95E-05	2.42E-05	1.20E-05	2.36E-05
C16C10.6	248	260	247	457	2.16E-05	2.14E-05	1.40E-05	3.20E-05
C16C10.7	174	286	223	267	2.12E-05	3.29E-05	1.77E-05	2.61E-05
C16C10.8.1	143	127	139	159	1.98E-05	1.66E-05	1.25E-05	1.77E-05
C16C10.9	3	7	7	1	2.80E-06	2.65E-06	1.82E-06	2.25E-06
C16C2.1	28	31	34	29	7.03E-06	7.33E-06	5.54E-06	5.85E-06
C16C2.2a.1	143	179	202	206	6.30E-06	7.43E-06	5.79E-06	7.29E-06
C16C2.2a.2	81	103	149	131	6.02E-06	7.25E-06	7.22E-06	7.83E-06
C16C2.2b	84	104	148	132	5.96E-06	6.98E-06	6.85E-06	7.53E-06
C16C4.1	1	0	0	0	2.80E-06	2.65E-06	1.82E-06	2.25E-06
C16C4.10	3	2	6	9	2.80E-06	2.65E-06	1.82E-06	2.25E-06
C16C4.11	1	5	5	3	2.80E-06	2.65E-06	1.82E-06	2.25E-06
C16C4.12	3	5	6	1	2.80E-06	2.65E-06	1.82E-06	2.25E-06
C16C4.13	22	17	29	21	2.80E-06	2.65E-06	1.82E-06	2.25E-06
C16C4.14	4	1	10	4	2.80E-06	2.65E-06	1.82E-06	2.25E-06
C16C4.15	11	5	8	5	2.80E-06	2.65E-06	1.82E-06	2.25E-06
C16C4.16	9	12	11	16	2.80E-06	2.65E-06	1.82E-06	2.90E-06
C16C4.17	1	0	1	0	2.80E-06	2.65E-06	1.82E-06	2.25E-06
C16C4.2	4	5	6	3	2.80E-06	2.65E-06	1.82E-06	2.25E-06
C16C4.3	3	4	5	1	2.80E-06	2.65E-06	1.82E-06	2.25E-06
C16C4.4	20	25	18	17	2.80E-06	2.86E-06	1.82E-06	2.25E-06
C16C4.5	13	5	7	3	2.80E-06	2.65E-06	1.82E-06	2.25E-06
C16C4.6	6	6	7	3	2.80E-06	2.65E-06	1.82E-06	2.25E-06
C16C4.7	23	28	29	23	2.80E-06	2.72E-06	1.95E-06	2.25E-06
C16C4.8	0	10	7	3	2.80E-06	2.65E-06	1.82E-06	2.25E-06
C16C4.9	4	3	3	2	2.80E-06	2.65E-06	1.82E-06	2.25E-06
C16C8.10	2	1	0	4	2.80E-06	2.65E-06	1.82E-06	2.25E-06
C16C8.11	53	50	99	58	7.95E-06	7.09E-06	9.66E-06	6.99E-06
C16C8.12	68	51	65	70	1.11E-05	7.86E-06	6.89E-06	9.18E-06
C16C8.13	26	26	36	41	3.95E-06	3.73E-06	3.57E-06	5.02E-06
C16C8.14	57	56	74	83	5.35E-06	4.97E-06	4.54E-06	6.27E-06
C16C8.16	244	218	299	232	1.53E-05	1.29E-05	1.22E-05	1.17E-05
C16C8.17	3	7	2	4	2.80E-06	2.65E-06	1.82E-06	2.25E-06
C16C8.18	25	39	19	15	3.44E-06	5.08E-06	1.82E-06	2.25E-06
C16C8.19	1	0	3	0	2.80E-06	2.65E-06	1.82E-06	2.25E-06
C16C8.2	116	147	36	40	5.18E-06	6.19E-06	1.82E-06	2.25E-06
C16C8.4	80	114	100	125	1.32E-05	1.77E-05	1.07E-05	1.65E-05
C16C8.5	74	80	51	85	1.53E-05	1.56E-05	6.85E-06	1.41E-05
C16C8.7	0	5	6	0	2.80E-06	2.65E-06	1.82E-06	2.25E-06
C16C8.8	1	1	3	0	2.80E-06	2.65E-06	1.82E-06	2.25E-06
C16C8.9	3	3	1	1	2.80E-06	2.65E-06	1.82E-06	2.25E-06
C16C8.t1	0	0	3	0	2.80E-06	2.65E-06	2.99E-06	2.25E-06
C16C8.t2	0	1	1	1	2.80E-06	2.65E-06	1.82E-06	2.25E-06
C16D6.1	8	2	1	4	2.80E-06	2.65E-06	1.82E-06	2.25E-06
C16D6.2	17	21	18	21	2.80E-06	2.65E-06	1.82E-06	2.25E-06
C16D6.3	4	2	0	4	2.80E-06	2.65E-06	1.82E-06	2.25E-06
C16D9.1	23	52	25	28	2.80E-06	4.05E-06	1.82E-06	2.25E-06
C16D9.10	2	0	0	0	2.94E-06	2.65E-06	1.82E-06	2.25E-06
C16D9.2a	109	193	111	157	2.80E-06	2.78E-06	1.82E-06	2.25E-06
C16D9.2b	61	83	61	77	2.80E-06	2.65E-06	1.82E-06	2.25E-06
C16D9.2c	62	124	59	95	2.80E-06	3.15E-06	1.82E-06	2.25E-06
C16D9.3	1	2	1	1	2.80E-06	2.65E-06	1.82E-06	2.25E-06
C16D9.4	56	46	36	31	4.79E-06	3.70E-06	2.00E-06	2.25E-06
C16D9.5	20	17	18	15	2.80E-06	2.65E-06	1.82E-06	2.25E-06
C16D9.6	20	27	25	41	2.80E-06	2.80E-06	1.82E-06	3.60E-06

C16D9.7	5	4	6	2	2.80E-06	2.65E-06	1.82E-06	2.25E-06
C16D9.8	1	0	2	1	2.80E-06	2.65E-06	1.82E-06	2.25E-06
C16D9.9	7	5	8	3	2.80E-06	2.65E-06	1.82E-06	2.25E-06
C16D9.t2	0	1	6	0	2.80E-06	2.65E-06	5.34E-06	2.25E-06
C16D9.t3	1	0	0	0	2.80E-06	2.65E-06	1.82E-06	2.25E-06
C16D9.t4	0	0	2	0	2.80E-06	2.65E-06	2.00E-06	2.25E-06
C16E9.1	35	71	53	52	2.80E-06	4.34E-06	2.22E-06	2.70E-06
C16E9.2a	65	78	79	95	4.31E-06	4.89E-06	3.41E-06	5.06E-06
C16E9.2b	13	24	10	15	4.06E-06	7.09E-06	2.04E-06	3.78E-06
C16E9.4a	126	82	121	131	5.54E-06	3.41E-06	3.46E-06	4.61E-06
C16E9.4b	31	27	29	53	2.80E-06	2.65E-06	1.82E-06	3.44E-06
C16H3.1	2	6	1	2	2.80E-06	2.65E-06	1.82E-06	2.25E-06
C16H3.2	844	734	1962	586	1.61E-04	1.32E-04	2.43E-04	8.96E-05
C16H3.3a	79	103	86	159	3.25E-06	3.99E-06	2.30E-06	5.24E-06
C16H3.3b	56	68	47	119	3.08E-06	3.54E-06	1.82E-06	5.26E-06
C17A2.1	2	4	7	5	2.80E-06	2.65E-06	1.82E-06	2.25E-06
C17A2.2	3	7	5	5	2.80E-06	2.65E-06	1.82E-06	2.25E-06
C17A2.3	2	1	1	2	2.80E-06	2.65E-06	1.82E-06	2.25E-06
C17A2.4	3	5	14	6	2.80E-06	2.65E-06	1.82E-06	2.25E-06
C17A2.5	15	10	13	6	2.80E-06	2.65E-06	1.82E-06	2.25E-06
C17A2.6	8	3	4	4	2.80E-06	2.65E-06	1.82E-06	2.25E-06
C17A2.7	6	3	4	4	2.80E-06	2.65E-06	1.82E-06	2.25E-06
C17A2.8	0	6	10	1	2.80E-06	2.65E-06	1.82E-06	2.25E-06
C17B7.1	5	5	4	4	2.80E-06	2.65E-06	1.82E-06	2.25E-06
C17B7.10	6	3	7	1	2.80E-06	2.65E-06	1.82E-06	2.25E-06
C17B7.11	89	123	63	87	9.49E-06	1.24E-05	4.37E-06	7.44E-06
C17B7.12	3	3	5	3	2.80E-06	2.65E-06	1.82E-06	2.25E-06
C17B7.13	14	37	22	42	2.80E-06	3.15E-06	1.82E-06	3.04E-06
C17B7.2	22	24	17	14	2.80E-06	2.65E-06	1.82E-06	2.25E-06
C17B7.3	3	4	2	3	2.80E-06	2.65E-06	1.82E-06	2.25E-06
C17B7.4	4	5	5	1	2.80E-06	2.65E-06	1.82E-06	2.25E-06
C17B7.5	21	28	27	39	2.80E-06	2.65E-06	1.82E-06	2.25E-06
C17B7.7	43	120	47	231	2.80E-06	5.26E-06	1.82E-06	8.59E-06
C17B7.8	16	8	12	8	2.80E-06	2.65E-06	1.82E-06	2.25E-06
C17B7.9	1	1	0	7	2.80E-06	2.65E-06	1.82E-06	2.25E-06
C17C3.10	2	8	8	5	2.80E-06	2.65E-06	1.82E-06	2.25E-06
C17C3.11	8	4	6	3	2.80E-06	2.65E-06	1.82E-06	2.25E-06
C17C3.12a	39	76	21	105	2.91E-06	5.37E-06	1.82E-06	6.30E-06
C17C3.12b	32	58	14	78	3.08E-06	5.26E-06	1.82E-06	6.03E-06
C17C3.12c.1	21	52	9	73	2.80E-06	4.55E-06	1.82E-06	5.44E-06
C17C3.12c.2	26	63	11	83	2.80E-06	6.00E-06	1.82E-06	6.72E-06
C17C3.13	0	2	0	1	2.80E-06	2.65E-06	1.82E-06	2.25E-06
C17C3.14	0	0	0	1	2.80E-06	2.65E-06	1.82E-06	2.25E-06
C17C3.15	16	76	30	37	3.02E-06	1.36E-05	3.70E-06	5.62E-06
C17C3.16	1	5	11	23	2.80E-06	4.81E-06	7.29E-06	1.88E-05
C17C3.17	1	5	11	23	2.80E-06	4.81E-06	7.29E-06	1.88E-05
C17C3.18	19	26	12	39	2.80E-06	3.20E-06	1.82E-06	4.09E-06
C17C3.19	0	3	1	1	2.80E-06	2.65E-06	1.82E-06	2.25E-06
C17C3.1a	84	342	94	240	6.86E-06	2.64E-05	4.99E-06	1.58E-05
C17C3.1b	75	325	90	226	7.45E-06	3.04E-05	5.81E-06	1.80E-05
C17C3.1c	1	11	3	8	2.80E-06	6.35E-06	1.82E-06	3.94E-06
C17C3.1e	76	313	91	216	8.68E-06	3.38E-05	6.76E-06	1.98E-05
C17C3.2	0	2	0	1	2.80E-06	2.65E-06	1.82E-06	2.25E-06
C17C3.20	1	1	0	0	2.80E-06	2.65E-06	1.82E-06	2.25E-06
C17C3.3	8	10	6	5	2.80E-06	2.65E-06	1.82E-06	2.25E-06
C17C3.4	2	5	0	2	2.80E-06	2.65E-06	1.82E-06	2.25E-06
C17C3.5	3	2	1	3	2.80E-06	2.65E-06	1.82E-06	2.25E-06
C17C3.6	1	8	3	4	2.80E-06	2.65E-06	1.82E-06	2.25E-06
C17C3.7	1	5	9	4	2.80E-06	2.65E-06	1.82E-06	2.25E-06

C17C3.8	6	9	15	8	2.80E-06	2.65E-06	1.82E-06	2.25E-06
C17C3.9	3	6	5	5	2.80E-06	2.65E-06	1.82E-06	2.25E-06
C17D12.1a	276	216	270	445	3.28E-05	2.42E-05	2.09E-05	4.25E-05
C17D12.1b.1	237	188	231	402	2.85E-05	2.14E-05	1.81E-05	3.88E-05
C17D12.1b.2	276	216	270	445	3.28E-05	2.42E-05	2.09E-05	4.25E-05
C17D12.2	34	31	51	44	2.80E-06	2.65E-06	2.04E-06	2.25E-06
C17D12.3	36	36	24	25	2.88E-06	2.72E-06	1.82E-06	2.25E-06
C17D12.5	13	6	5	5	3.36E-06	2.65E-06	1.82E-06	2.25E-06
C17D12.6	13	29	17	7	2.80E-06	2.65E-06	1.82E-06	2.25E-06
C17D12.7	161	136	302	462	9.13E-06	7.30E-06	1.12E-05	2.11E-05
C17D12.t1	0	0	3	0	2.80E-06	2.65E-06	2.99E-06	2.25E-06
C17D12.t2	0	0	3	0	2.80E-06	2.65E-06	2.99E-06	2.25E-06
C17E4.1	18	16	18	11	2.80E-06	2.65E-06	1.82E-06	2.25E-06
C17E4.11	57	65	50	82	8.79E-06	9.47E-06	5.01E-06	1.02E-05
C17E4.12	6	11	7	5	2.80E-06	2.65E-06	1.82E-06	2.25E-06
C17E4.2	99	118	86	152	4.26E-06	4.79E-06	2.41E-06	5.24E-06
C17E4.3	432	496	679	832	3.73E-05	4.05E-05	3.82E-05	5.78E-05
C17E4.4	78	70	229	79	2.31E-05	1.96E-05	4.42E-05	1.88E-05
C17E4.5	802	674	682	769	8.55E-05	6.79E-05	4.73E-05	6.59E-05
C17E4.6	481	477	597	891	3.15E-05	2.95E-05	2.54E-05	4.68E-05
C17E4.9	1037	1062	1580	1470	5.57E-05	5.39E-05	5.52E-05	6.34E-05
C17E7.1	7	17	8	15	2.80E-06	2.75E-06	1.82E-06	2.25E-06
C17E7.10	3	3	3	3	2.80E-06	2.65E-06	1.82E-06	2.25E-06
C17E7.11	6	5	22	5	2.80E-06	2.65E-06	1.82E-06	2.25E-06
C17E7.12	68	44	264	112	1.93E-05	1.18E-05	4.88E-05	2.56E-05
C17E7.13	6	7	9	3	2.80E-06	2.65E-06	1.82E-06	2.25E-06
C17E7.2	2	1	6	1	2.80E-06	2.65E-06	1.82E-06	2.25E-06
C17E7.3	5	6	9	7	2.80E-06	2.65E-06	1.82E-06	2.25E-06
C17E7.4	131	150	326	284	1.30E-05	1.40E-05	2.10E-05	2.26E-05
C17E7.5	4	4	3	2	2.80E-06	2.65E-06	1.82E-06	2.25E-06
C17E7.6	4	4	1	2	2.80E-06	2.65E-06	1.82E-06	2.25E-06
C17E7.7	12	5	10	5	2.80E-06	2.65E-06	1.82E-06	2.25E-06
C17E7.8	5	3	10	11	2.80E-06	2.65E-06	1.82E-06	2.25E-06
C17E7.9a	41	64	54	58	5.35E-06	7.88E-06	4.59E-06	6.07E-06
C17E7.9b	40	62	53	61	5.80E-06	8.46E-06	4.99E-06	7.08E-06
C17F3.1	59	113	44	18	2.17E-05	3.92E-05	1.05E-05	5.31E-06
C17F3.3	44	89	24	24	6.47E-06	1.24E-05	2.30E-06	2.83E-06
C17F4.1	1	0	1	0	2.80E-06	2.65E-06	1.82E-06	2.25E-06
C17F4.10	8	13	6	2	2.80E-06	2.65E-06	1.82E-06	2.25E-06
C17F4.11	2	1	2	0	2.80E-06	2.65E-06	1.82E-06	2.25E-06
C17F4.12	0	1	13	4	2.80E-06	2.65E-06	2.62E-06	2.25E-06
C17F4.2	6	9	14	3	2.80E-06	2.65E-06	2.57E-06	2.25E-06
C17F4.3	8	6	10	2	2.80E-06	2.65E-06	1.82E-06	2.25E-06
C17F4.4	3	4	19	6	2.80E-06	2.65E-06	1.82E-06	2.25E-06
C17F4.5	127	119	191	175	1.36E-05	1.20E-05	1.33E-05	1.50E-05
C17F4.6	19	14	17	7	2.80E-06	2.65E-06	1.82E-06	2.25E-06
C17F4.7	6027	9426	10806	7785	1.07E-03	1.58E-03	1.25E-03	1.11E-03
C17F4.8	8	6	10	6	2.80E-06	2.65E-06	1.82E-06	2.25E-06
C17F4.9	1	11	5	6	2.80E-06	2.65E-06	1.82E-06	2.25E-06
C17G1.1	6	4	3	5	2.80E-06	2.65E-06	1.82E-06	2.25E-06
C17G1.2	143	149	148	232	1.90E-05	1.87E-05	1.28E-05	2.48E-05
C17G1.4a	165	168	174	331	6.64E-06	6.37E-06	4.56E-06	1.07E-05
C17G1.4b	190	189	197	351	6.89E-06	6.45E-06	4.65E-06	1.02E-05
C17G1.5	32	74	25	39	2.80E-06	6.11E-06	1.82E-06	2.74E-06
C17G1.6a	274	387	161	300	1.34E-05	1.78E-05	5.10E-06	1.17E-05
C17G1.6b	296	413	178	319	1.44E-05	1.90E-05	5.65E-06	1.25E-05
C17G1.7.1	96	192	117	137	8.65E-06	1.64E-05	6.87E-06	9.94E-06
C17G1.8	33	44	31	44	3.50E-06	4.42E-06	2.15E-06	3.76E-06
C17G10.1.1	350	404	316	496	2.49E-05	2.72E-05	1.46E-05	2.84E-05

C17G10.1.2	355	386	382	521	2.71E-05	2.78E-05	1.90E-05	3.20E-05
C17G10.2	245	276	304	426	2.14E-05	2.28E-05	1.73E-05	3.00E-05
C17G10.3	20	57	9	11	2.80E-06	5.92E-06	1.82E-06	2.25E-06
C17G10.4a	612	577	980	1492	3.21E-05	2.86E-05	3.35E-05	6.29E-05
C17G10.4b	798	785	1345	1888	2.70E-05	2.51E-05	2.96E-05	5.13E-05
C17G10.4c.1	734	714	1209	1662	3.27E-05	3.00E-05	3.51E-05	5.95E-05
C17G10.4c.2	654	621	1080	1582	3.49E-05	3.13E-05	3.75E-05	6.78E-05
C17G10.4d	619	581	983	1499	2.68E-05	2.38E-05	2.77E-05	5.22E-05
C17G10.4e	613	568	970	1491	2.72E-05	2.39E-05	2.81E-05	5.32E-05
C17G10.6a	121	104	65	75	6.61E-06	5.37E-06	2.31E-06	3.28E-06
C17G10.6b	58	48	39	37	4.03E-06	3.15E-06	1.82E-06	2.25E-06
C17G10.7	14	14	9	15	2.80E-06	2.65E-06	1.82E-06	2.25E-06
C17G10.9a.1	1289	1441	1344	1856	7.40E-05	7.81E-05	5.02E-05	8.56E-05
C17G10.9a.2	1103	1191	1128	1657	7.64E-05	7.80E-05	5.09E-05	9.22E-05
C17G10.9b	1095	1190	1127	1655	7.62E-05	7.82E-05	5.10E-05	9.25E-05
C17H1.1	11	9	12	6	2.80E-06	2.65E-06	1.82E-06	2.25E-06
C17H1.10	29	22	25	18	3.67E-06	2.65E-06	2.06E-06	2.25E-06
C17H1.11	2	1	0	1	2.80E-06	2.65E-06	1.82E-06	2.25E-06
C17H1.12	12	4	6	4	3.08E-06	2.65E-06	1.82E-06	2.25E-06
C17H1.13	7	8	4	4	2.80E-06	2.65E-06	1.82E-06	2.25E-06
C17H1.14	5	12	5	10	2.80E-06	2.65E-06	1.82E-06	2.25E-06
C17H1.2	0	0	0	1	2.80E-06	2.65E-06	1.82E-06	2.25E-06
C17H1.3	6	10	7	7	2.80E-06	2.65E-06	1.82E-06	2.25E-06
C17H1.4	6	6	4	5	2.80E-06	2.65E-06	1.82E-06	2.25E-06
C17H1.5	18	15	15	11	2.80E-06	2.65E-06	1.82E-06	2.25E-06
C17H1.6	5	8	4	3	2.80E-06	2.65E-06	1.82E-06	2.25E-06
C17H1.7	16	20	16	13	2.80E-06	2.65E-06	1.82E-06	2.25E-06
C17H1.8	7	7	7	3	2.80E-06	2.65E-06	1.82E-06	2.25E-06
C17H1.9	11	6	2	5	2.80E-06	2.65E-06	1.82E-06	2.25E-06
C17H11.1	9	14	15	5	2.80E-06	2.65E-06	1.82E-06	2.25E-06
C17H11.2	131	145	232	261	5.60E-06	5.87E-06	6.47E-06	8.97E-06
C17H11.4	15	14	41	36	4.82E-06	4.26E-06	8.58E-06	9.31E-06
C17H11.5	2	1	1	2	2.80E-06	2.65E-06	1.82E-06	2.25E-06
C17H11.6a	86	145	81	159	3.98E-06	6.32E-06	2.42E-06	5.89E-06
C17H11.6b	102	168	116	176	4.17E-06	6.51E-06	3.10E-06	5.78E-06
C17H11.6c.1	115	189	128	192	4.09E-06	6.37E-06	2.97E-06	5.51E-06
C17H11.6c.2	88	144	81	159	4.12E-06	6.37E-06	2.48E-06	5.98E-06
C17H11.6c.3	86	140	79	158	4.03E-06	6.22E-06	2.41E-06	5.96E-06
C17H12.1.1	1282	1293	1089	1653	5.79E-05	5.52E-05	3.20E-05	6.00E-05
C17H12.1.2	964	979	803	1187	5.59E-05	5.36E-05	3.03E-05	5.52E-05
C17H12.10	11	9	6	6	2.80E-06	2.65E-06	1.82E-06	2.25E-06
C17H12.11	65	96	52	57	1.18E-05	1.64E-05	6.14E-06	8.30E-06
C17H12.12	59	130	46	60	3.47E-06	7.25E-06	1.82E-06	2.83E-06
C17H12.14.1	3014	4236	2709	4582	2.71E-04	3.60E-04	1.59E-04	3.31E-04
C17H12.14.2	1714	2265	1415	2454	2.72E-04	3.40E-04	1.46E-04	3.13E-04
C17H12.2	272	275	625	734	1.51E-05	1.45E-05	2.26E-05	3.28E-05
C17H12.3	56	97	48	29	5.40E-06	8.83E-06	3.02E-06	2.25E-06
C17H12.4	118	212	104	184	7.31E-06	1.24E-05	4.19E-06	9.13E-06
C17H12.5	33	60	15	18	2.80E-06	4.76E-06	1.82E-06	2.25E-06
C17H12.6	33	13	24	11	4.20E-06	2.65E-06	1.99E-06	2.25E-06
C17H12.7	2	2	4	3	2.80E-06	2.65E-06	1.82E-06	2.25E-06
C17H12.8	981	361	846	226	8.71E-05	3.03E-05	4.89E-05	1.61E-05
C17H12.9	41	37	19	38	2.80E-06	2.65E-06	1.82E-06	2.25E-06
C18A11.1	25	42	26	11	5.91E-06	9.36E-06	3.99E-06	2.25E-06
C18A11.2	5	4	3	1	2.80E-06	2.65E-06	1.82E-06	2.25E-06
C18A11.3	110	91	78	85	1.62E-05	1.27E-05	7.49E-06	1.01E-05
C18A11.4	4	6	1	7	2.80E-06	2.65E-06	1.82E-06	2.25E-06
C18A11.5a	9	6	4	3	2.80E-06	2.65E-06	1.82E-06	2.25E-06
C18A11.5b	9	8	6	4	2.80E-06	2.65E-06	1.82E-06	2.25E-06

C18A11.5c	19	22	19	14	2.80E-06	2.65E-06	1.82E-06	2.25E-06
C18A11.6	2	1	4	1	2.80E-06	2.65E-06	1.82E-06	2.25E-06
C18A11.7a	2023	1934	1275	1905	9.68E-05	8.74E-05	3.97E-05	7.32E-05
C18A11.7b.1	2715	2746	1865	2695	1.99E-04	1.91E-04	8.91E-05	1.59E-04
C18A11.7b.2	2007	1907	1243	1864	1.84E-04	1.65E-04	7.40E-05	1.37E-04
C18A3.1	85	96	74	155	8.68E-06	9.26E-06	4.92E-06	1.27E-05
C18A3.10	12	22	19	5	3.81E-06	6.59E-06	3.92E-06	2.25E-06
C18A3.11	7	13	12	3	2.80E-06	2.65E-06	1.82E-06	2.25E-06
C18A3.12	0	1	0	0	2.80E-06	2.65E-06	1.82E-06	2.25E-06
C18A3.2	264	299	295	399	2.41E-05	2.58E-05	1.75E-05	2.93E-05
C18A3.3	387	451	222	374	3.69E-05	4.06E-05	1.38E-05	2.86E-05
C18A3.4a	160	307	154	159	1.26E-05	2.28E-05	7.87E-06	1.00E-05
C18A3.4b.1	155	291	159	150	1.03E-05	1.83E-05	6.87E-06	8.01E-06
C18A3.4b.2	122	202	104	112	1.15E-05	1.80E-05	6.40E-06	8.50E-06
C18A3.4c	85	132	74	76	1.22E-05	1.78E-05	6.89E-06	8.73E-06
C18A3.5a	979	892	897	1365	8.81E-05	7.58E-05	5.25E-05	9.86E-05
C18A3.5b	919	812	787	1201	8.96E-05	7.48E-05	4.99E-05	9.40E-05
C18A3.5c	358	344	342	488	7.16E-05	6.50E-05	4.45E-05	7.84E-05
C18A3.5e.1	979	892	897	1365	8.81E-05	7.58E-05	5.25E-05	9.86E-05
C18A3.5e.2	962	862	865	1314	8.23E-05	6.97E-05	4.82E-05	9.03E-05
C18A3.5f.1	1397	1360	1294	2034	9.07E-05	8.34E-05	5.47E-05	1.06E-04
C18A3.5f.2	1385	1338	1270	1994	9.00E-05	8.21E-05	5.37E-05	1.04E-04
C18A3.5f.3	1379	1330	1267	1987	7.49E-05	6.83E-05	4.48E-05	8.67E-05
C18A3.5f.4	962	862	865	1314	8.23E-05	6.97E-05	4.82E-05	9.03E-05
C18A3.5f.5	919	812	787	1201	8.96E-05	7.48E-05	4.99E-05	9.40E-05
C18A3.6a	83	97	81	110	7.25E-06	7.99E-06	4.61E-06	7.71E-06
C18A3.6b.1	83	96	80	110	7.62E-06	8.33E-06	4.77E-06	8.12E-06
C18A3.6b.2	83	97	81	110	7.25E-06	7.99E-06	4.61E-06	7.71E-06
C18A3.7	25	39	32	8	6.83E-06	1.01E-05	5.69E-06	2.25E-06
C18A3.8	10	5	3	14	2.80E-06	2.65E-06	1.82E-06	2.25E-06
C18A3.9	5	11	7	7	2.80E-06	3.15E-06	1.82E-06	2.25E-06
C18B10.1	1	3	2	4	2.80E-06	2.65E-06	1.82E-06	2.25E-06
C18B10.10	7	5	6	3	2.80E-06	2.65E-06	1.82E-06	2.25E-06
C18B10.2	8	7	4	4	2.80E-06	2.65E-06	1.82E-06	2.25E-06
C18B10.3	2	6	9	1	2.80E-06	2.65E-06	1.82E-06	2.25E-06
C18B10.4	3	3	9	2	2.80E-06	2.65E-06	1.82E-06	2.25E-06
C18B10.5	7	3	19	3	2.80E-06	2.65E-06	1.82E-06	2.25E-06
C18B10.6	3	9	25	5	2.80E-06	2.65E-06	1.82E-06	2.25E-06
C18B10.7	2	3	22	9	2.80E-06	2.65E-06	1.82E-06	2.25E-06
C18B10.8	2	1	2	1	2.80E-06	2.65E-06	1.82E-06	2.25E-06
C18B10.9	7	10	4	2	2.80E-06	2.65E-06	1.82E-06	2.25E-06
C18B12.1	1	1	1	1	2.80E-06	2.65E-06	1.82E-06	2.25E-06
C18B12.2	27	26	24	24	2.80E-06	2.65E-06	1.82E-06	2.25E-06
C18B12.3	5	3	3	2	2.80E-06	2.65E-06	1.82E-06	2.25E-06
C18B12.4	185	303	209	286	1.22E-05	1.88E-05	8.93E-06	1.51E-05
C18B12.5	7	13	5	5	2.80E-06	2.65E-06	1.82E-06	2.25E-06
C18B12.6	88	100	61	85	5.01E-06	5.37E-06	2.26E-06	3.89E-06
C18B2.1	2	8	10	6	2.80E-06	2.65E-06	1.82E-06	2.25E-06
C18B2.2	12	13	14	6	2.80E-06	2.65E-06	1.82E-06	2.25E-06
C18B2.3	246	268	182	152	3.84E-05	3.95E-05	1.85E-05	1.90E-05
C18B2.5a.1	1217	1117	719	1099	6.73E-05	5.83E-05	2.59E-05	4.88E-05
C18B2.5a.2	1163	1084	704	1092	6.43E-05	5.67E-05	2.53E-05	4.85E-05
C18B2.5b	1199	1132	734	1127	5.99E-05	5.35E-05	2.39E-05	4.53E-05
C18B2.6	10	6	4	9	2.80E-06	2.65E-06	1.82E-06	2.25E-06
C18C4.10a	217	302	165	288	1.49E-05	1.96E-05	7.38E-06	1.59E-05
C18C4.10b.1	291	403	222	371	1.61E-05	2.10E-05	7.98E-06	1.64E-05
C18C4.10b.2	285	386	212	363	1.59E-05	2.03E-05	7.71E-06	1.63E-05
C18C4.10b.3	291	397	220	373	1.34E-05	1.72E-05	6.60E-06	1.38E-05
C18C4.10b.4	233	325	177	316	1.58E-05	2.09E-05	7.84E-06	1.72E-05

C18C4.10b.5	233	326	179	319	1.49E-05	1.97E-05	7.47E-06	1.64E-05
C18C4.10c.1	217	307	170	293	1.35E-05	1.80E-05	6.87E-06	1.46E-05
C18C4.10c.2	217	301	163	285	1.59E-05	2.08E-05	7.76E-06	1.68E-05
C18C4.10d.1	222	304	173	290	1.50E-05	1.93E-05	7.58E-06	1.57E-05
C18C4.10d.2	220	304	172	292	1.43E-05	1.87E-05	7.29E-06	1.53E-05
C18C4.2	38	72	39	44	2.80E-06	2.65E-06	1.82E-06	2.25E-06
C18C4.3.1	131	123	94	123	9.27E-06	8.20E-06	4.32E-06	6.97E-06
C18C4.3.2	141	126	101	123	9.94E-06	8.38E-06	4.63E-06	6.97E-06
C18C4.4	1	3	4	2	2.80E-06	2.65E-06	1.82E-06	2.25E-06
C18C4.5a	249	458	142	335	8.12E-06	1.41E-05	3.01E-06	8.77E-06
C18C4.5b	252	461	142	337	8.46E-06	1.46E-05	3.10E-06	9.09E-06
C18C4.6	3	7	0	2	2.80E-06	2.91E-06	1.82E-06	2.25E-06
C18C4.7	3	7	3	0	2.80E-06	2.65E-06	1.82E-06	2.25E-06
C18C4.9	6	10	8	10	2.80E-06	2.65E-06	1.82E-06	2.25E-06
C18D1.1.1	376	382	538	685	1.47E-05	1.41E-05	1.37E-05	2.15E-05
C18D1.1.2	372	380	531	681	1.50E-05	1.44E-05	1.39E-05	2.20E-05
C18D1.2	8	19	4	8	2.80E-06	2.65E-06	1.82E-06	2.25E-06
C18D1.3	21	43	19	12	7.95E-06	1.54E-05	4.68E-06	3.64E-06
C18D1.4	3	5	5	0	2.80E-06	2.65E-06	1.82E-06	2.25E-06
C18D11.1	205	257	165	150	2.51E-05	2.98E-05	1.32E-05	1.48E-05
C18D11.2	23	30	17	28	2.86E-06	3.52E-06	1.82E-06	2.79E-06
C18D11.3	264	169	326	440	5.08E-05	3.07E-05	4.08E-05	6.80E-05
C18D11.4.1	238	270	288	335	1.95E-05	2.09E-05	1.53E-05	2.20E-05
C18D11.4.2	142	145	157	195	1.69E-05	1.63E-05	1.21E-05	1.86E-05
C18D11.6	3	9	2	2	2.80E-06	2.65E-06	1.82E-06	2.25E-06
C18D11.7	10	7	17	9	2.80E-06	2.65E-06	1.82E-06	2.25E-06
C18D4.3	0	0	2	3	2.80E-06	2.65E-06	1.82E-06	2.25E-06
C18D4.4	0	5	3	1	2.80E-06	2.65E-06	1.82E-06	2.25E-06
C18D4.5	3	5	9	7	2.80E-06	2.65E-06	1.82E-06	2.25E-06
C18D4.6a	12	23	20	40	2.80E-06	2.65E-06	1.82E-06	3.49E-06
C18D4.6b	5	13	6	18	2.80E-06	3.52E-06	1.82E-06	4.14E-06
C18D4.6c	10	25	15	37	2.80E-06	2.70E-06	1.82E-06	3.40E-06
C18D4.7	7	9	4	1	2.80E-06	2.65E-06	1.82E-06	2.25E-06
C18D4.8	9	20	10	13	2.80E-06	2.65E-06	1.82E-06	2.25E-06
C18D4.9	3	5	25	4	2.80E-06	2.65E-06	1.82E-06	2.25E-06
C18D4.t1	0	0	2	0	2.80E-06	2.65E-06	2.00E-06	2.25E-06
C18E3.1	17	18	21	13	2.80E-06	2.65E-06	1.82E-06	2.25E-06
C18E3.3	95	137	151	175	3.70E-06	5.05E-06	3.84E-06	5.49E-06
C18E3.4	1	8	6	1	2.80E-06	2.65E-06	1.82E-06	2.25E-06
C18E3.5	162	180	231	238	1.62E-05	1.70E-05	1.50E-05	1.91E-05
C18E3.6	337	422	479	671	2.57E-05	3.04E-05	2.38E-05	4.12E-05
C18E3.7a	1182	1462	1403	2413	4.58E-05	5.35E-05	3.54E-05	7.51E-05
C18E3.7b	202	221	225	380	4.00E-05	4.14E-05	2.90E-05	6.05E-05
C18E3.7c.1	1140	1407	1351	2366	4.77E-05	5.56E-05	3.68E-05	7.95E-05
C18E3.7c.2	1113	1387	1328	2350	4.66E-05	5.48E-05	3.62E-05	7.90E-05
C18E3.8	199	172	299	340	2.07E-05	1.69E-05	2.02E-05	2.84E-05
C18E9.1	18	45	28	29	2.80E-06	6.45E-06	2.77E-06	3.53E-06
C18E9.10	148	176	157	236	1.88E-05	2.11E-05	1.30E-05	2.40E-05
C18E9.11a	381	466	359	420	4.05E-05	4.68E-05	2.49E-05	3.59E-05
C18E9.11b	379	464	357	419	3.96E-05	4.58E-05	2.43E-05	3.52E-05
C18E9.3a	653	487	638	980	3.41E-05	2.40E-05	2.17E-05	4.12E-05
C18E9.3b	587	430	563	870	3.54E-05	2.45E-05	2.21E-05	4.21E-05
C18E9.3c.1	585	429	561	869	3.74E-05	2.59E-05	2.34E-05	4.47E-05
C18E9.3c.2	582	428	559	868	3.82E-05	2.65E-05	2.39E-05	4.57E-05
C18E9.3c.3	564	413	548	863	3.70E-05	2.56E-05	2.34E-05	4.54E-05
C18E9.4	459	589	1150	353	1.21E-04	1.47E-04	1.98E-04	7.49E-05
C18E9.5	226	226	208	203	3.62E-05	3.42E-05	2.17E-05	2.61E-05
C18E9.6	636	796	936	989	5.50E-05	6.51E-05	5.27E-05	6.88E-05
C18E9.7	81	85	41	41	3.14E-06	3.09E-06	1.82E-06	2.25E-06

C18E9.8	35	66	43	26	2.80E-06	3.97E-06	1.82E-06	2.25E-06
C18E9.9	160	276	245	148	2.83E-05	4.61E-05	2.82E-05	2.10E-05
C18F10.2	19	15	17	14	2.80E-06	2.65E-06	1.82E-06	2.25E-06
C18F10.4	7	3	56	20	2.80E-06	2.65E-06	4.70E-06	2.25E-06
C18F10.5	6	4	7	4	2.80E-06	2.65E-06	1.82E-06	2.25E-06
C18F10.6	5	8	1	1	2.80E-06	2.65E-06	1.82E-06	2.25E-06
C18F10.7a.1	395	533	828	927	2.28E-05	2.90E-05	3.11E-05	4.29E-05
C18F10.7a.2	367	496	762	849	2.34E-05	2.99E-05	3.17E-05	4.35E-05
C18F10.7b	282	387	596	675	5.58E-05	7.23E-05	7.67E-05	1.07E-04
C18F10.8	3	8	14	5	2.80E-06	2.65E-06	1.82E-06	2.25E-06
C18F10.9	3	2	2	3	2.80E-06	2.65E-06	1.82E-06	2.25E-06
C18F3.2a	1300	1331	1794	2715	4.24E-05	4.10E-05	3.81E-05	7.11E-05
C18F3.2b	1857	1879	2541	3919	4.27E-05	4.08E-05	3.80E-05	7.23E-05
C18F3.2c	1440	1481	2006	3060	4.03E-05	3.92E-05	3.66E-05	6.89E-05
C18F3.2d	1310	1344	1814	2747	4.05E-05	3.92E-05	3.65E-05	6.82E-05
C18F3.4	58	69	58	61	7.06E-06	7.94E-06	4.59E-06	5.96E-06
C18G1.1	4	9	3	4	2.80E-06	2.65E-06	1.82E-06	2.25E-06
C18G1.3	6	15	13	5	2.80E-06	2.65E-06	1.82E-06	2.25E-06
C18G1.4a	1030	1109	1205	1639	5.08E-05	5.17E-05	3.87E-05	6.49E-05
C18G1.4b	842	929	1039	1446	4.83E-05	5.04E-05	3.88E-05	6.67E-05
C18G1.5.1	1698	1214	2812	2517	1.96E-04	1.32E-04	2.11E-04	2.33E-04
C18G1.5.2	1514	1107	2536	2441	1.81E-04	1.25E-04	1.97E-04	2.35E-04
C18G1.6	9	11	9	7	2.80E-06	2.65E-06	1.82E-06	2.25E-06
C18G1.7	8	12	7	7	2.80E-06	2.65E-06	1.82E-06	2.25E-06
C18G1.8	8	7	7	10	2.80E-06	2.65E-06	1.82E-06	2.25E-06
C18G1.9	24	42	9	10	2.80E-06	4.52E-06	1.82E-06	2.25E-06
C18H2.1	61	77	66	36	2.80E-06	2.65E-06	1.82E-06	2.25E-06
C18H2.2	208	209	355	392	1.59E-05	1.51E-05	1.77E-05	2.41E-05
C18H2.3	17	20	12	12	2.80E-06	2.65E-06	1.82E-06	2.25E-06
C18H2.4	25	29	17	7	2.80E-06	2.65E-06	1.82E-06	2.25E-06
C18H2.5	39	52	26	21	2.80E-06	2.65E-06	1.82E-06	2.25E-06
C18H7.10	11	10	11	7	2.80E-06	2.65E-06	1.82E-06	2.25E-06
C18H7.11	56	25	43	24	4.54E-06	2.65E-06	2.26E-06	2.25E-06
C18H7.2a	17	18	6	13	2.80E-06	2.65E-06	1.82E-06	2.25E-06
C18H7.2b.1	16	17	6	13	2.80E-06	2.65E-06	1.82E-06	2.25E-06
C18H7.2b.2	12	14	5	11	2.80E-06	2.65E-06	1.82E-06	2.25E-06
C18H7.3	60	60	33	22	4.87E-06	4.60E-06	1.82E-06	2.25E-06
C18H7.4	22	34	13	14	2.80E-06	2.70E-06	1.82E-06	2.25E-06
C18H7.5	4	5	14	7	2.80E-06	2.65E-06	3.10E-06	2.25E-06
C18H7.6	12	15	10	9	2.80E-06	2.65E-06	1.82E-06	2.25E-06
C18H7.7	9	23	6	5	2.80E-06	4.73E-06	1.82E-06	2.25E-06
C18H7.8	10	5	17	7	2.80E-06	2.65E-06	1.82E-06	2.25E-06
C18H7.9	2	1	2	2	2.80E-06	2.65E-06	1.82E-06	2.25E-06
C18H9.1	10	6	5	3	2.80E-06	2.65E-06	1.82E-06	2.25E-06
C18H9.2	43	35	82	141	1.24E-05	9.58E-06	1.55E-05	3.28E-05
C18H9.3	328	326	550	1149	1.33E-05	1.25E-05	1.45E-05	3.75E-05
C18H9.5	11	55	10	33	2.80E-06	4.18E-06	1.82E-06	2.25E-06
C18H9.6	113	173	117	133	1.99E-05	2.88E-05	1.34E-05	1.88E-05
C18H9.7	76	70	73	82	4.03E-06	3.49E-06	2.51E-06	3.49E-06
C18H9.8	16	24	25	9	2.80E-06	2.65E-06	1.82E-06	2.25E-06
C23F12.1a	946	1149	696	1381	1.51E-05	1.74E-05	7.25E-06	1.78E-05
C23F12.1b	1064	1304	786	1529	1.35E-05	1.57E-05	6.51E-06	1.56E-05
C23F12.2	64	79	34	70	2.80E-06	2.96E-06	1.82E-06	2.25E-06
C23F12.3	4	0	1	1	2.80E-06	2.65E-06	1.82E-06	2.25E-06
C23F12.4	39	41	18	39	5.54E-06	5.50E-06	1.82E-06	4.45E-06
C23G10.10	3	4	2	0	2.80E-06	2.65E-06	1.82E-06	2.25E-06
C23G10.11	4	3	2	1	2.80E-06	2.65E-06	1.82E-06	2.25E-06
C23G10.1a	9	18	9	9	2.80E-06	2.65E-06	1.82E-06	2.25E-06
C23G10.1b	15	22	15	18	2.80E-06	2.65E-06	1.82E-06	2.25E-06

C23G10.2b	221	311	418	216	5.59E-05	7.43E-05	6.88E-05	4.39E-05
C23G10.3.1	10762	10351	15207	14363	1.50E-03	1.36E-03	1.38E-03	1.61E-03
C23G10.3.2	9621	9347	13643	13607	1.41E-03	1.29E-03	1.30E-03	1.60E-03
C23G10.4a.1	2643	2388	4103	4828	1.45E-04	1.24E-04	1.47E-04	2.13E-04
C23G10.4a.2	2702	2448	4185	4873	1.50E-04	1.29E-04	1.52E-04	2.18E-04
C23G10.4b	3065	2819	4875	5738	1.12E-04	9.73E-05	1.16E-04	1.68E-04
C23G10.5	3	7	6	11	2.80E-06	2.65E-06	1.82E-06	2.25E-06
C23G10.6	31	63	27	15	2.80E-06	4.10E-06	1.82E-06	2.25E-06
C23G10.7a.1	708	719	724	1023	4.25E-05	4.08E-05	2.83E-05	4.93E-05
C23G10.7a.2	461	464	474	729	4.18E-05	3.98E-05	2.80E-05	5.31E-05
C23G10.7b	695	720	706	1036	4.11E-05	4.02E-05	2.72E-05	4.92E-05
C23G10.7c	699	723	709	1045	4.17E-05	4.08E-05	2.75E-05	5.01E-05
C23G10.8	855	861	1150	1762	3.25E-05	3.09E-05	2.84E-05	5.38E-05
C23H3.1	64	84	70	112	5.52E-06	6.85E-06	3.94E-06	7.76E-06
C23H3.2a	436	536	372	475	4.05E-05	4.70E-05	2.25E-05	3.54E-05
C23H3.2b	394	484	329	432	4.19E-05	4.87E-05	2.28E-05	3.70E-05
C23H3.3	110	160	130	162	1.08E-05	1.49E-05	8.33E-06	1.28E-05
C23H3.4a.1	553	1056	642	875	4.03E-05	7.27E-05	3.04E-05	5.12E-05
C23H3.4a.2	546	1028	582	835	3.81E-05	6.77E-05	2.64E-05	4.67E-05
C23H3.4a.3	503	956	547	793	4.07E-05	7.31E-05	2.88E-05	5.15E-05
C23H3.4b.1	503	956	547	793	4.07E-05	7.31E-05	2.88E-05	5.15E-05
C23H3.4b.2	472	871	514	755	3.88E-05	6.77E-05	2.75E-05	4.99E-05
C23H3.7	20	30	18	27	2.80E-06	2.65E-06	1.82E-06	2.25E-06
C23H3.9a	77	110	49	30	4.98E-06	6.72E-06	2.06E-06	2.25E-06
C23H3.9b	70	115	48	26	4.79E-06	7.43E-06	2.13E-06	2.25E-06
C23H3.9c	58	55	37	14	4.37E-06	3.91E-06	1.82E-06	2.25E-06
C23H3.9d	80	117	58	25	5.24E-06	7.22E-06	2.46E-06	2.25E-06
C23H4.1.1	566	786	783	1026	3.12E-05	4.09E-05	2.80E-05	4.54E-05
C23H4.1.2	560	775	760	1016	2.88E-05	3.76E-05	2.54E-05	4.20E-05
C23H4.1.3	568	785	785	1029	3.14E-05	4.10E-05	2.83E-05	4.57E-05
C23H4.1.4	560	775	757	1011	3.23E-05	4.22E-05	2.84E-05	4.68E-05
C23H4.1.5	314	425	408	546	2.61E-05	3.34E-05	2.21E-05	3.64E-05
C23H4.2	10	13	21	15	2.80E-06	2.65E-06	1.82E-06	2.25E-06
C23H4.3	66	69	83	98	3.81E-06	3.76E-06	3.12E-06	4.52E-06
C23H4.4	9	23	21	11	2.80E-06	2.65E-06	1.82E-06	2.25E-06
C23H4.6	35	43	32	35	2.80E-06	2.65E-06	1.82E-06	2.25E-06
C23H4.7	50	67	44	47	2.80E-06	3.47E-06	1.82E-06	2.25E-06
C23H4.8	12	13	3	8	2.80E-06	2.65E-06	1.82E-06	2.25E-06
C23H5.1	20	26	23	22	2.80E-06	2.65E-06	1.82E-06	2.25E-06
C23H5.10	6	4	23	28	2.80E-06	2.65E-06	4.70E-06	7.08E-06
C23H5.11	1	11	8	2	2.80E-06	2.65E-06	1.82E-06	2.25E-06
C23H5.2	5	5	3	5	2.80E-06	2.65E-06	1.82E-06	2.25E-06
C23H5.3	3	1	3	3	2.80E-06	2.65E-06	1.82E-06	2.25E-06
C23H5.4	0	2	5	4	2.80E-06	2.65E-06	1.82E-06	2.25E-06
C23H5.5	5	1	13	7	2.80E-06	2.65E-06	4.35E-06	2.88E-06
C23H5.6	3	0	2	2	2.80E-06	2.65E-06	1.82E-06	2.25E-06
C23H5.7	14	9	14	6	2.80E-06	2.65E-06	1.82E-06	2.25E-06
C23H5.8a	209	410	442	463	3.37E-05	6.25E-05	4.64E-05	6.00E-05
C23H5.9	1	11	10	7	2.80E-06	2.65E-06	1.82E-06	2.25E-06
C24A1.1.1	77	138	121	60	1.59E-05	2.69E-05	1.62E-05	9.94E-06
C24A1.1.2	55	99	93	46	1.35E-05	2.30E-05	1.49E-05	9.09E-06
C24A1.2a	33	35	45	41	2.80E-06	2.72E-06	2.42E-06	2.72E-06
C24A1.2b	52	58	74	77	3.05E-06	3.23E-06	2.82E-06	3.62E-06
C24A1.3a	66	84	42	89	2.88E-06	3.47E-06	1.82E-06	3.10E-06
C24A1.3b	69	86	42	90	3.11E-06	3.65E-06	1.82E-06	3.24E-06
C24A11.1	29	49	16	7	4.82E-06	7.67E-06	1.82E-06	2.25E-06
C24A11.2	10	11	7	6	2.80E-06	2.65E-06	1.82E-06	2.25E-06
C24A11.5	1	3	3	2	2.80E-06	2.65E-06	1.82E-06	2.25E-06
C24A11.6	4	3	9	4	2.80E-06	2.65E-06	3.43E-06	2.25E-06

C24A11.8a	797	972	1016	1425	3.81E-05	4.40E-05	3.17E-05	5.48E-05
C24A11.8b	635	760	791	1097	3.67E-05	4.15E-05	2.98E-05	5.09E-05
C24A11.9	193	277	239	322	1.41E-05	1.91E-05	1.13E-05	1.88E-05
C24A3.1	13	8	8	9	2.80E-06	2.65E-06	1.82E-06	2.25E-06
C24A3.2a.1	148	276	159	100	2.65E-05	4.67E-05	1.85E-05	1.44E-05
C24A3.2a.2	146	264	156	97	1.72E-05	2.93E-05	1.19E-05	9.18E-06
C24A3.2a.3	141	259	154	95	2.72E-05	4.72E-05	1.94E-05	1.47E-05
C24A3.2b.1	115	232	143	85	2.42E-05	4.61E-05	1.96E-05	1.44E-05
C24A3.2b.2	99	208	104	81	2.20E-05	4.37E-05	1.51E-05	1.45E-05
C24A3.4	24	41	27	28	2.80E-06	4.21E-06	1.91E-06	2.45E-06
C24A3.6.1	191	278	191	291	1.11E-05	1.53E-05	7.23E-06	1.36E-05
C24A3.6.2	157	225	155	241	1.13E-05	1.52E-05	7.23E-06	1.39E-05
C24A3.8	0	1	0	0	2.80E-06	2.65E-06	1.82E-06	2.25E-06
C24A3.9	2	4	0	1	2.80E-06	2.65E-06	1.82E-06	2.25E-06
C24A8.1	14	22	12	26	2.80E-06	2.65E-06	1.82E-06	2.25E-06
C24A8.3	93	71	77	137	2.86E-06	2.65E-06	1.82E-06	3.37E-06
C24A8.4	116	185	75	212	1.02E-05	1.54E-05	4.30E-06	1.50E-05
C24A8.5	2	1	2	2	2.80E-06	2.65E-06	1.82E-06	2.25E-06
C24A8.6	16	38	10	39	2.80E-06	4.66E-06	1.82E-06	4.07E-06
C24B5.1	15	24	21	10	2.80E-06	2.65E-06	1.82E-06	2.25E-06
C24B5.2a	372	409	694	689	2.75E-05	2.86E-05	3.34E-05	4.09E-05
C24B5.3	114	306	57	113	4.40E-06	1.11E-05	1.82E-06	3.49E-06
C24B5.4	22	43	27	33	2.80E-06	4.44E-06	1.91E-06	2.90E-06
C24B5.5	3	9	0	2	2.80E-06	2.65E-06	1.82E-06	2.25E-06
C24B9.1	3	2	7	5	2.80E-06	2.65E-06	1.82E-06	2.25E-06
C24B9.10	3	4	6	8	2.80E-06	2.65E-06	1.82E-06	2.25E-06
C24B9.11	3	7	4	4	2.80E-06	2.65E-06	1.82E-06	2.25E-06
C24B9.12	6	3	6	3	2.80E-06	2.65E-06	1.82E-06	2.25E-06
C24B9.13	2	0	7	2	2.80E-06	2.65E-06	1.82E-06	2.25E-06
C24B9.14	3	7	4	1	2.80E-06	2.65E-06	1.82E-06	2.25E-06
C24B9.15	4	7	2	3	2.80E-06	2.65E-06	1.82E-06	2.25E-06
C24B9.16	2	1	5	4	2.80E-06	2.65E-06	1.82E-06	2.25E-06
C24B9.2	9	8	5	3	2.80E-06	2.65E-06	1.82E-06	2.25E-06
C24B9.3a	338	308	510	559	2.72E-05	2.35E-05	2.68E-05	3.62E-05
C24B9.3b	234	197	340	421	1.94E-05	1.54E-05	1.83E-05	2.80E-05
C24B9.4	0	4	3	1	2.80E-06	2.65E-06	1.82E-06	2.25E-06
C24B9.5	2	0	4	1	2.80E-06	2.65E-06	1.82E-06	2.25E-06
C24B9.6	3	3	2	1	2.80E-06	2.65E-06	1.82E-06	2.25E-06
C24B9.7	2	4	9	1	2.80E-06	2.65E-06	1.82E-06	2.25E-06
C24B9.8	7	6	7	7	2.80E-06	2.65E-06	1.82E-06	2.25E-06
C24B9.9	10	34	14	18	2.80E-06	8.04E-06	2.28E-06	3.62E-06
C24D10.1	29	41	26	30	2.80E-06	2.86E-06	1.82E-06	2.25E-06
C24D10.3	0	0	1	0	2.80E-06	2.65E-06	1.82E-06	2.25E-06
C24D10.4	231	214	247	303	3.68E-05	3.22E-05	2.56E-05	3.88E-05
C24D10.5	25	30	45	32	4.12E-06	4.68E-06	4.83E-06	4.25E-06
C24D10.6	217	209	231	284	4.59E-05	4.17E-05	3.18E-05	4.82E-05
C24D10.9	0	1	1	0	2.80E-06	2.65E-06	1.82E-06	2.25E-06
C24F3.1a	1265	1362	988	1659	1.27E-04	1.29E-04	6.44E-05	1.34E-04
C24F3.1b.1	1753	1897	1347	2202	1.32E-04	1.35E-04	6.59E-05	1.33E-04
C24F3.1b.2	1272	1349	992	1659	1.04E-04	1.05E-04	5.30E-05	1.09E-04
C24F3.2	78	85	91	81	9.30E-06	9.58E-06	7.07E-06	7.76E-06
C24F3.3	11	20	7	3	2.80E-06	2.65E-06	1.82E-06	2.25E-06
C24F3.4	361	499	450	626	1.83E-05	2.40E-05	1.49E-05	2.55E-05
C24F3.5	50	112	51	49	2.80E-06	2.75E-06	1.82E-06	2.25E-06
C24G6.1	210	206	267	190	3.66E-05	3.39E-05	3.03E-05	2.66E-05
C24G6.2a	97	215	68	135	3.70E-06	7.75E-06	1.82E-06	4.14E-06
C24G6.2b	35	89	23	56	2.80E-06	6.03E-06	1.82E-06	3.24E-06
C24G6.3	506	575	641	1001	1.94E-05	2.08E-05	1.60E-05	3.09E-05
C24G6.4.1	53	99	47	62	2.97E-06	5.24E-06	1.82E-06	2.79E-06

C24G6.4.2	48	93	44	57	2.80E-06	5.11E-06	1.82E-06	2.65E-06
C24G6.6.1	415	585	429	483	2.51E-05	3.35E-05	1.69E-05	2.35E-05
C24G6.6.2	424	587	443	489	2.52E-05	3.30E-05	1.72E-05	2.34E-05
C24G6.6.3	412	578	423	479	2.55E-05	3.38E-05	1.70E-05	2.38E-05
C24G6.6.4	399	550	411	466	2.65E-05	3.46E-05	1.78E-05	2.49E-05
C24G6.7	12	10	7	15	2.80E-06	2.65E-06	1.82E-06	2.25E-06
C24G6.8.1	489	603	747	779	4.37E-05	5.10E-05	4.35E-05	5.60E-05
C24G6.8.2	429	539	641	722	3.95E-05	4.69E-05	3.84E-05	5.34E-05
C24G6.8.3	489	578	728	796	4.17E-05	4.66E-05	4.04E-05	5.45E-05
C24G7.1	12	12	24	11	2.80E-06	2.65E-06	1.82E-06	2.25E-06
C24G7.2	12	44	24	25	2.80E-06	2.65E-06	1.82E-06	2.25E-06
C24G7.4	31	46	30	60	2.80E-06	2.65E-06	1.82E-06	2.45E-06
C24H10.1	18	25	16	9	2.80E-06	3.62E-06	1.82E-06	2.25E-06
C24H10.2	51	66	47	78	5.80E-06	7.09E-06	3.48E-06	7.13E-06
C24H10.3	5	14	1	7	2.80E-06	3.07E-06	1.82E-06	2.25E-06
C24H10.4	1	2	0	0	2.80E-06	2.65E-06	1.82E-06	2.25E-06
C24H10.5	370	412	371	482	4.36E-05	4.59E-05	2.85E-05	4.56E-05
C24H11.1	20	40	20	19	2.80E-06	3.68E-06	1.82E-06	2.25E-06
C24H11.2	20	40	20	18	2.80E-06	3.68E-06	1.82E-06	2.25E-06
C24H11.3	4	4	5	3	2.80E-06	2.65E-06	1.82E-06	2.25E-06
C24H11.4	2	4	1	6	2.80E-06	2.65E-06	1.82E-06	2.25E-06
C24H11.6	23	40	33	29	6.47E-06	1.06E-05	6.03E-06	6.54E-06
C24H11.7	917	914	620	1177	1.88E-05	1.77E-05	8.27E-06	1.94E-05
C24H11.9	6	15	56	27	2.80E-06	4.05E-06	1.04E-05	6.18E-06
C24H12.1	12	10	12	11	2.80E-06	2.65E-06	1.82E-06	2.25E-06
C24H12.10	1	4	20	3	2.80E-06	2.65E-06	3.55E-06	2.25E-06
C24H12.11	6	8	11	8	2.80E-06	2.65E-06	1.82E-06	2.25E-06
C24H12.12	7	3	4	3	2.80E-06	2.65E-06	1.82E-06	2.25E-06
C24H12.2	6	3	6	3	2.80E-06	2.65E-06	1.82E-06	2.25E-06
C24H12.3	7	3	6	2	2.80E-06	2.65E-06	1.82E-06	2.25E-06
C24H12.4a	381	325	462	548	2.23E-05	1.80E-05	1.76E-05	2.58E-05
C24H12.4b	355	300	325	486	2.22E-05	1.77E-05	1.32E-05	2.44E-05
C24H12.5a	228	201	364	475	1.39E-05	1.15E-05	1.44E-05	2.32E-05
C24H12.5b	216	188	324	460	1.26E-05	1.03E-05	1.23E-05	2.15E-05
C24H12.6	5	7	3	3	2.80E-06	2.65E-06	1.82E-06	2.25E-06
C24H12.7	1	5	4	0	2.80E-06	2.65E-06	1.82E-06	2.25E-06
C24H12.8	6	4	3	1	2.80E-06	2.65E-06	1.82E-06	2.25E-06
C24H12.9	6	6	7	5	2.80E-06	2.65E-06	1.82E-06	2.25E-06
C25A1.1	391	347	539	608	4.36E-05	3.66E-05	3.91E-05	5.45E-05
C25A1.10a	4330	3415	3879	6640	1.52E-04	1.13E-04	8.86E-05	1.87E-04
C25A1.10b	2444	1894	2109	3775	1.30E-04	9.51E-05	7.30E-05	1.61E-04
C25A1.11	74	152	86	127	5.15E-06	1.00E-05	3.90E-06	7.13E-06
C25A1.12	217	265	326	328	2.46E-05	2.84E-05	2.41E-05	2.99E-05
C25A1.13	189	211	385	261	3.71E-05	3.92E-05	4.92E-05	4.12E-05
C25A1.15	6	4	4	3	2.80E-06	2.65E-06	1.82E-06	2.25E-06
C25A1.2	3	5	13	2	2.80E-06	2.65E-06	1.82E-06	2.25E-06
C25A1.3	197	233	208	223	1.77E-05	1.98E-05	1.22E-05	1.61E-05
C25A1.4.1	491	553	665	742	2.45E-05	2.60E-05	2.16E-05	2.97E-05
C25A1.4.2	404	442	511	651	3.23E-05	3.34E-05	2.66E-05	4.18E-05
C25A1.5	542	596	846	1059	4.66E-05	4.84E-05	4.73E-05	7.30E-05
C25A1.7.1	497	594	650	1021	1.70E-05	1.92E-05	1.44E-05	2.80E-05
C25A1.7.2	459	566	610	981	1.61E-05	1.88E-05	1.40E-05	2.77E-05
C25A1.7.3	494	590	645	1009	1.68E-05	1.90E-05	1.43E-05	2.76E-05
C25A1.8	3461	3647	6617	5672	4.45E-04	4.42E-04	5.53E-04	5.85E-04
C25A11.1	10	6	6	7	2.80E-06	2.65E-06	1.82E-06	2.25E-06
C25A11.2	142	190	184	252	7.34E-06	9.26E-06	6.18E-06	1.04E-05
C25A11.4a	565	766	456	796	1.43E-05	1.83E-05	7.49E-06	1.61E-05
C25A11.4b	429	575	346	592	1.69E-05	2.14E-05	8.86E-06	1.87E-05
C25A11.4c	496	697	428	707	1.46E-05	1.94E-05	8.22E-06	1.68E-05

C25A11.4d	585	803	463	812	1.43E-05	1.85E-05	7.34E-06	1.59E-05
C25A11.4e	446	610	346	628	1.58E-05	2.05E-05	8.00E-06	1.79E-05
C25A6.1	6	10	3	0	2.80E-06	2.65E-06	1.82E-06	2.25E-06
C25A8.1	2	4	2	0	2.80E-06	3.70E-06	1.82E-06	2.25E-06
C25A8.2	14	24	8	2	3.16E-06	5.11E-06	1.82E-06	2.25E-06
C25A8.4	1378	1876	2156	3400	4.77E-05	6.14E-05	4.86E-05	9.45E-05
C25A8.5	26	32	12	9	2.80E-06	2.70E-06	1.82E-06	2.25E-06
C25B8.1a	18	22	10	17	2.80E-06	2.65E-06	1.82E-06	2.25E-06
C25B8.1b	21	29	14	27	2.80E-06	2.65E-06	1.82E-06	2.25E-06
C25B8.3a	3303	4217	3705	5880	2.93E-04	3.54E-04	2.14E-04	4.19E-04
C25B8.3b	3046	3783	3569	5719	2.89E-04	3.39E-04	2.20E-04	4.35E-04
C25B8.4a	83	82	34	78	1.23E-05	1.15E-05	3.30E-06	9.33E-06
C25B8.4b	54	63	21	59	7.92E-06	8.76E-06	2.00E-06	6.97E-06
C25B8.4c	66	60	25	52	1.56E-05	1.34E-05	3.84E-06	9.85E-06
C25B8.5	3	10	16	7	2.80E-06	2.65E-06	1.82E-06	2.25E-06
C25B8.6	33	53	24	24	2.80E-06	3.33E-06	1.82E-06	2.25E-06
C25B8.7	4	6	8	2	2.80E-06	2.65E-06	1.82E-06	2.25E-06
C25B8.8	6	5	4	2	2.80E-06	2.65E-06	1.82E-06	2.25E-06
C25D7.1	29	54	31	9	7.00E-06	1.23E-05	4.87E-06	2.25E-06
C25D7.10	217	250	248	265	3.12E-05	3.39E-05	2.32E-05	3.06E-05
C25D7.12	11	18	12	11	2.80E-06	2.65E-06	1.82E-06	2.25E-06
C25D7.13	14	22	8	19	3.75E-06	5.58E-06	1.82E-06	4.09E-06
C25D7.14	13	19	14	12	2.80E-06	2.65E-06	1.82E-06	2.25E-06
C25D7.15	5	9	8	4	2.80E-06	2.65E-06	1.82E-06	2.25E-06
C25D7.2	8	21	12	10	2.80E-06	2.65E-06	1.82E-06	2.25E-06
C25D7.3	199	213	148	243	3.44E-06	3.49E-06	1.82E-06	3.40E-06
C25D7.4	4	7	15	10	2.80E-06	2.65E-06	1.82E-06	2.25E-06
C25D7.5	13	22	18	22	2.80E-06	2.65E-06	1.82E-06	2.25E-06
C25D7.6	1734	1703	3127	3517	7.24E-05	6.71E-05	8.49E-05	1.18E-04
C25D7.7	168	192	157	203	3.45E-05	3.72E-05	2.10E-05	3.34E-05
C25D7.8	505	528	650	832	5.06E-05	5.00E-05	4.24E-05	6.70E-05
C25D7.9	0	0	1	1	2.80E-06	2.65E-06	1.82E-06	2.25E-06
C25E10.1	7	4	3	5	2.80E-06	2.65E-06	1.82E-06	2.25E-06
C25E10.10	32	69	18	37	7.03E-06	1.43E-05	2.57E-06	6.52E-06
C25E10.11	2	4	2	0	2.80E-06	2.65E-06	1.82E-06	2.25E-06
C25E10.12	79	115	50	108	8.06E-06	1.11E-05	3.32E-06	8.86E-06
C25E10.13	1	2	5	1	2.80E-06	2.65E-06	1.82E-06	2.25E-06
C25E10.2	7	5	7	4	2.80E-06	2.65E-06	1.82E-06	2.25E-06
C25E10.3a	29	45	52	41	2.80E-06	3.23E-06	2.57E-06	2.50E-06
C25E10.3b	26	37	45	37	2.80E-06	2.65E-06	1.84E-06	2.25E-06
C25E10.3c	5	2	4	3	2.80E-06	2.65E-06	1.82E-06	2.25E-06
C25E10.4	19	29	16	17	2.80E-06	2.65E-06	1.82E-06	2.25E-06
C25E10.5	34	69	19	32	2.80E-06	4.58E-06	1.82E-06	2.25E-06
C25E10.7	0	1	0	2	2.80E-06	2.65E-06	1.82E-06	2.25E-06
C25E10.9a	50	122	99	81	1.37E-05	3.16E-05	1.77E-05	1.79E-05
C25E10.9b.1	68	188	176	117	1.41E-05	3.68E-05	2.38E-05	1.95E-05
C25E10.9b.2	53	139	117	92	1.33E-05	3.29E-05	1.91E-05	1.85E-05
C25E10.9b.3	50	122	99	81	1.35E-05	3.10E-05	1.73E-05	1.75E-05
C25E10.9b.4	63	160	131	93	1.74E-05	4.17E-05	2.35E-05	2.06E-05
C25F6.1	51	60	53	92	5.96E-06	6.61E-06	4.03E-06	8.61E-06
C25F6.2a.1	341	347	208	416	1.29E-05	1.24E-05	5.10E-06	1.26E-05
C25F6.2a.2	468	509	309	635	1.39E-05	1.43E-05	5.96E-06	1.51E-05
C25F6.2b.1	143	174	113	183	8.96E-06	1.03E-05	4.61E-06	9.22E-06
C25F6.2b.2	124	142	78	154	1.01E-05	1.09E-05	4.12E-06	1.00E-05
C25F6.3	885	1104	1034	1234	2.98E-05	3.51E-05	2.26E-05	3.33E-05
C25F6.4	11	20	23	39	2.80E-06	2.65E-06	1.82E-06	2.25E-06
C25F6.6	2	3	6	1	2.80E-06	2.65E-06	1.82E-06	2.25E-06
C25F6.7a	101	178	54	38	7.81E-06	1.30E-05	2.71E-06	2.36E-06
C25F6.7b	65	125	27	14	7.53E-06	1.37E-05	2.04E-06	2.25E-06

C25F6.8	5	5	1	2	2.80E-06	2.65E-06	1.82E-06	2.25E-06
C25F6.t1	1	0	0	0	2.80E-06	2.65E-06	1.82E-06	2.25E-06
C25F6.t2	1	0	0	0	2.80E-06	2.65E-06	1.82E-06	2.25E-06
C25F6.t3	1	0	0	0	2.80E-06	2.65E-06	1.82E-06	2.25E-06
C25F6.t4	1	0	3	0	2.80E-06	2.65E-06	2.99E-06	2.25E-06
C25F9.1	12	4	7	5	2.80E-06	2.65E-06	1.82E-06	2.25E-06
C25F9.10	20	41	28	22	3.95E-06	7.64E-06	3.61E-06	3.49E-06
C25F9.2	22	29	22	4	2.80E-06	2.65E-06	1.82E-06	2.25E-06
C25F9.4	56	86	43	59	3.53E-06	5.11E-06	1.82E-06	2.99E-06
C25F9.5.1	44	55	38	52	2.80E-06	2.65E-06	1.82E-06	2.25E-06
C25F9.5.2	42	52	37	50	2.80E-06	2.65E-06	1.82E-06	2.25E-06
C25F9.6	7	13	26	2	2.80E-06	2.65E-06	2.20E-06	2.25E-06
C25F9.7	10	26	28	7	2.80E-06	2.83E-06	2.11E-06	2.25E-06
C25F9.8	5	6	4	1	2.80E-06	2.65E-06	1.82E-06	2.25E-06
C25F9.9	20	24	17	14	4.65E-06	5.26E-06	2.57E-06	2.61E-06
C25F9.t1	0	0	3	0	2.80E-06	2.65E-06	2.99E-06	2.25E-06
C25F9.t2	0	0	0	1	2.80E-06	2.65E-06	1.82E-06	2.25E-06
C25F9.t3	0	0	2	0	2.80E-06	2.65E-06	2.08E-06	2.25E-06
C25F9.t4	1	0	0	0	2.80E-06	2.65E-06	1.82E-06	2.25E-06
C25G4.1	4	8	7	7	2.80E-06	2.65E-06	1.82E-06	2.25E-06
C25G4.10	252	346	330	559	7.64E-06	9.89E-06	6.51E-06	1.36E-05
C25G4.11	95	118	135	137	1.06E-05	1.24E-05	9.77E-06	1.22E-05
C25G4.2	71	78	152	109	1.14E-05	1.18E-05	1.59E-05	1.40E-05
C25G4.3	53	85	71	71	9.74E-06	1.47E-05	8.49E-06	1.05E-05
C25G4.4	140	125	185	191	9.86E-06	8.31E-06	8.47E-06	1.08E-05
C25G4.5	943	822	1158	1600	2.63E-05	2.17E-05	2.11E-05	3.59E-05
C25G4.6	122	182	127	70	1.55E-05	2.18E-05	1.05E-05	7.13E-06
C25G4.7	9	9	9	5	2.80E-06	2.65E-06	1.82E-06	2.25E-06
C25G4.8	12	14	12	7	2.80E-06	2.65E-06	1.82E-06	2.25E-06
C25G4.9	1	1	2	3	2.80E-06	2.65E-06	1.82E-06	2.25E-06
C25G6.1	4	3	7	2	2.80E-06	2.65E-06	1.82E-06	2.25E-06
C25G6.2	26	65	37	47	4.09E-06	9.68E-06	3.79E-06	5.94E-06
C25G6.3	11	7	6	8	2.80E-06	2.65E-06	1.82E-06	2.25E-06
C25G6.4	5	5	4	2	2.80E-06	2.65E-06	1.82E-06	2.25E-06
C25G6.5	10	6	13	8	2.80E-06	2.65E-06	1.82E-06	2.25E-06
C25H3.1	4	10	10	9	2.80E-06	2.65E-06	1.82E-06	2.25E-06
C25H3.10a	24	88	21	31	3.02E-06	1.05E-05	1.82E-06	3.15E-06
C25H3.10b	26	84	24	30	3.19E-06	9.73E-06	1.91E-06	2.95E-06
C25H3.11	167	162	199	360	6.58E-06	6.03E-06	5.10E-06	1.14E-05
C25H3.12	28	32	14	23	5.21E-06	5.61E-06	1.82E-06	3.44E-06
C25H3.14.1	65	71	143	83	1.35E-05	1.39E-05	1.93E-05	1.38E-05
C25H3.14.2	65	72	143	83	1.33E-05	1.39E-05	1.90E-05	1.36E-05
C25H3.15	6	6	2	5	2.80E-06	2.65E-06	1.82E-06	2.25E-06
C25H3.16	9	13	12	11	2.80E-06	3.02E-06	1.91E-06	2.25E-06
C25H3.3	54	59	134	104	1.35E-05	1.40E-05	2.18E-05	2.09E-05
C25H3.4	254	347	294	449	1.65E-05	2.13E-05	1.24E-05	2.35E-05
C25H3.5	25	38	32	34	5.07E-06	7.27E-06	4.23E-06	5.53E-06
C25H3.6a	450	524	672	940	2.56E-05	2.82E-05	2.49E-05	4.30E-05
C25H3.6b	168	170	238	331	2.37E-05	2.26E-05	2.18E-05	3.75E-05
C25H3.6c	30	38	67	48	8.79E-06	1.05E-05	1.28E-05	1.13E-05
C25H3.7a	113	181	107	108	1.97E-05	2.99E-05	1.22E-05	1.52E-05
C25H3.7b	252	407	272	259	2.94E-05	4.49E-05	2.07E-05	2.43E-05
C25H3.8	804	817	830	1442	1.30E-05	1.25E-05	8.71E-06	1.87E-05
C26B2.1	196	198	251	331	1.68E-05	1.60E-05	1.40E-05	2.28E-05
C26B2.2	15	19	24	15	2.80E-06	3.07E-06	2.68E-06	2.25E-06
C26B2.3a.1	382	444	387	683	1.77E-05	1.94E-05	1.16E-05	2.54E-05
C26B2.3a.2	265	304	264	518	1.68E-05	1.82E-05	1.09E-05	2.64E-05
C26B2.3b.1	359	418	366	653	1.70E-05	1.87E-05	1.13E-05	2.48E-05
C26B2.3b.2	58	65	52	80	1.25E-05	1.33E-05	7.32E-06	1.39E-05

C26B2.3c.1	254	294	255	496	1.73E-05	1.89E-05	1.13E-05	2.71E-05
C26B2.3c.2	284	323	278	535	1.72E-05	1.85E-05	1.10E-05	2.61E-05
C26B2.3c.3	253	291	254	494	1.72E-05	1.87E-05	1.13E-05	2.70E-05
C26B2.3c.4	265	304	264	518	1.68E-05	1.82E-05	1.09E-05	2.64E-05
C26B2.4	3	18	18	9	2.80E-06	2.65E-06	1.82E-06	2.25E-06
C26B2.5	1	3	1	2	2.80E-06	2.65E-06	1.82E-06	2.25E-06
C26B2.6	177	225	302	334	1.85E-05	2.21E-05	2.05E-05	2.80E-05
C26B2.7	126	146	275	110	2.61E-05	2.85E-05	3.70E-05	1.83E-05
C26B2.8	20	29	8	10	2.80E-06	2.72E-06	1.82E-06	2.25E-06
C26B9.1a	84	116	100	116	4.70E-06	6.14E-06	3.64E-06	5.22E-06
C26B9.1b	51	81	60	84	4.82E-06	7.25E-06	3.70E-06	6.39E-06
C26B9.2	11	6	6	4	2.80E-06	2.65E-06	1.82E-06	2.25E-06
C26B9.3	127	223	52	53	1.58E-05	2.61E-05	4.19E-06	5.29E-06
C26B9.5	505	690	762	769	3.32E-05	4.29E-05	3.26E-05	4.06E-05
C26B9.6	7	18	12	17	2.80E-06	2.65E-06	1.82E-06	2.25E-06
C26B9.7	14	20	25	6	3.89E-06	5.26E-06	4.54E-06	2.25E-06
C26C6.1a	1970	1650	2631	3712	3.68E-05	2.91E-05	3.20E-05	5.56E-05
C26C6.1b.1	704	668	978	1398	4.91E-05	4.40E-05	4.44E-05	7.83E-05
C26C6.1b.2	666	637	937	1340	4.44E-05	4.01E-05	4.07E-05	7.17E-05
C26C6.1b.3	664	633	936	1337	4.63E-05	4.17E-05	4.25E-05	7.49E-05
C26C6.2	1494	1345	1472	2398	8.29E-05	7.05E-05	5.32E-05	1.07E-04
C26C6.3	111	113	86	42	5.74E-06	5.53E-06	2.90E-06	2.25E-06
C26C6.4a	9	11	12	9	2.80E-06	2.65E-06	1.82E-06	2.25E-06
C26C6.4b	6	5	6	6	2.80E-06	2.65E-06	1.82E-06	2.25E-06
C26C6.5a	614	561	644	1033	3.14E-05	2.71E-05	2.14E-05	4.24E-05
C26C6.5b	627	568	654	1044	3.19E-05	2.73E-05	2.17E-05	4.27E-05
C26C6.6	8	19	7	7	2.80E-06	4.89E-06	1.82E-06	2.25E-06
C26C6.7	4	10	9	5	2.80E-06	2.65E-06	1.82E-06	2.25E-06
C26C6.8	7	3	4	7	2.80E-06	2.65E-06	1.82E-06	2.25E-06
C26C6.9	104	129	100	77	1.72E-05	2.02E-05	1.08E-05	1.03E-05
C26D10.1	1148	1304	1900	2412	5.95E-05	6.38E-05	6.40E-05	1.00E-04
C26D10.2a	2543	2814	2680	3490	1.87E-04	1.96E-04	1.28E-04	2.06E-04
C26D10.2b.1	1334	1333	1588	1792	1.46E-04	1.38E-04	1.13E-04	1.58E-04
C26D10.2b.2	1242	1248	1393	1690	1.72E-04	1.63E-04	1.26E-04	1.88E-04
C26D10.3	19	39	136	43	2.80E-06	3.04E-06	7.31E-06	2.86E-06
C26D10.4	27	51	31	38	2.80E-06	2.65E-06	1.82E-06	2.25E-06
C26D10.5a	52	101	47	61	2.80E-06	4.58E-06	1.82E-06	2.34E-06
C26D10.5b	42	96	44	58	2.80E-06	4.55E-06	1.82E-06	2.34E-06
C26D10.5c	10	30	14	19	2.80E-06	5.08E-06	1.82E-06	2.72E-06
C26D10.5d	20	57	25	32	2.80E-06	5.11E-06	1.82E-06	2.43E-06
C26D10.6a.1	61	106	60	122	3.92E-06	6.43E-06	2.51E-06	6.30E-06
C26D10.6a.2	55	98	53	111	3.89E-06	6.53E-06	2.44E-06	6.30E-06
C26D10.7	13	40	13	22	2.80E-06	2.65E-06	1.82E-06	2.25E-06
C26E1.1	4	2	3	0	2.80E-06	2.65E-06	1.82E-06	2.25E-06
C26E1.2	32	73	31	44	2.80E-06	5.50E-06	1.82E-06	2.83E-06
C26E1.3	51	44	42	41	3.42E-06	2.78E-06	1.82E-06	2.25E-06
C26E6.1	3	5	3	2	2.80E-06	2.65E-06	1.82E-06	2.25E-06
C26E6.11	160	184	192	239	2.36E-05	2.56E-05	1.84E-05	2.83E-05
C26E6.12	216	231	162	229	1.97E-05	1.99E-05	9.60E-06	1.68E-05
C26E6.2	150	160	176	313	8.82E-06	8.89E-06	6.74E-06	1.48E-05
C26E6.4	1223	1186	1540	2367	3.46E-05	3.17E-05	2.84E-05	5.38E-05
C26E6.5	609	533	791	1042	5.19E-05	4.29E-05	4.38E-05	7.13E-05
C26E6.7b	338	400	581	691	1.99E-05	2.22E-05	2.22E-05	3.26E-05
C26E6.8.1	242	355	386	442	1.55E-05	2.14E-05	1.61E-05	2.27E-05
C26E6.8.2	214	324	326	410	1.47E-05	2.11E-05	1.46E-05	2.26E-05
C26E6.9a	603	651	621	1031	1.47E-05	1.50E-05	9.84E-06	2.02E-05
C26E6.9b	239	284	261	457	1.18E-05	1.33E-05	8.42E-06	1.82E-05
C26E6.9c	542	565	575	972	1.32E-05	1.30E-05	9.09E-06	1.90E-05
C26F1.1a	24	33	24	22	7.14E-06	9.26E-06	4.65E-06	5.24E-06

C26F1.1b	80	88	39	33	1.37E-05	1.42E-05	4.34E-06	4.52E-06
C26F1.2	19	106	24	45	2.80E-06	6.80E-06	1.82E-06	2.45E-06
C26F1.3	289	285	388	214	5.01E-05	4.67E-05	4.38E-05	2.98E-05
C26F1.4.1	4485	4543	13829	4135	1.01E-03	9.69E-04	2.03E-03	7.50E-04
C26F1.4.2	3700	3783	10855	3681	9.19E-04	8.87E-04	1.75E-03	7.34E-04
C26F1.5	39	69	30	20	4.68E-06	7.83E-06	2.35E-06	2.25E-06
C26F1.6	6	8	4	4	2.80E-06	2.65E-06	1.82E-06	2.25E-06
C26F1.9.1	6822	5293	14787	4691	3.16E-03	2.31E-03	4.45E-03	1.74E-03
C26F1.9.2	4430	3455	9857	3451	2.07E-03	1.52E-03	2.99E-03	1.29E-03
C26G2.1	72	75	65	124	2.80E-06	2.65E-06	1.82E-06	2.74E-06
C26G2.2	51	77	54	95	2.80E-06	2.86E-06	1.82E-06	2.99E-06
C26G2.t1	0	0	1	0	2.80E-06	2.65E-06	1.82E-06	2.25E-06
C26H9A.1	559	731	945	1239	1.79E-05	2.21E-05	1.97E-05	3.19E-05
C26H9A.2	103	101	103	163	2.88E-06	2.67E-06	1.88E-06	3.67E-06
C27A12.10	150	165	184	157	2.18E-05	2.27E-05	1.74E-05	1.84E-05
C27A12.2	403	497	402	695	2.22E-05	2.59E-05	1.44E-05	3.08E-05
C27A12.3	317	285	411	636	2.12E-05	1.80E-05	1.79E-05	3.41E-05
C27A12.4	4	8	2	3	2.80E-06	2.65E-06	1.82E-06	2.25E-06
C27A12.6	324	450	522	633	1.63E-05	2.13E-05	1.71E-05	2.55E-05
C27A12.7a.1	296	375	470	619	2.22E-05	2.65E-05	2.29E-05	3.72E-05
C27A12.7a.2	286	370	435	595	1.95E-05	2.39E-05	1.94E-05	3.27E-05
C27A12.7b.1	321	405	502	648	1.77E-05	2.11E-05	1.80E-05	2.87E-05
C27A12.7b.2	304	395	457	605	2.03E-05	2.49E-05	1.98E-05	3.24E-05
C27A12.7b.3	296	375	470	619	2.22E-05	2.65E-05	2.29E-05	3.72E-05
C27A12.7b.4	286	370	435	595	1.95E-05	2.39E-05	1.94E-05	3.27E-05
C27A12.7b.5	283	370	433	592	2.06E-05	2.54E-05	2.05E-05	3.46E-05
C27A12.7b.6	283	369	432	592	2.12E-05	2.62E-05	2.11E-05	3.57E-05
C27A12.8.1	407	554	461	728	2.44E-05	3.14E-05	1.80E-05	3.51E-05
C27A12.8.2	330	443	387	608	2.49E-05	3.15E-05	1.90E-05	3.68E-05
C27A12.9	225	224	329	449	9.18E-06	8.62E-06	8.73E-06	1.47E-05
C27A2.1	884	1068	983	1770	2.95E-05	3.37E-05	2.14E-05	4.75E-05
C27A2.2a.1	4977	5210	14047	4127	1.23E-03	1.21E-03	2.25E-03	8.16E-04
C27A2.2a.2	4002	4185	10976	3671	1.02E-03	1.01E-03	1.83E-03	7.54E-04
C27A2.2b	3595	3804	10737	3359	8.23E-04	8.23E-04	1.60E-03	6.18E-04
C27A2.4	32	48	20	20	2.80E-06	2.65E-06	1.82E-06	2.25E-06
C27A2.5	6	20	4	7	2.80E-06	3.73E-06	1.82E-06	2.25E-06
C27A2.6	498	492	730	833	2.48E-05	2.31E-05	2.37E-05	3.33E-05
C27A2.7	0	1	8	0	2.80E-06	2.65E-06	1.82E-06	2.25E-06
C27A2.8	2	9	3	2	2.80E-06	2.65E-06	1.82E-06	2.25E-06
C27A2.t1	0	0	1	0	2.80E-06	2.65E-06	1.82E-06	2.25E-06
C27A7.1a	113	209	111	179	4.79E-06	8.38E-06	3.06E-06	6.09E-06
C27A7.1b	109	202	108	175	4.93E-06	8.62E-06	3.19E-06	6.36E-06
C27A7.2	12	16	10	3	2.80E-06	2.65E-06	1.82E-06	2.25E-06
C27A7.3a	26	44	24	46	2.80E-06	2.65E-06	1.82E-06	2.25E-06
C27A7.3b	25	42	19	45	2.80E-06	2.65E-06	1.82E-06	2.25E-06
C27A7.4	30	37	21	17	2.80E-06	2.65E-06	1.82E-06	2.25E-06
C27A7.5a	191	290	257	358	9.32E-06	1.34E-05	8.16E-06	1.40E-05
C27A7.5b	12	16	17	22	2.80E-06	3.28E-06	2.41E-06	3.85E-06
C27A7.5c	179	277	246	347	9.32E-06	1.36E-05	8.35E-06	1.45E-05
C27A7.6	330	349	455	617	2.53E-05	2.52E-05	2.26E-05	3.79E-05
C27A7.7	1	6	3	7	2.80E-06	2.65E-06	1.82E-06	2.25E-06
C27A7.8	14	36	6	19	6.38E-06	1.55E-05	1.82E-06	6.95E-06
C27A7.9	0	1	0	0	2.80E-06	2.65E-06	1.82E-06	2.25E-06
C27B7.1a	429	479	506	671	5.12E-05	5.40E-05	3.93E-05	6.43E-05
C27B7.1b	540	605	678	800	5.32E-05	5.63E-05	4.35E-05	6.33E-05
C27B7.2	107	118	121	109	1.69E-05	1.76E-05	1.24E-05	1.38E-05
C27B7.4	626	822	739	1367	1.69E-05	2.09E-05	1.30E-05	2.96E-05
C27B7.5	370	387	574	585	3.01E-05	2.97E-05	3.04E-05	3.82E-05
C27B7.6	26	27	23	28	2.80E-06	2.65E-06	1.82E-06	2.25E-06

C27B7.7	76	126	89	100	2.80E-06	2.91E-06	1.82E-06	2.25E-06
C27B7.8	472	456	544	706	7.74E-05	7.07E-05	5.81E-05	9.30E-05
C27B7.9	99	148	91	158	4.06E-05	5.73E-05	2.43E-05	5.21E-05
C27C12.1	67	87	90	120	6.83E-06	8.38E-06	5.98E-06	9.85E-06
C27C12.2	143	152	181	203	7.67E-06	7.70E-06	6.30E-06	8.75E-06
C27C12.3	160	145	244	280	1.49E-05	1.27E-05	1.48E-05	2.09E-05
C27C12.4	82	111	72	124	6.78E-06	8.68E-06	3.88E-06	8.25E-06
C27C12.5	33	38	35	31	2.80E-06	2.65E-06	1.82E-06	2.25E-06
C27C12.6	4	7	5	1	2.80E-06	2.65E-06	1.82E-06	2.25E-06
C27C12.7	190	267	177	391	8.32E-06	1.11E-05	5.05E-06	1.38E-05
C27C7.1	817	677	1715	1254	8.54E-05	6.68E-05	1.17E-04	1.05E-04
C27C7.2	1	3	0	0	2.80E-06	2.65E-06	1.82E-06	2.25E-06
C27C7.3	13	13	7	13	2.80E-06	2.65E-06	1.82E-06	2.25E-06
C27C7.4	26	40	20	7	2.80E-06	3.94E-06	1.82E-06	2.25E-06
C27C7.5	1	0	0	1	2.80E-06	2.65E-06	1.82E-06	2.25E-06
C27C7.7	8	8	7	2	2.80E-06	2.65E-06	1.82E-06	2.25E-06
C27C7.8	3	18	9	8	2.80E-06	3.09E-06	1.82E-06	2.25E-06
C27D11.1	2895	2914	3769	5394	9.06E-05	8.61E-05	7.67E-05	1.36E-04
C27D6.1	12	18	14	9	2.80E-06	2.65E-06	1.82E-06	2.25E-06
C27D6.10	6	6	10	2	2.80E-06	2.65E-06	1.82E-06	2.25E-06
C27D6.11	23	50	20	24	2.80E-06	5.26E-06	1.82E-06	2.25E-06
C27D6.12a	16	14	34	5	7.76E-06	6.40E-06	1.07E-05	2.25E-06
C27D6.12b	6	10	18	2	4.98E-06	7.83E-06	9.71E-06	2.25E-06
C27D6.3	84	90	20	17	2.41E-05	2.44E-05	3.74E-06	3.91E-06
C27D6.4a	223	347	258	371	3.61E-05	5.31E-05	2.72E-05	4.82E-05
C27D6.4b.1	313	423	405	557	3.48E-05	4.44E-05	2.93E-05	4.97E-05
C27D6.4b.2	304	405	387	550	3.39E-05	4.26E-05	2.80E-05	4.92E-05
C27D6.4c	617	731	873	1160	3.33E-05	3.73E-05	3.07E-05	5.03E-05
C27D6.6	3	4	9	6	2.80E-06	2.65E-06	1.82E-06	2.25E-06
C27D6.7	5	4	1	2	2.80E-06	2.65E-06	1.82E-06	2.25E-06
C27D6.8	3	6	10	10	2.80E-06	2.65E-06	1.82E-06	2.25E-06
C27D6.9	5	6	9	3	2.80E-06	2.65E-06	1.82E-06	2.25E-06
C27D8.1	53	64	38	30	5.54E-06	6.32E-06	2.59E-06	2.52E-06
C27D8.2	50	97	34	41	3.58E-06	6.59E-06	1.82E-06	2.36E-06
C27D8.3a	156	211	157	198	5.54E-06	7.09E-06	3.64E-06	5.67E-06
C27D8.3b	131	193	126	177	5.32E-06	7.41E-06	3.32E-06	5.76E-06
C27D8.4	139	150	181	285	1.53E-05	1.56E-05	1.30E-05	2.52E-05
C27D9.1	609	518	788	1070	4.02E-05	3.23E-05	3.39E-05	5.68E-05
C27D9.2	5	0	4	0	2.80E-06	2.65E-06	1.82E-06	2.25E-06
C27F2.1	62	30	46	34	3.44E-06	2.65E-06	1.82E-06	2.25E-06
C27F2.2	74	85	70	60	2.80E-06	2.65E-06	1.82E-06	2.25E-06
C27F2.4	303	252	339	369	3.08E-05	2.42E-05	2.24E-05	3.02E-05
C27F2.5.1	383	481	581	617	3.23E-05	3.83E-05	3.18E-05	4.17E-05
C27F2.5.2	183	270	238	312	2.53E-05	3.52E-05	2.14E-05	3.46E-05
C27F2.6	5	11	4	1	2.80E-06	2.65E-06	1.82E-06	2.25E-06
C27F2.7	117	144	149	162	1.07E-05	1.24E-05	8.86E-06	1.19E-05
C27F2.8	1953	1721	2728	3852	3.41E-05	2.84E-05	3.10E-05	5.41E-05
C27F2.9	222	222	301	281	2.79E-05	2.64E-05	2.47E-05	2.84E-05
C27H2.2a	87	155	64	117	3.28E-06	5.50E-06	1.82E-06	3.53E-06
C27H5.1	5	14	9	10	2.80E-06	3.09E-06	1.82E-06	2.25E-06
C27H5.2a	28	66	26	53	2.80E-06	4.07E-06	1.82E-06	2.79E-06
C27H5.2b	25	61	34	49	2.80E-06	3.89E-06	1.82E-06	2.65E-06
C27H5.2c	24	62	24	51	2.80E-06	4.26E-06	1.82E-06	2.99E-06
C27H5.2d	26	64	37	50	2.80E-06	3.91E-06	1.82E-06	2.59E-06
C27H5.3.1	385	433	302	357	2.58E-05	2.74E-05	1.32E-05	1.92E-05
C27H5.3.2	324	330	259	315	2.28E-05	2.19E-05	1.19E-05	1.78E-05
C27H5.4a	89	108	54	84	6.05E-06	6.93E-06	2.39E-06	4.59E-06
C27H5.4b	92	113	56	80	6.64E-06	7.70E-06	2.62E-06	4.63E-06
C27H5.5	34	31	22	19	3.47E-06	2.99E-06	1.82E-06	2.25E-06

C27H5.6	3	7	11	7	2.80E-06	2.65E-06	1.82E-06	2.25E-06
C27H5.7a	11	11	14	10	2.80E-06	2.65E-06	1.82E-06	2.25E-06
C27H5.7b.1	49	46	41	33	3.08E-06	2.75E-06	1.82E-06	2.25E-06
C27H5.7b.2	12	13	14	10	2.80E-06	2.65E-06	1.82E-06	2.25E-06
C27H5.8	26	44	159	65	2.80E-06	2.70E-06	6.71E-06	3.40E-06
C27H6.1	111	129	123	93	2.80E-06	2.65E-06	1.82E-06	2.25E-06
C27H6.2	910	770	1175	1702	6.07E-05	4.85E-05	5.10E-05	9.11E-05
C27H6.3	446	520	585	777	2.44E-05	2.69E-05	2.08E-05	3.41E-05
C27H6.4a	599	956	844	818	7.35E-05	1.11E-04	6.74E-05	8.06E-05
C27H6.4b	660	1052	890	894	6.00E-05	9.04E-05	5.27E-05	6.53E-05
C27H6.8.1	598	670	623	859	5.69E-05	6.02E-05	3.86E-05	6.56E-05
C27H6.8.2	522	590	502	728	5.52E-05	5.90E-05	3.45E-05	6.18E-05
C28A5.1	487	510	992	1156	1.44E-05	1.43E-05	1.91E-05	2.75E-05
C28A5.2	439	474	827	1025	1.31E-05	1.33E-05	1.60E-05	2.45E-05
C28A5.3	114	250	108	177	1.32E-05	2.74E-05	8.14E-06	1.65E-05
C28A5.4	27	24	19	25	2.94E-06	2.65E-06	1.82E-06	2.25E-06
C28A5.5	0	1	2	0	2.80E-06	2.65E-06	1.82E-06	2.25E-06
C28A5.6	65	167	62	63	2.80E-06	5.53E-06	1.82E-06	2.25E-06
C28A5.7	0	2	4	1	2.80E-06	2.65E-06	2.86E-06	2.25E-06
C28A5.t3	1	0	3	0	2.80E-06	2.65E-06	3.04E-06	2.25E-06
C28C12.1	28	44	21	22	5.24E-06	7.75E-06	2.55E-06	3.31E-06
C28C12.10	426	434	458	831	1.49E-05	1.43E-05	1.04E-05	2.33E-05
C28C12.11	38	53	21	20	5.57E-06	7.33E-06	2.00E-06	2.36E-06
C28C12.12.1	443	428	577	676	3.84E-05	3.50E-05	3.25E-05	4.71E-05
C28C12.12.2	354	351	490	584	3.76E-05	3.52E-05	3.39E-05	4.98E-05
C28C12.13	5	3	2	3	2.80E-06	2.65E-06	1.82E-06	2.25E-06
C28C12.2	687	643	1855	833	1.19E-04	1.05E-04	2.09E-04	1.16E-04
C28C12.3	2	3	3	4	2.80E-06	2.65E-06	1.82E-06	2.25E-06
C28C12.4	18	45	19	10	2.86E-06	6.72E-06	1.95E-06	2.25E-06
C28C12.5	467	553	526	489	3.77E-05	4.22E-05	2.76E-05	3.17E-05
C28C12.7a.1	1152	1756	1508	1795	1.55E-04	2.23E-04	1.32E-04	1.94E-04
C28C12.7a.2	1008	1488	1272	1559	1.57E-04	2.19E-04	1.29E-04	1.95E-04
C28C12.7b	1122	1657	1413	1699	9.28E-05	1.29E-04	7.61E-05	1.13E-04
C28C12.8	2	4	7	0	2.80E-06	2.65E-06	1.82E-06	2.25E-06
C28C12.9a.1	338	354	337	471	1.99E-05	1.97E-05	1.29E-05	2.22E-05
C28C12.9b	338	354	337	471	1.99E-05	1.97E-05	1.29E-05	2.23E-05
C28C12.9c	178	180	148	174	1.88E-05	1.80E-05	1.02E-05	1.48E-05
C28D4.1.1	83	171	43	88	5.99E-06	1.17E-05	2.02E-06	5.11E-06
C28D4.1.2	79	162	37	85	6.38E-06	1.24E-05	1.95E-06	5.53E-06
C28D4.10	2	4	1	1	2.80E-06	2.65E-06	1.82E-06	2.25E-06
C28D4.11	10	12	13	12	2.80E-06	2.65E-06	1.82E-06	2.25E-06
C28D4.2	300	434	361	554	1.97E-05	2.69E-05	1.54E-05	2.92E-05
C28D4.3.1	1666	1479	2598	2875	1.34E-04	1.13E-04	1.36E-04	1.86E-04
C28D4.3.2	1429	1260	2092	2548	1.39E-04	1.16E-04	1.33E-04	2.00E-04
C28D4.4	66	78	31	38	6.97E-06	7.78E-06	2.13E-06	3.22E-06
C28D4.5	79	119	34	46	8.34E-06	1.18E-05	2.33E-06	3.89E-06
C28D4.6	1	1	1	3	2.80E-06	2.65E-06	1.82E-06	2.25E-06
C28D4.7	56	108	31	20	6.16E-06	1.12E-05	2.22E-06	2.25E-06
C28D4.8	40	62	21	23	4.23E-06	6.19E-06	1.82E-06	2.25E-06
C28D4.9	145	308	97	183	9.77E-06	1.96E-05	4.25E-06	9.90E-06
C28F5.1	2	1	1	1	2.80E-06	2.65E-06	1.82E-06	2.25E-06
C28F5.2	6	10	10	2	2.80E-06	2.65E-06	1.82E-06	2.25E-06
C28F5.4	11	18	11	6	2.80E-06	2.65E-06	1.82E-06	2.25E-06
C28G1.1	30	39	27	35	2.80E-06	2.65E-06	1.82E-06	2.25E-06
C28G1.2	4	9	10	8	2.80E-06	2.65E-06	1.82E-06	2.25E-06
C28G1.4	26	31	20	33	2.80E-06	2.65E-06	1.82E-06	2.25E-06
C28G1.5	96	103	59	58	1.98E-05	2.00E-05	7.91E-06	9.58E-06
C28G1.6	25	24	23	11	2.97E-06	2.70E-06	1.82E-06	2.25E-06
C28H8.1	192	242	505	270	3.41E-05	4.07E-05	5.84E-05	3.85E-05

C28H8.11a	344	516	260	367	3.17E-05	4.49E-05	1.56E-05	2.71E-05
C28H8.11b.1	175	295	134	260	2.57E-05	4.09E-05	1.28E-05	3.07E-05
C28H8.11b.2	167	265	125	253	2.52E-05	3.78E-05	1.23E-05	3.07E-05
C28H8.11c.1	353	555	277	374	3.07E-05	4.57E-05	1.57E-05	2.62E-05
C28H8.11c.2	157	231	111	72	4.30E-05	5.98E-05	1.98E-05	1.58E-05
C28H8.11c.3	182	282	147	113	3.03E-05	4.44E-05	1.59E-05	1.51E-05
C28H8.12	332	415	300	437	3.29E-05	3.89E-05	1.94E-05	3.48E-05
C28H8.13	12	30	29	29	3.70E-06	8.76E-06	5.83E-06	7.20E-06
C28H8.2	11	12	7	4	2.80E-06	2.65E-06	1.82E-06	2.25E-06
C28H8.3.1	3297	3452	3709	5537	6.81E-05	6.73E-05	4.98E-05	9.18E-05
C28H8.3.2	3162	3337	3555	5338	6.51E-05	6.49E-05	4.76E-05	8.83E-05
C28H8.4	798	804	659	729	1.01E-04	9.62E-05	5.43E-05	7.42E-05
C28H8.5a	18	41	3	4	2.80E-06	4.76E-06	1.82E-06	2.25E-06
C28H8.5b	18	34	3	3	2.80E-06	4.71E-06	1.82E-06	2.25E-06
C28H8.6a	79	114	108	135	9.46E-06	1.29E-05	8.42E-06	1.30E-05
C28H8.6b	78	126	109	138	1.06E-05	1.63E-05	9.69E-06	1.51E-05
C28H8.7	3	1	5	3	2.80E-06	2.65E-06	1.82E-06	2.25E-06
C28H8.8	5	7	3	2	2.80E-06	2.65E-06	1.82E-06	2.25E-06
C28H8.9a	395	395	623	767	3.10E-05	2.93E-05	3.18E-05	4.83E-05
C28H8.9b	212	201	281	305	4.10E-05	3.67E-05	3.54E-05	4.74E-05
C29A12.1	261	323	341	371	3.63E-05	4.24E-05	3.08E-05	4.14E-05
C29A12.2	31	46	23	35	2.80E-06	2.65E-06	1.82E-06	2.25E-06
C29A12.3a	795	779	1095	1655	3.69E-05	3.41E-05	3.31E-05	6.17E-05
C29A12.3b	208	172	227	305	3.56E-05	2.78E-05	2.53E-05	4.19E-05
C29A12.4	81	89	82	82	2.80E-06	2.65E-06	1.82E-06	2.25E-06
C29A12.6	17	13	9	8	2.80E-06	2.65E-06	1.82E-06	2.25E-06
C29E4.1	628	812	360	256	6.63E-05	8.10E-05	2.47E-05	2.17E-05
C29E4.10	12	17	14	12	2.80E-06	2.65E-06	1.82E-06	2.25E-06
C29E4.12	193	173	465	158	4.43E-05	3.75E-05	6.94E-05	2.91E-05
C29E4.13	191	151	196	235	2.97E-05	2.22E-05	1.98E-05	2.94E-05
C29E4.14	17	21	18	8	5.07E-06	5.92E-06	3.50E-06	2.25E-06
C29E4.3a	656	916	923	1316	2.24E-05	2.95E-05	2.05E-05	3.61E-05
C29E4.3b	633	907	884	1279	2.29E-05	3.09E-05	2.08E-05	3.71E-05
C29E4.4	610	677	1078	1516	1.92E-05	2.02E-05	2.21E-05	3.84E-05
C29E4.5a	131	172	135	170	6.94E-06	8.60E-06	4.65E-06	7.22E-06
C29E4.5b	31	30	25	43	8.20E-06	7.51E-06	4.30E-06	9.15E-06
C29E4.7	44	76	33	45	6.55E-06	1.07E-05	3.19E-06	5.38E-06
C29E4.8.1	951	1042	1046	1157	9.81E-05	1.02E-04	7.02E-05	9.59E-05
C29E4.8.2	687	751	713	744	1.02E-04	1.05E-04	6.86E-05	8.83E-05
C29E4.9	73	64	82	43	3.63E-05	3.01E-05	2.66E-05	1.72E-05
C29E6.1a	124	180	124	149	6.66E-06	9.13E-06	4.34E-06	6.43E-06
C29E6.1b.1	96	162	92	121	4.82E-06	7.64E-06	2.99E-06	4.86E-06
C29E6.1b.2	84	143	80	113	4.79E-06	7.70E-06	2.97E-06	5.17E-06
C29E6.2	76	106	88	126	2.80E-06	3.07E-06	1.82E-06	3.10E-06
C29E6.3	46	64	33	20	3.98E-06	5.24E-06	1.86E-06	2.25E-06
C29E6.4	9	7	20	13	2.80E-06	2.65E-06	1.82E-06	2.25E-06
C29E6.5	32	84	22	10	2.80E-06	5.40E-06	1.82E-06	2.25E-06
C29F3.1	28	64	34	60	2.80E-06	2.86E-06	1.82E-06	2.29E-06
C29F3.2	22	52	15	26	2.80E-06	3.04E-06	1.82E-06	2.25E-06
C29F3.3	4	4	16	2	2.80E-06	2.65E-06	3.81E-06	2.25E-06
C29F3.4	1	3	2	0	2.80E-06	2.65E-06	1.82E-06	2.25E-06
C29F3.5	12	35	16	14	2.80E-06	6.85E-06	2.17E-06	2.34E-06
C29F3.6	12	21	9	12	2.80E-06	2.65E-06	1.82E-06	2.25E-06
C29F4.2	47	105	43	75	3.00E-06	6.32E-06	1.82E-06	3.85E-06
C29F4.3	8	12	23	6	2.80E-06	2.65E-06	1.82E-06	2.25E-06
C29F5.1	271	377	208	314	2.51E-05	3.30E-05	1.26E-05	2.34E-05
C29F5.2	12	10	13	5	2.80E-06	2.65E-06	1.82E-06	2.25E-06
C29F5.3	14	9	9	6	2.80E-06	2.65E-06	1.82E-06	2.25E-06
C29F5.4a	6	10	6	5	2.80E-06	2.65E-06	1.82E-06	2.25E-06

C29F5.4b	8	13	6	6	2.80E-06	2.65E-06	1.82E-06	2.25E-06
C29F5.5	3	1	3	1	2.80E-06	2.65E-06	1.82E-06	2.25E-06
C29F5.7	9	15	4	4	2.80E-06	2.67E-06	1.82E-06	2.25E-06
C29F5.8	2	2	4	0	2.80E-06	2.65E-06	1.82E-06	2.25E-06
C29F7.1	16	37	29	32	2.80E-06	3.31E-06	1.82E-06	2.43E-06
C29F7.2	287	310	205	237	2.48E-05	2.53E-05	1.15E-05	1.64E-05
C29F7.3	233	338	241	230	3.47E-05	4.76E-05	2.34E-05	2.76E-05
C29F7.4	52	70	56	54	4.54E-06	5.77E-06	3.17E-06	3.78E-06
C29F7.5	78	105	114	85	3.81E-06	4.84E-06	3.63E-06	3.33E-06
C29F7.6	23	21	48	39	2.80E-06	2.65E-06	1.82E-06	2.25E-06
C29F7.7	1	0	0	1	2.80E-06	2.65E-06	1.82E-06	2.25E-06
C29F7.8	4	2	1	0	2.80E-06	2.65E-06	1.82E-06	2.25E-06
C29F7.t1	0	0	1	0	2.80E-06	2.65E-06	1.82E-06	2.25E-06
C29F7.t2	3	2	3	2	4.09E-06	2.65E-06	2.66E-06	2.25E-06
C29F7.t3	0	0	1	0	2.80E-06	2.65E-06	1.82E-06	2.25E-06
C29F9.1	11	17	12	16	2.80E-06	2.65E-06	1.82E-06	2.25E-06
C29F9.10	7	13	7	5	2.80E-06	2.65E-06	1.82E-06	2.25E-06
C29F9.11	17	21	13	17	2.80E-06	2.65E-06	1.82E-06	2.25E-06
C29F9.12	20	14	36	8	1.18E-05	7.83E-06	1.39E-05	3.80E-06
C29F9.13	0	2	1	0	2.80E-06	2.65E-06	1.82E-06	2.25E-06
C29F9.2	14	31	16	7	3.00E-06	6.27E-06	2.22E-06	2.25E-06
C29F9.3a.1	59	70	27	56	7.03E-06	7.88E-06	2.10E-06	5.38E-06
C29F9.3a.2	55	66	25	51	6.66E-06	7.54E-06	1.97E-06	4.95E-06
C29F9.3b	57	68	26	57	5.82E-06	6.56E-06	1.82E-06	4.68E-06
C29F9.3c	53	59	23	46	6.78E-06	7.14E-06	1.91E-06	4.72E-06
C29F9.4	61	47	31	52	7.53E-06	5.50E-06	2.50E-06	5.17E-06
C29F9.5	2	2	1	1	2.80E-06	2.65E-06	1.82E-06	2.25E-06
C29F9.6	5	3	9	6	2.80E-06	2.65E-06	1.82E-06	2.25E-06
C29F9.8	2	2	10	2	2.80E-06	2.65E-06	1.82E-06	2.25E-06
C29F9.9	1	0	10	3	2.80E-06	2.65E-06	1.82E-06	2.25E-06
C29G2.1	9	11	8	7	2.80E-06	2.83E-06	1.82E-06	2.25E-06
C29G2.2	8	9	7	11	3.02E-06	3.20E-06	1.82E-06	3.33E-06
C29G2.3	1	5	4	4	2.80E-06	2.65E-06	1.82E-06	2.25E-06
C29G2.4	5	4	5	2	2.80E-06	2.65E-06	1.82E-06	2.25E-06
C29G2.5	6	7	6	2	2.80E-06	2.65E-06	1.82E-06	2.25E-06
C29G2.6	32	71	54	65	5.12E-06	1.07E-05	5.61E-06	8.34E-06
C29H12.1	293	301	351	492	1.86E-05	1.81E-05	1.45E-05	2.51E-05
C29H12.2.1	1417	1393	1593	1961	1.04E-04	9.63E-05	7.58E-05	1.15E-04
C29H12.2.2	1352	1337	1492	1905	1.28E-04	1.19E-04	9.16E-05	1.44E-04
C29H12.3a	19	23	24	20	2.80E-06	2.65E-06	1.82E-06	2.25E-06
C29H12.3b	23	26	18	18	2.80E-06	2.65E-06	1.82E-06	2.25E-06
C29H12.3c	16	17	15	13	2.80E-06	2.65E-06	1.82E-06	2.25E-06
C29H12.5	189	210	261	396	9.97E-06	1.05E-05	8.96E-06	1.68E-05
C29H12.6	26	36	19	16	2.80E-06	3.15E-06	1.82E-06	2.25E-06
C30A5.10a	11	15	11	7	2.80E-06	2.65E-06	1.82E-06	2.25E-06
C30A5.10b	13	15	11	6	2.80E-06	2.65E-06	1.82E-06	2.25E-06
C30A5.2	178	268	133	261	7.48E-06	1.07E-05	3.64E-06	8.82E-06
C30A5.3.1	647	713	818	949	7.03E-05	7.32E-05	5.78E-05	8.28E-05
C30A5.3.2	446	422	512	640	7.35E-05	6.57E-05	5.49E-05	8.47E-05
C30A5.4	14	19	11	8	2.80E-06	2.65E-06	1.82E-06	2.25E-06
C30A5.5	4	3	3	0	2.80E-06	2.65E-06	1.82E-06	2.25E-06
C30A5.6	7	9	8	3	2.80E-06	2.65E-06	1.82E-06	2.25E-06
C30A5.7a	14	19	9	14	2.80E-06	2.65E-06	1.82E-06	2.25E-06
C30A5.7b	28	30	15	29	2.80E-06	2.65E-06	1.82E-06	2.25E-06
C30B5.1	939	907	1247	1773	4.41E-05	4.02E-05	3.81E-05	6.68E-05
C30B5.2a	339	487	555	352	5.14E-05	6.97E-05	5.47E-05	4.28E-05
C30B5.2b	212	290	400	220	5.38E-05	6.96E-05	6.61E-05	4.49E-05
C30B5.3	30	86	30	19	2.80E-06	5.16E-06	1.82E-06	2.25E-06
C30B5.4	324	275	156	245	3.99E-05	3.20E-05	1.25E-05	2.42E-05

C30B5.5	28	36	13	8	2.80E-06	2.65E-06	1.82E-06	2.25E-06
C30B5.6	48	92	36	38	3.08E-06	5.55E-06	1.82E-06	2.25E-06
C30B5.7	4	12	2	7	2.80E-06	2.65E-06	1.82E-06	2.25E-06
C30C11.1	236	250	218	223	4.47E-05	4.47E-05	2.68E-05	3.39E-05
C30C11.4.1	4541	4589	3990	5802	1.88E-04	1.79E-04	1.07E-04	1.93E-04
C30C11.4.2	4101	4061	3738	5311	1.95E-04	1.83E-04	1.16E-04	2.03E-04
C30E1.2	2	3	4	3	2.80E-06	2.65E-06	1.82E-06	2.25E-06
C30E1.3	1	3	6	1	2.80E-06	2.65E-06	1.82E-06	2.25E-06
C30E1.4	9	7	5	1	2.80E-06	2.65E-06	1.82E-06	2.25E-06
C30E1.5	3	4	1	1	2.80E-06	2.65E-06	1.82E-06	2.25E-06
C30E1.6	2	4	5	6	2.80E-06	2.65E-06	1.82E-06	2.25E-06
C30E1.7	3	4	2	2	2.80E-06	2.65E-06	1.82E-06	2.25E-06
C30E1.8	4	14	9	7	2.80E-06	2.65E-06	1.82E-06	2.25E-06
C30E1.t1	0	1	0	1	2.80E-06	2.65E-06	1.82E-06	2.25E-06
C30F12.1	81	119	86	146	8.29E-06	1.15E-05	5.72E-06	1.20E-05
C30F12.2.1	318	250	291	465	2.19E-05	1.62E-05	1.30E-05	2.57E-05
C30F12.2.2	266	208	243	376	2.22E-05	1.64E-05	1.32E-05	2.52E-05
C30F12.3	4	11	6	6	2.80E-06	2.65E-06	1.82E-06	2.25E-06
C30F12.4	512	304	658	440	6.08E-05	3.41E-05	5.09E-05	4.20E-05
C30F12.5	180	261	156	177	1.64E-05	2.25E-05	9.27E-06	1.30E-05
C30F12.6	52	113	17	30	3.92E-06	8.01E-06	1.82E-06	2.25E-06
C30F12.7	142	246	148	95	1.29E-05	2.11E-05	8.75E-06	6.93E-06
C30F2.1	7	2	4	3	2.80E-06	2.65E-06	1.82E-06	2.25E-06
C30F2.2	8	8	11	8	2.80E-06	2.65E-06	1.82E-06	2.25E-06
C30F2.3	33	45	16	18	2.91E-06	3.76E-06	1.82E-06	2.25E-06
C30F2.4	1	1	9	0	2.80E-06	2.65E-06	3.08E-06	2.25E-06
C30F2.5	9	9	8	12	2.80E-06	2.65E-06	1.82E-06	2.25E-06
C30F8.2.1	1870	2491	2037	3293	1.43E-04	1.80E-04	1.01E-04	2.02E-04
C30F8.2.2	1352	1745	1440	2570	1.42E-04	1.73E-04	9.82E-05	2.16E-04
C30F8.2.3	1361	1759	1465	2592	1.43E-04	1.75E-04	1.00E-04	2.19E-04
C30F8.2.4	1328	1709	1384	2489	1.41E-04	1.71E-04	9.56E-05	2.12E-04
C30F8.3	11	47	5	8	2.80E-06	5.03E-06	1.82E-06	2.25E-06
C30F8.4a.1	55	81	55	85	2.80E-06	3.07E-06	1.82E-06	2.72E-06
C30F8.4a.2	52	73	51	83	2.80E-06	2.94E-06	1.82E-06	2.83E-06
C30F8.4b.1	52	71	50	83	2.80E-06	2.75E-06	1.82E-06	2.74E-06
C30F8.4b.2	52	73	51	83	2.80E-06	2.94E-06	1.82E-06	2.83E-06
C30G12.1	37	36	57	27	2.94E-06	2.70E-06	2.95E-06	2.25E-06
C30G12.2	627	288	346	480	8.00E-05	3.47E-05	2.87E-05	4.92E-05
C30G12.3	1	1	3	2	2.80E-06	2.65E-06	1.82E-06	2.25E-06
C30G12.4	13	30	11	5	2.80E-06	4.07E-06	1.82E-06	2.25E-06
C30G12.6a	460	513	501	856	1.54E-05	1.62E-05	1.09E-05	2.30E-05
C30G12.6b	456	511	502	850	1.54E-05	1.63E-05	1.10E-05	2.31E-05
C30G12.7	823	786	1427	1955	5.19E-05	4.68E-05	5.85E-05	9.90E-05
C30G4.2	1	2	0	4	2.80E-06	2.65E-06	1.82E-06	2.25E-06
C30G4.3	25	34	37	34	2.80E-06	2.65E-06	1.82E-06	2.25E-06
C30G4.4b	19	28	19	17	3.00E-06	4.15E-06	1.95E-06	2.25E-06
C30G4.5	1	7	0	3	2.80E-06	2.88E-06	1.82E-06	2.25E-06
C30G4.6	7	7	8	5	2.80E-06	2.65E-06	1.82E-06	2.25E-06
C30G4.7	26	39	31	25	3.92E-06	5.55E-06	3.04E-06	3.04E-06
C30G7.1	33	42	29	40	4.28E-06	5.16E-06	2.46E-06	4.18E-06
C30G7.2	16	26	27	15	3.14E-06	4.81E-06	3.44E-06	2.36E-06
C30G7.3	31	68	44	20	3.72E-06	7.72E-06	3.44E-06	2.25E-06
C30G7.5	4	5	4	1	2.80E-06	2.65E-06	1.82E-06	2.25E-06
C30H6.1	7	4	8	5	2.80E-06	2.65E-06	1.82E-06	2.25E-06
C30H6.10	9	14	13	1	2.80E-06	3.41E-06	2.19E-06	2.25E-06
C30H6.11	55	53	36	37	3.95E-06	3.60E-06	1.82E-06	2.25E-06
C30H6.12	18	8	11	9	3.11E-06	2.65E-06	1.82E-06	2.25E-06
C30H6.2	22	24	15	12	2.80E-06	2.65E-06	1.82E-06	2.25E-06
C30H6.3	1	2	1	3	2.80E-06	2.65E-06	1.82E-06	2.25E-06

C30H6.4	9	6	9	10	2.80E-06	2.65E-06	1.82E-06	2.25E-06
C30H6.5	76	86	51	31	5.66E-06	6.03E-06	2.46E-06	2.25E-06
C30H6.6	30	54	40	57	2.80E-06	2.70E-06	1.82E-06	2.43E-06
C30H6.7	589	768	662	878	6.03E-05	7.43E-05	4.41E-05	7.22E-05
C30H6.8	90	136	102	74	1.67E-05	2.38E-05	1.23E-05	1.10E-05
C30H6.9	141	166	259	194	2.42E-05	2.69E-05	2.90E-05	2.68E-05
C30H7.2a.1	482	627	490	759	4.56E-05	5.60E-05	3.01E-05	5.76E-05
C30H7.2a.2	461	601	463	746	4.36E-05	5.37E-05	2.85E-05	5.67E-05
C30H7.2a.3	463	603	463	747	4.07E-05	5.00E-05	2.65E-05	5.27E-05
C30H7.2a.4	463	603	463	747	3.94E-05	4.85E-05	2.56E-05	5.11E-05
C30H7.2b	652	802	691	997	4.22E-05	4.90E-05	2.91E-05	5.18E-05
C31A11.1	12	7	20	8	2.80E-06	2.65E-06	1.82E-06	2.25E-06
C31A11.10	10	13	4	1	2.80E-06	2.65E-06	1.82E-06	2.25E-06
C31A11.3	2	3	2	2	2.80E-06	2.65E-06	1.82E-06	2.25E-06
C31A11.4	14	8	11	9	2.80E-06	2.65E-06	1.82E-06	2.25E-06
C31A11.5	37	52	42	26	2.80E-06	2.65E-06	1.82E-06	2.25E-06
C31A11.6	2	4	6	1	2.80E-06	2.65E-06	1.82E-06	2.25E-06
C31A11.7	11	23	18	20	2.80E-06	2.65E-06	1.82E-06	2.25E-06
C31A11.9	4	7	2	3	2.80E-06	2.65E-06	1.82E-06	2.25E-06
C31B8.1	2	3	7	4	2.80E-06	2.65E-06	1.82E-06	2.25E-06
C31B8.10	6	0	1	1	2.80E-06	2.65E-06	1.82E-06	2.25E-06
C31B8.11	6	5	3	6	2.80E-06	2.65E-06	1.82E-06	2.25E-06
C31B8.12	6	10	5	9	2.80E-06	2.65E-06	1.82E-06	2.25E-06
C31B8.13	5	6	3	2	2.80E-06	2.65E-06	1.82E-06	2.25E-06
C31B8.14	3	4	22	7	2.80E-06	2.65E-06	1.82E-06	2.25E-06
C31B8.16	4	3	4	1	2.80E-06	2.65E-06	1.82E-06	2.25E-06
C31B8.2	2	2	3	0	2.80E-06	2.65E-06	1.82E-06	2.25E-06
C31B8.3	4	8	3	5	2.80E-06	2.65E-06	1.82E-06	2.25E-06
C31B8.4	12	19	9	10	2.80E-06	2.65E-06	1.82E-06	2.25E-06
C31B8.6	3	2	6	7	2.80E-06	2.65E-06	1.82E-06	2.25E-06
C31B8.7	16	23	16	18	2.80E-06	2.65E-06	1.82E-06	2.25E-06
C31B8.8	72	106	79	95	4.17E-06	5.82E-06	2.99E-06	4.43E-06
C31B8.9	4	1	9	0	2.80E-06	2.65E-06	1.82E-06	2.25E-06
C31C9.1a	144	216	141	132	1.05E-05	1.48E-05	6.67E-06	7.71E-06
C31C9.1b	136	206	136	128	1.04E-05	1.50E-05	6.80E-06	7.89E-06
C31C9.2.1	251	352	154	148	2.59E-05	3.43E-05	1.03E-05	1.23E-05
C31C9.2.2	196	300	118	125	2.09E-05	3.02E-05	8.18E-06	1.07E-05
C31C9.3	20	24	20	20	2.80E-06	2.65E-06	1.82E-06	2.25E-06
C31C9.4	5	9	15	7	2.80E-06	2.65E-06	1.82E-06	2.25E-06
C31C9.6	66	76	42	79	2.80E-06	2.78E-06	1.82E-06	2.45E-06
C31C9.7	133	69	120	16	3.17E-05	1.55E-05	1.86E-05	3.06E-06
C31E10.1	3	7	3	4	2.80E-06	2.65E-06	1.82E-06	2.25E-06
C31E10.3	7	6	7	11	2.80E-06	2.65E-06	1.82E-06	2.25E-06
C31E10.4	1	2	5	0	2.80E-06	2.65E-06	1.82E-06	2.25E-06
C31E10.5	132	162	165	283	5.96E-06	6.90E-06	4.85E-06	1.03E-05
C31E10.6	50	57	83	91	2.83E-06	3.04E-06	3.04E-06	4.12E-06
C31E10.7.1	333	549	468	328	6.94E-05	1.08E-04	6.35E-05	5.49E-05
C31E10.7.2	291	474	387	303	6.08E-05	9.36E-05	5.26E-05	5.09E-05
C31E10.8	34	58	36	76	2.80E-06	2.65E-06	1.82E-06	2.25E-06
C31G12.1	20	18	25	22	3.72E-06	3.17E-06	3.04E-06	3.31E-06
C31G12.2	4	5	0	0	2.80E-06	2.65E-06	1.82E-06	2.25E-06
C31G12.3	1	1	0	1	2.80E-06	2.65E-06	1.82E-06	2.25E-06
C31G12.4	5	7	8	6	2.80E-06	2.65E-06	1.82E-06	2.25E-06
C31H1.1	53	77	71	37	2.86E-06	3.91E-06	2.48E-06	2.25E-06
C31H1.2	9	11	13	4	2.80E-06	2.65E-06	1.82E-06	2.25E-06
C31H1.5	28	89	18	15	3.30E-06	9.95E-06	1.82E-06	2.25E-06
C31H1.6a	65	132	45	59	6.38E-06	1.23E-05	2.88E-06	4.66E-06
C31H1.6b	47	111	23	44	6.19E-06	1.38E-05	1.97E-06	4.66E-06
C31H1.7	2	3	2	0	2.80E-06	2.65E-06	1.82E-06	2.25E-06

C31H1.8	919	764	1393	1720	5.17E-05	4.06E-05	5.10E-05	7.77E-05
C31H2.1a	96	138	102	179	5.15E-06	6.98E-06	3.55E-06	7.69E-06
C31H2.1b	126	191	143	235	5.88E-06	8.44E-06	4.35E-06	8.82E-06
C31H2.2	247	377	183	69	1.89E-05	2.72E-05	9.11E-06	4.23E-06
C31H2.3	6	6	3	1	2.80E-06	2.65E-06	1.82E-06	2.25E-06
C31H2.4	28	30	26	17	2.86E-06	2.91E-06	1.82E-06	2.25E-06
C31H5.1	1	4	4	1	2.80E-06	2.65E-06	1.82E-06	2.25E-06
C31H5.2	15	15	8	4	2.80E-06	2.65E-06	1.82E-06	2.25E-06
C31H5.3	15	19	19	14	2.80E-06	2.65E-06	1.82E-06	2.25E-06
C31H5.5	8	30	8	11	2.80E-06	6.61E-06	1.82E-06	2.25E-06
C31H5.6	140	228	110	170	9.27E-06	1.42E-05	4.74E-06	9.02E-06
C31H5.7	2	4	7	3	2.80E-06	2.65E-06	1.82E-06	2.25E-06
C32A3.1a	169	166	230	406	1.20E-05	1.11E-05	1.06E-05	2.31E-05
C32A3.1b.1	169	166	230	406	1.20E-05	1.11E-05	1.06E-05	2.31E-05
C32A3.1b.2	157	158	225	398	1.17E-05	1.11E-05	1.09E-05	2.38E-05
C32A3.2	95	76	137	159	1.02E-05	7.72E-06	9.58E-06	1.37E-05
C32A3.3a.1	289	309	422	609	1.86E-05	1.88E-05	1.76E-05	3.14E-05
C32A3.3a.2	244	260	355	541	2.05E-05	2.06E-05	1.94E-05	3.65E-05
C32A3.3a.3	240	257	350	541	2.02E-05	2.05E-05	1.92E-05	3.67E-05
C32A3.3b	237	253	340	532	1.88E-05	1.90E-05	1.75E-05	3.39E-05
C32A9.1	4	2	5	3	2.80E-06	2.65E-06	1.82E-06	2.25E-06
C32B5.10	37	49	72	78	3.25E-06	4.07E-06	4.12E-06	5.51E-06
C32B5.11	2	4	10	3	2.80E-06	2.65E-06	1.82E-06	2.25E-06
C32B5.12	3	0	3	1	2.80E-06	2.65E-06	1.82E-06	2.25E-06
C32B5.13	2	0	0	1	2.80E-06	2.65E-06	1.82E-06	2.25E-06
C32B5.14	13	10	3	9	2.80E-06	2.65E-06	1.82E-06	2.25E-06
C32B5.15	3	3	3	4	2.80E-06	2.65E-06	1.82E-06	2.25E-06
C32B5.16	7	6	3	7	2.80E-06	2.65E-06	1.82E-06	2.25E-06
C32B5.17	6	8	13	6	2.80E-06	2.65E-06	2.55E-06	2.25E-06
C32B5.1a	19	16	23	22	2.80E-06	2.65E-06	1.82E-06	2.25E-06
C32B5.1b	6	8	7	12	2.80E-06	2.65E-06	1.82E-06	2.25E-06
C32B5.2	8	15	23	11	2.80E-06	2.65E-06	1.82E-06	2.25E-06
C32B5.3	2	3	3	0	2.80E-06	2.65E-06	1.82E-06	2.25E-06
C32B5.4	8	3	5	2	2.80E-06	2.65E-06	1.82E-06	2.25E-06
C32B5.5	5	7	4	5	2.80E-06	2.65E-06	1.82E-06	2.25E-06
C32B5.6	0	5	10	1	2.80E-06	2.65E-06	1.82E-06	2.25E-06
C32B5.7	4	7	6	7	2.80E-06	2.65E-06	1.82E-06	2.25E-06
C32B5.8	2	3	6	1	2.80E-06	2.65E-06	1.82E-06	2.25E-06
C32B5.9	2	6	7	3	2.80E-06	2.65E-06	1.82E-06	2.25E-06
C32C4.1	13	16	6	17	2.80E-06	2.65E-06	1.82E-06	2.25E-06
C32C4.2	5	15	8	6	2.80E-06	2.65E-06	1.82E-06	2.25E-06
C32C4.3	17	21	14	5	2.80E-06	2.65E-06	1.82E-06	2.25E-06
C32C4.5a	3	2	10	3	2.80E-06	2.65E-06	1.82E-06	2.25E-06
C32C4.5b	3	1	8	1	2.80E-06	2.65E-06	2.73E-06	2.25E-06
C32C4.7	8	10	16	6	2.80E-06	2.65E-06	1.82E-06	2.25E-06
C32D5.1	12	24	11	8	2.80E-06	3.25E-06	1.82E-06	2.25E-06
C32D5.10	267	308	394	587	1.35E-05	1.47E-05	1.30E-05	2.38E-05
C32D5.11	657	576	943	1274	2.91E-05	2.41E-05	2.71E-05	4.53E-05
C32D5.12	39	80	68	41	3.75E-06	7.27E-06	4.26E-06	3.17E-06
C32D5.14	2	2	2	6	2.80E-06	2.65E-06	1.82E-06	2.25E-06
C32D5.3	634	755	976	1313	2.62E-05	2.95E-05	2.63E-05	4.36E-05
C32D5.4	30	35	21	9	2.94E-06	3.23E-06	1.82E-06	2.25E-06
C32D5.6	59	83	39	85	3.67E-06	4.87E-06	1.82E-06	4.25E-06
C32D5.7	26	66	44	41	2.80E-06	5.34E-06	2.46E-06	2.81E-06
C32D5.8a.1	323	501	335	416	5.99E-05	8.78E-05	4.04E-05	6.20E-05
C32D5.8a.2	328	506	352	438	5.32E-05	7.76E-05	3.72E-05	5.71E-05
C32D5.8b	273	416	276	336	6.07E-05	8.73E-05	3.99E-05	6.00E-05
C32D5.9.1	1331	2483	1165	1759	2.11E-04	3.71E-04	1.20E-04	2.24E-04
C32D5.9.2	1169	2139	986	1573	1.92E-04	3.32E-04	1.06E-04	2.08E-04

C32E12.1	58	85	30	34	3.92E-06	5.42E-06	1.82E-06	2.25E-06
C32E12.2	6	10	11	5	2.80E-06	2.65E-06	1.82E-06	2.25E-06
C32E12.3	69	91	32	24	3.67E-06	4.55E-06	1.82E-06	2.25E-06
C32E12.4	192	261	125	216	4.51E-06	5.79E-06	1.91E-06	4.07E-06
C32E12.5.1	146	150	103	162	8.04E-06	7.80E-06	3.68E-06	7.15E-06
C32E12.5.2	76	69	42	79	6.97E-06	5.98E-06	2.50E-06	5.82E-06
C32E8.1	5	4	16	2	2.80E-06	2.65E-06	2.51E-06	2.25E-06
C32E8.10a	348	267	578	848	2.21E-05	1.60E-05	2.39E-05	4.33E-05
C32E8.10b	336	257	541	789	2.25E-05	1.62E-05	2.35E-05	4.24E-05
C32E8.10c	355	274	585	844	2.00E-05	1.46E-05	2.14E-05	3.82E-05
C32E8.10d	326	243	507	748	2.27E-05	1.60E-05	2.30E-05	4.19E-05
C32E8.10e	299	227	458	680	2.39E-05	1.71E-05	2.38E-05	4.36E-05
C32E8.10f	373	287	619	894	2.27E-05	1.65E-05	2.45E-05	4.37E-05
C32E8.10h	338	258	536	791	2.25E-05	1.62E-05	2.32E-05	4.23E-05
C32E8.11	1457	1480	1914	2746	2.63E-05	2.52E-05	2.25E-05	3.98E-05
C32E8.2a.1	6909	6564	9784	8773	1.13E-03	1.02E-03	1.04E-03	1.15E-03
C32E8.2a.2	6143	5698	8770	8332	1.00E-03	8.79E-04	9.32E-04	1.09E-03
C32E8.3	117	173	218	164	2.06E-05	2.87E-05	2.49E-05	2.32E-05
C32E8.6a	9	8	23	13	2.80E-06	2.65E-06	1.82E-06	2.25E-06
C32E8.6b	6	4	19	10	2.80E-06	2.65E-06	1.82E-06	2.25E-06
C32E8.7	25	39	27	28	2.80E-06	3.20E-06	1.82E-06	2.25E-06
C32E8.8	1322	1311	2869	3296	5.11E-05	4.78E-05	7.21E-05	1.02E-04
C32E8.9	53	66	52	81	7.25E-06	8.54E-06	4.63E-06	8.91E-06
C32F10.1b	416	558	640	796	3.23E-05	4.09E-05	3.23E-05	4.96E-05
C32F10.2	742	760	949	1666	2.58E-05	2.50E-05	2.15E-05	4.66E-05
C32F10.4.1	130	177	141	179	1.46E-05	1.88E-05	1.03E-05	1.62E-05
C32F10.4.2	124	164	127	173	1.45E-05	1.81E-05	9.66E-06	1.62E-05
C32F10.5	926	968	1078	1578	4.81E-05	4.75E-05	3.64E-05	6.59E-05
C32F10.6	100	77	110	152	6.13E-06	4.47E-06	4.39E-06	7.51E-06
C32F10.8a.1	1302	1774	1875	2466	8.39E-05	1.08E-04	7.86E-05	1.28E-04
C32F10.8a.2	1190	1643	1774	2368	8.79E-05	1.15E-04	8.53E-05	1.41E-04
C32F10.8b.1	1252	1725	1786	2340	7.61E-05	9.91E-05	7.07E-05	1.14E-04
C32F10.8b.2	1207	1661	1710	2304	1.27E-04	1.65E-04	1.17E-04	1.94E-04
C32F10.8b.3	869	1130	1213	1656	8.66E-05	1.06E-04	7.87E-05	1.33E-04
C32F10.8b.4	859	1117	1190	1646	8.57E-05	1.05E-04	7.72E-05	1.32E-04
C32F10.8b.5	823	1076	1134	1620	8.74E-05	1.08E-04	7.84E-05	1.38E-04
C32H11.1	9	19	16	8	2.80E-06	2.65E-06	1.82E-06	2.25E-06
C32H11.10	101	15	23	9	1.04E-05	2.65E-06	1.82E-06	2.25E-06
C32H11.11	19	31	10	4	3.61E-06	5.58E-06	1.82E-06	2.25E-06
C32H11.12	415	138	365	104	4.23E-05	1.33E-05	2.42E-05	8.50E-06
C32H11.13	1	4	8	4	2.80E-06	2.65E-06	1.82E-06	2.25E-06
C32H11.2	4	7	28	1	2.80E-06	2.65E-06	2.04E-06	2.25E-06
C32H11.3	10	5	3	3	2.80E-06	2.65E-06	1.82E-06	2.25E-06
C32H11.4	108	48	202	23	1.11E-05	4.66E-06	1.35E-05	2.25E-06
C32H11.5	36	35	27	7	5.57E-06	5.11E-06	2.71E-06	2.25E-06
C32H11.6	6	15	6	2	2.80E-06	2.72E-06	1.82E-06	2.25E-06
C32H11.7a	24	23	46	29	5.74E-06	5.18E-06	7.14E-06	5.56E-06
C32H11.8	19	31	10	4	3.61E-06	5.58E-06	1.82E-06	2.25E-06
C32H11.9	100	12	24	10	1.01E-05	2.65E-06	1.82E-06	2.25E-06
C33A11.1.1	179	271	155	149	8.12E-06	1.16E-05	4.57E-06	5.42E-06
C33A11.1.2	173	260	150	141	8.20E-06	1.17E-05	4.63E-06	5.38E-06
C33A11.2	33	56	40	54	3.47E-06	5.58E-06	2.75E-06	4.59E-06
C33A11.4	49	78	52	69	2.80E-06	3.97E-06	1.82E-06	2.99E-06
C33A12.1	303	409	826	344	6.28E-05	8.01E-05	1.11E-04	5.73E-05
C33A12.10	9	11	8	4	2.80E-06	2.65E-06	1.82E-06	2.25E-06
C33A12.11	12	12	9	10	2.80E-06	2.65E-06	1.82E-06	2.25E-06
C33A12.12	17	20	18	27	2.80E-06	2.65E-06	1.82E-06	2.25E-06
C33A12.13	6	13	10	7	2.80E-06	2.65E-06	1.82E-06	2.25E-06
C33A12.14	4	5	4	4	2.80E-06	2.65E-06	1.82E-06	2.25E-06

C33A12.15	3	13	2	4	2.80E-06	2.65E-06	1.82E-06	2.25E-06
C33A12.16	7	6	4	6	2.80E-06	2.65E-06	1.82E-06	2.25E-06
C33A12.17	1	2	0	1	2.80E-06	2.65E-06	1.82E-06	2.25E-06
C33A12.18	8	4	11	5	2.80E-06	2.65E-06	1.82E-06	2.25E-06
C33A12.19	56	258	86	262	7.90E-06	3.43E-05	7.89E-06	2.96E-05
C33A12.2	19	33	22	15	5.12E-06	8.38E-06	3.86E-06	3.24E-06
C33A12.4	30	55	39	22	8.18E-06	1.42E-05	6.92E-06	4.81E-06
C33A12.6	48	103	37	37	3.44E-06	6.98E-06	1.82E-06	2.25E-06
C33A12.7	41	61	52	48	5.99E-06	8.41E-06	4.94E-06	5.62E-06
C33A12.8	10	7	3	7	2.80E-06	2.65E-06	1.82E-06	2.25E-06
C33A12.9	9	19	31	16	2.80E-06	2.65E-06	1.82E-06	2.25E-06
C33B4.2	7	63	6	17	2.80E-06	7.04E-06	1.82E-06	2.25E-06
C33B4.3a	98	153	71	106	2.94E-06	4.34E-06	1.82E-06	2.54E-06
C33B4.3b	94	148	68	100	2.97E-06	4.42E-06	1.82E-06	2.54E-06
C33B4.4	46	69	85	75	1.07E-05	1.52E-05	1.28E-05	1.40E-05
C33B4.5	2	6	4	2	2.80E-06	2.65E-06	1.82E-06	2.25E-06
C33C12.1	3	2	6	6	2.80E-06	2.65E-06	1.82E-06	2.25E-06
C33C12.10	61	47	125	68	8.26E-06	6.00E-06	1.10E-05	7.40E-06
C33C12.11	1	2	1	2	2.80E-06	2.65E-06	1.82E-06	2.25E-06
C33C12.3a	82	99	110	119	5.35E-06	6.11E-06	4.66E-06	6.23E-06
C33C12.3b	60	85	92	109	4.76E-06	6.37E-06	4.74E-06	6.95E-06
C33C12.4	11	28	5	7	2.80E-06	5.90E-06	1.82E-06	2.25E-06
C33C12.5	5	3	15	13	2.80E-06	2.65E-06	1.82E-06	2.25E-06
C33C12.6	8	8	6	5	2.80E-06	2.65E-06	1.82E-06	2.25E-06
C33C12.7	8	9	4	4	2.80E-06	2.65E-06	1.82E-06	2.25E-06
C33C12.8	11	8	11	10	2.80E-06	2.65E-06	1.82E-06	2.25E-06
C33C12.9	47	36	54	60	7.48E-06	5.40E-06	5.58E-06	7.65E-06
C33D12.1	8	11	10	12	2.80E-06	2.65E-06	1.82E-06	2.25E-06
C33D12.2	50	55	31	85	3.72E-06	3.86E-06	1.82E-06	5.08E-06
C33D12.3	34	71	31	67	2.80E-06	4.81E-06	1.82E-06	3.87E-06
C33D12.6	11	22	17	21	2.80E-06	2.65E-06	1.82E-06	2.25E-06
C33D12.7	6	7	11	7	2.80E-06	2.65E-06	1.82E-06	2.25E-06
C33D3.1	67	93	68	64	4.84E-06	6.37E-06	3.21E-06	3.73E-06
C33D3.2	13	17	10	7	2.80E-06	2.65E-06	1.82E-06	2.25E-06
C33D3.3	22	16	24	10	2.80E-06	2.65E-06	1.86E-06	2.25E-06
C33D3.4	11	6	23	10	2.80E-06	2.65E-06	1.82E-06	2.25E-06
C33D3.5	7	12	6	5	2.80E-06	2.65E-06	1.82E-06	2.25E-06
C33D9.10	5	4	4	3	2.80E-06	2.65E-06	1.82E-06	2.25E-06
C33D9.1a	62	106	63	110	2.80E-06	3.78E-06	1.82E-06	3.33E-06
C33D9.1b	63	105	64	111	2.80E-06	3.81E-06	1.82E-06	3.42E-06
C33D9.2	4	3	4	2	2.80E-06	2.65E-06	1.82E-06	2.25E-06
C33D9.3a	112	161	64	122	7.50E-06	1.02E-05	2.79E-06	6.57E-06
C33D9.3b	70	98	45	83	7.34E-06	9.71E-06	3.08E-06	6.99E-06
C33D9.4	5	8	11	1	2.80E-06	2.65E-06	1.82E-06	2.25E-06
C33D9.5	28	40	35	41	2.80E-06	2.65E-06	1.82E-06	2.25E-06
C33D9.6	39	46	48	60	2.80E-06	2.78E-06	1.99E-06	3.06E-06
C33D9.8	40	45	41	55	2.80E-06	2.65E-06	1.82E-06	2.72E-06
C33D9.9	14	15	38	27	2.80E-06	2.65E-06	2.84E-06	2.50E-06
C33E10.1	5	12	10	6	2.80E-06	2.65E-06	1.82E-06	2.25E-06
C33E10.10	2	12	6	7	2.80E-06	3.12E-06	1.82E-06	2.25E-06
C33E10.2	14	22	16	37	2.80E-06	2.65E-06	1.82E-06	3.62E-06
C33E10.3	7	16	7	7	2.80E-06	2.65E-06	1.82E-06	2.25E-06
C33E10.4	35	43	33	47	2.80E-06	2.65E-06	1.82E-06	2.25E-06
C33E10.5	6	4	20	9	2.80E-06	2.65E-06	1.82E-06	2.25E-06
C33E10.6	21	29	52	30	2.80E-06	2.65E-06	1.82E-06	2.25E-06
C33E10.7	9	13	13	9	2.80E-06	2.65E-06	1.82E-06	2.25E-06
C33E10.8	6	9	6	7	2.80E-06	2.65E-06	1.82E-06	2.25E-06
C33E10.9	3	2	1	2	2.80E-06	2.65E-06	1.82E-06	2.25E-06
C33F10.1	105	265	77	35	2.11E-05	5.03E-05	1.01E-05	5.65E-06

C33F10.11	53	161	27	32	9.80E-06	2.81E-05	3.26E-06	4.77E-06
C33F10.12	53	133	41	36	5.04E-06	1.19E-05	2.53E-06	2.74E-06
C33F10.13	5	9	8	11	2.80E-06	2.65E-06	1.82E-06	2.25E-06
C33F10.2	134	162	230	298	5.77E-06	6.59E-06	6.45E-06	1.03E-05
C33F10.4a	196	191	253	361	1.55E-05	1.43E-05	1.31E-05	2.30E-05
C33F10.4b	97	106	121	168	1.11E-05	1.15E-05	9.04E-06	1.55E-05
C33F10.4c	89	80	108	184	1.52E-05	1.29E-05	1.20E-05	2.52E-05
C33F10.5a	114	214	78	173	4.20E-06	7.46E-06	1.88E-06	5.13E-06
C33F10.5b	120	218	83	184	3.64E-06	6.27E-06	1.82E-06	4.50E-06
C33F10.5c	98	181	59	146	4.51E-06	7.83E-06	1.82E-06	5.38E-06
C33F10.5d	122	219	82	185	3.75E-06	6.35E-06	1.82E-06	4.57E-06
C33F10.7a	29	66	35	25	2.80E-06	5.29E-06	1.93E-06	2.25E-06
C33F10.7b.1	20	56	26	18	2.80E-06	5.55E-06	1.82E-06	2.25E-06
C33F10.7b.2	19	53	22	18	2.80E-06	5.53E-06	1.82E-06	2.25E-06
C33F10.8	23	35	22	13	2.80E-06	2.65E-06	1.82E-06	2.25E-06
C33F10.9	824	1253	1196	316	2.16E-04	3.10E-04	2.04E-04	6.66E-05
C33G3.1a	52	66	32	64	2.80E-06	2.94E-06	1.82E-06	2.41E-06
C33G3.1b.1	78	106	59	92	2.80E-06	3.47E-06	1.82E-06	2.56E-06
C33G3.1b.2	79	111	60	92	2.80E-06	3.54E-06	1.82E-06	2.50E-06
C33G3.3	100	217	28	54	9.10E-06	1.87E-05	1.82E-06	3.96E-06
C33G3.4	61	83	48	63	2.80E-06	3.12E-06	1.82E-06	2.25E-06
C33G3.5	7	6	5	4	2.80E-06	2.65E-06	1.82E-06	2.25E-06
C33G3.6.1	64	82	61	87	3.30E-06	4.02E-06	2.06E-06	3.62E-06
C33G3.6.2	58	74	54	75	3.47E-06	4.18E-06	2.10E-06	3.60E-06
C33G3.t2	1	0	3	0	2.80E-06	2.65E-06	2.99E-06	2.25E-06
C33G8.1	5	9	8	7	2.80E-06	2.65E-06	1.82E-06	2.25E-06
C33G8.10	5	12	20	8	2.80E-06	2.65E-06	1.82E-06	2.25E-06
C33G8.12	12	10	7	12	2.80E-06	2.65E-06	1.82E-06	2.25E-06
C33G8.13	3	4	3	4	2.80E-06	2.65E-06	1.82E-06	2.25E-06
C33G8.2	185	264	85	133	1.77E-05	2.38E-05	5.28E-06	1.02E-05
C33G8.3	44	31	21	15	1.15E-05	7.64E-06	3.57E-06	3.15E-06
C33G8.4	30	45	82	20	7.31E-06	1.03E-05	1.30E-05	3.91E-06
C33G8.5	12	19	17	14	2.80E-06	2.65E-06	1.82E-06	2.25E-06
C33G8.6	27	58	17	25	2.80E-06	5.50E-06	1.82E-06	2.25E-06
C33G8.7	22	24	16	25	2.80E-06	2.65E-06	1.82E-06	2.25E-06
C33G8.8	10	14	18	10	2.80E-06	2.65E-06	1.82E-06	2.25E-06
C33G8.9	7	14	6	14	2.80E-06	2.65E-06	1.82E-06	2.25E-06
C33H5.1	6	5	7	4	2.80E-06	2.65E-06	1.82E-06	2.25E-06
C33H5.10	194	330	268	391	1.91E-05	3.07E-05	1.72E-05	3.09E-05
C33H5.11.1	299	423	362	604	1.91E-05	2.56E-05	1.51E-05	3.10E-05
C33H5.11.2	266	371	333	554	1.90E-05	2.50E-05	1.55E-05	3.17E-05
C33H5.12a.1	553	600	627	510	7.77E-05	7.96E-05	5.73E-05	5.76E-05
C33H5.12a.2	457	448	494	410	7.77E-05	7.19E-05	5.46E-05	5.60E-05
C33H5.12b.1	172	152	285	173	3.46E-05	2.89E-05	3.74E-05	2.80E-05
C33H5.12b.2	508	552	490	460	5.10E-05	5.23E-05	3.20E-05	3.71E-05
C33H5.12c	186	176	383	188	5.25E-05	4.69E-05	7.03E-05	4.26E-05
C33H5.13	49	49	132	49	1.97E-05	1.86E-05	3.45E-05	1.58E-05
C33H5.14.1	236	298	260	339	1.51E-05	1.80E-05	1.08E-05	1.74E-05
C33H5.14.2	224	288	234	330	1.63E-05	1.98E-05	1.11E-05	1.93E-05
C33H5.15	361	292	381	515	3.95E-05	3.02E-05	2.71E-05	4.52E-05
C33H5.16	13	27	8	9	2.80E-06	3.31E-06	1.82E-06	2.25E-06
C33H5.17	111	167	62	106	9.46E-06	1.35E-05	3.44E-06	7.26E-06
C33H5.18a	347	406	501	666	2.84E-05	3.14E-05	2.67E-05	4.38E-05
C33H5.18b	426	452	594	755	2.63E-05	2.64E-05	2.39E-05	3.74E-05
C33H5.19.1	230	299	265	270	2.88E-05	3.54E-05	2.16E-05	2.72E-05
C33H5.19.2	169	231	181	192	2.70E-05	3.49E-05	1.88E-05	2.46E-05
C33H5.2	9	9	16	9	2.80E-06	2.65E-06	1.82E-06	2.25E-06
C33H5.4a	660	565	1028	1101	2.75E-05	2.23E-05	2.79E-05	3.69E-05
C33H5.4b	400	348	647	661	2.64E-05	2.17E-05	2.78E-05	3.51E-05

C33H5.6	141	168	178	272	2.11E-05	2.38E-05	1.74E-05	3.27E-05
C33H5.7	482	480	596	692	5.03E-05	4.73E-05	4.05E-05	5.80E-05
C33H5.8	203	207	209	301	2.54E-05	2.45E-05	1.70E-05	3.03E-05
C33H5.9	281	352	508	594	1.38E-05	1.64E-05	1.63E-05	2.35E-05
C34B2.1	1	4	2	1	2.80E-06	2.65E-06	1.82E-06	2.25E-06
C34B2.10.1	271	373	586	302	5.82E-05	7.56E-05	8.18E-05	5.20E-05
C34B2.10.2	230	310	396	237	5.45E-05	6.94E-05	6.10E-05	4.51E-05
C34B2.11	57	74	44	55	7.36E-06	9.02E-06	3.70E-06	5.69E-06
C34B2.2	308	371	372	366	6.27E-05	7.14E-05	4.93E-05	5.99E-05
C34B2.3	28	50	13	15	2.97E-06	5.00E-06	1.82E-06	2.25E-06
C34B2.4	2	7	7	3	2.80E-06	2.65E-06	1.82E-06	2.25E-06
C34B2.5	166	208	228	279	2.56E-05	3.03E-05	2.29E-05	3.46E-05
C34B2.6	818	865	873	1339	3.00E-05	3.00E-05	2.09E-05	3.95E-05
C34B2.7.1	686	741	793	1140	3.74E-05	3.81E-05	2.81E-05	4.99E-05
C34B2.7.2	652	700	739	1101	3.79E-05	3.84E-05	2.80E-05	5.14E-05
C34B2.8	706	872	695	733	1.11E-04	1.30E-04	7.11E-05	9.26E-05
C34B2.9	21	26	10	16	3.11E-06	3.62E-06	1.82E-06	2.25E-06
C34B4.1a	33	79	52	81	2.80E-06	2.72E-06	1.82E-06	2.38E-06
C34B4.1b	37	90	56	88	2.80E-06	2.91E-06	1.82E-06	2.43E-06
C34B4.2a.1	443	423	494	752	2.60E-05	2.35E-05	1.89E-05	3.55E-05
C34B4.2a.2	271	259	247	414	2.83E-05	2.55E-05	1.68E-05	3.47E-05
C34B4.3	9	22	8	4	2.80E-06	2.65E-06	1.82E-06	2.25E-06
C34B4.4	8	19	24	28	2.80E-06	2.65E-06	1.82E-06	2.25E-06
C34B4.5	3	14	13	7	2.80E-06	2.65E-06	1.82E-06	2.25E-06
C34B7.1	18	34	19	14	2.80E-06	2.65E-06	1.82E-06	2.25E-06
C34B7.2	456	560	556	1001	1.78E-05	2.07E-05	1.42E-05	3.15E-05
C34B7.3	59	141	64	201	3.84E-06	8.65E-06	2.71E-06	1.05E-05
C34B7.4	143	140	313	391	8.65E-06	7.99E-06	1.23E-05	1.90E-05
C34C12.1	3	12	8	7	2.80E-06	2.65E-06	1.82E-06	2.25E-06
C34C12.2	334	295	409	610	1.70E-05	1.42E-05	1.36E-05	2.50E-05
C34C12.3	353	245	861	713	2.35E-05	1.54E-05	3.73E-05	3.82E-05
C34C12.4.1	181	185	183	137	3.40E-05	3.28E-05	2.23E-05	2.06E-05
C34C12.4.2	147	162	157	123	2.97E-05	3.09E-05	2.07E-05	2.00E-05
C34C12.5.1	220	337	230	219	2.59E-05	3.75E-05	1.76E-05	2.07E-05
C34C12.5.2	206	327	215	202	2.60E-05	3.90E-05	1.76E-05	2.05E-05
C34C12.6	32	74	26	57	2.80E-06	6.11E-06	1.82E-06	4.00E-06
C34C12.7	28	42	22	32	5.10E-06	7.22E-06	2.61E-06	4.68E-06
C34C12.8	388	384	412	520	4.91E-05	4.59E-05	3.39E-05	5.29E-05
C34C12.9	8	7	8	7	2.80E-06	2.65E-06	1.82E-06	2.25E-06
C34C6.1	8	5	5	4	2.80E-06	2.65E-06	1.82E-06	2.25E-06
C34C6.2	198	190	189	443	8.99E-06	8.15E-06	5.59E-06	1.62E-05
C34C6.3	41	60	20	15	2.94E-06	4.07E-06	1.82E-06	2.25E-06
C34C6.4	227	366	340	466	1.38E-05	2.11E-05	1.35E-05	2.28E-05
C34C6.5a	133	138	97	143	1.05E-05	1.03E-05	4.97E-06	9.04E-06
C34C6.5b.1	156	166	110	167	1.05E-05	1.05E-05	4.81E-06	9.00E-06
C34C6.5b.2	132	134	96	142	1.13E-05	1.08E-05	5.36E-06	9.76E-06
C34C6.5b.3	132	137	96	142	1.09E-05	1.07E-05	5.17E-06	9.45E-06
C34C6.6a	73	178	62	86	5.40E-06	1.25E-05	2.99E-06	5.13E-06
C34C6.6b	63	165	58	80	4.70E-06	1.16E-05	2.81E-06	4.79E-06
C34C6.7	24	57	14	37	2.80E-06	4.36E-06	1.82E-06	2.41E-06
C34C6.8	0	3	2	0	2.80E-06	2.65E-06	1.82E-06	2.25E-06
C34D1.1	1	1	0	0	2.80E-06	2.65E-06	1.82E-06	2.25E-06
C34D1.2	2	3	2	3	2.80E-06	2.65E-06	1.82E-06	2.25E-06
C34D1.3	20	17	12	3	2.80E-06	2.65E-06	1.82E-06	2.25E-06
C34D1.4	17	33	23	24	2.80E-06	4.55E-06	2.19E-06	2.81E-06
C34D1.5	22	30	24	11	3.78E-06	4.87E-06	2.68E-06	2.25E-06
C34D10.1	28	83	22	45	4.03E-06	1.13E-05	2.06E-06	5.20E-06
C34D10.2	210	347	248	434	6.36E-06	9.92E-06	4.88E-06	1.05E-05
C34D4.1	32	24	13	13	2.80E-06	2.65E-06	1.82E-06	2.25E-06

C34D4.10	52	62	44	54	5.35E-06	6.03E-06	2.95E-06	4.45E-06
C34D4.11	25	28	24	8	6.80E-06	7.22E-06	4.26E-06	2.25E-06
C34D4.12	174	205	301	172	3.24E-05	3.60E-05	3.64E-05	2.57E-05
C34D4.13	135	136	196	161	3.82E-05	3.63E-05	3.61E-05	3.66E-05
C34D4.14	3765	3034	3650	5548	4.79E-05	3.65E-05	3.02E-05	5.67E-05
C34D4.15	109	175	52	54	1.15E-05	1.74E-05	3.55E-06	4.57E-06
C34D4.16	7	3	10	1	2.80E-06	2.65E-06	1.82E-06	2.25E-06
C34D4.2	31	53	23	6	3.22E-06	5.21E-06	1.82E-06	2.25E-06
C34D4.3	58	87	73	35	1.49E-05	2.12E-05	1.22E-05	7.24E-06
C34D4.4a	377	407	406	481	5.68E-05	5.79E-05	3.98E-05	5.82E-05
C34D4.4b	329	346	366	401	4.82E-05	4.78E-05	3.49E-05	4.72E-05
C34D4.6	4	0	19	2	2.80E-06	2.65E-06	1.82E-06	2.25E-06
C34D4.7	10	7	17	8	2.80E-06	2.65E-06	1.82E-06	2.25E-06
C34D4.8	5	5	11	4	2.80E-06	2.65E-06	1.82E-06	2.25E-06
C34D4.9	33	49	12	21	2.80E-06	3.44E-06	1.82E-06	2.25E-06
C34E10.1.1	484	587	457	648	3.56E-05	4.08E-05	2.19E-05	3.83E-05
C34E10.1.2	454	561	449	634	3.34E-05	3.90E-05	2.15E-05	3.75E-05
C34E10.10	138	159	135	154	1.99E-05	2.16E-05	1.26E-05	1.78E-05
C34E10.11	411	487	357	380	4.25E-05	4.76E-05	2.40E-05	3.16E-05
C34E10.2	264	348	290	283	2.48E-05	3.08E-05	1.77E-05	2.13E-05
C34E10.3	236	344	290	520	8.93E-06	1.23E-05	7.14E-06	1.58E-05
C34E10.4a	203	308	163	288	1.09E-05	1.57E-05	5.72E-06	1.25E-05
C34E10.4b	198	313	168	305	1.15E-05	1.71E-05	6.32E-06	1.42E-05
C34E10.5.1	742	701	690	1075	3.33E-05	2.98E-05	2.02E-05	3.88E-05
C34E10.5.2	702	664	668	1029	3.34E-05	2.99E-05	2.07E-05	3.94E-05
C34E10.5.3	702	651	688	1033	3.53E-05	3.09E-05	2.25E-05	4.17E-05
C34E10.6.1	16075	14581	14751	20028	9.27E-04	7.94E-04	5.53E-04	9.27E-04
C34E10.6.2	14572	13578	13438	18387	8.81E-04	7.76E-04	5.29E-04	8.93E-04
C34E10.6.3	15700	14181	14240	19602	1.08E-03	9.22E-04	6.38E-04	1.08E-03
C34E10.7	19	10	19	14	3.67E-06	2.65E-06	2.39E-06	2.25E-06
C34E10.8	667	653	748	1238	2.29E-05	2.12E-05	1.67E-05	3.41E-05
C34E10.9	6	6	5	3	2.80E-06	2.65E-06	1.82E-06	2.25E-06
C34E11.1.1	270	356	273	396	1.63E-05	2.03E-05	1.08E-05	1.92E-05
C34E11.1.2	215	281	211	337	1.63E-05	2.02E-05	1.04E-05	2.06E-05
C34E11.2	37	49	22	22	2.80E-06	2.65E-06	1.82E-06	2.25E-06
C34E11.3	116	163	67	129	2.80E-06	3.47E-06	1.82E-06	2.34E-06
C34E11.4	6	12	15	10	2.80E-06	3.23E-06	2.79E-06	2.29E-06
C34E7.1.1	69	101	41	73	3.78E-06	5.24E-06	1.82E-06	3.22E-06
C34E7.1.2	60	81	36	61	4.20E-06	5.37E-06	1.82E-06	3.44E-06
C34E7.3	6	14	6	5	2.80E-06	2.65E-06	1.82E-06	2.25E-06
C34E7.4	281	401	129	192	2.94E-05	3.96E-05	8.78E-06	1.61E-05
C34F11.1	10	14	5	7	2.80E-06	2.65E-06	1.82E-06	2.25E-06
C34F11.2	24	39	45	13	6.83E-06	1.05E-05	8.35E-06	2.97E-06
C34F11.3a.1	699	973	634	850	3.31E-05	4.35E-05	1.95E-05	3.23E-05
C34F11.3a.2	699	973	634	850	3.32E-05	4.36E-05	1.96E-05	3.24E-05
C34F11.3b	672	926	606	822	2.97E-05	3.87E-05	1.74E-05	2.92E-05
C34F11.3c	667	929	607	835	3.10E-05	4.08E-05	1.83E-05	3.11E-05
C34F11.4	586	742	869	205	1.67E-04	1.99E-04	1.61E-04	4.68E-05
C34F11.5	96	149	38	36	4.00E-06	5.87E-06	1.82E-06	2.25E-06
C34F11.6	490	655	638	158	1.24E-04	1.56E-04	1.05E-04	3.21E-05
C34F11.8	12	54	19	30	2.80E-06	6.14E-06	1.82E-06	2.90E-06
C34F11.9a	166	217	142	305	7.70E-06	9.50E-06	4.28E-06	1.14E-05
C34F11.9b	110	153	107	212	7.08E-06	9.31E-06	4.48E-06	1.10E-05
C34F11.9c	115	143	94	171	6.58E-06	7.75E-06	3.50E-06	7.87E-06
C34F6.1	95	142	45	35	3.08E-06	4.34E-06	1.82E-06	2.25E-06
C34F6.10	192	298	177	326	5.82E-06	8.54E-06	3.50E-06	7.94E-06
C34F6.11	21	34	11	25	3.64E-06	5.55E-06	1.82E-06	3.46E-06
C34F6.2	6753	8326	9066	14245	8.34E-04	9.71E-04	7.29E-04	1.41E-03
C34F6.3	3149	4151	5066	7911	3.94E-04	4.90E-04	4.12E-04	7.94E-04

C34F6.4	55	82	68	80	4.79E-06	6.74E-06	3.86E-06	5.60E-06
C34F6.5	13	17	6	10	4.14E-06	5.13E-06	1.82E-06	2.56E-06
C34F6.6	14	20	11	9	3.70E-06	5.00E-06	1.90E-06	2.25E-06
C34F6.7	130	131	205	167	7.28E-06	6.93E-06	7.47E-06	7.51E-06
C34F6.8.1	208	383	239	305	1.40E-05	2.43E-05	1.04E-05	1.64E-05
C34F6.8.2	223	401	259	309	1.44E-05	2.45E-05	1.09E-05	1.61E-05
C34F6.8.3	197	359	220	293	1.68E-05	2.89E-05	1.22E-05	2.01E-05
C34F6.9	95	119	112	160	2.80E-06	3.25E-06	2.10E-06	3.71E-06
C34G6.1	1394	1149	1739	2445	2.94E-05	2.29E-05	2.39E-05	4.14E-05
C34G6.2	306	573	394	659	1.46E-05	2.58E-05	1.22E-05	2.52E-05
C34G6.3	32	61	18	7	9.80E-06	1.76E-05	3.59E-06	2.25E-06
C34G6.4	340	454	336	485	9.30E-06	1.17E-05	5.98E-06	1.06E-05
C34G6.5	318	392	371	560	2.55E-05	2.97E-05	1.94E-05	3.61E-05
C34G6.6b	1067	1324	825	844	3.65E-05	4.28E-05	1.84E-05	2.32E-05
C34G6.7b.1	783	803	990	1263	5.25E-05	5.09E-05	4.32E-05	6.80E-05
C34G6.7b.2	705	748	878	1141	6.52E-05	6.53E-05	5.28E-05	8.47E-05
C34H3.1	97	98	207	178	5.57E-06	5.32E-06	7.73E-06	8.21E-06
C34H3.2	18	29	30	27	2.80E-06	4.02E-06	2.86E-06	3.17E-06
C34H4.1	61	35	71	27	5.60E-06	3.04E-06	4.25E-06	2.25E-06
C34H4.2	134	107	193	134	1.01E-05	7.64E-06	9.49E-06	8.14E-06
C34H4.3	29	37	38	35	2.80E-06	3.02E-06	2.13E-06	2.43E-06
C34H4.4a	886	793	599	280	1.21E-04	1.02E-04	5.33E-05	3.08E-05
C34H4.4b	956	877	675	338	1.14E-04	9.88E-05	5.24E-05	3.24E-05
C34H4.5	18	12	18	26	4.79E-06	3.02E-06	3.12E-06	5.58E-06
C35A11.1	15	31	14	24	2.80E-06	2.65E-06	1.82E-06	2.25E-06
C35A11.2	16	22	9	3	2.80E-06	2.65E-06	1.82E-06	2.25E-06
C35A11.3	8	8	6	5	2.80E-06	2.65E-06	1.82E-06	2.25E-06
C35A11.4	109	96	99	105	7.20E-06	5.98E-06	4.25E-06	5.56E-06
C35A5.1	9	2	3	7	2.80E-06	2.65E-06	1.82E-06	2.25E-06
C35A5.10	18	27	3	6	2.80E-06	3.31E-06	1.82E-06	2.25E-06
C35A5.11	16	21	11	9	3.75E-06	4.66E-06	1.82E-06	2.25E-06
C35A5.2	27	28	33	25	2.80E-06	2.65E-06	1.82E-06	2.25E-06
C35A5.3	114	158	144	248	9.10E-06	1.19E-05	7.49E-06	1.59E-05
C35A5.4	23	28	26	11	2.80E-06	2.65E-06	1.82E-06	2.25E-06
C35A5.5	20	27	17	16	2.80E-06	2.65E-06	1.82E-06	2.25E-06
C35A5.6a	33	53	48	47	4.51E-06	6.82E-06	4.26E-06	5.15E-06
C35A5.6b	11	34	24	23	2.80E-06	7.94E-06	3.86E-06	4.57E-06
C35A5.7	9	18	12	8	2.80E-06	2.65E-06	1.82E-06	2.25E-06
C35A5.9	1	9	7	6	2.80E-06	2.65E-06	1.82E-06	2.25E-06
C35B1.2	115	210	200	248	7.56E-06	1.31E-05	8.56E-06	1.31E-05
C35B1.3	5	3	1	1	2.86E-06	2.65E-06	1.82E-06	2.25E-06
C35B1.4	349	294	1200	494	1.01E-04	8.02E-05	2.25E-04	1.15E-04
C35B1.5.1	368	537	492	429	7.70E-05	1.06E-04	6.70E-05	7.21E-05
C35B1.5.2	324	458	411	406	7.16E-05	9.56E-05	5.91E-05	7.20E-05
C35B1.7	10	11	8	10	2.80E-06	2.65E-06	1.82E-06	2.25E-06
C35B1.8	0	1	0	0	2.80E-06	2.65E-06	1.82E-06	2.25E-06
C35B8.1	702	941	237	194	8.87E-05	1.12E-04	1.95E-05	1.97E-05
C35B8.2a.1	178	220	162	305	5.52E-06	6.45E-06	3.26E-06	7.60E-06
C35B8.2a.2	134	168	137	249	5.12E-06	6.06E-06	3.41E-06	7.62E-06
C35B8.2b	135	167	137	249	5.01E-06	5.85E-06	3.30E-06	7.40E-06
C35B8.3a.1	56	102	53	73	3.64E-06	6.24E-06	2.24E-06	3.80E-06
C35B8.3a.2	50	97	49	71	3.95E-06	7.25E-06	2.51E-06	4.50E-06
C35B8.3b	55	102	56	76	3.67E-06	6.40E-06	2.42E-06	4.07E-06
C35B8.4	22	19	10	13	8.12E-06	6.64E-06	2.41E-06	3.87E-06
C35C5.11	11	6	11	8	2.80E-06	2.65E-06	1.82E-06	2.25E-06
C35C5.2	8	8	14	6	2.80E-06	2.65E-06	1.82E-06	2.25E-06
C35C5.3a	193	333	280	275	2.81E-05	4.59E-05	2.66E-05	3.22E-05
C35C5.3b	242	444	349	359	2.53E-05	4.39E-05	2.37E-05	3.02E-05
C35C5.4	70	85	79	90	1.33E-05	1.53E-05	9.78E-06	1.38E-05

C35C5.5	20	42	18	30	2.80E-06	2.65E-06	1.82E-06	2.25E-06
C35C5.6	154	164	230	329	4.26E-06	4.29E-06	4.14E-06	7.31E-06
C35C5.8a	21	64	21	33	2.80E-06	4.89E-06	1.82E-06	2.25E-06
C35C5.8b	3	12	8	3	2.80E-06	2.65E-06	1.82E-06	2.25E-06
C35C5.9	43	68	84	51	2.17E-05	3.24E-05	2.76E-05	2.07E-05
C35D10.1	306	427	381	303	4.02E-05	5.30E-05	3.26E-05	3.20E-05
C35D10.10	353	299	323	396	4.09E-05	3.27E-05	2.43E-05	3.68E-05
C35D10.11	252	383	502	107	7.18E-05	1.03E-04	9.31E-05	2.45E-05
C35D10.12	4	6	14	5	2.80E-06	2.65E-06	1.82E-06	2.25E-06
C35D10.13	357	345	416	365	9.32E-05	8.51E-05	7.07E-05	7.65E-05
C35D10.14	100	191	149	198	8.82E-06	1.59E-05	8.56E-06	1.40E-05
C35D10.15	8	12	10	11	2.80E-06	2.65E-06	1.82E-06	2.25E-06
C35D10.16	514	493	389	575	1.13E-04	1.02E-04	5.56E-05	1.01E-04
C35D10.17	75	92	231	79	2.33E-05	2.70E-05	4.68E-05	1.97E-05
C35D10.2	133	372	97	117	1.28E-05	3.38E-05	6.07E-06	9.02E-06
C35D10.3	15	15	16	10	2.80E-06	2.65E-06	1.82E-06	2.25E-06
C35D10.4	578	603	674	924	2.74E-05	2.70E-05	2.08E-05	3.51E-05
C35D10.5	122	136	110	214	2.39E-05	2.51E-05	1.40E-05	3.36E-05
C35D10.6	281	378	474	461	3.62E-05	4.60E-05	3.98E-05	4.77E-05
C35D10.7a	272	272	485	714	1.78E-05	1.68E-05	2.06E-05	3.74E-05
C35D10.7b.1	253	246	440	674	1.79E-05	1.64E-05	2.02E-05	3.82E-05
C35D10.7b.2	258	257	461	682	1.78E-05	1.67E-05	2.07E-05	3.78E-05
C35D10.8	27	30	12	16	5.94E-06	6.22E-06	1.82E-06	2.81E-06
C35D10.9a	265	290	357	515	1.60E-05	1.65E-05	1.40E-05	2.49E-05
C35D10.9b	236	257	313	483	1.54E-05	1.58E-05	1.33E-05	2.53E-05
C35D6.1	1	4	2	0	2.80E-06	2.65E-06	1.82E-06	2.25E-06
C35D6.10	2	3	16	4	2.80E-06	2.65E-06	1.82E-06	2.25E-06
C35D6.2	1	4	2	0	2.80E-06	2.65E-06	1.82E-06	2.25E-06
C35D6.3	1	0	0	0	2.80E-06	2.65E-06	1.82E-06	2.25E-06
C35D6.4	9	4	10	5	2.80E-06	2.65E-06	1.82E-06	2.25E-06
C35D6.5	3	6	4	2	2.80E-06	2.65E-06	1.82E-06	2.25E-06
C35D6.6	0	0	0	1	2.80E-06	2.65E-06	1.82E-06	2.25E-06
C35D6.7	1	1	0	0	2.80E-06	2.65E-06	1.82E-06	2.25E-06
C35D6.8	1	5	9	2	2.80E-06	2.65E-06	1.82E-06	2.25E-06
C35D6.9a	12	13	8	6	2.80E-06	2.65E-06	1.82E-06	2.25E-06
C35D6.9b	1	3	2	0	2.80E-06	2.65E-06	1.82E-06	2.25E-06
C35E7.1	65	92	73	107	3.16E-06	4.23E-06	2.31E-06	4.18E-06
C35E7.10a	45	90	12	10	3.89E-06	7.35E-06	1.82E-06	2.25E-06
C35E7.10b	39	59	10	8	5.01E-06	7.14E-06	1.82E-06	2.25E-06
C35E7.11	15	20	12	6	2.80E-06	2.99E-06	1.82E-06	2.25E-06
C35E7.2a	35	31	35	35	2.80E-06	2.65E-06	1.82E-06	2.25E-06
C35E7.2b	30	19	25	17	2.80E-06	2.65E-06	1.82E-06	2.25E-06
C35E7.3	11	9	8	8	2.80E-06	2.65E-06	1.82E-06	2.25E-06
C35E7.4	27	50	39	39	2.80E-06	2.65E-06	1.82E-06	2.25E-06
C35E7.5a	224	198	145	322	6.24E-06	5.21E-06	2.62E-06	7.20E-06
C35E7.5b	161	137	87	249	6.30E-06	5.05E-06	2.20E-06	7.83E-06
C35E7.6	32	38	43	68	2.97E-06	3.33E-06	2.61E-06	5.08E-06
C35E7.7	2	6	2	1	2.80E-06	2.65E-06	1.82E-06	2.25E-06
C35E7.8	72	111	114	137	7.81E-06	1.14E-05	8.05E-06	1.19E-05
C35E7.9	76	117	38	37	8.12E-06	1.18E-05	2.64E-06	3.17E-06
C36A4.1	129	110	106	141	9.58E-06	7.72E-06	5.12E-06	8.41E-06
C36A4.10	1	2	0	0	2.80E-06	2.65E-06	1.82E-06	2.25E-06
C36A4.11	8	13	11	11	4.34E-06	6.64E-06	3.88E-06	4.79E-06
C36A4.2	147	145	118	158	1.09E-05	1.02E-05	5.70E-06	9.42E-06
C36A4.3	98	122	60	87	7.28E-06	8.54E-06	2.90E-06	5.20E-06
C36A4.4.1	1010	1003	1334	1854	6.69E-05	6.28E-05	5.75E-05	9.86E-05
C36A4.4.2	870	849	1063	1551	6.68E-05	6.16E-05	5.31E-05	9.56E-05
C36A4.5	1660	1295	2607	3010	6.49E-05	4.78E-05	6.63E-05	9.45E-05
C36A4.6	31	52	43	43	2.80E-06	3.65E-06	2.08E-06	2.56E-06

C36A4.8	259	223	320	463	1.67E-05	1.36E-05	1.34E-05	2.40E-05
C36A4.9a.1	2199	2285	3706	3661	1.06E-04	1.04E-04	1.16E-04	1.41E-04
C36A4.9a.2	1856	1910	3023	3062	1.01E-04	9.82E-05	1.07E-04	1.34E-04
C36A4.9a.3	1897	1954	3132	3092	1.01E-04	9.80E-05	1.08E-04	1.32E-04
C36A4.9b	1847	1904	3022	3058	1.00E-04	9.75E-05	1.07E-04	1.33E-04
C36B1.10	56	107	24	15	5.46E-06	9.87E-06	1.82E-06	2.25E-06
C36B1.11	672	667	743	1060	6.19E-05	5.80E-05	4.45E-05	7.84E-05
C36B1.12a.1	1394	1201	1783	2503	7.06E-05	5.75E-05	5.88E-05	1.02E-04
C36B1.12a.2	1343	1144	1738	2415	6.61E-05	5.32E-05	5.56E-05	9.54E-05
C36B1.12b	1398	1206	1789	2515	7.10E-05	5.78E-05	5.91E-05	1.03E-04
C36B1.12c.1	1388	1198	1779	2495	7.13E-05	5.82E-05	5.95E-05	1.03E-04
C36B1.12c.2	1306	1115	1706	2375	6.36E-05	5.13E-05	5.40E-05	9.29E-05
C36B1.13	1	12	16	6	2.80E-06	2.65E-06	2.13E-06	2.25E-06
C36B1.14	89	135	240	124	2.18E-05	3.12E-05	3.82E-05	2.44E-05
C36B1.1a	334	431	391	555	1.09E-05	1.34E-05	8.35E-06	1.46E-05
C36B1.1b	324	420	385	550	1.55E-05	1.90E-05	1.20E-05	2.12E-05
C36B1.1c	412	520	483	699	2.05E-05	2.44E-05	1.56E-05	2.79E-05
C36B1.1d	334	429	388	549	1.06E-05	1.29E-05	8.02E-06	1.40E-05
C36B1.3	523	540	774	752	6.90E-05	6.73E-05	6.65E-05	7.97E-05
C36B1.4.1	764	900	991	903	9.58E-05	1.07E-04	8.09E-05	9.10E-05
C36B1.5.1	240	274	311	426	1.69E-05	1.83E-05	1.43E-05	2.41E-05
C36B1.5.2	223	248	291	409	1.66E-05	1.75E-05	1.41E-05	2.45E-05
C36B1.6	24	35	17	24	5.10E-06	7.01E-06	2.35E-06	4.09E-06
C36B1.7	338	300	197	348	6.61E-05	5.54E-05	2.51E-05	5.46E-05
C36B1.8a	1225	899	1231	2093	4.36E-05	3.02E-05	2.85E-05	5.98E-05
C36B1.8b	1408	1106	1441	2470	4.21E-05	3.13E-05	2.81E-05	5.94E-05
C36B1.9	143	152	185	264	7.90E-06	7.94E-06	6.65E-06	1.17E-05
C36B7.1	3	6	24	4	2.80E-06	2.65E-06	1.82E-06	2.25E-06
C36B7.2	9	2	2	6	2.80E-06	2.65E-06	1.82E-06	2.25E-06
C36B7.3	2	2	1	1	2.80E-06	2.65E-06	1.82E-06	2.25E-06
C36B7.4	1	5	0	1	2.80E-06	2.65E-06	1.82E-06	2.25E-06
C36B7.5a	31	45	23	18	2.80E-06	2.65E-06	1.82E-06	2.25E-06
C36B7.5b	34	48	24	20	2.80E-06	2.65E-06	1.82E-06	2.25E-06
C36B7.6.1	220	342	243	337	1.17E-05	1.72E-05	8.40E-06	1.44E-05
C36B7.6.2	189	298	222	308	1.17E-05	1.74E-05	8.95E-06	1.53E-05
C36B7.7	5	14	8	12	2.80E-06	2.65E-06	1.82E-06	2.25E-06
C36B7.8	8	2	2	2	3.28E-06	2.65E-06	1.82E-06	2.25E-06
C36C5.1	2	2	2	4	2.80E-06	2.65E-06	1.82E-06	2.25E-06
C36C5.10	4	4	7	3	2.80E-06	2.65E-06	1.82E-06	2.25E-06
C36C5.11	4	6	1	3	2.80E-06	2.65E-06	1.82E-06	2.25E-06
C36C5.12	7	34	6	7	2.80E-06	5.45E-06	1.82E-06	2.25E-06
C36C5.14	6	16	5	7	2.80E-06	2.80E-06	1.82E-06	2.25E-06
C36C5.15	5	9	2	5	2.80E-06	2.65E-06	1.82E-06	2.25E-06
C36C5.2	1	8	9	3	2.80E-06	2.65E-06	1.82E-06	2.25E-06
C36C5.3	1	2	14	4	2.80E-06	2.65E-06	1.82E-06	2.25E-06
C36C5.4	2	6	0	2	2.80E-06	2.65E-06	1.82E-06	2.25E-06
C36C5.5	46	118	51	24	8.37E-06	2.03E-05	6.05E-06	3.51E-06
C36C5.6	5	8	1	4	2.80E-06	2.65E-06	1.82E-06	2.25E-06
C36C5.7	1	5	4	4	2.80E-06	2.65E-06	1.82E-06	2.25E-06
C36C5.8	4	7	6	8	2.80E-06	2.65E-06	1.82E-06	2.25E-06
C36C5.9	1	3	3	2	2.80E-06	2.65E-06	1.82E-06	2.25E-06
C36C9.1	222	210	383	388	9.30E-06	8.31E-06	1.04E-05	1.30E-05
C36C9.2	5	9	21	7	2.80E-06	2.65E-06	1.82E-06	2.25E-06
C36C9.3	72	55	61	106	6.55E-06	4.71E-06	3.61E-06	7.74E-06
C36C9.4	10	7	8	6	2.80E-06	2.65E-06	1.82E-06	2.25E-06
C36C9.5	8	11	9	6	2.80E-06	2.65E-06	1.82E-06	2.25E-06
C36C9.6	4	2	3	2	2.80E-06	2.65E-06	1.82E-06	2.25E-06
C36C9.t1	0	0	0	1	2.80E-06	2.65E-06	1.82E-06	2.25E-06
C36C9.t3	0	0	0	1	2.80E-06	2.65E-06	1.82E-06	2.25E-06

C36E6.1a	63	90	95	99	8.01E-06	1.08E-05	7.85E-06	1.01E-05
C36E6.1b	158	246	280	347	7.92E-06	1.16E-05	9.13E-06	1.40E-05
C36E6.2	115	135	99	135	1.13E-05	1.25E-05	6.32E-06	1.06E-05
C36E6.3	2481	2651	5291	3682	2.33E-04	2.35E-04	3.24E-04	2.78E-04
C36E6.5.1	1972	2672	5598	3604	3.42E-04	4.38E-04	6.32E-04	5.02E-04
C36E6.5.2	1823	2422	4923	3463	3.27E-04	4.10E-04	5.74E-04	4.98E-04
C36E6.8	24	50	22	6	6.83E-06	1.35E-05	4.08E-06	2.25E-06
C36E8.1.1	1078	1403	970	1518	4.79E-05	5.89E-05	2.80E-05	5.42E-05
C36E8.1.2	943	1204	876	1327	4.96E-05	5.98E-05	3.00E-05	5.60E-05
C36E8.2	31	31	21	23	3.53E-06	3.33E-06	1.82E-06	2.25E-06
C36E8.3	69	106	69	107	4.12E-06	5.98E-06	2.68E-06	5.13E-06
C36E8.4	37	40	31	46	2.80E-06	2.65E-06	1.82E-06	2.47E-06
C36E8.5.1	7136	7236	8592	9815	4.81E-04	4.61E-04	3.77E-04	5.32E-04
C36E8.5.2	6673	6847	8139	9575	5.18E-04	5.02E-04	4.11E-04	5.97E-04
C36F7.1	70	66	69	77	6.80E-06	6.08E-06	4.37E-06	6.03E-06
C36F7.2	89	109	128	113	1.02E-05	1.18E-05	9.57E-06	1.04E-05
C36F7.4a.1	5	14	12	8	2.80E-06	2.65E-06	1.82E-06	2.25E-06
C36F7.4a.2	3	11	13	7	2.80E-06	2.65E-06	1.82E-06	2.25E-06
C36F7.4b	10	21	17	15	2.80E-06	2.65E-06	1.82E-06	2.25E-06
C36F7.5	37	65	16	14	4.28E-06	7.09E-06	1.82E-06	2.25E-06
C36F7.t2	0	0	1	0	2.80E-06	2.65E-06	1.82E-06	2.25E-06
C36F7.t3	0	0	1	0	2.80E-06	2.65E-06	1.82E-06	2.25E-06
C36H8.1	60	109	52	46	5.35E-06	9.18E-06	3.01E-06	3.28E-06
C36H8.2	37	38	33	30	3.02E-06	2.94E-06	1.82E-06	2.25E-06
C37A2.2	1455	931	2173	3319	4.17E-05	2.52E-05	4.05E-05	7.64E-05
C37A2.3	27	62	31	19	2.80E-06	4.84E-06	1.82E-06	2.25E-06
C37A2.4a	760	588	1291	1661	5.29E-05	3.87E-05	5.85E-05	9.29E-05
C37A2.4b	1090	746	1617	1792	5.97E-05	3.86E-05	5.76E-05	7.88E-05
C37A2.5a	271	244	291	365	1.04E-05	8.86E-06	7.29E-06	1.13E-05
C37A2.5b	340	320	378	461	1.29E-05	1.14E-05	9.31E-06	1.40E-05
C37A2.6	16	16	21	15	2.80E-06	2.65E-06	2.08E-06	2.25E-06
C37A2.7.1	4059	3950	16987	3097	1.06E-03	9.72E-04	2.88E-03	6.48E-04
C37A2.7.2	3238	3119	13555	2701	9.67E-04	8.80E-04	2.63E-03	6.48E-04
C37A2.8a	287	303	137	283	3.03E-05	3.02E-05	9.40E-06	2.40E-05
C37A2.8b	275	300	144	268	2.95E-05	3.04E-05	1.01E-05	2.31E-05
C37A5.1.1	14	33	13	19	2.80E-06	2.65E-06	1.82E-06	2.25E-06
C37A5.1.2	13	30	13	19	2.80E-06	2.65E-06	1.82E-06	2.25E-06
C37A5.2	2	69	3	8	2.80E-06	1.16E-05	1.82E-06	2.25E-06
C37A5.3	2	16	1	5	2.80E-06	7.06E-06	1.82E-06	2.25E-06
C37A5.4	3	43	3	8	2.80E-06	6.74E-06	1.82E-06	2.25E-06
C37A5.5	0	13	0	3	2.80E-06	5.11E-06	1.82E-06	2.25E-06
C37A5.6	1	11	0	2	2.80E-06	4.42E-06	1.82E-06	2.25E-06
C37A5.7.1	33	54	77	100	2.80E-06	3.73E-06	3.66E-06	5.87E-06
C37A5.7.2	28	36	52	80	2.80E-06	3.15E-06	3.13E-06	5.94E-06
C37A5.8	0	17	0	3	2.80E-06	6.67E-06	1.82E-06	2.25E-06
C37A5.t1	3	2	3	2	4.09E-06	2.65E-06	2.66E-06	2.25E-06
C37C3.1	134	171	148	253	9.77E-06	1.18E-05	7.03E-06	1.48E-05
C37C3.10	2	3	0	2	2.80E-06	2.65E-06	1.82E-06	2.25E-06
C37C3.11	2	3	4	1	2.80E-06	2.65E-06	1.82E-06	2.25E-06
C37C3.12	10	29	13	9	2.80E-06	5.08E-06	1.82E-06	2.25E-06
C37C3.13.1	49	116	35	46	9.02E-06	2.02E-05	4.19E-06	6.79E-06
C37C3.13.2	35	91	17	35	7.34E-06	1.80E-05	2.31E-06	5.89E-06
C37C3.2a.1	1255	1560	1248	1651	7.56E-05	8.88E-05	4.89E-05	7.99E-05
C37C3.2a.2	1244	1552	1240	1635	8.61E-05	1.01E-04	5.58E-05	9.09E-05
C37C3.2b.1	1053	1332	1062	1411	6.69E-05	8.00E-05	4.39E-05	7.20E-05
C37C3.2b.2	1101	1344	1102	1445	6.96E-05	8.03E-05	4.53E-05	7.34E-05
C37C3.2b.3	1179	1467	1197	1512	7.46E-05	8.77E-05	4.93E-05	7.68E-05
C37C3.2b.4	1116	1378	1124	1471	8.29E-05	9.68E-05	5.44E-05	8.78E-05
C37C3.2c.1	1093	1349	1086	1447	7.40E-05	8.63E-05	4.78E-05	7.87E-05

C37C3.2c.2	1047	1289	1051	1385	7.57E-05	8.80E-05	4.94E-05	8.04E-05
C37C3.3	10	10	9	9	2.80E-06	2.65E-06	1.82E-06	2.25E-06
C37C3.4	3	6	3	1	2.80E-06	2.65E-06	1.82E-06	2.25E-06
C37C3.6a.1	4238	4468	2815	6144	9.51E-05	9.47E-05	4.11E-05	1.11E-04
C37C3.6a.2	3997	4248	2677	5764	9.43E-05	9.47E-05	4.11E-05	1.09E-04
C37C3.6b.1	4028	4317	2701	5828	6.78E-05	6.86E-05	2.96E-05	7.88E-05
C37C3.6b.2	4056	4329	2723	5824	6.91E-05	6.97E-05	3.02E-05	7.97E-05
C37C3.6c.1	4042	4282	2702	5912	9.43E-05	9.44E-05	4.10E-05	1.11E-04
C37C3.6c.2	3799	4055	2562	5514	9.39E-05	9.47E-05	4.12E-05	1.09E-04
C37C3.7	24	21	25	24	2.80E-06	2.65E-06	1.82E-06	2.25E-06
C37C3.8a.1	94	141	79	112	7.34E-06	1.04E-05	4.01E-06	7.02E-06
C37C3.8a.2	71	131	66	101	6.24E-06	1.09E-05	3.77E-06	7.13E-06
C37C3.8a.3	76	120	67	100	6.55E-06	9.76E-06	3.75E-06	6.90E-06
C37C3.8b	76	120	67	100	6.55E-06	9.76E-06	3.75E-06	6.90E-06
C37C3.9	705	593	849	783	6.42E-05	5.10E-05	5.03E-05	5.73E-05
C37E2.1.1	527	678	443	720	3.30E-05	4.02E-05	1.81E-05	3.63E-05
C37E2.1.2	511	654	424	710	3.35E-05	4.05E-05	1.81E-05	3.74E-05
C37E2.1.3	328	445	265	439	3.22E-05	4.13E-05	1.69E-05	3.46E-05
C37E2.2a	10	16	6	3	2.80E-06	2.65E-06	1.82E-06	2.25E-06
C37E2.3	1	3	3	3	2.80E-06	2.65E-06	1.82E-06	2.25E-06
C37E2.4	5	3	9	7	2.80E-06	2.65E-06	1.82E-06	2.25E-06
C37E2.5	49	75	21	47	4.48E-06	6.48E-06	1.82E-06	3.46E-06
C37H5.1	31	39	32	38	2.80E-06	2.91E-06	1.82E-06	2.43E-06
C37H5.10	1	1	2	1	2.80E-06	2.65E-06	1.82E-06	2.25E-06
C37H5.11	2	1	0	0	2.80E-06	2.65E-06	1.82E-06	2.25E-06
C37H5.13a	102	169	110	175	8.62E-06	1.35E-05	6.05E-06	1.19E-05
C37H5.13b	45	61	53	71	6.08E-06	7.80E-06	4.66E-06	7.71E-06
C37H5.13c	15	17	18	21	5.60E-06	6.00E-06	4.37E-06	6.30E-06
C37H5.2	17	49	32	34	2.80E-06	4.76E-06	2.15E-06	2.81E-06
C37H5.3a	46	119	40	65	3.81E-06	9.31E-06	2.15E-06	4.32E-06
C37H5.3b	39	101	33	55	4.03E-06	9.89E-06	2.22E-06	4.59E-06
C37H5.4	2	2	0	2	2.80E-06	2.65E-06	1.82E-06	2.25E-06
C37H5.6a	843	968	1036	1598	6.87E-05	7.45E-05	5.50E-05	1.05E-04
C37H5.6b.1	1124	1335	1538	2324	7.41E-05	8.31E-05	6.60E-05	1.23E-04
C37H5.6b.2	1097	1290	1464	2278	6.59E-05	7.32E-05	5.72E-05	1.10E-04
C37H5.8	3721	3631	3177	4948	1.68E-04	1.54E-04	9.31E-05	1.79E-04
C37H5.9a	37	69	35	54	2.80E-06	4.71E-06	1.82E-06	3.13E-06
C37H5.9b	41	77	37	60	2.80E-06	4.97E-06	1.82E-06	3.28E-06
C37H5.9c.1	46	83	43	61	2.97E-06	5.08E-06	1.82E-06	3.17E-06
C37H5.9c.2	46	81	44	60	2.94E-06	4.92E-06	1.84E-06	3.10E-06
C37H5.9c.3	45	80	43	59	2.97E-06	4.97E-06	1.84E-06	3.10E-06
C38C10.1	11	14	8	11	2.80E-06	2.65E-06	1.82E-06	2.25E-06
C38C10.2.1	899	1014	1351	1551	5.63E-05	6.00E-05	5.51E-05	7.80E-05
C38C10.2.2	883	997	1301	1522	5.55E-05	5.92E-05	5.32E-05	7.68E-05
C38C10.3	30	42	18	6	3.70E-06	4.89E-06	1.82E-06	2.25E-06
C38C10.4	195	279	229	318	1.35E-05	1.83E-05	1.03E-05	1.77E-05
C38C10.5a	704	748	1076	1580	1.85E-05	1.86E-05	1.84E-05	3.34E-05
C38C10.5b	680	705	1049	1554	1.85E-05	1.81E-05	1.85E-05	3.39E-05
C38C10.6	3	2	5	2	2.80E-06	2.65E-06	1.82E-06	2.25E-06
C38C3.1	2	3	8	4	2.80E-06	2.65E-06	1.82E-06	2.25E-06
C38C3.2	2	5	6	1	2.80E-06	2.65E-06	1.82E-06	2.25E-06
C38C3.3	17	36	16	12	2.80E-06	3.41E-06	1.82E-06	2.25E-06
C38C3.4a	3	10	3	7	2.80E-06	2.65E-06	1.82E-06	2.25E-06
C38C3.4b	4	19	8	11	2.80E-06	2.65E-06	1.82E-06	2.25E-06
C38C3.5a	2930	2253	2590	3760	2.61E-04	1.89E-04	1.50E-04	2.69E-04
C38C3.5c.1	718	727	615	529	1.14E-04	1.09E-04	6.33E-05	6.72E-05
C38C3.5c.2	407	421	378	398	6.64E-05	6.48E-05	4.01E-05	5.21E-05
C38C3.6	1	1	2	2	2.80E-06	2.65E-06	1.82E-06	2.25E-06
C38C3.7	19	16	12	8	2.80E-06	2.65E-06	1.82E-06	2.25E-06

C38C3.8	5	3	3	3	2.80E-06	2.65E-06	1.82E-06	2.25E-06
C38C3.9	6	4	2	5	2.80E-06	2.65E-06	1.82E-06	2.25E-06
C38C5.1	0	1	1	1	2.80E-06	2.65E-06	1.82E-06	2.25E-06
C38C6.2	243	201	130	226	1.23E-05	9.63E-06	4.28E-06	9.20E-06
C38C6.3	17	51	8	8	2.80E-06	5.40E-06	1.82E-06	2.25E-06
C38C6.4	4	8	3	4	2.80E-06	2.65E-06	1.82E-06	2.25E-06
C38C6.5	16	17	32	8	2.80E-06	2.65E-06	1.82E-06	2.25E-06
C38C6.6.1	322	777	143	229	1.87E-05	4.26E-05	5.39E-06	1.07E-05
C38C6.t1	0	0	1	0	2.80E-06	2.65E-06	1.82E-06	2.25E-06
C38D4.10	33	43	76	26	1.64E-05	2.02E-05	2.46E-05	1.04E-05
C38D4.1a	396	278	562	544	2.98E-05	1.97E-05	2.75E-05	3.28E-05
C38D4.1b	149	107	161	133	3.40E-05	2.31E-05	2.39E-05	2.44E-05
C38D4.3	1907	1585	2568	3572	3.92E-05	3.08E-05	3.43E-05	5.89E-05
C38D4.4	557	557	840	1038	3.45E-05	3.26E-05	3.38E-05	5.16E-05
C38D4.5.1	487	529	683	957	1.87E-05	1.92E-05	1.71E-05	2.96E-05
C38D4.5.2	438	450	622	900	1.90E-05	1.84E-05	1.75E-05	3.13E-05
C38D4.6a.1	603	657	744	902	6.63E-05	6.82E-05	5.32E-05	7.96E-05
C38D4.6a.2	572	621	682	872	4.86E-05	4.99E-05	3.77E-05	5.96E-05
C38D4.6b.1	595	654	740	899	5.10E-05	5.30E-05	4.13E-05	6.19E-05
C38D4.6b.2	397	425	508	583	5.50E-05	5.57E-05	4.58E-05	6.49E-05
C38D4.7	15	22	17	19	2.80E-06	2.78E-06	1.82E-06	2.25E-06
C38D4.8	3	1	5	1	2.80E-06	2.65E-06	1.82E-06	2.25E-06
C38D9.1	4	7	9	1	2.80E-06	2.65E-06	1.82E-06	2.25E-06
C38D9.10	4	1	9	5	2.80E-06	2.65E-06	1.82E-06	2.25E-06
C38D9.2	14	26	9	7	2.80E-06	2.65E-06	1.82E-06	2.25E-06
C38D9.3	22	15	31	12	2.80E-06	2.65E-06	1.82E-06	2.25E-06
C38D9.4	6	3	6	0	2.80E-06	2.65E-06	1.82E-06	2.25E-06
C38D9.5	16	22	13	11	2.80E-06	2.65E-06	1.82E-06	2.25E-06
C38D9.6	6	4	5	7	2.80E-06	2.65E-06	1.82E-06	2.25E-06
C38D9.7	2	2	5	5	2.80E-06	2.65E-06	1.82E-06	2.25E-06
C38D9.8	7	6	5	6	2.80E-06	2.65E-06	1.82E-06	2.25E-06
C38D9.9	4	5	4	3	2.80E-06	2.65E-06	1.82E-06	2.25E-06
C38H2.1	26	41	35	31	2.80E-06	2.65E-06	1.82E-06	2.25E-06
C38H2.2	101	141	143	132	6.50E-06	8.57E-06	5.99E-06	6.84E-06
C38H2.3	11	20	8	11	3.16E-06	5.42E-06	1.82E-06	2.54E-06
C39B10.1	7	10	16	10	2.80E-06	2.65E-06	1.82E-06	2.25E-06
C39B10.3	5	4	4	4	2.80E-06	2.65E-06	1.82E-06	2.25E-06
C39B10.4	1	1	1	0	2.80E-06	2.65E-06	1.82E-06	2.25E-06
C39B10.5	1	0	2	0	2.80E-06	2.65E-06	1.82E-06	2.25E-06
C39B10.6	7	9	8	4	2.80E-06	2.86E-06	1.82E-06	2.25E-06
C39B5.1	2	12	9	2	2.80E-06	2.65E-06	1.82E-06	2.25E-06
C39B5.10	2	1	6	4	2.80E-06	2.65E-06	1.82E-06	2.25E-06
C39B5.11	4	1	2	0	2.80E-06	2.65E-06	1.82E-06	2.25E-06
C39B5.12	2	6	6	3	2.80E-06	2.65E-06	1.82E-06	2.25E-06
C39B5.2	31	30	15	34	2.80E-06	2.65E-06	1.82E-06	2.47E-06
C39B5.3	3	2	11	7	2.80E-06	2.65E-06	1.82E-06	2.25E-06
C39B5.4	11	19	7	12	2.80E-06	2.65E-06	1.82E-06	2.25E-06
C39B5.5	90	88	87	63	7.59E-06	7.01E-06	4.77E-06	4.27E-06
C39B5.6	77	94	75	139	5.21E-06	6.00E-06	3.30E-06	7.56E-06
C39B5.7	1	5	3	2	2.80E-06	2.65E-06	1.82E-06	2.25E-06
C39B5.8	1	3	5	2	2.80E-06	2.65E-06	1.82E-06	2.25E-06
C39B5.9	3	4	9	2	2.80E-06	2.65E-06	1.82E-06	2.25E-06
C39D10.1	1	0	1	0	2.80E-06	2.65E-06	1.82E-06	2.25E-06
C39D10.11	23	23	13	31	2.80E-06	2.65E-06	1.82E-06	2.25E-06
C39D10.2	19	23	23	5	3.28E-06	3.76E-06	2.59E-06	2.25E-06
C39D10.3a	43	61	29	43	4.34E-06	5.82E-06	1.91E-06	3.49E-06
C39D10.3b	24	32	14	26	4.20E-06	5.26E-06	1.82E-06	3.64E-06
C39D10.5	6	10	9	6	2.80E-06	2.65E-06	1.82E-06	2.25E-06
C39D10.6	10	10	12	8	2.80E-06	2.65E-06	1.82E-06	2.25E-06

C39D10.8a	357	300	160	239	5.39E-05	4.28E-05	1.57E-05	2.90E-05
C39D10.8b	349	293	158	239	9.44E-05	7.49E-05	2.78E-05	5.19E-05
C39D10.8c	355	295	163	239	4.21E-05	3.30E-05	1.26E-05	2.28E-05
C39D10.9	2	5	9	2	2.80E-06	2.65E-06	1.82E-06	2.25E-06
C39E6.1	79	124	98	145	4.62E-06	6.85E-06	3.74E-06	6.81E-06
C39E6.2	0	3	0	1	2.80E-06	2.65E-06	1.82E-06	2.25E-06
C39E6.4	20	16	11	14	2.80E-06	2.65E-06	1.82E-06	2.25E-06
C39E6.6	11	13	4	4	2.80E-06	2.65E-06	1.82E-06	2.25E-06
C39E9.1	0	3	2	0	2.80E-06	2.65E-06	1.82E-06	2.25E-06
C39E9.10	13	36	19	21	2.80E-06	2.65E-06	1.82E-06	2.25E-06
C39E9.11	328	433	306	514	2.72E-05	3.40E-05	1.65E-05	3.43E-05
C39E9.12	445	501	982	998	3.69E-05	3.92E-05	5.30E-05	6.65E-05
C39E9.13	915	645	934	1152	9.06E-05	6.03E-05	6.02E-05	9.16E-05
C39E9.14a.1	651	569	798	1128	4.76E-05	3.93E-05	3.80E-05	6.63E-05
C39E9.14a.2	549	511	642	969	4.61E-05	4.05E-05	3.51E-05	6.53E-05
C39E9.14b	594	533	732	1075	4.62E-05	3.92E-05	3.70E-05	6.72E-05
C39E9.2	11	25	30	18	2.80E-06	4.21E-06	3.48E-06	2.59E-06
C39E9.3	18	37	16	10	2.80E-06	4.23E-06	1.82E-06	2.25E-06
C39E9.4	26	40	11	11	4.62E-06	6.72E-06	1.82E-06	2.25E-06
C39E9.5	0	2	2	1	2.80E-06	2.65E-06	1.82E-06	2.25E-06
C39E9.6	1	4	7	1	2.80E-06	2.65E-06	1.82E-06	2.25E-06
C39E9.7	51	36	33	40	3.58E-06	2.65E-06	1.82E-06	2.25E-06
C39E9.8a	157	724	166	331	1.85E-05	8.05E-05	1.27E-05	3.13E-05
C39E9.8b	176	802	204	379	2.44E-05	1.05E-04	1.84E-05	4.22E-05
C39E9.9	9	20	12	5	2.80E-06	2.65E-06	1.82E-06	2.25E-06
C39F7.1	9	6	3	4	2.80E-06	2.65E-06	1.82E-06	2.25E-06
C39F7.4.1	1900	1974	1847	2811	1.94E-04	1.90E-04	1.23E-04	2.30E-04
C39F7.4.2	1078	1068	947	1326	1.85E-04	1.74E-04	1.06E-04	1.83E-04
C39F7.5	38	34	36	47	2.80E-06	2.65E-06	1.82E-06	2.25E-06
C39H7.1	104	171	61	60	1.19E-05	1.85E-05	4.56E-06	5.53E-06
C39H7.2	2	5	8	4	2.80E-06	2.65E-06	1.82E-06	2.25E-06
C39H7.4	90	134	113	119	7.73E-06	1.09E-05	6.32E-06	8.23E-06
C39H7.5	7	8	12	6	2.80E-06	2.65E-06	1.82E-06	2.25E-06
C39H7.6	8	8	6	7	2.80E-06	2.65E-06	1.82E-06	2.25E-06
C39H7.7	8	5	6	7	2.80E-06	2.65E-06	1.82E-06	2.25E-06
C39H7.8	4	4	10	6	2.80E-06	2.65E-06	1.82E-06	2.25E-06
C39H7.9	5	6	6	3	2.80E-06	2.65E-06	1.82E-06	2.25E-06
C40A11.1	27	29	22	22	3.14E-06	3.17E-06	1.82E-06	2.25E-06
C40A11.10	1	1	6	6	2.80E-06	2.65E-06	1.82E-06	2.25E-06
C40A11.2.1	7	5	6	3	2.80E-06	2.65E-06	1.82E-06	2.25E-06
C40A11.2.2	6	5	6	2	2.80E-06	2.65E-06	1.82E-06	2.25E-06
C40A11.3	3	4	5	4	2.80E-06	2.65E-06	1.82E-06	2.25E-06
C40A11.4	2	5	7	5	2.80E-06	2.65E-06	1.82E-06	2.25E-06
C40A11.5	6	3	5	2	2.80E-06	2.65E-06	1.82E-06	2.25E-06
C40A11.6	12	5	9	12	2.80E-06	2.65E-06	1.82E-06	2.25E-06
C40A11.7	13	5	3	10	2.80E-06	2.65E-06	1.82E-06	2.25E-06
C40A11.8	2	9	10	5	2.80E-06	2.65E-06	1.82E-06	2.25E-06
C40C9.1	9	6	5	1	2.80E-06	2.65E-06	1.82E-06	2.25E-06
C40C9.2	16	20	18	17	2.80E-06	2.65E-06	1.82E-06	2.25E-06
C40C9.3	7	17	10	10	2.80E-06	2.94E-06	1.82E-06	2.25E-06
C40C9.4	2	1	2	2	2.80E-06	2.65E-06	1.82E-06	2.25E-06
C40C9.5a	58	90	57	77	2.80E-06	3.52E-06	1.82E-06	2.56E-06
C40C9.5b	58	90	55	77	2.80E-06	3.60E-06	1.82E-06	2.61E-06
C40C9.t1	0	0	1	0	2.80E-06	2.65E-06	1.82E-06	2.25E-06
C40C9.t2	0	0	3	0	2.80E-06	2.65E-06	2.99E-06	2.25E-06
C40D2.1	9	6	6	7	2.80E-06	2.65E-06	1.82E-06	2.25E-06
C40D2.2.1	73	75	70	53	5.94E-06	5.77E-06	3.70E-06	3.46E-06
C40D2.2.2	67	60	64	45	5.66E-06	4.76E-06	3.50E-06	3.04E-06
C40D2.3	5	14	14	9	2.80E-06	2.65E-06	1.82E-06	2.25E-06

C40D2.4	2	0	2	2	2.80E-06	2.65E-06	1.82E-06	2.25E-06
C40H1.1	621	562	1007	1177	4.15E-05	3.54E-05	4.38E-05	6.31E-05
C40H1.2	7	7	8	12	2.80E-06	2.65E-06	1.82E-06	2.25E-06
C40H1.3	26	20	16	14	2.80E-06	2.65E-06	1.82E-06	2.25E-06
C40H1.4	53	122	29	32	6.72E-06	1.46E-05	2.39E-06	3.26E-06
C40H1.5	63	126	147	75	1.49E-05	2.82E-05	2.26E-05	1.43E-05
C40H1.6	221	269	464	231	4.58E-05	5.26E-05	6.25E-05	3.84E-05
C40H1.7	43	54	87	104	4.09E-06	4.84E-06	5.36E-06	7.92E-06
C40H1.9	4	6	6	4	2.80E-06	2.65E-06	1.82E-06	2.25E-06
C40H5.1	4	1	0	0	4.26E-06	2.65E-06	1.82E-06	2.25E-06
C40H5.2	7	5	7	4	2.80E-06	2.65E-06	1.82E-06	2.25E-06
C40H5.3	12	12	8	6	2.80E-06	2.65E-06	1.82E-06	2.25E-06
C40H5.4	6	23	3	7	2.80E-06	2.65E-06	1.82E-06	2.25E-06
C40H5.5	2	7	11	11	2.80E-06	2.65E-06	1.82E-06	2.25E-06
C40H5.6	77	104	112	166	1.90E-05	2.43E-05	1.80E-05	3.30E-05
C40H5.7	3	0	1	3	2.80E-06	2.65E-06	1.82E-06	2.25E-06
C40H5.8	51	46	78	50	8.15E-06	6.93E-06	8.09E-06	6.41E-06
C41A3.1	190	196	297	454	2.80E-06	2.65E-06	1.82E-06	2.25E-06
C41A3.2a	41	67	33	68	2.80E-06	2.86E-06	1.82E-06	2.47E-06
C41A3.2b	22	52	21	51	2.80E-06	3.31E-06	1.82E-06	2.77E-06
C41C4.1	26	34	46	26	2.80E-06	3.23E-06	3.01E-06	2.25E-06
C41C4.10	118	191	103	102	1.16E-05	1.78E-05	6.61E-06	8.07E-06
C41C4.2	11	13	12	3	2.80E-06	2.65E-06	1.82E-06	2.25E-06
C41C4.3	14	19	17	12	2.80E-06	2.65E-06	1.82E-06	2.25E-06
C41C4.4	580	715	739	1141	1.97E-05	2.29E-05	1.63E-05	3.11E-05
C41C4.6	407	305	738	784	3.04E-05	2.15E-05	3.59E-05	4.70E-05
C41C4.7a	572	646	685	943	4.77E-05	5.09E-05	3.72E-05	6.32E-05
C41C4.7b.1	587	647	733	952	4.79E-05	4.98E-05	3.89E-05	6.23E-05
C41C4.7b.2	509	562	621	870	5.03E-05	5.24E-05	3.99E-05	6.90E-05
C41C4.8.1	2459	2623	3007	4422	1.07E-04	1.08E-04	8.50E-05	1.54E-04
C41C4.8.2	2566	2701	3114	4512	1.09E-04	1.09E-04	8.62E-05	1.54E-04
C41C4.8.3	2420	2567	2978	4390	1.11E-04	1.11E-04	8.87E-05	1.61E-04
C41C4.9	2	4	5	2	2.80E-06	2.65E-06	1.82E-06	2.25E-06
C41D11.1	33	37	20	50	3.81E-06	4.02E-06	1.82E-06	4.63E-06
C41D11.2	364	528	643	898	2.84E-05	3.89E-05	3.26E-05	5.62E-05
C41D11.3	245	282	296	475	1.87E-05	2.03E-05	1.47E-05	2.91E-05
C41D11.4	135	125	176	253	1.52E-05	1.33E-05	1.29E-05	2.29E-05
C41D11.5	319	349	397	468	4.59E-05	4.75E-05	3.72E-05	5.41E-05
C41D11.6	9	15	20	18	2.80E-06	2.65E-06	1.82E-06	2.25E-06
C41D11.8.1	219	274	245	296	2.44E-05	2.89E-05	1.78E-05	2.65E-05
C41D11.8.2	206	256	207	280	2.47E-05	2.90E-05	1.62E-05	2.70E-05
C41D11.9	61	51	101	56	1.16E-05	9.18E-06	1.25E-05	8.57E-06
C41D7.1	6	6	11	4	2.80E-06	2.65E-06	3.12E-06	2.25E-06
C41D7.2	28	52	26	41	2.80E-06	2.65E-06	1.82E-06	2.25E-06
C41G11.1a	114	217	176	379	9.49E-06	1.71E-05	9.55E-06	2.54E-05
C41G11.1b	88	191	116	308	7.92E-06	1.63E-05	6.81E-06	2.23E-05
C41G11.3	47	44	28	35	2.80E-06	2.65E-06	1.82E-06	2.25E-06
C41G11.4a	13	23	19	20	2.80E-06	2.65E-06	1.82E-06	2.25E-06
C41G11.4b	13	24	18	20	2.80E-06	2.65E-06	1.82E-06	2.25E-06
C41G11.4c	11	24	15	19	2.80E-06	2.65E-06	1.82E-06	2.25E-06
C41G6.1	7	3	10	4	2.80E-06	2.65E-06	1.82E-06	2.25E-06
C41G6.10	8	4	6	1	2.80E-06	2.65E-06	1.82E-06	2.25E-06
C41G6.11	4	5	18	5	2.80E-06	2.65E-06	1.82E-06	2.25E-06
C41G6.12	1	1	2	0	2.80E-06	2.65E-06	1.82E-06	2.25E-06
C41G6.13	4	2	1	2	2.80E-06	2.65E-06	1.82E-06	2.25E-06
C41G6.14	7	5	6	3	2.80E-06	2.65E-06	1.82E-06	2.25E-06
C41G6.15	6	4	2	4	2.80E-06	2.65E-06	1.82E-06	2.25E-06
C41G6.16	4	8	5	7	2.80E-06	2.65E-06	1.82E-06	2.25E-06
C41G6.2	4	4	12	5	2.80E-06	2.65E-06	1.82E-06	2.25E-06

C41G6.3	9	11	16	8	2.80E-06	2.65E-06	1.82E-06	2.25E-06
C41G6.5	5	6	10	4	2.80E-06	2.65E-06	1.82E-06	2.25E-06
C41G6.6	1	6	5	3	2.80E-06	2.65E-06	1.82E-06	2.25E-06
C41G6.7	5	9	1	5	2.80E-06	2.65E-06	1.82E-06	2.25E-06
C41G6.8	5	4	4	5	2.80E-06	2.65E-06	1.82E-06	2.25E-06
C41G6.9	2	3	4	3	2.80E-06	2.65E-06	1.82E-06	2.25E-06
C41G7.1a	207	213	204	267	3.21E-05	3.12E-05	2.06E-05	3.32E-05
C41G7.1b	225	219	218	283	3.49E-05	3.21E-05	2.20E-05	3.52E-05
C41G7.3	754	948	996	1239	4.71E-05	5.59E-05	4.05E-05	6.22E-05
C41G7.4	85	80	100	110	7.78E-06	6.90E-06	5.96E-06	8.07E-06
C41G7.5a	18	20	16	21	2.80E-06	2.65E-06	1.82E-06	2.25E-06
C41G7.5b.1	18	20	16	21	2.80E-06	2.65E-06	1.82E-06	2.25E-06
C41G7.5b.2	18	20	16	21	2.80E-06	2.65E-06	1.82E-06	2.25E-06
C41G7.6	114	161	38	51	1.66E-05	2.22E-05	3.61E-06	5.98E-06
C41G7.7	6	7	5	7	2.80E-06	2.65E-06	1.82E-06	2.25E-06
C41H7.1	7	7	7	6	2.80E-06	2.65E-06	1.82E-06	2.25E-06
C41H7.2	10	9	5	8	2.80E-06	2.65E-06	1.82E-06	2.25E-06
C41H7.3	89	107	144	171	7.98E-06	9.05E-06	8.40E-06	1.23E-05
C41H7.4	20	30	35	33	2.80E-06	3.15E-06	2.53E-06	2.95E-06
C41H7.5	36	42	41	57	5.35E-06	5.90E-06	3.95E-06	6.79E-06
C41H7.6	101	119	187	204	1.00E-05	1.12E-05	1.21E-05	1.63E-05
C41H7.7	7	9	5	7	2.80E-06	2.65E-06	1.82E-06	2.25E-06
C41H7.8	8	6	1	3	2.80E-06	2.65E-06	1.82E-06	2.25E-06
C41H7.9	6	5	4	6	4.40E-06	3.47E-06	1.91E-06	3.53E-06
C42C1.1	7	11	6	5	2.80E-06	2.65E-06	1.82E-06	2.25E-06
C42C1.10.1	144	164	171	184	1.49E-05	1.61E-05	1.15E-05	1.53E-05
C42C1.10.2	157	172	173	193	1.49E-05	1.54E-05	1.07E-05	1.47E-05
C42C1.11a.1	298	399	351	465	1.58E-05	2.00E-05	1.21E-05	1.98E-05
C42C1.11a.2	343	469	498	554	2.09E-05	2.71E-05	1.98E-05	2.72E-05
C42C1.11b	78	115	124	146	1.38E-05	1.93E-05	1.43E-05	2.08E-05
C42C1.11c.1	325	448	446	532	1.72E-05	2.24E-05	1.54E-05	2.26E-05
C42C1.11c.2	294	396	347	465	1.80E-05	2.29E-05	1.38E-05	2.29E-05
C42C1.12	92	139	202	97	2.84E-05	4.05E-05	4.06E-05	2.40E-05
C42C1.14.1	6113	6033	18764	3120	1.64E-03	1.53E-03	3.28E-03	6.73E-04
C42C1.14.2	4584	4677	14631	2642	1.33E-03	1.28E-03	2.76E-03	6.16E-04
C42C1.15	119	184	110	155	1.24E-05	1.81E-05	7.45E-06	1.30E-05
C42C1.16	73	117	89	126	9.04E-06	1.37E-05	7.18E-06	1.25E-05
C42C1.2	12	13	8	9	2.80E-06	2.65E-06	1.82E-06	2.25E-06
C42C1.3	9	19	11	2	2.80E-06	3.28E-06	1.82E-06	2.25E-06
C42C1.4b	98	80	97	124	1.47E-05	1.14E-05	9.49E-06	1.50E-05
C42C1.5	407	417	371	513	3.17E-05	3.06E-05	1.88E-05	3.20E-05
C42C1.6	4	3	5	5	2.80E-06	2.65E-06	1.82E-06	2.25E-06
C42C1.7	82	115	107	132	4.03E-06	5.34E-06	3.43E-06	5.22E-06
C42C1.8	684	614	820	1241	4.45E-05	3.77E-05	3.47E-05	6.49E-05
C42C1.9	19	36	34	8	2.80E-06	4.73E-06	3.08E-06	2.25E-06
C42D4.1	582	1313	864	1114	7.69E-05	1.64E-04	7.43E-05	1.18E-04
C42D4.10	5	4	21	7	2.80E-06	2.65E-06	1.82E-06	2.25E-06
C42D4.11	1	2	1	2	2.80E-06	2.65E-06	1.82E-06	2.25E-06
C42D4.12	5	1	5	1	2.80E-06	2.65E-06	1.82E-06	2.25E-06
C42D4.13	14	33	14	8	2.80E-06	5.82E-06	1.82E-06	2.25E-06
C42D4.2	55	66	43	72	3.28E-06	3.73E-06	1.82E-06	3.44E-06
C42D4.3	216	544	98	180	2.87E-05	6.82E-05	8.45E-06	1.92E-05
C42D4.4	7	7	9	4	2.80E-06	2.65E-06	1.82E-06	2.25E-06
C42D4.5	13	6	5	3	2.80E-06	2.65E-06	1.82E-06	2.25E-06
C42D4.6	64	50	46	50	1.23E-05	9.05E-06	5.74E-06	7.69E-06
C42D4.8	994	832	1170	1797	2.55E-05	2.02E-05	1.96E-05	3.71E-05
C42D4.9	3	2	13	6	2.80E-06	2.65E-06	1.82E-06	2.25E-06
C42D8.1	44	44	60	42	6.13E-06	5.79E-06	5.43E-06	4.70E-06
C42D8.3	56	95	57	116	2.80E-06	4.05E-06	1.82E-06	4.21E-06

C42D8.4	0	2	5	1	2.80E-06	2.65E-06	1.82E-06	2.25E-06
C42D8.5a	473	595	231	259	1.95E-05	2.31E-05	6.20E-06	8.57E-06
C42D8.5b.1	263	290	119	110	2.12E-05	2.21E-05	6.25E-06	7.13E-06
C42D8.8a	1553	2076	1240	2274	6.35E-05	8.02E-05	3.30E-05	7.47E-05
C42D8.8b.1	1549	2072	1234	2244	6.33E-05	8.00E-05	3.28E-05	7.36E-05
C42D8.8b.2	1191	1569	912	1696	4.87E-05	6.06E-05	2.43E-05	5.57E-05
C42D8.9	1	1	1	5	2.80E-06	2.65E-06	1.82E-06	2.25E-06
C42D8.t1	0	0	1	0	2.80E-06	2.65E-06	1.82E-06	2.25E-06
C43C3.1	303	332	180	212	1.31E-05	1.36E-05	5.08E-06	7.38E-06
C43C3.2	19	25	18	5	2.80E-06	2.65E-06	1.82E-06	2.25E-06
C43C3.3	39	50	17	59	2.80E-06	2.86E-06	1.82E-06	2.88E-06
C43D7.1	12	13	18	15	2.80E-06	2.65E-06	1.82E-06	2.25E-06
C43D7.2	33	19	57	33	3.92E-06	2.65E-06	4.41E-06	3.15E-06
C43D7.3	2	4	18	7	2.80E-06	2.65E-06	1.82E-06	2.25E-06
C43D7.4	3	2	1	4	2.80E-06	2.65E-06	1.82E-06	2.25E-06
C43D7.5	7	6	19	6	2.80E-06	2.65E-06	3.02E-06	2.25E-06
C43D7.6	1	2	3	1	2.80E-06	2.65E-06	1.82E-06	2.25E-06
C43D7.7	3	0	1	1	2.80E-06	2.65E-06	1.82E-06	2.25E-06
C43D7.8	1	0	4	1	2.80E-06	2.65E-06	1.82E-06	2.25E-06
C43D7.9	3	6	3	1	2.80E-06	2.65E-06	1.82E-06	2.25E-06
C43E11.10	460	516	600	737	2.98E-05	3.16E-05	2.53E-05	3.84E-05
C43E11.11	457	480	612	711	2.50E-05	2.48E-05	2.18E-05	3.12E-05
C43E11.12a	200	134	158	154	3.89E-05	2.46E-05	2.00E-05	2.40E-05
C43E11.12b	111	88	105	106	2.47E-05	1.85E-05	1.52E-05	1.89E-05
C43E11.2a	169	238	279	407	1.41E-05	1.88E-05	1.52E-05	2.73E-05
C43E11.2b	55	75	110	166	1.12E-05	1.44E-05	1.45E-05	2.71E-05
C43E11.4	328	393	335	428	2.54E-05	2.87E-05	1.69E-05	2.66E-05
C43E11.5	39	108	31	23	3.95E-06	1.03E-05	2.04E-06	2.25E-06
C43E11.6b	96	138	71	153	6.38E-06	8.65E-06	3.06E-06	8.16E-06
C43E11.6c	18	24	13	23	5.43E-06	6.82E-06	2.55E-06	5.56E-06
C43E11.6d.1	138	187	104	214	6.24E-06	8.01E-06	3.06E-06	7.78E-06
C43E11.6d.2	133	179	101	202	6.16E-06	7.83E-06	3.04E-06	7.49E-06
C43E11.6e.1	97	144	74	157	6.08E-06	8.52E-06	3.02E-06	7.89E-06
C43E11.6e.2	113	157	86	173	6.05E-06	7.94E-06	2.99E-06	7.42E-06
C43E11.8	330	384	463	613	1.86E-05	2.04E-05	1.70E-05	2.77E-05
C43E11.9.1	272	289	334	209	4.55E-05	4.56E-05	3.63E-05	2.81E-05
C43E11.9.2	227	240	223	170	4.22E-05	4.22E-05	2.70E-05	2.54E-05
C43F9.10	10	18	20	13	2.80E-06	2.65E-06	1.82E-06	2.25E-06
C43F9.4	13	15	13	3	2.80E-06	2.65E-06	1.82E-06	2.25E-06
C43F9.5	4	10	5	2	2.80E-06	2.65E-06	1.82E-06	2.25E-06
C43F9.6	36	67	18	20	3.56E-06	6.24E-06	1.82E-06	2.25E-06
C43F9.7	3	1	7	1	2.80E-06	2.65E-06	2.26E-06	2.25E-06
C43F9.8	74	132	44	66	9.27E-06	1.56E-05	3.59E-06	6.63E-06
C43F9.9	50	101	43	45	2.80E-06	5.21E-06	1.82E-06	2.25E-06
C43G2.1.1	864	921	1047	1392	4.46E-05	4.49E-05	3.51E-05	5.77E-05
C43G2.1.2	626	694	798	1077	3.89E-05	4.08E-05	3.23E-05	5.38E-05
C43G2.2	177	287	178	368	7.59E-06	1.16E-05	4.97E-06	1.27E-05
C43G2.3	29	37	13	10	5.12E-06	6.19E-06	1.82E-06	2.25E-06
C43G2.4	28	36	25	14	2.80E-06	2.65E-06	1.82E-06	2.25E-06
C43G2.5	18	20	23	14	2.80E-06	2.65E-06	1.82E-06	2.25E-06
C43G2.t2	1	0	0	0	2.80E-06	2.65E-06	1.82E-06	2.25E-06
C43H6.1	80	80	43	76	6.52E-06	6.16E-06	2.28E-06	4.97E-06
C43H6.3	71	96	74	78	1.10E-05	1.40E-05	7.45E-06	9.69E-06
C43H6.4	46	75	49	81	2.80E-06	3.73E-06	1.82E-06	3.42E-06
C43H6.5	8	6	8	5	2.80E-06	2.65E-06	1.82E-06	2.25E-06
C43H6.6	1	2	2	0	2.80E-06	2.65E-06	1.82E-06	2.25E-06
C43H6.7	36	54	33	46	4.37E-06	6.22E-06	2.61E-06	4.50E-06
C43H6.8	1	0	0	2	2.80E-06	2.65E-06	1.82E-06	2.25E-06
C43H6.9	8	9	7	1	2.80E-06	2.65E-06	1.82E-06	2.25E-06

C43H8.2	243	264	358	424	2.63E-05	2.70E-05	2.52E-05	3.69E-05
C44B11.1	5	6	7	1	2.80E-06	2.65E-06	1.82E-06	2.25E-06
C44B11.4	21	34	52	9	6.19E-06	9.47E-06	9.97E-06	2.25E-06
C44B11.6	45	77	111	56	1.15E-05	1.86E-05	1.85E-05	1.15E-05
C44B12.2	2219	3192	1676	2423	2.29E-04	3.11E-04	1.12E-04	2.01E-04
C44B12.3	6	3	1	2	2.80E-06	2.65E-06	1.82E-06	2.25E-06
C44B12.4	6	3	10	5	2.80E-06	2.65E-06	1.82E-06	2.25E-06
C44B12.6	7	5	5	1	2.80E-06	2.65E-06	1.82E-06	2.25E-06
C44B12.8	6	5	6	4	2.80E-06	2.65E-06	1.82E-06	2.25E-06
C44B12.9	2	1	7	2	2.80E-06	2.65E-06	1.82E-06	2.25E-06
C44B7.1.1	179	196	183	211	2.68E-05	2.77E-05	1.78E-05	2.53E-05
C44B7.1.2	192	207	201	238	2.43E-05	2.48E-05	1.65E-05	2.42E-05
C44B7.1.3	178	191	150	205	2.51E-05	2.55E-05	1.38E-05	2.33E-05
C44B7.10.1	1684	2497	1589	2032	1.19E-04	1.67E-04	7.30E-05	1.15E-04
C44B7.10.2	1586	2393	1480	1971	1.16E-04	1.65E-04	7.02E-05	1.15E-04
C44B7.10.3	1584	2385	1478	1970	1.14E-04	1.62E-04	6.92E-05	1.14E-04
C44B7.11	177	229	225	211	7.67E-06	9.36E-06	6.34E-06	7.33E-06
C44B7.12	388	430	622	792	2.71E-05	2.84E-05	2.83E-05	4.45E-05
C44B7.2a.1	191	255	224	323	1.07E-05	1.35E-05	8.18E-06	1.46E-05
C44B7.2a.2	182	229	213	315	1.06E-05	1.26E-05	8.09E-06	1.48E-05
C44B7.2b	111	123	102	152	1.17E-05	1.22E-05	6.98E-06	1.28E-05
C44B7.3	19	40	20	15	2.80E-06	2.65E-06	1.82E-06	2.25E-06
C44B7.4	18	34	11	18	2.80E-06	3.20E-06	1.82E-06	2.25E-06
C44B7.5	425	659	380	571	6.69E-05	9.81E-05	3.90E-05	7.22E-05
C44B7.6a.1	13	29	32	21	2.80E-06	2.65E-06	1.82E-06	2.25E-06
C44B7.6a.2	12	27	31	21	2.80E-06	2.65E-06	1.82E-06	2.25E-06
C44B7.6b	13	24	26	15	2.80E-06	2.65E-06	1.82E-06	2.25E-06
C44B7.7	61	95	46	73	1.03E-05	1.52E-05	5.07E-06	9.90E-06
C44B7.8	204	340	126	210	1.09E-05	1.72E-05	4.39E-06	9.04E-06
C44B7.9	201	459	138	242	1.08E-05	2.32E-05	4.79E-06	1.04E-05
C44B9.1	28	41	105	110	2.80E-06	2.65E-06	2.68E-06	3.46E-06
C44B9.2	41	48	21	39	3.95E-06	4.36E-06	1.82E-06	3.01E-06
C44B9.3	184	152	299	349	2.39E-05	1.86E-05	2.52E-05	3.63E-05
C44B9.4	348	301	446	746	1.10E-05	8.99E-06	9.18E-06	1.89E-05
C44B9.5	209	203	289	329	1.54E-05	1.41E-05	1.38E-05	1.95E-05
C44B9.6	2	0	11	4	2.80E-06	2.65E-06	1.82E-06	2.25E-06
C44C1.1	92	175	108	155	1.13E-05	2.03E-05	8.62E-06	1.53E-05
C44C1.2	73	117	81	124	6.55E-06	9.95E-06	4.74E-06	8.95E-06
C44C1.3	7	8	4	4	2.80E-06	2.65E-06	1.82E-06	2.25E-06
C44C1.4a	75	114	66	126	4.73E-06	6.80E-06	2.71E-06	6.39E-06
C44C1.4b	21	37	22	41	3.70E-06	6.16E-06	2.53E-06	5.80E-06
C44C1.5a	176	138	188	158	1.46E-05	1.08E-05	1.02E-05	1.05E-05
C44C1.5b.1	186	151	205	170	1.74E-05	1.34E-05	1.25E-05	1.28E-05
C44C1.5b.2	173	138	182	157	2.55E-05	1.92E-05	1.75E-05	1.86E-05
C44C1.6	12	19	13	12	2.80E-06	3.73E-06	1.82E-06	2.25E-06
C44C10.1	719	735	611	312	8.92E-05	8.61E-05	4.93E-05	3.11E-05
C44C10.10	5	4	2	2	2.80E-06	2.65E-06	1.82E-06	2.25E-06
C44C10.12	1	3	0	2	2.80E-06	2.65E-06	1.82E-06	2.25E-06
C44C10.2	2	5	8	4	2.80E-06	2.65E-06	1.82E-06	2.25E-06
C44C10.3	12	27	17	19	2.80E-06	2.65E-06	1.82E-06	2.25E-06
C44C10.4	52	71	67	77	3.30E-06	4.26E-06	2.77E-06	3.94E-06
C44C10.5	6	9	6	6	2.80E-06	2.65E-06	1.82E-06	2.25E-06
C44C10.6	1	2	1	1	2.80E-06	2.65E-06	1.82E-06	2.25E-06
C44C10.7	0	4	6	9	2.80E-06	2.65E-06	1.82E-06	2.25E-06
C44C10.8	9	7	2	11	2.80E-06	2.65E-06	1.82E-06	2.25E-06
C44C11.1b.1	44	25	28	41	8.48E-06	4.55E-06	3.52E-06	6.34E-06
C44C11.1b.2	42	24	28	39	8.12E-06	4.39E-06	3.52E-06	6.05E-06
C44C3.1	10	11	10	5	2.80E-06	2.65E-06	1.82E-06	2.25E-06
C44C3.10	5	3	12	4	2.80E-06	2.65E-06	1.82E-06	2.25E-06

C44C3.11	6	6	3	10	2.80E-06	2.65E-06	1.82E-06	2.25E-06
C44C3.2	2	1	13	1	2.80E-06	2.65E-06	1.82E-06	2.25E-06
C44C3.3	3	5	8	1	2.80E-06	2.65E-06	1.82E-06	2.25E-06
C44C3.4	2	2	5	4	2.80E-06	2.65E-06	1.82E-06	2.25E-06
C44C3.5	3	6	5	4	2.80E-06	2.65E-06	1.82E-06	2.25E-06
C44C3.6	6	5	1	1	2.80E-06	2.65E-06	1.82E-06	2.25E-06
C44C3.7	7	4	5	1	2.80E-06	2.65E-06	1.82E-06	2.25E-06
C44C3.8	7	4	11	5	2.80E-06	2.65E-06	1.82E-06	2.25E-06
C44C3.9	2	3	3	4	2.80E-06	2.65E-06	1.82E-06	2.25E-06
C44C8.1	91	226	105	113	5.71E-06	1.34E-05	4.28E-06	5.69E-06
C44C8.2	92	226	104	115	4.20E-06	9.73E-06	3.08E-06	4.21E-06
C44C8.3	92	226	104	115	4.20E-06	9.73E-06	3.08E-06	4.21E-06
C44C8.4	91	226	105	113	4.14E-06	9.73E-06	3.12E-06	4.14E-06
C44C8.6a	129	208	136	203	8.90E-06	1.35E-05	6.10E-06	1.12E-05
C44E12.1	20	52	30	36	2.80E-06	4.95E-06	1.97E-06	2.90E-06
C44E12.3a	28	23	23	19	2.80E-06	2.65E-06	1.82E-06	2.25E-06
C44E12.3b	28	22	23	19	2.80E-06	2.65E-06	1.82E-06	2.25E-06
C44E12.3c	29	25	28	21	2.80E-06	2.65E-06	1.82E-06	2.25E-06
C44E12.3d	65	51	56	35	3.28E-06	2.65E-06	1.84E-06	2.25E-06
C44E12.3e	65	56	76	49	2.80E-06	2.65E-06	2.06E-06	2.25E-06
C44E12.3f	29	23	23	19	2.80E-06	2.65E-06	1.82E-06	2.25E-06
C44E4.1a	1410	1271	1473	2346	1.94E-05	1.65E-05	1.32E-05	2.59E-05
C44E4.1b.1	574	626	557	893	2.25E-05	2.31E-05	1.42E-05	2.81E-05
C44E4.1b.2	533	566	498	791	2.25E-05	2.25E-05	1.36E-05	2.68E-05
C44E4.1c	1411	1270	1469	2344	1.94E-05	1.65E-05	1.32E-05	2.59E-05
C44E4.2	65	53	113	137	6.94E-06	5.34E-06	7.84E-06	1.17E-05
C44E4.3	566	656	505	559	4.87E-05	5.33E-05	2.83E-05	3.86E-05
C44E4.5	294	303	359	418	1.48E-05	1.44E-05	1.18E-05	1.69E-05
C44E4.6.1	934	1014	5278	861	2.65E-04	2.72E-04	9.74E-04	1.96E-04
C44E4.6.2	791	865	3984	708	2.38E-04	2.46E-04	7.81E-04	1.71E-04
C44E4.6.3	773	842	3864	678	2.30E-04	2.37E-04	7.49E-04	1.62E-04
C44E4.7	255	225	403	585	8.40E-06	7.01E-06	8.66E-06	1.55E-05
C44F1.1	33	49	22	40	2.80E-06	2.91E-06	1.82E-06	2.25E-06
C44F1.2	12	18	5	4	2.80E-06	2.65E-06	1.82E-06	2.25E-06
C44F1.3	195	341	272	179	2.32E-05	3.82E-05	2.10E-05	1.71E-05
C44F1.5	94	182	78	129	2.91E-06	5.34E-06	1.82E-06	3.22E-06
C44H4.1	22	16	8	12	2.80E-06	2.65E-06	1.82E-06	2.25E-06
C44H4.2	90	142	68	70	4.40E-06	6.53E-06	2.15E-06	2.74E-06
C44H4.3	846	953	495	488	3.95E-05	4.21E-05	1.51E-05	1.83E-05
C44H4.4	130	143	102	190	6.50E-06	6.77E-06	3.32E-06	7.65E-06
C44H4.5	52	86	68	108	5.01E-06	7.83E-06	4.26E-06	8.37E-06
C44H4.6	8	6	11	10	2.80E-06	2.65E-06	1.82E-06	2.25E-06
C44H4.7a	119	139	130	215	4.48E-06	4.95E-06	3.19E-06	6.52E-06
C44H4.7b	120	143	133	220	4.62E-06	5.21E-06	3.33E-06	6.81E-06
C44H4.8	10	8	12	8	2.80E-06	2.65E-06	1.82E-06	2.25E-06
C44H9.1	16	20	12	11	2.80E-06	2.65E-06	1.82E-06	2.25E-06
C44H9.2	7	10	13	5	2.80E-06	2.65E-06	1.82E-06	2.25E-06
C44H9.4	39	63	40	57	2.80E-06	2.65E-06	1.82E-06	2.25E-06
C44H9.5	4	14	10	23	2.80E-06	2.65E-06	1.82E-06	2.97E-06
C44H9.6.1	46	72	42	57	2.80E-06	3.57E-06	1.82E-06	2.41E-06
C44H9.6.2	43	65	38	50	2.80E-06	3.70E-06	1.82E-06	2.41E-06
C44H9.7a	12	4	7	5	2.80E-06	2.65E-06	1.82E-06	2.25E-06
C44H9.7b	14	4	7	11	2.80E-06	2.65E-06	1.82E-06	2.25E-06
C44H9.8	15	24	23	17	2.80E-06	2.65E-06	1.82E-06	2.25E-06
C45B11.1a	486	513	726	930	3.34E-05	3.33E-05	3.25E-05	5.14E-05
C45B11.1b	465	479	681	879	2.72E-05	2.65E-05	2.60E-05	4.14E-05
C45B11.2	7	9	7	1	2.80E-06	2.65E-06	1.82E-06	2.25E-06
C45B11.3.1	113	194	81	130	1.34E-05	2.17E-05	6.23E-06	1.24E-05
C45B11.3.2	97	180	71	120	1.21E-05	2.13E-05	5.78E-06	1.21E-05

C45B11.4	16	19	13	7	2.80E-06	2.65E-06	1.82E-06	2.25E-06
C45B11.5	7	1	2	6	2.80E-06	2.65E-06	1.82E-06	2.25E-06
C45B2.1	441	695	1206	339	1.34E-04	1.99E-04	2.38E-04	8.27E-05
C45B2.2	130	453	242	124	3.97E-05	1.31E-04	4.81E-05	3.04E-05
C45B2.3	6	15	14	8	2.80E-06	4.89E-06	3.15E-06	2.25E-06
C45B2.4a	9	12	9	11	2.80E-06	2.65E-06	1.82E-06	2.25E-06
C45B2.4b.1	9	12	8	8	2.80E-06	2.65E-06	1.82E-06	2.25E-06
C45B2.4b.2	7	10	6	7	2.80E-06	2.65E-06	1.82E-06	2.25E-06
C45B2.5.1	86	122	90	119	7.08E-06	9.50E-06	4.83E-06	7.87E-06
C45B2.5.2	82	115	82	111	7.06E-06	9.34E-06	4.59E-06	7.65E-06
C45B2.6	85	122	90	137	3.61E-06	4.92E-06	2.50E-06	4.70E-06
C45B2.7	381	599	168	193	1.37E-05	2.03E-05	3.94E-06	5.58E-06
C45B2.8	4	12	3	2	2.80E-06	4.13E-06	1.82E-06	2.25E-06
C45E1.1a	30	58	32	41	3.00E-06	5.50E-06	2.10E-06	3.31E-06
C45E1.1b	28	55	29	41	2.80E-06	5.18E-06	1.88E-06	3.28E-06
C45E1.4	23	48	19	35	3.89E-06	7.67E-06	2.10E-06	4.75E-06
C45E5.1	61	98	43	72	5.71E-06	8.68E-06	2.62E-06	5.42E-06
C45E5.2	8	9	12	5	2.80E-06	2.65E-06	1.82E-06	2.25E-06
C45E5.3	3	4	5	2	2.80E-06	2.65E-06	1.82E-06	2.25E-06
C45E5.4	26	23	34	12	5.68E-06	4.73E-06	4.83E-06	2.25E-06
C45E5.6a.1	162	213	210	273	8.37E-06	1.04E-05	7.07E-06	1.13E-05
C45E5.6b	178	244	212	280	9.27E-06	1.20E-05	7.18E-06	1.17E-05
C45E5.6c.1	147	198	198	253	1.04E-05	1.33E-05	9.17E-06	1.44E-05
C45E5.6c.2	145	197	191	252	1.03E-05	1.33E-05	8.84E-06	1.44E-05
C45E5.6c.3	138	189	178	235	8.68E-06	1.12E-05	7.29E-06	1.19E-05
C45E5.6c.4	138	188	176	235	8.71E-06	1.12E-05	7.22E-06	1.19E-05
C45E5.6c.5	149	201	200	259	9.74E-06	1.24E-05	8.51E-06	1.36E-05
C45E5.6c.6	127	182	170	224	1.04E-05	1.41E-05	9.09E-06	1.48E-05
C45E5.6c.7	130	178	174	229	7.48E-06	9.65E-06	6.51E-06	1.06E-05
C45G3.1	588	774	877	1201	1.77E-05	2.20E-05	1.71E-05	2.90E-05
C45G3.3	72	115	115	117	9.27E-06	1.40E-05	9.64E-06	1.21E-05
C45G3.4	14	9	5	2	4.51E-06	2.72E-06	1.82E-06	2.25E-06
C45G7.1	0	1	2	0	2.80E-06	2.65E-06	1.82E-06	2.25E-06
C45G7.2	2	48	2	19	2.80E-06	1.21E-05	1.82E-06	4.07E-06
C45G7.3	3	13	4	5	2.80E-06	3.25E-06	1.82E-06	2.25E-06
C45G7.4	19	27	32	22	2.80E-06	2.65E-06	1.82E-06	2.25E-06
C45G7.5	166	284	122	88	2.80E-06	4.31E-06	1.82E-06	2.25E-06
C45G9.1	3	4	2	0	2.80E-06	2.65E-06	1.82E-06	2.25E-06
C45G9.10a	14	14	22	10	2.80E-06	2.65E-06	1.82E-06	2.25E-06
C45G9.10b	12	16	22	10	2.80E-06	2.65E-06	1.82E-06	2.25E-06
C45G9.11	8	9	27	11	2.80E-06	2.65E-06	1.84E-06	2.25E-06
C45G9.12	19	35	22	16	7.56E-06	1.31E-05	5.69E-06	5.11E-06
C45G9.13	14	45	18	19	2.80E-06	8.20E-06	2.26E-06	2.95E-06
C45G9.14	4	2	7	2	2.80E-06	2.65E-06	1.82E-06	2.25E-06
C45G9.2	132	126	199	222	1.70E-05	1.54E-05	1.67E-05	2.30E-05
C45G9.4	74	147	30	47	9.44E-06	1.77E-05	2.50E-06	4.81E-06
C45G9.5	233	294	287	310	2.19E-05	2.61E-05	1.75E-05	2.34E-05
C45G9.6a	37	94	47	35	2.80E-06	5.71E-06	1.97E-06	2.25E-06
C45G9.6b	24	105	41	26	4.45E-06	1.84E-05	4.94E-06	3.87E-06
C45G9.7	29	42	36	26	6.58E-06	9.02E-06	5.32E-06	4.75E-06
C45G9.8	21	37	22	19	2.80E-06	2.80E-06	1.82E-06	2.25E-06
C45G9.9	78	164	36	53	9.88E-06	1.96E-05	2.97E-06	5.40E-06
C45H4.1	7	6	2	2	2.80E-06	2.65E-06	1.82E-06	2.25E-06
C45H4.10	8	9	5	4	2.80E-06	2.65E-06	1.82E-06	2.25E-06
C45H4.11	9	2	6	0	2.80E-06	2.65E-06	1.82E-06	2.25E-06
C45H4.12	4	4	2	4	2.80E-06	2.65E-06	1.82E-06	2.25E-06
C45H4.13	3	6	8	3	2.80E-06	2.65E-06	1.82E-06	2.25E-06
C45H4.14a	48	47	64	82	8.04E-06	7.43E-06	6.98E-06	1.10E-05
C45H4.14b	34	32	38	62	7.22E-06	6.40E-06	5.25E-06	1.06E-05

C45H4.15	3	2	3	2	2.80E-06	2.65E-06	1.82E-06	2.25E-06
C45H4.16	5	3	5	6	2.80E-06	2.65E-06	1.82E-06	2.25E-06
C45H4.17	5	5	10	4	2.80E-06	2.65E-06	1.82E-06	2.25E-06
C45H4.18	5	2	6	5	2.80E-06	2.65E-06	1.82E-06	2.25E-06
C45H4.2	8	3	11	3	2.80E-06	2.65E-06	1.82E-06	2.25E-06
C45H4.3	5	7	10	3	2.80E-06	2.65E-06	1.82E-06	2.25E-06
C45H4.5	7	5	8	5	2.80E-06	2.65E-06	1.82E-06	2.25E-06
C45H4.6	6	8	6	5	2.80E-06	2.65E-06	1.82E-06	2.25E-06
C45H4.7	19	14	16	17	2.80E-06	2.65E-06	1.82E-06	2.25E-06
C45H4.8	12	12	7	10	2.80E-06	2.65E-06	1.82E-06	2.25E-06
C45H4.9	15	12	22	10	2.80E-06	2.65E-06	1.82E-06	2.25E-06
C45H4.t1	1	1	16	4	2.80E-06	2.65E-06	1.60E-05	4.93E-06
C46A5.1	36	51	20	15	2.80E-06	3.36E-06	1.82E-06	2.25E-06
C46A5.2	9	5	9	3	2.80E-06	2.65E-06	1.82E-06	2.25E-06
C46A5.3a	832	1120	348	347	8.69E-05	1.11E-04	2.37E-05	2.91E-05
C46A5.3b	668	903	279	307	7.63E-05	9.74E-05	2.07E-05	2.82E-05
C46A5.4	34	45	26	24	2.80E-06	2.65E-06	1.82E-06	2.25E-06
C46A5.5	128	130	163	196	1.42E-05	1.36E-05	1.18E-05	1.75E-05
C46A5.6	216	237	283	395	2.55E-05	2.64E-05	2.17E-05	3.74E-05
C46A5.8	16	22	9	10	3.05E-06	3.97E-06	1.82E-06	2.25E-06
C46A5.9	1048	1065	1651	2219	4.19E-05	4.02E-05	4.30E-05	7.13E-05
C46C11.1a	117	144	93	226	4.12E-06	4.76E-06	2.13E-06	6.36E-06
C46C11.1b	94	126	79	191	3.86E-06	4.89E-06	2.11E-06	6.30E-06
C46C11.2a	70	101	62	106	5.63E-06	7.67E-06	3.24E-06	6.86E-06
C46C11.2b	56	90	54	101	4.51E-06	6.88E-06	2.84E-06	6.54E-06
C46C11.3	23	36	25	17	2.80E-06	4.02E-06	1.91E-06	2.25E-06
C46C11.4	4	10	11	10	2.80E-06	2.65E-06	1.82E-06	2.25E-06
C46C2.1a	2165	1748	2363	3737	4.10E-05	3.13E-05	2.92E-05	5.69E-05
C46C2.1b	2159	1777	2362	3570	4.03E-05	3.14E-05	2.87E-05	5.36E-05
C46C2.2.1	338	459	351	540	2.09E-05	2.68E-05	1.41E-05	2.69E-05
C46C2.2.2	305	430	313	485	2.03E-05	2.71E-05	1.36E-05	2.59E-05
C46C2.3	5	7	4	5	2.80E-06	2.65E-06	1.82E-06	2.25E-06
C46C2.4	18	21	93	24	2.80E-06	2.65E-06	3.68E-06	2.25E-06
C46C2.5	107	245	115	191	1.78E-05	3.86E-05	1.25E-05	2.56E-05
C46C2.6a	4	10	12	6	2.80E-06	2.65E-06	1.82E-06	2.25E-06
C46C2.6b	2	3	1	1	2.80E-06	2.65E-06	1.82E-06	2.25E-06
C46E1.1	10	8	5	5	2.80E-06	2.65E-06	1.82E-06	2.25E-06
C46E1.2	10	11	14	8	2.80E-06	2.65E-06	1.82E-06	2.25E-06
C46E1.3	13	22	9	19	2.80E-06	2.65E-06	1.82E-06	2.25E-06
C46E10.1	28	55	25	15	3.33E-06	6.19E-06	1.93E-06	2.25E-06
C46E10.10	6	4	3	5	2.80E-06	2.65E-06	1.82E-06	2.25E-06
C46E10.2	4	4	0	1	2.80E-06	2.65E-06	1.82E-06	2.25E-06
C46E10.3	6	3	4	4	2.80E-06	2.65E-06	1.82E-06	2.25E-06
C46E10.4	47	72	61	70	3.67E-06	5.29E-06	3.08E-06	4.36E-06
C46E10.5	4	12	9	3	2.80E-06	2.65E-06	1.82E-06	2.25E-06
C46E10.6	2	3	1	5	2.80E-06	2.65E-06	1.82E-06	2.25E-06
C46E10.7	8	5	2	2	2.80E-06	2.65E-06	1.82E-06	2.25E-06
C46E10.8	14	18	17	9	2.80E-06	2.65E-06	1.82E-06	2.25E-06
C46E10.9	14	16	13	23	2.80E-06	2.65E-06	1.82E-06	2.25E-06
C46F11.1a	26	44	44	30	2.80E-06	2.65E-06	1.82E-06	2.25E-06
C46F11.1b	17	42	26	24	2.80E-06	2.65E-06	1.82E-06	2.25E-06
C46F11.2a	628	986	875	1103	4.51E-05	6.68E-05	4.09E-05	6.36E-05
C46F11.2b.1	598	946	839	1071	4.36E-05	6.52E-05	3.98E-05	6.28E-05
C46F11.2b.2	587	929	816	1060	4.74E-05	7.09E-05	4.29E-05	6.88E-05
C46F11.3	187	144	157	198	2.32E-05	1.69E-05	1.27E-05	1.97E-05
C46F11.5a	204	236	266	355	1.28E-05	1.40E-05	1.09E-05	1.79E-05
C46F11.5b	189	214	258	329	1.24E-05	1.33E-05	1.10E-05	1.73E-05
C46F11.6	40	60	23	64	7.78E-06	1.10E-05	2.92E-06	1.00E-05
C46F2.1	35	47	34	23	4.37E-06	5.55E-06	2.77E-06	2.32E-06

C46F4.1	16	39	22	41	2.80E-06	3.47E-06	1.82E-06	3.10E-06
C46F4.2.1	123	346	141	242	5.12E-06	1.36E-05	3.81E-06	8.07E-06
C46F4.2.2	97	269	106	203	5.01E-06	1.31E-05	3.57E-06	8.43E-06
C46F4.3	1	2	4	0	2.80E-06	2.65E-06	1.82E-06	2.25E-06
C46F9.1	18	24	10	22	3.64E-06	4.60E-06	1.82E-06	3.58E-06
C46F9.2	7	14	23	12	2.80E-06	2.65E-06	1.82E-06	2.25E-06
C46F9.3	32	60	20	34	2.80E-06	4.79E-06	1.82E-06	2.32E-06
C46F9.4	6	8	9	2	2.80E-06	2.65E-06	1.82E-06	2.25E-06
C46G7.1.1	267	367	568	173	7.31E-05	9.49E-05	1.01E-04	3.81E-05
C46G7.1.2	237	316	429	159	6.38E-05	8.04E-05	7.52E-05	3.44E-05
C46G7.2	502	818	773	773	6.40E-05	9.86E-05	6.42E-05	7.92E-05
C46G7.3	8	12	4	0	2.80E-06	2.65E-06	1.82E-06	2.25E-06
C46G7.4a	1421	1752	1185	2020	4.51E-05	5.25E-05	2.45E-05	5.15E-05
C46G7.4b	1219	1545	1050	1752	6.10E-05	7.30E-05	3.42E-05	7.04E-05
C46G7.4c	1294	1586	1039	1735	5.22E-05	6.04E-05	2.73E-05	5.62E-05
C46G7.5	6	8	10	11	2.80E-06	2.65E-06	1.82E-06	2.25E-06
C46H11.1	0	4	2	1	2.80E-06	2.65E-06	1.82E-06	2.25E-06
C46H11.10a	7	16	10	8	2.80E-06	2.65E-06	1.82E-06	2.25E-06
C46H11.10b.	9	16	18	12	2.80E-06	2.65E-06	1.82E-06	2.25E-06
C46H11.10b.	6	12	10	8	2.80E-06	2.65E-06	1.82E-06	2.25E-06
C46H11.2	32	56	30	47	2.86E-06	4.71E-06	1.82E-06	3.35E-06
C46H11.3	2	4	4	0	2.80E-06	2.65E-06	1.82E-06	2.25E-06
C46H11.4a	118	130	87	148	5.77E-06	6.00E-06	2.77E-06	5.82E-06
C46H11.4b	115	132	92	147	7.62E-06	8.25E-06	3.95E-06	7.80E-06
C46H11.4c	176	213	139	188	1.08E-05	1.23E-05	5.54E-06	9.24E-06
C46H11.6	14	20	16	5	2.80E-06	2.65E-06	1.82E-06	2.25E-06
C46H11.7	20	17	10	2	3.11E-06	2.65E-06	1.82E-06	2.25E-06
C46H11.8	18	30	8	6	2.80E-06	3.60E-06	1.82E-06	2.25E-06
C46H11.9	50	67	21	13	7.34E-06	9.28E-06	2.00E-06	2.25E-06
C46H3.1	3	1	4	2	2.80E-06	2.65E-06	1.82E-06	2.25E-06
C46H3.2a	81	96	113	153	3.86E-06	4.34E-06	3.52E-06	5.89E-06
C46H3.2b.1	55	74	73	119	3.14E-06	3.97E-06	2.70E-06	5.44E-06
C46H3.2b.2	62	78	74	120	3.50E-06	4.15E-06	2.71E-06	5.42E-06
C46H3.3	1	8	2	0	2.80E-06	2.65E-06	1.82E-06	2.25E-06
C47A10.1	137	143	110	136	3.86E-06	3.81E-06	2.02E-06	3.08E-06
C47A10.10	5	4	3	4	2.80E-06	2.65E-06	1.82E-06	2.25E-06
C47A10.11	5	1	8	1	2.80E-06	2.65E-06	1.82E-06	2.25E-06
C47A10.12	2	6	1	4	2.80E-06	2.65E-06	1.82E-06	2.25E-06
C47A10.2	7	9	1	3	2.80E-06	2.65E-06	1.82E-06	2.25E-06
C47A10.3	2	5	2	0	2.80E-06	2.65E-06	1.82E-06	2.25E-06
C47A10.4	9	8	8	6	2.80E-06	2.65E-06	1.82E-06	2.25E-06
C47A10.5	13	48	10	19	2.80E-06	5.05E-06	1.82E-06	2.25E-06
C47A10.6	6	5	5	3	2.80E-06	2.65E-06	1.82E-06	2.25E-06
C47A10.7	2	2	2	2	2.80E-06	2.65E-06	1.82E-06	2.25E-06
C47A10.8	7	4	6	7	2.80E-06	2.65E-06	1.82E-06	2.25E-06
C47A10.9	3	1	9	3	2.80E-06	2.65E-06	1.82E-06	2.25E-06
C47A10.t3	1	0	0	0	2.80E-06	2.65E-06	1.82E-06	2.25E-06
C47A10.t5	0	0	4	0	2.80E-06	2.65E-06	3.99E-06	2.25E-06
C47A4.1	105	118	71	125	2.15E-05	2.29E-05	9.48E-06	2.06E-05
C47A4.2b	73	122	57	82	5.94E-06	9.36E-06	3.01E-06	5.35E-06
C47A4.2c.1	73	122	56	81	6.16E-06	9.73E-06	3.08E-06	5.49E-06
C47A4.2c.2	80	127	61	88	6.50E-06	9.76E-06	3.23E-06	5.76E-06
C47A4.3	13	30	10	3	2.80E-06	3.33E-06	1.82E-06	2.25E-06
C47A4.4	5	17	4	3	2.80E-06	2.65E-06	1.82E-06	2.25E-06
C47A4.5	11	13	11	1	5.40E-06	6.03E-06	3.52E-06	2.25E-06
C47B2.1	82	96	54	83	6.13E-06	6.80E-06	2.62E-06	4.99E-06
C47B2.2a	263	278	264	301	1.94E-05	1.94E-05	1.27E-05	1.78E-05
C47B2.3.1	6136	6051	7883	8873	3.95E-04	3.68E-04	3.30E-04	4.59E-04
C47B2.3.2	5793	5775	7311	8548	3.32E-04	3.13E-04	2.73E-04	3.94E-04

C47B2.3.3	5635	5592	7153	8412	4.66E-04	4.37E-04	3.85E-04	5.58E-04
C47B2.4	724	778	1117	1051	8.04E-05	8.16E-05	8.07E-05	9.37E-05
C47B2.5.1	462	526	820	813	5.33E-05	5.74E-05	6.16E-05	7.54E-05
C47B2.5.2	411	454	689	727	5.31E-05	5.55E-05	5.80E-05	7.55E-05
C47B2.6a	522	407	539	548	4.45E-05	3.28E-05	2.99E-05	3.75E-05
C47B2.6b	467	357	485	496	4.88E-05	3.53E-05	3.30E-05	4.17E-05
C47B2.7a	41	57	56	91	3.28E-06	4.31E-06	2.92E-06	5.85E-06
C47B2.7b	46	62	59	101	3.42E-06	4.36E-06	2.86E-06	6.05E-06
C47B2.8	24	52	45	21	4.17E-06	8.52E-06	5.08E-06	2.92E-06
C47B2.9	25	60	70	55	6.92E-06	1.57E-05	1.26E-05	1.22E-05
C47C12.2	1	2	1	2	2.80E-06	2.65E-06	1.82E-06	2.25E-06
C47C12.3a.1	13	17	21	17	2.80E-06	2.65E-06	1.82E-06	2.25E-06
C47C12.3a.2	8	5	11	10	2.80E-06	2.65E-06	1.82E-06	2.25E-06
C47C12.3b	12	13	16	17	2.80E-06	2.65E-06	1.82E-06	2.25E-06
C47C12.4	116	104	139	232	1.09E-05	9.20E-06	8.45E-06	1.74E-05
C47D12.1a	1435	1129	1502	2602	1.29E-05	9.58E-06	8.76E-06	1.88E-05
C47D12.1c	1362	1067	1426	2522	1.25E-05	9.28E-06	8.55E-06	1.86E-05
C47D12.3	31	37	18	17	3.53E-06	3.99E-06	1.82E-06	2.25E-06
C47D12.4	4	15	3	2	2.80E-06	2.65E-06	1.82E-06	2.25E-06
C47D12.5	53	91	69	117	4.82E-06	7.80E-06	4.08E-06	8.55E-06
C47D12.6a	1844	1533	1563	2525	9.36E-05	7.35E-05	5.16E-05	1.03E-04
C47D12.6b.1	1998	1696	1744	2798	9.22E-05	7.39E-05	5.24E-05	1.04E-04
C47D12.6b.2	1996	1698	1785	2806	9.40E-05	7.55E-05	5.47E-05	1.06E-04
C47D12.6b.3	1973	1678	1747	2785	9.70E-05	7.79E-05	5.59E-05	1.10E-04
C47D12.6b.4	1803	1500	1548	2506	8.42E-05	6.62E-05	4.70E-05	9.40E-05
C47D12.7	7	10	9	16	2.80E-06	2.65E-06	1.82E-06	2.25E-06
C47D12.8	365	377	642	834	1.43E-05	1.39E-05	1.64E-05	2.62E-05
C47D2.1	5	8	4	3	2.80E-06	2.65E-06	1.82E-06	2.25E-06
C47D2.2	92	107	94	40	1.97E-05	2.16E-05	1.31E-05	6.88E-06
C47E12.1.1	1249	1499	1409	1927	8.36E-05	9.47E-05	6.14E-05	1.04E-04
C47E12.1.2	1206	1443	1370	1876	8.57E-05	9.69E-05	6.34E-05	1.07E-04
C47E12.10	15	14	9	12	2.80E-06	2.65E-06	1.82E-06	2.25E-06
C47E12.11	23	41	24	17	2.80E-06	3.94E-06	1.82E-06	2.25E-06
C47E12.12	10	12	20	5	2.88E-06	3.28E-06	3.77E-06	2.25E-06
C47E12.13	1	1	4	2	2.80E-06	2.65E-06	1.82E-06	2.25E-06
C47E12.14	1	0	6	0	2.80E-06	2.65E-06	1.82E-06	2.25E-06
C47E12.2.1	272	299	341	444	2.67E-05	2.77E-05	2.18E-05	3.50E-05
C47E12.2.2	267	295	331	421	2.74E-05	2.86E-05	2.21E-05	3.47E-05
C47E12.3	347	382	383	552	2.01E-05	2.09E-05	1.44E-05	2.57E-05
C47E12.4b	1183	1513	1837	1765	1.03E-04	1.24E-04	1.04E-04	1.23E-04
C47E12.4c.1	1257	1589	1972	1808	1.04E-04	1.25E-04	1.06E-04	1.20E-04
C47E12.4c.2	1248	1577	1960	1798	1.07E-04	1.28E-04	1.10E-04	1.24E-04
C47E12.4c.3	1245	1576	1958	1795	1.09E-04	1.30E-04	1.11E-04	1.26E-04
C47E12.4d.1	1183	1513	1837	1765	1.03E-04	1.24E-04	1.04E-04	1.23E-04
C47E12.4d.2	1245	1576	1958	1795	1.09E-04	1.30E-04	1.11E-04	1.26E-04
C47E12.4d.3	1126	1465	1772	1722	1.36E-04	1.68E-04	1.40E-04	1.67E-04
C47E12.5a.1	5250	4472	6839	7750	1.58E-04	1.27E-04	1.34E-04	1.88E-04
C47E12.5a.2	5178	4414	6714	7695	1.58E-04	1.27E-04	1.33E-04	1.88E-04
C47E12.5b.1	5178	4414	6714	7695	1.72E-04	1.39E-04	1.45E-04	2.06E-04
C47E12.5b.2	4908	4220	6419	7405	1.75E-04	1.42E-04	1.49E-04	2.12E-04
C47E12.5b.3	5132	4380	6672	7638	1.71E-04	1.38E-04	1.45E-04	2.04E-04
C47E12.5b.4	5073	4345	6553	7580	1.71E-04	1.38E-04	1.44E-04	2.05E-04
C47E12.5b.5	5143	4386	6674	7641	1.71E-04	1.38E-04	1.45E-04	2.04E-04
C47E12.6a	85	107	49	40	3.89E-06	4.63E-06	1.82E-06	2.25E-06
C47E12.6b	66	94	45	35	3.44E-06	4.66E-06	1.82E-06	2.25E-06
C47E12.7	276	307	205	260	2.34E-05	2.45E-05	1.13E-05	1.77E-05
C47E12.8	116	208	101	104	7.95E-06	1.35E-05	4.50E-06	5.71E-06
C47E12.9	10	14	8	6	2.80E-06	2.65E-06	1.82E-06	2.25E-06
C47E8.1	10	15	7	3	2.80E-06	2.83E-06	1.82E-06	2.25E-06

C47E8.10	7	8	4	5	2.80E-06	2.65E-06	1.82E-06	2.25E-06
C47E8.11	18	17	22	5	8.51E-06	7.59E-06	6.76E-06	2.25E-06
C47E8.2	2	7	11	5	2.80E-06	2.65E-06	1.82E-06	2.25E-06
C47E8.3	11	15	6	8	2.80E-06	2.65E-06	1.82E-06	2.25E-06
C47E8.4	238	286	300	329	2.40E-05	2.73E-05	1.97E-05	2.67E-05
C47E8.5.1	17244	18217	22018	26348	8.19E-04	8.18E-04	6.81E-04	1.01E-03
C47E8.5.2	17548	18552	22424	26479	7.84E-04	7.83E-04	6.52E-04	9.51E-04
C47E8.5.3	17338	18313	22407	26457	9.19E-04	9.17E-04	7.73E-04	1.13E-03
C47E8.6	9	14	15	5	2.80E-06	2.65E-06	1.82E-06	2.25E-06
C47E8.7.1	657	857	453	857	2.66E-05	3.28E-05	1.19E-05	2.79E-05
C47E8.7.2	568	743	388	719	2.91E-05	3.59E-05	1.29E-05	2.96E-05
C47E8.8	279	254	354	543	6.05E-06	5.21E-06	5.01E-06	9.47E-06
C47E8.9	5	2	0	2	2.80E-06	2.65E-06	1.82E-06	2.25E-06
C47E8.t1	0	0	2	0	2.80E-06	2.65E-06	2.00E-06	2.25E-06
C47F8.1	14	18	13	26	2.80E-06	2.65E-06	1.82E-06	2.77E-06
C47F8.2	5	3	13	14	2.80E-06	2.65E-06	1.82E-06	2.25E-06
C47F8.3	5	3	6	2	2.80E-06	2.65E-06	1.82E-06	2.25E-06
C47F8.4	2	1	4	3	2.80E-06	2.65E-06	1.82E-06	2.25E-06
C47F8.5	4	9	13	6	2.80E-06	2.65E-06	1.82E-06	2.25E-06
C47F8.6	5	4	3	0	2.80E-06	2.65E-06	1.82E-06	2.25E-06
C47F8.7	3	2	2	4	2.80E-06	2.65E-06	1.82E-06	2.25E-06
C47F8.8	3	5	1	1	2.80E-06	2.65E-06	1.82E-06	2.25E-06
C47F8.9	0	3	1	1	2.80E-06	2.65E-06	1.82E-06	2.25E-06
C47G2.1	4	7	1	0	2.80E-06	2.65E-06	1.82E-06	2.25E-06
C47G2.2	29	28	12	21	2.80E-06	2.65E-06	1.82E-06	2.25E-06
C47G2.3.1	114	116	187	121	1.70E-05	1.63E-05	1.81E-05	1.45E-05
C47G2.4	446	493	557	852	2.34E-05	2.45E-05	1.91E-05	3.60E-05
C47G2.6	3	3	5	2	2.80E-06	2.65E-06	1.82E-06	2.25E-06
C47G2.7	74	60	123	171	2.08E-05	1.59E-05	2.25E-05	3.87E-05
C47G2.8	0	0	1	0	2.80E-06	2.65E-06	1.82E-06	2.25E-06
C48A7.1a	269	395	258	352	4.84E-06	6.72E-06	3.02E-06	5.11E-06
C48A7.1b	241	350	209	295	4.76E-06	6.53E-06	2.70E-06	4.70E-06
C48A7.2	1867	1645	2267	3333	8.04E-05	6.70E-05	6.36E-05	1.15E-04
C48B4.1.1	243	297	165	265	1.30E-05	1.51E-05	5.76E-06	1.14E-05
C48B4.1.2	239	279	150	252	1.35E-05	1.48E-05	5.50E-06	1.14E-05
C48B4.10	176	182	209	237	3.59E-05	3.51E-05	2.78E-05	3.88E-05
C48B4.11	273	270	282	397	3.31E-05	3.09E-05	2.22E-05	3.87E-05
C48B4.12a	1	10	2	1	2.80E-06	2.65E-06	1.82E-06	2.25E-06
C48B4.12b	1	10	2	1	2.80E-06	2.65E-06	1.82E-06	2.25E-06
C48B4.13	5	20	12	5	2.80E-06	3.91E-06	1.82E-06	2.25E-06
C48B4.2	8	20	11	6	2.80E-06	2.65E-06	1.82E-06	2.25E-06
C48B4.3	11	15	6	7	2.80E-06	2.65E-06	1.82E-06	2.25E-06
C48B4.4a	1309	1309	1266	2610	2.89E-05	2.73E-05	1.82E-05	4.63E-05
C48B4.4b	1367	1377	1321	2689	2.85E-05	2.71E-05	1.79E-05	4.50E-05
C48B4.4c	1312	1306	1260	2619	2.65E-05	2.49E-05	1.66E-05	4.25E-05
C48B4.6	162	228	206	220	3.42E-05	4.54E-05	2.83E-05	3.73E-05
C48B4.7	208	214	440	225	4.24E-05	4.12E-05	5.84E-05	3.69E-05
C48B4.8	74	82	105	84	1.25E-05	1.31E-05	1.16E-05	1.14E-05
C48B4.9	230	163	301	271	4.69E-05	3.14E-05	4.00E-05	4.44E-05
C48B6.10	458	531	1046	314	1.38E-04	1.51E-04	2.05E-04	7.59E-05
C48B6.3.1	267	214	755	708	4.72E-05	3.58E-05	8.69E-05	1.01E-04
C48B6.3.2	110	113	322	341	1.95E-05	1.89E-05	3.72E-05	4.86E-05
C48B6.4	16	47	23	18	2.80E-06	2.65E-06	1.82E-06	2.25E-06
C48B6.5	12	11	12	9	2.80E-06	2.65E-06	1.82E-06	2.25E-06
C48B6.6a	924	806	948	1702	1.36E-05	1.12E-05	9.09E-06	2.02E-05
C48B6.6b	768	656	787	1411	1.30E-05	1.04E-05	8.64E-06	1.91E-05
C48B6.8	15	19	21	11	2.80E-06	2.65E-06	1.82E-06	2.25E-06
C48B6.9	21	20	46	45	2.97E-06	2.67E-06	4.23E-06	5.11E-06
C48C5.1	1	5	3	5	2.80E-06	2.65E-06	1.82E-06	2.25E-06

C48C5.3	6	6	4	4	2.80E-06	2.65E-06	1.82E-06	2.25E-06
C48C5.t4	0	0	1	0	2.80E-06	2.65E-06	1.82E-06	2.25E-06
C48D1.1a	15	22	17	19	2.80E-06	2.65E-06	1.82E-06	2.25E-06
C48D1.1b	18	23	18	22	2.80E-06	2.65E-06	1.82E-06	2.25E-06
C48D1.2	1216	1065	1913	2280	5.40E-05	4.47E-05	5.53E-05	8.14E-05
C48D1.5	3	3	7	4	2.80E-06	2.65E-06	1.82E-06	2.25E-06
C48D1.6	3	7	5	3	2.80E-06	2.65E-06	1.82E-06	2.25E-06
C48D5.2a	122	144	87	141	3.86E-06	4.29E-06	1.82E-06	3.58E-06
C48D5.2b	61	83	39	67	3.53E-06	4.52E-06	1.82E-06	3.10E-06
C48D5.2c	58	84	40	65	3.67E-06	5.03E-06	1.82E-06	3.31E-06
C48D5.3	7	9	1	1	2.80E-06	2.65E-06	1.82E-06	2.25E-06
C48E7.1	405	711	335	470	5.73E-05	9.51E-05	3.09E-05	5.35E-05
C48E7.10	100	156	313	185	2.70E-05	3.98E-05	5.50E-05	4.01E-05
C48E7.11	191	141	214	135	5.91E-05	4.12E-05	4.31E-05	3.36E-05
C48E7.2	338	413	401	490	2.04E-05	2.36E-05	1.58E-05	2.38E-05
C48E7.3	256	261	151	308	2.88E-05	2.78E-05	1.11E-05	2.79E-05
C48E7.5	34	30	17	20	2.80E-06	2.65E-06	1.82E-06	2.25E-06
C48E7.6	120	187	171	159	2.80E-06	3.49E-06	2.19E-06	2.52E-06
C48E7.7	325	626	295	133	7.93E-05	1.44E-04	4.68E-05	2.61E-05
C48E7.8	44	65	13	29	2.80E-06	2.80E-06	1.82E-06	2.25E-06
C48E7.9	7	9	4	2	2.80E-06	2.65E-06	1.82E-06	2.25E-06
C48G7.1	278	301	367	635	1.57E-05	1.61E-05	1.35E-05	2.89E-05
C48G7.2	124	129	158	254	9.74E-06	9.58E-06	8.09E-06	1.60E-05
C48G7.3	605	761	646	1168	2.02E-05	2.40E-05	1.40E-05	3.13E-05
C49A1.1	7	8	12	8	2.80E-06	2.65E-06	1.82E-06	2.25E-06
C49A1.10	62	119	37	57	1.10E-05	1.99E-05	4.26E-06	8.10E-06
C49A1.2	3	20	6	6	2.80E-06	2.65E-06	1.82E-06	2.25E-06
C49A1.3	12	24	6	8	2.80E-06	2.65E-06	1.82E-06	2.25E-06
C49A1.4a	12	16	11	18	2.80E-06	2.65E-06	1.82E-06	2.25E-06
C49A1.4b.1	11	14	9	16	2.80E-06	2.65E-06	1.82E-06	2.25E-06
C49A1.4b.2	10	14	10	16	2.80E-06	2.65E-06	1.82E-06	2.25E-06
C49A1.5	0	1	7	1	2.80E-06	2.65E-06	1.82E-06	2.25E-06
C49A1.6	1	3	2	0	2.80E-06	2.65E-06	1.82E-06	2.25E-06
C49A1.9	7	10	12	6	2.80E-06	2.65E-06	1.82E-06	2.25E-06
C49A9.1	16	15	13	5	2.80E-06	2.65E-06	1.82E-06	2.25E-06
C49A9.10	102	105	80	92	3.34E-05	3.25E-05	1.71E-05	2.42E-05
C49A9.2	108	112	89	114	6.52E-06	6.40E-06	3.50E-06	5.53E-06
C49A9.3	46	58	51	29	2.80E-06	3.20E-06	1.95E-06	2.25E-06
C49A9.4	77	75	70	87	4.45E-06	4.07E-06	2.62E-06	4.03E-06
C49A9.5	43	44	33	30	3.02E-06	2.94E-06	1.82E-06	2.25E-06
C49A9.6	46	68	27	41	4.03E-06	5.63E-06	1.82E-06	2.88E-06
C49A9.7	2	13	3	2	2.80E-06	2.65E-06	1.82E-06	2.25E-06
C49A9.8.1	22	30	20	17	2.80E-06	2.65E-06	1.82E-06	2.25E-06
C49A9.8.2	21	29	20	15	2.80E-06	2.65E-06	1.82E-06	2.25E-06
C49A9.9b	110	137	116	158	6.97E-06	8.20E-06	4.79E-06	8.05E-06
C49C3.1	70	188	98	159	3.25E-06	8.23E-06	2.95E-06	5.91E-06
C49C3.10	42	55	26	52	3.72E-06	4.63E-06	1.82E-06	3.71E-06
C49C3.11	9	5	8	1	2.80E-06	2.65E-06	1.82E-06	2.25E-06
C49C3.12	0	3	2	1	2.80E-06	2.65E-06	1.82E-06	2.25E-06
C49C3.13	34	24	10	13	2.80E-06	2.65E-06	1.82E-06	2.25E-06
C49C3.15	18	24	11	22	2.80E-06	3.17E-06	1.82E-06	2.47E-06
C49C3.2	5	8	6	2	2.80E-06	2.65E-06	1.82E-06	2.25E-06
C49C3.3	9	18	4	5	2.80E-06	4.26E-06	1.82E-06	2.25E-06
C49C3.4	1263	1775	2438	2410	2.99E-05	3.97E-05	3.76E-05	4.58E-05
C49C3.5	23	36	15	22	3.02E-06	4.44E-06	1.82E-06	2.32E-06
C49C3.6	155	213	191	221	2.13E-05	2.77E-05	1.71E-05	2.44E-05
C49C3.7	262	275	370	406	3.28E-05	3.25E-05	3.01E-05	4.08E-05
C49C3.8	20	40	39	39	3.00E-06	5.66E-06	3.81E-06	4.70E-06
C49C3.9	89	136	82	90	6.22E-06	8.97E-06	3.72E-06	5.04E-06

C49C8.1	20	30	41	15	2.80E-06	2.65E-06	1.93E-06	2.25E-06
C49C8.2	7	12	6	5	2.80E-06	2.65E-06	1.82E-06	2.25E-06
C49C8.3	15	17	19	20	3.47E-06	3.73E-06	2.86E-06	3.73E-06
C49C8.4	47	88	38	49	3.53E-06	6.22E-06	1.86E-06	2.95E-06
C49C8.5.1	162	294	188	178	1.31E-05	2.24E-05	9.86E-06	1.15E-05
C49C8.5.2	157	292	182	168	1.29E-05	2.27E-05	9.75E-06	1.11E-05
C49C8.6	4	3	4	2	2.80E-06	2.65E-06	1.82E-06	2.25E-06
C49D10.10	7	14	9	8	2.80E-06	2.65E-06	1.82E-06	2.25E-06
C49D10.11	1	0	2	2	2.80E-06	2.65E-06	1.82E-06	2.25E-06
C49D10.2	0	2	3	2	2.80E-06	2.65E-06	1.82E-06	2.25E-06
C49D10.3	6	3	3	2	2.80E-06	2.65E-06	1.82E-06	2.25E-06
C49D10.4	14	26	11	11	2.80E-06	4.21E-06	1.82E-06	2.25E-06
C49D10.6	0	4	2	0	2.80E-06	2.65E-06	1.82E-06	2.25E-06
C49D10.7	1	4	0	1	2.80E-06	2.65E-06	1.82E-06	2.25E-06
C49D10.8	6	8	4	5	2.80E-06	2.65E-06	1.82E-06	2.25E-06
C49D10.9	7	3	8	4	2.80E-06	2.65E-06	1.82E-06	2.25E-06
C49F5.1.1	2959	4087	2970	5006	1.65E-04	2.16E-04	1.08E-04	2.25E-04
C49F5.1.2	1713	2439	1924	3094	1.37E-04	1.84E-04	1.00E-04	1.99E-04
C49F5.1.3	2492	3471	2599	4129	1.57E-04	2.06E-04	1.06E-04	2.08E-04
C49F5.2	45	74	52	80	2.80E-06	3.25E-06	1.82E-06	2.99E-06
C49F5.3	83	79	131	88	1.20E-05	1.08E-05	1.23E-05	1.02E-05
C49F5.4	11	2	4	7	2.80E-06	2.65E-06	1.82E-06	2.25E-06
C49F5.5	7	8	2	2	2.80E-06	2.65E-06	1.82E-06	2.25E-06
C49F5.6	207	253	340	480	8.68E-06	1.00E-05	9.27E-06	1.62E-05
C49F5.7.1	185	170	531	142	5.29E-05	4.59E-05	9.87E-05	3.26E-05
C49F5.7.2	186	169	531	141	5.38E-05	4.62E-05	1.00E-04	3.28E-05
C49F5.8	19	26	32	15	2.80E-06	2.65E-06	1.82E-06	2.25E-06
C49F8.1	41	50	23	20	3.75E-06	4.31E-06	1.82E-06	2.25E-06
C49F8.2	91	81	87	108	4.20E-06	3.54E-06	2.62E-06	4.03E-06
C49F8.3	144	204	69	31	2.66E-05	3.55E-05	8.29E-06	4.59E-06
C49G7.1	7	27	12	10	2.80E-06	2.65E-06	1.82E-06	2.25E-06
C49G7.11	2	3	1	1	2.80E-06	2.65E-06	1.82E-06	2.25E-06
C49G7.2	10	5	15	3	2.80E-06	2.65E-06	1.82E-06	2.25E-06
C49G7.4	169	312	159	132	2.02E-05	3.52E-05	1.24E-05	1.27E-05
C49G7.5	11	12	41	15	2.80E-06	2.65E-06	3.21E-06	2.25E-06
C49G7.6	8	15	5	6	3.44E-06	6.11E-06	1.82E-06	2.25E-06
C49G7.7	30	32	26	20	2.80E-06	2.65E-06	1.82E-06	2.25E-06
C49G7.8	55	44	49	36	4.14E-06	3.15E-06	2.41E-06	2.25E-06
C49G9.1	5	7	7	4	2.80E-06	2.65E-06	1.82E-06	2.25E-06
C49H3.1	19	18	21	15	2.80E-06	2.65E-06	1.82E-06	2.25E-06
C49H3.10	1015	870	1364	2135	3.28E-05	2.66E-05	2.87E-05	5.55E-05
C49H3.11.1	6851	6950	10605	13043	8.09E-04	7.75E-04	8.14E-04	1.24E-03
C49H3.11.2	7215	7307	11367	13346	8.66E-04	8.29E-04	8.88E-04	1.29E-03
C49H3.11.3	6261	6214	9956	12375	8.42E-04	7.89E-04	8.71E-04	1.34E-03
C49H3.12	12	12	11	5	3.33E-06	3.15E-06	1.99E-06	2.25E-06
C49H3.4	61	61	25	64	9.16E-06	8.65E-06	2.44E-06	7.71E-06
C49H3.5a	1349	1246	1521	2646	4.52E-05	3.94E-05	3.31E-05	7.12E-05
C49H3.5b	1025	967	1138	2055	4.53E-05	4.04E-05	3.27E-05	7.30E-05
C49H3.6a	298	287	407	586	1.76E-05	1.60E-05	1.56E-05	2.77E-05
C49H3.6b	285	265	375	563	1.84E-05	1.62E-05	1.58E-05	2.92E-05
C49H3.8	191	258	267	325	2.33E-05	2.97E-05	2.12E-05	3.18E-05
C49H3.9	540	436	603	911	4.16E-05	3.17E-05	3.02E-05	5.64E-05
C50A2.2	138	101	187	312	9.21E-06	6.37E-06	8.13E-06	1.67E-05
C50A2.3	34	51	54	36	6.78E-06	9.63E-06	7.02E-06	5.78E-06
C50A2.4	0	3	8	3	2.80E-06	2.65E-06	1.82E-06	2.25E-06
C50A2.t1	0	0	1	0	2.80E-06	2.65E-06	1.82E-06	2.25E-06
C50B6.1	6	4	3	0	2.80E-06	2.65E-06	1.82E-06	2.25E-06
C50B6.10	4	2	4	1	2.80E-06	2.65E-06	1.82E-06	2.25E-06
C50B6.11	5	4	7	4	2.80E-06	2.65E-06	1.82E-06	2.25E-06

C50B6.12	6	4	6	4	2.80E-06	2.65E-06	1.82E-06	2.25E-06
C50B6.13	8	9	4	5	2.80E-06	2.65E-06	1.82E-06	2.25E-06
C50B6.2.1	3074	2859	4428	5188	1.78E-04	1.57E-04	1.67E-04	2.42E-04
C50B6.2.2	2941	2739	4242	5092	1.87E-04	1.64E-04	1.75E-04	2.60E-04
C50B6.3	525	479	717	1040	3.14E-05	2.71E-05	2.79E-05	5.00E-05
C50B6.4	1067	1046	522	374	1.30E-04	1.20E-04	4.14E-05	3.66E-05
C50B6.5	10	4	8	5	2.80E-06	2.65E-06	1.82E-06	2.25E-06
C50B6.6	1	7	2	3	2.80E-06	2.65E-06	1.82E-06	2.25E-06
C50B6.7	125	109	171	101	6.41E-06	5.26E-06	5.70E-06	4.16E-06
C50B6.8	26	16	14	23	2.80E-06	2.65E-06	1.82E-06	2.25E-06
C50B6.9	5	8	6	2	2.80E-06	2.65E-06	1.82E-06	2.25E-06
C50B8.3	117	161	137	139	1.28E-05	1.67E-05	9.77E-06	1.22E-05
C50B8.4	13	22	6	5	3.00E-06	4.76E-06	1.82E-06	2.25E-06
C50B8.5	21	39	19	20	3.53E-06	6.16E-06	2.08E-06	2.70E-06
C50B8.6	27	46	16	25	4.93E-06	7.91E-06	1.90E-06	3.67E-06
C50C10.1	10	9	15	14	2.80E-06	2.65E-06	1.82E-06	2.25E-06
C50C10.2	3	4	2	4	2.80E-06	2.65E-06	1.82E-06	2.25E-06
C50C10.3	9	11	7	4	2.80E-06	2.65E-06	1.82E-06	2.25E-06
C50C10.4	7	9	5	1	2.80E-06	2.65E-06	1.82E-06	2.25E-06
C50C10.5	8	6	4	2	2.80E-06	2.65E-06	1.82E-06	2.25E-06
C50C10.6	3	3	2	2	2.80E-06	2.65E-06	1.82E-06	2.25E-06
C50C10.7	3	1	3	2	2.80E-06	2.65E-06	1.82E-06	2.25E-06
C50C10.8	6	7	15	5	2.80E-06	2.65E-06	1.82E-06	2.25E-06
C50C10.9	13	11	25	13	2.80E-06	2.65E-06	1.82E-06	2.25E-06
C50C3.1	325	253	381	542	2.58E-05	1.90E-05	1.97E-05	3.46E-05
C50C3.12	7	12	23	9	2.80E-06	2.65E-06	2.20E-06	2.25E-06
C50C3.2	78	106	92	53	2.80E-06	2.65E-06	1.82E-06	2.25E-06
C50C3.5	17	27	11	20	3.14E-06	4.68E-06	1.82E-06	2.95E-06
C50C3.6	4111	3759	5714	8247	6.24E-05	5.39E-05	5.64E-05	1.01E-04
C50C3.7	127	172	331	309	1.02E-05	1.31E-05	1.73E-05	2.00E-05
C50C3.8.1	1222	1211	1775	2243	8.00E-05	7.49E-05	7.57E-05	1.18E-04
C50C3.8.2	919	914	1318	1676	7.98E-05	7.50E-05	7.45E-05	1.17E-04
C50C3.9a	139	192	112	145	3.86E-06	5.03E-06	2.02E-06	3.24E-06
C50C3.9b	32	43	21	16	5.88E-06	7.46E-06	2.51E-06	2.36E-06
C50D2.1	57	111	29	16	3.50E-06	6.43E-06	1.82E-06	2.25E-06
C50D2.10	9	7	2	5	5.32E-06	3.91E-06	1.82E-06	2.38E-06
C50D2.2	27	40	21	20	2.80E-06	2.65E-06	1.82E-06	2.25E-06
C50D2.3	8	14	2	7	2.80E-06	3.23E-06	1.82E-06	2.25E-06
C50D2.4	44	48	29	22	4.54E-06	4.68E-06	1.95E-06	2.25E-06
C50D2.5	232	265	342	227	5.49E-05	5.93E-05	5.27E-05	4.32E-05
C50D2.6	13	15	290	45	2.80E-06	2.65E-06	1.71E-05	3.28E-06
C50D2.7.1	230	333	299	405	1.44E-05	1.96E-05	1.21E-05	2.03E-05
C50D2.7.2	181	284	224	351	1.32E-05	1.96E-05	1.07E-05	2.06E-05
C50D2.8	78	72	145	218	8.32E-06	7.25E-06	1.00E-05	1.86E-05
C50D2.9	138	149	130	163	1.25E-05	1.27E-05	7.67E-06	1.19E-05
C50E10.1	28	34	56	8	6.19E-06	7.12E-06	8.07E-06	2.25E-06
C50E10.10	5	3	9	4	2.80E-06	2.65E-06	1.82E-06	2.25E-06
C50E10.11	7	5	15	7	2.80E-06	2.65E-06	1.82E-06	2.25E-06
C50E10.2	10	21	7	10	2.80E-06	2.65E-06	1.82E-06	2.25E-06
C50E10.3	3	3	3	4	2.80E-06	2.65E-06	1.82E-06	2.25E-06
C50E10.4	82	130	171	152	3.58E-06	5.37E-06	4.87E-06	5.33E-06
C50E10.5	4	9	10	6	2.80E-06	2.65E-06	1.82E-06	2.25E-06
C50E10.6	11	6	29	8	2.80E-06	2.65E-06	1.93E-06	2.25E-06
C50E10.7	4	6	6	8	2.80E-06	2.65E-06	1.82E-06	2.25E-06
C50E10.8	5	11	6	3	2.80E-06	2.65E-06	1.82E-06	2.25E-06
C50E10.9	1	3	10	5	2.80E-06	2.65E-06	1.82E-06	2.25E-06
C50E3.1	3	2	0	2	2.80E-06	2.65E-06	1.82E-06	2.25E-06
C50E3.10	3	2	6	0	2.80E-06	2.65E-06	1.82E-06	2.25E-06
C50E3.11	73	74	104	114	1.13E-05	1.08E-05	1.05E-05	1.42E-05

C50E3.12	78	85	143	127	7.62E-06	7.83E-06	9.07E-06	9.94E-06
C50E3.13	80	74	121	110	6.78E-06	5.92E-06	6.67E-06	7.49E-06
C50E3.14	0	3	3	0	2.80E-06	2.65E-06	1.82E-06	2.25E-06
C50E3.15	6	2	5	4	2.80E-06	2.65E-06	1.82E-06	2.25E-06
C50E3.16	6	6	4	10	2.80E-06	2.65E-06	1.82E-06	2.25E-06
C50E3.2	1	1	2	2	2.80E-06	2.65E-06	1.82E-06	2.25E-06
C50E3.3	3	1	1	2	2.80E-06	2.65E-06	1.82E-06	2.25E-06
C50E3.5	264	273	395	556	1.89E-05	1.85E-05	1.84E-05	3.20E-05
C50E3.6	19	28	9	13	2.80E-06	2.65E-06	1.82E-06	2.25E-06
C50E3.7	7	2	4	1	2.80E-06	2.65E-06	1.82E-06	2.25E-06
C50E3.8	0	4	9	1	2.80E-06	2.65E-06	2.26E-06	2.25E-06
C50E3.9	7	11	14	6	2.80E-06	2.65E-06	1.82E-06	2.25E-06
C50F2.1	10	13	6	5	2.80E-06	2.65E-06	1.82E-06	2.25E-06
C50F2.10	3	47	17	18	2.80E-06	1.45E-05	3.63E-06	4.75E-06
C50F2.2	535	423	544	853	1.88E-05	1.41E-05	1.25E-05	2.41E-05
C50F2.4	27	40	51	35	5.43E-06	7.59E-06	6.67E-06	5.65E-06
C50F2.5	45	130	33	26	4.23E-06	1.16E-05	2.02E-06	2.25E-06
C50F2.6a.1	300	347	144	104	4.20E-05	4.59E-05	1.31E-05	1.17E-05
C50F2.6a.2	272	303	121	100	3.83E-05	4.03E-05	1.11E-05	1.13E-05
C50F2.6b	294	338	134	118	3.19E-05	3.47E-05	9.46E-06	1.03E-05
C50F2.7	3	27	6	9	2.80E-06	5.24E-06	1.82E-06	2.25E-06
C50F2.8	21	29	14	29	2.80E-06	2.65E-06	1.82E-06	2.25E-06
C50F2.9	3	9	6	5	2.80E-06	2.65E-06	1.82E-06	2.25E-06
C50F4.10	26	55	15	9	3.05E-06	6.11E-06	1.82E-06	2.25E-06
C50F4.11	439	591	575	817	2.23E-05	2.84E-05	1.90E-05	3.34E-05
C50F4.12	157	143	242	327	1.26E-05	1.08E-05	1.26E-05	2.11E-05
C50F4.13.1	239	256	300	201	5.30E-05	5.36E-05	4.33E-05	3.58E-05
C50F4.13.2	214	230	253	180	4.72E-05	4.79E-05	3.63E-05	3.19E-05
C50F4.14	329	388	369	433	3.00E-05	3.34E-05	2.19E-05	3.17E-05
C50F4.15	9	7	26	5	2.80E-06	2.65E-06	1.82E-06	2.25E-06
C50F4.16.1	343	413	478	615	2.65E-05	3.01E-05	2.40E-05	3.82E-05
C50F4.2	39	67	24	13	2.80E-06	2.96E-06	1.82E-06	2.25E-06
C50F4.3	5	12	17	16	2.80E-06	2.65E-06	1.82E-06	2.25E-06
C50F4.4	36	48	48	62	7.14E-06	8.99E-06	6.20E-06	9.90E-06
C50F4.5	392	563	424	233	4.21E-05	5.72E-05	2.97E-05	2.01E-05
C50F4.6	53	48	91	30	1.56E-05	1.34E-05	1.75E-05	7.11E-06
C50F4.7	213	321	493	243	5.33E-05	7.58E-05	8.02E-05	4.88E-05
C50F4.9	8	11	18	13	2.80E-06	3.04E-06	3.43E-06	3.04E-06
C50F7.10	70	145	60	79	5.18E-06	1.01E-05	2.90E-06	4.70E-06
C50F7.1a	10	5	11	5	2.80E-06	2.65E-06	1.82E-06	2.25E-06
C50F7.1b	11	12	21	11	2.80E-06	2.65E-06	1.82E-06	2.25E-06
C50F7.2	8	32	37	27	2.80E-06	2.65E-06	1.82E-06	2.25E-06
C50F7.3	21	27	17	8	2.80E-06	3.04E-06	1.82E-06	2.25E-06
C50F7.4	679	759	591	918	5.52E-05	5.82E-05	3.12E-05	5.99E-05
C50F7.5	54	185	51	68	6.16E-06	2.00E-05	3.79E-06	6.25E-06
C50F7.6	26	58	38	32	2.80E-06	4.66E-06	2.10E-06	2.25E-06
C50F7.9	6	5	3	4	2.80E-06	2.65E-06	1.82E-06	2.25E-06
C50H11.1	19	41	14	23	2.80E-06	2.80E-06	1.82E-06	2.25E-06
C50H11.10	4	6	1	3	2.80E-06	2.65E-06	1.82E-06	2.25E-06
C50H11.11	7	7	6	1	2.80E-06	2.65E-06	1.82E-06	2.25E-06
C50H11.12	2	11	2	3	2.80E-06	2.65E-06	1.82E-06	2.25E-06
C50H11.13	5	6	2	4	2.80E-06	2.65E-06	1.82E-06	2.25E-06
C50H11.14	5	3	4	3	2.80E-06	2.65E-06	1.82E-06	2.25E-06
C50H11.15	38	61	43	49	2.80E-06	4.23E-06	2.06E-06	2.90E-06
C50H11.16	3	3	10	5	2.80E-06	2.65E-06	1.82E-06	2.25E-06
C50H11.17	6	4	7	3	2.80E-06	2.65E-06	1.82E-06	2.25E-06
C50H11.2	2	3	12	1	2.80E-06	2.65E-06	1.82E-06	2.25E-06
C50H11.3	14	4	29	14	2.80E-06	2.65E-06	1.93E-06	2.25E-06
C50H11.4	1	1	3	2	2.80E-06	2.65E-06	1.82E-06	2.25E-06

C50H11.5	6	2	8	3	2.80E-06	2.65E-06	1.82E-06	2.25E-06
C50H11.6	4	1	6	3	2.80E-06	2.65E-06	1.82E-06	2.25E-06
C50H11.7	4	3	4	3	2.80E-06	2.65E-06	1.82E-06	2.25E-06
C50H11.8	92	102	367	297	2.16E-05	2.26E-05	5.61E-05	5.60E-05
C50H11.9	3	3	1	2	2.80E-06	2.65E-06	1.82E-06	2.25E-06
C50H2.1	215	319	191	352	7.98E-06	1.12E-05	4.61E-06	1.05E-05
C50H2.10	31	19	50	21	1.54E-05	8.94E-06	1.62E-05	8.39E-06
C50H2.12	44	28	56	16	2.19E-05	1.32E-05	1.81E-05	6.39E-06
C50H2.13	16	36	25	29	2.80E-06	5.87E-06	2.81E-06	4.03E-06
C50H2.2a	17	12	22	11	2.80E-06	2.65E-06	1.82E-06	2.25E-06
C50H2.2b	14	8	14	6	2.80E-06	2.65E-06	1.82E-06	2.25E-06
C50H2.3	27	58	48	42	2.80E-06	2.65E-06	1.82E-06	2.25E-06
C50H2.4	4	5	1	5	2.80E-06	2.65E-06	1.82E-06	2.25E-06
C50H2.5	4	5	26	3	2.80E-06	2.65E-06	2.37E-06	2.25E-06
C50H2.6	2	5	6	2	2.80E-06	2.65E-06	1.82E-06	2.25E-06
C50H2.7	1	5	10	1	2.80E-06	2.65E-06	1.82E-06	2.25E-06
C50H2.9	3	4	3	2	2.80E-06	2.65E-06	1.82E-06	2.25E-06
C51E3.1	4	11	18	4	2.80E-06	2.65E-06	1.82E-06	2.25E-06
C51E3.10	37	39	71	20	2.19E-05	2.18E-05	2.74E-05	9.51E-06
C51E3.2	6	6	7	4	2.80E-06	2.65E-06	1.82E-06	2.25E-06
C51E3.3	6	11	8	1	2.80E-06	2.65E-06	1.82E-06	2.25E-06
C51E3.4	5	4	3	2	2.80E-06	2.65E-06	1.82E-06	2.25E-06
C51E3.5	3	4	9	4	2.80E-06	2.65E-06	1.82E-06	2.25E-06
C51E3.6	47	94	49	128	3.16E-06	5.95E-06	2.15E-06	6.90E-06
C51E3.7a.1	469	668	623	556	2.68E-05	3.60E-05	2.31E-05	2.55E-05
C51E3.7a.2	462	646	607	551	2.08E-05	2.75E-05	1.78E-05	2.00E-05
C51E3.8	5	2	13	2	2.80E-06	2.65E-06	1.82E-06	2.25E-06
C51E3.9	127	217	131	162	1.29E-05	2.09E-05	8.67E-06	1.32E-05
C51F7.1	636	868	535	1027	2.07E-05	2.67E-05	1.13E-05	2.69E-05
C51F7.2	6	4	4	4	2.80E-06	2.65E-06	1.82E-06	2.25E-06
C52A10.1	93	195	87	168	6.10E-06	1.21E-05	3.72E-06	8.84E-06
C52A10.2	23	17	31	15	2.80E-06	2.65E-06	1.82E-06	2.25E-06
C52A10.3	13	5	7	14	2.80E-06	2.65E-06	1.82E-06	2.25E-06
C52A11.2	4	11	5	4	2.80E-06	2.65E-06	1.82E-06	2.25E-06
C52A11.3	1	0	0	0	2.80E-06	2.65E-06	1.82E-06	2.25E-06
C52A11.4a	124	190	74	153	2.80E-06	2.83E-06	1.82E-06	2.25E-06
C52A11.4b	29	30	15	26	2.80E-06	2.65E-06	1.82E-06	2.25E-06
C52B11.2	27	32	25	48	3.14E-06	3.49E-06	1.88E-06	4.45E-06
C52B11.3	13	14	13	19	2.80E-06	2.65E-06	1.82E-06	2.25E-06
C52B11.4	11	10	2	2	4.96E-06	4.26E-06	1.82E-06	2.25E-06
C52B11.5	75	136	47	105	1.17E-05	2.01E-05	4.77E-06	1.32E-05
C52B9.10	3	5	1	1	2.80E-06	2.65E-06	1.82E-06	2.25E-06
C52B9.11	3	11	3	2	2.80E-06	2.65E-06	1.82E-06	2.25E-06
C52B9.1a	39	70	54	46	2.80E-06	4.50E-06	2.39E-06	2.52E-06
C52B9.1b	31	55	43	37	2.80E-06	4.55E-06	2.46E-06	2.61E-06
C52B9.2a	14	31	13	32	3.00E-06	6.24E-06	1.82E-06	5.49E-06
C52B9.2b	29	49	19	44	2.80E-06	4.36E-06	1.82E-06	3.33E-06
C52B9.3a	27	26	23	36	2.80E-06	2.65E-06	1.82E-06	2.41E-06
C52B9.3b	31	31	25	45	2.80E-06	2.65E-06	1.82E-06	2.38E-06
C52B9.5	2	6	4	5	2.80E-06	2.65E-06	1.82E-06	2.25E-06
C52B9.6	18	15	10	11	2.80E-06	2.65E-06	1.82E-06	2.25E-06
C52B9.7a	33	58	36	49	2.94E-06	4.87E-06	2.08E-06	3.49E-06
C52B9.7b	17	28	18	16	2.94E-06	4.58E-06	2.02E-06	2.25E-06
C52B9.8	206	198	99	238	5.46E-06	4.95E-06	1.82E-06	5.06E-06
C52B9.9	23	34	16	23	2.80E-06	2.65E-06	1.82E-06	2.25E-06
C52D10.1	56	85	58	42	4.98E-06	7.14E-06	3.35E-06	3.01E-06
C52D10.10	8	9	7	5	2.80E-06	2.65E-06	1.82E-06	2.25E-06
C52D10.11	32	57	55	42	5.18E-06	8.73E-06	5.79E-06	5.47E-06
C52D10.12.1	117	207	127	214	4.76E-06	7.94E-06	3.35E-06	6.97E-06

C52D10.12.2	97	182	99	193	4.40E-06	7.78E-06	2.92E-06	7.02E-06
C52D10.13	681	883	200	268	8.30E-05	1.02E-04	1.59E-05	2.62E-05
C52D10.3	30	46	31	38	3.95E-06	5.69E-06	2.64E-06	4.00E-06
C52D10.4	2	3	4	2	2.80E-06	2.65E-06	1.82E-06	2.25E-06
C52D10.7	23	44	17	11	3.92E-06	7.12E-06	1.90E-06	2.25E-06
C52D10.8	29	24	12	13	5.35E-06	4.18E-06	1.82E-06	2.25E-06
C52D10.9.1	29	48	15	18	4.87E-06	7.64E-06	1.82E-06	2.43E-06
C52D10.9.2	29	48	15	18	4.90E-06	7.67E-06	1.82E-06	2.45E-06
C52E12.1.1	786	822	687	1203	2.88E-05	2.84E-05	1.64E-05	3.54E-05
C52E12.1.2	725	771	652	1152	2.91E-05	2.92E-05	1.70E-05	3.72E-05
C52E12.2b	156	257	171	190	3.47E-06	5.42E-06	2.48E-06	3.40E-06
C52E12.3	562	542	504	736	5.08E-05	4.63E-05	2.96E-05	5.34E-05
C52E12.4	661	702	1027	1387	1.52E-05	1.52E-05	1.54E-05	2.56E-05
C52E12.6	44	52	38	52	4.73E-06	5.29E-06	2.66E-06	4.50E-06
C52E12.t1	0	0	1	0	2.80E-06	2.65E-06	1.82E-06	2.25E-06
C52E12.t2	1	0	2	2	2.80E-06	2.65E-06	2.02E-06	2.50E-06
C52E12.t3	1	0	3	0	2.80E-06	2.65E-06	3.04E-06	2.25E-06
C52E2.1	3	7	5	6	2.80E-06	2.65E-06	1.82E-06	2.25E-06
C52E2.2	1	1	1	2	2.80E-06	2.65E-06	1.82E-06	2.25E-06
C52E2.3	3	2	3	0	2.80E-06	2.65E-06	1.82E-06	2.25E-06
C52E2.4	29	40	32	29	2.80E-06	2.65E-06	1.82E-06	2.25E-06
C52E2.5	13	17	23	15	2.80E-06	2.65E-06	1.82E-06	2.25E-06
C52E2.6	6	13	5	10	2.80E-06	2.65E-06	1.82E-06	2.25E-06
C52E2.7	4	4	7	3	2.80E-06	2.65E-06	1.82E-06	2.25E-06
C52E2.8	5	6	8	3	2.80E-06	2.65E-06	1.82E-06	2.25E-06
C52E4.1	794	1476	546	1793	8.26E-05	1.45E-04	3.70E-05	1.50E-04
C52E4.3.1	983	895	679	817	1.95E-04	1.68E-04	8.76E-05	1.30E-04
C52E4.3.2	841	771	606	764	1.70E-04	1.47E-04	7.96E-05	1.24E-04
C52E4.4.1	1776	1684	2082	2459	1.24E-04	1.11E-04	9.44E-05	1.38E-04
C52E4.4.2	1495	1417	1675	2135	1.04E-04	9.35E-05	7.62E-05	1.20E-04
C52E4.5	292	485	278	436	1.63E-05	2.55E-05	1.01E-05	1.95E-05
C52E4.6a.1	376	472	587	736	2.56E-05	3.03E-05	2.60E-05	4.02E-05
C52E4.6a.2	348	432	534	709	2.41E-05	2.82E-05	2.40E-05	3.94E-05
C52E4.7	32	56	26	12	4.20E-06	6.93E-06	2.22E-06	2.25E-06
C52E4.8	1	0	1	1	2.80E-06	2.65E-06	1.82E-06	2.25E-06
C52G5.2	153	237	62	71	2.56E-05	3.74E-05	6.74E-06	9.54E-06
C52G5.3	2	2	1	2	2.80E-06	2.65E-06	1.82E-06	2.25E-06
C53A3.1	5	17	6	5	2.80E-06	2.65E-06	1.82E-06	2.25E-06
C53A3.2	246	940	294	534	2.30E-05	8.31E-05	1.79E-05	4.01E-05
C53A3.3	0	1	0	0	2.80E-06	2.65E-06	1.82E-06	2.25E-06
C53A5.1.1	2016	2160	3122	1704	3.80E-04	3.85E-04	3.83E-04	2.58E-04
C53A5.1.2	1706	1881	2579	1602	3.26E-04	3.40E-04	3.21E-04	2.46E-04
C53A5.1.3	1919	1998	2912	1607	3.62E-04	3.56E-04	3.57E-04	2.43E-04
C53A5.10	5	3	4	3	2.80E-06	2.65E-06	1.82E-06	2.25E-06
C53A5.11	12	8	5	3	2.80E-06	2.65E-06	1.82E-06	2.25E-06
C53A5.13	45	83	55	74	2.80E-06	4.07E-06	1.86E-06	3.10E-06
C53A5.2	310	354	367	447	1.01E-05	1.08E-05	7.74E-06	1.16E-05
C53A5.3.1	1544	1442	1882	2654	9.44E-05	8.33E-05	7.49E-05	1.30E-04
C53A5.3.2	1168	996	1343	1931	9.31E-05	7.50E-05	6.97E-05	1.24E-04
C53A5.4	11	16	9	3	2.80E-06	2.65E-06	1.82E-06	2.25E-06
C53A5.5	14	23	21	11	2.80E-06	2.65E-06	1.82E-06	2.25E-06
C53A5.6	201	171	360	513	1.31E-05	1.06E-05	1.53E-05	2.69E-05
C53A5.8	7	1	12	4	2.80E-06	2.65E-06	1.82E-06	2.25E-06
C53A5.9	8	2	9	2	2.80E-06	2.65E-06	1.82E-06	2.25E-06
C53B4.1	9	17	16	22	2.80E-06	2.65E-06	1.82E-06	2.25E-06
C53B4.2	61	99	45	43	5.04E-06	7.70E-06	2.41E-06	2.86E-06
C53B4.3.1	103	108	67	75	6.83E-06	6.74E-06	2.88E-06	3.98E-06
C53B4.3.2	94	97	57	66	8.37E-06	8.15E-06	3.30E-06	4.72E-06
C53B4.4a	1320	1199	1844	2632	3.11E-05	2.67E-05	2.83E-05	4.98E-05

C53B4.4b	1469	1341	2118	2997	3.08E-05	2.66E-05	2.89E-05	5.05E-05
C53B4.4c	1329	1196	1818	2590	3.70E-05	3.15E-05	3.30E-05	5.80E-05
C53B4.4d	1461	1330	2062	2920	3.56E-05	3.07E-05	3.27E-05	5.72E-05
C53B4.5	7176	10296	11037	16698	8.26E-04	1.12E-03	8.27E-04	1.54E-03
C53B4.6	81	93	49	103	8.85E-06	9.60E-06	3.48E-06	9.04E-06
C53B4.7b	141	215	70	107	1.28E-05	1.84E-05	4.14E-06	7.80E-06
C53B4.8a	57	88	37	34	2.94E-06	4.26E-06	1.82E-06	2.25E-06
C53B4.8b	57	89	40	35	2.80E-06	4.15E-06	1.82E-06	2.25E-06
C53B4.9	0	1	1	0	2.80E-06	2.65E-06	1.82E-06	2.25E-06
C53B7.2	132	135	150	121	2.51E-05	2.43E-05	1.86E-05	1.85E-05
C53B7.3	154	296	130	196	2.11E-05	3.84E-05	1.16E-05	2.16E-05
C53B7.5	5	13	7	10	2.80E-06	2.65E-06	1.82E-06	2.25E-06
C53B7.6	0	0	0	1	2.80E-06	2.65E-06	1.82E-06	2.25E-06
C53B7.7	20	36	26	44	2.80E-06	2.91E-06	1.82E-06	3.01E-06
C53C11.1	2	5	4	3	2.80E-06	2.65E-06	1.82E-06	2.25E-06
C53C11.2	19	30	55	21	6.22E-06	9.28E-06	1.17E-05	5.53E-06
C53C11.3	56	83	52	91	2.80E-06	2.65E-06	1.82E-06	2.32E-06
C53C11.4	2	5	7	1	2.80E-06	2.65E-06	1.82E-06	2.25E-06
C53C11.5	4	5	5	9	2.80E-06	2.65E-06	1.82E-06	2.25E-06
C53C7.1a	5	11	5	4	2.80E-06	2.65E-06	1.82E-06	2.25E-06
C53C7.1b	5	10	5	4	2.80E-06	2.65E-06	1.82E-06	2.25E-06
C53C7.3	2	2	3	2	2.80E-06	2.65E-06	1.82E-06	2.25E-06
C53C7.4	5	10	7	5	2.80E-06	2.65E-06	1.82E-06	2.25E-06
C53C9.2	312	343	214	259	1.88E-05	1.95E-05	8.40E-06	1.25E-05
C53C9.3a	19	32	12	14	2.80E-06	2.65E-06	1.82E-06	2.25E-06
C53C9.3b	12	19	8	11	2.80E-06	2.65E-06	1.82E-06	2.25E-06
C53C9.3c	10	19	5	10	2.80E-06	2.65E-06	1.82E-06	2.25E-06
C53D5.1a	99	147	162	149	6.61E-06	9.28E-06	7.05E-06	8.01E-06
C53D5.1b	67	103	120	118	5.96E-06	8.65E-06	6.94E-06	8.41E-06
C53D5.1c.1	79	116	138	126	6.83E-06	9.47E-06	7.76E-06	8.75E-06
C53D5.1c.2	62	102	116	115	6.08E-06	9.44E-06	7.40E-06	9.04E-06
C53D5.2	16	25	29	24	2.80E-06	2.80E-06	2.24E-06	2.27E-06
C53D5.3	0	0	0	2	2.80E-06	2.65E-06	1.82E-06	2.25E-06
C53D5.4	70	61	65	86	7.11E-06	5.85E-06	4.30E-06	7.02E-06
C53D5.5.1	90	117	105	165	4.79E-06	5.87E-06	3.64E-06	7.06E-06
C53D5.5.2	78	95	87	150	4.26E-06	4.89E-06	3.10E-06	6.59E-06
C53D5.6	5587	4295	6596	10064	1.69E-04	1.22E-04	1.30E-04	2.44E-04
C53D6.10	24	43	14	5	5.10E-06	8.62E-06	1.93E-06	2.25E-06
C53D6.11	14	18	16	6	2.80E-06	2.65E-06	1.82E-06	2.25E-06
C53D6.2	63	62	36	50	4.62E-06	4.31E-06	1.82E-06	2.95E-06
C53D6.3	14	7	11	7	2.80E-06	2.65E-06	1.82E-06	2.25E-06
C53D6.4	301	272	420	353	3.17E-05	2.71E-05	2.88E-05	2.98E-05
C53D6.5	9	6	10	2	2.80E-06	2.65E-06	1.82E-06	2.25E-06
C53D6.6	51	64	56	74	2.80E-06	3.33E-06	2.00E-06	3.28E-06
C53D6.7	103	147	112	92	1.01E-05	1.35E-05	7.11E-06	7.22E-06
C53D6.8	5	3	4	6	2.80E-06	2.65E-06	1.82E-06	2.25E-06
C53D6.9	2	4	6	1	2.80E-06	2.65E-06	1.82E-06	2.25E-06
C53D6.t1	0	0	1	0	2.80E-06	2.65E-06	1.82E-06	2.25E-06
C53H9.1.1	6476	7328	14540	4872	1.57E-03	1.67E-03	2.29E-03	9.47E-04
C53H9.1.2	5723	6589	10492	3985	1.39E-03	1.51E-03	1.66E-03	7.76E-04
C53H9.2a	784	819	829	1422	5.25E-05	5.18E-05	3.61E-05	7.65E-05
C53H9.2b.1	779	816	827	1415	5.32E-05	5.26E-05	3.68E-05	7.76E-05
C53H9.2b.2	779	803	810	1395	5.24E-05	5.10E-05	3.55E-05	7.54E-05
C53H9.2b.3	751	787	787	1353	5.21E-05	5.16E-05	3.55E-05	7.54E-05
C53H9.2b.4	779	803	810	1395	5.32E-05	5.18E-05	3.60E-05	7.65E-05
C53H9.2c.1	722	761	759	1308	5.32E-05	5.30E-05	3.64E-05	7.74E-05
C53H9.2c.2	656	687	710	1219	5.87E-05	5.81E-05	4.14E-05	8.77E-05
C53H9.2c.3	628	658	670	1157	5.86E-05	5.80E-05	4.07E-05	8.67E-05
C53H9.2c.4	656	674	693	1199	5.99E-05	5.82E-05	4.12E-05	8.80E-05

C53H9.3	102	102	388	88	4.48E-05	4.23E-05	1.11E-04	3.10E-05
C54A12.1	98	181	52	71	3.61E-06	6.27E-06	1.82E-06	2.25E-06
C54A12.2	7	14	9	13	2.80E-06	2.65E-06	1.82E-06	2.25E-06
C54A12.3	0	2	0	1	2.80E-06	2.65E-06	1.82E-06	2.25E-06
C54A12.4	12	29	10	10	2.80E-06	4.26E-06	1.82E-06	2.25E-06
C54C6.1.1	6469	4786	15796	2955	2.04E-03	1.42E-03	3.23E-03	7.47E-04
C54C6.1.2	4129	3242	8634	2180	1.33E-03	9.86E-04	1.81E-03	5.64E-04
C54C6.2	291	338	325	406	2.36E-05	2.59E-05	1.71E-05	2.64E-05
C54C6.4	6	4	4	2	2.80E-06	2.65E-06	1.82E-06	2.25E-06
C54C6.5	8	7	7	6	2.80E-06	2.65E-06	1.82E-06	2.25E-06
C54C6.6	98	126	129	115	1.79E-05	2.18E-05	1.54E-05	1.69E-05
C54C6.7	3	4	9	2	2.80E-06	2.65E-06	1.93E-06	2.25E-06
C54C8.1	5	3	27	4	2.80E-06	2.65E-06	1.84E-06	2.25E-06
C54C8.11	2	8	9	6	2.80E-06	2.65E-06	1.82E-06	2.25E-06
C54C8.12	0	2	2	0	2.80E-06	2.65E-06	1.82E-06	2.25E-06
C54C8.2	13	10	4	6	2.80E-06	2.65E-06	1.82E-06	2.25E-06
C54C8.3	8	7	2	4	2.80E-06	2.65E-06	1.82E-06	2.25E-06
C54C8.4	4	18	11	2	2.80E-06	2.65E-06	1.82E-06	2.25E-06
C54C8.5	1	4	0	0	2.80E-06	2.65E-06	1.82E-06	2.25E-06
C54C8.6	3	1	2	1	2.80E-06	2.65E-06	1.82E-06	2.25E-06
C54C8.7	5	4	3	0	2.80E-06	2.65E-06	1.82E-06	2.25E-06
C54C8.8	0	5	3	2	2.80E-06	2.65E-06	1.82E-06	2.25E-06
C54C8.9	5	4	1	0	2.80E-06	2.65E-06	1.82E-06	2.25E-06
C54D1.1	70	106	61	85	2.80E-06	2.83E-06	1.82E-06	2.25E-06
C54D1.2	22	14	25	7	4.93E-06	2.96E-06	3.64E-06	2.25E-06
C54D1.3	52	67	39	56	2.80E-06	2.65E-06	1.82E-06	2.25E-06
C54D1.5	574	915	1000	1655	1.31E-05	1.97E-05	1.48E-05	3.03E-05
C54D1.6.1	66	98	81	108	2.86E-06	4.02E-06	2.30E-06	3.78E-06
C54D1.6.2	67	101	83	109	2.88E-06	4.13E-06	2.33E-06	3.78E-06
C54D1.7	15	23	15	11	3.61E-06	5.24E-06	2.35E-06	2.25E-06
C54D10.1	93	143	142	81	1.19E-05	1.72E-05	1.18E-05	8.30E-06
C54D10.10	37	57	16	19	6.02E-06	8.78E-06	1.82E-06	2.50E-06
C54D10.12	35	42	24	20	2.80E-06	2.96E-06	1.82E-06	2.25E-06
C54D10.2	3	6	7	2	2.80E-06	2.65E-06	1.82E-06	2.25E-06
C54D10.3.1	172	405	145	108	3.09E-05	6.87E-05	1.69E-05	1.56E-05
C54D10.3.2	147	346	119	98	3.11E-05	6.92E-05	1.64E-05	1.67E-05
C54D10.4	41	54	30	34	3.75E-06	4.66E-06	1.82E-06	2.50E-06
C54D10.5	12	10	4	10	2.80E-06	2.65E-06	1.82E-06	2.25E-06
C54D10.6	4	2	6	4	2.80E-06	2.65E-06	1.82E-06	2.25E-06
C54D10.7	57	65	47	35	2.80E-06	2.65E-06	1.82E-06	2.25E-06
C54D10.8	25	16	6	3	2.80E-06	2.65E-06	1.82E-06	2.25E-06
C54D10.9	9	3	5	4	2.80E-06	2.65E-06	1.82E-06	2.25E-06
C54D2.1	14	18	11	7	2.80E-06	2.65E-06	1.82E-06	2.25E-06
C54D2.2	8	4	3	4	4.14E-06	2.65E-06	1.82E-06	2.25E-06
C54D2.4a	17	36	19	19	2.80E-06	2.65E-06	1.82E-06	2.25E-06
C54D2.5a	125	177	107	128	2.80E-06	3.07E-06	1.82E-06	2.25E-06
C54D2.5c	125	177	108	127	2.80E-06	3.04E-06	1.82E-06	2.25E-06
C54D2.5d	126	177	108	130	2.80E-06	2.99E-06	1.82E-06	2.25E-06
C54D2.5e	125	174	104	125	2.80E-06	2.99E-06	1.82E-06	2.25E-06
C54D2.5f	76	92	64	87	2.80E-06	2.65E-06	1.82E-06	2.25E-06
C54E10.1	3	6	12	1	2.80E-06	2.65E-06	1.82E-06	2.25E-06
C54E10.2	5	10	4	8	2.80E-06	2.65E-06	1.82E-06	2.25E-06
C54E10.3	3	3	4	4	2.80E-06	2.65E-06	1.82E-06	2.25E-06
C54E10.4	4	6	3	3	2.80E-06	2.65E-06	1.82E-06	2.25E-06
C54E10.5	6	22	5	9	2.80E-06	2.65E-06	1.82E-06	2.25E-06
C54E10.6	11	25	30	14	3.33E-06	7.17E-06	5.92E-06	3.42E-06
C54E4.1	3	4	7	5	2.80E-06	2.65E-06	1.82E-06	2.25E-06
C54E4.2a.1	294	453	340	348	4.79E-05	6.97E-05	3.60E-05	4.55E-05
C54E4.2a.2	266	418	277	325	4.34E-05	6.45E-05	2.94E-05	4.26E-05

C54E4.2b	324	513	373	355	4.64E-05	6.94E-05	3.48E-05	4.08E-05
C54E4.3	15	13	20	12	2.80E-06	2.65E-06	1.82E-06	2.25E-06
C54E4.4	5	5	6	3	2.80E-06	2.65E-06	1.82E-06	2.25E-06
C54E4.5	26	43	34	34	5.01E-06	7.83E-06	4.26E-06	5.26E-06
C54F6.1	4	4	12	3	2.80E-06	2.65E-06	1.82E-06	2.25E-06
C54F6.10	5	4	6	2	2.80E-06	2.65E-06	1.82E-06	2.25E-06
C54F6.11	4	3	7	1	2.80E-06	2.65E-06	1.82E-06	2.25E-06
C54F6.12	3	2	0	5	2.80E-06	2.65E-06	1.82E-06	2.25E-06
C54F6.13a	67	100	68	71	3.61E-06	5.08E-06	2.39E-06	3.08E-06
C54F6.13b	62	92	66	70	3.56E-06	4.97E-06	2.46E-06	3.22E-06
C54F6.14	8	19	7	4	2.80E-06	3.57E-06	1.82E-06	2.25E-06
C54F6.15	1	0	1	1	2.80E-06	2.65E-06	1.82E-06	2.25E-06
C54F6.2	4	1	1	2	2.80E-06	2.65E-06	1.82E-06	2.25E-06
C54F6.3	6	17	9	9	2.80E-06	2.65E-06	1.82E-06	2.25E-06
C54F6.4	3	3	5	2	2.80E-06	2.65E-06	1.82E-06	2.25E-06
C54F6.5	1	8	5	2	2.80E-06	4.87E-06	2.10E-06	2.25E-06
C54F6.6	12	6	8	4	4.76E-06	2.65E-06	2.06E-06	2.25E-06
C54F6.7	6	3	8	2	2.80E-06	2.65E-06	1.82E-06	2.25E-06
C54F6.8	3	5	4	2	2.80E-06	2.65E-06	1.82E-06	2.25E-06
C54F6.9	5	15	23	16	2.80E-06	2.65E-06	1.82E-06	2.25E-06
C54G10.1	1	2	1	0	2.80E-06	2.65E-06	1.82E-06	2.25E-06
C54G10.2	720	902	459	1021	3.01E-05	3.57E-05	1.25E-05	3.43E-05
C54G10.3	656	670	892	1439	2.84E-05	2.74E-05	2.51E-05	5.00E-05
C54G10.4a	14	12	6	20	2.80E-06	2.65E-06	1.82E-06	2.25E-06
C54G4.1a	356	521	346	605	1.40E-05	1.94E-05	8.86E-06	1.91E-05
C54G4.1b	65	92	68	103	1.28E-05	1.71E-05	8.69E-06	1.63E-05
C54G4.2	23	35	23	25	2.80E-06	2.72E-06	1.82E-06	2.25E-06
C54G4.3	35	55	27	12	5.32E-06	7.88E-06	2.66E-06	2.25E-06
C54G4.4	25	50	20	13	2.80E-06	2.65E-06	1.82E-06	2.25E-06
C54G4.5	15	14	13	10	3.61E-06	3.20E-06	2.04E-06	2.25E-06
C54G4.6	88	111	67	55	1.74E-05	2.07E-05	8.62E-06	8.73E-06
C54G4.7.1	190	282	239	281	1.22E-05	1.71E-05	9.99E-06	1.45E-05
C54G4.7.2	175	261	234	269	1.46E-05	2.06E-05	1.27E-05	1.80E-05
C54G4.7.3	170	255	228	263	1.42E-05	2.01E-05	1.24E-05	1.76E-05
C54G4.8.1	1014	1228	2091	1806	7.85E-05	8.99E-05	1.05E-04	1.12E-04
C54G4.8.2	571	772	1205	1334	7.29E-05	9.31E-05	1.00E-04	1.37E-04
C54G4.9	174	179	162	269	1.33E-05	1.29E-05	8.05E-06	1.65E-05
C54G6.1a	82	74	122	116	1.02E-05	8.65E-06	9.84E-06	1.15E-05
C54G6.1b	52	50	81	83	7.20E-06	6.56E-06	7.31E-06	9.24E-06
C54G6.2	6	9	9	6	2.80E-06	2.65E-06	1.82E-06	2.25E-06
C54G6.3	2	0	1	2	2.80E-06	2.65E-06	1.82E-06	2.25E-06
C54G6.5	1321	1172	5200	910	4.17E-04	3.49E-04	1.07E-03	2.31E-04
C54G7.1	3	9	3	3	2.80E-06	2.65E-06	1.82E-06	2.25E-06
C54G7.2.1	212	335	179	303	1.34E-05	2.01E-05	7.38E-06	1.54E-05
C54G7.2.2	190	293	163	283	1.47E-05	2.14E-05	8.18E-06	1.75E-05
C54G7.3a	152	168	62	90	2.94E-06	3.07E-06	1.82E-06	2.25E-06
C54G7.3b	123	139	50	64	3.58E-06	3.84E-06	1.82E-06	2.25E-06
C54G7.4	17	31	33	25	2.80E-06	2.65E-06	1.82E-06	2.25E-06
C54H2.1a	31	65	54	67	2.88E-06	5.69E-06	3.26E-06	4.99E-06
C54H2.1b	14	25	31	37	2.91E-06	4.89E-06	4.19E-06	6.16E-06
C54H2.3	98	131	173	228	5.94E-06	7.51E-06	6.81E-06	1.11E-05
C54H2.4	3	10	4	1	2.80E-06	2.65E-06	1.82E-06	2.25E-06
C55A1.1	2	5	1	1	2.80E-06	2.65E-06	1.82E-06	2.25E-06
C55A1.10	37	40	58	60	2.80E-06	2.65E-06	1.82E-06	2.25E-06
C55A1.11	3	1	0	0	2.80E-06	2.65E-06	1.82E-06	2.25E-06
C55A1.12	6	6	7	3	2.80E-06	2.65E-06	1.82E-06	2.25E-06
C55A1.13	5	5	4	0	2.80E-06	2.65E-06	1.82E-06	2.25E-06
C55A1.14	3	2	2	2	2.80E-06	2.65E-06	1.82E-06	2.25E-06
C55A1.15	3	1	2	2	2.80E-06	2.65E-06	1.82E-06	2.25E-06

C55A1.16	10	9	13	8	2.80E-06	2.65E-06	1.82E-06	2.25E-06
C55A1.2	3	8	1	2	2.80E-06	2.65E-06	1.82E-06	2.25E-06
C55A1.3	1	3	5	2	2.80E-06	2.65E-06	1.82E-06	2.25E-06
C55A1.4	1	2	0	0	2.80E-06	2.65E-06	1.82E-06	2.25E-06
C55A1.5	3	4	5	3	2.80E-06	2.65E-06	1.82E-06	2.25E-06
C55A1.6	3	6	2	3	2.80E-06	2.65E-06	1.82E-06	2.25E-06
C55A1.7	1	2	0	0	2.80E-06	2.65E-06	1.82E-06	2.25E-06
C55A1.8	5	5	4	4	2.80E-06	2.65E-06	1.82E-06	2.25E-06
C55A1.9	0	1	4	0	2.80E-06	2.65E-06	1.82E-06	2.25E-06
C55A6.1	352	418	619	434	5.22E-05	5.86E-05	5.97E-05	5.17E-05
C55A6.10	159	193	222	237	1.02E-05	1.17E-05	9.27E-06	1.22E-05
C55A6.11	8	13	3	3	2.80E-06	2.65E-06	1.82E-06	2.25E-06
C55A6.12	226	338	148	168	3.61E-05	5.09E-05	1.54E-05	2.15E-05
C55A6.2	560	696	792	1175	2.71E-05	3.18E-05	2.49E-05	4.56E-05
C55A6.3	19	27	8	10	3.19E-06	4.26E-06	1.82E-06	2.25E-06
C55A6.4	65	95	55	42	9.66E-06	1.34E-05	5.32E-06	5.02E-06
C55A6.5	16	20	26	5	2.80E-06	2.80E-06	2.51E-06	2.25E-06
C55A6.6	27	37	26	12	3.58E-06	4.66E-06	2.26E-06	2.25E-06
C55A6.7	86	47	46	53	1.03E-05	5.32E-06	3.59E-06	5.11E-06
C55A6.8	5	2	6	3	2.80E-06	2.65E-06	1.82E-06	2.25E-06
C55A6.9	526	568	524	673	4.08E-05	4.16E-05	2.65E-05	4.20E-05
C55B6.1a	81	127	130	164	5.10E-06	7.54E-06	5.32E-06	8.28E-06
C55B6.1b	39	72	54	87	3.44E-06	5.98E-06	3.10E-06	6.14E-06
C55B6.2	317	276	248	374	1.82E-05	1.49E-05	9.26E-06	1.72E-05
C55B6.4	3	4	1	1	2.80E-06	2.65E-06	1.82E-06	2.25E-06
C55B6.5	4	2	5	0	2.80E-06	2.65E-06	1.82E-06	2.25E-06
C55B7.1	913	981	514	1302	3.28E-05	3.32E-05	1.20E-05	3.75E-05
C55B7.10	8	20	5	2	2.80E-06	5.50E-06	1.82E-06	2.25E-06
C55B7.11	196	176	241	341	2.25E-05	1.90E-05	1.80E-05	3.14E-05
C55B7.2.1	74	101	70	110	3.98E-06	5.13E-06	2.46E-06	4.75E-06
C55B7.2.2	68	89	62	102	3.08E-06	3.81E-06	1.84E-06	3.73E-06
C55B7.3	48	86	30	38	4.37E-06	7.41E-06	1.82E-06	2.79E-06
C55B7.4a	2593	9697	1786	8525	2.08E-04	7.37E-04	9.34E-05	5.51E-04
C55B7.4b.1	2024	7610	1498	7285	1.62E-04	5.74E-04	7.79E-05	4.67E-04
C55B7.4b.2	2292	8823	1598	7836	1.91E-04	6.93E-04	8.65E-05	5.23E-04
C55B7.4b.3	2495	9250	1751	8070	2.08E-04	7.28E-04	9.49E-05	5.40E-04
C55B7.4b.4	1596	6936	1192	5974	1.71E-04	7.00E-04	8.29E-05	5.13E-04
C55B7.5	388	534	461	742	2.80E-05	3.64E-05	2.16E-05	4.30E-05
C55B7.6	16	12	25	11	2.80E-06	2.65E-06	1.82E-06	2.25E-06
C55B7.8	184	236	260	382	1.25E-05	1.51E-05	1.14E-05	2.08E-05
C55B7.9.1	147	256	195	234	1.43E-05	2.35E-05	1.23E-05	1.83E-05
C55B7.9.2	112	221	144	198	1.76E-05	3.27E-05	1.47E-05	2.49E-05
C55C2.1	2	4	6	0	2.80E-06	2.65E-06	1.82E-06	2.25E-06
C55C2.2	27	20	96	11	8.40E-06	5.87E-06	1.94E-05	2.74E-06
C55C2.3	7	13	4	5	2.80E-06	2.65E-06	1.82E-06	2.25E-06
C55C2.4	5	7	5	5	2.80E-06	2.65E-06	1.82E-06	2.25E-06
C55C2.5a	47	88	35	57	2.88E-06	5.08E-06	1.82E-06	2.79E-06
C55C2.5b	27	65	25	42	2.80E-06	3.76E-06	1.82E-06	2.25E-06
C55C2.5c	47	86	36	60	2.80E-06	4.81E-06	1.82E-06	2.86E-06
C55C3.1	19	16	37	27	2.80E-06	2.65E-06	1.82E-06	2.25E-06
C55C3.2	1	1	4	3	2.80E-06	2.65E-06	1.82E-06	2.25E-06
C55C3.3	48	46	45	25	3.44E-06	3.12E-06	2.10E-06	2.25E-06
C55C3.4	45	62	10	7	3.75E-06	4.89E-06	1.82E-06	2.25E-06
C55C3.5	615	715	665	1127	3.29E-05	3.61E-05	2.31E-05	4.84E-05
C55C3.6	11	11	9	5	2.80E-06	2.65E-06	1.82E-06	2.25E-06
C55C3.7	1	0	1	0	2.80E-06	2.65E-06	1.82E-06	2.25E-06
C55C3.8	6	10	4	4	2.80E-06	2.65E-06	1.82E-06	2.25E-06
C55F2.1a	122	290	132	235	1.04E-05	2.33E-05	7.31E-06	1.61E-05
C55F2.1c	99	248	111	218	9.38E-06	2.22E-05	6.85E-06	1.66E-05

C55F2.2	14	47	13	17	2.80E-06	7.09E-06	1.82E-06	2.25E-06
C55F2.3	0	7	6	2	2.80E-06	8.91E-06	5.27E-06	2.25E-06
C55H1.1	5	5	4	3	2.80E-06	2.65E-06	1.82E-06	2.25E-06
C55H1.2	10	10	11	5	2.80E-06	2.65E-06	1.82E-06	2.25E-06
C56A3.1	14	18	18	12	2.80E-06	2.65E-06	1.82E-06	2.25E-06
C56A3.3	10	17	15	15	2.80E-06	2.65E-06	1.82E-06	2.25E-06
C56A3.4	217	236	284	356	2.70E-05	2.78E-05	2.30E-05	3.56E-05
C56A3.5	347	334	400	498	3.19E-05	2.90E-05	2.40E-05	3.68E-05
C56A3.7	30	28	26	15	3.19E-06	2.80E-06	1.82E-06	2.25E-06
C56A3.9	35	35	25	38	8.62E-06	8.15E-06	4.01E-06	7.51E-06
C56C10.1	339	341	508	637	1.97E-05	1.87E-05	1.92E-05	2.97E-05
C56C10.10	156	275	166	181	1.57E-05	2.61E-05	1.09E-05	1.46E-05
C56C10.11	347	353	499	607	1.57E-05	1.51E-05	1.47E-05	2.20E-05
C56C10.12	249	318	377	487	5.82E-06	7.01E-06	5.72E-06	9.13E-06
C56C10.13a	530	657	791	1043	2.43E-05	2.85E-05	2.36E-05	3.84E-05
C56C10.13b.[illegible]	568	708	811	1077	2.31E-05	2.72E-05	2.15E-05	3.52E-05
C56C10.13b.[illegible]	557	698	789	1059	2.42E-05	2.87E-05	2.24E-05	3.70E-05
C56C10.3.1	598	833	874	1156	4.76E-05	6.27E-05	4.53E-05	7.40E-05
C56C10.3.2	534	735	792	1095	8.94E-05	1.16E-04	8.63E-05	1.47E-04
C56C10.4	21	52	22	12	2.97E-06	6.90E-06	2.02E-06	2.25E-06
C56C10.5	11	6	13	7	2.80E-06	2.65E-06	1.82E-06	2.25E-06
C56C10.6	23	57	30	14	2.80E-06	4.58E-06	1.82E-06	2.25E-06
C56C10.7a.1	176	324	258	351	1.47E-05	2.55E-05	1.40E-05	2.35E-05
C56C10.7a.2	180	328	276	363	1.55E-05	2.68E-05	1.55E-05	2.52E-05
C56C10.7b.1	180	329	282	364	1.39E-05	2.39E-05	1.41E-05	2.25E-05
C56C10.7b.2	160	297	241	326	1.43E-05	2.50E-05	1.40E-05	2.33E-05
C56C10.8.1	7016	6150	9780	6988	8.60E-04	7.12E-04	7.80E-04	6.88E-04
C56C10.8.2	6054	5287	8144	6281	1.03E-03	8.48E-04	8.99E-04	8.56E-04
C56C10.9	154	176	125	180	1.72E-05	1.85E-05	9.07E-06	1.61E-05
C56E10.1	15	36	22	36	2.80E-06	2.65E-06	1.82E-06	2.25E-06
C56E10.3a	14	8	29	11	2.80E-06	2.65E-06	1.82E-06	2.25E-06
C56E10.3b	7	6	14	6	2.80E-06	2.65E-06	1.82E-06	2.25E-06
C56E10.4a	19	57	11	32	2.80E-06	5.53E-06	1.82E-06	2.63E-06
C56E10.4b	19	67	11	35	2.80E-06	5.45E-06	1.82E-06	2.43E-06
C56E10.4c	20	59	15	35	2.80E-06	5.18E-06	1.82E-06	2.61E-06
C56E6.1	346	325	391	628	7.76E-06	6.88E-06	5.70E-06	1.13E-05
C56E6.2	117	133	120	146	1.15E-05	1.24E-05	7.71E-06	1.16E-05
C56E6.3a	443	514	671	959	2.18E-05	2.39E-05	2.15E-05	3.80E-05
C56E6.3b	413	493	633	931	2.31E-05	2.60E-05	2.30E-05	4.18E-05
C56E6.4	11	6	5	4	2.80E-06	2.65E-06	1.82E-06	2.25E-06
C56E6.5	21	21	12	14	2.80E-06	2.65E-06	1.82E-06	2.25E-06
C56E6.6	21	56	32	32	2.80E-06	2.65E-06	1.82E-06	2.25E-06
C56E6.7	89	125	157	236	8.74E-06	1.16E-05	1.00E-05	1.86E-05
C56G2.15	61	63	70	64	1.04E-05	1.02E-05	7.80E-06	8.79E-06
C56G2.1a	1814	1449	2025	2841	7.39E-05	5.58E-05	5.37E-05	9.29E-05
C56G2.1b	1298	1035	1446	1991	8.61E-05	6.49E-05	6.24E-05	1.06E-04
C56G2.3	26	26	41	56	3.05E-06	2.88E-06	3.12E-06	5.26E-06
C56G2.4.1	18	37	15	30	2.80E-06	2.65E-06	1.82E-06	2.25E-06
C56G2.4.2	15	30	15	28	2.80E-06	2.65E-06	1.82E-06	2.25E-06
C56G2.5	36	57	42	82	3.11E-06	4.66E-06	2.37E-06	5.69E-06
C56G2.6.1	1434	1846	1737	2138	1.55E-04	1.88E-04	1.22E-04	1.85E-04
C56G2.6.2	1441	1855	1749	2145	1.69E-04	2.05E-04	1.33E-04	2.02E-04
C56G2.7	2676	2896	3322	3878	1.11E-04	1.14E-04	8.98E-05	1.29E-04
C56G2.9	13	8	13	4	2.80E-06	2.65E-06	1.82E-06	2.25E-06
C56G3.1a.1	19	20	16	12	2.80E-06	2.65E-06	1.82E-06	2.25E-06
C56G3.1a.2	19	19	18	12	2.80E-06	2.65E-06	1.82E-06	2.25E-06
C56G3.1b	19	25	30	13	2.80E-06	2.65E-06	1.82E-06	2.25E-06
C56G3.2	12	16	11	10	2.80E-06	2.65E-06	1.82E-06	2.25E-06
C56G3.t1	1	0	3	0	2.80E-06	2.65E-06	3.04E-06	2.25E-06

C56G7.1.1	867	1091	713	904	1.37E-04	1.63E-04	7.34E-05	1.15E-04
C56G7.1.2	588	765	493	667	1.18E-04	1.45E-04	6.42E-05	1.07E-04
C56G7.2	10	15	13	12	2.80E-06	2.65E-06	1.82E-06	2.25E-06
C56G7.3	48	69	77	37	5.12E-06	6.96E-06	5.36E-06	3.17E-06
C56G7.t1	0	0	3	0	2.80E-06	2.65E-06	2.99E-06	2.25E-06
CC4.2	33	55	44	68	5.01E-06	7.91E-06	4.35E-06	8.30E-06
CC4.3.1	315	334	454	593	1.98E-05	1.98E-05	1.86E-05	3.00E-05
CC4.3.2	365	482	570	681	2.28E-05	2.85E-05	2.32E-05	3.42E-05
CC4.3.3	315	334	453	594	1.98E-05	1.98E-05	1.85E-05	2.99E-05
CC8.1	3	7	6	8	2.80E-06	2.65E-06	1.82E-06	2.25E-06
CC8.2b	101	159	153	181	1.15E-05	1.72E-05	1.14E-05	1.66E-05
CD4.1	11	29	13	9	3.28E-06	8.17E-06	2.53E-06	2.25E-06
CD4.10	43	38	73	44	1.23E-05	1.03E-05	1.36E-05	1.01E-05
CD4.11	2	2	2	1	2.80E-06	2.65E-06	1.82E-06	2.25E-06
CD4.2	80	114	128	138	9.41E-06	1.27E-05	9.80E-06	1.30E-05
CD4.3.1	145	151	170	175	2.64E-05	2.59E-05	2.01E-05	2.55E-05
CD4.3.2	126	137	136	158	2.32E-05	2.38E-05	1.63E-05	2.33E-05
CD4.4	308	334	420	468	4.42E-05	4.53E-05	3.92E-05	5.39E-05
CD4.5	2	4	4	3	2.80E-06	2.65E-06	1.82E-06	2.25E-06
CD4.6.1	1135	1045	1282	1493	1.35E-04	1.18E-04	9.94E-05	1.43E-04
CD4.6.2	1057	966	1196	1447	1.49E-04	1.29E-04	1.10E-04	1.64E-04
CD4.7	111	108	201	153	3.19E-05	2.93E-05	3.76E-05	3.53E-05
CD4.8	78	100	121	124	4.06E-06	4.92E-06	4.10E-06	5.17E-06
CE7X_3.1	43	30	54	88	5.29E-06	3.49E-06	4.34E-06	8.70E-06
CE7X_3.2	21	30	53	82	3.53E-06	4.73E-06	5.78E-06	1.10E-05
cTel54X.1	18	27	14	8	2.80E-06	2.65E-06	2.99E-06	2.25E-06
cTel7X.1	2	0	1	0	2.80E-06	2.65E-06	1.82E-06	2.25E-06
D1005.1	721	899	820	1169	2.80E-06	2.78E-06	1.82E-06	2.25E-06
D1005.2	50	82	69	74	2.80E-06	2.65E-06	1.82E-06	2.25E-06
D1005.3	67	160	56	79	2.21E-05	2.60E-05	1.63E-05	2.87E-05
D1005.4	12	9	13	1	3.89E-06	6.00E-06	3.48E-06	4.61E-06
D1005.5	7	6	2	3	6.36E-06	1.43E-05	3.44E-06	6.00E-06
D1005.6	12	10	23	17	3.98E-06	2.80E-06	2.79E-06	2.25E-06
D1005.t1	33	12	14	4	2.80E-06	2.65E-06	1.82E-06	2.25E-06
D1007.1	3	4	8	3	3.39E-06	2.67E-06	4.23E-06	3.87E-06
D1007.10a	39	48	59	64	4.51E-05	1.55E-05	1.24E-05	4.39E-06
D1007.10b	70	80	96	126	2.80E-06	2.65E-06	1.82E-06	2.25E-06
D1007.10c	29	25	31	57	5.74E-06	6.67E-06	5.65E-06	7.56E-06
D1007.12.1	8872	7941	16926	7839	6.52E-06	7.04E-06	5.83E-06	9.45E-06
D1007.12.2	7278	6467	13143	6891	2.80E-06	2.65E-06	1.82E-06	4.00E-06
D1007.14	78	157	74	92	1.82E-03	1.54E-03	2.26E-03	1.29E-03
D1007.15	88	87	85	148	1.48E-03	1.24E-03	1.74E-03	1.13E-03
D1007.16	488	453	508	635	1.05E-05	1.99E-05	6.47E-06	9.92E-06
D1007.18	2	5	5	4	2.80E-06	2.65E-06	1.82E-06	3.67E-06
D1007.2	4	5	7	6	8.85E-05	7.76E-05	5.99E-05	9.24E-05
D1007.3	24	18	14	13	2.80E-06	2.65E-06	1.82E-06	2.25E-06
D1007.4	45	66	78	73	2.80E-06	2.65E-06	1.82E-06	2.25E-06
D1007.5b.1	400	371	538	723	3.39E-06	2.65E-06	1.82E-06	2.25E-06
D1007.5b.2	274	247	367	484	9.94E-06	1.38E-05	1.12E-05	1.30E-05
D1007.6.1	9836	7122	11526	5469	2.64E-05	2.31E-05	2.31E-05	3.83E-05
D1007.6.2	7685	5699	8196	4696	2.53E-05	2.15E-05	2.20E-05	3.58E-05
D1007.7	818	662	1105	1637	2.04E-03	1.40E-03	1.56E-03	9.11E-04
D1007.8	97	129	118	122	1.61E-03	1.13E-03	1.12E-03	7.91E-04
D1007.9	7	5	12	12	2.73E-05	2.09E-05	2.40E-05	4.39E-05
D1009.1a	322	440	287	335	1.32E-05	1.66E-05	1.05E-05	1.34E-05
D1009.1b	34	44	38	44	2.80E-06	2.65E-06	1.82E-06	2.25E-06
D1009.2a	92	124	133	117	1.51E-05	1.95E-05	8.76E-06	1.26E-05
D1009.2b	55	79	78	78	1.06E-05	1.29E-05	7.69E-06	1.10E-05
D1009.3a	12	12	15	19	5.38E-06	6.85E-06	5.07E-06	5.51E-06

D1009.3b	26	24	24	26	4.59E-06	6.22E-06	4.23E-06	5.22E-06
D1009.4	63	78	61	54	2.80E-06	2.65E-06	1.82E-06	2.25E-06
D1009.5	4	2	2	0	2.80E-06	2.65E-06	1.82E-06	2.25E-06
D1009.t2	0	0	1	0	7.34E-06	8.57E-06	4.61E-06	5.04E-06
D1014.1	28	28	32	27	2.80E-06	2.65E-06	1.82E-06	2.25E-06
D1014.2	31	28	38	43	2.80E-06	2.65E-06	1.82E-06	2.25E-06
D1014.3.1	559	651	586	901	2.80E-06	2.65E-06	1.82E-06	2.25E-06
D1014.3.2	396	492	424	714	2.80E-06	2.65E-06	2.17E-06	3.04E-06
D1014.4	106	150	76	142	4.42E-05	4.87E-05	3.02E-05	5.73E-05
D1014.5	88	202	37	43	4.98E-05	5.84E-05	3.47E-05	7.21E-05
D1014.6	54	4	6	6	1.95E-05	2.61E-05	9.09E-06	2.10E-05
D1014.7	28	11	12	8	4.34E-06	9.44E-06	1.82E-06	2.25E-06
D1014.8	311	352	266	432	4.23E-06	2.65E-06	1.82E-06	2.25E-06
D1022.1a	363	418	490	503	2.80E-06	2.65E-06	1.82E-06	2.25E-06
D1022.1b	301	365	405	452	1.91E-05	2.04E-05	1.06E-05	2.13E-05
D1022.2	4	5	0	3	3.28E-05	3.57E-05	2.88E-05	3.65E-05
D1022.3	13	5	44	8	3.65E-05	4.17E-05	3.19E-05	4.40E-05
D1022.4	125	146	138	90	2.80E-06	2.65E-06	1.82E-06	2.25E-06
D1022.5	1	5	2	1	2.80E-06	2.65E-06	4.34E-06	2.25E-06
D1022.6	10	9	7	13	1.68E-05	1.86E-05	1.21E-05	9.74E-06
D1022.7a	1822	1882	2620	3932	2.80E-06	2.65E-06	1.82E-06	2.25E-06
D1022.7b	1623	1671	2353	3595	2.80E-06	2.65E-06	1.82E-06	2.25E-06
D1022.7c	726	739	1137	1761	4.79E-05	4.67E-05	4.48E-05	8.30E-05
D1022.8	17	18	14	18	4.72E-05	4.59E-05	4.45E-05	8.39E-05
D1022.9	15	38	6	11	4.17E-05	4.01E-05	4.25E-05	8.13E-05
D1025.1	43	49	30	44	2.80E-06	2.65E-06	1.82E-06	2.25E-06
D1025.10	30	30	39	45	2.80E-06	4.63E-06	1.82E-06	2.25E-06
D1025.2	68	60	64	67	3.61E-06	3.86E-06	1.82E-06	2.97E-06
D1025.3	7	10	9	17	2.97E-06	2.80E-06	2.51E-06	3.58E-06
D1025.4	249	512	270	114	1.44E-05	1.20E-05	8.86E-06	1.14E-05
D1025.6	233	474	225	91	2.80E-06	2.65E-06	1.82E-06	2.25E-06
D1025.7	59	120	34	36	7.30E-05	1.42E-04	5.15E-05	2.69E-05
D1025.8	120	287	139	79	7.31E-05	1.40E-04	4.59E-05	2.29E-05
D1025.9	137	294	103	70	1.75E-05	3.36E-05	6.56E-06	8.57E-06
D1037.2	70	95	104	212	3.52E-05	7.95E-05	2.65E-05	1.86E-05
D1037.3.1	1207	1504	873	975	4.00E-05	8.10E-05	1.96E-05	1.64E-05
D1037.3.2	1203	1495	870	968	3.92E-06	5.03E-06	3.79E-06	9.51E-06
D1037.4	619	566	540	704	2.01E-04	2.37E-04	9.48E-05	1.31E-04
D1037.5	19	37	36	18	2.07E-04	2.43E-04	9.74E-05	1.34E-04
D1043.1	478	471	868	1183	6.43E-05	5.55E-05	3.65E-05	5.87E-05
D1044.1	21	35	45	29	2.80E-06	2.65E-06	1.82E-06	2.25E-06
D1044.2a	95	114	58	126	1.86E-05	1.74E-05	2.20E-05	3.70E-05
D1044.2b	53	63	26	79	2.80E-06	3.07E-06	2.71E-06	2.25E-06
D1044.2c	105	123	64	136	3.16E-06	3.60E-06	1.82E-06	3.37E-06
D1044.3	26	66	42	24	3.47E-06	3.91E-06	1.82E-06	4.18E-06
D1044.4	1	0	3	0	3.02E-06	3.36E-06	1.82E-06	3.15E-06
D1044.6	1001	680	1209	1755	2.80E-06	3.36E-06	1.82E-06	2.25E-06
D1044.7	9	23	6	8	2.80E-06	2.65E-06	1.82E-06	2.25E-06
D1044.8	15	14	17	10	3.15E-05	2.02E-05	2.48E-05	4.44E-05
D1046.1.1	638	612	715	916	2.80E-06	2.65E-06	1.82E-06	2.25E-06
D1046.1.2	613	597	699	884	2.80E-06	2.65E-06	1.82E-06	2.25E-06
D1046.1.3	673	663	745	969	4.17E-05	3.78E-05	3.04E-05	4.81E-05
D1046.1.4	676	660	749	969	3.94E-05	3.62E-05	2.92E-05	4.56E-05
D1046.3	104	138	135	175	3.79E-05	3.53E-05	2.74E-05	4.39E-05
D1046.4	34	42	28	55	3.54E-05	3.27E-05	2.55E-05	4.08E-05
D1046.5	80	194	93	127	9.35E-06	1.17E-05	7.91E-06	1.27E-05
D1046.t1	1	0	0	1	2.80E-06	2.65E-06	1.82E-06	2.83E-06
D1053.1	243	263	221	253	4.90E-06	1.12E-05	3.70E-06	6.23E-06
D1053.2	2	9	3	2	2.80E-06	2.65E-06	1.82E-06	2.25E-06

D1053.3	21	40	24	19	3.13E-05	3.20E-05	1.85E-05	2.62E-05
D1053.4	12	17	15	13	2.80E-06	2.65E-06	1.82E-06	2.25E-06
D1054.1	66	98	51	62	2.94E-06	5.32E-06	2.19E-06	2.25E-06
D1054.10	1528	2400	1510	2998	3.50E-06	4.68E-06	2.84E-06	3.04E-06
D1054.11	2508	3093	3450	5969	9.24E-06	1.29E-05	4.65E-06	6.97E-06
D1054.12	6	9	3	9	2.76E-04	4.10E-04	1.78E-04	4.36E-04
D1054.13	106	119	114	143	2.76E-04	3.22E-04	2.47E-04	5.28E-04
D1054.14.1	303	329	509	511	2.80E-06	2.65E-06	1.82E-06	2.25E-06
D1054.15.1	697	742	867	988	7.98E-06	8.46E-06	5.58E-06	8.64E-06
D1054.15.2	608	678	763	929	3.32E-05	3.41E-05	3.63E-05	4.50E-05
D1054.16	11	11	13	17	5.01E-05	5.04E-05	4.05E-05	5.70E-05
D1054.17	2	0	2	0	4.57E-05	4.82E-05	3.74E-05	5.61E-05
D1054.4	16	47	38	65	2.80E-06	2.65E-06	1.82E-06	2.25E-06
D1054.5	19	36	23	31	2.80E-06	2.65E-06	1.82E-06	2.25E-06
D1054.6	1	0	1	0	2.80E-06	4.95E-06	2.75E-06	5.82E-06
D1054.7	0	0	1	1	2.80E-06	3.20E-06	1.82E-06	2.34E-06
D1054.8	92	102	91	113	2.80E-06	2.65E-06	1.82E-06	2.25E-06
D1054.9a	41	82	64	16	2.80E-06	2.65E-06	1.82E-06	2.25E-06
D1054.9b.1	80	128	71	22	1.16E-05	1.22E-05	7.51E-06	1.15E-05
D1054.9b.2	61	107	71	19	3.00E-06	5.69E-06	3.06E-06	2.25E-06
D1054.9c	34	51	16	5	4.12E-06	6.24E-06	2.39E-06	2.25E-06
D1054.9d	48	88	68	16	3.53E-06	5.87E-06	2.68E-06	2.25E-06
D1054.9e	41	84	64	16	5.38E-06	7.59E-06	1.82E-06	2.25E-06
D1065.1	12	8	21	13	3.44E-06	5.95E-06	3.17E-06	2.25E-06
D1065.2	3	3	8	3	3.16E-06	6.14E-06	3.23E-06	2.25E-06
D1065.3	15	21	10	12	2.80E-06	2.65E-06	1.82E-06	2.25E-06
D1065.4a	4	0	7	4	2.80E-06	2.65E-06	1.82E-06	2.25E-06
D1065.4b	4	0	9	4	2.80E-06	2.65E-06	1.82E-06	2.25E-06
D1065.5	2	9	4	4	2.80E-06	2.65E-06	1.82E-06	2.25E-06
D1069.1	6	6	22	7	2.80E-06	2.65E-06	1.82E-06	2.25E-06
D1069.2.1	47	114	24	28	2.80E-06	2.65E-06	1.82E-06	2.25E-06
D1069.2.2	31	88	18	20	2.80E-06	2.65E-06	1.82E-06	2.25E-06
D1069.3a	131	127	159	261	4.45E-06	1.02E-05	1.82E-06	2.25E-06
D1069.3b	167	163	157	278	5.52E-06	1.48E-05	2.08E-06	2.86E-06
D1069.4	14	13	10	16	1.02E-05	9.31E-06	8.04E-06	1.63E-05
D1079.1	2	0	2	1	1.22E-05	1.13E-05	7.47E-06	1.64E-05
D1081.1	2	3	4	2	2.80E-06	2.65E-06	1.82E-06	2.25E-06
D1081.2	54	67	40	58	2.80E-06	2.65E-06	1.82E-06	2.25E-06
D1081.3	35	31	14	10	2.80E-06	2.65E-06	1.82E-06	2.25E-06
D1081.4	19	43	12	7	5.99E-06	7.04E-06	2.90E-06	5.17E-06
D1081.5	29	46	3	9	2.80E-06	2.65E-06	1.82E-06	2.25E-06
D1081.6	31	23	60	49	2.80E-06	3.97E-06	1.82E-06	2.25E-06
D1081.9	168	130	220	252	3.92E-06	5.87E-06	1.82E-06	2.25E-06
D1086.1	24	23	17	18	6.33E-06	4.44E-06	7.96E-06	8.03E-06
D1086.11	457	568	279	1036	2.22E-05	1.62E-05	1.89E-05	2.67E-05
D1086.12a	57	78	29	43	4.51E-06	4.10E-06	2.08E-06	2.72E-06
D1086.12b	59	81	27	42	8.39E-05	9.85E-05	3.33E-05	1.53E-04
D1086.2	6	7	6	7	9.74E-06	1.26E-05	3.23E-06	5.89E-06
D1086.3	199	77	167	17	9.55E-06	1.24E-05	2.84E-06	5.47E-06
D1086.4	99	94	90	149	2.80E-06	2.65E-06	1.82E-06	2.25E-06
D1086.5	10	31	7	21	3.21E-05	1.17E-05	1.75E-05	2.25E-06
D1086.6	509	704	605	994	1.46E-05	1.31E-05	8.64E-06	1.77E-05
D1086.7	201	353	238	317	2.80E-06	4.42E-06	1.82E-06	2.54E-06
D1086.8	46	70	30	21	5.51E-05	7.20E-05	4.26E-05	8.64E-05
D1086.9	10	7	6	3	2.14E-05	3.56E-05	1.65E-05	2.72E-05
D2005.1	223	207	256	454	9.24E-06	1.33E-05	3.92E-06	3.40E-06
D2005.2	52	109	57	46	2.80E-06	2.65E-06	1.82E-06	2.25E-06
D2005.3	172	233	336	157	2.57E-05	2.25E-05	1.92E-05	4.20E-05
D2005.4	582	529	650	940	7.14E-06	1.41E-05	5.08E-06	5.06E-06

D2005.5	752	742	749	1366	3.77E-05	4.82E-05	4.79E-05	2.76E-05
D2005.6	18	22	15	20	1.72E-05	1.48E-05	1.25E-05	2.23E-05
D2007.1	33	52	113	56	2.42E-05	2.26E-05	1.57E-05	3.54E-05
D2007.2	15	14	7	4	2.80E-06	2.65E-06	1.82E-06	2.25E-06
D2007.3	1	2	2	0	1.10E-05	1.64E-05	2.45E-05	1.50E-05
D2007.4	163	202	282	234	2.86E-06	2.65E-06	1.82E-06	2.25E-06
D2007.5.1	233	348	243	383	2.80E-06	2.65E-06	1.82E-06	2.25E-06
D2007.5.2	213	316	219	355	2.88E-05	3.36E-05	3.24E-05	3.32E-05
D2013.1	93	125	100	128	1.78E-05	2.51E-05	1.21E-05	2.35E-05
D2013.10	50	82	79	75	1.76E-05	2.47E-05	1.18E-05	2.36E-05
D2013.2	319	539	440	725	1.51E-05	1.92E-05	1.06E-05	1.67E-05
D2013.3	22	25	22	31	7.56E-06	1.17E-05	7.76E-06	9.11E-06
D2013.5	729	803	774	1172	2.30E-05	3.68E-05	2.07E-05	4.21E-05
D2013.6	169	156	214	294	2.80E-06	2.65E-06	1.82E-06	2.36E-06
D2013.7	951	1123	843	1124	2.56E-05	2.67E-05	1.77E-05	3.31E-05
D2013.8a.1	338	533	397	645	2.67E-05	2.33E-05	2.20E-05	3.74E-05
D2013.8a.2	317	494	374	620	8.78E-05	9.79E-05	5.07E-05	8.34E-05
D2013.8b	154	286	155	224	1.09E-05	1.63E-05	8.38E-06	1.68E-05
D2013.9.1	502	665	576	842	1.08E-05	1.58E-05	8.25E-06	1.69E-05
D2021.1	157	155	162	228	1.14E-05	2.00E-05	7.47E-06	1.33E-05
D2021.2a	97	146	83	118	2.56E-05	3.21E-05	1.91E-05	3.45E-05
D2021.2b	42	57	53	51	4.51E-06	4.21E-06	3.02E-06	5.26E-06
D2021.4a	8	40	12	18	7.70E-06	1.10E-05	4.28E-06	7.53E-06
D2021.4b	5	27	11	11	4.68E-06	6.00E-06	3.84E-06	4.57E-06
D2021.8	31	53	34	32	2.80E-06	4.79E-06	1.82E-06	2.25E-06
D2023.1b.1	39	32	36	43	2.80E-06	4.73E-06	1.82E-06	2.25E-06
D2023.1b.2	33	29	36	33	5.38E-06	8.68E-06	3.84E-06	4.45E-06
D2023.2.1	1977	3014	2649	4169	3.89E-06	3.02E-06	2.33E-06	3.44E-06
D2023.2.2	1990	3043	2699	4198	5.40E-06	4.47E-06	3.83E-06	4.34E-06
D2023.2.3	1965	2994	2639	4160	5.53E-05	7.97E-05	4.82E-05	9.37E-05
D2023.3	9	9	9	6	5.72E-05	8.27E-05	5.05E-05	9.70E-05
D2023.4.1	126	155	151	99	5.73E-05	8.24E-05	5.01E-05	9.74E-05
D2023.4.2	113	141	115	83	2.80E-06	2.65E-06	1.82E-06	2.25E-06
D2023.5	210	294	221	342	2.36E-05	2.74E-05	1.84E-05	1.49E-05
D2023.6	322	428	448	601	2.21E-05	2.61E-05	1.46E-05	1.30E-05
D2023.7	33	48	22	41	2.19E-05	2.90E-05	1.50E-05	2.87E-05
D2024.1	6	26	4	5	2.09E-05	2.63E-05	1.89E-05	3.14E-05
D2024.10	2	5	11	5	3.11E-06	4.29E-06	1.82E-06	3.10E-06
D2024.2	25	33	21	24	2.80E-06	2.65E-06	1.82E-06	2.25E-06
D2024.3	679	656	929	1188	2.80E-06	2.65E-06	3.43E-06	2.25E-06
D2024.4	17	19	6	16	3.14E-06	3.91E-06	1.82E-06	2.43E-06
D2024.5a.1	250	247	323	335	7.90E-05	7.21E-05	7.03E-05	1.11E-04
D2024.5a.2	202	213	206	250	2.80E-06	2.65E-06	1.82E-06	2.25E-06
D2024.5b.1	248	243	322	322	4.00E-05	3.73E-05	3.36E-05	4.30E-05
D2024.5b.2	190	194	201	223	3.64E-05	3.63E-05	2.42E-05	3.62E-05
D2024.6.1	540	637	610	665	3.62E-05	3.35E-05	3.06E-05	3.78E-05
D2024.6.2	524	612	564	637	3.43E-05	3.31E-05	2.36E-05	3.23E-05
D2024.7	17	21	57	14	5.56E-05	6.19E-05	4.09E-05	5.50E-05
D2024.8	52	35	17	24	5.17E-05	5.70E-05	3.62E-05	5.04E-05
D2030.1.1	452	670	426	704	2.80E-06	2.94E-06	5.47E-06	2.25E-06
D2030.1.2	418	637	390	663	6.02E-06	3.84E-06	1.82E-06	2.25E-06
D2030.1.3	378	554	361	611	2.62E-05	3.67E-05	1.61E-05	3.28E-05
D2030.10b	74	109	76	146	2.28E-05	3.29E-05	1.39E-05	2.91E-05
D2030.11	52	79	82	43	2.49E-05	3.45E-05	1.55E-05	3.24E-05
D2030.12	10	19	25	17	2.80E-06	3.57E-06	1.82E-06	4.07E-06
D2030.2a.1	358	663	518	687	1.29E-05	1.85E-05	1.32E-05	8.57E-06
D2030.2a.2	312	572	456	657	2.80E-06	3.91E-06	3.55E-06	2.99E-06
D2030.2a.3	323	592	494	670	1.94E-05	3.39E-05	1.82E-05	2.99E-05
D2030.2b.1	307	570	443	643	1.98E-05	3.43E-05	1.88E-05	3.35E-05

D2030.2b.2	299	551	424	628	2.01E-05	3.48E-05	2.00E-05	3.35E-05
D2030.2b.3	312	572	456	657	2.04E-05	3.59E-05	1.92E-05	3.44E-05
D2030.2b.4	323	592	494	670	1.52E-05	2.65E-05	1.40E-05	2.57E-05
D2030.2b.5	299	550	424	628	1.98E-05	3.43E-05	1.88E-05	3.35E-05
D2030.2b.6	306	563	443	640	2.05E-05	3.55E-05	2.04E-05	3.42E-05
D2030.3	379	418	461	685	1.86E-05	3.23E-05	1.72E-05	3.14E-05
D2030.4.1	411	669	719	282	2.04E-05	3.54E-05	1.92E-05	3.43E-05
D2030.4.2	349	557	556	242	2.60E-05	2.70E-05	2.05E-05	3.77E-05
D2030.6.1	1844	1764	2928	3429	9.24E-05	1.42E-04	1.05E-04	5.09E-05
D2030.6.2	1806	1705	2834	3303	8.23E-05	1.24E-04	8.53E-05	4.58E-05
D2030.7.1	404	496	584	869	7.78E-05	7.03E-05	8.04E-05	1.16E-04
D2030.7.2	402	488	575	857	8.15E-05	7.27E-05	8.33E-05	1.20E-04
D2030.8	446	528	665	809	3.08E-05	3.57E-05	2.90E-05	5.33E-05
D2030.9b	298	456	463	530	3.10E-05	3.55E-05	2.88E-05	5.30E-05
D2045.1a	2068	1700	2372	3362	2.32E-05	2.60E-05	2.26E-05	3.39E-05
D2045.1b.1	1020	896	1409	2009	1.97E-05	2.84E-05	1.99E-05	2.81E-05
D2045.1b.2	2068	1700	2372	3362	6.41E-05	4.98E-05	4.79E-05	8.37E-05
D2045.2	759	866	1362	1624	5.97E-05	4.95E-05	5.37E-05	9.45E-05
D2045.5	7	20	7	4	6.41E-05	4.98E-05	4.79E-05	8.37E-05
D2045.6	610	653	627	997	1.53E-05	1.65E-05	1.79E-05	2.63E-05
D2045.7	28	39	13	15	2.80E-06	2.65E-06	1.82E-06	2.25E-06
D2045.8	31	38	15	17	2.23E-05	2.26E-05	1.49E-05	2.93E-05
D2045.9	43	80	57	66	3.25E-06	4.26E-06	1.82E-06	2.25E-06
D2062.1	4	4	5	3	4.48E-06	5.18E-06	1.82E-06	2.25E-06
D2062.10	5	8	6	6	2.80E-06	4.31E-06	2.11E-06	3.01E-06
D2062.12	16	31	16	19	2.80E-06	2.65E-06	1.82E-06	2.25E-06
D2062.2	4	3	2	4	2.80E-06	2.65E-06	1.82E-06	2.25E-06
D2062.3	2	3	3	4	2.80E-06	2.75E-06	1.82E-06	2.25E-06
D2062.4	9	11	8	4	2.80E-06	2.65E-06	1.82E-06	2.25E-06
D2062.5	10	23	10	8	2.80E-06	2.65E-06	1.82E-06	2.25E-06
D2062.6	48	70	40	17	2.80E-06	2.91E-06	1.82E-06	2.25E-06
D2062.7	69	99	73	28	2.80E-06	5.21E-06	1.82E-06	2.25E-06
D2062.8	7	5	6	3	1.76E-05	2.42E-05	9.53E-06	4.99E-06
D2062.9	6	9	9	6	1.91E-05	2.59E-05	1.31E-05	6.23E-06
D2063.1	8	57	12	23	2.80E-06	2.65E-06	1.82E-06	2.25E-06
D2063.2	4	4	9	2	2.80E-06	2.65E-06	1.82E-06	2.25E-06
D2063.3a	652	533	666	750	2.80E-06	6.14E-06	1.82E-06	2.25E-06
D2063.3b	621	506	643	711	2.80E-06	2.65E-06	1.82E-06	2.25E-06
D2063.4	16	25	14	9	6.27E-05	4.85E-05	4.17E-05	5.80E-05
D2085.1	1649	1569	1414	1690	6.20E-05	4.78E-05	4.18E-05	5.71E-05
D2085.2	8	13	12	10	2.80E-06	3.60E-06	1.82E-06	2.25E-06
D2085.3	466	438	489	705	2.64E-05	2.38E-05	1.47E-05	2.17E-05
D2085.4	456	619	635	953	2.80E-06	2.91E-06	1.84E-06	2.25E-06
D2085.5a	509	635	421	944	2.29E-05	2.03E-05	1.57E-05	2.78E-05
D2085.5b.1	527	668	432	973	1.65E-05	2.11E-05	1.49E-05	2.77E-05
D2085.5b.2	529	669	433	980	1.81E-05	2.13E-05	9.75E-06	2.70E-05
D2085.5b.3	530	669	433	980	1.77E-05	2.12E-05	9.46E-06	2.63E-05
D2085.6	140	164	179	226	1.80E-05	2.15E-05	9.58E-06	2.68E-05
D2085.7	8	19	12	13	1.77E-05	2.11E-05	9.40E-06	2.62E-05
D2089.1a	517	590	574	731	1.13E-05	1.25E-05	9.40E-06	1.46E-05
D2089.1b.1	157	148	161	151	2.80E-06	3.23E-06	1.82E-06	2.25E-06
D2089.1b.2	214	243	211	272	3.75E-05	4.04E-05	2.71E-05	4.26E-05
D2089.1b.3	174	187	165	204	3.07E-05	2.74E-05	2.05E-05	2.37E-05
D2089.2	8	14	2	3	3.18E-05	3.40E-05	2.04E-05	3.24E-05
D2089.3	43	29	91	55	3.04E-05	3.09E-05	1.88E-05	2.86E-05
D2089.4a	120	155	91	144	2.80E-06	2.72E-06	1.82E-06	2.25E-06
D2089.4b.1	75	94	57	81	5.96E-06	3.81E-06	8.22E-06	6.14E-06
D2089.4b.2	73	94	57	81	5.63E-06	6.88E-06	2.79E-06	5.44E-06
D2089.5	56	118	28	61	5.57E-06	6.59E-06	2.75E-06	4.84E-06

D2092.10	1	1	3	1	5.54E-06	6.74E-06	2.81E-06	4.93E-06
D2092.1a	74	123	52	85	4.70E-06	9.36E-06	1.82E-06	4.12E-06
D2092.1b	88	149	71	102	2.80E-06	2.65E-06	1.82E-06	2.25E-06
D2092.2.1	349	438	646	763	3.44E-06	5.40E-06	1.82E-06	3.17E-06
D2092.2.2	276	330	498	585	3.50E-06	5.58E-06	1.84E-06	3.26E-06
D2092.3	11	9	6	10	2.63E-05	3.12E-05	3.17E-05	4.63E-05
D2092.4	402	460	401	501	2.70E-05	3.05E-05	3.17E-05	4.60E-05
D2092.5	531	464	944	1383	2.80E-06	2.65E-06	1.82E-06	2.25E-06
D2092.6	27	21	10	19	3.34E-05	3.61E-05	2.17E-05	3.35E-05
D2092.7	18	30	6	6	2.18E-05	1.80E-05	2.52E-05	4.56E-05
D2092.8	24	53	32	12	3.16E-06	2.65E-06	1.82E-06	2.25E-06
D2096.1	30	62	92	32	2.80E-06	2.65E-06	1.82E-06	2.25E-06
D2096.10	14	8	2	22	5.82E-06	1.22E-05	5.07E-06	2.34E-06
D2096.11	395	435	456	849	7.06E-06	1.38E-05	1.40E-05	6.03E-06
D2096.12	336	300	430	528	4.23E-06	2.65E-06	1.82E-06	5.33E-06
D2096.2a	700	622	609	705	2.02E-05	2.11E-05	1.52E-05	3.50E-05
D2096.2b	192	183	230	290	1.47E-05	1.24E-05	1.22E-05	1.86E-05
D2096.3.1	634	513	1204	697	1.17E-04	9.85E-05	6.65E-05	9.50E-05
D2096.3.2	637	518	1206	699	8.53E-05	7.68E-05	6.65E-05	1.04E-04
D2096.3.3	631	509	1196	695	2.42E-05	1.85E-05	2.99E-05	2.13E-05
D2096.4.1	661	696	712	1010	2.39E-05	1.84E-05	2.94E-05	2.11E-05
D2096.4.2	516	597	586	840	2.51E-05	1.91E-05	3.10E-05	2.22E-05
D2096.5	3	7	12	4	4.18E-05	4.16E-05	2.93E-05	5.13E-05
D2096.6	48	63	17	24	4.02E-05	4.39E-05	2.97E-05	5.26E-05
D2096.7a	136	193	151	235	2.80E-06	2.65E-06	2.11E-06	2.25E-06
D2096.7b	134	188	139	223	7.76E-06	9.63E-06	1.82E-06	3.13E-06
D2096.8	3347	3469	3819	4945	1.48E-05	1.98E-05	1.07E-05	2.05E-05
D2096.9	7	13	17	2	1.43E-05	1.89E-05	9.66E-06	1.91E-05
DC2.1	2	12	3	2	2.80E-04	2.75E-04	2.08E-04	3.33E-04
DC2.2	8	13	11	17	2.94E-06	5.16E-06	4.65E-06	2.25E-06
DC2.3a	143	149	198	199	2.80E-06	2.65E-06	1.82E-06	2.25E-06
DC2.3b	210	266	275	287	2.80E-06	2.65E-06	1.82E-06	2.25E-06
DC2.5	47	51	41	30	2.34E-05	2.30E-05	2.11E-05	2.62E-05
DC2.6	1	2	4	3	1.54E-05	1.84E-05	1.31E-05	1.69E-05
DC2.7a	6	10	14	16	6.61E-06	6.80E-06	3.75E-06	3.40E-06
DC2.7b	5	6	6	10	2.80E-06	2.65E-06	1.82E-06	2.25E-06
DC2.7c	6	10	11	10	2.80E-06	2.65E-06	1.82E-06	2.25E-06
DH11.1.1	84	132	66	104	2.80E-06	2.65E-06	1.82E-06	2.25E-06
DH11.1.2	73	118	61	97	2.80E-06	2.65E-06	1.82E-06	2.25E-06
DH11.2	73	369	57	253	4.45E-06	6.61E-06	2.28E-06	4.43E-06
DH11.3	67	70	60	124	4.48E-06	6.82E-06	2.44E-06	4.77E-06
DH11.4	18	32	16	12	5.32E-06	2.54E-05	2.71E-06	1.48E-05
DH11.5a	55	51	45	51	2.80E-06	2.65E-06	1.82E-06	2.83E-06
DH11.5b	52	53	45	51	2.80E-06	2.65E-06	1.82E-06	2.25E-06
DH11.5c	28	38	31	33	3.72E-06	3.28E-06	1.99E-06	2.79E-06
DY3.1	366	450	914	387	3.75E-06	3.62E-06	2.11E-06	2.97E-06
DY3.2.1	1534	1968	2172	2846	2.91E-06	3.73E-06	2.10E-06	2.74E-06
DY3.2.2	1175	1491	1647	2255	9.51E-05	1.10E-04	1.55E-04	8.08E-05
DY3.3	4	9	8	6	7.76E-05	9.41E-05	7.15E-05	1.16E-04
DY3.4a.1	218	260	205	362	7.67E-05	9.19E-05	7.00E-05	1.18E-04
DY3.4b.1	198	252	181	343	2.80E-06	2.65E-06	1.82E-06	2.25E-06
DY3.4b.2	200	253	183	345	1.36E-05	1.53E-05	8.31E-06	1.81E-05
DY3.4b.3	207	258	203	360	1.31E-05	1.57E-05	7.78E-06	1.82E-05
DY3.4b.4	216	258	201	361	1.33E-05	1.59E-05	7.91E-06	1.84E-05
DY3.5	89	91	47	34	1.37E-05	1.61E-05	8.75E-06	1.91E-05
DY3.6	52	108	57	79	1.43E-05	1.61E-05	8.66E-06	1.92E-05
DY3.7	1329	1402	1622	2497	4.17E-06	4.02E-06	1.82E-06	2.25E-06
DY3.8	357	429	517	467	3.44E-06	6.74E-06	2.44E-06	4.18E-06
E_BE45912.2	25	19	15	23	2.80E-06	2.65E-06	1.82E-06	2.25E-06

E01A2.2a	842	873	834	1342	4.47E-05	4.46E-05	3.55E-05	6.75E-05
E01A2.2b.1	621	664	599	1049	5.43E-05	6.16E-05	5.11E-05	5.70E-05
E01A2.2b.2	790	815	769	1278	4.12E-06	2.96E-06	1.82E-06	3.04E-06
E01A2.3a	9	30	14	10	4.07E-05	3.99E-05	2.62E-05	5.21E-05
E01A2.3b	26	48	33	36	3.25E-05	3.28E-05	2.04E-05	4.41E-05
E01A2.4	680	512	449	779	5.07E-05	4.94E-05	3.21E-05	6.58E-05
E01A2.5	58	75	80	72	2.80E-06	2.65E-06	1.82E-06	2.25E-06
E01A2.6	1254	1212	1492	2339	3.50E-06	6.11E-06	2.90E-06	3.89E-06
E01A2.7	47	65	58	61	4.60E-05	3.27E-05	1.98E-05	4.23E-05
E01A2.8	110	109	122	63	8.65E-06	1.06E-05	7.78E-06	8.64E-06
E01B7.1	126	129	155	231	1.13E-04	1.03E-04	8.71E-05	1.69E-04
E01B7.2	92	114	132	130	4.70E-06	6.14E-06	3.77E-06	4.90E-06
E01F3.1a	33	39	45	76	8.20E-06	7.70E-06	5.92E-06	3.78E-06
E01F3.1b	49	52	67	105	1.13E-05	1.09E-05	9.04E-06	1.66E-05
E01G4.1	150	173	230	371	1.62E-05	1.90E-05	1.51E-05	1.84E-05
E01G4.4	598	394	722	908	2.80E-06	2.65E-06	1.82E-06	3.51E-06
E01G4.5	22	37	37	37	2.80E-06	2.65E-06	1.99E-06	3.82E-06
E01G4.6	1276	2109	431	484	1.39E-05	1.51E-05	1.38E-05	2.75E-05
E01G6.1	88	134	34	56	7.89E-05	4.91E-05	6.20E-05	9.62E-05
E01G6.2	30	51	20	26	2.80E-06	3.09E-06	2.13E-06	2.63E-06
E01G6.3	36	34	31	28	5.18E-05	8.09E-05	1.14E-05	1.58E-05
E01H11.1b	162	266	236	357	2.80E-06	3.23E-06	1.82E-06	2.25E-06
E01H11.1c	187	266	274	349	3.22E-06	5.16E-06	1.82E-06	2.25E-06
E01H11.3	21	15	30	11	2.80E-06	2.65E-06	1.82E-06	2.25E-06
E01H11.4	3	3	4	3	7.67E-06	1.19E-05	7.29E-06	1.36E-05
E01H11.t1	0	0	0	1	7.45E-06	1.00E-05	7.11E-06	1.12E-05
E02A10.1.1	535	614	619	595	5.32E-06	3.60E-06	4.94E-06	2.25E-06
E02A10.1.2	476	555	533	562	2.80E-06	2.65E-06	1.82E-06	2.25E-06
E02A10.2	90	80	28	46	2.80E-06	2.65E-06	1.82E-06	2.25E-06
E02A10.3	14	25	18	13	4.14E-05	4.49E-05	3.12E-05	3.70E-05
E02A10.4	17	14	10	10	4.07E-05	4.48E-05	2.96E-05	3.86E-05
E02C12.10	13	11	15	7	8.71E-06	7.30E-06	1.82E-06	3.58E-06
E02C12.11	1	2	2	2	2.80E-06	4.02E-06	2.00E-06	2.25E-06
E02C12.12	2	3	4	0	3.25E-06	2.65E-06	1.82E-06	2.25E-06
E02C12.13	54	57	99	45	2.80E-06	2.65E-06	1.82E-06	2.25E-06
E02C12.2	2	6	7	4	2.80E-06	2.65E-06	1.82E-06	2.25E-06
E02C12.3	3	7	8	8	2.80E-06	2.65E-06	1.82E-06	2.25E-06
E02C12.4	21	21	24	11	1.15E-05	1.15E-05	1.37E-05	7.71E-06
E02C12.5	11	19	9	11	2.80E-06	2.65E-06	1.82E-06	2.25E-06
E02C12.6	5	13	10	8	2.80E-06	2.65E-06	1.82E-06	2.25E-06
E02C12.8a	16	19	9	12	4.93E-06	4.66E-06	3.68E-06	2.25E-06
E02C12.8b	3	3	2	3	2.80E-06	2.65E-06	1.82E-06	2.25E-06
E02C12.8c	12	17	6	12	2.80E-06	2.65E-06	1.82E-06	2.25E-06
E02C12.9	2	4	2	3	2.80E-06	2.65E-06	1.82E-06	2.25E-06
E02D9.1a	19	35	13	28	2.80E-06	2.65E-06	1.82E-06	2.25E-06
E02D9.1b.1	424	491	282	462	2.80E-06	2.65E-06	1.82E-06	2.25E-06
E02D9.1b.2	408	466	264	449	2.80E-06	2.65E-06	1.82E-06	2.25E-06
E02D9.1c	317	355	207	381	2.80E-06	2.88E-06	1.82E-06	2.25E-06
E02D9.1d	9	18	5	13	3.65E-05	3.99E-05	1.58E-05	3.19E-05
E02H1.2	132	167	214	269	3.74E-05	4.04E-05	1.58E-05	3.31E-05
E02H1.4	458	498	607	875	2.13E-05	2.25E-05	9.04E-06	2.05E-05
E02H1.6	137	153	149	91	2.80E-06	3.33E-06	1.82E-06	2.25E-06
E02H1.7	54	88	57	60	1.25E-05	1.49E-05	1.32E-05	2.04E-05
E02H1.8	279	251	191	133	2.76E-05	2.83E-05	2.38E-05	4.23E-05
E02H4.1	12	17	8	10	2.26E-05	2.39E-05	1.60E-05	1.21E-05
E02H4.2	11	10	18	19	3.53E-06	5.45E-06	2.42E-06	3.15E-06
E02H4.3a	185	224	185	296	8.01E-05	6.81E-05	3.57E-05	3.07E-05
E02H4.3b	98	126	116	203	2.80E-06	2.65E-06	1.82E-06	2.25E-06
E02H4.4	49	89	58	46	2.80E-06	2.65E-06	1.82E-06	2.25E-06

E02H4.5	4	5	9	4	5.63E-06	6.45E-06	3.66E-06	7.24E-06
E02H4.6	57	80	111	89	8.93E-06	1.08E-05	6.87E-06	1.48E-05
E02H9.1	0	5	1	3	2.80E-06	3.70E-06	1.82E-06	2.25E-06
E02H9.2	12	21	7	23	2.80E-06	2.65E-06	1.82E-06	2.25E-06
E02H9.4	8	8	7	7	7.00E-06	9.28E-06	8.87E-06	8.77E-06
E02H9.5	17	29	25	36	2.80E-06	2.65E-06	1.82E-06	2.25E-06
E02H9.6	18	26	28	18	2.80E-06	3.20E-06	1.82E-06	2.97E-06
E02H9.7	45	52	106	69	2.80E-06	2.65E-06	1.82E-06	2.25E-06
E02H9.9	7	12	13	6	2.80E-06	2.65E-06	1.82E-06	2.27E-06
E03A3.1	27	28	16	11	2.80E-06	2.65E-06	1.82E-06	2.25E-06
E03A3.2	234	292	207	304	6.38E-06	6.98E-06	9.78E-06	7.87E-06
E03A3.3	4	7	11	8	2.80E-06	3.89E-06	2.90E-06	2.25E-06
E03A3.4	19	22	13	7	2.80E-06	2.65E-06	1.82E-06	2.25E-06
E03A3.5	7	7	14	5	1.03E-05	1.21E-05	5.92E-06	1.07E-05
E03A3.6	68	79	75	87	2.80E-06	2.65E-06	2.10E-06	2.25E-06
E03D2.1	105	116	106	76	5.57E-06	6.11E-06	2.48E-06	2.25E-06
E03D2.2a	41	71	44	49	2.80E-06	2.65E-06	1.82E-06	2.25E-06
E03D2.2b	33	62	34	42	3.00E-06	3.28E-06	2.15E-06	3.06E-06
E03D2.3	6	3	5	4	1.63E-05	1.70E-05	1.07E-05	9.45E-06
E03D2.4	6	2	4	2	6.58E-06	1.07E-05	4.59E-06	6.30E-06
E03E2.1.1	75	149	91	134	6.13E-06	1.09E-05	4.12E-06	6.27E-06
E03E2.1.2	72	133	81	127	2.80E-06	2.65E-06	1.82E-06	2.25E-06
E03E2.t1	3	2	3	2	2.80E-06	2.65E-06	1.82E-06	2.25E-06
E03E2.t2	3	2	3	2	4.45E-06	8.36E-06	3.52E-06	6.39E-06
E03E2.t3	1	0	3	0	5.10E-06	8.89E-06	3.74E-06	7.22E-06
E03G2.1	14	14	8	15	4.09E-06	2.65E-06	2.66E-06	2.25E-06
E03G2.2	145	242	164	294	4.09E-06	2.65E-06	2.66E-06	2.25E-06
E03G2.3	85	102	99	93	2.80E-06	2.65E-06	2.99E-06	2.25E-06
E03G2.4	13	20	11	13	2.80E-06	2.65E-06	1.82E-06	2.25E-06
E03H12.1	4	6	6	4	3.84E-06	6.03E-06	2.81E-06	6.23E-06
E03H12.2	5	5	2	9	8.43E-06	9.55E-06	6.40E-06	7.40E-06
E03H12.3	11	9	10	6	2.80E-06	2.65E-06	1.82E-06	2.25E-06
E03H12.4	0	3	5	3	2.80E-06	2.65E-06	1.82E-06	2.25E-06
E03H12.5	114	190	58	42	2.80E-06	2.65E-06	1.82E-06	2.25E-06
E03H12.6a	4	5	7	5	2.80E-06	2.65E-06	1.82E-06	2.25E-06
E03H12.6b	5	5	6	3	2.80E-06	2.65E-06	1.82E-06	2.25E-06
E03H12.7	35	39	23	10	1.89E-05	2.97E-05	6.25E-06	5.60E-06
E03H12.8	5	8	2	0	2.80E-06	2.65E-06	1.82E-06	2.25E-06
E03H12.9	3	1	1	2	2.80E-06	2.65E-06	1.82E-06	2.25E-06
E03H4.10	21	50	54	28	7.00E-06	7.38E-06	3.01E-06	2.25E-06
E03H4.11	3	1	1	4	2.80E-06	2.65E-06	1.82E-06	2.25E-06
E03H4.12	3	3	4	1	2.80E-06	2.65E-06	1.82E-06	2.25E-06
E03H4.13	2	3	5	8	2.80E-06	4.10E-06	3.04E-06	2.25E-06
E03H4.2	5	5	2	4	2.80E-06	2.65E-06	1.82E-06	2.25E-06
E03H4.3	0	1	4	1	2.80E-06	2.65E-06	1.82E-06	2.25E-06
E03H4.4	5	3	10	7	2.80E-06	2.65E-06	1.82E-06	2.25E-06
E03H4.5	7	9	9	3	2.80E-06	2.65E-06	1.82E-06	2.25E-06
E03H4.6	6	9	10	3	2.80E-06	2.65E-06	1.82E-06	2.25E-06
E03H4.7	5	3	2	4	2.80E-06	2.65E-06	1.82E-06	2.25E-06
E03H4.8	83	54	86	86	2.80E-06	2.65E-06	1.82E-06	2.25E-06
E03H4.9	4	7	8	4	2.80E-06	2.65E-06	1.82E-06	2.25E-06
E04A4.1	7	7	5	1	2.80E-06	2.65E-06	1.82E-06	2.25E-06
E04A4.2	4	4	3	2	6.33E-06	3.89E-06	4.26E-06	5.26E-06
E04A4.3	2	6	5	2	2.80E-06	2.65E-06	1.82E-06	2.25E-06
E04A4.4a	682	707	786	1062	2.80E-06	2.65E-06	1.82E-06	2.25E-06
E04A4.4b.1	668	685	805	1052	2.80E-06	2.65E-06	1.82E-06	2.25E-06
E04A4.4b.2	661	676	777	1042	2.80E-06	2.65E-06	1.82E-06	2.25E-06
E04A4.5	450	618	611	661	2.85E-05	2.79E-05	2.14E-05	3.57E-05
E04A4.6	9	14	11	6	2.96E-05	2.86E-05	2.32E-05	3.74E-05

E04A4.7.1	2311	3031	10029	2429	2.94E-05	2.84E-05	2.25E-05	3.72E-05
E04A4.7.2	1954	2640	7836	2138	5.63E-05	7.31E-05	4.98E-05	6.64E-05
E04A4.7.3	1935	2604	7698	2111	2.80E-06	2.65E-06	1.82E-06	2.25E-06
E04A4.7.4	1944	2625	7707	2123	5.15E-04	6.38E-04	1.45E-03	4.34E-04
E04A4.8.1	11552	8193	8918	9331	4.17E-04	5.32E-04	1.09E-03	3.66E-04
E04A4.8.2	10314	7246	8038	8876	4.48E-04	5.69E-04	1.16E-03	3.92E-04
E04D5.1a.1	1556	2073	1870	2435	4.37E-04	5.58E-04	1.13E-03	3.84E-04
E04D5.1a.2	1311	1712	1531	2060	1.79E-03	1.20E-03	8.99E-04	1.16E-03
E04D5.1b	342	423	379	528	1.90E-03	1.26E-03	9.64E-04	1.31E-03
E04D5.2	5	20	11	5	8.52E-05	1.07E-04	6.66E-05	1.07E-04
E04D5.3	65	103	53	75	8.42E-05	1.04E-04	6.40E-05	1.06E-04
E04D5.4	11	14	12	5	7.13E-05	8.33E-05	5.14E-05	8.85E-05
E04D5.5	33	48	44	33	2.80E-06	2.65E-06	1.82E-06	2.25E-06
E04F6.1	4	5	6	1	4.79E-06	7.14E-06	2.53E-06	4.43E-06
E04F6.10	11	8	8	7	2.80E-06	2.65E-06	1.82E-06	2.25E-06
E04F6.11a	188	210	261	355	5.29E-06	7.25E-06	4.57E-06	4.25E-06
E04F6.11b	114	127	150	205	2.80E-06	2.65E-06	1.82E-06	2.25E-06
E04F6.12	5	1	6	1	2.80E-06	2.65E-06	1.82E-06	2.25E-06
E04F6.13	4	4	4	1	5.99E-06	6.32E-06	5.41E-06	9.06E-06
E04F6.14	6	2	7	1	5.24E-06	5.53E-06	4.48E-06	7.58E-06
E04F6.15	43	111	41	45	2.80E-06	2.65E-06	1.82E-06	2.25E-06
E04F6.2	5	8	2	1	2.80E-06	2.65E-06	1.82E-06	2.25E-06
E04F6.3	264	562	183	316	2.80E-06	2.65E-06	1.82E-06	2.25E-06
E04F6.4	49	120	43	74	5.01E-06	1.22E-05	3.12E-06	4.23E-06
E04F6.5a	623	1030	549	920	2.80E-06	2.65E-06	1.82E-06	2.25E-06
E04F6.5b.1	542	907	480	790	2.88E-05	5.80E-05	1.30E-05	2.77E-05
E04F6.5b.2	371	621	356	628	2.80E-06	6.32E-06	1.82E-06	3.31E-06
E04F6.6	86	187	61	73	3.51E-05	5.48E-05	2.01E-05	4.16E-05
E04F6.7	28	46	39	21	3.51E-05	5.54E-05	2.02E-05	4.11E-05
E04F6.9	78	245	254	112	3.37E-05	5.32E-05	2.10E-05	4.58E-05
EEED8.10a	349	490	460	740	5.96E-06	1.22E-05	2.75E-06	4.07E-06
EEED8.11	11	7	4	9	2.80E-06	4.13E-06	2.41E-06	2.25E-06
EEED8.12	16	27	14	15	2.20E-05	6.53E-05	4.66E-05	2.54E-05
EEED8.13	16	4	9	22	1.65E-05	2.19E-05	1.41E-05	2.81E-05
EEED8.14	139	150	122	248	2.80E-06	2.65E-06	1.82E-06	2.25E-06
EEED8.15	62	45	48	68	2.83E-06	4.50E-06	1.82E-06	2.25E-06
EEED8.16	315	420	263	392	3.72E-06	2.65E-06	1.82E-06	4.09E-06
EEED8.2	24	29	17	19	1.30E-05	1.32E-05	7.42E-06	1.86E-05
EEED8.3	402	388	611	818	5.60E-06	3.84E-06	2.81E-06	4.93E-06
EEED8.4	21	25	12	14	1.81E-05	2.27E-05	9.80E-06	1.80E-05
EEED8.5	722	803	812	1325	2.91E-06	3.33E-06	1.82E-06	2.25E-06
EEED8.6	9	3	9	4	7.70E-05	7.02E-05	7.61E-05	1.26E-04
EEED8.7a	544	536	660	737	3.61E-06	4.07E-06	1.82E-06	2.25E-06
EEED8.7b	264	229	250	295	2.17E-05	2.28E-05	1.59E-05	3.20E-05
EEED8.8	52	79	41	49	2.80E-06	2.65E-06	1.82E-06	2.25E-06
EEED8.9	350	469	513	706	7.32E-05	6.82E-05	5.78E-05	7.97E-05
EGAP1.1	11	21	67	38	7.76E-05	6.36E-05	4.78E-05	6.97E-05
EGAP1.3	49	58	60	49	7.25E-06	1.04E-05	3.72E-06	5.49E-06
EGAP2.1	55	66	45	60	1.86E-05	2.35E-05	1.77E-05	3.01E-05
EGAP2.2	1	1	2	0	2.80E-06	2.72E-06	5.96E-06	4.18E-06
EGAP2.3.1	493	585	356	517	3.16E-06	3.54E-06	2.51E-06	2.54E-06
EGAP2.3.2	453	540	328	484	5.71E-06	6.48E-06	3.04E-06	5.02E-06
EGAP4.1	5	8	27	7	2.80E-06	2.65E-06	1.82E-06	2.25E-06
EGAP5.1	0	2	0	2	3.47E-05	3.90E-05	1.63E-05	2.93E-05
EGAP7.1	166	180	131	63	3.72E-05	4.19E-05	1.75E-05	3.19E-05
EGAP798.1	2	2	3	1	2.80E-06	2.65E-06	5.76E-06	2.25E-06
EGAP9.2	16	14	7	7	2.80E-06	2.65E-06	1.82E-06	2.25E-06
EGAP9.3	10	18	24	19	1.95E-05	2.00E-05	1.00E-05	5.94E-06
EGAP9.4	6	3	6	1	2.80E-06	2.65E-06	1.82E-06	2.25E-06

F01D4.1	30	16	39	14	2.80E-06	2.65E-06	1.82E-06	2.25E-06
F01D4.2	302	442	323	286	2.80E-06	2.65E-06	1.82E-06	2.25E-06
F01D4.3	22	30	18	14	2.80E-06	2.65E-06	1.82E-06	2.25E-06
F01D4.4.1	406	573	634	583	1.89E-05	2.62E-05	1.32E-05	1.44E-05
F01D4.4.2	387	543	600	558	2.80E-06	2.65E-06	1.82E-06	2.25E-06
F01D4.4.3	382	540	584	547	2.69E-05	3.59E-05	2.74E-05	3.11E-05
F01D4.5a	143	166	124	167	2.60E-05	3.45E-05	2.63E-05	3.02E-05
F01D4.5b	103	142	108	147	2.63E-05	3.52E-05	2.62E-05	3.03E-05
F01D4.6a	2	7	9	6	2.06E-05	2.25E-05	1.16E-05	1.93E-05
F01D4.6b	2	4	10	6	1.53E-05	1.99E-05	1.04E-05	1.75E-05
F01D4.7	5	4	10	4	2.80E-06	2.65E-06	1.82E-06	2.25E-06
F01D4.8	18	16	10	7	2.80E-06	2.65E-06	1.82E-06	2.25E-06
F01D4.9	8	3	19	6	2.80E-06	2.65E-06	1.82E-06	2.25E-06
F01D5.1	118	89	348	48	2.80E-06	2.65E-06	1.82E-06	2.25E-06
F01D5.10	23	28	9	10	2.80E-06	2.65E-06	1.82E-06	2.25E-06
F01D5.11	0	1	0	0	2.39E-05	1.70E-05	4.58E-05	7.80E-06
F01D5.2	24	14	66	5	2.80E-06	2.83E-06	1.82E-06	2.25E-06
F01D5.3	35	43	110	20	2.80E-06	2.65E-06	1.82E-06	2.25E-06
F01D5.4	58	58	167	57	6.69E-06	3.68E-06	1.20E-05	2.25E-06
F01D5.5	105	132	71	33	9.83E-06	1.14E-05	2.01E-05	4.52E-06
F01D5.6	87	112	42	28	1.07E-05	1.01E-05	2.01E-05	8.46E-06
F01D5.7a	4	11	35	5	2.24E-05	2.66E-05	9.86E-06	5.65E-06
F01D5.7b	4	11	36	5	7.48E-06	9.10E-06	2.35E-06	2.25E-06
F01D5.8	34	50	28	36	2.80E-06	2.65E-06	2.51E-06	2.25E-06
F01D5.9	97	109	76	79	2.80E-06	2.65E-06	2.55E-06	2.25E-06
F01E11.1	65	63	37	33	3.05E-06	4.23E-06	1.82E-06	2.59E-06
F01E11.3	18	21	13	17	6.97E-06	7.38E-06	3.55E-06	4.54E-06
F01E11.4	12	10	11	15	3.81E-06	3.49E-06	1.82E-06	2.25E-06
F01E11.5a	3	18	14	8	2.80E-06	2.65E-06	1.82E-06	2.25E-06
F01E11.5b	4	17	13	9	2.80E-06	2.65E-06	1.82E-06	2.25E-06
F01E11.5c	4	18	13	9	2.80E-06	2.65E-06	1.82E-06	2.25E-06
F01F1.10a	95	127	132	190	2.80E-06	2.65E-06	1.82E-06	2.25E-06
F01F1.10b	97	128	138	195	2.80E-06	2.65E-06	1.82E-06	2.25E-06
F01F1.10c	96	119	131	193	7.90E-06	9.97E-06	7.14E-06	1.27E-05
F01F1.11	310	367	335	492	8.09E-06	1.01E-05	7.49E-06	1.31E-05
F01F1.12b.1	3019	3430	4934	5144	8.40E-06	9.84E-06	7.45E-06	1.36E-05
F01F1.12b.2	2713	3146	4149	4602	1.88E-05	2.11E-05	1.32E-05	2.40E-05
F01F1.13	6	11	9	8	3.36E-04	3.60E-04	3.57E-04	4.60E-04
F01F1.14	6	13	15	5	3.79E-04	4.16E-04	3.78E-04	5.17E-04
F01F1.15.1	134	159	192	143	2.80E-06	2.65E-06	1.82E-06	2.25E-06
F01F1.15.2	112	136	140	112	2.80E-06	3.04E-06	2.42E-06	2.25E-06
F01F1.1a	143	196	196	247	1.87E-05	2.09E-05	1.74E-05	1.60E-05
F01F1.1b	36	35	36	46	1.97E-05	2.26E-05	1.60E-05	1.58E-05
F01F1.1c	127	163	165	212	6.94E-06	8.99E-06	6.20E-06	9.63E-06
F01F1.3	27	73	36	33	9.02E-06	8.28E-06	5.87E-06	9.27E-06
F01F1.4	339	351	468	572	7.34E-06	8.91E-06	6.21E-06	9.85E-06
F01F1.5.1	394	476	491	656	3.67E-06	9.36E-06	3.19E-06	3.60E-06
F01F1.5.2	356	445	453	622	2.70E-05	2.64E-05	2.43E-05	3.66E-05
F01F1.6.1	937	1019	1339	1253	2.17E-05	2.48E-05	1.76E-05	2.90E-05
F01F1.7	633	695	579	1022	2.09E-05	2.47E-05	1.73E-05	2.93E-05
F01F1.8a.1	2698	2559	2845	3808	5.40E-05	5.54E-05	5.02E-05	5.80E-05
F01F1.8a.2	2301	2139	2348	3217	3.11E-05	3.22E-05	1.85E-05	4.03E-05
F01F1.8b.1	2715	2553	2873	3745	1.59E-04	1.43E-04	1.09E-04	1.81E-04
F01F1.8b.2	1798	1701	1788	2382	1.59E-04	1.40E-04	1.06E-04	1.79E-04
F01F1.8b.3	1780	1692	1767	2365	1.60E-04	1.42E-04	1.10E-04	1.77E-04
F01F1.9	742	835	1109	1092	1.56E-04	1.39E-04	1.01E-04	1.65E-04
F01G10.1.1	3238	3445	5049	6026	1.54E-04	1.39E-04	9.98E-05	1.65E-04
F01G10.1.2	3187	3388	4917	5976	5.39E-05	5.73E-05	5.24E-05	6.37E-05
F01G10.10	68	160	19	38	1.83E-04	1.84E-04	1.86E-04	2.74E-04

F01G10.2	43	106	34	83	1.82E-04	1.83E-04	1.83E-04	2.74E-04
F01G10.3	22	35	21	18	4.65E-06	1.03E-05	1.82E-06	2.25E-06
F01G10.4	3	2	2	2	3.50E-06	8.17E-06	1.82E-06	5.44E-06
F01G10.5	54	63	31	31	2.80E-06	2.65E-06	1.82E-06	2.25E-06
F01G10.6	13	25	15	5	2.80E-06	2.65E-06	1.82E-06	2.25E-06
F01G10.7	69	159	87	145	2.80E-06	2.65E-06	1.82E-06	2.25E-06
F01G10.8	54	108	48	39	2.80E-06	3.15E-06	1.82E-06	2.25E-06
F01G10.9	108	93	55	41	6.02E-06	1.31E-05	4.96E-06	1.02E-05
F01G12.1	2	5	1	0	5.24E-06	9.89E-06	3.02E-06	3.04E-06
F01G12.2a	53	83	46	79	6.55E-06	5.32E-06	2.17E-06	2.25E-06
F01G12.2b	159	209	138	211	2.80E-06	2.65E-06	1.82E-06	2.25E-06
F01G12.5a	4421	5276	3963	6337	6.80E-06	1.01E-05	3.84E-06	8.14E-06
F01G12.5b.1	5172	6200	4480	7425	1.22E-05	1.52E-05	6.92E-06	1.30E-05
F01G12.5b.2	5034	6026	4405	7197	9.38E-05	1.06E-04	5.47E-05	1.08E-04
F01G12.6	176	179	87	187	1.02E-04	1.15E-04	5.73E-05	1.17E-04
F01G4.3	549	560	578	1025	9.96E-05	1.13E-04	5.67E-05	1.14E-04
F01G4.4	776	682	1197	1380	1.41E-05	1.35E-05	4.52E-06	1.20E-05
F01G4.5	87	171	106	123	1.59E-05	1.53E-05	1.09E-05	2.39E-05
F02A9.1	2	15	6	2	3.81E-05	3.16E-05	3.83E-05	5.44E-05
F02A9.2.1	3683	4824	6380	4947	1.09E-05	2.02E-05	8.62E-06	1.23E-05
F02A9.2.2	3380	4395	5339	4636	2.80E-06	2.65E-06	1.82E-06	2.25E-06
F02A9.3.1	10112	15658	15568	16274	6.25E-04	7.73E-04	7.05E-04	6.74E-04
F02A9.3.2	9200	14370	12890	15057	5.74E-04	7.06E-04	5.90E-04	6.33E-04
F02A9.4a	879	1245	795	1059	1.84E-03	2.69E-03	1.84E-03	2.37E-03
F02A9.4b	461	572	478	533	1.74E-03	2.56E-03	1.58E-03	2.28E-03
F02A9.6	1384	1225	2303	2938	4.66E-05	6.24E-05	2.74E-05	4.51E-05
F02A9.7	8	7	2	1	3.02E-05	3.53E-05	2.04E-05	2.80E-05
F02C12.1	70	105	52	82	3.66E-05	3.06E-05	3.96E-05	6.23E-05
F02C12.2	4	1	3	2	2.80E-06	2.65E-06	1.82E-06	2.25E-06
F02C12.3	3	10	9	4	3.05E-06	4.31E-06	1.82E-06	2.88E-06
F02C12.4	2	3	4	3	2.80E-06	2.65E-06	1.82E-06	2.25E-06
F02C12.5a	12	49	20	21	2.80E-06	2.65E-06	1.82E-06	2.25E-06
F02C12.5c	12	47	20	20	2.80E-06	2.65E-06	1.82E-06	2.25E-06
F02C9.1	4	4	6	1	2.80E-06	3.23E-06	1.82E-06	2.25E-06
F02C9.2	6	7	3	5	2.80E-06	3.07E-06	1.82E-06	2.25E-06
F02C9.3	10	11	11	7	2.80E-06	2.65E-06	1.82E-06	2.25E-06
F02C9.4	15	29	6	7	2.80E-06	2.65E-06	1.82E-06	2.25E-06
F02D10.1	58	70	37	35	2.80E-06	2.65E-06	1.82E-06	2.25E-06
F02D10.2	2	3	0	7	2.80E-06	5.03E-06	1.82E-06	2.25E-06
F02D10.3	4	2	2	1	6.83E-06	7.78E-06	2.84E-06	3.31E-06
F02D10.4	4	3	9	6	2.80E-06	2.65E-06	1.82E-06	4.12E-06
F02D10.5	49	39	41	64	2.80E-06	2.65E-06	1.82E-06	2.25E-06
F02D10.6	34	25	26	33	2.80E-06	2.65E-06	1.82E-06	2.25E-06
F02D10.7	6	5	2	3	2.80E-06	2.65E-06	1.82E-06	2.92E-06
F02D10.t2	3	2	3	2	6.36E-06	4.42E-06	3.15E-06	4.95E-06
F02D10.t3	0	1	0	0	2.80E-06	2.65E-06	1.82E-06	2.25E-06
F02D8.1	1	5	4	1	4.09E-06	2.65E-06	2.66E-06	2.25E-06
F02D8.2	17	19	9	3	2.80E-06	2.65E-06	1.82E-06	2.25E-06
F02D8.3	3	11	7	11	2.80E-06	2.65E-06	1.82E-06	2.25E-06
F02D8.5	14	14	7	17	2.80E-06	2.65E-06	1.82E-06	2.25E-06
F02E11.1	15	22	15	10	2.80E-06	2.65E-06	1.82E-06	2.25E-06
F02E11.2	75	79	182	102	5.80E-06	5.48E-06	1.90E-06	5.67E-06
F02E11.3	2	1	2	1	2.80E-06	2.65E-06	1.82E-06	2.25E-06
F02E11.4	0	1	7	0	5.43E-06	5.42E-06	8.58E-06	5.94E-06
F02E11.5	6	4	1	1	2.80E-06	2.65E-06	1.82E-06	2.25E-06
F02E8.1.1	1519	1731	2011	2374	2.80E-06	2.65E-06	1.82E-06	2.25E-06
F02E8.1.2	1424	1577	1907	2268	2.80E-06	2.65E-06	1.82E-06	2.25E-06
F02E8.2a	26	24	42	12	1.44E-04	1.55E-04	1.24E-04	1.80E-04
F02E8.2b	16	17	20	5	1.50E-04	1.57E-04	1.31E-04	1.92E-04

F02E8.3	97	171	106	66	2.80E-06	2.65E-06	1.82E-06	2.25E-06
F02E8.4	407	418	499	796	2.80E-06	2.65E-06	1.82E-06	2.25E-06
F02E8.5	67	122	59	81	1.89E-05	3.15E-05	1.35E-05	1.03E-05
F02E8.6	130	205	154	179	2.30E-05	2.24E-05	1.84E-05	3.62E-05
F02E9.1	144	140	85	143	3.84E-06	6.59E-06	2.20E-06	3.73E-06
F02E9.10a.1	422	399	620	801	3.16E-06	4.71E-06	2.44E-06	3.51E-06
F02E9.10a.2	399	373	560	757	3.47E-05	3.18E-05	1.33E-05	2.77E-05
F02E9.10b.1	338	337	451	642	3.44E-05	3.07E-05	3.29E-05	5.24E-05
F02E9.10b.2	348	346	507	668	3.28E-05	2.89E-05	2.99E-05	4.99E-05
F02E9.2a	116	109	553	233	3.00E-05	2.82E-05	2.60E-05	4.58E-05
F02E9.2b	19	37	117	98	3.30E-05	3.09E-05	3.12E-05	5.08E-05
F02E9.3	28	55	65	26	1.07E-05	9.52E-06	3.33E-05	1.73E-05
F02E9.7	230	279	234	410	2.88E-06	5.32E-06	1.16E-05	1.20E-05
F02E9.8	4	4	6	4	3.28E-06	6.06E-06	4.94E-06	2.43E-06
F02E9.9a.1	717	828	732	971	1.93E-05	2.21E-05	1.28E-05	2.76E-05
F02E9.9a.2	742	858	753	984	2.80E-06	2.65E-06	1.82E-06	2.25E-06
F02E9.9b.1	780	901	772	1018	3.69E-05	4.03E-05	2.45E-05	4.01E-05
F02E9.9b.2	742	858	753	984	3.96E-05	4.33E-05	2.62E-05	4.22E-05
F02G3.1a	82	132	99	137	3.76E-05	4.10E-05	2.42E-05	3.94E-05
F02G3.1b	84	133	96	135	3.82E-05	4.17E-05	2.52E-05	4.07E-05
F02G3.1c	73	106	94	121	2.86E-06	4.34E-06	2.24E-06	3.85E-06
F02H6.1	24	25	14	20	2.88E-06	4.31E-06	2.13E-06	3.71E-06
F02H6.2	145	143	145	226	2.80E-06	3.84E-06	2.33E-06	3.71E-06
F02H6.3a	30	32	16	27	2.80E-06	2.65E-06	1.82E-06	2.25E-06
F02H6.3b	49	59	42	58	1.75E-05	1.63E-05	1.14E-05	2.19E-05
F02H6.4	104	153	154	185	9.58E-06	9.65E-06	3.32E-06	6.93E-06
F02H6.5	51	65	38	42	1.03E-05	1.17E-05	5.74E-06	9.78E-06
F02H6.6	2	2	1	2	1.30E-05	1.81E-05	1.26E-05	1.86E-05
F02H6.7	4	6	12	11	3.50E-06	4.23E-06	1.82E-06	2.32E-06
F07A11.1	7	12	9	7	2.80E-06	2.65E-06	1.82E-06	2.25E-06
F07A11.2a	3033	2507	5184	5771	2.80E-06	2.65E-06	1.82E-06	2.25E-06
F07A11.2b.1	3233	2651	5693	6211	2.80E-06	2.65E-06	1.82E-06	2.25E-06
F07A11.2b.2	3189	2607	5460	6035	1.54E-04	1.20E-04	1.71E-04	2.36E-04
F07A11.2b.3	3065	2532	5254	5848	1.56E-04	1.21E-04	1.78E-04	2.40E-04
F07A11.3	560	550	857	1206	1.60E-04	1.24E-04	1.78E-04	2.43E-04
F07A11.4	227	237	172	202	1.59E-04	1.24E-04	1.77E-04	2.43E-04
F07A11.5	70	74	80	68	2.42E-05	2.25E-05	2.41E-05	4.19E-05
F07A11.6a	393	399	339	587	7.45E-06	7.35E-06	3.68E-06	5.33E-06
F07A11.6b	508	504	419	744	7.25E-06	7.25E-06	5.39E-06	5.65E-06
F07A11.6c	485	482	400	720	6.61E-06	6.35E-06	3.72E-06	7.94E-06
F07A11.6d	263	238	189	323	7.25E-06	6.80E-06	3.88E-06	8.52E-06
F07A11.6e	409	427	356	616	7.11E-06	6.69E-06	3.83E-06	8.50E-06
F07A5.1a	519	549	711	1154	9.66E-06	8.25E-06	4.52E-06	9.54E-06
F07A5.1b	566	607	797	1254	6.78E-06	6.69E-06	3.84E-06	8.21E-06
F07A5.2	106	313	107	103	4.17E-05	4.16E-05	3.72E-05	7.44E-05
F07A5.3	62	125	89	119	4.20E-05	4.26E-05	3.85E-05	7.48E-05
F07A5.4.1	4	19	7	10	1.26E-05	3.51E-05	8.25E-06	9.81E-06
F07A5.4.2	4	21	8	11	3.81E-06	7.25E-06	3.55E-06	5.87E-06
F07A5.5	13	18	2	20	2.80E-06	2.65E-06	1.82E-06	2.25E-06
F07A5.6	16	26	9	6	2.80E-06	2.72E-06	1.82E-06	2.25E-06
F07A5.7.1	10479	10763	8686	13760	2.80E-06	2.65E-06	1.82E-06	2.25E-06
F07A5.7.2	9538	9872	7492	12402	2.80E-06	2.86E-06	1.82E-06	2.25E-06
F07A5.7.3	9688	10223	7411	12460	3.45E-04	3.34E-04	1.86E-04	3.63E-04
F07B10.1	64	89	58	86	3.51E-04	3.43E-04	1.80E-04	3.67E-04
F07B10.2	8	4	2	4	3.61E-04	3.59E-04	1.80E-04	3.73E-04
F07B10.3	8	5	3	2	5.63E-06	7.38E-06	3.32E-06	6.07E-06
F07B10.4	10	16	20	9	2.80E-06	2.65E-06	1.82E-06	2.25E-06
F07B10.5	6	6	7	2	2.80E-06	2.65E-06	1.82E-06	2.25E-06
F07B10.6	5	5	1	3	2.80E-06	2.65E-06	1.82E-06	2.25E-06

F07B7.1	6	3	5	8	2.80E-06	2.65E-06	1.82E-06	2.25E-06
F07B7.10	41	76	29	11	2.80E-06	2.65E-06	1.82E-06	2.25E-06
F07B7.11	21	63	30	13	2.80E-06	2.65E-06	1.82E-06	2.25E-06
F07B7.12	99	105	108	200	1.20E-05	2.09E-05	5.50E-06	2.59E-06
F07B7.13	7	10	4	5	5.52E-06	1.57E-05	5.14E-06	2.74E-06
F07B7.14	2	7	8	4	2.80E-06	2.65E-06	1.82E-06	3.01E-06
F07B7.2	8	54	7	30	2.80E-06	2.65E-06	1.82E-06	2.25E-06
F07B7.3	41	76	29	11	2.80E-06	2.65E-06	1.82E-06	2.25E-06
F07B7.4	21	63	30	13	2.80E-06	6.00E-06	1.82E-06	2.83E-06
F07B7.5	27	31	34	22	1.20E-05	2.09E-05	5.50E-06	2.59E-06
F07B7.7	1	3	3	1	5.52E-06	1.57E-05	5.14E-06	2.74E-06
F07B7.8	5	5	4	0	7.36E-06	7.99E-06	6.03E-06	4.81E-06
F07B7.9	9	19	34	10	2.80E-06	2.65E-06	1.82E-06	2.25E-06
F07C3.1	16	27	18	17	2.80E-06	2.65E-06	1.82E-06	2.25E-06
F07C3.10	13	18	22	14	3.22E-06	6.45E-06	7.94E-06	2.88E-06
F07C3.2	27	60	26	16	2.80E-06	2.65E-06	1.82E-06	2.25E-06
F07C3.3	19	15	29	16	2.80E-06	2.65E-06	1.82E-06	2.25E-06
F07C3.4	198	306	217	359	5.49E-06	1.15E-05	3.43E-06	2.61E-06
F07C3.7	85	110	91	127	2.80E-06	2.65E-06	1.82E-06	2.25E-06
F07C3.8	4	6	0	1	4.87E-06	7.14E-06	3.48E-06	7.11E-06
F07C3.9	8	28	34	19	5.52E-06	6.74E-06	3.84E-06	6.61E-06
F07C4.1	4	4	2	5	2.80E-06	2.65E-06	1.82E-06	2.25E-06
F07C4.10	2	5	2	1	5.54E-06	1.83E-05	1.53E-05	1.05E-05
F07C4.11	1	2	2	2	2.80E-06	2.65E-06	1.82E-06	2.25E-06
F07C4.12a	10	6	7	3	2.80E-06	2.65E-06	1.82E-06	2.25E-06
F07C4.12b	12	8	10	4	2.80E-06	2.65E-06	1.82E-06	2.25E-06
F07C4.13	5	10	14	6	2.80E-06	2.65E-06	1.82E-06	2.25E-06
F07C4.14	3	5	8	5	2.80E-06	2.65E-06	1.82E-06	2.25E-06
F07C4.2	3	2	0	3	2.80E-06	2.65E-06	1.82E-06	2.25E-06
F07C4.3	3	3	1	4	2.80E-06	2.65E-06	1.82E-06	2.25E-06
F07C4.4	3	5	9	4	2.80E-06	2.65E-06	1.82E-06	2.25E-06
F07C4.5	2	4	4	1	2.80E-06	2.65E-06	1.82E-06	2.25E-06
F07C4.6	7	9	6	2	2.80E-06	2.65E-06	1.82E-06	2.25E-06
F07C4.7	70	118	117	46	2.80E-06	2.65E-06	1.82E-06	2.25E-06
F07C4.8	8	2	5	0	2.80E-06	2.65E-06	1.82E-06	2.25E-06
F07C4.9	0	3	0	1	1.44E-05	2.29E-05	1.56E-05	7.58E-06
F07C6.1	26	37	15	14	2.80E-06	2.65E-06	1.82E-06	2.25E-06
F07C6.2	16	20	33	9	2.80E-06	2.65E-06	1.82E-06	2.25E-06
F07C6.3	38	46	53	16	2.94E-06	3.94E-06	1.82E-06	2.25E-06
F07C6.4a	188	165	209	277	3.56E-06	4.21E-06	4.77E-06	2.25E-06
F07C6.4c.1	251	315	302	493	6.10E-06	6.98E-06	5.56E-06	2.25E-06
F07C6.4c.2	277	336	327	528	1.04E-05	8.62E-06	7.53E-06	1.23E-05
F07C6.5	1	1	2	0	1.60E-05	1.90E-05	1.26E-05	2.53E-05
F07D10.1.1	2323	3636	2723	2429	1.67E-05	1.92E-05	1.28E-05	2.56E-05
F07D10.1.2	2133	3238	2291	2230	2.80E-06	2.65E-06	1.82E-06	2.25E-06
F07D3.2	85	171	124	155	3.64E-04	5.38E-04	2.78E-04	3.06E-04
F07D3.3	14	15	4	4	3.69E-04	5.29E-04	2.58E-04	3.10E-04
F07E5.1	6	12	22	18	1.51E-05	2.86E-05	1.43E-05	2.21E-05
F07E5.10	0	2	4	1	2.80E-06	2.65E-06	1.82E-06	2.25E-06
F07E5.2	8	2	7	7	2.80E-06	2.65E-06	1.82E-06	2.25E-06
F07E5.4	2	6	1	1	2.80E-06	2.65E-06	1.82E-06	2.25E-06
F07E5.5	302	204	257	344	2.80E-06	2.65E-06	1.82E-06	2.25E-06
F07E5.6	25	23	27	11	2.80E-06	2.65E-06	1.82E-06	2.25E-06
F07E5.7	9	3	9	4	2.72E-05	1.73E-05	1.50E-05	2.48E-05
F07E5.8	14	28	17	15	2.80E-06	2.65E-06	1.82E-06	2.25E-06
F07E5.9	12	8	6	5	2.80E-06	2.65E-06	1.82E-06	2.25E-06
F07F6.1	21	80	35	25	2.80E-06	2.65E-06	1.82E-06	2.25E-06
F07F6.2	12	17	13	8	2.80E-06	2.65E-06	1.82E-06	2.25E-06
F07F6.4.1	405	400	656	879	2.80E-06	5.66E-06	1.82E-06	2.25E-06

F07F6.4.2	377	374	620	862	2.80E-06	2.65E-06	1.82E-06	2.25E-06
F07F6.5	27	60	6	12	2.84E-05	2.65E-05	2.99E-05	4.95E-05
F07F6.6	14	23	18	14	2.65E-05	2.48E-05	2.83E-05	4.86E-05
F07F6.7	23	16	21	19	8.06E-06	1.69E-05	1.82E-06	2.88E-06
F07F6.9	3	6	2	0	2.80E-06	2.65E-06	1.82E-06	2.25E-06
F07G11.1	6	5	4	6	3.22E-06	2.65E-06	1.91E-06	2.25E-06
F07G11.2	2	4	5	3	2.80E-06	2.65E-06	1.82E-06	2.25E-06
F07G11.3	4	12	5	2	2.80E-06	2.65E-06	1.82E-06	2.25E-06
F07G11.4	2	9	4	3	2.80E-06	2.65E-06	1.82E-06	2.25E-06
F07G11.5	10	7	3	2	2.80E-06	2.65E-06	1.82E-06	2.25E-06
F07G11.6	2	0	4	5	2.80E-06	2.65E-06	1.82E-06	2.25E-06
F07G11.7	8	7	7	4	2.80E-06	2.65E-06	1.82E-06	2.25E-06
F07G11.8	9	9	1	3	2.80E-06	2.65E-06	1.82E-06	2.25E-06
F07G11.9	27	36	26	22	2.80E-06	2.65E-06	1.82E-06	2.25E-06
F07G6.1	6	10	14	16	2.80E-06	2.65E-06	1.82E-06	2.25E-06
F07G6.2	5	5	3	3	2.80E-06	2.65E-06	1.82E-06	2.25E-06
F07G6.3	3	8	14	6	2.80E-06	2.65E-06	1.82E-06	2.25E-06
F07G6.5	1	0	0	0	2.80E-06	2.65E-06	1.82E-06	2.25E-06
F07G6.6	3	5	13	5	2.80E-06	2.65E-06	1.82E-06	2.25E-06
F07G6.7	5	15	13	9	2.80E-06	2.65E-06	1.82E-06	2.25E-06
F07G6.9	2	1	0	1	2.80E-06	2.65E-06	1.82E-06	2.25E-06
F07H5.10	173	227	230	242	2.80E-06	2.65E-06	1.82E-06	2.25E-06
F07H5.13	31	29	10	33	2.80E-06	2.65E-06	1.82E-06	2.25E-06
F07H5.2	17	68	20	20	1.70E-05	2.11E-05	1.47E-05	1.91E-05
F07H5.3a	432	689	422	392	8.60E-06	7.59E-06	1.82E-06	7.35E-06
F07H5.3b	437	690	430	402	2.80E-06	6.56E-06	1.82E-06	2.25E-06
F07H5.4	185	227	128	166	7.43E-05	1.12E-04	4.72E-05	5.42E-05
F07H5.5a	3215	6406	5199	6657	7.22E-05	1.08E-04	4.62E-05	5.33E-05
F07H5.5b	3079	6218	4882	6296	3.37E-05	3.90E-05	1.52E-05	2.43E-05
F07H5.6	3	1	3	2	2.36E-04	4.44E-04	2.48E-04	3.92E-04
F07H5.7	3	2	6	3	2.18E-04	4.16E-04	2.25E-04	3.58E-04
F07H5.8	55	66	18	34	2.80E-06	2.65E-06	1.82E-06	2.25E-06
F08A10.1a	26	38	15	15	2.80E-06	2.65E-06	1.82E-06	2.25E-06
F08A10.1b	26	36	15	16	2.80E-06	3.04E-06	1.82E-06	2.25E-06
F08A10.1c	26	41	16	21	2.80E-06	2.65E-06	1.82E-06	2.25E-06
F08A10.1d	28	45	17	21	2.80E-06	2.65E-06	1.82E-06	2.25E-06
F08A10.2	6	8	16	6	2.80E-06	2.65E-06	1.82E-06	2.25E-06
F08A8.1a.1	278	360	230	367	2.80E-06	2.65E-06	1.82E-06	2.25E-06
F08A8.1a.2	268	349	216	363	2.80E-06	2.65E-06	1.82E-06	2.25E-06
F08A8.1b.1	258	336	204	344	1.47E-05	1.79E-05	7.89E-06	1.55E-05
F08A8.1b.2	245	293	179	317	1.48E-05	1.82E-05	7.76E-06	1.61E-05
F08A8.1b.3	265	348	210	354	1.51E-05	1.86E-05	7.78E-06	1.62E-05
F08A8.1b.4	268	349	216	363	1.58E-05	1.78E-05	7.49E-06	1.64E-05
F08A8.1c.1	265	348	210	354	1.46E-05	1.82E-05	7.56E-06	1.57E-05
F08A8.1c.2	268	349	216	363	1.48E-05	1.82E-05	7.74E-06	1.61E-05
F08A8.2	320	374	172	200	1.46E-05	1.82E-05	7.56E-06	1.57E-05
F08A8.3	196	195	150	174	1.48E-05	1.82E-05	7.74E-06	1.61E-05
F08A8.4	257	317	171	217	1.71E-05	1.88E-05	5.98E-06	8.57E-06
F08A8.6	5	4	6	4	1.11E-05	1.04E-05	5.52E-06	7.89E-06
F08A8.7	203	215	223	267	1.32E-05	1.53E-05	5.70E-06	8.93E-06
F08B1.1a.1	384	592	408	688	2.80E-06	2.65E-06	1.82E-06	2.25E-06
F08B1.1a.2	343	513	381	608	2.13E-05	2.13E-05	1.52E-05	2.25E-05
F08B1.1a.3	261	431	279	505	1.69E-05	2.46E-05	1.17E-05	2.43E-05
F08B1.1b	215	360	243	453	1.67E-05	2.35E-05	1.20E-05	2.37E-05
F08B1.1c.1	209	338	231	420	1.30E-05	2.02E-05	9.02E-06	2.02E-05
F08B1.1c.2	225	389	244	441	1.32E-05	2.09E-05	9.73E-06	2.24E-05
F08B1.1c.3	191	327	217	405	1.41E-05	2.16E-05	1.02E-05	2.29E-05
F08B1.2	116	265	246	407	1.41E-05	2.30E-05	9.95E-06	2.22E-05
F08B1.3	24	44	59	114	1.55E-05	2.52E-05	1.15E-05	2.65E-05

F08B12.1	134	250	76	132	3.39E-06	7.30E-06	4.66E-06	9.54E-06
F08B12.2	33	53	32	43	2.80E-06	3.36E-06	3.12E-06	7.42E-06
F08B12.3a	98	171	73	140	5.04E-06	8.89E-06	1.86E-06	4.00E-06
F08B12.3c	101	171	70	141	3.28E-06	4.97E-06	2.08E-06	3.44E-06
F08B12.4.1	271	579	470	313	3.00E-06	4.97E-06	1.82E-06	3.46E-06
F08B12.4.2	167	373	308	247	3.42E-06	5.48E-06	1.82E-06	3.85E-06
F08B12.5	0	3	0	0	6.21E-05	1.25E-04	7.01E-05	5.76E-05
F08B4.1a	313	317	425	667	4.40E-05	9.29E-05	5.28E-05	5.23E-05
F08B4.1b	381	369	492	763	2.80E-06	2.65E-06	1.82E-06	2.25E-06
F08B4.2a	116	246	111	84	1.39E-05	1.33E-05	1.23E-05	2.38E-05
F08B4.2b	65	106	64	67	1.49E-05	1.36E-05	1.25E-05	2.39E-05
F08B4.3	6	9	10	6	2.80E-06	5.61E-06	1.82E-06	2.25E-06
F08B4.4	2	2	1	3	2.80E-06	2.67E-06	1.82E-06	2.25E-06
F08B4.5	289	374	410	521	4.31E-06	6.11E-06	4.66E-06	3.46E-06
F08B4.6	350	341	517	676	2.80E-06	2.65E-06	1.82E-06	2.25E-06
F08B4.7.1	284	316	466	267	2.02E-05	2.47E-05	1.86E-05	2.92E-05
F08B4.7.2	178	207	384	195	1.43E-05	1.31E-05	1.37E-05	2.21E-05
F08B6.1	54	80	137	84	5.01E-05	5.27E-05	5.35E-05	3.78E-05
F08B6.3	45	69	37	40	3.13E-05	3.44E-05	4.40E-05	2.76E-05
F08B6.4a	2190	2787	2268	3124	1.82E-05	2.54E-05	3.00E-05	2.27E-05
F08B6.4b	2700	3542	3004	3810	5.57E-06	8.07E-06	2.97E-06	3.98E-06
F08B6.4c.1	2537	3303	2752	3705	1.44E-04	1.74E-04	9.73E-05	1.65E-04
F08B6.4c.2	2190	2787	2268	3124	2.27E-04	2.81E-04	1.64E-04	2.57E-04
F08B6.4c.3	2325	2965	2583	3274	1.21E-04	1.49E-04	8.57E-05	1.42E-04
F08C6.1a.1	202	395	152	340	1.44E-04	1.74E-04	9.73E-05	1.65E-04
F08C6.1a.2	151	341	112	283	1.55E-04	1.87E-04	1.12E-04	1.75E-04
F08C6.1b	26	62	10	35	5.82E-06	1.08E-05	2.86E-06	7.89E-06
F08C6.1c	144	317	108	272	5.35E-06	1.14E-05	2.59E-06	8.05E-06
F08C6.2a	239	418	154	280	5.43E-06	1.22E-05	1.82E-06	5.87E-06
F08C6.2b	198	351	135	243	5.32E-06	1.11E-05	2.61E-06	8.07E-06
F08C6.5	5	6	3	5	1.61E-05	2.66E-05	6.76E-06	1.52E-05
F08C6.6	400	570	382	273	2.07E-05	3.47E-05	9.20E-06	2.04E-05
F08C6.7	69	117	64	90	2.80E-06	2.65E-06	1.82E-06	2.25E-06
F08D12.1	590	287	811	939	3.80E-05	5.12E-05	2.36E-05	2.08E-05
F08D12.10	21	10	10	11	5.94E-06	9.50E-06	3.59E-06	6.21E-06
F08D12.11	14	10	8	4	2.34E-05	1.08E-05	2.09E-05	2.99E-05
F08D12.12.1	10	11	12	10	2.80E-06	2.65E-06	1.82E-06	2.25E-06
F08D12.12.2	10	8	11	7	2.80E-06	2.65E-06	1.82E-06	2.25E-06
F08D12.13	10	4	21	24	2.80E-06	2.65E-06	1.82E-06	2.25E-06
F08D12.2	16	21	19	17	2.80E-06	2.65E-06	1.82E-06	2.25E-06
F08D12.3a	23	23	41	25	2.80E-06	2.65E-06	1.97E-06	2.77E-06
F08D12.3b	20	19	40	22	2.80E-06	3.44E-06	2.15E-06	2.36E-06
F08D12.4	1	1	2	2	2.80E-06	2.65E-06	3.26E-06	2.45E-06
F08D12.6	7	8	10	1	2.80E-06	2.65E-06	2.41E-06	2.25E-06
F08D12.7	16	48	40	38	2.80E-06	2.65E-06	1.82E-06	2.25E-06
F08D12.8	5	4	10	7	2.80E-06	2.65E-06	1.82E-06	2.25E-06
F08D12.9	10	6	2	5	2.80E-06	5.03E-06	2.88E-06	3.37E-06
F08E10.1	3	3	3	1	2.80E-06	2.65E-06	1.82E-06	2.25E-06
F08E10.2	6	4	0	0	2.80E-06	2.65E-06	1.82E-06	2.25E-06
F08E10.3	1	2	6	0	2.80E-06	2.65E-06	1.82E-06	2.25E-06
F08E10.4	5	2	2	4	2.80E-06	2.65E-06	1.82E-06	2.25E-06
F08E10.5	0	5	2	0	2.80E-06	2.65E-06	1.82E-06	2.25E-06
F08E10.6	3	3	0	2	2.80E-06	2.65E-06	1.82E-06	2.25E-06
F08E10.7	2	14	5	2	2.80E-06	2.65E-06	1.82E-06	2.25E-06
F08E10.8	9	14	6	12	2.80E-06	2.65E-06	1.82E-06	2.25E-06
F08F1.1a	14	13	8	14	2.80E-06	2.65E-06	1.82E-06	2.25E-06
F08F1.1b	17	16	10	15	2.80E-06	2.65E-06	1.82E-06	2.25E-06
F08F1.3	16	22	23	18	2.80E-06	2.65E-06	1.82E-06	2.25E-06
F08F1.4a	56	85	57	68	2.80E-06	2.65E-06	1.82E-06	2.25E-06

F08F1.4b.1	57	73	50	60	2.80E-06	3.41E-06	2.46E-06	2.38E-06
F08F1.4b.2	51	74	32	55	9.88E-06	1.42E-05	6.54E-06	9.63E-06
F08F1.5	30	32	30	40	1.01E-05	1.23E-05	5.79E-06	8.59E-06
F08F1.6	79	147	39	11	8.23E-06	1.13E-05	3.35E-06	7.13E-06
F08F1.7	505	757	533	694	2.80E-06	2.65E-06	1.82E-06	2.61E-06
F08F1.8.1	112	227	151	205	2.34E-05	4.12E-05	7.53E-06	2.61E-06
F08F1.8.2	81	175	94	146	2.33E-05	3.30E-05	1.60E-05	2.58E-05
F08F1.9	9	5	3	3	1.71E-05	3.27E-05	1.50E-05	2.51E-05
F08F1.t1	0	0	1	0	1.55E-05	3.17E-05	1.17E-05	2.24E-05
F08F1.t2	0	0	1	0	2.80E-06	2.65E-06	1.82E-06	2.25E-06
F08F3.1	10	17	10	8	2.80E-06	2.65E-06	1.82E-06	2.25E-06
F08F3.10	2	2	2	1	2.80E-06	2.65E-06	1.82E-06	2.25E-06
F08F3.2a.1	464	539	672	962	3.70E-06	5.92E-06	2.41E-06	2.38E-06
F08F3.2a.2	448	517	652	937	2.80E-06	2.65E-06	1.82E-06	2.25E-06
F08F3.2b	349	376	476	686	2.27E-05	2.49E-05	2.14E-05	3.77E-05
F08F3.3.1	934	1773	1589	2504	2.30E-05	2.51E-05	2.18E-05	3.86E-05
F08F3.3.2	899	1699	1469	2432	2.27E-05	2.31E-05	2.01E-05	3.58E-05
F08F3.4	185	322	169	375	6.45E-05	1.16E-04	7.14E-05	1.39E-04
F08F3.6	251	245	392	351	6.69E-05	1.19E-04	7.11E-05	1.45E-04
F08F3.7	101	166	83	238	1.78E-05	2.93E-05	1.06E-05	2.90E-05
F08F3.8	8	18	12	11	2.32E-05	2.14E-05	2.36E-05	2.61E-05
F08F3.9a	14	19	17	18	7.22E-06	1.12E-05	3.86E-06	1.37E-05
F08F3.9b	15	20	18	18	2.80E-06	2.65E-06	1.82E-06	2.25E-06
F08F8.1	29	91	14	46	2.80E-06	2.65E-06	1.82E-06	2.25E-06
F08F8.10a	285	293	323	419	2.80E-06	2.65E-06	1.82E-06	2.25E-06
F08F8.10b	280	293	322	417	3.08E-06	9.10E-06	1.82E-06	3.91E-06
F08F8.2	305	398	343	497	2.03E-05	1.97E-05	1.50E-05	2.40E-05
F08F8.3a	30	28	28	36	1.93E-05	1.91E-05	1.44E-05	2.31E-05
F08F8.3b	23	27	24	33	1.78E-05	2.20E-05	1.30E-05	2.33E-05
F08F8.4	249	316	255	311	2.80E-06	2.65E-06	1.82E-06	2.25E-06
F08F8.5	15	90	8	32	2.80E-06	2.65E-06	1.82E-06	2.25E-06
F08F8.6	10	22	4	3	2.49E-05	2.98E-05	1.66E-05	2.50E-05
F08F8.7.1	193	251	150	120	2.80E-06	1.13E-05	1.82E-06	3.40E-06
F08F8.7.3	184	229	127	108	2.80E-06	2.72E-06	1.82E-06	2.25E-06
F08F8.8	133	166	188	203	2.73E-05	3.36E-05	1.38E-05	1.37E-05
F08F8.9a	552	609	506	843	2.98E-05	3.51E-05	1.34E-05	1.41E-05
F08F8.9b	537	587	493	828	1.43E-05	1.68E-05	1.31E-05	1.75E-05
F08F8.9c.1	528	557	478	786	3.07E-05	3.20E-05	1.83E-05	3.76E-05
F08F8.9c.2	472	503	435	732	3.10E-05	3.20E-05	1.85E-05	3.83E-05
F08G12.1	66	90	44	101	3.09E-05	3.08E-05	1.82E-05	3.70E-05
F08G12.10	161	143	182	252	3.36E-05	3.39E-05	2.02E-05	4.19E-05
F08G12.11	3	1	2	1	3.47E-06	4.47E-06	1.82E-06	4.27E-06
F08G12.2	130	184	188	184	1.22E-05	1.02E-05	8.95E-06	1.53E-05
F08G12.3	30	35	41	55	2.80E-06	2.65E-06	1.82E-06	2.25E-06
F08G12.4	13	47	38	33	1.24E-05	1.66E-05	1.16E-05	1.41E-05
F08G12.5	6	23	9	7	2.80E-06	2.65E-06	2.04E-06	3.40E-06
F08G12.8	4	4	11	1	2.80E-06	9.44E-06	5.25E-06	5.62E-06
F08G2.1	35	39	62	17	2.80E-06	4.36E-06	1.82E-06	2.25E-06
F08G2.10	0	3	1	1	2.80E-06	2.65E-06	2.00E-06	2.25E-06
F08G2.11	0	3	1	1	1.06E-05	1.12E-05	1.22E-05	4.14E-06
F08G2.2	17	23	47	17	2.80E-06	2.65E-06	1.82E-06	2.25E-06
F08G2.3	16	16	10	9	2.80E-06	2.65E-06	1.82E-06	2.25E-06
F08G2.4	33	40	50	36	4.96E-06	6.35E-06	8.93E-06	3.98E-06
F08G2.5	6	9	26	7	4.37E-06	4.13E-06	1.82E-06	2.25E-06
F08G2.6	7	9	8	4	5.94E-06	6.80E-06	5.85E-06	5.20E-06
F08G2.7	44	33	55	50	2.80E-06	2.65E-06	3.53E-06	2.25E-06
F08G2.8	5	12	20	9	2.80E-06	2.65E-06	1.82E-06	2.25E-06
F08G2.9	0	3	1	1	3.30E-06	2.65E-06	2.68E-06	3.01E-06
F08G5.1	326	319	549	601	2.80E-06	2.65E-06	2.02E-06	2.25E-06

F08G5.2	25	33	17	11	2.80E-06	2.65E-06	1.82E-06	2.25E-06
F08G5.3a.1	28	56	21	17	3.25E-05	3.01E-05	3.57E-05	4.82E-05
F08G5.3a.2	22	45	16	13	2.80E-06	3.20E-06	1.82E-06	2.25E-06
F08G5.3b	25	45	17	12	4.76E-06	8.97E-06	2.31E-06	2.32E-06
F08G5.4	466	484	236	181	3.86E-06	7.49E-06	1.84E-06	2.25E-06
F08G5.5.1	69	70	52	32	4.14E-06	7.04E-06	1.82E-06	2.25E-06
F08G5.5.2	66	61	48	32	5.44E-05	5.34E-05	1.79E-05	1.70E-05
F08G5.6	274	1024	371	573	4.76E-06	4.58E-06	2.33E-06	2.25E-06
F08G5.7	17	16	12	9	4.59E-06	4.02E-06	2.17E-06	2.25E-06
F08H9.1	193	204	268	423	2.91E-05	1.03E-04	2.56E-05	4.88E-05
F08H9.10	0	1	6	1	2.80E-06	2.65E-06	1.82E-06	2.25E-06
F08H9.11	0	1	6	1	1.18E-05	1.18E-05	1.07E-05	2.09E-05
F08H9.12	7	12	7	4	2.80E-06	2.65E-06	2.64E-06	2.25E-06
F08H9.2	62	75	61	35	2.80E-06	2.65E-06	2.64E-06	2.25E-06
F08H9.3	3	6	5	2	2.80E-06	2.65E-06	1.82E-06	2.25E-06
F08H9.4	1	6	11	6	1.60E-05	1.83E-05	1.02E-05	7.24E-06
F08H9.5	28	10	41	39	2.80E-06	2.65E-06	1.82E-06	2.25E-06
F08H9.7	266	199	294	399	2.80E-06	2.65E-06	1.82E-06	2.25E-06
F08H9.8	58	71	87	77	3.00E-06	2.65E-06	2.86E-06	3.35E-06
F08H9.9	5	11	11	11	2.47E-05	1.74E-05	1.77E-05	2.97E-05
F09A5.1	22	31	21	10	5.66E-06	6.56E-06	5.54E-06	6.05E-06
F09A5.2	26	37	22	15	2.80E-06	2.65E-06	1.82E-06	2.25E-06
F09A5.3	5	9	7	2	2.80E-06	2.72E-06	1.82E-06	2.25E-06
F09A5.4a	132	229	99	181	2.80E-06	2.65E-06	1.82E-06	2.25E-06
F09A5.4b.1	63	114	56	101	2.80E-06	2.65E-06	1.82E-06	2.25E-06
F09A5.4b.2	65	120	58	103	9.60E-06	1.57E-05	4.68E-06	1.06E-05
F09A5.4c	56	93	54	84	6.58E-06	1.12E-05	3.81E-06	8.48E-06
F09A5.4d	51	90	50	83	6.78E-06	1.18E-05	3.94E-06	8.64E-06
F09A5.4e	52	91	51	84	5.88E-06	9.23E-06	3.68E-06	7.08E-06
F09B12.1a	199	295	66	58	6.05E-06	1.01E-05	3.86E-06	7.89E-06
F09B12.1b.1	237	388	87	68	5.94E-06	9.79E-06	3.79E-06	7.69E-06
F09B12.1b.2	206	304	67	60	1.29E-05	1.81E-05	2.79E-06	3.04E-06
F09B12.2	61	64	60	65	1.19E-05	1.84E-05	2.84E-06	2.74E-06
F09B12.3	712	776	772	553	1.34E-05	1.87E-05	2.84E-06	3.15E-06
F09B12.5	11	13	12	10	7.03E-06	6.96E-06	4.50E-06	6.00E-06
F09B12.6	27	37	26	33	4.32E-05	4.45E-05	3.05E-05	2.69E-05
F09B9.1	54	203	52	46	2.80E-06	2.65E-06	1.82E-06	2.25E-06
F09B9.2a	654	909	694	1079	2.80E-06	2.65E-06	1.82E-06	2.25E-06
F09B9.2b	644	890	682	1069	2.80E-06	9.63E-06	1.82E-06	2.25E-06
F09B9.3	442	593	329	380	2.45E-05	3.21E-05	1.69E-05	3.24E-05
F09B9.4	127	205	101	144	3.76E-05	4.90E-05	2.59E-05	5.01E-05
F09B9.5	8	12	16	6	3.19E-05	4.04E-05	1.54E-05	2.20E-05
F09C11.1	12	20	37	16	1.78E-05	2.71E-05	9.22E-06	1.62E-05
F09C12.1	7	8	8	8	2.80E-06	2.65E-06	1.90E-06	2.25E-06
F09C12.2	5	1	7	3	2.94E-06	4.63E-06	5.92E-06	3.15E-06
F09C12.6	7	12	10	4	2.80E-06	2.65E-06	1.82E-06	2.25E-06
F09C12.7	104	163	420	62	2.80E-06	2.65E-06	1.82E-06	2.25E-06
F09C12.8	33	59	22	11	2.80E-06	2.65E-06	1.82E-06	2.25E-06
F09C3.1	182	254	95	204	4.98E-05	7.37E-05	1.31E-04	2.38E-05
F09C3.2	23	52	19	32	3.92E-06	6.64E-06	1.82E-06	2.25E-06
F09C3.3	8	16	13	9	4.79E-06	6.30E-06	1.82E-06	4.30E-06
F09C3.4	5	3	2	3	2.80E-06	5.92E-06	1.82E-06	3.10E-06
F09C3.5	3	4	6	3	2.80E-06	2.65E-06	1.82E-06	2.25E-06
F09C6.1	2	2	0	0	2.80E-06	2.65E-06	1.82E-06	2.25E-06
F09C6.10	4	5	2	4	2.80E-06	2.65E-06	1.82E-06	2.25E-06
F09C6.11	1	0	2	1	2.80E-06	2.65E-06	1.82E-06	2.25E-06
F09C6.12	1	3	1	1	2.80E-06	2.65E-06	1.82E-06	2.25E-06
F09C6.13	0	6	0	0	2.80E-06	2.65E-06	1.82E-06	2.25E-06
F09C6.14	1	1	1	0	2.80E-06	2.65E-06	1.82E-06	2.25E-06

F09C6.2	4	4	7	6	2.80E-06	2.72E-06	1.82E-06	2.25E-06
F09C6.3	1	8	2	2	2.80E-06	2.65E-06	1.82E-06	2.25E-06
F09C6.4	4	7	4	6	2.80E-06	2.65E-06	1.82E-06	2.25E-06
F09C6.5	5	7	1	2	2.80E-06	2.65E-06	1.82E-06	2.25E-06
F09C6.6	4	4	8	4	2.80E-06	2.65E-06	1.82E-06	2.25E-06
F09C6.7	5	4	4	1	2.80E-06	2.65E-06	1.82E-06	2.25E-06
F09C6.8	6	10	15	5	2.80E-06	2.65E-06	1.82E-06	2.25E-06
F09C6.9	5	14	6	8	2.80E-06	2.65E-06	1.82E-06	2.25E-06
F09C8.1	174	213	251	391	2.80E-06	2.65E-06	1.82E-06	2.25E-06
F09C8.2.1	295	237	335	417	2.80E-06	2.65E-06	1.82E-06	2.25E-06
F09C8.2.2	154	143	192	261	1.60E-05	1.85E-05	1.50E-05	2.89E-05
F09D1.1	285	329	322	480	1.02E-05	7.75E-06	7.54E-06	1.16E-05
F09D12.1	121	116	110	50	7.25E-06	6.37E-06	5.89E-06	9.87E-06
F09D12.2	8	3	7	4	1.67E-05	1.83E-05	1.23E-05	2.26E-05
F09E10.1	28	43	131	40	4.38E-05	3.97E-05	2.59E-05	1.46E-05
F09E10.10	1	6	6	5	2.80E-06	2.65E-06	1.82E-06	2.25E-06
F09E10.3	214	245	220	251	1.15E-05	1.67E-05	3.50E-05	1.32E-05
F09E10.5	5	4	10	1	2.80E-06	2.65E-06	1.82E-06	2.25E-06
F09E10.6	14	8	10	13	2.52E-05	2.73E-05	1.69E-05	2.38E-05
F09E10.7a.1	14	15	15	5	2.80E-06	2.65E-06	1.82E-06	2.25E-06
F09E10.7a.2	3	5	4	1	3.00E-06	2.65E-06	1.82E-06	2.25E-06
F09E10.7b	4	7	6	1	2.80E-06	2.72E-06	1.88E-06	2.25E-06
F09E10.8a	145	216	152	212	2.80E-06	2.65E-06	1.82E-06	2.25E-06
F09E5.1.1	572	741	806	1029	2.80E-06	2.65E-06	1.82E-06	2.25E-06
F09E5.1.2	600	761	854	1050	8.68E-06	1.22E-05	5.92E-06	1.02E-05
F09E5.10	191	255	255	262	2.74E-05	3.36E-05	2.52E-05	3.97E-05
F09E5.11	178	201	133	193	3.74E-05	4.49E-05	3.47E-05	5.26E-05
F09E5.12	6	6	5	8	1.22E-05	1.53E-05	1.06E-05	1.34E-05
F09E5.13	260	220	235	312	3.04E-05	3.24E-05	1.48E-05	2.64E-05
F09E5.14	35	41	49	42	2.80E-06	2.65E-06	1.82E-06	2.25E-06
F09E5.15.1	4695	4384	5452	4267	3.53E-05	2.82E-05	2.08E-05	3.40E-05
F09E5.15.2	4696	4379	5453	4252	8.68E-06	9.63E-06	7.93E-06	8.39E-06
F09E5.16	6	9	7	2	7.17E-04	6.33E-04	5.42E-04	5.24E-04
F09E5.17	192	221	292	204	7.71E-04	6.79E-04	5.83E-04	5.61E-04
F09E5.2	214	309	215	281	2.80E-06	3.15E-06	1.82E-06	2.25E-06
F09E5.3.1	608	703	510	563	3.83E-05	4.17E-05	3.79E-05	3.27E-05
F09E5.3.2	522	607	385	476	1.83E-05	2.50E-05	1.20E-05	1.93E-05
F09E5.4	8	6	1	4	6.82E-05	7.45E-05	3.72E-05	5.07E-05
F09E5.5.1	357	461	403	552	6.36E-05	6.99E-05	3.05E-05	4.66E-05
F09E5.5.2	352	437	418	549	2.80E-06	2.65E-06	1.82E-06	2.25E-06
F09E5.7.1	315	416	330	456	1.57E-05	1.92E-05	1.16E-05	1.95E-05
F09E5.7.2	223	324	207	337	1.64E-05	1.93E-05	1.27E-05	2.06E-05
F09E5.8	141	187	122	181	2.49E-05	3.10E-05	1.69E-05	2.89E-05
F09E5.9	114	133	176	114	2.64E-05	3.62E-05	1.59E-05	3.20E-05
F09E8.1	3	2	3	2	2.08E-05	2.61E-05	1.17E-05	2.15E-05
F09E8.3	338	395	529	746	2.03E-05	2.24E-05	2.04E-05	1.63E-05
F09E8.5	5	3	3	4	2.80E-06	2.65E-06	1.82E-06	2.25E-06
F09E8.6	45	54	16	29	9.10E-06	1.01E-05	9.27E-06	1.61E-05
F09E8.7	40	45	27	38	2.80E-06	2.65E-06	1.82E-06	2.25E-06
F09E8.8	11	9	16	3	3.33E-06	3.78E-06	1.82E-06	2.25E-06
F09F3.1	3	9	5	3	2.80E-06	2.65E-06	1.82E-06	2.25E-06
F09F3.10	5	6	4	8	5.63E-06	4.34E-06	5.32E-06	2.25E-06
F09F3.11	2	3	2	3	2.80E-06	2.65E-06	1.82E-06	2.25E-06
F09F3.12	6	1	7	3	2.80E-06	2.65E-06	1.82E-06	2.25E-06
F09F3.13	0	3	3	1	2.80E-06	2.65E-06	1.82E-06	2.25E-06
F09F3.2	4	7	10	0	2.80E-06	2.65E-06	1.82E-06	2.25E-06
F09F3.3	5	9	10	9	2.80E-06	2.65E-06	1.82E-06	2.25E-06
F09F3.4	10	14	4	3	2.80E-06	2.65E-06	1.82E-06	2.25E-06
F09F3.5	10	3	7	1	2.80E-06	2.65E-06	1.82E-06	2.25E-06

F09F3.6	50	91	67	23	2.80E-06	2.65E-06	1.82E-06	2.25E-06
F09F3.7	5	6	18	10	2.80E-06	2.65E-06	1.82E-06	2.25E-06
F09F3.8	16	29	22	11	1.06E-05	1.82E-05	9.24E-06	3.91E-06
F09F3.9.1	84	154	72	138	2.80E-06	2.65E-06	1.82E-06	2.25E-06
F09F3.9.2	79	148	68	130	2.80E-06	2.65E-06	1.82E-06	2.25E-06
F09F7.1	5	8	5	9	4.09E-06	7.09E-06	2.28E-06	5.42E-06
F09F7.2b	589	737	1085	728	4.00E-06	7.06E-06	2.24E-06	5.29E-06
F09F7.3.1	1263	1196	1222	2299	2.80E-06	2.65E-06	1.82E-06	2.25E-06
F09F7.4a.1	812	1348	878	2235	7.73E-05	9.14E-05	9.27E-05	7.68E-05
F09F7.4a.2	674	1116	668	1837	3.89E-05	3.48E-05	2.45E-05	5.69E-05
F09F7.4b.1	674	1116	668	1837	6.46E-05	1.01E-04	4.54E-05	1.43E-04
F09F7.4b.2	660	1084	642	1803	6.48E-05	1.01E-04	4.18E-05	1.42E-04
F09F7.4b.3	674	1119	672	1833	6.48E-05	1.01E-04	4.18E-05	1.42E-04
F09F7.5a	81	93	42	75	6.68E-05	1.04E-04	4.23E-05	1.47E-04
F09F7.5b	80	95	48	76	6.08E-05	9.54E-05	3.95E-05	1.33E-04
F09F7.6	17	38	32	21	5.12E-06	5.55E-06	1.82E-06	3.80E-06
F09F7.7a	91	136	118	136	4.73E-06	5.29E-06	1.84E-06	3.60E-06
F09F7.7b	69	97	89	103	7.22E-06	1.52E-05	8.84E-06	7.15E-06
F09F7.8	14	15	17	5	1.07E-05	1.51E-05	9.04E-06	1.29E-05
F09F9.1	24	26	24	21	1.20E-05	1.59E-05	1.01E-05	1.44E-05
F09F9.2	91	125	46	20	4.28E-06	4.34E-06	3.39E-06	2.25E-06
F09F9.3	3	2	6	2	2.80E-06	2.67E-06	1.82E-06	2.25E-06
F09F9.4	23	26	12	34	2.05E-05	2.66E-05	6.72E-06	3.62E-06
F09G2.1	102	160	75	118	2.80E-06	2.65E-06	1.82E-06	2.25E-06
F09G2.2	851	707	1047	1220	2.80E-06	2.65E-06	1.82E-06	2.25E-06
F09G2.3	30	75	19	51	3.78E-06	5.61E-06	1.82E-06	3.51E-06
F09G2.4	407	455	422	745	8.43E-05	6.61E-05	6.75E-05	9.70E-05
F09G2.5	27	49	19	33	2.80E-06	4.23E-06	1.82E-06	2.45E-06
F09G2.6	14	17	6	11	1.76E-05	1.85E-05	1.18E-05	2.58E-05
F09G2.7	2	2	2	1	2.80E-06	2.91E-06	1.82E-06	2.25E-06
F09G2.8.1	370	446	483	642	2.80E-06	2.65E-06	1.82E-06	2.25E-06
F09G2.8.2	360	439	468	624	2.80E-06	2.65E-06	1.82E-06	2.25E-06
F09G2.9.1	1769	1637	1989	2646	2.52E-05	2.87E-05	2.14E-05	3.52E-05
F09G2.9.2	1032	1002	1185	1489	2.59E-05	2.99E-05	2.19E-05	3.61E-05
F09G8.10	13	21	15	15	1.07E-04	9.38E-05	7.85E-05	1.29E-04
F09G8.2	36	54	32	57	9.25E-05	8.48E-05	6.91E-05	1.07E-04
F09G8.3	369	330	384	485	2.80E-06	2.65E-06	1.82E-06	2.25E-06
F09G8.4	45	76	41	41	3.64E-06	5.18E-06	2.11E-06	4.66E-06
F09G8.5	33	37	21	31	3.26E-05	2.76E-05	2.21E-05	3.44E-05
F09G8.6	254	249	127	89	2.80E-06	2.65E-06	1.82E-06	2.25E-06
F09G8.7	206	239	148	128	2.80E-06	2.70E-06	1.82E-06	2.25E-06
F09G8.8	90	151	83	91	2.96E-05	2.74E-05	9.64E-06	8.32E-06
F09G8.9	0	5	3	1	7.61E-05	8.35E-05	3.56E-05	3.80E-05
F10A3.1	2	3	5	1	5.15E-06	8.17E-06	3.10E-06	4.18E-06
F10A3.10	9	9	9	10	2.80E-06	2.65E-06	1.82E-06	2.25E-06
F10A3.11	0	2	1	2	2.80E-06	2.65E-06	1.82E-06	2.25E-06
F10A3.12	2	6	3	3	2.80E-06	2.65E-06	1.82E-06	2.25E-06
F10A3.13	9	5	4	0	2.80E-06	2.65E-06	1.82E-06	2.25E-06
F10A3.15	3	4	8	6	2.80E-06	2.65E-06	1.82E-06	2.25E-06
F10A3.16	3	7	17	3	2.80E-06	2.65E-06	1.82E-06	2.25E-06
F10A3.2	3	9	17	7	2.80E-06	2.65E-06	1.82E-06	2.25E-06
F10A3.3	3	6	3	0	2.80E-06	2.65E-06	1.82E-06	2.25E-06
F10A3.4	105	36	85	44	2.80E-06	2.65E-06	1.82E-06	2.25E-06
F10A3.5	9	6	7	2	2.80E-06	2.65E-06	1.82E-06	2.25E-06
F10A3.6	5	6	13	8	6.64E-06	2.65E-06	3.50E-06	2.25E-06
F10A3.7	3	1	7	2	2.80E-06	2.65E-06	1.82E-06	2.25E-06
F10A3.8	9	3	6	4	2.80E-06	2.65E-06	1.82E-06	2.25E-06
F10A3.9	6	2	4	2	2.80E-06	2.65E-06	1.82E-06	2.25E-06
F10B5.1.1	9910	10326	14700	8082	2.80E-06	2.65E-06	1.82E-06	2.25E-06

F10B5.1.2	8808	9422	10643	7352	2.80E-06	2.65E-06	1.82E-06	2.25E-06
F10B5.2	244	206	283	492	1.58E-03	1.55E-03	1.52E-03	1.03E-03
F10B5.4	11	8	43	14	1.52E-03	1.54E-03	1.20E-03	1.02E-03
F10B5.5	238	272	326	443	2.55E-05	2.03E-05	1.92E-05	4.12E-05
F10B5.6.1	456	511	805	1058	2.80E-06	2.65E-06	1.82E-06	2.25E-06
F10B5.6.2	446	476	768	1027	2.08E-05	2.24E-05	1.85E-05	3.11E-05
F10B5.7	478	514	665	942	2.51E-05	2.65E-05	2.88E-05	4.67E-05
F10B5.8	574	661	966	1061	2.54E-05	2.56E-05	2.84E-05	4.69E-05
F10B5.9	0	5	3	1	9.94E-06	1.01E-05	9.00E-06	1.57E-05
F10C1.1	0	0	3	1	3.23E-05	3.51E-05	3.53E-05	4.79E-05
F10C1.2a.1	1545	2118	1262	2434	2.80E-06	2.65E-06	1.82E-06	2.25E-06
F10C1.2a.2	1526	2095	1236	2418	2.80E-06	2.65E-06	1.82E-06	2.25E-06
F10C1.2b	2040	2843	1561	3114	9.90E-05	1.28E-04	5.27E-05	1.25E-04
F10C1.3	20	23	17	4	1.01E-04	1.31E-04	5.32E-05	1.28E-04
F10C1.5	9	11	18	17	1.02E-04	1.34E-04	5.08E-05	1.25E-04
F10C1.7a	1341	1286	1398	1487	6.22E-06	6.74E-06	3.43E-06	2.25E-06
F10C1.7c.1	1369	1329	1413	1497	2.80E-06	2.65E-06	1.82E-06	2.25E-06
F10C1.7c.2	1321	1262	1365	1481	8.34E-05	7.55E-05	5.66E-05	7.43E-05
F10C1.8b	5	3	4	5	8.74E-05	8.01E-05	5.87E-05	7.67E-05
F10C1.9	68	118	44	90	8.53E-05	7.70E-05	5.74E-05	7.68E-05
F10C1.t1	0	0	4	0	2.80E-06	2.65E-06	1.82E-06	2.25E-06
F10C2.2	374	481	583	652	1.08E-05	1.76E-05	4.54E-06	1.14E-05
F10C2.3	18	43	20	28	2.80E-06	2.65E-06	4.10E-06	2.25E-06
F10C2.4	956	866	1456	1975	3.39E-05	4.12E-05	3.44E-05	4.75E-05
F10C2.5	187	256	184	264	2.80E-06	2.65E-06	1.82E-06	2.25E-06
F10C2.6	213	242	232	312	3.18E-05	2.72E-05	3.15E-05	5.28E-05
F10C2.7	6	12	9	12	8.26E-06	1.07E-05	5.30E-06	9.38E-06
F10D11.1.1	443	630	519	422	1.29E-05	1.38E-05	9.11E-06	1.51E-05
F10D11.1.2	413	611	442	415	2.80E-06	2.65E-06	1.82E-06	2.25E-06
F10D11.2	364	389	469	695	6.34E-05	8.53E-05	4.84E-05	4.86E-05
F10D11.3	13	17	8	4	5.74E-05	8.02E-05	4.00E-05	4.63E-05
F10D11.4	16	18	9	7	2.73E-05	2.75E-05	2.29E-05	4.19E-05
F10D11.5	23	30	35	19	3.78E-06	4.68E-06	1.82E-06	2.25E-06
F10D11.6	445	820	176	369	3.53E-06	3.76E-06	1.82E-06	2.25E-06
F10D2.1	4	10	5	16	2.80E-06	2.65E-06	1.82E-06	2.25E-06
F10D2.10	168	813	138	967	1.91E-05	3.32E-05	4.90E-06	1.27E-05
F10D2.11	107	246	112	165	2.80E-06	2.65E-06	1.82E-06	2.25E-06
F10D2.12	6	9	10	8	1.85E-05	8.47E-05	9.89E-06	8.56E-05
F10D2.2a	49	133	55	140	6.80E-06	1.48E-05	4.63E-06	8.41E-06
F10D2.2b	45	129	46	133	2.80E-06	2.65E-06	1.82E-06	2.25E-06
F10D2.3	3	4	12	3	3.05E-06	7.83E-06	2.22E-06	6.99E-06
F10D2.4	13	6	15	4	3.05E-06	8.28E-06	2.04E-06	7.26E-06
F10D2.5	16	19	27	20	2.80E-06	2.65E-06	1.82E-06	2.25E-06
F10D2.6	29	46	31	36	2.80E-06	2.65E-06	1.82E-06	2.25E-06
F10D2.7	18	72	28	61	2.80E-06	2.65E-06	1.82E-06	2.25E-06
F10D2.8	2	3	5	3	2.80E-06	2.83E-06	1.82E-06	2.25E-06
F10D2.9	127	813	65	985	2.80E-06	4.84E-06	1.82E-06	3.49E-06
F10D7.1	21	36	34	18	2.80E-06	2.65E-06	1.82E-06	2.25E-06
F10D7.2.1	70	118	76	88	1.38E-05	8.33E-05	4.59E-06	8.59E-05
F10D7.2.2	61	104	61	81	2.80E-06	2.65E-06	1.82E-06	2.25E-06
F10D7.3	99	114	51	42	5.18E-06	8.23E-06	3.66E-06	5.22E-06
F10D7.4	10	8	12	7	5.10E-06	8.20E-06	3.32E-06	5.44E-06
F10D7.5a	164	193	179	276	1.15E-05	1.25E-05	3.86E-06	3.91E-06
F10D7.5b	35	48	49	57	2.80E-06	2.65E-06	1.82E-06	2.25E-06
F10D7.5c	66	90	76	108	7.73E-06	8.60E-06	5.48E-06	1.04E-05
F10D7.5e	32	44	46	54	6.05E-06	7.83E-06	5.50E-06	7.89E-06
F10E7.1	10	16	8	13	5.63E-06	7.25E-06	4.21E-06	7.40E-06
F10E7.10	10	22	11	4	6.36E-06	8.25E-06	5.94E-06	8.59E-06
F10E7.11	337	260	466	531	2.80E-06	2.65E-06	1.82E-06	2.25E-06

F10E7.2	293	232	333	202	2.80E-06	2.65E-06	1.82E-06	2.25E-06
F10E7.3	11	9	5	6	3.75E-05	2.74E-05	3.38E-05	4.75E-05
F10E7.4	157	284	155	323	4.54E-05	3.40E-05	3.36E-05	2.51E-05
F10E7.5	743	646	692	937	2.80E-06	2.65E-06	1.82E-06	2.25E-06
F10E7.6	133	174	232	127	6.83E-06	1.17E-05	4.39E-06	1.13E-05
F10E7.7.1	8249	5991	20772	4675	1.05E-04	8.59E-05	6.34E-05	1.06E-04
F10E7.7.2	8209	5944	20744	4626	3.23E-05	3.98E-05	3.66E-05	2.47E-05
F10E7.8	1300	1417	1542	2163	1.56E-03	1.07E-03	2.55E-03	7.09E-04
F10E7.9	28	46	54	50	2.08E-03	1.43E-03	3.43E-03	9.44E-04
F10E9.1	7	18	5	6	4.77E-05	4.91E-05	3.68E-05	6.37E-05
F10E9.10	6	7	3	2	2.80E-06	2.65E-06	2.13E-06	2.43E-06
F10E9.11	371	225	464	216	2.80E-06	3.20E-06	1.82E-06	2.25E-06
F10E9.12	25	69	37	18	2.80E-06	2.65E-06	1.82E-06	2.25E-06
F10E9.2	20	21	12	10	1.24E-04	7.09E-05	1.01E-04	5.78E-05
F10E9.4	328	303	221	247	5.40E-06	1.41E-05	5.21E-06	3.13E-06
F10E9.5	148	125	121	161	2.80E-06	2.65E-06	1.82E-06	2.25E-06
F10E9.6a.1	186	255	145	268	4.66E-05	4.06E-05	2.04E-05	2.82E-05
F10E9.6a.2	186	255	145	268	3.21E-05	2.56E-05	1.71E-05	2.81E-05
F10E9.6b	184	248	142	268	1.03E-05	1.34E-05	5.23E-06	1.19E-05
F10E9.6c	225	311	192	320	1.03E-05	1.34E-05	5.25E-06	1.20E-05
F10E9.7	180	212	240	224	1.04E-05	1.33E-05	5.25E-06	1.22E-05
F10E9.8	455	385	701	857	9.74E-06	1.27E-05	5.41E-06	1.12E-05
F10F2.1	1760	1714	2199	2812	4.07E-05	4.53E-05	3.53E-05	4.07E-05
F10F2.2	227	364	219	410	1.95E-05	1.56E-05	1.95E-05	2.95E-05
F10F2.3	44	105	18	17	2.52E-05	2.32E-05	2.05E-05	3.23E-05
F10F2.4	12	37	24	22	6.24E-06	9.47E-06	3.92E-06	9.06E-06
F10F2.5	17	29	9	3	5.46E-06	1.23E-05	1.82E-06	2.25E-06
F10F2.6	28	36	12	15	2.80E-06	2.65E-06	1.82E-06	2.25E-06
F10F2.7	47	90	28	20	2.80E-06	2.65E-06	1.82E-06	2.25E-06
F10F2.8	34	48	24	14	2.80E-06	2.65E-06	1.82E-06	2.25E-06
F10F2.9	22	34	18	8	2.80E-06	5.05E-06	1.82E-06	2.25E-06
F10G2.1	168	322	193	167	2.80E-06	2.91E-06	1.82E-06	2.25E-06
F10G2.2	5	3	6	4	2.80E-06	2.65E-06	1.82E-06	2.25E-06
F10G2.3	34	65	29	49	2.77E-05	5.01E-05	2.07E-05	2.21E-05
F10G2.4	4	14	8	4	2.80E-06	2.65E-06	1.82E-06	2.25E-06
F10G2.6a.1	4	3	5	1	2.80E-06	4.73E-06	1.82E-06	3.04E-06
F10G2.6a.2	6	3	9	1	2.80E-06	2.80E-06	1.82E-06	2.25E-06
F10G2.6b	7	6	10	1	2.80E-06	2.65E-06	1.82E-06	2.25E-06
F10G2.7	8	7	11	3	2.80E-06	2.65E-06	1.82E-06	2.25E-06
F10G2.8	5	3	8	4	2.80E-06	2.65E-06	1.82E-06	2.25E-06
F10G2.9	0	4	3	2	2.80E-06	2.65E-06	1.82E-06	2.25E-06
F10G7.1.1	710	856	1030	1427	2.80E-06	2.65E-06	1.82E-06	2.25E-06
F10G7.1.2	693	826	1027	1405	2.80E-06	2.65E-06	1.82E-06	2.25E-06
F10G7.10a	459	475	581	700	3.12E-05	3.55E-05	2.94E-05	5.03E-05
F10G7.10b	420	437	545	638	3.29E-05	3.71E-05	3.17E-05	5.36E-05
F10G7.10c	411	422	540	629	8.29E-06	8.12E-06	6.83E-06	1.02E-05
F10G7.11	578	531	961	295	8.15E-06	8.01E-06	6.89E-06	9.94E-06
F10G7.12	7	10	6	0	8.40E-06	8.15E-06	7.20E-06	1.03E-05
F10G7.2.1	6482	4688	5899	7993	1.35E-04	1.17E-04	1.46E-04	5.54E-05
F10G7.2.2	6413	4651	5810	7961	2.80E-06	3.76E-06	1.82E-06	2.25E-06
F10G7.4	399	422	706	680	2.30E-04	1.57E-04	1.36E-04	2.27E-04
F10G7.5a	83	110	61	97	2.28E-04	1.56E-04	1.34E-04	2.27E-04
F10G7.5b	92	127	75	117	2.10E-05	2.09E-05	2.41E-05	2.87E-05
F10G7.6	4	3	9	0	6.72E-06	8.44E-06	3.23E-06	6.32E-06
F10G7.7	3	9	3	2	6.41E-06	8.38E-06	3.41E-06	6.57E-06
F10G7.8.1	1455	1350	2255	2628	2.80E-06	2.65E-06	1.82E-06	2.25E-06
F10G7.8.2	1341	1257	1983	2424	2.80E-06	2.65E-06	1.82E-06	2.25E-06
F10G7.9a	425	472	457	722	9.53E-05	8.35E-05	9.61E-05	1.38E-04
F10G7.9b.1	451	503	493	754	1.02E-04	9.02E-05	9.80E-05	1.48E-04

F10G7.9b.2	472	521	503	781	2.18E-05	2.29E-05	1.53E-05	2.97E-05
F10G8.1	30	50	14	15	2.03E-05	2.14E-05	1.44E-05	2.72E-05
F10G8.2	10	15	6	4	2.14E-05	2.23E-05	1.48E-05	2.84E-05
F10G8.3.1	500	584	978	893	3.30E-06	5.21E-06	1.82E-06	2.25E-06
F10G8.3.2	410	460	673	688	2.80E-06	2.65E-06	1.82E-06	2.25E-06
F10G8.4	8	9	20	9	3.85E-05	4.25E-05	4.90E-05	5.52E-05
F10G8.5	887	1182	967	1294	4.09E-05	4.33E-05	4.37E-05	5.51E-05
F10G8.6	305	368	343	432	2.80E-06	2.65E-06	1.82E-06	2.25E-06
F10G8.7	163	169	232	186	7.31E-05	9.19E-05	5.18E-05	8.56E-05
F10G8.9a	43	65	40	40	3.11E-05	3.54E-05	2.27E-05	3.53E-05
F10G8.9b	71	93	75	65	2.28E-05	2.23E-05	2.11E-05	2.09E-05
F11A1.2	11	11	4	7	6.08E-06	8.68E-06	3.68E-06	4.54E-06
F11A1.3a	186	235	177	297	6.47E-06	8.01E-06	4.45E-06	4.77E-06
F11A1.3b	121	150	143	189	2.80E-06	2.65E-06	1.82E-06	2.25E-06
F11A1.3c	32	37	45	75	5.66E-06	6.74E-06	3.50E-06	7.26E-06
F11A1.3d	121	150	143	189	5.40E-06	6.32E-06	4.15E-06	6.77E-06
F11A10.2	204	260	247	258	4.45E-06	4.87E-06	4.08E-06	8.39E-06
F11A10.3	537	412	653	834	5.40E-06	6.32E-06	4.15E-06	6.77E-06
F11A10.4	1179	1026	1711	2182	3.02E-05	3.63E-05	2.38E-05	3.07E-05
F11A10.5	494	621	793	802	3.35E-05	2.43E-05	2.65E-05	4.17E-05
F11A10.6	178	258	193	208	2.50E-05	2.06E-05	2.36E-05	3.71E-05
F11A10.7	413	429	277	388	2.94E-05	3.49E-05	3.07E-05	3.83E-05
F11A10.8	429	405	728	814	1.50E-05	2.06E-05	1.06E-05	1.41E-05
F11A3.1.1	325	631	310	448	3.85E-05	3.78E-05	1.68E-05	2.91E-05
F11A3.1.2	311	600	288	435	4.97E-05	4.44E-05	5.49E-05	7.58E-05
F11A3.2a	355	338	685	755	2.11E-05	3.86E-05	1.31E-05	2.33E-05
F11A3.2b	262	275	500	646	2.13E-05	3.88E-05	1.28E-05	2.39E-05
F11A3.3	7	6	6	14	1.93E-05	1.74E-05	2.43E-05	3.30E-05
F11A5.1	2	2	4	0	1.91E-05	1.89E-05	2.37E-05	3.78E-05
F11A5.10	59	66	64	45	2.80E-06	2.65E-06	1.82E-06	2.25E-06
F11A5.11	6	2	5	5	2.80E-06	2.65E-06	1.82E-06	2.25E-06
F11A5.12	11	15	9	14	4.20E-06	4.44E-06	2.97E-06	2.56E-06
F11A5.13	3	5	2	2	2.80E-06	2.65E-06	1.82E-06	2.25E-06
F11A5.14	0	3	0	0	2.80E-06	2.65E-06	1.82E-06	2.25E-06
F11A5.15	6	3	6	4	2.80E-06	2.65E-06	1.82E-06	2.25E-06
F11A5.16	4	6	0	0	2.80E-06	2.65E-06	1.82E-06	2.25E-06
F11A5.2	3	5	3	0	2.80E-06	2.65E-06	1.82E-06	2.25E-06
F11A5.3	1	1	6	2	2.80E-06	2.65E-06	1.82E-06	2.25E-06
F11A5.4	4	2	4	4	2.80E-06	2.65E-06	1.82E-06	2.25E-06
F11A5.7	0	4	6	5	2.80E-06	2.65E-06	1.82E-06	2.25E-06
F11A5.8	10	7	11	5	2.80E-06	2.65E-06	1.82E-06	2.25E-06
F11A5.9.1	91	264	109	277	2.80E-06	2.65E-06	1.82E-06	2.25E-06
F11A5.9.2	85	217	108	261	2.80E-06	2.65E-06	1.82E-06	2.25E-06
F11A6.1b	125	255	113	184	5.32E-06	1.46E-05	4.14E-06	1.30E-05
F11A6.2	33	53	40	49	5.85E-06	1.42E-05	4.85E-06	1.45E-05
F11C1.1	74	78	109	44	5.15E-06	9.92E-06	3.02E-06	6.09E-06
F11C1.2	37	51	34	48	4.62E-06	7.04E-06	3.66E-06	5.53E-06
F11C1.3	106	106	81	112	1.38E-05	1.38E-05	1.33E-05	6.61E-06
F11C1.4	2	6	7	1	5.66E-06	7.38E-06	3.39E-06	5.91E-06
F11C1.5a	514	802	474	1001	6.50E-06	6.14E-06	3.23E-06	5.51E-06
F11C1.6a.1	81	84	67	108	2.80E-06	2.65E-06	1.82E-06	2.25E-06
F11C1.6a.2	48	32	38	58	1.08E-05	1.58E-05	6.45E-06	1.68E-05
F11C1.6b.1	46	29	37	53	3.53E-06	3.47E-06	1.90E-06	3.78E-06
F11C1.6b.2	48	32	38	58	3.11E-06	2.65E-06	1.82E-06	3.01E-06
F11C1.6b.3	79	82	66	102	3.25E-06	2.65E-06	1.82E-06	2.99E-06
F11C3.1	9	10	9	15	3.11E-06	2.65E-06	1.82E-06	3.01E-06
F11C3.2	23	24	17	27	3.75E-06	3.70E-06	2.04E-06	3.91E-06
F11C3.3.1	20409	21000	16083	27550	2.80E-06	2.65E-06	1.82E-06	2.25E-06
F11C3.3.2	19898	20554	15811	27137	2.80E-06	2.65E-06	1.82E-06	2.25E-06

F11C7.1	7	10	7	12	3.36E-04	3.26E-04	1.72E-04	3.64E-04
F11C7.2	20	16	25	8	3.60E-04	3.51E-04	1.86E-04	3.94E-04
F11C7.3a	39	68	64	48	2.80E-06	2.65E-06	1.82E-06	2.25E-06
F11C7.3b	43	72	69	52	4.76E-06	3.60E-06	3.86E-06	2.25E-06
F11C7.4	66	63	35	69	3.42E-06	5.63E-06	3.64E-06	3.37E-06
F11C7.5	987	1417	1951	1894	3.25E-06	5.16E-06	3.41E-06	3.17E-06
F11C7.6a	3	8	8	2	2.80E-06	2.65E-06	1.82E-06	2.25E-06
F11C7.6b	3	8	8	2	9.65E-05	1.31E-04	1.24E-04	1.49E-04
F11C7.7	1	5	2	2	2.80E-06	2.65E-06	1.82E-06	2.25E-06
F11D11.1	2	2	1	3	2.80E-06	2.65E-06	1.82E-06	2.25E-06
F11D11.3	3	8	9	3	2.80E-06	2.65E-06	1.82E-06	2.25E-06
F11D11.4	1	5	5	1	2.80E-06	2.65E-06	1.82E-06	2.25E-06
F11D11.5	5	3	2	2	2.80E-06	2.65E-06	1.82E-06	2.25E-06
F11D11.6	5	4	4	0	2.80E-06	2.65E-06	1.82E-06	2.25E-06
F11D11.7	2	3	6	4	2.80E-06	2.65E-06	1.82E-06	2.25E-06
F11D11.8	7	1	2	1	2.80E-06	2.65E-06	1.82E-06	2.25E-06
F11D5.1a	203	175	93	140	2.80E-06	2.65E-06	1.82E-06	2.25E-06
F11D5.1b	58	64	31	46	2.80E-06	2.65E-06	1.82E-06	2.25E-06
F11D5.1c	180	130	81	118	8.06E-06	6.56E-06	2.41E-06	4.48E-06
F11D5.3a	32	56	31	52	6.52E-06	6.80E-06	2.26E-06	4.16E-06
F11D5.3b.1	27	49	24	46	8.74E-06	5.95E-06	2.55E-06	4.59E-06
F11D5.3b.2	26	49	23	46	2.80E-06	2.65E-06	1.82E-06	2.25E-06
F11D5.3b.3	28	50	26	48	2.80E-06	2.65E-06	1.82E-06	2.25E-06
F11D5.5	7	4	14	3	2.80E-06	2.65E-06	1.82E-06	2.25E-06
F11D5.6	1	3	4	0	2.80E-06	2.65E-06	1.82E-06	2.25E-06
F11D5.7	16	7	26	15	2.80E-06	2.65E-06	1.82E-06	2.25E-06
F11E6.10	2	6	7	1	2.80E-06	2.65E-06	1.82E-06	2.25E-06
F11E6.11.1	23	32	11	19	2.80E-06	2.65E-06	1.82E-06	2.25E-06
F11E6.11.2	20	27	11	16	2.80E-06	2.65E-06	1.82E-06	2.25E-06
F11E6.1a	59	74	59	89	4.14E-06	5.45E-06	1.82E-06	2.74E-06
F11E6.1b	59	76	61	87	3.61E-06	4.60E-06	1.82E-06	2.32E-06
F11E6.1c	65	80	63	90	4.12E-06	4.89E-06	2.68E-06	4.99E-06
F11E6.2	4	1	3	1	4.17E-06	5.08E-06	2.81E-06	4.95E-06
F11E6.3.1	7047	4232	8375	4584	3.98E-06	4.63E-06	2.51E-06	4.43E-06
F11E6.3.2	5831	3565	7091	4090	2.80E-06	2.65E-06	1.82E-06	2.25E-06
F11E6.4	6	11	5	4	6.55E-04	3.72E-04	5.07E-04	3.42E-04
F11E6.6	4	16	6	2	6.26E-04	3.61E-04	4.95E-04	3.52E-04
F11E6.7	901	905	1543	1945	2.80E-06	2.99E-06	1.82E-06	2.25E-06
F11E6.8	70	92	38	50	2.80E-06	2.65E-06	1.82E-06	2.25E-06
F11E6.9	27	70	20	12	3.17E-05	3.00E-05	3.53E-05	5.49E-05
F11F1.1	33	65	34	49	4.42E-06	5.50E-06	1.82E-06	2.54E-06
F11F1.2	6	9	4	5	3.25E-06	7.96E-06	1.82E-06	2.25E-06
F11F1.4	5	2	5	4	2.80E-06	3.78E-06	1.82E-06	2.41E-06
F11F1.5	1	3	3	4	2.80E-06	2.65E-06	1.82E-06	2.25E-06
F11F1.6	26	46	18	24	2.80E-06	2.65E-06	1.82E-06	2.25E-06
F11F1.7	3	4	4	1	2.80E-06	2.65E-06	1.82E-06	2.25E-06
F11F1.8	3	3	4	1	4.23E-06	7.09E-06	1.91E-06	3.15E-06
F11G11.1	29	33	14	16	2.80E-06	2.65E-06	1.82E-06	2.25E-06
F11G11.10	2463	2923	1132	793	2.80E-06	2.65E-06	1.82E-06	2.25E-06
F11G11.11	4398	5995	5427	9896	5.24E-06	5.63E-06	1.82E-06	2.32E-06
F11G11.12	2484	2981	952	738	2.54E-04	2.85E-04	7.61E-05	6.58E-05
F11G11.13	6	5	4	3	4.73E-04	6.09E-04	3.80E-04	8.54E-04
F11G11.14	16	23	19	4	2.84E-04	3.22E-04	7.10E-05	6.79E-05
F11G11.2	450	705	425	365	2.80E-06	2.65E-06	1.82E-06	2.25E-06
F11G11.3	106	92	101	39	2.97E-06	4.05E-06	2.30E-06	2.25E-06
F11G11.4	23	33	8	14	6.96E-05	1.03E-04	4.28E-05	4.54E-05
F11G11.5	421	512	458	488	1.62E-05	1.33E-05	1.01E-05	4.79E-06
F11G11.7	472	478	613	872	6.66E-06	9.02E-06	1.82E-06	3.26E-06
F11G11.9	30	65	27	27	4.83E-05	5.54E-05	3.42E-05	4.49E-05

F11H8.2	19	20	19	12	2.97E-05	2.85E-05	2.51E-05	4.41E-05
F11H8.3	2321	2836	4355	4658	3.81E-06	7.78E-06	2.22E-06	2.74E-06
F11H8.4a	382	375	471	774	2.80E-06	2.65E-06	1.82E-06	2.25E-06
F11H8.4b	386	379	480	782	2.86E-04	3.30E-04	3.49E-04	4.60E-04
F11H8.t1	0	1	1	1	9.49E-06	8.81E-06	7.62E-06	1.55E-05
F12A10.1	5	27	5	2	9.58E-06	8.89E-06	7.76E-06	1.56E-05
F12A10.2	20	25	14	39	2.80E-06	2.65E-06	1.82E-06	2.25E-06
F12A10.3a	13	15	12	10	2.80E-06	7.88E-06	1.82E-06	2.25E-06
F12A10.3b	13	15	13	11	4.65E-06	5.48E-06	2.11E-06	7.26E-06
F12A10.4	22	29	18	13	2.80E-06	2.65E-06	1.82E-06	2.25E-06
F12A10.5	10	16	8	5	2.80E-06	2.65E-06	1.82E-06	2.25E-06
F12A10.6	8	14	25	22	2.80E-06	2.65E-06	1.82E-06	2.25E-06
F12A10.7	1	4	0	2	2.80E-06	3.86E-06	1.82E-06	2.25E-06
F12A10.8	145	166	251	315	2.80E-06	3.91E-06	4.83E-06	5.24E-06
F12B6.1	141	143	74	156	2.80E-06	2.65E-06	1.82E-06	2.25E-06
F12B6.2a	69	135	55	110	7.87E-06	8.52E-06	8.86E-06	1.37E-05
F12B6.2b.1	61	126	52	104	2.80E-06	2.65E-06	1.82E-06	2.25E-06
F12B6.2b.2	61	127	52	105	4.40E-06	8.09E-06	2.28E-06	5.62E-06
F12B6.2c	60	110	38	91	4.20E-06	8.17E-06	2.33E-06	5.74E-06
F12B6.2d	60	124	50	105	4.20E-06	8.28E-06	2.33E-06	5.82E-06
F12B6.3	16	26	18	17	4.34E-06	7.54E-06	1.82E-06	5.31E-06
F12D9.1a	7	15	5	5	3.53E-06	6.90E-06	1.91E-06	4.97E-06
F12D9.1b	2	11	4	2	2.80E-06	2.65E-06	1.82E-06	2.25E-06
F12D9.2	0	4	3	0	2.80E-06	2.65E-06	1.82E-06	2.25E-06
F12E12.1	47	60	159	59	2.80E-06	2.65E-06	1.82E-06	2.25E-06
F12E12.10	5	7	7	9	2.80E-06	2.65E-06	1.82E-06	2.25E-06
F12E12.11	11	14	15	16	1.40E-05	1.68E-05	3.07E-05	1.41E-05
F12E12.2	2	1	0	3	2.80E-06	2.65E-06	1.82E-06	2.25E-06
F12E12.3	3	10	6	7	2.80E-06	2.65E-06	1.82E-06	2.25E-06
F12E12.4	9	5	14	9	2.80E-06	2.65E-06	1.82E-06	2.25E-06
F12E12.5	2	5	5	0	2.80E-06	2.65E-06	1.82E-06	2.25E-06
F12E12.6	4	2	3	5	2.80E-06	2.65E-06	1.82E-06	2.25E-06
F12E12.7	4	4	7	3	2.80E-06	2.65E-06	1.82E-06	2.25E-06
F12E12.8	10	20	12	8	2.80E-06	2.65E-06	1.82E-06	2.25E-06
F12E12.9	6	7	4	4	2.80E-06	2.65E-06	1.82E-06	2.25E-06
F12F3.1a.1	21	39	25	18	2.80E-06	2.65E-06	1.82E-06	2.25E-06
F12F3.1a.2	18	38	25	17	2.80E-06	2.65E-06	1.82E-06	2.25E-06
F12F3.1b	18	38	25	17	2.80E-06	2.65E-06	1.82E-06	2.25E-06
F12F3.1c	18	38	26	17	2.80E-06	2.70E-06	1.82E-06	2.25E-06
F12F3.1d	28	44	29	22	2.80E-06	2.70E-06	1.82E-06	2.25E-06
F12F6.1	608	655	903	1216	2.80E-06	2.65E-06	1.82E-06	2.25E-06
F12F6.2	25	34	31	51	2.80E-06	2.72E-06	1.82E-06	2.25E-06
F12F6.3.1	165	244	229	359	2.41E-05	2.45E-05	2.33E-05	3.87E-05
F12F6.3.2	172	245	261	394	9.52E-06	1.22E-05	7.69E-06	1.56E-05
F12F6.5	2067	1925	2995	4235	1.12E-05	1.57E-05	1.01E-05	1.96E-05
F12F6.6	1770	1661	1898	2633	1.15E-05	1.54E-05	1.13E-05	2.11E-05
F12F6.7.1	383	491	510	675	6.52E-05	5.73E-05	6.15E-05	1.07E-04
F12F6.7.2	355	444	447	619	5.17E-05	4.58E-05	3.61E-05	6.18E-05
F12F6.8	19	7	23	11	2.81E-05	3.41E-05	2.44E-05	3.99E-05
F12F6.9	38	72	34	25	2.93E-05	3.46E-05	2.40E-05	4.10E-05
F13A2.1	0	3	1	2	2.80E-06	2.65E-06	1.82E-06	2.25E-06
F13A2.2	0	2	1	2	3.19E-06	5.74E-06	1.86E-06	2.25E-06
F13A2.3	6	6	8	3	2.80E-06	2.65E-06	1.82E-06	2.25E-06
F13A2.4	13	8	32	9	2.80E-06	2.65E-06	1.82E-06	2.25E-06
F13A2.5	4	3	4	4	2.80E-06	2.65E-06	1.82E-06	2.25E-06
F13A2.6	7	4	6	5	3.61E-06	2.65E-06	5.79E-06	2.25E-06
F13A2.8	3	3	2	3	2.80E-06	2.65E-06	1.82E-06	2.25E-06
F13A2.9	1	1	2	1	2.80E-06	2.65E-06	1.82E-06	2.25E-06
F13A7.1	23	54	24	18	2.80E-06	2.65E-06	1.82E-06	2.25E-06

F13A7.10	14	8	18	11	2.80E-06	2.65E-06	1.82E-06	2.25E-06
F13A7.11	11	22	13	18	2.80E-06	5.45E-06	1.82E-06	2.25E-06
F13A7.12	7	6	14	7	2.80E-06	2.65E-06	1.82E-06	2.25E-06
F13A7.13	6	5	7	1	2.80E-06	3.57E-06	1.82E-06	2.47E-06
F13A7.2	2	0	5	3	2.80E-06	2.65E-06	1.82E-06	2.25E-06
F13A7.3	6	7	10	10	2.80E-06	2.65E-06	1.82E-06	2.25E-06
F13A7.4	5	8	8	4	2.80E-06	2.65E-06	1.82E-06	2.25E-06
F13A7.6	8	6	16	6	2.80E-06	2.65E-06	1.82E-06	2.25E-06
F13A7.7	19	17	11	4	2.80E-06	2.65E-06	1.82E-06	2.25E-06
F13A7.8	4	3	3	5	2.80E-06	2.65E-06	1.82E-06	2.25E-06
F13A7.9	18	21	18	22	2.80E-06	2.65E-06	1.82E-06	2.25E-06
F13B10.1a	119	208	91	207	2.80E-06	2.65E-06	1.82E-06	2.25E-06
F13B10.1b	138	249	163	271	3.42E-06	3.78E-06	2.22E-06	3.37E-06
F13B10.1c	119	207	92	212	4.76E-06	7.88E-06	2.37E-06	6.66E-06
F13B10.1d.1	75	155	64	164	6.58E-06	1.12E-05	5.05E-06	1.04E-05
F13B10.1d.2	75	154	64	163	4.42E-06	7.30E-06	2.22E-06	6.34E-06
F13B10.1e.1	161	259	149	280	6.78E-06	1.32E-05	3.75E-06	1.19E-05
F13B10.1e.2	160	262	152	286	6.78E-06	1.31E-05	3.77E-06	1.18E-05
F13B10.1e.3	118	208	96	207	5.10E-06	7.75E-06	3.08E-06	7.13E-06
F13B10.2a.1	20714	19450	28138	30371	5.04E-06	7.80E-06	3.12E-06	7.26E-06
F13B10.2a.2	19966	18516	26831	29922	4.73E-06	7.86E-06	2.50E-06	6.66E-06
F13B10.2d.1	16022	14911	21949	23604	1.90E-03	1.69E-03	1.68E-03	2.24E-03
F13B10.2d.2	15825	14674	21857	23451	1.79E-03	1.57E-03	1.57E-03	2.16E-03
F13B12.1.1	534	714	732	1000	1.83E-03	1.61E-03	1.63E-03	2.16E-03
F13B12.1.2	492	663	663	948	1.90E-03	1.66E-03	1.70E-03	2.26E-03
F13B12.2	31	41	40	39	3.57E-05	4.51E-05	3.19E-05	5.37E-05
F13B12.3	27	27	21	31	3.55E-05	4.52E-05	3.11E-05	5.49E-05
F13B12.5	11	24	33	7	2.80E-06	2.65E-06	1.82E-06	2.25E-06
F13B12.6	560	436	820	1181	2.80E-06	2.65E-06	1.82E-06	2.25E-06
F13B12.7	2	6	9	1	2.80E-06	3.20E-06	3.02E-06	2.25E-06
F13B6.1	24	48	16	27	3.84E-05	2.83E-05	3.66E-05	6.51E-05
F13B6.2	53	73	78	76	2.80E-06	2.65E-06	1.82E-06	2.25E-06
F13B6.3.1	72	113	72	66	2.80E-06	4.84E-06	1.82E-06	2.32E-06
F13B6.3.2	67	104	70	65	3.89E-06	5.08E-06	3.74E-06	4.48E-06
F13B9.1a	756	704	547	846	5.99E-06	8.89E-06	3.90E-06	4.41E-06
F13B9.1b	755	702	548	843	5.77E-06	8.46E-06	3.92E-06	4.50E-06
F13B9.1c	699	654	527	815	1.60E-05	1.41E-05	7.54E-06	1.44E-05
F13B9.2	33	35	17	17	1.62E-05	1.42E-05	7.65E-06	1.45E-05
F13B9.5.1	63	85	62	95	1.62E-05	1.43E-05	7.94E-06	1.52E-05
F13B9.5.2	61	83	61	95	5.01E-06	5.03E-06	1.82E-06	2.25E-06
F13B9.6	13	19	10	11	2.80E-06	3.44E-06	1.82E-06	3.26E-06
F13B9.8a	36	67	43	43	2.80E-06	3.36E-06	1.82E-06	3.28E-06
F13B9.8b	44	83	52	60	2.80E-06	2.65E-06	1.82E-06	2.25E-06
F13C5.1	19	24	21	20	7.39E-06	1.30E-05	5.76E-06	7.11E-06
F13C5.2	141	142	107	196	7.92E-06	1.42E-05	6.10E-06	8.68E-06
F13C5.3	10	9	11	2	2.80E-06	2.65E-06	1.82E-06	2.25E-06
F13C5.4	5	4	7	5	9.58E-06	9.13E-06	4.74E-06	1.07E-05
F13C5.5	48	69	58	51	2.80E-06	2.65E-06	1.82E-06	2.25E-06
F13C5.6a	145	191	108	211	2.80E-06	2.65E-06	2.30E-06	2.25E-06
F13C5.6b	152	210	117	223	6.75E-06	9.15E-06	5.30E-06	5.76E-06
F13D11.1	37	66	59	51	1.08E-05	1.34E-05	5.21E-06	1.26E-05
F13D11.2a	168	141	121	471	1.02E-05	1.33E-05	5.10E-06	1.20E-05
F13D11.2b	104	85	68	270	3.05E-06	5.16E-06	3.17E-06	3.37E-06
F13D11.3	12	33	16	23	4.20E-06	3.33E-06	1.97E-06	9.47E-06
F13D12.10	13	15	10	12	3.95E-06	3.04E-06	1.82E-06	8.23E-06
F13D12.2	314	593	370	599	2.80E-06	6.06E-06	2.02E-06	3.58E-06
F13D12.3	67	126	90	123	2.80E-06	2.65E-06	1.82E-06	2.25E-06
F13D12.4a.1	4779	5536	6513	9308	3.17E-05	5.65E-05	2.43E-05	4.86E-05
F13D12.4a.2	4648	5359	6316	9228	7.95E-06	1.41E-05	6.96E-06	1.17E-05

F13D12.4a.3	4619	5341	6245	9179	2.93E-04	3.21E-04	2.60E-04	4.59E-04
F13D12.4b	3334	3887	4285	6140	2.91E-04	3.17E-04	2.58E-04	4.65E-04
F13D12.5	150	190	232	235	2.82E-04	3.08E-04	2.48E-04	4.50E-04
F13D12.6.1	1329	1404	2113	1820	2.87E-04	3.16E-04	2.40E-04	4.24E-04
F13D12.6.2	1312	1367	2045	1792	1.33E-05	1.59E-05	1.34E-05	1.67E-05
F13D12.7.1	1815	1588	1731	2264	9.58E-05	9.56E-05	9.91E-05	1.05E-04
F13D12.7.2	846	748	746	941	9.71E-05	9.55E-05	9.85E-05	1.06E-04
F13D12.7.3	828	729	725	925	8.62E-05	7.13E-05	5.35E-05	8.64E-05
F13D12.8	23	46	21	8	7.45E-05	6.22E-05	4.27E-05	6.65E-05
F13D2.1	118	201	101	213	7.81E-05	6.50E-05	4.45E-05	7.01E-05
F13D2.2	2	12	9	4	3.81E-06	7.19E-06	2.26E-06	2.25E-06
F13D2.3	8	9	6	10	3.19E-06	5.13E-06	1.82E-06	4.61E-06
F13D2.4	23	26	15	39	2.80E-06	2.65E-06	1.82E-06	2.25E-06
F13E6.1	205	372	181	265	2.80E-06	2.65E-06	1.82E-06	2.25E-06
F13E6.2	20	12	23	31	7.08E-06	7.59E-06	3.01E-06	9.67E-06
F13E6.3	10	15	30	8	2.58E-05	4.43E-05	1.49E-05	2.69E-05
F13E6.4	218	312	227	339	2.80E-06	2.65E-06	1.82E-06	2.25E-06
F13E6.5	103	156	142	151	2.80E-06	2.65E-06	2.70E-06	2.25E-06
F13E6.6	61	93	44	75	1.20E-05	1.63E-05	8.16E-06	1.50E-05
F13E9.1	327	441	471	700	1.10E-05	1.57E-05	9.84E-06	1.29E-05
F13E9.10	2	3	1	4	2.80E-06	2.65E-06	1.82E-06	2.25E-06
F13E9.12.1	6	10	10	2	1.84E-05	2.35E-05	1.73E-05	3.17E-05
F13E9.12.2	6	10	10	1	2.80E-06	2.65E-06	1.82E-06	2.25E-06
F13E9.13	19	29	20	18	2.80E-06	3.62E-06	2.50E-06	2.25E-06
F13E9.2	0	0	0	3	2.80E-06	3.44E-06	2.37E-06	2.25E-06
F13E9.3	3	0	1	2	2.80E-06	3.68E-06	1.82E-06	2.25E-06
F13E9.4	3	8	10	2	2.80E-06	2.65E-06	1.82E-06	2.25E-06
F13E9.5	17	52	9	12	2.80E-06	2.65E-06	1.82E-06	2.25E-06
F13E9.6	3	2	6	1	2.80E-06	2.65E-06	1.82E-06	2.25E-06
F13E9.8	4	5	3	9	2.80E-06	5.29E-06	1.82E-06	2.25E-06
F13E9.9	3	4	2	4	2.80E-06	2.65E-06	1.82E-06	2.25E-06
F13G11.1a	262	419	301	594	2.80E-06	2.65E-06	1.82E-06	2.25E-06
F13G11.1b.1	219	324	226	452	2.80E-06	2.65E-06	1.82E-06	2.25E-06
F13G11.1b.2	225	331	227	459	1.57E-05	2.36E-05	1.17E-05	2.85E-05
F13G11.1b.3	213	319	218	448	1.55E-05	2.16E-05	1.04E-05	2.56E-05
F13G11.2	19	44	20	5	1.65E-05	2.30E-05	1.08E-05	2.71E-05
F13G3.1	47	60	42	32	1.68E-05	2.38E-05	1.12E-05	2.85E-05
F13G3.10	276	323	452	136	3.25E-06	7.12E-06	2.22E-06	2.25E-06
F13G3.12	13	17	16	14	3.00E-06	3.62E-06	1.82E-06	2.25E-06
F13G3.2	5	14	4	9	8.01E-05	8.85E-05	8.53E-05	3.17E-05
F13G3.3	23	34	22	24	2.80E-06	2.65E-06	1.82E-06	2.25E-06
F13G3.6	81	133	102	129	2.80E-06	2.65E-06	1.82E-06	2.25E-06
F13G3.7a	168	215	220	322	2.80E-06	2.65E-06	1.82E-06	2.25E-06
F13G3.7b.1	221	316	301	400	9.18E-06	1.43E-05	7.53E-06	1.18E-05
F13G3.7b.2	168	211	218	323	1.49E-05	1.81E-05	1.27E-05	2.30E-05
F13G3.9	90	123	197	107	1.51E-05	2.05E-05	1.34E-05	2.20E-05
F13H10.1	4	5	12	6	1.50E-05	1.79E-05	1.27E-05	2.32E-05
F13H10.2	175	269	233	275	2.08E-05	2.68E-05	2.96E-05	1.98E-05
F13H10.3a	485	624	731	969	2.80E-06	2.65E-06	2.44E-06	2.25E-06
F13H10.3b	400	499	621	844	1.65E-05	2.39E-05	1.43E-05	2.08E-05
F13H10.5	16	16	16	12	2.44E-05	2.96E-05	2.39E-05	3.91E-05
F13H10.6	77	177	202	92	2.42E-05	2.85E-05	2.44E-05	4.10E-05
F13H10.8	355	531	552	305	2.80E-06	2.65E-06	1.82E-06	2.25E-06
F13H6.1	218	332	133	230	2.82E-05	6.12E-05	4.81E-05	2.71E-05
F13H6.3	212	263	222	200	8.82E-05	1.25E-04	8.92E-05	6.08E-05
F13H6.4	15	14	8	14	1.10E-05	1.58E-05	4.35E-06	9.29E-06
F13H6.5	17	35	36	22	1.35E-05	1.58E-05	9.22E-06	1.03E-05
F13H8.10a	253	280	270	397	2.80E-06	2.65E-06	1.82E-06	2.25E-06
F13H8.10b	209	234	214	314	2.80E-06	2.65E-06	1.82E-06	2.25E-06

F13H8.10c.1	197	221	203	302	8.79E-06	9.20E-06	6.12E-06	1.11E-05
F13H8.10c.2	206	229	209	310	1.19E-05	1.25E-05	7.91E-06	1.43E-05
F13H8.11	37	41	33	59	1.25E-05	1.33E-05	8.40E-06	1.54E-05
F13H8.12	7	8	6	6	1.25E-05	1.31E-05	8.25E-06	1.51E-05
F13H8.1a	11	26	6	3	2.80E-06	2.65E-06	1.82E-06	3.15E-06
F13H8.1b	18	42	17	7	3.14E-06	3.41E-06	1.82E-06	2.25E-06
F13H8.2	652	665	823	1151	2.80E-06	5.85E-06	1.82E-06	2.25E-06
F13H8.3	85	176	108	216	2.80E-06	5.32E-06	1.82E-06	2.25E-06
F13H8.4	32	38	29	32	2.40E-05	2.31E-05	1.97E-05	3.40E-05
F13H8.5	337	417	95	179	7.67E-06	1.50E-05	6.34E-06	1.56E-05
F13H8.6	4	5	5	6	4.34E-06	4.87E-06	2.55E-06	3.49E-06
F13H8.7.1	581	1004	680	760	2.09E-05	2.44E-05	3.83E-06	8.91E-06
F13H8.7.2	519	896	600	703	2.80E-06	2.65E-06	1.82E-06	2.25E-06
F13H8.7.3	526	910	619	716	5.13E-05	8.38E-05	3.91E-05	5.39E-05
F13H8.8	31	67	25	58	4.98E-05	8.12E-05	3.75E-05	5.42E-05
F13H8.9	25	53	21	24	5.03E-05	8.23E-05	3.86E-05	5.51E-05
F14A5.1	6	7	7	2	2.80E-06	4.52E-06	1.82E-06	3.33E-06
F14B4.1	37	37	43	59	2.80E-06	4.73E-06	1.82E-06	2.25E-06
F14B4.2a.1	1536	1285	1990	2677	2.80E-06	2.65E-06	1.82E-06	2.25E-06
F14B4.2a.2	1473	1248	1868	2614	2.80E-06	2.65E-06	1.82E-06	2.29E-06
F14B4.2b	1654	1422	2166	2962	1.13E-04	8.95E-05	9.55E-05	1.59E-04
F14B4.3	782	753	948	1439	1.09E-04	8.70E-05	8.98E-05	1.55E-04
F14B6.1	12	16	13	19	9.22E-05	7.49E-05	7.86E-05	1.33E-04
F14B6.2	44	57	29	35	2.35E-05	2.14E-05	1.85E-05	3.47E-05
F14B6.3	13	19	12	9	2.80E-06	2.65E-06	1.82E-06	2.25E-06
F14B6.4	3	4	5	2	2.80E-06	2.72E-06	1.82E-06	2.25E-06
F14B6.5	8	6	13	1	2.80E-06	2.65E-06	1.82E-06	2.25E-06
F14B6.6a	19	24	5	8	2.80E-06	2.65E-06	1.82E-06	2.25E-06
F14B6.t1	0	0	0	1	2.80E-06	2.65E-06	1.82E-06	2.25E-06
F14B6.t2	0	0	0	1	2.80E-06	2.65E-06	1.82E-06	2.25E-06
F14B8.1a	111	136	113	194	2.80E-06	2.65E-06	1.82E-06	2.25E-06
F14B8.1b.1	78	100	78	167	2.80E-06	2.65E-06	1.82E-06	2.25E-06
F14B8.1b.2	94	114	84	172	4.20E-06	4.87E-06	2.79E-06	5.89E-06
F14B8.2	17	24	8	13	3.81E-06	4.63E-06	2.48E-06	6.57E-06
F14B8.3	69	90	78	198	4.09E-06	4.71E-06	2.39E-06	6.03E-06
F14B8.4	65	57	109	46	9.30E-06	1.24E-05	2.84E-06	5.71E-06
F14B8.5a.1	99	136	61	111	5.60E-06	6.88E-06	4.12E-06	1.29E-05
F14B8.5a.2	76	112	47	78	2.09E-05	1.73E-05	2.28E-05	1.19E-05
F14B8.5a.3	77	113	49	80	5.24E-06	6.80E-06	2.10E-06	4.72E-06
F14B8.5b	71	108	46	81	5.21E-06	7.27E-06	2.10E-06	4.30E-06
F14B8.6	88	115	108	91	5.26E-06	7.30E-06	2.19E-06	4.39E-06
F14B8.7	44	48	45	54	5.10E-06	7.33E-06	2.15E-06	4.68E-06
F14B8.t1	0	0	1	0	6.41E-06	7.91E-06	5.12E-06	5.33E-06
F14D12.1a	26	36	17	35	2.80E-06	2.88E-06	1.88E-06	2.77E-06
F14D12.1b	28	39	28	39	2.80E-06	2.65E-06	1.82E-06	2.25E-06
F14D12.4a	30	37	19	22	2.80E-06	2.65E-06	1.82E-06	2.25E-06
F14D12.4b	39	60	20	32	2.80E-06	2.65E-06	1.82E-06	2.25E-06
F14D12.4c	19	28	13	17	2.80E-06	2.65E-06	1.82E-06	2.25E-06
F14D12.5	44	68	39	73	2.80E-06	3.76E-06	1.82E-06	2.25E-06
F14D12.6a	9	11	14	14	2.80E-06	2.91E-06	1.82E-06	2.25E-06
F14D12.6b	8	10	11	12	2.80E-06	3.31E-06	1.82E-06	3.04E-06
F14D2.1	130	141	101	177	2.80E-06	2.65E-06	1.82E-06	2.25E-06
F14D2.11	14	24	9	10	2.80E-06	2.65E-06	1.82E-06	2.25E-06
F14D2.12	189	202	352	313	1.62E-05	1.66E-05	8.18E-06	1.77E-05
F14D2.13	58	79	99	134	4.70E-06	7.62E-06	1.97E-06	2.70E-06
F14D2.14	11	16	13	13	2.22E-05	2.24E-05	2.69E-05	2.95E-05
F14D2.15	128	122	101	168	6.33E-06	8.15E-06	7.03E-06	1.17E-05
F14D2.2	133	154	144	182	2.80E-06	3.12E-06	1.82E-06	2.25E-06
F14D2.4a	114	131	236	190	1.59E-05	1.43E-05	8.18E-06	1.68E-05

F14D2.4b	92	83	134	139	1.11E-05	1.22E-05	7.84E-06	1.22E-05
F14D2.5	8	5	7	1	1.27E-05	1.38E-05	1.71E-05	1.70E-05
F14D2.6	19	11	21	7	1.29E-05	1.10E-05	1.22E-05	1.56E-05
F14D2.7	25	23	32	23	2.80E-06	2.65E-06	1.82E-06	2.25E-06
F14D2.8	33	53	26	36	2.80E-06	2.65E-06	1.82E-06	2.25E-06
F14D2.9	33	25	43	43	2.80E-06	2.65E-06	1.82E-06	2.25E-06
F14D7.1	1	4	2	0	2.80E-06	4.26E-06	1.82E-06	2.45E-06
F14D7.10	18	25	15	9	2.80E-06	2.65E-06	1.82E-06	2.25E-06
F14D7.11	0	1	1	2	2.80E-06	2.65E-06	1.82E-06	2.25E-06
F14D7.2	194	309	352	431	3.72E-06	4.87E-06	2.00E-06	2.25E-06
F14D7.3	1	1	3	0	2.80E-06	2.65E-06	1.82E-06	2.25E-06
F14D7.4	0	4	3	3	1.50E-05	2.26E-05	1.77E-05	2.68E-05
F14D7.5	1	2	1	2	2.80E-06	2.65E-06	1.82E-06	2.25E-06
F14D7.6	44	65	33	30	2.80E-06	2.65E-06	1.82E-06	2.25E-06
F14D7.7	18	24	16	10	2.80E-06	2.65E-06	1.82E-06	2.25E-06
F14D7.8	2	6	5	5	3.25E-06	4.55E-06	1.82E-06	2.25E-06
F14D7.9	2	5	10	3	6.47E-06	8.15E-06	3.74E-06	2.88E-06
F14E5.1	17	39	14	33	2.80E-06	2.65E-06	1.82E-06	2.25E-06
F14E5.2a	1288	1657	1651	2271	2.80E-06	2.65E-06	1.82E-06	2.25E-06
F14E5.2b	1408	1793	1710	2354	2.80E-06	2.91E-06	1.82E-06	2.25E-06
F14E5.3	25	30	10	12	4.16E-05	5.06E-05	3.47E-05	5.89E-05
F14E5.5	108	452	58	102	4.15E-05	4.99E-05	3.28E-05	5.58E-05
F14E5.6	9	15	7	4	2.80E-06	2.65E-06	1.82E-06	2.25E-06
F14F11.1a.1	28	49	21	43	1.23E-05	4.86E-05	4.30E-06	9.31E-06
F14F11.1a.2	28	49	21	43	2.80E-06	2.65E-06	1.82E-06	2.25E-06
F14F11.1a.3	28	49	21	43	2.80E-06	3.49E-06	1.82E-06	2.59E-06
F14F11.1a.4	28	49	21	43	2.80E-06	3.33E-06	1.82E-06	2.50E-06
F14F11.1b	28	50	24	43	2.80E-06	3.36E-06	1.82E-06	2.52E-06
F14F11.1d	28	49	21	43	2.80E-06	3.33E-06	1.82E-06	2.50E-06
F14F11.1e	28	50	21	43	2.80E-06	2.99E-06	1.82E-06	2.25E-06
F14F11.1f	31	55	24	44	2.80E-06	3.44E-06	1.82E-06	2.56E-06
F14F11.1g	30	50	23	45	2.80E-06	3.41E-06	1.82E-06	2.50E-06
F14F11.1h	22	56	30	36	2.80E-06	3.41E-06	1.82E-06	2.32E-06
F14F11.2	27	33	30	20	2.80E-06	2.78E-06	1.82E-06	2.25E-06
F14F3.1a	19	17	12	20	2.80E-06	3.02E-06	1.82E-06	2.25E-06
F14F3.1b	14	11	8	14	5.99E-06	6.93E-06	4.34E-06	3.58E-06
F14F3.1c	37	32	30	39	2.80E-06	2.65E-06	1.82E-06	2.25E-06
F14F3.2	113	138	126	165	2.80E-06	2.65E-06	1.82E-06	2.25E-06
F14F3.3	89	108	100	118	2.80E-06	2.65E-06	1.82E-06	2.25E-06
F14F3.4	7	15	5	1	5.49E-06	6.32E-06	3.97E-06	6.43E-06
F14F4.1	4	6	2	3	6.33E-06	7.25E-06	4.63E-06	6.75E-06
F14F4.3a	613	686	632	1136	2.91E-06	5.87E-06	1.82E-06	2.25E-06
F14F4.3b	556	628	584	1066	2.80E-06	2.65E-06	1.82E-06	2.25E-06
F14F7.1.1	996	1710	1789	1934	1.48E-05	1.57E-05	9.95E-06	2.21E-05
F14F7.1.2	824	1427	1391	1742	1.45E-05	1.55E-05	9.93E-06	2.24E-05
F14F7.2	6	9	4	11	1.00E-04	1.63E-04	1.17E-04	1.56E-04
F14F7.3	4	6	7	13	8.75E-05	1.43E-04	9.61E-05	1.49E-04
F14F7.4	28	26	25	20	2.80E-06	2.65E-06	1.82E-06	2.25E-06
F14F7.5	23	32	33	24	2.80E-06	2.65E-06	1.82E-06	2.25E-06
F14F8.1	5	9	15	4	3.50E-06	3.07E-06	2.02E-06	2.25E-06
F14F8.10	8	7	6	11	2.80E-06	2.65E-06	1.82E-06	2.25E-06
F14F8.11	13	14	19	4	2.80E-06	2.65E-06	1.82E-06	2.25E-06
F14F8.12	6	16	12	10	2.80E-06	2.65E-06	1.82E-06	2.25E-06
F14F8.13	11	10	16	14	2.80E-06	2.65E-06	1.82E-06	2.25E-06
F14F8.14	0	1	0	2	2.80E-06	2.65E-06	1.82E-06	2.25E-06
F14F8.2	12	7	20	13	2.80E-06	2.65E-06	1.82E-06	2.25E-06
F14F8.3	15	6	14	7	2.80E-06	2.65E-06	1.82E-06	2.25E-06
F14F8.4	6	6	5	2	2.80E-06	2.65E-06	1.82E-06	2.25E-06
F14F8.5	4	6	11	5	2.80E-06	2.65E-06	1.82E-06	2.25E-06

F14F8.6	8	4	4	2	2.80E-06	2.65E-06	1.82E-06	2.25E-06
F14F8.7	10	8	31	15	2.80E-06	2.65E-06	1.82E-06	2.25E-06
F14F8.8	1	3	4	0	2.80E-06	2.65E-06	1.82E-06	2.25E-06
F14F8.9	1	5	3	1	2.80E-06	2.65E-06	2.35E-06	2.25E-06
F14F9.1	3	3	3	5	2.80E-06	2.65E-06	1.82E-06	2.25E-06
F14F9.2	8	2	4	2	2.80E-06	2.65E-06	1.82E-06	2.25E-06
F14F9.3	17	23	15	15	2.80E-06	2.65E-06	1.82E-06	2.25E-06
F14F9.4	55	40	34	65	2.80E-06	2.65E-06	1.82E-06	2.25E-06
F14F9.5	25	34	30	23	2.80E-06	2.65E-06	1.82E-06	2.25E-06
F14F9.6	1	1	1	1	2.80E-06	2.65E-06	1.82E-06	2.25E-06
F14F9.7	0	3	6	3	2.83E-06	3.65E-06	2.20E-06	2.25E-06
F14F9.8	17	34	16	12	2.80E-06	2.65E-06	1.82E-06	2.25E-06
F14H12.1	61	51	27	37	2.80E-06	2.65E-06	1.82E-06	2.25E-06
F14H12.2	9	4	1	1	6.97E-06	1.32E-05	4.26E-06	3.96E-06
F14H12.3	22	28	16	11	6.61E-06	5.21E-06	1.90E-06	3.22E-06
F14H12.4a	134	229	95	233	2.80E-06	2.65E-06	1.82E-06	2.25E-06
F14H12.4b	127	209	84	227	2.83E-06	3.41E-06	1.82E-06	2.25E-06
F14H12.6	11	4	18	5	9.32E-06	1.50E-05	4.30E-06	1.30E-05
F14H12.7	5	8	14	2	9.46E-06	1.47E-05	4.08E-06	1.36E-05
F14H12.8	5	5	7	8	3.22E-06	2.65E-06	3.44E-06	2.25E-06
F14H3.1	5	3	3	1	2.80E-06	2.83E-06	3.41E-06	2.25E-06
F14H3.10	10	34	8	18	2.80E-06	2.65E-06	1.82E-06	2.25E-06
F14H3.11	11	32	19	24	2.80E-06	2.65E-06	1.82E-06	2.25E-06
F14H3.12	9	27	123	25	2.80E-06	2.65E-06	1.82E-06	2.25E-06
F14H3.13	1	2	4	0	2.80E-06	3.25E-06	1.82E-06	2.25E-06
F14H3.2	4	14	7	8	2.80E-06	6.67E-06	2.09E-05	5.24E-06
F14H3.3	64	62	46	64	2.80E-06	2.65E-06	1.82E-06	2.25E-06
F14H3.4	80	138	125	126	2.80E-06	2.65E-06	1.82E-06	2.25E-06
F14H3.5	54	67	79	76	7.31E-06	6.69E-06	3.41E-06	5.87E-06
F14H3.6	590	770	1254	1105	1.06E-05	1.73E-05	1.08E-05	1.34E-05
F14H3.7	3	6	13	5	1.02E-05	1.20E-05	9.75E-06	1.16E-05
F14H3.8	7	5	7	5	6.39E-05	7.88E-05	8.84E-05	9.61E-05
F14H3.9	2	6	7	2	2.80E-06	2.65E-06	1.82E-06	2.25E-06
F14H8.1	66	155	60	54	2.80E-06	2.65E-06	1.82E-06	2.25E-06
F14H8.2	6	5	1	3	2.80E-06	2.65E-06	1.82E-06	2.25E-06
F14H8.3	2	3	4	2	5.32E-06	1.18E-05	3.15E-06	3.49E-06
F14H8.4	3	2	6	0	2.80E-06	2.65E-06	1.82E-06	2.25E-06
F14H8.5	3	3	4	1	2.80E-06	2.65E-06	1.82E-06	2.25E-06
F14H8.6	18	26	11	36	2.80E-06	2.65E-06	1.82E-06	2.25E-06
F15A2.1	2699	3771	4583	5147	2.80E-06	2.65E-06	1.82E-06	2.25E-06
F15A2.2	32	45	30	46	2.80E-06	2.65E-06	1.82E-06	2.25E-06
F15A2.3	5	4	2	4	3.33E-04	4.39E-04	3.67E-04	5.09E-04
F15A2.4	5	2	9	9	2.80E-06	2.65E-06	1.82E-06	2.25E-06
F15A2.5	21	16	14	29	2.80E-06	2.65E-06	1.82E-06	2.25E-06
F15A2.7	1	2	1	2	2.80E-06	2.65E-06	1.82E-06	2.25E-06
F15A4.1	4	5	3	1	5.10E-06	3.68E-06	2.20E-06	5.65E-06
F15A4.10	24	35	85	30	2.80E-06	2.65E-06	1.82E-06	2.25E-06
F15A4.11	44	31	56	37	2.80E-06	2.65E-06	1.82E-06	2.25E-06
F15A4.12	26	36	51	27	7.00E-06	9.65E-06	1.61E-05	7.04E-06
F15A4.13	8	3	4	5	1.09E-05	7.30E-06	9.07E-06	7.40E-06
F15A4.2	13	5	14	8	7.00E-06	9.15E-06	8.93E-06	5.85E-06
F15A4.3	2	4	6	8	2.80E-06	2.65E-06	1.82E-06	2.25E-06
F15A4.4	4	5	2	4	2.80E-06	2.65E-06	1.82E-06	2.25E-06
F15A4.5	12	12	9	8	2.80E-06	2.65E-06	1.82E-06	2.25E-06
F15A4.6	68	143	140	32	2.80E-06	2.65E-06	1.82E-06	2.25E-06
F15A4.7	5	4	7	2	2.80E-06	2.65E-06	1.82E-06	2.25E-06
F15A4.8a	11	10	18	6	1.47E-05	2.93E-05	1.97E-05	5.58E-06
F15A4.8b	10	5	8	5	2.80E-06	2.65E-06	1.82E-06	2.25E-06
F15A4.9	3	4	12	3	2.80E-06	2.65E-06	1.82E-06	2.25E-06

F15A8.1	1	0	4	2	2.80E-06	2.65E-06	1.82E-06	2.25E-06
F15A8.3	7	4	1	1	2.80E-06	2.65E-06	1.82E-06	2.25E-06
F15A8.4	1	3	2	1	2.80E-06	2.65E-06	1.82E-06	2.25E-06
F15A8.5a	5	9	7	7	2.80E-06	2.65E-06	1.82E-06	2.25E-06
F15A8.5b	8	11	5	13	2.80E-06	2.65E-06	1.82E-06	2.25E-06
F15A8.5c	5	6	5	6	2.80E-06	2.65E-06	1.82E-06	2.25E-06
F15A8.5d	38	70	60	46	2.80E-06	2.65E-06	1.82E-06	2.25E-06
F15A8.6	28	60	36	32	2.80E-06	2.65E-06	1.82E-06	2.25E-06
F15A8.7	6	19	8	3	3.61E-06	6.27E-06	3.70E-06	3.51E-06
F15B10.1a	406	741	496	516	2.80E-06	3.31E-06	1.82E-06	2.25E-06
F15B10.1b	376	686	463	483	2.80E-06	2.65E-06	1.82E-06	2.25E-06
F15B10.3	13	18	9	12	4.06E-05	7.01E-05	3.23E-05	4.15E-05
F15B9.1	46	105	64	31	4.30E-05	7.41E-05	3.44E-05	4.43E-05
F15B9.2	5	5	1	6	2.80E-06	3.41E-06	1.82E-06	2.25E-06
F15B9.3	57	91	54	47	8.57E-06	1.85E-05	7.76E-06	4.63E-06
F15B9.4	558	503	724	1162	2.80E-06	2.65E-06	1.82E-06	2.25E-06
F15B9.5	30	37	20	55	1.13E-05	1.71E-05	6.98E-06	7.49E-06
F15B9.6	10	16	25	10	1.66E-05	1.41E-05	1.40E-05	2.77E-05
F15B9.7	101	124	121	163	2.80E-06	2.65E-06	1.82E-06	3.01E-06
F15B9.8	132	239	36	43	2.80E-06	2.65E-06	1.82E-06	2.25E-06
F15B9.9	10	9	4	2	2.80E-06	2.65E-06	1.82E-06	2.25E-06
F15B9.t1	1	0	3	0	2.27E-05	3.88E-05	4.03E-06	5.94E-06
F15C11.1.1	126	187	115	138	2.80E-06	2.65E-06	1.82E-06	2.25E-06
F15C11.1.2	97	139	98	105	2.80E-06	2.65E-06	3.04E-06	2.25E-06
F15C11.2a	1264	1393	1523	1971	4.96E-06	6.96E-06	2.95E-06	4.36E-06
F15C11.2b	1045	1141	1232	1614	4.84E-06	6.56E-06	3.19E-06	4.21E-06
F15C11.2c	1235	1348	1472	1913	7.82E-05	8.14E-05	6.13E-05	9.80E-05
F15D3.1a	374	542	198	495	8.04E-05	8.30E-05	6.17E-05	9.98E-05
F15D3.1b	68	81	69	76	7.52E-05	7.76E-05	5.83E-05	9.36E-05
F15D3.2	3	4	0	0	3.81E-06	5.21E-06	1.82E-06	4.05E-06
F15D3.3	5	7	6	5	7.00E-06	7.88E-06	4.63E-06	6.27E-06
F15D3.4	20	29	34	19	2.80E-06	2.65E-06	1.82E-06	2.25E-06
F15D3.5	0	0	1	1	2.80E-06	2.65E-06	1.82E-06	2.25E-06
F15D3.6	367	406	327	615	2.80E-06	2.65E-06	1.82E-06	2.25E-06
F15D3.7	264	339	501	395	2.80E-06	2.65E-06	1.82E-06	2.25E-06
F15D3.8	7	19	5	5	4.29E-05	4.48E-05	2.49E-05	5.78E-05
F15D3.t1	0	0	3	0	3.49E-05	4.23E-05	4.31E-05	4.19E-05
F15D4.1	333	360	445	715	2.80E-06	2.65E-06	1.82E-06	2.25E-06
F15D4.2.1	66	58	156	86	2.80E-06	2.65E-06	2.99E-06	2.25E-06
F15D4.2.2	54	52	101	67	7.67E-06	7.86E-06	6.69E-06	1.32E-05
F15D4.3	273	197	524	144	1.21E-05	1.01E-05	1.86E-05	1.27E-05
F15D4.4	20	13	18	21	1.09E-05	9.89E-06	1.32E-05	1.08E-05
F15D4.5	22	26	26	19	8.47E-05	5.77E-05	1.06E-04	3.59E-05
F15D4.6	6	6	3	7	2.80E-06	2.65E-06	1.82E-06	2.25E-06
F15D4.7	24	26	30	13	2.80E-06	2.65E-06	1.82E-06	2.25E-06
F15D4.8	41	65	49	50	2.80E-06	2.65E-06	1.82E-06	2.25E-06
F15E11.1	6758	255	10623	135	2.80E-06	2.65E-06	1.82E-06	2.25E-06
F15E11.10	10	118	9	45	8.20E-06	1.23E-05	6.38E-06	8.03E-06
F15E11.11	8	12	9	17	1.63E-03	5.80E-05	1.67E-03	2.61E-05
F15E11.12	606	12	1398	1	2.80E-06	1.45E-05	1.82E-06	4.70E-06
F15E11.13	5188	224	11504	83	2.80E-06	2.65E-06	1.82E-06	2.25E-06
F15E11.14	6320	229	10470	127	1.25E-04	2.65E-06	1.88E-04	2.25E-06
F15E11.15a	1734	14	2604	9	1.27E-03	5.20E-05	1.84E-03	1.64E-05
F15E11.15b.[illegible]	1777	13	2747	10	1.52E-03	5.21E-05	1.64E-03	2.46E-05
F15E11.15b.[illegible]	1558	13	2261	8	2.50E-04	2.65E-06	2.44E-04	2.25E-06
F15E11.2	3	2	4	4	3.27E-04	2.65E-06	3.29E-04	2.25E-06
F15E11.3	2	9	4	4	2.99E-04	2.65E-06	2.82E-04	2.25E-06
F15E11.4	3	4	17	7	2.80E-06	2.65E-06	1.82E-06	2.25E-06
F15E11.5	2	4	2	0	2.80E-06	2.65E-06	1.82E-06	2.25E-06

F15E6.1	887	684	1292	1690	2.80E-06	2.65E-06	1.82E-06	2.25E-06
F15E6.10	2	4	1	2	2.80E-06	2.65E-06	1.82E-06	2.25E-06
F15E6.2	195	355	137	162	2.00E-05	1.46E-05	1.90E-05	3.06E-05
F15E6.3	6	20	8	14	2.80E-06	2.65E-06	1.82E-06	2.25E-06
F15E6.4	20	61	80	72	1.56E-05	2.68E-05	7.12E-06	1.04E-05
F15E6.5	2	0	1	1	2.80E-06	4.36E-06	1.82E-06	2.61E-06
F15E6.6	94	76	37	92	7.95E-06	2.29E-05	2.07E-05	2.30E-05
F15E6.7	7	5	3	2	2.80E-06	2.65E-06	1.82E-06	2.25E-06
F15E6.8	3	9	2	2	2.83E-06	2.65E-06	1.82E-06	2.25E-06
F15E6.9	13	12	14	11	2.80E-06	2.65E-06	1.82E-06	2.25E-06
F15G9.1a	259	428	230	299	2.80E-06	4.52E-06	1.82E-06	2.25E-06
F15G9.1b	155	233	156	208	2.80E-06	2.65E-06	1.82E-06	2.25E-06
F15G9.1c	187	294	167	230	2.93E-05	4.57E-05	1.69E-05	2.72E-05
F15G9.2	2	2	3	2	2.66E-05	3.78E-05	1.74E-05	2.87E-05
F15G9.3	6	1	6	4	2.50E-05	3.72E-05	1.45E-05	2.47E-05
F15G9.4a	966	1327	1177	1710	2.80E-06	2.65E-06	1.82E-06	2.25E-06
F15G9.4b	949	1304	1165	1691	2.80E-06	2.65E-06	1.82E-06	2.25E-06
F15G9.5	11	8	18	12	6.89E-06	8.94E-06	5.47E-06	9.78E-06
F15G9.6	2	1	1	0	6.80E-06	8.83E-06	5.45E-06	9.76E-06
F15G9.t1	0	0	1	0	2.80E-06	2.65E-06	1.82E-06	2.25E-06
F15G9.t2	1	0	3	0	2.80E-06	2.65E-06	1.82E-06	2.25E-06
F15H10.1.1	2644	3082	2202	1767	2.80E-06	2.65E-06	1.82E-06	2.25E-06
F15H10.1.2	2507	2947	2061	1706	2.80E-06	2.65E-06	3.04E-06	2.25E-06
F15H10.10	1	4	6	5	2.74E-04	3.01E-04	1.48E-04	1.47E-04
F15H10.2	2463	2876	2062	1620	2.65E-04	2.95E-04	1.42E-04	1.45E-04
F15H10.5	5	3	13	9	2.80E-06	2.65E-06	1.82E-06	2.25E-06
F15H10.6	4	10	7	4	2.90E-04	3.20E-04	1.58E-04	1.53E-04
F15H10.7	15	40	26	19	2.80E-06	2.65E-06	1.82E-06	2.25E-06
F15H10.8	66	60	26	15	2.80E-06	2.65E-06	1.82E-06	2.25E-06
F15H10.9	5	6	8	0	2.80E-06	2.65E-06	1.82E-06	2.25E-06
F15H9.1	12	19	16	9	6.52E-06	5.58E-06	1.82E-06	2.25E-06
F15H9.2	3	5	7	2	2.80E-06	2.65E-06	1.82E-06	2.25E-06
F15H9.3	2	3	2	2	2.80E-06	2.65E-06	1.82E-06	2.25E-06
F15H9.4	4	6	4	2	2.80E-06	2.65E-06	1.82E-06	2.25E-06
F15H9.5	0	3	0	1	2.80E-06	2.65E-06	1.82E-06	2.25E-06
F15H9.6	0	3	0	1	2.80E-06	2.65E-06	1.82E-06	2.25E-06
F15H9.t1	0	0	0	1	2.80E-06	2.65E-06	1.82E-06	2.25E-06
F16A11.1	532	653	789	1115	2.80E-06	2.65E-06	1.82E-06	2.25E-06
F16A11.2	216	266	272	388	2.80E-06	2.65E-06	1.82E-06	2.25E-06
F16A11.3a	1239	1207	1637	2319	2.35E-05	2.73E-05	2.27E-05	3.97E-05
F16A11.3b	999	1005	1406	1926	1.50E-05	1.74E-05	1.23E-05	2.16E-05
F16A11.3c	672	648	947	1240	2.71E-05	2.49E-05	2.33E-05	4.07E-05
F16B12.1	6	12	6	8	2.70E-05	2.57E-05	2.47E-05	4.18E-05
F16B12.2	3	0	7	4	2.36E-05	2.15E-05	2.16E-05	3.50E-05
F16B12.4	38	42	36	71	2.80E-06	2.65E-06	1.82E-06	2.25E-06
F16B12.5	8	6	4	2	2.80E-06	2.65E-06	1.82E-06	2.25E-06
F16B12.6	308	292	417	510	5.26E-06	5.50E-06	3.24E-06	7.92E-06
F16B12.7	6	5	3	2	2.80E-06	2.65E-06	1.82E-06	2.25E-06
F16B12.8	6	3	4	3	7.95E-06	7.12E-06	7.00E-06	1.06E-05
F16B3.1	17	29	42	37	2.80E-06	2.65E-06	1.82E-06	2.25E-06
F16B3.2	7	9	6	6	2.80E-06	2.65E-06	1.82E-06	2.25E-06
F16B3.3	8	15	8	4	2.80E-06	2.65E-06	1.82E-06	2.25E-06
F16B4.1	18	31	15	29	2.80E-06	3.17E-06	1.82E-06	2.25E-06
F16B4.10	9	6	12	7	2.80E-06	2.72E-06	1.82E-06	2.25E-06
F16B4.11	6	13	11	7	2.80E-06	2.65E-06	1.82E-06	2.25E-06
F16B4.12a	18	37	19	8	2.80E-06	2.65E-06	1.82E-06	2.25E-06
F16B4.12b	14	31	9	8	2.80E-06	2.65E-06	1.82E-06	2.25E-06
F16B4.2a	11	23	9	16	2.80E-06	2.65E-06	1.82E-06	2.25E-06
F16B4.2b	10	22	9	16	2.80E-06	2.65E-06	1.82E-06	2.25E-06

F16B4.3	5	11	19	8	2.80E-06	2.65E-06	1.82E-06	2.25E-06
F16B4.5a	0	2	1	1	2.80E-06	2.65E-06	1.82E-06	2.25E-06
F16B4.5b	0	3	4	2	2.80E-06	2.65E-06	1.82E-06	2.25E-06
F16B4.6	7	7	7	3	2.80E-06	2.65E-06	1.82E-06	2.25E-06
F16B4.7	1	1	5	1	2.80E-06	2.65E-06	1.82E-06	2.25E-06
F16B4.8	127	87	237	297	2.80E-06	2.65E-06	1.82E-06	2.25E-06
F16B4.9	11	6	6	5	2.80E-06	2.65E-06	1.82E-06	2.25E-06
F16C3.1	20	18	13	34	9.55E-06	6.16E-06	1.16E-05	1.79E-05
F16C3.2	19	65	13	19	2.80E-06	2.65E-06	1.82E-06	2.25E-06
F16C3.3	9	10	15	4	2.80E-06	2.65E-06	1.82E-06	2.54E-06
F16C3.4	11	23	16	8	2.80E-06	6.35E-06	1.82E-06	2.25E-06
F16D3.1	5	9	13	12	2.80E-06	2.65E-06	1.82E-06	2.25E-06
F16D3.2.1	897	878	1296	1693	2.80E-06	4.58E-06	2.20E-06	2.25E-06
F16D3.2.2	753	759	1096	1433	2.80E-06	2.65E-06	1.82E-06	2.25E-06
F16D3.4	738	801	1119	1733	3.93E-05	3.64E-05	3.70E-05	5.96E-05
F16D3.6	2	5	8	4	3.34E-05	3.18E-05	3.17E-05	5.11E-05
F16D3.7	9	17	19	8	2.09E-05	2.14E-05	2.06E-05	3.94E-05
F16F9.1	45	47	29	28	2.80E-06	2.65E-06	1.82E-06	2.25E-06
F16F9.2	203	226	145	127	2.80E-06	2.65E-06	1.82E-06	2.25E-06
F16F9.3	15	6	3	17	5.32E-06	5.26E-06	2.24E-06	2.68E-06
F16F9.4	59	120	53	42	5.63E-06	5.92E-06	2.62E-06	2.83E-06
F16F9.5	20	28	19	14	4.03E-06	2.65E-06	1.82E-06	3.67E-06
F16G10.1	1	2	2	2	4.68E-06	8.97E-06	2.73E-06	2.68E-06
F16G10.10	3	0	2	1	2.80E-06	2.65E-06	1.82E-06	2.25E-06
F16G10.11	4	2	5	0	2.80E-06	2.65E-06	1.82E-06	2.25E-06
F16G10.13	1	1	0	1	2.80E-06	2.65E-06	1.82E-06	2.25E-06
F16G10.14	3	3	6	4	2.80E-06	2.65E-06	1.82E-06	2.25E-06
F16G10.15	7	11	8	4	2.80E-06	2.65E-06	1.82E-06	2.25E-06
F16G10.2	1	4	0	0	2.80E-06	2.65E-06	1.82E-06	2.25E-06
F16G10.3	0	0	3	0	2.80E-06	2.65E-06	1.82E-06	2.25E-06
F16G10.4	0	8	7	2	2.80E-06	2.65E-06	1.82E-06	2.25E-06
F16G10.5	2	7	2	1	2.80E-06	2.65E-06	1.82E-06	2.25E-06
F16G10.6	2	0	1	1	2.80E-06	2.65E-06	1.82E-06	2.25E-06
F16G10.7	1	0	0	0	2.80E-06	2.65E-06	1.82E-06	2.25E-06
F16G10.8	3	0	1	0	2.80E-06	2.65E-06	1.82E-06	2.25E-06
F16G10.9	3	0	0	0	2.80E-06	2.65E-06	1.82E-06	2.25E-06
F16H11.1	24	54	55	45	2.80E-06	2.65E-06	1.82E-06	2.25E-06
F16H11.2	135	95	313	402	2.80E-06	2.65E-06	1.82E-06	2.25E-06
F16H11.3	124	128	236	219	2.80E-06	3.44E-06	2.42E-06	2.45E-06
F16H11.4	0	0	3	1	8.69E-05	5.78E-05	1.31E-04	2.08E-04
F16H11.5	200	208	291	249	1.06E-05	1.04E-05	1.32E-05	1.51E-05
F16H6.1	22	49	27	37	2.80E-06	2.65E-06	1.82E-06	2.25E-06
F16H6.10	51	146	81	80	1.10E-05	1.08E-05	1.04E-05	1.10E-05
F16H6.2	2	2	0	2	2.80E-06	3.02E-06	1.82E-06	2.25E-06
F16H6.3	4	1	3	0	5.15E-06	1.39E-05	5.32E-06	6.50E-06
F16H6.4	0	4	5	1	2.80E-06	2.65E-06	1.82E-06	2.25E-06
F16H6.5	5	10	3	4	2.80E-06	2.65E-06	1.82E-06	2.25E-06
F16H6.6	0	1	0	0	2.80E-06	2.65E-06	1.82E-06	2.25E-06
F16H6.7	11	9	8	8	2.80E-06	2.65E-06	1.82E-06	2.25E-06
F16H6.8	3	0	10	4	2.80E-06	2.65E-06	1.82E-06	2.25E-06
F16H6.9	7	3	14	6	2.80E-06	2.65E-06	1.82E-06	2.25E-06
F16H9.1a	41	59	41	66	2.80E-06	2.65E-06	1.82E-06	2.25E-06
F16H9.1b	14	21	17	22	2.80E-06	2.65E-06	1.82E-06	2.25E-06
F16H9.2	0	5	7	5	2.94E-06	3.99E-06	1.91E-06	3.80E-06
F17A2.1	11	7	8	7	2.80E-06	3.73E-06	2.08E-06	3.33E-06
F17A2.10	10	14	15	10	2.80E-06	2.65E-06	1.82E-06	2.25E-06
F17A2.11	0	5	1	4	2.80E-06	2.65E-06	1.82E-06	2.25E-06
F17A2.12	5	6	6	3	2.80E-06	2.65E-06	1.82E-06	2.25E-06
F17A2.13	54	29	30	38	2.80E-06	2.65E-06	1.82E-06	2.25E-06

F17A2.3	4	10	9	3	2.80E-06	2.65E-06	1.82E-06	2.25E-06
F17A2.4	2	1	0	0	6.24E-06	3.17E-06	2.26E-06	3.53E-06
F17A2.5	23	24	29	28	2.80E-06	2.65E-06	1.82E-06	2.25E-06
F17A2.6	34	11	15	4	2.80E-06	2.65E-06	1.82E-06	2.25E-06
F17A2.7	2	12	11	5	2.80E-06	2.65E-06	2.13E-06	2.54E-06
F17A2.8	5	6	7	3	3.89E-06	2.65E-06	1.82E-06	2.25E-06
F17A2.9	4	5	6	1	2.80E-06	2.65E-06	1.82E-06	2.25E-06
F17A9.1	3	0	28	2	2.80E-06	2.65E-06	1.82E-06	2.25E-06
F17A9.2	425	413	461	548	2.80E-06	2.65E-06	1.82E-06	2.25E-06
F17A9.3	25	77	20	17	2.80E-06	2.65E-06	3.61E-06	2.25E-06
F17A9.4	111	102	86	97	2.75E-05	2.53E-05	1.94E-05	2.85E-05
F17A9.5	63	60	82	44	3.11E-06	9.07E-06	1.82E-06	2.25E-06
F17A9.6	96	103	115	119	7.81E-06	6.77E-06	3.94E-06	5.49E-06
F17B5.1a	93	94	57	40	5.21E-06	4.68E-06	4.41E-06	2.92E-06
F17B5.2	4	5	16	8	1.02E-05	1.03E-05	7.93E-06	1.01E-05
F17B5.3	3	5	2	2	4.12E-06	3.94E-06	1.82E-06	2.25E-06
F17B5.4	3	3	6	2	2.80E-06	2.65E-06	1.82E-06	2.25E-06
F17B5.5	6	0	1	3	2.80E-06	2.65E-06	1.82E-06	2.25E-06
F17B5.6	7	4	22	8	2.80E-06	2.65E-06	1.82E-06	2.25E-06
F17C11.1	120	226	151	199	2.80E-06	2.65E-06	1.82E-06	2.25E-06
F17C11.10	1146	1043	1942	2693	2.80E-06	2.65E-06	1.82E-06	2.25E-06
F17C11.11	57	454	49	138	2.03E-05	3.61E-05	1.66E-05	2.70E-05
F17C11.12	66	127	83	95	3.82E-05	3.29E-05	4.22E-05	7.22E-05
F17C11.13	4	7	1	1	7.28E-06	5.48E-05	4.06E-06	1.41E-05
F17C11.2.1	35	85	39	25	4.00E-06	7.27E-06	3.28E-06	4.63E-06
F17C11.2.2	30	77	33	25	2.80E-06	2.65E-06	1.82E-06	2.25E-06
F17C11.3	41	87	45	36	6.24E-06	1.43E-05	4.52E-06	3.58E-06
F17C11.4	70	196	56	123	5.63E-06	1.36E-05	4.03E-06	3.76E-06
F17C11.5	4	8	5	0	4.20E-06	8.44E-06	3.01E-06	2.97E-06
F17C11.6	40	85	86	35	1.00E-05	2.64E-05	5.19E-06	1.41E-05
F17C11.7a	378	431	568	735	2.80E-06	2.65E-06	1.82E-06	2.25E-06
F17C11.7b	356	428	542	695	1.10E-05	2.21E-05	1.54E-05	7.74E-06
F17C11.8.1	315	347	316	477	2.65E-05	2.85E-05	2.59E-05	4.14E-05
F17C11.8.2	301	332	292	465	2.31E-05	2.62E-05	2.29E-05	3.63E-05
F17C11.9a	12551	13541	14954	17411	2.16E-05	2.25E-05	1.41E-05	2.63E-05
F17C11.9c	10276	11370	12323	14271	2.90E-05	3.03E-05	1.83E-05	3.60E-05
F17C8.1	89	120	98	136	9.82E-04	1.00E-03	7.61E-04	1.09E-03
F17C8.2	27	58	27	29	9.95E-04	1.04E-03	7.76E-04	1.11E-03
F17C8.3	87	170	69	89	2.80E-06	3.12E-06	1.82E-06	3.01E-06
F17C8.4.1	143	303	154	210	3.28E-06	6.64E-06	2.13E-06	2.81E-06
F17C8.4.2	137	285	148	207	4.54E-06	8.36E-06	2.33E-06	3.71E-06
F17C8.4.3	109	236	102	167	2.09E-05	4.19E-05	1.47E-05	2.47E-05
F17C8.5	14	16	12	3	2.26E-05	4.44E-05	1.59E-05	2.74E-05
F17C8.6	11	10	13	5	1.91E-05	3.91E-05	1.16E-05	2.35E-05
F17C8.7	55	115	30	35	2.80E-06	2.65E-06	1.82E-06	2.25E-06
F17C8.8	30	43	30	29	2.80E-06	2.65E-06	1.82E-06	2.25E-06
F17E5.1a	51	63	85	126	6.92E-06	1.36E-05	2.46E-06	3.53E-06
F17E5.1b	34	50	65	91	6.58E-06	8.91E-06	4.28E-06	5.11E-06
F17E5.2	87	104	69	115	2.80E-06	2.65E-06	1.84E-06	3.37E-06
F17E9.1	8	16	4	14	2.80E-06	2.65E-06	2.02E-06	3.51E-06
F17E9.10	14	17	22	14	4.54E-06	5.13E-06	2.35E-06	4.84E-06
F17E9.11	10	28	11	30	2.80E-06	2.65E-06	1.82E-06	2.25E-06
F17E9.12	8	9	12	6	3.36E-06	3.84E-06	3.43E-06	2.70E-06
F17E9.13	12	10	32	6	2.80E-06	4.29E-06	1.82E-06	3.89E-06
F17E9.14	8	12	14	6	2.88E-06	3.04E-06	2.81E-06	2.25E-06
F17E9.2	17	20	21	34	3.50E-06	2.75E-06	6.07E-06	2.25E-06
F17E9.3	2	4	3	4	2.80E-06	2.65E-06	1.82E-06	2.25E-06
F17E9.4	448	622	738	570	3.75E-06	4.18E-06	3.02E-06	6.03E-06
F17E9.5	257	407	255	136	2.80E-06	2.65E-06	1.82E-06	2.25E-06

F17E9.7	3	6	5	0	1.17E-04	1.54E-04	1.26E-04	1.20E-04
F17E9.8	4	7	29	9	4.82E-05	7.21E-05	3.11E-05	2.05E-05
F17E9.9	9	10	12	3	2.80E-06	2.65E-06	1.82E-06	2.25E-06
F17H10.2a	125	251	71	178	2.80E-06	2.65E-06	1.82E-06	2.25E-06
F17H10.2b	92	169	48	116	2.80E-06	2.86E-06	2.37E-06	2.25E-06
F17H10.4	3	8	8	3	1.35E-05	2.57E-05	4.99E-06	1.55E-05
F17H10.t1	0	1	0	1	1.64E-05	2.84E-05	5.56E-06	1.66E-05
F18A1.1	27	27	29	21	2.80E-06	2.65E-06	1.82E-06	2.25E-06
F18A1.2.1	768	652	1133	1378	2.80E-06	2.65E-06	1.82E-06	2.25E-06
F18A1.2.2	781	679	1098	1378	2.80E-06	2.65E-06	1.82E-06	2.25E-06
F18A1.2.3	751	644	1077	1343	4.54E-05	3.64E-05	4.35E-05	6.54E-05
F18A1.3a.1	1041	963	1524	1799	4.02E-05	3.30E-05	3.68E-05	5.70E-05
F18A1.3a.2	274	315	384	423	4.43E-05	3.59E-05	4.14E-05	6.36E-05
F18A1.3b	269	301	383	412	7.34E-05	6.41E-05	6.99E-05	1.02E-04
F18A1.3c	219	237	332	348	2.56E-05	2.78E-05	2.33E-05	3.17E-05
F18A1.3d	263	304	385	415	2.69E-05	2.84E-05	2.49E-05	3.31E-05
F18A1.3e	269	301	383	412	3.05E-05	3.12E-05	3.01E-05	3.90E-05
F18A1.4a	79	124	86	133	2.59E-05	2.83E-05	2.47E-05	3.29E-05
F18A1.4b	78	122	85	129	2.69E-05	2.84E-05	2.49E-05	3.31E-05
F18A1.6a	438	497	724	1031	4.98E-06	7.41E-06	3.53E-06	6.75E-06
F18A1.6b.1	473	531	787	1088	5.10E-06	7.54E-06	3.61E-06	6.77E-06
F18A1.6b.2	460	509	748	1055	2.21E-05	2.38E-05	2.38E-05	4.19E-05
F18A1.7.1	1742	1487	1879	2260	2.12E-05	2.25E-05	2.30E-05	3.92E-05
F18A1.8	216	165	198	241	2.31E-05	2.42E-05	2.45E-05	4.26E-05
F18A11.2	21	38	21	17	2.32E-04	1.87E-04	1.63E-04	2.41E-04
F18A11.3	106	154	234	60	4.74E-05	3.42E-05	2.83E-05	4.25E-05
F18A11.5	14	22	15	8	2.80E-06	3.44E-06	1.82E-06	2.25E-06
F18A11.6	60	76	80	95	3.26E-05	4.48E-05	4.68E-05	1.48E-05
F18A11.7	4	2	4	2	2.80E-06	2.65E-06	1.82E-06	2.25E-06
F18A12.1	32	44	60	33	5.26E-06	6.30E-06	4.56E-06	6.68E-06
F18A12.2	11	10	4	5	2.80E-06	2.65E-06	1.82E-06	2.25E-06
F18A12.3	9	13	14	7	2.80E-06	2.65E-06	2.00E-06	2.25E-06
F18A12.4	26	31	17	16	2.80E-06	2.65E-06	1.82E-06	2.25E-06
F18A12.5	20	29	33	15	2.80E-06	2.65E-06	1.82E-06	2.25E-06
F18A12.6	16	26	22	22	2.80E-06	2.65E-06	1.82E-06	2.25E-06
F18A12.7	19	13	8	11	2.80E-06	2.65E-06	1.82E-06	2.25E-06
F18A12.8a	39	75	34	45	2.80E-06	2.65E-06	1.82E-06	2.25E-06
F18A12.8b	41	75	35	45	2.80E-06	2.65E-06	1.82E-06	2.25E-06
F18C12.1	70	112	115	85	2.80E-06	2.75E-06	1.82E-06	2.25E-06
F18C12.2a	1841	1775	2639	3850	2.80E-06	2.65E-06	1.82E-06	2.25E-06
F18C12.2b	1740	1666	2548	3708	2.80E-06	2.65E-06	1.82E-06	2.25E-06
F18C12.4	10	11	3	2	2.80E-05	2.55E-05	2.61E-05	4.70E-05
F18C5.1	9	10	12	11	2.86E-05	2.59E-05	2.72E-05	4.89E-05
F18C5.10.1	228	476	244	361	2.80E-06	2.65E-06	1.82E-06	2.25E-06
F18C5.10.2	167	327	143	231	2.80E-06	2.65E-06	1.82E-06	2.25E-06
F18C5.2	454	496	1572	1143	1.85E-05	3.64E-05	1.29E-05	2.35E-05
F18C5.3	744	764	710	1147	2.13E-05	3.93E-05	1.18E-05	2.36E-05
F18C5.4	32	51	23	28	1.55E-05	1.60E-05	3.49E-05	3.14E-05
F18C5.5	63	85	35	19	1.79E-05	1.74E-05	1.11E-05	2.22E-05
F18C5.6	11	6	11	7	3.84E-06	5.77E-06	1.82E-06	2.70E-06
F18C5.8	10	6	5	6	9.83E-06	1.25E-05	3.55E-06	2.38E-06
F18C5.9	4	3	2	2	2.80E-06	2.65E-06	1.82E-06	2.25E-06
F18C5.t1	1	0	3	0	2.80E-06	2.65E-06	1.82E-06	2.25E-06
F18E2.1	229	444	189	504	2.80E-06	2.65E-06	1.82E-06	2.25E-06
F18E2.2	821	1057	632	1144	2.80E-06	2.65E-06	2.99E-06	2.25E-06
F18E2.3	1180	1078	1885	2704	1.78E-05	3.26E-05	9.55E-06	3.14E-05
F18E2.4	2	2	4	3	4.56E-05	5.55E-05	2.28E-05	5.11E-05
F18E2.5	9	10	9	4	3.73E-05	3.22E-05	3.87E-05	6.86E-05
F18E2.6	1	3	0	1	2.80E-06	2.65E-06	1.82E-06	2.25E-06

F18E3.1	4	5	6	1	2.80E-06	2.65E-06	1.82E-06	2.25E-06
F18E3.10	2	3	5	3	2.80E-06	2.65E-06	1.82E-06	2.25E-06
F18E3.11	115	270	144	85	2.80E-06	2.65E-06	1.82E-06	2.25E-06
F18E3.2	2	5	0	1	2.80E-06	2.65E-06	1.82E-06	2.25E-06
F18E3.3	1	0	2	0	3.19E-05	7.07E-05	2.60E-05	1.89E-05
F18E3.4a	4	5	2	4	2.80E-06	2.65E-06	1.82E-06	2.25E-06
F18E3.4b	3	6	2	5	2.80E-06	2.65E-06	1.82E-06	2.25E-06
F18E3.5	2	3	0	1	2.80E-06	2.65E-06	1.82E-06	2.25E-06
F18E3.6	17	10	7	3	2.80E-06	2.65E-06	1.82E-06	2.25E-06
F18E3.7a	54	248	28	99	2.80E-06	2.65E-06	1.82E-06	2.25E-06
F18E3.7b	12	60	8	30	2.80E-06	2.65E-06	1.82E-06	2.25E-06
F18E3.9	1	12	7	5	5.88E-06	2.56E-05	1.99E-06	8.68E-06
F18E9.1	5	7	3	3	2.83E-06	1.33E-05	1.82E-06	5.67E-06
F18E9.2	16	29	28	27	2.80E-06	2.65E-06	1.82E-06	2.25E-06
F18E9.3	58	96	60	68	2.80E-06	2.65E-06	1.82E-06	2.25E-06
F18E9.4	11	32	26	21	4.90E-06	8.38E-06	5.58E-06	6.63E-06
F18E9.5a	98	128	62	104	4.73E-06	7.38E-06	3.19E-06	4.45E-06
F18E9.5b	117	150	83	128	2.80E-06	4.68E-06	2.62E-06	2.61E-06
F18E9.6	0	5	1	0	3.42E-06	4.21E-06	1.82E-06	2.90E-06
F18E9.7	1	2	5	3	3.64E-06	4.42E-06	1.82E-06	3.22E-06
F18E9.8	1	4	4	1	2.80E-06	2.65E-06	1.82E-06	2.25E-06
F18F11.1	51	98	50	78	2.80E-06	2.65E-06	1.82E-06	2.25E-06
F18F11.3	54	49	87	72	2.80E-06	2.65E-06	1.82E-06	2.25E-06
F18F11.4	21	43	19	7	7.84E-06	1.42E-05	4.99E-06	9.63E-06
F18F11.5	38	46	28	44	2.80E-06	2.65E-06	1.82E-06	2.25E-06
F18G5.1	3	0	2	2	2.80E-06	2.65E-06	1.82E-06	2.25E-06
F18G5.2	113	182	65	85	2.80E-06	2.99E-06	1.82E-06	2.45E-06
F18G5.3	44	66	28	25	2.80E-06	2.65E-06	1.82E-06	2.25E-06
F18G5.4	18	24	10	24	9.02E-06	1.37E-05	3.37E-06	5.44E-06
F18G5.5	2	2	4	1	4.56E-06	6.48E-06	1.90E-06	2.25E-06
F18G5.6	54	139	80	69	2.80E-06	2.65E-06	1.82E-06	2.25E-06
F18G5.t1	1	0	3	0	2.80E-06	2.65E-06	1.82E-06	2.25E-06
F18G5.t2	1	0	3	0	6.72E-06	1.63E-05	6.49E-06	6.90E-06
F18H3.1	22	28	33	30	2.80E-06	2.65E-06	3.04E-06	2.25E-06
F18H3.3a.1	961	973	710	1186	2.80E-06	2.65E-06	3.04E-06	2.25E-06
F18H3.3a.2	942	929	687	1166	4.03E-06	4.84E-06	3.94E-06	4.41E-06
F18H3.3a.3	941	932	689	1170	4.07E-05	3.90E-05	1.96E-05	4.04E-05
F18H3.3a.4	520	528	384	670	4.09E-05	3.81E-05	1.94E-05	4.06E-05
F18H3.3b	630	580	418	679	4.06E-05	3.80E-05	1.94E-05	4.06E-05
F18H3.4	89	125	139	143	2.80E-05	2.68E-05	1.34E-05	2.89E-05
F18H3.5a	34	41	50	93	3.29E-05	2.86E-05	1.42E-05	2.85E-05
F18H3.5b	29	42	43	84	5.24E-06	6.93E-06	5.32E-06	6.75E-06
F19B10.1	42	34	88	74	3.11E-06	3.54E-06	2.97E-06	6.81E-06
F19B10.10.1	159	156	161	191	2.80E-06	3.65E-06	2.57E-06	6.18E-06
F19B10.10.2	154	152	152	186	5.12E-06	3.91E-06	6.98E-06	7.24E-06
F19B10.11	5	3	11	3	8.34E-06	7.75E-06	5.50E-06	8.05E-06
F19B10.12	2	3	2	2	8.71E-06	8.12E-06	5.59E-06	8.43E-06
F19B10.2	34	32	65	39	2.80E-06	2.65E-06	1.82E-06	2.25E-06
F19B10.3	0	2	0	0	2.80E-06	2.65E-06	1.82E-06	2.25E-06
F19B10.4	10	10	12	8	5.21E-06	4.63E-06	6.49E-06	4.79E-06
F19B10.5	17	22	31	40	2.80E-06	2.65E-06	1.82E-06	2.25E-06
F19B10.6	0	1	1	0	2.80E-06	2.65E-06	1.82E-06	2.25E-06
F19B10.7	1	4	2	0	2.80E-06	2.65E-06	2.41E-06	3.82E-06
F19B10.8	3	7	3	8	2.80E-06	2.65E-06	1.82E-06	2.25E-06
F19B10.9	77	69	73	85	2.80E-06	2.65E-06	1.82E-06	2.25E-06
F19B2.1	9	8	6	6	2.80E-06	2.65E-06	1.82E-06	2.25E-06
F19B2.10	5	8	5	2	7.06E-06	5.98E-06	4.35E-06	6.25E-06
F19B2.2	5	8	5	3	2.80E-06	2.65E-06	1.82E-06	2.25E-06
F19B2.3	0	3	12	3	2.80E-06	2.65E-06	1.82E-06	2.25E-06

F19B2.4	8	6	9	4	2.80E-06	2.65E-06	1.82E-06	2.25E-06
F19B2.5	115	376	72	74	2.80E-06	2.65E-06	1.82E-06	2.25E-06
F19B2.6	24	31	25	24	2.80E-06	2.65E-06	1.82E-06	2.25E-06
F19B2.7	6	15	9	4	2.11E-05	6.52E-05	8.60E-06	1.09E-05
F19B2.8	4	5	10	3	2.80E-06	2.65E-06	1.82E-06	2.25E-06
F19B6.1a	1568	1682	1956	2443	2.80E-06	2.65E-06	1.82E-06	2.25E-06
F19B6.1b	853	872	1072	1373	2.80E-06	2.65E-06	1.82E-06	2.25E-06
F19B6.2a	438	459	526	699	5.03E-05	5.10E-05	4.08E-05	6.29E-05
F19B6.2b	426	451	520	697	5.72E-05	5.53E-05	4.68E-05	7.40E-05
F19B6.3	7	14	5	2	4.69E-05	4.64E-05	3.66E-05	6.01E-05
F19B6.4	39	74	32	23	4.28E-05	4.28E-05	3.40E-05	5.62E-05
F19C6.1	190	213	219	358	2.80E-06	2.65E-06	1.82E-06	2.25E-06
F19C6.2a	98	156	77	113	2.80E-06	3.65E-06	1.82E-06	2.25E-06
F19C6.2b	68	101	46	81	7.64E-06	8.09E-06	5.72E-06	1.16E-05
F19C6.3	6	12	9	12	5.68E-06	8.54E-06	2.90E-06	5.26E-06
F19C6.5	31	40	29	22	5.63E-06	7.91E-06	2.48E-06	5.40E-06
F19C7.1	944	1131	1231	1153	2.80E-06	2.65E-06	1.82E-06	2.25E-06
F19C7.2	75	74	72	71	4.59E-06	5.61E-06	2.79E-06	2.61E-06
F19C7.3	8	7	6	13	1.07E-04	1.21E-04	9.07E-05	1.05E-04
F19C7.4	26	17	55	32	4.73E-06	4.42E-06	2.95E-06	3.60E-06
F19C7.5	4	6	4	6	2.80E-06	2.65E-06	1.82E-06	2.25E-06
F19C7.6	3	3	3	3	2.80E-06	2.65E-06	2.41E-06	2.25E-06
F19C7.7	84	135	25	29	2.80E-06	2.65E-06	1.82E-06	2.25E-06
F19C7.8a	14	18	1	5	2.80E-06	2.65E-06	1.82E-06	2.25E-06
F19C7.8b	19	21	8	13	9.21E-06	1.40E-05	1.82E-06	2.56E-06
F19D8.1	6	11	9	4	2.80E-06	2.65E-06	1.82E-06	2.25E-06
F19D8.2	5	8	12	4	2.80E-06	2.65E-06	1.82E-06	2.25E-06
F19F10.1	4	5	7	1	2.80E-06	2.65E-06	1.82E-06	2.25E-06
F19F10.10	259	285	375	538	2.80E-06	2.65E-06	1.82E-06	2.25E-06
F19F10.11a	404	406	577	884	2.80E-06	2.65E-06	1.82E-06	2.25E-06
F19F10.11b	311	291	486	776	1.07E-05	1.11E-05	1.01E-05	1.78E-05
F19F10.12	297	284	319	451	1.29E-05	1.22E-05	1.20E-05	2.27E-05
F19F10.2	5	8	9	4	1.32E-05	1.16E-05	1.34E-05	2.64E-05
F19F10.3	17	14	33	6	1.61E-05	1.45E-05	1.12E-05	1.96E-05
F19F10.4	34	38	15	17	2.80E-06	2.65E-06	1.82E-06	2.25E-06
F19F10.5	6	8	18	2	4.82E-06	3.73E-06	6.07E-06	2.25E-06
F19F10.6	2	5	2	0	5.74E-06	6.06E-06	1.82E-06	2.29E-06
F19F10.7	0	0	1	0	2.80E-06	2.65E-06	1.82E-06	2.25E-06
F19F10.8	0	4	1	1	2.80E-06	2.65E-06	1.82E-06	2.25E-06
F19F10.9	674	723	461	956	2.80E-06	2.65E-06	1.82E-06	2.25E-06
F19G12.1	1	0	1	0	2.80E-06	2.65E-06	1.82E-06	2.25E-06
F19G12.2	6	4	9	6	2.82E-05	2.85E-05	1.25E-05	3.21E-05
F19G12.3	0	0	2	2	2.80E-06	2.65E-06	1.82E-06	2.25E-06
F19G12.4	2	2	2	0	2.80E-06	2.65E-06	1.82E-06	2.25E-06
F19G12.5	3	8	4	4	2.80E-06	2.65E-06	1.82E-06	2.25E-06
F19G12.7	7	7	8	6	2.80E-06	2.65E-06	1.82E-06	2.25E-06
F19G12.t1	5	2	2	2	2.80E-06	2.65E-06	1.82E-06	2.25E-06
F19H6.2	8	18	22	15	2.80E-06	2.65E-06	1.82E-06	2.25E-06
F19H6.3	15	20	26	9	6.92E-06	2.65E-06	1.82E-06	2.25E-06
F19H6.4	70	91	107	88	2.80E-06	2.72E-06	2.30E-06	2.25E-06
F19H6.5	6	3	4	2	2.80E-06	2.65E-06	1.82E-06	2.25E-06
F19H6.6	3	0	4	2	1.07E-05	1.31E-05	1.07E-05	1.08E-05
F19H6.7	2	7	1	3	2.80E-06	2.65E-06	1.82E-06	2.25E-06
F19H8.1	320	549	328	450	2.80E-06	2.65E-06	1.82E-06	2.25E-06
F19H8.2	72	135	66	55	2.80E-06	3.39E-06	1.82E-06	2.25E-06
F19H8.3	3	7	8	2	9.35E-06	1.51E-05	6.23E-06	1.05E-05
F19H8.4	165	184	92	69	8.85E-06	1.57E-05	5.27E-06	5.42E-06
F19H8.5	15	40	17	27	2.80E-06	2.65E-06	1.82E-06	2.25E-06
F20A1.1	132	142	346	66	6.86E-06	7.22E-06	2.48E-06	2.29E-06

F20A1.2	6	14	6	9	2.80E-06	2.75E-06	1.82E-06	2.25E-06
F20A1.3	2	2	3	3	3.46E-05	3.51E-05	5.89E-05	1.39E-05
F20A1.4	9	14	7	14	2.80E-06	2.65E-06	1.82E-06	2.25E-06
F20A1.6a	16	20	18	5	2.80E-06	2.65E-06	1.82E-06	2.25E-06
F20A1.6b	9	11	6	4	2.80E-06	2.65E-06	1.82E-06	2.25E-06
F20A1.7a	44	59	30	30	2.80E-06	2.65E-06	1.82E-06	2.25E-06
F20A1.7b	50	68	41	35	2.80E-06	2.65E-06	1.82E-06	2.25E-06
F20A1.7c	39	55	29	23	2.80E-06	2.65E-06	1.82E-06	2.25E-06
F20A1.8	1	1	2	2	2.80E-06	2.65E-06	1.82E-06	2.25E-06
F20A1.9	365	282	412	545	2.80E-06	2.65E-06	1.82E-06	2.25E-06
F20B10.1	77	96	60	105	2.80E-06	2.65E-06	1.82E-06	2.25E-06
F20B10.2	20	25	13	21	3.06E-05	2.23E-05	2.25E-05	3.67E-05
F20B10.3	16	17	3	7	2.80E-06	2.72E-06	1.82E-06	2.54E-06
F20B4.2	149	122	136	155	2.80E-06	3.23E-06	1.82E-06	2.32E-06
F20B4.3	0	2	6	3	2.80E-06	2.65E-06	1.82E-06	2.25E-06
F20B4.4	0	2	0	1	1.65E-05	1.27E-05	9.77E-06	1.37E-05
F20B4.6	20	52	23	51	2.80E-06	2.65E-06	1.82E-06	2.25E-06
F20B6.1	9	8	9	8	2.80E-06	2.65E-06	1.82E-06	2.25E-06
F20B6.2.1	3883	4996	5101	7064	2.80E-06	3.15E-06	1.82E-06	2.63E-06
F20B6.2.2	3926	5072	5163	7108	2.80E-06	2.65E-06	1.82E-06	2.25E-06
F20B6.3	76	88	68	121	2.29E-04	2.78E-04	1.96E-04	3.34E-04
F20B6.4	1	4	0	4	2.98E-04	3.63E-04	2.55E-04	4.33E-04
F20B6.5	9	3	4	4	2.80E-06	2.65E-06	1.82E-06	2.41E-06
F20B6.6	5	15	6	5	2.80E-06	2.65E-06	1.82E-06	2.25E-06
F20B6.7	11	12	6	9	2.80E-06	2.65E-06	1.82E-06	2.25E-06
F20B6.8a	277	361	283	645	2.80E-06	3.62E-06	1.82E-06	2.25E-06
F20B6.8b	536	640	481	995	2.80E-06	2.70E-06	1.82E-06	2.25E-06
F20B6.8c.1	522	625	467	980	1.26E-05	1.55E-05	8.36E-06	2.35E-05
F20B6.8c.2	520	621	462	974	1.54E-05	1.74E-05	8.98E-06	2.30E-05
F20B6.8c.3	536	640	481	995	1.61E-05	1.82E-05	9.37E-06	2.43E-05
F20B6.8c.4	522	622	462	974	1.66E-05	1.87E-05	9.58E-06	2.49E-05
F20B6.8c.5	520	621	462	974	1.54E-05	1.74E-05	8.98E-06	2.30E-05
F20B6.9	10	15	17	9	1.64E-05	1.84E-05	9.42E-06	2.45E-05
F20C5.1b	431	454	496	818	1.66E-05	1.87E-05	9.58E-06	2.49E-05
F20C5.1c	313	344	371	613	3.11E-06	4.42E-06	3.44E-06	2.25E-06
F20C5.2a	35	67	42	55	1.88E-05	1.87E-05	1.41E-05	2.87E-05
F20C5.2b	46	115	83	77	1.86E-05	1.94E-05	1.44E-05	2.93E-05
F20C5.3	84	98	422	248	2.80E-06	2.67E-06	1.82E-06	2.25E-06
F20C5.4	24	41	16	35	2.80E-06	3.36E-06	1.82E-06	2.25E-06
F20C5.5	25	28	22	13	1.36E-05	1.50E-05	4.44E-05	3.22E-05
F20C5.6	51	54	34	52	2.80E-06	3.99E-06	1.82E-06	2.90E-06
F20C5.7	4	3	8	3	2.80E-06	2.65E-06	1.82E-06	2.25E-06
F20D1.1.1	240	268	114	209	5.38E-06	5.37E-06	2.33E-06	4.41E-06
F20D1.1.2	181	179	78	164	2.80E-06	2.65E-06	1.82E-06	2.25E-06
F20D1.10.1	45	47	67	28	3.06E-05	3.23E-05	9.46E-06	2.14E-05
F20D1.10.2	38	44	45	20	2.93E-05	2.74E-05	8.24E-06	2.13E-05
F20D1.2	119	172	138	194	7.34E-06	7.22E-06	7.11E-06	3.67E-06
F20D1.3.1	394	536	538	765	1.11E-05	1.22E-05	8.58E-06	4.70E-06
F20D1.3.2	342	482	456	670	6.16E-06	8.41E-06	4.65E-06	8.05E-06
F20D1.4	278	377	227	352	2.84E-05	3.65E-05	2.53E-05	4.43E-05
F20D1.5	7	4	5	1	2.75E-05	3.66E-05	2.39E-05	4.33E-05
F20D1.6	161	218	181	285	5.38E-05	6.89E-05	2.86E-05	5.47E-05
F20D1.7	21	11	14	10	2.80E-06	2.65E-06	1.82E-06	2.25E-06
F20D1.8	14	28	34	16	6.22E-06	7.94E-06	4.54E-06	8.82E-06
F20D1.9	132	154	127	136	2.80E-06	2.65E-06	1.82E-06	2.25E-06
F20D12.1a	2633	2613	3565	5481	2.80E-06	2.65E-06	1.82E-06	2.25E-06
F20D12.1b	2219	2177	3006	4806	1.43E-05	1.58E-05	8.96E-06	1.19E-05
F20D12.2	705	729	688	1190	8.44E-05	7.91E-05	7.44E-05	1.41E-04
F20D12.3	16	11	28	8	9.55E-05	8.85E-05	8.41E-05	1.66E-04

F20D12.4.1	990	1134	1544	1832	2.27E-05	2.22E-05	1.44E-05	3.08E-05
F20D12.4.2	1050	1195	1625	1890	2.80E-06	2.65E-06	1.82E-06	2.25E-06
F20D12.4.3	1011	1155	1575	1842	4.35E-05	4.71E-05	4.42E-05	6.47E-05
F20D12.5	55	72	28	29	4.58E-05	4.92E-05	4.61E-05	6.61E-05
F20D12.6a	8	13	7	5	4.81E-05	5.19E-05	4.88E-05	7.04E-05
F20D12.6b	11	11	15	7	1.32E-05	1.63E-05	4.35E-06	5.58E-06
F20D12.7	5	5	2	1	2.80E-06	2.65E-06	1.82E-06	2.25E-06
F20D6.1	19	16	9	7	2.80E-06	2.65E-06	1.82E-06	2.25E-06
F20D6.10	27	42	18	31	2.80E-06	2.65E-06	1.82E-06	2.25E-06
F20D6.12	14	10	40	10	2.80E-06	2.65E-06	1.82E-06	2.25E-06
F20D6.2	6	7	10	10	2.80E-06	2.86E-06	1.82E-06	2.25E-06
F20D6.3	7	18	11	21	2.80E-06	2.65E-06	1.90E-06	2.25E-06
F20D6.4a	608	769	890	1161	2.80E-06	2.65E-06	1.82E-06	2.25E-06
F20D6.4c	535	688	755	964	2.80E-06	2.65E-06	1.82E-06	2.25E-06
F20D6.5	49	36	27	11	5.62E-05	6.72E-05	5.36E-05	8.62E-05
F20D6.6	34	45	18	15	5.45E-05	6.62E-05	5.01E-05	7.89E-05
F20D6.8	28	25	10	26	5.04E-06	3.49E-06	1.82E-06	2.25E-06
F20D6.9	1	1	0	1	4.70E-06	5.87E-06	1.82E-06	2.25E-06
F20E11.1	2	8	9	3	2.80E-06	2.65E-06	1.82E-06	2.25E-06
F20E11.10	3	8	3	0	2.80E-06	2.65E-06	1.82E-06	2.25E-06
F20E11.11	2	5	5	1	2.80E-06	2.65E-06	1.82E-06	2.25E-06
F20E11.12	3	4	4	1	2.80E-06	2.65E-06	1.82E-06	2.25E-06
F20E11.13	36	30	47	46	2.80E-06	2.65E-06	1.82E-06	2.25E-06
F20E11.14	4	9	6	3	2.80E-06	2.65E-06	1.82E-06	2.25E-06
F20E11.15	3	8	12	10	2.80E-06	2.65E-06	1.82E-06	2.25E-06
F20E11.16	16	14	7	4	2.80E-06	2.65E-06	1.82E-06	2.25E-06
F20E11.2	4	6	5	2	2.80E-06	2.65E-06	1.82E-06	2.25E-06
F20E11.3	5	12	19	6	2.80E-06	2.65E-06	1.82E-06	2.25E-06
F20E11.4	1	6	3	1	2.80E-06	2.65E-06	1.82E-06	2.25E-06
F20E11.5	1	1	0	0	2.80E-06	2.65E-06	1.82E-06	2.25E-06
F20E11.6	3	6	13	4	2.80E-06	2.65E-06	1.82E-06	2.25E-06
F20E11.7	1	1	1	1	2.80E-06	2.65E-06	1.82E-06	2.25E-06
F20E11.8	4	8	5	5	2.80E-06	2.65E-06	1.82E-06	2.25E-06
F20E11.9	9	7	16	8	2.80E-06	2.65E-06	1.82E-06	2.25E-06
F20G2.1	5	16	9	11	2.80E-06	2.65E-06	1.82E-06	2.25E-06
F20G2.2	174	209	366	247	2.80E-06	2.65E-06	1.82E-06	2.25E-06
F20G2.3	97	197	60	84	2.80E-06	2.65E-06	1.82E-06	2.25E-06
F20G2.4	4	5	1	2	2.46E-05	2.79E-05	3.36E-05	2.80E-05
F20G2.5	18	22	16	6	8.74E-06	1.68E-05	3.52E-06	6.07E-06
F20G2.6a	6	6	5	3	2.80E-06	2.65E-06	1.82E-06	2.25E-06
F20G2.6b	20	15	12	8	2.80E-06	2.65E-06	1.82E-06	2.25E-06
F20G4.1	810	937	970	1613	2.80E-06	2.65E-06	1.82E-06	2.25E-06
F20G4.2	146	103	138	253	2.80E-06	2.65E-06	1.82E-06	2.25E-06
F20G4.3	3719	3679	4760	7547	1.79E-05	1.96E-05	1.40E-05	2.87E-05
F20H11.1	197	196	296	291	1.14E-05	7.62E-06	7.03E-06	1.59E-05
F20H11.2	607	661	875	1812	6.34E-05	5.92E-05	5.28E-05	1.03E-04
F20H11.3.1	4290	3747	3435	3741	1.42E-05	1.34E-05	1.39E-05	1.69E-05
F20H11.3.2	3106	2763	2545	2908	1.32E-05	1.36E-05	1.24E-05	3.17E-05
F20H11.4	95	100	49	40	2.98E-04	2.46E-04	1.55E-04	2.09E-04
F20H11.5	339	416	304	476	2.66E-04	2.24E-04	1.42E-04	2.00E-04
F20H11.6	97	143	172	311	7.70E-06	7.64E-06	2.59E-06	2.61E-06
F21A10.2a.1	482	596	465	748	3.30E-05	3.82E-05	1.92E-05	3.72E-05
F21A10.2a.2	473	584	449	734	8.76E-06	1.22E-05	1.01E-05	2.25E-05
F21A10.2a.3	332	394	334	557	1.30E-05	1.52E-05	8.16E-06	1.62E-05
F21A10.2a.4	331	398	329	555	1.42E-05	1.66E-05	8.76E-06	1.77E-05
F21A10.2b	328	393	329	556	1.25E-05	1.40E-05	8.18E-06	1.68E-05
F21A10.2c	331	398	329	555	1.28E-05	1.45E-05	8.29E-06	1.72E-05
F21A10.4	7	8	6	3	1.29E-05	1.46E-05	8.44E-06	1.76E-05
F21A3.1	4	5	7	2	1.28E-05	1.45E-05	8.29E-06	1.72E-05

F21A3.2a	35	83	34	67	2.80E-06	2.65E-06	1.82E-06	2.25E-06
F21A3.2b	29	77	29	60	2.80E-06	2.65E-06	1.82E-06	2.25E-06
F21A3.3	63	81	41	47	2.80E-06	5.63E-06	1.82E-06	3.87E-06
F21A3.4	2	14	5	5	2.80E-06	4.97E-06	1.82E-06	3.31E-06
F21A3.5	99	116	67	138	6.61E-06	8.04E-06	2.81E-06	3.96E-06
F21A3.6	7	10	6	2	2.80E-06	2.65E-06	1.82E-06	2.25E-06
F21A3.7	15	28	14	18	6.94E-06	7.67E-06	3.06E-06	7.76E-06
F21A3.8	4	11	3	2	2.80E-06	2.65E-06	1.82E-06	2.25E-06
F21A9.1	2	0	1	2	2.80E-06	2.65E-06	1.82E-06	2.25E-06
F21A9.2	15	13	14	11	2.80E-06	2.65E-06	1.82E-06	2.25E-06
F21C10.1	1	2	2	0	2.80E-06	2.65E-06	1.82E-06	2.25E-06
F21C10.10.1	128	276	217	138	2.80E-06	2.65E-06	1.82E-06	2.25E-06
F21C10.10.2	109	248	177	130	2.80E-06	2.65E-06	1.82E-06	2.25E-06
F21C10.11a	19	24	81	19	1.38E-05	2.81E-05	1.52E-05	1.19E-05
F21C10.11b	2	9	27	5	1.78E-05	3.84E-05	1.89E-05	1.71E-05
F21C10.12	5	7	11	4	8.76E-06	1.04E-05	2.43E-05	7.04E-06
F21C10.3a	9	23	7	9	2.80E-06	7.56E-06	1.56E-05	3.58E-06
F21C10.3b	7	24	6	9	2.80E-06	2.65E-06	1.82E-06	2.25E-06
F21C10.4	7	10	2	4	2.80E-06	2.86E-06	1.82E-06	2.25E-06
F21C10.5	3	3	5	2	2.80E-06	2.96E-06	1.82E-06	2.25E-06
F21C10.6	1	0	1	1	2.80E-06	2.65E-06	1.82E-06	2.25E-06
F21C10.7	661	821	281	743	2.80E-06	2.65E-06	1.82E-06	2.25E-06
F21C10.8a	97	122	58	68	2.80E-06	2.65E-06	1.82E-06	2.25E-06
F21C10.8b	96	122	59	67	9.55E-06	1.12E-05	2.64E-06	8.61E-06
F21C3.1	41	82	21	43	9.35E-06	1.11E-05	3.63E-06	5.26E-06
F21C3.2	22	36	20	16	9.32E-06	1.12E-05	3.74E-06	5.22E-06
F21C3.3	323	370	423	300	3.19E-06	6.03E-06	1.82E-06	2.68E-06
F21C3.4a	281	323	371	480	2.80E-06	2.65E-06	1.82E-06	2.25E-06
F21C3.4b	268	302	367	461	9.21E-05	9.96E-05	7.84E-05	6.87E-05
F21C3.5	327	382	1148	550	1.71E-05	1.86E-05	1.47E-05	2.35E-05
F21C3.6	12	15	11	6	1.75E-05	1.86E-05	1.56E-05	2.42E-05
F21C3.t1	0	0	2	0	6.20E-05	6.84E-05	1.42E-04	8.37E-05
F21D12.1a	54	66	36	51	2.80E-06	2.83E-06	1.82E-06	2.25E-06
F21D12.1b	52	66	33	46	2.80E-06	2.65E-06	2.00E-06	2.25E-06
F21D12.1c.1	54	64	33	51	4.06E-06	4.71E-06	1.82E-06	3.08E-06
F21D12.1c.2	55	65	37	53	4.09E-06	4.92E-06	1.82E-06	2.92E-06
F21D12.1d	57	68	35	50	4.54E-06	5.08E-06	1.82E-06	3.44E-06
F21D12.1e	54	58	38	52	4.45E-06	4.97E-06	1.95E-06	3.46E-06
F21D12.2	1	4	12	2	4.20E-06	4.73E-06	1.82E-06	2.95E-06
F21D12.3	99	223	62	137	4.37E-06	4.42E-06	2.00E-06	3.37E-06
F21D12.5	4	5	6	1	2.80E-06	2.65E-06	2.33E-06	2.25E-06
F21D5.1.1	840	1043	1275	1737	5.52E-06	1.17E-05	2.24E-06	6.12E-06
F21D5.1.2	687	819	1028	1432	2.80E-06	2.65E-06	1.82E-06	2.25E-06
F21D5.2.1	529	630	493	844	4.72E-05	5.53E-05	4.66E-05	7.83E-05
F21D5.2.2	391	454	365	597	4.65E-05	5.23E-05	4.53E-05	7.78E-05
F21D5.3.1	171	392	168	311	4.39E-05	4.94E-05	2.66E-05	5.63E-05
F21D5.3.2	162	371	153	286	4.56E-05	5.00E-05	2.77E-05	5.59E-05
F21D5.3.3	162	375	153	287	7.98E-06	1.73E-05	5.10E-06	1.16E-05
F21D5.4	36	33	35	23	8.09E-06	1.75E-05	4.97E-06	1.15E-05
F21D5.5	234	302	138	300	8.09E-06	1.77E-05	4.97E-06	1.15E-05
F21D5.6	305	395	348	435	5.18E-06	4.47E-06	3.26E-06	2.65E-06
F21D5.7.1	629	975	528	997	1.94E-05	2.37E-05	7.45E-06	2.00E-05
F21D5.7.2	543	814	475	900	3.42E-05	4.18E-05	2.54E-05	3.91E-05
F21D5.8	133	155	199	100	3.72E-05	5.45E-05	2.03E-05	4.74E-05
F21D5.9	5	7	1	7	4.06E-05	5.75E-05	2.31E-05	5.41E-05
F21D9.1	6	8	9	8	3.91E-05	4.30E-05	3.81E-05	2.36E-05
F21D9.2	16	30	11	19	2.80E-06	2.65E-06	1.82E-06	2.25E-06
F21D9.3	2	1	0	0	2.80E-06	2.65E-06	1.82E-06	2.25E-06
F21D9.4	3	5	7	4	2.80E-06	3.09E-06	1.82E-06	2.25E-06

F21D9.5	9	14	17	11	2.80E-06	2.65E-06	1.82E-06	2.25E-06
F21D9.6	6	6	6	8	2.80E-06	2.65E-06	1.82E-06	2.25E-06
F21D9.7	12	16	15	13	2.80E-06	2.65E-06	1.82E-06	2.25E-06
F21D9.8	3	1	0	1	2.80E-06	2.65E-06	1.82E-06	2.25E-06
F21E9.2	3	4	2	3	2.80E-06	2.65E-06	1.82E-06	2.25E-06
F21E9.3	14	67	20	15	2.80E-06	2.65E-06	1.82E-06	2.25E-06
F21E9.4	4	1	3	0	2.80E-06	2.65E-06	1.82E-06	2.25E-06
F21E9.6	2	1	1	0	2.80E-06	1.26E-05	2.61E-06	2.41E-06
F21F12.1	4	2	5	5	2.80E-06	2.65E-06	1.82E-06	2.25E-06
F21F3.2	24	35	16	4	2.80E-06	2.65E-06	1.82E-06	2.25E-06
F21F3.3	18	51	12	20	2.80E-06	2.65E-06	1.82E-06	2.25E-06
F21F3.4	55	82	57	89	2.80E-06	2.72E-06	1.82E-06	2.25E-06
F21F3.5	38	39	27	32	2.80E-06	5.69E-06	1.82E-06	2.25E-06
F21F3.6.1	1191	1496	1397	1950	5.15E-06	7.25E-06	3.46E-06	6.68E-06
F21F3.6.2	726	843	731	1042	2.80E-06	2.65E-06	1.82E-06	2.25E-06
F21F3.7	76	76	108	121	1.15E-04	1.36E-04	8.75E-05	1.51E-04
F21F8.1	2	9	3	1	1.15E-04	1.26E-04	7.53E-05	1.32E-04
F21F8.10	4	6	9	2	1.68E-05	1.59E-05	1.55E-05	2.15E-05
F21F8.11	50	68	29	67	2.80E-06	2.65E-06	1.82E-06	2.25E-06
F21F8.2	5	8	0	1	2.80E-06	2.65E-06	1.82E-06	2.25E-06
F21F8.3.1	3993	4503	5748	5557	3.53E-06	4.52E-06	1.82E-06	3.78E-06
F21F8.3.2	3710	4103	5313	5366	2.80E-06	2.65E-06	1.82E-06	2.25E-06
F21F8.4.1	56	53	61	66	3.60E-04	3.83E-04	3.37E-04	4.02E-04
F21F8.4.2	52	51	57	62	3.51E-04	3.67E-04	3.27E-04	4.08E-04
F21F8.5	5	11	4	2	4.73E-06	4.23E-06	3.35E-06	4.48E-06
F21F8.6	4	10	12	6	4.90E-06	4.52E-06	3.50E-06	4.68E-06
F21F8.7.1	7189	6818	9623	11558	2.80E-06	3.33E-06	1.82E-06	2.25E-06
F21F8.7.2	6628	6243	9116	11255	2.80E-06	2.65E-06	1.82E-06	2.25E-06
F21F8.8	0	0	5	6	6.49E-04	5.82E-04	5.66E-04	8.39E-04
F21F8.9	4	6	6	2	6.33E-04	5.64E-04	5.67E-04	8.64E-04
F21G4.1	54	81	76	63	2.80E-06	2.65E-06	1.82E-06	2.25E-06
F21G4.2	1852	1693	3436	3594	2.80E-06	2.65E-06	1.82E-06	2.25E-06
F21G4.3	8	4	3	3	3.14E-06	4.44E-06	2.86E-06	2.92E-06
F21G4.4	27	37	33	25	4.15E-05	3.58E-05	5.01E-05	6.46E-05
F21G4.5	19	18	14	13	2.80E-06	2.65E-06	1.82E-06	2.25E-06
F21G4.6	88	131	99	184	2.80E-06	3.25E-06	2.00E-06	2.25E-06
F21H11.2a	1116	1062	1703	2057	2.80E-06	2.65E-06	1.82E-06	2.25E-06
F21H11.2b	349	334	436	524	2.80E-06	2.65E-06	1.82E-06	2.88E-06
F21H11.3.1	70	69	87	63	1.42E-05	1.28E-05	1.41E-05	2.11E-05
F21H11.3.2	78	73	97	73	1.65E-05	1.49E-05	1.34E-05	1.99E-05
F21H12.1	245	330	306	349	4.79E-06	4.44E-06	3.86E-06	3.46E-06
F21H12.2	4	6	8	21	4.79E-06	4.23E-06	3.88E-06	3.60E-06
F21H12.3	3	2	2	0	1.97E-05	2.51E-05	1.60E-05	2.25E-05
F21H12.4	631	598	1443	2253	2.80E-06	3.25E-06	2.99E-06	9.69E-06
F21H12.5	332	359	429	520	2.80E-06	2.65E-06	1.82E-06	2.25E-06
F21H12.6	1760	1807	2257	3169	3.53E-05	3.16E-05	5.25E-05	1.01E-04
F21H12.7	5	8	7	3	1.64E-05	1.67E-05	1.38E-05	2.06E-05
F21H7.1	34	25	24	16	4.47E-05	4.34E-05	3.73E-05	6.47E-05
F21H7.10	2	2	6	9	2.80E-06	2.65E-06	1.82E-06	2.25E-06
F21H7.11	2	2	5	2	5.80E-06	4.02E-06	2.66E-06	2.25E-06
F21H7.12	6	13	3	0	2.80E-06	2.65E-06	1.82E-06	2.25E-06
F21H7.13	3	6	4	6	2.80E-06	2.65E-06	1.82E-06	2.25E-06
F21H7.14	8	2	9	1	2.80E-06	2.65E-06	1.82E-06	2.25E-06
F21H7.2.1	56	72	31	30	2.80E-06	2.65E-06	1.82E-06	2.25E-06
F21H7.2.2	60	76	36	35	2.80E-06	2.65E-06	1.82E-06	2.25E-06
F21H7.3	6	10	9	9	4.03E-06	4.89E-06	1.82E-06	2.25E-06
F21H7.4	2	4	11	4	3.72E-06	4.47E-06	1.82E-06	2.25E-06
F21H7.5	107	189	90	67	2.80E-06	2.65E-06	1.82E-06	2.25E-06
F21H7.6	4	6	7	4	2.80E-06	2.65E-06	1.82E-06	2.25E-06

F21H7.7	7	5	10	5	1.11E-05	1.84E-05	6.05E-06	5.56E-06
F21H7.9	10	7	14	6	2.80E-06	2.65E-06	1.82E-06	2.25E-06
F22A3.1	112	202	79	147	2.80E-06	2.65E-06	1.82E-06	2.25E-06
F22A3.2	119	172	128	88	2.80E-06	2.65E-06	1.82E-06	2.25E-06
F22A3.3	9	7	8	5	6.47E-06	1.10E-05	2.97E-06	6.84E-06
F22A3.5	18	38	14	34	2.44E-05	3.33E-05	1.71E-05	1.45E-05
F22A3.6a	2226	2447	5492	2116	2.80E-06	2.65E-06	1.82E-06	2.25E-06
F22A3.6b	1012	1151	2780	790	2.80E-06	4.92E-06	1.82E-06	3.73E-06
F22A3.7	126	253	187	93	5.94E-04	6.16E-04	9.53E-04	4.53E-04
F22B3.1	25	24	118	24	1.76E-04	1.89E-04	3.14E-04	1.10E-04
F22B3.2	35	53	76	64	3.56E-05	6.74E-05	3.43E-05	2.11E-05
F22B3.4	2931	2936	5743	6034	7.34E-06	6.67E-06	2.26E-05	5.67E-06
F22B3.5	2	2	1	0	9.55E-06	1.36E-05	1.35E-05	1.40E-05
F22B3.7	3	1	1	1	1.43E-04	1.35E-04	1.82E-04	2.36E-04
F22B3.8	11	24	6	6	2.80E-06	2.65E-06	1.82E-06	2.25E-06
F22B3.9	1	5	9	3	2.80E-06	2.65E-06	1.82E-06	2.25E-06
F22B5.1	123	107	93	109	2.80E-06	2.65E-06	1.82E-06	2.25E-06
F22B5.2	413	421	760	682	2.80E-06	2.65E-06	1.82E-06	2.25E-06
F22B5.3	48	33	27	82	2.48E-05	2.04E-05	1.22E-05	1.77E-05
F22B5.4	10	21	10	10	4.08E-05	3.93E-05	4.89E-05	5.42E-05
F22B5.5	26	52	19	15	4.23E-06	2.75E-06	1.82E-06	5.80E-06
F22B5.6	19	25	17	10	2.80E-06	4.42E-06	1.82E-06	2.25E-06
F22B5.7	1817	1543	2678	3395	2.86E-06	5.40E-06	1.82E-06	2.25E-06
F22B5.9	1097	1102	1390	1699	3.16E-06	3.94E-06	1.84E-06	2.25E-06
F22B7.1	11	25	11	13	4.50E-05	3.61E-05	4.32E-05	6.76E-05
F22B7.10	43	97	55	76	6.45E-05	6.12E-05	5.32E-05	8.02E-05
F22B7.13	178	256	179	279	2.80E-06	2.65E-06	1.82E-06	2.25E-06
F22B7.2	4	5	4	3	2.80E-06	5.00E-06	1.95E-06	3.33E-06
F22B7.3	1	4	0	0	1.18E-05	1.60E-05	7.73E-06	1.48E-05
F22B7.4	1	2	0	0	2.80E-06	2.65E-06	1.82E-06	2.25E-06
F22B7.5a.1	302	342	196	299	2.80E-06	2.65E-06	1.82E-06	2.25E-06
F22B7.5a.2	248	285	164	262	2.80E-06	2.65E-06	1.82E-06	2.25E-06
F22B7.5b	284	319	186	285	2.07E-05	2.22E-05	8.76E-06	1.65E-05
F22B7.6	289	399	337	586	2.01E-05	2.18E-05	8.66E-06	1.71E-05
F22B7.7	28	34	44	43	2.06E-05	2.18E-05	8.78E-06	1.66E-05
F22B7.9	32	338	35	32	1.99E-05	2.59E-05	1.51E-05	3.23E-05
F22B7.t1	0	1	0	0	2.80E-06	2.65E-06	1.82E-06	2.25E-06
F22B8.1	9	6	4	3	3.72E-06	3.73E-05	2.66E-06	2.99E-06
F22B8.2	6	7	8	5	2.80E-06	2.65E-06	1.82E-06	2.25E-06
F22B8.3	7	3	10	7	2.80E-06	2.65E-06	1.82E-06	2.25E-06
F22B8.4	20	14	20	16	2.80E-06	2.65E-06	1.82E-06	2.25E-06
F22B8.5	7	19	10	9	2.80E-06	2.65E-06	1.82E-06	2.25E-06
F22B8.6.1	407	454	402	340	2.80E-06	2.65E-06	1.82E-06	2.25E-06
F22B8.6.2	390	437	396	328	2.80E-06	2.65E-06	1.82E-06	2.25E-06
F22B8.7	140	185	131	146	3.57E-05	3.76E-05	2.29E-05	2.39E-05
F22B8.8	9	3	7	2	3.65E-05	3.86E-05	2.41E-05	2.46E-05
F22D3.1a	624	642	924	1332	1.46E-05	1.83E-05	8.91E-06	1.23E-05
F22D3.1b	863	869	1276	1928	2.80E-06	2.65E-06	1.82E-06	2.25E-06
F22D3.2a	546	591	775	955	3.68E-05	3.58E-05	3.55E-05	6.31E-05
F22D3.2b	333	348	473	582	3.55E-05	3.38E-05	3.41E-05	6.37E-05
F22D3.2c	128	141	208	233	1.91E-05	1.95E-05	1.77E-05	2.69E-05
F22D3.4	15	18	24	19	1.86E-05	1.84E-05	1.72E-05	2.61E-05
F22D3.5	22	19	44	12	1.50E-05	1.57E-05	1.59E-05	2.20E-05
F22D3.6	180	214	236	297	2.80E-06	2.65E-06	1.82E-06	2.25E-06
F22D6.1	36	86	46	47	1.64E-05	1.34E-05	2.14E-05	7.20E-06
F22D6.10	272	291	112	107	9.97E-06	1.12E-05	8.51E-06	1.32E-05
F22D6.11	2	14	9	15	3.19E-06	7.17E-06	2.64E-06	3.33E-06
F22D6.12.1	53	56	30	49	3.35E-05	3.39E-05	8.98E-06	1.06E-05
F22D6.12.2	50	55	29	42	2.80E-06	2.65E-06	1.82E-06	2.25E-06

F22D6.14	68	64	225	91	3.86E-06	3.84E-06	1.82E-06	2.86E-06
F22D6.15	28	23	14	9	3.86E-06	4.02E-06	1.82E-06	2.61E-06
F22D6.2.1	1664	1844	3161	3293	2.86E-05	2.54E-05	6.16E-05	3.08E-05
F22D6.2.2	713	773	1457	1421	8.18E-06	6.35E-06	2.66E-06	2.25E-06
F22D6.3a.1	1194	1236	1607	1930	1.32E-04	1.38E-04	1.64E-04	2.10E-04
F22D6.3a.2	1118	1143	1479	1839	1.35E-04	1.39E-04	1.80E-04	2.17E-04
F22D6.4.1	408	525	684	307	7.44E-05	7.28E-05	6.52E-05	9.66E-05
F22D6.4.2	370	489	469	254	7.62E-05	7.36E-05	6.56E-05	1.01E-04
F22D6.5	773	1012	472	1180	8.75E-05	1.06E-04	9.55E-05	5.29E-05
F22D6.6	413	483	581	769	7.78E-05	9.71E-05	6.41E-05	4.29E-05
F22D6.7	7	4	7	4	3.50E-05	4.33E-05	1.39E-05	4.29E-05
F22D6.8	16	28	18	11	2.22E-05	2.45E-05	2.03E-05	3.32E-05
F22D6.9	75	126	49	99	5.80E-06	3.15E-06	3.77E-06	2.68E-06
F22E10.1	27	32	35	22	5.91E-06	9.79E-06	4.34E-06	3.26E-06
F22E10.2	25	58	47	50	6.30E-06	1.00E-05	2.68E-06	6.70E-06
F22E10.4	22	18	22	10	2.80E-06	2.65E-06	1.82E-06	2.25E-06
F22E10.5	133	343	137	269	2.80E-06	2.65E-06	1.82E-06	2.25E-06
F22E12.1	202	317	186	332	2.80E-06	2.65E-06	1.82E-06	2.25E-06
F22E12.2	29	63	17	32	1.22E-05	2.97E-05	8.18E-06	1.98E-05
F22E12.3	12	11	9	5	8.60E-06	1.27E-05	5.14E-06	1.13E-05
F22E12.4a.1	150	185	159	262	5.21E-06	1.07E-05	1.99E-06	4.61E-06
F22E12.4a.2	123	152	137	236	2.80E-06	2.65E-06	1.82E-06	2.25E-06
F22E12.4b.1	153	176	162	257	6.30E-06	7.33E-06	4.34E-06	8.82E-06
F22E12.4b.2	64	73	73	120	6.33E-06	7.38E-06	4.57E-06	9.74E-06
F22E12.4c	70	89	83	140	6.52E-06	7.09E-06	4.48E-06	8.79E-06
F22E12.4d	145	177	156	256	6.52E-06	7.01E-06	4.83E-06	9.81E-06
F22E12.4e.1	123	152	137	236	5.60E-06	6.74E-06	4.32E-06	9.02E-06
F22E12.4e.2	112	138	127	229	6.38E-06	7.35E-06	4.46E-06	9.06E-06
F22E5.1	45	42	32	39	6.33E-06	7.38E-06	4.57E-06	9.74E-06
F22E5.10	1	2	3	4	6.38E-06	7.41E-06	4.70E-06	1.05E-05
F22E5.11	3	3	13	5	4.54E-06	3.99E-06	2.10E-06	3.15E-06
F22E5.12	1	1	1	1	2.80E-06	2.65E-06	1.82E-06	2.25E-06
F22E5.13	16	30	19	18	2.80E-06	2.65E-06	1.82E-06	2.25E-06
F22E5.14	5	2	4	0	2.80E-06	2.65E-06	1.82E-06	2.25E-06
F22E5.15	4	4	9	6	2.80E-06	3.09E-06	1.82E-06	2.25E-06
F22E5.16	6	5	1	2	2.80E-06	2.65E-06	1.82E-06	2.25E-06
F22E5.17	53	75	101	93	2.80E-06	2.65E-06	1.82E-06	2.25E-06
F22E5.18	8	2	8	1	2.80E-06	2.65E-06	1.82E-06	2.25E-06
F22E5.19	2	1	2	0	6.64E-06	8.89E-06	8.24E-06	9.36E-06
F22E5.2	2	3	3	0	2.80E-06	2.65E-06	1.82E-06	2.25E-06
F22E5.20	9	6	10	4	2.80E-06	2.65E-06	1.82E-06	2.25E-06
F22E5.21	6	5	22	9	2.80E-06	2.65E-06	1.82E-06	2.25E-06
F22E5.3	12	26	26	13	2.80E-06	2.65E-06	1.82E-06	2.25E-06
F22E5.4	3	8	6	3	2.80E-06	2.65E-06	1.86E-06	2.25E-06
F22E5.5	4	4	4	0	2.80E-06	2.65E-06	1.82E-06	2.25E-06
F22E5.6	2	1	9	4	2.80E-06	2.65E-06	1.82E-06	2.25E-06
F22E5.7	3	4	3	5	2.80E-06	2.65E-06	1.82E-06	2.25E-06
F22E5.8	5	8	5	1	2.80E-06	2.65E-06	1.82E-06	2.25E-06
F22E5.9	62	64	96	93	2.80E-06	2.65E-06	1.82E-06	2.25E-06
F22F1.1	95	85	67	136	2.80E-06	2.65E-06	1.82E-06	2.25E-06
F22F1.2	19	17	29	23	1.45E-05	1.41E-05	1.46E-05	1.74E-05
F22F1.3	10	7	7	8	1.22E-05	1.03E-05	5.58E-06	1.40E-05
F22F1.t6	0	0	0	1	2.80E-06	2.65E-06	2.35E-06	2.29E-06
F22F1.t7	0	0	1	0	2.80E-06	2.65E-06	1.82E-06	2.25E-06
F22F4.1	136	215	55	40	2.80E-06	2.65E-06	1.82E-06	2.25E-06
F22F4.3	21	16	25	20	2.80E-06	2.65E-06	1.82E-06	2.25E-06
F22F4.4	363	282	342	128	8.01E-06	1.20E-05	2.11E-06	2.25E-06
F22F4.5	16	26	18	34	2.80E-06	2.65E-06	1.82E-06	2.25E-06
F22F7.1a.1	1397	1635	1670	2253	4.60E-05	3.38E-05	2.82E-05	1.30E-05

F22F7.1a.2	1288	1529	1550	2130	3.02E-06	4.66E-06	2.22E-06	5.17E-06
F22F7.1b	1322	1551	1561	2154	1.11E-04	1.22E-04	8.61E-05	1.43E-04
F22F7.2	12	13	25	4	1.13E-04	1.26E-04	8.81E-05	1.49E-04
F22F7.3	5	9	5	6	1.01E-04	1.12E-04	7.79E-05	1.33E-04
F22F7.4	3	7	9	3	2.80E-06	2.65E-06	1.82E-06	2.25E-06
F22F7.5	53	85	72	78	2.80E-06	2.65E-06	1.82E-06	2.25E-06
F22F7.6	6	4	3	2	2.80E-06	2.65E-06	1.82E-06	2.25E-06
F22F7.7.1	77	70	145	84	5.18E-06	7.86E-06	4.57E-06	6.12E-06
F22F7.7.2	76	69	145	84	2.80E-06	2.65E-06	1.82E-06	2.25E-06
F22G12.1	4	4	0	6	6.27E-06	5.37E-06	7.67E-06	5.49E-06
F22G12.3	3	5	2	2	6.61E-06	5.66E-06	8.20E-06	5.87E-06
F22G12.4	224	234	336	396	2.80E-06	2.65E-06	1.82E-06	2.25E-06
F22G12.5	970	785	1154	1829	2.80E-06	2.65E-06	1.82E-06	2.25E-06
F22G12.6	60	66	74	97	9.24E-06	9.13E-06	9.02E-06	1.31E-05
F22G12.7	11	13	11	4	1.48E-05	1.13E-05	1.14E-05	2.24E-05
F22G12.8	16	12	9	11	8.09E-06	8.41E-06	6.49E-06	1.05E-05
F22H10.1	0	3	3	0	2.80E-06	2.65E-06	1.82E-06	2.25E-06
F22H10.2	23	17	55	25	2.80E-06	2.65E-06	1.82E-06	2.25E-06
F22H10.4	6	2	26	29	2.80E-06	2.65E-06	1.82E-06	2.25E-06
F22H10.5	9	5	4	6	8.34E-06	5.82E-06	1.30E-05	7.29E-06
F22H10.6	96	59	124	131	2.80E-06	2.65E-06	2.57E-06	3.55E-06
F22H10.t1	3	2	3	2	2.80E-06	2.65E-06	1.82E-06	2.25E-06
F23A7.1	2	4	6	6	1.63E-05	9.47E-06	1.37E-05	1.79E-05
F23A7.2	4	3	8	3	4.09E-06	2.65E-06	2.66E-06	2.25E-06
F23A7.3	12	5	6	13	2.80E-06	2.65E-06	1.82E-06	2.25E-06
F23A7.5	18	14	8	11	2.80E-06	2.65E-06	1.82E-06	2.25E-06
F23A7.6	12	15	7	12	2.80E-06	2.65E-06	1.82E-06	2.25E-06
F23A7.7	22	78	27	49	2.80E-06	2.65E-06	1.82E-06	2.25E-06
F23A7.8	1754	1288	4068	824	2.80E-06	2.65E-06	1.82E-06	2.25E-06
F23A7.t1	1	0	3	0	2.80E-06	2.86E-06	1.82E-06	2.25E-06
F23B12.1	22	32	25	18	8.81E-04	6.11E-04	1.33E-03	3.32E-04
F23B12.2	6	5	3	2	2.80E-06	2.65E-06	2.99E-06	2.25E-06
F23B12.3.1	145	180	125	146	2.80E-06	2.80E-06	1.82E-06	2.25E-06
F23B12.3.2	134	165	113	140	2.80E-06	2.65E-06	1.82E-06	2.25E-06
F23B12.4	491	651	618	626	2.47E-05	2.89E-05	1.38E-05	2.00E-05
F23B12.5	2117	2063	2888	3532	2.29E-05	2.67E-05	1.26E-05	1.92E-05
F23B12.6.1	443	485	442	524	4.37E-05	5.48E-05	3.58E-05	4.48E-05
F23B12.6.2	459	487	455	522	1.32E-04	1.22E-04	1.17E-04	1.77E-04
F23B12.7.1	822	712	772	1257	3.61E-05	3.73E-05	2.34E-05	3.43E-05
F23B12.7.2	830	697	806	1283	4.26E-05	4.27E-05	2.75E-05	3.89E-05
F23B12.8.1	566	561	792	1008	3.12E-05	2.55E-05	1.90E-05	3.83E-05
F23B12.8.2	566	559	786	999	3.23E-05	2.56E-05	2.04E-05	4.01E-05
F23B12.9	6	9	11	7	2.12E-05	1.99E-05	1.93E-05	3.03E-05
F23B2.10	8	19	23	9	2.12E-05	1.98E-05	1.92E-05	3.01E-05
F23B2.11.1	1148	1031	1185	1224	2.80E-06	2.65E-06	1.82E-06	2.25E-06
F23B2.11.2	1148	1033	1185	1224	2.80E-06	2.65E-06	1.82E-06	2.25E-06
F23B2.13	210	146	552	132	3.70E-05	3.13E-05	2.48E-05	3.16E-05
F23B2.14	0	0	1	2	3.71E-05	3.15E-05	2.49E-05	3.18E-05
F23B2.15	0	1	2	2	1.24E-04	8.17E-05	2.13E-04	6.28E-05
F23B2.3	9	15	15	3	2.80E-06	2.65E-06	1.82E-06	2.25E-06
F23B2.4	19	35	29	19	2.80E-06	2.65E-06	1.82E-06	2.25E-06
F23B2.5a	79	82	89	40	2.80E-06	2.65E-06	1.82E-06	2.25E-06
F23B2.5b	77	74	77	42	2.80E-06	2.65E-06	1.82E-06	2.25E-06
F23B2.5c	65	72	80	35	1.31E-05	1.29E-05	9.62E-06	5.33E-06
F23B2.6	778	786	1191	1074	1.31E-05	1.19E-05	8.51E-06	5.74E-06
F23B2.7	8	28	15	6	1.70E-05	1.77E-05	1.36E-05	7.33E-06
F23B2.8	1	2	0	1	1.11E-04	1.06E-04	1.10E-04	1.23E-04
F23C8.1	12	10	4	4	2.80E-06	3.54E-06	1.82E-06	2.25E-06
F23C8.10	0	5	3	1	2.80E-06	2.65E-06	1.82E-06	2.25E-06

F23C8.11	5	10	10	6	2.80E-06	2.65E-06	1.82E-06	2.25E-06
F23C8.12	1	1	11	1	2.80E-06	2.65E-06	1.82E-06	2.25E-06
F23C8.13	9	20	14	25	2.80E-06	2.65E-06	1.82E-06	2.25E-06
F23C8.2	3	0	0	0	2.80E-06	2.65E-06	3.26E-06	2.25E-06
F23C8.3	7	7	6	3	2.80E-06	2.86E-06	1.82E-06	3.04E-06
F23C8.4	639	386	856	1004	2.80E-06	2.65E-06	1.82E-06	2.25E-06
F23C8.5.1	830	977	932	1093	2.80E-06	2.65E-06	1.82E-06	2.25E-06
F23C8.5.2	704	808	773	976	7.39E-05	4.22E-05	6.44E-05	9.33E-05
F23C8.6	250	384	312	318	1.02E-04	1.14E-04	7.48E-05	1.08E-04
F23C8.7	22	25	10	12	8.72E-05	9.46E-05	6.23E-05	9.71E-05
F23C8.8	15	27	18	5	3.56E-05	5.16E-05	2.89E-05	3.63E-05
F23C8.9	129	148	100	109	2.80E-06	2.65E-06	1.82E-06	2.25E-06
F23D12.1	229	211	863	132	2.80E-06	3.15E-06	1.82E-06	2.25E-06
F23D12.2	96	71	124	162	1.78E-05	1.93E-05	8.96E-06	1.21E-05
F23D12.3	8	22	8	2	2.83E-05	2.47E-05	6.94E-05	1.31E-05
F23D12.4	9	5	5	0	2.80E-06	2.65E-06	1.82E-06	2.25E-06
F23D12.5	22	32	27	21	2.80E-06	3.81E-06	1.82E-06	2.25E-06
F23D12.7	96	110	357	96	2.80E-06	2.65E-06	1.82E-06	2.25E-06
F23D12.8	9	10	6	9	2.80E-06	2.65E-06	1.82E-06	2.25E-06
F23D12.9	5	9	14	8	5.97E-05	6.46E-05	1.45E-04	4.80E-05
F23F1.1	298	223	463	410	2.80E-06	2.65E-06	1.82E-06	2.25E-06
F23F1.10	89	92	307	110	2.80E-06	2.65E-06	1.82E-06	2.25E-06
F23F1.2	27	37	5	7	4.70E-05	3.32E-05	4.75E-05	5.20E-05
F23F1.3	24	27	14	19	3.42E-05	3.35E-05	7.69E-05	3.40E-05
F23F1.4	75	113	49	69	5.26E-06	6.82E-06	1.82E-06	2.25E-06
F23F1.5	360	329	302	429	2.80E-06	2.83E-06	1.82E-06	2.25E-06
F23F1.6	47	62	42	55	1.72E-05	2.44E-05	7.31E-06	1.27E-05
F23F1.7	18	32	20	18	4.16E-05	3.59E-05	2.27E-05	3.98E-05
F23F1.8a	1017	1226	895	1202	2.88E-06	3.60E-06	1.82E-06	2.72E-06
F23F1.8b.1	1016	1225	940	1155	3.64E-06	6.11E-06	2.62E-06	2.92E-06
F23F1.8b.2	975	1181	841	1143	7.80E-05	8.88E-05	4.46E-05	7.40E-05
F23F1.8b.3	961	1167	823	1111	9.47E-05	1.08E-04	5.70E-05	8.65E-05
F23F1.9	318	309	421	572	8.37E-05	9.58E-05	4.70E-05	7.88E-05
F23F12.10	5	10	5	6	8.97E-05	1.03E-04	5.00E-05	8.33E-05
F23F12.11	9	15	36	10	2.55E-05	2.34E-05	2.19E-05	3.67E-05
F23F12.13	21	32	30	31	2.80E-06	2.65E-06	1.82E-06	2.25E-06
F23F12.3	35	50	35	48	2.80E-06	2.65E-06	2.51E-06	2.25E-06
F23F12.4	5	7	5	5	2.80E-06	2.65E-06	1.82E-06	2.25E-06
F23F12.6.1	1248	1320	1912	2185	2.80E-06	2.94E-06	1.82E-06	2.38E-06
F23F12.6.2	1117	1172	1712	2051	2.80E-06	2.65E-06	1.82E-06	2.25E-06
F23F12.8	35	33	44	22	1.03E-04	1.03E-04	1.03E-04	1.45E-04
F23F12.9a	17	24	28	16	1.00E-04	9.93E-05	9.99E-05	1.48E-04
F23F12.9b	3	7	2	8	2.80E-06	2.65E-06	1.82E-06	2.25E-06
F23G4.1	5	14	2	6	2.80E-06	3.07E-06	2.46E-06	2.25E-06
F23G4.t1	1	0	0	0	2.80E-06	2.65E-06	1.82E-06	2.25E-06
F23H11.1	386	346	380	543	2.80E-06	2.67E-06	1.82E-06	2.25E-06
F23H11.2	204	203	311	432	2.80E-06	2.65E-06	1.82E-06	2.25E-06
F23H11.3.1	673	726	824	973	5.01E-05	4.24E-05	3.21E-05	5.66E-05
F23H11.3.2	567	646	704	870	8.60E-06	8.07E-06	8.51E-06	1.46E-05
F23H11.4a	416	432	668	848	6.74E-05	6.87E-05	5.37E-05	7.83E-05
F23H11.4b	301	300	488	631	6.49E-05	6.98E-05	5.24E-05	8.00E-05
F23H11.5.1	1696	1471	4032	900	1.44E-05	1.41E-05	1.51E-05	2.36E-05
F23H11.5.2	1444	1256	3273	791	1.63E-05	1.54E-05	1.72E-05	2.75E-05
F23H11.5.3	1441	1267	3046	755	4.33E-04	3.55E-04	6.69E-04	1.84E-04
F23H11.6	5	7	13	6	4.03E-04	3.31E-04	5.95E-04	1.77E-04
F23H11.7	20	15	17	8	3.82E-04	3.17E-04	5.25E-04	1.61E-04
F23H11.8a	28	32	13	34	2.80E-06	2.65E-06	1.82E-06	2.25E-06
F23H11.8b.1	26	29	10	31	2.80E-06	2.65E-06	1.82E-06	2.25E-06
F23H11.8b.2	19	22	6	29	2.80E-06	2.65E-06	1.82E-06	2.25E-06

F23H11.9a	261	349	392	323	2.80E-06	2.65E-06	1.82E-06	2.25E-06
F23H11.9b.1	298	396	551	372	2.80E-06	2.65E-06	1.82E-06	2.25E-06
F23H11.9b.2	261	349	392	323	3.77E-05	4.76E-05	3.68E-05	3.74E-05
F23H12.1	100	136	49	65	3.97E-05	4.98E-05	4.78E-05	3.98E-05
F23H12.10	32	25	19	26	4.07E-05	5.14E-05	3.98E-05	4.05E-05
F23H12.2.1	436	510	489	319	1.89E-05	2.43E-05	6.03E-06	9.87E-06
F23H12.2.2	422	490	479	312	5.99E-06	4.42E-06	2.31E-06	3.91E-06
F23H12.3	23	20	20	18	6.74E-05	7.44E-05	4.92E-05	3.96E-05
F23H12.5	165	278	90	92	6.96E-05	7.64E-05	5.14E-05	4.13E-05
F23H12.6	11	7	17	10	4.28E-06	3.52E-06	2.42E-06	2.68E-06
F23H12.7	1	3	7	3	6.75E-06	1.08E-05	2.41E-06	3.04E-06
F23H12.8	163	125	348	71	2.80E-06	2.65E-06	1.82E-06	2.25E-06
F25A2.1	4	3	3	3	2.80E-06	2.65E-06	1.82E-06	2.25E-06
F25B3.1	721	767	1019	1546	4.74E-05	3.44E-05	6.59E-05	1.66E-05
F25B3.2	9	8	6	3	2.80E-06	2.65E-06	1.82E-06	2.25E-06
F25B3.3	21	23	18	21	2.70E-05	2.71E-05	2.48E-05	4.65E-05
F25B3.4	15	41	12	8	2.80E-06	2.65E-06	1.82E-06	2.25E-06
F25B3.5.1	32	99	34	36	2.80E-06	2.65E-06	1.82E-06	2.25E-06
F25B3.6	923	985	1168	1616	2.80E-06	4.95E-06	1.82E-06	2.25E-06
F25B4.1.1	461	699	591	521	2.80E-06	7.41E-06	1.82E-06	2.29E-06
F25B4.1.2	413	640	529	474	4.39E-05	4.42E-05	3.61E-05	6.17E-05
F25B4.2	152	161	148	186	3.72E-05	5.32E-05	3.10E-05	3.37E-05
F25B4.3	8	5	10	5	3.78E-05	5.53E-05	3.15E-05	3.48E-05
F25B4.4	117	147	121	172	9.63E-06	9.63E-06	6.09E-06	9.45E-06
F25B4.5.1	710	695	1344	1396	2.80E-06	2.65E-06	1.82E-06	2.25E-06
F25B4.6	153	238	183	212	1.99E-05	2.36E-05	1.34E-05	2.34E-05
F25B4.7	217	308	231	257	3.31E-05	3.06E-05	4.07E-05	5.22E-05
F25B4.8a	36	47	66	29	1.12E-05	1.64E-05	8.69E-06	1.24E-05
F25B4.8b	43	50	75	36	2.15E-05	2.88E-05	1.49E-05	2.04E-05
F25B4.9	776	1060	634	820	9.49E-06	1.17E-05	1.13E-05	6.14E-06
F25B5.1a	176	336	122	244	7.00E-06	7.70E-06	7.96E-06	4.72E-06
F25B5.1b	186	354	130	258	9.53E-05	1.23E-04	5.07E-05	8.09E-05
F25B5.2.1	578	613	955	1191	7.17E-06	1.29E-05	3.24E-06	7.98E-06
F25B5.2.2	555	585	910	1164	7.06E-06	1.27E-05	3.21E-06	7.87E-06
F25B5.3a	1100	1237	1021	1459	2.46E-05	2.47E-05	2.65E-05	4.07E-05
F25B5.3b	1495	1644	1440	1883	2.46E-05	2.44E-05	2.62E-05	4.14E-05
F25B5.3c.1	1110	1244	1036	1461	1.11E-04	1.18E-04	6.70E-05	1.18E-04
F25B5.3c.2	1101	1241	1028	1474	1.16E-04	1.20E-04	7.26E-05	1.17E-04
F25B5.3c.3	1235	1339	1144	1544	1.03E-04	1.10E-04	6.28E-05	1.09E-04
F25B5.3c.4	1198	1316	1121	1537	1.05E-04	1.12E-04	6.40E-05	1.13E-04
F25B5.3d	1098	1235	1023	1459	1.21E-04	1.24E-04	7.30E-05	1.22E-04
F25B5.3e	504	549	433	544	1.12E-04	1.16E-04	6.83E-05	1.16E-04
F25B5.4a.1	7895	10705	16553	18468	1.10E-04	1.17E-04	6.65E-05	1.17E-04
F25B5.4a.2	7719	10536	16240	18344	9.13E-05	9.40E-05	5.11E-05	7.92E-05
F25B5.4a.3	7709	10521	16200	18324	3.33E-04	4.26E-04	4.54E-04	6.25E-04
F25B5.4c.1	5125	6918	10989	12322	3.06E-04	3.94E-04	4.19E-04	5.84E-04
F25B5.4c.2	5127	6923	10990	12325	3.43E-04	4.42E-04	4.68E-04	6.54E-04
F25B5.5	1432	1830	2759	3013	3.54E-04	4.52E-04	4.94E-04	6.84E-04
F25B5.6a	433	617	572	778	3.54E-04	4.52E-04	4.94E-04	6.84E-04
F25B5.6b	343	545	465	665	9.22E-05	1.11E-04	1.16E-04	1.56E-04
F25B5.6c.1	343	547	466	666	2.25E-05	3.02E-05	1.93E-05	3.24E-05
F25B5.6c.2	342	525	461	658	2.45E-05	3.68E-05	2.16E-05	3.82E-05
F25B5.7a	279	313	290	408	2.27E-05	3.42E-05	2.01E-05	3.54E-05
F25B5.7b	137	114	98	134	2.54E-05	3.68E-05	2.23E-05	3.93E-05
F25B5.7c	293	299	282	394	1.85E-05	1.97E-05	1.25E-05	2.18E-05
F25B5.7d	256	246	227	320	2.66E-05	2.09E-05	1.24E-05	2.09E-05
F25C8.1	10	11	8	19	1.85E-05	1.78E-05	1.16E-05	2.00E-05
F25C8.2	33	47	31	54	2.48E-05	2.25E-05	1.43E-05	2.49E-05
F25C8.3a	177	179	173	183	2.80E-06	2.65E-06	1.82E-06	2.25E-06

F25C8.3b	178	184	181	185	2.80E-06	3.65E-06	1.82E-06	3.58E-06
F25C8.4	61	100	30	66	2.80E-06	2.65E-06	1.82E-06	2.25E-06
F25C8.5	8	11	9	3	2.80E-06	2.65E-06	1.82E-06	2.25E-06
F25D1.1a	1375	1675	1570	2404	3.61E-06	5.61E-06	1.82E-06	3.15E-06
F25D1.1b	1296	1559	1490	2314	2.80E-06	2.65E-06	1.82E-06	2.25E-06
F25D1.2	7	6	5	4	7.19E-05	8.27E-05	5.34E-05	1.01E-04
F25D1.3	18	13	11	5	1.16E-04	1.32E-04	8.68E-05	1.66E-04
F25D1.4	23	26	37	21	2.80E-06	2.65E-06	1.82E-06	2.25E-06
F25D1.5	14	40	21	31	2.91E-06	2.65E-06	1.82E-06	2.25E-06
F25D7.2	731	759	1032	1165	2.80E-06	2.65E-06	1.82E-06	2.25E-06
F25D7.3	144	159	129	142	2.80E-06	5.08E-06	1.84E-06	3.35E-06
F25D7.4	1933	1576	3255	3882	5.22E-05	5.12E-05	4.80E-05	6.69E-05
F25D7.5	18	27	17	9	6.58E-06	6.85E-06	3.83E-06	5.20E-06
F25E2.1	5	7	14	2	7.09E-05	5.46E-05	7.77E-05	1.14E-04
F25E2.2	312	422	702	559	2.80E-06	2.65E-06	1.82E-06	2.25E-06
F25E2.3	16	38	15	20	2.80E-06	2.65E-06	1.82E-06	2.25E-06
F25E2.4	123	158	77	174	1.76E-05	2.25E-05	2.58E-05	2.54E-05
F25E2.5a	115	136	101	213	2.80E-06	3.81E-06	1.82E-06	2.25E-06
F25E2.5b	134	160	126	247	9.10E-06	1.10E-05	3.70E-06	1.03E-05
F25E2.5c	129	151	121	235	4.73E-06	5.26E-06	2.70E-06	7.02E-06
F25E5.1	221	223	277	362	4.98E-06	5.63E-06	3.04E-06	7.38E-06
F25E5.10	4	5	10	3	5.18E-06	5.74E-06	3.17E-06	7.60E-06
F25E5.11	6	7	5	6	7.03E-06	6.69E-06	5.72E-06	9.24E-06
F25E5.12	4	2	5	6	2.80E-06	2.65E-06	1.82E-06	2.25E-06
F25E5.13	2	3	8	4	2.80E-06	2.65E-06	1.82E-06	2.25E-06
F25E5.14	6	4	5	3	2.80E-06	2.65E-06	1.82E-06	2.25E-06
F25E5.15	3	0	3	3	2.80E-06	2.65E-06	1.82E-06	2.25E-06
F25E5.16	22	11	12	9	2.80E-06	2.65E-06	1.82E-06	2.25E-06
F25E5.2	12	22	14	6	2.80E-06	2.65E-06	1.82E-06	2.25E-06
F25E5.3	5	6	5	10	2.80E-06	2.65E-06	1.82E-06	2.25E-06
F25E5.4	8	16	9	16	2.80E-06	2.65E-06	1.82E-06	2.25E-06
F25E5.5	23	51	13	47	2.80E-06	2.65E-06	1.82E-06	2.25E-06
F25E5.6a	14	16	16	11	2.80E-06	2.65E-06	1.82E-06	2.25E-06
F25E5.6b	9	7	8	5	2.80E-06	4.36E-06	1.82E-06	3.42E-06
F25E5.7	4	4	3	4	2.80E-06	2.65E-06	1.82E-06	2.25E-06
F25E5.8b.1	151	393	228	272	2.80E-06	2.65E-06	1.82E-06	2.25E-06
F25E5.8b.2	48	154	54	63	2.80E-06	2.65E-06	1.82E-06	2.25E-06
F25E5.9	9	7	1	2	1.67E-05	4.11E-05	1.64E-05	2.42E-05
F25F2.1a	50	51	38	23	9.27E-06	2.81E-05	6.78E-06	9.76E-06
F25F2.1b	53	57	41	25	2.80E-06	2.65E-06	1.82E-06	2.25E-06
F25F2.2	575	525	797	1262	3.98E-06	3.84E-06	1.97E-06	2.25E-06
F25F6.1	6	7	1	4	3.25E-06	3.31E-06	1.82E-06	2.25E-06
F25F6.t2	0	1	1	1	4.87E-06	4.18E-06	4.39E-06	8.57E-06
F25F8.1	39	71	29	55	2.80E-06	2.65E-06	1.82E-06	2.25E-06
F25F8.2	40	80	57	84	2.80E-06	2.65E-06	1.82E-06	2.25E-06
F25G6.1	18	39	25	38	2.80E-06	3.62E-06	1.82E-06	2.38E-06
F25G6.2	504	541	625	982	3.08E-06	5.79E-06	2.84E-06	5.17E-06
F25G6.3b	26	38	27	26	2.80E-06	2.65E-06	1.82E-06	2.25E-06
F25G6.4	20	24	18	16	1.56E-05	1.58E-05	1.26E-05	2.44E-05
F25G6.5	5	5	0	2	2.80E-06	2.70E-06	1.82E-06	2.25E-06
F25G6.6	85	117	47	52	2.80E-06	2.65E-06	1.82E-06	2.25E-06
F25G6.7a	23	22	17	14	2.80E-06	2.65E-06	1.82E-06	2.25E-06
F25G6.7b	31	31	21	20	4.73E-06	6.16E-06	1.82E-06	2.32E-06
F25G6.8.1	381	374	471	355	2.80E-06	2.65E-06	1.82E-06	2.25E-06
F25G6.8.2	357	353	346	311	2.80E-06	2.65E-06	1.82E-06	2.25E-06
F25G6.9	848	891	955	1671	7.08E-05	6.56E-05	5.69E-05	5.30E-05
F25H2.1	45	91	38	65	6.45E-05	6.02E-05	4.07E-05	4.51E-05
F25H2.10.1	14505	16463	23788	21329	2.23E-05	2.22E-05	1.64E-05	3.53E-05
F25H2.10.2	12994	14760	20257	19915	3.67E-06	7.01E-06	2.02E-06	4.25E-06

F25H2.11.1	6330	7677	6314	6954	1.58E-03	1.69E-03	1.68E-03	1.86E-03
F25H2.11.2	5649	6914	5163	6249	1.49E-03	1.60E-03	1.51E-03	1.83E-03
F25H2.12a	116	134	104	172	1.07E-03	1.22E-03	6.92E-04	9.41E-04
F25H2.12b	115	128	100	167	9.53E-04	1.10E-03	5.67E-04	8.47E-04
F25H2.13	371	374	489	806	9.02E-06	9.84E-06	5.27E-06	1.07E-05
F25H2.2	83	151	81	124	8.99E-06	9.44E-06	5.08E-06	1.05E-05
F25H2.3	1	2	5	2	1.34E-05	1.28E-05	1.15E-05	2.34E-05
F25H2.4	257	328	679	307	4.70E-06	8.07E-06	2.99E-06	5.65E-06
F25H2.5.1	11191	10063	9654	8764	2.80E-06	2.65E-06	1.82E-06	2.25E-06
F25H2.5.2	11155	10017	9624	8749	4.52E-05	5.46E-05	7.78E-05	4.34E-05
F25H2.5.3	11139	9989	9597	8719	2.02E-03	1.72E-03	1.14E-03	1.27E-03
F25H2.6.1	345	573	471	655	2.06E-03	1.75E-03	1.16E-03	1.30E-03
F25H2.6.2	377	596	524	685	2.03E-03	1.72E-03	1.14E-03	1.27E-03
F25H2.7	74	102	57	106	1.85E-05	2.89E-05	1.64E-05	2.81E-05
F25H2.8.1	1143	1394	1557	2170	1.99E-05	2.98E-05	1.80E-05	2.91E-05
F25H2.8.2	794	977	1025	1484	7.48E-06	9.76E-06	3.75E-06	8.61E-06
F25H2.9	835	1080	726	636	7.33E-05	8.44E-05	6.50E-05	1.12E-04
F25H5.1a	286	395	212	417	7.51E-05	8.73E-05	6.31E-05	1.13E-04
F25H5.1b	269	354	199	390	1.10E-04	1.34E-04	6.21E-05	6.72E-05
F25H5.1c.1	289	398	212	421	1.71E-05	2.23E-05	8.24E-06	2.00E-05
F25H5.1e	251	326	177	354	1.55E-05	1.92E-05	7.45E-06	1.80E-05
F25H5.1f	552	754	393	755	1.72E-05	2.24E-05	8.24E-06	2.02E-05
F25H5.2	13	30	11	16	1.95E-05	2.40E-05	8.96E-06	2.21E-05
F25H5.3a	3749	3625	4673	5748	2.18E-05	2.82E-05	1.01E-05	2.40E-05
F25H5.3b	2950	2843	3674	4685	2.80E-06	4.55E-06	1.82E-06	2.25E-06
F25H5.3c.1	2994	2883	3760	4730	2.01E-04	1.84E-04	1.63E-04	2.48E-04
F25H5.3c.2	2960	2846	3695	4694	1.80E-04	1.64E-04	1.46E-04	2.30E-04
F25H5.3d	3275	3132	3996	4941	1.65E-04	1.50E-04	1.35E-04	2.10E-04
F25H5.4.1	27230	30058	43769	52104	1.99E-04	1.81E-04	1.62E-04	2.53E-04
F25H5.4.2	27235	30064	43773	52107	2.15E-04	1.94E-04	1.71E-04	2.60E-04
F25H5.4.3	25825	28600	41517	50480	1.08E-03	1.13E-03	1.13E-03	1.67E-03
F25H5.4.4	25811	28582	41509	50461	1.10E-03	1.15E-03	1.15E-03	1.69E-03
F25H5.5	347	414	423	673	1.05E-03	1.10E-03	1.10E-03	1.65E-03
F25H5.6.1	139	154	302	95	1.07E-03	1.12E-03	1.12E-03	1.68E-03
F25H5.6.2	116	135	199	72	1.67E-05	1.88E-05	1.32E-05	2.60E-05
F25H5.7	38	71	20	25	2.18E-05	2.28E-05	3.08E-05	1.20E-05
F25H5.8	183	191	260	138	2.56E-05	2.81E-05	2.86E-05	1.28E-05
F25H8.3	178	244	160	268	2.86E-06	5.03E-06	1.82E-06	2.25E-06
F25H8.5a	263	401	170	140	1.08E-04	1.07E-04	1.00E-04	6.57E-05
F25H8.5b	321	456	231	148	3.08E-06	3.97E-06	1.82E-06	3.71E-06
F25H8.5c	263	401	170	141	1.23E-05	1.77E-05	5.19E-06	5.26E-06
F25H8.5d	40	74	22	28	2.70E-05	3.62E-05	1.26E-05	9.99E-06
F25H8.6	119	162	92	109	1.34E-05	1.93E-05	5.63E-06	5.76E-06
F25H8.7	3	1	2	2	3.28E-06	5.74E-06	1.82E-06	2.25E-06
F25H9.1	5	4	7	6	6.05E-06	7.78E-06	3.04E-06	4.45E-06
F25H9.2	22	22	18	30	2.80E-06	2.65E-06	1.82E-06	2.25E-06
F25H9.3	1	2	1	3	2.80E-06	2.65E-06	1.82E-06	2.25E-06
F25H9.4	26	39	19	29	2.80E-06	2.65E-06	1.82E-06	2.45E-06
F25H9.6	131	150	273	150	2.80E-06	2.65E-06	1.82E-06	2.25E-06
F25H9.7	95	93	184	78	2.80E-06	3.28E-06	1.82E-06	2.25E-06
F25H9.9	13	22	20	9	1.62E-05	1.75E-05	2.20E-05	1.49E-05
F26A1.1	282	320	350	315	2.06E-05	1.90E-05	2.59E-05	1.35E-05
F26A1.10	11	19	12	11	2.80E-06	2.65E-06	1.82E-06	2.25E-06
F26A1.11	5	5	5	5	3.24E-05	3.47E-05	2.61E-05	2.90E-05
F26A1.12	6	5	6	1	4.48E-06	7.30E-06	3.19E-06	3.60E-06
F26A1.13	57	80	171	208	2.80E-06	2.65E-06	1.82E-06	2.25E-06
F26A1.14	36	52	54	68	2.80E-06	2.65E-06	1.82E-06	2.25E-06
F26A1.15	4	16	10	8	4.70E-06	6.24E-06	9.18E-06	1.38E-05
F26A1.2	2	11	5	1	8.23E-06	1.12E-05	8.05E-06	1.25E-05

F26A1.3	30	56	14	18	2.80E-06	2.65E-06	1.82E-06	2.25E-06
F26A1.4	3	8	3	3	2.80E-06	2.65E-06	1.82E-06	2.25E-06
F26A1.6	14	129	10	57	2.80E-06	3.86E-06	1.82E-06	2.25E-06
F26A1.7	0	5	7	4	2.80E-06	2.65E-06	1.82E-06	2.25E-06
F26A1.8	16	348	16	163	2.80E-06	1.80E-05	1.82E-06	6.75E-06
F26A1.9	10	28	8	11	2.80E-06	2.65E-06	1.82E-06	2.25E-06
F26A10.1	2	8	5	7	2.80E-06	3.02E-05	1.82E-06	1.20E-05
F26A10.2	77	73	42	60	2.80E-06	6.77E-06	1.82E-06	2.27E-06
F26A3.1	128	195	81	193	2.80E-06	5.03E-06	2.17E-06	3.76E-06
F26A3.2	56	109	263	200	4.62E-06	4.15E-06	1.82E-06	2.90E-06
F26A3.3	772	881	1091	1749	1.34E-05	1.93E-05	5.52E-06	1.62E-05
F26A3.4.1	200	645	127	260	1.35E-05	2.48E-05	4.12E-05	3.87E-05
F26A3.4.2	145	458	85	187	1.66E-05	1.79E-05	1.53E-05	3.02E-05
F26A3.5	19	83	17	20	2.41E-05	7.35E-05	9.97E-06	2.52E-05
F26A3.6	11	11	8	12	2.34E-05	6.99E-05	8.95E-06	2.43E-05
F26A3.7	122	153	146	162	2.80E-06	9.31E-06	1.82E-06	2.25E-06
F26A3.8	479	637	667	972	2.80E-06	2.65E-06	1.82E-06	2.25E-06
F26B1.1	232	372	148	75	1.48E-05	1.75E-05	1.15E-05	1.58E-05
F26B1.2a	1744	1591	1793	2957	1.07E-05	1.34E-05	9.68E-06	1.74E-05
F26B1.2b	340	328	258	459	2.84E-05	4.30E-05	1.18E-05	7.38E-06
F26B1.2c.1	971	883	849	1438	1.02E-04	8.82E-05	6.85E-05	1.39E-04
F26B1.2c.2	954	857	810	1424	7.76E-05	7.07E-05	3.83E-05	8.41E-05
F26B1.2c.3	1025	931	917	1482	9.30E-05	7.99E-05	5.29E-05	1.11E-04
F26B1.2d	227	204	182	279	9.01E-05	7.64E-05	4.98E-05	1.08E-04
F26B1.2e	207	206	163	198	1.00E-04	8.60E-05	5.83E-05	1.16E-04
F26B1.3.1	2605	2509	4237	4395	3.98E-05	3.38E-05	2.08E-05	3.93E-05
F26B1.3.2	2238	2164	3537	3782	5.34E-05	5.02E-05	2.74E-05	4.10E-05
F26B1.4	55	59	35	23	1.61E-04	1.46E-04	1.70E-04	2.18E-04
F26B1.5	9	15	3	10	1.56E-04	1.42E-04	1.60E-04	2.11E-04
F26B1.6	4	8	5	3	6.72E-06	6.80E-06	2.79E-06	2.25E-06
F26B1.7	6	14	10	14	2.80E-06	2.65E-06	1.82E-06	2.25E-06
F26C11.1	152	206	212	199	2.80E-06	2.65E-06	1.82E-06	2.25E-06
F26C11.2	2	6	2	2	2.80E-06	2.65E-06	1.82E-06	2.25E-06
F26C11.3	21	16	14	22	7.50E-06	9.60E-06	6.81E-06	7.89E-06
F26C11.4	2	1	3	0	2.80E-06	2.65E-06	1.82E-06	2.25E-06
F26D10.10	1226	921	1712	1921	2.80E-06	2.65E-06	1.82E-06	2.25E-06
F26D10.11	11	12	6	6	2.80E-06	2.65E-06	1.82E-06	2.25E-06
F26D10.12	9	10	10	16	1.10E-04	7.77E-05	9.95E-05	1.38E-04
F26D10.13	3	3	0	3	2.80E-06	2.65E-06	1.82E-06	2.25E-06
F26D10.3.1	28251	19813	35826	40307	2.80E-06	2.65E-06	1.82E-06	3.08E-06
F26D10.3.2	26260	18549	34049	39390	2.80E-06	2.65E-06	1.82E-06	2.25E-06
F26D10.8	2	5	1	3	1.52E-03	1.01E-03	1.26E-03	1.74E-03
F26D11.1	13	26	34	31	1.46E-03	9.77E-04	1.24E-03	1.76E-03
F26D11.10	6	12	6	4	2.80E-06	2.65E-06	1.82E-06	2.25E-06
F26D11.11a	264	314	242	406	2.80E-06	5.24E-06	4.72E-06	5.31E-06
F26D11.11b	193	243	181	316	2.80E-06	2.65E-06	1.82E-06	2.25E-06
F26D11.2	13	13	14	14	9.13E-06	1.03E-05	5.45E-06	1.13E-05
F26D11.4	4	7	7	2	9.32E-06	1.11E-05	5.69E-06	1.23E-05
F26D11.5	2	0	4	1	2.80E-06	2.65E-06	1.82E-06	2.25E-06
F26D11.6	12	8	6	5	2.80E-06	2.65E-06	1.82E-06	2.25E-06
F26D11.9	4	5	5	2	2.80E-06	2.65E-06	1.82E-06	2.25E-06
F26D12.1a	125	238	130	226	2.80E-06	2.65E-06	1.82E-06	2.25E-06
F26D12.1b	61	130	70	126	2.80E-06	2.65E-06	1.82E-06	2.25E-06
F26D12.1c	15	33	21	46	4.56E-06	8.20E-06	3.08E-06	6.61E-06
F26D12.1d	60	105	50	102	3.72E-06	7.49E-06	2.79E-06	6.18E-06
F26D2.1	5	4	7	4	2.97E-06	6.16E-06	2.70E-06	7.31E-06
F26D2.10	44	90	45	28	7.03E-06	1.16E-05	3.81E-06	9.60E-06
F26D2.11	3	4	3	2	2.80E-06	2.65E-06	1.82E-06	2.25E-06
F26D2.12	0	4	1	1	2.80E-06	2.65E-06	1.82E-06	2.25E-06

F26D2.13	4	4	2	3	2.80E-06	2.65E-06	1.82E-06	2.25E-06
F26D2.14	1	1	0	2	2.80E-06	2.65E-06	1.82E-06	2.25E-06
F26D2.15	1	2	10	5	2.80E-06	2.65E-06	1.82E-06	2.25E-06
F26D2.16	3	4	5	1	2.80E-06	2.65E-06	1.82E-06	2.25E-06
F26D2.2	245	296	183	355	2.80E-06	2.65E-06	1.82E-06	2.25E-06
F26D2.3a	4	8	2	2	2.80E-06	2.65E-06	1.82E-06	2.25E-06
F26D2.3b	4	3	5	0	1.77E-05	2.02E-05	8.60E-06	2.06E-05
F26D2.4	4	2	4	1	2.80E-06	2.65E-06	1.82E-06	2.25E-06
F26D2.5	7	1	5	3	2.80E-06	2.65E-06	1.82E-06	2.25E-06
F26D2.7	2	3	3	1	2.80E-06	2.65E-06	1.82E-06	2.25E-06
F26D2.8	9	2	10	5	2.80E-06	2.65E-06	1.82E-06	2.25E-06
F26D2.9	5	6	2	6	2.80E-06	2.65E-06	1.82E-06	2.25E-06
F26E4.1	1504	1679	2131	3101	2.80E-06	2.65E-06	1.82E-06	2.25E-06
F26E4.10	456	475	648	978	2.80E-06	2.65E-06	1.82E-06	2.25E-06
F26E4.11.1	897	1063	1712	1951	6.67E-05	7.03E-05	6.15E-05	1.10E-04
F26E4.11.2	736	807	1409	1583	1.50E-05	1.48E-05	1.39E-05	2.58E-05
F26E4.12	83	138	66	57	4.59E-05	5.14E-05	5.71E-05	8.02E-05
F26E4.2	46	70	29	34	4.74E-05	4.91E-05	5.90E-05	8.18E-05
F26E4.3	126	195	102	185	1.89E-05	2.97E-05	9.78E-06	1.04E-05
F26E4.5	17	44	13	12	1.24E-05	1.79E-05	5.10E-06	7.40E-06
F26E4.6.1	968	845	5830	1229	8.57E-06	1.25E-05	4.52E-06	1.01E-05
F26E4.6.2	784	676	3934	981	2.80E-06	3.23E-06	1.82E-06	2.25E-06
F26E4.7a	35	79	39	60	2.74E-04	2.26E-04	1.08E-03	2.80E-04
F26E4.7b	36	83	52	63	2.25E-04	1.83E-04	7.35E-04	2.26E-04
F26E4.8.1	3818	3989	4573	5765	2.80E-06	4.10E-06	1.82E-06	2.65E-06
F26E4.8.2	3488	3560	4229	5504	2.80E-06	4.18E-06	1.82E-06	2.70E-06
F26E4.9.1	1201	1324	1708	1137	2.81E-04	2.77E-04	2.19E-04	3.41E-04
F26E4.9.2	1345	1496	2040	1205	2.87E-04	2.76E-04	2.26E-04	3.63E-04
F26E4.9.3	1130	1228	1619	1100	2.09E-04	2.18E-04	1.93E-04	1.59E-04
F26F12.2	7	13	4	2	2.12E-04	2.23E-04	2.09E-04	1.53E-04
F26F12.3a.1	349	691	459	750	2.54E-04	2.61E-04	2.37E-04	1.99E-04
F26F12.3a.2	345	688	454	749	2.80E-06	2.65E-06	1.82E-06	2.25E-06
F26F12.3b	345	688	454	749	1.44E-05	2.69E-05	1.23E-05	2.48E-05
F26F12.3c.1	488	927	645	1057	1.42E-05	2.68E-05	1.22E-05	2.48E-05
F26F12.3c.2	302	644	423	689	1.42E-05	2.68E-05	1.22E-05	2.48E-05
F26F12.4	17	19	9	8	1.50E-05	2.68E-05	1.29E-05	2.60E-05
F26F12.5a	50	40	66	56	1.47E-05	2.96E-05	1.34E-05	2.69E-05
F26F12.5b	6	14	5	8	2.80E-06	2.75E-06	1.82E-06	2.25E-06
F26F12.6a	7	4	4	3	5.91E-06	4.47E-06	5.08E-06	5.33E-06
F26F12.6b	5	4	6	4	2.80E-06	3.52E-06	1.82E-06	2.25E-06
F26F12.8	7	15	7	4	2.80E-06	2.65E-06	1.82E-06	2.25E-06
F26F2.1	12	4	9	3	2.80E-06	2.65E-06	1.82E-06	2.25E-06
F26F2.2	2	0	1	2	3.00E-06	6.08E-06	1.95E-06	2.25E-06
F26F2.3	7	5	11	12	2.80E-06	2.65E-06	1.82E-06	2.25E-06
F26F2.4	4	2	1	1	2.80E-06	2.65E-06	1.82E-06	2.25E-06
F26F2.5	0	1	0	2	2.80E-06	2.65E-06	1.82E-06	2.25E-06
F26F2.6	4	6	4	0	2.80E-06	2.65E-06	1.82E-06	2.25E-06
F26F2.7	182	212	517	584	2.80E-06	2.65E-06	1.82E-06	2.25E-06
F26F2.8	17	16	24	9	2.80E-06	2.65E-06	1.82E-06	2.25E-06
F26F4.1	546	504	744	805	8.37E-06	9.23E-06	1.55E-05	2.16E-05
F26F4.10a.1	1226	1363	1261	1656	3.78E-06	3.36E-06	3.46E-06	2.25E-06
F26F4.10a.2	1211	1340	1331	1668	5.16E-05	4.50E-05	4.58E-05	6.11E-05
F26F4.10b.1	1232	1361	1375	1701	5.95E-05	6.25E-05	3.98E-05	6.46E-05
F26F4.10b.2	1126	1285	1164	1553	6.27E-05	6.55E-05	4.48E-05	6.93E-05
F26F4.11	354	388	613	316	5.92E-05	6.18E-05	4.30E-05	6.57E-05
F26F4.12	117	154	152	163	6.04E-05	6.51E-05	4.06E-05	6.69E-05
F26F4.13	92	98	127	77	6.75E-05	6.99E-05	7.61E-05	4.84E-05
F26F4.2	28	64	24	10	1.71E-05	2.13E-05	1.45E-05	1.91E-05
F26F4.3	45	42	58	38	2.75E-05	2.76E-05	2.47E-05	1.85E-05

F26F4.4.1	445	506	594	735	5.32E-06	1.15E-05	2.97E-06	2.25E-06
F26F4.4.2	434	504	582	727	4.70E-06	4.15E-06	3.95E-06	3.19E-06
F26F4.4.3	382	435	469	632	1.98E-05	2.13E-05	1.72E-05	2.63E-05
F26F4.5.1	217	298	393	437	3.11E-05	3.41E-05	2.71E-05	4.19E-05
F26F4.5.2	168	245	319	388	3.11E-05	3.35E-05	2.49E-05	4.14E-05
F26F4.6	196	198	207	283	1.41E-05	1.83E-05	1.66E-05	2.28E-05
F26F4.7	1049	1005	1284	1977	1.34E-05	1.85E-05	1.66E-05	2.49E-05
F26F4.8	59	109	34	63	1.86E-05	1.77E-05	1.28E-05	2.15E-05
F26F4.9a	81	103	107	82	3.14E-05	2.85E-05	2.51E-05	4.76E-05
F26F4.9b	173	259	200	158	6.44E-06	1.12E-05	2.41E-06	5.51E-06
F26G1.1	484	500	934	1150	1.93E-05	2.32E-05	1.66E-05	1.57E-05
F26G1.10	3	9	2	0	1.10E-05	1.56E-05	8.29E-06	8.10E-06
F26G1.2a	9	23	23	26	1.70E-05	1.66E-05	2.14E-05	3.25E-05
F26G1.2b	2	13	5	15	2.80E-06	2.65E-06	1.82E-06	2.25E-06
F26G1.3	3	4	3	2	3.47E-06	8.36E-06	5.76E-06	8.03E-06
F26G1.4	22	58	23	26	2.80E-06	3.31E-06	1.82E-06	3.24E-06
F26G1.5	81	175	38	37	2.80E-06	2.65E-06	1.82E-06	2.25E-06
F26G1.6	33	50	20	23	2.80E-06	5.69E-06	1.82E-06	2.25E-06
F26G1.7	778	1210	1195	289	1.34E-05	2.73E-05	4.08E-06	4.90E-06
F26G1.8	238	321	434	84	2.80E-06	2.65E-06	1.82E-06	2.25E-06
F26G1.9	4	20	3	2	2.03E-04	2.98E-04	2.03E-04	6.06E-05
F26G5.1	124	158	269	388	7.36E-05	9.38E-05	8.74E-05	2.09E-05
F26G5.10	5	7	12	4	2.80E-06	4.97E-06	1.82E-06	2.25E-06
F26G5.11	1	3	11	2	7.98E-06	9.60E-06	1.13E-05	2.01E-05
F26G5.12	5	6	4	4	2.80E-06	2.65E-06	1.82E-06	2.25E-06
F26G5.2	4	4	33	7	2.80E-06	2.65E-06	1.82E-06	2.25E-06
F26G5.3	3	0	6	4	2.80E-06	2.65E-06	1.82E-06	2.25E-06
F26G5.4	7	5	10	5	2.80E-06	2.65E-06	2.30E-06	2.25E-06
F26G5.5	3	6	9	5	2.80E-06	2.65E-06	1.82E-06	2.25E-06
F26G5.9	765	818	1058	1631	2.80E-06	2.65E-06	1.82E-06	2.25E-06
F26H11.1	281	278	372	186	2.80E-06	2.65E-06	1.82E-06	2.25E-06
F26H11.2b	1020	988	970	1584	2.59E-05	2.62E-05	2.33E-05	4.44E-05
F26H11.2c	1283	1550	1209	1912	6.54E-05	6.12E-05	5.64E-05	3.48E-05
F26H11.2d	569	828	505	746	2.17E-05	1.99E-05	1.34E-05	2.71E-05
F26H11.2e	234	530	202	272	2.07E-05	2.36E-05	1.27E-05	2.48E-05
F26H11.2f	300	669	224	308	3.03E-05	4.17E-05	1.75E-05	3.19E-05
F26H11.2g	248	547	169	251	1.97E-05	4.21E-05	1.11E-05	1.84E-05
F26H11.4	174	206	238	285	1.92E-05	4.05E-05	9.35E-06	1.59E-05
F26H11.5	133	157	139	164	2.17E-05	4.53E-05	9.64E-06	1.77E-05
F26H9.1	454	442	606	925	8.68E-06	9.71E-06	7.73E-06	1.14E-05
F26H9.2	460	414	485	800	2.08E-05	2.32E-05	1.41E-05	2.06E-05
F26H9.4	89	79	116	119	3.23E-05	2.97E-05	2.80E-05	5.28E-05
F26H9.5	478	470	541	566	3.19E-05	2.71E-05	2.19E-05	4.46E-05
F26H9.6.1	1007	1123	1100	1460	1.48E-05	1.24E-05	1.26E-05	1.59E-05
F26H9.6.2	578	645	524	782	4.20E-05	3.90E-05	3.10E-05	4.00E-05
F26H9.7	65	83	67	112	1.04E-04	1.09E-04	7.38E-05	1.21E-04
F26H9.8	43	55	38	24	1.01E-04	1.07E-04	5.97E-05	1.10E-04
F27B3.2	8	9	12	12	1.10E-05	1.33E-05	7.40E-06	1.53E-05
F27B3.5	370	484	434	571	2.80E-06	2.65E-06	1.82E-06	2.25E-06
F27B3.6	4	8	13	11	2.80E-06	2.65E-06	1.82E-06	2.25E-06
F27B3.7	16	15	5	23	1.45E-05	1.79E-05	1.11E-05	1.80E-05
F27B3.8	3	4	1	2	2.80E-06	2.65E-06	1.82E-06	2.25E-06
F27C1.1	197	433	135	115	4.17E-06	3.70E-06	1.82E-06	4.81E-06
F27C1.10	12	8	13	7	2.80E-06	2.65E-06	1.82E-06	2.25E-06
F27C1.11	31	35	37	29	2.72E-05	5.66E-05	1.22E-05	1.28E-05
F27C1.13	1	3	2	1	2.80E-06	2.65E-06	1.82E-06	2.25E-06
F27C1.2b	351	548	506	599	2.80E-06	2.65E-06	1.82E-06	2.25E-06
F27C1.3	26	32	21	22	2.80E-06	2.65E-06	1.82E-06	2.25E-06
F27C1.4	10	3	13	10	3.68E-05	5.43E-05	3.45E-05	5.04E-05

F27C1.6.1	397	413	300	618	3.19E-06	3.70E-06	1.82E-06	2.25E-06
F27C1.6.2	393	393	299	625	2.80E-06	2.65E-06	1.97E-06	2.25E-06
F27C1.7a.1	1900	2113	2141	1921	2.07E-05	2.04E-05	1.02E-05	2.59E-05
F27C1.7a.2	1744	1970	1838	1840	2.25E-05	2.12E-05	1.11E-05	2.87E-05
F27C1.7a.3	1728	1948	1801	1821	3.16E-04	3.32E-04	2.32E-04	2.57E-04
F27C1.7a.4	1710	1932	1737	1798	3.08E-04	3.29E-04	2.11E-04	2.61E-04
F27C1.7b	2128	2506	2167	2301	2.85E-04	3.03E-04	1.93E-04	2.41E-04
F27C1.8.1	1425	1759	707	418	3.05E-04	3.26E-04	2.02E-04	2.58E-04
F27C1.8.2	1263	1544	582	394	2.51E-04	2.80E-04	1.67E-04	2.18E-04
F27C8.1	86	133	78	123	1.81E-04	2.11E-04	5.83E-05	4.25E-05
F27C8.2	10	13	5	16	1.65E-04	1.90E-04	4.94E-05	4.13E-05
F27C8.3	3	7	14	5	4.98E-06	7.30E-06	2.95E-06	5.74E-06
F27C8.4	228	283	405	228	2.80E-06	2.65E-06	1.82E-06	2.25E-06
F27C8.5	37	95	43	22	2.80E-06	2.65E-06	3.12E-06	2.25E-06
F27C8.6.1	1379	1859	2520	2836	6.70E-05	7.86E-05	7.75E-05	5.38E-05
F27C8.6.2	1339	1802	2503	2797	2.80E-06	5.92E-06	1.84E-06	2.25E-06
F27D4.3	63	98	45	81	1.02E-04	1.30E-04	1.22E-04	1.69E-04
F27D4.4b	27	15	12	16	1.01E-04	1.28E-04	1.23E-04	1.69E-04
F27D4.6.1	74	111	46	120	8.29E-06	1.22E-05	3.86E-06	8.57E-06
F27D4.6.2	73	103	42	116	3.88E-05	2.03E-05	1.12E-05	1.85E-05
F27D4.6.3	73	105	42	118	2.80E-06	3.15E-06	1.82E-06	2.90E-06
F27D4.7	128	152	184	101	2.80E-06	2.99E-06	1.82E-06	2.86E-06
F27D9.1b	34	28	11	34	2.80E-06	3.07E-06	1.82E-06	2.95E-06
F27D9.1c	370	428	301	480	2.93E-05	3.29E-05	2.74E-05	1.86E-05
F27D9.2	37	53	32	38	1.06E-05	8.23E-06	2.22E-06	8.50E-06
F27D9.3	2	5	2	1	1.47E-05	1.61E-05	7.78E-06	1.53E-05
F27D9.4	2	9	1	1	2.80E-06	3.39E-06	1.82E-06	2.25E-06
F27D9.5	1177	1400	1157	1446	2.80E-06	2.65E-06	1.82E-06	2.25E-06
F27D9.6	72	122	91	41	2.80E-06	2.65E-06	1.82E-06	2.25E-06
F27D9.7	19	32	23	16	5.38E-05	6.05E-05	3.44E-05	5.31E-05
F27D9.8a	24	39	24	31	5.85E-06	9.39E-06	4.83E-06	2.68E-06
F27D9.8b	27	42	24	33	2.80E-06	2.65E-06	1.82E-06	2.25E-06
F27E11.1	18	21	20	12	2.80E-06	2.65E-06	1.82E-06	2.25E-06
F27E11.2a.1	10	21	10	9	2.80E-06	2.65E-06	1.82E-06	2.25E-06
F27E11.2a.2	8	21	10	6	2.80E-06	2.65E-06	1.82E-06	2.25E-06
F27E11.2a.3	8	20	10	6	2.80E-06	2.65E-06	1.82E-06	2.25E-06
F27E11.2b	5	18	8	4	2.80E-06	2.65E-06	1.82E-06	2.25E-06
F27E11.3a	36	35	20	41	2.80E-06	2.65E-06	1.82E-06	2.25E-06
F27E11.3b	32	25	16	29	2.80E-06	2.65E-06	1.82E-06	2.25E-06
F27E11.t1	1	0	0	0	2.80E-06	2.65E-06	1.82E-06	2.25E-06
F27E5.1	226	160	338	266	2.80E-06	2.65E-06	1.82E-06	2.25E-06
F27E5.2	8	2	16	10	2.80E-06	2.65E-06	1.82E-06	2.25E-06
F27E5.3	34	49	37	23	2.10E-05	1.40E-05	2.04E-05	1.98E-05
F27E5.4	52	79	21	22	2.80E-06	2.65E-06	1.82E-06	2.25E-06
F27E5.5	8	10	7	5	2.80E-06	3.52E-06	1.82E-06	2.25E-06
F27E5.7	13	22	3	11	6.86E-06	9.84E-06	1.82E-06	2.34E-06
F27E5.8	5	8	14	5	2.80E-06	2.65E-06	1.82E-06	2.25E-06
F28A10.1	13	21	10	7	5.15E-06	8.25E-06	1.82E-06	3.51E-06
F28A10.10	2	0	2	0	2.80E-06	2.65E-06	1.82E-06	2.25E-06
F28A10.2	8	2	6	5	2.80E-06	2.65E-06	1.82E-06	2.25E-06
F28A10.3	2	3	1	1	2.80E-06	2.65E-06	1.82E-06	2.25E-06
F28A10.4	2	2	7	6	2.80E-06	2.65E-06	1.82E-06	2.25E-06
F28A10.5	5	9	2	4	2.80E-06	2.65E-06	1.82E-06	2.25E-06
F28A10.6	294	593	366	678	2.80E-06	2.65E-06	1.82E-06	2.25E-06
F28A10.7	2	5	3	1	2.80E-06	2.65E-06	1.82E-06	2.25E-06
F28A10.8	1	1	5	0	2.51E-05	4.78E-05	2.03E-05	4.65E-05
F28A10.9	1	5	3	5	2.80E-06	2.65E-06	1.82E-06	2.25E-06
F28A12.1	9	13	12	9	2.80E-06	2.65E-06	1.82E-06	2.25E-06
F28A12.2	6	13	6	3	2.80E-06	2.65E-06	1.82E-06	2.25E-06

F28A12.3	5	11	9	6	2.80E-06	2.65E-06	1.82E-06	2.25E-06
F28A12.4.1	743	2148	702	2979	2.80E-06	2.65E-06	1.82E-06	2.25E-06
F28A12.4.2	684	1970	643	2850	2.80E-06	2.65E-06	1.82E-06	2.25E-06
F28B1.1	6	6	5	2	6.67E-05	1.82E-04	4.10E-05	2.15E-04
F28B1.2	10	9	7	8	6.53E-05	1.78E-04	4.00E-05	2.19E-04
F28B1.3	10	9	3	2	2.80E-06	2.65E-06	1.82E-06	2.25E-06
F28B1.4	1	0	2	0	2.80E-06	2.65E-06	1.82E-06	2.25E-06
F28B1.5	3	6	4	4	2.80E-06	2.65E-06	1.82E-06	2.25E-06
F28B1.6	2	3	1	3	2.80E-06	2.65E-06	1.82E-06	2.25E-06
F28B1.7	2	3	2	4	2.80E-06	2.65E-06	1.82E-06	2.25E-06
F28B1.8	6	0	9	1	2.80E-06	2.65E-06	1.82E-06	2.25E-06
F28B12.2a	180	334	154	259	2.80E-06	2.65E-06	1.82E-06	2.25E-06
F28B12.2b	121	239	105	179	2.80E-06	2.65E-06	1.82E-06	2.25E-06
F28B12.2c	118	235	101	174	9.97E-06	1.75E-05	5.56E-06	1.15E-05
F28B12.2d	118	235	101	174	9.86E-06	1.84E-05	5.58E-06	1.17E-05
F28B12.2e.1	82	165	72	146	9.77E-06	1.84E-05	5.45E-06	1.16E-05
F28B12.2e.2	132	249	110	192	9.77E-06	1.84E-05	5.45E-06	1.16E-05
F28B12.2e.3	120	250	105	187	1.07E-05	2.03E-05	6.12E-06	1.53E-05
F28B12.2f.1	121	238	99	178	1.02E-05	1.81E-05	5.52E-06	1.19E-05
F28B12.2f.2	121	239	105	179	9.46E-06	1.86E-05	5.39E-06	1.18E-05
F28B12.3.1	639	680	994	1349	9.88E-06	1.84E-05	5.27E-06	1.17E-05
F28B12.3.2	535	562	837	1173	9.86E-06	1.84E-05	5.58E-06	1.17E-05
F28B3.1	546	621	663	1213	3.23E-05	3.24E-05	3.27E-05	5.47E-05
F28B3.10.1	158	252	269	184	3.27E-05	3.24E-05	3.33E-05	5.75E-05
F28B3.10.2	129	222	177	159	1.22E-05	1.31E-05	9.66E-06	2.18E-05
F28B3.3	50	68	59	109	2.84E-05	4.28E-05	3.15E-05	2.66E-05
F28B3.4	37	33	49	71	2.59E-05	4.22E-05	2.32E-05	2.57E-05
F28B3.5a	466	426	462	781	4.84E-06	6.22E-06	3.72E-06	8.48E-06
F28B3.5b	274	250	297	527	6.78E-06	5.71E-06	5.83E-06	1.04E-05
F28B3.7a.1	1703	1446	1964	2710	3.50E-05	3.02E-05	2.26E-05	4.71E-05
F28B3.7a.2	1605	1369	1815	2549	2.86E-05	2.46E-05	2.02E-05	4.41E-05
F28B3.7b	199	173	230	292	4.67E-05	3.75E-05	3.50E-05	5.97E-05
F28B3.8	1625	1206	1764	2522	4.66E-05	3.76E-05	3.43E-05	5.95E-05
F28B3.9	118	138	173	196	5.27E-05	4.33E-05	3.96E-05	6.21E-05
F28B4.1	5	6	5	2	5.42E-05	3.80E-05	3.83E-05	6.75E-05
F28B4.2	119	212	120	197	9.21E-06	1.02E-05	8.78E-06	1.23E-05
F28B4.4	6	12	19	8	2.80E-06	2.65E-06	1.82E-06	2.25E-06
F28C1.1	306	310	207	411	4.23E-06	7.09E-06	2.77E-06	5.60E-06
F28C1.2	51	53	77	113	2.80E-06	2.65E-06	1.82E-06	2.25E-06
F28C1.3a	244	345	226	426	1.61E-05	1.54E-05	7.09E-06	1.74E-05
F28C1.3b	242	336	221	424	2.80E-06	2.65E-06	1.82E-06	3.24E-06
F28C10.1	1	2	4	1	9.04E-06	1.21E-05	5.45E-06	1.27E-05
F28C10.3	62	54	59	98	9.02E-06	1.18E-05	5.36E-06	1.27E-05
F28C10.4	29	15	18	6	2.80E-06	2.65E-06	1.82E-06	2.25E-06
F28C12.1	0	3	2	1	4.82E-06	3.94E-06	2.97E-06	6.09E-06
F28C12.2	6	1	7	1	2.80E-06	2.65E-06	1.82E-06	2.25E-06
F28C12.3	4	2	6	0	2.80E-06	2.65E-06	1.82E-06	2.25E-06
F28C12.4	1	2	1	2	2.80E-06	2.65E-06	1.82E-06	2.25E-06
F28C12.5	3	8	0	2	2.80E-06	2.65E-06	1.82E-06	2.25E-06
F28C12.6	2	3	5	5	2.80E-06	2.65E-06	1.82E-06	2.25E-06
F28C12.7	2	4	4	2	2.80E-06	2.65E-06	1.82E-06	2.25E-06
F28C6.1	31	26	37	52	2.80E-06	2.65E-06	1.82E-06	2.25E-06
F28C6.10	3	5	8	12	2.80E-06	2.65E-06	1.82E-06	2.25E-06
F28C6.2	295	247	393	536	2.80E-06	2.65E-06	1.82E-06	2.95E-06
F28C6.3	440	427	511	725	2.80E-06	2.65E-06	1.82E-06	2.32E-06
F28C6.4a.1	181	262	184	294	2.06E-05	1.62E-05	1.78E-05	3.00E-05
F28C6.4a.2	217	306	209	318	3.75E-05	3.44E-05	2.84E-05	4.97E-05
F28C6.4a.3	193	273	184	298	8.74E-06	1.19E-05	5.78E-06	1.14E-05
F28C6.4b.1	177	256	182	292	9.10E-06	1.21E-05	5.70E-06	1.07E-05

F28C6.4b.2	210	288	201	310	9.27E-06	1.24E-05	5.74E-06	1.15E-05
F28C6.4b.3	186	255	176	290	8.65E-06	1.18E-05	5.79E-06	1.15E-05
F28C6.5	36	66	22	15	9.41E-06	1.22E-05	5.87E-06	1.12E-05
F28C6.7a.1	6107	5528	22225	5470	9.63E-06	1.25E-05	5.92E-06	1.21E-05
F28C6.7a.2	5135	4657	18363	5018	6.10E-06	1.06E-05	2.42E-06	2.25E-06
F28C6.7b	4854	4325	17479	4718	1.45E-03	1.24E-03	3.43E-03	1.04E-03
F28C6.7c	4846	4308	17471	4712	1.22E-03	1.05E-03	2.85E-03	9.60E-04
F28C6.8	206	272	202	214	1.08E-03	9.06E-04	2.52E-03	8.40E-04
F28C6.9	5	7	2	2	1.15E-03	9.62E-04	2.69E-03	8.94E-04
F28D1.1.1	398	446	368	476	3.41E-05	4.26E-05	2.18E-05	2.85E-05
F28D1.1.2	357	403	326	450	2.80E-06	2.65E-06	1.82E-06	2.25E-06
F28D1.10	734	758	1102	1585	2.26E-05	2.39E-05	1.36E-05	2.17E-05
F28D1.11	338	183	398	142	2.29E-05	2.44E-05	1.36E-05	2.32E-05
F28D1.12	3	3	2	3	2.24E-05	2.18E-05	2.19E-05	3.88E-05
F28D1.2	54	48	25	44	1.01E-04	5.16E-05	7.73E-05	3.41E-05
F28D1.3	31	27	37	39	2.80E-06	2.65E-06	1.82E-06	2.25E-06
F28D1.4	5	3	0	1	6.16E-06	5.18E-06	1.86E-06	4.03E-06
F28D1.5	100	77	119	91	4.96E-06	4.07E-06	3.84E-06	4.99E-06
F28D1.6	24	28	23	9	2.80E-06	2.65E-06	1.82E-06	2.25E-06
F28D1.7.1	8653	10469	18064	6212	1.42E-05	1.03E-05	1.10E-05	1.04E-05
F28D1.7.2	8649	10458	18076	6201	3.89E-06	4.29E-06	2.42E-06	2.25E-06
F28D1.8	27	36	8	14	1.83E-03	2.09E-03	2.48E-03	1.05E-03
F28D1.9	216	398	138	176	1.94E-03	2.22E-03	2.64E-03	1.12E-03
F28D9.1	296	247	430	623	2.80E-06	3.52E-06	1.82E-06	2.25E-06
F28D9.2a	2	2	0	0	9.80E-06	1.71E-05	4.08E-06	6.41E-06
F28D9.2b	2	1	1	1	1.64E-05	1.29E-05	1.55E-05	2.76E-05
F28D9.3	4	4	10	5	2.80E-06	2.65E-06	1.82E-06	2.25E-06
F28D9.4	236	199	177	304	2.80E-06	2.65E-06	1.82E-06	2.25E-06
F28E10.1a	65	80	55	72	2.80E-06	2.65E-06	1.82E-06	2.25E-06
F28E10.1b.1	39	45	37	48	2.71E-05	2.16E-05	1.32E-05	2.80E-05
F28E10.1b.2	43	51	37	53	2.80E-06	2.65E-06	1.82E-06	2.25E-06
F28E10.1c	40	47	37	49	2.80E-06	2.65E-06	1.82E-06	2.25E-06
F28E10.1d	31	47	20	39	2.80E-06	2.65E-06	1.82E-06	2.25E-06
F28E10.2	18	48	8	21	2.80E-06	2.65E-06	1.82E-06	2.25E-06
F28E10.4	19	19	18	1	2.80E-06	2.65E-06	1.82E-06	2.25E-06
F28E10.5	21	24	11	13	2.80E-06	6.06E-06	1.82E-06	2.25E-06
F28F5.3a	222	292	181	282	2.80E-06	2.65E-06	1.82E-06	2.25E-06
F28F5.3b	179	225	156	229	3.42E-06	3.68E-06	1.82E-06	2.25E-06
F28F5.3c	190	255	128	224	8.06E-06	1.00E-05	4.28E-06	8.21E-06
F28F5.4	3	3	9	4	7.45E-06	8.86E-06	4.23E-06	7.67E-06
F28F5.6	45	57	34	16	6.41E-06	8.15E-06	2.81E-06	6.07E-06
F28F8.1	10	23	30	17	2.80E-06	2.65E-06	3.46E-06	2.25E-06
F28F8.10	14	20	14	18	9.18E-06	1.10E-05	4.52E-06	2.63E-06
F28F8.3.1	129	154	521	128	2.80E-06	2.65E-06	1.82E-06	2.25E-06
F28F8.3.2	115	129	416	124	2.80E-06	2.65E-06	1.82E-06	2.25E-06
F28F8.4	43	46	73	86	3.76E-05	4.24E-05	9.89E-05	3.00E-05
F28F8.5	100	119	168	159	1.83E-05	1.94E-05	4.32E-05	1.59E-05
F28F8.7	29	21	54	74	5.99E-06	6.06E-06	6.61E-06	9.63E-06
F28F8.8	2	10	16	11	1.79E-05	2.02E-05	1.96E-05	2.29E-05
F28F8.9	76	86	101	70	2.80E-06	2.65E-06	2.82E-06	4.77E-06
F28F9.2	1	2	0	3	2.80E-06	2.65E-06	1.82E-06	2.25E-06
F28F9.3	13	13	22	16	1.59E-05	1.70E-05	1.38E-05	1.18E-05
F28F9.4	7	10	7	5	2.80E-06	2.65E-06	1.82E-06	2.25E-06
F28G4.1	25	56	55	41	3.25E-06	3.07E-06	3.57E-06	3.22E-06
F28G4.2	4	12	9	11	2.80E-06	2.65E-06	1.82E-06	2.25E-06
F28G4.3	5	5	23	7	2.80E-06	3.57E-06	2.42E-06	2.25E-06
F28G4.4	10	10	7	2	2.80E-06	2.65E-06	1.82E-06	2.25E-06
F28G4.5	11	18	11	10	2.80E-06	2.65E-06	1.82E-06	2.25E-06
F28H1.1	32	62	31	45	2.80E-06	2.65E-06	1.82E-06	2.25E-06

F28H1.3.1	1882	1999	2162	2760	2.80E-06	2.65E-06	1.82E-06	2.25E-06
F28H1.3.2	1752	1856	1957	2486	5.32E-06	9.73E-06	3.35E-06	6.00E-06
F28H1.3.3	1758	1864	1962	2488	6.07E-05	6.09E-05	4.54E-05	7.15E-05
F28H1.4a	54	77	45	56	6.70E-05	6.71E-05	4.87E-05	7.64E-05
F28H1.4b	53	77	45	56	6.72E-05	6.73E-05	4.88E-05	7.64E-05
F28H1.5	12	11	8	4	7.45E-06	1.00E-05	4.03E-06	6.21E-06
F28H6.1a	69	151	72	84	7.53E-06	1.03E-05	4.15E-06	6.39E-06
F28H6.2	13	8	13	6	3.33E-06	2.88E-06	1.82E-06	2.25E-06
F28H6.3	13	9	7	6	3.92E-06	8.09E-06	2.66E-06	3.82E-06
F28H6.4	114	114	121	178	2.80E-06	2.65E-06	1.82E-06	2.25E-06
F28H6.6	85	119	99	95	2.80E-06	2.65E-06	1.82E-06	2.25E-06
F28H6.7	0	1	0	1	4.03E-06	3.81E-06	2.77E-06	5.04E-06
F28H6.8	0	2	1	0	3.50E-06	4.63E-06	2.66E-06	3.15E-06
F28H6.t1	0	0	4	2	2.80E-06	2.65E-06	1.82E-06	2.25E-06
F28H7.1	5	8	8	5	2.80E-06	2.65E-06	1.82E-06	2.25E-06
F28H7.10a	9	20	35	11	2.80E-06	2.65E-06	3.99E-06	2.47E-06
F28H7.11	8	5	11	3	2.80E-06	2.65E-06	1.82E-06	2.25E-06
F28H7.2	8	3	7	4	2.80E-06	2.65E-06	1.82E-06	2.25E-06
F28H7.3	620	628	816	597	2.80E-06	2.65E-06	1.82E-06	2.25E-06
F28H7.4	11	11	15	4	2.80E-06	2.65E-06	1.82E-06	2.25E-06
F28H7.6	25	58	17	15	7.12E-05	6.81E-05	6.10E-05	5.51E-05
F28H7.7	9	8	18	9	2.80E-06	2.65E-06	1.82E-06	2.25E-06
F28H7.8	44	109	38	55	2.80E-06	3.84E-06	1.82E-06	2.25E-06
F28H7.9	10	10	10	5	2.80E-06	2.65E-06	1.82E-06	2.25E-06
F29A7.1	7	4	15	3	4.00E-06	9.36E-06	2.24E-06	4.00E-06
F29A7.2	5	3	4	1	2.80E-06	2.65E-06	1.82E-06	2.25E-06
F29A7.3	1	0	1	1	2.80E-06	2.65E-06	1.82E-06	2.25E-06
F29A7.4	5	5	11	2	2.80E-06	2.65E-06	1.82E-06	2.25E-06
F29A7.5	0	0	1	0	2.80E-06	2.65E-06	1.82E-06	2.25E-06
F29A7.7	3	4	3	0	2.80E-06	2.65E-06	1.82E-06	2.25E-06
F29A7.8	4	0	0	1	2.80E-06	2.65E-06	1.82E-06	2.25E-06
F29B9.1	105	107	141	205	2.80E-06	2.65E-06	1.82E-06	2.25E-06
F29B9.10	95	112	238	78	2.80E-06	2.65E-06	1.82E-06	2.25E-06
F29B9.11.1	1923	1603	5677	982	1.27E-05	1.22E-05	1.11E-05	1.99E-05
F29B9.11.2	1519	1316	4378	846	4.49E-05	5.00E-05	7.32E-05	2.96E-05
F29B9.12	9	8	3	5	5.32E-04	4.19E-04	1.02E-03	2.18E-04
F29B9.2a	725	700	877	1263	4.14E-04	3.39E-04	7.76E-04	1.85E-04
F29B9.2b	671	612	771	1188	2.80E-06	2.65E-06	1.82E-06	2.25E-06
F29B9.4a.1	221	307	344	345	2.70E-05	2.47E-05	2.13E-05	3.78E-05
F29B9.4a.2	208	283	315	313	2.79E-05	2.40E-05	2.08E-05	3.96E-05
F29B9.5	19	56	138	51	1.05E-05	1.38E-05	1.07E-05	1.32E-05
F29B9.6.1	1844	1464	3791	2272	9.91E-06	1.27E-05	9.77E-06	1.20E-05
F29B9.6.2	616	606	1139	1119	2.80E-06	6.64E-06	1.13E-05	5.13E-06
F29B9.7	18	26	7	8	1.64E-04	1.23E-04	2.20E-04	1.63E-04
F29B9.8.1	380	511	300	522	1.23E-04	1.15E-04	1.49E-04	1.80E-04
F29B9.8.2	298	428	230	444	4.31E-06	5.87E-06	1.82E-06	2.25E-06
F29B9.9	78	131	34	43	2.28E-05	2.89E-05	1.17E-05	2.51E-05
F29C12.1b	484	580	204	169	2.91E-05	3.95E-05	1.46E-05	3.48E-05
F29C12.3	617	634	863	1221	8.74E-06	1.39E-05	2.48E-06	3.87E-06
F29C12.4	677	664	538	814	2.78E-05	3.15E-05	7.63E-06	7.80E-06
F29C12.5	1	4	11	5	1.34E-05	1.30E-05	1.22E-05	2.13E-05
F29C12.6	0	2	3	1	3.06E-05	2.84E-05	1.58E-05	2.96E-05
F29C12.7	22	22	10	5	2.80E-06	2.65E-06	1.82E-06	2.25E-06
F29C4.1a	144	147	139	202	2.80E-06	2.65E-06	1.82E-06	2.25E-06
F29C4.1b	114	111	103	147	1.91E-05	1.80E-05	5.65E-06	3.49E-06
F29C4.2.1	406	513	1436	378	6.47E-06	6.24E-06	4.06E-06	7.29E-06
F29C4.2.2	274	319	945	259	6.24E-06	5.74E-06	3.68E-06	6.48E-06
F29C4.3	10	13	24	16	1.13E-04	1.34E-04	2.59E-04	8.42E-05
F29C4.4.1	3	4	3	1	1.11E-04	1.22E-04	2.50E-04	8.44E-05

F29C4.4.2	3	2	3	0	2.80E-06	2.65E-06	1.82E-06	2.25E-06
F29C4.5	4	7	3	6	2.80E-06	2.65E-06	1.82E-06	2.25E-06
F29C4.6.1	171	133	143	191	2.80E-06	2.65E-06	1.82E-06	2.25E-06
F29C4.6.2	163	127	158	195	2.80E-06	2.65E-06	1.82E-06	2.25E-06
F29C4.7b.1	320	331	494	592	1.53E-05	1.13E-05	8.35E-06	1.38E-05
F29C4.7b.2	227	231	318	394	1.61E-05	1.19E-05	1.02E-05	1.55E-05
F29C4.7c	263	270	375	477	1.80E-05	1.76E-05	1.81E-05	2.67E-05
F29C4.8	32	37	31	38	1.99E-05	1.91E-05	1.81E-05	2.77E-05
F29C4.t1	0	0	4	2	1.96E-05	1.90E-05	1.82E-05	2.86E-05
F29C6.1a	111	279	99	110	2.80E-06	2.65E-06	1.82E-06	2.25E-06
F29C6.1b.1	58	166	42	64	2.80E-06	2.65E-06	3.99E-06	2.47E-06
F29C6.1b.2	34	84	18	37	1.52E-05	3.62E-05	8.84E-06	1.21E-05
F29D10.1	10	13	15	11	9.38E-06	2.53E-05	4.41E-06	8.30E-06
F29D10.2	8	13	3	7	8.51E-06	1.99E-05	2.93E-06	7.44E-06
F29D10.3	10	6	5	3	2.80E-06	3.07E-06	2.44E-06	2.25E-06
F29D10.4	702	728	1014	1366	2.80E-06	2.96E-06	1.82E-06	2.25E-06
F29D11.1	1334	2130	1254	1845	2.80E-06	2.65E-06	1.82E-06	2.25E-06
F29D11.2	1385	1156	1825	2563	2.23E-05	2.19E-05	2.10E-05	3.49E-05
F29F11.1.1	682	945	833	942	1.02E-05	1.54E-05	6.25E-06	1.14E-05
F29F11.1.2	622	878	679	875	4.07E-05	3.21E-05	3.49E-05	6.05E-05
F29F11.2	12	15	14	11	4.82E-05	6.31E-05	3.83E-05	5.35E-05
F29F11.3	172	181	186	226	4.81E-05	6.41E-05	3.42E-05	5.44E-05
F29F11.4	18	29	36	29	2.80E-06	2.65E-06	1.82E-06	2.25E-06
F29G6.1	50	46	23	26	1.82E-05	1.81E-05	1.28E-05	1.92E-05
F29G6.2	10	11	7	9	2.80E-06	2.65E-06	1.82E-06	2.25E-06
F29G6.3a	1525	1919	1735	2601	2.80E-06	2.65E-06	1.82E-06	2.25E-06
F29G6.3b.1	1730	1839	1864	2775	2.80E-06	2.65E-06	1.82E-06	2.25E-06
F29G6.3b.2	1677	1774	1804	2714	7.23E-05	8.60E-05	5.35E-05	9.91E-05
F29G6.3c.1	2033	2413	2205	3271	3.51E-05	3.53E-05	2.46E-05	4.53E-05
F29G6.3c.2	2014	2381	2183	3248	3.48E-05	3.48E-05	2.43E-05	4.52E-05
F29G9.1	27	33	14	30	4.97E-05	5.57E-05	3.51E-05	6.43E-05
F29G9.2a	642	631	1235	1316	5.01E-05	5.59E-05	3.53E-05	6.48E-05
F29G9.2b.1	550	494	1029	1182	5.46E-06	6.30E-06	1.84E-06	4.86E-06
F29G9.2b.2	584	528	1111	1229	3.94E-05	3.66E-05	4.93E-05	6.49E-05
F29G9.3	263	263	303	186	3.89E-05	3.30E-05	4.73E-05	6.71E-05
F29G9.4a	109	248	107	183	4.04E-05	3.45E-05	5.00E-05	6.83E-05
F29G9.4b	109	243	100	180	4.89E-05	4.62E-05	3.67E-05	2.78E-05
F29G9.5	1329	1494	1561	2056	1.00E-05	2.15E-05	6.40E-06	1.35E-05
F29G9.6a	49	60	73	71	8.62E-06	1.82E-05	5.16E-06	1.14E-05
F29G9.6b	34	47	40	45	1.02E-04	1.08E-04	7.80E-05	1.27E-04
F29G9.7	19	19	15	29	3.78E-06	4.36E-06	3.66E-06	4.41E-06
F30A10.1	34	37	31	27	4.42E-06	5.77E-06	3.39E-06	4.70E-06
F30A10.10	1317	1067	1345	2250	2.80E-06	2.65E-06	1.82E-06	3.01E-06
F30A10.11	2	2	4	0	3.84E-06	3.94E-06	2.28E-06	2.45E-06
F30A10.12	26	25	9	4	3.56E-05	2.73E-05	2.37E-05	4.89E-05
F30A10.2	54	152	44	22	2.80E-06	2.65E-06	1.82E-06	2.25E-06
F30A10.3.1	496	493	642	762	6.75E-06	6.11E-06	1.82E-06	2.25E-06
F30A10.3.2	379	363	481	606	7.25E-06	1.93E-05	3.84E-06	2.38E-06
F30A10.4	13	15	17	5	3.96E-05	3.72E-05	3.34E-05	4.89E-05
F30A10.5.1	366	455	304	365	4.03E-05	3.64E-05	3.33E-05	5.17E-05
F30A10.5.2	297	356	238	332	2.80E-06	2.65E-06	1.82E-06	2.25E-06
F30A10.6	615	828	868	1073	3.58E-05	4.21E-05	1.94E-05	2.87E-05
F30A10.7	15	20	14	2	3.37E-05	3.82E-05	1.76E-05	3.02E-05
F30A10.8a	70	97	32	54	3.63E-05	4.62E-05	3.34E-05	5.09E-05
F30A10.8b	52	74	24	46	2.80E-06	2.65E-06	1.82E-06	2.25E-06
F30A10.9	184	371	266	243	5.60E-06	7.33E-06	1.82E-06	3.46E-06
F30B5.1	1653	1994	854	533	4.40E-06	5.90E-06	1.82E-06	3.13E-06
F30B5.4.1	368	183	537	294	2.39E-05	4.56E-05	2.25E-05	2.54E-05
F30B5.4.2	307	146	437	251	1.89E-04	2.15E-04	6.35E-05	4.89E-05

F30B5.6	3	1	2	2	2.29E-05	1.08E-05	2.18E-05	1.47E-05
F30B5.7	13	22	13	11	2.37E-05	1.06E-05	2.19E-05	1.55E-05
F30B5.8	5	2	0	1	2.80E-06	2.65E-06	1.82E-06	2.25E-06
F30B5.9	1	4	12	6	2.80E-06	2.65E-06	1.82E-06	2.25E-06
F30D4.1	1	3	1	1	2.80E-06	2.65E-06	1.82E-06	2.25E-06
F30F8.1	683	622	1274	1493	2.80E-06	2.65E-06	1.82E-06	2.25E-06
F30F8.2	48	110	41	45	2.80E-06	2.65E-06	1.82E-06	2.25E-06
F30F8.3	599	570	648	824	3.49E-05	3.00E-05	4.23E-05	6.12E-05
F30F8.8.1	374	488	423	695	2.83E-06	6.14E-06	1.82E-06	2.25E-06
F30F8.8.2	368	488	444	722	6.81E-05	6.12E-05	4.79E-05	7.53E-05
F30F8.8.3	351	475	405	675	1.87E-05	2.30E-05	1.38E-05	2.79E-05
F30F8.9b.2	216	228	174	216	1.91E-05	2.39E-05	1.50E-05	3.01E-05
F30H5.1	278	280	475	638	2.01E-05	2.57E-05	1.51E-05	3.10E-05
F30H5.2	4	4	3	5	2.79E-05	2.78E-05	1.46E-05	2.24E-05
F30H5.3	413	468	160	153	1.08E-05	1.03E-05	1.20E-05	1.99E-05
F30H5.4	2	2	3	1	2.80E-06	2.65E-06	1.82E-06	2.25E-06
F30H5.5	0	1	8	2	8.68E-06	9.31E-06	2.19E-06	2.59E-06
F31A3.1	2	3	6	0	2.80E-06	2.65E-06	2.51E-06	2.25E-06
F31A3.2	8	6	6	4	2.80E-06	2.65E-06	1.82E-06	2.25E-06
F31A3.3	3	9	14	9	2.80E-06	2.65E-06	1.82E-06	2.25E-06
F31A3.4	8	7	3	3	2.80E-06	2.65E-06	1.82E-06	2.25E-06
F31A3.5	93	91	89	148	2.80E-06	2.65E-06	1.99E-06	2.25E-06
F31A9.1	1	0	1	2	2.80E-06	2.65E-06	1.82E-06	2.25E-06
F31A9.2	4	6	7	2	4.00E-06	3.70E-06	2.50E-06	5.13E-06
F31A9.3a	3	1	0	2	2.80E-06	2.65E-06	1.82E-06	2.25E-06
F31A9.3b	3	2	0	1	2.80E-06	2.65E-06	1.82E-06	2.25E-06
F31A9.4	2	10	6	1	2.80E-06	2.65E-06	1.82E-06	2.25E-06
F31B12.1a	93	141	70	167	2.80E-06	2.65E-06	1.82E-06	2.25E-06
F31B12.1b	95	145	64	170	2.80E-06	2.65E-06	1.82E-06	2.25E-06
F31B12.1c	96	146	64	166	2.80E-06	2.65E-06	1.82E-06	2.52E-06
F31B12.2	19	31	12	26	2.80E-06	2.65E-06	1.82E-06	2.59E-06
F31B12.3	7	18	8	8	2.80E-06	2.65E-06	1.82E-06	2.54E-06
F31B12.4	1	6	2	1	2.80E-06	2.65E-06	1.82E-06	2.25E-06
F31B9.1	6	4	1	2	2.80E-06	2.65E-06	1.82E-06	2.25E-06
F31B9.2	5	7	5	2	2.80E-06	2.65E-06	1.82E-06	2.25E-06
F31B9.3	27	32	44	41	2.80E-06	2.65E-06	1.82E-06	2.25E-06
F31B9.4	7	12	3	6	2.80E-06	2.65E-06	1.82E-06	2.25E-06
F31C3.1.1	1457	1773	2712	1495	4.68E-06	5.24E-06	4.97E-06	5.71E-06
F31C3.1.2	1336	1629	2363	1399	2.80E-06	2.65E-06	1.82E-06	2.25E-06
F31C3.10	3796	4941	22333	45936	2.02E-04	2.33E-04	2.45E-04	1.67E-04
F31C3.11	179	146	409	476	2.33E-04	2.69E-04	2.69E-04	1.96E-04
F31C3.2a	413	493	790	1023	4.99E-04	6.14E-04	1.91E-03	4.85E-03
F31C3.2b	382	458	744	1008	1.31E-04	1.01E-04	1.95E-04	2.80E-04
F31C3.3	1187	920	1296	2156	1.85E-05	2.08E-05	2.30E-05	3.67E-05
F31C3.4	549	514	924	1185	1.76E-05	2.00E-05	2.23E-05	3.74E-05
F31C3.5	104	105	265	184	2.04E-05	1.49E-05	1.45E-05	2.97E-05
F31C3.6a	28	72	29	34	4.97E-05	4.39E-05	5.44E-05	8.61E-05
F31C3.7	32383	23126	84992	142771	2.14E-05	2.04E-05	3.56E-05	3.05E-05
F31C3.8	32383	23126	84992	142771	2.80E-06	3.73E-06	1.82E-06	2.25E-06
F31C3.9	27316	39518	178996	325471	2.07E-03	1.39E-03	3.53E-03	7.32E-03
F31D4.1	197	179	217	293	2.07E-03	1.39E-03	3.53E-03	7.32E-03
F31D4.2	485	421	979	1058	8.72E-04	1.19E-03	3.72E-03	8.34E-03
F31D4.3.1	1106	1226	1337	1658	2.17E-05	1.86E-05	1.56E-05	2.60E-05
F31D4.3.2	1104	1267	1335	1594	2.80E-05	2.30E-05	3.68E-05	4.91E-05
F31D4.4	11	25	12	15	7.60E-05	7.96E-05	5.98E-05	9.15E-05
F31D4.5	25	28	19	27	9.38E-05	1.02E-04	7.38E-05	1.09E-04
F31D4.6	3	8	4	2	2.80E-06	2.65E-06	1.82E-06	2.25E-06
F31D4.7	7	14	12	11	2.80E-06	2.65E-06	1.82E-06	2.25E-06
F31D4.8	54	66	64	38	2.80E-06	2.65E-06	1.82E-06	2.25E-06

F31D5.1	14	8	9	12	2.80E-06	2.65E-06	1.82E-06	2.25E-06
F31D5.2	37	53	54	57	8.48E-06	9.79E-06	6.54E-06	4.79E-06
F31D5.3a	390	474	327	516	2.80E-06	2.65E-06	1.82E-06	2.25E-06
F31D5.3b	422	508	342	542	2.88E-06	3.89E-06	2.73E-06	3.55E-06
F31D5.3c	153	194	120	189	1.33E-05	1.53E-05	7.25E-06	1.41E-05
F31D5.3d	304	368	243	376	1.33E-05	1.51E-05	7.02E-06	1.37E-05
F31D5.4	35	45	43	54	8.96E-06	1.07E-05	4.57E-06	8.88E-06
F31D5.5	60	51	41	40	1.28E-05	1.46E-05	6.65E-06	1.27E-05
F31D5.6	5	3	8	2	2.80E-06	2.65E-06	1.82E-06	2.25E-06
F31E3.1	107	156	89	154	2.80E-06	2.65E-06	1.82E-06	2.25E-06
F31E3.2a	13	28	17	12	2.80E-06	2.65E-06	1.82E-06	2.25E-06
F31E3.2b	15	26	14	14	9.32E-06	1.29E-05	5.05E-06	1.08E-05
F31E3.2c	3	9	3	5	2.80E-06	2.65E-06	1.82E-06	2.25E-06
F31E3.3	457	659	730	674	2.80E-06	2.65E-06	1.82E-06	2.25E-06
F31E3.4	517	538	748	941	2.80E-06	2.65E-06	1.82E-06	2.25E-06
F31E3.5.1	60182	62010	83761	99892	4.90E-05	6.67E-05	5.09E-05	5.80E-05
F31E3.5.2	57693	59156	78840	97541	1.55E-05	1.53E-05	1.46E-05	2.27E-05
F31E3.6	167	140	364	169	4.41E-03	4.29E-03	4.00E-03	5.88E-03
F31E8.1	4	8	2	1	4.17E-03	4.04E-03	3.71E-03	5.66E-03
F31E8.2a	65	89	56	55	4.05E-05	3.21E-05	5.74E-05	3.29E-05
F31E8.2b	47	57	35	46	2.80E-06	2.65E-06	1.82E-06	2.25E-06
F31E8.3	3	4	2	5	4.06E-06	5.26E-06	2.28E-06	2.77E-06
F31E8.4	113	117	131	117	3.95E-06	4.52E-06	1.91E-06	3.10E-06
F31E8.5	51	109	33	25	2.80E-06	2.65E-06	1.82E-06	2.25E-06
F31E8.6	5	7	6	7	2.59E-05	2.53E-05	1.95E-05	2.15E-05
F31E9.1	9	22	10	16	3.92E-06	7.94E-06	1.82E-06	2.25E-06
F31E9.2	2	3	7	2	2.80E-06	2.65E-06	1.82E-06	2.25E-06
F31E9.3	10	21	9	3	2.80E-06	2.65E-06	1.82E-06	2.25E-06
F31E9.4	9	11	11	4	2.80E-06	2.65E-06	1.82E-06	2.25E-06
F31E9.5	4	6	13	1	2.80E-06	2.65E-06	1.82E-06	2.25E-06
F31E9.6	11	23	12	27	2.80E-06	2.65E-06	1.82E-06	2.25E-06
F31E9.7	3	6	10	5	2.80E-06	2.65E-06	1.82E-06	2.25E-06
F31E9.t1	0	0	6	0	2.80E-06	2.65E-06	1.82E-06	2.41E-06
F31F4.1	13	13	8	17	2.80E-06	2.65E-06	1.82E-06	2.25E-06
F31F4.10	2	3	9	2	2.80E-06	2.65E-06	5.99E-06	2.25E-06
F31F4.11	5	8	23	5	2.80E-06	2.65E-06	1.82E-06	2.25E-06
F31F4.12	11	32	17	16	2.80E-06	2.65E-06	1.82E-06	2.25E-06
F31F4.13	2	4	5	8	2.80E-06	2.65E-06	1.97E-06	2.25E-06
F31F4.14	6	7	9	6	2.80E-06	2.83E-06	1.82E-06	2.25E-06
F31F4.15	149	560	133	363	2.80E-06	2.65E-06	1.82E-06	2.25E-06
F31F4.16	5	6	3	1	2.80E-06	2.65E-06	1.82E-06	2.25E-06
F31F4.17	9	7	19	13	1.60E-05	5.68E-05	9.29E-06	3.13E-05
F31F4.18	6	2	4	1	2.80E-06	2.65E-06	1.82E-06	2.25E-06
F31F4.2	3	1	6	3	2.80E-06	2.65E-06	1.82E-06	2.25E-06
F31F4.3	3	5	5	4	2.80E-06	2.65E-06	1.82E-06	2.25E-06
F31F4.4	5	4	1	2	2.80E-06	2.65E-06	1.82E-06	2.25E-06
F31F4.6	2	3	0	3	2.80E-06	2.65E-06	1.82E-06	2.25E-06
F31F4.7	12	16	16	10	2.80E-06	2.65E-06	1.82E-06	2.25E-06
F31F4.8	6	8	4	1	2.80E-06	2.65E-06	1.82E-06	2.25E-06
F31F4.9	1	6	5	1	2.80E-06	2.65E-06	1.82E-06	2.25E-06
F31F6.1	72	68	115	97	2.80E-06	2.65E-06	1.82E-06	2.25E-06
F31F6.2	54	37	90	97	2.80E-06	2.65E-06	1.82E-06	2.25E-06
F31F6.3	56	53	112	85	8.57E-06	7.64E-06	8.89E-06	9.27E-06
F31F6.4a	25	38	23	4	6.75E-06	4.36E-06	7.31E-06	9.74E-06
F31F6.4b	85	94	79	56	7.31E-06	6.53E-06	9.51E-06	8.91E-06
F31F6.5	39	81	26	29	6.86E-06	9.87E-06	4.10E-06	2.25E-06
F31F6.6	68	96	114	96	1.51E-05	1.58E-05	9.15E-06	8.01E-06
F31F6.7	4	5	4	3	2.80E-06	2.99E-06	1.82E-06	2.25E-06
F31F6.8	4	9	9	2	3.95E-06	5.24E-06	4.30E-06	4.45E-06

F31F7.1a	126	397	207	230	2.80E-06	2.65E-06	1.82E-06	2.25E-06
F31F7.1b.1	91	296	159	168	2.80E-06	2.65E-06	1.82E-06	2.25E-06
F31F7.1b.2	99	309	167	181	9.16E-06	2.72E-05	9.78E-06	1.34E-05
F31F7.1c	100	325	168	176	9.07E-06	2.79E-05	1.03E-05	1.34E-05
F31F7.2	44	40	31	22	9.97E-06	2.94E-05	1.09E-05	1.46E-05
F31F7.3	14	11	6	10	1.07E-05	3.28E-05	1.17E-05	1.51E-05
F32A11.1	367	379	523	620	2.80E-06	2.65E-06	1.82E-06	2.25E-06
F32A11.2	187	194	278	367	3.00E-06	2.65E-06	1.82E-06	2.25E-06
F32A11.3	147	236	105	66	3.23E-05	3.15E-05	2.99E-05	4.38E-05
F32A11.4	107	64	84	120	1.27E-05	1.25E-05	1.23E-05	2.01E-05
F32A11.5	49	26	42	62	1.35E-05	2.05E-05	6.29E-06	4.88E-06
F32A11.6	60	87	33	51	6.10E-06	3.44E-06	3.12E-06	5.49E-06
F32A11.7	46	38	25	12	2.80E-06	2.65E-06	1.82E-06	2.65E-06
F32A5.1a	304	450	327	474	6.08E-06	8.33E-06	2.19E-06	4.16E-06
F32A5.1b	250	370	240	412	2.80E-06	2.65E-06	1.82E-06	2.25E-06
F32A5.2a	513	540	467	533	1.50E-05	2.10E-05	1.05E-05	1.88E-05
F32A5.2b	484	512	443	509	1.59E-05	2.23E-05	9.95E-06	2.11E-05
F32A5.3	572	583	546	640	1.79E-05	1.79E-05	1.06E-05	1.50E-05
F32A5.4a.1	376	583	185	186	2.10E-05	2.10E-05	1.25E-05	1.77E-05
F32A5.4a.2	297	472	141	157	3.54E-05	3.40E-05	2.20E-05	3.18E-05
F32A5.4b	260	416	135	143	3.35E-05	4.90E-05	1.07E-05	1.33E-05
F32A5.5a	162	150	120	116	4.54E-05	6.81E-05	1.40E-05	1.93E-05
F32A5.5b.1	152	137	117	106	4.38E-05	6.63E-05	1.48E-05	1.94E-05
F32A5.5b.2	139	125	103	103	1.77E-05	1.55E-05	8.53E-06	1.02E-05
F32A5.6	31	78	23	41	1.90E-05	1.62E-05	9.51E-06	1.06E-05
F32A5.7	176	235	358	193	1.74E-05	1.48E-05	8.40E-06	1.04E-05
F32A5.8	99	136	77	73	3.42E-06	8.15E-06	1.82E-06	3.64E-06
F32A5.9	32	59	14	24	3.23E-05	4.07E-05	4.27E-05	2.84E-05
F32A6.1	4	6	2	1	1.43E-05	1.86E-05	7.25E-06	8.48E-06
F32A6.2	18	26	47	28	8.12E-06	1.42E-05	2.31E-06	4.90E-06
F32A6.3a	88	110	84	111	2.80E-06	2.65E-06	1.82E-06	2.25E-06
F32A6.3b	91	114	94	118	2.80E-06	2.96E-06	3.70E-06	2.72E-06
F32A6.4a	92	142	76	152	3.53E-06	4.15E-06	2.19E-06	3.58E-06
F32A6.4b	62	94	53	113	3.33E-06	3.97E-06	2.24E-06	3.49E-06
F32A6.4c	13	21	8	11	4.82E-06	7.01E-06	2.59E-06	6.39E-06
F32A6.5	21	22	7	13	3.92E-06	5.61E-06	2.19E-06	5.74E-06
F32A7.1	7	5	7	1	3.89E-06	5.92E-06	1.82E-06	2.63E-06
F32A7.2	3	3	2	4	2.80E-06	2.65E-06	1.82E-06	2.25E-06
F32A7.3a	22	28	16	30	2.80E-06	2.65E-06	1.82E-06	2.25E-06
F32A7.3b	20	26	15	29	2.80E-06	2.65E-06	1.82E-06	2.25E-06
F32A7.4	339	278	326	469	2.80E-06	2.65E-06	1.82E-06	2.25E-06
F32A7.5a	673	686	717	766	2.80E-06	2.65E-06	1.82E-06	2.25E-06
F32A7.5b	554	573	584	632	2.21E-05	1.71E-05	1.38E-05	2.46E-05
F32A7.5c	667	678	708	760	2.60E-05	2.50E-05	1.80E-05	2.37E-05
F32A7.6	3308	3071	2315	4031	2.42E-05	2.37E-05	1.66E-05	2.22E-05
F32A7.7	3	5	11	1	2.58E-05	2.48E-05	1.78E-05	2.36E-05
F32A7.8	1	1	0	0	2.13E-04	1.86E-04	9.68E-05	2.08E-04
F32B4.1	8	4	7	0	2.80E-06	2.65E-06	1.82E-06	2.25E-06
F32B4.2	22	34	23	6	2.80E-06	2.65E-06	1.82E-06	2.25E-06
F32B4.4a	197	192	222	251	2.80E-06	2.65E-06	1.82E-06	2.25E-06
F32B4.4b.1	202	186	207	240	4.59E-06	6.69E-06	3.12E-06	2.25E-06
F32B4.4b.2	199	182	204	236	9.52E-06	8.78E-06	6.98E-06	9.76E-06
F32B4.5	18	18	8	12	1.02E-05	8.83E-06	6.78E-06	9.69E-06
F32B4.6	2	6	4	1	1.04E-05	8.97E-06	6.92E-06	9.87E-06
F32B4.8	22	17	20	15	2.80E-06	2.65E-06	1.82E-06	2.25E-06
F32B5.1	597	1017	746	564	2.80E-06	2.65E-06	1.82E-06	2.25E-06
F32B5.2	13	11	10	10	2.80E-06	2.65E-06	1.82E-06	2.25E-06
F32B5.3	4	4	1	0	5.21E-05	8.38E-05	4.23E-05	3.95E-05
F32B5.4	30	55	46	18	2.80E-06	2.65E-06	1.82E-06	2.25E-06

F32B5.6a	140	315	117	207	2.80E-06	2.65E-06	1.82E-06	2.25E-06
F32B5.6b	141	308	118	204	8.93E-06	1.55E-05	8.91E-06	4.30E-06
F32B5.6c	33	72	22	43	9.10E-06	1.93E-05	4.96E-06	1.08E-05
F32B5.6d.1	90	189	82	141	8.34E-06	1.72E-05	4.54E-06	9.69E-06
F32B5.6d.2	92	201	92	141	7.03E-06	1.45E-05	3.06E-06	7.38E-06
F32B5.6d.3	98	213	90	152	8.15E-06	1.61E-05	4.83E-06	1.02E-05
F32B5.6d.4	83	174	80	131	8.51E-06	1.76E-05	5.54E-06	1.05E-05
F32B5.6d.5	94	208	90	151	8.46E-06	1.73E-05	5.05E-06	1.05E-05
F32B5.7.1	71	102	56	75	7.22E-06	1.43E-05	4.52E-06	9.15E-06
F32B5.7.2	61	87	49	72	9.69E-06	2.02E-05	6.03E-06	1.25E-05
F32B5.8	956	1581	1014	1685	2.91E-06	3.94E-06	1.82E-06	2.45E-06
F32B6.1.1	61	81	80	112	2.80E-06	3.68E-06	1.82E-06	2.59E-06
F32B6.1.2	63	82	85	115	1.05E-04	1.65E-04	7.27E-05	1.49E-04
F32B6.10	22	27	20	17	4.06E-06	5.08E-06	3.46E-06	5.98E-06
F32B6.11	5	3	9	3	4.76E-06	5.85E-06	4.17E-06	6.97E-06
F32B6.2.1	956	1095	851	1148	2.80E-06	2.65E-06	1.82E-06	2.25E-06
F32B6.3	239	264	285	239	2.80E-06	2.65E-06	1.82E-06	2.25E-06
F32B6.4	33	84	24	15	4.86E-05	5.26E-05	2.82E-05	4.69E-05
F32B6.5	258	563	242	134	2.38E-05	2.48E-05	1.85E-05	1.91E-05
F32B6.6	2033	2675	3246	709	5.18E-06	1.25E-05	2.46E-06	2.25E-06
F32B6.7	8	7	8	4	3.34E-05	6.89E-05	2.04E-05	1.39E-05
F32B6.8b.1	463	547	640	760	5.20E-04	6.46E-04	5.40E-04	1.46E-04
F32B6.8b.2	435	514	603	741	2.80E-06	2.65E-06	1.82E-06	2.25E-06
F32B6.9	53	85	41	71	2.98E-05	3.33E-05	2.68E-05	3.93E-05
F32D1.1	136	170	226	287	3.25E-05	3.62E-05	2.93E-05	4.44E-05
F32D1.10.1	2330	1985	3729	4533	4.79E-06	7.25E-06	2.41E-06	5.15E-06
F32D1.10.2	2110	1785	3336	4188	7.50E-06	8.86E-06	8.13E-06	1.27E-05
F32D1.11	27	24	9	16	1.07E-04	8.60E-05	1.11E-04	1.67E-04
F32D1.3	9	29	17	13	1.06E-04	8.49E-05	1.09E-04	1.69E-04
F32D1.4	7	5	6	1	5.66E-06	4.76E-06	1.82E-06	2.70E-06
F32D1.5.1	1587	2053	1640	1753	2.80E-06	2.65E-06	1.82E-06	2.25E-06
F32D1.5.2	1452	1842	1424	1567	2.80E-06	2.65E-06	1.82E-06	2.25E-06
F32D1.6	365	330	418	418	1.46E-04	1.78E-04	9.81E-05	1.29E-04
F32D1.7	466	362	666	798	1.35E-04	1.62E-04	8.61E-05	1.17E-04
F32D1.8	1	0	0	0	8.26E-05	7.05E-05	6.16E-05	7.60E-05
F32D8.1	4	15	3	4	4.82E-05	3.54E-05	4.49E-05	6.63E-05
F32D8.10	29	53	38	43	2.80E-06	2.65E-06	1.82E-06	2.25E-06
F32D8.12a	131	463	78	332	2.80E-06	2.65E-06	1.82E-06	2.25E-06
F32D8.12b.1	150	491	90	369	2.80E-06	3.97E-06	1.95E-06	2.72E-06
F32D8.12b.2	141	481	87	348	1.03E-05	3.43E-05	3.97E-06	2.09E-05
F32D8.12b.3	143	488	89	349	7.59E-06	2.35E-05	2.97E-06	1.50E-05
F32D8.12c.1	136	471	88	347	8.40E-06	2.71E-05	3.37E-06	1.67E-05
F32D8.12c.2	131	454	84	338	8.57E-06	2.76E-05	3.46E-06	1.68E-05
F32D8.12c.3	117	423	69	308	1.04E-05	3.41E-05	4.39E-06	2.14E-05
F32D8.12c.4	127	456	75	327	1.11E-05	3.65E-05	4.65E-06	2.31E-05
F32D8.14	357	324	433	359	9.21E-06	3.15E-05	3.53E-06	1.95E-05
F32D8.2	18	13	26	18	9.97E-06	3.38E-05	3.83E-06	2.06E-05
F32D8.3	17	24	7	6	4.65E-05	3.99E-05	3.67E-05	3.76E-05
F32D8.4	163	183	179	214	2.80E-06	2.65E-06	1.82E-06	2.25E-06
F32D8.5a	179	197	289	220	2.80E-06	3.44E-06	1.82E-06	2.25E-06
F32D8.6.1	1800	2057	3176	1072	1.46E-05	1.54E-05	1.04E-05	1.53E-05
F32D8.6.2	1282	1468	2101	825	2.72E-05	2.82E-05	2.85E-05	2.68E-05
F32D8.8	3	9	15	5	4.59E-04	4.96E-04	5.27E-04	2.20E-04
F32D8.9	116	118	260	77	3.27E-04	3.54E-04	3.49E-04	1.69E-04
F32E10.1	554	595	746	1061	2.80E-06	2.65E-06	1.82E-06	2.25E-06
F32E10.2	192	222	270	261	2.37E-05	2.27E-05	3.45E-05	1.26E-05
F32E10.3	318	473	125	109	2.50E-05	2.54E-05	2.19E-05	3.85E-05
F32E10.4.1	3974	3452	4038	6476	2.23E-05	2.44E-05	2.04E-05	2.44E-05
F32E10.4.2	2067	1863	2102	3224	1.29E-05	1.81E-05	3.30E-06	3.55E-06

F32E10.5	150	236	217	268	1.69E-04	1.38E-04	1.12E-04	2.21E-04
F32E10.6.1	953	1078	908	1266	1.49E-04	1.27E-04	9.89E-05	1.87E-04
F32E10.6.2	751	829	726	1088	1.01E-05	1.50E-05	9.48E-06	1.45E-05
F32E10.7	3	7	5	4	7.78E-05	8.31E-05	4.82E-05	8.30E-05
F32E10.8	6	10	3	5	7.42E-05	7.74E-05	4.67E-05	8.64E-05
F32E10.9	19	30	25	26	2.80E-06	2.65E-06	1.82E-06	2.25E-06
F32F2.1	131	228	138	148	2.80E-06	2.65E-06	1.82E-06	2.25E-06
F32G8.1	12	5	14	6	3.30E-06	4.92E-06	2.82E-06	3.62E-06
F32G8.2	4	10	13	8	5.26E-06	8.65E-06	3.61E-06	4.77E-06
F32G8.3	1	5	3	1	2.80E-06	2.65E-06	1.82E-06	2.25E-06
F32G8.4	241	473	187	285	2.80E-06	2.65E-06	1.82E-06	2.25E-06
F32G8.5	24	13	16	7	2.80E-06	2.65E-06	1.82E-06	2.25E-06
F32G8.6	288	335	208	242	1.92E-05	3.56E-05	9.69E-06	1.82E-05
F32H2.10	88	99	125	102	3.02E-06	2.65E-06	1.82E-06	2.25E-06
F32H2.11	6	17	5	6	4.56E-05	5.01E-05	2.14E-05	3.07E-05
F32H2.1a	591	705	849	1072	1.87E-05	1.99E-05	1.73E-05	1.75E-05
F32H2.1b	600	718	868	1090	2.80E-06	2.65E-06	1.82E-06	2.25E-06
F32H2.2	63	63	107	89	2.20E-05	2.48E-05	2.06E-05	3.21E-05
F32H2.3.1	1069	983	1619	1907	2.23E-05	2.52E-05	2.10E-05	3.26E-05
F32H2.3.2	1017	953	1541	1850	1.43E-05	1.35E-05	1.59E-05	1.63E-05
F32H2.4	185	215	234	272	4.53E-05	3.93E-05	4.46E-05	6.49E-05
F32H2.5	2389	2754	3119	3977	4.58E-05	4.06E-05	4.52E-05	6.70E-05
F32H2.6	2	7	6	7	2.10E-05	2.30E-05	1.73E-05	2.48E-05
F32H2.7	149	223	105	63	3.21E-05	3.50E-05	2.73E-05	4.29E-05
F32H2.8	1	4	7	5	2.80E-06	2.65E-06	1.82E-06	2.25E-06
F32H2.9	15	15	13	5	1.38E-05	1.95E-05	6.32E-06	4.68E-06
F32H5.1	117	173	90	155	2.80E-06	2.65E-06	1.82E-06	2.25E-06
F32H5.3a	9	23	21	29	2.80E-06	2.65E-06	1.82E-06	2.25E-06
F32H5.3b	8	19	20	33	1.14E-05	1.59E-05	5.70E-06	1.21E-05
F32H5.4	25	33	25	34	2.80E-06	4.21E-06	2.64E-06	4.52E-06
F32H5.5	3	7	4	1	2.80E-06	3.49E-06	2.53E-06	5.15E-06
F32H5.6a	4	3	6	6	2.80E-06	2.65E-06	1.82E-06	2.25E-06
F32H5.6b	2	1	3	2	2.80E-06	2.65E-06	1.82E-06	2.25E-06
F32H5.7	28	42	24	25	2.80E-06	2.65E-06	1.82E-06	2.25E-06
F33A8.1.1	561	575	758	1104	2.80E-06	2.65E-06	1.82E-06	2.25E-06
F33A8.1.2	536	546	729	1075	2.80E-06	2.70E-06	1.82E-06	2.25E-06
F33A8.10	14	14	6	8	2.24E-05	2.17E-05	1.97E-05	3.54E-05
F33A8.2.1	42	77	26	30	2.23E-05	2.14E-05	1.97E-05	3.58E-05
F33A8.2.2	40	77	25	30	2.80E-06	2.65E-06	1.82E-06	2.25E-06
F33A8.3.1	2304	2171	1134	2065	8.93E-06	1.54E-05	3.59E-06	5.13E-06
F33A8.3.2	956	818	440	776	8.60E-06	1.56E-05	3.50E-06	5.17E-06
F33A8.4.1	308	313	368	443	1.95E-04	1.74E-04	6.26E-05	1.41E-04
F33A8.5.1	657	612	487	525	1.52E-04	1.23E-04	4.54E-05	9.89E-05
F33A8.5.2	600	553	429	498	1.82E-05	1.75E-05	1.41E-05	2.10E-05
F33A8.6	14	13	21	15	1.14E-04	1.01E-04	5.52E-05	7.35E-05
F33A8.7	53	69	13	14	1.07E-04	9.35E-05	4.99E-05	7.16E-05
F33A8.9	8	11	5	7	2.80E-06	2.65E-06	2.10E-06	2.25E-06
F33C8.1a	355	550	396	485	1.14E-05	1.40E-05	1.82E-06	2.41E-06
F33C8.1b	322	512	377	463	2.80E-06	2.65E-06	1.82E-06	2.25E-06
F33C8.2	4	5	8	9	9.44E-06	1.38E-05	6.85E-06	1.04E-05
F33C8.3	70	119	78	60	9.74E-06	1.46E-05	7.42E-06	1.12E-05
F33C8.4	54	85	43	49	2.80E-06	2.65E-06	1.82E-06	2.25E-06
F33D11.1	3	6	6	1	9.04E-06	1.45E-05	6.56E-06	6.23E-06
F33D11.10.1	654	935	915	1324	9.46E-06	1.41E-05	4.90E-06	6.90E-06
F33D11.10.2	545	772	698	1091	2.80E-06	2.65E-06	1.82E-06	2.25E-06
F33D11.11	1359	1281	1724	2016	5.22E-05	7.05E-05	4.75E-05	8.49E-05
F33D11.12	255	372	303	367	5.05E-05	6.75E-05	4.20E-05	8.11E-05
F33D11.2	9	19	19	11	1.14E-04	1.01E-04	9.37E-05	1.35E-04
F33D11.3	100	137	29	30	2.13E-05	2.94E-05	1.65E-05	2.46E-05

F33D11.4	3	2	1	2	2.80E-06	2.65E-06	1.82E-06	2.25E-06
F33D11.5	13	11	20	11	1.32E-05	1.71E-05	2.50E-06	3.19E-06
F33D11.6	1	8	7	2	2.80E-06	2.65E-06	1.82E-06	2.25E-06
F33D11.7	29	51	15	13	2.80E-06	2.65E-06	1.82E-06	2.25E-06
F33D11.8	8	22	19	13	2.80E-06	2.65E-06	1.82E-06	2.25E-06
F33D11.9a.1	470	576	561	709	3.11E-06	5.16E-06	1.82E-06	2.25E-06
F33D11.9a.2	390	464	458	588	2.80E-06	7.09E-06	4.23E-06	3.58E-06
F33D11.9b	566	712	690	1004	4.31E-05	4.99E-05	3.35E-05	5.22E-05
F33D4.1a	100	188	103	145	4.91E-05	5.52E-05	3.76E-05	5.95E-05
F33D4.1b	101	188	98	149	3.12E-05	3.71E-05	2.48E-05	4.45E-05
F33D4.2d	661	922	710	1238	5.96E-06	1.06E-05	4.01E-06	6.95E-06
F33D4.2e	655	926	700	1241	6.10E-06	1.07E-05	3.84E-06	7.22E-06
F33D4.2g	334	412	392	623	8.32E-06	1.10E-05	5.81E-06	1.25E-05
F33D4.3	28	35	15	15	8.29E-06	1.11E-05	5.76E-06	1.26E-05
F33D4.4.1	545	623	724	980	6.89E-06	8.01E-06	5.25E-06	1.03E-05
F33D4.4.2	437	489	653	844	6.50E-06	7.67E-06	2.26E-06	2.79E-06
F33D4.5.1	499	608	483	486	4.35E-05	4.69E-05	3.76E-05	6.27E-05
F33D4.5.2	365	448	315	353	4.51E-05	4.76E-05	4.38E-05	6.99E-05
F33D4.6a	390	595	143	130	3.72E-05	4.28E-05	2.34E-05	2.91E-05
F33D4.6b	739	1084	238	201	3.60E-05	4.18E-05	2.02E-05	2.80E-05
F33D4.7	143	198	192	154	3.67E-05	5.28E-05	8.75E-06	9.83E-06
F33D4.8	165	284	320	185	3.91E-05	5.42E-05	8.20E-06	8.55E-06
F33E11.1	7	5	3	14	4.60E-05	6.02E-05	4.02E-05	3.98E-05
F33E11.2	257	177	405	358	3.18E-05	5.17E-05	4.01E-05	2.87E-05
F33E11.3	239	223	418	557	2.80E-06	2.65E-06	1.82E-06	2.25E-06
F33E11.6a	107	89	225	274	2.39E-05	1.56E-05	2.45E-05	2.67E-05
F33E11.6b	132	120	316	344	1.18E-05	1.04E-05	1.35E-05	2.21E-05
F33E2.2a	138	304	126	276	1.15E-05	8.99E-06	1.57E-05	2.35E-05
F33E2.2b	154	346	159	304	7.70E-06	6.61E-06	1.20E-05	1.61E-05
F33E2.2c	91	181	83	166	5.54E-06	1.15E-05	3.30E-06	8.88E-06
F33E2.3	5	4	9	5	5.54E-06	1.18E-05	3.74E-06	8.82E-06
F33E2.4	9	5	3	1	4.98E-06	9.36E-06	2.95E-06	7.31E-06
F33E2.6	11	27	20	12	2.80E-06	2.65E-06	1.82E-06	2.25E-06
F33E2.7	11	9	8	8	2.80E-06	2.65E-06	1.82E-06	2.25E-06
F33G12.2	102	148	102	199	2.80E-06	2.65E-06	1.82E-06	2.25E-06
F33G12.4.1	412	455	687	719	2.80E-06	2.65E-06	1.82E-06	2.25E-06
F33G12.4.2	411	453	679	713	1.24E-05	1.71E-05	8.09E-06	1.95E-05
F33G12.5.1	848	919	953	1430	3.02E-05	3.16E-05	3.28E-05	4.24E-05
F33G12.5.2	771	841	873	1333	3.05E-05	3.18E-05	3.28E-05	4.26E-05
F33G12.6a	95	166	84	138	3.01E-05	3.08E-05	2.20E-05	4.08E-05
F33G12.6b	94	163	83	133	3.04E-05	3.13E-05	2.24E-05	4.22E-05
F33G12.7	79	105	363	103	4.06E-06	6.69E-06	2.33E-06	4.75E-06
F33G12.t1	0	0	1	0	4.17E-06	6.82E-06	2.41E-06	4.75E-06
F33H1.1a	109	115	142	214	2.42E-05	3.04E-05	7.25E-05	2.54E-05
F33H1.1b	108	113	142	213	2.80E-06	2.65E-06	1.82E-06	2.25E-06
F33H1.2.1	2126	1947	3310	3197	4.40E-06	4.39E-06	3.74E-06	6.95E-06
F33H1.2.2	2146	1938	3400	3210	5.01E-06	4.95E-06	4.28E-06	7.92E-06
F33H1.3	561	436	643	927	2.23E-04	1.93E-04	2.26E-04	2.69E-04
F33H1.4	823	675	1171	1765	2.32E-04	1.98E-04	2.39E-04	2.79E-04
F33H1.5	6	4	1	4	5.82E-05	4.27E-05	4.34E-05	7.72E-05
F33H1.6	1	0	1	0	1.99E-05	1.54E-05	1.85E-05	3.43E-05
F33H12.1	4	3	4	1	2.80E-06	2.65E-06	1.82E-06	2.25E-06
F33H12.2	11	4	9	2	2.80E-06	2.65E-06	1.82E-06	2.25E-06
F33H12.3	17	12	4	5	2.80E-06	2.65E-06	1.82E-06	2.25E-06
F33H12.4	6	5	6	0	2.80E-06	2.65E-06	1.82E-06	2.25E-06
F33H12.5	6	1	22	9	3.11E-06	2.65E-06	1.82E-06	2.25E-06
F33H12.6	13	28	24	14	2.80E-06	2.65E-06	1.82E-06	2.25E-06
F33H2.1	394	321	499	706	2.80E-06	2.65E-06	1.82E-06	2.25E-06
F33H2.2	409	456	728	942	2.80E-06	2.65E-06	1.82E-06	2.25E-06

F33H2.5	910	935	1385	2117	1.41E-05	1.08E-05	1.16E-05	2.03E-05
F33H2.6	174	236	177	254	1.76E-05	1.85E-05	2.04E-05	3.26E-05
F33H2.7	29	39	34	33	1.57E-05	1.52E-05	1.55E-05	2.92E-05
F33H2.8	10	33	15	8	2.07E-05	2.65E-05	1.37E-05	2.43E-05
F34D10.2.1	494	412	928	1175	2.80E-06	3.20E-06	1.91E-06	2.29E-06
F34D10.3	12	15	17	8	2.80E-06	3.41E-06	1.82E-06	2.25E-06
F34D10.4.1	486	584	558	929	1.84E-05	1.45E-05	2.25E-05	3.52E-05
F34D10.4.2	382	443	458	766	2.83E-06	3.33E-06	2.61E-06	2.25E-06
F34D10.5	5	17	5	6	2.19E-05	2.49E-05	1.64E-05	3.37E-05
F34D10.6	8	9	13	5	2.04E-05	2.23E-05	1.59E-05	3.28E-05
F34D10.7.1	26	29	18	14	2.80E-06	2.65E-06	1.82E-06	2.25E-06
F34D10.7.2	25	27	16	13	2.80E-06	2.65E-06	1.82E-06	2.25E-06
F34D10.8	29	46	36	5	2.80E-06	2.65E-06	1.82E-06	2.25E-06
F34D6.1	13	19	19	10	2.80E-06	2.65E-06	1.82E-06	2.25E-06
F34D6.2	1	1	5	4	1.54E-05	2.31E-05	1.24E-05	2.25E-06
F34D6.3	7	7	6	7	2.80E-06	2.65E-06	1.82E-06	2.25E-06
F34D6.4	7	11	11	4	2.80E-06	2.65E-06	1.82E-06	2.25E-06
F34D6.5	5	9	3	1	2.80E-06	2.65E-06	1.82E-06	2.25E-06
F34D6.6	2	2	7	2	2.80E-06	2.65E-06	1.82E-06	2.25E-06
F34D6.7	3	3	1	2	2.80E-06	2.65E-06	1.82E-06	2.25E-06
F34D6.8	2	1	2	1	2.80E-06	2.65E-06	1.82E-06	2.25E-06
F34D6.9	1	5	2	1	2.80E-06	2.65E-06	1.82E-06	2.25E-06
F34H10.1	25	21	14	7	2.80E-06	2.65E-06	1.82E-06	2.25E-06
F34H10.2	2	0	0	3	2.80E-06	2.65E-06	1.82E-06	2.25E-06
F34H10.3.1	91	138	86	150	6.52E-06	5.18E-06	2.39E-06	2.25E-06
F34H10.4	14	21	9	13	2.80E-06	2.65E-06	1.82E-06	2.25E-06
F34H10.5	1	0	0	0	7.48E-06	1.07E-05	4.59E-06	9.90E-06
F34H10.t1	1	0	3	0	2.80E-06	2.65E-06	1.82E-06	2.25E-06
F35A5.1	230	241	119	225	2.80E-06	2.65E-06	1.82E-06	2.25E-06
F35A5.2	27	34	25	10	2.80E-06	2.65E-06	3.04E-06	2.25E-06
F35A5.3	45	53	14	12	6.75E-06	6.67E-06	2.26E-06	5.29E-06
F35A5.4	23	41	14	13	8.99E-06	1.07E-05	5.43E-06	2.68E-06
F35A5.5	4	5	2	2	4.00E-06	4.47E-06	1.82E-06	2.25E-06
F35A5.8a	70	64	52	76	2.80E-06	2.65E-06	1.82E-06	2.25E-06
F35A5.8b	111	121	105	153	2.80E-06	2.65E-06	1.82E-06	2.25E-06
F35B12.1	1	6	3	3	6.19E-06	5.34E-06	2.99E-06	5.40E-06
F35B12.10	33	70	32	41	9.72E-06	1.00E-05	5.98E-06	1.08E-05
F35B12.2	60	88	38	41	2.80E-06	2.65E-06	1.82E-06	2.25E-06
F35B12.3	97	175	74	82	2.80E-06	4.89E-06	1.82E-06	2.43E-06
F35B12.4	12	17	7	10	6.52E-06	9.05E-06	2.70E-06	3.58E-06
F35B12.5	465	477	731	756	6.52E-06	1.11E-05	3.23E-06	4.43E-06
F35B12.6	34	62	34	22	2.80E-06	2.72E-06	1.82E-06	2.25E-06
F35B12.7.1	583	528	820	156	2.85E-05	2.76E-05	2.92E-05	3.72E-05
F35B12.7.2	449	374	500	94	4.42E-06	7.62E-06	2.88E-06	2.29E-06
F35B12.8	13	13	6	9	1.71E-04	1.46E-04	1.56E-04	3.67E-05
F35B12.9	103	144	89	105	1.33E-04	1.04E-04	9.62E-05	2.23E-05
F35B3.1	4	4	4	5	2.80E-06	2.65E-06	1.82E-06	2.25E-06
F35B3.3	2	1	1	0	1.01E-05	1.34E-05	5.70E-06	8.30E-06
F35B3.4	69	286	18	52	2.80E-06	2.65E-06	1.82E-06	2.25E-06
F35B3.5a	233	247	161	322	2.80E-06	2.65E-06	1.82E-06	2.25E-06
F35B3.5b	84	98	58	123	9.58E-06	3.75E-05	1.82E-06	5.78E-06
F35B3.7	18	35	29	16	6.78E-06	6.77E-06	3.04E-06	7.51E-06
F35C11.1	4	9	8	15	5.77E-06	6.35E-06	2.59E-06	6.77E-06
F35C11.2	27	52	15	12	2.80E-06	2.65E-06	1.82E-06	2.25E-06
F35C11.3	31	65	17	6	2.80E-06	2.65E-06	1.82E-06	2.83E-06
F35C11.4	41	35	33	45	2.80E-06	4.60E-06	1.82E-06	2.25E-06
F35C11.5	615	612	917	1081	3.19E-06	6.32E-06	1.82E-06	2.25E-06
F35C11.6	11	39	14	13	4.23E-06	3.41E-06	2.22E-06	3.73E-06
F35C11.7	9	16	7	7	4.25E-05	3.99E-05	4.12E-05	6.00E-05

F35C12.1	3	5	1	0	2.80E-06	6.56E-06	1.82E-06	2.25E-06
F35C12.2a	30	44	26	32	2.80E-06	4.13E-06	1.82E-06	2.25E-06
F35C12.2b	30	44	26	32	2.80E-06	3.52E-06	1.82E-06	2.25E-06
F35C12.3a	52	84	37	37	2.80E-06	2.65E-06	1.82E-06	2.25E-06
F35C12.3b.1	52	69	42	40	2.80E-06	2.65E-06	1.82E-06	2.25E-06
F35C12.3b.2	41	57	23	33	5.26E-06	8.04E-06	2.44E-06	3.01E-06
F35C12.3c	56	89	45	42	7.92E-06	9.92E-06	4.15E-06	4.88E-06
F35C5.1	10	5	7	3	7.73E-06	1.02E-05	2.82E-06	4.99E-06
F35C5.10	2	2	4	2	6.10E-06	9.15E-06	3.19E-06	3.67E-06
F35C5.11	2	2	2	4	2.80E-06	2.65E-06	1.82E-06	2.25E-06
F35C5.12	12	20	23	8	2.80E-06	2.65E-06	1.82E-06	2.25E-06
F35C5.2	9	15	13	9	2.80E-06	2.65E-06	1.82E-06	2.25E-06
F35C5.3	5	5	4	4	2.80E-06	4.05E-06	3.21E-06	2.25E-06
F35C5.4	1	1	0	1	2.80E-06	2.65E-06	1.82E-06	2.25E-06
F35C5.5a	126	166	193	194	2.80E-06	2.65E-06	1.82E-06	2.25E-06
F35C5.5b	125	161	190	185	2.80E-06	2.65E-06	1.82E-06	2.25E-06
F35C5.6.1	5578	4905	7171	5389	1.15E-05	1.43E-05	1.15E-05	1.43E-05
F35C5.6.2	4913	4368	6568	5203	1.12E-05	1.36E-05	1.11E-05	1.33E-05
F35C5.7	12	20	8	7	4.75E-04	3.95E-04	3.97E-04	3.69E-04
F35C5.8	328	431	565	378	4.44E-04	3.73E-04	3.87E-04	3.78E-04
F35C5.9	133	130	151	95	2.80E-06	2.65E-06	1.82E-06	2.25E-06
F35C8.1	0	2	1	1	3.12E-05	3.88E-05	3.50E-05	2.89E-05
F35C8.2	15	30	14	14	1.26E-05	1.16E-05	9.29E-06	7.22E-06
F35C8.5	252	248	339	316	2.80E-06	2.65E-06	1.82E-06	2.25E-06
F35C8.6	496	644	469	622	2.80E-06	4.05E-06	1.82E-06	2.25E-06
F35C8.7a.1	254	246	521	576	3.12E-05	2.91E-05	2.74E-05	3.15E-05
F35C8.7a.2	249	238	513	575	6.57E-05	8.05E-05	4.04E-05	6.61E-05
F35C8.7b	259	245	528	601	1.17E-05	1.07E-05	1.56E-05	2.13E-05
F35C8.8	21	18	21	22	1.20E-05	1.08E-05	1.61E-05	2.22E-05
F35D11.1	2	3	8	2	1.16E-05	1.04E-05	1.55E-05	2.17E-05
F35D11.10	7	10	5	10	2.80E-06	2.65E-06	1.82E-06	2.25E-06
F35D11.11a	160	231	97	189	2.80E-06	2.65E-06	1.82E-06	2.25E-06
F35D11.11b	163	236	99	196	2.80E-06	2.65E-06	1.82E-06	2.25E-06
F35D11.11c	163	236	99	196	3.11E-06	4.23E-06	1.82E-06	2.95E-06
F35D11.11d	150	218	92	180	2.97E-06	4.07E-06	1.82E-06	2.88E-06
F35D11.2a.1	114	118	62	77	2.94E-06	4.02E-06	1.82E-06	2.83E-06
F35D11.2a.2	111	118	61	76	3.25E-06	4.44E-06	1.82E-06	3.13E-06
F35D11.2b	121	128	69	84	8.34E-06	8.15E-06	2.95E-06	4.52E-06
F35D11.3.1	45	80	49	67	8.12E-06	8.15E-06	2.90E-06	4.48E-06
F35D11.3.2	45	76	43	64	7.48E-06	7.49E-06	2.77E-06	4.18E-06
F35D11.5.1	91	112	99	77	2.80E-06	4.23E-06	1.82E-06	3.01E-06
F35D11.5.2	73	95	75	60	2.80E-06	4.23E-06	1.82E-06	3.04E-06
F35D11.7	5	5	0	3	1.79E-05	2.08E-05	1.27E-05	1.22E-05
F35D11.8	0	5	1	0	1.40E-05	1.72E-05	9.35E-06	9.22E-06
F35D11.9	2	2	3	7	2.80E-06	2.65E-06	1.82E-06	2.25E-06
F35D2.1	22	17	10	11	2.80E-06	2.65E-06	1.82E-06	2.25E-06
F35D2.2	21	30	11	8	2.80E-06	2.65E-06	1.82E-06	2.25E-06
F35D2.3	36	45	44	34	2.80E-06	2.65E-06	1.82E-06	2.25E-06
F35D2.4	12	18	18	20	2.80E-06	2.80E-06	1.82E-06	2.25E-06
F35D2.5a	19	31	16	29	3.44E-06	4.07E-06	2.75E-06	2.63E-06
F35D2.5b	20	33	18	32	2.80E-06	2.65E-06	1.82E-06	2.25E-06
F35D2.5c.1	19	31	19	28	2.80E-06	2.65E-06	1.82E-06	2.25E-06
F35D2.5c.2	15	23	14	24	2.80E-06	2.65E-06	1.82E-06	2.25E-06
F35D6.1a	440	513	524	686	2.80E-06	2.65E-06	1.82E-06	2.25E-06
F35D6.1b	41	50	49	63	2.80E-06	2.65E-06	1.82E-06	2.25E-06
F35E12.1	5	9	6	4	2.16E-05	2.38E-05	1.67E-05	2.70E-05
F35E12.10	257	418	318	396	1.18E-05	1.36E-05	9.20E-06	1.46E-05
F35E12.2	4	11	11	12	2.80E-06	2.65E-06	1.82E-06	2.25E-06
F35E12.3	7	16	12	10	1.82E-05	2.80E-05	1.47E-05	2.26E-05

F35E12.4	4	7	15	12	2.80E-06	2.65E-06	1.82E-06	2.25E-06
F35E12.5	770	4219	1288	1776	2.80E-06	2.65E-06	1.82E-06	2.25E-06
F35E12.7a	79	137	144	98	2.80E-06	2.65E-06	1.82E-06	2.25E-06
F35E12.7b	71	119	134	89	8.36E-05	4.33E-04	9.10E-05	1.55E-04
F35E12.7c	74	127	140	91	3.98E-06	6.53E-06	4.72E-06	3.96E-06
F35E12.7d	73	128	142	92	4.98E-06	7.88E-06	6.12E-06	5.02E-06
F35E12.8	120	107	71	93	5.01E-06	8.12E-06	6.16E-06	4.95E-06
F35E12.9a	43	91	23	41	4.59E-06	7.59E-06	5.79E-06	4.63E-06
F35E12.9b	46	94	39	52	8.43E-06	7.12E-06	3.24E-06	5.26E-06
F35E2.1	2	4	3	3	3.05E-06	6.14E-06	1.82E-06	2.34E-06
F35E2.10	26	39	14	17	3.78E-06	7.30E-06	2.08E-06	3.42E-06
F35E2.2	4	2	8	3	2.80E-06	2.65E-06	1.82E-06	2.25E-06
F35E2.3	6	5	4	3	2.80E-06	2.65E-06	1.82E-06	2.25E-06
F35E2.4	3	4	1	3	2.80E-06	2.65E-06	1.82E-06	2.25E-06
F35E2.5	30	34	29	17	2.80E-06	2.65E-06	1.82E-06	2.25E-06
F35E2.6	22	34	10	15	2.80E-06	2.65E-06	1.82E-06	2.25E-06
F35E2.7	8	9	8	4	2.80E-06	2.65E-06	1.82E-06	2.25E-06
F35E2.8	3	4	6	2	2.80E-06	2.65E-06	1.82E-06	2.25E-06
F35E2.9	44	102	34	41	2.80E-06	2.65E-06	1.82E-06	2.25E-06
F35E8.1	3	1	2	0	2.80E-06	2.65E-06	1.82E-06	2.25E-06
F35E8.10	5	1	3	1	2.80E-06	5.53E-06	1.82E-06	2.25E-06
F35E8.11	16	7	12	7	2.80E-06	2.65E-06	1.82E-06	2.25E-06
F35E8.12	12	19	16	34	2.80E-06	2.65E-06	1.82E-06	2.25E-06
F35E8.13	5	1	3	1	2.80E-06	2.65E-06	1.82E-06	2.25E-06
F35E8.2	3	1	3	0	2.80E-06	2.65E-06	1.82E-06	2.65E-06
F35E8.4	3	1	2	0	2.80E-06	2.65E-06	1.82E-06	2.25E-06
F35E8.6	3	3	1	3	2.80E-06	2.65E-06	1.82E-06	2.25E-06
F35E8.7	2	3	3	0	2.80E-06	2.65E-06	1.82E-06	2.25E-06
F35E8.8	49	20	75	11	2.80E-06	2.65E-06	1.82E-06	2.25E-06
F35E8.9	2	1	1	0	2.80E-06	2.65E-06	1.82E-06	2.25E-06
F35F10.1	23	25	41	10	8.71E-06	3.36E-06	8.67E-06	2.25E-06
F35F10.10	10	8	19	2	2.80E-06	2.65E-06	1.82E-06	2.25E-06
F35F10.11	68	59	61	64	2.80E-06	2.83E-06	3.19E-06	2.25E-06
F35F10.12.1	30	49	56	59	2.80E-06	2.65E-06	1.82E-06	2.25E-06
F35F10.12.2	28	42	53	56	5.10E-06	4.18E-06	2.97E-06	3.85E-06
F35F10.13	9	11	7	3	2.80E-06	3.07E-06	2.41E-06	3.15E-06
F35F10.14	1	0	0	1	2.80E-06	3.12E-06	2.71E-06	3.53E-06
F35F10.2	3	5	10	4	2.80E-06	2.65E-06	1.82E-06	2.25E-06
F35F10.4	13	15	13	9	2.80E-06	2.65E-06	1.82E-06	2.25E-06
F35F10.5	15	3	15	6	2.80E-06	2.65E-06	1.82E-06	2.25E-06
F35F10.6	14	12	8	6	2.80E-06	2.65E-06	1.82E-06	2.25E-06
F35F10.7	5	3	6	4	2.80E-06	2.65E-06	1.82E-06	2.25E-06
F35F10.8	3	5	5	2	2.80E-06	2.65E-06	1.82E-06	2.25E-06
F35F10.9	4	5	3	2	2.80E-06	2.65E-06	1.82E-06	2.25E-06
F35F11.1.1	279	222	421	509	2.80E-06	2.65E-06	1.82E-06	2.25E-06
F35F11.1.2	247	191	368	475	2.80E-06	2.65E-06	1.82E-06	2.25E-06
F35F11.2	10	12	5	3	1.64E-05	1.23E-05	1.61E-05	2.40E-05
F35F11.3	8	9	14	7	1.72E-05	1.25E-05	1.66E-05	2.65E-05
F35G12.12.1	394	523	402	650	2.80E-06	2.65E-06	1.82E-06	2.25E-06
F35G12.1a	208	287	333	404	2.80E-06	2.65E-06	1.82E-06	2.25E-06
F35G12.2.1	1016	1323	1025	1615	2.72E-05	3.41E-05	1.81E-05	3.60E-05
F35G12.2.2	1056	1356	1049	1619	1.58E-05	2.07E-05	1.65E-05	2.47E-05
F35G12.2.3	1004	1309	1015	1597	5.46E-05	6.71E-05	3.58E-05	6.97E-05
F35G12.2.4	830	1084	837	1348	5.68E-05	6.89E-05	3.67E-05	7.00E-05
F35G12.3a	747	773	923	1470	7.59E-05	9.35E-05	4.99E-05	9.70E-05
F35G12.3b	526	535	706	1025	7.73E-05	9.54E-05	5.07E-05	1.01E-04
F35G12.4b	396	429	521	629	2.36E-05	2.31E-05	1.90E-05	3.73E-05
F35G12.5	77	94	104	151	2.49E-05	2.39E-05	2.17E-05	3.89E-05
F35G12.6	40	51	17	20	2.03E-05	2.08E-05	1.74E-05	2.59E-05

F35G12.7	72	44	94	64	8.43E-06	9.73E-06	7.42E-06	1.33E-05
F35G12.8	1471	1469	1337	2726	3.56E-06	4.29E-06	1.82E-06	2.25E-06
F35G12.9	138	170	278	178	1.76E-05	1.02E-05	1.50E-05	1.26E-05
F35G2.1a.1	1201	1230	2015	2664	3.33E-05	3.15E-05	1.97E-05	4.97E-05
F35G2.1a.2	803	839	1358	1763	2.77E-05	3.22E-05	3.63E-05	2.87E-05
F35G2.2.1	188	302	206	167	4.98E-05	4.82E-05	5.44E-05	8.88E-05
F35G2.2.2	155	263	158	154	4.98E-05	4.91E-05	5.48E-05	8.78E-05
F35G2.3	16	6	10	7	2.98E-05	4.53E-05	2.13E-05	2.13E-05
F35G2.4.1	208	416	145	241	2.88E-05	4.62E-05	1.91E-05	2.30E-05
F35G2.4.2	169	354	110	201	3.44E-06	2.65E-06	1.82E-06	2.25E-06
F35G8.1	10	20	5	3	1.36E-05	2.56E-05	6.16E-06	1.26E-05
F35G8.2	5	22	13	8	1.17E-05	2.31E-05	4.94E-06	1.12E-05
F35H10.1	12	10	32	6	2.80E-06	2.65E-06	1.82E-06	2.25E-06
F35H10.10	921	1044	591	836	2.80E-06	5.48E-06	2.22E-06	2.25E-06
F35H10.11	9	10	12	3	3.50E-06	2.75E-06	6.07E-06	2.25E-06
F35H10.12	12	10	9	7	2.26E-05	2.41E-05	9.42E-06	1.64E-05
F35H10.2	14	34	11	7	2.80E-06	2.86E-06	2.37E-06	2.25E-06
F35H10.3	7	15	16	10	1.37E-05	1.08E-05	6.69E-06	6.43E-06
F35H10.4.1	582	1333	1073	2129	3.08E-06	7.09E-06	1.82E-06	2.25E-06
F35H10.4.2	516	1208	930	1975	2.80E-06	2.75E-06	2.02E-06	2.25E-06
F35H10.4.3	547	1297	1001	2071	2.20E-05	4.76E-05	2.64E-05	6.46E-05
F35H10.5	38	40	230	55	2.18E-05	4.83E-05	2.56E-05	6.72E-05
F35H10.6	134	185	176	127	2.23E-05	5.00E-05	2.66E-05	6.79E-05
F35H12.1	7	10	15	11	1.77E-05	1.76E-05	6.98E-05	2.06E-05
F35H12.2a	69	116	62	118	2.60E-05	3.39E-05	2.22E-05	1.98E-05
F35H12.2b	72	120	68	120	2.80E-06	2.65E-06	1.82E-06	2.25E-06
F35H12.3	71	101	101	140	2.80E-06	4.39E-06	1.82E-06	3.80E-06
F35H12.4	63	95	71	118	2.80E-06	4.39E-06	1.82E-06	3.73E-06
F35H12.5a	28	41	22	46	5.43E-06	7.30E-06	5.03E-06	8.61E-06
F35H12.5b.1	31	44	25	47	3.44E-06	4.89E-06	2.51E-06	5.17E-06
F35H12.5b.2	26	40	22	44	2.91E-06	4.05E-06	1.82E-06	3.85E-06
F35H12.6	5	10	11	7	2.80E-06	3.70E-06	1.82E-06	3.35E-06
F35H12.7	2	1	1	3	2.80E-06	3.97E-06	1.82E-06	3.71E-06
F35H8.1	9	22	9	5	2.80E-06	2.65E-06	1.82E-06	2.25E-06
F35H8.2	19	66	20	20	2.80E-06	2.65E-06	1.82E-06	2.25E-06
F35H8.3	374	323	603	660	2.80E-06	5.13E-06	1.82E-06	2.25E-06
F35H8.4	8	21	8	2	2.80E-06	5.74E-06	1.82E-06	2.25E-06
F35H8.5	106	128	66	121	2.76E-05	2.25E-05	2.89E-05	3.90E-05
F35H8.6	218	436	299	405	2.94E-06	7.27E-06	1.91E-06	2.25E-06
F35H8.7	11	8	25	12	6.94E-06	7.94E-06	2.82E-06	6.36E-06
F36A2.10	34	84	34	28	1.42E-05	2.69E-05	1.27E-05	2.12E-05
F36A2.11	18	29	8	8	2.80E-06	2.65E-06	1.82E-06	2.25E-06
F36A2.12	28	49	16	3	7.06E-06	1.65E-05	4.59E-06	4.68E-06
F36A2.13	2698	2361	3034	5275	2.80E-06	4.18E-06	1.82E-06	2.25E-06
F36A2.14	37	92	26	23	8.76E-06	1.45E-05	3.26E-06	2.25E-06
F36A2.1a.1	645	725	810	1185	3.26E-05	2.70E-05	2.39E-05	5.12E-05
F36A2.1a.2	576	638	769	1127	4.37E-06	1.02E-05	1.99E-06	2.25E-06
F36A2.1b	566	632	759	1117	3.02E-05	3.21E-05	2.47E-05	4.46E-05
F36A2.2	183	228	237	300	3.15E-05	3.29E-05	2.73E-05	4.95E-05
F36A2.3	161	291	154	200	3.12E-05	3.29E-05	2.72E-05	4.94E-05
F36A2.4	32	27	21	21	1.29E-05	1.52E-05	1.09E-05	1.70E-05
F36A2.6.1	6122	7194	9804	4476	1.52E-05	2.60E-05	9.49E-06	1.52E-05
F36A2.6.2	5477	6402	8268	4170	2.80E-06	2.65E-06	1.82E-06	2.25E-06
F36A2.7.1	2356	2187	3701	2130	1.34E-03	1.49E-03	1.40E-03	7.90E-04
F36A2.7.2	1939	1842	3072	1961	1.20E-03	1.33E-03	1.18E-03	7.36E-04
F36A2.8	98	117	248	89	4.35E-04	3.81E-04	4.44E-04	3.16E-04
F36A2.9a	494	613	698	932	3.61E-04	3.24E-04	3.73E-04	2.94E-04
F36A4.1	3	7	2	0	2.57E-05	2.90E-05	4.23E-05	1.88E-05
F36A4.10	129	209	111	101	2.84E-05	3.33E-05	2.61E-05	4.30E-05

F36A4.11	1	5	16	3	2.80E-06	2.65E-06	1.82E-06	2.25E-06
F36A4.14	17	26	28	30	1.50E-05	2.30E-05	8.42E-06	9.45E-06
F36A4.2	11	18	8	6	2.80E-06	2.65E-06	3.57E-06	2.25E-06
F36A4.3	6	10	1	3	2.80E-06	2.65E-06	1.82E-06	2.32E-06
F36A4.4	22	49	17	13	2.91E-06	4.50E-06	1.82E-06	2.25E-06
F36A4.5	1	4	3	3	2.80E-06	2.99E-06	1.82E-06	2.25E-06
F36A4.6	44	44	21	25	5.96E-06	1.25E-05	2.99E-06	2.83E-06
F36A4.7	1562	1474	1649	3020	2.80E-06	2.65E-06	1.82E-06	2.25E-06
F36A4.9	2	3	5	1	5.38E-06	5.08E-06	1.82E-06	2.45E-06
F36D1.1	35	30	55	46	2.95E-05	2.63E-05	2.03E-05	4.58E-05
F36D1.10	0	0	1	1	2.80E-06	2.65E-06	1.82E-06	2.25E-06
F36D1.2	5	4	6	4	3.95E-06	3.20E-06	4.05E-06	4.16E-06
F36D1.3	5	4	5	5	2.80E-06	2.65E-06	1.82E-06	2.25E-06
F36D1.4	8	9	8	1	2.80E-06	2.65E-06	1.82E-06	2.25E-06
F36D1.5	5	4	0	3	2.80E-06	2.65E-06	1.82E-06	2.25E-06
F36D1.6	6	9	3	3	4.59E-06	4.89E-06	2.99E-06	2.25E-06
F36D1.7	9	11	6	4	2.80E-06	2.65E-06	1.82E-06	2.25E-06
F36D1.8	9	7	14	9	2.80E-06	2.65E-06	1.82E-06	2.25E-06
F36D1.9	11	8	9	5	2.80E-06	2.96E-06	1.82E-06	2.25E-06
F36D3.10	0	1	1	1	2.80E-06	2.65E-06	1.82E-06	2.25E-06
F36D3.11	0	2	0	1	3.28E-06	2.65E-06	1.82E-06	2.25E-06
F36D3.13	5	0	6	3	2.80E-06	2.65E-06	1.82E-06	2.25E-06
F36D3.14	0	2	0	1	2.80E-06	2.65E-06	1.82E-06	2.25E-06
F36D3.2	7	7	14	6	2.80E-06	2.65E-06	1.82E-06	2.25E-06
F36D3.3	8	9	15	2	2.80E-06	2.65E-06	1.82E-06	2.25E-06
F36D3.4	50	85	24	22	2.80E-06	2.65E-06	1.82E-06	2.25E-06
F36D3.5	36	74	34	28	2.80E-06	2.65E-06	1.82E-06	2.25E-06
F36D3.6	4	2	11	3	5.54E-06	8.91E-06	1.82E-06	2.25E-06
F36D3.7	5	6	12	6	2.80E-06	2.65E-06	1.82E-06	2.25E-06
F36D3.8	20	46	19	25	2.80E-06	2.65E-06	1.82E-06	2.25E-06
F36D3.9	4	5	3	4	2.80E-06	2.65E-06	1.82E-06	2.25E-06
F36D4.1	9	9	8	2	2.80E-06	2.65E-06	1.82E-06	2.25E-06
F36D4.2	110	137	85	132	2.80E-06	2.65E-06	1.82E-06	2.25E-06
F36D4.3a	1131	1249	1866	2172	2.80E-06	2.65E-06	1.82E-06	2.25E-06
F36D4.3b	1127	1249	1865	2177	1.83E-05	2.15E-05	9.18E-06	1.76E-05
F36D4.3c.1	472	516	622	724	2.16E-05	2.25E-05	2.32E-05	3.33E-05
F36D4.3c.2	468	514	617	722	2.15E-05	2.25E-05	2.31E-05	3.34E-05
F36D4.3e	468	514	617	722	3.29E-05	3.40E-05	2.82E-05	4.06E-05
F36D4.3f	431	468	544	660	3.32E-05	3.44E-05	2.85E-05	4.11E-05
F36D4.4	12	10	16	9	3.32E-05	3.44E-05	2.85E-05	4.11E-05
F36D4.5a	621	538	827	1162	3.92E-05	4.02E-05	3.22E-05	4.82E-05
F36D4.5b.1	930	882	1290	1798	2.80E-06	2.65E-06	1.82E-06	2.25E-06
F36D4.5b.2	959	906	1326	1819	6.92E-05	5.66E-05	6.00E-05	1.04E-04
F36D4.5b.3	936	884	1298	1807	6.52E-05	5.84E-05	5.89E-05	1.01E-04
F36D4.5b.4	620	538	827	1161	6.92E-05	6.18E-05	6.23E-05	1.06E-04
F36D4.6	1	1	1	2	6.26E-05	5.58E-05	5.64E-05	9.70E-05
F36D4.7	1	0	0	0	6.94E-05	5.69E-05	6.03E-05	1.04E-04
F36D4.t2	0	1	1	1	2.80E-06	2.65E-06	1.82E-06	2.25E-06
F36F12.1	12	3	12	7	2.80E-06	2.65E-06	1.82E-06	2.25E-06
F36F12.2	5	5	2	6	2.80E-06	2.65E-06	1.82E-06	2.25E-06
F36F12.3	0	1	4	4	2.80E-06	2.65E-06	1.82E-06	2.25E-06
F36F12.4	20	21	31	31	2.80E-06	2.65E-06	1.82E-06	2.25E-06
F36F12.5	0	1	7	1	2.80E-06	2.65E-06	1.82E-06	2.25E-06
F36F12.6	1	2	2	1	3.39E-06	3.36E-06	3.43E-06	4.23E-06
F36F12.7	5	5	11	1	2.80E-06	2.65E-06	1.82E-06	2.25E-06
F36F12.8	72	36	124	80	2.80E-06	2.65E-06	1.82E-06	2.25E-06
F36F2.1	43	51	22	25	2.80E-06	2.65E-06	2.17E-06	2.25E-06
F36F2.2	27	54	40	37	9.86E-06	4.66E-06	1.10E-05	8.79E-06
F36F2.3a	1020	1060	626	1170	7.90E-06	8.83E-06	2.62E-06	3.69E-06

F36F2.3b	302	348	171	296	3.42E-06	6.43E-06	3.28E-06	3.76E-06
F36F2.5	16	21	9	8	3.35E-05	3.29E-05	1.34E-05	3.08E-05
F36F2.6	357	347	520	786	3.02E-05	3.29E-05	1.11E-05	2.38E-05
F36F2.7	5	4	3	4	2.80E-06	2.65E-06	1.82E-06	2.25E-06
F36F2.8	0	2	4	1	2.02E-05	1.85E-05	1.92E-05	3.57E-05
F36G3.1	168	254	113	188	2.80E-06	2.65E-06	1.82E-06	2.25E-06
F36G3.2	91	112	41	64	2.80E-06	2.65E-06	1.82E-06	2.25E-06
F36G3.3	3	5	2	1	5.80E-06	8.25E-06	2.53E-06	5.20E-06
F36G9.1	15	25	25	28	8.12E-06	9.42E-06	2.37E-06	4.59E-06
F36G9.10	1	1	0	1	2.80E-06	2.65E-06	1.82E-06	2.25E-06
F36G9.11	3	11	6	5	2.80E-06	2.65E-06	1.82E-06	2.36E-06
F36G9.12	75	35	44	28	2.80E-06	2.65E-06	1.82E-06	2.25E-06
F36G9.13	32	28	19	19	2.80E-06	2.65E-06	1.82E-06	2.25E-06
F36G9.14	16	16	14	17	3.86E-06	2.65E-06	1.82E-06	2.25E-06
F36G9.15	0	2	2	2	2.80E-06	2.65E-06	1.82E-06	2.25E-06
F36G9.16	15	4	20	6	2.80E-06	2.65E-06	1.82E-06	2.25E-06
F36G9.2	4	6	7	9	2.80E-06	2.65E-06	1.82E-06	2.25E-06
F36G9.3	21	10	11	8	2.80E-06	2.65E-06	1.82E-06	2.25E-06
F36G9.4	16	21	37	30	2.80E-06	2.65E-06	1.82E-06	2.25E-06
F36G9.5	3	4	7	1	2.80E-06	2.65E-06	1.82E-06	2.25E-06
F36G9.6	3	2	6	1	2.80E-06	2.65E-06	1.82E-06	2.25E-06
F36G9.7	7	12	9	3	2.80E-06	2.65E-06	1.82E-06	2.25E-06
F36G9.8	8	8	3	5	2.80E-06	2.65E-06	1.82E-06	2.25E-06
F36G9.9	6	2	4	2	2.80E-06	2.65E-06	1.82E-06	2.25E-06
F36H1.10	18	14	19	10	2.80E-06	2.65E-06	1.82E-06	2.25E-06
F36H1.11	8	16	11	3	2.80E-06	2.65E-06	1.82E-06	2.25E-06
F36H1.12	2	3	1	3	4.14E-06	3.04E-06	2.84E-06	2.25E-06
F36H1.2a	643	728	995	1311	3.56E-06	6.72E-06	3.19E-06	2.25E-06
F36H1.2b	645	731	1002	1319	2.80E-06	2.65E-06	1.82E-06	2.25E-06
F36H1.3	47	72	27	15	1.58E-05	1.68E-05	1.59E-05	2.58E-05
F36H1.4a	351	399	368	572	1.74E-05	1.87E-05	1.76E-05	2.87E-05
F36H1.4b.1	226	216	242	403	2.94E-06	4.26E-06	1.82E-06	2.25E-06
F36H1.4b.2	237	230	246	409	2.06E-05	2.21E-05	1.40E-05	2.69E-05
F36H1.4c	272	258	308	491	1.89E-05	1.71E-05	1.32E-05	2.71E-05
F36H1.5	33	41	19	16	1.89E-05	1.73E-05	1.28E-05	2.62E-05
F36H1.6	596	883	666	892	1.88E-05	1.68E-05	1.38E-05	2.73E-05
F36H1.9	2	2	4	1	5.94E-06	6.96E-06	2.22E-06	2.32E-06
F36H12.1	39	87	92	60	2.39E-05	3.35E-05	1.74E-05	2.87E-05
F36H12.10	46	76	36	17	2.80E-06	2.65E-06	1.82E-06	2.25E-06
F36H12.11	22	66	28	11	6.89E-06	1.45E-05	1.06E-05	8.52E-06
F36H12.13	1	4	1	1	4.31E-06	6.72E-06	2.19E-06	2.25E-06
F36H12.14	24	33	14	11	3.81E-06	1.08E-05	3.15E-06	2.25E-06
F36H12.15	0	1	2	3	2.80E-06	2.72E-06	1.82E-06	2.25E-06
F36H12.16	4	4	7	10	3.28E-06	4.26E-06	1.82E-06	2.25E-06
F36H12.17	9	7	11	13	2.80E-06	2.65E-06	1.82E-06	2.25E-06
F36H12.19	0	2	0	7	2.80E-06	2.65E-06	1.82E-06	2.43E-06
F36H12.2	58	95	60	76	2.80E-06	2.65E-06	1.82E-06	2.25E-06
F36H12.20	0	0	1	2	2.80E-06	2.65E-06	1.82E-06	5.74E-06
F36H12.3	89	122	47	48	6.13E-06	9.50E-06	4.14E-06	6.45E-06
F36H12.4	23	32	20	3	2.80E-06	2.65E-06	1.82E-06	2.25E-06
F36H12.5	46	49	28	17	9.88E-06	1.28E-05	3.41E-06	4.27E-06
F36H12.6	1540	2006	1717	533	1.00E-05	1.32E-05	5.67E-06	2.25E-06
F36H12.7	2387	3276	4097	993	8.68E-06	8.73E-06	3.44E-06	2.56E-06
F36H12.8	46	87	66	37	7.37E-04	9.07E-04	5.35E-04	2.05E-04
F36H12.9	44	77	27	15	5.89E-04	7.63E-04	6.58E-04	1.97E-04
F36H2.1a	1238	1150	1439	2246	5.01E-06	8.94E-06	4.68E-06	3.24E-06
F36H2.1b	1500	1350	1804	2747	4.31E-06	7.12E-06	1.82E-06	2.25E-06
F36H2.1c	1237	1151	1437	2247	4.21E-05	3.69E-05	3.18E-05	6.13E-05
F36H2.2	300	399	406	521	4.53E-05	3.85E-05	3.55E-05	6.66E-05

F36H2.3	74	128	75	79	4.21E-05	3.70E-05	3.19E-05	6.15E-05
F36H2.4	2	4	5	3	1.97E-05	2.47E-05	1.73E-05	2.74E-05
F36H2.5	16	36	12	16	2.80E-06	3.17E-06	1.82E-06	2.25E-06
F36H5.1.1	88	208	49	137	2.80E-06	2.65E-06	1.82E-06	2.25E-06
F36H5.1.2	84	204	47	133	2.80E-06	2.72E-06	1.82E-06	2.25E-06
F36H5.11	4	4	4	4	3.84E-06	8.57E-06	1.82E-06	4.79E-06
F36H5.2a.1	15	48	18	20	3.81E-06	8.73E-06	1.82E-06	4.84E-06
F36H5.2a.2	18	52	18	18	2.80E-06	2.65E-06	1.82E-06	2.25E-06
F36H5.2c.1	49	127	20	82	2.80E-06	7.56E-06	1.95E-06	2.68E-06
F36H5.2c.2	49	131	20	86	3.28E-06	8.97E-06	2.13E-06	2.63E-06
F36H5.3a	9	12	6	8	3.47E-06	8.52E-06	1.82E-06	4.68E-06
F36H5.3b	9	12	6	8	3.28E-06	8.28E-06	1.82E-06	4.63E-06
F36H5.4	3	8	14	10	2.80E-06	2.65E-06	1.82E-06	2.25E-06
F36H5.5	6	14	12	5	2.80E-06	2.65E-06	1.82E-06	2.25E-06
F36H5.6	3	1	1	2	2.80E-06	2.65E-06	1.82E-06	2.25E-06
F36H5.7	1	4	0	4	2.80E-06	2.65E-06	1.82E-06	2.25E-06
F36H5.8	6	8	6	5	2.80E-06	2.65E-06	1.82E-06	2.25E-06
F36H5.9	0	1	4	1	2.80E-06	2.65E-06	1.82E-06	2.25E-06
F36H9.1	4	3	4	3	2.80E-06	2.65E-06	1.82E-06	2.25E-06
F36H9.2	4	6	1	2	2.80E-06	2.65E-06	1.82E-06	2.25E-06
F36H9.3	219	145	186	331	2.80E-06	2.65E-06	1.82E-06	2.25E-06
F36H9.4	43	55	59	30	2.80E-06	2.65E-06	1.82E-06	2.25E-06
F36H9.5	24	36	11	9	3.17E-05	1.98E-05	1.75E-05	3.85E-05
F36H9.6	3	3	1	1	7.20E-06	8.70E-06	6.43E-06	4.03E-06
F36H9.7	2	2	11	1	2.80E-06	3.60E-06	1.82E-06	2.25E-06
F36H9.8	6	9	7	3	2.80E-06	2.65E-06	1.82E-06	2.25E-06
F37A4.1.1	549	735	743	1004	2.80E-06	2.65E-06	1.82E-06	2.25E-06
F37A4.1.2	748	960	958	1188	2.80E-06	2.65E-06	1.82E-06	2.25E-06
F37A4.1.3	506	670	695	949	3.70E-05	4.68E-05	3.26E-05	5.44E-05
F37A4.3	23	24	100	29	5.72E-05	6.94E-05	4.77E-05	7.30E-05
F37A4.4	76	137	55	69	3.88E-05	4.85E-05	3.47E-05	5.84E-05
F37A4.5	35	54	23	19	3.72E-06	3.68E-06	1.06E-05	3.78E-06
F37A4.6	9	10	7	6	2.80E-06	4.02E-06	1.82E-06	2.25E-06
F37A4.7a.1	34	47	31	42	4.09E-06	5.95E-06	1.82E-06	2.25E-06
F37A4.7a.2	34	46	31	41	2.80E-06	2.65E-06	1.82E-06	2.25E-06
F37A4.7b	42	54	36	42	2.80E-06	2.65E-06	1.82E-06	2.25E-06
F37A4.7c	34	46	31	41	2.80E-06	2.65E-06	1.82E-06	2.25E-06
F37A4.7d	57	78	58	78	2.80E-06	2.65E-06	1.82E-06	2.25E-06
F37A4.8	1210	1248	2041	2523	2.80E-06	2.65E-06	1.82E-06	2.25E-06
F37A4.9	668	753	869	1229	2.80E-06	2.65E-06	1.82E-06	2.25E-06
F37A8.1	11	15	7	3	4.12E-05	4.02E-05	4.52E-05	6.90E-05
F37A8.2	7	26	21	10	5.01E-05	5.34E-05	4.25E-05	7.41E-05
F37A8.4	24	56	41	30	2.80E-06	2.96E-06	1.82E-06	2.25E-06
F37A8.5	11	19	19	6	2.80E-06	2.65E-06	1.82E-06	2.25E-06
F37B1.1	17	45	30	17	4.54E-06	9.97E-06	5.03E-06	4.54E-06
F37B1.2	22	8	12	10	2.97E-06	4.87E-06	3.35E-06	2.25E-06
F37B1.3	8	5	5	2	3.02E-06	7.56E-06	3.46E-06	2.43E-06
F37B1.4	9	13	18	7	3.92E-06	2.65E-06	1.82E-06	2.25E-06
F37B1.5	11	22	16	24	2.80E-06	2.65E-06	1.82E-06	2.25E-06
F37B1.6	8	4	4	0	2.80E-06	2.65E-06	2.04E-06	2.25E-06
F37B1.7	0	3	5	0	2.80E-06	3.70E-06	1.86E-06	3.44E-06
F37B1.8	2	5	1	2	2.80E-06	2.65E-06	1.82E-06	2.25E-06
F37B12.1	13	53	10	19	2.80E-06	2.65E-06	1.82E-06	2.25E-06
F37B12.2.1	599	747	839	992	2.80E-06	2.65E-06	1.82E-06	2.25E-06
F37B12.2.2	510	613	718	906	2.80E-06	5.92E-06	1.82E-06	2.25E-06
F37B12.3	272	391	383	348	2.89E-05	3.40E-05	2.63E-05	3.84E-05
F37B4.1	5	7	5	8	2.91E-05	3.30E-05	2.66E-05	4.14E-05
F37B4.10	31	36	43	34	2.30E-05	3.12E-05	2.10E-05	2.36E-05
F37B4.11	1	6	5	1	2.80E-06	2.65E-06	1.82E-06	2.25E-06

F37B4.12	2	5	4	2	3.53E-06	3.86E-06	3.17E-06	3.10E-06
F37B4.13	2	6	8	1	2.80E-06	2.65E-06	1.82E-06	2.25E-06
F37B4.14	0	0	2	1	2.80E-06	2.65E-06	1.82E-06	2.25E-06
F37B4.2.1	100	138	92	120	2.80E-06	2.65E-06	1.82E-06	2.25E-06
F37B4.2.2	94	131	89	117	2.80E-06	2.65E-06	1.82E-06	2.25E-06
F37B4.3	7	2	5	4	6.64E-06	8.65E-06	3.97E-06	6.39E-06
F37B4.4	7	8	10	3	7.00E-06	9.20E-06	4.32E-06	6.99E-06
F37B4.5	2	5	7	3	2.80E-06	2.65E-06	1.82E-06	2.25E-06
F37B4.6	6	3	4	1	2.80E-06	2.65E-06	1.82E-06	2.25E-06
F37B4.8	5	7	6	1	2.80E-06	2.65E-06	1.82E-06	2.25E-06
F37B4.9	5	9	6	2	2.80E-06	2.65E-06	1.82E-06	2.25E-06
F37C12.1	205	252	218	306	2.80E-06	2.65E-06	1.82E-06	2.25E-06
F37C12.10	10	32	12	9	2.80E-06	2.65E-06	1.82E-06	2.25E-06
F37C12.11.1	3951	3260	19279	3039	2.32E-05	2.70E-05	1.61E-05	2.79E-05
F37C12.11.2	2890	2423	13338	2457	2.80E-06	5.61E-06	1.82E-06	2.25E-06
F37C12.12	6	9	14	10	1.26E-03	9.80E-04	3.99E-03	7.77E-04
F37C12.13a.1	229	264	278	393	9.25E-04	7.32E-04	2.78E-03	6.32E-04
F37C12.13a.2	224	258	242	372	2.80E-06	2.65E-06	1.82E-06	2.25E-06
F37C12.13b	97	117	110	145	1.81E-05	1.97E-05	1.43E-05	2.50E-05
F37C12.14	3	4	8	2	1.92E-05	2.08E-05	1.35E-05	2.55E-05
F37C12.15	7	12	21	6	1.83E-05	2.08E-05	1.35E-05	2.19E-05
F37C12.16	7	12	21	6	2.80E-06	2.65E-06	1.82E-06	2.25E-06
F37C12.17	9	9	8	6	2.80E-06	2.65E-06	1.82E-06	2.25E-06
F37C12.18	24	35	14	12	2.80E-06	2.65E-06	1.82E-06	2.25E-06
F37C12.2	395	432	654	768	2.80E-06	2.65E-06	1.82E-06	2.25E-06
F37C12.3.1	511	685	624	477	3.86E-06	5.32E-06	1.82E-06	2.25E-06
F37C12.3.2	452	642	486	425	3.19E-05	3.29E-05	3.43E-05	4.98E-05
F37C12.4.1	5863	5225	21193	4426	9.55E-05	1.21E-04	7.59E-05	7.16E-05
F37C12.4.2	5245	4651	15644	3807	8.77E-05	1.18E-04	6.14E-05	6.63E-05
F37C12.7	3348	3186	4429	6607	1.71E-03	1.44E-03	4.03E-03	1.04E-03
F37C12.9.1	6738	7727	15194	5386	1.65E-03	1.38E-03	3.19E-03	9.59E-04
F37C12.9.2	5728	6583	11658	4762	1.33E-04	1.19E-04	1.14E-04	2.10E-04
F37C4.1	6	5	7	1	1.44E-03	1.56E-03	2.11E-03	9.23E-04
F37C4.2	7	8	12	9	1.20E-03	1.31E-03	1.59E-03	8.04E-04
F37C4.3	13	16	15	7	2.80E-06	2.65E-06	1.82E-06	2.25E-06
F37C4.4a	38	52	23	32	2.80E-06	2.65E-06	1.82E-06	2.25E-06
F37C4.4b.1	30	43	18	19	2.80E-06	2.65E-06	1.82E-06	2.25E-06
F37C4.4b.2	39	54	24	24	4.48E-06	5.79E-06	1.82E-06	3.04E-06
F37C4.5a.1	557	789	978	1212	4.62E-06	6.27E-06	1.82E-06	2.36E-06
F37C4.5a.2	580	814	1023	1223	3.84E-06	5.00E-06	1.82E-06	2.25E-06
F37C4.5b	316	431	591	759	3.51E-05	4.69E-05	4.01E-05	6.13E-05
F37C4.6.1	106	153	168	151	3.88E-05	5.14E-05	4.45E-05	6.56E-05
F37C4.6.2	99	148	156	141	3.72E-05	4.80E-05	4.53E-05	7.18E-05
F37C4.7	3	4	1	2	6.78E-06	9.23E-06	6.98E-06	7.76E-06
F37C4.8	5	13	4	4	6.78E-06	9.58E-06	6.94E-06	7.76E-06
F37D6.1	471	472	654	904	2.80E-06	2.65E-06	1.82E-06	2.25E-06
F37D6.2a.1	321	372	515	703	2.80E-06	2.65E-06	1.82E-06	2.25E-06
F37D6.2a.2	301	349	483	666	1.39E-05	1.32E-05	1.26E-05	2.15E-05
F37D6.2b	314	371	516	699	1.86E-05	2.03E-05	1.94E-05	3.27E-05
F37D6.3	10	11	12	3	1.90E-05	2.08E-05	1.99E-05	3.38E-05
F37D6.4	0	1	3	0	1.98E-05	2.21E-05	2.11E-05	3.53E-05
F37D6.6	18	25	16	28	2.80E-06	2.65E-06	1.82E-06	2.25E-06
F37E3.1	800	772	1098	1665	2.80E-06	2.65E-06	1.82E-06	2.25E-06
F37E3.2	3	11	12	5	2.80E-06	2.65E-06	1.82E-06	2.25E-06
F37E3.3	33	63	26	21	3.29E-05	3.00E-05	2.94E-05	5.50E-05
F37F2.2	107	119	133	100	2.80E-06	2.65E-06	1.82E-06	2.25E-06
F37F2.3	5	4	1	0	2.94E-06	5.29E-06	1.82E-06	2.25E-06
F37H8.1	14	15	18	19	2.48E-05	2.61E-05	2.01E-05	1.86E-05
F37H8.2	13	22	17	26	2.80E-06	2.65E-06	1.82E-06	2.25E-06

F37H8.3	28	34	20	21	2.80E-06	2.65E-06	1.82E-06	2.25E-06
F37H8.4	12	37	9	8	2.80E-06	2.65E-06	1.82E-06	2.25E-06
F37H8.5	527	735	1040	647	3.95E-06	4.55E-06	1.84E-06	2.38E-06
F38A1.1	40	65	29	34	2.80E-06	3.97E-06	1.82E-06	2.25E-06
F38A1.10	4	9	6	4	6.43E-05	8.47E-05	8.26E-05	6.34E-05
F38A1.11	2	5	2	0	3.81E-06	5.82E-06	1.82E-06	2.59E-06
F38A1.13	0	2	1	1	2.80E-06	2.65E-06	1.82E-06	2.25E-06
F38A1.14	23	24	26	21	2.80E-06	2.65E-06	1.82E-06	2.25E-06
F38A1.4	13	6	15	5	2.80E-06	2.65E-06	1.82E-06	2.25E-06
F38A1.5	46	48	55	28	2.80E-06	2.65E-06	1.82E-06	2.25E-06
F38A1.6	0	0	3	1	2.80E-06	2.65E-06	1.82E-06	2.25E-06
F38A1.7	6	5	4	1	2.97E-06	2.94E-06	2.31E-06	2.25E-06
F38A1.8	101	117	180	313	2.80E-06	2.65E-06	1.82E-06	2.25E-06
F38A1.9	3	4	4	4	2.80E-06	2.65E-06	1.82E-06	2.25E-06
F38A3.1	2863	2478	2678	2676	8.12E-06	8.89E-06	9.42E-06	2.02E-05
F38A3.2	1245	1517	597	499	2.80E-06	2.65E-06	1.82E-06	2.25E-06
F38A5.10	28	20	9	4	3.09E-04	2.52E-04	1.88E-04	2.32E-04
F38A5.11	10	20	10	7	1.34E-04	1.54E-04	4.17E-05	4.30E-05
F38A5.12	35	44	36	5	1.26E-05	8.49E-06	2.64E-06	2.25E-06
F38A5.13	1218	1281	1058	1777	2.80E-06	2.65E-06	1.82E-06	2.25E-06
F38A5.14	45	49	30	7	1.57E-05	1.87E-05	1.05E-05	2.25E-06
F38A5.1a	495	720	707	902	6.68E-05	6.64E-05	3.78E-05	7.83E-05
F38A5.1b	444	644	643	839	2.02E-05	2.08E-05	8.78E-06	2.52E-06
F38A5.2a	705	713	748	942	2.80E-05	3.85E-05	2.60E-05	4.10E-05
F38A5.2b.1	560	560	615	706	2.88E-05	3.94E-05	2.71E-05	4.37E-05
F38A5.3a	56	93	65	74	4.35E-05	4.15E-05	3.00E-05	4.67E-05
F38A5.5	36	43	33	4	4.12E-05	3.89E-05	2.94E-05	4.17E-05
F38A5.6	13	33	11	3	7.73E-06	1.21E-05	5.83E-06	8.21E-06
F38A5.7	86	116	75	108	1.62E-05	1.83E-05	9.66E-06	2.25E-06
F38A5.8	2	2	2	0	2.80E-06	4.42E-06	1.82E-06	2.25E-06
F38A5.9	34	44	27	6	1.50E-05	1.90E-05	8.49E-06	1.51E-05
F38A6.1a	95	115	66	99	2.80E-06	2.65E-06	1.82E-06	2.25E-06
F38A6.1b	61	77	38	75	1.55E-05	1.89E-05	8.00E-06	2.25E-06
F38A6.2	38	72	47	52	4.68E-06	5.37E-06	2.11E-06	3.91E-06
F38A6.3a	1055	1537	1077	1666	4.62E-06	5.50E-06	1.88E-06	4.57E-06
F38A6.3b	888	1288	901	1405	2.80E-06	2.75E-06	1.82E-06	2.25E-06
F38A6.3c	570	808	626	972	4.11E-05	5.66E-05	2.73E-05	5.22E-05
F38A6.3d	738	1001	714	1117	4.01E-05	5.50E-05	2.65E-05	5.10E-05
F38A6.4	24	29	20	6	4.05E-05	5.43E-05	2.90E-05	5.55E-05
F38B2.1a	499	746	608	884	3.96E-05	5.08E-05	2.49E-05	4.82E-05
F38B2.1b	471	703	583	857	4.65E-06	5.32E-06	2.53E-06	2.25E-06
F38B2.2	0	13	3	3	2.90E-05	4.10E-05	2.30E-05	4.13E-05
F38B2.3	7	12	13	14	2.88E-05	4.05E-05	2.32E-05	4.20E-05
F38B2.4.1	142	313	215	253	2.80E-06	2.65E-06	1.82E-06	2.25E-06
F38B2.4.2	103	231	158	209	2.80E-06	2.65E-06	1.82E-06	2.25E-06
F38B6.1	3	5	0	0	1.75E-05	3.64E-05	1.72E-05	2.50E-05
F38B6.2	9	6	7	4	1.81E-05	3.84E-05	1.81E-05	2.96E-05
F38B6.3	5	10	7	3	2.80E-06	2.65E-06	1.82E-06	2.25E-06
F38B6.4	138	229	103	168	2.80E-06	2.65E-06	1.82E-06	2.25E-06
F38B6.5a	22	54	28	26	2.80E-06	2.65E-06	1.82E-06	2.25E-06
F38B6.5b.1	8	23	11	14	5.21E-06	8.17E-06	2.53E-06	5.11E-06
F38B6.5b.2	9	26	12	19	2.80E-06	4.44E-06	1.82E-06	2.25E-06
F38B6.6	26	22	27	33	2.80E-06	4.10E-06	1.82E-06	2.25E-06
F38B6.7	18	14	12	7	2.80E-06	3.76E-06	1.82E-06	2.34E-06
F38B6.8	3	8	13	5	2.80E-06	2.65E-06	1.82E-06	2.25E-06
F38B7.1a	346	581	330	618	2.80E-06	2.65E-06	1.82E-06	2.25E-06
F38B7.1b	192	309	196	338	2.80E-06	2.65E-06	1.82E-06	2.25E-06
F38B7.3	43	89	46	73	1.55E-05	2.46E-05	9.64E-06	2.23E-05
F38B7.4	4	4	2	4	1.47E-05	2.23E-05	9.75E-06	2.08E-05

F38B7.5	332	289	512	579	3.47E-06	6.80E-06	2.42E-06	4.75E-06
F38B7.6	7	4	6	2	2.80E-06	2.65E-06	1.82E-06	2.25E-06
F38B7.7	4	1	10	1	1.43E-05	1.18E-05	1.44E-05	2.01E-05
F38B7.8	4	6	5	3	2.80E-06	2.65E-06	1.82E-06	2.25E-06
F38B7.9	6	5	5	5	2.80E-06	2.65E-06	1.82E-06	2.25E-06
F38C2.1	2	1	2	0	2.80E-06	2.65E-06	1.82E-06	2.25E-06
F38C2.2	10	18	26	14	2.80E-06	2.65E-06	1.82E-06	2.25E-06
F38C2.4	10	9	8	16	2.80E-06	2.65E-06	1.82E-06	2.25E-06
F38C2.5	8	10	14	7	2.80E-06	3.65E-06	3.64E-06	2.41E-06
F38C2.6	1	4	4	1	2.80E-06	2.65E-06	1.82E-06	2.25E-06
F38C2.7	3	6	8	2	2.80E-06	2.65E-06	1.82E-06	2.25E-06
F38C2.8	1	5	1	1	2.80E-06	2.65E-06	1.82E-06	2.25E-06
F38E1.10	2	1	1	2	2.80E-06	2.65E-06	1.82E-06	2.25E-06
F38E1.11	1	1	2	1	2.80E-06	2.65E-06	1.82E-06	2.25E-06
F38E1.12	3	5	10	3	2.80E-06	2.65E-06	1.82E-06	2.25E-06
F38E1.3	16	29	4	9	2.80E-06	2.65E-06	1.82E-06	2.25E-06
F38E1.5	15	13	15	11	2.80E-06	2.65E-06	1.82E-06	2.25E-06
F38E1.6	11	8	19	4	2.80E-06	3.12E-06	1.82E-06	2.25E-06
F38E1.7	826	666	1477	1678	2.80E-06	2.65E-06	1.82E-06	2.25E-06
F38E1.8	6	6	17	7	2.80E-06	2.65E-06	1.82E-06	2.25E-06
F38E1.9	307	313	380	379	6.02E-05	4.58E-05	7.00E-05	9.82E-05
F38E11.1	3	10	6	4	2.80E-06	2.65E-06	1.82E-06	2.25E-06
F38E11.10	1	1	2	0	4.34E-05	4.18E-05	3.50E-05	4.30E-05
F38E11.11	1	1	2	0	2.80E-06	3.20E-06	1.82E-06	2.25E-06
F38E11.12	13	7	9	10	2.80E-06	2.65E-06	1.82E-06	2.25E-06
F38E11.13	6	4	5	2	2.80E-06	2.65E-06	1.82E-06	2.25E-06
F38E11.2	2	7	4	5	2.80E-06	2.65E-06	1.82E-06	2.25E-06
F38E11.3	51	87	55	107	2.80E-06	2.65E-06	1.82E-06	2.25E-06
F38E11.4	15	15	16	19	2.80E-06	2.65E-06	1.82E-06	2.25E-06
F38E11.5	2234	1972	2278	2766	4.48E-06	7.22E-06	3.15E-06	7.56E-06
F38E11.6a	12	36	17	21	2.80E-06	2.65E-06	1.82E-06	2.25E-06
F38E11.6b	13	36	17	22	6.72E-05	5.60E-05	4.46E-05	6.68E-05
F38E11.7	15	16	13	25	2.80E-06	2.65E-06	1.82E-06	2.25E-06
F38E11.8	2	9	6	4	2.80E-06	2.65E-06	1.82E-06	2.25E-06
F38E11.9	21	35	33	23	2.80E-06	2.65E-06	1.82E-06	3.17E-06
F38E9.1	76	110	82	172	2.80E-06	2.65E-06	1.82E-06	2.25E-06
F38E9.2	32	48	23	56	2.80E-06	4.13E-06	2.68E-06	2.29E-06
F38E9.4	3	1	4	1	3.70E-06	5.05E-06	2.61E-06	6.72E-06
F38E9.5.1	300	292	264	371	2.80E-06	2.65E-06	1.82E-06	2.25E-06
F38E9.5.2	242	243	166	268	2.80E-06	2.65E-06	1.82E-06	2.25E-06
F38E9.6	15	13	17	11	2.15E-05	1.98E-05	1.23E-05	2.13E-05
F38E9.t1	0	0	0	1	1.97E-05	1.87E-05	8.82E-06	1.76E-05
F38G1.1.1	11	12	3	3	3.08E-06	2.65E-06	2.28E-06	2.25E-06
F38G1.1.2	12	12	6	3	2.80E-06	2.65E-06	1.82E-06	2.25E-06
F38G1.2	10	5	11	6	2.80E-06	2.65E-06	1.82E-06	2.25E-06
F38G1.3	11	18	19	13	2.80E-06	2.65E-06	1.82E-06	2.25E-06
F38G1.t1	0	0	1	0	2.80E-06	2.65E-06	1.82E-06	2.25E-06
F38H12.1	4	8	4	4	2.80E-06	3.15E-06	2.28E-06	2.25E-06
F38H12.2	2	4	9	3	2.80E-06	2.65E-06	1.82E-06	2.25E-06
F38H12.3	16	28	17	22	2.80E-06	2.65E-06	1.82E-06	2.25E-06
F38H12.5	6	2	5	3	2.80E-06	2.65E-06	1.82E-06	2.25E-06
F38H4.1	2	4	47	8	2.80E-06	2.65E-06	1.82E-06	2.25E-06
F38H4.10	115	145	200	144	2.80E-06	2.65E-06	1.82E-06	2.25E-06
F38H4.2	9	11	9	10	2.80E-06	2.65E-06	7.00E-06	2.25E-06
F38H4.3	29	29	21	24	1.93E-05	2.30E-05	2.18E-05	1.94E-05
F38H4.4	47	76	38	26	2.80E-06	2.65E-06	1.82E-06	2.25E-06
F38H4.5	7	15	1	11	2.80E-06	2.65E-06	1.82E-06	2.25E-06
F38H4.6	20	45	15	3	3.36E-06	5.11E-06	1.82E-06	2.25E-06
F38H4.7.1	213	229	235	425	2.80E-06	2.65E-06	1.82E-06	2.25E-06

F38H4.7.2	210	227	235	425	3.78E-06	8.01E-06	1.84E-06	2.25E-06
F38H4.9.1	1744	1777	2692	3182	1.22E-05	1.24E-05	8.76E-06	1.96E-05
F38H4.9.2	882	860	1144	1575	1.21E-05	1.23E-05	8.80E-06	1.96E-05
F39B1.1	175	238	171	314	1.13E-04	1.09E-04	1.14E-04	1.66E-04
F39B1.t2	0	0	3	0	1.03E-04	9.47E-05	8.68E-05	1.47E-04
F39B2.1	724	587	1352	1434	3.84E-06	4.95E-06	2.44E-06	5.53E-06
F39B2.10.1	1318	1456	980	1646	2.80E-06	2.65E-06	2.99E-06	2.25E-06
F39B2.10.2	985	1107	728	1265	4.78E-05	3.66E-05	5.80E-05	7.60E-05
F39B2.11	393	372	609	884	9.33E-05	9.74E-05	4.52E-05	9.36E-05
F39B2.2.1	471	541	512	519	8.10E-05	8.60E-05	3.90E-05	8.36E-05
F39B2.2.2	361	403	395	453	3.79E-05	3.39E-05	3.83E-05	6.86E-05
F39B2.3	99	108	122	141	9.03E-05	9.80E-05	6.39E-05	8.00E-05
F39B2.4a	615	624	927	1325	6.92E-05	7.30E-05	4.93E-05	6.98E-05
F39B2.4b	630	631	939	1337	1.09E-05	1.12E-05	8.75E-06	1.25E-05
F39B2.5.1	131	118	150	128	1.44E-05	1.39E-05	1.42E-05	2.50E-05
F39B2.5.2	109	101	106	103	1.46E-05	1.38E-05	1.41E-05	2.49E-05
F39B2.6.1	6999	5747	18066	4762	2.30E-05	1.96E-05	1.71E-05	1.81E-05
F39B2.6.2	6046	4916	14430	4357	1.94E-05	1.70E-05	1.23E-05	1.47E-05
F39B2.7	27	45	39	52	1.61E-03	1.25E-03	2.71E-03	8.81E-04
F39B2.8	37	45	28	33	1.42E-03	1.09E-03	2.20E-03	8.22E-04
F39B3.2	13	21	19	16	2.80E-06	3.57E-06	2.13E-06	3.51E-06
F39B3.3	1	1	1	0	2.80E-06	2.65E-06	1.82E-06	2.25E-06
F39C12.1	496	513	504	834	2.80E-06	2.65E-06	1.82E-06	2.25E-06
F39C12.2a	33	47	31	43	2.80E-06	2.65E-06	1.82E-06	2.25E-06
F39C12.2b	44	62	41	55	3.61E-06	3.52E-06	2.39E-06	4.86E-06
F39C12.2c	40	60	41	52	2.80E-06	2.65E-06	1.82E-06	2.25E-06
F39C12.2d	34	48	31	42	2.80E-06	2.65E-06	1.82E-06	2.25E-06
F39C12.2e	45	63	41	56	2.80E-06	2.65E-06	1.82E-06	2.25E-06
F39C12.3a.1	87	117	54	73	2.80E-06	2.65E-06	1.82E-06	2.25E-06
F39C12.3a.2	87	116	54	73	2.80E-06	2.65E-06	1.82E-06	2.25E-06
F39C12.3b	110	152	73	91	7.36E-06	9.36E-06	2.97E-06	4.97E-06
F39C12.4	5	6	4	1	7.36E-06	9.28E-06	2.99E-06	4.97E-06
F39D8.1a	34	52	24	24	6.08E-06	7.94E-06	2.62E-06	4.05E-06
F39D8.1b	28	53	22	17	2.80E-06	2.65E-06	1.82E-06	2.25E-06
F39D8.1c	31	50	24	21	2.80E-06	2.65E-06	1.82E-06	2.25E-06
F39D8.3	6	18	13	9	2.80E-06	2.65E-06	1.82E-06	2.25E-06
F39D8.4	10	27	11	14	2.80E-06	2.65E-06	1.82E-06	2.25E-06
F39E9.1	69	70	67	72	2.80E-06	2.65E-06	1.82E-06	2.25E-06
F39E9.11	23	33	21	19	2.80E-06	2.65E-06	1.82E-06	2.25E-06
F39E9.12	7	5	8	7	5.80E-06	5.55E-06	3.66E-06	4.86E-06
F39E9.14	2	0	7	7	2.80E-06	3.62E-06	1.82E-06	2.25E-06
F39E9.2	16	39	15	17	2.80E-06	2.65E-06	1.82E-06	2.25E-06
F39E9.3	2	2	2	6	2.80E-06	2.65E-06	3.21E-06	3.96E-06
F39E9.4	21	42	14	11	2.80E-06	4.50E-06	1.82E-06	2.25E-06
F39E9.6	2	1	0	0	2.80E-06	2.65E-06	1.82E-06	2.25E-06
F39E9.7	9	10	9	6	2.80E-06	2.65E-06	1.82E-06	2.25E-06
F39E9.8	1	2	4	1	2.80E-06	2.65E-06	1.82E-06	2.25E-06
F39E9.9	2	3	3	2	2.80E-06	2.65E-06	1.82E-06	2.25E-06
F39F10.2	4	3	7	5	2.80E-06	2.65E-06	1.82E-06	2.25E-06
F39F10.3	9	14	26	16	2.80E-06	2.65E-06	1.82E-06	2.25E-06
F39F10.4	5	6	4	4	2.80E-06	2.65E-06	1.82E-06	2.25E-06
F39F10.5	0	1	2	4	2.80E-06	2.65E-06	2.11E-06	2.25E-06
F39G3.1	35	45	58	50	2.80E-06	2.65E-06	1.82E-06	2.25E-06
F39G3.2	21	20	15	20	2.80E-06	2.65E-06	1.82E-06	2.25E-06
F39G3.3	222	186	280	330	2.80E-06	2.70E-06	2.41E-06	2.56E-06
F39G3.4	2	3	4	2	2.80E-06	2.65E-06	1.82E-06	2.25E-06
F39G3.5a	36	76	43	34	1.59E-05	1.26E-05	1.31E-05	1.90E-05
F39G3.5b	42	79	50	35	2.80E-06	2.65E-06	1.82E-06	2.25E-06
F39G3.6	22	10	20	14	4.76E-06	9.47E-06	3.70E-06	3.60E-06

F39G3.7	38	49	57	71	4.65E-06	8.25E-06	3.59E-06	3.10E-06
F39G3.8	28	42	50	29	2.80E-06	2.65E-06	1.82E-06	2.25E-06
F39H11.1	315	275	189	240	2.80E-06	2.65E-06	1.82E-06	2.81E-06
F39H11.2	575	581	669	958	2.80E-06	3.81E-06	3.12E-06	2.25E-06
F39H11.3	339	368	398	616	4.94E-05	4.08E-05	1.93E-05	3.02E-05
F39H11.5.1	1008	1213	1517	1362	2.77E-05	2.65E-05	2.10E-05	3.71E-05
F39H12.1	40	63	28	35	1.93E-05	1.98E-05	1.47E-05	2.82E-05
F39H12.2	8	10	14	3	1.29E-04	1.46E-04	1.26E-04	1.40E-04
F39H12.3	75	79	51	76	3.22E-06	4.79E-06	1.82E-06	2.27E-06
F39H12.4	62	98	43	53	2.83E-06	3.33E-06	3.21E-06	2.25E-06
F39H2.2a	240	323	323	396	1.47E-05	1.47E-05	6.52E-06	1.20E-05
F39H2.2b.1	180	252	231	321	2.80E-06	3.09E-06	1.82E-06	2.25E-06
F39H2.2b.2	225	309	303	382	1.90E-05	2.42E-05	1.67E-05	2.52E-05
F39H2.4	167	211	173	223	1.78E-05	2.36E-05	1.49E-05	2.55E-05
F39H2.5.1	237	255	379	496	1.96E-05	2.55E-05	1.72E-05	2.68E-05
F39H2.5.2	222	241	358	485	2.79E-05	3.33E-05	1.88E-05	2.99E-05
F40A3.1	32	85	33	41	1.42E-05	1.45E-05	1.48E-05	2.39E-05
F40A3.2	116	196	103	120	1.36E-05	1.39E-05	1.42E-05	2.38E-05
F40A3.3a	345	349	537	267	4.03E-06	1.01E-05	2.70E-06	4.14E-06
F40A3.4	9	8	13	4	1.58E-05	2.53E-05	9.15E-06	1.32E-05
F40A3.5	24	29	22	37	4.98E-05	4.76E-05	5.04E-05	3.09E-05
F40A3.6	119	193	100	115	2.80E-06	2.65E-06	2.30E-06	2.25E-06
F40A3.7	12	16	11	14	2.80E-06	2.65E-06	1.82E-06	2.25E-06
F40B1.1	4	6	10	2	1.72E-05	2.63E-05	9.38E-06	1.33E-05
F40B1.2	1	8	9	8	2.80E-06	2.65E-06	1.82E-06	2.25E-06
F40B5.1	7	10	14	16	2.80E-06	2.65E-06	1.82E-06	2.25E-06
F40B5.2a	83	111	71	126	2.80E-06	2.65E-06	1.82E-06	2.25E-06
F40B5.2b	74	104	66	118	2.80E-06	2.65E-06	1.82E-06	2.50E-06
F40B5.3	7	5	2	7	5.99E-06	7.56E-06	3.33E-06	7.29E-06
F40C5.1	2	3	2	2	6.24E-06	8.31E-06	3.63E-06	8.01E-06
F40C5.2	41	27	22	26	2.80E-06	2.65E-06	1.82E-06	2.25E-06
F40C5.3	34	14	31	3	2.80E-06	2.65E-06	1.82E-06	2.25E-06
F40D4.1	3	2	4	1	9.32E-06	5.82E-06	3.26E-06	4.75E-06
F40D4.10	6	5	3	0	9.07E-06	3.52E-06	5.38E-06	2.25E-06
F40D4.11	4	7	0	2	2.80E-06	2.65E-06	1.82E-06	2.25E-06
F40D4.12	6	9	11	3	2.80E-06	2.65E-06	1.82E-06	2.25E-06
F40D4.13	8	13	6	3	2.80E-06	2.65E-06	1.82E-06	2.25E-06
F40D4.14	24	36	48	40	2.80E-06	2.65E-06	1.82E-06	2.25E-06
F40D4.2	3	2	5	2	2.80E-06	2.65E-06	1.82E-06	2.25E-06
F40D4.3	4	6	4	2	2.80E-06	2.65E-06	1.82E-06	2.25E-06
F40D4.4	11	13	12	13	2.80E-06	2.65E-06	1.82E-06	2.25E-06
F40D4.5	2	5	6	2	2.80E-06	2.65E-06	1.82E-06	2.25E-06
F40D4.6	22	20	26	27	2.80E-06	2.65E-06	1.82E-06	2.25E-06
F40D4.7	8	13	6	12	2.80E-06	2.65E-06	1.82E-06	2.25E-06
F40D4.8	3	8	4	3	2.80E-06	2.65E-06	2.11E-06	2.72E-06
F40D4.9a	5	9	6	9	2.80E-06	2.65E-06	1.82E-06	2.25E-06
F40E10.1	49	28	22	119	2.80E-06	2.65E-06	1.82E-06	2.25E-06
F40E10.2	9	17	11	10	2.80E-06	2.65E-06	1.82E-06	2.25E-06
F40E10.3.1	1205	1682	1561	1805	2.86E-06	2.65E-06	1.82E-06	5.56E-06
F40E10.3.2	1070	1507	1368	1663	2.80E-06	2.80E-06	1.82E-06	2.25E-06
F40E10.4	53	85	94	76	9.63E-05	1.27E-04	8.12E-05	1.16E-04
F40E10.5	104	111	25	21	9.55E-05	1.27E-04	7.94E-05	1.19E-04
F40E10.6.1	442	474	397	635	2.80E-06	2.65E-06	1.82E-06	2.25E-06
F40E10.6.2	448	490	413	652	1.16E-05	1.17E-05	1.82E-06	2.25E-06
F40E10.6.3	443	479	396	637	3.27E-05	3.31E-05	1.91E-05	3.77E-05
F40E12.1	1	1	0	0	2.98E-05	3.08E-05	1.79E-05	3.48E-05
F40E12.2	38	50	70	39	3.25E-05	3.32E-05	1.89E-05	3.76E-05
F40E3.2	131	168	258	242	2.80E-06	2.65E-06	1.82E-06	2.25E-06
F40E3.3	7	8	2	2	2.80E-06	2.65E-06	1.82E-06	2.25E-06

F40E3.5.1	11	22	14	9	1.93E-05	2.33E-05	2.47E-05	2.86E-05
F40E3.5.2	12	22	15	10	2.80E-06	2.65E-06	1.82E-06	2.25E-06
F40E3.6	1	2	1	3	2.80E-06	2.65E-06	1.82E-06	2.25E-06
F40F11.1.1	9347	9796	36242	9389	2.80E-06	2.65E-06	1.82E-06	2.25E-06
F40F11.1.2	8422	8788	28362	8649	2.80E-06	2.65E-06	1.82E-06	2.25E-06
F40F11.3	135	164	140	110	1.91E-03	1.89E-03	4.82E-03	1.54E-03
F40F11.4	4	10	4	1	1.72E-03	1.70E-03	3.77E-03	1.42E-03
F40F12.1	104	139	341	157	4.20E-05	4.82E-05	2.84E-05	2.75E-05
F40F12.10	8	3	2	7	2.80E-06	2.65E-06	1.82E-06	2.25E-06
F40F12.3	11	28	5	5	3.08E-05	3.89E-05	6.57E-05	3.74E-05
F40F12.4	7	9	41	10	4.68E-06	2.65E-06	1.82E-06	3.28E-06
F40F12.5	1476	1540	1978	2869	2.80E-06	3.04E-06	1.82E-06	2.25E-06
F40F12.7.1	126	292	143	127	2.80E-06	2.65E-06	1.84E-06	2.25E-06
F40F12.7.2	123	287	142	127	4.33E-05	4.26E-05	3.77E-05	6.75E-05
F40F12.8	12	19	7	4	1.68E-05	3.68E-05	1.24E-05	1.36E-05
F40F12.9	2	2	1	0	1.47E-05	3.24E-05	1.10E-05	1.22E-05
F40F4.1	0	3	12	4	2.80E-06	3.12E-06	1.82E-06	2.25E-06
F40F4.4a	181	301	143	240	2.80E-06	2.65E-06	1.82E-06	2.25E-06
F40F4.4b	121	204	94	183	2.80E-06	2.65E-06	1.82E-06	2.25E-06
F40F4.5	31	24	40	19	2.20E-05	3.45E-05	1.13E-05	2.34E-05
F40F4.6	5797	7004	10204	9325	2.99E-05	4.76E-05	1.51E-05	3.63E-05
F40F4.7	108	187	144	136	2.80E-06	2.65E-06	2.13E-06	2.25E-06
F40F4.8	19	11	5	12	9.57E-05	1.09E-04	1.10E-04	1.24E-04
F40F8.1.1	288	370	522	367	9.91E-06	1.62E-05	8.58E-06	1.00E-05
F40F8.1.2	261	336	416	321	3.05E-06	2.65E-06	1.82E-06	2.25E-06
F40F8.10.1	9176	8401	16939	11294	3.22E-05	3.90E-05	3.79E-05	3.29E-05
F40F8.10.2	8180	7447	14307	10673	2.98E-05	3.63E-05	3.09E-05	2.95E-05
F40F8.3	27	37	117	27	1.60E-03	1.38E-03	1.92E-03	1.58E-03
F40F8.4	43	82	57	21	1.51E-03	1.30E-03	1.72E-03	1.59E-03
F40F8.5	329	506	444	623	4.40E-06	5.66E-06	1.24E-05	3.53E-06
F40F9.10	22	24	23	11	8.12E-06	1.46E-05	7.00E-06	3.19E-06
F40F9.11	4	3	6	2	1.29E-05	1.86E-05	1.13E-05	1.95E-05
F40F9.1a	767	833	955	1180	2.80E-06	2.65E-06	1.82E-06	2.25E-06
F40F9.1b	772	845	959	1191	2.80E-06	2.65E-06	1.82E-06	2.25E-06
F40F9.2	210	281	219	224	7.47E-05	7.66E-05	6.05E-05	9.23E-05
F40F9.3	47	70	24	22	9.68E-05	1.00E-04	7.83E-05	1.20E-04
F40F9.4	2	2	3	3	2.24E-05	2.83E-05	1.52E-05	1.92E-05
F40F9.5	91	175	70	122	6.36E-06	8.94E-06	2.11E-06	2.38E-06
F40F9.6a	1612	1914	2022	2859	2.80E-06	2.65E-06	1.82E-06	2.25E-06
F40F9.7b.1	466	540	571	733	5.35E-06	9.73E-06	2.68E-06	5.76E-06
F40F9.7b.2	480	574	577	738	6.51E-05	7.30E-05	5.31E-05	9.27E-05
F40F9.7b.3	481	581	577	734	5.35E-05	5.86E-05	4.27E-05	6.76E-05
F40F9.7b.4	478	572	572	731	3.63E-05	4.11E-05	2.84E-05	4.49E-05
F40F9.8	3	3	2	3	4.48E-05	5.11E-05	3.50E-05	5.49E-05
F40G12.1	2	7	7	4	4.46E-05	5.04E-05	3.47E-05	5.48E-05
F40G12.10	46	80	24	11	2.80E-06	2.65E-06	1.82E-06	2.25E-06
F40G12.11	122	160	228	213	2.80E-06	2.65E-06	1.82E-06	2.25E-06
F40G12.13	0	1	6	1	3.92E-06	6.43E-06	1.82E-06	2.25E-06
F40G12.14	0	1	6	1	9.83E-06	1.22E-05	1.20E-05	1.38E-05
F40G12.15	3	4	6	3	2.80E-06	2.65E-06	2.64E-06	2.25E-06
F40G12.2	6	2	7	5	2.80E-06	2.65E-06	2.64E-06	2.25E-06
F40G12.3	13	14	29	13	2.80E-06	2.65E-06	1.82E-06	2.25E-06
F40G12.4	5	2	0	1	2.80E-06	2.65E-06	1.82E-06	2.25E-06
F40G12.5	1	2	1	1	2.80E-06	2.65E-06	1.82E-06	2.25E-06
F40G12.6	2	4	11	4	2.80E-06	2.65E-06	1.82E-06	2.25E-06
F40G12.7	6	7	8	2	2.80E-06	2.65E-06	1.82E-06	2.25E-06
F40G12.8	2	5	2	6	2.80E-06	2.65E-06	1.82E-06	2.25E-06
F40G12.9	4	0	0	1	2.80E-06	2.65E-06	1.82E-06	2.25E-06
F40G9.1	85	121	287	211	2.80E-06	2.65E-06	1.82E-06	2.25E-06

F40G9.10	5	0	0	2	2.80E-06	2.65E-06	1.82E-06	2.25E-06
F40G9.11	163	108	267	232	4.98E-06	6.69E-06	1.10E-05	9.94E-06
F40G9.12	2	0	3	1	2.80E-06	2.65E-06	1.82E-06	2.25E-06
F40G9.14	3	7	7	5	2.88E-05	1.80E-05	3.07E-05	3.30E-05
F40G9.2	61	75	51	46	2.80E-06	2.65E-06	1.82E-06	2.25E-06
F40G9.3	972	762	870	1009	2.80E-06	2.65E-06	1.82E-06	2.25E-06
F40G9.4	5	14	11	11	2.11E-05	2.46E-05	1.15E-05	1.28E-05
F40G9.5	15	13	18	20	1.12E-04	8.29E-05	6.52E-05	9.33E-05
F40G9.6	4	5	1	7	2.80E-06	2.65E-06	1.82E-06	2.25E-06
F40G9.7	5	2	0	1	2.80E-06	2.65E-06	1.82E-06	2.25E-06
F40G9.8	4	9	0	1	2.80E-06	2.65E-06	1.82E-06	2.25E-06
F40G9.9	9	13	13	6	2.80E-06	2.65E-06	1.82E-06	2.25E-06
F40H3.1a	103	139	121	136	2.80E-06	2.65E-06	1.82E-06	2.25E-06
F40H3.1b	81	117	94	119	2.80E-06	2.65E-06	1.82E-06	2.25E-06
F40H3.2	36	62	49	34	7.73E-06	9.87E-06	5.92E-06	8.21E-06
F40H3.3	7	16	3	5	1.15E-05	1.56E-05	8.66E-06	1.35E-05
F40H3.4	5	12	6	5	9.41E-06	1.53E-05	8.35E-06	7.15E-06
F40H3.5	4	14	9	6	2.80E-06	3.89E-06	1.82E-06	2.25E-06
F40H3.6	15	11	8	11	2.80E-06	2.65E-06	1.82E-06	2.25E-06
F40H6.1	18	41	17	15	2.80E-06	2.65E-06	1.82E-06	2.25E-06
F40H6.2	10	13	3	3	5.52E-06	3.84E-06	1.91E-06	3.26E-06
F40H6.4	7	19	12	11	3.72E-06	8.04E-06	2.30E-06	2.50E-06
F40H6.5	13	11	21	12	2.80E-06	2.65E-06	1.82E-06	2.25E-06
F40H6.6	43	44	41	61	2.80E-06	2.65E-06	1.82E-06	2.25E-06
F40H6.t1	3	2	3	2	2.80E-06	2.65E-06	1.82E-06	2.25E-06
F40H7.1	2	7	6	2	1.08E-05	1.04E-05	6.69E-06	1.23E-05
F40H7.10	2	3	2	4	4.09E-06	2.65E-06	2.66E-06	2.25E-06
F40H7.11	0	2	3	1	2.80E-06	2.65E-06	1.82E-06	2.25E-06
F40H7.12	2	1	3	2	2.80E-06	2.65E-06	1.82E-06	2.25E-06
F40H7.2	4	1	6	1	2.80E-06	2.65E-06	1.82E-06	2.25E-06
F40H7.3	10	4	1	2	2.80E-06	2.65E-06	1.82E-06	2.25E-06
F40H7.4	0	3	6	0	2.80E-06	2.65E-06	1.82E-06	2.25E-06
F40H7.5	2	0	32	4	2.80E-06	2.65E-06	1.82E-06	2.25E-06
F40H7.6	2	3	1	3	2.80E-06	2.65E-06	1.82E-06	2.25E-06
F40H7.7	1	3	2	2	2.80E-06	2.65E-06	2.42E-06	2.25E-06
F40H7.8	3	5	10	3	2.80E-06	2.65E-06	1.82E-06	2.25E-06
F40H7.9	0	2	1	1	2.80E-06	2.65E-06	1.82E-06	2.25E-06
F41A4.1	38	42	42	40	2.80E-06	2.65E-06	1.82E-06	2.25E-06
F41B4.1	95	114	112	129	2.80E-06	2.65E-06	1.82E-06	2.25E-06
F41B4.2a	10	28	19	11	2.80E-06	2.65E-06	1.82E-06	2.25E-06
F41B4.2b.1	12	30	19	13	8.15E-06	9.23E-06	6.25E-06	8.88E-06
F41B4.2b.2	12	30	20	13	2.80E-06	6.14E-06	2.86E-06	2.25E-06
F41B4.2b.3	11	27	16	12	2.80E-06	5.48E-06	2.39E-06	2.25E-06
F41B4.3	10	19	26	13	2.80E-06	6.00E-06	2.77E-06	2.25E-06
F41B4.4a	30	49	15	28	2.80E-06	5.05E-06	2.06E-06	2.25E-06
F41B4.4b	13	20	8	8	2.80E-06	4.76E-06	4.48E-06	2.77E-06
F41B5.1	16	8	6	6	2.80E-06	2.65E-06	1.82E-06	2.25E-06
F41B5.10	7	6	14	12	2.80E-06	2.65E-06	1.82E-06	2.25E-06
F41B5.2	45	40	33	40	3.11E-06	2.65E-06	1.82E-06	2.25E-06
F41B5.3	17	20	13	13	2.80E-06	2.65E-06	1.82E-06	2.25E-06
F41B5.4	11	20	8	18	3.56E-06	2.99E-06	1.82E-06	2.54E-06
F41B5.6	22	19	15	35	2.80E-06	2.65E-06	1.82E-06	2.25E-06
F41B5.7	5	2	9	7	2.80E-06	2.65E-06	1.82E-06	2.25E-06
F41B5.8	3	4	7	3	2.80E-06	2.65E-06	1.82E-06	2.25E-06
F41B5.9	32	39	61	40	2.80E-06	2.65E-06	1.82E-06	2.25E-06
F41C3.1	1	4	2	2	2.80E-06	2.65E-06	1.82E-06	2.25E-06
F41C3.11	70	105	27	57	2.80E-06	2.72E-06	2.95E-06	2.38E-06
F41C3.2	109	418	48	85	2.80E-06	2.65E-06	1.82E-06	2.25E-06
F41C3.3.1	213	334	232	365	6.05E-06	8.54E-06	1.82E-06	3.96E-06

F41C3.3.2	211	334	236	358	7.00E-06	2.54E-05	2.00E-06	4.39E-06
F41C3.4	189	240	197	225	1.43E-05	2.12E-05	1.01E-05	1.97E-05
F41C3.5.1	2936	4393	3450	4910	1.55E-05	2.33E-05	1.13E-05	2.12E-05
F41C3.5.2	2711	4134	3208	4765	2.70E-05	3.24E-05	1.83E-05	2.58E-05
F41C3.6	5	11	29	9	2.01E-04	2.84E-04	1.54E-04	2.70E-04
F41C3.7	5	4	9	3	2.15E-04	3.10E-04	1.66E-04	3.04E-04
F41C3.8a	24	18	12	18	2.80E-06	2.65E-06	2.30E-06	2.25E-06
F41C3.8b	27	23	29	21	2.80E-06	2.65E-06	1.82E-06	2.25E-06
F41C6.1.1	58	84	46	58	2.80E-06	2.65E-06	1.82E-06	2.25E-06
F41C6.1.2	51	70	40	56	2.80E-06	2.65E-06	1.91E-06	2.25E-06
F41C6.2	5	1	1	1	2.80E-06	3.70E-06	1.82E-06	2.25E-06
F41C6.3	1	1	0	0	2.80E-06	3.36E-06	1.82E-06	2.29E-06
F41C6.4	1	14	5	9	2.80E-06	2.65E-06	1.82E-06	2.25E-06
F41C6.5	74	116	35	33	2.80E-06	2.65E-06	1.82E-06	2.25E-06
F41C6.6	2	1	0	0	2.80E-06	2.65E-06	1.82E-06	2.25E-06
F41C6.7	9	16	38	18	7.84E-06	1.16E-05	2.41E-06	2.81E-06
F41D3.1	2	1	3	5	2.80E-06	2.65E-06	1.82E-06	2.25E-06
F41D3.10	9	7	21	8	2.80E-06	2.65E-06	2.35E-06	2.25E-06
F41D3.11	4	4	3	4	2.80E-06	2.65E-06	1.82E-06	2.25E-06
F41D3.12	2	3	2	1	2.80E-06	2.65E-06	1.82E-06	2.25E-06
F41D3.13	0	1	0	0	2.80E-06	2.65E-06	1.82E-06	2.25E-06
F41D3.2	5	8	7	5	2.80E-06	2.65E-06	1.82E-06	2.25E-06
F41D3.3	2	8	7	3	2.80E-06	2.65E-06	1.82E-06	2.25E-06
F41D3.4	3	4	4	6	2.80E-06	2.65E-06	1.82E-06	2.25E-06
F41D3.5	9	7	22	8	2.80E-06	2.65E-06	1.82E-06	2.25E-06
F41D3.6	3	4	2	1	2.80E-06	2.65E-06	1.82E-06	2.25E-06
F41D3.7	3	1	1	0	2.80E-06	2.65E-06	1.82E-06	2.25E-06
F41D3.8	1	4	0	1	2.80E-06	2.65E-06	1.82E-06	2.25E-06
F41D3.9	1	2	8	2	2.80E-06	2.65E-06	1.82E-06	2.25E-06
F41D9.1	24	30	37	36	2.80E-06	2.65E-06	1.82E-06	2.25E-06
F41D9.2	48	65	49	60	2.80E-06	2.65E-06	1.82E-06	2.25E-06
F41D9.3a	158	195	126	215	2.80E-06	2.65E-06	1.82E-06	2.25E-06
F41D9.3c	192	234	166	265	1.06E-05	1.36E-05	7.05E-06	1.06E-05
F41D9.3d	59	76	46	86	1.35E-05	1.57E-05	7.00E-06	1.48E-05
F41D9.3e	173	203	139	227	1.40E-05	1.61E-05	7.89E-06	1.55E-05
F41D9.5	18	32	18	19	1.01E-05	1.23E-05	5.12E-06	1.18E-05
F41D9.t1	0	0	1	1	1.43E-05	1.58E-05	7.45E-06	1.50E-05
F41D9.t2	0	0	1	1	2.80E-06	2.65E-06	1.82E-06	2.25E-06
F41D9.t4	0	0	0	1	2.80E-06	2.65E-06	1.82E-06	2.25E-06
F41E6.1	4	12	3	1	2.80E-06	2.65E-06	1.82E-06	2.25E-06
F41E6.10	2	3	3	0	2.80E-06	2.65E-06	1.82E-06	2.25E-06
F41E6.11	42	41	8	14	2.80E-06	2.65E-06	1.82E-06	2.25E-06
F41E6.12	92	200	107	113	2.80E-06	2.65E-06	1.82E-06	2.25E-06
F41E6.13a	1121	1113	1357	1757	4.87E-06	4.47E-06	1.82E-06	2.25E-06
F41E6.13b.1	1150	1147	1421	1794	1.13E-05	2.31E-05	8.53E-06	1.11E-05
F41E6.14	56	48	31	12	7.22E-05	6.77E-05	5.69E-05	9.08E-05
F41E6.15	15	58	32	27	7.55E-05	7.11E-05	6.07E-05	9.45E-05
F41E6.2	820	822	361	465	3.19E-06	2.65E-06	1.82E-06	2.25E-06
F41E6.4a	1473	1432	1724	2945	6.08E-06	2.22E-05	8.45E-06	8.79E-06
F41E6.4b	1135	1117	1453	2463	1.88E-04	1.78E-04	5.39E-05	8.57E-05
F41E6.4c	711	682	712	1205	5.07E-05	4.65E-05	3.86E-05	8.13E-05
F41E6.5	108	176	162	165	4.80E-05	4.47E-05	4.00E-05	8.38E-05
F41E6.6.1	204	536	134	364	4.34E-05	3.93E-05	2.83E-05	5.91E-05
F41E6.6.2	182	477	127	352	7.92E-06	1.22E-05	7.73E-06	9.72E-06
F41E6.7	4	4	0	0	1.44E-05	3.57E-05	6.14E-06	2.06E-05
F41E6.8	3	6	3	3	1.42E-05	3.51E-05	6.43E-06	2.20E-05
F41E6.9	440	634	569	664	2.80E-06	2.65E-06	1.82E-06	2.25E-06
F41E7.1	80	100	100	113	2.80E-06	2.65E-06	1.82E-06	2.25E-06
F41E7.2.1	27	48	33	51	4.41E-05	6.00E-05	3.71E-05	5.35E-05

F41E7.2.2	27	42	27	45	5.15E-06	6.08E-06	4.19E-06	5.85E-06
F41E7.2.3	26	41	26	45	2.80E-06	2.67E-06	1.82E-06	2.41E-06
F41E7.3	17	26	12	8	2.80E-06	2.65E-06	1.82E-06	2.38E-06
F41E7.4	32	54	120	35	2.80E-06	2.65E-06	1.82E-06	2.41E-06
F41E7.5	332	339	537	182	2.80E-06	2.65E-06	1.82E-06	2.25E-06
F41E7.6	3	5	45	8	9.16E-06	1.46E-05	2.23E-05	8.03E-06
F41E7.7	3	4	9	4	8.53E-05	8.23E-05	8.98E-05	3.76E-05
F41E7.9	19	16	16	14	2.80E-06	2.65E-06	1.97E-06	2.25E-06
F41F3.1	10	12	14	7	2.80E-06	2.65E-06	3.43E-06	2.25E-06
F41F3.2	48	66	54	52	2.80E-06	2.65E-06	1.82E-06	2.25E-06
F41F3.3.1	1062	1494	1102	289	2.80E-06	2.65E-06	1.82E-06	2.25E-06
F41F3.3.2	810	1113	816	257	3.98E-06	5.18E-06	2.92E-06	3.46E-06
F41F3.4	2723	2600	3786	2817	2.03E-04	2.70E-04	1.37E-04	4.44E-05
F41F3.5	2	4	1	3	1.55E-04	2.01E-04	1.01E-04	3.94E-05
F41F3.6	5	7	11	7	3.08E-04	2.78E-04	2.78E-04	2.56E-04
F41F3.7	6	3	5	1	2.80E-06	2.65E-06	1.82E-06	2.25E-06
F41F3.8	13	10	20	10	2.80E-06	2.65E-06	1.82E-06	2.25E-06
F41G3.1.1	22	28	37	19	2.80E-06	2.65E-06	1.82E-06	2.25E-06
F41G3.1.2	21	26	23	19	4.09E-06	2.96E-06	4.08E-06	2.52E-06
F41G3.10	66	180	158	76	6.44E-06	7.72E-06	7.03E-06	4.45E-06
F41G3.11	3	1	4	3	6.30E-06	7.35E-06	4.48E-06	4.57E-06
F41G3.12	42	47	33	55	9.83E-06	2.53E-05	1.53E-05	9.09E-06
F41G3.14	196	193	222	275	2.80E-06	2.65E-06	1.82E-06	2.25E-06
F41G3.16	1	3	8	1	2.80E-06	2.65E-06	1.82E-06	2.25E-06
F41G3.17	1	2	0	1	2.60E-05	2.41E-05	1.91E-05	2.92E-05
F41G3.18	19	37	21	20	2.80E-06	2.65E-06	2.37E-06	2.25E-06
F41G3.19	5	6	3	3	2.80E-06	2.65E-06	1.82E-06	2.25E-06
F41G3.2	6	15	28	16	6.27E-06	1.16E-05	4.52E-06	5.31E-06
F41G3.20	3	3	1	1	2.80E-06	2.65E-06	1.82E-06	2.25E-06
F41G3.3	40	37	23	19	2.80E-06	2.65E-06	2.11E-06	2.25E-06
F41G3.4	9	19	10	9	2.80E-06	2.65E-06	1.82E-06	2.25E-06
F41G3.5	13	42	14	14	2.80E-06	2.65E-06	1.82E-06	2.25E-06
F41G3.6	336	376	452	515	2.80E-06	4.66E-06	1.82E-06	2.25E-06
F41G4.1	6	10	4	3	2.80E-06	5.61E-06	1.82E-06	2.25E-06
F41G4.2a	369	541	416	678	3.73E-05	3.94E-05	3.27E-05	4.59E-05
F41G4.2b.1	314	507	414	633	2.80E-06	2.65E-06	1.82E-06	2.25E-06
F41G4.2b.2	301	491	391	616	1.09E-05	1.52E-05	8.04E-06	1.62E-05
F41G4.3a	25	55	12	21	2.04E-05	3.11E-05	1.75E-05	3.30E-05
F41G4.3b	19	42	9	12	1.99E-05	3.07E-05	1.69E-05	3.28E-05
F41G4.3c	14	28	8	8	2.80E-06	4.87E-06	1.82E-06	2.25E-06
F41G4.4	0	0	1	0	2.80E-06	4.73E-06	1.82E-06	2.25E-06
F41G4.5	3	4	3	4	2.97E-06	5.61E-06	1.82E-06	2.25E-06
F41G4.7	6	12	13	11	2.80E-06	2.65E-06	1.82E-06	2.25E-06
F41G4.8	10	33	6	11	2.80E-06	2.65E-06	1.82E-06	2.25E-06
F41H10.10	233	364	388	423	2.80E-06	2.65E-06	1.82E-06	2.25E-06
F41H10.11	399	491	598	748	2.80E-06	4.79E-06	1.82E-06	2.25E-06
F41H10.12	3	5	4	1	2.46E-05	3.64E-05	2.67E-05	3.59E-05
F41H10.2	218	358	107	133	2.95E-05	3.43E-05	2.88E-05	4.44E-05
F41H10.3a	1214	1068	1509	2049	2.80E-06	2.65E-06	1.82E-06	2.25E-06
F41H10.3b	1199	1055	1489	2020	1.40E-05	2.18E-05	4.48E-06	6.88E-06
F41H10.4.1	142	280	169	237	3.48E-05	2.89E-05	2.81E-05	4.71E-05
F41H10.4.2	131	252	151	222	3.48E-05	2.89E-05	2.81E-05	4.71E-05
F41H10.5	9	18	8	6	8.32E-06	1.55E-05	6.45E-06	1.12E-05
F41H10.6a	766	770	1213	1491	8.48E-06	1.54E-05	6.36E-06	1.16E-05
F41H10.6b	697	702	1141	1416	2.80E-06	2.65E-06	1.82E-06	2.25E-06
F41H10.7	1685	1668	1470	2027	2.56E-05	2.43E-05	2.64E-05	4.01E-05
F41H10.9	1	0	2	1	2.65E-05	2.52E-05	2.83E-05	4.33E-05
F41H8.1	3	4	5	2	2.00E-04	1.87E-04	1.14E-04	1.93E-04
F41H8.2	3	4	3	2	2.80E-06	2.65E-06	1.82E-06	2.25E-06

F41H8.3	1	1	3	2	2.80E-06	2.65E-06	1.82E-06	2.25E-06
F41H8.4	1	0	5	0	2.80E-06	2.65E-06	1.82E-06	2.25E-06
F42A10.1.1	958	1015	1160	1583	2.80E-06	2.65E-06	1.82E-06	2.25E-06
F42A10.1.2	934	996	1126	1563	2.80E-06	2.65E-06	1.82E-06	2.25E-06
F42A10.1.3	922	977	1107	1549	4.43E-05	4.43E-05	3.49E-05	5.88E-05
F42A10.2a	239	285	73	183	4.29E-05	4.32E-05	3.37E-05	5.76E-05
F42A10.2b	96	177	59	168	4.48E-05	4.48E-05	3.50E-05	6.04E-05
F42A10.2c	92	172	57	163	1.22E-05	1.37E-05	2.42E-06	7.49E-06
F42A10.3	119	243	133	79	4.56E-06	7.94E-06	1.82E-06	6.41E-06
F42A10.4a.1	741	889	903	1350	4.31E-06	7.64E-06	1.82E-06	6.14E-06
F42A10.4a.2	613	746	730	1124	1.21E-05	2.33E-05	8.78E-06	6.43E-06
F42A10.4b	617	738	734	1130	3.05E-05	3.46E-05	2.42E-05	4.46E-05
F42A10.5.1	839	1058	1406	1405	2.95E-05	3.40E-05	2.29E-05	4.35E-05
F42A10.5.2	437	476	844	854	3.00E-05	3.39E-05	2.33E-05	4.42E-05
F42A10.7.1	188	125	86	122	6.24E-05	7.44E-05	6.81E-05	8.40E-05
F42A10.7.2	144	101	64	102	5.77E-05	5.94E-05	7.25E-05	9.06E-05
F42A10.8	6	9	15	7	3.19E-05	2.00E-05	9.49E-06	1.66E-05
F42A10.9	65	72	48	46	2.67E-05	1.77E-05	7.73E-06	1.52E-05
F42A6.1	3	4	6	4	2.80E-06	2.65E-06	1.82E-06	2.25E-06
F42A6.2	4	2	1	3	1.38E-05	1.44E-05	6.63E-06	7.83E-06
F42A6.3	881	692	1397	1654	2.80E-06	2.65E-06	1.82E-06	2.25E-06
F42A6.4	22	41	32	30	2.80E-06	2.65E-06	1.82E-06	2.25E-06
F42A6.5	20	18	23	23	4.21E-05	3.12E-05	4.35E-05	6.35E-05
F42A6.6	25	32	52	61	2.80E-06	3.02E-06	1.82E-06	2.25E-06
F42A6.7a.1	992	1133	1004	1368	7.25E-06	6.16E-06	5.43E-06	6.70E-06
F42A6.7a.2	730	834	812	1166	5.71E-06	6.93E-06	7.74E-06	1.12E-05
F42A6.7b.1	940	1103	978	1303	7.47E-05	8.06E-05	4.92E-05	8.27E-05
F42A6.7b.2	698	878	806	1175	6.77E-05	7.31E-05	4.90E-05	8.68E-05
F42A6.7b.3	921	1073	961	1294	5.61E-05	6.22E-05	3.80E-05	6.25E-05
F42A6.7b.4	729	839	809	1161	5.71E-05	6.78E-05	4.29E-05	7.72E-05
F42A6.7c	893	1054	928	1320	6.97E-05	7.67E-05	4.73E-05	7.86E-05
F42A6.7d	892	1059	925	1315	6.90E-05	7.50E-05	4.98E-05	8.83E-05
F42A6.8	7	2	2	3	8.48E-05	9.45E-05	5.73E-05	1.01E-04
F42A6.9	95	132	104	168	8.45E-05	9.47E-05	5.70E-05	1.00E-04
F42A8.1	314	387	271	438	2.80E-06	2.65E-06	1.82E-06	2.25E-06
F42A8.2.1	994	1237	1666	1816	3.75E-06	4.92E-06	2.66E-06	5.31E-06
F42A8.2.2	953	1202	1559	1764	2.86E-05	3.33E-05	1.61E-05	3.20E-05
F42A8.3	208	257	311	288	9.85E-05	1.16E-04	1.07E-04	1.45E-04
F42A9.1a	620	574	1137	1707	9.77E-05	1.16E-04	1.04E-04	1.45E-04
F42A9.1b	446	440	907	1454	2.20E-05	2.57E-05	2.14E-05	2.44E-05
F42A9.2	690	708	925	1312	1.44E-05	1.26E-05	1.72E-05	3.20E-05
F42A9.3	7	15	7	3	1.26E-05	1.17E-05	1.67E-05	3.30E-05
F42A9.4	9	14	15	2	2.10E-05	2.03E-05	1.83E-05	3.20E-05
F42A9.5	48	70	46	31	2.80E-06	3.89E-06	1.82E-06	2.25E-06
F42A9.6	175	204	134	177	2.80E-06	2.65E-06	1.82E-06	2.25E-06
F42A9.7	44	59	20	17	3.30E-06	4.55E-06	2.06E-06	2.25E-06
F42A9.8	62	91	225	85	5.04E-05	5.55E-05	2.51E-05	4.09E-05
F42A9.9	1	6	1	5	6.13E-06	7.78E-06	1.82E-06	2.25E-06
F42C5.10	507	543	453	589	1.93E-05	2.67E-05	4.56E-05	2.12E-05
F42C5.2	4	12	21	13	2.80E-06	2.65E-06	1.82E-06	2.25E-06
F42C5.3	6	13	11	7	1.41E-05	1.43E-05	8.18E-06	1.31E-05
F42C5.4	27	30	27	28	2.80E-06	2.65E-06	1.82E-06	2.25E-06
F42C5.5	19	36	7	19	2.80E-06	2.65E-06	1.82E-06	2.25E-06
F42C5.6	6	6	4	2	2.80E-06	2.65E-06	1.82E-06	2.25E-06
F42C5.7	338	499	205	224	2.80E-06	3.91E-06	1.82E-06	2.25E-06
F42C5.8.1	10510	10854	19208	17392	2.80E-06	2.65E-06	1.82E-06	2.25E-06
F42C5.8.2	10064	10267	16909	16903	4.59E-05	6.41E-05	1.81E-05	2.44E-05
F42C5.9.1	310	434	273	420	1.70E-03	1.66E-03	2.02E-03	2.26E-03
F42C5.9.2	253	370	238	377	1.79E-03	1.73E-03	1.96E-03	2.42E-03

F42D1.2.1	620	1146	536	1048	1.74E-05	2.30E-05	9.95E-06	1.89E-05
F42D1.2.2	491	895	423	800	1.70E-05	2.35E-05	1.04E-05	2.03E-05
F42D1.4	0	3	1	1	3.91E-05	6.83E-05	2.20E-05	5.31E-05
F42E11.2a	212	292	156	321	3.80E-05	6.55E-05	2.13E-05	4.98E-05
F42E11.2b	38	45	25	57	2.80E-06	2.65E-06	1.82E-06	2.25E-06
F42E11.2c	196	274	144	296	1.25E-05	1.62E-05	5.96E-06	1.52E-05
F42E11.3	5	5	3	1	1.06E-05	1.19E-05	4.54E-06	1.28E-05
F42E8.1	8	9	10	5	1.21E-05	1.59E-05	5.76E-06	1.46E-05
F42E8.2	51	59	28	12	2.80E-06	2.65E-06	1.82E-06	2.25E-06
F42F12.1	172	234	118	41	2.80E-06	2.65E-06	1.82E-06	2.25E-06
F42F12.10	238	306	239	81	1.55E-05	1.69E-05	5.54E-06	2.92E-06
F42F12.11	1	7	6	1	5.41E-05	6.95E-05	2.42E-05	1.04E-05
F42F12.12	77	118	42	23	7.15E-05	8.68E-05	4.67E-05	1.95E-05
F42F12.13	55	95	44	16	2.80E-06	2.65E-06	1.82E-06	2.25E-06
F42F12.2	10	22	6	8	2.79E-05	4.04E-05	9.91E-06	6.70E-06
F42F12.3	48	80	97	70	2.81E-05	4.59E-05	1.46E-05	6.57E-06
F42F12.4.1	44	66	42	41	2.80E-06	3.25E-06	1.82E-06	2.25E-06
F42F12.4.2	39	59	36	39	7.34E-06	1.16E-05	9.66E-06	8.61E-06
F42F12.6	138	210	146	37	7.87E-06	1.11E-05	4.88E-06	5.89E-06
F42F12.7	203	293	271	52	7.84E-06	1.12E-05	4.70E-06	6.30E-06
F42F12.9	124	196	148	47	5.05E-05	7.26E-05	3.48E-05	1.09E-05
F42F12.t1	0	1	0	0	5.47E-05	7.45E-05	4.75E-05	1.12E-05
F42G10.1.1	167	265	203	322	4.45E-05	6.65E-05	3.46E-05	1.36E-05
F42G10.1.2	170	267	188	320	2.80E-06	2.65E-06	1.82E-06	2.25E-06
F42G10.2	67	101	55	93	9.86E-06	1.48E-05	7.80E-06	1.52E-05
F42G2.2	42	76	39	49	8.82E-06	1.31E-05	6.34E-06	1.33E-05
F42G2.3	9	3	19	13	4.79E-06	6.80E-06	2.55E-06	5.33E-06
F42G2.4	54	56	68	45	2.80E-06	4.73E-06	1.82E-06	2.59E-06
F42G2.5	8	11	6	6	2.80E-06	2.65E-06	1.82E-06	2.25E-06
F42G2.6	38	30	24	30	2.80E-06	2.75E-06	2.30E-06	2.25E-06
F42G2.7	1	4	0	2	2.80E-06	2.65E-06	1.82E-06	2.25E-06
F42G2.8	15	20	15	11	2.80E-06	2.65E-06	1.82E-06	2.25E-06
F42G2.t1	0	1	0	0	2.80E-06	2.65E-06	1.82E-06	2.25E-06
F42G4.2	38	58	29	19	2.80E-06	2.65E-06	1.82E-06	2.25E-06
F42G4.3a.1	960	1140	1001	1526	2.80E-06	2.65E-06	1.82E-06	2.25E-06
F42G4.3a.2	779	857	743	1179	3.19E-06	4.60E-06	1.82E-06	2.25E-06
F42G4.3b.1	386	479	376	532	4.51E-05	5.06E-05	3.06E-05	5.76E-05
F42G4.3b.2	332	422	333	494	4.67E-05	4.85E-05	2.90E-05	5.67E-05
F42G4.5	33	52	36	47	4.91E-05	5.76E-05	3.11E-05	5.44E-05
F42G4.6	27	85	21	20	4.75E-05	5.71E-05	3.10E-05	5.68E-05
F42G4.7	30	33	101	48	4.23E-06	6.27E-06	2.99E-06	4.84E-06
F42G8.10a	326	578	386	366	3.61E-06	1.07E-05	1.82E-06	2.25E-06
F42G8.10b.1	408	707	524	450	8.96E-06	9.31E-06	1.96E-05	1.15E-05
F42G8.10b.2	312	534	300	359	6.26E-05	1.05E-04	4.83E-05	5.65E-05
F42G8.10b.3	326	578	386	366	6.57E-05	1.08E-04	5.50E-05	5.82E-05
F42G8.11	62	147	71	76	5.67E-05	9.17E-05	3.55E-05	5.24E-05
F42G8.12.1	1950	2304	2014	2131	6.26E-05	1.05E-04	4.83E-05	5.65E-05
F42G8.12.2	1535	1840	1482	1712	7.59E-06	1.70E-05	5.65E-06	7.47E-06
F42G8.3a	31	58	38	43	1.80E-04	2.01E-04	1.21E-04	1.58E-04
F42G8.3b	30	58	29	42	2.03E-04	2.30E-04	1.28E-04	1.82E-04
F42G8.4.1	82	148	59	118	2.80E-06	3.89E-06	1.82E-06	2.45E-06
F42G8.4.2	76	141	56	112	2.80E-06	4.05E-06	1.82E-06	2.50E-06
F42G8.5	36	53	30	45	4.98E-06	8.49E-06	2.33E-06	5.76E-06
F42G8.6	129	170	145	193	4.79E-06	8.38E-06	2.30E-06	5.67E-06
F42G8.7	26	35	25	15	3.75E-06	5.24E-06	2.04E-06	3.78E-06
F42G8.8	36	74	36	28	1.16E-05	1.44E-05	8.45E-06	1.39E-05
F42G8.9	17	44	14	15	2.80E-06	2.65E-06	1.82E-06	2.25E-06
F42G9.1a	611	555	945	1341	2.80E-06	5.34E-06	1.82E-06	2.25E-06
F42G9.1b	429	401	692	1102	2.80E-06	3.86E-06	1.82E-06	2.25E-06

F42G9.2	212	283	125	87	3.53E-05	3.03E-05	3.56E-05	6.23E-05
F42G9.4	7	10	12	4	3.28E-05	2.89E-05	3.44E-05	6.76E-05
F42G9.5a	211	283	285	396	3.34E-05	4.21E-05	1.28E-05	1.10E-05
F42G9.5b.1	195	267	245	360	2.80E-06	2.65E-06	1.82E-06	2.25E-06
F42G9.5b.2	191	261	243	353	1.10E-05	1.39E-05	9.66E-06	1.66E-05
F42G9.6a	412	403	593	826	1.39E-05	1.79E-05	1.13E-05	2.06E-05
F42G9.6b	483	480	708	968	1.41E-05	1.82E-05	1.17E-05	2.09E-05
F42G9.6c	385	387	565	822	2.33E-05	2.15E-05	2.18E-05	3.75E-05
F42G9.7	149	93	267	180	2.14E-05	2.01E-05	2.04E-05	3.45E-05
F42G9.8	1	1	7	2	2.18E-05	2.07E-05	2.08E-05	3.74E-05
F42G9.9a.1	49	70	41	39	8.82E-06	5.21E-06	1.03E-05	8.57E-06
F42G9.9a.2	32	45	16	28	2.80E-06	2.65E-06	1.82E-06	2.25E-06
F42G9.9b.1	32	42	17	29	3.64E-06	4.92E-06	1.99E-06	2.34E-06
F42G9.9b.2	32	40	15	29	2.80E-06	3.49E-06	1.82E-06	2.25E-06
F42G9.9c.1	37	45	19	31	2.80E-06	2.75E-06	1.82E-06	2.25E-06
F42G9.9c.2	30	40	15	26	2.80E-06	3.07E-06	1.82E-06	2.25E-06
F42G9.9d.1	47	68	41	38	2.80E-06	2.91E-06	1.82E-06	2.25E-06
F42G9.9d.2	30	40	15	26	2.80E-06	2.67E-06	1.82E-06	2.25E-06
F42H10.2	131	97	231	79	3.58E-06	4.92E-06	2.04E-06	2.34E-06
F42H10.3	131	164	80	133	2.80E-06	3.49E-06	1.82E-06	2.25E-06
F42H10.5	74	127	70	125	4.22E-05	2.95E-05	4.84E-05	2.04E-05
F42H10.6.1	37	54	117	43	1.46E-05	1.72E-05	5.78E-06	1.19E-05
F42H10.6.2	37	60	119	48	2.80E-06	4.39E-06	1.82E-06	3.69E-06
F42H10.7a	318	316	393	622	6.86E-06	9.47E-06	1.41E-05	6.41E-06
F42H10.7b	324	322	405	628	7.08E-06	1.08E-05	1.48E-05	7.38E-06
F42H10.9	78	53	61	91	2.13E-05	2.00E-05	1.71E-05	3.34E-05
F42H10.t1	0	0	3	0	2.14E-05	2.01E-05	1.74E-05	3.33E-05
F42H11.1	27	46	14	16	9.38E-06	6.03E-06	4.77E-06	8.79E-06
F42H11.2.1	441	393	657	733	2.80E-06	2.65E-06	2.99E-06	2.25E-06
F43A11.1	6	4	5	1	3.11E-06	5.00E-06	1.82E-06	2.25E-06
F43A11.3	5	7	4	3	2.08E-05	1.75E-05	2.01E-05	2.77E-05
F43A11.4	7	3	6	4	2.80E-06	2.65E-06	1.82E-06	2.25E-06
F43A11.5	5	2	3	1	2.80E-06	2.65E-06	1.82E-06	2.25E-06
F43A11.6	6	11	2	5	2.80E-06	2.65E-06	1.82E-06	2.25E-06
F43A11.7	3	2	3	4	2.80E-06	2.65E-06	1.82E-06	2.25E-06
F43B10.1	11	10	33	47	2.80E-06	2.65E-06	1.82E-06	2.25E-06
F43B10.2a	123	130	76	100	2.80E-06	2.65E-06	1.82E-06	2.25E-06
F43B10.2b	94	88	51	78	2.80E-06	2.65E-06	2.57E-06	4.52E-06
F43C1.1	19	25	34	16	4.17E-06	4.15E-06	1.82E-06	2.72E-06
F43C1.2a.1	937	736	1088	1501	4.54E-06	3.99E-06	1.82E-06	3.01E-06
F43C1.2b	625	504	692	1087	2.80E-06	2.65E-06	1.82E-06	2.25E-06
F43C1.3	96	95	140	83	5.39E-05	4.00E-05	4.07E-05	6.94E-05
F43C1.5	22	22	14	14	3.56E-05	2.71E-05	2.56E-05	4.97E-05
F43C1.6.1	142	170	119	120	1.64E-05	1.53E-05	1.55E-05	1.14E-05
F43C1.6.2	123	143	90	102	3.70E-06	3.49E-06	1.82E-06	2.25E-06
F43C1.7	1	2	1	2	2.62E-05	2.96E-05	1.43E-05	1.78E-05
F43C11.1	1	2	1	1	2.24E-05	2.47E-05	1.07E-05	1.49E-05
F43C11.10	1	5	5	2	2.80E-06	2.65E-06	1.82E-06	2.25E-06
F43C11.11	0	1	1	1	2.80E-06	2.65E-06	1.82E-06	2.25E-06
F43C11.12	1	1	0	0	2.80E-06	2.65E-06	1.82E-06	2.25E-06
F43C11.2	0	2	0	3	2.80E-06	2.65E-06	1.82E-06	2.25E-06
F43C11.3	85	85	189	42	2.80E-06	2.65E-06	1.82E-06	2.25E-06
F43C11.4	3	2	3	3	2.80E-06	2.65E-06	1.82E-06	2.25E-06
F43C11.5	3	1	0	1	2.28E-05	2.16E-05	3.30E-05	9.06E-06
F43C11.6	6	13	8	8	2.80E-06	2.65E-06	1.82E-06	2.25E-06
F43C11.7	15	42	16	25	2.80E-06	2.65E-06	1.82E-06	2.25E-06
F43C11.8	3	7	56	5	2.80E-06	2.65E-06	1.82E-06	2.25E-06
F43C11.9	58	62	79	96	2.80E-06	4.79E-06	1.82E-06	2.43E-06
F43C9.1	26	49	14	25	2.80E-06	2.65E-06	4.01E-06	2.25E-06

F43C9.2	78	89	86	101	6.02E-06	6.08E-06	5.34E-06	8.01E-06
F43C9.3	11	13	15	11	2.80E-06	4.02E-06	1.82E-06	2.25E-06
F43C9.4a	21	48	32	45	1.16E-05	1.25E-05	8.35E-06	1.21E-05
F43C9.4b	9	27	17	31	2.80E-06	2.65E-06	1.82E-06	2.25E-06
F43C9.t1	1	0	3	0	2.80E-06	3.41E-06	1.82E-06	2.72E-06
F43D2.1	196	260	338	445	2.80E-06	2.65E-06	1.82E-06	2.25E-06
F43D2.2	121	104	86	190	2.80E-06	2.65E-06	2.99E-06	2.25E-06
F43D2.3	9	6	10	3	2.89E-05	3.62E-05	3.25E-05	5.27E-05
F43D2.4	4	21	13	13	1.74E-05	1.42E-05	8.07E-06	2.20E-05
F43D2.6	73	154	124	140	2.80E-06	2.65E-06	1.82E-06	2.25E-06
F43D9.1	108	130	65	124	2.80E-06	3.28E-06	1.82E-06	2.25E-06
F43D9.2	156	177	282	205	1.75E-05	3.48E-05	1.93E-05	2.69E-05
F43D9.4.1	10222	6802	11113	9432	3.05E-06	3.47E-06	1.82E-06	2.81E-06
F43D9.4.2	10155	6734	11034	9349	1.89E-05	2.03E-05	2.22E-05	1.99E-05
F43D9.5	31	48	33	56	1.81E-03	1.14E-03	1.28E-03	1.34E-03
F43E12.1	1	3	1	1	1.84E-03	1.15E-03	1.30E-03	1.36E-03
F43E12.t1	0	1	1	1	5.21E-06	7.62E-06	3.61E-06	7.56E-06
F43E2.1	84	110	141	157	2.80E-06	2.65E-06	1.82E-06	2.25E-06
F43E2.11	0	1	2	2	2.80E-06	2.65E-06	1.82E-06	2.25E-06
F43E2.2	345	447	486	319	5.32E-06	6.56E-06	5.79E-06	7.96E-06
F43E2.3	46	85	36	41	2.80E-06	2.65E-06	1.82E-06	2.25E-06
F43E2.4	648	637	878	1136	3.88E-05	4.74E-05	3.55E-05	2.88E-05
F43E2.6a	10	10	3	6	2.80E-06	4.58E-06	1.82E-06	2.25E-06
F43E2.6b.1	6	11	2	5	2.86E-05	2.65E-05	2.52E-05	4.02E-05
F43E2.6b.2	7	11	2	6	2.97E-06	2.80E-06	1.82E-06	2.25E-06
F43E2.7a	640	865	681	958	2.80E-06	3.33E-06	1.82E-06	2.25E-06
F43E2.7b	1008	1379	1002	1415	2.80E-06	3.15E-06	1.82E-06	2.25E-06
F43E2.8.1	2843	2746	3116	3561	8.75E-05	1.12E-04	6.06E-05	1.05E-04
F43E2.8.2	2692	2604	2922	3395	8.39E-05	1.08E-04	5.43E-05	9.46E-05
F43E2.9	3	3	0	3	1.44E-04	1.31E-04	1.02E-04	1.45E-04
F43G6.10	67	38	204	53	1.49E-04	1.37E-04	1.06E-04	1.51E-04
F43G6.11a	58	93	80	68	2.80E-06	2.65E-06	1.82E-06	2.25E-06
F43G6.11b	50	72	60	52	1.97E-05	1.06E-05	3.91E-05	1.25E-05
F43G6.1a	548	510	892	1144	4.14E-06	6.30E-06	3.72E-06	3.91E-06
F43G6.1b	520	483	818	1091	3.67E-06	5.00E-06	2.88E-06	3.08E-06
F43G6.2	149	115	275	90	1.81E-05	1.59E-05	1.92E-05	3.04E-05
F43G6.3	61	76	117	109	1.81E-05	1.59E-05	1.86E-05	3.06E-05
F43G6.4	41	63	52	70	4.12E-05	3.00E-05	4.95E-05	2.00E-05
F43G6.5	9	15	16	10	8.93E-06	1.05E-05	1.12E-05	1.28E-05
F43G6.6	18	33	14	7	4.17E-06	6.06E-06	3.44E-06	5.71E-06
F43G6.7	41	42	236	45	2.80E-06	2.65E-06	1.82E-06	2.25E-06
F43G6.8	26	61	26	28	2.80E-06	2.65E-06	1.82E-06	2.25E-06
F43G6.9.1	2787	2247	4897	5872	1.22E-05	1.18E-05	4.56E-05	1.07E-05
F43G6.9.2	2359	1898	4121	5010	3.39E-06	7.49E-06	2.20E-06	2.92E-06
F43G9.1.1	1620	1654	1616	1973	1.08E-04	8.23E-05	1.24E-04	1.83E-04
F43G9.1.2	1131	1191	1166	1387	1.05E-04	7.95E-05	1.19E-04	1.79E-04
F43G9.10	645	652	419	703	1.12E-04	1.09E-04	7.30E-05	1.10E-04
F43G9.11	2	3	4	5	1.17E-04	1.16E-04	7.83E-05	1.15E-04
F43G9.12	365	491	318	537	4.89E-05	4.67E-05	2.07E-05	4.28E-05
F43G9.13.1	192	175	203	209	2.80E-06	2.65E-06	1.82E-06	2.25E-06
F43G9.13.2	165	145	152	155	1.55E-05	1.97E-05	8.78E-06	1.83E-05
F43G9.3	329	308	299	403	4.37E-05	3.76E-05	3.01E-05	3.82E-05
F43G9.4	227	248	251	279	3.94E-05	3.27E-05	2.36E-05	2.97E-05
F43G9.5	311	302	489	559	4.05E-05	3.58E-05	2.39E-05	3.98E-05
F43G9.6	92	129	64	31	2.38E-05	2.46E-05	1.71E-05	2.35E-05
F43G9.8	5	18	5	8	4.51E-05	4.13E-05	4.61E-05	6.51E-05
F43H9.1	48	52	26	41	2.80E-06	2.65E-06	1.82E-06	2.25E-06
F43H9.2a	164	201	192	268	2.80E-06	2.65E-06	1.82E-06	2.25E-06
F43H9.2b	168	209	204	279	5.91E-06	6.03E-06	2.08E-06	4.05E-06

F43H9.3	140	156	120	161	1.08E-05	1.25E-05	8.22E-06	1.41E-05
F43H9.4	54	212	51	113	9.91E-06	1.16E-05	7.82E-06	1.32E-05
F44A2.1a	852	743	1093	1525	1.18E-05	1.24E-05	6.58E-06	1.09E-05
F44A2.1b	1376	1273	1874	2689	6.55E-06	2.43E-05	4.03E-06	1.10E-05
F44A2.2	12	9	4	8	5.04E-05	4.15E-05	4.21E-05	7.24E-05
F44A2.3	43	114	77	84	5.48E-05	4.79E-05	4.86E-05	8.60E-05
F44A2.4	6	6	17	3	2.80E-06	2.65E-06	1.82E-06	2.25E-06
F44A2.5b	115	173	133	112	2.80E-06	6.40E-06	2.97E-06	4.00E-06
F44A2.7	4	3	11	2	2.80E-06	2.65E-06	1.82E-06	2.25E-06
F44A2.t1	1	0	0	0	7.31E-06	1.04E-05	5.50E-06	5.71E-06
F44A6.1a	444	535	422	666	2.80E-06	2.65E-06	1.82E-06	2.25E-06
F44A6.1b	449	552	447	677	2.80E-06	2.65E-06	1.82E-06	2.25E-06
F44A6.2.1	175	146	176	278	3.16E-05	3.60E-05	1.96E-05	3.81E-05
F44A6.2.2	175	146	174	277	3.02E-05	3.51E-05	1.96E-05	3.66E-05
F44A6.3	1	3	1	1	1.03E-05	8.15E-06	6.76E-06	1.32E-05
F44A6.4	48	99	41	40	1.04E-05	8.20E-06	6.74E-06	1.32E-05
F44A6.5	40	278	46	69	2.80E-06	2.65E-06	1.82E-06	2.25E-06
F44B9.10	6	15	4	3	1.14E-05	2.22E-05	6.34E-06	7.65E-06
F44B9.1a	89	121	84	32	4.82E-06	3.17E-05	3.61E-06	6.68E-06
F44B9.1b	86	114	75	27	2.80E-06	2.70E-06	1.82E-06	2.25E-06
F44B9.1c	71	88	66	22	4.00E-06	5.13E-06	2.46E-06	2.25E-06
F44B9.2	156	212	116	146	4.20E-06	5.26E-06	2.39E-06	2.25E-06
F44B9.3a	379	400	521	711	4.59E-06	5.40E-06	2.79E-06	2.25E-06
F44B9.3b	317	330	454	629	1.13E-05	1.45E-05	5.47E-06	8.48E-06
F44B9.4a	389	330	428	631	2.02E-05	2.01E-05	1.80E-05	3.04E-05
F44B9.4b	269	229	307	436	2.11E-05	2.08E-05	1.97E-05	3.37E-05
F44B9.5.1	260	334	217	304	2.21E-05	1.77E-05	1.58E-05	2.88E-05
F44B9.5.2	221	280	198	263	2.25E-05	1.81E-05	1.67E-05	2.93E-05
F44B9.6	1141	800	1376	1643	2.33E-05	2.83E-05	1.26E-05	2.19E-05
F44B9.7	297	290	321	466	2.11E-05	2.52E-05	1.23E-05	2.02E-05
F44B9.8	548	589	590	605	3.80E-05	2.52E-05	2.98E-05	4.39E-05
F44B9.9	2	10	2	1	2.28E-05	2.10E-05	1.60E-05	2.87E-05
F44C4.2	5	9	586	79	4.77E-05	4.84E-05	3.34E-05	4.23E-05
F44C4.4a	867	785	1197	1699	2.80E-06	2.65E-06	1.82E-06	2.25E-06
F44C4.4b.1	870	793	1216	1701	2.80E-06	2.65E-06	3.31E-05	5.51E-06
F44C4.5	8	16	10	20	3.10E-05	2.65E-05	2.79E-05	4.88E-05
F44C4.6	1	4	2	4	3.15E-05	2.71E-05	2.86E-05	4.94E-05
F44C8.1	16	36	24	22	2.80E-06	2.65E-06	1.82E-06	2.25E-06
F44C8.10	5	21	18	22	2.80E-06	2.65E-06	1.82E-06	2.25E-06
F44C8.11	16	25	6	16	2.80E-06	2.65E-06	1.82E-06	2.25E-06
F44C8.3	11	29	20	23	2.80E-06	2.65E-06	1.82E-06	2.25E-06
F44C8.4	16	17	13	17	2.80E-06	2.65E-06	1.82E-06	2.25E-06
F44C8.5a	4	6	6	6	2.80E-06	2.65E-06	1.82E-06	2.25E-06
F44C8.5b	21	19	24	16	2.80E-06	2.65E-06	1.82E-06	2.25E-06
F44C8.6a	13	29	42	28	2.80E-06	2.65E-06	1.82E-06	2.25E-06
F44C8.6b	11	27	41	28	2.80E-06	2.65E-06	1.82E-06	2.25E-06
F44C8.7	8	8	5	4	2.80E-06	2.65E-06	2.22E-06	2.25E-06
F44C8.8	21	36	35	33	2.80E-06	2.65E-06	2.28E-06	2.25E-06
F44C8.9a	20	16	10	8	2.80E-06	2.65E-06	1.82E-06	2.25E-06
F44C8.9b	3	0	5	7	2.80E-06	2.96E-06	1.99E-06	2.32E-06
F44D12.1	131	174	77	175	2.80E-06	2.65E-06	1.82E-06	2.25E-06
F44D12.10	1	3	2	0	2.80E-06	2.65E-06	1.82E-06	2.25E-06
F44D12.11	4	5	1	4	4.62E-06	5.79E-06	1.82E-06	4.95E-06
F44D12.2	50	99	54	76	2.80E-06	2.65E-06	1.82E-06	2.25E-06
F44D12.3	329	447	490	105	2.80E-06	2.65E-06	1.82E-06	2.25E-06
F44D12.4	185	500	173	115	4.70E-06	8.81E-06	3.32E-06	5.76E-06
F44D12.6	14	23	13	8	8.97E-05	1.15E-04	8.69E-05	2.30E-05
F44D12.7	223	338	179	62	1.84E-05	4.70E-05	1.12E-05	9.20E-06
F44D12.8	22	56	18	19	2.80E-06	2.78E-06	1.82E-06	2.25E-06

F44D12.9.1	76	166	83	139	7.57E-05	1.08E-04	3.95E-05	1.69E-05
F44D12.9.2	72	163	83	142	2.80E-06	4.42E-06	1.82E-06	2.25E-06
F44E2.10	180	260	240	237	5.29E-06	1.10E-05	3.77E-06	7.78E-06
F44E2.2a	2272	1688	1957	3239	5.96E-06	1.27E-05	4.46E-06	9.42E-06
F44E2.2b	1790	1175	1516	2738	2.20E-05	3.01E-05	1.91E-05	2.33E-05
F44E2.2c	1520	1196	1602	2178	3.89E-05	2.73E-05	2.18E-05	4.45E-05
F44E2.3	131	156	112	143	3.05E-05	1.89E-05	1.68E-05	3.75E-05
F44E2.4	416	766	359	366	3.00E-04	2.23E-04	2.06E-04	3.46E-04
F44E2.6	61	75	204	90	1.79E-05	2.01E-05	9.95E-06	1.57E-05
F44E2.6b	62	77	213	93	1.11E-05	1.93E-05	6.23E-06	7.83E-06
F44E2.7a	337	366	414	607	1.49E-05	1.73E-05	3.24E-05	1.76E-05
F44E2.7b.1	289	321	354	530	1.46E-05	1.72E-05	3.27E-05	1.77E-05
F44E2.7b.2	249	294	314	461	2.28E-05	2.34E-05	1.82E-05	3.29E-05
F44E2.7b.3	268	297	315	471	2.34E-05	2.45E-05	1.86E-05	3.44E-05
F44E2.7b.4	306	332	385	560	2.25E-05	2.51E-05	1.85E-05	3.34E-05
F44E2.7b.5	309	341	394	572	2.11E-05	2.21E-05	1.61E-05	2.97E-05
F44E2.7c	249	294	312	461	2.40E-05	2.46E-05	1.97E-05	3.53E-05
F44E2.7d	306	332	385	560	2.42E-05	2.52E-05	2.01E-05	3.59E-05
F44E2.8	448	494	547	648	2.26E-05	2.52E-05	1.84E-05	3.36E-05
F44E2.9	125	140	379	130	2.40E-05	2.46E-05	1.97E-05	3.53E-05
F44E5.1.1	1624	1177	5694	1063	5.61E-05	5.84E-05	4.46E-05	6.51E-05
F44E5.1.2	1376	1015	3830	837	2.88E-05	3.04E-05	5.67E-05	2.40E-05
F44E5.2	5	11	29	6	4.84E-04	3.31E-04	1.10E-03	2.54E-04
F44E5.3	4	10	19	3	4.29E-04	2.99E-04	7.78E-04	2.10E-04
F44E7.2	345	955	455	715	2.80E-06	2.65E-06	1.82E-06	2.25E-06
F44E7.3	9	10	2	2	2.80E-06	2.65E-06	1.82E-06	2.25E-06
F44E7.4a	2411	2401	3034	4040	3.19E-05	8.34E-05	2.74E-05	5.31E-05
F44E7.4b	2328	2315	2925	3969	4.93E-06	5.18E-06	1.82E-06	2.25E-06
F44E7.4c.1	2319	2303	2904	3944	8.01E-05	7.53E-05	6.56E-05	1.08E-04
F44E7.4c.2	2261	2251	2838	3886	8.24E-05	7.74E-05	6.74E-05	1.13E-04
F44E7.4d	2137	2094	2736	3683	8.08E-05	7.58E-05	6.59E-05	1.10E-04
F44E7.5a	171	218	297	405	8.18E-05	7.69E-05	6.68E-05	1.13E-04
F44E7.5b	172	219	294	401	8.08E-05	7.48E-05	6.73E-05	1.12E-04
F44E7.6	11	16	26	9	1.37E-05	1.66E-05	1.55E-05	2.62E-05
F44E7.7	9	15	16	17	1.38E-05	1.66E-05	1.53E-05	2.58E-05
F44E7.8	11	25	9	14	2.80E-06	2.65E-06	1.91E-06	2.25E-06
F44E7.9	193	275	446	189	2.80E-06	2.65E-06	1.82E-06	2.25E-06
F44F1.1	45	37	12	18	2.80E-06	2.65E-06	1.82E-06	2.25E-06
F44F1.3	17	39	9	15	5.91E-05	7.95E-05	8.88E-05	4.65E-05
F44F1.4	18	16	12	14	2.80E-06	2.65E-06	1.82E-06	2.25E-06
F44F1.5	14	21	15	9	2.80E-06	2.65E-06	1.82E-06	2.25E-06
F44F1.6a	10	11	6	14	2.80E-06	2.65E-06	1.82E-06	2.25E-06
F44F1.6b	2	2	3	3	2.80E-06	2.65E-06	1.82E-06	2.25E-06
F44F1.7	45	39	27	35	2.80E-06	2.65E-06	1.82E-06	2.25E-06
F44F4.1	13	30	5	12	2.80E-06	2.65E-06	1.82E-06	2.25E-06
F44F4.10	18	19	5	7	2.80E-06	2.65E-06	1.82E-06	2.25E-06
F44F4.11	543	790	605	658	2.80E-06	5.74E-06	1.82E-06	2.25E-06
F44F4.13	4	3	5	3	2.80E-06	2.65E-06	1.82E-06	2.25E-06
F44F4.2.1	648	728	1115	1136	4.33E-05	5.95E-05	3.14E-05	4.21E-05
F44F4.2.2	590	661	956	1047	2.80E-06	2.65E-06	1.82E-06	2.25E-06
F44F4.3	10	9	7	9	4.03E-05	4.27E-05	4.51E-05	5.67E-05
F44F4.4	98	313	68	163	3.96E-05	4.19E-05	4.17E-05	5.64E-05
F44F4.5a	3	9	2	2	2.80E-06	2.65E-06	1.82E-06	2.25E-06
F44F4.5b	4	8	2	2	3.95E-06	1.19E-05	1.82E-06	5.26E-06
F44F4.6	16	15	15	9	2.80E-06	2.65E-06	1.82E-06	2.25E-06
F44F4.7	16	15	15	9	2.80E-06	2.65E-06	1.82E-06	2.25E-06
F44F4.9	4	6	6	0	2.80E-06	2.65E-06	1.82E-06	2.25E-06
F44G3.1	5	2	4	2	2.80E-06	2.65E-06	1.82E-06	2.25E-06
F44G3.10	4	4	2	2	2.80E-06	2.65E-06	1.82E-06	2.25E-06

F44G3.11	9	3	7	1	2.80E-06	2.65E-06	1.82E-06	2.25E-06
F44G3.12	3	6	12	2	2.80E-06	2.65E-06	1.82E-06	2.25E-06
F44G3.13	3	6	4	3	2.80E-06	2.65E-06	1.82E-06	2.25E-06
F44G3.14	6	13	29	16	2.80E-06	2.65E-06	1.82E-06	2.25E-06
F44G3.2	244	342	154	146	2.80E-06	2.65E-06	1.82E-06	2.25E-06
F44G3.3	22	21	31	31	2.80E-06	2.65E-06	1.82E-06	2.25E-06
F44G3.4	5	2	8	2	2.26E-05	2.99E-05	9.29E-06	1.09E-05
F44G3.5	3	2	6	4	2.80E-06	2.65E-06	1.82E-06	2.25E-06
F44G3.7	31	97	22	21	2.80E-06	2.65E-06	1.82E-06	2.25E-06
F44G3.8	3	6	12	6	2.80E-06	2.65E-06	1.82E-06	2.25E-06
F44G3.9	3	0	2	6	2.80E-06	4.84E-06	1.82E-06	2.25E-06
F44G4.1	412	511	315	583	2.80E-06	2.65E-06	1.82E-06	2.25E-06
F44G4.2.1	597	786	1055	544	2.80E-06	2.65E-06	1.82E-06	2.25E-06
F44G4.2.2	551	734	776	457	3.47E-05	4.06E-05	1.73E-05	3.94E-05
F44G4.3	86	78	133	79	1.06E-04	1.32E-04	1.22E-04	7.74E-05
F44G4.4a	435	369	539	754	1.01E-04	1.26E-04	9.21E-05	6.70E-05
F44G4.4b	132	112	171	164	2.92E-05	2.50E-05	2.94E-05	2.15E-05
F44G4.5	15	33	12	14	3.41E-05	2.74E-05	2.75E-05	4.75E-05
F44G4.6	4	5	7	2	3.55E-05	2.85E-05	3.00E-05	3.55E-05
F44G4.7	5	6	7	8	3.11E-06	6.45E-06	1.82E-06	2.34E-06
F44G4.8	146	248	158	200	2.80E-06	2.65E-06	1.82E-06	2.25E-06
F45B8.1	63	74	88	101	2.80E-06	2.65E-06	1.82E-06	2.25E-06
F45B8.2	5	15	17	26	3.64E-06	5.85E-06	2.57E-06	4.03E-06
F45B8.3	10	10	5	10	6.97E-06	7.72E-06	6.32E-06	8.95E-06
F45B8.4	6	8	6	6	2.80E-06	2.65E-06	1.82E-06	2.88E-06
F45C12.1	4	8	8	2	2.80E-06	2.65E-06	1.82E-06	2.25E-06
F45C12.10a	2	2	10	4	2.80E-06	2.65E-06	1.82E-06	2.25E-06
F45C12.10b	3	4	10	4	2.80E-06	2.65E-06	1.82E-06	2.25E-06
F45C12.11	1	7	3	0	2.80E-06	2.65E-06	1.82E-06	2.25E-06
F45C12.12	11	18	22	26	2.80E-06	2.65E-06	1.82E-06	2.25E-06
F45C12.13	6	6	7	4	2.80E-06	2.65E-06	1.82E-06	2.25E-06
F45C12.14	1	1	1	2	2.80E-06	2.65E-06	1.95E-06	2.86E-06
F45C12.16	7	8	6	4	2.80E-06	2.65E-06	1.82E-06	2.25E-06
F45C12.2	5	8	6	5	2.80E-06	2.65E-06	1.82E-06	2.25E-06
F45C12.3	5	11	4	9	2.80E-06	2.65E-06	1.82E-06	2.25E-06
F45C12.4	15	15	24	9	2.80E-06	2.65E-06	1.82E-06	2.25E-06
F45C12.5	3	6	8	6	2.80E-06	2.65E-06	1.82E-06	2.25E-06
F45C12.6	7	5	5	4	2.80E-06	2.65E-06	2.00E-06	2.25E-06
F45C12.7	51	59	60	48	2.80E-06	2.65E-06	1.82E-06	2.25E-06
F45C12.8	9	7	5	11	2.80E-06	2.65E-06	1.82E-06	2.25E-06
F45C12.9	0	0	2	1	4.90E-06	5.34E-06	3.74E-06	3.69E-06
F45D11.1.1	43	19	25	6	2.80E-06	2.65E-06	1.82E-06	2.25E-06
F45D11.1.2	45	19	25	6	2.80E-06	2.65E-06	1.82E-06	2.25E-06
F45D11.10	5	12	21	12	2.88E-06	2.65E-06	1.82E-06	2.25E-06
F45D11.12	4	5	34	14	2.97E-06	2.65E-06	1.82E-06	2.25E-06
F45D11.13	24	18	9	10	2.80E-06	2.65E-06	1.82E-06	2.25E-06
F45D11.14	330	41	577	9	2.80E-06	2.65E-06	3.66E-06	2.25E-06
F45D11.15	320	39	536	9	2.91E-06	2.65E-06	1.82E-06	2.25E-06
F45D11.16	320	39	536	9	2.42E-05	2.83E-06	2.75E-05	2.25E-06
F45D11.2	24	5	33	4	2.38E-05	2.75E-06	2.59E-05	2.25E-06
F45D11.3	24	5	33	4	2.38E-05	2.75E-06	2.59E-05	2.25E-06
F45D11.4	24	5	33	4	2.80E-06	2.65E-06	2.17E-06	2.25E-06
F45D11.5	1	0	10	4	2.80E-06	2.65E-06	2.17E-06	2.25E-06
F45D11.6	1	4	4	2	2.80E-06	2.65E-06	2.17E-06	2.25E-06
F45D11.8	5	1	5	3	2.80E-06	2.65E-06	1.82E-06	2.25E-06
F45D11.9	14	21	32	36	2.80E-06	2.65E-06	1.82E-06	2.25E-06
F45D3.1	10	10	14	4	2.80E-06	2.65E-06	1.82E-06	2.25E-06
F45D3.3	277	765	189	363	2.80E-06	2.67E-06	2.82E-06	3.91E-06
F45D3.4a	242	964	126	340	2.80E-06	2.65E-06	2.10E-06	2.25E-06

F45D3.4b	175	660	109	275	3.04E-05	7.93E-05	1.35E-05	3.20E-05
F45E1.1	19	21	7	13	2.81E-05	1.06E-04	9.53E-06	3.17E-05
F45E1.2	2	3	1	0	2.53E-05	9.00E-05	1.02E-05	3.19E-05
F45E1.3	43	108	29	19	7.62E-06	7.96E-06	1.82E-06	4.18E-06
F45E1.4	10	24	12	5	2.80E-06	2.65E-06	1.82E-06	2.25E-06
F45E1.5	15	26	12	14	6.22E-06	1.48E-05	2.73E-06	2.25E-06
F45E1.6.1	112	224	220	91	5.66E-06	1.28E-05	4.41E-06	2.27E-06
F45E1.6.2	100	205	172	86	2.80E-06	2.65E-06	1.82E-06	2.25E-06
F45E1.7a.1	85	119	61	108	2.12E-05	4.00E-05	2.71E-05	1.38E-05
F45E1.7a.2	85	119	60	106	1.90E-05	3.67E-05	2.12E-05	1.31E-05
F45E1.7b	99	149	67	122	6.38E-06	8.44E-06	2.99E-06	6.52E-06
F45E10.1a	218	289	206	244	6.47E-06	8.57E-06	2.97E-06	6.48E-06
F45E10.1b	206	274	184	234	6.22E-06	8.83E-06	2.73E-06	6.16E-06
F45E10.1c	219	287	207	246	4.87E-06	6.08E-06	2.99E-06	4.36E-06
F45E10.1d	212	272	192	239	4.93E-06	6.19E-06	2.86E-06	4.50E-06
F45E10.1e	173	230	153	189	4.70E-06	5.82E-06	2.88E-06	4.23E-06
F45E10.2a	28	38	28	34	4.73E-06	5.74E-06	2.79E-06	4.30E-06
F45E10.2b	24	36	15	28	5.24E-06	6.56E-06	3.01E-06	4.59E-06
F45E12.1	363	362	447	592	2.80E-06	2.65E-06	1.82E-06	2.25E-06
F45E12.2	533	470	685	929	2.80E-06	2.65E-06	1.82E-06	2.25E-06
F45E12.3	432	453	569	910	3.20E-05	3.01E-05	2.56E-05	4.19E-05
F45E12.5a	114	142	144	112	2.35E-05	1.96E-05	1.96E-05	3.29E-05
F45E12.5b.1	194	241	237	206	1.75E-05	1.74E-05	1.50E-05	2.96E-05
F45E12.5b.2	174	193	196	188	2.15E-05	2.53E-05	1.77E-05	1.70E-05
F45E12.6	1	3	2	0	3.79E-05	4.44E-05	3.01E-05	3.23E-05
F45E4.1	15	19	22	7	3.45E-05	3.61E-05	2.53E-05	2.99E-05
F45E4.10a	512	500	533	934	2.80E-06	2.65E-06	1.82E-06	2.25E-06
F45E4.10b.1	533	519	555	965	3.11E-06	3.73E-06	2.97E-06	2.25E-06
F45E4.10b.2	529	512	545	963	2.44E-05	2.25E-05	1.65E-05	3.57E-05
F45E4.11	45	60	74	88	2.43E-05	2.23E-05	1.65E-05	3.53E-05
F45E4.2.1	1183	1653	1487	1737	2.42E-05	2.22E-05	1.63E-05	3.54E-05
F45E4.2.2	842	1210	1042	1279	2.91E-06	3.65E-06	3.12E-06	4.57E-06
F45E4.3a	70	81	78	55	1.38E-04	1.82E-04	1.13E-04	1.62E-04
F45E4.3b.1	69	54	49	47	1.26E-04	1.70E-04	1.01E-04	1.53E-04
F45E4.3b.2	58	50	45	42	2.80E-06	2.65E-06	1.82E-06	2.25E-06
F45E4.4	74	92	72	84	2.80E-06	2.65E-06	1.82E-06	2.25E-06
F45E4.5	28	82	49	40	2.80E-06	2.65E-06	1.82E-06	2.25E-06
F45E4.6	13	36	9	10	2.80E-06	2.65E-06	1.82E-06	2.25E-06
F45E4.7a	16	37	20	13	1.02E-05	2.82E-05	1.16E-05	1.17E-05
F45E4.7b	4	18	11	4	2.80E-06	3.94E-06	1.82E-06	2.25E-06
F45E4.7c	13	28	19	10	2.80E-06	2.65E-06	1.82E-06	2.25E-06
F45E4.8.1	77	148	130	45	2.80E-06	2.65E-06	1.82E-06	2.25E-06
F45E4.8.2	68	122	96	40	2.80E-06	2.65E-06	1.82E-06	2.25E-06
F45E4.9	281	340	264	346	1.72E-05	3.13E-05	1.89E-05	8.07E-06
F45E6.1	4	2	0	2	1.56E-05	2.65E-05	1.43E-05	7.38E-06
F45E6.2	116	143	124	195	5.03E-05	5.75E-05	3.07E-05	4.97E-05
F45E6.3	50	62	70	69	2.80E-06	2.65E-06	1.82E-06	2.25E-06
F45E6.4	8	11	7	11	5.46E-06	6.35E-06	3.79E-06	7.35E-06
F45E6.6	1	4	1	2	3.22E-06	3.76E-06	2.92E-06	3.55E-06
F45F2.1	5	7	5	1	2.80E-06	2.65E-06	1.82E-06	2.25E-06
F45F2.10	1582	1330	1749	2700	2.80E-06	2.65E-06	1.82E-06	2.25E-06
F45F2.11	535	365	790	1011	2.80E-06	2.65E-06	1.82E-06	2.25E-06
F45F2.12	14	39	8	11	4.81E-05	3.82E-05	3.46E-05	6.59E-05
F45F2.13	32	28	22	21	4.92E-05	3.17E-05	4.72E-05	7.46E-05
F45F2.2	9	24	7	5	4.23E-06	1.11E-05	1.82E-06	2.65E-06
F45F2.3	14	36	176	19	8.71E-06	7.22E-06	3.90E-06	4.59E-06
F45F2.4	36	59	32	10	3.08E-06	7.78E-06	1.82E-06	2.25E-06
F45F2.5	14	10	15	10	4.14E-06	1.01E-05	3.39E-05	4.52E-06
F45F2.6	9	12	11	6	1.05E-05	1.63E-05	6.07E-06	2.34E-06

F45F2.7	5	15	13	13	2.80E-06	2.65E-06	1.82E-06	2.25E-06
F45F2.9.1	57	82	99	60	2.80E-06	2.65E-06	1.82E-06	2.25E-06
F45F2.9.2	24	33	35	43	2.80E-06	2.65E-06	1.82E-06	2.25E-06
F45G2.1	11	8	3	4	1.17E-05	1.59E-05	1.32E-05	9.90E-06
F45G2.10	94	127	168	129	6.97E-06	9.07E-06	6.63E-06	1.01E-05
F45G2.2a	100	142	111	112	2.80E-06	2.65E-06	1.82E-06	2.25E-06
F45G2.2b.1	31	46	36	40	2.18E-05	2.78E-05	2.53E-05	2.40E-05
F45G2.2b.2	27	40	29	36	2.86E-06	3.84E-06	2.06E-06	2.56E-06
F45G2.3	293	294	345	511	2.80E-06	2.88E-06	1.82E-06	2.25E-06
F45G2.5	6	30	5	12	2.80E-06	3.25E-06	1.82E-06	2.47E-06
F45G2.6	4	5	0	1	1.59E-05	1.51E-05	1.22E-05	2.23E-05
F45G2.7	20	43	58	35	2.80E-06	5.21E-06	1.82E-06	2.25E-06
F45G2.8.1	149	170	119	113	2.80E-06	2.65E-06	1.82E-06	2.25E-06
F45G2.8.2	132	157	97	101	3.98E-06	8.07E-06	7.49E-06	5.58E-06
F45G2.9	18	45	58	38	3.00E-05	3.23E-05	1.56E-05	1.83E-05
F45G2.t1	0	0	1	0	2.54E-05	2.85E-05	1.21E-05	1.56E-05
F45H10.1.1	61	107	195	204	2.97E-06	7.04E-06	6.25E-06	5.06E-06
F45H10.1.2	57	102	176	200	2.80E-06	2.65E-06	1.82E-06	2.25E-06
F45H10.2.1	692	728	1632	486	3.98E-06	6.56E-06	8.24E-06	1.06E-05
F45H10.2.2	613	671	1066	363	3.70E-06	6.27E-06	7.45E-06	1.05E-05
F45H10.3.1	481	418	1113	491	1.85E-04	1.84E-04	2.84E-04	1.04E-04
F45H10.3.2	397	357	894	466	1.49E-04	1.54E-04	1.69E-04	7.10E-05
F45H10.4	45	61	90	39	9.19E-05	7.55E-05	1.38E-04	7.54E-05
F45H10.5	6	16	10	2	8.13E-05	6.91E-05	1.19E-04	7.66E-05
F45H11.1a	19	19	6	17	7.73E-06	9.89E-06	1.01E-05	5.38E-06
F45H11.1b	21	27	9	19	2.80E-06	3.07E-06	1.82E-06	2.25E-06
F45H11.2	947	629	909	325	2.80E-06	2.65E-06	1.82E-06	2.25E-06
F45H11.3	793	648	1020	1577	2.80E-06	2.65E-06	1.82E-06	2.25E-06
F45H11.4	70	110	71	79	2.51E-04	1.58E-04	1.57E-04	6.93E-05
F45H11.5	132	112	213	85	4.01E-05	3.09E-05	3.35E-05	6.40E-05
F45H7.1	29	30	28	35	2.80E-06	2.83E-06	1.82E-06	2.25E-06
F45H7.2a	92	113	98	152	4.21E-05	3.38E-05	4.42E-05	2.18E-05
F45H7.2b	52	64	59	116	2.80E-06	2.65E-06	1.82E-06	2.50E-06
F45H7.4.1	32	57	38	57	3.50E-06	4.07E-06	2.42E-06	4.66E-06
F45H7.4.2	25	43	21	48	2.80E-06	3.12E-06	1.99E-06	4.81E-06
F45H7.6	37	51	18	25	2.80E-06	3.39E-06	1.82E-06	2.88E-06
F45H7.t1	1	0	0	0	2.80E-06	3.20E-06	1.82E-06	3.04E-06
F46A8.1	3	3	7	3	2.80E-06	2.65E-06	1.82E-06	2.25E-06
F46A8.10	8	22	5	4	2.80E-06	2.65E-06	1.82E-06	2.25E-06
F46A8.11	10	17	9	8	2.80E-06	2.65E-06	1.82E-06	2.25E-06
F46A8.2	3	5	6	2	2.80E-06	2.65E-06	1.82E-06	2.25E-06
F46A8.3	2	2	2	1	2.80E-06	2.65E-06	1.82E-06	2.25E-06
F46A8.4	2	5	6	6	2.80E-06	2.65E-06	1.82E-06	2.25E-06
F46A8.5	5	4	3	1	2.80E-06	2.65E-06	1.82E-06	2.25E-06
F46A8.6	14	17	6	12	2.80E-06	2.65E-06	1.82E-06	2.25E-06
F46A8.7	11	15	28	8	2.80E-06	2.65E-06	1.82E-06	2.25E-06
F46A8.8	4	5	3	1	2.80E-06	2.86E-06	1.82E-06	2.25E-06
F46A8.9	7	9	4	3	1.11E-05	1.43E-05	1.84E-05	6.48E-06
F46A9.1	22	75	19	21	2.80E-06	2.65E-06	1.82E-06	2.25E-06
F46A9.2	12	19	16	9	2.80E-06	2.65E-06	1.82E-06	2.25E-06
F46A9.3a	16	30	28	18	3.44E-06	1.11E-05	1.93E-06	2.65E-06
F46A9.3b	14	31	30	18	2.80E-06	2.65E-06	1.82E-06	2.25E-06
F46A9.4	698	682	1212	993	2.80E-06	2.65E-06	1.82E-06	2.25E-06
F46A9.5.1	984	1125	968	1148	2.80E-06	2.65E-06	1.82E-06	2.25E-06
F46A9.5.2	638	783	577	752	1.09E-04	1.00E-04	1.23E-04	1.24E-04
F46A9.5.3	541	697	526	692	1.27E-04	1.37E-04	8.12E-05	1.19E-04
F46A9.6.1	195	268	273	357	1.24E-04	1.44E-04	7.29E-05	1.17E-04
F46A9.6.2	199	267	275	360	1.11E-04	1.36E-04	7.05E-05	1.14E-04
F46A9.6.3	76	100	122	170	1.37E-05	1.79E-05	1.25E-05	2.02E-05

F46B3.1	0	1	3	1	2.29E-05	2.90E-05	2.06E-05	3.33E-05
F46B3.11	5	3	9	6	5.63E-06	7.01E-06	5.89E-06	1.01E-05
F46B3.12	1	3	10	10	2.80E-06	2.65E-06	1.82E-06	2.25E-06
F46B3.13	13	6	28	18	2.80E-06	2.65E-06	1.82E-06	2.25E-06
F46B3.14	3	1	2	0	2.80E-06	2.65E-06	1.82E-06	2.25E-06
F46B3.15	0	1	0	0	2.80E-06	2.65E-06	3.13E-06	2.50E-06
F46B3.16	2	0	1	0	2.80E-06	2.65E-06	1.82E-06	2.25E-06
F46B3.17	10	13	12	11	2.80E-06	2.65E-06	1.82E-06	2.25E-06
F46B3.2	1	3	5	1	2.80E-06	2.65E-06	1.82E-06	2.25E-06
F46B3.3	4	1	2	5	2.80E-06	2.65E-06	1.82E-06	2.25E-06
F46B3.4	22	28	10	8	2.80E-06	2.65E-06	1.82E-06	2.25E-06
F46B3.5	62	49	19	14	2.80E-06	2.65E-06	1.82E-06	2.25E-06
F46B3.7	0	0	2	1	5.07E-06	6.08E-06	1.82E-06	2.25E-06
F46B3.8	0	1	1	0	2.80E-06	2.65E-06	1.82E-06	2.25E-06
F46B3.9	3	4	1	1	2.80E-06	2.65E-06	1.82E-06	2.25E-06
F46B6.10	3	3	0	1	2.80E-06	2.65E-06	1.82E-06	2.25E-06
F46B6.11	12	14	18	8	2.80E-06	2.65E-06	1.82E-06	2.25E-06
F46B6.12.1	127	191	123	137	2.80E-06	2.65E-06	1.82E-06	2.25E-06
F46B6.12.2	123	192	114	127	2.80E-06	2.65E-06	1.82E-06	2.25E-06
F46B6.13	1	0	1	0	2.38E-05	3.39E-05	1.50E-05	2.06E-05
F46B6.2	12	12	8	13	2.23E-05	3.28E-05	1.34E-05	1.85E-05
F46B6.3a	443	468	433	607	2.80E-06	2.65E-06	1.82E-06	2.25E-06
F46B6.3b	448	469	446	622	2.80E-06	2.65E-06	1.82E-06	2.25E-06
F46B6.4	126	188	177	207	3.82E-05	3.81E-05	2.43E-05	4.21E-05
F46B6.5a	831	884	1048	1377	3.87E-05	3.82E-05	2.50E-05	4.31E-05
F46B6.5b	714	743	926	1175	1.00E-05	1.41E-05	9.17E-06	1.32E-05
F46B6.5c	738	765	943	1197	3.19E-05	3.20E-05	2.62E-05	4.24E-05
F46B6.6a	358	386	271	431	3.36E-05	3.30E-05	2.84E-05	4.44E-05
F46B6.7.1	5032	4771	6111	8171	3.50E-05	3.43E-05	2.91E-05	4.57E-05
F46B6.7.2	3627	3493	4331	5769	1.73E-05	1.76E-05	8.53E-06	1.67E-05
F46B6.8	92	137	96	70	2.28E-04	2.04E-04	1.80E-04	2.97E-04
F46B6.9	8	17	5	12	2.13E-04	1.94E-04	1.66E-04	2.73E-04
F46C3.1	205	503	201	360	8.34E-06	1.17E-05	5.67E-06	5.11E-06
F46C3.2	12	17	12	5	2.80E-06	2.65E-06	1.82E-06	2.25E-06
F46C3.3	468	452	211	392	6.36E-06	1.47E-05	4.05E-06	8.95E-06
F46C3.4	15	28	13	21	2.80E-06	2.65E-06	1.82E-06	2.25E-06
F46C3.5	1	1	5	0	6.61E-06	6.03E-06	1.93E-06	4.45E-06
F46C5.1	19	15	10	5	2.80E-06	2.65E-06	1.82E-06	2.25E-06
F46C5.10	52	141	64	108	2.80E-06	2.65E-06	1.82E-06	2.25E-06
F46C5.3	4	9	4	5	2.86E-06	2.65E-06	1.82E-06	2.25E-06
F46C5.4	3	5	2	3	1.01E-05	2.58E-05	8.09E-06	1.68E-05
F46C5.6	93	111	103	111	2.80E-06	2.65E-06	1.82E-06	2.25E-06
F46C5.7	46	24	27	6	2.80E-06	2.65E-06	1.82E-06	2.25E-06
F46C5.9	476	532	558	618	3.44E-06	3.89E-06	2.48E-06	3.31E-06
F46C8.1	3	4	0	1	8.60E-06	4.23E-06	3.28E-06	2.25E-06
F46C8.2	46	46	22	23	3.53E-05	3.72E-05	2.69E-05	3.68E-05
F46C8.3	28	45	19	26	2.80E-06	2.65E-06	1.82E-06	2.25E-06
F46C8.5	10	24	21	30	4.31E-06	4.07E-06	1.82E-06	2.25E-06
F46C8.6.1	159	297	107	73	5.18E-06	7.86E-06	2.28E-06	3.87E-06
F46C8.6.2	122	246	85	61	2.80E-06	2.65E-06	1.82E-06	2.56E-06
F46C8.7	9	23	20	14	1.55E-05	2.74E-05	6.80E-06	5.71E-06
F46C8.8	101	200	38	55	1.42E-05	2.71E-05	6.45E-06	5.71E-06
F46E10.10a	2203	2502	3201	3509	2.80E-06	2.65E-06	1.82E-06	2.25E-06
F46E10.10b.1	2639	2907	3842	3970	1.11E-05	2.07E-05	2.70E-06	4.84E-06
F46E10.10b.2	1712	2041	2615	2679	2.44E-04	2.62E-04	2.31E-04	3.12E-04
F46E10.10c.1	2487	2761	3697	3965	2.46E-04	2.56E-04	2.33E-04	2.97E-04
F46E10.10c.2	1608	1730	2336	2796	2.33E-04	2.62E-04	2.32E-04	2.93E-04
F46E10.11	11	21	26	14	2.41E-04	2.53E-04	2.33E-04	3.09E-04
F46E10.1a	868	1298	1114	1674	2.31E-04	2.34E-04	2.18E-04	3.22E-04

F46E10.1c.1	687	1069	916	1403	2.80E-06	3.94E-06	3.35E-06	2.25E-06
F46E10.1c.2	697	1081	926	1416	4.07E-05	5.74E-05	3.39E-05	6.30E-05
F46E10.1c.3	686	1068	916	1403	4.31E-05	6.34E-05	3.74E-05	7.07E-05
F46E10.2.1	13	17	7	10	4.26E-05	6.25E-05	3.69E-05	6.96E-05
F46E10.3	3	3	0	0	4.31E-05	6.33E-05	3.74E-05	7.08E-05
F46E10.8	61	87	69	64	2.80E-06	3.28E-06	1.82E-06	2.25E-06
F46E10.9.1	926	1152	746	953	2.80E-06	2.65E-06	1.82E-06	2.25E-06
F46E10.9.2	889	1112	687	929	9.13E-06	1.23E-05	6.72E-06	7.69E-06
F46E10.9.3	567	689	443	547	8.36E-05	9.82E-05	4.38E-05	6.91E-05
F46F11.10	59	93	73	116	7.22E-05	8.53E-05	3.63E-05	6.06E-05
F46F11.11	3	0	0	1	8.56E-05	9.82E-05	4.35E-05	6.63E-05
F46F11.1a	482	623	571	851	5.63E-06	8.38E-06	4.54E-06	8.91E-06
F46F11.1b	453	576	540	813	2.80E-06	2.65E-06	1.82E-06	2.25E-06
F46F11.2.1	7933	6862	10284	12545	1.34E-05	1.64E-05	1.03E-05	1.90E-05
F46F11.2.2	5951	5146	7531	10017	1.33E-05	1.60E-05	1.03E-05	1.92E-05
F46F11.3	57	48	39	23	8.81E-04	7.20E-04	7.43E-04	1.12E-03
F46F11.4	223	305	502	152	7.96E-04	6.51E-04	6.56E-04	1.08E-03
F46F11.5.1	1563	2056	924	1361	8.04E-06	6.40E-06	3.57E-06	2.61E-06
F46F11.5.2	1438	1863	779	1223	7.59E-05	9.81E-05	1.11E-04	4.16E-05
F46F11.6	514	659	605	739	2.57E-04	3.19E-04	9.89E-05	1.80E-04
F46F11.7	55	109	52	37	2.37E-04	2.90E-04	8.35E-05	1.62E-04
F46F11.8	365	330	563	676	3.91E-05	4.73E-05	2.99E-05	4.51E-05
F46F11.9a	471	532	582	852	6.38E-06	1.20E-05	3.94E-06	3.46E-06
F46F11.9b	490	564	613	882	3.83E-05	3.27E-05	3.84E-05	5.69E-05
F46F2.1	22	24	31	8	1.36E-05	1.46E-05	1.10E-05	1.98E-05
F46F2.2a.1	310	379	228	387	1.34E-05	1.46E-05	1.09E-05	1.94E-05
F46F2.2a.2	166	188	115	195	2.80E-06	2.65E-06	1.82E-06	2.25E-06
F46F2.2b	139	167	104	180	1.57E-05	1.81E-05	7.51E-06	1.57E-05
F46F2.2c	300	365	218	374	1.26E-05	1.35E-05	5.69E-06	1.19E-05
F46F2.2d.1	175	193	122	204	9.18E-06	1.04E-05	4.48E-06	9.56E-06
F46F2.2d.2	167	186	115	195	1.63E-05	1.87E-05	7.71E-06	1.63E-05
F46F2.3	1357	1363	1882	840	1.16E-05	1.21E-05	5.28E-06	1.09E-05
F46F2.4	7	3	5	2	1.27E-05	1.33E-05	5.67E-06	1.19E-05
F46F2.5	12	12	10	24	2.42E-04	2.30E-04	2.19E-04	1.21E-04
F46F3.1	25	13	14	18	2.80E-06	2.65E-06	1.82E-06	2.25E-06
F46F3.2	49	79	58	49	2.80E-06	2.65E-06	1.82E-06	2.29E-06
F46F3.3	17	42	18	10	3.11E-06	2.65E-06	1.82E-06	2.25E-06
F46F3.4	1079	995	1454	1978	2.91E-06	4.44E-06	2.26E-06	2.34E-06
F46F5.1	2	6	1	1	2.80E-06	4.60E-06	1.82E-06	2.25E-06
F46F5.10	5	7	2	3	4.42E-05	3.85E-05	3.87E-05	6.50E-05
F46F5.11	27	37	12	8	2.80E-06	2.65E-06	1.82E-06	2.25E-06
F46F5.12	1	0	0	0	2.80E-06	2.65E-06	1.82E-06	2.25E-06
F46F5.13	2	1	0	0	2.80E-06	2.65E-06	1.82E-06	2.25E-06
F46F5.14	9	9	4	1	2.80E-06	2.65E-06	1.82E-06	2.25E-06
F46F5.15	2	2	4	1	2.80E-06	2.65E-06	1.82E-06	2.25E-06
F46F5.16	7	5	5	1	2.80E-06	2.65E-06	1.82E-06	2.25E-06
F46F5.2	3	3	7	9	2.80E-06	2.65E-06	1.82E-06	2.25E-06
F46F5.3	3	3	5	2	2.80E-06	2.65E-06	1.82E-06	2.25E-06
F46F5.4	3	8	3	3	2.80E-06	2.65E-06	1.82E-06	2.25E-06
F46F5.5	2	4	3	1	2.80E-06	2.65E-06	1.82E-06	2.25E-06
F46F5.6	20	36	45	15	2.80E-06	2.65E-06	1.82E-06	2.25E-06
F46F5.7	15	17	9	8	2.80E-06	2.65E-06	1.82E-06	2.25E-06
F46F5.8	0	1	0	1	6.97E-06	1.19E-05	1.02E-05	4.21E-06
F46F5.9	11	14	9	4	2.80E-06	2.65E-06	1.82E-06	2.25E-06
F46F6.1a.1	114	143	129	227	2.80E-06	2.65E-06	1.82E-06	2.25E-06
F46F6.1a.2	108	134	124	225	3.92E-06	4.71E-06	2.08E-06	2.25E-06
F46F6.1b	62	57	60	96	5.99E-06	7.12E-06	4.41E-06	9.60E-06
F46F6.2	417	445	568	899	5.94E-06	6.96E-06	4.43E-06	9.94E-06
F46G10.2	42	133	34	53	7.00E-06	6.08E-06	4.41E-06	8.70E-06

F46G10.3	5	9	7	8	1.22E-05	1.23E-05	1.08E-05	2.11E-05
F46G10.4	29	52	42	55	2.80E-06	6.35E-06	1.82E-06	2.25E-06
F46G10.5	296	414	225	488	2.80E-06	2.65E-06	1.82E-06	2.25E-06
F46G10.6	170	813	106	288	2.80E-06	3.57E-06	1.99E-06	3.19E-06
F46G10.7a	43	80	26	33	1.21E-05	1.60E-05	5.99E-06	1.60E-05
F46G10.7b	40	77	22	33	1.91E-05	8.63E-05	7.74E-06	2.60E-05
F46G11.1	13	24	23	23	5.10E-06	8.97E-06	2.00E-06	3.15E-06
F46G11.2	33	63	20	20	5.15E-06	9.36E-06	1.84E-06	3.42E-06
F46G11.3	93	115	61	110	2.80E-06	2.65E-06	1.82E-06	2.25E-06
F46G11.4	15	24	15	6	3.05E-06	5.50E-06	1.82E-06	2.25E-06
F46H5.2a	138	194	184	273	5.29E-06	6.19E-06	2.26E-06	5.02E-06
F46H5.2b	105	141	143	215	5.12E-06	7.78E-06	3.33E-06	2.25E-06
F46H5.2c	122	178	168	238	8.34E-06	1.11E-05	7.25E-06	1.33E-05
F46H5.3a	6341	6977	8711	8021	8.57E-06	1.09E-05	7.60E-06	1.41E-05
F46H5.3b.1	6869	7564	9676	8853	7.67E-06	1.06E-05	6.87E-06	1.20E-05
F46H5.3b.2	6864	7560	9659	8860	5.96E-04	6.20E-04	5.33E-04	6.06E-04
F46H5.3b.3	6862	7550	9669	8843	5.40E-04	5.62E-04	4.95E-04	5.59E-04
F46H5.4	257	290	318	511	5.93E-04	6.17E-04	5.43E-04	6.15E-04
F46H5.5	8	3	6	1	6.45E-04	6.71E-04	5.92E-04	6.68E-04
F46H5.6	19	9	11	11	4.51E-06	4.79E-06	3.63E-06	7.17E-06
F46H5.7a.1	511	870	463	809	2.80E-06	2.65E-06	1.82E-06	2.25E-06
F46H5.7a.2	430	755	410	753	2.80E-06	2.65E-06	1.82E-06	2.25E-06
F46H5.7b	484	843	438	779	2.67E-05	4.29E-05	1.57E-05	3.39E-05
F46H5.7c.1	310	542	288	577	2.79E-05	4.63E-05	1.73E-05	3.93E-05
F46H5.7c.2	35	53	17	29	2.62E-05	4.31E-05	1.55E-05	3.39E-05
F46H5.8	27	50	22	40	1.67E-05	2.77E-05	1.01E-05	2.51E-05
F46H6.1.1	221	300	186	244	6.92E-06	9.89E-06	2.19E-06	4.61E-06
F46H6.1.2	150	208	123	182	2.80E-06	4.47E-06	1.82E-06	3.04E-06
F46H6.2a	101	232	85	151	3.12E-05	4.01E-05	1.71E-05	2.77E-05
F46H6.2b.1	64	116	47	86	2.88E-05	3.77E-05	1.54E-05	2.81E-05
F46H6.2b.2	51	87	37	66	5.96E-06	1.29E-05	3.26E-06	7.15E-06
F46H6.2b.3	38	59	27	54	5.60E-06	9.60E-06	2.68E-06	6.05E-06
F46H6.2c	88	206	79	138	6.58E-06	1.06E-05	3.10E-06	6.84E-06
F46H6.5	4	10	5	2	7.53E-06	1.11E-05	3.48E-06	8.59E-06
F47A4.1b	6	12	4	13	6.58E-06	1.45E-05	3.84E-06	8.28E-06
F47A4.2	523	543	252	681	2.80E-06	2.65E-06	1.82E-06	2.25E-06
F47A4.3a	43	63	55	46	2.80E-06	2.65E-06	1.82E-06	2.25E-06
F47A4.3b	37	53	45	47	5.46E-06	5.34E-06	1.82E-06	5.71E-06
F47A4.5	158	211	221	313	2.80E-06	2.67E-06	1.82E-06	2.25E-06
F47A4.t1	0	0	1	0	2.80E-06	2.65E-06	1.82E-06	2.25E-06
F47B10.1.1	1604	1700	1570	1885	4.87E-06	6.16E-06	4.45E-06	7.76E-06
F47B10.1.2	1205	1235	1254	1519	2.80E-06	2.65E-06	1.82E-06	2.25E-06
F47B10.2	311	768	494	642	1.08E-04	1.08E-04	6.86E-05	1.02E-04
F47B10.3	5	11	7	6	1.03E-04	9.93E-05	6.95E-05	1.04E-04
F47B10.4	1	1	1	0	1.55E-05	3.62E-05	1.60E-05	2.57E-05
F47B10.5	33	52	22	32	2.80E-06	2.65E-06	1.82E-06	2.25E-06
F47B10.6	1	2	0	1	2.80E-06	2.65E-06	1.82E-06	2.25E-06
F47B10.8	6	12	3	1	9.88E-06	1.47E-05	4.28E-06	7.69E-06
F47B10.9	24	59	37	42	2.80E-06	2.65E-06	1.82E-06	2.25E-06
F47B3.1	34	43	27	24	2.80E-06	2.65E-06	1.82E-06	2.25E-06
F47B3.2	14	18	8	8	4.09E-06	9.50E-06	4.10E-06	5.76E-06
F47B3.3	25	40	14	31	3.50E-06	4.18E-06	1.82E-06	2.25E-06
F47B3.4	34	47	21	14	3.75E-06	4.55E-06	1.82E-06	2.25E-06
F47B3.5	24	39	17	13	5.94E-06	8.99E-06	2.17E-06	5.91E-06
F47B3.6	31	39	18	15	4.51E-06	5.87E-06	1.82E-06	2.25E-06
F47B3.7	31	73	36	24	4.48E-06	6.88E-06	2.06E-06	2.25E-06
F47B3.8	170	252	251	353	3.70E-06	4.39E-06	1.82E-06	2.25E-06
F47B7.2a	284	383	237	340	2.91E-06	6.48E-06	2.20E-06	2.25E-06
F47B7.2b	263	360	227	332	2.80E-06	3.36E-06	2.31E-06	4.00E-06

F47B7.2c	247	342	217	313	1.04E-05	1.33E-05	5.65E-06	1.00E-05
F47B7.3	2	0	2	2	1.31E-05	1.70E-05	7.36E-06	1.33E-05
F47B7.4	6	4	3	3	1.36E-05	1.77E-05	7.74E-06	1.38E-05
F47B7.5	1	5	3	2	2.80E-06	2.65E-06	1.82E-06	2.25E-06
F47B7.6	1	2	3	2	2.80E-06	2.65E-06	1.82E-06	2.25E-06
F47B7.7	61	89	58	77	2.80E-06	2.65E-06	1.82E-06	2.25E-06
F47B8.1	4	7	1	2	2.80E-06	2.65E-06	1.82E-06	2.25E-06
F47B8.10	9	21	9	6	4.59E-06	6.32E-06	2.82E-06	4.63E-06
F47B8.11	105	239	86	43	2.80E-06	2.65E-06	1.82E-06	2.25E-06
F47B8.12	3	7	7	8	2.80E-06	2.65E-06	1.82E-06	2.25E-06
F47B8.13	13	16	10	4	1.39E-05	2.99E-05	7.42E-06	4.57E-06
F47B8.14	7	14	6	3	2.80E-06	2.65E-06	1.82E-06	2.25E-06
F47B8.2	24	44	15	27	2.80E-06	2.65E-06	1.82E-06	2.25E-06
F47B8.3	11	37	15	24	2.80E-06	2.65E-06	1.82E-06	2.25E-06
F47B8.4	9	14	8	3	4.93E-06	8.52E-06	2.00E-06	4.45E-06
F47B8.5	31	31	17	14	2.80E-06	4.58E-06	1.82E-06	2.52E-06
F47B8.6	5	5	5	9	2.80E-06	2.65E-06	1.82E-06	2.25E-06
F47B8.7	9	9	14	9	2.80E-06	2.65E-06	1.82E-06	2.25E-06
F47B8.8	38	46	51	32	2.80E-06	2.65E-06	1.82E-06	2.25E-06
F47B8.9a	3	5	9	3	2.80E-06	2.65E-06	1.82E-06	2.25E-06
F47B8.9b	4	5	10	3	6.86E-06	7.83E-06	5.99E-06	4.63E-06
F47C10.1	0	4	4	0	2.80E-06	2.65E-06	1.82E-06	2.25E-06
F47C10.2	13	9	4	3	2.80E-06	2.65E-06	1.82E-06	2.25E-06
F47C10.3	3	6	1	2	2.80E-06	2.65E-06	1.82E-06	2.25E-06
F47C10.4	5	5	1	4	2.80E-06	2.65E-06	1.82E-06	2.25E-06
F47C10.6	5	17	15	8	2.80E-06	2.65E-06	1.82E-06	2.25E-06
F47C10.7	4	4	0	3	2.80E-06	2.65E-06	1.82E-06	2.25E-06
F47C10.8	6	7	19	8	2.80E-06	2.65E-06	1.82E-06	2.25E-06
F47C12.1	36	37	17	49	2.80E-06	2.65E-06	1.82E-06	2.25E-06
F47C12.10	5	6	1	8	2.80E-06	2.65E-06	1.82E-06	2.25E-06
F47C12.11	0	2	0	0	2.80E-06	2.65E-06	1.82E-06	2.25E-06
F47C12.2	4	8	7	11	2.80E-06	2.65E-06	1.82E-06	2.25E-06
F47C12.3	4	4	2	5	2.80E-06	2.65E-06	1.82E-06	2.25E-06
F47C12.4	46	114	24	29	2.80E-06	2.65E-06	1.82E-06	2.25E-06
F47C12.5	3	2	2	1	2.80E-06	2.65E-06	1.82E-06	2.25E-06
F47C12.6	0	0	0	1	2.86E-06	6.72E-06	1.82E-06	2.25E-06
F47C12.7	2	1	0	0	2.80E-06	2.65E-06	1.82E-06	2.25E-06
F47C12.8	1	1	3	1	2.80E-06	2.65E-06	1.82E-06	2.25E-06
F47D12.10	9	17	16	20	2.80E-06	2.65E-06	1.82E-06	2.25E-06
F47D12.1a	36	52	23	33	2.80E-06	2.65E-06	1.82E-06	2.25E-06
F47D12.1c	32	52	23	34	2.80E-06	4.92E-06	3.19E-06	4.93E-06
F47D12.1d	7	13	5	4	2.80E-06	2.65E-06	1.82E-06	2.25E-06
F47D12.1e	27	38	20	32	2.80E-06	2.65E-06	1.82E-06	2.25E-06
F47D12.3	5	8	6	3	2.80E-06	2.65E-06	1.82E-06	2.25E-06
F47D12.4b	501	546	517	717	2.80E-06	2.91E-06	1.82E-06	2.25E-06
F47D12.4c	492	544	515	718	2.80E-06	2.65E-06	1.82E-06	2.25E-06
F47D12.5	20	23	34	22	4.28E-05	4.40E-05	2.87E-05	4.92E-05
F47D12.6	58	76	48	18	7.85E-05	8.20E-05	5.35E-05	9.20E-05
F47D12.7	45	87	25	21	2.80E-06	2.65E-06	1.82E-06	2.25E-06
F47D12.9a.1	594	629	985	1111	1.68E-05	2.08E-05	9.04E-06	4.18E-06
F47D12.9a.2	592	628	984	1110	2.80E-06	5.05E-06	1.82E-06	2.25E-06
F47D12.9b.1	754	838	1278	1387	3.68E-05	3.68E-05	3.97E-05	5.52E-05
F47D12.9b.2	632	715	1075	1202	4.02E-05	4.03E-05	4.35E-05	6.06E-05
F47D12.9b.3	678	742	1119	1257	4.01E-05	4.21E-05	4.43E-05	5.93E-05
F47D12.9b.4	469	505	777	924	4.12E-05	4.40E-05	4.56E-05	6.30E-05
F47D2.1	3	4	5	4	4.12E-05	4.26E-05	4.42E-05	6.13E-05
F47D2.10a	4	7	3	10	4.16E-05	4.23E-05	4.49E-05	6.59E-05
F47D2.10b	4	6	3	10	2.80E-06	2.65E-06	1.82E-06	2.25E-06
F47D2.2	5	3	4	5	2.80E-06	2.65E-06	1.82E-06	2.25E-06

F47D2.3	8	4	4	4	2.80E-06	2.65E-06	1.82E-06	2.25E-06
F47D2.4	1	0	4	4	2.80E-06	2.65E-06	1.82E-06	2.25E-06
F47D2.5	1	4	8	0	2.80E-06	2.65E-06	1.82E-06	2.25E-06
F47D2.6	3	3	1	4	2.80E-06	2.65E-06	1.82E-06	2.25E-06
F47D2.7	4	3	4	2	2.80E-06	2.65E-06	1.82E-06	2.25E-06
F47D2.8	1	5	8	0	2.80E-06	2.65E-06	1.82E-06	2.25E-06
F47D2.9	7	2	8	5	2.80E-06	2.65E-06	1.82E-06	2.25E-06
F47E1.1	0	1	0	0	2.80E-06	2.65E-06	1.82E-06	2.25E-06
F47E1.2	40	53	59	60	2.80E-06	2.65E-06	1.82E-06	2.25E-06
F47E1.3	4	11	19	5	2.80E-06	2.65E-06	1.82E-06	2.25E-06
F47E1.5	3	5	2	3	2.80E-06	2.65E-06	1.82E-06	2.25E-06
F47E1.t3	0	1	0	0	2.80E-06	2.65E-06	1.82E-06	2.25E-06
F47F2.1a	12	30	20	24	2.80E-06	2.65E-06	1.82E-06	2.25E-06
F47F2.1b	28	46	36	30	2.80E-06	2.65E-06	1.82E-06	2.25E-06
F47F2.1c.1	19	38	28	27	2.80E-06	3.15E-06	1.82E-06	2.25E-06
F47F2.1c.2	21	38	26	27	2.80E-06	3.17E-06	1.82E-06	2.25E-06
F47F2.3	5	1	1	3	2.80E-06	3.07E-06	1.82E-06	2.25E-06
F47F6.1a	89	80	70	65	2.80E-06	3.57E-06	1.82E-06	2.25E-06
F47F6.1b	146	127	123	103	2.80E-06	2.65E-06	1.82E-06	2.25E-06
F47F6.1c	33	39	35	19	1.05E-05	8.89E-06	5.36E-06	6.14E-06
F47F6.3	20	36	24	9	8.01E-06	6.59E-06	4.39E-06	4.54E-06
F47F6.4	0	2	2	1	2.80E-06	2.67E-06	1.82E-06	2.25E-06
F47F6.5	20	41	9	10	2.80E-06	2.65E-06	1.82E-06	2.25E-06
F47F6.9	7	2	6	4	2.80E-06	2.65E-06	1.82E-06	2.25E-06
F47G3.1	45	83	47	62	2.80E-06	2.65E-06	1.82E-06	2.25E-06
F47G3.2	35	29	29	38	2.80E-06	2.65E-06	1.82E-06	2.25E-06
F47G3.3	52	74	75	103	2.80E-06	3.17E-06	1.82E-06	2.25E-06
F47G4.1	0	2	1	2	3.89E-06	3.04E-06	2.10E-06	3.40E-06
F47G4.2	66	81	95	144	4.03E-06	5.45E-06	3.79E-06	6.43E-06
F47G4.3	17	71	28	43	2.80E-06	2.65E-06	1.82E-06	2.25E-06
F47G4.4.1	58	62	65	137	3.33E-06	3.86E-06	3.12E-06	5.82E-06
F47G4.4.2	49	52	55	119	2.80E-06	5.77E-06	1.82E-06	2.97E-06
F47G4.5	3	3	2	1	2.80E-06	2.78E-06	2.00E-06	5.24E-06
F47G4.6	5	2	3	3	2.80E-06	2.65E-06	1.82E-06	4.86E-06
F47G4.8	1	0	2	0	2.80E-06	2.65E-06	1.82E-06	2.25E-06
F47G6.1	43	34	35	40	2.80E-06	2.65E-06	1.82E-06	2.25E-06
F47G6.2	27	44	244	59	2.80E-06	2.65E-06	1.82E-06	2.25E-06
F47G6.3	14	26	19	8	2.80E-06	2.65E-06	1.82E-06	2.25E-06
F47G6.4	150	262	128	136	2.80E-06	2.67E-06	1.02E-05	3.06E-06
F47G6.t1	0	0	1	1	2.80E-06	2.65E-06	1.82E-06	2.25E-06
F47G9.1.1	992	1179	788	1020	4.42E-06	7.33E-06	2.46E-06	3.24E-06
F47G9.1.2	675	782	513	699	2.80E-06	2.65E-06	1.82E-06	2.25E-06
F47G9.2	5	1	6	0	1.19E-04	1.33E-04	6.13E-05	9.79E-05
F47G9.3	64	68	44	54	1.19E-04	1.31E-04	5.91E-05	9.93E-05
F47G9.4	51	61	40	48	2.80E-06	2.65E-06	1.82E-06	2.25E-06
F47G9.6	0	4	3	3	2.91E-06	2.91E-06	1.82E-06	2.25E-06
F47H4.1	75	110	126	113	2.80E-06	2.65E-06	1.82E-06	2.25E-06
F47H4.10	5	7	9	4	2.80E-06	2.65E-06	1.82E-06	2.25E-06
F47H4.11	8	5	8	3	8.43E-06	1.17E-05	9.22E-06	1.02E-05
F47H4.12	15	34	23	15	2.80E-06	2.65E-06	1.82E-06	2.25E-06
F47H4.2	12	36	22	13	2.80E-06	2.65E-06	1.82E-06	2.25E-06
F47H4.3	2	4	5	3	2.80E-06	2.65E-06	1.82E-06	2.25E-06
F47H4.4	5	7	7	4	2.80E-06	2.65E-06	1.82E-06	2.25E-06
F47H4.5	7	2	2	0	2.80E-06	2.65E-06	1.82E-06	2.25E-06
F47H4.6	4	6	7	6	2.80E-06	2.65E-06	1.82E-06	2.25E-06
F47H4.7	7	6	10	7	2.80E-06	2.65E-06	1.82E-06	2.25E-06
F47H4.8	10	11	8	6	2.80E-06	2.65E-06	1.82E-06	2.25E-06
F47H4.9	18	27	21	22	2.80E-06	2.65E-06	1.82E-06	2.25E-06
F48A11.1	79	134	63	72	2.80E-06	2.65E-06	1.82E-06	2.25E-06

F48A11.2	5	6	4	1	2.80E-06	2.65E-06	1.82E-06	2.25E-06
F48A11.4	319	225	420	540	2.80E-06	2.70E-06	1.82E-06	2.25E-06
F48A11.5a	1140	1007	1204	1498	2.80E-06	2.65E-06	1.82E-06	2.25E-06
F48A11.5b	1270	1110	1383	1630	2.60E-05	1.73E-05	2.23E-05	3.54E-05
F48A9.1	23	43	37	44	6.72E-05	5.61E-05	4.62E-05	7.10E-05
F48A9.2	8	16	4	11	7.40E-05	6.11E-05	5.24E-05	7.63E-05
F48A9.3	12	30	88	14	2.80E-06	4.47E-06	2.64E-06	3.89E-06
F48B9.1	17	27	21	18	2.80E-06	2.65E-06	1.82E-06	2.25E-06
F48B9.2	6	11	12	12	2.80E-06	2.94E-06	5.92E-06	2.25E-06
F48B9.3	2	11	7	1	2.80E-06	2.65E-06	1.82E-06	2.25E-06
F48B9.4	20	27	15	3	2.80E-06	2.65E-06	1.82E-06	2.25E-06
F48B9.5	1	15	6	2	2.80E-06	2.65E-06	1.82E-06	2.25E-06
F48B9.8	31	54	34	32	8.29E-06	1.06E-05	4.05E-06	2.25E-06
F48C1.1	45	129	81	68	2.80E-06	2.65E-06	1.82E-06	2.25E-06
F48C1.2	24	36	30	32	4.40E-06	7.22E-06	3.13E-06	3.64E-06
F48C1.3	1	2	4	5	2.80E-06	4.13E-06	1.82E-06	2.25E-06
F48C1.4	35	40	67	73	2.80E-06	3.49E-06	2.00E-06	2.65E-06
F48C1.5	87	71	127	111	2.80E-06	2.65E-06	1.82E-06	2.25E-06
F48C1.6	262	260	310	355	1.24E-05	1.34E-05	1.55E-05	2.08E-05
F48C1.7	57	120	26	29	2.60E-05	2.00E-05	2.47E-05	2.66E-05
F48C1.8	13	11	9	13	3.98E-05	3.73E-05	3.06E-05	4.33E-05
F48C1.9	8	19	8	3	7.08E-06	1.41E-05	2.11E-06	2.90E-06
F48C11.1	4	7	5	2	2.80E-06	2.65E-06	1.82E-06	2.25E-06
F48C11.2	9	14	12	17	3.08E-06	6.88E-06	2.00E-06	2.25E-06
F48C11.3	38	74	40	29	2.80E-06	2.65E-06	1.82E-06	2.25E-06
F48C5.1	15	30	11	13	2.80E-06	2.65E-06	1.82E-06	2.25E-06
F48C5.2	6	17	5	2	8.06E-06	1.48E-05	5.52E-06	4.95E-06
F48D6.2a	0	8	5	0	2.80E-06	3.12E-06	1.82E-06	2.25E-06
F48D6.2b	0	8	5	1	2.80E-06	3.52E-06	1.82E-06	2.25E-06
F48D6.3	3	5	11	4	2.80E-06	2.65E-06	1.82E-06	2.25E-06
F48D6.4a	591	814	1376	323	2.80E-06	2.65E-06	1.82E-06	2.25E-06
F48D6.4b	597	847	1370	330	2.80E-06	2.65E-06	1.82E-06	2.25E-06
F48D6.4c	573	782	1347	308	1.25E-04	1.63E-04	1.90E-04	5.49E-05
F48E3.1a.1	44	67	40	70	1.25E-04	1.68E-04	1.87E-04	5.56E-05
F48E3.1a.2	49	73	43	73	1.25E-04	1.61E-04	1.91E-04	5.40E-05
F48E3.1b	50	73	43	71	2.80E-06	3.84E-06	1.82E-06	3.40E-06
F48E3.2	29	51	48	55	2.80E-06	3.57E-06	1.82E-06	3.04E-06
F48E3.3	786	1077	906	1269	2.80E-06	3.25E-06	1.82E-06	2.70E-06
F48E3.4	444	534	419	564	2.80E-06	3.52E-06	2.30E-06	3.24E-06
F48E3.6	16	12	6	14	1.86E-05	2.41E-05	1.40E-05	2.42E-05
F48E3.7	8	15	9	3	4.98E-05	5.66E-05	3.06E-05	5.09E-05
F48E3.8a	60	83	59	81	2.80E-06	2.65E-06	1.82E-06	2.25E-06
F48E3.8b	38	55	43	70	2.80E-06	2.65E-06	1.82E-06	2.25E-06
F48E3.8c	11	25	7	27	2.80E-06	2.65E-06	1.82E-06	2.25E-06
F48E3.9	9	14	12	10	2.80E-06	2.65E-06	1.82E-06	2.25E-06
F48E3.t1	1	0	3	0	2.80E-06	2.65E-06	1.82E-06	2.25E-06
F48E8.1a	249	378	307	402	2.80E-06	2.65E-06	1.82E-06	2.25E-06
F48E8.1b	238	366	290	377	2.80E-06	2.65E-06	3.04E-06	2.25E-06
F48E8.1c	226	356	276	358	2.40E-05	3.44E-05	1.92E-05	3.11E-05
F48E8.2	172	198	332	381	2.32E-05	3.37E-05	1.84E-05	2.96E-05
F48E8.3.1	244	345	240	357	2.32E-05	3.46E-05	1.85E-05	2.96E-05
F48E8.3.2	193	269	204	299	1.05E-05	1.14E-05	1.32E-05	1.87E-05
F48E8.4	321	327	626	787	1.48E-05	1.98E-05	9.46E-06	1.74E-05
F48E8.5.1	2835	2907	3678	4856	1.18E-05	1.56E-05	8.16E-06	1.48E-05
F48E8.5.2	2822	2892	3644	4824	1.47E-05	1.41E-05	1.86E-05	2.89E-05
F48E8.5.3	2147	2205	2695	3613	1.41E-04	1.36E-04	1.19E-04	1.93E-04
F48E8.6	434	379	620	943	1.41E-04	1.36E-04	1.18E-04	1.93E-04
F48E8.7a	567	512	1006	1181	1.12E-04	1.08E-04	9.11E-05	1.51E-04
F48E8.7b	541	482	953	1128	1.83E-05	1.51E-05	1.70E-05	3.19E-05

F48E8.8	4	11	5	6	4.35E-05	3.71E-05	5.03E-05	7.28E-05
F48F5.1	56	86	28	27	4.30E-05	3.62E-05	4.93E-05	7.20E-05
F48F5.2	3	12	2	3	2.80E-06	2.65E-06	1.82E-06	2.25E-06
F48F5.3	2	2	5	1	2.80E-06	2.65E-06	1.82E-06	2.25E-06
F48F5.4	2	7	6	3	2.80E-06	2.78E-06	1.82E-06	2.25E-06
F48F5.5	138	180	179	282	2.80E-06	2.65E-06	1.84E-06	2.25E-06
F48F5.6	12	21	16	11	2.80E-06	2.65E-06	1.82E-06	2.25E-06
F48F7.1	449	480	731	908	1.32E-05	1.62E-05	1.11E-05	2.16E-05
F48F7.2.1	50	94	46	54	3.19E-06	5.29E-06	2.77E-06	2.36E-06
F48F7.2.2	25	47	17	27	1.10E-05	1.11E-05	1.16E-05	1.78E-05
F48F7.3	2	6	1	1	4.09E-06	7.27E-06	2.46E-06	3.55E-06
F48F7.4	114	154	156	217	3.05E-06	5.42E-06	1.82E-06	2.65E-06
F48F7.5	13	38	12	10	2.80E-06	2.65E-06	1.82E-06	2.25E-06
F48F7.7	12	21	2	2	4.48E-06	5.71E-06	3.97E-06	6.84E-06
F48F7.8	36	37	47	80	2.80E-06	4.55E-06	1.82E-06	2.25E-06
F48G7.1	6	2	10	5	2.80E-06	2.65E-06	1.82E-06	2.25E-06
F48G7.10	1	5	1	2	5.99E-06	5.82E-06	5.10E-06	1.07E-05
F48G7.11	13	15	17	13	2.80E-06	2.65E-06	1.82E-06	2.25E-06
F48G7.12	4	8	9	4	2.80E-06	2.65E-06	1.82E-06	2.25E-06
F48G7.13	10	9	46	19	2.80E-06	2.65E-06	1.82E-06	2.25E-06
F48G7.2	4	8	2	5	2.80E-06	2.65E-06	1.82E-06	2.25E-06
F48G7.3	10	13	12	12	2.80E-06	2.65E-06	4.10E-06	2.25E-06
F48G7.4	8	17	10	6	2.80E-06	2.65E-06	1.82E-06	2.25E-06
F48G7.5	34	39	27	14	2.80E-06	2.65E-06	1.82E-06	2.25E-06
F48G7.6	4	3	5	5	2.80E-06	2.65E-06	1.82E-06	2.25E-06
F48G7.7	2	7	0	2	5.63E-06	6.11E-06	2.92E-06	2.25E-06
F48G7.8	14	15	5	3	2.80E-06	2.65E-06	1.82E-06	2.25E-06
F48G7.9	1	2	0	1	2.80E-06	2.65E-06	1.82E-06	2.25E-06
F49A5.2	7	8	7	4	3.14E-06	3.17E-06	1.82E-06	2.25E-06
F49A5.3	3	6	1	0	2.80E-06	2.65E-06	1.82E-06	2.25E-06
F49A5.4	1	5	4	1	2.80E-06	2.65E-06	1.82E-06	2.25E-06
F49A5.5a	10	10	12	3	2.80E-06	2.65E-06	1.82E-06	2.25E-06
F49A5.5b	10	8	12	3	2.80E-06	2.65E-06	1.82E-06	2.25E-06
F49A5.6	2	3	2	1	2.80E-06	2.65E-06	1.82E-06	2.25E-06
F49A5.7	10	8	2	3	2.80E-06	2.65E-06	1.82E-06	2.25E-06
F49A5.8	1	2	3	1	2.80E-06	2.65E-06	1.82E-06	2.25E-06
F49A5.9	2	6	3	0	2.80E-06	2.65E-06	1.82E-06	2.25E-06
F49B2.1	4	6	6	5	2.80E-06	2.65E-06	1.82E-06	2.25E-06
F49B2.2	15	19	22	17	2.80E-06	2.65E-06	1.82E-06	2.25E-06
F49B2.3	12	18	356	361	2.80E-06	2.65E-06	1.82E-06	2.25E-06
F49B2.4	5	6	2	2	2.80E-06	2.65E-06	1.82E-06	2.25E-06
F49B2.5	29	46	36	35	2.80E-06	2.65E-06	2.68E-05	3.35E-05
F49B2.6	39	38	36	33	2.80E-06	2.65E-06	1.82E-06	2.25E-06
F49C12.1	7	7	6	3	2.80E-06	2.94E-06	1.82E-06	2.25E-06
F49C12.10	5	4	12	11	3.02E-06	2.78E-06	1.82E-06	2.25E-06
F49C12.11.1	594	722	465	310	2.80E-06	2.65E-06	1.82E-06	2.25E-06
F49C12.11.2	504	640	330	255	2.80E-06	2.65E-06	1.82E-06	2.25E-06
F49C12.13.1	804	1177	1184	878	1.71E-04	1.97E-04	8.74E-05	7.19E-05
F49C12.13.2	671	948	1001	825	1.45E-04	1.75E-04	6.20E-05	5.91E-05
F49C12.14	46	71	46	19	1.61E-04	2.23E-04	1.55E-04	1.42E-04
F49C12.15	192	458	103	124	1.34E-04	1.79E-04	1.31E-04	1.33E-04
F49C12.2	6	7	4	6	7.06E-06	1.03E-05	4.59E-06	2.34E-06
F49C12.3	5	11	6	4	7.98E-06	1.80E-05	2.79E-06	4.14E-06
F49C12.4	25	12	6	6	2.80E-06	2.65E-06	1.82E-06	2.25E-06
F49C12.5a	6	8	16	8	2.80E-06	2.65E-06	1.82E-06	2.25E-06
F49C12.5b	5	8	16	9	2.88E-06	2.65E-06	1.82E-06	2.25E-06
F49C12.6	21	29	16	22	2.80E-06	2.65E-06	1.82E-06	2.25E-06
F49C12.7a	129	71	97	92	2.80E-06	2.65E-06	1.82E-06	2.25E-06
F49C12.7b	136	79	99	95	2.80E-06	3.09E-06	1.82E-06	2.25E-06

F49C12.7c.1	144	82	109	101	1.08E-05	5.63E-06	5.30E-06	6.21E-06
F49C12.7c.2	139	78	101	97	1.02E-05	5.58E-06	4.83E-06	5.71E-06
F49C12.7c.3	125	65	96	90	1.13E-05	6.08E-06	5.58E-06	6.36E-06
F49C12.8	2129	2069	2991	3199	1.20E-05	6.35E-06	5.67E-06	6.72E-06
F49C12.9	699	601	1119	1121	1.11E-05	5.45E-06	5.54E-06	6.41E-06
F49C5.1	1	1	4	7	1.76E-04	1.62E-04	1.61E-04	2.13E-04
F49C5.10	2	3	0	0	7.08E-05	5.76E-05	7.38E-05	9.13E-05
F49C5.11	36	14	8	2	2.80E-06	2.65E-06	1.82E-06	2.25E-06
F49C5.2	8	9	15	5	2.80E-06	2.65E-06	1.82E-06	2.25E-06
F49C5.3	11	11	7	8	1.39E-05	5.08E-06	2.00E-06	2.25E-06
F49C5.4	5	7	4	4	2.80E-06	2.65E-06	1.82E-06	2.25E-06
F49C5.5	1	5	5	5	2.80E-06	2.65E-06	1.82E-06	2.25E-06
F49C5.6	6	7	3	1	2.80E-06	2.65E-06	1.82E-06	2.25E-06
F49C5.7	4	1	1	6	2.80E-06	2.65E-06	1.82E-06	2.25E-06
F49C5.9	5	7	4	1	2.80E-06	2.65E-06	1.82E-06	2.25E-06
F49C5.t2	1	0	3	0	2.80E-06	2.65E-06	1.82E-06	2.25E-06
F49C5.t4	1	0	3	0	2.80E-06	2.65E-06	1.82E-06	2.25E-06
F49D11.1	409	548	502	614	2.80E-06	2.65E-06	3.04E-06	2.25E-06
F49D11.2	8	8	14	9	2.80E-06	2.65E-06	2.99E-06	2.25E-06
F49D11.3a	4	14	5	5	2.28E-05	2.88E-05	1.82E-05	2.75E-05
F49D11.3b	3	11	4	5	2.80E-06	2.65E-06	1.82E-06	2.25E-06
F49D11.4	4	8	9	3	2.80E-06	2.65E-06	1.82E-06	2.25E-06
F49D11.6	20	48	21	12	2.80E-06	2.65E-06	1.82E-06	2.25E-06
F49D11.7	3	1	4	0	2.80E-06	2.65E-06	1.82E-06	2.25E-06
F49D11.8	204	305	213	126	2.80E-06	5.16E-06	1.82E-06	2.25E-06
F49E10.1	25	25	21	18	2.80E-06	2.65E-06	1.82E-06	2.25E-06
F49E10.2a	107	115	48	47	4.91E-05	6.94E-05	3.34E-05	2.44E-05
F49E10.2b	104	112	47	46	2.80E-06	2.65E-06	1.82E-06	2.25E-06
F49E10.4a	16	20	12	17	4.96E-06	5.03E-06	1.82E-06	2.25E-06
F49E10.4b	13	20	13	15	4.09E-06	4.15E-06	1.82E-06	2.25E-06
F49E10.5	31	41	38	41	2.80E-06	2.65E-06	1.82E-06	2.25E-06
F49E11.10	78	232	106	108	2.80E-06	2.65E-06	1.82E-06	2.25E-06
F49E11.11	29	43	30	27	2.80E-06	2.65E-06	1.82E-06	2.25E-06
F49E11.1a	1210	1032	1866	2394	1.26E-05	3.55E-05	1.12E-05	1.40E-05
F49E11.1b	2156	1854	3393	4657	4.62E-06	6.48E-06	3.12E-06	3.46E-06
F49E11.1c	2165	1862	3415	4678	8.34E-05	6.72E-05	8.37E-05	1.33E-04
F49E11.1d.1	1104	946	1699	2236	6.72E-05	5.46E-05	6.88E-05	1.17E-04
F49E11.1d.2	1127	976	1730	2275	6.86E-05	5.58E-05	7.04E-05	1.19E-04
F49E11.1d.3	1106	951	1706	2238	8.09E-05	6.55E-05	8.10E-05	1.32E-04
F49E11.1e	1116	964	1723	2246	8.53E-05	6.98E-05	8.52E-05	1.38E-04
F49E11.2	26	46	16	12	8.10E-05	6.58E-05	8.13E-05	1.32E-04
F49E11.3	8	5	9	6	6.63E-05	5.41E-05	6.66E-05	1.07E-04
F49E11.4	2	1	1	2	4.17E-06	6.98E-06	1.82E-06	2.25E-06
F49E11.5	5	3	5	5	2.80E-06	2.65E-06	1.82E-06	2.25E-06
F49E11.6	3	2	1	1	2.80E-06	2.65E-06	1.82E-06	2.25E-06
F49E11.7	10	18	6	3	2.80E-06	2.65E-06	1.82E-06	2.25E-06
F49E11.8	3	3	1	3	2.80E-06	2.65E-06	1.82E-06	2.25E-06
F49E11.9	2	2	0	1	2.80E-06	2.65E-06	1.82E-06	2.25E-06
F49E12.10	18	27	13	20	2.80E-06	2.65E-06	1.82E-06	2.25E-06
F49E12.12	21	58	17	18	2.80E-06	2.65E-06	1.82E-06	2.25E-06
F49E12.2	430	282	946	302	2.80E-06	3.31E-06	1.82E-06	2.25E-06
F49E12.4	11	17	10	6	2.80E-06	5.71E-06	1.82E-06	2.25E-06
F49E12.5a	6	4	9	3	9.50E-05	5.89E-05	1.36E-04	5.36E-05
F49E12.5b	5	3	3	3	2.80E-06	3.73E-06	1.82E-06	2.25E-06
F49E12.6	136	148	137	150	2.80E-06	2.65E-06	1.82E-06	2.25E-06
F49E12.7	3	5	3	2	2.80E-06	2.65E-06	1.82E-06	2.25E-06
F49E12.8	11	18	2	3	7.06E-06	7.25E-06	4.63E-06	6.25E-06
F49E2.1a	35	42	32	33	2.80E-06	2.65E-06	1.82E-06	2.25E-06
F49E2.1b	59	84	55	48	2.80E-06	2.80E-06	1.82E-06	2.25E-06

F49E2.2b	198	268	167	250	3.19E-06	3.60E-06	1.90E-06	2.41E-06
F49E2.4	7	17	2	6	3.67E-06	4.92E-06	2.22E-06	2.38E-06
F49E2.5a.1	1196	1219	634	1130	1.24E-05	1.58E-05	6.80E-06	1.26E-05
F49E2.5a.2	1141	1184	618	1113	2.80E-06	2.75E-06	1.82E-06	2.25E-06
F49E2.5b.1	918	944	519	911	3.49E-05	3.36E-05	1.20E-05	2.65E-05
F49E2.5b.2	863	909	503	894	3.46E-05	3.39E-05	1.22E-05	2.71E-05
F49E2.5c.1	993	1022	559	988	3.39E-05	3.29E-05	1.25E-05	2.70E-05
F49E2.5c.2	938	987	543	971	3.34E-05	3.32E-05	1.27E-05	2.78E-05
F49E2.5d.1	963	977	528	936	3.39E-05	3.29E-05	1.24E-05	2.71E-05
F49E2.5d.2	908	942	512	919	3.34E-05	3.32E-05	1.26E-05	2.78E-05
F49E2.5e.1	618	613	276	516	3.77E-05	3.61E-05	1.34E-05	2.94E-05
F49E2.5e.2	540	560	247	476	3.74E-05	3.66E-05	1.37E-05	3.04E-05
F49E2.5f.1	629	634	278	527	3.31E-05	3.10E-05	9.62E-06	2.22E-05
F49E2.5f.2	574	599	262	510	3.37E-05	3.30E-05	1.00E-05	2.38E-05
F49E2.5g.1	545	546	235	445	3.47E-05	3.31E-05	9.99E-06	2.34E-05
F49E2.5g.2	490	511	219	428	3.41E-05	3.36E-05	1.01E-05	2.43E-05
F49E2.5h.1	916	929	513	901	3.43E-05	3.25E-05	9.62E-06	2.25E-05
F49E2.5h.2	861	894	497	884	3.35E-05	3.30E-05	9.75E-06	2.35E-05
F49E2.5i.1	1125	1130	589	1044	3.76E-05	3.60E-05	1.37E-05	2.97E-05
F49E2.5i.2	1070	1095	573	1027	3.73E-05	3.66E-05	1.40E-05	3.07E-05
F49E2.5j.1	1471	1546	772	1367	3.84E-05	3.64E-05	1.31E-05	2.86E-05
F49E2.5j.2	1121	1153	606	1082	3.81E-05	3.69E-05	1.33E-05	2.94E-05
F49E7.1a	92	126	108	195	3.73E-05	3.71E-05	1.28E-05	2.79E-05
F49E7.1b	14	13	13	21	3.74E-05	3.64E-05	1.32E-05	2.90E-05
F49E7.2	26	28	8	19	3.00E-06	3.89E-06	2.30E-06	5.13E-06
F49E7.t1	0	0	1	0	3.19E-06	2.80E-06	1.93E-06	3.85E-06
F49E7.t2	0	0	1	0	3.98E-06	4.05E-06	1.82E-06	2.34E-06
F49E7.t3	0	0	1	0	2.80E-06	2.65E-06	1.82E-06	2.25E-06
F49E8.1	1043	951	1295	2040	2.80E-06	2.65E-06	1.82E-06	2.25E-06
F49E8.2	370	396	453	533	2.80E-06	2.65E-06	1.82E-06	2.25E-06
F49E8.3a.1	2913	2907	3404	4037	6.54E-05	5.63E-05	5.28E-05	1.03E-04
F49E8.3a.2	2784	2734	3168	3836	3.26E-05	3.29E-05	2.60E-05	3.77E-05
F49E8.3b	2796	2742	3176	3845	1.16E-04	1.09E-04	8.82E-05	1.29E-04
F49E8.4	227	195	192	190	1.12E-04	1.04E-04	8.30E-05	1.24E-04
F49E8.5.1	436	585	525	707	1.10E-04	1.02E-04	8.13E-05	1.21E-04
F49E8.5.2	381	528	455	674	4.17E-05	3.38E-05	2.29E-05	2.80E-05
F49E8.6	40	30	39	47	4.18E-05	5.30E-05	3.28E-05	5.44E-05
F49E8.7a.1	386	358	454	576	4.14E-05	5.42E-05	3.22E-05	5.89E-05
F49E8.7a.2	319	302	376	508	5.88E-06	4.18E-06	3.74E-06	5.56E-06
F49E8.7b.1	341	320	409	545	2.46E-05	2.16E-05	1.89E-05	2.96E-05
F49E8.7b.2	319	302	376	508	2.33E-05	2.08E-05	1.79E-05	2.98E-05
F49F1.1	33	78	153	80	2.41E-05	2.14E-05	1.88E-05	3.09E-05
F49F1.10	4	0	4	2	2.33E-05	2.08E-05	1.79E-05	2.98E-05
F49F1.11	0	0	2	2	4.62E-06	1.03E-05	1.40E-05	9.02E-06
F49F1.12	8	10	1	5	2.80E-06	2.65E-06	1.82E-06	2.25E-06
F49F1.13	4	3	1	1	2.80E-06	2.65E-06	1.82E-06	2.25E-06
F49F1.14	4	3	2	5	2.80E-06	3.04E-06	1.82E-06	2.25E-06
F49F1.3	3	3	0	2	2.80E-06	2.65E-06	1.82E-06	2.25E-06
F49F1.5	36	79	51	19	2.80E-06	2.65E-06	1.82E-06	2.25E-06
F49F1.6	20	20	43	23	2.80E-06	2.65E-06	1.82E-06	2.25E-06
F49F1.7	34	43	77	26	7.62E-06	1.58E-05	7.03E-06	3.24E-06
F49F1.8	12	12	10	6	2.88E-06	2.72E-06	4.03E-06	2.65E-06
F49F1.9	3	3	1	2	4.93E-06	5.90E-06	7.27E-06	3.04E-06
F49H12.1a	114	133	108	141	2.80E-06	2.65E-06	1.82E-06	2.25E-06
F49H12.1b.1	66	95	77	103	2.80E-06	2.65E-06	1.82E-06	2.25E-06
F49H12.1b.2	63	93	75	102	7.31E-06	8.07E-06	4.52E-06	7.26E-06
F49H12.2	4	2	6	3	6.83E-06	9.28E-06	5.19E-06	8.57E-06
F49H12.3	6	11	12	4	6.58E-06	9.18E-06	5.10E-06	8.57E-06
F49H12.4	1	5	3	0	2.80E-06	2.65E-06	1.82E-06	2.25E-06

F49H12.5	493	630	175	227	2.80E-06	2.65E-06	1.99E-06	2.25E-06
F49H12.6a.1	331	452	260	476	2.80E-06	2.65E-06	1.82E-06	2.25E-06
F49H12.6a.2	259	341	220	390	5.61E-05	6.77E-05	1.30E-05	2.08E-05
F49H12.6b	319	436	255	467	1.60E-05	2.07E-05	8.20E-06	1.85E-05
F49H12.7	1	2	3	3	1.53E-05	1.90E-05	8.44E-06	1.85E-05
F49H6.1	1	1	0	2	1.54E-05	1.99E-05	8.00E-06	1.81E-05
F49H6.10	3	6	3	3	2.80E-06	2.65E-06	1.82E-06	2.25E-06
F49H6.11	2	5	3	1	2.80E-06	2.65E-06	1.82E-06	2.25E-06
F49H6.12	0	4	5	0	2.80E-06	2.65E-06	1.82E-06	2.25E-06
F49H6.13	2	4	2	0	2.80E-06	2.65E-06	1.82E-06	2.25E-06
F49H6.2	6	4	5	4	2.80E-06	2.65E-06	1.82E-06	2.25E-06
F49H6.3	0	1	1	0	2.80E-06	2.65E-06	1.82E-06	2.25E-06
F49H6.4	3	4	14	1	2.80E-06	2.65E-06	1.82E-06	2.25E-06
F49H6.5	9	18	7	3	2.80E-06	2.65E-06	1.82E-06	2.25E-06
F49H6.6	5	7	9	3	2.80E-06	2.65E-06	1.82E-06	2.25E-06
F49H6.7	2	6	0	1	2.80E-06	2.65E-06	1.82E-06	2.25E-06
F49H6.8	2	0	0	0	2.80E-06	2.65E-06	1.82E-06	2.25E-06
F49H6.9	1	6	6	5	2.80E-06	2.65E-06	1.82E-06	2.25E-06
F52A8.1.1	839	1271	1099	682	2.80E-06	2.65E-06	1.82E-06	2.25E-06
F52A8.1.2	668	995	743	556	2.80E-06	2.65E-06	1.82E-06	2.25E-06
F52A8.2	80	168	100	107	1.52E-04	2.18E-04	1.30E-04	9.94E-05
F52A8.3a	6	7	4	1	1.40E-04	1.96E-04	1.01E-04	9.33E-05
F52A8.3b	6	7	3	0	6.27E-06	1.25E-05	5.10E-06	6.75E-06
F52A8.4	17	33	26	14	2.80E-06	2.65E-06	1.82E-06	2.25E-06
F52A8.5.1	329	407	279	254	2.80E-06	2.65E-06	1.82E-06	2.25E-06
F52A8.5.2	347	414	269	246	2.80E-06	3.54E-06	1.93E-06	2.25E-06
F52A8.6a	290	381	398	554	6.74E-05	7.87E-05	3.72E-05	4.18E-05
F52A8.6b	103	100	161	163	6.75E-05	7.60E-05	3.40E-05	3.84E-05
F52A8.6c.1	204	231	252	394	2.35E-05	2.91E-05	2.09E-05	3.60E-05
F52A8.6c.2	215	247	283	408	1.58E-05	1.45E-05	1.61E-05	2.01E-05
F52B10.1	2213	2868	1445	3363	2.73E-05	2.92E-05	2.19E-05	4.23E-05
F52B10.2	4	5	1	6	2.59E-05	2.81E-05	2.22E-05	3.94E-05
F52B10.3	10	14	7	12	3.88E-05	4.75E-05	1.65E-05	4.74E-05
F52B11.1a.1	343	343	456	507	2.80E-06	2.65E-06	1.82E-06	2.25E-06
F52B11.1a.2	355	366	445	511	2.80E-06	2.65E-06	1.82E-06	2.25E-06
F52B11.1a.3	323	302	423	463	2.09E-05	1.97E-05	1.80E-05	2.48E-05
F52B11.1b	237	226	311	367	1.97E-05	1.92E-05	1.60E-05	2.27E-05
F52B11.2	115	137	131	168	2.35E-05	2.08E-05	2.01E-05	2.71E-05
F52B11.4	1434	1338	1244	862	2.12E-05	1.91E-05	1.81E-05	2.64E-05
F52B11.5	18	19	14	4	1.90E-05	2.14E-05	1.41E-05	2.23E-05
F52B11.6	30	27	28	18	1.76E-04	1.55E-04	9.91E-05	8.47E-05
F52B5.1	1206	1322	1450	2148	3.72E-06	3.73E-06	1.90E-06	2.25E-06
F52B5.2	468	567	437	622	8.06E-06	6.85E-06	4.90E-06	3.89E-06
F52B5.3	852	832	1031	1527	3.58E-05	3.71E-05	2.80E-05	5.12E-05
F52B5.5a.1	376	392	498	644	4.36E-05	4.99E-05	2.65E-05	4.65E-05
F52B5.5a.2	372	384	487	638	2.12E-05	1.96E-05	1.67E-05	3.06E-05
F52B5.5b.1	151	162	180	215	1.99E-05	1.95E-05	1.71E-05	2.73E-05
F52B5.5b.2	372	384	487	638	2.00E-05	1.95E-05	1.70E-05	2.76E-05
F52B5.6.1	10456	7000	20154	5445	2.17E-05	2.20E-05	1.69E-05	2.49E-05
F52B5.6.2	8589	5867	15524	4905	2.00E-05	1.95E-05	1.70E-05	2.76E-05
F52B5.7	34	45	59	67	2.35E-03	1.49E-03	2.95E-03	9.84E-04
F52C12.2	291	219	245	297	1.94E-03	1.25E-03	2.29E-03	8.91E-04
F52C12.3	31	38	47	80	4.34E-06	5.42E-06	4.90E-06	6.88E-06
F52C12.4	257	226	448	777	3.50E-05	2.49E-05	1.92E-05	2.87E-05
F52C12.5.1	32	40	21	36	3.05E-06	3.52E-06	3.01E-06	6.32E-06
F52C6.1	20	16	15	24	5.85E-06	4.87E-06	6.65E-06	1.42E-05
F52C6.10	27	40	33	50	2.80E-06	2.83E-06	1.82E-06	2.25E-06
F52C6.11	64	91	87	90	3.22E-06	2.65E-06	1.82E-06	3.10E-06
F52C6.12.1	218	206	195	299	3.33E-06	4.68E-06	2.66E-06	4.97E-06

F52C6.12.2	109	97	94	143	7.84E-06	1.05E-05	6.92E-06	8.84E-06
F52C6.13	32	30	24	6	5.40E-05	4.82E-05	3.15E-05	5.95E-05
F52C6.14	6	15	6	4	3.45E-05	2.90E-05	1.94E-05	3.63E-05
F52C6.2.1	108	90	103	120	8.01E-06	7.09E-06	3.92E-06	2.25E-06
F52C6.2.2	82	67	91	91	2.80E-06	2.65E-06	1.82E-06	2.25E-06
F52C6.2.3	81	65	90	90	1.80E-05	1.42E-05	1.12E-05	1.61E-05
F52C6.2.4	103	84	102	116	1.71E-05	1.32E-05	1.23E-05	1.52E-05
F52C6.3	47	33	29	39	1.71E-05	1.30E-05	1.24E-05	1.53E-05
F52C6.4	30	27	50	34	1.75E-05	1.35E-05	1.13E-05	1.59E-05
F52C6.5	4	5	1	0	6.33E-06	4.21E-06	2.55E-06	4.23E-06
F52C6.6	2	0	0	2	3.84E-06	3.25E-06	4.15E-06	3.49E-06
F52C6.7	15	24	7	11	2.80E-06	2.65E-06	1.82E-06	2.25E-06
F52C6.8	52	56	83	62	2.80E-06	2.65E-06	1.82E-06	2.25E-06
F52C6.9	17	15	13	27	2.80E-06	2.75E-06	1.82E-06	2.25E-06
F52C9.1a	162	210	263	292	6.33E-06	6.43E-06	6.56E-06	6.05E-06
F52C9.1b	156	200	255	282	2.80E-06	2.65E-06	1.82E-06	2.63E-06
F52C9.3	226	224	230	270	4.26E-06	5.21E-06	4.50E-06	6.16E-06
F52C9.5	14	33	15	17	4.37E-06	5.29E-06	4.65E-06	6.34E-06
F52C9.8a	669	574	871	1322	1.86E-05	1.74E-05	1.23E-05	1.78E-05
F52C9.8b	1036	949	1291	2074	2.80E-06	2.65E-06	1.82E-06	2.25E-06
F52C9.8c	606	587	691	1191	1.97E-05	1.59E-05	1.67E-05	3.12E-05
F52C9.8d.1	443	442	542	948	2.34E-05	2.03E-05	1.90E-05	3.76E-05
F52C9.8d.2	502	475	583	1014	2.84E-05	2.60E-05	2.11E-05	4.49E-05
F52C9.8e	439	438	529	943	2.71E-05	2.56E-05	2.16E-05	4.66E-05
F52C9.8f	304	311	335	605	2.77E-05	2.48E-05	2.10E-05	4.50E-05
F52C9.8g	479	460	555	982	2.72E-05	2.56E-05	2.13E-05	4.69E-05
F52C9.t1	0	0	1	0	2.56E-05	2.47E-05	1.84E-05	4.09E-05
F52D1.1	579	754	393	430	2.66E-05	2.41E-05	2.00E-05	4.38E-05
F52D1.2	8	9	5	3	2.80E-06	2.65E-06	1.82E-06	2.25E-06
F52D1.3	160	186	55	50	2.28E-05	2.80E-05	1.01E-05	1.36E-05
F52D10.1	40	58	43	48	2.80E-06	2.65E-06	1.82E-06	2.25E-06
F52D10.2	80	102	95	108	6.80E-06	7.46E-06	1.82E-06	2.25E-06
F52D10.3a.1	2447	2968	2477	3401	2.80E-06	2.78E-06	1.82E-06	2.25E-06
F52D10.3a.2	2140	2607	2041	2869	5.82E-06	7.01E-06	4.50E-06	6.32E-06
F52D10.3b.1	2569	3129	2581	3416	2.44E-04	2.80E-04	1.61E-04	2.73E-04
F52D10.3b.2	1292	1540	1259	1626	2.48E-04	2.85E-04	1.54E-04	2.67E-04
F52D10.4	5	6	2	1	2.18E-04	2.51E-04	1.43E-04	2.33E-04
F52D10.5	1	3	3	1	2.36E-04	2.66E-04	1.50E-04	2.39E-04
F52D10.6	23	28	17	21	2.80E-06	2.65E-06	1.82E-06	2.25E-06
F52D2.1	11	13	8	6	2.80E-06	2.65E-06	1.82E-06	2.25E-06
F52D2.10	1	2	5	3	2.80E-06	2.65E-06	1.82E-06	2.25E-06
F52D2.2	99	140	193	146	2.80E-06	2.65E-06	1.82E-06	2.25E-06
F52D2.4	336	305	551	641	2.80E-06	2.65E-06	1.82E-06	2.25E-06
F52D2.5	1	1	2	1	1.61E-05	2.15E-05	2.04E-05	1.90E-05
F52D2.6	179	137	231	337	1.38E-05	1.19E-05	1.48E-05	2.12E-05
F52D2.8a	2	3	3	1	2.80E-06	2.65E-06	1.82E-06	2.25E-06
F52D2.8b	3	4	5	1	6.80E-06	4.92E-06	5.72E-06	1.03E-05
F52D2.9	12	2	6	3	2.80E-06	2.65E-06	1.82E-06	2.25E-06
F52E1.1	4128	3294	6104	6764	2.80E-06	2.65E-06	1.82E-06	2.25E-06
F52E1.10	404	455	506	656	2.80E-06	2.65E-06	1.82E-06	2.25E-06
F52E1.12	7	5	10	4	4.14E-04	3.12E-04	3.98E-04	5.44E-04
F52E1.13a	1039	1042	1105	1567	3.10E-05	3.30E-05	2.53E-05	4.05E-05
F52E1.13b.1	1224	1245	1382	2115	2.80E-06	2.65E-06	1.82E-06	2.25E-06
F52E1.13b.2	738	767	764	1158	4.61E-05	4.37E-05	3.19E-05	5.59E-05
F52E1.13c	1033	1031	1097	1558	6.57E-05	6.32E-05	4.83E-05	9.13E-05
F52E1.13d	665	656	666	900	6.15E-05	6.04E-05	4.14E-05	7.75E-05
F52E1.14	718	1026	799	716	4.63E-05	4.37E-05	3.20E-05	5.62E-05
F52E1.2	25	14	20	12	2.53E-05	2.36E-05	1.65E-05	2.76E-05
F52E1.3	33	24	19	12	1.18E-04	1.59E-04	8.53E-05	9.43E-05

F52E1.4a	5	18	13	13	4.56E-06	2.65E-06	2.37E-06	2.25E-06
F52E1.4b	5	18	14	15	5.12E-06	3.52E-06	1.93E-06	2.25E-06
F52E1.5	23	56	11	17	2.80E-06	2.65E-06	1.82E-06	2.25E-06
F52E1.7a	155	300	221	167	2.80E-06	2.65E-06	1.82E-06	2.25E-06
F52E1.7b	136	272	162	144	3.39E-06	7.78E-06	1.82E-06	2.25E-06
F52E1.8	11	11	4	6	1.99E-05	3.64E-05	1.85E-05	1.72E-05
F52E1.9	71	92	56	54	2.83E-05	5.35E-05	2.19E-05	2.41E-05
F52E10.1	93	105	117	165	2.80E-06	2.65E-06	1.82E-06	2.25E-06
F52E10.2	3	2	0	0	8.37E-06	1.03E-05	4.30E-06	5.13E-06
F52E10.3	3	2	3	5	2.80E-06	2.96E-06	2.28E-06	3.96E-06
F52E10.4	9	17	4	8	2.80E-06	2.65E-06	1.82E-06	2.25E-06
F52E10.5	59	52	35	70	2.80E-06	2.65E-06	1.82E-06	2.25E-06
F52E4.1a.1	1286	1510	1447	1752	5.01E-06	8.94E-06	1.82E-06	3.58E-06
F52E4.1a.2	1251	1435	1379	1716	3.33E-06	2.78E-06	1.82E-06	3.17E-06
F52E4.1b	1338	1563	1454	1833	8.09E-05	8.97E-05	5.93E-05	8.85E-05
F52E4.4	12	28	19	32	8.64E-05	9.37E-05	6.20E-05	9.52E-05
F52E4.5	21	93	16	30	8.02E-05	8.85E-05	5.67E-05	8.83E-05
F52E4.6	186	295	101	73	2.80E-06	2.65E-06	1.82E-06	2.25E-06
F52E4.7	115	215	149	175	2.80E-06	8.68E-06	1.82E-06	2.38E-06
F52E4.8	60	56	48	67	1.34E-05	2.01E-05	4.74E-06	4.23E-06
F52F10.1	8	15	7	11	4.65E-06	8.20E-06	3.92E-06	5.69E-06
F52F10.2	40	73	20	20	5.85E-06	5.16E-06	3.04E-06	5.24E-06
F52F10.3	16	28	17	13	2.80E-06	2.65E-06	1.82E-06	2.25E-06
F52F10.4	17	13	16	4	2.80E-06	4.10E-06	1.82E-06	2.25E-06
F52F10.5	2	6	10	8	2.80E-06	2.65E-06	1.82E-06	2.25E-06
F52F12.1a	25	43	37	33	2.80E-06	2.65E-06	1.82E-06	2.25E-06
F52F12.1b	22	41	35	32	2.80E-06	2.65E-06	1.82E-06	2.25E-06
F52F12.2	14	7	12	10	2.80E-06	2.65E-06	1.82E-06	2.25E-06
F52F12.3	323	363	435	597	2.80E-06	2.65E-06	1.82E-06	2.25E-06
F52F12.4	525	495	844	858	2.80E-06	2.65E-06	1.82E-06	2.25E-06
F52F12.5	26	60	7	4	1.72E-05	1.82E-05	1.50E-05	2.55E-05
F52F12.6	51	52	81	98	5.90E-05	5.26E-05	6.18E-05	7.75E-05
F52F12.7	329	384	410	398	6.52E-06	1.42E-05	1.82E-06	2.25E-06
F52F12.8	6	20	7	4	3.42E-06	3.31E-06	3.53E-06	5.29E-06
F52F12.9	4	6	9	10	4.79E-05	5.28E-05	3.89E-05	4.66E-05
F52G2.1a	512	529	684	977	2.80E-06	5.69E-06	1.82E-06	2.25E-06
F52G2.1b	474	503	650	932	2.80E-06	2.65E-06	1.82E-06	2.25E-06
F52G2.2a	977	859	1050	1564	2.19E-05	2.14E-05	1.90E-05	3.36E-05
F52G2.3	56	80	40	71	2.05E-05	2.06E-05	1.83E-05	3.24E-05
F52G3.1	377	434	383	552	2.88E-05	2.39E-05	2.02E-05	3.70E-05
F52G3.3	28	49	48	35	3.19E-06	4.31E-06	1.82E-06	3.26E-06
F52G3.4	10	22	14	9	1.07E-05	1.16E-05	7.07E-06	1.26E-05
F52G3.5	15	19	12	7	2.80E-06	2.65E-06	1.82E-06	2.25E-06
F52H2.1	104	99	146	197	2.80E-06	2.65E-06	1.82E-06	2.25E-06
F52H2.2	126	121	169	192	2.80E-06	2.65E-06	1.82E-06	2.25E-06
F52H2.3	16	22	5	9	5.15E-06	4.66E-06	4.72E-06	7.85E-06
F52H2.4	10	17	7	19	8.18E-06	7.41E-06	7.12E-06	9.99E-06
F52H2.5	36	49	39	41	4.23E-06	5.50E-06	1.82E-06	2.25E-06
F52H2.6	107	118	184	220	2.80E-06	2.65E-06	1.82E-06	2.25E-06
F52H2.7	48	70	63	100	4.42E-06	5.69E-06	3.12E-06	4.05E-06
F52H3.1.1	191	217	158	200	6.08E-06	6.35E-06	6.81E-06	1.01E-05
F52H3.1.2	171	205	142	190	2.80E-06	2.70E-06	1.82E-06	3.26E-06
F52H3.1.3	171	206	141	192	9.18E-06	9.87E-06	4.94E-06	7.71E-06
F52H3.3	77	124	71	63	8.65E-06	9.79E-06	4.68E-06	7.71E-06
F52H3.4	145	153	194	204	8.40E-06	9.58E-06	4.52E-06	7.58E-06
F52H3.5	70	181	96	82	6.50E-06	9.87E-06	3.90E-06	4.27E-06
F52H3.6	32	84	24	21	1.33E-05	1.33E-05	1.16E-05	1.51E-05
F52H3.7a	1799	2189	1718	2844	1.27E-05	3.10E-05	1.13E-05	1.19E-05
F52H3.7b.1	1770	2046	1826	2835	3.61E-06	8.97E-06	1.82E-06	2.25E-06

F52H3.7b.2	1568	1653	1587	2167	5.15E-05	5.92E-05	3.20E-05	6.54E-05
F53A10.2a	117	194	151	185	6.76E-05	7.39E-05	4.54E-05	8.70E-05
F53A10.2b	71	104	92	94	1.72E-04	1.72E-04	1.14E-04	1.91E-04
F53A10.2c	82	156	105	149	5.32E-06	8.33E-06	4.48E-06	6.77E-06
F53A2.1	27	45	56	38	5.07E-06	7.04E-06	4.28E-06	5.40E-06
F53A2.2	3	6	6	1	3.78E-06	6.77E-06	3.13E-06	5.51E-06
F53A2.3	19	26	29	28	2.80E-06	2.65E-06	1.82E-06	2.25E-06
F53A2.4	451	506	508	516	2.80E-06	2.65E-06	1.82E-06	2.25E-06
F53A2.5	219	155	459	513	2.80E-06	2.86E-06	2.19E-06	2.61E-06
F53A2.6.1	692	774	1446	864	4.96E-05	5.25E-05	3.63E-05	4.56E-05
F53A2.6.2	652	742	1138	778	4.42E-05	2.95E-05	6.03E-05	8.31E-05
F53A2.6.3	596	669	1059	702	6.52E-05	6.89E-05	8.87E-05	6.54E-05
F53A2.7.1	650	1139	643	989	1.01E-04	1.09E-04	1.15E-04	9.68E-05
F53A2.7.2	711	1196	700	1016	1.04E-04	1.10E-04	1.20E-04	9.85E-05
F53A2.8a	306	325	392	482	5.39E-05	8.93E-05	3.47E-05	6.59E-05
F53A2.8b.1	303	321	363	468	5.90E-05	9.38E-05	3.78E-05	6.78E-05
F53A2.8b.2	303	320	363	468	1.67E-05	1.68E-05	1.39E-05	2.11E-05
F53A2.8c	341	348	426	522	1.39E-05	1.40E-05	1.09E-05	1.73E-05
F53A2.9	21	28	36	44	1.43E-05	1.43E-05	1.12E-05	1.78E-05
F53A3.1	10	11	26	9	1.69E-05	1.63E-05	1.37E-05	2.08E-05
F53A3.2	241	214	334	470	2.80E-06	2.65E-06	2.06E-06	3.10E-06
F53A3.3.1	4071	4423	12540	3270	2.80E-06	2.65E-06	3.35E-06	2.25E-06
F53A3.3.2	4195	4545	13216	3321	1.54E-05	1.29E-05	1.39E-05	2.41E-05
F53A3.3.3	4764	5120	14488	3495	9.50E-04	9.75E-04	1.90E-03	6.13E-04
F53A3.4	215	178	227	330	9.95E-04	1.02E-03	2.04E-03	6.33E-04
F53A3.5	66	44	55	76	1.13E-03	1.15E-03	2.24E-03	6.66E-04
F53A3.6	137	93	124	186	1.28E-05	1.01E-05	8.82E-06	1.58E-05
F53A3.7	29	32	122	30	4.23E-06	2.67E-06	2.30E-06	3.91E-06
F53A9.1	55	137	180	28	1.23E-05	7.94E-06	7.27E-06	1.35E-05
F53A9.10a.1	2941	2873	2640	4146	8.62E-06	8.99E-06	2.36E-05	7.15E-06
F53A9.10a.2	2808	2789	2520	4072	2.63E-05	6.19E-05	5.61E-05	1.08E-05
F53A9.10b.1	3196	3304	3122	4674	2.54E-04	2.34E-04	1.48E-04	2.88E-04
F53A9.10b.2	2971	3123	2899	4486	2.43E-04	2.28E-04	1.42E-04	2.83E-04
F53A9.10b.3	2875	3038	2826	4388	2.38E-04	2.33E-04	1.51E-04	2.80E-04
F53A9.10b.4	2808	2789	2520	4072	2.22E-04	2.20E-04	1.41E-04	2.69E-04
F53A9.2	7	17	7	5	1.90E-04	1.89E-04	1.21E-04	2.32E-04
F53A9.3	4	1	3	2	2.43E-04	2.28E-04	1.42E-04	2.83E-04
F53A9.4	13	15	13	14	3.11E-06	7.14E-06	2.02E-06	2.25E-06
F53A9.5	1	7	4	4	2.80E-06	2.65E-06	1.82E-06	2.25E-06
F53A9.7	24	85	46	43	2.80E-06	2.65E-06	1.82E-06	2.25E-06
F53A9.8	88	351	288	66	2.80E-06	2.65E-06	1.82E-06	2.25E-06
F53A9.9	7	24	26	7	7.14E-06	2.39E-05	8.89E-06	1.03E-05
F53B1.3	4	5	2	2	2.57E-05	9.67E-05	5.47E-05	1.55E-05
F53B1.4	470	509	148	165	2.80E-06	5.71E-06	4.26E-06	2.25E-06
F53B1.6	41	43	38	20	2.80E-06	2.65E-06	1.82E-06	2.25E-06
F53B1.7	6	2	2	3	5.12E-05	5.23E-05	1.05E-05	1.44E-05
F53B1.8	237	227	271	289	3.14E-06	3.09E-06	1.90E-06	2.25E-06
F53B1.9	7	4	1	3	2.80E-06	2.65E-06	1.82E-06	2.25E-06
F53B1.t1	0	1	4	1	1.07E-05	9.68E-06	7.96E-06	1.05E-05
F53B2.1	30	32	24	27	4.93E-06	2.67E-06	1.82E-06	2.25E-06
F53B2.2	4	2	3	1	2.80E-06	2.65E-06	4.05E-06	2.25E-06
F53B2.3	1	5	2	0	3.75E-06	3.78E-06	1.95E-06	2.70E-06
F53B2.4	5	9	11	3	2.80E-06	2.65E-06	1.82E-06	2.25E-06
F53B2.5	18	44	13	3	2.80E-06	2.65E-06	1.82E-06	2.25E-06
F53B2.6	55	52	56	85	2.80E-06	2.65E-06	1.82E-06	2.25E-06
F53B2.7	20	41	16	38	2.80E-06	3.47E-06	1.82E-06	2.25E-06
F53B2.8	37	160	19	25	3.39E-06	3.04E-06	2.26E-06	4.23E-06
F53B2.9	7	7	9	5	2.80E-06	4.79E-06	1.82E-06	3.78E-06
F53B3.1	114	118	114	176	5.29E-06	2.16E-05	1.82E-06	2.88E-06

F53B3.3	21	10	7	7	2.80E-06	2.65E-06	1.82E-06	2.25E-06
F53B3.5	123	85	98	170	6.50E-06	6.35E-06	4.23E-06	8.05E-06
F53B3.6	126	231	133	120	2.80E-06	2.65E-06	1.82E-06	2.25E-06
F53B6.1	39	92	38	84	5.68E-06	3.70E-06	2.95E-06	6.30E-06
F53B6.2a	59	74	52	54	1.29E-05	2.22E-05	8.82E-06	9.83E-06
F53B6.2b	49	71	52	46	4.03E-06	8.99E-06	2.55E-06	6.97E-06
F53B6.4	102	241	51	55	2.80E-06	2.65E-06	1.82E-06	2.25E-06
F53B6.5	17	11	10	19	2.80E-06	3.17E-06	1.82E-06	2.25E-06
F53B6.6	6	16	16	8	1.08E-05	2.41E-05	3.52E-06	4.68E-06
F53B6.7	24	58	41	18	3.47E-06	2.65E-06	1.82E-06	3.10E-06
F53B6.8	2	6	0	1	2.80E-06	2.65E-06	1.82E-06	2.25E-06
F53B6.9	34	146	28	42	2.83E-06	6.45E-06	3.13E-06	2.25E-06
F53B6.t1	1	0	3	0	2.80E-06	2.65E-06	1.82E-06	2.25E-06
F53B7.2	33	36	29	19	1.19E-05	4.84E-05	6.40E-06	1.19E-05
F53B7.3	247	265	322	254	2.80E-06	2.65E-06	2.99E-06	2.25E-06
F53B7.4	6	6	9	0	2.80E-06	2.65E-06	1.82E-06	2.25E-06
F53B7.7	4	8	5	7	3.12E-05	3.16E-05	2.65E-05	2.58E-05
F53C11.1	107	115	139	56	2.80E-06	2.65E-06	1.82E-06	2.25E-06
F53C11.2	11	12	1630	238	2.80E-06	2.65E-06	1.82E-06	2.25E-06
F53C11.3	230	423	262	470	7.67E-06	7.80E-06	6.49E-06	3.22E-06
F53C11.4.1	760	745	1037	1412	2.80E-06	2.65E-06	5.71E-05	1.03E-05
F53C11.4.2	490	484	689	1016	2.35E-05	4.08E-05	1.74E-05	3.85E-05
F53C11.5a	1054	1046	1267	1809	3.06E-05	2.83E-05	2.72E-05	4.57E-05
F53C11.5b	963	964	1120	1656	3.41E-05	3.18E-05	3.12E-05	5.67E-05
F53C11.5c	942	936	1108	1640	4.11E-05	3.85E-05	3.22E-05	5.67E-05
F53C11.6	8	12	9	9	3.88E-05	3.66E-05	2.93E-05	5.35E-05
F53C11.7.1	196	181	239	275	4.22E-05	3.96E-05	3.23E-05	5.90E-05
F53C11.7.2	148	139	185	216	2.80E-06	2.65E-06	1.82E-06	2.25E-06
F53C11.8.1	152	139	151	220	1.04E-05	9.10E-06	8.29E-06	1.18E-05
F53C11.8.2	148	135	143	218	7.59E-06	6.74E-06	6.18E-06	8.91E-06
F53C3.1	19	26	17	12	1.09E-05	9.44E-06	7.07E-06	1.27E-05
F53C3.10	9	3	5	4	1.39E-05	1.20E-05	8.73E-06	1.64E-05
F53C3.11	5	4	12	6	2.80E-06	2.78E-06	1.82E-06	2.25E-06
F53C3.12	36	37	42	33	2.80E-06	2.65E-06	1.82E-06	2.25E-06
F53C3.13a	420	403	550	769	2.80E-06	2.65E-06	1.82E-06	2.25E-06
F53C3.13b.1	412	393	537	740	2.80E-06	2.65E-06	1.86E-06	2.25E-06
F53C3.13b.2	412	391	528	739	4.02E-05	3.65E-05	3.43E-05	5.92E-05
F53C3.13c.1	498	488	701	918	3.32E-05	3.00E-05	2.82E-05	4.80E-05
F53C3.13c.2	419	401	535	755	3.50E-05	3.14E-05	2.92E-05	5.05E-05
F53C3.2	8	6	7	9	3.74E-05	3.46E-05	3.42E-05	5.53E-05
F53C3.3	5	8	4	10	4.28E-05	3.87E-05	3.55E-05	6.19E-05
F53C3.4	6	10	10	11	2.80E-06	2.65E-06	1.82E-06	2.25E-06
F53C3.5	1	11	5	5	2.80E-06	2.65E-06	1.82E-06	2.25E-06
F53C3.6a.1	9	13	6	9	2.80E-06	2.65E-06	1.82E-06	2.25E-06
F53C3.6a.2	7	9	3	8	2.80E-06	2.65E-06	1.82E-06	2.25E-06
F53C3.6b.1	8	9	3	9	2.80E-06	2.65E-06	1.82E-06	2.25E-06
F53C3.6b.2	7	9	3	8	2.80E-06	2.65E-06	1.82E-06	2.25E-06
F53C3.7	7	3	9	5	2.80E-06	2.65E-06	1.82E-06	2.25E-06
F53C3.8	4	6	4	0	2.80E-06	2.65E-06	1.82E-06	2.25E-06
F53E10.2a	4	9	8	7	2.80E-06	2.65E-06	1.82E-06	2.25E-06
F53E10.2b	4	10	9	9	2.80E-06	2.65E-06	1.82E-06	2.25E-06
F53E10.3	7	7	28	12	2.80E-06	2.65E-06	1.82E-06	2.25E-06
F53E10.4	61	31	46	17	2.80E-06	2.65E-06	1.82E-06	2.25E-06
F53E10.5	7	13	8	5	3.22E-06	3.04E-06	8.40E-06	4.45E-06
F53E10.6.1	201	162	204	180	1.00E-05	4.79E-06	4.90E-06	2.25E-06
F53E10.6.2	175	138	172	166	2.80E-06	2.65E-06	1.82E-06	2.25E-06
F53E2.1	49	98	64	93	3.63E-05	2.76E-05	2.39E-05	2.61E-05
F53F1.1	3	3	7	1	3.39E-05	2.52E-05	2.16E-05	2.58E-05
F53F1.10	5	6	4	1	4.03E-06	7.64E-06	3.44E-06	6.16E-06

F53F1.11	4	1	3	1	2.80E-06	2.65E-06	1.82E-06	2.25E-06
F53F1.2	290	503	460	501	2.80E-06	2.65E-06	1.82E-06	2.25E-06
F53F1.3	101	136	74	113	2.80E-06	2.65E-06	1.82E-06	2.25E-06
F53F1.4	2523	2070	1416	686	3.14E-05	5.15E-05	3.25E-05	4.36E-05
F53F1.5	1073	672	600	294	1.27E-05	1.61E-05	6.03E-06	1.14E-05
F53F1.6	28	44	18	17	5.38E-04	4.17E-04	1.97E-04	1.18E-04
F53F1.7	8	11	5	2	1.38E-04	8.16E-05	5.02E-05	3.04E-05
F53F1.8	5	10	19	9	3.39E-06	5.03E-06	1.82E-06	2.25E-06
F53F1.9	10	8	6	6	2.80E-06	2.65E-06	1.82E-06	2.25E-06
F53F10.1	16	29	18	11	2.80E-06	2.65E-06	1.82E-06	2.25E-06
F53F10.2a.1	1074	1537	1156	1770	2.80E-06	2.65E-06	1.82E-06	2.25E-06
F53F10.2a.2	860	1253	868	1359	2.80E-06	4.02E-06	1.82E-06	2.25E-06
F53F10.2b.1	805	1125	886	1279	4.97E-05	6.72E-05	3.48E-05	6.58E-05
F53F10.2b.2	527	789	557	848	4.02E-05	5.53E-05	2.64E-05	5.10E-05
F53F10.2b.3	577	820	627	964	7.02E-05	9.27E-05	5.03E-05	8.96E-05
F53F10.2b.4	478	703	482	748	2.80E-05	3.96E-05	1.93E-05	3.62E-05
F53F10.5.1	668	645	1409	1684	4.47E-05	6.01E-05	3.17E-05	6.00E-05
F53F10.5.2	612	591	1353	1629	4.54E-05	6.30E-05	2.98E-05	5.70E-05
F53F10.6	44	36	70	55	2.84E-05	2.59E-05	3.89E-05	5.74E-05
F53F10.7	16	31	45	32	2.83E-05	2.58E-05	4.07E-05	6.05E-05
F53F10.8	38	63	53	57	1.05E-05	8.09E-06	1.08E-05	1.05E-05
F53F4.1	2	3	0	5	5.35E-06	9.79E-06	9.78E-06	8.59E-06
F53F4.10.1	929	1415	1009	891	1.11E-05	1.74E-05	1.01E-05	1.34E-05
F53F4.10.2	810	1238	837	819	2.80E-06	2.65E-06	1.82E-06	2.25E-06
F53F4.11.1	769	899	825	1092	1.22E-04	1.75E-04	8.61E-05	9.39E-05
F53F4.12	163	181	234	311	1.23E-04	1.77E-04	8.27E-05	9.98E-05
F53F4.14.1	254	340	208	403	4.73E-05	5.22E-05	3.30E-05	5.40E-05
F53F4.14.2	242	323	216	415	1.56E-05	1.63E-05	1.46E-05	2.39E-05
F53F4.15	3	8	0	0	1.69E-05	2.14E-05	9.04E-06	2.16E-05
F53F4.16	182	217	558	177	1.71E-05	2.15E-05	9.91E-06	2.35E-05
F53F4.2	3	5	2	3	2.80E-06	2.88E-06	1.82E-06	2.25E-06
F53F4.3	239	259	210	278	5.41E-05	6.09E-05	1.08E-04	4.22E-05
F53F4.4	7	12	16	11	2.80E-06	2.65E-06	1.82E-06	2.25E-06
F53F4.5.1	302	458	188	285	3.88E-05	3.97E-05	2.22E-05	3.63E-05
F53F4.5.2	276	416	168	267	2.80E-06	2.65E-06	1.82E-06	2.25E-06
F53F4.6	9	8	13	10	1.69E-05	2.42E-05	6.85E-06	1.28E-05
F53F4.7	1	9	11	8	1.65E-05	2.35E-05	6.52E-06	1.28E-05
F53F4.8	18	16	14	8	2.80E-06	2.65E-06	1.82E-06	2.25E-06
F53F4.9	2	3	3	3	2.80E-06	2.65E-06	1.82E-06	2.25E-06
F53F8.1	4	12	11	9	2.80E-06	2.65E-06	1.82E-06	2.25E-06
F53F8.2	2	1	3	1	2.80E-06	2.65E-06	1.82E-06	2.25E-06
F53F8.3	98	119	155	133	2.80E-06	2.65E-06	1.82E-06	2.25E-06
F53F8.4	55	99	78	76	2.80E-06	2.65E-06	1.82E-06	2.25E-06
F53F8.5	173	123	269	397	1.63E-05	1.87E-05	1.68E-05	1.78E-05
F53F8.6	3	3	8	1	8.06E-06	1.37E-05	7.45E-06	8.95E-06
F53G12.1	3044	3097	2207	3137	1.75E-05	1.17E-05	1.77E-05	3.23E-05
F53G12.10.1	10651	10314	12695	12076	2.80E-06	2.65E-06	1.82E-06	2.25E-06
F53G12.10.2	10538	10159	12639	12001	2.77E-04	2.66E-04	1.31E-04	2.29E-04
F53G12.11	0	1	0	0	1.45E-03	1.33E-03	1.13E-03	1.32E-03
F53G12.12	3	1	1	1	1.57E-03	1.43E-03	1.23E-03	1.44E-03
F53G12.3	63	93	61	94	2.80E-06	2.65E-06	1.82E-06	2.25E-06
F53G12.4	11	4	6	6	2.80E-06	2.65E-06	1.82E-06	2.25E-06
F53G12.5a.1	1706	1224	2379	2807	2.80E-06	2.65E-06	1.82E-06	2.25E-06
F53G12.5a.2	1260	915	1827	2047	2.80E-06	2.65E-06	1.82E-06	2.25E-06
F53G12.5b	1958	1459	2646	3452	1.29E-04	8.76E-05	1.17E-04	1.71E-04
F53G12.6	23	45	19	12	1.13E-04	7.75E-05	1.07E-04	1.47E-04
F53G12.7	18	22	10	16	1.10E-04	7.75E-05	9.68E-05	1.56E-04
F53G12.8	38	33	27	18	2.80E-06	3.09E-06	1.82E-06	2.25E-06
F53G12.9	1	3	4	0	2.80E-06	2.65E-06	1.82E-06	2.25E-06

F53G2.1	10	10	15	5	7.98E-06	6.53E-06	3.68E-06	3.04E-06
F53G2.2	2	7	7	5	2.80E-06	2.65E-06	1.82E-06	2.25E-06
F53G2.3	6	3	6	1	2.80E-06	2.65E-06	1.82E-06	2.25E-06
F53G2.4a	11	13	18	18	2.80E-06	2.65E-06	1.82E-06	2.25E-06
F53G2.4b	10	10	6	14	2.80E-06	2.65E-06	1.82E-06	2.25E-06
F53G2.6	241	238	433	412	2.80E-06	2.65E-06	1.82E-06	2.25E-06
F53G2.7.1	469	538	616	789	2.80E-06	2.65E-06	1.82E-06	2.25E-06
F53G2.7.2	388	465	494	728	8.48E-06	7.94E-06	9.93E-06	1.17E-05
F53G2.8	2	6	9	11	2.84E-05	3.08E-05	2.43E-05	3.84E-05
F53H1.1	440	497	279	651	2.71E-05	3.07E-05	2.24E-05	4.08E-05
F53H1.2	2	3	1	4	2.80E-06	2.65E-06	1.82E-06	2.43E-06
F53H1.3	182	151	232	326	1.63E-05	1.74E-05	6.71E-06	1.93E-05
F53H1.4a	538	478	540	1134	2.80E-06	2.65E-06	1.82E-06	2.25E-06
F53H1.4b	149	136	105	247	1.13E-05	8.83E-06	9.35E-06	1.62E-05
F53H1.4c	185	148	112	270	1.45E-05	1.22E-05	9.48E-06	2.46E-05
F53H10.2	316	525	385	659	1.61E-05	1.39E-05	7.38E-06	2.15E-05
F53H2.1	13	25	16	11	1.78E-05	1.34E-05	7.00E-06	2.08E-05
F53H2.2	12	40	9	76	1.12E-05	1.76E-05	8.91E-06	1.88E-05
F53H2.3	222	170	287	449	2.80E-06	2.65E-06	1.82E-06	2.25E-06
F53H4.1	127	119	198	229	5.35E-06	1.68E-05	2.61E-06	2.72E-05
F53H4.2	603	643	471	1097	1.24E-05	8.97E-06	1.04E-05	2.02E-05
F53H4.3	38	52	12	13	4.42E-06	3.91E-06	4.50E-06	6.41E-06
F53H4.4	13	25	18	23	6.11E-05	6.16E-05	3.11E-05	8.93E-05
F53H4.5	27	29	16	20	5.46E-06	7.06E-06	1.82E-06	2.25E-06
F53H4.6	41	39	38	62	2.80E-06	3.17E-06	1.82E-06	2.50E-06
F53H8.1	71	133	54	98	2.80E-06	2.65E-06	1.82E-06	2.25E-06
F53H8.2	69	133	114	138	2.80E-06	2.65E-06	1.82E-06	2.25E-06
F53H8.3	66	79	57	65	6.38E-06	1.13E-05	3.15E-06	7.08E-06
F53H8.4	71	109	61	117	5.04E-06	9.20E-06	5.43E-06	8.12E-06
F54A3.1	48	38	87	157	4.09E-06	4.60E-06	2.30E-06	3.22E-06
F54A3.2	64	48	72	83	5.49E-06	7.96E-06	3.08E-06	7.26E-06
F54A3.3	982	821	1975	2374	4.20E-06	3.15E-06	4.96E-06	1.10E-05
F54A3.4	26	33	15	7	1.05E-05	7.46E-06	7.71E-06	1.10E-05
F54A3.5.1	417	442	295	301	4.23E-05	3.34E-05	5.53E-05	8.21E-05
F54A3.5.2	384	416	231	240	2.80E-06	2.65E-06	1.82E-06	2.25E-06
F54A3.6	233	189	250	377	1.04E-04	1.04E-04	4.79E-05	6.03E-05
F54A5.2	40	29	24	21	9.62E-05	9.85E-05	3.77E-05	4.83E-05
F54A5.3a	53	49	41	53	1.61E-05	1.24E-05	1.12E-05	2.09E-05
F54A5.3b	9	8	12	10	3.81E-06	2.65E-06	1.82E-06	2.25E-06
F54A5.3c	18	12	16	15	5.74E-06	5.03E-06	2.90E-06	4.61E-06
F54A5.3d	28	21	23	24	2.80E-06	2.65E-06	2.33E-06	2.41E-06
F54B11.1	8	13	3	6	3.50E-06	2.65E-06	2.02E-06	2.34E-06
F54B11.10	2	13	7	2	2.80E-06	2.65E-06	1.82E-06	2.25E-06
F54B11.11	57	78	52	86	2.80E-06	2.65E-06	1.82E-06	2.25E-06
F54B11.2	74	82	61	51	2.80E-06	4.58E-06	1.82E-06	2.25E-06
F54B11.3a	426	413	470	708	3.50E-06	4.52E-06	2.08E-06	4.23E-06
F54B11.4	2	5	7	3	9.04E-06	9.47E-06	4.87E-06	5.02E-06
F54B11.5	53	68	82	81	1.30E-05	1.19E-05	9.35E-06	1.74E-05
F54B11.6	19	40	14	24	2.80E-06	2.65E-06	1.82E-06	2.25E-06
F54B11.7	4	4	3	1	5.68E-06	6.90E-06	5.72E-06	6.99E-06
F54B11.8	8	13	19	10	2.80E-06	5.05E-06	1.82E-06	2.59E-06
F54B11.9	4	3	1	4	2.80E-06	2.65E-06	1.82E-06	2.25E-06
F54B3.2	9	19	6	4	2.80E-06	2.65E-06	2.35E-06	2.25E-06
F54B3.3.1	974	931	919	1281	2.80E-06	2.65E-06	1.82E-06	2.25E-06
F54B3.3.2	985	946	922	1286	2.80E-06	2.65E-06	1.82E-06	2.25E-06
F54B8.1	0	0	2	1	5.39E-05	4.86E-05	3.31E-05	5.69E-05
F54B8.10	3	6	3	6	5.30E-05	4.81E-05	3.23E-05	5.56E-05
F54B8.11	2	9	15	3	2.80E-06	2.65E-06	1.82E-06	2.25E-06
F54B8.12	1	5	4	4	2.80E-06	2.65E-06	1.82E-06	2.25E-06

F54B8.13	3	2	2	2	2.80E-06	2.65E-06	1.82E-06	2.25E-06
F54B8.14	8	6	11	5	2.80E-06	2.65E-06	1.82E-06	2.25E-06
F54B8.15	36	33	57	35	2.80E-06	2.65E-06	1.82E-06	2.25E-06
F54B8.16	4	3	4	4	2.80E-06	2.65E-06	1.82E-06	2.25E-06
F54B8.2	1	1	5	4	2.80E-06	2.65E-06	1.82E-06	2.25E-06
F54B8.3	22	50	49	26	2.80E-06	2.65E-06	1.82E-06	2.25E-06
F54B8.4	23	24	29	20	2.80E-06	2.65E-06	1.82E-06	2.25E-06
F54B8.5	10	7	2	3	2.80E-06	3.62E-06	2.44E-06	2.25E-06
F54B8.6	9	1	4	3	8.85E-06	8.73E-06	7.27E-06	6.18E-06
F54B8.7a	3	5	3	4	4.14E-06	2.75E-06	1.82E-06	2.25E-06
F54B8.7b	2	2	0	0	2.80E-06	2.65E-06	1.82E-06	2.25E-06
F54B8.8	4	5	14	6	2.80E-06	2.65E-06	1.82E-06	2.25E-06
F54B8.9	6	5	3	5	2.80E-06	2.65E-06	1.82E-06	2.25E-06
F54C1.1	19	26	19	19	2.80E-06	2.65E-06	1.82E-06	2.25E-06
F54C1.2.1	156	156	267	245	2.80E-06	2.65E-06	1.82E-06	2.25E-06
F54C1.2.2	142	148	217	234	2.80E-06	2.65E-06	1.82E-06	2.25E-06
F54C1.3a	399	421	605	889	1.34E-05	1.26E-05	1.49E-05	1.69E-05
F54C1.3b	301	301	415	699	1.27E-05	1.25E-05	1.27E-05	1.69E-05
F54C1.5a	19	18	17	13	1.45E-05	1.45E-05	1.43E-05	2.60E-05
F54C1.5b.1	19	17	18	17	1.60E-05	1.52E-05	1.44E-05	2.99E-05
F54C1.5b.2	16	13	16	10	2.80E-06	2.65E-06	1.82E-06	2.25E-06
F54C1.6	4	0	4	1	2.80E-06	2.65E-06	1.82E-06	2.25E-06
F54C1.7.1	3696	5694	4646	3702	2.80E-06	2.65E-06	1.82E-06	2.25E-06
F54C1.7.2	3539	5490	4530	3472	2.80E-06	2.65E-06	1.82E-06	2.25E-06
F54C1.7.3	3534	5484	4529	3465	5.69E-04	8.28E-04	4.65E-04	4.57E-04
F54C1.8	27	37	22	8	6.63E-04	9.71E-04	5.52E-04	5.22E-04
F54C1.9	20	53	29	9	5.79E-04	8.48E-04	4.83E-04	4.56E-04
F54C4.1	107	124	229	209	4.68E-06	6.06E-06	2.48E-06	2.25E-06
F54C4.2	92	162	248	116	2.80E-06	6.98E-06	2.62E-06	2.25E-06
F54C4.3	217	162	374	666	1.84E-05	2.02E-05	2.56E-05	2.89E-05
F54C4.4	22	31	39	21	2.03E-05	3.38E-05	3.56E-05	2.05E-05
F54C8.1	27	65	21	17	7.06E-06	4.97E-06	7.93E-06	1.74E-05
F54C8.10	0	2	4	0	6.83E-06	9.10E-06	7.89E-06	5.24E-06
F54C8.2	205	162	442	395	3.36E-06	7.67E-06	1.82E-06	2.25E-06
F54C8.3	574	581	646	976	2.80E-06	2.65E-06	2.86E-06	2.25E-06
F54C8.4	512	571	657	974	2.92E-05	2.18E-05	4.10E-05	4.52E-05
F54C8.5	195	206	355	301	1.75E-05	1.68E-05	1.28E-05	2.40E-05
F54C8.6	10	11	2	10	3.85E-05	4.06E-05	3.22E-05	5.89E-05
F54C8.7a	209	307	174	252	1.97E-05	1.97E-05	2.34E-05	2.45E-05
F54C8.8	0	2	4	1	2.80E-06	2.65E-06	1.82E-06	2.25E-06
F54C8.9	0	2	4	1	2.11E-05	2.92E-05	1.14E-05	2.04E-05
F54C9.1.1	2873	3162	1587	2054	2.80E-06	2.65E-06	2.86E-06	2.25E-06
F54C9.1.2	3040	3319	1662	2085	2.80E-06	2.65E-06	2.86E-06	2.25E-06
F54C9.1.3	2514	2651	1338	1618	3.46E-04	3.60E-04	1.24E-04	1.99E-04
F54C9.11	34	76	33	33	3.81E-04	3.93E-04	1.35E-04	2.10E-04
F54C9.2	333	406	366	471	4.56E-04	4.54E-04	1.58E-04	2.36E-04
F54C9.3	185	385	272	103	3.86E-06	8.15E-06	2.44E-06	3.01E-06
F54C9.4	1265	1778	569	579	2.27E-05	2.61E-05	1.62E-05	2.58E-05
F54C9.5.1	15493	13044	18280	18134	6.58E-05	1.29E-04	6.29E-05	2.94E-05
F54C9.5.2	13580	11581	16133	17070	1.59E-04	2.11E-04	4.65E-05	5.85E-05
F54C9.6a.1	294	293	308	400	1.79E-03	1.42E-03	1.37E-03	1.68E-03
F54C9.6a.2	311	299	341	415	1.71E-03	1.38E-03	1.32E-03	1.73E-03
F54C9.6b.1	286	283	305	391	2.37E-05	2.23E-05	1.62E-05	2.59E-05
F54C9.6b.2	268	274	294	386	2.60E-05	2.36E-05	1.85E-05	2.79E-05
F54C9.6b.3	311	299	341	415	2.41E-05	2.25E-05	1.67E-05	2.65E-05
F54C9.7	8	12	5	7	2.32E-05	2.24E-05	1.66E-05	2.69E-05
F54C9.8	6406	5392	10426	11063	2.60E-05	2.36E-05	1.85E-05	2.79E-05
F54C9.9	677	623	289	708	2.80E-06	2.65E-06	1.82E-06	2.25E-06
F54D1.1	29	59	16	15	3.58E-04	2.85E-04	3.79E-04	4.97E-04

F54D1.2	304	419	329	221	3.23E-05	2.81E-05	8.96E-06	2.71E-05
F54D1.3	308	420	340	226	3.89E-06	7.46E-06	1.82E-06	2.25E-06
F54D1.5	54	56	63	53	3.85E-05	5.01E-05	2.71E-05	2.25E-05
F54D1.6	366	611	365	569	3.70E-05	4.76E-05	2.66E-05	2.18E-05
F54D10.1	14	44	51	23	2.80E-06	2.65E-06	1.82E-06	2.25E-06
F54D10.2	83	131	75	116	8.40E-06	1.33E-05	5.47E-06	1.05E-05
F54D10.3	14	9	9	12	2.80E-06	6.61E-06	5.27E-06	2.92E-06
F54D10.4	10	8	12	19	5.57E-06	8.28E-06	3.26E-06	6.25E-06
F54D10.5	198	155	371	384	2.80E-06	2.65E-06	1.82E-06	2.25E-06
F54D10.6	12	22	11	13	2.80E-06	2.65E-06	1.82E-06	3.01E-06
F54D10.7	58	63	85	77	2.40E-05	1.77E-05	2.93E-05	3.74E-05
F54D10.8	9	2	4	3	2.80E-06	4.68E-06	1.82E-06	2.34E-06
F54D10.9	3	1	2	3	8.12E-06	8.33E-06	7.74E-06	8.66E-06
F54D11.2.1	1005	670	1435	2104	2.80E-06	2.65E-06	1.82E-06	2.25E-06
F54D11.2.2	975	663	1373	2066	2.80E-06	2.65E-06	1.82E-06	2.25E-06
F54D11.2.3	939	653	1335	2020	2.99E-05	1.89E-05	2.78E-05	5.03E-05
F54D11.3	15	29	33	32	2.95E-05	1.89E-05	2.70E-05	5.01E-05
F54D11.4	320	163	275	351	2.88E-05	1.89E-05	2.67E-05	4.98E-05
F54D12.1	4	6	10	5	2.80E-06	2.80E-06	2.20E-06	2.63E-06
F54D12.10	7	3	17	8	5.26E-05	2.53E-05	2.94E-05	4.64E-05
F54D12.11	3	2	3	2	2.80E-06	2.65E-06	1.82E-06	2.25E-06
F54D12.2	0	0	1	2	2.80E-06	2.65E-06	2.70E-06	2.25E-06
F54D12.3	4	5	11	3	2.80E-06	2.65E-06	1.82E-06	2.25E-06
F54D12.4	6	10	13	12	2.80E-06	2.65E-06	1.82E-06	2.25E-06
F54D12.5	113	70	209	231	2.80E-06	2.65E-06	1.82E-06	2.25E-06
F54D12.6	67	59	96	109	4.87E-06	7.67E-06	6.87E-06	7.83E-06
F54D12.7	7	8	37	13	1.26E-05	7.35E-06	1.51E-05	2.06E-05
F54D12.8	2	0	1	2	1.28E-05	1.06E-05	1.19E-05	1.67E-05
F54D12.9	1	5	2	3	2.80E-06	2.65E-06	1.90E-06	2.25E-06
F54D5.11	249	266	354	309	2.80E-06	2.65E-06	1.82E-06	2.25E-06
F54D5.12.1	555	1174	705	1278	2.80E-06	2.65E-06	1.82E-06	2.25E-06
F54D5.14	1132	1189	1853	2579	3.15E-05	3.18E-05	2.92E-05	3.14E-05
F54D5.15a	25	36	24	19	3.67E-05	7.34E-05	3.04E-05	6.79E-05
F54D5.15b.1	24	36	25	18	3.42E-05	3.40E-05	3.65E-05	6.27E-05
F54D5.15b.2	24	36	23	18	2.80E-06	2.65E-06	1.82E-06	2.25E-06
F54D5.15c	27	37	26	19	2.80E-06	2.65E-06	1.82E-06	2.25E-06
F54D5.16	66	76	77	80	2.80E-06	2.65E-06	1.82E-06	2.25E-06
F54D5.2	198	216	408	401	2.80E-06	2.65E-06	1.82E-06	2.25E-06
F54D5.3.1	740	1111	1144	801	2.37E-05	2.58E-05	1.80E-05	2.31E-05
F54D5.3.2	627	981	936	715	1.70E-05	1.75E-05	2.28E-05	2.76E-05
F54D5.4.1	959	765	1123	458	1.36E-04	1.93E-04	1.37E-04	1.18E-04
F54D5.4.2	811	657	866	381	1.19E-04	1.76E-04	1.15E-04	1.09E-04
F54D5.5a.1	182	212	279	352	1.74E-04	1.31E-04	1.33E-04	6.68E-05
F54D5.5a.2	177	200	268	339	1.52E-04	1.16E-04	1.06E-04	5.74E-05
F54D5.5b	177	200	268	340	1.43E-05	1.58E-05	1.43E-05	2.23E-05
F54D5.7.1	386	548	479	656	1.52E-05	1.63E-05	1.50E-05	2.34E-05
F54D5.7.2	338	466	402	573	1.34E-05	1.43E-05	1.32E-05	2.07E-05
F54D5.8	1517	2052	1455	1893	3.16E-05	4.23E-05	2.55E-05	4.31E-05
F54D5.9	456	466	828	958	3.06E-05	3.98E-05	2.37E-05	4.16E-05
F54D7.2	476	652	630	850	1.22E-04	1.56E-04	7.60E-05	1.22E-04
F54D7.3	11	4	7	7	2.67E-05	2.57E-05	3.15E-05	4.50E-05
F54D7.4	5580	5741	6680	2593	3.68E-05	4.76E-05	3.17E-05	5.28E-05
F54D8.1.1	113	85	85	305	2.80E-06	2.65E-06	1.82E-06	2.25E-06
F54D8.1.2	106	82	72	298	8.68E-04	8.44E-04	6.76E-04	3.24E-04
F54D8.2.1	1147	2221	1890	820	1.13E-05	8.01E-06	5.52E-06	2.44E-05
F54D8.2.2	955	1883	1502	735	1.12E-05	8.17E-06	4.94E-06	2.52E-05
F54D8.2.3	964	1892	1508	740	2.39E-04	4.38E-04	2.57E-04	1.37E-04
F54D8.3a.1	1093	1409	1383	1446	1.95E-04	3.63E-04	1.99E-04	1.20E-04
F54D8.3a.2	1141	1468	1459	1467	1.97E-04	3.65E-04	2.00E-04	1.21E-04

F54D8.3b.1	1025	1337	1268	1363	7.22E-05	8.79E-05	5.94E-05	7.67E-05
F54D8.3b.2	1080	1389	1359	1431	8.33E-05	1.01E-04	6.93E-05	8.60E-05
F54D8.3b.3	1019	1293	1300	1381	7.18E-05	8.85E-05	5.78E-05	7.67E-05
F54D8.4	23	21	62	23	7.89E-05	9.58E-05	6.46E-05	8.39E-05
F54D8.6	361	325	374	492	7.45E-05	8.92E-05	6.18E-05	8.10E-05
F54E12.1	24	32	44	43	2.80E-06	2.65E-06	4.12E-06	2.25E-06
F54E12.2.1	1968	1486	2303	3044	1.58E-05	1.35E-05	1.07E-05	1.74E-05
F54E12.2.2	1961	1471	2266	3011	6.55E-06	8.23E-06	7.80E-06	9.40E-06
F54E12.3	23	26	34	16	6.44E-05	4.60E-05	4.91E-05	8.00E-05
F54E12.4	133	108	411	151	6.39E-05	4.53E-05	4.80E-05	7.88E-05
F54E12.5	33	41	101	57	8.26E-06	8.81E-06	7.94E-06	4.61E-06
F54E2.1	379	698	434	528	4.00E-05	3.07E-05	8.05E-05	3.65E-05
F54E2.2	4	7	9	4	9.63E-06	1.13E-05	1.92E-05	1.34E-05
F54E2.3a	1501	2239	914	2530	3.41E-05	5.93E-05	2.54E-05	3.81E-05
F54E2.3b	1397	2096	858	2361	2.80E-06	2.65E-06	1.82E-06	2.25E-06
F54E2.3c	1336	1980	799	2323	1.10E-05	1.55E-05	4.35E-06	1.49E-05
F54E2.3d	1439	2158	886	2456	1.16E-05	1.65E-05	4.65E-06	1.58E-05
F54E2.5	16	8	15	8	1.12E-05	1.57E-05	4.37E-06	1.57E-05
F54E2.6	2	3	0	1	1.18E-05	1.67E-05	4.72E-06	1.61E-05
F54E4.1	598	728	515	974	2.80E-06	2.65E-06	1.82E-06	2.25E-06
F54E4.2	3	5	6	2	2.80E-06	2.65E-06	1.82E-06	2.25E-06
F54E4.3	70	98	79	73	7.11E-06	8.17E-06	3.97E-06	9.29E-06
F54E4.4	13	10	14	18	2.80E-06	2.65E-06	1.82E-06	2.25E-06
F54E7.1a	190	221	172	262	7.17E-06	9.47E-06	5.27E-06	6.00E-06
F54E7.1b	133	182	122	220	2.80E-06	2.65E-06	1.82E-06	2.25E-06
F54E7.2.1	5973	6219	16437	5521	1.94E-05	2.13E-05	1.14E-05	2.15E-05
F54E7.2.2	4812	5104	13762	5124	1.46E-05	1.89E-05	8.71E-06	1.94E-05
F54E7.3a	1101	1061	1624	2224	1.35E-03	1.32E-03	2.41E-03	9.99E-04
F54E7.3b	1319	1258	1903	2615	1.18E-03	1.19E-03	2.20E-03	1.01E-03
F54E7.3c	1044	991	1522	2092	2.98E-05	2.72E-05	2.87E-05	4.84E-05
F54E7.5	15	17	22	12	3.05E-05	2.75E-05	2.86E-05	4.86E-05
F54E7.6	7	7	3	3	2.91E-05	2.61E-05	2.76E-05	4.68E-05
F54E7.7	122	241	80	101	2.80E-06	2.65E-06	1.82E-06	2.25E-06
F54E7.8	280	335	391	392	2.80E-06	2.65E-06	1.82E-06	2.25E-06
F54F11.1	3	2	16	4	1.39E-05	2.59E-05	5.92E-06	9.22E-06
F54F11.2	3846	3402	4087	5174	3.49E-05	3.95E-05	3.17E-05	3.93E-05
F54F11.3	4	8	1	6	2.80E-06	2.65E-06	1.82E-06	2.25E-06
F54F12.1	31	84	26	35	8.81E-05	7.36E-05	6.10E-05	9.52E-05
F54F2.1	728	922	588	934	2.80E-06	2.65E-06	1.82E-06	2.25E-06
F54F2.2a.1	1111	1023	1185	1965	2.80E-06	2.65E-06	1.82E-06	2.25E-06
F54F2.2a.2	871	799	934	1531	2.05E-05	2.45E-05	1.08E-05	2.11E-05
F54F2.2a.3	859	795	927	1520	3.72E-05	3.24E-05	2.58E-05	5.29E-05
F54F2.2b	22	16	12	18	3.48E-05	3.01E-05	2.43E-05	4.91E-05
F54F2.5a	343	341	374	573	3.57E-05	3.12E-05	2.51E-05	5.08E-05
F54F2.5b	153	168	162	237	3.86E-06	2.67E-06	1.82E-06	2.54E-06
F54F2.6	0	0	1	2	2.04E-05	1.92E-05	1.45E-05	2.74E-05
F54F2.7	53	55	71	53	1.83E-05	1.89E-05	1.26E-05	2.27E-05
F54F2.9	137	226	143	181	2.80E-06	2.65E-06	1.82E-06	2.25E-06
F54F3.1	736	1014	950	1126	1.05E-05	1.03E-05	9.17E-06	8.43E-06
F54F3.2	7	7	13	4	1.13E-05	1.76E-05	7.69E-06	1.20E-05
F54F3.3	48	25	51	16	1.65E-05	2.15E-05	1.39E-05	2.03E-05
F54F3.4	39	101	35	33	2.80E-06	2.65E-06	1.82E-06	2.25E-06
F54F7.1	20	29	15	26	4.20E-06	2.65E-06	2.90E-06	2.25E-06
F54F7.2	65	138	100	181	5.26E-06	1.29E-05	3.08E-06	3.58E-06
F54F7.3	244	196	293	212	2.94E-06	4.02E-06	1.82E-06	3.06E-06
F54F7.4	10	24	12	10	7.50E-06	1.51E-05	7.53E-06	1.68E-05
F54F7.5	173	168	202	307	4.64E-05	3.52E-05	3.63E-05	3.24E-05
F54F7.6	23	35	24	31	2.80E-06	2.65E-06	1.82E-06	2.25E-06
F54F7.7	2	2	4	2	5.71E-06	5.24E-06	4.35E-06	8.16E-06

F54F7.8	10	6	3	3	2.80E-06	3.97E-06	1.88E-06	2.97E-06
F54G2.1a	94	133	80	112	2.80E-06	2.65E-06	1.82E-06	2.25E-06
F54G2.1b	101	142	89	122	2.80E-06	2.65E-06	1.82E-06	2.25E-06
F54G2.2	23	22	28	15	2.80E-06	3.31E-06	1.82E-06	2.36E-06
F54G8.1	8	6	10	3	3.28E-06	4.34E-06	1.88E-06	3.17E-06
F54G8.2	10	14	15	8	2.80E-06	2.65E-06	1.82E-06	2.25E-06
F54G8.3	364	500	423	683	2.80E-06	2.65E-06	1.82E-06	2.25E-06
F54G8.4	103	144	63	133	2.80E-06	2.65E-06	1.82E-06	2.25E-06
F54G8.5	14	12	10	3	1.07E-05	1.39E-05	8.11E-06	1.61E-05
F54G8.6	5	7	4	3	3.58E-06	4.73E-06	1.82E-06	3.71E-06
F54H12.1a	2776	3174	3519	5110	2.80E-06	2.65E-06	1.82E-06	2.25E-06
F54H12.1b	1993	2309	2557	3855	3.86E-06	5.11E-06	2.00E-06	2.25E-06
F54H12.1c.1	2293	2659	3019	4575	1.15E-04	1.24E-04	9.47E-05	1.70E-04
F54H12.1c.2	2586	2981	3287	4933	1.12E-04	1.22E-04	9.33E-05	1.74E-04
F54H12.2	6	7	5	4	1.17E-04	1.28E-04	1.00E-04	1.88E-04
F54H12.4	11	7	8	7	1.24E-04	1.35E-04	1.02E-04	1.89E-04
F54H12.5	45	76	41	69	2.80E-06	2.65E-06	1.82E-06	2.25E-06
F54H12.6	3009	3172	5260	3681	2.80E-06	2.65E-06	1.82E-06	2.25E-06
F54H12.7	39	63	21	15	2.80E-06	3.04E-06	1.82E-06	2.34E-06
F54H5.2	24	47	30	14	4.17E-04	4.15E-04	4.74E-04	4.09E-04
F54H5.3	6	6	9	7	9.97E-06	1.52E-05	3.50E-06	3.08E-06
F54H5.4a	40	42	42	71	2.80E-06	3.89E-06	1.82E-06	2.25E-06
F54H5.4b	58	60	60	86	2.80E-06	2.65E-06	1.82E-06	2.25E-06
F54H5.5	34	71	15	27	4.82E-06	4.79E-06	3.30E-06	6.86E-06
F55A11.1	543	749	718	666	5.26E-06	5.13E-06	3.53E-06	6.25E-06
F55A11.10	0	0	1	0	2.80E-06	3.78E-06	1.82E-06	2.25E-06
F55A11.11	59	145	57	80	7.11E-05	9.27E-05	6.12E-05	7.01E-05
F55A11.2	490	555	594	735	2.80E-06	2.65E-06	1.82E-06	2.25E-06
F55A11.4	169	234	226	334	4.14E-06	9.63E-06	2.61E-06	4.52E-06
F55A11.5	23	28	11	16	3.68E-05	3.93E-05	2.90E-05	4.43E-05
F55A11.6a	78	135	65	86	1.01E-05	1.33E-05	8.84E-06	1.61E-05
F55A11.6b	51	109	45	69	2.80E-06	2.65E-06	1.82E-06	2.25E-06
F55A11.7	630	490	835	1145	1.04E-05	1.70E-05	5.61E-06	9.18E-06
F55A12.1	694	735	541	879	8.34E-06	1.68E-05	4.79E-06	9.09E-06
F55A12.10	79	107	174	88	4.86E-05	3.57E-05	4.19E-05	7.10E-05
F55A12.2a	78	110	113	120	2.90E-05	2.90E-05	1.47E-05	2.95E-05
F55A12.2b	76	106	112	117	1.31E-05	1.67E-05	1.88E-05	1.17E-05
F55A12.2c	78	109	114	119	8.82E-06	1.18E-05	8.33E-06	1.09E-05
F55A12.3	683	598	859	1236	9.21E-06	1.21E-05	8.84E-06	1.14E-05
F55A12.4a	45	85	40	40	9.04E-06	1.19E-05	8.60E-06	1.11E-05
F55A12.4b	41	64	40	34	3.18E-05	2.63E-05	2.61E-05	4.63E-05
F55A12.4c	40	68	35	34	4.40E-06	7.86E-06	2.55E-06	3.15E-06
F55A12.5	637	691	946	1306	3.33E-06	4.89E-06	2.11E-06	2.25E-06
F55A12.6	22	21	22	10	4.34E-06	6.98E-06	2.48E-06	2.97E-06
F55A12.7	318	381	206	371	2.65E-05	2.72E-05	2.56E-05	4.37E-05
F55A12.9a	411	895	249	543	2.80E-06	2.65E-06	1.82E-06	2.25E-06
F55A12.9b	412	888	249	543	2.22E-05	2.52E-05	9.37E-06	2.08E-05
F55A12.9c	436	928	274	560	3.26E-05	6.70E-05	1.28E-05	3.46E-05
F55A12.9d.1	562	1235	385	751	3.36E-05	6.84E-05	1.32E-05	3.56E-05
F55A12.9d.2	557	1222	383	736	3.40E-05	6.83E-05	1.39E-05	3.51E-05
F55A12.9d.3	578	1242	405	752	3.02E-05	6.27E-05	1.35E-05	3.24E-05
F55A12.9d.4	561	1232	386	747	3.18E-05	6.59E-05	1.42E-05	3.38E-05
F55A12.9d.5	410	887	249	541	3.13E-05	6.36E-05	1.43E-05	3.27E-05
F55A3.1	519	637	975	1573	3.16E-05	6.55E-05	1.41E-05	3.38E-05
F55A3.2	169	176	136	266	3.40E-05	6.95E-05	1.34E-05	3.61E-05
F55A3.3	2646	2225	3708	4631	1.61E-05	1.87E-05	1.97E-05	3.92E-05
F55A3.5	0	3	9	4	7.20E-06	7.09E-06	3.77E-06	9.11E-06
F55A3.6	16	12	11	4	8.86E-05	7.04E-05	8.08E-05	1.25E-04
F55A3.7	428	420	552	746	2.80E-06	2.65E-06	1.82E-06	2.25E-06

F55A4.10	12	15	16	11	5.01E-06	3.54E-06	2.24E-06	2.25E-06
F55A4.2	41	57	45	64	3.25E-05	3.01E-05	2.73E-05	4.55E-05
F55A4.3	0	1	0	1	2.80E-06	2.65E-06	1.82E-06	2.25E-06
F55A4.4	8	7	5	9	2.91E-06	3.81E-06	2.08E-06	3.64E-06
F55A4.5	211	225	204	285	2.80E-06	2.65E-06	1.82E-06	2.25E-06
F55A4.7	4	10	3	6	2.80E-06	2.65E-06	1.82E-06	2.25E-06
F55A4.8a	57	110	34	93	8.96E-06	9.02E-06	5.63E-06	9.72E-06
F55A4.8b	49	96	23	80	2.80E-06	2.65E-06	1.82E-06	2.25E-06
F55A4.8c.1	23	38	9	29	3.33E-06	6.08E-06	1.82E-06	4.39E-06
F55A4.8c.2	25	43	11	35	3.25E-06	6.00E-06	1.82E-06	4.25E-06
F55A8.1.1	169	153	158	288	2.80E-06	4.29E-06	1.82E-06	2.79E-06
F55A8.1.2	169	152	157	285	3.58E-06	5.82E-06	1.82E-06	4.03E-06
F55A8.2a.1	1605	1638	1807	2539	1.14E-05	9.79E-06	6.96E-06	1.57E-05
F55A8.2a.2	1485	1523	1677	2309	1.17E-05	9.92E-06	7.05E-06	1.58E-05
F55A8.2b	1169	1213	1322	1920	5.81E-05	5.60E-05	4.26E-05	7.38E-05
F55A8.2c	1178	1221	1332	1923	5.77E-05	5.59E-05	4.24E-05	7.21E-05
F55A8.2d	178	169	191	270	5.70E-05	5.58E-05	4.19E-05	7.51E-05
F55A8.2e	1172	1216	1330	1926	5.78E-05	5.66E-05	4.25E-05	7.58E-05
F55A8.2f	767	825	876	1335	3.39E-05	3.04E-05	2.37E-05	4.13E-05
F55B11.1	329	505	270	332	5.56E-05	5.44E-05	4.10E-05	7.33E-05
F55B11.2	884	995	913	1168	5.57E-05	5.66E-05	4.14E-05	7.79E-05
F55B11.3	345	579	719	682	8.93E-06	1.30E-05	4.77E-06	7.24E-06
F55B11.4	131	154	331	116	1.47E-04	1.56E-04	9.89E-05	1.56E-04
F55B11.5	62	103	85	128	5.55E-05	8.80E-05	7.53E-05	8.82E-05
F55B11.6	2	4	0	1	2.69E-05	2.98E-05	4.42E-05	1.91E-05
F55B12.1	8	14	22	15	1.13E-05	1.78E-05	1.01E-05	1.88E-05
F55B12.2	5	2	10	5	2.80E-06	2.65E-06	1.82E-06	2.25E-06
F55B12.3a	964	855	1275	1606	2.80E-06	2.65E-06	1.82E-06	2.25E-06
F55B12.3b.1	1067	971	1444	1796	2.80E-06	2.65E-06	1.82E-06	2.25E-06
F55B12.3b.2	716	604	942	1232	4.55E-05	3.81E-05	3.92E-05	6.09E-05
F55B12.4.1	212	249	257	346	3.92E-05	3.37E-05	3.45E-05	5.31E-05
F55B12.4.2	193	229	244	329	4.55E-05	3.63E-05	3.90E-05	6.29E-05
F55B12.4.3	186	221	219	318	1.61E-05	1.79E-05	1.27E-05	2.11E-05
F55B12.5.1	278	496	386	567	1.59E-05	1.79E-05	1.31E-05	2.18E-05
F55B12.5.2	259	465	357	531	1.55E-05	1.74E-05	1.19E-05	2.13E-05
F55B12.6	6	6	4	2	1.62E-05	2.73E-05	1.46E-05	2.66E-05
F55B12.7	6	8	14	2	1.75E-05	2.97E-05	1.57E-05	2.88E-05
F55B12.8	2	8	14	13	2.80E-06	2.65E-06	1.82E-06	2.25E-06
F55B12.9	3	4	7	4	2.80E-06	2.65E-06	1.82E-06	2.25E-06
F55C10.1	137	225	136	143	2.80E-06	2.65E-06	1.82E-06	2.25E-06
F55C10.2	1393	1260	892	740	2.80E-06	2.65E-06	1.82E-06	2.25E-06
F55C10.3	1307	1058	813	783	1.67E-05	2.59E-05	1.08E-05	1.40E-05
F55C10.4	10	28	20	9	1.65E-04	1.41E-04	6.89E-05	7.05E-05
F55C10.5	49	74	60	31	1.83E-04	1.40E-04	7.40E-05	8.79E-05
F55C12.1a.1	266	437	201	298	2.80E-06	2.65E-06	1.82E-06	2.25E-06
F55C12.1a.2	272	447	204	303	1.62E-05	2.31E-05	1.29E-05	8.23E-06
F55C12.1b	276	453	206	306	2.00E-05	3.11E-05	9.86E-06	1.80E-05
F55C12.1c	253	416	192	286	2.22E-05	3.44E-05	1.08E-05	1.98E-05
F55C12.1d.1	359	560	247	386	2.04E-05	3.16E-05	9.91E-06	1.81E-05
F55C12.1d.2	272	447	204	303	2.11E-05	3.27E-05	1.04E-05	1.91E-05
F55C12.4	17	26	21	8	2.09E-05	3.08E-05	9.35E-06	1.80E-05
F55C12.5a	472	649	649	1079	2.05E-05	3.18E-05	1.00E-05	1.84E-05
F55C12.5b	586	803	775	1251	2.80E-06	3.15E-06	1.82E-06	2.25E-06
F55C12.5c	574	786	755	1211	2.01E-05	2.61E-05	1.80E-05	3.69E-05
F55C12.6	7	17	12	13	2.02E-05	2.62E-05	1.74E-05	3.47E-05
F55C12.7.1	33	47	43	26	2.02E-05	2.62E-05	1.73E-05	3.43E-05
F55C12.7.2	22	37	33	16	2.80E-06	2.65E-06	1.82E-06	2.25E-06
F55C5.1	52	103	74	22	6.13E-06	8.25E-06	5.19E-06	3.87E-06
F55C5.10	7	3	8	9	6.64E-06	1.06E-05	6.49E-06	3.89E-06

F55C5.11	1	2	1	2	1.63E-05	3.05E-05	1.51E-05	5.53E-06
F55C5.2	28	56	19	9	2.80E-06	2.65E-06	1.82E-06	2.25E-06
F55C5.3a	51	67	32	61	2.80E-06	2.65E-06	1.82E-06	2.25E-06
F55C5.3b	52	73	39	66	3.53E-06	6.64E-06	1.82E-06	2.25E-06
F55C5.4	763	666	1020	1452	3.33E-06	4.15E-06	1.82E-06	3.22E-06
F55C5.5	301	371	384	355	3.08E-06	4.07E-06	1.82E-06	3.15E-06
F55C5.6	6	10	11	6	2.68E-05	2.21E-05	2.33E-05	4.10E-05
F55C5.7	467	543	792	1154	3.08E-05	3.58E-05	2.55E-05	2.91E-05
F55C5.8	1156	1294	1299	2045	2.80E-06	2.65E-06	1.82E-06	2.25E-06
F55C5.9	4	6	13	1	1.93E-05	2.12E-05	2.13E-05	3.83E-05
F55C7.2	6	5	0	2	5.71E-05	6.04E-05	4.18E-05	8.11E-05
F55C7.7a	323	388	261	486	2.80E-06	2.65E-06	1.82E-06	2.25E-06
F55C7.7b	149	196	147	296	2.80E-06	2.65E-06	1.82E-06	2.25E-06
F55C7.7c	185	212	122	206	4.84E-06	5.50E-06	2.55E-06	5.85E-06
F55C7.7e	173	192	112	187	3.39E-06	4.21E-06	2.19E-06	5.42E-06
F55C7.7f	303	369	250	462	6.83E-06	7.38E-06	2.93E-06	6.09E-06
F55C9.1	4	3	9	2	1.01E-05	1.06E-05	4.28E-06	8.82E-06
F55C9.10	6	5	2	3	4.62E-06	5.32E-06	2.48E-06	5.67E-06
F55C9.11	1	2	0	1	2.80E-06	2.65E-06	1.82E-06	2.25E-06
F55C9.12	3	4	5	3	2.80E-06	2.65E-06	1.82E-06	2.25E-06
F55C9.13	4	12	8	4	2.80E-06	2.65E-06	1.82E-06	2.25E-06
F55C9.3	3	12	18	4	2.80E-06	2.65E-06	1.82E-06	2.25E-06
F55C9.4	6	5	5	3	2.80E-06	2.65E-06	1.82E-06	2.25E-06
F55C9.5	0	1	3	1	2.80E-06	2.65E-06	2.61E-06	2.25E-06
F55C9.6	2	2	1	3	2.80E-06	2.65E-06	1.82E-06	2.25E-06
F55C9.7	4	7	6	9	2.80E-06	2.65E-06	1.82E-06	2.25E-06
F55C9.8	3	5	6	3	2.80E-06	2.65E-06	1.82E-06	2.25E-06
F55D1.1	1	4	3	1	2.80E-06	2.65E-06	1.82E-06	2.25E-06
F55D1.2	0	1	0	0	2.80E-06	2.65E-06	1.82E-06	2.25E-06
F55D10.1	222	401	232	565	2.80E-06	2.65E-06	1.82E-06	2.25E-06
F55D10.2.1	1464	1387	3876	997	2.80E-06	2.65E-06	1.82E-06	2.25E-06
F55D10.2.2	1354	1304	2800	852	8.12E-06	1.39E-05	5.52E-06	1.66E-05
F55D10.3	74	86	70	123	3.22E-04	2.88E-04	5.55E-04	1.76E-04
F55D10.4	8	8	11	11	3.01E-04	2.74E-04	4.05E-04	1.52E-04
F55D10.5	14	11	8	8	3.42E-06	3.73E-06	2.10E-06	4.54E-06
F55D12.1	15	24	20	24	2.80E-06	2.65E-06	1.82E-06	2.25E-06
F55D12.2	118	217	70	111	2.80E-06	2.65E-06	1.82E-06	2.25E-06
F55D12.3	7	9	12	6	3.95E-06	5.95E-06	3.43E-06	5.06E-06
F55D12.4	13	10	12	8	5.38E-06	9.36E-06	2.08E-06	4.07E-06
F55D12.5	822	627	712	1117	2.80E-06	2.65E-06	1.82E-06	2.25E-06
F55D12.6	16	44	22	7	2.80E-06	2.65E-06	1.82E-06	2.25E-06
F55E10.1	1	2	1	2	4.23E-05	3.05E-05	2.39E-05	4.62E-05
F55E10.2	3	2	0	1	3.14E-06	8.17E-06	2.81E-06	2.25E-06
F55E10.4	1	1	2	0	2.80E-06	2.65E-06	1.82E-06	2.25E-06
F55E10.5	8	5	7	2	2.80E-06	2.65E-06	1.82E-06	2.25E-06
F55E10.6	50	89	36	67	2.80E-06	2.65E-06	1.82E-06	2.25E-06
F55E10.7	51	62	44	70	2.80E-06	2.65E-06	1.82E-06	2.25E-06
F55F1.1	6	5	1	0	6.10E-06	1.03E-05	2.86E-06	6.57E-06
F55F1.2	4	4	0	0	2.80E-06	3.02E-06	1.82E-06	2.88E-06
F55F1.3	3	7	1	8	2.80E-06	2.65E-06	1.82E-06	2.25E-06
F55F10.1	1577	1289	670	1831	2.80E-06	2.65E-06	1.82E-06	2.25E-06
F55F3.1.1	78	109	69	100	2.80E-06	2.65E-06	1.82E-06	2.25E-06
F55F3.1.2	70	86	64	92	1.32E-05	1.02E-05	3.64E-06	1.23E-05
F55F3.2a	105	81	95	78	7.92E-06	1.05E-05	4.56E-06	8.16E-06
F55F3.2b	107	85	101	84	8.96E-06	1.04E-05	5.34E-06	9.47E-06
F55F3.3.1	780	779	730	910	7.00E-06	5.11E-06	4.12E-06	4.16E-06
F55F3.3.2	429	447	401	540	7.03E-06	5.29E-06	4.32E-06	4.43E-06
F55F3.4	20	34	26	6	5.10E-05	4.82E-05	3.11E-05	4.78E-05
F55F3.t1	0	0	3	0	4.80E-05	4.73E-05	2.92E-05	4.86E-05

F55F3.t2	0	0	3	0	2.80E-06	2.65E-06	1.82E-06	2.25E-06
F55F8.1.1	60	110	39	34	2.80E-06	2.65E-06	2.99E-06	2.25E-06
F55F8.1.2	56	106	36	29	2.80E-06	2.65E-06	2.99E-06	2.25E-06
F55F8.2a	570	596	475	760	2.80E-06	3.97E-06	1.82E-06	2.25E-06
F55F8.2b	559	575	469	745	2.80E-06	4.15E-06	1.82E-06	2.25E-06
F55F8.3	492	468	421	680	2.74E-05	2.71E-05	1.49E-05	2.94E-05
F55F8.4.1	497	553	423	736	2.71E-05	2.64E-05	1.48E-05	2.91E-05
F55F8.4.2	465	516	389	679	1.84E-05	1.65E-05	1.02E-05	2.04E-05
F55F8.4.3	477	526	393	688	3.08E-05	3.24E-05	1.71E-05	3.67E-05
F55F8.5.1	371	404	317	469	3.05E-05	3.19E-05	1.66E-05	3.57E-05
F55F8.5.2	350	366	283	437	3.11E-05	3.23E-05	1.67E-05	3.60E-05
F55F8.6	358	428	402	470	2.86E-05	2.95E-05	1.59E-05	2.91E-05
F55F8.7	63	76	33	29	2.95E-05	2.91E-05	1.55E-05	2.96E-05
F55F8.8	7	0	5	4	3.70E-05	4.18E-05	2.70E-05	3.90E-05
F55F8.9	496	497	799	1011	4.54E-06	5.16E-06	1.82E-06	2.25E-06
F55G1.1	10	6	6	5	2.80E-06	2.65E-06	1.82E-06	2.25E-06
F55G1.10	30	27	110	36	2.82E-05	2.67E-05	2.96E-05	4.62E-05
F55G1.11	31	50	110	67	2.80E-06	2.65E-06	1.82E-06	2.25E-06
F55G1.12	10	4	4	3	7.14E-06	6.08E-06	1.71E-05	6.88E-06
F55G1.13	7	8	6	6	9.72E-06	1.48E-05	2.24E-05	1.69E-05
F55G1.15	13	24	21	27	2.80E-06	2.65E-06	1.82E-06	2.25E-06
F55G1.2	34	51	76	54	2.80E-06	2.65E-06	1.82E-06	2.25E-06
F55G1.3	15	36	42	27	2.80E-06	2.65E-06	1.82E-06	2.38E-06
F55G1.4	1837	2039	3129	3893	9.27E-06	1.31E-05	1.35E-05	1.18E-05
F55G1.5	102	96	117	169	4.51E-06	1.02E-05	8.24E-06	6.52E-06
F55G1.6	353	374	489	532	3.25E-05	3.41E-05	3.60E-05	5.53E-05
F55G1.7	14	21	23	28	1.23E-05	1.09E-05	9.17E-06	1.64E-05
F55G1.8.1	2037	2059	3036	3899	3.18E-05	3.18E-05	2.86E-05	3.84E-05
F55G1.9	135	171	199	233	2.80E-06	2.65E-06	1.82E-06	2.72E-06
F55G11.1	6	8	3	10	1.05E-04	1.00E-04	1.02E-04	1.62E-04
F55G11.10	2	3	3	4	1.66E-05	1.99E-05	1.59E-05	2.30E-05
F55G11.2	195	48	206	52	2.80E-06	2.65E-06	1.82E-06	2.25E-06
F55G11.3	4	1	6	2	2.80E-06	2.65E-06	1.82E-06	2.25E-06
F55G11.4	200	303	239	193	2.13E-05	4.95E-06	1.46E-05	4.57E-06
F55G11.5	65	29	49	9	2.80E-06	2.65E-06	1.82E-06	2.25E-06
F55G11.6	11	9	3	6	2.09E-05	3.00E-05	1.63E-05	1.62E-05
F55G11.7	4	1	5	3	6.50E-06	2.75E-06	3.19E-06	2.25E-06
F55G11.8	138	90	162	67	2.80E-06	2.65E-06	1.82E-06	2.25E-06
F55G11.9	16	24	22	7	2.80E-06	2.65E-06	1.82E-06	2.25E-06
F55G7.1	8	4	3	4	1.40E-05	8.62E-06	1.07E-05	5.47E-06
F55G7.2	8	16	6	11	2.80E-06	2.65E-06	1.82E-06	2.25E-06
F55G7.3	5	3	4	1	2.80E-06	2.65E-06	1.82E-06	2.25E-06
F55H12.1	28	58	24	23	2.80E-06	2.65E-06	1.82E-06	2.25E-06
F55H12.2	53	139	61	36	2.80E-06	2.65E-06	1.82E-06	2.25E-06
F55H12.3	184	263	197	249	2.80E-06	2.65E-06	1.82E-06	2.25E-06
F55H12.4	511	880	469	467	1.66E-05	4.11E-05	1.24E-05	9.04E-06
F55H12.5	34	47	14	14	2.80E-06	3.09E-06	1.82E-06	2.47E-06
F55H12.6a	15	27	14	15	7.90E-05	1.29E-04	4.72E-05	5.80E-05
F55H12.6b	17	19	13	18	4.20E-06	5.48E-06	1.82E-06	2.25E-06
F55H2.1	45	87	54	57	2.80E-06	3.09E-06	1.82E-06	2.25E-06
F55H2.2.1	1183	1823	1383	2189	2.80E-06	2.65E-06	1.82E-06	2.25E-06
F55H2.2.2	870	1286	1008	1631	6.89E-06	1.26E-05	5.38E-06	6.99E-06
F55H2.4	360	225	549	321	1.15E-04	1.67E-04	8.75E-05	1.71E-04
F55H2.5	22	40	96	58	1.25E-04	1.74E-04	9.39E-05	1.88E-04
F55H2.6.1	2673	2305	3043	4860	2.21E-05	1.30E-05	2.19E-05	1.58E-05
F56A11.1	267	326	267	495	3.08E-06	5.29E-06	8.73E-06	6.52E-06
F56A11.3	37	43	43	55	6.79E-05	5.53E-05	5.03E-05	9.92E-05
F56A11.4	7	8	9	6	7.08E-06	8.17E-06	4.61E-06	1.05E-05
F56A11.5	234	272	330	344	3.25E-06	3.57E-06	2.46E-06	3.87E-06

F56A11.6	43	61	37	23	2.80E-06	2.65E-06	1.82E-06	2.25E-06
F56A11.7	2	3	3	1	2.44E-05	2.68E-05	2.24E-05	2.88E-05
F56A12.1	8	5	7	9	3.39E-06	4.55E-06	1.90E-06	2.25E-06
F56A12.2	6	6	8	7	2.80E-06	2.65E-06	1.82E-06	2.25E-06
F56A3.1	162	175	202	316	2.80E-06	2.65E-06	1.82E-06	2.25E-06
F56A3.2	202	221	307	354	2.80E-06	2.65E-06	1.82E-06	2.25E-06
F56A3.3a.1	937	977	1598	2101	7.53E-06	7.70E-06	6.12E-06	1.18E-05
F56A3.3a.2	934	972	1597	2099	1.60E-05	1.66E-05	1.59E-05	2.26E-05
F56A3.3b	454	448	900	1192	2.15E-05	2.12E-05	2.39E-05	3.88E-05
F56A3.4	1632	1706	2402	3253	2.15E-05	2.11E-05	2.39E-05	3.88E-05
F56A3.5	2	2	1	0	1.90E-05	1.77E-05	2.46E-05	4.02E-05
F56A4.1	3	1	1	0	4.82E-05	4.76E-05	4.62E-05	7.72E-05
F56A4.10	7	93	32	77	2.80E-06	2.65E-06	1.82E-06	2.25E-06
F56A4.11	11	15	9	22	2.80E-06	2.65E-06	1.82E-06	2.25E-06
F56A4.12	14	100	21	87	2.80E-06	7.25E-06	1.82E-06	5.11E-06
F56A4.2	2156	18158	5271	15876	2.80E-06	2.65E-06	1.82E-06	2.25E-06
F56A4.3	220	763	296	192	2.80E-06	7.46E-06	1.82E-06	5.51E-06
F56A4.4	10	42	0	6	2.71E-04	2.16E-03	4.31E-04	1.60E-03
F56A4.5	4	9	10	3	6.26E-05	2.05E-04	5.48E-05	4.38E-05
F56A4.6	3	8	11	9	2.80E-06	1.01E-05	1.82E-06	2.25E-06
F56A4.7	4	8	5	4	2.80E-06	2.65E-06	1.82E-06	2.25E-06
F56A4.9	9	283	10	180	2.80E-06	2.65E-06	1.82E-06	2.25E-06
F56A6.1a	1004	1243	1192	2116	2.80E-06	2.65E-06	1.82E-06	2.25E-06
F56A6.1b	995	1210	1188	2087	2.80E-06	2.16E-05	1.82E-06	1.16E-05
F56A6.2	108	138	105	139	3.99E-05	4.67E-05	3.08E-05	6.76E-05
F56A6.4	91	138	135	153	3.95E-05	4.54E-05	3.07E-05	6.66E-05
F56A6.5	1	1	4	1	2.80E-06	2.65E-06	1.82E-06	2.25E-06
F56A8.1	65	70	48	45	7.08E-06	1.01E-05	6.83E-06	9.56E-06
F56A8.3a.1	1027	943	832	1049	2.80E-06	2.65E-06	1.82E-06	2.25E-06
F56A8.3a.2	942	857	778	995	2.80E-06	2.65E-06	1.82E-06	2.25E-06
F56A8.3b.1	596	560	511	629	6.74E-05	5.85E-05	3.55E-05	5.53E-05
F56A8.3b.2	557	530	482	602	6.47E-05	5.56E-05	3.48E-05	5.49E-05
F56A8.4	118	143	195	194	6.22E-05	5.52E-05	3.47E-05	5.27E-05
F56A8.5	91	138	173	182	7.73E-05	6.95E-05	4.35E-05	6.71E-05
F56A8.6.1	324	221	472	496	1.54E-05	1.76E-05	1.65E-05	2.03E-05
F56A8.6.2	291	197	435	477	1.08E-05	1.54E-05	1.33E-05	1.73E-05
F56A8.7b	73	159	105	124	3.28E-05	2.11E-05	3.11E-05	4.03E-05
F56A8.8	13	12	3	6	3.20E-05	2.04E-05	3.11E-05	4.21E-05
F56B3.1	890	1600	1782	1765	8.18E-06	1.68E-05	7.65E-06	1.12E-05
F56B3.10	6	12	5	5	2.80E-06	2.65E-06	1.82E-06	2.25E-06
F56B3.11a	233	255	240	238	8.53E-05	1.45E-04	1.11E-04	1.36E-04
F56B3.11b	215	231	227	217	2.80E-06	2.65E-06	1.82E-06	2.25E-06
F56B3.12	144	150	207	241	3.99E-05	4.13E-05	2.67E-05	3.27E-05
F56B3.2a	78	115	68	108	3.48E-05	3.53E-05	2.39E-05	2.82E-05
F56B3.2b	66	91	50	85	2.92E-05	2.88E-05	2.73E-05	3.93E-05
F56B3.3	9	6	29	9	5.57E-06	7.78E-06	3.17E-06	6.21E-06
F56B3.4a	139	152	127	244	5.88E-06	7.67E-06	2.90E-06	6.07E-06
F56B3.4b	115	133	111	227	2.80E-06	2.65E-06	3.12E-06	2.25E-06
F56B3.5	77	93	300	159	7.64E-06	7.91E-06	4.56E-06	1.08E-05
F56B3.6	18	44	13	12	7.08E-06	7.72E-06	4.45E-06	1.12E-05
F56B3.7	17	21	21	16	7.78E-06	8.89E-06	1.98E-05	1.29E-05
F56B3.8	181	214	244	292	3.28E-06	7.54E-06	1.82E-06	2.25E-06
F56B3.9	7	10	8	11	2.80E-06	2.65E-06	1.82E-06	2.25E-06
F56B6.2a	125	175	199	282	1.93E-05	2.15E-05	1.69E-05	2.50E-05
F56B6.2b	90	131	150	235	2.80E-06	2.65E-06	1.82E-06	2.25E-06
F56B6.2c	55	102	86	135	4.70E-06	6.24E-06	4.88E-06	8.55E-06
F56B6.2d	38	60	47	72	4.20E-06	5.79E-06	4.56E-06	8.82E-06
F56B6.2e.1	55	102	86	135	4.65E-06	8.12E-06	4.72E-06	9.15E-06
F56B6.2e.2	52	92	83	123	6.24E-06	9.31E-06	5.03E-06	9.51E-06

F56B6.4a	598	753	511	553	4.65E-06	8.12E-06	4.72E-06	9.15E-06
F56B6.4b	592	745	506	549	4.79E-06	7.99E-06	4.97E-06	9.09E-06
F56B6.4c	581	726	493	527	4.17E-05	4.97E-05	2.32E-05	3.10E-05
F56B6.5a	9	16	16	11	5.71E-05	6.79E-05	3.18E-05	4.25E-05
F56B6.5b	7	14	14	10	5.53E-05	6.53E-05	3.05E-05	4.03E-05
F56B6.6	6	12	1	1	2.80E-06	2.65E-06	1.82E-06	2.25E-06
F56C11.1	210	293	216	343	2.80E-06	2.65E-06	1.82E-06	2.25E-06
F56C11.2	146	227	122	109	2.80E-06	3.49E-06	1.82E-06	2.25E-06
F56C11.3	21	35	56	31	5.10E-06	6.72E-06	3.41E-06	6.68E-06
F56C11.4	23	26	26	41	6.05E-06	8.91E-06	3.30E-06	3.64E-06
F56C11.5	146	139	169	268	4.84E-06	7.62E-06	8.40E-06	5.74E-06
F56C11.6a	19	77	35	41	3.72E-06	3.99E-06	2.75E-06	5.35E-06
F56C11.6b	19	74	34	40	2.36E-05	2.12E-05	1.78E-05	3.48E-05
F56C3.1	3	3	3	4	2.80E-06	4.79E-06	1.82E-06	2.25E-06
F56C3.10	0	3	0	1	2.80E-06	4.73E-06	1.82E-06	2.25E-06
F56C3.2	16	37	34	16	2.80E-06	2.65E-06	1.82E-06	2.25E-06
F56C3.3	2	6	2	1	2.80E-06	2.65E-06	1.82E-06	2.25E-06
F56C3.4	3	4	10	2	2.80E-06	2.67E-06	1.82E-06	2.25E-06
F56C3.5	7	7	8	3	2.80E-06	2.65E-06	1.82E-06	2.25E-06
F56C3.6	4	7	5	3	2.80E-06	2.65E-06	1.82E-06	2.25E-06
F56C3.7	1	1	1	0	2.80E-06	2.65E-06	1.82E-06	2.25E-06
F56C3.8	4	2	4	0	2.80E-06	2.65E-06	1.82E-06	2.25E-06
F56C3.9	12	14	18	10	2.80E-06	2.65E-06	1.82E-06	2.25E-06
F56C4.1	5	5	9	3	2.80E-06	2.65E-06	1.82E-06	2.25E-06
F56C4.2	6	4	1	0	2.80E-06	2.65E-06	1.82E-06	2.25E-06
F56C4.3	7	10	10	10	2.80E-06	2.65E-06	1.82E-06	2.25E-06
F56C9.10a	1620	1813	2646	3463	2.80E-06	2.65E-06	1.82E-06	2.25E-06
F56C9.10b.1	1771	2011	2913	3779	2.80E-06	2.65E-06	1.82E-06	2.25E-06
F56C9.10b.2	1771	2011	2909	3774	5.86E-05	6.19E-05	6.22E-05	1.01E-04
F56C9.11	205	238	318	344	5.51E-05	5.91E-05	5.90E-05	9.44E-05
F56C9.3	930	785	1490	1623	5.52E-05	5.92E-05	5.90E-05	9.45E-05
F56C9.5	12	11	12	4	2.54E-05	2.79E-05	2.56E-05	3.42E-05
F56C9.6	740	633	826	1034	6.65E-05	5.30E-05	6.93E-05	9.32E-05
F56C9.7.1	569	867	756	663	3.05E-06	2.65E-06	1.99E-06	2.25E-06
F56C9.7.2	492	751	657	620	7.30E-05	5.90E-05	5.30E-05	8.19E-05
F56C9.8	112	154	80	165	8.13E-05	1.17E-04	7.03E-05	7.61E-05
F56D1.1	484	462	467	631	7.70E-05	1.11E-04	6.69E-05	7.79E-05
F56D1.2	500	545	1044	1316	2.28E-05	2.97E-05	1.06E-05	2.70E-05
F56D1.3.1	343	358	548	634	3.85E-05	3.47E-05	2.42E-05	4.03E-05
F56D1.3.2	209	244	330	370	1.83E-05	1.89E-05	2.49E-05	3.87E-05
F56D1.4a	1220	1153	1678	2419	3.32E-05	3.28E-05	3.45E-05	4.93E-05
F56D1.4b	1183	1126	1637	2375	3.53E-05	3.89E-05	3.62E-05	5.01E-05
F56D1.4c	1216	1149	1667	2403	2.88E-05	2.57E-05	2.57E-05	4.58E-05
F56D1.5	78	153	50	87	2.79E-05	2.51E-05	2.52E-05	4.51E-05
F56D1.6	52	76	21	76	2.87E-05	2.56E-05	2.56E-05	4.55E-05
F56D1.7	2828	2002	3589	5608	6.64E-06	1.23E-05	2.77E-06	5.96E-06
F56D12.1a	295	411	388	505	9.46E-06	1.31E-05	2.50E-06	1.11E-05
F56D12.1b	266	355	327	455	1.59E-04	1.07E-04	1.32E-04	2.54E-04
F56D12.1c.1	363	486	479	550	1.95E-05	2.57E-05	1.67E-05	2.68E-05
F56D12.1c.2	342	459	448	533	1.93E-05	2.44E-05	1.55E-05	2.65E-05
F56D12.1d.1	338	457	440	541	2.21E-05	2.80E-05	1.90E-05	2.69E-05
F56D12.1d.2	253	338	349	445	3.23E-05	4.09E-05	2.75E-05	4.04E-05
F56D12.4a	44	45	39	43	2.08E-05	2.66E-05	1.76E-05	2.67E-05
F56D12.4b	24	26	32	30	1.87E-05	2.36E-05	1.68E-05	2.64E-05
F56D12.4c	13	18	21	23	2.80E-06	2.65E-06	1.82E-06	2.25E-06
F56D12.5a.1	14334	11507	12121	15815	2.80E-06	2.65E-06	1.82E-06	2.25E-06
F56D12.5a.2	7602	5742	6681	8377	2.80E-06	2.65E-06	1.82E-06	2.25E-06
F56D12.5b	3436	2924	2857	4498	9.04E-04	6.86E-04	4.97E-04	8.01E-04
F56D12.6a	540	444	665	993	6.82E-04	4.87E-04	3.90E-04	6.04E-04

F56D12.6b	472	405	621	931	7.73E-04	6.21E-04	4.18E-04	8.13E-04
F56D2.1.1	2445	3205	3256	3631	1.94E-05	1.51E-05	1.55E-05	2.86E-05
F56D2.1.2	2361	3115	3127	3582	1.82E-05	1.47E-05	1.56E-05	2.88E-05
F56D2.2	176	180	189	257	1.61E-04	1.99E-04	1.39E-04	1.92E-04
F56D2.3	40	86	24	14	1.84E-04	2.29E-04	1.58E-04	2.24E-04
F56D2.4	3	1	2	0	1.42E-05	1.37E-05	9.91E-06	1.66E-05
F56D2.5	42	47	23	49	3.14E-06	6.35E-06	1.82E-06	2.25E-06
F56D2.6a	927	924	1716	2068	2.80E-06	2.65E-06	1.82E-06	2.25E-06
F56D2.6b.1	953	928	1733	2062	2.88E-06	3.04E-06	1.82E-06	2.70E-06
F56D2.6b.2	856	825	1519	1892	4.19E-05	3.95E-05	5.05E-05	7.52E-05
F56D2.8	1	5	5	3	4.41E-05	4.06E-05	5.22E-05	7.66E-05
F56D3.1.1	540	554	195	167	4.53E-05	4.13E-05	5.23E-05	8.05E-05
F56D3.1.2	452	479	165	144	2.80E-06	2.65E-06	1.82E-06	2.25E-06
F56D5.1	20	21	13	36	3.79E-05	3.67E-05	8.91E-06	9.42E-06
F56D5.10	3	2	7	3	3.80E-05	3.81E-05	9.04E-06	9.74E-06
F56D5.2	20	24	14	11	2.80E-06	2.65E-06	1.82E-06	2.86E-06
F56D5.3	16	35	15	17	2.80E-06	2.65E-06	1.82E-06	2.25E-06
F56D5.4	86	94	92	125	2.80E-06	2.65E-06	1.82E-06	2.25E-06
F56D5.5	22	21	17	19	2.80E-06	2.75E-06	1.82E-06	2.25E-06
F56D5.6	111	238	99	110	9.52E-06	9.84E-06	6.63E-06	1.11E-05
F56D5.8	32	29	44	18	3.00E-06	2.70E-06	1.82E-06	2.25E-06
F56D5.9	18	24	40	8	7.53E-06	1.53E-05	4.37E-06	6.00E-06
F56D6.1	9	5	3	2	2.80E-06	2.65E-06	1.82E-06	2.25E-06
F56D6.10	2	7	3	1	2.80E-06	2.65E-06	1.82E-06	2.25E-06
F56D6.11	3	3	2	0	2.80E-06	2.65E-06	1.82E-06	2.25E-06
F56D6.12	8	7	6	7	2.80E-06	3.52E-06	1.82E-06	2.25E-06
F56D6.13	13	20	8	7	2.80E-06	2.65E-06	1.82E-06	2.25E-06
F56D6.14	5	16	2	4	2.80E-06	2.65E-06	1.82E-06	2.25E-06
F56D6.15	29	14	51	6	3.61E-06	5.24E-06	1.82E-06	2.25E-06
F56D6.2	294	86	447	80	2.80E-06	6.19E-06	1.82E-06	2.25E-06
F56D6.4	4	4	7	3	2.80E-06	2.65E-06	3.13E-06	2.25E-06
F56D6.5	5	6	8	1	1.60E-05	4.42E-06	1.59E-05	3.51E-06
F56D6.6	9	13	15	4	2.80E-06	2.65E-06	1.82E-06	2.25E-06
F56D6.7	1	1	3	5	2.80E-06	2.65E-06	1.82E-06	2.25E-06
F56D6.8	6	1	7	3	2.80E-06	2.65E-06	1.82E-06	2.25E-06
F56D6.9	0	3	1	1	2.80E-06	2.65E-06	1.82E-06	2.25E-06
F56E10.1	90	67	93	189	3.33E-06	2.65E-06	2.53E-06	2.25E-06
F56E10.2	31	38	42	62	2.80E-06	2.65E-06	1.82E-06	2.25E-06
F56E10.3	14	14	31	22	9.35E-06	6.59E-06	6.29E-06	1.58E-05
F56E10.4.1	9474	6072	20617	3129	2.80E-06	2.65E-06	1.82E-06	2.25E-06
F56E10.4.2	7610	4666	14482	2644	2.80E-06	2.65E-06	1.82E-06	2.25E-06
F56E10.4.3	7641	4714	14496	2663	2.86E-03	1.73E-03	4.05E-03	7.59E-04
F56E10.4.4	7584	4645	14460	2622	2.34E-03	1.36E-03	2.90E-03	6.53E-04
F56E3.3a	235	334	158	244	2.36E-03	1.37E-03	2.91E-03	6.60E-04
F56E3.3b	234	332	155	241	2.63E-03	1.52E-03	3.26E-03	7.30E-04
F56E3.3c	218	311	134	227	5.21E-06	6.98E-06	2.28E-06	4.34E-06
F56E3.4	8	8	11	5	5.15E-06	6.90E-06	2.22E-06	4.25E-06
F56F10.1.1	519	624	544	620	5.15E-06	6.96E-06	2.06E-06	4.32E-06
F56F10.1.2	475	573	486	604	2.80E-06	2.65E-06	1.82E-06	2.25E-06
F56F10.2	24	41	16	21	3.25E-05	3.69E-05	2.22E-05	3.11E-05
F56F10.3	94	385	57	160	3.27E-05	3.73E-05	2.18E-05	3.34E-05
F56F10.t1	0	0	2	0	2.80E-06	3.81E-06	1.82E-06	2.25E-06
F56F11.2	18	25	14	23	1.05E-05	4.08E-05	4.15E-06	1.44E-05
F56F11.3.1	46	45	47	46	2.80E-06	2.65E-06	2.00E-06	2.25E-06
F56F11.3.2	46	45	46	44	2.80E-06	2.65E-06	1.82E-06	2.25E-06
F56F11.4a	369	281	480	572	3.28E-06	3.04E-06	2.19E-06	2.63E-06
F56F11.4b	376	286	499	582	3.28E-06	3.04E-06	2.13E-06	2.52E-06
F56F11.5	18	17	29	19	3.34E-05	2.40E-05	2.83E-05	4.16E-05
F56F12.1	46	63	31	28	2.62E-05	1.88E-05	2.26E-05	3.25E-05

F56F3.1	2981	2941	3133	5795	2.80E-06	2.65E-06	1.82E-06	2.25E-06
F56F3.2a	357	474	573	713	3.02E-06	3.91E-06	1.82E-06	2.25E-06
F56F3.2b	366	492	590	726	1.01E-04	9.40E-05	6.90E-05	1.57E-04
F56F3.3	1	4	4	1	1.85E-05	2.31E-05	1.93E-05	2.96E-05
F56F3.4	32	70	25	16	1.88E-05	2.39E-05	1.98E-05	3.00E-05
F56F3.6.1	28	39	36	22	2.80E-06	2.65E-06	1.82E-06	2.25E-06
F56F3.6.2	22	25	24	17	3.44E-06	7.09E-06	1.82E-06	2.25E-06
F56F4.1	3	1	7	1	5.24E-06	6.90E-06	4.39E-06	3.31E-06
F56F4.2	5	8	8	1	4.42E-06	4.76E-06	3.13E-06	2.74E-06
F56F4.3	27	50	25	18	2.80E-06	2.65E-06	1.82E-06	2.25E-06
F56F4.4	8	11	12	2	2.80E-06	2.65E-06	1.82E-06	2.25E-06
F56F4.5	83	117	76	95	2.80E-06	2.65E-06	1.82E-06	2.25E-06
F56F4.6	4	0	1	0	2.80E-06	2.65E-06	1.82E-06	2.25E-06
F56F4.t1	0	0	2	0	3.81E-06	5.05E-06	2.26E-06	3.49E-06
F56F4.t2	0	0	3	0	2.80E-06	2.65E-06	1.82E-06	2.25E-06
F56G4.1	51	79	54	56	2.80E-06	2.65E-06	2.00E-06	2.25E-06
F56G4.2	64	130	49	52	2.80E-06	2.65E-06	2.99E-06	2.25E-06
F56G4.3	64	130	49	52	2.80E-06	2.86E-06	1.82E-06	2.25E-06
F56G4.4	170	196	143	261	7.20E-06	1.38E-05	3.59E-06	4.70E-06
F56G4.5.1	105	149	116	203	7.20E-06	1.38E-05	3.59E-06	4.70E-06
F56G4.5.2	109	159	123	204	1.30E-05	1.42E-05	7.11E-06	1.60E-05
F56G4.6	108	106	165	218	6.19E-06	8.31E-06	4.45E-06	9.60E-06
F56G4.7	3	11	8	3	6.69E-06	9.20E-06	4.90E-06	1.00E-05
F56H1.1	58	94	46	42	5.18E-06	4.81E-06	5.16E-06	8.41E-06
F56H1.2	15	24	24	15	2.80E-06	2.65E-06	1.82E-06	2.25E-06
F56H1.3	32	44	38	23	2.80E-06	3.41E-06	1.82E-06	2.25E-06
F56H1.4.1	1841	1668	2475	2619	2.80E-06	2.65E-06	1.82E-06	2.25E-06
F56H1.4.2	1803	1605	2398	2596	2.80E-06	2.65E-06	1.82E-06	2.25E-06
F56H1.5	425	496	691	1011	1.38E-04	1.18E-04	1.21E-04	1.58E-04
F56H1.6	198	273	279	252	1.54E-04	1.30E-04	1.33E-04	1.78E-04
F56H11.1a.1	68	167	101	115	1.24E-05	1.37E-05	1.31E-05	2.37E-05
F56H11.1a.2	68	161	98	111	1.93E-05	2.52E-05	1.77E-05	1.98E-05
F56H11.1b	54	168	99	97	3.33E-06	7.72E-06	3.21E-06	4.52E-06
F56H11.1c	49	156	99	114	3.50E-06	7.80E-06	3.28E-06	4.57E-06
F56H11.2	20	63	14	24	2.86E-06	8.41E-06	3.41E-06	4.14E-06
F56H11.3	18	27	14	14	2.80E-06	7.41E-06	3.23E-06	4.59E-06
F56H11.4	2302	3282	3306	3791	4.14E-06	1.24E-05	1.90E-06	4.00E-06
F56H11.6	4	3	4	1	2.80E-06	3.07E-06	1.82E-06	2.25E-06
F56H6.1	0	0	1	1	2.44E-04	3.29E-04	2.28E-04	3.23E-04
F56H6.11	4	9	9	9	2.80E-06	2.65E-06	1.82E-06	2.25E-06
F56H6.12	4	7	10	5	2.80E-06	2.65E-06	1.82E-06	2.25E-06
F56H6.13	4	3	0	1	2.80E-06	2.65E-06	1.82E-06	2.25E-06
F56H6.14	0	3	1	1	2.80E-06	2.65E-06	1.82E-06	2.25E-06
F56H6.15	0	3	1	0	2.80E-06	2.65E-06	1.82E-06	2.25E-06
F56H6.16	0	3	1	1	2.80E-06	2.65E-06	1.82E-06	2.25E-06
F56H6.2	2	2	6	3	2.80E-06	2.65E-06	1.82E-06	2.25E-06
F56H6.3	4	3	8	2	2.80E-06	2.65E-06	1.82E-06	2.25E-06
F56H6.4	8	3	5	0	2.80E-06	2.65E-06	1.82E-06	2.25E-06
F56H6.5	4	1	4	2	2.80E-06	2.65E-06	1.82E-06	2.25E-06
F56H6.6	7	6	5	4	2.80E-06	2.65E-06	1.82E-06	2.25E-06
F56H6.7	6	7	8	5	2.80E-06	2.65E-06	1.82E-06	2.25E-06
F56H6.8	4	8	6	3	2.80E-06	2.65E-06	1.82E-06	2.25E-06
F56H6.9	5	8	8	1	2.80E-06	2.65E-06	1.82E-06	2.25E-06
F56H9.1	0	2	18	4	2.80E-06	2.65E-06	1.82E-06	2.25E-06
F56H9.2.1	503	633	3077	473	2.80E-06	2.65E-06	1.82E-06	2.25E-06
F56H9.2.2	402	530	2400	384	2.80E-06	2.65E-06	1.82E-06	2.25E-06
F56H9.3	6	15	14	9	1.69E-04	2.01E-04	6.71E-04	1.27E-04
F56H9.4	6	14	11	8	1.36E-04	1.69E-04	5.28E-04	1.04E-04
F56H9.5	141	176	167	222	2.80E-06	2.65E-06	1.82E-06	2.25E-06

F56H9.6	13	10	10	15	2.80E-06	2.65E-06	1.82E-06	2.25E-06
F57A10.1	1	3	5	2	4.48E-06	5.29E-06	3.44E-06	5.67E-06
F57A10.2	5	12	19	6	2.80E-06	2.65E-06	1.82E-06	2.25E-06
F57A10.3	412	374	599	833	2.80E-06	2.65E-06	1.82E-06	2.25E-06
F57A10.4	53	58	61	59	2.80E-06	2.65E-06	2.53E-06	2.25E-06
F57A10.5	5	10	11	6	1.92E-05	1.64E-05	1.81E-05	3.11E-05
F57A10.6	5	1	6	1	8.74E-06	9.02E-06	6.54E-06	7.80E-06
F57A8.1.1	80	91	68	101	2.80E-06	2.65E-06	1.82E-06	2.25E-06
F57A8.1.2	74	80	60	100	2.80E-06	2.65E-06	1.82E-06	2.25E-06
F57A8.2a	287	372	365	360	8.62E-06	9.28E-06	4.77E-06	8.75E-06
F57A8.2b	273	354	345	320	8.65E-06	8.83E-06	4.56E-06	9.38E-06
F57A8.3	4	6	9	6	1.96E-05	2.40E-05	1.62E-05	1.97E-05
F57A8.4	17	22	17	18	2.20E-05	2.70E-05	1.81E-05	2.07E-05
F57A8.5	6	4	4	2	2.80E-06	2.65E-06	1.82E-06	2.25E-06
F57A8.6	20	21	13	14	2.80E-06	2.65E-06	1.82E-06	2.25E-06
F57A8.7	7	8	2	10	2.80E-06	2.65E-06	1.82E-06	2.25E-06
F57A8.8	43	58	56	58	2.80E-06	2.65E-06	1.82E-06	2.25E-06
F57B1.1	9	12	12	9	2.80E-06	2.65E-06	1.82E-06	2.25E-06
F57B1.3	5070	4821	5922	7402	2.03E-05	2.59E-05	1.72E-05	2.20E-05
F57B1.4.1	9457	10296	11720	13715	2.80E-06	2.65E-06	1.82E-06	2.25E-06
F57B1.4.2	7162	7720	9480	12451	5.47E-04	4.91E-04	4.16E-04	6.42E-04
F57B1.5	12	29	4	7	9.65E-04	9.92E-04	7.78E-04	1.12E-03
F57B1.6a	10	13	9	6	7.23E-04	7.36E-04	6.22E-04	1.01E-03
F57B1.6b	8	9	5	3	2.83E-06	6.48E-06	1.82E-06	2.25E-06
F57B1.7	5	7	3	4	2.80E-06	2.65E-06	1.82E-06	2.25E-06
F57B1.8	1	8	4	2	2.80E-06	2.65E-06	1.82E-06	2.25E-06
F57B10.1.1	1342	1530	1767	2599	2.80E-06	2.65E-06	1.82E-06	2.25E-06
F57B10.1.2	1074	1220	1479	2039	2.80E-06	2.65E-06	1.82E-06	2.25E-06
F57B10.10.1	605	826	811	406	5.16E-05	5.56E-05	4.42E-05	8.03E-05
F57B10.10.2	567	779	664	390	4.07E-05	4.37E-05	3.65E-05	6.21E-05
F57B10.11	206	311	308	280	1.41E-04	1.82E-04	1.23E-04	7.62E-05
F57B10.12	754	720	1257	1181	1.33E-04	1.72E-04	1.01E-04	7.34E-05
F57B10.14.1	1057	1065	2483	544	3.62E-05	5.17E-05	3.52E-05	3.95E-05
F57B10.14.2	855	898	1630	399	8.76E-05	7.90E-05	9.50E-05	1.10E-04
F57B10.3a	697	1116	721	1000	4.18E-04	3.98E-04	6.39E-04	1.73E-04
F57B10.3b.1	643	1041	665	937	3.40E-04	3.37E-04	4.21E-04	1.27E-04
F57B10.3b.2	645	1042	669	940	4.11E-05	6.22E-05	2.77E-05	4.74E-05
F57B10.3b.3	647	1043	678	943	4.30E-05	6.58E-05	2.89E-05	5.03E-05
F57B10.3b.4	663	1061	713	959	4.35E-05	6.64E-05	2.94E-05	5.10E-05
F57B10.4	416	432	510	781	4.61E-05	7.03E-05	3.15E-05	5.40E-05
F57B10.5	472	595	401	409	4.73E-05	7.16E-05	3.31E-05	5.50E-05
F57B10.6	315	376	271	542	2.18E-05	2.13E-05	1.73E-05	3.28E-05
F57B10.7	876	967	1013	1546	7.18E-05	8.55E-05	3.97E-05	5.00E-05
F57B10.8	137	145	99	120	1.42E-05	1.60E-05	7.93E-06	1.96E-05
F57B10.9	60	98	50	102	4.59E-05	4.78E-05	3.45E-05	6.50E-05
F57B7.1a	43	54	43	54	1.90E-05	1.90E-05	8.95E-06	1.34E-05
F57B7.1b	42	55	41	54	4.26E-06	6.53E-06	2.30E-06	5.80E-06
F57B7.2	11	24	15	24	2.80E-06	3.20E-06	1.82E-06	2.72E-06
F57B7.3	172	183	92	56	2.80E-06	3.31E-06	1.82E-06	2.74E-06
F57B7.4	31	92	42	34	2.80E-06	2.91E-06	1.82E-06	2.47E-06
F57B9.1	105	184	140	122	2.17E-05	2.18E-05	7.54E-06	5.67E-06
F57B9.10a	1620	1459	2084	2500	2.80E-06	6.14E-06	1.93E-06	2.25E-06
F57B9.10b.1	1321	1204	1665	2018	1.44E-05	2.39E-05	1.25E-05	1.35E-05
F57B9.10b.2	1257	1160	1588	1985	1.09E-04	9.27E-05	9.12E-05	1.35E-04
F57B9.2	4655	3407	6988	10383	1.16E-04	1.00E-04	9.53E-05	1.43E-04
F57B9.3	892	683	736	985	1.11E-04	9.66E-05	9.11E-05	1.40E-04
F57B9.4a	113	151	123	145	6.37E-05	4.40E-05	6.22E-05	1.14E-04
F57B9.4b	116	161	127	153	9.15E-05	6.62E-05	4.91E-05	8.11E-05
F57B9.4c	80	121	90	111	1.04E-05	1.31E-05	7.34E-06	1.07E-05

F57B9.4d	70	105	82	100	1.00E-05	1.31E-05	7.12E-06	1.06E-05
F57B9.4e	110	161	126	150	1.10E-05	1.58E-05	8.07E-06	1.23E-05
F57B9.5.1	788	652	851	1065	1.26E-05	1.79E-05	9.62E-06	1.45E-05
F57B9.5.2	700	589	709	942	1.04E-05	1.44E-05	7.74E-06	1.14E-05
F57B9.6a.1	8687	7280	8918	11583	5.64E-05	4.41E-05	3.96E-05	6.12E-05
F57B9.6a.2	8215	6908	8579	11401	5.79E-05	4.60E-05	3.82E-05	6.26E-05
F57B9.6b.1	8215	6908	8579	11401	5.81E-04	4.60E-04	3.88E-04	6.22E-04
F57B9.6b.2	3905	3437	4694	6235	5.81E-04	4.61E-04	3.95E-04	6.47E-04
F57B9.7a	236	303	221	314	5.81E-04	4.61E-04	3.95E-04	6.47E-04
F57B9.7b	239	285	239	310	1.10E-03	9.14E-04	8.60E-04	1.41E-03
F57B9.8	19	31	17	12	1.99E-05	2.41E-05	1.21E-05	2.13E-05
F57B9.9	258	353	85	97	2.37E-05	2.68E-05	1.55E-05	2.47E-05
F57C12.1	51	162	38	65	2.80E-06	2.65E-06	1.82E-06	2.25E-06
F57C12.2	24	29	22	25	2.37E-05	3.06E-05	5.07E-06	7.15E-06
F57C12.3	0	0	2	3	2.80E-06	7.41E-06	1.82E-06	2.52E-06
F57C12.4	85	151	99	179	2.80E-06	2.65E-06	1.82E-06	2.25E-06
F57C12.5a	315	608	319	620	2.80E-06	2.65E-06	1.82E-06	2.25E-06
F57C12.5b	322	623	329	641	2.80E-06	3.41E-06	1.82E-06	3.44E-06
F57C12.5c	315	609	326	628	7.59E-06	1.38E-05	4.99E-06	1.20E-05
F57C12.5d.1	73	173	78	120	7.73E-06	1.41E-05	5.14E-06	1.23E-05
F57C12.5d.2	79	186	85	143	7.56E-06	1.38E-05	5.08E-06	1.21E-05
F57C12.5d.3	335	638	347	652	5.91E-06	1.32E-05	4.10E-06	7.78E-06
F57C12.5e	336	637	350	654	6.52E-06	1.45E-05	4.56E-06	9.47E-06
F57C2.1	93	126	150	135	7.78E-06	1.40E-05	5.25E-06	1.22E-05
F57C2.2	129	160	163	201	7.78E-06	1.39E-05	5.27E-06	1.21E-05
F57C2.3	114	110	135	169	1.15E-05	1.48E-05	1.21E-05	1.34E-05
F57C2.4	126	156	93	81	1.60E-05	1.88E-05	1.32E-05	2.01E-05
F57C2.5.1	270	348	379	437	1.69E-05	1.54E-05	1.30E-05	2.01E-05
F57C2.5.2	266	335	365	434	4.63E-05	5.41E-05	2.22E-05	2.39E-05
F57C2.6	232	242	443	523	2.31E-05	2.81E-05	2.11E-05	3.00E-05
F57C7.1a	321	375	413	660	2.38E-05	2.83E-05	2.12E-05	3.12E-05
F57C7.1b	326	372	407	660	1.11E-05	1.10E-05	1.38E-05	2.02E-05
F57C7.2a	124	166	118	169	8.71E-06	9.60E-06	7.29E-06	1.44E-05
F57C7.2b	127	169	120	171	7.95E-06	8.57E-06	6.47E-06	1.29E-05
F57C7.3a	273	345	289	442	5.82E-06	7.38E-06	3.61E-06	6.39E-06
F57C7.3b	167	192	145	263	5.85E-06	7.35E-06	3.59E-06	6.32E-06
F57C7.4	18	29	38	18	2.04E-05	2.44E-05	1.41E-05	2.65E-05
F57C9.1a	103	171	167	120	2.09E-05	2.27E-05	1.18E-05	2.64E-05
F57C9.1b	113	184	165	124	2.80E-06	2.65E-06	1.82E-06	2.25E-06
F57C9.2	31	60	42	71	1.10E-05	1.73E-05	1.16E-05	1.03E-05
F57C9.3	17	22	15	18	1.07E-05	1.65E-05	1.01E-05	9.42E-06
F57C9.4a	395	375	453	684	2.80E-06	4.29E-06	2.06E-06	4.30E-06
F57C9.4b	468	502	555	839	4.93E-06	6.00E-06	2.82E-06	4.18E-06
F57C9.5	388	383	671	844	2.16E-05	1.94E-05	1.61E-05	3.00E-05
F57C9.6	9	14	9	4	1.97E-05	1.99E-05	1.52E-05	2.83E-05
F57C9.7	63	100	106	126	1.87E-05	1.75E-05	2.11E-05	3.27E-05
F57E7.1	5	3	3	1	2.80E-06	2.65E-06	1.82E-06	2.25E-06
F57E7.2	7	3	4	7	5.68E-06	8.54E-06	6.23E-06	9.15E-06
F57E7.3	3	1	5	3	2.80E-06	2.65E-06	1.82E-06	2.25E-06
F57F10.1a	155	206	137	187	2.80E-06	2.65E-06	1.82E-06	2.25E-06
F57F10.1b	133	165	118	138	2.80E-06	2.65E-06	1.82E-06	2.25E-06
F57F10.1c	143	186	130	159	5.38E-06	6.74E-06	3.10E-06	5.22E-06
F57F4.1	32	65	18	18	5.57E-06	6.53E-06	3.21E-06	4.63E-06
F57F4.2	9	10	11	4	5.35E-06	6.59E-06	3.17E-06	4.79E-06
F57F4.3	17617	13327	16708	24428	2.80E-06	4.97E-06	1.82E-06	2.25E-06
F57F4.4.1	18474	14275	17472	25466	2.80E-06	2.65E-06	1.82E-06	2.25E-06
F57F5.1	3963	4857	4160	5933	2.98E-04	2.13E-04	1.84E-04	3.32E-04
F57F5.2	26	28	26	29	3.21E-04	2.35E-04	1.98E-04	3.56E-04
F57F5.3	12	18	11	9	3.44E-04	3.98E-04	2.35E-04	4.13E-04

F57F5.4a.1	82	173	101	137	2.80E-06	2.65E-06	1.82E-06	2.25E-06
F57F5.4a.2	84	174	104	139	2.80E-06	2.65E-06	1.82E-06	2.25E-06
F57G12.1.1	356	301	413	585	4.59E-06	9.18E-06	3.68E-06	6.18E-06
F57G12.1.2	346	289	406	581	4.87E-06	9.55E-06	3.94E-06	6.48E-06
F57G12.2	6	7	6	6	1.88E-05	1.50E-05	1.42E-05	2.48E-05
F57G12.t1	1	0	0	0	1.83E-05	1.44E-05	1.39E-05	2.46E-05
F57G4.1	14	11	18	10	2.80E-06	2.65E-06	1.82E-06	2.25E-06
F57G4.4	3	38	7	38	2.80E-06	2.65E-06	1.82E-06	2.25E-06
F57G4.5	5	38	9	63	2.80E-06	2.65E-06	1.82E-06	2.25E-06
F57G4.6	7	41	15	45	2.80E-06	3.81E-06	1.82E-06	3.24E-06
F57G4.7	25	172	43	235	2.80E-06	5.16E-06	1.82E-06	7.26E-06
F57G4.8	28	80	53	165	2.80E-06	5.66E-06	1.82E-06	5.26E-06
F57G4.9	6	35	13	32	2.80E-06	8.31E-06	1.82E-06	9.67E-06
F57G8.1	3	8	3	2	2.80E-06	7.14E-06	3.26E-06	1.25E-05
F57G8.3	4	5	4	4	2.80E-06	5.32E-06	1.82E-06	4.14E-06
F57G8.4	4	3	5	3	2.80E-06	2.65E-06	1.82E-06	2.25E-06
F57G8.5	21	22	36	23	2.80E-06	2.65E-06	1.82E-06	2.25E-06
F57G8.6	12	24	15	20	2.80E-06	2.65E-06	1.82E-06	2.25E-06
F57G8.7	1	2	1	0	2.80E-06	2.65E-06	1.82E-06	2.25E-06
F57G8.8	0	2	3	1	2.80E-06	2.65E-06	1.82E-06	2.25E-06
F57G9.1	5	2	3	3	2.80E-06	2.65E-06	1.82E-06	2.25E-06
F57G9.2	6	9	4	2	2.80E-06	2.65E-06	1.82E-06	2.25E-06
F57G9.3	3	10	6	6	2.80E-06	2.65E-06	1.82E-06	2.25E-06
F57G9.4	5	5	4	4	2.80E-06	2.65E-06	1.82E-06	2.25E-06
F57G9.6	5	4	3	1	2.80E-06	2.65E-06	1.82E-06	2.25E-06
F57G9.7	5	11	2	3	2.80E-06	2.65E-06	1.82E-06	2.25E-06
F57H12.1	2530	2711	2081	2680	2.80E-06	2.65E-06	1.82E-06	2.25E-06
F57H12.2	10	17	9	14	2.80E-06	2.65E-06	1.82E-06	2.25E-06
F57H12.3	63	103	14	18	2.78E-04	2.82E-04	1.49E-04	2.37E-04
F57H12.4	6	15	10	2	2.80E-06	2.65E-06	1.82E-06	2.25E-06
F57H12.5	89	97	37	49	1.11E-05	1.71E-05	1.82E-06	2.54E-06
F57H12.6	224	125	50	24	2.80E-06	2.65E-06	1.82E-06	2.25E-06
F57H12.7	96	160	100	83	6.02E-06	6.19E-06	1.82E-06	2.65E-06
F58A3.1a.1	152	164	95	227	3.63E-05	1.92E-05	5.27E-06	3.13E-06
F58A3.1a.2	144	162	93	224	1.11E-05	1.74E-05	7.51E-06	7.69E-06
F58A3.1b	141	153	87	214	8.04E-06	8.17E-06	3.26E-06	9.63E-06
F58A3.1c	200	214	143	251	8.37E-06	8.89E-06	3.52E-06	1.05E-05
F58A3.2a	78	120	42	121	8.65E-06	8.86E-06	3.46E-06	1.05E-05
F58A3.2b.1	91	129	54	125	9.07E-06	9.18E-06	4.23E-06	9.15E-06
F58A3.2b.2	89	127	54	126	2.80E-06	3.44E-06	1.82E-06	2.97E-06
F58A3.2b.3	88	127	42	117	2.94E-06	3.91E-06	1.82E-06	3.24E-06
F58A3.2c	85	127	44	125	2.83E-06	3.78E-06	1.82E-06	3.19E-06
F58A3.2d	97	131	57	133	2.88E-06	3.94E-06	1.82E-06	3.10E-06
F58A3.2e	74	114	41	118	2.80E-06	3.94E-06	1.82E-06	3.28E-06
F58A3.3	7	6	11	5	2.83E-06	3.62E-06	1.82E-06	3.13E-06
F58A3.4	3	4	7	2	2.80E-06	3.78E-06	1.82E-06	3.33E-06
F58A3.5	97	166	85	76	2.80E-06	2.65E-06	1.82E-06	2.25E-06
F58A4.1	3	4	3	8	2.80E-06	2.65E-06	1.82E-06	2.25E-06
F58A4.10.1	1207	1503	1265	1403	2.43E-05	3.93E-05	1.39E-05	1.53E-05
F58A4.10.2	967	1177	969	1154	2.80E-06	2.65E-06	1.82E-06	2.25E-06
F58A4.11	268	314	170	270	1.55E-04	1.83E-04	1.06E-04	1.45E-04
F58A4.12	23	32	5	4	1.72E-04	1.97E-04	1.12E-04	1.65E-04
F58A4.14	12	7	7	9	1.03E-05	1.14E-05	4.26E-06	8.34E-06
F58A4.2	154	127	467	237	3.11E-06	4.07E-06	1.82E-06	2.25E-06
F58A4.3	367	378	762	678	2.80E-06	2.65E-06	1.82E-06	2.25E-06
F58A4.4	282	313	514	458	3.59E-05	2.80E-05	7.09E-05	4.44E-05
F58A4.5	20	10	16	12	4.06E-05	3.95E-05	5.49E-05	6.03E-05
F58A4.6	89	83	76	143	2.49E-05	2.61E-05	2.95E-05	3.25E-05
F58A4.7a.1	278	487	285	415	2.80E-06	2.65E-06	1.82E-06	2.25E-06

F58A4.7a.2	170	291	175	238	2.00E-05	1.76E-05	1.11E-05	2.58E-05
F58A4.7b.1	174	294	176	240	1.55E-05	2.57E-05	1.04E-05	1.86E-05
F58A4.7b.2	174	296	178	242	1.40E-05	2.27E-05	9.38E-06	1.58E-05
F58A4.7c	212	363	204	305	1.43E-05	2.28E-05	9.40E-06	1.58E-05
F58A4.7d	50	74	34	46	1.42E-05	2.29E-05	9.48E-06	1.59E-05
F58A4.8.1	462	493	610	799	1.51E-05	2.44E-05	9.46E-06	1.75E-05
F58A6.1	49	106	29	40	1.13E-05	1.58E-05	4.99E-06	8.34E-06
F58A6.10	7	4	8	1	3.52E-05	3.55E-05	3.02E-05	4.89E-05
F58A6.11	9	7	6	3	6.55E-06	1.34E-05	2.53E-06	4.30E-06
F58A6.2	4	4	5	1	2.80E-06	2.65E-06	1.82E-06	2.25E-06
F58A6.4	20	31	9	9	2.80E-06	2.65E-06	1.82E-06	2.25E-06
F58A6.5	43	58	19	19	2.80E-06	2.65E-06	1.82E-06	2.25E-06
F58A6.6	14	2	13	19	2.80E-06	2.65E-06	1.82E-06	2.25E-06
F58B3.1	337	1592	282	1213	5.10E-06	6.51E-06	1.82E-06	2.25E-06
F58B3.2	37	127	19	139	2.80E-06	2.65E-06	1.82E-06	2.25E-06
F58B3.3	40	136	19	140	5.44E-05	2.43E-04	2.96E-05	1.57E-04
F58B3.4	518	571	314	518	6.38E-06	2.07E-05	2.13E-06	1.93E-05
F58B3.5	1539	1736	1879	2584	6.83E-06	2.19E-05	2.11E-06	1.92E-05
F58B3.7	186	204	225	181	2.95E-05	3.07E-05	1.16E-05	2.37E-05
F58B3.8	3	8	8	3	5.77E-05	6.15E-05	4.59E-05	7.78E-05
F58B4.1a	17	24	11	25	1.74E-05	1.80E-05	1.37E-05	1.36E-05
F58B4.1b	23	37	22	29	2.80E-06	2.65E-06	1.82E-06	2.25E-06
F58B4.2	2	2	9	2	2.80E-06	2.65E-06	1.82E-06	2.25E-06
F58B4.3	21	50	26	12	2.80E-06	2.65E-06	1.82E-06	2.25E-06
F58B4.4	1	3	3	2	2.80E-06	2.65E-06	2.42E-06	2.25E-06
F58B4.5	188	210	157	199	3.89E-06	8.78E-06	3.13E-06	2.25E-06
F58B4.6	6	11	8	10	2.80E-06	2.65E-06	1.82E-06	2.25E-06
F58B6.1	5	8	14	3	1.50E-05	1.59E-05	8.18E-06	1.28E-05
F58B6.2	75	114	75	106	2.80E-06	2.91E-06	1.82E-06	2.25E-06
F58B6.3a	106	85	130	152	2.80E-06	2.65E-06	1.82E-06	2.25E-06
F58B6.3b	307	227	379	524	3.08E-06	4.42E-06	2.00E-06	3.49E-06
F58D12.1	2	2	22	5	8.65E-06	6.56E-06	6.91E-06	9.96E-06
F58D12.2	12	17	41	11	1.72E-05	1.20E-05	1.38E-05	2.36E-05
F58D12.3	19	26	62	15	2.80E-06	2.65E-06	3.72E-06	2.25E-06
F58D2.1	6	5	14	6	2.80E-06	2.65E-06	1.82E-06	2.25E-06
F58D2.2	53	109	34	32	2.80E-06	2.65E-06	1.97E-06	2.25E-06
F58D5.1.1	1448	1202	793	1377	2.80E-06	2.65E-06	1.82E-06	2.25E-06
F58D5.1.2	1420	1180	764	1344	3.25E-06	6.30E-06	1.82E-06	2.25E-06
F58D5.2a	10	35	40	17	6.73E-05	5.28E-05	2.40E-05	5.14E-05
F58D5.3	11	15	19	14	6.79E-05	5.33E-05	2.38E-05	5.16E-05
F58D5.4a	289	273	334	444	2.80E-06	6.53E-06	5.14E-06	2.70E-06
F58D5.4b	277	256	317	412	2.80E-06	2.65E-06	1.82E-06	2.25E-06
F58D5.5	106	116	210	191	1.76E-05	1.57E-05	1.32E-05	2.17E-05
F58D5.6	19	31	23	10	1.80E-05	1.57E-05	1.34E-05	2.15E-05
F58D5.7	33	54	16	19	1.97E-05	2.03E-05	2.54E-05	2.85E-05
F58D5.8	10	17	10	9	2.80E-06	2.65E-06	1.82E-06	2.25E-06
F58D5.9	8	17	38	34	4.56E-06	7.04E-06	1.82E-06	2.25E-06
F58D7.1	3	6	4	1	2.80E-06	2.65E-06	1.82E-06	2.25E-06
F58E1.10	2	5	5	4	2.80E-06	2.72E-06	4.17E-06	4.61E-06
F58E1.11	4	7	11	5	2.80E-06	2.65E-06	1.82E-06	2.25E-06
F58E1.12	3	8	9	4	2.80E-06	2.65E-06	1.82E-06	2.25E-06
F58E1.13	2	8	3	3	2.80E-06	2.65E-06	1.82E-06	2.25E-06
F58E1.14	3	3	7	2	2.80E-06	2.65E-06	1.82E-06	2.25E-06
F58E1.2	12	9	4	3	2.80E-06	2.65E-06	1.82E-06	2.25E-06
F58E1.3	5	15	10	7	2.80E-06	2.65E-06	1.82E-06	2.25E-06
F58E1.4	3	4	9	7	2.80E-06	2.65E-06	1.82E-06	2.25E-06
F58E1.5	8	18	4	3	2.80E-06	2.65E-06	1.82E-06	2.25E-06
F58E1.6a	7	10	3	4	2.80E-06	2.65E-06	1.82E-06	2.25E-06
F58E1.6b	8	10	3	4	2.80E-06	2.65E-06	1.82E-06	2.25E-06

F58E1.7	4	2	4	1	2.80E-06	2.65E-06	1.82E-06	2.25E-06
F58E1.8	3	6	3	5	2.80E-06	2.65E-06	1.82E-06	2.25E-06
F58E1.9	3	6	4	3	2.80E-06	2.65E-06	1.82E-06	2.25E-06
F58E10.1a	430	456	638	874	2.80E-06	2.65E-06	1.82E-06	2.25E-06
F58E10.1b	567	569	828	1117	2.80E-06	2.65E-06	1.82E-06	2.25E-06
F58E10.2	5	5	3	3	2.31E-05	2.31E-05	2.23E-05	3.77E-05
F58E10.3a.1	1878	2221	1742	2950	2.34E-05	2.22E-05	2.23E-05	3.71E-05
F58E10.3a.2	1754	2058	1602	2826	2.80E-06	2.65E-06	1.82E-06	2.25E-06
F58E10.3a.3	1528	1802	1478	2538	8.72E-05	9.74E-05	5.26E-05	1.10E-04
F58E10.3a.4	1570	1864	1539	2584	9.59E-05	1.06E-04	5.70E-05	1.24E-04
F58E10.3a.5	1465	1719	1420	2478	9.45E-05	1.05E-04	5.95E-05	1.26E-04
F58E10.3a.6	1486	1747	1437	2494	9.15E-05	1.03E-04	5.84E-05	1.21E-04
F58E10.4	219	409	275	243	9.45E-05	1.05E-04	5.96E-05	1.28E-04
F58E10.5	6	9	9	4	9.29E-05	1.03E-04	5.85E-05	1.25E-04
F58E10.6	6	3	3	1	2.32E-05	4.09E-05	1.89E-05	2.06E-05
F58E10.7	10	36	32	13	2.80E-06	2.65E-06	1.82E-06	2.25E-06
F58E2.1	2	3	2	0	2.80E-06	2.65E-06	1.82E-06	2.25E-06
F58E2.10	5	5	24	3	2.80E-06	5.37E-06	3.28E-06	2.25E-06
F58E2.2	2	4	1	1	2.80E-06	2.65E-06	1.82E-06	2.25E-06
F58E2.3	21	19	20	26	2.80E-06	2.65E-06	1.82E-06	2.25E-06
F58E2.4	17	36	19	18	2.80E-06	2.65E-06	1.82E-06	2.25E-06
F58E2.5	2	2	3	1	2.80E-06	2.65E-06	1.82E-06	2.25E-06
F58E2.6	7	3	2	2	2.80E-06	2.65E-06	1.82E-06	2.25E-06
F58E2.7	3	4	3	0	2.80E-06	2.65E-06	1.82E-06	2.25E-06
F58E2.8	6	0	2	3	2.80E-06	2.65E-06	1.82E-06	2.25E-06
F58E2.9	5	7	8	4	2.80E-06	2.65E-06	1.82E-06	2.25E-06
F58E6.10	11	5	6	6	2.80E-06	2.65E-06	1.82E-06	2.25E-06
F58E6.11	7	15	7	3	2.80E-06	2.65E-06	1.82E-06	2.25E-06
F58E6.1a	243	359	96	87	2.80E-06	2.65E-06	1.82E-06	2.25E-06
F58E6.1b	46	73	44	51	2.80E-06	2.65E-06	1.82E-06	2.25E-06
F58E6.3	3	4	2	2	9.30E-06	1.30E-05	2.39E-06	2.68E-06
F58E6.4	2	1	3	0	2.80E-06	3.36E-06	1.82E-06	2.25E-06
F58E6.5	56	130	41	34	2.80E-06	2.65E-06	1.82E-06	2.25E-06
F58E6.6	3	6	3	1	2.80E-06	2.65E-06	1.82E-06	2.25E-06
F58E6.7	16	8	4	4	6.30E-06	1.38E-05	2.99E-06	3.06E-06
F58E6.8	3	4	6	3	2.80E-06	2.65E-06	1.82E-06	2.25E-06
F58F12.1.1	2275	1874	1698	1899	4.76E-06	2.65E-06	1.82E-06	2.25E-06
F58F12.1.2	2125	1723	1537	1814	2.80E-06	2.65E-06	1.82E-06	2.25E-06
F58F12.2	6	20	18	8	3.49E-04	2.72E-04	1.70E-04	2.34E-04
F58F12.3	22	35	34	20	3.80E-04	2.91E-04	1.79E-04	2.61E-04
F58F12.4	24	23	6	13	2.80E-06	5.61E-06	3.46E-06	2.25E-06
F58F6.1	397	498	225	144	2.80E-06	2.67E-06	1.82E-06	2.25E-06
F58F6.2	7	6	7	2	5.94E-06	5.37E-06	1.82E-06	2.59E-06
F58F6.3	4	3	3	1	5.19E-05	6.16E-05	1.92E-05	1.51E-05
F58F6.4	132	166	270	318	2.80E-06	2.65E-06	1.82E-06	2.25E-06
F58F6.5	2	3	3	1	2.80E-06	2.65E-06	1.82E-06	2.25E-06
F58F6.6	7	4	8	8	1.37E-05	1.62E-05	1.82E-05	2.65E-05
F58F6.t1	0	0	5	0	2.80E-06	2.65E-06	1.82E-06	2.25E-06
F58F9.1	4	4	2	2	2.80E-06	2.65E-06	1.82E-06	2.25E-06
F58F9.10	1	2	4	2	2.80E-06	2.65E-06	4.99E-06	2.25E-06
F58F9.11	2	0	1	2	2.80E-06	2.65E-06	1.82E-06	2.25E-06
F58F9.3a	22	37	15	19	2.80E-06	2.65E-06	1.82E-06	2.25E-06
F58F9.3b	39	67	34	38	2.80E-06	2.65E-06	1.82E-06	2.25E-06
F58F9.4	27	21	9	18	4.51E-06	7.17E-06	2.00E-06	3.13E-06
F58F9.6	6	5	5	5	4.31E-06	6.98E-06	2.44E-06	3.37E-06
F58F9.7.1	109	240	96	140	3.19E-06	2.65E-06	1.82E-06	2.25E-06
F58F9.7.2	107	231	94	138	2.80E-06	2.65E-06	1.82E-06	2.25E-06
F58F9.8	1	5	5	1	5.88E-06	1.22E-05	3.37E-06	6.07E-06
F58F9.9	3	8	2	4	5.96E-06	1.21E-05	3.41E-06	6.16E-06

F58G1.1	1224	1228	1542	2343	2.80E-06	2.65E-06	1.82E-06	2.25E-06
F58G1.10	2	1	3	0	2.80E-06	2.65E-06	1.82E-06	2.25E-06
F58G1.2.1	256	226	349	481	4.37E-05	4.14E-05	3.58E-05	6.71E-05
F58G1.2.2	241	209	320	428	2.80E-06	2.65E-06	1.82E-06	2.25E-06
F58G1.3	47	81	38	40	1.78E-05	1.48E-05	1.58E-05	2.68E-05
F58G1.4	722	753	1134	643	1.83E-05	1.50E-05	1.59E-05	2.62E-05
F58G1.5	42	73	13	12	4.70E-06	7.67E-06	2.48E-06	3.22E-06
F58G1.6	48	65	46	47	1.06E-04	1.04E-04	1.08E-04	7.55E-05
F58G1.7	41	35	94	38	4.87E-06	8.01E-06	1.82E-06	2.25E-06
F58G1.8	2	4	2	7	4.06E-06	5.21E-06	2.53E-06	3.19E-06
F58G1.9	2	3	2	0	2.80E-06	2.65E-06	3.33E-06	2.25E-06
F58G11.1a	1015	1008	1232	1691	2.80E-06	2.65E-06	1.82E-06	2.50E-06
F58G11.1b	998	984	1205	1669	2.80E-06	2.65E-06	1.82E-06	2.25E-06
F58G11.2	589	578	780	1478	4.27E-05	4.00E-05	3.37E-05	5.71E-05
F58G11.3a	610	601	1095	1423	4.29E-05	3.99E-05	3.37E-05	5.76E-05
F58G11.4	7	19	12	17	1.99E-05	1.85E-05	1.72E-05	4.02E-05
F58G11.5.1	322	342	301	558	3.04E-05	2.82E-05	3.55E-05	5.69E-05
F58G11.5.2	304	327	279	544	2.80E-06	2.65E-06	1.82E-06	2.25E-06
F58G11.6	308	341	469	593	1.69E-05	1.70E-05	1.03E-05	2.36E-05
F58G4.1	287	321	193	285	1.67E-05	1.70E-05	1.00E-05	2.41E-05
F58G4.2	8	3	4	4	2.00E-05	2.09E-05	1.98E-05	3.09E-05
F58G4.3	5	6	14	6	5.24E-06	5.53E-06	2.30E-06	4.16E-06
F58G4.4	10	10	8	6	2.80E-06	2.65E-06	1.82E-06	2.25E-06
F58G4.5	8	3	15	1	2.80E-06	2.65E-06	1.82E-06	2.25E-06
F58G4.6	7	2	6	1	2.80E-06	2.65E-06	1.82E-06	2.25E-06
F58G4.7	8	7	23	6	2.80E-06	2.65E-06	1.82E-06	2.25E-06
F58G6.1.1	222	354	154	237	2.80E-06	2.65E-06	1.82E-06	2.25E-06
F58G6.1.2	167	286	118	200	2.80E-06	2.65E-06	1.82E-06	2.25E-06
F58G6.2	8	9	21	14	1.27E-05	1.91E-05	5.72E-06	1.09E-05
F58G6.3	81	93	133	37	1.34E-05	2.17E-05	6.18E-06	1.29E-05
F58G6.4	16	25	12	12	2.80E-06	2.65E-06	1.82E-06	2.25E-06
F58G6.5a	88	146	111	186	2.24E-05	2.43E-05	2.39E-05	8.21E-06
F58G6.5b	86	145	109	192	2.80E-06	2.65E-06	1.82E-06	2.25E-06
F58G6.5c	95	160	116	205	5.32E-06	8.36E-06	4.37E-06	9.06E-06
F58G6.6	25	27	24	12	5.18E-06	8.25E-06	4.28E-06	9.29E-06
F58G6.7	159	95	146	103	4.79E-06	7.62E-06	3.81E-06	8.32E-06
F58G6.8	2	8	5	0	2.80E-06	2.65E-06	1.82E-06	2.25E-06
F58H1.1a	797	890	880	1394	2.18E-05	1.23E-05	1.31E-05	1.14E-05
F58H1.1b	826	939	911	1425	2.80E-06	2.65E-06	1.82E-06	2.25E-06
F58H1.2	41	127	31	28	2.60E-05	2.74E-05	1.87E-05	3.65E-05
F58H1.3a	229	263	356	304	2.57E-05	2.76E-05	1.85E-05	3.56E-05
F58H1.4	7	9	6	6	6.69E-06	1.96E-05	3.30E-06	3.69E-06
F58H1.5.1	29	30	33	30	1.63E-05	1.77E-05	1.65E-05	1.74E-05
F58H1.5.2	23	28	30	26	2.80E-06	3.02E-06	1.82E-06	2.25E-06
F58H1.6	53	64	18	22	2.88E-06	2.80E-06	2.13E-06	2.38E-06
F58H1.7a	34	39	34	15	2.80E-06	3.02E-06	2.22E-06	2.38E-06
F58H1.7b	11	25	21	7	7.22E-06	8.23E-06	1.82E-06	2.41E-06
F58H10.1	50	49	21	27	4.68E-06	5.05E-06	3.04E-06	2.25E-06
F58H12.1	222	279	171	342	2.80E-06	4.79E-06	2.77E-06	2.25E-06
F58H7.1	6	17	13	12	1.56E-05	1.44E-05	4.25E-06	6.75E-06
F58H7.2	94	116	108	134	7.22E-06	8.57E-06	3.63E-06	8.95E-06
F58H7.3	11	26	14	8	2.80E-06	2.65E-06	1.82E-06	2.25E-06
F58H7.5	4	1	17	2	6.13E-06	7.17E-06	4.59E-06	7.04E-06
F58H7.7	15	16	16	11	2.80E-06	2.65E-06	1.82E-06	2.25E-06
F58H7.8	89	204	101	113	2.80E-06	2.65E-06	1.82E-06	2.25E-06
F59A1.10	38	107	52	41	2.80E-06	2.65E-06	1.82E-06	2.25E-06
F59A1.12	3	7	14	6	9.80E-06	2.12E-05	7.23E-06	9.99E-06
F59A1.13	4	12	3	5	3.84E-06	1.02E-05	3.43E-06	3.33E-06
F59A1.14	7	5	40	6	2.80E-06	2.65E-06	1.82E-06	2.25E-06

F59A1.15	20	29	13	8	2.80E-06	2.65E-06	1.82E-06	2.25E-06
F59A1.16	0	2	1	2	2.80E-06	2.65E-06	2.75E-06	2.25E-06
F59A1.2	9	5	7	13	2.80E-06	2.65E-06	1.82E-06	2.25E-06
F59A1.3	6	14	98	19	2.80E-06	2.65E-06	1.82E-06	2.25E-06
F59A1.4	7	5	1	4	2.80E-06	2.65E-06	1.82E-06	2.25E-06
F59A1.5	15	8	17	14	2.80E-06	2.65E-06	6.92E-06	2.25E-06
F59A1.6	0	2	2	0	2.80E-06	2.65E-06	1.82E-06	2.25E-06
F59A1.7	37	70	42	71	2.80E-06	2.65E-06	1.82E-06	2.25E-06
F59A1.8	22	27	25	30	2.80E-06	2.65E-06	1.82E-06	2.25E-06
F59A1.9	21	18	13	26	4.31E-06	7.72E-06	3.19E-06	6.66E-06
F59A1.t1	1	0	0	0	2.97E-06	3.41E-06	2.19E-06	3.24E-06
F59A1.t4	0	0	1	0	2.80E-06	2.65E-06	1.82E-06	2.34E-06
F59A1.t5	0	0	0	1	2.80E-06	2.65E-06	1.82E-06	2.25E-06
F59A2.1a	4132	3415	5042	6938	2.80E-06	2.65E-06	1.82E-06	2.25E-06
F59A2.1b.1	4715	3974	5708	7904	2.80E-06	2.65E-06	1.82E-06	2.25E-06
F59A2.1b.2	4788	4032	5806	7934	1.78E-04	1.39E-04	1.42E-04	2.40E-04
F59A2.1b.3	4124	3413	5042	6939	1.74E-04	1.38E-04	1.37E-04	2.34E-04
F59A2.2	146	136	109	244	1.73E-04	1.38E-04	1.37E-04	2.30E-04
F59A2.3.1	1000	1021	1215	1189	1.73E-04	1.35E-04	1.38E-04	2.34E-04
F59A2.3.2	751	727	940	959	1.05E-05	9.20E-06	5.08E-06	1.40E-05
F59A2.4a	205	177	268	326	1.19E-04	1.14E-04	9.38E-05	1.13E-04
F59A2.4b	76	72	90	123	1.17E-04	1.07E-04	9.57E-05	1.21E-04
F59A2.5	72	82	80	71	1.70E-05	1.39E-05	1.45E-05	2.17E-05
F59A2.6	203	240	225	486	1.48E-05	1.33E-05	1.14E-05	1.93E-05
F59A3.1	155	275	154	229	2.16E-05	2.32E-05	1.56E-05	1.71E-05
F59A3.10	5	6	23	8	6.69E-06	7.46E-06	4.83E-06	1.28E-05
F59A3.2a	607	591	763	972	5.29E-06	8.86E-06	3.43E-06	6.27E-06
F59A3.2b	378	340	458	624	2.80E-06	2.65E-06	4.74E-06	2.25E-06
F59A3.3	236	247	243	206	2.31E-05	2.12E-05	1.89E-05	2.97E-05
F59A3.4	433	433	692	756	1.99E-05	1.69E-05	1.57E-05	2.63E-05
F59A3.5.1	50	90	26	70	2.78E-05	2.75E-05	1.86E-05	1.95E-05
F59A3.5.2	48	78	23	70	3.78E-05	3.57E-05	3.93E-05	5.31E-05
F59A3.6.1	120	185	50	148	8.04E-06	1.37E-05	2.71E-06	9.04E-06
F59A3.6.2	114	173	47	144	7.39E-06	1.13E-05	2.31E-06	8.66E-06
F59A3.6.3	87	126	37	104	7.64E-06	1.11E-05	2.08E-06	7.56E-06
F59A3.7	4	8	16	2	8.04E-06	1.15E-05	2.15E-06	8.14E-06
F59A3.8	21	33	21	11	7.92E-06	1.08E-05	2.19E-06	7.60E-06
F59A3.9	32	48	51	60	2.80E-06	2.65E-06	1.82E-06	2.25E-06
F59A6.1	463	599	411	799	2.80E-06	2.65E-06	1.82E-06	2.25E-06
F59A6.10	3	9	6	5	2.80E-06	3.33E-06	2.44E-06	3.53E-06
F59A6.11	3	3	1	0	1.08E-05	1.31E-05	6.21E-06	1.49E-05
F59A6.12	70	116	174	117	2.80E-06	2.65E-06	1.82E-06	2.25E-06
F59A6.2	21	18	4	10	2.80E-06	2.65E-06	1.82E-06	2.25E-06
F59A6.3	12	13	10	14	2.75E-05	4.31E-05	4.45E-05	3.69E-05
F59A6.4	48	84	28	22	6.58E-06	5.34E-06	1.82E-06	2.52E-06
F59A6.5	765	754	1145	1488	2.80E-06	2.65E-06	1.82E-06	2.25E-06
F59A6.6a	43	39	57	42	2.80E-06	3.78E-06	1.82E-06	2.25E-06
F59A6.6c	51	108	58	97	2.14E-05	1.99E-05	2.08E-05	3.34E-05
F59A6.6d	39	45	57	47	7.76E-06	6.67E-06	6.71E-06	6.09E-06
F59A6.8	857	1284	1234	387	7.56E-06	1.51E-05	5.59E-06	1.15E-05
F59A7.1	16	25	23	8	7.11E-06	7.78E-06	6.78E-06	6.90E-06
F59A7.10	3	3	2	0	2.39E-04	3.38E-04	2.24E-04	8.66E-05
F59A7.11	1	0	0	1	2.80E-06	2.65E-06	1.82E-06	2.25E-06
F59A7.12	1	1	5	0	2.80E-06	2.65E-06	1.82E-06	2.25E-06
F59A7.2	7	19	28	6	2.80E-06	2.65E-06	1.82E-06	2.25E-06
F59A7.3	1	7	5	4	2.80E-06	2.65E-06	1.82E-06	2.25E-06
F59A7.4	12	3	12	6	2.80E-06	4.36E-06	4.41E-06	2.25E-06
F59A7.5a	41	25	18	20	2.80E-06	2.65E-06	1.82E-06	2.25E-06
F59A7.5b	30	17	14	7	2.80E-06	2.65E-06	1.82E-06	2.25E-06

F59A7.7	2	4	4	4	6.38E-06	3.68E-06	1.82E-06	2.50E-06
F59A7.8	68	71	52	72	5.91E-06	3.17E-06	1.82E-06	2.25E-06
F59A7.9	7	16	6	8	2.80E-06	2.65E-06	1.82E-06	2.25E-06
F59B1.1	5	2	3	2	4.00E-06	3.94E-06	1.99E-06	3.40E-06
F59B1.10	14	22	34	26	2.80E-06	2.65E-06	1.82E-06	2.25E-06
F59B1.2.1	475	744	464	431	2.80E-06	2.65E-06	1.82E-06	2.25E-06
F59B1.2.2	376	602	381	404	2.80E-06	2.65E-06	1.93E-06	2.25E-06
F59B1.3	5	4	9	4	8.43E-05	1.25E-04	5.36E-05	6.14E-05
F59B1.4	5	6	28	5	6.61E-05	1.00E-04	4.36E-05	5.71E-05
F59B1.5	3	9	6	3	2.80E-06	2.65E-06	1.82E-06	2.25E-06
F59B1.6	5	6	13	4	2.80E-06	2.65E-06	2.55E-06	2.25E-06
F59B1.7	3	5	2	0	2.80E-06	2.65E-06	1.82E-06	2.25E-06
F59B1.8.1	54	145	64	135	2.80E-06	2.65E-06	1.82E-06	2.25E-06
F59B1.8.2	50	137	55	127	2.80E-06	2.65E-06	1.82E-06	2.25E-06
F59B1.9	3	14	21	6	4.59E-06	1.16E-05	3.53E-06	9.22E-06
F59B10.1	572	620	434	514	4.42E-06	1.14E-05	3.15E-06	9.00E-06
F59B10.2	22	21	38	12	2.80E-06	2.65E-06	1.82E-06	2.25E-06
F59B10.3	24	36	8	6	1.75E-05	1.80E-05	8.66E-06	1.27E-05
F59B10.4a	6	42	6	32	2.80E-06	2.65E-06	1.91E-06	2.25E-06
F59B10.4b	17	50	14	41	2.91E-06	4.13E-06	1.82E-06	2.25E-06
F59B10.5	30	42	38	11	2.80E-06	5.40E-06	1.82E-06	3.49E-06
F59B10.6	9	11	4	4	2.80E-06	5.13E-06	1.82E-06	3.58E-06
F59B2.11	23	23	18	6	6.61E-06	8.73E-06	5.45E-06	2.25E-06
F59B2.12	15	10	18	15	3.16E-06	3.65E-06	1.82E-06	2.25E-06
F59B2.13	27	47	40	31	2.80E-06	2.65E-06	1.82E-06	2.25E-06
F59B2.2	688	636	909	1133	2.80E-06	2.65E-06	1.82E-06	2.25E-06
F59B2.5	100	180	123	141	2.80E-06	3.02E-06	1.82E-06	2.25E-06
F59B2.6	1036	849	1484	1762	4.54E-05	3.96E-05	3.90E-05	6.00E-05
F59B2.8	3	17	17	18	6.69E-06	1.14E-05	5.36E-06	7.58E-06
F59B2.9	9	14	20	16	6.54E-05	5.06E-05	6.10E-05	8.94E-05
F59B8.1a	3	6	14	5	2.80E-06	2.65E-06	1.82E-06	2.25E-06
F59B8.1b	3	4	15	4	2.80E-06	2.65E-06	1.82E-06	2.25E-06
F59B8.2.1	2104	2725	2745	3249	2.80E-06	2.65E-06	1.82E-06	2.25E-06
F59B8.2.2	2289	2874	2899	3307	2.80E-06	2.65E-06	1.82E-06	2.25E-06
F59C12.1	23	31	11	24	1.64E-04	2.00E-04	1.39E-04	2.03E-04
F59C12.2a	12	19	28	22	2.03E-04	2.41E-04	1.67E-04	2.36E-04
F59C12.3	81	157	47	128	2.80E-06	2.65E-06	1.82E-06	2.25E-06
F59C6.10	8	10	15	8	2.80E-06	2.65E-06	1.82E-06	2.25E-06
F59C6.11	36	45	29	14	4.62E-06	8.44E-06	1.82E-06	5.85E-06
F59C6.12	19	11	35	22	2.80E-06	2.65E-06	1.82E-06	2.25E-06
F59C6.14a	26	28	21	12	4.87E-06	5.77E-06	2.55E-06	2.25E-06
F59C6.14b	20	15	10	6	4.09E-06	2.65E-06	4.88E-06	3.80E-06
F59C6.2	12	9	11	7	6.55E-06	6.67E-06	3.44E-06	2.43E-06
F59C6.3	16	25	8	16	6.72E-06	4.76E-06	2.19E-06	2.25E-06
F59C6.4	192	201	310	284	2.80E-06	2.65E-06	1.82E-06	2.25E-06
F59C6.5.1	1115	1729	1243	998	2.86E-06	4.21E-06	1.82E-06	2.29E-06
F59C6.5.2	1056	1669	971	925	3.14E-05	3.11E-05	3.30E-05	3.73E-05
F59C6.6	18	28	10	8	1.34E-04	1.96E-04	9.72E-05	9.63E-05
F59C6.7	12	18	9	11	1.51E-04	2.25E-04	9.03E-05	1.06E-04
F59C6.8	12	16	13	14	3.00E-06	4.42E-06	1.82E-06	2.25E-06
F59D12.1	35	36	45	35	2.80E-06	2.65E-06	1.82E-06	2.25E-06
F59D12.2	8	10	3	7	2.80E-06	2.65E-06	1.82E-06	2.25E-06
F59D12.3	17	20	19	14	2.80E-06	2.65E-06	2.00E-06	2.25E-06
F59D12.4	16	23	15	14	2.80E-06	2.65E-06	1.82E-06	2.25E-06
F59D12.5	18	19	28	26	2.80E-06	2.65E-06	1.82E-06	2.25E-06
F59D6.1	2	2	10	3	2.80E-06	2.65E-06	1.82E-06	2.25E-06
F59D6.2	5	3	8	4	4.26E-06	4.23E-06	4.30E-06	4.93E-06
F59D6.3	64	25	77	27	2.80E-06	2.65E-06	2.04E-06	2.25E-06
F59D6.4	9	6	9	5	2.80E-06	2.65E-06	1.82E-06	2.25E-06

F59D6.5	0	9	5	3	5.04E-06	2.65E-06	3.94E-06	2.25E-06
F59D6.6	12	8	19	8	2.80E-06	2.65E-06	1.82E-06	2.25E-06
F59D6.7	10	9	10	14	2.80E-06	2.65E-06	1.82E-06	2.25E-06
F59D8.1	157508	109462	312715	479047	2.80E-06	2.65E-06	1.82E-06	2.25E-06
F59D8.2	160241	114551	321987	495899	2.80E-06	2.65E-06	1.82E-06	2.25E-06
F59E10.1	447	359	566	658	3.63E-03	2.38E-03	4.69E-03	8.87E-03
F59E10.3	487	621	366	385	3.71E-03	2.50E-03	4.85E-03	9.21E-03
F59E11.1	4	3	4	7	2.72E-05	2.07E-05	2.24E-05	3.22E-05
F59E11.10	9	12	9	7	8.28E-05	9.97E-05	4.05E-05	5.26E-05
F59E11.11	24	46	15	18	2.80E-06	2.65E-06	1.82E-06	2.25E-06
F59E11.12a	1	5	0	4	2.80E-06	2.65E-06	1.82E-06	2.25E-06
F59E11.12b.1	13	23	8	17	2.80E-06	3.84E-06	1.82E-06	2.25E-06
F59E11.12b.2	5	11	4	7	2.80E-06	2.72E-06	1.82E-06	2.25E-06
F59E11.13	6	5	11	7	2.80E-06	2.65E-06	1.82E-06	2.25E-06
F59E11.14	4	4	4	0	2.80E-06	2.65E-06	1.82E-06	2.25E-06
F59E11.15	1	2	5	2	2.80E-06	2.65E-06	1.82E-06	2.25E-06
F59E11.16	2	4	3	3	2.80E-06	2.65E-06	1.82E-06	2.25E-06
F59E11.2	6	7	5	7	2.80E-06	2.65E-06	1.82E-06	2.25E-06
F59E11.3	8	9	18	6	2.80E-06	2.65E-06	1.82E-06	2.25E-06
F59E11.5	91	138	96	53	2.80E-06	2.65E-06	1.82E-06	2.25E-06
F59E11.6	1	10	5	3	2.80E-06	2.65E-06	1.82E-06	2.25E-06
F59E11.7a	14	27	11	11	1.29E-05	1.85E-05	8.87E-06	6.05E-06
F59E11.7b	13	27	8	9	2.80E-06	2.65E-06	1.82E-06	2.25E-06
F59E11.8	3	7	4	6	2.80E-06	4.29E-06	1.82E-06	2.25E-06
F59E12.1	831	785	1209	1613	2.80E-06	4.73E-06	1.82E-06	2.25E-06
F59E12.10	80	92	116	129	2.80E-06	2.65E-06	1.82E-06	2.25E-06
F59E12.11	550	431	493	736	3.59E-05	3.20E-05	3.40E-05	5.59E-05
F59E12.12	418	502	124	81	1.57E-05	1.71E-05	1.48E-05	2.04E-05
F59E12.13.1	348	328	376	441	5.19E-05	3.85E-05	3.03E-05	5.58E-05
F59E12.2.1	265	316	328	531	4.85E-05	5.50E-05	9.37E-06	7.56E-06
F59E12.2.2	267	326	328	527	2.91E-05	2.59E-05	2.05E-05	2.97E-05
F59E12.3	5	7	2	1	1.29E-05	1.46E-05	1.04E-05	2.08E-05
F59E12.4a	1245	1202	1731	2435	1.28E-05	1.47E-05	1.02E-05	2.02E-05
F59E12.4b	1208	1169	1595	2341	2.80E-06	2.65E-06	1.82E-06	2.25E-06
F59E12.5b	1218	1177	1613	2374	8.44E-05	7.70E-05	7.64E-05	1.33E-04
F59E12.6a	175	244	138	315	8.38E-05	7.66E-05	7.20E-05	1.30E-04
F59E12.6b	109	144	68	191	8.13E-05	7.42E-05	7.01E-05	1.27E-04
F59E12.8	5	8	4	5	7.53E-06	9.92E-06	3.86E-06	1.09E-05
F59E12.9	1135	1009	1102	1969	8.06E-06	1.01E-05	3.26E-06	1.13E-05
F59F3.1	260	309	319	491	2.80E-06	2.65E-06	1.82E-06	2.25E-06
F59F3.2	17	17	33	10	2.43E-05	2.04E-05	1.54E-05	3.39E-05
F59F3.5	43	30	37	28	7.87E-06	8.83E-06	6.27E-06	1.19E-05
F59F3.6	7	18	7	5	2.80E-06	2.65E-06	1.82E-06	2.25E-06
F59F4.3	76	97	48	85	2.80E-06	2.65E-06	1.82E-06	2.25E-06
F59F4.4	174	220	312	304	2.80E-06	2.99E-06	1.82E-06	2.25E-06
F59F5.1	25	57	41	59	5.01E-06	6.06E-06	2.06E-06	4.50E-06
F59F5.2	11	19	18	15	2.06E-05	2.46E-05	2.40E-05	2.89E-05
F59F5.3	19	37	17	15	2.80E-06	4.81E-06	2.39E-06	4.23E-06
F59F5.4	0	0	1	2	2.80E-06	2.88E-06	1.88E-06	2.25E-06
F59F5.5	1	9	9	3	2.80E-06	2.65E-06	1.82E-06	2.25E-06
F59F5.6	94	122	105	166	2.80E-06	2.65E-06	1.82E-06	2.25E-06
F59F5.7	50	57	53	38	2.80E-06	2.65E-06	1.82E-06	2.25E-06
F59F5.8	28	65	16	24	2.80E-06	3.39E-06	2.00E-06	3.91E-06
F59G1.1a	639	866	879	1383	2.80E-06	2.65E-06	1.82E-06	2.25E-06
F59G1.1b.1	806	1049	1146	1622	2.80E-06	5.77E-06	1.82E-06	2.25E-06
F59G1.1b.2	671	891	913	1422	4.96E-05	6.35E-05	4.44E-05	8.62E-05
F59G1.1c.1	755	957	1008	1523	5.08E-05	6.25E-05	4.71E-05	8.22E-05
F59G1.1c.2	557	730	835	1169	5.15E-05	6.46E-05	4.56E-05	8.76E-05
F59G1.1d.1	639	865	879	1383	4.70E-05	5.62E-05	4.08E-05	7.61E-05

F59G1.1d.2	671	891	913	1422	5.00E-05	6.19E-05	4.88E-05	8.43E-05
F59G1.1d.3	704	938	1028	1463	5.29E-05	6.77E-05	4.74E-05	9.20E-05
F59G1.1d.4	639	865	879	1383	5.15E-05	6.46E-05	4.56E-05	8.76E-05
F59G1.1d.5	660	885	904	1411	5.19E-05	6.53E-05	4.93E-05	8.66E-05
F59G1.2	11	20	11	10	5.31E-05	6.78E-05	4.75E-05	9.22E-05
F59G1.3	1273	1120	1358	1983	5.06E-05	6.41E-05	4.51E-05	8.69E-05
F59G1.4	55	39	83	51	2.80E-06	2.65E-06	1.82E-06	2.25E-06
F59G1.5.1	903	804	1300	1931	4.80E-05	3.99E-05	3.34E-05	6.01E-05
F59G1.5.2	648	561	898	1330	5.80E-06	3.89E-06	5.70E-06	4.32E-06
F59G1.7.1	72	86	82	77	3.69E-05	3.10E-05	3.45E-05	6.34E-05
F59G1.7.2	64	76	51	68	3.50E-05	2.86E-05	3.16E-05	5.78E-05
F59H5.1	210	218	322	506	1.34E-05	1.51E-05	9.91E-06	1.15E-05
F59H5.3	15	34	24	34	1.22E-05	1.37E-05	6.34E-06	1.04E-05
F59H6.1	45	36	62	69	9.41E-06	9.23E-06	9.38E-06	1.82E-05
F59H6.10	33	45	32	57	2.80E-06	3.89E-06	1.90E-06	3.31E-06
F59H6.11	81	109	165	227	4.76E-06	3.60E-06	4.28E-06	5.87E-06
F59H6.12	43	58	48	34	4.00E-06	5.16E-06	2.53E-06	5.58E-06
F59H6.2	9	13	8	9	9.88E-06	1.26E-05	1.31E-05	2.22E-05
F59H6.3	9	9	6	17	7.67E-06	9.79E-06	5.58E-06	4.88E-06
F59H6.4	16	23	54	16	2.80E-06	2.65E-06	1.82E-06	2.25E-06
F59H6.5	15	32	24	14	4.68E-06	4.42E-06	2.02E-06	7.08E-06
F59H6.6	2	4	8	4	2.80E-06	2.72E-06	4.41E-06	2.25E-06
F59H6.7	5	3	3	1	2.80E-06	2.65E-06	1.82E-06	2.25E-06
F59H6.8	13	21	20	19	2.80E-06	2.65E-06	1.82E-06	2.25E-06
F59H6.9	44	68	57	80	2.80E-06	2.65E-06	1.82E-06	2.25E-06
H01A20.1	56	131	65	145	2.80E-06	2.72E-06	1.82E-06	2.25E-06
H01A20.2	51	58	33	74	4.96E-06	7.22E-06	4.17E-06	7.24E-06
H01G02.1	88	142	92	110	3.61E-06	7.96E-06	2.73E-06	7.51E-06
H01G02.2	5	10	8	4	7.31E-06	7.86E-06	3.08E-06	8.52E-06
H01G02.3a	39	63	38	59	1.06E-05	1.62E-05	7.22E-06	1.06E-05
H01G02.3b	10	13	9	20	2.80E-06	2.65E-06	1.82E-06	2.25E-06
H01M10.1	32	42	35	45	2.80E-06	3.84E-06	1.82E-06	3.06E-06
H01M10.2	4	5	3	5	2.80E-06	2.65E-06	1.82E-06	2.97E-06
H02F09.2	2	1	5	3	3.47E-06	4.29E-06	2.46E-06	3.91E-06
H02F09.3	17	31	35	9	2.80E-06	2.65E-06	1.82E-06	2.25E-06
H02I12.1	16937	12384	27165	32281	2.80E-06	2.65E-06	1.82E-06	2.25E-06
H02I12.2	6	2	4	3	2.80E-06	2.65E-06	1.82E-06	2.25E-06
H02I12.3	19	22	7	10	4.53E-04	3.13E-04	4.72E-04	6.93E-04
H02I12.4	8	10	14	19	2.80E-06	2.65E-06	1.82E-06	2.25E-06
H02I12.5	569	532	1105	814	2.80E-06	2.65E-06	1.82E-06	2.25E-06
H02I12.6	80	77	104	85	2.80E-06	2.65E-06	1.82E-06	2.25E-06
H02I12.7	33	41	101	57	5.12E-05	4.53E-05	6.47E-05	5.89E-05
H02I12.8	430	513	754	843	2.41E-05	2.19E-05	2.04E-05	2.06E-05
H02K04.1	4	10	4	4	9.63E-06	1.13E-05	1.92E-05	1.34E-05
H02K04.2	0	0	3	2	3.14E-05	3.53E-05	3.58E-05	4.94E-05
H03A11.1	213	233	162	286	2.80E-06	2.65E-06	1.82E-06	2.25E-06
H03A11.2	3156	4353	1863	2599	2.80E-06	2.65E-06	1.82E-06	2.25E-06
H03E18.1	371	536	156	133	1.11E-05	1.15E-05	5.48E-06	1.20E-05
H03E18.2	5	7	2	6	8.81E-05	1.15E-04	3.39E-05	5.83E-05
H03G16.1	2	4	2	3	1.12E-05	1.53E-05	3.06E-06	3.24E-06
H03G16.2	2	2	3	1	2.80E-06	2.65E-06	1.82E-06	2.25E-06
H03G16.3	7	8	44	8	2.80E-06	2.65E-06	1.82E-06	2.25E-06
H03G16.4	5	4	6	3	2.80E-06	2.65E-06	1.82E-06	2.25E-06
H03G16.5	3	9	16	8	2.80E-06	2.65E-06	1.82E-06	2.25E-06
H03G16.6	2	1	4	0	2.80E-06	2.65E-06	1.82E-06	2.25E-06
H04D03.1	203	167	358	201	2.80E-06	2.65E-06	1.82E-06	2.25E-06
H04D03.2a	170	153	343	313	2.80E-06	2.65E-06	1.82E-06	2.25E-06
H04D03.2b	180	160	413	331	2.42E-05	1.88E-05	2.78E-05	1.92E-05
H04D03.3	177	197	292	351	8.48E-06	7.22E-06	1.11E-05	1.25E-05

H04D03.4	12	18	17	18	8.96E-06	7.51E-06	1.34E-05	1.32E-05
H04J21.1	23	43	17	17	1.61E-05	1.70E-05	1.73E-05	2.57E-05
H04J21.2	5	1	2	4	2.80E-06	2.65E-06	1.82E-06	2.25E-06
H04J21.3a.1	396	371	529	803	2.80E-06	2.65E-06	1.82E-06	2.25E-06
H04J21.3a.2	382	332	481	754	2.80E-06	2.65E-06	1.82E-06	2.25E-06
H04J21.3b	422	380	535	795	1.58E-05	1.39E-05	1.37E-05	2.57E-05
H04J21.3c	182	139	207	310	1.60E-05	1.31E-05	1.31E-05	2.53E-05
H04M03.1	39	76	21	22	1.32E-05	1.12E-05	1.09E-05	1.99E-05
H04M03.10	20	5	8	7	1.71E-05	1.23E-05	1.26E-05	2.33E-05
H04M03.11	3	4	2	3	2.80E-06	4.07E-06	1.82E-06	2.25E-06
H04M03.12	6	16	12	11	2.80E-06	2.65E-06	1.82E-06	2.25E-06
H04M03.2	21	24	50	9	2.80E-06	2.65E-06	1.82E-06	2.25E-06
H04M03.3	90	69	132	145	2.80E-06	2.65E-06	1.82E-06	2.25E-06
H04M03.5	3	1	4	3	9.21E-06	9.95E-06	1.43E-05	3.17E-06
H04M03.6	2	5	2	4	9.10E-06	6.61E-06	8.69E-06	1.18E-05
H04M03.7	4	4	4	1	2.80E-06	2.65E-06	1.82E-06	2.25E-06
H04M03.8	4	8	5	7	2.80E-06	2.65E-06	1.82E-06	2.25E-06
H04M03.9	22	7	4	5	2.80E-06	2.65E-06	1.82E-06	2.25E-06
H05B21.1	0	5	3	1	2.80E-06	2.65E-06	1.82E-06	2.25E-06
H05B21.2	3	9	7	3	2.80E-06	2.65E-06	1.82E-06	2.25E-06
H05B21.3	12	7	13	5	2.80E-06	2.65E-06	1.82E-06	2.25E-06
H05B21.4	5	7	18	4	2.80E-06	2.65E-06	1.82E-06	2.25E-06
H05C05.1a	292	230	550	739	2.80E-06	2.65E-06	1.82E-06	2.25E-06
H05C05.1b	59	58	174	212	2.80E-06	2.65E-06	1.82E-06	2.25E-06
H05C05.2a	276	283	338	437	1.25E-05	9.31E-06	1.53E-05	2.55E-05
H05C05.2b	372	382	438	590	7.73E-06	7.19E-06	1.49E-05	2.23E-05
H05C05.3	5	8	14	6	2.35E-05	2.27E-05	1.87E-05	2.98E-05
H05G16.1	90	70	115	116	2.36E-05	2.29E-05	1.81E-05	3.01E-05
H05L03.3.1	130	209	219	96	2.80E-06	2.65E-06	1.82E-06	2.25E-06
H05L03.3.2	110	186	183	93	2.80E-06	2.65E-06	1.99E-06	2.47E-06
H05L14.1	67	140	61	46	2.33E-05	3.53E-05	2.55E-05	1.38E-05
H05L14.2	542	369	1053	1275	2.05E-05	3.27E-05	2.22E-05	1.39E-05
H06A10.1	45	67	37	39	3.00E-06	5.92E-06	1.82E-06	2.25E-06
H06A10.2	64	75	40	48	8.79E-06	5.66E-06	1.11E-05	1.66E-05
H06H21.1	3	8	3	7	5.74E-06	8.09E-06	3.08E-06	4.00E-06
H06H21.10a	76	125	61	93	7.81E-06	8.65E-06	3.17E-06	4.70E-06
H06H21.10b	74	125	71	87	2.80E-06	2.65E-06	1.82E-06	2.25E-06
H06H21.11	178	148	267	208	2.80E-06	3.60E-06	1.82E-06	2.27E-06
H06H21.2	6	10	7	3	2.80E-06	3.94E-06	1.82E-06	2.34E-06
H06H21.6.1	776	825	955	1429	3.67E-05	2.88E-05	3.58E-05	3.45E-05
H06H21.6.2	778	825	943	1412	2.80E-06	2.65E-06	1.82E-06	2.25E-06
H06H21.6.3	789	821	964	1419	4.78E-05	4.80E-05	3.83E-05	7.08E-05
H06H21.6.4	700	732	845	1267	5.33E-05	5.34E-05	4.20E-05	7.77E-05
H06H21.6.5	687	736	836	1277	4.89E-05	4.81E-05	3.89E-05	7.06E-05
H06H21.8a	152	262	159	160	5.33E-05	5.26E-05	4.18E-05	7.74E-05
H06H21.8b	151	266	139	151	5.21E-05	5.27E-05	4.13E-05	7.78E-05
H06H21.9	20	39	12	16	1.36E-05	2.21E-05	9.24E-06	1.15E-05
H06I04.1a.1	68	109	98	112	1.45E-05	2.41E-05	8.67E-06	1.16E-05
H06I04.1a.2	63	94	87	100	2.80E-06	3.36E-06	1.82E-06	2.25E-06
H06I04.1b	95	132	148	142	4.90E-06	7.43E-06	4.61E-06	6.50E-06
H06I04.1c.1	55	82	80	94	5.07E-06	7.14E-06	4.56E-06	6.45E-06
H06I04.1c.2	40	70	66	80	6.10E-06	8.01E-06	6.20E-06	7.33E-06
H06I04.2	53	31	30	69	4.93E-06	6.93E-06	4.66E-06	6.77E-06
H06I04.3a	1056	926	411	1079	4.14E-06	6.85E-06	4.45E-06	6.66E-06
H06I04.3b	867	768	317	883	6.05E-06	3.36E-06	2.22E-06	6.34E-06
H06I04.4a.1	5675	4768	10661	5411	4.32E-05	3.58E-05	1.09E-05	3.54E-05
H06I04.4a.2	5233	4464	8021	4884	4.45E-05	3.73E-05	1.06E-05	3.64E-05
H06I04.5	42	63	29	28	1.12E-03	8.87E-04	1.37E-03	8.56E-04
H06I04.6	13	24	13	29	1.04E-03	8.34E-04	1.03E-03	7.76E-04

H06I04.7	1	4	4	1	2.80E-06	2.65E-06	1.82E-06	2.25E-06
H06O01.1.1	5090	5831	4988	5407	2.80E-06	2.65E-06	1.82E-06	2.70E-06
H06O01.1.2	4908	5641	4758	5310	2.80E-06	2.65E-06	1.82E-06	2.25E-06
H06O01.1.3	4903	5635	4756	5307	3.34E-04	3.61E-04	2.13E-04	2.85E-04
H06O01.2	571	591	451	876	3.74E-04	4.06E-04	2.36E-04	3.25E-04
H06O01.3	205	390	173	243	3.72E-04	4.04E-04	2.35E-04	3.23E-04
H06O01.4	7	12	11	8	1.35E-05	1.32E-05	6.92E-06	1.66E-05
H08J11.2	58	58	38	78	1.50E-05	2.69E-05	8.22E-06	1.43E-05
H08J19.1	1	1	1	1	2.80E-06	2.65E-06	1.82E-06	2.25E-06
H08M01.1	16	21	13	8	6.78E-06	6.40E-06	2.88E-06	7.31E-06
H08M01.2a	72	92	98	116	2.80E-06	2.65E-06	1.82E-06	2.25E-06
H08M01.2b	80	98	103	120	2.80E-06	2.65E-06	1.82E-06	2.25E-06
H09F14.1	3	6	9	8	2.80E-06	2.65E-06	1.82E-06	2.63E-06
H09G03.1	9	15	14	12	2.80E-06	2.65E-06	1.82E-06	2.63E-06
H09G03.2a	49	85	71	76	2.80E-06	2.65E-06	1.82E-06	2.25E-06
H09G03.2b.1	38	76	53	64	2.80E-06	2.65E-06	1.82E-06	2.25E-06
H09G03.2b.2	35	75	53	64	2.80E-06	3.44E-06	1.99E-06	2.63E-06
H09G03.2c	34	68	50	56	2.80E-06	3.12E-06	1.82E-06	2.25E-06
H09I01.1	5	2	1	0	2.80E-06	3.09E-06	1.82E-06	2.25E-06
H09I01.2	1	0	0	0	2.80E-06	3.02E-06	1.82E-06	2.25E-06
H10D12.2	3	8	6	7	2.80E-06	2.65E-06	1.82E-06	2.25E-06
H10D18.1	3	1	1	3	2.80E-06	2.65E-06	1.82E-06	2.25E-06
H10D18.2	0	3	2	2	2.80E-06	2.65E-06	1.82E-06	2.25E-06
H10D18.3	10	6	4	7	2.80E-06	2.65E-06	1.82E-06	2.25E-06
H10D18.4	0	1	1	1	2.80E-06	2.65E-06	1.82E-06	2.25E-06
H10D18.5	11	21	11	19	2.80E-06	2.65E-06	1.82E-06	2.25E-06
H10D18.6	3	5	2	5	2.80E-06	2.65E-06	1.82E-06	2.25E-06
H10E21.1	17	33	24	23	2.80E-06	2.65E-06	1.82E-06	2.25E-06
H10E21.2	8	8	5	3	2.80E-06	2.65E-06	1.82E-06	2.25E-06
H10E21.3b	29	70	30	51	3.05E-06	5.61E-06	2.81E-06	3.33E-06
H10E21.4	97	200	45	48	2.80E-06	2.65E-06	1.82E-06	2.25E-06
H10E21.5	46	60	41	63	2.80E-06	4.89E-06	1.82E-06	3.04E-06
H11E01.1	2	1	3	3	1.38E-05	2.69E-05	4.17E-06	5.49E-06
H11E01.2	9	20	33	35	3.11E-06	3.84E-06	1.82E-06	3.42E-06
H11E01.3	172	203	141	186	2.80E-06	2.65E-06	1.82E-06	2.25E-06
H11E01.t1	0	0	0	3	2.80E-06	2.65E-06	1.82E-06	2.25E-06
H11L12.1	295	232	661	445	4.34E-06	4.84E-06	2.31E-06	3.78E-06
H12C20.2a	509	533	740	1121	2.80E-06	2.65E-06	1.82E-06	3.80E-06
H12C20.2b	147	140	186	310	4.35E-05	3.23E-05	6.35E-05	5.27E-05
H12C20.3	170	613	136	315	2.17E-05	2.14E-05	2.05E-05	3.83E-05
H12C20.4	12	10	15	10	1.64E-05	1.48E-05	1.35E-05	2.78E-05
H12C20.5	4	5	9	7	1.43E-05	4.88E-05	7.45E-06	2.13E-05
H12C20.6a	37	98	35	70	2.80E-06	2.65E-06	1.82E-06	2.25E-06
H12D21.1	111	285	250	162	2.80E-06	2.65E-06	1.82E-06	2.25E-06
H12D21.10	43	68	61	59	3.33E-06	8.33E-06	2.06E-06	5.06E-06
H12D21.11	5	3	5	6	1.18E-04	2.87E-04	1.74E-04	1.39E-04
H12D21.12	17	46	15	5	2.83E-06	4.23E-06	2.62E-06	3.13E-06
H12D21.13	18	56	16	5	2.80E-06	2.65E-06	1.82E-06	2.25E-06
H12D21.14	1	20	2	1	1.81E-05	4.63E-05	1.04E-05	4.27E-06
H12D21.15	1	20	2	1	1.92E-05	5.64E-05	1.11E-05	4.27E-06
H12D21.2	8	12	6	5	2.80E-06	2.02E-05	1.82E-06	2.25E-06
H12D21.3	8	14	5	2	2.80E-06	2.02E-05	1.82E-06	2.25E-06
H12D21.4	3	3	4	3	2.80E-06	2.65E-06	1.82E-06	2.25E-06
H12D21.5	13	24	16	4	3.56E-06	5.87E-06	1.82E-06	2.25E-06
H12D21.6	4	3	6	0	2.80E-06	2.65E-06	1.82E-06	2.25E-06
H12D21.9	4	22	6	16	2.86E-06	4.97E-06	2.28E-06	2.25E-06
H12I13.1	6	12	9	10	2.80E-06	2.65E-06	1.82E-06	2.25E-06
H12I13.2	2	4	3	2	2.80E-06	2.86E-06	1.82E-06	2.25E-06
H12I13.3	10	5	8	6	2.80E-06	2.65E-06	1.82E-06	2.25E-06

H12I13.4	415	366	485	635	2.80E-06	2.65E-06	1.82E-06	2.25E-06
H12I13.5	3	11	1	0	2.80E-06	2.65E-06	1.82E-06	2.25E-06
H12I13.6	7	14	4	0	2.42E-05	2.02E-05	1.85E-05	2.98E-05
H12I19.1	7	5	12	7	2.80E-06	4.42E-06	1.82E-06	2.25E-06
H12I19.2	3	6	13	1	3.02E-06	5.71E-06	1.82E-06	2.25E-06
H12I19.3	8	5	12	9	2.80E-06	2.65E-06	1.82E-06	2.25E-06
H12I19.4	5	9	11	7	2.80E-06	2.65E-06	1.82E-06	2.25E-06
H12I19.5a	14	16	13	9	2.80E-06	2.65E-06	1.82E-06	2.25E-06
H12I19.5b	13	12	13	7	2.80E-06	2.65E-06	1.82E-06	2.25E-06
H12I19.7	4	4	6	4	2.80E-06	2.65E-06	1.82E-06	2.25E-06
H12I19.8	2	2	2	1	2.80E-06	2.65E-06	1.82E-06	2.25E-06
H13N06.2	17	36	20	27	2.80E-06	2.65E-06	1.82E-06	2.25E-06
H13N06.3a	191	329	240	320	2.80E-06	2.65E-06	1.82E-06	2.25E-06
H13N06.3b	123	210	133	221	2.80E-06	2.65E-06	1.82E-06	2.25E-06
H13N06.4a	98	189	79	123	1.11E-05	1.80E-05	9.06E-06	1.49E-05
H13N06.4b	83	170	68	111	1.01E-05	1.63E-05	7.12E-06	1.46E-05
H13N06.5	419	582	373	387	6.24E-06	1.13E-05	3.26E-06	6.27E-06
H13N06.7	2	1	1	1	1.55E-05	2.99E-05	8.25E-06	1.66E-05
H14A12.2a	2039	2123	2030	2472	3.00E-05	3.93E-05	1.73E-05	2.22E-05
H14A12.2b.1	1776	1888	1748	2187	2.80E-06	2.65E-06	1.82E-06	2.25E-06
H14A12.2b.2	1772	1880	1743	2183	1.37E-04	1.35E-04	8.88E-05	1.33E-04
H14A12.2b.3	1816	1924	1783	2243	1.30E-04	1.31E-04	8.33E-05	1.29E-04
H14A12.2b.4	1669	1786	1647	2088	1.34E-04	1.34E-04	8.57E-05	1.33E-04
H14A12.2b.5	1744	1864	1713	2169	1.37E-04	1.37E-04	8.77E-05	1.36E-04
H14A12.3	455	349	697	689	1.41E-04	1.43E-04	9.07E-05	1.42E-04
H14A12.4	7	12	2	3	1.39E-04	1.40E-04	8.86E-05	1.38E-04
H14A12.5	14	14	15	11	4.49E-05	3.25E-05	4.47E-05	5.46E-05
H14A12.6	0	2	0	1	2.80E-06	2.65E-06	1.82E-06	2.25E-06
H14A12.7	3	2	3	1	3.92E-06	3.70E-06	2.73E-06	2.47E-06
H14E04.1	18	23	7	8	2.80E-06	2.65E-06	1.82E-06	2.25E-06
H14E04.2a	353	309	398	579	2.80E-06	2.65E-06	1.82E-06	2.25E-06
H14E04.2b	405	340	438	657	2.80E-06	2.65E-06	1.82E-06	2.25E-06
H14E04.2c	334	305	375	558	2.28E-05	1.89E-05	1.67E-05	3.00E-05
H14E04.2d	353	309	398	579	2.27E-05	1.80E-05	1.60E-05	2.96E-05
H14E04.3	4	13	10	5	2.13E-05	1.84E-05	1.56E-05	2.86E-05
H14E04.4	13	8	37	14	2.28E-05	1.89E-05	1.67E-05	3.00E-05
H14E04.5	137	143	218	212	2.80E-06	2.65E-06	1.82E-06	2.25E-06
H14N18.1a	392	1007	196	313	3.02E-06	2.65E-06	5.58E-06	2.61E-06
H14N18.1b.1	304	808	169	255	1.54E-05	1.52E-05	1.59E-05	1.91E-05
H14N18.1b.2	288	787	160	249	2.21E-05	5.36E-05	7.18E-06	1.41E-05
H14N18.1c	304	808	169	255	2.48E-05	6.22E-05	8.96E-06	1.67E-05
H14N18.2	1	6	3	3	2.36E-05	6.10E-05	8.55E-06	1.64E-05
H14N18.3	238	541	346	325	2.48E-05	6.22E-05	8.96E-06	1.67E-05
H14N18.4a	69	119	84	132	2.80E-06	2.65E-06	1.82E-06	2.25E-06
H14N18.4b	75	127	90	138	5.18E-05	1.11E-04	4.90E-05	5.68E-05
H15N14.1c	724	637	837	1305	4.06E-06	6.61E-06	3.23E-06	6.25E-06
H15N14.1d	669	604	746	1229	3.53E-06	5.63E-06	2.75E-06	5.22E-06
H15N14.1e	671	602	754	1236	2.68E-05	2.23E-05	2.02E-05	3.88E-05
H15N14.1f	696	617	785	1278	2.63E-05	2.25E-05	1.91E-05	3.88E-05
H15N14.1g	690	614	780	1269	2.69E-05	2.28E-05	1.97E-05	3.98E-05
H16D19.1	15	6	26	3	2.50E-05	2.09E-05	1.83E-05	3.69E-05
H16D19.2	3	5	4	2	2.63E-05	2.22E-05	1.94E-05	3.89E-05
H16D19.3	4	13	10	15	2.80E-06	2.65E-06	1.82E-06	2.25E-06
H16D19.4	17	16	29	18	2.80E-06	2.65E-06	1.82E-06	2.25E-06
H16O14.1	347	355	281	415	2.80E-06	2.65E-06	1.82E-06	2.25E-06
H16O14.2	10	8	11	7	2.80E-06	2.65E-06	1.82E-06	2.25E-06
H17B01.1a	457	544	583	777	1.32E-05	1.28E-05	6.98E-06	1.27E-05
H17B01.1b	358	430	459	661	4.12E-06	3.09E-06	2.93E-06	2.32E-06
H17B01.2	161	221	167	263	3.17E-05	3.56E-05	2.63E-05	4.32E-05

H17B01.3	5	6	7	2	2.62E-05	2.97E-05	2.18E-05	3.88E-05
H17B01.4b.1	318	289	340	515	1.13E-05	1.47E-05	7.63E-06	1.48E-05
H17B01.4b.2	293	274	304	502	2.80E-06	2.65E-06	1.82E-06	2.25E-06
H17B01.5	4	7	2	1	2.98E-05	2.56E-05	2.08E-05	3.88E-05
H17B01.t1	0	0	4	2	2.75E-05	2.43E-05	1.86E-05	3.79E-05
H18N23.2a	80	116	83	102	2.80E-06	2.65E-06	1.82E-06	2.25E-06
H18N23.2b	261	326	236	324	2.80E-06	2.65E-06	3.99E-06	2.47E-06
H18N23.2c	80	116	83	102	8.54E-06	1.17E-05	5.78E-06	8.75E-06
H19J13.1	10	10	13	7	1.32E-05	1.56E-05	7.80E-06	1.32E-05
H19J13.2	3	3	3	0	8.37E-06	1.15E-05	5.65E-06	8.57E-06
H19M22.2a	1505	1870	971	2019	2.80E-06	2.65E-06	1.82E-06	2.25E-06
H19M22.2b	1647	2000	1035	2146	2.80E-06	2.65E-06	1.82E-06	2.25E-06
H19M22.2c	220	275	148	284	1.27E-05	1.49E-05	5.34E-06	1.37E-05
H19M22.2d	125	165	147	178	1.34E-05	1.54E-05	5.48E-06	1.40E-05
H19M22.3a	68	68	46	29	1.98E-05	2.34E-05	8.67E-06	2.05E-05
H19M22.3b	11	16	10	5	4.84E-06	6.03E-06	3.72E-06	5.53E-06
H19M22.3c	29	40	21	10	3.28E-06	3.09E-06	1.82E-06	2.25E-06
H19M22.4	10	11	22	12	2.80E-06	2.65E-06	1.82E-06	2.25E-06
H19N07.1.1	1069	1342	1093	1553	2.80E-06	3.09E-06	1.82E-06	2.25E-06
H19N07.1.2	884	1064	955	1369	2.80E-06	2.65E-06	2.24E-06	2.25E-06
H19N07.2a	1913	1776	2110	3271	5.94E-05	7.04E-05	3.95E-05	6.93E-05
H19N07.2b	2204	2061	2454	3818	6.11E-05	6.95E-05	4.30E-05	7.60E-05
H19N07.2c	2201	2044	2452	3826	6.06E-05	5.32E-05	4.35E-05	8.33E-05
H19N07.3.1	95	166	72	112	5.91E-05	5.22E-05	4.28E-05	8.22E-05
H19N07.3.2	87	153	66	100	5.96E-05	5.23E-05	4.32E-05	8.32E-05
H19N07.4.1	331	560	455	658	1.49E-05	2.46E-05	7.34E-06	1.41E-05
H19N07.4.2	272	459	361	580	1.55E-05	2.57E-05	7.62E-06	1.43E-05
H20E11.1a	77	86	76	83	1.99E-05	3.18E-05	1.78E-05	3.18E-05
H20E11.1b	20	36	20	19	2.01E-05	3.20E-05	1.74E-05	3.44E-05
H20E11.2	37	24	19	20	5.21E-06	5.50E-06	3.35E-06	4.52E-06
H20E11.3a	55	46	28	30	2.80E-06	4.15E-06	1.82E-06	2.25E-06
H20E11.3b	67	49	34	31	3.81E-06	2.65E-06	1.82E-06	2.25E-06
H20J04.1	38	55	25	11	5.85E-06	4.63E-06	1.93E-06	2.56E-06
H20J04.2	701	699	933	1476	6.92E-06	4.76E-06	2.28E-06	2.56E-06
H20J04.3	106	106	188	228	2.80E-06	2.65E-06	1.82E-06	2.25E-06
H20J04.4a	62	63	77	168	1.67E-05	1.57E-05	1.44E-05	2.82E-05
H20J04.4b	83	77	113	201	1.27E-05	1.20E-05	1.46E-05	2.18E-05
H20J04.6	14	31	74	36	4.70E-06	4.52E-06	3.81E-06	1.02E-05
H20J04.7	5	10	7	10	4.87E-06	4.26E-06	4.30E-06	9.47E-06
H20J04.8.1	241	334	301	345	2.80E-06	4.21E-06	6.92E-06	4.16E-06
H20J04.8.2	194	274	201	257	2.80E-06	2.65E-06	1.82E-06	2.25E-06
H20J04.9	63	76	80	78	2.72E-05	3.56E-05	2.21E-05	3.13E-05
H20J18.1a	303	394	299	477	2.83E-05	3.77E-05	1.91E-05	3.01E-05
H20J18.1b	202	282	217	326	8.74E-06	9.97E-06	7.23E-06	8.70E-06
H21P03.1	953	739	902	1060	9.24E-06	1.13E-05	5.92E-06	1.17E-05
H21P03.2	426	319	575	734	8.01E-06	1.06E-05	5.59E-06	1.04E-05
H21P03.3a	452	542	648	980	1.39E-04	1.02E-04	8.55E-05	1.24E-04
H21P03.3b	412	463	569	846	3.78E-05	2.67E-05	3.32E-05	5.23E-05
H22D07.1	9	21	13	11	2.94E-05	3.33E-05	2.74E-05	5.12E-05
H22D14.1	5	4	5	0	3.13E-05	3.32E-05	2.82E-05	5.17E-05
H22K11.1	4662	6562	6010	7426	2.80E-06	2.65E-06	1.82E-06	2.25E-06
H22K11.2	123	173	158	243	2.80E-06	2.65E-06	1.82E-06	2.25E-06
H22K11.3	5	8	2	0	3.84E-04	5.11E-04	3.22E-04	4.92E-04
H22K11.4a	37	67	29	29	7.08E-06	9.44E-06	5.94E-06	1.13E-05
H22K11.4b	40	73	30	29	2.80E-06	2.65E-06	1.82E-06	2.25E-06
H23L24.1	2	4	9	4	2.97E-06	5.08E-06	1.82E-06	2.25E-06
H23L24.2	10	24	5	12	3.19E-06	5.53E-06	1.82E-06	2.25E-06
H23L24.3a	13	11	14	3	2.80E-06	2.65E-06	1.82E-06	2.25E-06
H23L24.3b	18	15	20	6	2.80E-06	2.65E-06	1.82E-06	2.25E-06

H23L24.4	10	9	3	4	2.80E-06	2.65E-06	1.82E-06	2.25E-06
H23L24.5	25	35	14	56	2.80E-06	2.65E-06	1.82E-06	2.25E-06
H23N18.1	97	202	90	68	2.80E-06	2.65E-06	1.82E-06	2.25E-06
H23N18.2	11	21	10	7	2.80E-06	2.65E-06	1.82E-06	3.46E-06
H23N18.3	45	153	62	24	6.80E-06	1.34E-05	4.10E-06	3.82E-06
H23N18.4	11	10	17	5	2.80E-06	2.65E-06	1.82E-06	2.25E-06
H23N18.5	193	247	68	26	3.16E-06	1.01E-05	2.82E-06	2.25E-06
H23N18.6	2	3	8	0	2.80E-06	2.65E-06	1.82E-06	2.25E-06
H24D24.1	3	6	12	9	6.61E-05	7.99E-05	1.52E-05	7.15E-06
H24D24.2	8	4	8	5	2.80E-06	2.65E-06	1.82E-06	2.25E-06
H24G06.1a	368	538	252	422	2.80E-06	2.65E-06	1.82E-06	2.25E-06
H24G06.1b	389	581	272	449	2.80E-06	2.65E-06	1.82E-06	2.25E-06
H24G06.1c	140	200	84	151	8.18E-06	1.13E-05	3.64E-06	7.53E-06
H24G06.1d.1	218	328	156	260	9.07E-06	1.28E-05	4.12E-06	8.41E-06
H24G06.1d.2	205	314	152	254	6.86E-06	9.26E-06	2.68E-06	5.96E-06
H24K24.2	6	10	10	9	1.06E-05	1.51E-05	4.96E-06	1.02E-05
H24K24.4	124	146	257	379	9.13E-06	1.32E-05	4.41E-06	9.09E-06
H24K24.5	53	137	28	41	2.80E-06	2.65E-06	1.82E-06	2.25E-06
H24O09.1	6	6	3	1	7.59E-06	8.44E-06	1.02E-05	1.86E-05
H24O09.2	5	5	9	2	3.58E-06	8.76E-06	1.82E-06	2.25E-06
H25K10.1	6	35	30	36	2.80E-06	2.65E-06	1.82E-06	2.25E-06
H25K10.2	1	1	0	1	2.80E-06	2.65E-06	1.82E-06	2.25E-06
H25K10.3	8	3	4	2	2.80E-06	3.49E-06	2.06E-06	3.06E-06
H25K10.4	1	1	1	0	2.80E-06	2.65E-06	1.82E-06	2.25E-06
H25K10.5	1	7	19	7	2.80E-06	2.65E-06	1.82E-06	2.25E-06
H25K10.6	2	9	14	6	2.80E-06	2.65E-06	1.82E-06	2.25E-06
H25K10.7	5	4	3	1	2.80E-06	2.65E-06	1.82E-06	2.25E-06
H25P06.1.1	395	443	450	546	2.80E-06	2.65E-06	1.82E-06	2.25E-06
H25P06.1.2	323	374	329	445	2.80E-06	2.65E-06	1.82E-06	2.25E-06
H25P06.2a	182	212	269	371	2.24E-05	2.37E-05	1.66E-05	2.49E-05
H25P06.2b	224	258	325	441	2.16E-05	2.36E-05	1.43E-05	2.39E-05
H25P06.3	1	0	0	5	1.50E-05	1.65E-05	1.45E-05	2.46E-05
H25P06.4	6	8	11	5	1.58E-05	1.72E-05	1.49E-05	2.49E-05
H25P19.1	124	118	187	218	2.80E-06	2.65E-06	1.82E-06	2.25E-06
H26D21.1	111	141	127	178	2.80E-06	2.65E-06	1.82E-06	2.25E-06
H26D21.2	836	774	1126	1551	1.19E-05	1.07E-05	1.17E-05	1.68E-05
H27A22.1a	278	311	350	443	1.36E-05	1.63E-05	1.01E-05	1.76E-05
H27A22.1b	309	348	399	475	3.57E-05	3.12E-05	3.13E-05	5.32E-05
H27C11.1a	215	309	392	536	2.67E-05	2.82E-05	2.18E-05	3.41E-05
H27C11.1b.1	92	185	173	369	2.55E-05	2.72E-05	2.14E-05	3.15E-05
H27C11.1b.2	85	192	209	395	1.10E-05	1.49E-05	1.30E-05	2.20E-05
H27C11.1b.3	81	176	169	355	6.86E-06	1.30E-05	8.40E-06	2.21E-05
H27C11.1b.4	75	168	160	338	6.44E-06	1.38E-05	1.03E-05	2.41E-05
H27D07.1	7	3	4	2	7.45E-06	1.53E-05	1.01E-05	2.62E-05
H27D07.2	9	10	9	4	7.22E-06	1.53E-05	1.00E-05	2.62E-05
H27D07.3	4	5	6	3	2.80E-06	2.65E-06	1.82E-06	2.25E-06
H27D07.4	2	5	4	0	2.80E-06	2.65E-06	1.82E-06	2.25E-06
H27D07.5	10	6	4	10	2.80E-06	2.65E-06	1.82E-06	2.25E-06
H27D07.6	1	7	6	2	2.80E-06	2.65E-06	1.82E-06	2.25E-06
H27M09.1	248	354	223	372	2.80E-06	2.65E-06	1.82E-06	2.25E-06
H27M09.2	524	541	758	752	2.80E-06	2.65E-06	1.82E-06	2.25E-06
H27M09.3	369	486	252	555	1.42E-05	1.92E-05	8.31E-06	1.71E-05
H27M09.5	14	26	13	22	8.35E-05	8.14E-05	7.86E-05	9.62E-05
H28G03.1a.1	277	367	304	454	2.27E-05	2.83E-05	1.01E-05	2.75E-05
H28G03.1a.2	270	365	283	442	2.80E-06	2.67E-06	1.82E-06	2.25E-06
H28G03.1b	230	331	250	413	2.97E-05	3.71E-05	2.12E-05	3.90E-05
H28G03.1c.1	637	884	587	1079	2.90E-05	3.71E-05	1.98E-05	3.82E-05
H28G03.1c.2	263	357	275	432	2.72E-05	3.70E-05	1.92E-05	3.92E-05
H28G03.2a	96	165	93	166	3.37E-05	4.42E-05	2.02E-05	4.59E-05

H28G03.2b	52	80	47	84	2.84E-05	3.64E-05	1.93E-05	3.74E-05
H28G03.2c.1	51	80	45	84	5.63E-06	9.15E-06	3.55E-06	7.83E-06
H28G03.2c.2	62	91	56	109	4.40E-06	6.37E-06	2.59E-06	5.69E-06
H28G03.3	3	5	2	4	5.15E-06	7.64E-06	2.97E-06	6.84E-06
H28G03.4	9	11	15	6	6.13E-06	8.49E-06	3.59E-06	8.64E-06
H28G03.5	15	50	38	66	2.80E-06	2.65E-06	1.82E-06	2.25E-06
H28G03.6	176	241	176	294	2.80E-06	2.65E-06	1.82E-06	2.25E-06
H28O16.1a	13901	13087	14186	18578	2.80E-06	5.18E-06	2.71E-06	5.82E-06
H28O16.1b	3122	2865	2927	3529	3.58E-06	4.63E-06	2.33E-06	4.81E-06
H28O16.1c	15544	14209	15972	20883	9.00E-04	8.00E-04	5.98E-04	9.66E-04
H28O16.1d.1	13844	13029	14135	18535	6.83E-04	5.92E-04	4.17E-04	6.20E-04
H28O16.1d.2	13849	13034	14136	18540	9.25E-04	7.99E-04	6.19E-04	9.98E-04
H28O16.1d.3	13789	12955	14079	18459	9.31E-04	8.27E-04	6.18E-04	1.00E-03
H28O16.1d.4	13818	13008	14057	18480	9.27E-04	8.24E-04	6.16E-04	9.97E-04
H28O16.2	238	188	227	350	9.30E-04	8.26E-04	6.18E-04	1.00E-03
H30A04.1a	267	275	225	316	9.85E-04	8.75E-04	6.52E-04	1.06E-03
H30A04.1b.1	266	273	224	318	2.39E-05	1.79E-05	1.49E-05	2.83E-05
H30A04.1b.2	264	273	224	316	9.44E-06	9.18E-06	5.17E-06	8.95E-06
H31B20.1	8	6	4	3	9.18E-06	8.89E-06	5.03E-06	8.82E-06
H31B20.2	1	3	3	3	9.30E-06	9.10E-06	5.14E-06	8.95E-06
H31G24.1	2	5	3	2	2.80E-06	2.65E-06	1.82E-06	2.25E-06
H31G24.2	10	9	19	8	2.80E-06	2.65E-06	1.82E-06	2.25E-06
H31G24.3	84	107	144	138	2.80E-06	2.65E-06	1.82E-06	2.25E-06
H31G24.4	1252	1492	1918	2186	2.80E-06	2.65E-06	1.82E-06	2.25E-06
H32C10.1	29	40	14	12	1.30E-05	1.56E-05	1.44E-05	1.71E-05
H32C10.2	68	148	73	70	1.11E-04	1.25E-04	1.10E-04	1.55E-04
H32C10.3	24	49	35	31	2.80E-06	3.36E-06	1.82E-06	2.25E-06
H32K16.1	34	38	32	35	8.60E-06	1.77E-05	6.01E-06	7.11E-06
H32K16.2	9	10	14	0	2.80E-06	2.65E-06	1.82E-06	2.25E-06
H32K21.1	0	7	12	1	2.80E-06	2.78E-06	1.82E-06	2.25E-06
H34C03.1	81	93	53	131	4.73E-06	4.97E-06	4.79E-06	2.25E-06
H34C03.2	1034	1175	2344	2607	2.80E-06	3.39E-06	3.99E-06	2.25E-06
H34I24.1	13	21	6	7	5.71E-06	6.19E-06	2.44E-06	7.42E-06
H34I24.2	558	609	965	1006	4.23E-05	4.54E-05	6.25E-05	8.58E-05
H34I24.3	4	4	1	4	2.80E-06	2.86E-06	1.82E-06	2.25E-06
H34P18.1	6	10	10	5	3.84E-05	3.96E-05	4.32E-05	5.56E-05
H35B03.1	48	89	50	97	2.80E-06	2.65E-06	1.82E-06	2.25E-06
H35B03.2a	202	229	221	297	2.80E-06	2.65E-06	1.82E-06	2.25E-06
H35B03.2b	68	77	61	116	3.92E-06	6.85E-06	2.64E-06	6.34E-06
H35N03.1	8	17	12	12	1.36E-05	1.45E-05	9.66E-06	1.60E-05
H35N09.1	2	4	2	2	1.41E-05	1.51E-05	8.24E-06	1.93E-05
H35N09.2	7	9	11	12	2.80E-06	2.65E-06	1.82E-06	2.25E-06
H36L18.1	6	11	13	4	2.80E-06	2.65E-06	1.82E-06	2.25E-06
H36L18.2.1	616	916	515	405	2.80E-06	2.65E-06	1.82E-06	2.25E-06
H36L18.2.2	507	766	389	337	2.80E-06	2.65E-06	1.82E-06	2.25E-06
H37A05.1	1117	2069	1203	2610	1.28E-04	1.79E-04	6.94E-05	6.73E-05
H37A05.2	7	13	5	18	9.86E-05	1.41E-04	4.92E-05	5.26E-05
H37A05.3	10	11	11	24	4.31E-05	7.54E-05	3.02E-05	8.09E-05
H37A05.4	13	22	16	11	2.80E-06	2.65E-06	1.82E-06	2.25E-06
H37N21.1	259	198	433	657	2.80E-06	2.65E-06	1.82E-06	2.45E-06
H38K22.1	902	772	968	1729	2.80E-06	4.42E-06	2.20E-06	2.25E-06
H38K22.2a	343	375	431	603	1.49E-05	1.07E-05	1.62E-05	3.03E-05
H38K22.2b	565	582	708	939	2.06E-05	1.66E-05	1.44E-05	3.16E-05
H38K22.3	172	251	315	284	4.23E-05	4.36E-05	3.46E-05	5.97E-05
H38K22.4	0	1	0	0	4.65E-05	4.53E-05	3.79E-05	6.21E-05
H38K22.5a	173	261	161	264	1.52E-05	2.10E-05	1.82E-05	2.02E-05
H38K22.5b	192	297	183	276	2.80E-06	2.65E-06	1.82E-06	2.25E-06
H38K22.5c.1	199	315	194	290	9.24E-06	1.32E-05	5.59E-06	1.13E-05
H38K22.5c.2	174	258	160	260	8.96E-06	1.31E-05	5.56E-06	1.04E-05

H39E20.1	6	0	2	4	8.15E-06	1.22E-05	5.16E-06	9.54E-06
H39E23.1a	1006	1008	1016	1795	9.10E-06	1.27E-05	5.45E-06	1.09E-05
H39E23.1b	1162	1227	1138	2174	4.48E-06	2.65E-06	1.82E-06	2.41E-06
H39E23.1c	928	941	939	1710	2.58E-05	2.44E-05	1.70E-05	3.70E-05
H39E23.2	3	2	6	4	3.20E-05	3.19E-05	2.04E-05	4.81E-05
H39E23.3	20	3	16	4	3.12E-05	2.98E-05	2.05E-05	4.61E-05
H40L08.1	122	143	123	132	2.80E-06	2.65E-06	1.82E-06	2.25E-06
H40L08.3	11	14	22	17	2.80E-06	2.65E-06	1.82E-06	2.25E-06
H41C03.1	44	62	41	40	8.09E-06	8.94E-06	5.30E-06	7.02E-06
H41C03.2	2	4	12	2	2.80E-06	2.65E-06	1.82E-06	2.25E-06
H41C03.3.1	57	69	37	69	4.14E-06	5.50E-06	2.51E-06	3.01E-06
H42K12.1a	101	112	129	162	2.80E-06	2.65E-06	1.82E-06	2.25E-06
H42K12.1b	102	114	128	165	3.78E-06	4.34E-06	1.82E-06	3.69E-06
H42K12.2	1	4	2	6	4.70E-06	4.95E-06	3.92E-06	6.07E-06
H42K12.3.1	509	657	225	179	4.73E-06	5.00E-06	3.86E-06	6.16E-06
H42K12.3.2	485	614	206	168	2.80E-06	2.65E-06	1.82E-06	2.25E-06
H43E16.1	219	182	223	150	3.37E-05	4.11E-05	9.69E-06	9.51E-06
H43I07.1	105	91	173	219	3.43E-05	4.11E-05	9.49E-06	9.56E-06
H43I07.2	287	264	358	455	6.58E-06	5.16E-06	4.35E-06	3.62E-06
H43I07.3	197	219	212	296	1.47E-05	1.20E-05	1.57E-05	2.46E-05
JC8.10a	251	324	398	489	2.63E-05	2.29E-05	2.14E-05	3.35E-05
JC8.10b	251	324	393	485	1.69E-05	1.78E-05	1.18E-05	2.04E-05
JC8.11a	144	137	183	249	7.70E-06	9.36E-06	7.93E-06	1.20E-05
JC8.12a	35	28	18	9	7.64E-06	9.34E-06	7.80E-06	1.19E-05
JC8.13	175	146	263	274	1.67E-05	1.50E-05	1.38E-05	2.32E-05
JC8.14	215	375	711	350	3.61E-06	2.72E-06	1.82E-06	2.25E-06
JC8.2.1	553	692	565	746	1.94E-05	1.53E-05	1.90E-05	2.44E-05
JC8.2.2	510	654	477	655	5.69E-05	9.38E-05	1.23E-04	7.44E-05
JC8.3c.1	6221	6132	11085	7229	3.86E-05	4.57E-05	2.57E-05	4.18E-05
JC8.3c.2	6067	5977	10597	7027	4.05E-05	4.91E-05	2.47E-05	4.18E-05
JC8.3c.3	5818	5730	10629	6788	1.30E-03	1.21E-03	1.50E-03	1.21E-03
JC8.3c.4	5456	5373	10275	6286	9.57E-04	8.91E-04	1.09E-03	8.90E-04
JC8.3c.5	5837	5743	10643	6796	9.98E-04	9.28E-04	1.19E-03	9.35E-04
JC8.3c.6	6049	5947	10618	7017	1.00E-03	9.33E-04	1.23E-03	9.29E-04
JC8.3c.7	6014	5926	10522	6994	1.20E-03	1.12E-03	1.43E-03	1.12E-03
JC8.3c.8	5736	5616	10529	6659	1.07E-03	9.91E-04	1.22E-03	9.94E-04
JC8.4	23	30	43	26	1.20E-03	1.12E-03	1.37E-03	1.12E-03
JC8.5	164	152	179	234	1.14E-03	1.06E-03	1.37E-03	1.07E-03
JC8.6a	432	528	695	785	4.84E-06	5.98E-06	5.90E-06	4.41E-06
JC8.6b	337	427	507	595	2.29E-05	2.00E-05	1.63E-05	2.62E-05
JC8.7	159	178	167	255	3.15E-05	3.64E-05	3.30E-05	4.60E-05
JC8.8.1	690	1335	1331	746	2.91E-05	3.49E-05	2.85E-05	4.13E-05
K01A11.1	12	24	24	13	1.75E-05	1.85E-05	1.20E-05	2.26E-05
K01A11.2	78	75	85	67	1.68E-04	3.06E-04	2.10E-04	1.46E-04
K01A11.3	3	3	1	3	2.80E-06	4.26E-06	2.93E-06	2.25E-06
K01A11.4.1	64	89	56	42	1.27E-05	1.15E-05	8.98E-06	8.73E-06
K01A11.4.2	65	89	57	42	2.80E-06	2.65E-06	1.82E-06	2.25E-06
K01A11.5	190	292	266	284	2.80E-06	3.52E-06	1.82E-06	2.25E-06
K01A11.t1	0	0	3	0	2.80E-06	3.54E-06	1.82E-06	2.25E-06
K01A12.2	1	1	2	0	1.13E-05	1.64E-05	1.03E-05	1.36E-05
K01A12.3	18	5	11	5	2.80E-06	2.65E-06	2.99E-06	2.25E-06
K01A12.4	93	152	131	64	2.80E-06	2.65E-06	1.82E-06	2.25E-06
K01A2.1	27	50	26	32	2.80E-06	2.65E-06	1.82E-06	2.25E-06
K01A2.10	16	38	16	22	2.94E-05	4.54E-05	2.70E-05	1.63E-05
K01A2.11a	48	73	27	20	3.50E-06	6.11E-06	2.19E-06	3.33E-06
K01A2.11b	48	61	25	19	2.80E-06	4.66E-06	1.82E-06	2.29E-06
K01A2.11c	37	53	15	12	2.94E-06	4.23E-06	1.82E-06	2.25E-06
K01A2.11d	55	87	36	24	3.44E-06	4.13E-06	1.82E-06	2.25E-06
K01A2.2b	5	17	6	8	2.97E-06	4.02E-06	1.82E-06	2.25E-06

K01A2.3	10	8	6	0	2.91E-06	4.36E-06	1.82E-06	2.25E-06
K01A2.4	43	36	32	16	2.80E-06	3.41E-06	1.82E-06	2.25E-06
K01A2.5	49	110	43	67	2.80E-06	2.65E-06	1.82E-06	2.25E-06
K01A2.6	2	2	2	3	3.67E-06	2.88E-06	1.82E-06	2.25E-06
K01A2.7	7	13	3	6	6.75E-06	1.43E-05	3.86E-06	7.42E-06
K01A2.8a	188	210	132	180	2.80E-06	2.65E-06	1.82E-06	2.25E-06
K01A2.8c	53	67	39	52	2.80E-06	2.65E-06	1.82E-06	2.25E-06
K01A2.8d	38	51	33	40	1.86E-05	1.96E-05	8.49E-06	1.43E-05
K01A2.9	13	26	17	24	6.22E-06	7.43E-06	2.99E-06	4.90E-06
K01A6.2	201	349	338	520	8.29E-06	1.05E-05	4.68E-06	7.02E-06
K01A6.4	6	6	6	1	3.22E-06	6.08E-06	2.73E-06	4.77E-06
K01A6.5	3	5	0	5	6.33E-06	1.04E-05	6.92E-06	1.31E-05
K01A6.6	12	16	9	8	2.80E-06	2.65E-06	1.82E-06	2.25E-06
K01A6.t1	0	0	0	1	2.80E-06	2.65E-06	1.82E-06	2.25E-06
K01B6.1	102	127	89	94	2.80E-06	2.65E-06	1.82E-06	2.25E-06
K01B6.2	10	18	7	12	2.80E-06	2.65E-06	1.82E-06	2.25E-06
K01B6.3.1	20	22	13	29	4.00E-06	4.71E-06	2.28E-06	2.97E-06
K01B6.3.2	20	20	14	29	2.80E-06	2.65E-06	1.82E-06	2.25E-06
K01B6.4	1	2	3	1	2.80E-06	2.65E-06	1.82E-06	2.25E-06
K01C8.1.1	276	734	279	452	2.80E-06	2.65E-06	1.82E-06	2.25E-06
K01C8.1.2	281	753	296	451	2.80E-06	2.65E-06	1.82E-06	2.25E-06
K01C8.1.3	272	697	261	424	1.92E-05	4.83E-05	1.26E-05	2.53E-05
K01C8.10.1	2555	2638	2515	3523	1.71E-05	4.32E-05	1.17E-05	2.20E-05
K01C8.10.3	2262	2309	2225	3274	2.01E-05	4.87E-05	1.26E-05	2.52E-05
K01C8.2	18	14	11	15	1.54E-04	1.50E-04	9.86E-05	1.70E-04
K01C8.3a	943	957	1686	2070	1.39E-04	1.35E-04	8.93E-05	1.62E-04
K01C8.3b	848	859	1562	1931	2.80E-06	2.65E-06	1.82E-06	2.25E-06
K01C8.5	388	373	386	725	5.04E-05	4.84E-05	5.87E-05	8.89E-05
K01C8.6	294	319	413	379	4.40E-05	4.21E-05	5.27E-05	8.04E-05
K01C8.8	27	34	50	27	1.99E-05	1.80E-05	1.29E-05	2.98E-05
K01C8.9	925	1112	883	1342	4.36E-05	4.47E-05	3.99E-05	4.52E-05
K01D12.1	13	7	7	10	2.80E-06	2.65E-06	2.20E-06	2.25E-06
K01D12.10	16	24	13	14	4.89E-05	5.55E-05	3.04E-05	5.70E-05
K01D12.11	102	170	100	80	2.80E-06	2.65E-06	1.82E-06	2.25E-06
K01D12.12	219	377	409	339	3.05E-06	4.34E-06	1.82E-06	2.25E-06
K01D12.13a	50	85	58	50	1.23E-05	1.93E-05	7.84E-06	7.74E-06
K01D12.13b	46	86	59	51	2.72E-05	4.42E-05	3.31E-05	3.38E-05
K01D12.14	8	11	12	5	6.64E-06	1.07E-05	5.01E-06	5.33E-06
K01D12.15	39	48	53	18	5.57E-06	9.87E-06	4.66E-06	4.97E-06
K01D12.2	24	24	26	30	2.80E-06	2.65E-06	1.82E-06	2.25E-06
K01D12.3	2	8	4	5	1.11E-05	1.30E-05	9.86E-06	4.14E-06
K01D12.4	6	4	10	5	2.80E-06	2.65E-06	1.82E-06	2.25E-06
K01D12.5	33	46	6	5	2.80E-06	2.65E-06	1.82E-06	2.25E-06
K01D12.8	32	35	6	13	2.80E-06	2.65E-06	1.82E-06	2.25E-06
K01D12.9	159	132	60	21	4.54E-06	5.98E-06	1.82E-06	2.25E-06
K01F9.2	16	28	24	13	8.54E-06	8.81E-06	1.82E-06	2.79E-06
K01G12.3	40	30	43	42	5.40E-05	4.23E-05	1.32E-05	5.74E-06
K01G5.10	33	28	33	20	3.81E-06	6.30E-06	3.72E-06	2.47E-06
K01G5.2b	208	246	385	357	2.80E-06	2.65E-06	1.82E-06	2.25E-06
K01G5.2c	196	228	381	358	4.96E-06	3.97E-06	3.23E-06	2.41E-06
K01G5.3	206	137	264	293	2.40E-05	2.68E-05	2.89E-05	3.30E-05
K01G5.4.1	3726	4029	4935	4355	2.40E-05	2.63E-05	3.03E-05	3.52E-05
K01G5.4.2	2560	2801	3411	3209	1.97E-05	1.24E-05	1.64E-05	2.25E-05
K01G5.5.1	2414	2074	2167	2584	4.35E-04	4.45E-04	3.75E-04	4.09E-04
K01G5.5.2	2121	1836	2023	2453	2.98E-04	3.08E-04	2.58E-04	3.00E-04
K01G5.6	241	292	311	404	1.79E-04	1.46E-04	1.05E-04	1.54E-04
K01G5.7.1	4609	4241	5663	6463	1.76E-04	1.44E-04	1.09E-04	1.63E-04
K01G5.7.2	4075	3842	4835	5926	1.02E-05	1.17E-05	8.58E-06	1.38E-05
K01G5.8b	127	144	370	155	3.48E-04	3.03E-04	2.79E-04	3.92E-04

K01G5.9.1	194	267	301	465	3.36E-04	3.00E-04	2.60E-04	3.93E-04
K01G5.9.2	207	283	319	487	3.51E-05	3.76E-05	6.66E-05	3.44E-05
K01G5.9.3	165	230	239	387	1.61E-05	2.09E-05	1.62E-05	3.09E-05
K01H12.1	73	77	273	74	1.57E-05	2.02E-05	1.57E-05	2.96E-05
K01H12.2	133	225	100	64	1.69E-05	2.22E-05	1.59E-05	3.18E-05
K01H12.3	2	4	3	1	3.37E-05	3.35E-05	8.19E-05	2.74E-05
K01H12.4	55	87	25	20	1.58E-05	2.53E-05	7.74E-06	6.12E-06
K02A11.1a	797	856	929	1324	2.80E-06	2.65E-06	1.82E-06	2.25E-06
K02A11.1b.1	877	913	977	1467	5.74E-06	8.57E-06	1.82E-06	2.25E-06
K02A11.1b.2	916	970	1036	1531	5.43E-05	5.51E-05	4.12E-05	7.25E-05
K02A11.2	7	14	4	3	4.30E-05	4.23E-05	3.12E-05	5.78E-05
K02A11.3	51	109	53	88	4.64E-05	4.64E-05	3.41E-05	6.23E-05
K02A11.4	34	48	31	15	2.80E-06	2.65E-06	1.82E-06	2.25E-06
K02A2.1	4	1	0	1	6.19E-06	1.25E-05	4.17E-06	8.57E-06
K02A2.2	9	8	7	6	1.12E-05	1.50E-05	6.67E-06	3.98E-06
K02A2.3	37	55	44	41	2.80E-06	2.65E-06	1.82E-06	2.25E-06
K02A2.4	8	9	5	11	2.80E-06	2.65E-06	1.82E-06	2.25E-06
K02A2.5	17	16	6	5	2.80E-06	2.65E-06	1.82E-06	2.25E-06
K02A2.7	4	6	11	5	2.80E-06	2.65E-06	1.82E-06	2.25E-06
K02A4.1	989	1983	1053	2305	3.70E-06	3.28E-06	1.82E-06	2.25E-06
K02A6.1	0	2	1	3	2.80E-06	2.65E-06	1.82E-06	2.25E-06
K02A6.2	2	0	0	1	6.00E-05	1.14E-04	4.16E-05	1.12E-04
K02A6.3a	35	66	23	39	2.80E-06	2.65E-06	1.82E-06	2.25E-06
K02A6.3b.1	29	68	18	35	2.80E-06	2.65E-06	1.82E-06	2.25E-06
K02A6.3b.2	14	36	7	9	2.80E-06	4.47E-06	1.82E-06	2.25E-06
K02B12.1	21	30	29	19	2.80E-06	5.21E-06	1.82E-06	2.29E-06
K02B12.2	15	17	16	23	2.80E-06	5.58E-06	1.82E-06	2.25E-06
K02B12.3.1	312	338	389	521	2.80E-06	2.65E-06	1.82E-06	2.25E-06
K02B12.4a	7	10	11	4	2.86E-06	3.04E-06	1.97E-06	3.51E-06
K02B12.4b	12	16	34	11	2.31E-05	2.37E-05	1.88E-05	3.10E-05
K02B12.5	430	489	569	877	2.80E-06	2.65E-06	1.82E-06	2.25E-06
K02B12.6	9	2	2	1	2.80E-06	2.65E-06	1.88E-06	2.25E-06
K02B12.7	353	343	298	504	1.31E-05	1.41E-05	1.13E-05	2.15E-05
K02B12.8	219	227	215	416	2.80E-06	2.65E-06	1.82E-06	2.25E-06
K02B12.9	4	2	6	3	2.61E-05	2.40E-05	1.44E-05	3.00E-05
K02B2.1	397	478	544	768	2.06E-05	2.02E-05	1.31E-05	3.14E-05
K02B2.3.1	399	458	500	623	2.80E-06	2.65E-06	1.82E-06	2.25E-06
K02B2.3.2	318	361	381	494	2.88E-05	3.27E-05	2.57E-05	4.47E-05
K02B2.3.3	324	368	386	498	3.62E-05	3.93E-05	2.95E-05	4.54E-05
K02B2.4	131	137	229	318	3.51E-05	3.77E-05	2.74E-05	4.38E-05
K02B2.5.1	8008	9377	15551	4311	3.56E-05	3.82E-05	2.76E-05	4.40E-05
K02B2.5.2	6316	7451	11454	3676	7.67E-06	7.59E-06	8.75E-06	1.50E-05
K02B2.6	1	1	1	1	2.21E-03	2.45E-03	2.80E-03	9.58E-04
K02B7.1	46	120	39	211	1.76E-03	1.96E-03	2.07E-03	8.21E-04
K02B7.2	11	40	22	49	2.80E-06	2.65E-06	1.82E-06	2.25E-06
K02B9.1	706	661	1461	1452	2.80E-06	5.69E-06	1.82E-06	8.52E-06
K02B9.2	122	134	174	237	2.80E-06	3.60E-06	1.82E-06	3.76E-06
K02B9.3a	4	4	2	7	3.36E-05	2.98E-05	4.53E-05	5.56E-05
K02B9.4	112	172	107	181	5.52E-06	5.74E-06	5.14E-06	8.64E-06
K02C4.2	4	7	10	6	2.80E-06	2.65E-06	1.82E-06	2.25E-06
K02C4.3	1135	1238	1649	2411	1.00E-05	1.45E-05	6.23E-06	1.30E-05
K02C4.4	87	162	46	96	2.80E-06	2.65E-06	1.82E-06	2.25E-06
K02C4.5	117	188	71	120	3.25E-05	3.35E-05	3.07E-05	5.55E-05
K02D10.1a	520	623	492	700	3.75E-06	6.59E-06	1.82E-06	3.33E-06
K02D10.1b.1	686	752	654	872	9.83E-06	1.49E-05	3.88E-06	8.10E-06
K02D10.1b.2	509	569	455	628	3.68E-05	4.17E-05	2.27E-05	3.98E-05
K02D10.2	2	6	2	3	6.73E-05	6.97E-05	4.17E-05	6.87E-05
K02D10.3	7	5	3	4	6.46E-05	6.82E-05	3.76E-05	6.41E-05
K02D10.4	35	42	73	59	2.80E-06	2.65E-06	1.82E-06	2.25E-06

K02D10.5	460	588	752	750	2.80E-06	2.65E-06	1.82E-06	2.25E-06
K02D3.1	3	13	7	5	2.80E-06	2.96E-06	3.55E-06	3.55E-06
K02D3.2	5	16	5	1	4.34E-05	5.25E-05	4.62E-05	5.69E-05
K02D7.1	314	617	300	464	2.80E-06	2.65E-06	1.82E-06	2.25E-06
K02D7.2	5	4	5	3	2.80E-06	2.65E-06	1.82E-06	2.25E-06
K02D7.3	3130	3428	4946	5616	3.07E-05	5.70E-05	1.91E-05	3.65E-05
K02D7.5	47	147	40	71	2.80E-06	2.65E-06	1.82E-06	2.25E-06
K02D7.6	2	5	5	2	3.41E-04	3.53E-04	3.51E-04	4.92E-04
K02E10.1	31	53	26	32	4.34E-06	1.28E-05	2.41E-06	5.26E-06
K02E10.2a	142	203	114	214	2.80E-06	2.65E-06	1.82E-06	2.25E-06
K02E10.2b	92	169	99	199	3.70E-06	5.95E-06	2.02E-06	3.06E-06
K02E10.4a	77	94	59	60	6.33E-06	8.54E-06	3.30E-06	7.65E-06
K02E10.4b	77	93	61	60	3.33E-06	5.77E-06	2.33E-06	5.78E-06
K02E10.5	1	5	0	3	5.91E-06	6.80E-06	2.95E-06	3.69E-06
K02E10.6	7	16	9	10	5.68E-06	6.51E-06	2.93E-06	3.55E-06
K02E10.7	18	22	48	30	2.80E-06	2.65E-06	1.82E-06	2.25E-06
K02E10.8	48	71	46	70	2.80E-06	2.65E-06	1.82E-06	2.25E-06
K02E11.1	48	87	38	27	2.80E-06	2.65E-06	1.82E-06	2.25E-06
K02E11.10	368	435	120	102	2.80E-06	2.99E-06	1.82E-06	2.50E-06
K02E11.2	7	6	5	7	2.80E-06	4.10E-06	1.82E-06	2.25E-06
K02E11.3	14	21	21	5	3.81E-05	4.25E-05	8.07E-06	8.48E-06
K02E11.4	11	6	20	3	2.80E-06	2.65E-06	1.82E-06	2.25E-06
K02E11.5	28	24	42	14	3.95E-06	5.61E-06	3.86E-06	2.25E-06
K02E11.6	20	23	25	6	3.95E-06	2.65E-06	4.66E-06	2.25E-06
K02E11.7	20	20	51	3	8.23E-06	6.67E-06	8.04E-06	3.31E-06
K02E11.8	4	5	5	3	5.80E-06	6.30E-06	4.70E-06	2.25E-06
K02E11.9	6	4	2	3	4.65E-06	4.39E-06	7.73E-06	2.25E-06
K02E2.1	2	3	8	1	2.80E-06	2.65E-06	1.82E-06	2.25E-06
K02E2.2	14	11	7	10	2.80E-06	2.65E-06	1.82E-06	2.25E-06
K02E2.3	3	3	2	0	2.80E-06	2.65E-06	1.82E-06	2.25E-06
K02E2.4	21	14	21	2	2.80E-06	2.65E-06	1.82E-06	2.25E-06
K02E2.5	3	5	2	4	2.80E-06	2.65E-06	1.82E-06	2.25E-06
K02E2.6.1	62	117	158	62	6.22E-06	3.91E-06	4.05E-06	2.25E-06
K02E2.6.2	72	125	182	76	2.80E-06	2.65E-06	1.82E-06	2.25E-06
K02E2.7	7	4	1	4	1.30E-05	2.33E-05	2.16E-05	1.05E-05
K02E7.10	3	12	11	8	1.50E-05	2.46E-05	2.47E-05	1.27E-05
K02E7.11	7	5	11	6	2.80E-06	2.65E-06	1.82E-06	2.25E-06
K02E7.12.1	9	10	28	6	2.80E-06	2.65E-06	1.82E-06	2.25E-06
K02E7.12.2	8	6	18	4	2.80E-06	2.65E-06	1.95E-06	2.25E-06
K02E7.1a	7	13	3	0	2.80E-06	2.94E-06	5.67E-06	2.25E-06
K02E7.1b	5	9	2	0	3.00E-06	2.65E-06	4.41E-06	2.25E-06
K02E7.2	13	37	21	44	2.80E-06	2.65E-06	1.82E-06	2.25E-06
K02E7.3	44	126	47	232	2.80E-06	2.65E-06	1.82E-06	2.25E-06
K02E7.4	6	7	10	5	2.80E-06	3.17E-06	1.82E-06	3.19E-06
K02E7.5	1	1	0	0	2.80E-06	5.50E-06	1.82E-06	8.64E-06
K02E7.6.1	52	32	28	8	2.80E-06	2.65E-06	1.82E-06	2.25E-06
K02E7.6.2	46	32	26	7	2.80E-06	2.65E-06	1.82E-06	2.25E-06
K02E7.7	7	6	12	11	3.33E-06	2.65E-06	1.82E-06	2.25E-06
K02E7.8	8	13	21	9	2.91E-06	2.65E-06	1.82E-06	2.25E-06
K02E7.9	10	12	13	6	2.80E-06	2.65E-06	1.82E-06	2.25E-06
K02F2.1a	1495	1500	1951	2505	2.80E-06	2.65E-06	2.88E-06	2.25E-06
K02F2.1b	1540	1580	1972	2560	2.80E-06	2.65E-06	1.82E-06	2.25E-06
K02F2.2.1	9881	13652	9986	15436	5.99E-05	5.68E-05	5.09E-05	8.06E-05
K02F2.2.2	8386	11684	8415	13605	5.70E-05	5.52E-05	4.75E-05	7.61E-05
K02F2.3	963	939	1366	1738	7.23E-04	9.43E-04	4.75E-04	9.07E-04
K02F2.4	72	77	102	120	7.06E-04	9.29E-04	4.61E-04	9.20E-04
K02F2.5	84	121	90	90	2.74E-05	2.52E-05	2.53E-05	3.97E-05
K02F2.6	38	70	34	41	8.62E-06	8.70E-06	7.94E-06	1.15E-05
K02F3.1	72	92	117	164	9.83E-06	1.34E-05	6.85E-06	8.46E-06

K02F3.10	443	457	492	529	2.80E-06	3.44E-06	1.82E-06	2.25E-06
K02F3.12a	290	345	262	466	4.98E-06	6.03E-06	5.28E-06	9.13E-06
K02F3.12b	280	329	243	452	7.60E-05	7.40E-05	5.49E-05	7.29E-05
K02F3.2	121	153	338	449	1.69E-05	1.90E-05	9.93E-06	2.18E-05
K02F3.3	186	329	224	104	1.71E-05	1.90E-05	9.68E-06	2.22E-05
K02F3.4.1	675	1265	485	821	6.33E-06	7.56E-06	1.15E-05	1.89E-05
K02F3.4.2	685	1317	519	840	2.83E-05	4.73E-05	2.22E-05	1.27E-05
K02F3.5	42	49	25	27	5.52E-05	9.78E-05	2.58E-05	5.40E-05
K02F3.6	58	79	61	74	4.19E-05	7.62E-05	2.07E-05	4.13E-05
K02F3.7	9	6	5	3	5.07E-06	5.58E-06	1.97E-06	2.63E-06
K02F3.8	11	9	3	4	5.35E-06	6.88E-06	3.64E-06	5.47E-06
K02F3.9	144	214	179	104	2.80E-06	2.65E-06	1.82E-06	2.25E-06
K02F6.1	14	22	11	1	2.80E-06	2.65E-06	1.82E-06	2.25E-06
K02F6.2	17	14	11	7	2.79E-05	3.92E-05	2.26E-05	1.62E-05
K02F6.3	35	68	39	34	2.80E-06	2.65E-06	1.82E-06	2.25E-06
K02F6.4	19	21	11	8	2.80E-06	2.65E-06	1.82E-06	2.25E-06
K02F6.5	2	5	9	5	2.80E-06	2.86E-06	1.82E-06	2.25E-06
K02F6.6	0	5	5	0	2.80E-06	2.65E-06	1.82E-06	2.25E-06
K02F6.7	105	121	170	168	2.80E-06	2.65E-06	1.82E-06	2.25E-06
K02F6.8	3	5	9	5	2.80E-06	2.65E-06	1.82E-06	2.25E-06
K02F6.9	25	17	16	9	7.11E-06	7.75E-06	7.49E-06	9.13E-06
K02G10.1	32	42	48	45	2.80E-06	2.65E-06	1.82E-06	2.25E-06
K02G10.3.1	60	112	57	60	2.80E-06	2.65E-06	1.82E-06	2.25E-06
K02G10.4a	65	102	61	64	4.23E-06	5.26E-06	4.14E-06	4.79E-06
K02G10.4b	33	61	30	33	4.62E-06	8.15E-06	2.86E-06	3.71E-06
K02G10.4c	54	93	51	48	1.23E-05	1.83E-05	7.54E-06	9.78E-06
K02G10.5	52	80	30	37	1.12E-05	1.95E-05	6.63E-06	9.00E-06
K02G10.6	397	462	354	605	9.02E-06	1.47E-05	5.54E-06	6.45E-06
K02G10.7a	151	400	163	329	2.80E-06	3.73E-06	1.82E-06	2.25E-06
K02G10.7b	150	416	169	344	3.16E-05	3.48E-05	1.84E-05	3.87E-05
K02G10.8	147	225	93	166	1.78E-05	4.45E-05	1.25E-05	3.11E-05
K02H11.1	6	2	3	4	1.79E-05	4.70E-05	1.32E-05	3.31E-05
K02H11.2	4	3	16	3	1.42E-05	2.05E-05	5.85E-06	1.29E-05
K02H11.3	4	2	3	0	2.80E-06	2.65E-06	1.82E-06	2.25E-06
K02H11.4	7	8	5	3	2.80E-06	2.65E-06	1.82E-06	2.25E-06
K02H11.5	1	7	3	1	2.80E-06	2.65E-06	1.82E-06	2.25E-06
K02H11.6	0	2	3	1	2.80E-06	2.65E-06	1.82E-06	2.25E-06
K02H11.7	3	3	1	0	2.80E-06	2.65E-06	1.82E-06	2.25E-06
K02H11.8	1	2	3	2	2.80E-06	2.65E-06	1.82E-06	2.25E-06
K02H11.9	3	5	2	0	2.80E-06	2.65E-06	1.82E-06	2.25E-06
K02H8.1	91	86	75	120	2.80E-06	2.65E-06	1.82E-06	2.25E-06
K03A1.1	32	40	54	29	2.80E-06	2.65E-06	1.82E-06	2.25E-06
K03A1.4a	10	21	13	3	1.04E-05	9.34E-06	5.61E-06	1.11E-05
K03A1.4b	0	2	0	0	8.74E-06	1.03E-05	9.60E-06	6.36E-06
K03A1.5	699	856	1002	1250	2.80E-06	2.80E-06	1.82E-06	2.25E-06
K03A1.6	11	20	38	10	2.80E-06	2.65E-06	1.82E-06	2.25E-06
K03A11.1	39	32	41	41	3.12E-05	3.61E-05	2.91E-05	4.48E-05
K03A11.2	2	2	4	0	3.95E-06	6.77E-06	8.87E-06	2.88E-06
K03A11.3	4	1	1	2	2.80E-06	2.65E-06	1.82E-06	2.25E-06
K03A11.4	9	7	11	6	2.80E-06	2.65E-06	1.82E-06	2.25E-06
K03A11.5	10	25	17	14	2.80E-06	2.65E-06	1.82E-06	2.25E-06
K03B4.1	214	184	338	396	2.80E-06	2.65E-06	1.82E-06	2.25E-06
K03B4.2	542	450	595	859	2.80E-06	3.04E-06	1.82E-06	2.25E-06
K03B4.3b	157	137	226	218	1.98E-05	1.61E-05	2.04E-05	2.94E-05
K03B4.4	9	9	7	5	7.47E-05	5.86E-05	5.33E-05	9.50E-05
K03B4.5	7	6	9	5	3.26E-05	2.68E-05	3.05E-05	3.63E-05
K03B4.6	7	10	11	8	3.81E-06	3.60E-06	1.93E-06	2.25E-06
K03B4.7a.1	306	307	1116	449	2.80E-06	2.65E-06	1.82E-06	2.25E-06
K03B4.7a.2	250	241	989	415	2.80E-06	2.65E-06	1.82E-06	2.25E-06

K03B4.7b	290	287	1110	423	6.57E-05	6.22E-05	1.56E-04	7.74E-05
K03B8.1	9	4	12	4	5.40E-05	4.91E-05	1.39E-04	7.19E-05
K03B8.10	1	0	1	1	3.92E-05	3.66E-05	9.76E-05	4.59E-05
K03B8.2	3	9	5	6	2.80E-06	2.65E-06	1.82E-06	2.25E-06
K03B8.3	1	4	3	2	2.80E-06	2.65E-06	1.82E-06	2.25E-06
K03B8.4	15	17	33	27	2.80E-06	2.65E-06	1.82E-06	2.25E-06
K03B8.5	1	2	8	2	2.80E-06	2.65E-06	1.82E-06	2.25E-06
K03B8.6.1	175	192	110	126	5.77E-06	6.19E-06	8.27E-06	8.34E-06
K03B8.6.2	98	109	65	60	2.80E-06	2.65E-06	1.82E-06	2.25E-06
K03B8.7	4	11	5	1	1.34E-05	1.39E-05	5.48E-06	7.74E-06
K03B8.8	30	34	29	37	1.10E-05	1.16E-05	4.76E-06	5.42E-06
K03B8.9.1	32	42	45	35	2.80E-06	2.65E-06	1.82E-06	2.25E-06
K03B8.9.2	32	43	45	35	6.47E-06	6.93E-06	4.08E-06	6.41E-06
K03C7.1	268	304	148	277	2.80E-06	2.65E-06	1.82E-06	2.25E-06
K03C7.2a	337	451	332	550	2.80E-06	2.65E-06	1.82E-06	2.25E-06
K03C7.2b.2	117	172	133	181	1.07E-05	1.15E-05	3.86E-06	8.91E-06
K03C7.2c.1	75	103	93	132	1.85E-05	2.34E-05	1.18E-05	2.42E-05
K03C7.2c.2	103	149	122	168	1.38E-05	1.91E-05	1.02E-05	1.71E-05
K03C7.2c.3	75	103	92	131	1.23E-05	1.60E-05	9.95E-06	1.74E-05
K03C7.3	4	5	2	4	1.37E-05	1.87E-05	1.06E-05	1.79E-05
K03D10.1	33	50	43	64	1.74E-05	2.26E-05	1.39E-05	2.44E-05
K03D10.3	297	223	528	614	2.80E-06	2.65E-06	1.82E-06	2.25E-06
K03D3.1	8	6	5	2	2.80E-06	2.65E-06	1.82E-06	2.50E-06
K03D3.10	1	2	6	1	1.68E-05	1.19E-05	1.95E-05	2.79E-05
K03D3.11	2	6	5	0	2.80E-06	2.65E-06	1.82E-06	2.25E-06
K03D3.12	7	3	2	2	2.80E-06	2.65E-06	1.82E-06	2.25E-06
K03D3.13	1	3	5	0	2.80E-06	2.65E-06	1.82E-06	2.25E-06
K03D3.2	3	0	4	2	2.80E-06	2.65E-06	1.82E-06	2.25E-06
K03D3.3	10	10	22	9	2.80E-06	2.65E-06	1.82E-06	2.25E-06
K03D3.4	7	5	3	2	2.80E-06	2.65E-06	1.82E-06	2.25E-06
K03D3.5	0	0	2	2	2.80E-06	2.65E-06	2.28E-06	2.25E-06
K03D7.1	399	470	675	930	2.80E-06	2.65E-06	1.82E-06	2.25E-06
K03D7.10	6	7	3	2	2.80E-06	2.65E-06	1.82E-06	2.25E-06
K03D7.11	5	3	7	2	9.94E-06	1.11E-05	1.10E-05	1.86E-05
K03D7.2	7	6	7	7	2.80E-06	2.65E-06	1.82E-06	2.25E-06
K03D7.3	41	33	58	37	2.80E-06	2.65E-06	1.82E-06	2.25E-06
K03D7.4	1	3	3	3	2.80E-06	2.65E-06	1.82E-06	2.25E-06
K03D7.5	3	4	2	4	2.80E-06	2.65E-06	1.82E-06	2.25E-06
K03D7.6	4	3	4	2	2.80E-06	2.65E-06	1.82E-06	2.25E-06
K03D7.7	3	5	7	9	2.80E-06	2.65E-06	1.82E-06	2.25E-06
K03D7.8	11	11	11	10	2.80E-06	2.65E-06	1.82E-06	2.25E-06
K03D7.9	1	5	3	1	2.80E-06	2.65E-06	1.82E-06	2.25E-06
K03E5.1	8	11	11	4	2.80E-06	2.65E-06	1.82E-06	2.25E-06
K03E5.2a	127	174	117	149	2.80E-06	2.65E-06	1.82E-06	2.25E-06
K03E5.2b	113	157	111	139	2.80E-06	2.65E-06	1.82E-06	2.25E-06
K03E5.2c	68	67	66	93	1.70E-05	2.20E-05	1.02E-05	1.60E-05
K03E5.3	28	54	138	128	1.85E-05	2.42E-05	1.18E-05	1.82E-05
K03E6.1	18	17	9	26	6.05E-05	5.63E-05	3.82E-05	6.64E-05
K03E6.3	16	43	8	11	2.83E-06	5.16E-06	9.09E-06	1.04E-05
K03E6.4	5	2	2	2	2.80E-06	2.65E-06	1.82E-06	2.70E-06
K03E6.5	101	134	81	108	3.00E-06	7.59E-06	1.82E-06	2.25E-06
K03E6.6	69	115	160	129	2.80E-06	2.65E-06	1.82E-06	2.25E-06
K03E6.7.1	119	177	74	139	9.91E-06	1.24E-05	5.17E-06	8.50E-06
K03E6.7.2	95	147	56	112	1.78E-05	2.80E-05	2.68E-05	2.67E-05
K03F8.1	2	1	0	1	8.01E-06	1.13E-05	3.24E-06	7.51E-06
K03F8.2	5	8	8	7	7.92E-06	1.16E-05	3.04E-06	7.51E-06
K03F8.t1	0	0	0	1	2.80E-06	2.65E-06	1.82E-06	2.25E-06
K03H1.1	55	108	39	26	2.80E-06	2.65E-06	1.82E-06	2.25E-06
K03H1.10.1	68	154	65	79	2.80E-06	2.65E-06	1.82E-06	2.25E-06

K03H1.10.2	71	159	64	77	5.10E-06	9.47E-06	2.35E-06	2.25E-06
K03H1.11	40	84	124	160	6.30E-06	1.35E-05	3.92E-06	5.89E-06
K03H1.12	34	25	29	14	6.80E-06	1.44E-05	3.99E-06	5.91E-06
K03H1.3	118	165	364	169	5.04E-06	9.97E-06	1.01E-05	1.61E-05
K03H1.5	170	189	92	153	8.62E-06	6.00E-06	4.79E-06	2.86E-06
K03H1.6	30	56	49	67	2.96E-05	3.91E-05	5.93E-05	3.40E-05
K03H1.7	264	278	435	340	4.45E-06	4.68E-06	1.82E-06	3.22E-06
K03H1.8	6	2	4	4	7.14E-06	1.26E-05	7.58E-06	1.28E-05
K03H1.9	9	17	10	8	4.99E-05	4.96E-05	5.35E-05	5.16E-05
K03H4.1	8	10	21	9	2.80E-06	2.65E-06	1.82E-06	2.25E-06
K03H4.2	246	215	349	462	2.80E-06	3.02E-06	1.82E-06	2.25E-06
K03H6.1	6	8	11	6	2.80E-06	2.65E-06	1.82E-06	2.25E-06
K03H6.2	103	113	149	78	3.47E-05	2.86E-05	3.20E-05	5.23E-05
K03H6.4	4	3	3	3	2.80E-06	2.65E-06	1.82E-06	2.25E-06
K03H6.5	8	12	5	4	1.10E-05	1.14E-05	1.04E-05	6.70E-06
K03H6.6	17	22	36	43	2.80E-06	2.65E-06	1.82E-06	2.25E-06
K03H6.7	2	6	13	4	2.80E-06	2.65E-06	1.82E-06	2.25E-06
K03H9.1	2	1	2	1	2.80E-06	2.65E-06	2.95E-06	4.36E-06
K03H9.2	5	10	5	4	2.80E-06	2.65E-06	1.84E-06	2.25E-06
K03H9.3	7	17	9	4	2.80E-06	2.65E-06	1.82E-06	2.25E-06
K04A8.10	12	15	16	8	2.80E-06	2.65E-06	1.82E-06	2.25E-06
K04A8.2	7	3	4	4	2.80E-06	2.65E-06	1.82E-06	2.25E-06
K04A8.3	2	4	11	7	2.80E-06	2.65E-06	1.82E-06	2.25E-06
K04A8.4	6	9	5	3	2.80E-06	2.65E-06	1.82E-06	2.25E-06
K04A8.5	3	14	12	4	2.80E-06	2.65E-06	2.26E-06	2.25E-06
K04A8.6	307	425	226	378	2.80E-06	2.65E-06	1.82E-06	2.25E-06
K04A8.8	3	2	1	1	2.80E-06	2.65E-06	1.82E-06	2.25E-06
K04A8.9	1	4	2	1	1.04E-05	1.36E-05	4.99E-06	1.03E-05
K04A8.t2	3	2	3	2	2.80E-06	2.65E-06	1.82E-06	2.25E-06
K04B12.1	77	76	60	96	2.80E-06	2.65E-06	1.82E-06	2.25E-06
K04B12.2a	69	86	112	180	4.09E-06	2.65E-06	2.66E-06	2.25E-06
K04B12.2b.1	70	89	121	177	2.80E-06	2.65E-06	1.82E-06	2.25E-06
K04B12.2b.2	79	96	121	190	6.08E-06	7.17E-06	6.41E-06	1.27E-05
K04B12.2b.3	69	86	112	180	5.49E-06	6.59E-06	6.16E-06	1.11E-05
K04B12.3	214	258	338	499	4.82E-06	5.53E-06	4.79E-06	9.29E-06
K04C1.1	4	3	3	1	6.72E-06	7.91E-06	7.09E-06	1.41E-05
K04C1.2a	86	94	116	126	8.74E-06	9.95E-06	8.96E-06	1.64E-05
K04C1.3.1	31	44	31	46	2.80E-06	2.65E-06	1.82E-06	2.25E-06
K04C1.3.2	25	28	24	40	6.05E-06	6.24E-06	5.30E-06	7.11E-06
K04C1.4	9	10	15	8	2.94E-06	3.97E-06	1.91E-06	3.51E-06
K04C1.5	170	177	258	306	2.97E-06	3.12E-06	1.84E-06	3.80E-06
K04C1.6	2	4	10	1	2.80E-06	2.65E-06	2.53E-06	2.25E-06
K04C2.2	1012	866	1069	1620	2.48E-05	2.44E-05	2.45E-05	3.58E-05
K04C2.3a.1	289	316	616	417	2.80E-06	2.65E-06	1.82E-06	2.25E-06
K04C2.3a.2	338	387	716	449	3.62E-05	2.93E-05	2.49E-05	4.66E-05
K04C2.3b	34	35	39	36	5.77E-05	5.96E-05	8.00E-05	6.69E-05
K04C2.5	3	16	4	4	7.01E-05	7.58E-05	9.66E-05	7.48E-05
K04C2.6	2	3	5	6	2.54E-05	2.47E-05	1.90E-05	2.16E-05
K04C2.7	8	8	10	12	2.80E-06	5.18E-06	1.82E-06	2.25E-06
K04C2.8	7	12	9	7	2.80E-06	2.65E-06	1.82E-06	2.25E-06
K04C2.t3	1	0	3	0	2.80E-06	2.65E-06	1.82E-06	2.25E-06
K04D7.1.1	11525	13281	17887	15439	2.80E-06	2.72E-06	1.82E-06	2.25E-06
K04D7.1.2	11383	13101	17779	15284	2.80E-06	2.65E-06	2.99E-06	2.25E-06
K04D7.1.3	10120	11739	15217	14406	1.18E-03	1.28E-03	1.19E-03	1.27E-03
K04D7.1.4	10296	11932	16091	14878	1.23E-03	1.33E-03	1.25E-03	1.32E-03
K04D7.2a	437	417	416	638	1.13E-03	1.24E-03	1.10E-03	1.29E-03
K04D7.2b	287	277	280	451	1.14E-03	1.25E-03	1.16E-03	1.33E-03
K04D7.3	1452	2540	1996	2663	3.56E-05	3.21E-05	2.20E-05	4.17E-05
K04D7.4	42	46	46	53	3.15E-05	2.87E-05	2.00E-05	3.98E-05

K04D7.6	16	17	11	6	9.90E-05	1.64E-04	8.86E-05	1.46E-04
K04E7.1	1	2	1	0	2.80E-06	2.65E-06	1.82E-06	2.25E-06
K04E7.2	1057	1115	766	1458	2.80E-06	2.65E-06	1.82E-06	2.25E-06
K04E7.3	16	20	6	21	2.80E-06	2.65E-06	1.82E-06	2.25E-06
K04F1.1	10	11	6	6	4.39E-05	4.38E-05	2.07E-05	4.87E-05
K04F1.10	6	7	7	5	2.80E-06	2.65E-06	1.82E-06	2.25E-06
K04F1.11	7	2	2	1	2.80E-06	2.65E-06	1.82E-06	2.25E-06
K04F1.12	4	3	7	6	2.80E-06	2.65E-06	1.82E-06	2.25E-06
K04F1.13	7	7	6	6	2.80E-06	2.65E-06	1.82E-06	2.25E-06
K04F1.14a	12	10	11	11	2.80E-06	2.65E-06	1.82E-06	2.25E-06
K04F1.14b	7	9	5	8	2.80E-06	2.65E-06	1.82E-06	2.25E-06
K04F1.15	4	3	6	2	2.80E-06	2.65E-06	1.82E-06	2.25E-06
K04F1.16	4	4	9	6	2.80E-06	2.65E-06	1.82E-06	2.25E-06
K04F1.2	4	13	6	4	2.80E-06	2.65E-06	1.82E-06	2.25E-06
K04F1.3	10	6	11	1	2.80E-06	2.65E-06	1.82E-06	2.25E-06
K04F1.4	4	13	8	6	2.80E-06	2.65E-06	1.82E-06	2.25E-06
K04F1.5	3	5	20	1	2.80E-06	2.65E-06	1.82E-06	2.25E-06
K04F1.6	4	9	12	8	2.80E-06	2.65E-06	1.82E-06	2.25E-06
K04F1.7	3	3	10	5	2.80E-06	2.65E-06	1.82E-06	2.25E-06
K04F1.8	3	2	0	0	2.80E-06	2.65E-06	1.82E-06	2.25E-06
K04F1.9	24	74	34	44	2.80E-06	2.65E-06	1.82E-06	2.25E-06
K04F10.2	10	17	19	7	2.80E-06	2.65E-06	1.82E-06	2.25E-06
K04F10.3	386	355	401	519	6.41E-06	1.86E-05	5.90E-06	9.42E-06
K04F10.4a.1	789	985	1123	1485	2.80E-06	2.65E-06	1.82E-06	2.25E-06
K04F10.4a.2	761	959	1099	1445	3.79E-05	3.29E-05	2.56E-05	4.09E-05
K04F10.4c	879	1098	1231	1592	3.80E-05	4.48E-05	3.52E-05	5.75E-05
K04F10.4d	1031	1271	1442	1801	3.70E-05	4.41E-05	3.48E-05	5.65E-05
K04F10.4e	778	980	1117	1468	3.73E-05	4.40E-05	3.40E-05	5.42E-05
K04F10.4f	781	990	1133	1474	3.60E-05	4.19E-05	3.28E-05	5.05E-05
K04F10.6a	355	394	444	670	3.86E-05	4.59E-05	3.61E-05	5.85E-05
K04F10.6b	352	390	422	651	3.19E-05	3.82E-05	3.02E-05	4.84E-05
K04F10.7	343	361	345	434	2.79E-05	2.93E-05	2.27E-05	4.23E-05
K04G11.1	10	5	9	11	2.65E-05	2.78E-05	2.07E-05	3.94E-05
K04G11.2	20	22	40	30	2.65E-05	2.63E-05	1.73E-05	2.69E-05
K04G11.3	18	19	79	30	2.80E-06	2.65E-06	1.82E-06	2.25E-06
K04G11.4	18	27	24	42	2.80E-06	2.65E-06	1.82E-06	2.25E-06
K04G11.5	24	38	21	42	2.80E-06	2.65E-06	5.19E-06	2.43E-06
K04G2.1.1	862	1097	731	977	2.80E-06	2.65E-06	1.82E-06	2.92E-06
K04G2.1.2	697	932	586	814	2.80E-06	2.65E-06	1.82E-06	2.34E-06
K04G2.10	79	81	71	59	8.80E-05	1.06E-04	4.86E-05	8.01E-05
K04G2.11	36	57	47	42	9.97E-05	1.26E-04	5.46E-05	9.35E-05
K04G2.2	777	638	1261	1403	8.96E-06	8.70E-06	5.25E-06	5.38E-06
K04G2.3	237	274	295	429	8.57E-06	1.28E-05	7.27E-06	8.03E-06
K04G2.4	87	92	37	49	4.98E-05	3.87E-05	5.26E-05	7.23E-05
K04G2.6.1	605	554	991	1172	1.18E-05	1.28E-05	9.51E-06	1.71E-05
K04G2.6.2	557	519	917	1114	1.27E-05	1.27E-05	3.52E-06	5.76E-06
K04G2.7	6	11	5	5	3.02E-05	2.62E-05	3.23E-05	4.71E-05
K04G2.8a	763	732	1303	1816	2.99E-05	2.63E-05	3.20E-05	4.80E-05
K04G2.9	119	167	99	109	2.80E-06	2.65E-06	1.82E-06	2.25E-06
K04G7.1	387	443	677	794	2.35E-05	2.13E-05	2.61E-05	4.48E-05
K04G7.10	322	327	174	279	2.13E-05	2.82E-05	1.15E-05	1.56E-05
K04G7.11	368	398	353	440	2.39E-05	2.58E-05	2.72E-05	3.94E-05
K04G7.3a	945	1318	1234	1964	2.64E-05	2.53E-05	9.29E-06	1.84E-05
K04G7.3b	692	967	947	1508	4.77E-05	4.87E-05	2.97E-05	4.58E-05
K04G7.4a.1	1439	2044	1556	1854	2.57E-05	3.39E-05	2.18E-05	4.29E-05
K04G7.4a.2	1335	1913	1498	1762	1.90E-05	2.51E-05	1.69E-05	3.32E-05
K04G7.4a.3	1258	1790	1301	1659	9.85E-05	1.32E-04	6.93E-05	1.02E-04
K04G7.4b.1	1374	1935	1267	1699	9.72E-05	1.32E-04	7.10E-05	1.03E-04
K04G7.4b.2	1403	1974	1418	1764	9.99E-05	1.34E-04	6.73E-05	1.06E-04

K04H4.1a	2893	3758	3791	5366	9.58E-05	1.27E-04	5.75E-05	9.52E-05
K04H4.1b	2759	3556	3444	4917	9.77E-05	1.30E-04	6.43E-05	9.87E-05
K04H4.2a.1	375	436	124	155	6.08E-05	7.46E-05	5.19E-05	9.06E-05
K04H4.2a.2	374	436	124	155	6.39E-05	7.78E-05	5.19E-05	9.15E-05
K04H4.2a.3	299	347	94	110	1.74E-05	1.91E-05	3.74E-06	5.76E-06
K04H4.2b	322	371	97	117	1.73E-05	1.91E-05	3.74E-06	5.76E-06
K04H4.2c.1	399	471	130	172	1.67E-05	1.83E-05	3.43E-06	4.95E-06
K04H4.2c.2	401	473	133	173	1.45E-05	1.58E-05	2.84E-06	4.23E-06
K04H4.5	57	111	57	40	1.31E-05	1.46E-05	2.77E-06	4.52E-06
K04H4.6a	56	35	35	35	1.31E-05	1.46E-05	2.82E-06	4.54E-06
K04H4.6b	59	35	39	36	8.46E-06	1.55E-05	5.50E-06	4.77E-06
K04H4.7	23	10	13	11	5.60E-06	3.31E-06	2.28E-06	2.81E-06
K04H8.1	0	2	2	1	5.63E-06	3.15E-06	2.42E-06	2.74E-06
K04H8.2	1	3	8	6	4.14E-06	2.65E-06	1.82E-06	2.25E-06
K04H8.3	3	5	4	2	2.80E-06	2.65E-06	1.82E-06	2.25E-06
K05B2.2a	91	82	107	110	2.80E-06	2.65E-06	1.82E-06	2.25E-06
K05B2.2b	35	40	50	51	2.80E-06	2.65E-06	1.82E-06	2.25E-06
K05B2.4	18	34	18	11	7.95E-06	6.77E-06	6.09E-06	7.71E-06
K05B2.5a.1	531	618	510	549	6.94E-06	7.51E-06	6.47E-06	8.14E-06
K05B2.5a.2	401	449	409	451	2.80E-06	2.88E-06	1.82E-06	2.25E-06
K05B2.5b	410	459	420	453	2.10E-05	2.30E-05	1.31E-05	1.74E-05
K05B2.5c	521	610	503	543	2.12E-05	2.24E-05	1.40E-05	1.91E-05
K05C4.1.1	840	1009	1244	1200	1.97E-05	2.08E-05	1.31E-05	1.75E-05
K05C4.1.2	672	778	909	971	1.62E-05	1.79E-05	1.02E-05	1.36E-05
K05C4.10	4	2	4	4	9.01E-05	1.02E-04	8.68E-05	1.03E-04
K05C4.11	16	25	17	13	8.65E-05	9.46E-05	7.61E-05	1.00E-04
K05C4.2.1	391	484	765	568	2.80E-06	2.65E-06	1.82E-06	2.25E-06
K05C4.2.2	426	532	847	591	2.80E-06	2.65E-06	1.82E-06	2.25E-06
K05C4.3	10	9	18	10	6.04E-05	7.06E-05	7.69E-05	7.05E-05
K05C4.4	46	37	45	52	8.01E-05	9.44E-05	1.04E-04	8.92E-05
K05C4.6	538	530	864	1012	2.80E-06	2.65E-06	1.82E-06	2.25E-06
K05C4.7	251	274	412	505	3.98E-06	3.02E-06	2.53E-06	3.60E-06
K05C4.8	2	6	2	1	2.73E-05	2.54E-05	2.86E-05	4.13E-05
K05C4.9	21	17	11	16	2.86E-05	2.95E-05	3.06E-05	4.62E-05
K05D4.1	8	6	11	7	2.80E-06	2.65E-06	1.82E-06	2.25E-06
K05D4.2	5	2	5	1	2.80E-06	2.65E-06	1.82E-06	2.25E-06
K05D4.3	5	12	10	7	2.80E-06	2.65E-06	1.82E-06	2.25E-06
K05D4.4	13	7	8	3	2.80E-06	2.65E-06	1.82E-06	2.25E-06
K05D4.5	5	4	4	5	2.80E-06	2.65E-06	1.82E-06	2.25E-06
K05D4.6	5	3	5	5	2.80E-06	2.65E-06	1.82E-06	2.25E-06
K05D4.7	1	3	0	2	2.80E-06	2.65E-06	1.82E-06	2.25E-06
K05D4.8	3	6	1	4	2.80E-06	2.65E-06	1.82E-06	2.25E-06
K05D4.9	3	3	9	5	2.80E-06	2.65E-06	1.82E-06	2.25E-06
K05F1.1	21	22	31	15	2.80E-06	2.65E-06	1.82E-06	2.25E-06
K05F1.10	26	40	34	20	2.80E-06	2.65E-06	1.82E-06	2.25E-06
K05F1.2	1058	1149	2111	366	2.80E-06	2.65E-06	1.82E-06	2.25E-06
K05F1.3	77	172	74	50	6.89E-06	1.00E-05	5.87E-06	4.25E-06
K05F1.5	49	55	40	33	2.68E-04	2.75E-04	3.48E-04	7.45E-05
K05F1.6a	52	82	69	89	6.72E-06	1.42E-05	4.21E-06	3.51E-06
K05F1.6b	26	50	40	56	3.58E-06	3.78E-06	1.90E-06	2.25E-06
K05F1.7	252	308	370	64	2.80E-06	2.96E-06	1.82E-06	2.74E-06
K05F1.8	8	10	6	0	2.80E-06	2.80E-06	1.82E-06	2.68E-06
K05F1.9	96	147	37	43	7.35E-05	8.49E-05	7.02E-05	1.50E-05
K05F6.1	13	8	8	4	3.78E-06	4.47E-06	1.84E-06	2.25E-06
K05F6.10	7	4	3	1	1.54E-05	2.23E-05	3.86E-06	5.53E-06
K05F6.11	73	66	92	38	2.80E-06	2.65E-06	1.82E-06	2.25E-06
K05F6.2	4	4	8	5	2.80E-06	2.65E-06	1.82E-06	2.25E-06
K05F6.3	6	7	4	10	7.92E-06	6.77E-06	6.51E-06	3.31E-06
K05F6.4	10	11	11	11	2.80E-06	2.65E-06	1.82E-06	2.25E-06

K05F6.5	3	4	6	6	2.80E-06	2.65E-06	1.82E-06	2.25E-06
K05F6.6	5	9	4	3	2.80E-06	2.65E-06	1.82E-06	2.25E-06
K05F6.7	11	12	14	2	2.80E-06	2.65E-06	1.82E-06	2.25E-06
K05F6.8	1	2	1	0	2.80E-06	2.65E-06	1.82E-06	2.25E-06
K05F6.9	6	1	13	5	2.80E-06	2.65E-06	1.82E-06	2.25E-06
K05G3.1	4	3	3	0	2.80E-06	2.65E-06	1.82E-06	2.25E-06
K05G3.2	2	5	2	0	2.80E-06	2.65E-06	1.82E-06	2.25E-06
K05G3.3	260	382	352	532	2.80E-06	2.65E-06	1.82E-06	2.25E-06
K06A1.1	8	7	14	6	2.80E-06	2.65E-06	1.82E-06	2.25E-06
K06A1.2	27	28	24	23	2.81E-05	3.90E-05	2.48E-05	4.62E-05
K06A1.3	51	47	37	88	2.80E-06	2.65E-06	1.82E-06	2.25E-06
K06A1.5.1	119	186	108	184	2.80E-06	2.65E-06	1.82E-06	2.25E-06
K06A1.5.2	105	167	94	173	3.19E-06	2.78E-06	1.82E-06	4.43E-06
K06A1.6	58	63	68	110	7.62E-06	1.13E-05	4.50E-06	9.47E-06
K06A4.1	28	40	41	96	7.31E-06	1.10E-05	4.26E-06	9.67E-06
K06A4.2	6	7	5	1	2.80E-06	2.65E-06	1.82E-06	3.15E-06
K06A4.4	15	25	15	13	3.56E-06	4.81E-06	3.41E-06	9.83E-06
K06A4.5	122	245	99	176	2.80E-06	2.65E-06	1.82E-06	2.25E-06
K06A4.6	3	2	1	2	2.80E-06	3.68E-06	1.82E-06	2.25E-06
K06A4.8	10	19	8	11	1.62E-05	3.06E-05	8.53E-06	1.87E-05
K06A4.t1	1	0	0	0	2.80E-06	2.65E-06	1.82E-06	2.25E-06
K06A5.1	463	468	596	859	2.80E-06	2.75E-06	1.82E-06	2.25E-06
K06A5.2	113	224	77	89	2.80E-06	2.65E-06	1.82E-06	2.25E-06
K06A5.3	21	37	13	5	2.53E-05	2.42E-05	2.12E-05	3.77E-05
K06A5.4	603	671	698	1067	1.22E-05	2.28E-05	5.39E-06	7.69E-06
K06A5.6.1	944	1067	1261	1481	5.01E-06	8.36E-06	2.02E-06	2.25E-06
K06A5.6.2	803	919	1125	1356	2.25E-05	2.37E-05	1.70E-05	3.20E-05
K06A5.7.1	1472	1285	2950	3324	7.77E-05	8.30E-05	6.76E-05	9.80E-05
K06A5.7.2	1036	1048	2213	2658	6.41E-05	6.94E-05	5.85E-05	8.70E-05
K06A5.8a.1	18	27	22	28	7.20E-05	5.94E-05	9.39E-05	1.31E-04
K06A5.8a.2	17	21	17	27	6.32E-05	6.04E-05	8.79E-05	1.30E-04
K06A5.8b	18	25	18	27	2.80E-06	2.65E-06	1.82E-06	2.25E-06
K06A5.8d	86	95	110	151	2.80E-06	2.65E-06	1.82E-06	2.25E-06
K06A9.1a	121	91	89	84	2.80E-06	2.65E-06	1.82E-06	2.25E-06
K06A9.1b	207	217	177	148	3.72E-06	3.89E-06	3.12E-06	5.26E-06
K06A9.1c	102	85	73	69	4.37E-06	3.09E-06	2.10E-06	2.43E-06
K06A9.2	20	45	14	14	3.28E-06	3.23E-06	1.82E-06	2.25E-06
K06A9.3	3	7	7	5	4.62E-06	3.62E-06	2.15E-06	2.50E-06
K06B4.1	11	13	10	15	2.80E-06	2.65E-06	1.82E-06	2.25E-06
K06B4.10	4	7	3	3	2.80E-06	2.65E-06	1.82E-06	2.25E-06
K06B4.11	10	23	13	13	2.80E-06	2.65E-06	1.82E-06	2.25E-06
K06B4.12	44	59	47	59	2.80E-06	2.65E-06	1.82E-06	2.25E-06
K06B4.13	2	6	5	2	2.80E-06	2.65E-06	1.82E-06	2.25E-06
K06B4.2	2	5	4	7	2.80E-06	2.86E-06	1.82E-06	2.43E-06
K06B4.3	7	1	2	0	2.80E-06	2.65E-06	1.82E-06	2.25E-06
K06B4.4	10	18	4	12	2.80E-06	2.65E-06	1.82E-06	2.25E-06
K06B4.5	2	1	3	1	3.11E-06	2.65E-06	1.82E-06	2.25E-06
K06B4.6	3	3	7	5	2.80E-06	2.65E-06	1.82E-06	2.25E-06
K06B4.7	1	2	6	5	2.80E-06	2.65E-06	1.82E-06	2.25E-06
K06B4.8	10	6	7	3	2.80E-06	2.65E-06	1.82E-06	2.25E-06
K06B4.9	2	3	5	2	2.80E-06	2.65E-06	1.82E-06	2.25E-06
K06B9.1	10	13	17	8	2.80E-06	2.65E-06	1.82E-06	2.25E-06
K06B9.2	521	485	646	917	2.80E-06	2.65E-06	1.82E-06	2.25E-06
K06B9.4	145	160	362	405	2.80E-06	2.65E-06	1.82E-06	2.25E-06
K06B9.5	6	6	3	4	7.26E-05	6.38E-05	5.86E-05	1.03E-04
K06B9.6	11	15	22	13	1.25E-05	1.31E-05	2.04E-05	2.81E-05
K06C4.1	5	5	4	0	2.80E-06	2.65E-06	1.82E-06	2.25E-06
K06C4.10	15	46	224	38	2.80E-06	3.15E-06	3.17E-06	2.32E-06
K06C4.11	41	76	29	11	2.80E-06	2.65E-06	1.82E-06	2.25E-06

K06C4.12	16	43	10	12	5.38E-06	1.56E-05	5.23E-05	1.10E-05
K06C4.13	27	31	34	22	1.20E-05	2.09E-05	5.50E-06	2.59E-06
K06C4.15	7	5	5	2	4.82E-06	1.22E-05	1.97E-06	2.90E-06
K06C4.17	16	18	21	16	7.36E-06	7.99E-06	6.03E-06	4.81E-06
K06C4.2	9	19	34	10	2.80E-06	2.65E-06	1.82E-06	2.25E-06
K06C4.3	41	76	29	11	2.80E-06	2.65E-06	1.82E-06	2.25E-06
K06C4.4	16	43	10	12	3.22E-06	6.45E-06	7.94E-06	2.88E-06
K06C4.5	38	36	43	28	1.20E-05	2.09E-05	5.50E-06	2.59E-06
K06C4.6a	15	22	20	18	4.82E-06	1.22E-05	1.97E-06	2.90E-06
K06C4.6b	15	22	20	18	9.10E-06	8.15E-06	6.71E-06	5.38E-06
K06C4.6c.1	14	22	19	17	2.80E-06	2.65E-06	1.82E-06	2.25E-06
K06C4.6c.2	12	18	18	17	2.80E-06	2.65E-06	1.82E-06	2.25E-06
K06C4.7	1	4	5	2	2.80E-06	2.65E-06	1.82E-06	2.25E-06
K06C4.8	22	31	13	20	2.80E-06	2.65E-06	1.82E-06	2.25E-06
K06C4.9	22	19	21	15	2.80E-06	2.65E-06	1.82E-06	2.25E-06
K06G5.1a.1	1731	2070	2276	2034	2.80E-06	3.07E-06	1.82E-06	2.25E-06
K06G5.1a.2	1625	1952	2135	1989	2.80E-06	2.65E-06	1.82E-06	2.25E-06
K06G5.2	18	24	27	23	1.16E-04	1.32E-04	9.96E-05	1.10E-04
K06G5.3	9	3	0	0	1.19E-04	1.35E-04	1.02E-04	1.17E-04
K06H6.1	5	5	4	4	2.80E-06	2.65E-06	1.82E-06	2.25E-06
K06H6.2	4	5	16	6	2.80E-06	2.65E-06	1.82E-06	2.25E-06
K06H6.3	2	4	14	4	2.80E-06	2.65E-06	1.82E-06	2.25E-06
K06H6.4	3	5	9	5	2.80E-06	2.65E-06	1.82E-06	2.25E-06
K06H6.5	0	5	2	5	2.80E-06	2.65E-06	1.82E-06	2.25E-06
K06H6.6	4	1	9	4	2.80E-06	2.65E-06	1.82E-06	2.25E-06
K06H7.1	7	7	18	9	2.80E-06	2.65E-06	1.82E-06	2.25E-06
K06H7.2	37	47	39	56	2.80E-06	2.65E-06	1.82E-06	2.25E-06
K06H7.3.1	364	459	364	580	2.80E-06	2.65E-06	1.82E-06	2.25E-06
K06H7.3.2	345	430	318	536	3.39E-06	4.07E-06	2.33E-06	4.12E-06
K06H7.3.3	347	433	318	536	2.01E-05	2.39E-05	1.31E-05	2.57E-05
K06H7.4.1	413	477	591	679	2.08E-05	2.45E-05	1.25E-05	2.60E-05
K06H7.4.2	322	372	454	574	2.09E-05	2.46E-05	1.24E-05	2.59E-05
K06H7.6	404	424	536	840	2.89E-05	3.15E-05	2.69E-05	3.81E-05
K06H7.7	113	123	125	193	2.94E-05	3.21E-05	2.69E-05	4.21E-05
K06H7.8	22	32	7	6	2.03E-05	2.01E-05	1.75E-05	3.39E-05
K06H7.9	148	185	147	148	1.30E-05	1.34E-05	9.40E-06	1.79E-05
K07A1.1	278	308	302	383	2.80E-06	3.25E-06	1.82E-06	2.25E-06
K07A1.10	260	357	249	305	1.98E-05	2.34E-05	1.28E-05	1.59E-05
K07A1.11	413	457	592	787	2.82E-05	2.95E-05	1.99E-05	3.12E-05
K07A1.12.1	1141	1250	1700	1586	4.37E-05	5.67E-05	2.72E-05	4.12E-05
K07A1.12.2	1074	1186	1559	1527	3.49E-05	3.65E-05	3.25E-05	5.34E-05
K07A1.13	37	44	38	46	8.90E-05	9.22E-05	8.64E-05	9.94E-05
K07A1.14	3	1	0	0	8.31E-05	8.67E-05	7.85E-05	9.49E-05
K07A1.15	103	112	149	180	7.31E-06	8.20E-06	4.88E-06	7.31E-06
K07A1.16	28	41	20	27	2.80E-06	2.65E-06	1.82E-06	2.25E-06
K07A1.2.1	910	1057	1821	1889	3.13E-05	3.21E-05	2.94E-05	4.39E-05
K07A1.2.2	845	997	1671	1805	4.26E-06	5.90E-06	1.99E-06	3.31E-06
K07A1.3	3	23	7	18	6.38E-05	7.00E-05	8.31E-05	1.06E-04
K07A1.4	31	54	33	10	6.10E-05	6.80E-05	7.85E-05	1.05E-04
K07A1.5	31	51	14	13	2.80E-06	5.34E-06	1.82E-06	3.55E-06
K07A1.6	119	261	242	177	3.92E-06	6.43E-06	2.71E-06	2.25E-06
K07A1.7	1	5	0	2	3.78E-06	5.85E-06	1.82E-06	2.25E-06
K07A1.8.1	1731	1494	1604	2018	3.36E-05	6.96E-05	4.44E-05	4.01E-05
K07A1.8.2	1346	1151	1218	1554	2.80E-06	2.65E-06	1.82E-06	2.25E-06
K07A1.9a	257	308	241	329	1.06E-04	8.62E-05	6.37E-05	9.89E-05
K07A1.9b	239	282	216	293	1.02E-04	8.22E-05	5.99E-05	9.44E-05
K07A1.9c	239	282	231	315	1.82E-05	2.06E-05	1.11E-05	1.87E-05
K07A12.1	618	794	539	849	1.94E-05	2.16E-05	1.14E-05	1.91E-05
K07A12.2	3098	2573	4601	5689	1.64E-05	1.82E-05	1.03E-05	1.73E-05

K07A12.3.1	681	724	1329	563	3.40E-05	4.13E-05	1.93E-05	3.76E-05
K07A12.3.2	633	644	961	517	1.03E-04	8.08E-05	9.95E-05	1.52E-04
K07A12.4a	252	306	328	457	1.61E-04	1.61E-04	2.04E-04	1.07E-04
K07A12.4b	248	313	324	443	1.49E-04	1.43E-04	1.47E-04	9.75E-05
K07A12.5	22	49	32	14	1.54E-05	1.77E-05	1.30E-05	2.24E-05
K07A12.6	8	19	7	4	1.43E-05	1.71E-05	1.22E-05	2.06E-05
K07A12.7	392	414	290	437	2.80E-06	4.73E-06	2.13E-06	2.25E-06
K07A3.1.1	638	851	897	1010	2.80E-06	3.57E-06	1.82E-06	2.25E-06
K07A3.1.2	529	712	724	878	4.42E-05	4.41E-05	2.13E-05	3.96E-05
K07A3.1.3	629	848	886	1005	5.53E-05	6.97E-05	5.06E-05	7.03E-05
K07A3.2a	75	92	43	70	5.76E-05	7.33E-05	5.13E-05	7.68E-05
K07A3.2b	29	44	13	31	5.78E-05	7.37E-05	5.30E-05	7.42E-05
K07A3.3a	17	35	12	16	2.94E-06	3.41E-06	1.82E-06	2.25E-06
K07A3.3b	18	36	12	18	3.08E-06	4.42E-06	1.82E-06	2.65E-06
K07A9.3	65	86	151	152	2.80E-06	2.65E-06	1.82E-06	2.25E-06
K07A9.4	16	16	24	18	2.80E-06	2.65E-06	1.82E-06	2.25E-06
K07B1.1	5	12	9	7	1.28E-05	1.60E-05	1.93E-05	2.40E-05
K07B1.2	207	234	339	371	2.80E-06	2.65E-06	2.68E-06	2.47E-06
K07B1.3	121	147	152	169	2.80E-06	2.65E-06	1.82E-06	2.25E-06
K07B1.4a	139	200	182	197	1.69E-05	1.81E-05	1.80E-05	2.43E-05
K07B1.4b.1	127	176	160	180	1.12E-05	1.29E-05	9.17E-06	1.26E-05
K07B1.5a.1	404	522	493	745	1.20E-05	1.63E-05	1.03E-05	1.37E-05
K07B1.5a.2	352	428	405	621	1.30E-05	1.71E-05	1.07E-05	1.49E-05
K07B1.5b	342	418	393	602	2.94E-05	3.59E-05	2.33E-05	4.35E-05
K07B1.6b	293	485	238	326	3.07E-05	3.53E-05	2.30E-05	4.35E-05
K07B1.7a	128	143	85	140	2.90E-05	3.35E-05	2.17E-05	4.10E-05
K07B1.7b	186	206	124	210	2.82E-05	4.40E-05	1.49E-05	2.52E-05
K07B1.8	84	408	62	161	1.35E-05	1.42E-05	5.83E-06	1.18E-05
K07C10.1	69	88	119	204	1.32E-05	1.38E-05	5.72E-06	1.20E-05
K07C10.t1	0	0	3	0	7.84E-06	3.59E-05	3.75E-06	1.21E-05
K07C11.1	6	2	5	2	3.02E-06	3.65E-06	3.39E-06	7.17E-06
K07C11.10	7	4	0	0	2.80E-06	2.65E-06	2.99E-06	2.25E-06
K07C11.2.1	1395	1361	1649	2054	2.80E-06	2.65E-06	1.82E-06	2.25E-06
K07C11.2.2	973	999	1215	1531	2.80E-06	2.65E-06	1.82E-06	2.25E-06
K07C11.3	83	177	99	106	1.12E-04	1.03E-04	8.58E-05	1.32E-04
K07C11.4	135	129	105	158	1.10E-04	1.06E-04	8.91E-05	1.39E-04
K07C11.7a	249	549	324	1018	1.68E-05	3.39E-05	1.31E-05	1.73E-05
K07C11.7b.1	169	360	196	685	7.56E-06	6.82E-06	3.83E-06	7.11E-06
K07C11.7b.2	173	362	202	692	2.09E-05	4.35E-05	1.77E-05	6.86E-05
K07C11.8.1	14	54	18	21	2.27E-05	4.57E-05	1.71E-05	7.40E-05
K07C11.8.2	16	48	14	19	2.22E-05	4.38E-05	1.68E-05	7.12E-05
K07C11.9	345	520	365	579	2.91E-06	1.06E-05	2.42E-06	3.49E-06
K07C5.1	798	883	1064	1409	3.11E-06	8.83E-06	1.82E-06	2.97E-06
K07C5.10	1	1	0	2	1.74E-05	2.48E-05	1.20E-05	2.35E-05
K07C5.2	160	230	201	197	5.94E-05	6.21E-05	5.16E-05	8.43E-05
K07C5.3	286	360	263	378	2.80E-06	2.65E-06	1.82E-06	2.25E-06
K07C5.4	4335	3081	3999	5643	1.81E-05	2.45E-05	1.48E-05	1.79E-05
K07C5.5	54	88	43	51	2.00E-05	2.38E-05	1.20E-05	2.13E-05
K07C5.6.1	710	706	441	905	3.02E-04	2.03E-04	1.81E-04	3.16E-04
K07C5.6.2	656	657	418	880	5.29E-06	8.17E-06	2.75E-06	4.03E-06
K07C5.7	40	92	43	50	3.94E-05	3.70E-05	1.59E-05	4.03E-05
K07C5.8	1096	1076	2273	2759	3.76E-05	3.56E-05	1.56E-05	4.05E-05
K07C5.9	8	10	14	10	2.80E-06	5.90E-06	1.90E-06	2.72E-06
K07C6.1	10	7	11	7	4.27E-05	3.96E-05	5.76E-05	8.63E-05
K07C6.10	3	4	3	2	2.80E-06	2.75E-06	2.64E-06	2.34E-06
K07C6.11	3	10	5	3	2.80E-06	2.65E-06	1.82E-06	2.25E-06
K07C6.13	3	4	5	1	2.80E-06	2.65E-06	1.82E-06	2.25E-06
K07C6.15	6	13	22	21	2.80E-06	2.65E-06	1.82E-06	2.25E-06
K07C6.2	5	5	4	2	2.80E-06	2.65E-06	1.82E-06	2.25E-06

K07C6.3	9	15	6	4	2.80E-06	2.65E-06	1.82E-06	2.25E-06
K07C6.4	17	21	22	5	2.80E-06	2.65E-06	1.82E-06	2.25E-06
K07C6.5	87	64	111	53	2.80E-06	2.65E-06	1.82E-06	2.25E-06
K07C6.6	2	3	2	4	2.80E-06	2.65E-06	1.82E-06	2.25E-06
K07C6.7	2	5	2	1	6.55E-06	4.55E-06	5.45E-06	3.22E-06
K07C6.8	2	7	1	2	2.80E-06	2.65E-06	1.82E-06	2.25E-06
K07C6.9	3	3	5	3	2.80E-06	2.65E-06	1.82E-06	2.25E-06
K07D4.1	19	20	30	32	2.80E-06	2.65E-06	1.82E-06	2.25E-06
K07D4.2	2	3	3	1	2.80E-06	2.65E-06	1.82E-06	2.25E-06
K07D4.3.1	940	844	1246	1351	4.65E-06	4.60E-06	4.76E-06	6.27E-06
K07D4.3.2	790	704	976	1092	2.80E-06	2.65E-06	1.82E-06	2.25E-06
K07D4.4	7	7	10	4	9.50E-05	8.06E-05	8.20E-05	1.10E-04
K07D4.5	5	2	5	0	9.36E-05	7.88E-05	7.53E-05	1.04E-04
K07D4.6	9	9	6	5	2.80E-06	2.65E-06	1.82E-06	2.25E-06
K07D4.7a	103	194	123	197	2.80E-06	2.65E-06	1.82E-06	2.25E-06
K07D4.7b	70	125	69	122	2.80E-06	2.65E-06	1.82E-06	2.25E-06
K07D4.8	84	84	70	55	3.08E-06	5.48E-06	2.39E-06	4.72E-06
K07D4.9	16	27	51	11	3.78E-06	6.37E-06	2.42E-06	5.29E-06
K07D8.1.1	1808	2543	1382	2758	8.85E-06	8.36E-06	4.79E-06	4.66E-06
K07D8.1.2	1816	2556	1393	2770	4.26E-06	6.80E-06	8.84E-06	2.36E-06
K07E1.1	199	366	101	182	3.02E-05	4.01E-05	1.50E-05	3.70E-05
K07E12.1a	1790	2134	1872	2367	3.07E-05	4.08E-05	1.53E-05	3.76E-05
K07E12.1b	1420	1681	1576	1854	2.34E-05	4.07E-05	7.74E-06	1.72E-05
K07E12.2	54	41	60	8	5.04E-06	5.69E-06	3.43E-06	5.35E-06
K07E3.1	232	262	223	347	4.31E-06	4.84E-06	3.12E-06	4.52E-06
K07E3.2	79	82	65	82	2.19E-05	1.57E-05	1.59E-05	2.61E-06
K07E3.3	129	277	125	211	8.15E-06	8.70E-06	5.10E-06	9.81E-06
K07E3.4b	452	585	427	573	3.58E-06	3.52E-06	1.91E-06	2.99E-06
K07E3.7a.1	194	187	167	177	1.31E-05	2.66E-05	8.25E-06	1.72E-05
K07E3.7a.2	196	188	168	178	2.48E-05	3.04E-05	1.53E-05	2.53E-05
K07E3.7a.3	168	168	144	157	5.29E-06	4.81E-06	2.95E-06	3.87E-06
K07E3.7b	165	160	141	152	5.21E-06	4.73E-06	2.92E-06	3.80E-06
K07E3.8a	101	156	119	92	5.10E-06	4.81E-06	2.84E-06	3.82E-06
K07E8.1	1	3	0	0	5.12E-06	4.68E-06	2.84E-06	3.78E-06
K07E8.10	3	4	1	3	1.20E-05	1.75E-05	9.18E-06	8.77E-06
K07E8.11	1	4	1	4	2.80E-06	2.65E-06	1.82E-06	2.25E-06
K07E8.12	2	3	1	0	2.80E-06	2.65E-06	1.82E-06	2.25E-06
K07E8.2	6	8	21	10	2.80E-06	2.65E-06	1.82E-06	2.25E-06
K07E8.4	8	7	5	2	2.80E-06	2.65E-06	1.82E-06	2.25E-06
K07E8.5	5	5	8	7	2.80E-06	2.65E-06	1.82E-06	2.25E-06
K07E8.6	10	7	5	9	2.80E-06	2.65E-06	1.82E-06	2.25E-06
K07E8.7	86	73	118	133	2.80E-06	2.65E-06	1.82E-06	2.25E-06
K07E8.8	1	1	2	2	2.80E-06	2.65E-06	1.82E-06	2.25E-06
K07E8.9	3	4	2	2	7.34E-06	5.87E-06	6.54E-06	9.11E-06
K07F5.1	1800	2230	3609	740	2.80E-06	2.65E-06	1.82E-06	2.25E-06
K07F5.11	97	140	68	43	2.80E-06	2.65E-06	1.82E-06	2.25E-06
K07F5.12	64	80	65	84	4.73E-04	5.54E-04	6.17E-04	1.56E-04
K07F5.13a	347	370	523	804	1.22E-05	1.66E-05	5.56E-06	4.34E-06
K07F5.13b.1	341	383	534	781	6.78E-06	8.01E-06	4.48E-06	7.15E-06
K07F5.13b.2	330	366	509	771	2.05E-05	2.06E-05	2.01E-05	3.81E-05
K07F5.13c	390	431	576	902	2.31E-05	2.45E-05	2.35E-05	4.24E-05
K07F5.14	295	341	181	280	2.23E-05	2.34E-05	2.24E-05	4.19E-05
K07F5.15.1	333	448	578	261	1.91E-05	1.99E-05	1.84E-05	3.55E-05
K07F5.15.2	245	345	385	188	2.54E-05	2.77E-05	1.01E-05	1.94E-05
K07F5.15.3	295	409	418	220	6.34E-05	8.06E-05	7.16E-05	3.99E-05
K07F5.16	11	8	15	6	6.74E-05	8.97E-05	6.89E-05	4.16E-05
K07F5.2	2126	2744	3513	837	5.75E-05	7.53E-05	5.30E-05	3.44E-05
K07F5.3	2344	3052	3680	839	2.80E-06	2.65E-06	2.33E-06	2.25E-06
K07F5.4a	15	23	11	5	5.49E-04	6.69E-04	5.90E-04	1.73E-04

K07F5.4b	16	22	11	5	5.99E-04	7.37E-04	6.12E-04	1.72E-04
K07F5.6	18	34	12	12	2.80E-06	2.65E-06	1.82E-06	2.25E-06
K07F5.7	0	2	1	1	2.80E-06	2.65E-06	1.82E-06	2.25E-06
K07F5.8	10	12	7	3	2.80E-06	2.65E-06	1.82E-06	2.25E-06
K07F5.9	192	277	506	77	2.80E-06	2.65E-06	1.82E-06	2.25E-06
K07G5.1	985	1047	1616	2240	2.80E-06	2.65E-06	1.82E-06	2.25E-06
K07G5.2	195	197	145	211	5.36E-05	7.31E-05	9.20E-05	1.73E-05
K07G5.3	25	28	25	28	3.15E-05	3.16E-05	3.36E-05	5.76E-05
K07G5.4	8	10	3	12	3.01E-05	2.87E-05	1.46E-05	2.62E-05
K07G5.5	56	61	42	56	2.80E-06	2.65E-06	1.82E-06	2.25E-06
K07G5.6.1	399	519	347	497	2.80E-06	2.65E-06	1.82E-06	2.25E-06
K07G5.6.2	354	469	290	452	5.99E-06	6.16E-06	2.92E-06	4.81E-06
K07G6.1	2	0	0	1	3.51E-05	4.31E-05	1.98E-05	3.51E-05
K07H8.1	455	533	677	883	3.44E-05	4.31E-05	1.83E-05	3.53E-05
K07H8.10.1	3606	3579	1574	3825	2.80E-06	2.65E-06	1.82E-06	2.25E-06
K07H8.10.2	3199	3166	1444	3539	2.40E-05	2.66E-05	2.33E-05	3.74E-05
K07H8.11	8	6	16	4	1.50E-04	1.41E-04	4.26E-05	1.28E-04
K07H8.12	9	10	6	4	1.49E-04	1.39E-04	4.37E-05	1.32E-04
K07H8.2a	780	997	1083	1465	2.80E-06	2.65E-06	1.82E-06	2.25E-06
K07H8.2b.1	681	857	1000	1335	3.70E-06	3.89E-06	1.82E-06	2.25E-06
K07H8.2c.1	659	842	938	1289	4.75E-05	5.74E-05	4.29E-05	7.17E-05
K07H8.2c.2	610	785	878	1212	4.89E-05	5.82E-05	4.68E-05	7.71E-05
K07H8.2c.3	675	856	953	1301	4.88E-05	5.89E-05	4.52E-05	7.67E-05
K07H8.3	404	463	565	516	5.07E-05	6.16E-05	4.74E-05	8.08E-05
K07H8.5	7	25	8	6	4.96E-05	5.94E-05	4.56E-05	7.68E-05
K07H8.6b	12600	10978	14190	26912	6.09E-05	6.59E-05	5.54E-05	6.25E-05
K07H8.7	6	11	2	7	2.80E-06	2.65E-06	1.82E-06	2.25E-06
K07H8.8	8	9	6	5	2.02E-03	1.66E-03	1.48E-03	3.46E-03
K07H8.9	204	169	191	293	2.80E-06	2.65E-06	1.82E-06	2.25E-06
K08A2.2	9	22	22	12	2.80E-06	2.65E-06	1.82E-06	2.25E-06
K08A2.4	45	48	82	49	2.53E-05	1.98E-05	1.54E-05	2.91E-05
K08A2.5a.1	45	70	63	77	2.80E-06	2.65E-06	1.82E-06	2.25E-06
K08A2.5a.2	45	68	65	83	4.31E-06	4.36E-06	5.12E-06	3.78E-06
K08A2.5a.3	43	61	59	77	2.80E-06	4.10E-06	2.53E-06	3.82E-06
K08A2.5b.1	31	47	42	41	2.80E-06	3.84E-06	2.51E-06	3.98E-06
K08A2.5b.2	33	47	41	40	2.80E-06	3.44E-06	2.30E-06	3.69E-06
K08A2.5c.1	21	40	33	32	2.80E-06	3.52E-06	2.17E-06	2.61E-06
K08A2.5c.2	23	38	31	31	2.80E-06	3.39E-06	2.02E-06	2.45E-06
K08A8.1a	353	404	415	468	2.94E-06	5.29E-06	3.01E-06	3.60E-06
K08A8.1b	201	250	266	272	3.22E-06	5.03E-06	2.82E-06	3.49E-06
K08A8.2a.1	83	121	56	134	2.69E-05	2.90E-05	2.05E-05	2.86E-05
K08A8.2a.2	36	60	28	61	2.47E-05	2.90E-05	2.13E-05	2.69E-05
K08A8.2b.1	22	49	20	50	4.98E-06	6.85E-06	2.19E-06	6.45E-06
K08A8.2b.2	19	45	17	51	4.54E-06	7.14E-06	2.30E-06	6.16E-06
K08A8.2b.3	36	60	28	61	3.64E-06	7.64E-06	2.15E-06	6.63E-06
K08A8.3	230	202	255	366	2.80E-06	5.50E-06	1.82E-06	5.31E-06
K08B12.1	96	186	70	32	4.54E-06	7.14E-06	2.30E-06	6.16E-06
K08B12.2a	347	354	330	418	8.51E-06	7.06E-06	6.14E-06	1.09E-05
K08B12.2b	58	73	58	80	9.35E-06	1.71E-05	4.45E-06	2.50E-06
K08B12.3	21	39	22	15	1.78E-05	1.71E-05	1.10E-05	1.72E-05
K08B12.4	6	6	3	3	1.09E-05	1.29E-05	7.09E-06	1.21E-05
K08B4.1a	770	627	1092	1448	3.05E-06	5.34E-06	2.08E-06	2.25E-06
K08B4.1b	605	477	837	1083	2.80E-06	2.65E-06	1.82E-06	2.25E-06
K08B4.2	5	4	6	6	3.38E-05	2.60E-05	3.12E-05	5.11E-05
K08B4.3	48	84	60	58	3.36E-05	2.50E-05	3.02E-05	4.83E-05
K08B4.4	23	38	28	50	2.80E-06	2.65E-06	1.82E-06	2.25E-06
K08B4.5	1	7	3	3	3.36E-06	5.55E-06	2.73E-06	3.26E-06
K08B4.6	452	901	378	474	2.80E-06	2.65E-06	1.82E-06	2.83E-06
K08B4.7	29	56	22	22	2.80E-06	2.65E-06	1.82E-06	2.25E-06

K08B5.1	9	19	11	6	1.02E-04	1.92E-04	5.54E-05	8.58E-05
K08B5.2	14	28	11	14	6.19E-06	1.13E-05	3.06E-06	3.78E-06
K08C7.1	91	101	102	146	2.80E-06	2.65E-06	1.82E-06	2.25E-06
K08C7.2.1	103	188	83	69	2.80E-06	3.25E-06	1.82E-06	2.25E-06
K08C7.2.2	99	185	85	65	6.72E-06	7.06E-06	4.90E-06	8.66E-06
K08C7.3a	2174	2934	2055	3620	5.77E-06	9.95E-06	3.02E-06	3.10E-06
K08C7.3b	2150	2896	2016	3566	5.91E-06	1.04E-05	3.30E-06	3.10E-06
K08C7.4	13	8	11	3	2.17E-05	2.77E-05	1.34E-05	2.91E-05
K08C7.5	7	16	11	3	2.17E-05	2.76E-05	1.32E-05	2.89E-05
K08C7.6	52	86	56	81	2.80E-06	2.65E-06	1.82E-06	2.25E-06
K08C7.7	24	40	20	30	2.80E-06	2.65E-06	1.82E-06	2.25E-06
K08C9.1	17	18	10	10	1.10E-05	1.72E-05	7.73E-06	1.38E-05
K08C9.2	59	103	25	23	2.94E-06	4.63E-06	1.82E-06	2.97E-06
K08C9.4	165	203	73	80	2.80E-06	2.65E-06	1.82E-06	2.25E-06
K08C9.5	13	5	11	2	8.88E-06	1.46E-05	2.44E-06	2.77E-06
K08C9.6	3	1	5	2	2.04E-05	2.37E-05	5.87E-06	7.94E-06
K08C9.7	24	19	7	7	2.80E-06	2.65E-06	1.82E-06	2.25E-06
K08D10.1	395	403	581	901	2.80E-06	2.65E-06	1.82E-06	2.25E-06
K08D10.10	34	26	27	23	7.78E-06	5.82E-06	1.82E-06	2.25E-06
K08D10.11	2	3	23	4	1.65E-05	1.59E-05	1.58E-05	3.02E-05
K08D10.2	47	53	59	51	3.14E-06	2.65E-06	1.82E-06	2.25E-06
K08D10.3	313	330	556	460	2.80E-06	2.65E-06	3.63E-06	2.25E-06
K08D10.4	167	163	195	190	1.20E-05	1.28E-05	9.82E-06	1.05E-05
K08D10.5	3	7	9	8	4.59E-05	4.57E-05	5.30E-05	5.42E-05
K08D10.7	14	26	15	8	2.61E-05	2.40E-05	1.98E-05	2.38E-05
K08D10.9	8	12	10	4	2.80E-06	2.65E-06	1.82E-06	2.25E-06
K08D12.1	1180	891	1616	1128	2.80E-06	3.04E-06	1.82E-06	2.25E-06
K08D12.2	3	6	5	4	2.80E-06	2.65E-06	1.82E-06	2.25E-06
K08D12.3a.1	2260	1888	1652	2159	1.62E-04	1.16E-04	1.44E-04	1.24E-04
K08D12.3a.2	1967	1635	1348	1965	2.80E-06	2.65E-06	1.82E-06	2.25E-06
K08D12.3b	1923	1598	1339	1948	3.77E-04	2.97E-04	1.79E-04	2.89E-04
K08D12.4	0	4	1	6	3.43E-04	2.69E-04	1.53E-04	2.75E-04
K08D12.5	9	17	9	10	5.73E-04	4.50E-04	2.60E-04	4.66E-04
K08D12.7	0	0	1	0	2.80E-06	2.65E-06	1.82E-06	2.25E-06
K08D8.1	7	8	9	10	2.80E-06	2.65E-06	1.82E-06	2.25E-06
K08D8.2	0	2	11	1	2.80E-06	2.65E-06	1.82E-06	2.25E-06
K08D8.3	52	148	10	63	2.80E-06	2.65E-06	2.00E-06	2.74E-06
K08D8.4a	33	132	49	54	2.80E-06	2.65E-06	2.15E-06	2.25E-06
K08D8.4b	28	109	37	48	3.44E-06	9.26E-06	1.82E-06	3.35E-06
K08D8.4c	31	139	50	54	2.80E-06	8.25E-06	2.11E-06	2.86E-06
K08D8.4d	32	124	48	48	2.80E-06	8.38E-06	1.97E-06	3.15E-06
K08D8.5	51	54	150	21	2.80E-06	7.72E-06	1.91E-06	2.54E-06
K08D8.6	328	388	294	382	2.80E-06	8.23E-06	2.20E-06	2.72E-06
K08D9.1	3	1	3	3	5.32E-06	5.32E-06	1.02E-05	2.25E-06
K08D9.2	6	4	2	2	2.25E-05	2.51E-05	1.31E-05	2.10E-05
K08D9.3	495	393	862	840	2.80E-06	2.65E-06	1.82E-06	2.25E-06
K08D9.4	8	22	16	12	2.80E-06	2.65E-06	1.82E-06	2.25E-06
K08D9.5	4	2	3	4	2.42E-05	1.82E-05	2.74E-05	3.30E-05
K08D9.6	4	9	8	8	2.80E-06	2.65E-06	1.82E-06	2.25E-06
K08E3.1	202	445	90	205	2.80E-06	2.65E-06	1.82E-06	2.25E-06
K08E3.10	115	166	115	121	2.80E-06	2.65E-06	1.82E-06	2.25E-06
K08E3.2	30	32	18	13	1.00E-05	2.08E-05	2.90E-06	8.14E-06
K08E3.3a	201	274	354	538	2.26E-05	3.08E-05	1.47E-05	1.91E-05
K08E3.3b	184	265	338	516	4.03E-06	4.07E-06	1.82E-06	2.25E-06
K08E3.4	582	571	861	1109	1.16E-05	1.49E-05	1.32E-05	2.49E-05
K08E3.5a.1	1732	1692	2178	2976	1.08E-05	1.47E-05	1.29E-05	2.43E-05
K08E3.5a.2	1727	1679	2169	2973	3.12E-05	2.89E-05	3.00E-05	4.77E-05
K08E3.5a.3	1730	1685	2171	2973	1.24E-04	1.15E-04	1.02E-04	1.72E-04
K08E3.5b	1727	1704	2189	2984	1.26E-04	1.16E-04	1.03E-04	1.74E-04

K08E3.5c	1797	1738	2283	3043	1.26E-04	1.16E-04	1.03E-04	1.74E-04
K08E3.5d	1629	1607	2033	2836	1.18E-04	1.10E-04	9.75E-05	1.64E-04
K08E3.5e	1730	1680	2170	2973	1.30E-04	1.19E-04	1.08E-04	1.77E-04
K08E3.5f	1927	1875	2408	3227	1.20E-04	1.12E-04	9.76E-05	1.68E-04
K08E3.7.1	219	268	250	366	1.17E-04	1.07E-04	9.53E-05	1.61E-04
K08E3.7.2	197	239	229	345	1.21E-04	1.11E-04	9.84E-05	1.63E-04
K08E4.1	1163	1442	1365	2049	1.85E-05	2.13E-05	1.37E-05	2.48E-05
K08E4.2	54	62	87	68	1.88E-05	2.16E-05	1.42E-05	2.64E-05
K08E4.3	55	85	81	93	3.40E-05	3.98E-05	2.59E-05	4.81E-05
K08E4.4	1	4	3	3	7.25E-06	7.88E-06	7.62E-06	7.35E-06
K08E4.5	12	15	10	9	5.82E-06	8.49E-06	5.58E-06	7.89E-06
K08E4.6	434	469	434	502	2.80E-06	2.65E-06	1.82E-06	2.25E-06
K08E5.1	428	406	492	698	2.80E-06	2.65E-06	1.82E-06	2.25E-06
K08E5.2a	132	271	113	227	5.52E-05	5.64E-05	3.60E-05	5.13E-05
K08E5.2b.1	85	175	75	141	1.17E-05	1.05E-05	8.75E-06	1.53E-05
K08E5.2b.2	98	204	83	171	6.64E-06	1.29E-05	3.70E-06	9.18E-06
K08E5.2b.3	132	271	113	227	5.74E-06	1.11E-05	3.30E-06	7.62E-06
K08E5.3a	1398	1892	1063	1237	6.05E-06	1.19E-05	3.33E-06	8.48E-06
K08E5.3b	1349	1829	1040	1217	6.64E-06	1.29E-05	3.70E-06	9.18E-06
K08E5.4	37	50	41	51	1.36E-05	1.73E-05	6.71E-06	9.63E-06
K08E7.3	812	739	1426	1470	1.56E-05	2.00E-05	7.84E-06	1.13E-05
K08E7.4	6	5	6	5	1.39E-05	1.78E-05	1.01E-05	1.55E-05
K08E7.5a	114	156	48	44	4.03E-05	3.47E-05	4.61E-05	5.87E-05
K08E7.5b	47	77	22	20	2.80E-06	2.65E-06	1.82E-06	2.25E-06
K08E7.5c	96	123	46	43	3.47E-06	4.50E-06	1.82E-06	2.25E-06
K08E7.5d	51	83	26	21	3.56E-06	5.50E-06	1.82E-06	2.25E-06
K08E7.6	9	12	7	5	3.84E-06	4.66E-06	1.82E-06	2.25E-06
K08E7.7	38	35	21	41	3.50E-06	5.40E-06	1.82E-06	2.25E-06
K08E7.8a	24	28	101	28	2.80E-06	2.65E-06	1.82E-06	2.25E-06
K08E7.8b	16	20	88	23	2.80E-06	2.65E-06	1.82E-06	2.25E-06
K08E7.9	144	211	288	322	4.68E-06	5.13E-06	1.28E-05	4.36E-06
K08E7.t1	0	0	3	0	4.17E-06	4.92E-06	1.50E-05	4.81E-06
K08F11.1	1	6	3	3	3.95E-06	5.48E-06	5.14E-06	7.11E-06
K08F11.2	394	401	584	899	2.80E-06	2.65E-06	2.99E-06	2.25E-06
K08F11.3.1	1339	1164	1642	2032	2.80E-06	2.65E-06	1.82E-06	2.25E-06
K08F11.3.2	1378	1204	1741	2088	1.64E-05	1.57E-05	1.58E-05	3.00E-05
K08F11.3.3	1292	1102	1498	1841	1.07E-04	8.77E-05	8.52E-05	1.30E-04
K08F11.4a	155	192	196	236	1.07E-04	8.79E-05	8.76E-05	1.30E-04
K08F11.5.1	340	355	531	705	1.23E-04	9.94E-05	9.31E-05	1.41E-04
K08F11.5.2	311	330	467	650	1.25E-05	1.46E-05	1.03E-05	1.53E-05
K08F11.6	40	49	30	39	1.85E-05	1.82E-05	1.88E-05	3.08E-05
K08F4.1.1	539	622	685	1126	1.85E-05	1.86E-05	1.81E-05	3.11E-05
K08F4.1.2	529	610	663	1104	4.26E-06	4.92E-06	2.08E-06	3.33E-06
K08F4.10	85	119	441	77	2.18E-05	2.37E-05	1.80E-05	3.65E-05
K08F4.12	7	12	10	15	2.20E-05	2.40E-05	1.79E-05	3.69E-05
K08F4.2	3436	2719	3302	4809	5.26E-05	6.96E-05	1.78E-04	3.83E-05
K08F4.3	468	485	677	533	2.80E-06	4.50E-06	2.59E-06	4.79E-06
K08F4.4	35	64	65	55	2.01E-04	1.51E-04	1.26E-04	2.26E-04
K08F4.5	42	66	29	26	8.76E-05	8.58E-05	8.25E-05	8.02E-05
K08F4.6	1	8	13	11	2.80E-06	3.89E-06	2.71E-06	2.83E-06
K08F4.8	1303	1889	1594	463	5.99E-06	8.91E-06	2.70E-06	2.99E-06
K08F4.9	207	247	237	253	2.80E-06	2.65E-06	1.82E-06	2.25E-06
K08F8.1a.1	408	1468	409	892	3.28E-04	4.49E-04	2.61E-04	9.36E-05
K08F8.1a.2	406	1437	397	875	2.78E-05	3.13E-05	2.07E-05	2.73E-05
K08F8.1a.3	375	1341	340	786	2.55E-05	8.67E-05	1.66E-05	4.48E-05
K08F8.1b.1	298	1117	301	653	2.63E-05	8.80E-05	1.68E-05	4.56E-05
K08F8.1b.2	358	1293	374	788	2.22E-05	7.49E-05	1.31E-05	3.73E-05
K08F8.1c.1	375	1341	340	786	2.36E-05	8.35E-05	1.55E-05	4.15E-05
K08F8.1c.2	368	1322	338	777	2.43E-05	8.29E-05	1.65E-05	4.30E-05

K08F8.1c.3	318	1120	291	702	2.60E-05	8.77E-05	1.53E-05	4.37E-05
K08F8.1c.4	332	1154	308	714	2.55E-05	8.65E-05	1.52E-05	4.32E-05
K08F8.1d.1	357	1240	346	793	2.65E-05	8.82E-05	1.58E-05	4.70E-05
K08F8.1d.2	351	1218	340	776	2.63E-05	8.64E-05	1.59E-05	4.55E-05
K08F8.1e.1	209	709	179	471	2.30E-05	7.56E-05	1.45E-05	4.11E-05
K08F8.1e.2	265	927	228	555	2.20E-05	7.22E-05	1.39E-05	3.91E-05
K08F8.1e.3	258	908	226	546	2.41E-05	7.72E-05	1.34E-05	4.36E-05
K08F8.1e.4	208	706	179	471	2.74E-05	9.06E-05	1.54E-05	4.61E-05
K08F8.1e.5	222	740	196	483	2.32E-05	7.73E-05	1.32E-05	3.95E-05
K08F8.2	64	68	38	52	1.88E-05	6.01E-05	1.05E-05	3.41E-05
K08F8.3.1	80	121	69	94	2.56E-05	8.08E-05	1.47E-05	4.48E-05
K08F8.3.2	60	92	56	79	5.10E-06	5.11E-06	1.97E-06	3.33E-06
K08F8.4	308	570	358	506	5.38E-06	7.67E-06	3.01E-06	5.06E-06
K08F8.5a	16	24	6	9	5.10E-06	7.38E-06	3.10E-06	5.40E-06
K08F8.5b	16	25	6	9	2.21E-05	3.87E-05	1.67E-05	2.92E-05
K08F8.6	568	669	391	820	2.80E-06	2.65E-06	1.82E-06	2.25E-06
K08F8.7	12	10	4	8	2.80E-06	2.80E-06	1.82E-06	2.25E-06
K08F9.1	13	11	17	12	6.86E-06	7.62E-06	3.08E-06	7.94E-06
K08F9.2.1	582	653	1004	1367	2.80E-06	2.65E-06	1.82E-06	2.25E-06
K08F9.2.2	536	595	925	1298	2.80E-06	2.65E-06	1.82E-06	2.25E-06
K08F9.3	6	7	7	8	3.38E-05	3.58E-05	3.80E-05	6.38E-05
K08F9.4	296	286	333	530	3.15E-05	3.30E-05	3.53E-05	6.12E-05
K08G2.10	3	4	2	0	2.80E-06	2.65E-06	1.82E-06	2.25E-06
K08G2.11	6	9	13	3	1.94E-05	1.77E-05	1.42E-05	2.79E-05
K08G2.12	2	5	2	2	2.80E-06	2.65E-06	1.82E-06	2.25E-06
K08G2.13	2	2	3	2	2.80E-06	2.65E-06	1.82E-06	2.25E-06
K08G2.14	1	1	3	1	2.80E-06	2.65E-06	1.82E-06	2.25E-06
K08G2.15	1	2	1	1	2.80E-06	2.65E-06	1.82E-06	2.25E-06
K08G2.2	7	5	9	3	2.80E-06	2.65E-06	1.82E-06	2.25E-06
K08G2.3	5	7	6	7	2.80E-06	2.65E-06	1.82E-06	2.25E-06
K08G2.4	14	15	8	6	2.80E-06	2.65E-06	1.82E-06	2.25E-06
K08G2.5	3	5	2	1	2.80E-06	2.65E-06	1.82E-06	2.25E-06
K08G2.6	4	6	15	4	2.80E-06	2.65E-06	1.82E-06	2.25E-06
K08G2.7	13	9	11	9	2.80E-06	2.65E-06	1.82E-06	2.25E-06
K08G2.8	7	3	5	1	2.80E-06	2.65E-06	1.82E-06	2.25E-06
K08G2.9	4	3	1	0	2.80E-06	2.65E-06	1.82E-06	2.25E-06
K08H10.1.1	1159	2194	1787	3600	2.80E-06	2.65E-06	1.82E-06	2.25E-06
K08H10.1.2	1107	2118	1702	3499	2.80E-06	2.65E-06	1.82E-06	2.25E-06
K08H10.2b	723	855	646	1334	5.49E-05	9.82E-05	5.51E-05	1.37E-04
K08H10.3a	47	35	13	35	5.51E-05	9.96E-05	5.51E-05	1.40E-04
K08H10.3b	37	25	10	29	3.88E-05	4.34E-05	2.26E-05	5.75E-05
K08H10.4.1	721	970	1051	1139	1.45E-05	1.02E-05	2.61E-06	8.68E-06
K08H10.4.2	644	853	907	1051	1.32E-05	8.38E-06	2.31E-06	8.28E-06
K08H10.5	9	10	3	8	4.16E-05	5.29E-05	3.95E-05	5.29E-05
K08H10.6	10	14	13	9	4.36E-05	5.46E-05	4.00E-05	5.72E-05
K08H10.7	497	528	550	890	2.80E-06	2.65E-06	1.82E-06	2.25E-06
K08H10.9	173	192	184	231	2.80E-06	2.65E-06	1.82E-06	2.25E-06
K08H2.1	27	36	13	50	1.73E-05	1.74E-05	1.25E-05	2.49E-05
K08H2.2	9	13	10	11	3.32E-05	3.48E-05	2.30E-05	3.56E-05
K08H2.3	30	35	50	58	3.53E-06	4.47E-06	1.82E-06	5.26E-06
K08H2.4	4	2	6	7	2.80E-06	2.65E-06	1.82E-06	2.25E-06
K08H2.5	8	12	1	1	4.17E-06	4.60E-06	4.54E-06	6.50E-06
K08H2.6	18	34	45	38	2.80E-06	2.65E-06	1.82E-06	2.25E-06
K08H2.7	2	12	5	4	2.80E-06	2.65E-06	1.82E-06	2.25E-06
K08H2.8	72	89	52	63	2.80E-06	4.58E-06	4.15E-06	4.34E-06
K08H2.9	4	11	11	16	2.80E-06	2.96E-06	1.82E-06	2.25E-06
K08H2.t1	0	1	1	1	5.12E-06	5.98E-06	2.41E-06	3.60E-06
K09A11.1	42	47	51	63	2.80E-06	2.65E-06	1.82E-06	2.25E-06
K09A11.2	13	14	23	13	2.80E-06	2.65E-06	1.82E-06	2.25E-06

K09A11.3	21	20	35	19	2.80E-06	2.72E-06	2.04E-06	3.13E-06
K09A11.4	4	4	10	3	2.80E-06	2.65E-06	1.82E-06	2.25E-06
K09A11.5	5	5	20	7	2.80E-06	2.65E-06	1.82E-06	2.25E-06
K09A11.t1	0	0	2	0	2.80E-06	2.65E-06	1.82E-06	2.25E-06
K09A9.1	251	570	278	406	2.80E-06	2.65E-06	1.82E-06	2.25E-06
K09A9.2.1	207	232	276	299	2.80E-06	2.65E-06	2.00E-06	2.25E-06
K09A9.2.2	71	84	103	128	7.98E-06	1.71E-05	5.76E-06	1.04E-05
K09A9.3.1	668	688	786	1077	1.98E-05	2.10E-05	1.72E-05	2.30E-05
K09A9.3.2	545	530	601	899	1.22E-05	1.36E-05	1.15E-05	1.77E-05
K09A9.6	146	270	122	211	4.16E-05	4.04E-05	3.18E-05	5.38E-05
K09B11.1	53	74	119	220	4.49E-05	4.13E-05	3.22E-05	5.95E-05
K09B11.10	57	103	32	30	5.71E-06	1.00E-05	3.12E-06	6.63E-06
K09B11.11	0	2	4	1	4.06E-06	5.34E-06	5.92E-06	1.35E-05
K09B11.12	0	2	4	1	2.88E-06	4.92E-06	1.82E-06	2.25E-06
K09B11.13	0	2	4	1	2.80E-06	2.65E-06	2.86E-06	2.25E-06
K09B11.14	0	2	4	1	2.80E-06	2.65E-06	2.86E-06	2.25E-06
K09B11.15	0	2	4	1	2.80E-06	2.65E-06	2.86E-06	2.25E-06
K09B11.16	0	2	4	1	2.80E-06	2.65E-06	2.86E-06	2.25E-06
K09B11.2	194	200	199	326	2.80E-06	2.65E-06	2.86E-06	2.25E-06
K09B11.3	2	1	9	1	2.80E-06	2.65E-06	2.86E-06	2.25E-06
K09B11.4	6	5	0	4	1.27E-05	1.23E-05	8.45E-06	1.71E-05
K09B11.5a	28	34	32	19	2.80E-06	2.65E-06	1.90E-06	2.25E-06
K09B11.9a	182	231	279	481	2.80E-06	2.65E-06	1.82E-06	2.25E-06
K09B11.9b	179	224	267	473	2.80E-06	2.65E-06	1.82E-06	2.25E-06
K09B3.1	3	2	2	0	7.22E-06	8.65E-06	7.20E-06	1.53E-05
K09C4.10	77	82	62	123	7.62E-06	9.02E-06	7.40E-06	1.62E-05
K09C4.1a	44	44	78	33	2.80E-06	2.65E-06	1.82E-06	2.25E-06
K09C4.1b	12	25	22	12	2.80E-06	2.67E-06	1.82E-06	3.40E-06
K09C4.3	919	560	485	590	2.80E-06	2.65E-06	3.23E-06	2.25E-06
K09C4.4	6	9	6	3	2.80E-06	4.02E-06	2.42E-06	2.25E-06
K09C4.5	48	84	84	98	1.23E-04	7.06E-05	4.21E-05	6.33E-05
K09C4.6	2	2	3	2	2.80E-06	2.65E-06	1.82E-06	2.25E-06
K09C4.8	34	39	41	32	3.25E-06	5.37E-06	3.70E-06	5.33E-06
K09C4.9	8	2	4	2	2.80E-06	2.65E-06	1.82E-06	2.25E-06
K09C6.1	20	44	15	17	2.80E-06	2.65E-06	1.82E-06	2.25E-06
K09C6.10	3	2	1	0	2.80E-06	2.65E-06	1.82E-06	2.25E-06
K09C6.2	14	29	9	13	2.80E-06	2.65E-06	1.82E-06	2.25E-06
K09C6.3	4	4	8	5	2.80E-06	2.65E-06	1.82E-06	2.25E-06
K09C6.4	7	8	6	2	2.80E-06	2.96E-06	1.82E-06	2.25E-06
K09C6.5	6	7	6	3	2.80E-06	2.65E-06	1.82E-06	2.25E-06
K09C6.6	3	4	1	1	2.80E-06	2.65E-06	1.82E-06	2.25E-06
K09C6.7	48	75	34	33	2.80E-06	2.65E-06	1.82E-06	2.25E-06
K09C6.8	36	52	11	21	2.80E-06	2.65E-06	1.82E-06	2.25E-06
K09C6.9	34	26	28	10	3.05E-06	4.50E-06	1.82E-06	2.25E-06
K09C8.1	161	194	161	125	2.80E-06	3.33E-06	1.82E-06	2.25E-06
K09C8.2	2	5	2	2	6.47E-06	4.68E-06	3.46E-06	2.25E-06
K09C8.3	12	11	6	2	7.14E-06	8.15E-06	4.65E-06	4.45E-06
K09C8.4	8	12	22	12	2.80E-06	2.65E-06	1.82E-06	2.25E-06
K09C8.5	80	94	107	121	2.80E-06	2.65E-06	1.82E-06	2.25E-06
K09C8.6	12	23	20	13	2.80E-06	2.65E-06	1.82E-06	2.25E-06
K09C8.7	2	5	1	2	2.80E-06	2.65E-06	1.91E-06	2.68E-06
K09C8.8	2	4	1	0	3.25E-06	5.90E-06	3.53E-06	2.83E-06
K09D9.1	8	33	7	14	2.80E-06	2.65E-06	1.82E-06	2.25E-06
K09D9.10	5	4	9	7	2.80E-06	2.65E-06	1.82E-06	2.25E-06
K09D9.11	17	16	26	14	2.80E-06	3.94E-06	1.82E-06	2.25E-06
K09D9.12	27	24	18	26	2.80E-06	2.65E-06	1.82E-06	2.25E-06
K09D9.13	3	6	9	5	2.80E-06	2.65E-06	1.82E-06	2.25E-06
K09D9.2	199	173	224	128	2.80E-06	2.65E-06	1.82E-06	2.25E-06
K09D9.3	8	9	10	9	2.80E-06	2.65E-06	1.82E-06	2.25E-06

K09D9.4	2	9	9	4	1.50E-05	1.23E-05	1.10E-05	7.76E-06
K09D9.5	3	3	2	2	2.80E-06	2.65E-06	1.82E-06	2.25E-06
K09D9.6	2	4	5	3	2.80E-06	2.65E-06	1.82E-06	2.25E-06
K09D9.7	3	6	5	4	2.80E-06	2.65E-06	1.82E-06	2.25E-06
K09D9.8	1	6	7	2	2.80E-06	2.65E-06	1.82E-06	2.25E-06
K09D9.9	4	6	10	1	2.80E-06	2.65E-06	1.82E-06	2.25E-06
K09E10.1	38	53	32	12	2.80E-06	2.65E-06	1.82E-06	2.25E-06
K09E10.2	26	41	22	16	2.80E-06	2.65E-06	1.82E-06	2.25E-06
K09E2.1	17	20	9	5	2.80E-06	2.70E-06	1.82E-06	2.25E-06
K09E2.2	13	16	14	6	2.80E-06	2.65E-06	1.82E-06	2.25E-06
K09E2.3	267	397	243	331	2.80E-06	2.65E-06	1.82E-06	2.25E-06
K09E2.4a	76	116	54	104	2.80E-06	2.65E-06	1.82E-06	2.25E-06
K09E2.4b	31	45	11	37	4.40E-05	6.18E-05	2.60E-05	4.38E-05
K09E3.1	2	5	3	1	2.80E-06	3.17E-06	1.82E-06	2.41E-06
K09E3.2	0	1	5	2	3.81E-06	5.24E-06	1.82E-06	3.64E-06
K09E3.5	11	18	12	16	2.80E-06	2.65E-06	1.82E-06	2.25E-06
K09E3.6	16	29	22	17	2.80E-06	2.65E-06	1.82E-06	2.25E-06
K09E3.7	75	113	138	164	2.80E-06	2.65E-06	1.82E-06	2.25E-06
K09E4.1	26	46	29	8	2.80E-06	3.12E-06	1.82E-06	2.25E-06
K09E4.2	144	151	254	360	3.25E-06	4.63E-06	3.90E-06	5.71E-06
K09E4.3	115	146	244	155	2.80E-06	4.15E-06	1.82E-06	2.25E-06
K09E4.4.1	14	31	23	22	7.70E-06	7.62E-06	8.84E-06	1.55E-05
K09E4.4.2	9	27	12	19	1.16E-05	1.39E-05	1.59E-05	1.25E-05
K09E4.5	8	17	9	14	2.80E-06	2.65E-06	1.82E-06	2.25E-06
K09E4.6	164	311	98	51	2.80E-06	2.65E-06	1.82E-06	2.25E-06
K09E9.1.1	74	103	88	95	2.80E-06	2.65E-06	1.82E-06	2.25E-06
K09E9.1.2	70	100	85	93	4.32E-05	7.74E-05	1.68E-05	1.08E-05
K09E9.1.3	66	83	78	82	4.28E-06	5.61E-06	3.32E-06	4.41E-06
K09E9.1.4	63	80	74	77	4.14E-06	5.61E-06	3.28E-06	4.43E-06
K09E9.2.1	139	176	126	182	4.62E-06	5.50E-06	3.55E-06	4.61E-06
K09E9.2.2	117	140	104	155	4.62E-06	5.53E-06	3.53E-06	4.54E-06
K09E9.3	42	65	47	73	8.82E-06	1.06E-05	5.21E-06	9.29E-06
K09E9.4	5	4	13	1	1.14E-05	1.29E-05	6.60E-06	1.21E-05
K09F5.1	10	10	5	2	3.22E-06	4.73E-06	2.35E-06	4.52E-06
K09F5.2	59203	46040	88313	135486	2.80E-06	2.65E-06	4.32E-06	2.25E-06
K09F5.3.1	1105	1475	4785	830	2.80E-06	2.65E-06	1.82E-06	2.25E-06
K09F5.3.2	940	1254	3571	713	1.34E-03	9.83E-04	1.30E-03	2.46E-03
K09F5.4	2	4	7	7	3.33E-04	4.20E-04	9.38E-04	2.01E-04
K09F5.5	13	20	21	18	2.91E-04	3.67E-04	7.19E-04	1.77E-04
K09F5.6	57	78	57	80	2.80E-06	2.65E-06	1.82E-06	2.25E-06
K09F6.10	24	34	25	35	2.80E-06	2.65E-06	1.82E-06	2.25E-06
K09F6.11	4	1	7	8	2.80E-06	2.70E-06	1.82E-06	2.34E-06
K09F6.2	54	93	51	125	2.80E-06	2.65E-06	1.82E-06	2.25E-06
K09F6.3	105	147	41	65	2.80E-06	2.65E-06	1.82E-06	2.25E-06
K09F6.4	21	41	19	21	4.06E-06	6.59E-06	2.50E-06	7.53E-06
K09F6.5	10	6	6	6	3.22E-06	4.26E-06	1.82E-06	2.25E-06
K09F6.6	25	44	29	52	2.80E-06	2.65E-06	1.82E-06	2.25E-06
K09F6.7	7	16	5	13	2.80E-06	2.65E-06	1.82E-06	2.25E-06
K09F6.8	7	6	5	9	2.80E-06	2.65E-06	1.82E-06	2.25E-06
K09F6.9	27	36	21	44	2.80E-06	2.65E-06	1.82E-06	2.25E-06
K09G1.1a	488	981	412	635	2.80E-06	2.65E-06	1.82E-06	2.25E-06
K09G1.2	10	14	10	0	2.80E-06	2.65E-06	1.82E-06	2.25E-06
K09G1.3	7	5	2	3	8.49E-05	1.61E-04	4.66E-05	8.87E-05
K09G1.4a	17	33	27	32	2.91E-06	3.86E-06	1.90E-06	2.25E-06
K09G1.4b	24	37	28	34	4.84E-06	3.25E-06	1.82E-06	2.25E-06
K09H11.1	159	271	153	272	2.80E-06	2.65E-06	1.82E-06	2.25E-06
K09H11.10	0	4	1	3	2.80E-06	2.65E-06	1.82E-06	2.25E-06
K09H11.11	2	4	4	1	5.91E-06	9.50E-06	3.70E-06	8.12E-06
K09H11.2	3	3	5	2	2.80E-06	2.88E-06	1.82E-06	2.25E-06

K09H11.3	1146	748	2179	2220	2.80E-06	2.65E-06	1.82E-06	2.25E-06
K09H11.4	24	25	25	20	2.80E-06	2.65E-06	1.82E-06	2.25E-06
K09H11.5	6	4	8	9	3.42E-05	2.11E-05	4.23E-05	5.32E-05
K09H11.6	4	6	3	3	2.80E-06	2.65E-06	1.82E-06	2.25E-06
K09H11.7	239	819	277	506	2.80E-06	2.65E-06	2.31E-06	3.22E-06
K09H11.8	6	4	12	4	2.80E-06	2.65E-06	1.82E-06	2.25E-06
K09H11.9	4	5	9	2	2.76E-05	8.94E-05	2.08E-05	4.70E-05
K09H9.1	44	39	41	61	2.80E-06	2.65E-06	1.82E-06	2.25E-06
K09H9.3	450	473	184	148	2.80E-06	2.65E-06	1.82E-06	2.25E-06
K09H9.4	9	8	12	1	4.37E-06	3.68E-06	2.66E-06	4.88E-06
K09H9.6	433	476	235	498	5.92E-05	5.87E-05	1.57E-05	1.56E-05
K09H9.7	30	60	25	40	2.80E-06	2.65E-06	1.82E-06	2.25E-06
K10B2.2a	94	143	74	79	2.66E-05	2.76E-05	9.38E-06	2.46E-05
K10B2.2b	42	55	13	23	6.75E-06	1.27E-05	3.66E-06	7.22E-06
K10B2.4	251	239	704	218	6.52E-06	9.39E-06	3.35E-06	4.41E-06
K10B2.5	1795	1466	2580	3763	7.70E-06	9.50E-06	1.82E-06	3.37E-06
K10B3.10	1443	1869	1170	2251	6.69E-05	6.02E-05	1.22E-04	4.67E-05
K10B3.1a	24	35	31	15	5.80E-05	4.48E-05	5.43E-05	9.77E-05
K10B3.1b	71	104	162	38	2.07E-05	2.54E-05	1.09E-05	2.60E-05
K10B3.5	106	88	93	137	1.21E-05	1.67E-05	1.02E-05	6.07E-06
K10B3.6a	66	124	50	63	1.74E-05	2.41E-05	2.59E-05	7.49E-06
K10B3.6b	69	128	56	64	5.46E-06	4.29E-06	3.12E-06	5.67E-06
K10B3.6c	37	55	22	28	3.14E-06	5.58E-06	1.82E-06	2.41E-06
K10B3.7.1	2563	3930	3552	4472	5.18E-06	9.07E-06	2.73E-06	3.87E-06
K10B3.7.2	2607	3934	3723	4563	5.07E-06	7.14E-06	1.97E-06	3.08E-06
K10B3.9.1	2303	3559	3242	3901	2.48E-04	3.60E-04	2.24E-04	3.48E-04
K10B3.9.2	2305	3577	3246	3912	2.47E-04	3.52E-04	2.30E-04	3.48E-04
K10B3.9.3	18	60	25	27	1.48E-04	2.16E-04	1.36E-04	2.01E-04
K10B4.1	20	17	11	9	1.19E-04	1.75E-04	1.09E-04	1.63E-04
K10B4.2	3	3	12	3	3.72E-06	1.17E-05	3.37E-06	4.50E-06
K10B4.3	73	127	63	105	2.80E-06	2.65E-06	1.82E-06	2.25E-06
K10B4.4	6	16	2	2	2.80E-06	2.65E-06	1.82E-06	2.25E-06
K10B4.5	4	11	9	3	3.14E-06	5.13E-06	1.82E-06	3.62E-06
K10B4.6a	40	71	29	42	2.80E-06	2.65E-06	1.82E-06	2.25E-06
K10B4.6b	30	59	21	35	2.80E-06	2.65E-06	1.82E-06	2.25E-06
K10C2.1	3885	3224	3988	5986	3.42E-06	5.71E-06	1.82E-06	2.88E-06
K10C2.2	2	3	3	3	2.80E-06	3.78E-06	1.82E-06	2.25E-06
K10C2.3	1973	527	1686	704	6.15E-05	4.82E-05	4.11E-05	7.61E-05
K10C2.4.1	800	805	938	914	2.80E-06	2.65E-06	1.82E-06	2.25E-06
K10C2.4.2	739	728	827	849	1.79E-04	4.51E-05	9.93E-05	5.12E-05
K10C2.5	7	9	2	2	5.85E-05	5.56E-05	4.47E-05	5.37E-05
K10C2.6	4	2	14	6	6.54E-05	6.09E-05	4.76E-05	6.04E-05
K10C2.7	34	22	43	39	2.80E-06	2.65E-06	1.82E-06	2.25E-06
K10C3.2.1	914	976	1294	1689	2.80E-06	2.65E-06	2.95E-06	2.25E-06
K10C3.2.2	358	336	519	645	6.05E-06	3.68E-06	4.97E-06	5.56E-06
K10C3.3	366	467	468	543	8.50E-05	8.57E-05	7.83E-05	1.26E-04
K10C3.4	524	421	789	1089	6.72E-05	5.95E-05	6.34E-05	9.72E-05
K10C3.5a	414	441	361	538	4.03E-05	4.86E-05	3.35E-05	4.80E-05
K10C3.5b	329	353	287	442	5.27E-05	4.00E-05	5.17E-05	8.80E-05
K10C3.6a	642	944	838	1216	1.65E-05	1.66E-05	9.38E-06	1.72E-05
K10C3.6b.1	490	718	678	903	1.65E-05	1.67E-05	9.38E-06	1.78E-05
K10C3.6b.2	472	705	652	890	5.01E-05	6.96E-05	4.25E-05	7.62E-05
K10C3.6c.1	647	956	852	1230	2.79E-05	3.86E-05	2.51E-05	4.13E-05
K10C3.6c.2	644	946	840	1217	3.69E-05	5.21E-05	3.32E-05	5.59E-05
K10C3.6d	645	946	840	1222	3.43E-05	4.79E-05	2.94E-05	5.24E-05
K10C8.1	68	93	95	127	3.55E-05	4.93E-05	3.01E-05	5.39E-05
K10C8.2	8	10	4	11	4.73E-05	6.56E-05	4.01E-05	7.20E-05
K10C8.4	10	7	16	6	4.48E-06	5.79E-06	4.06E-06	6.72E-06
K10C9.1	4	7	14	7	2.80E-06	2.65E-06	1.82E-06	2.25E-06

K10C9.3	141	180	151	137	2.80E-06	2.65E-06	2.62E-06	2.25E-06
K10C9.4	7	6	5	3	2.80E-06	3.44E-06	4.72E-06	2.92E-06
K10C9.6	4	5	16	14	1.87E-05	2.25E-05	1.30E-05	1.46E-05
K10C9.7	20	16	14	11	2.80E-06	2.65E-06	1.82E-06	2.25E-06
K10C9.8	5	4	6	2	2.80E-06	2.65E-06	1.82E-06	2.25E-06
K10C9.9	4	1	1	1	2.80E-06	2.65E-06	1.82E-06	2.25E-06
K10D11.1	119	58	157	27	2.80E-06	2.65E-06	1.82E-06	2.25E-06
K10D11.2	8	5	7	3	2.80E-06	2.65E-06	1.82E-06	2.25E-06
K10D11.3	15	21	21	13	1.30E-05	5.98E-06	1.12E-05	2.36E-06
K10D11.4	5	3	4	3	2.80E-06	2.65E-06	1.82E-06	2.25E-06
K10D11.5	94	97	97	69	2.80E-06	2.65E-06	1.82E-06	2.25E-06
K10D11.6	28	35	21	17	2.80E-06	2.65E-06	1.82E-06	2.25E-06
K10D2.1a	499	575	592	832	6.86E-06	6.69E-06	4.61E-06	4.05E-06
K10D2.1b	302	327	416	623	2.80E-06	2.65E-06	1.82E-06	2.25E-06
K10D2.2.1	2798	2241	3013	4116	1.44E-05	1.56E-05	1.11E-05	1.93E-05
K10D2.2.2	1933	1513	1954	2650	1.67E-05	1.70E-05	1.49E-05	2.76E-05
K10D2.3	1408	1347	1732	2840	1.32E-04	1.00E-04	9.29E-05	1.57E-04
K10D2.4	159	119	456	88	1.33E-04	9.86E-05	8.78E-05	1.47E-04
K10D2.5	118	138	235	101	3.30E-05	2.98E-05	2.64E-05	5.35E-05
K10D2.6.1	1475	1828	1952	2375	5.86E-05	4.14E-05	1.09E-04	2.60E-05
K10D2.6.2	1304	1603	1711	2111	3.38E-05	3.73E-05	4.38E-05	2.32E-05
K10D2.7	10	14	28	14	7.22E-05	8.45E-05	6.22E-05	9.33E-05
K10D3.1	9	16	17	10	7.28E-05	8.45E-05	6.22E-05	9.47E-05
K10D3.2	309	427	259	487	4.40E-06	5.82E-06	8.00E-06	4.95E-06
K10D3.3	174	287	189	234	2.80E-06	2.65E-06	1.82E-06	2.25E-06
K10D3.4	356	430	310	227	1.27E-05	1.65E-05	6.91E-06	1.60E-05
K10D6.1	17	20	11	6	1.93E-05	3.00E-05	1.36E-05	2.08E-05
K10D6.2a	57	159	62	91	1.16E-05	1.33E-05	6.60E-06	5.96E-06
K10D6.2b	55	161	59	87	2.80E-06	2.65E-06	1.82E-06	2.25E-06
K10D6.2c	64	162	62	92	5.46E-06	1.44E-05	3.86E-06	7.02E-06
K10D6.3	0	0	2	1	6.22E-06	1.72E-05	4.34E-06	7.92E-06
K10D6.4.1	64	96	113	76	6.69E-06	1.60E-05	4.21E-06	7.71E-06
K10D6.4.2	60	90	114	73	2.80E-06	2.65E-06	1.82E-06	2.25E-06
K10E9.1	196	185	254	466	4.17E-06	5.90E-06	4.79E-06	3.98E-06
K10F12.1	2	8	10	2	6.97E-06	9.89E-06	8.64E-06	6.81E-06
K10F12.3a	27	44	29	25	5.04E-06	4.50E-06	4.25E-06	9.63E-06
K10F12.3b	31	48	43	38	2.80E-06	2.65E-06	1.82E-06	2.25E-06
K10F12.4a	58	81	52	73	2.80E-06	2.65E-06	1.82E-06	2.25E-06
K10F12.4b.1	43	61	31	49	2.80E-06	2.65E-06	1.82E-06	2.25E-06
K10F12.4b.2	36	56	30	45	6.97E-06	9.20E-06	4.08E-06	7.06E-06
K10F12.6	0	1	2	1	6.33E-06	8.49E-06	2.97E-06	5.80E-06
K10F12.7	8	6	14	3	5.94E-06	8.73E-06	3.23E-06	5.96E-06
K10G4.1	14	8	14	4	2.80E-06	2.65E-06	1.82E-06	2.25E-06
K10G4.10	2	6	8	6	2.80E-06	2.65E-06	1.82E-06	2.25E-06
K10G4.11	1	1	0	0	2.80E-06	2.65E-06	1.82E-06	2.25E-06
K10G4.2	7	6	8	3	2.80E-06	2.65E-06	1.82E-06	2.25E-06
K10G4.3	8	13	12	9	2.80E-06	2.65E-06	1.82E-06	2.25E-06
K10G4.4	11	7	14	3	2.80E-06	2.65E-06	1.82E-06	2.25E-06
K10G4.5	14	47	25	20	2.80E-06	2.65E-06	1.82E-06	2.25E-06
K10G4.6	5	4	1	1	2.80E-06	2.65E-06	1.82E-06	2.25E-06
K10G4.7	10	18	68	20	2.80E-06	2.65E-06	1.82E-06	2.25E-06
K10G4.8	1	3	4	1	2.80E-06	2.65E-06	1.82E-06	2.25E-06
K10G4.9	3	6	4	1	2.80E-06	2.65E-06	4.96E-06	2.25E-06
K10G6.1	24	38	25	37	2.80E-06	2.65E-06	1.82E-06	2.25E-06
K10G6.2	11	20	0	3	2.80E-06	2.65E-06	1.82E-06	2.25E-06
K10G6.3	252	234	242	363	3.39E-06	5.05E-06	2.30E-06	4.18E-06
K10G6.4	9	13	14	9	2.80E-06	2.65E-06	1.82E-06	2.25E-06
K10G6.5	8	6	2	7	4.82E-06	4.23E-06	3.01E-06	5.56E-06
K10G9.1	70	178	55	114	2.80E-06	2.65E-06	1.82E-06	2.25E-06

K10G9.2	33	50	48	46	2.80E-06	2.65E-06	1.82E-06	2.25E-06
K10G9.3	31	34	37	41	3.72E-06	8.91E-06	1.90E-06	4.86E-06
K10H10.1	319	286	388	547	3.28E-06	4.68E-06	3.10E-06	3.67E-06
K10H10.10	11	10	13	5	2.80E-06	2.65E-06	1.91E-06	2.61E-06
K10H10.2.1	264	305	238	271	2.16E-05	1.83E-05	1.71E-05	2.98E-05
K10H10.2.2	239	278	224	261	2.80E-06	2.65E-06	1.82E-06	2.25E-06
K10H10.3a	10	15	12	10	2.49E-05	2.72E-05	1.46E-05	2.05E-05
K10H10.4	49	43	17	8	2.56E-05	2.81E-05	1.56E-05	2.24E-05
K10H10.5	20	19	10	11	2.80E-06	2.65E-06	1.82E-06	2.25E-06
K10H10.6	1	3	9	3	5.49E-06	4.55E-06	1.82E-06	2.25E-06
K10H10.7	9	11	21	7	3.89E-06	3.49E-06	1.82E-06	2.25E-06
K10H10.9	2	6	5	4	2.80E-06	2.65E-06	1.82E-06	2.25E-06
K11B4.2	111	92	145	102	2.80E-06	2.91E-06	3.84E-06	2.25E-06
K11C4.1	11	40	10	7	2.80E-06	2.65E-06	1.82E-06	2.25E-06
K11C4.2	16	40	13	15	2.28E-05	1.78E-05	1.94E-05	1.68E-05
K11C4.3a	1691	1585	1630	2260	2.80E-06	4.58E-06	1.82E-06	2.25E-06
K11C4.3b	1692	1586	1640	2282	2.80E-06	4.26E-06	1.82E-06	2.25E-06
K11C4.5	949	1221	737	1296	2.23E-05	1.97E-05	1.40E-05	2.39E-05
K11D12.1	12	16	12	12	2.44E-05	2.16E-05	1.54E-05	2.64E-05
K11D12.10a	99	156	71	128	6.80E-06	8.25E-06	3.43E-06	7.44E-06
K11D12.10b	114	178	81	152	2.80E-06	2.65E-06	1.82E-06	2.25E-06
K11D12.11	7	9	9	3	3.56E-06	5.29E-06	1.82E-06	3.69E-06
K11D12.12	39	33	43	54	3.84E-06	5.66E-06	1.82E-06	4.12E-06
K11D12.13	165	318	328	436	2.80E-06	2.65E-06	1.82E-06	2.25E-06
K11D12.2.1	938	792	1337	1655	6.27E-06	5.00E-06	4.50E-06	6.97E-06
K11D12.2.2	871	718	1208	1564	3.26E-05	5.93E-05	4.22E-05	6.92E-05
K11D12.2.3	924	765	1309	1633	6.24E-05	4.98E-05	5.79E-05	8.85E-05
K11D12.2.4	639	518	848	1142	5.97E-05	4.65E-05	5.39E-05	8.61E-05
K11D12.4	140	194	61	51	7.11E-05	5.56E-05	6.55E-05	1.01E-04
K11D12.5	197	277	250	181	5.84E-05	4.47E-05	5.04E-05	8.38E-05
K11D12.6	3	5	5	3	6.75E-06	8.83E-06	1.91E-06	2.25E-06
K11D12.7.1	147	218	92	154	3.04E-05	4.04E-05	2.51E-05	2.24E-05
K11D12.7.2	129	187	77	119	2.80E-06	2.65E-06	1.82E-06	2.25E-06
K11D12.8	11	21	12	16	2.33E-05	3.26E-05	9.48E-06	1.96E-05
K11D12.9	14	13	21	14	2.28E-05	3.12E-05	8.86E-06	1.69E-05
K11D2.1	8	12	10	14	2.80E-06	2.65E-06	1.82E-06	2.25E-06
K11D2.2	397	306	546	456	2.80E-06	2.65E-06	1.82E-06	2.25E-06
K11D2.3a.1	194	175	427	399	2.80E-06	2.65E-06	1.82E-06	2.34E-06
K11D2.3a.2	113	104	205	297	3.50E-05	2.55E-05	3.13E-05	3.23E-05
K11D2.3b	8	3	11	23	1.16E-05	9.87E-06	1.66E-05	1.91E-05
K11D2.4a	140	116	274	290	9.46E-06	8.23E-06	1.12E-05	2.00E-05
K11D2.4b	120	102	236	257	5.07E-06	2.65E-06	4.54E-06	1.17E-05
K11D2.5	62	66	88	118	1.36E-05	1.06E-05	1.73E-05	2.26E-05
K11D9.1a	930	778	1159	1354	1.41E-05	1.13E-05	1.80E-05	2.42E-05
K11D9.1b.1	949	781	1163	1326	6.19E-06	6.22E-06	5.72E-06	9.47E-06
K11D9.1b.2	845	679	1023	1206	4.18E-05	3.30E-05	3.39E-05	4.88E-05
K11D9.2a.1	6024	6303	6256	9046	4.70E-05	3.65E-05	3.74E-05	5.27E-05
K11D9.2a.2	6024	6303	6256	9046	4.56E-05	3.46E-05	3.59E-05	5.23E-05
K11D9.3.1	75	114	94	108	1.86E-04	1.83E-04	1.25E-04	2.24E-04
K11D9.3.2	76	114	89	105	1.86E-04	1.84E-04	1.25E-04	2.24E-04
K11D9.3.3	70	109	81	95	5.10E-06	7.33E-06	4.15E-06	5.89E-06
K11E4.1	8	14	15	15	4.90E-06	6.93E-06	3.74E-06	5.42E-06
K11E4.2	18	17	17	16	5.43E-06	7.99E-06	4.08E-06	5.91E-06
K11E4.3	9	9	11	10	2.80E-06	2.65E-06	1.82E-06	2.25E-06
K11E4.4	79	135	137	126	2.80E-06	2.65E-06	1.82E-06	2.25E-06
K11E4.5a	101	100	133	141	2.80E-06	2.65E-06	1.82E-06	2.25E-06
K11E4.5b	133	127	161	193	2.80E-06	4.21E-06	2.93E-06	3.33E-06
K11E4.t1	0	0	3	0	4.93E-06	4.60E-06	4.23E-06	5.53E-06
K11E4.t2	0	0	1	0	6.86E-06	6.19E-06	5.41E-06	7.98E-06

K11E4.t3	0	0	3	0	2.80E-06	2.65E-06	2.99E-06	2.25E-06
K11E4.t4	0	0	2	0	2.80E-06	2.65E-06	1.82E-06	2.25E-06
K11E4.t5	0	0	3	0	2.80E-06	2.65E-06	2.99E-06	2.25E-06
K11E4.t6	0	0	3	0	2.80E-06	2.65E-06	2.00E-06	2.25E-06
K11E8.1a.1	838	1057	626	1089	2.80E-06	2.65E-06	2.99E-06	2.25E-06
K11E8.1a.2	520	637	405	546	2.80E-06	2.65E-06	2.99E-06	2.25E-06
K11E8.1b.1	158	168	126	146	4.93E-05	5.87E-05	2.40E-05	5.15E-05
K11E8.1b.2	129	142	100	129	2.93E-05	3.40E-05	1.49E-05	2.47E-05
K11E8.1b.3	153	164	124	144	2.77E-05	2.78E-05	1.44E-05	2.06E-05
K11E8.1c.1	510	619	388	546	2.27E-05	2.35E-05	1.14E-05	1.82E-05
K11E8.1c.2	531	640	407	556	2.38E-05	2.41E-05	1.26E-05	1.80E-05
K11E8.1d.1	827	1047	605	1070	2.39E-05	2.74E-05	1.18E-05	2.06E-05
K11E8.1d.2	509	627	384	527	2.41E-05	2.74E-05	1.20E-05	2.03E-05
K11E8.1e.1	494	608	369	529	3.86E-05	4.62E-05	1.84E-05	4.01E-05
K11E8.1e.2	515	629	388	539	3.25E-05	3.78E-05	1.60E-05	2.70E-05
K11E8.1f.1	499	616	385	537	3.03E-05	3.52E-05	1.47E-05	2.61E-05
K11E8.1f.2	520	637	404	547	3.02E-05	3.49E-05	1.48E-05	2.54E-05
K11E8.1g.1	509	632	390	539	3.14E-05	3.66E-05	1.58E-05	2.71E-05
K11E8.1g.2	530	653	409	549	3.13E-05	3.62E-05	1.58E-05	2.64E-05
K11E8.1h.1	515	634	394	551	3.19E-05	3.74E-05	1.59E-05	2.71E-05
K11E8.1h.2	536	655	413	561	3.18E-05	3.70E-05	1.60E-05	2.64E-05
K11E8.1i.1	320	381	278	347	2.97E-05	3.46E-05	1.48E-05	2.55E-05
K11E8.1i.2	325	394	264	338	2.97E-05	3.43E-05	1.49E-05	2.50E-05
K11E8.1k.1	797	1000	596	1047	1.83E-05	2.05E-05	1.03E-05	1.59E-05
K11E8.1k.2	280	330	237	299	2.68E-05	3.07E-05	1.42E-05	2.24E-05
K11E8.1l.1	807	1016	593	1056	3.77E-05	4.47E-05	1.84E-05	3.98E-05
K11E8.1l.2	280	330	237	299	2.58E-05	2.88E-05	1.42E-05	2.22E-05
K11E8.1m	264	348	194	269	3.85E-05	4.57E-05	1.84E-05	4.04E-05
K11G12.1a	199	401	215	421	2.58E-05	2.88E-05	1.42E-05	2.22E-05
K11G12.1b	196	372	195	388	2.69E-05	3.35E-05	1.29E-05	2.20E-05
K11G12.2	8	17	17	16	1.25E-05	2.39E-05	8.80E-06	2.13E-05
K11G12.3	5	9	6	2	1.31E-05	2.35E-05	8.49E-06	2.09E-05
K11G12.4a	43	95	50	65	2.80E-06	2.65E-06	1.82E-06	2.25E-06
K11G12.4b	38	78	46	54	2.80E-06	2.65E-06	1.82E-06	2.25E-06
K11G12.6a	103	157	158	239	2.80E-06	5.53E-06	2.00E-06	3.22E-06
K11G12.6b	132	182	190	265	2.80E-06	5.34E-06	2.17E-06	3.15E-06
K11G12.7	8	21	19	17	6.69E-06	9.63E-06	6.69E-06	1.25E-05
K11G9.1	10	11	18	2	6.05E-06	7.88E-06	5.67E-06	9.76E-06
K11G9.2	42	51	49	38	2.80E-06	2.65E-06	1.82E-06	2.25E-06
K11G9.3	9	12	13	14	2.80E-06	2.65E-06	1.82E-06	2.25E-06
K11G9.4	17	16	13	25	2.97E-06	3.39E-06	2.24E-06	2.25E-06
K11G9.5	47	59	44	46	2.80E-06	2.65E-06	1.82E-06	2.25E-06
K11H12.1	61	68	145	48	2.80E-06	2.65E-06	1.82E-06	2.41E-06
K11H12.10	2	1	0	1	3.28E-06	3.89E-06	1.99E-06	2.56E-06
K11H12.11	22	13	11	6	1.87E-05	1.97E-05	2.90E-05	1.18E-05
K11H12.2.1	9408	7999	14058	10099	2.80E-06	2.65E-06	1.82E-06	2.25E-06
K11H12.2.2	7688	6767	10993	9267	2.80E-06	2.65E-06	1.82E-06	2.25E-06
K11H12.3	6	3	4	4	1.45E-03	1.17E-03	1.41E-03	1.25E-03
K11H12.4	73	29	64	29	1.18E-03	9.83E-04	1.10E-03	1.15E-03
K11H12.5	5	9	14	8	2.80E-06	2.65E-06	1.82E-06	2.25E-06
K11H12.6	2	5	7	3	6.64E-06	2.65E-06	3.77E-06	2.25E-06
K11H12.7	258	321	210	202	2.80E-06	2.65E-06	1.82E-06	2.25E-06
K11H12.8a	349	500	352	497	2.80E-06	2.65E-06	1.82E-06	2.25E-06
K11H12.8b	306	437	284	455	4.24E-05	4.98E-05	2.24E-05	2.67E-05
K11H12.9	6	13	21	16	3.25E-05	4.39E-05	2.13E-05	3.71E-05
K11H3.1a	499	853	425	559	2.84E-05	3.83E-05	1.71E-05	3.39E-05
K11H3.2	13	35	17	11	2.80E-06	2.65E-06	1.82E-06	2.25E-06
K11H3.3	537	466	1179	913	4.80E-05	7.75E-05	2.66E-05	4.32E-05
K11H3.4	289	278	389	403	2.80E-06	5.40E-06	1.82E-06	2.25E-06

K11H3.5	2	8	4	1	3.94E-05	3.23E-05	5.62E-05	5.38E-05
K11H3.6	219	158	831	207	1.50E-05	1.36E-05	1.32E-05	1.68E-05
K11H3.7	22	29	15	12	2.80E-06	2.65E-06	1.82E-06	2.25E-06
K12B6.1	251	313	285	473	8.10E-05	5.52E-05	2.00E-04	6.15E-05
K12B6.2	17	26	17	11	2.80E-06	2.75E-06	1.82E-06	2.25E-06
K12B6.3	6	5	6	6	9.38E-06	1.11E-05	6.94E-06	1.42E-05
K12B6.4	6	3	4	4	2.80E-06	2.65E-06	1.82E-06	2.25E-06
K12B6.5	5	7	11	12	2.80E-06	2.65E-06	1.82E-06	2.25E-06
K12B6.6	0	1	0	2	2.80E-06	2.65E-06	1.82E-06	2.25E-06
K12B6.7	52	14	140	70	2.80E-06	2.65E-06	1.82E-06	2.25E-06
K12B6.8	37	41	25	49	2.80E-06	2.65E-06	1.82E-06	2.25E-06
K12B6.9	49	64	46	31	4.03E-06	2.65E-06	7.05E-06	4.36E-06
K12C11.1.1	275	399	412	515	2.80E-06	2.65E-06	1.82E-06	2.25E-06
K12C11.3	7	8	7	4	9.18E-06	1.13E-05	5.61E-06	4.66E-06
K12C11.4	97	205	93	217	1.88E-05	2.58E-05	1.84E-05	2.84E-05
K12D12.1	3104	2785	3991	5287	2.80E-06	2.65E-06	1.82E-06	2.25E-06
K12D12.2.1	1573	1157	2165	2939	2.80E-06	5.08E-06	1.82E-06	4.57E-06
K12D12.2.2	1542	1126	2123	2884	7.17E-05	6.08E-05	6.00E-05	9.81E-05
K12D12.3	22	26	10	12	3.35E-05	2.33E-05	3.00E-05	5.03E-05
K12D12.4a	9	17	13	7	3.37E-05	2.32E-05	3.02E-05	5.06E-05
K12D12.5	366	289	382	521	2.80E-06	2.65E-06	1.82E-06	2.25E-06
K12D9.1	6	4	8	2	2.80E-06	2.65E-06	1.82E-06	2.25E-06
K12D9.10	5	8	2	5	2.11E-05	1.57E-05	1.43E-05	2.41E-05
K12D9.11	3	3	7	3	2.80E-06	2.65E-06	1.82E-06	2.25E-06
K12D9.12	21	21	36	34	2.80E-06	2.65E-06	1.82E-06	2.25E-06
K12D9.13	4	5	10	5	2.80E-06	2.65E-06	1.82E-06	2.25E-06
K12D9.2	4	4	7	3	2.80E-06	2.65E-06	1.82E-06	2.25E-06
K12D9.3	5	6	9	6	2.80E-06	2.65E-06	1.82E-06	2.25E-06
K12D9.4	6	4	4	3	2.80E-06	2.65E-06	1.82E-06	2.25E-06
K12D9.5	5	7	6	3	2.80E-06	2.65E-06	1.82E-06	2.25E-06
K12D9.6	1	3	5	2	2.80E-06	2.65E-06	1.82E-06	2.25E-06
K12D9.7	3	6	3	3	2.80E-06	2.65E-06	1.82E-06	2.25E-06
K12D9.8	5	3	16	1	2.80E-06	2.65E-06	1.82E-06	2.25E-06
K12D9.9	2	8	11	1	2.80E-06	2.65E-06	1.82E-06	2.25E-06
K12F2.1	2460	2703	2224	3001	2.80E-06	2.65E-06	1.82E-06	2.25E-06
K12F2.2a	138	193	100	193	2.80E-06	2.65E-06	1.82E-06	2.25E-06
K12F2.2b	77	109	76	112	4.55E-05	4.72E-05	2.67E-05	4.46E-05
K12G11.1	23	55	37	51	4.56E-06	6.03E-06	2.15E-06	5.13E-06
K12G11.2	18	17	16	18	4.90E-06	6.56E-06	3.15E-06	5.71E-06
K12G11.4	3	7	3	0	2.80E-06	2.65E-06	1.82E-06	2.25E-06
K12G11.5	6	6	5	5	2.80E-06	2.65E-06	1.82E-06	2.25E-06
K12G11.6	4	2	2	3	2.80E-06	2.65E-06	1.82E-06	2.25E-06
K12H4.1	48	58	34	44	2.80E-06	2.65E-06	1.82E-06	2.25E-06
K12H4.2	85	81	72	90	2.80E-06	2.65E-06	1.82E-06	2.25E-06
K12H4.3	364	400	328	459	2.80E-06	2.78E-06	1.82E-06	2.25E-06
K12H4.4.1	500	526	555	604	1.66E-05	1.49E-05	9.17E-06	1.41E-05
K12H4.4.2	428	471	334	437	3.46E-05	3.59E-05	2.03E-05	3.51E-05
K12H4.5.1	559	608	1485	389	7.79E-05	7.74E-05	5.63E-05	7.56E-05
K12H4.5.2	515	579	1211	363	7.61E-05	7.91E-05	3.86E-05	6.24E-05
K12H4.6	0	1	6	1	1.45E-04	1.49E-04	2.51E-04	8.10E-05
K12H4.7a	2754	2775	3740	4720	1.31E-04	1.39E-04	2.01E-04	7.42E-05
K12H4.8	842	933	1056	1675	2.80E-06	2.65E-06	1.82E-06	2.25E-06
K12H6.1	11	12	30	48	1.92E-04	1.83E-04	1.70E-04	2.65E-04
K12H6.10	0	0	0	3	1.65E-05	1.72E-05	1.34E-05	2.63E-05
K12H6.11	3	3	3	1	2.80E-06	2.65E-06	1.88E-06	3.71E-06
K12H6.12	9	12	4	8	2.80E-06	2.65E-06	1.82E-06	2.25E-06
K12H6.2	36	40	28	47	2.80E-06	2.65E-06	1.82E-06	2.25E-06
K12H6.3	5	11	54	16	2.80E-06	2.65E-06	1.82E-06	2.25E-06
K12H6.4	1	1	5	5	4.45E-06	4.66E-06	2.24E-06	4.66E-06

K12H6.5	0	3	4	4	2.80E-06	2.65E-06	3.43E-06	2.25E-06
K12H6.6a	113	191	132	217	2.80E-06	2.65E-06	1.82E-06	2.25E-06
K12H6.6b	113	186	130	213	2.80E-06	2.65E-06	1.82E-06	2.25E-06
K12H6.7	4	4	4	2	1.13E-05	1.80E-05	8.56E-06	1.74E-05
K12H6.9	0	0	0	2	1.01E-05	1.57E-05	7.56E-06	1.53E-05
LLC1.1	271	248	257	339	2.80E-06	2.65E-06	1.82E-06	2.25E-06
LLC1.3.1	1505	1669	1377	1769	2.80E-06	2.65E-06	1.82E-06	2.25E-06
LLC1.3.2	1296	1416	1121	1511	1.32E-05	1.15E-05	8.16E-06	1.33E-05
M01A10.1	219	240	305	478	9.25E-05	9.69E-05	5.50E-05	8.73E-05
M01A10.2a	94	202	97	150	9.72E-05	1.00E-04	5.47E-05	9.10E-05
M01A10.2b	15	55	21	24	1.18E-05	1.22E-05	1.07E-05	2.06E-05
M01A10.2c	90	191	86	156	3.22E-06	6.53E-06	2.17E-06	4.12E-06
M01A10.2d	70	156	75	125	2.80E-06	6.69E-06	1.82E-06	2.47E-06
M01A12.1	10	9	1	5	2.80E-06	5.55E-06	1.82E-06	3.87E-06
M01A12.3	5	11	4	3	2.80E-06	4.71E-06	1.82E-06	3.22E-06
M01A12.4	6	13	3	7	2.80E-06	2.65E-06	1.82E-06	2.25E-06
M01A8.1	8	11	6	7	2.80E-06	3.25E-06	1.82E-06	2.25E-06
M01A8.2a	127	188	96	156	2.80E-06	2.65E-06	1.82E-06	2.25E-06
M01A8.2b	141	199	116	174	2.80E-06	2.65E-06	1.82E-06	2.25E-06
M01B12.3	158	169	157	184	5.01E-06	7.01E-06	2.46E-06	4.95E-06
M01B12.4a.1	614	669	782	1253	5.32E-06	7.09E-06	2.84E-06	5.26E-06
M01B12.4a.2	482	498	613	966	3.14E-05	3.18E-05	2.03E-05	2.94E-05
M01B12.4b	474	490	606	950	2.69E-05	2.77E-05	2.23E-05	4.41E-05
M01B12.4c	605	658	749	1197	2.78E-05	2.71E-05	2.30E-05	4.48E-05
M01B12.5a	483	498	447	651	2.69E-05	2.63E-05	2.24E-05	4.33E-05
M01B12.5b.1	175	164	147	210	2.53E-05	2.60E-05	2.04E-05	4.02E-05
M01B12.5b.2	164	151	134	204	3.53E-05	3.44E-05	2.12E-05	3.82E-05
M01B2.1	15	12	18	9	2.24E-05	1.98E-05	1.22E-05	2.16E-05
M01B2.10	21	35	28	32	2.28E-05	1.98E-05	1.21E-05	2.27E-05
M01B2.11	1	6	7	5	2.80E-06	2.65E-06	1.82E-06	2.25E-06
M01B2.2	7	10	1	5	2.80E-06	3.09E-06	1.82E-06	2.41E-06
M01B2.3	7	7	14	7	2.80E-06	2.65E-06	1.82E-06	2.25E-06
M01B2.4	2	10	11	9	2.80E-06	2.65E-06	1.82E-06	2.25E-06
M01B2.5	3	1	13	4	2.80E-06	2.65E-06	1.82E-06	2.25E-06
M01B2.6	15	10	6	8	2.80E-06	2.65E-06	1.82E-06	2.25E-06
M01B2.7	4	1	2	0	2.80E-06	2.65E-06	1.82E-06	2.25E-06
M01B2.8	1	1	4	1	2.80E-06	2.65E-06	1.82E-06	2.25E-06
M01B2.9	5	3	10	0	2.80E-06	2.65E-06	1.82E-06	2.25E-06
M01D1.1	5	5	6	4	2.80E-06	2.65E-06	1.82E-06	2.25E-06
M01D1.10	9	7	3	4	2.80E-06	2.65E-06	1.82E-06	2.25E-06
M01D1.2a	6	14	5	4	2.80E-06	2.65E-06	1.82E-06	2.25E-06
M01D1.2b	16	17	17	16	2.80E-06	2.65E-06	1.82E-06	2.25E-06
M01D1.3	16	10	5	9	2.80E-06	2.65E-06	1.82E-06	2.25E-06
M01D1.7	2	2	2	0	2.80E-06	2.65E-06	1.82E-06	2.25E-06
M01D1.8	12	11	3	6	2.80E-06	2.65E-06	1.82E-06	2.25E-06
M01D1.9	4	5	5	7	2.80E-06	2.65E-06	1.82E-06	2.25E-06
M01D7.1	6	6	6	2	2.80E-06	2.65E-06	1.82E-06	2.25E-06
M01D7.2	340	241	584	596	2.80E-06	2.65E-06	1.82E-06	2.25E-06
M01D7.4	54	76	57	72	2.80E-06	2.65E-06	1.82E-06	2.25E-06
M01D7.5	53	59	73	17	2.73E-05	1.83E-05	3.05E-05	3.85E-05
M01D7.6.1	439	308	475	465	4.59E-06	6.11E-06	3.15E-06	4.93E-06
M01D7.6.2	311	224	341	365	1.62E-05	1.70E-05	1.45E-05	4.16E-06
M01D7.6.3	375	278	400	443	7.67E-05	5.08E-05	5.40E-05	6.53E-05
M01D7.7a	742	909	646	1174	6.14E-05	4.18E-05	4.38E-05	5.79E-05
M01D7.7b	452	525	404	678	7.07E-05	4.95E-05	4.91E-05	6.71E-05
M01E10.2	63	69	29	27	5.08E-05	5.87E-05	2.88E-05	6.45E-05
M01E10.3	1	0	2	1	3.78E-05	4.15E-05	2.20E-05	4.56E-05
M01E11.1	120	157	202	178	2.80E-06	2.65E-06	1.82E-06	2.25E-06
M01E11.2.1	418	476	476	622	2.80E-06	2.65E-06	1.82E-06	2.25E-06

M01E11.3	275	264	249	529	1.52E-05	1.88E-05	1.67E-05	1.81E-05
M01E11.4a	817	920	1141	1233	2.76E-05	2.96E-05	2.04E-05	3.29E-05
M01E11.4b	918	1087	1436	1572	1.07E-05	9.73E-06	6.32E-06	1.66E-05
M01E11.4c	971	1125	1247	1411	1.24E-04	1.32E-04	1.13E-04	1.51E-04
M01E11.5.1	2592	2440	3103	4119	9.92E-05	1.11E-04	1.01E-04	1.36E-04
M01E11.5.2	2040	1951	2331	3358	6.13E-05	6.71E-05	5.12E-05	7.15E-05
M01E11.5.3	2043	1958	2357	3375	2.93E-04	2.60E-04	2.28E-04	3.74E-04
M01E11.6	689	735	1528	1407	2.76E-04	2.50E-04	2.05E-04	3.65E-04
M01E11.7a	358	467	294	417	2.76E-04	2.50E-04	2.07E-04	3.66E-04
M01E11.7b.1	191	226	136	186	3.82E-05	3.85E-05	5.51E-05	6.27E-05
M01E11.7b.2	187	223	134	184	1.20E-05	1.48E-05	6.41E-06	1.12E-05
M01E11.7c	341	451	280	391	1.16E-05	1.30E-05	5.38E-06	9.09E-06
M01E11.7d	183	252	165	248	1.20E-05	1.35E-05	5.59E-06	9.49E-06
M01E5.1	1	4	0	2	1.34E-05	1.67E-05	7.16E-06	1.23E-05
M01E5.2	89	104	84	104	9.86E-06	1.28E-05	5.79E-06	1.08E-05
M01E5.4	327	354	334	503	2.80E-06	2.65E-06	1.82E-06	2.25E-06
M01E5.5a	1413	1531	1961	2918	5.94E-06	6.53E-06	3.64E-06	5.56E-06
M01E5.5b	955	1009	1347	2065	3.20E-05	3.27E-05	2.13E-05	3.95E-05
M01E5.6	52	43	20	31	4.92E-05	5.03E-05	4.44E-05	8.15E-05
M01E5.7	0	1	6	7	4.62E-05	4.61E-05	4.24E-05	8.02E-05
M01E5.t1	0	0	3	0	2.80E-06	2.65E-06	1.82E-06	2.25E-06
M01F1.1	17	29	39	14	2.80E-06	2.65E-06	4.01E-06	5.78E-06
M01F1.2.1	11312	9172	11541	9822	2.80E-06	2.65E-06	2.99E-06	2.25E-06
M01F1.2.2	10197	8366	10230	9486	2.80E-06	2.65E-06	2.15E-06	2.25E-06
M01F1.3.1	11561	9621	11788	10134	1.88E-03	1.44E-03	1.25E-03	1.31E-03
M01F1.3.2	200	385	195	252	1.73E-03	1.34E-03	1.13E-03	1.29E-03
M01F1.3.3	185	365	169	243	6.18E-04	4.86E-04	4.10E-04	4.35E-04
M01F1.4a	353	470	594	932	1.98E-05	3.61E-05	1.26E-05	2.01E-05
M01F1.4b	333	442	556	895	1.94E-05	3.62E-05	1.16E-05	2.05E-05
M01F1.5.1	169	249	144	170	1.48E-05	1.87E-05	1.63E-05	3.15E-05
M01F1.5.2	162	237	139	167	1.54E-05	1.93E-05	1.67E-05	3.32E-05
M01F1.7	134	173	138	220	8.99E-06	1.25E-05	4.99E-06	7.26E-06
M01F1.8a	146	158	223	283	9.27E-06	1.28E-05	5.17E-06	7.67E-06
M01F1.8b	158	174	252	298	4.34E-06	5.29E-06	2.92E-06	5.71E-06
M01F1.9	344	278	726	821	1.52E-05	1.56E-05	1.51E-05	2.37E-05
M01G12.1	9	5	4	9	4.69E-05	4.88E-05	4.87E-05	7.11E-05
M01G12.10	3	2	4	1	6.83E-05	5.22E-05	9.38E-05	1.31E-04
M01G12.11	0	0	1	0	2.80E-06	2.65E-06	1.82E-06	2.25E-06
M01G12.12	47	53	41	66	2.80E-06	2.65E-06	1.82E-06	2.25E-06
M01G12.13	9	5	4	9	2.80E-06	2.65E-06	1.82E-06	2.25E-06
M01G12.14	8	11	7	7	2.80E-06	2.65E-06	1.82E-06	2.25E-06
M01G12.2	1	5	5	0	2.80E-06	2.65E-06	1.82E-06	2.25E-06
M01G12.3	2	1	2	3	2.80E-06	2.65E-06	1.82E-06	2.25E-06
M01G12.4	3	5	0	3	2.80E-06	2.65E-06	1.82E-06	2.25E-06
M01G12.5	1	6	5	1	2.80E-06	2.65E-06	1.82E-06	2.25E-06
M01G12.6	6	6	4	3	2.80E-06	2.65E-06	1.82E-06	2.25E-06
M01G12.7	6	5	6	4	2.80E-06	2.65E-06	1.82E-06	2.25E-06
M01G12.8	22	86	11	25	2.80E-06	2.65E-06	1.82E-06	2.25E-06
M01G12.9	31	45	15	15	2.80E-06	2.65E-06	1.82E-06	2.25E-06
M01G4.1	0	0	2	0	4.26E-06	1.57E-05	1.82E-06	3.87E-06
M01G5.1	194	172	288	505	4.23E-06	5.82E-06	1.82E-06	2.25E-06
M01G5.3	160	179	184	250	2.80E-06	2.65E-06	1.82E-06	2.25E-06
M01G5.5	106	91	384	536	8.15E-06	6.82E-06	7.85E-06	1.70E-05
M01G5.6	90	92	68	64	1.55E-05	1.64E-05	1.16E-05	1.95E-05
M01H9.1	29	42	11	12	5.54E-06	4.50E-06	1.30E-05	2.25E-05
M01H9.2	8	3	3	4	3.26E-05	3.15E-05	1.60E-05	1.86E-05
M01H9.3a.1	788	1031	862	1298	6.80E-06	9.31E-06	1.82E-06	2.27E-06
M01H9.3a.2	457	550	449	663	2.80E-06	2.65E-06	1.82E-06	2.25E-06
M01H9.3b	578	792	687	1090	7.82E-05	9.67E-05	5.57E-05	1.04E-04

M01H9.3c	457	550	449	663	6.90E-05	7.84E-05	4.41E-05	8.04E-05
M01H9.5	4	12	7	2	4.55E-05	5.89E-05	3.52E-05	6.89E-05
M02A10.1	1	0	1	2	6.90E-05	7.84E-05	4.41E-05	8.04E-05
M02A10.2	26	45	42	32	2.80E-06	2.65E-06	1.82E-06	2.25E-06
M02A10.3a	217	254	251	325	2.80E-06	2.65E-06	1.82E-06	2.25E-06
M02A10.3b	168	201	207	260	2.80E-06	2.65E-06	1.82E-06	2.25E-06
M02A10.3c	196	231	230	298	1.09E-05	1.21E-05	8.24E-06	1.32E-05
M02B1.1	47	52	79	76	1.07E-05	1.21E-05	8.60E-06	1.33E-05
M02B1.2	32	50	18	49	1.12E-05	1.25E-05	8.56E-06	1.37E-05
M02B1.3.1	1048	1005	1455	2063	5.32E-06	5.58E-06	5.83E-06	6.93E-06
M02B1.3.2	702	671	1013	1466	6.30E-06	9.28E-06	2.30E-06	7.74E-06
M02B1.4	33	36	21	14	7.20E-05	6.52E-05	6.50E-05	1.14E-04
M02B7.1	104	100	92	136	8.03E-05	7.25E-05	7.54E-05	1.35E-04
M02B7.2	86	58	93	109	3.92E-06	4.05E-06	1.82E-06	2.25E-06
M02B7.3a.1	15	6	8	5	1.27E-05	1.15E-05	7.31E-06	1.33E-05
M02B7.3a.2	14	6	8	5	1.20E-05	7.67E-06	8.45E-06	1.22E-05
M02B7.3b	15	6	8	6	2.80E-06	2.65E-06	1.82E-06	2.25E-06
M02B7.4	56	34	103	116	2.80E-06	2.65E-06	1.82E-06	2.25E-06
M02B7.5	393	391	673	983	2.80E-06	2.65E-06	1.82E-06	2.25E-06
M02B7.6	6	14	11	5	7.11E-06	4.07E-06	8.51E-06	1.18E-05
M02B7.7	17	7	59	23	1.88E-05	1.77E-05	2.10E-05	3.78E-05
M02D8.1	596	805	653	464	2.80E-06	2.65E-06	1.82E-06	2.25E-06
M02D8.2	10	14	9	14	5.82E-06	2.65E-06	1.32E-05	6.32E-06
M02D8.3	110	120	102	112	8.68E-05	1.11E-04	6.19E-05	5.43E-05
M02D8.4a	145	378	124	237	2.91E-06	3.86E-06	1.82E-06	3.28E-06
M02D8.4b.1	146	398	138	241	4.20E-06	4.34E-06	2.53E-06	3.44E-06
M02D8.4b.2	142	392	136	234	8.85E-06	2.18E-05	4.92E-06	1.16E-05
M02D8.4c	125	316	108	218	8.26E-06	2.12E-05	5.07E-06	1.09E-05
M02D8.5	23	29	24	24	8.12E-06	2.12E-05	5.07E-06	1.08E-05
M02D8.6	120	185	154	79	8.65E-06	2.07E-05	4.87E-06	1.21E-05
M02D8.7	6	9	2	5	2.80E-06	2.65E-06	1.82E-06	2.25E-06
M02E1.1a	105	161	137	202	2.56E-05	3.73E-05	2.14E-05	1.36E-05
M02E1.1b.1	62	94	76	120	2.80E-06	2.65E-06	1.82E-06	2.25E-06
M02E1.1b.2	74	117	112	136	3.28E-06	4.73E-06	2.77E-06	5.04E-06
M02E1.2	9	11	16	8	3.02E-06	4.34E-06	2.41E-06	4.70E-06
M02E1.3	30	29	44	26	3.14E-06	4.68E-06	3.10E-06	4.63E-06
M02F4.1	2	7	8	4	2.83E-06	3.25E-06	3.26E-06	2.25E-06
M02F4.2	6	1	2	2	6.44E-06	5.90E-06	6.16E-06	4.50E-06
M02F4.3	55	68	41	68	2.80E-06	2.65E-06	1.82E-06	2.25E-06
M02F4.7	269	311	255	258	2.80E-06	2.65E-06	1.82E-06	2.25E-06
M02F4.9	5	1	1	1	3.92E-06	4.55E-06	1.90E-06	3.87E-06
M02G9.1	104	102	26	34	2.69E-05	2.94E-05	1.66E-05	2.07E-05
M02G9.2	27	19	9	8	2.80E-06	2.65E-06	1.82E-06	2.25E-06
M02G9.3	10	7	2	2	3.75E-06	3.47E-06	1.82E-06	2.25E-06
M02H5.1	9	26	8	8	2.80E-06	2.65E-06	1.82E-06	2.25E-06
M02H5.10	5	7	6	4	2.80E-06	2.65E-06	1.82E-06	2.25E-06
M02H5.11	5	1	18	8	2.80E-06	2.65E-06	1.82E-06	2.25E-06
M02H5.12	5	7	11	5	2.80E-06	2.65E-06	1.82E-06	2.25E-06
M02H5.2	2	4	6	2	2.80E-06	2.65E-06	1.82E-06	2.25E-06
M02H5.3	9	26	8	17	2.80E-06	2.65E-06	1.82E-06	2.25E-06
M02H5.4	25	35	9	24	2.80E-06	2.65E-06	1.82E-06	2.25E-06
M02H5.5	14	40	31	31	2.80E-06	2.65E-06	1.82E-06	2.25E-06
M02H5.6	12	27	13	22	2.80E-06	2.96E-06	1.82E-06	2.25E-06
M02H5.7	10	17	8	13	2.80E-06	3.44E-06	1.84E-06	2.27E-06
M02H5.8	79	43	305	27	2.80E-06	2.65E-06	1.82E-06	2.25E-06
M02H5.9	5	4	1	1	2.80E-06	2.65E-06	1.82E-06	2.25E-06
M03A1.1a	695	595	1223	1706	2.34E-05	1.20E-05	5.88E-05	6.43E-06
M03A1.1b	926	803	1621	2247	2.80E-06	2.65E-06	1.82E-06	2.25E-06
M03A1.3	15	36	18	30	2.32E-05	1.88E-05	2.66E-05	4.58E-05

M03A1.6a	504	646	872	1168	2.67E-05	2.19E-05	3.04E-05	5.21E-05
M03A1.6b.1	512	661	897	1161	2.80E-06	2.65E-06	1.82E-06	2.25E-06
M03A1.6b.2	524	673	916	1171	2.34E-05	2.83E-05	2.63E-05	4.35E-05
M03A1.6c	751	896	1295	1679	2.49E-05	3.04E-05	2.84E-05	4.54E-05
M03A1.6d	513	662	897	1161	2.49E-05	3.02E-05	2.84E-05	4.47E-05
M03A1.7.1	463	881	453	103	2.88E-05	3.25E-05	3.24E-05	5.18E-05
M03A1.7.2	338	663	285	80	2.44E-05	2.97E-05	2.78E-05	4.44E-05
M03A1.t1	1	0	3	0	8.60E-05	1.55E-04	5.48E-05	1.54E-05
M03A1.t2	0	0	3	0	7.65E-05	1.42E-04	4.20E-05	1.45E-05
M03A8.1	626	1094	578	677	2.80E-06	2.65E-06	3.04E-06	2.25E-06
M03A8.2	297	516	218	493	2.80E-06	2.65E-06	3.04E-06	2.25E-06
M03A8.3	21	46	20	16	5.04E-05	8.32E-05	3.03E-05	4.38E-05
M03A8.4.1	270	300	182	259	4.54E-06	7.46E-06	2.17E-06	6.05E-06
M03A8.4.2	193	199	144	214	2.80E-06	3.39E-06	1.82E-06	2.25E-06
M03B6.1	10	24	17	11	2.00E-05	2.09E-05	8.76E-06	1.54E-05
M03B6.2	349	810	374	730	1.61E-05	1.57E-05	7.82E-06	1.43E-05
M03B6.3	109	138	61	50	2.80E-06	5.61E-06	2.73E-06	2.25E-06
M03B6.4	11	23	13	33	1.80E-05	3.95E-05	1.26E-05	3.03E-05
M03B6.5	6	6	6	3	6.05E-06	7.22E-06	2.20E-06	2.25E-06
M03C11.1	13	18	17	13	2.80E-06	2.65E-06	1.82E-06	3.22E-06
M03C11.2	173	174	302	439	2.80E-06	2.65E-06	1.82E-06	2.25E-06
M03C11.3	478	368	800	1065	2.80E-06	2.65E-06	1.82E-06	2.25E-06
M03C11.4	437	378	1255	1040	7.45E-06	7.06E-06	8.45E-06	1.52E-05
M03C11.5.1	399	429	395	641	1.95E-05	1.42E-05	2.12E-05	3.49E-05
M03C11.5.2	352	354	336	567	2.23E-05	1.82E-05	4.16E-05	4.26E-05
M03C11.6	30	34	37	75	1.78E-05	1.81E-05	1.15E-05	2.30E-05
M03C11.7.1	339	382	331	515	1.93E-05	1.83E-05	1.20E-05	2.50E-05
M03C11.8	732	731	733	1279	3.72E-06	3.99E-06	2.99E-06	7.47E-06
M03D4.1a	945	876	1456	1801	1.94E-05	2.07E-05	1.24E-05	2.37E-05
M03D4.1b	916	846	1423	1756	2.38E-05	2.25E-05	1.55E-05	3.35E-05
M03D4.1c	1056	962	1602	1999	4.55E-05	3.98E-05	4.56E-05	6.96E-05
M03D4.3	8	3	6	2	4.42E-05	3.86E-05	4.47E-05	6.81E-05
M03D4.4a	28	59	39	58	4.45E-05	3.83E-05	4.39E-05	6.76E-05
M03D4.4b.1	20	38	22	40	4.98E-06	2.65E-06	2.42E-06	2.25E-06
M03D4.4b.2	20	36	20	36	2.80E-06	3.33E-06	1.82E-06	2.79E-06
M03D4.5	2	2	3	3	2.80E-06	2.65E-06	1.82E-06	2.36E-06
M03D4.6	7	8	7	12	2.80E-06	2.78E-06	1.82E-06	2.36E-06
M03E7.1	4	11	4	1	2.80E-06	2.65E-06	1.82E-06	2.25E-06
M03E7.2	9	18	1	4	2.80E-06	2.65E-06	1.82E-06	2.25E-06
M03E7.3	3	5	4	2	2.80E-06	2.65E-06	1.82E-06	2.25E-06
M03E7.4	26	37	9	8	2.80E-06	4.23E-06	1.82E-06	2.25E-06
M03E7.5.1	178	203	169	160	2.80E-06	2.65E-06	1.82E-06	2.25E-06
M03E7.5.2	165	179	145	143	3.86E-06	5.21E-06	1.82E-06	2.25E-06
M03F4.2b.1	13080	12550	13057	19533	2.77E-05	2.99E-05	1.71E-05	2.00E-05
M03F4.2b.2	13090	12563	13058	19536	2.24E-05	2.29E-05	1.28E-05	1.56E-05
M03F4.2c	12493	11968	12336	18991	1.25E-03	1.13E-03	8.11E-04	1.50E-03
M03F4.3a	7	18	17	16	1.25E-03	1.13E-03	8.11E-04	1.50E-03
M03F4.3b	6	13	12	15	1.17E-03	1.05E-03	7.49E-04	1.42E-03
M03F4.3c.1	6	13	11	14	2.80E-06	2.65E-06	1.82E-06	2.25E-06
M03F4.3c.2	6	13	11	14	2.80E-06	2.65E-06	1.82E-06	2.25E-06
M03F4.4	4	3	2	1	2.80E-06	2.65E-06	1.82E-06	2.25E-06
M03F4.6	1126	1678	1208	1823	2.80E-06	2.65E-06	1.82E-06	2.25E-06
M03F4.7a	1320	1443	1447	1335	2.80E-06	2.65E-06	1.82E-06	2.25E-06
M03F4.7b	1136	1241	1157	1193	9.26E-05	1.30E-04	6.46E-05	1.20E-04
M03F8.1	24	63	12	12	1.22E-04	1.26E-04	8.70E-05	9.90E-05
M03F8.2a	288	314	452	615	1.03E-04	1.06E-04	6.82E-05	8.68E-05
M03F8.2b	273	304	413	602	2.80E-06	6.43E-06	1.82E-06	2.25E-06
M03F8.2c	288	301	408	599	2.49E-05	2.57E-05	2.55E-05	4.28E-05
M03F8.3	614	773	722	990	2.31E-05	2.43E-05	2.28E-05	4.09E-05

M03F8.4	25	16	13	15	2.01E-05	1.98E-05	1.85E-05	3.36E-05
M03F8.5	13	12	17	26	2.84E-05	3.38E-05	2.17E-05	3.68E-05
M03F8.6	19	16	13	12	3.39E-06	2.65E-06	1.82E-06	2.25E-06
M04B2.1	1282	1275	1704	2513	2.80E-06	2.65E-06	1.82E-06	2.25E-06
M04B2.2	79	121	79	118	2.97E-06	2.65E-06	1.82E-06	2.25E-06
M04B2.3	122	151	103	143	4.70E-05	4.42E-05	4.07E-05	7.41E-05
M04B2.4.1	229	298	189	272	7.53E-06	1.09E-05	4.90E-06	9.02E-06
M04B2.4.2	236	287	211	276	2.15E-05	2.51E-05	1.18E-05	2.02E-05
M04B2.5	29	38	16	35	1.47E-05	1.81E-05	7.91E-06	1.41E-05
M04B2.6	10	14	8	4	1.67E-05	1.92E-05	9.69E-06	1.57E-05
M04B2.7	4	10	5	6	2.80E-06	2.65E-06	1.82E-06	2.25E-06
M04C3.1a	77	145	87	173	4.06E-06	5.37E-06	2.11E-06	2.25E-06
M04C3.1b	105	201	116	201	2.80E-06	2.65E-06	1.82E-06	2.25E-06
M04C3.2	71	101	42	47	3.02E-06	5.40E-06	2.22E-06	5.49E-06
M04C3.3	25	49	28	34	4.20E-06	7.62E-06	3.02E-06	6.48E-06
M04C7.1	6	18	13	9	3.86E-06	5.18E-06	1.82E-06	2.25E-06
M04C7.3	1	4	2	0	4.17E-06	7.72E-06	3.04E-06	4.57E-06
M04C7.4	0	2	4	1	2.80E-06	2.65E-06	1.82E-06	2.25E-06
M04C9.2	2	2	8	5	2.80E-06	2.65E-06	1.82E-06	2.25E-06
M04C9.3	50	67	37	57	2.80E-06	2.65E-06	1.82E-06	2.25E-06
M04C9.4	18	20	19	13	2.80E-06	2.65E-06	3.13E-06	2.43E-06
M04C9.5	15	14	15	15	7.70E-06	9.73E-06	3.70E-06	7.04E-06
M04D5.1	40	34	73	52	2.80E-06	2.65E-06	1.82E-06	2.25E-06
M04D5.2	2	2	2	3	2.80E-06	2.65E-06	1.82E-06	2.25E-06
M04D8.1	4	4	14	2	2.80E-06	2.65E-06	1.82E-06	2.25E-06
M04D8.3	3	2	3	0	2.80E-06	2.65E-06	1.82E-06	2.25E-06
M04D8.4	4	3	0	6	2.80E-06	2.65E-06	4.41E-06	2.25E-06
M04D8.5	3	0	1	1	2.80E-06	2.65E-06	1.82E-06	2.25E-06
M04D8.6	2	9	6	3	2.80E-06	2.65E-06	1.82E-06	2.25E-06
M04D8.7	5	8	9	3	2.80E-06	2.65E-06	1.82E-06	2.25E-06
M04D8.8	3	0	6	1	2.80E-06	2.65E-06	1.82E-06	2.25E-06
M04F3.1.1	542	689	899	673	2.80E-06	2.65E-06	1.82E-06	2.25E-06
M04F3.1.2	489	617	771	605	2.80E-06	2.65E-06	1.82E-06	2.25E-06
M04F3.2	15	13	5	10	6.27E-05	7.53E-05	6.77E-05	6.25E-05
M04F3.3	88	101	85	63	6.23E-05	7.43E-05	6.39E-05	6.19E-05
M04F3.4	99	145	160	145	2.80E-06	2.65E-06	1.82E-06	2.25E-06
M04F3.5	475	432	574	844	7.48E-06	8.09E-06	4.70E-06	4.30E-06
M04F3.6	225	208	314	325	2.14E-05	2.96E-05	2.25E-05	2.51E-05
M04G12.1a	166	241	166	201	2.44E-05	2.09E-05	1.92E-05	3.48E-05
M04G12.1b	157	224	158	190	5.87E-05	5.13E-05	5.34E-05	6.81E-05
M04G12.2	460	778	424	352	1.00E-05	1.37E-05	6.51E-06	9.72E-06
M04G12.3	19	20	19	10	9.97E-06	1.34E-05	6.52E-06	9.69E-06
M04G12.4a	166	192	114	188	3.33E-05	5.32E-05	2.00E-05	2.05E-05
M04G12.4b.1	217	254	136	253	2.80E-06	2.65E-06	1.82E-06	2.25E-06
M04G12.4b.2	208	241	130	251	1.06E-05	1.16E-05	4.76E-06	9.67E-06
M04G12.4b.3	157	178	108	186	1.04E-05	1.16E-05	4.26E-06	9.78E-06
M04G7.1	20	45	22	26	1.01E-05	1.11E-05	4.12E-06	9.81E-06
M04G7.2	18	18	10	5	1.06E-05	1.13E-05	4.74E-06	1.01E-05
M04G7.3a	20	33	17	25	2.80E-06	5.92E-06	1.99E-06	2.90E-06
M04G7.3b.1	20	34	17	26	2.80E-06	2.65E-06	1.82E-06	2.25E-06
M04G7.3b.2	18	28	16	22	2.80E-06	2.65E-06	1.82E-06	2.25E-06
M05B5.1	25	60	44	27	2.80E-06	2.65E-06	1.82E-06	2.25E-06
M05B5.2	99	167	50	91	2.80E-06	2.65E-06	1.82E-06	2.25E-06
M05B5.3	34	41	51	26	2.80E-06	4.73E-06	2.41E-06	2.25E-06
M05B5.4	99	191	117	220	1.27E-05	2.03E-05	4.17E-06	9.38E-06
M05D6.1	36	73	24	8	2.91E-06	3.31E-06	2.84E-06	2.25E-06
M05D6.10	2	7	9	7	8.74E-06	1.59E-05	6.71E-06	1.56E-05
M05D6.2	476	592	497	797	3.72E-06	7.12E-06	1.82E-06	2.25E-06
M05D6.3	26	47	16	13	2.80E-06	2.65E-06	1.82E-06	2.25E-06

M05D6.4	48	152	37	54	3.05E-05	3.59E-05	2.08E-05	4.11E-05
M05D6.5.1	147	186	125	170	2.80E-06	4.15E-06	1.82E-06	2.25E-06
M05D6.5.2	116	149	90	119	4.09E-06	1.22E-05	2.06E-06	3.71E-06
M05D6.6.1	146	191	336	153	2.30E-05	2.75E-05	1.27E-05	2.13E-05
M05D6.6.2	137	188	323	151	2.20E-05	2.67E-05	1.11E-05	1.81E-05
M05D6.7	23	109	25	42	2.02E-05	2.50E-05	3.03E-05	1.70E-05
M05D6.8	6	7	5	3	2.04E-05	2.64E-05	3.12E-05	1.80E-05
M05D6.9	13	37	12	8	2.80E-06	9.36E-06	1.82E-06	3.08E-06
M106.1	805	840	707	1399	2.80E-06	2.65E-06	1.82E-06	2.25E-06
M106.2	365	330	495	558	2.80E-06	4.79E-06	1.82E-06	2.25E-06
M106.4a	784	727	949	1236	2.25E-05	2.21E-05	1.28E-05	3.14E-05
M106.4b	652	619	774	1045	2.31E-05	1.97E-05	2.04E-05	2.83E-05
M110.1	87	132	120	100	3.05E-05	2.67E-05	2.40E-05	3.86E-05
M110.2	4	9	5	5	3.06E-05	2.75E-05	2.37E-05	3.94E-05
M110.3	140	204	288	265	6.08E-06	8.70E-06	5.45E-06	5.60E-06
M110.4a	3504	2360	4922	5443	2.80E-06	2.65E-06	1.82E-06	2.25E-06
M110.4b	2257	1880	2758	4435	2.75E-05	3.79E-05	3.68E-05	4.18E-05
M110.5a.1	2634	2128	3226	4535	8.49E-05	5.40E-05	7.76E-05	1.06E-04
M110.5a.2	1970	1604	2515	3326	7.24E-05	5.70E-05	5.76E-05	1.14E-04
M110.5b	1996	1606	2540	3372	1.34E-04	1.03E-04	1.07E-04	1.86E-04
M110.5c	1844	1499	2374	3147	1.48E-04	1.14E-04	1.23E-04	2.01E-04
M110.7	30	44	25	15	1.50E-04	1.14E-04	1.24E-04	2.03E-04
M110.8	4	1	2	1	1.34E-04	1.03E-04	1.12E-04	1.83E-04
M110.9	14	14	5	4	2.80E-06	2.65E-06	1.82E-06	2.25E-06
M116.1	1	3	1	4	2.80E-06	2.65E-06	1.82E-06	2.25E-06
M116.2	24	24	25	25	3.61E-06	3.41E-06	1.82E-06	2.25E-06
M116.4	22	38	18	29	2.80E-06	2.65E-06	1.82E-06	2.25E-06
M116.5	508	555	620	1037	3.22E-06	3.04E-06	2.19E-06	2.70E-06
M117.1	48	99	80	147	2.80E-06	4.07E-06	1.82E-06	2.63E-06
M117.2.1	4377	5100	5163	5686	1.63E-05	1.68E-05	1.29E-05	2.67E-05
M117.2.2	4244	4915	4914	5494	3.19E-06	6.19E-06	3.44E-06	7.83E-06
M117.2.3	3217	3772	3887	4546	3.89E-04	4.29E-04	2.99E-04	4.06E-04
M117.3	26	21	5	6	4.37E-04	4.78E-04	3.29E-04	4.54E-04
M117.4	29	45	31	13	4.70E-04	5.20E-04	3.69E-04	5.33E-04
M117.5	9	7	6	12	7.70E-06	5.87E-06	1.82E-06	2.25E-06
M117.6	25	32	47	35	2.80E-06	2.65E-06	1.82E-06	2.25E-06
M117.7	5	8	4	8	2.80E-06	2.65E-06	1.82E-06	2.25E-06
M142.1	57	65	54	84	7.48E-06	9.02E-06	9.13E-06	8.39E-06
M142.3	6	8	2	2	2.80E-06	2.65E-06	1.82E-06	2.25E-06
M142.4	5	9	5	5	8.34E-06	8.97E-06	5.14E-06	9.87E-06
M142.5	43	60	230	129	2.80E-06	2.65E-06	1.82E-06	2.25E-06
M142.6	604	535	937	1350	2.80E-06	2.65E-06	1.82E-06	2.25E-06
M142.7	11	7	16	7	5.32E-06	7.01E-06	1.85E-05	1.28E-05
M142.8	150	116	207	171	2.10E-05	1.76E-05	2.12E-05	3.78E-05
M151.1	5	7	7	3	2.80E-06	2.65E-06	2.04E-06	2.25E-06
M151.2	29	29	42	51	2.12E-05	1.54E-05	1.90E-05	1.94E-05
M151.3	74	59	90	114	2.80E-06	2.65E-06	1.82E-06	2.25E-06
M151.4	23	20	28	20	2.80E-06	2.65E-06	1.82E-06	2.27E-06
M151.5	5	8	7	4	3.30E-06	2.65E-06	2.61E-06	4.09E-06
M151.6	2	3	3	4	2.80E-06	2.65E-06	1.82E-06	2.25E-06
M151.7	116	89	174	219	2.80E-06	2.65E-06	1.82E-06	2.25E-06
M151.8	6	1	3	1	2.80E-06	2.65E-06	1.82E-06	2.25E-06
M153.1	121	176	74	81	9.66E-06	6.98E-06	9.42E-06	1.46E-05
M153.2	17	17	25	18	2.80E-06	2.65E-06	1.82E-06	2.25E-06
M153.3	6	8	5	4	1.05E-05	1.44E-05	4.17E-06	5.65E-06
M153.4	2	5	4	1	2.80E-06	2.65E-06	1.82E-06	2.25E-06
M162.1	2	3	3	2	2.80E-06	2.65E-06	1.82E-06	2.25E-06
M162.10	17	22	21	23	2.80E-06	2.65E-06	1.82E-06	2.25E-06
M162.11	4	2	2	4	2.80E-06	2.65E-06	1.82E-06	2.25E-06

M162.2	31	35	14	19	2.80E-06	2.65E-06	1.82E-06	2.25E-06
M162.3	2	5	3	1	2.80E-06	2.65E-06	1.82E-06	2.25E-06
M162.4	4	0	3	2	6.78E-06	7.22E-06	1.99E-06	3.33E-06
M162.5.1	12	21	12	16	2.80E-06	2.65E-06	1.82E-06	2.25E-06
M162.5.2	12	18	12	16	2.80E-06	2.65E-06	1.82E-06	2.25E-06
M162.6	1	1	0	0	2.80E-06	2.65E-06	1.82E-06	2.25E-06
M162.7	26	55	19	22	2.80E-06	2.65E-06	1.82E-06	2.25E-06
M162.8	5	5	7	0	2.80E-06	2.65E-06	1.82E-06	2.25E-06
M162.9	20	44	56	28	2.80E-06	2.80E-06	1.82E-06	2.25E-06
M163.1	105	188	113	141	2.80E-06	2.65E-06	1.82E-06	2.25E-06
M163.10	1	1	0	0	2.80E-06	2.96E-06	2.59E-06	2.25E-06
M163.11	4	5	7	6	1.08E-05	1.84E-05	7.60E-06	1.17E-05
M163.2	12	20	26	13	2.80E-06	2.65E-06	1.82E-06	2.25E-06
M163.3	241	191	181	307	2.80E-06	2.65E-06	1.82E-06	2.25E-06
M163.4	79	114	106	190	2.80E-06	2.65E-06	1.82E-06	2.25E-06
M163.5	8	4	5	5	2.99E-05	2.24E-05	1.46E-05	3.06E-05
M163.6	0	1	0	0	3.42E-06	4.66E-06	2.99E-06	6.61E-06
M163.7	3	5	6	1	2.80E-06	2.65E-06	1.82E-06	2.25E-06
M163.8	14	22	21	9	2.80E-06	2.65E-06	1.82E-06	2.25E-06
M163.9	3	0	0	0	2.80E-06	2.65E-06	1.82E-06	2.25E-06
M163.t1	0	0	9	0	2.80E-06	3.44E-06	2.26E-06	2.25E-06
M163.t2	0	0	3	0	2.80E-06	2.65E-06	1.82E-06	2.25E-06
M176.1	3	3	4	2	2.80E-06	2.65E-06	8.98E-06	2.25E-06
M176.10	8	7	10	10	2.80E-06	2.65E-06	2.99E-06	2.25E-06
M176.11	51	56	80	55	2.80E-06	2.65E-06	1.82E-06	2.25E-06
M176.2.1	396	488	464	699	2.80E-06	2.65E-06	1.82E-06	2.25E-06
M176.2.2	375	446	410	666	8.62E-06	8.94E-06	8.80E-06	7.47E-06
M176.3.1	404	420	559	596	2.77E-05	3.22E-05	2.11E-05	3.92E-05
M176.3.2	383	385	495	551	2.85E-05	3.20E-05	2.03E-05	4.06E-05
M176.4	60	145	62	82	6.34E-05	6.22E-05	5.71E-05	7.51E-05
M176.5	33	67	24	38	6.36E-05	6.04E-05	5.35E-05	7.35E-05
M176.6a	29	118	32	100	8.15E-06	1.86E-05	5.47E-06	8.93E-06
M176.6b	33	116	52	94	4.17E-06	7.99E-06	1.97E-06	3.85E-06
M176.7	51	50	24	29	2.80E-06	8.57E-06	1.82E-06	6.18E-06
M176.8	30	46	32	53	2.80E-06	8.94E-06	2.77E-06	6.16E-06
M176.9	28	44	21	19	3.56E-06	3.31E-06	1.82E-06	2.25E-06
M18.1	3308	3354	4099	2815	2.80E-06	3.65E-06	1.82E-06	3.58E-06
M18.2	28	70	63	30	2.80E-06	2.65E-06	1.82E-06	2.25E-06
M18.3	136	167	175	207	3.53E-04	3.38E-04	2.84E-04	2.41E-04
M18.5	699	1015	809	1248	6.83E-06	1.61E-05	1.00E-05	5.87E-06
M18.6	175	176	188	209	1.07E-05	1.24E-05	8.93E-06	1.30E-05
M18.8	357	452	415	515	2.05E-05	2.81E-05	1.54E-05	2.93E-05
M195.1	1053	1159	522	451	1.57E-05	1.49E-05	1.10E-05	1.51E-05
M195.2	32	48	24	13	2.83E-05	3.38E-05	2.14E-05	3.27E-05
M195.4	7	7	5	9	1.20E-04	1.25E-04	3.87E-05	4.12E-05
M199.1	2	0	2	1	2.80E-06	3.57E-06	1.82E-06	2.25E-06
M199.2	78	53	48	46	2.80E-06	2.65E-06	1.82E-06	2.25E-06
M199.3	7	10	9	8	2.80E-06	2.65E-06	1.82E-06	2.25E-06
M199.4	12	117	15	61	6.50E-06	4.15E-06	2.61E-06	3.08E-06
M199.5	257	357	73	301	3.84E-06	5.18E-06	3.21E-06	3.53E-06
M199.6	6	4	9	6	2.80E-06	1.76E-05	1.82E-06	7.83E-06
M199.7	2	3	4	5	1.45E-05	1.90E-05	2.68E-06	1.37E-05
M199.8	2	2	0	1	2.80E-06	2.65E-06	1.82E-06	2.25E-06
M28.1	24	21	15	17	2.80E-06	2.65E-06	1.82E-06	2.25E-06
M28.10	355	433	746	589	2.80E-06	2.65E-06	1.82E-06	2.25E-06
M28.2	35	38	31	31	2.80E-06	2.65E-06	1.82E-06	2.25E-06
M28.4	16	29	14	6	4.42E-05	5.09E-05	6.04E-05	5.89E-05
M28.5.1	2494	2607	4900	2513	2.80E-06	2.65E-06	1.82E-06	2.25E-06
M28.5.2	1802	1860	3478	1846	2.80E-06	2.65E-06	1.82E-06	2.25E-06

M28.6	147	197	140	209	5.33E-04	5.26E-04	6.82E-04	4.31E-04
M28.7	15	16	15	8	3.80E-04	3.71E-04	4.77E-04	3.13E-04
M28.8	83	113	76	170	9.94E-06	1.26E-05	6.16E-06	1.14E-05
M28.9.1	93	137	38	60	2.80E-06	2.65E-06	1.82E-06	2.25E-06
M28.9.2	95	138	39	63	3.53E-06	4.55E-06	2.11E-06	5.82E-06
M4.1	247	189	524	612	3.92E-06	5.48E-06	1.82E-06	2.25E-06
M4.2	6	8	3	3	4.03E-06	5.55E-06	1.82E-06	2.25E-06
M57.1	77	98	62	94	1.58E-05	1.14E-05	2.18E-05	3.15E-05
M57.2.1	324	382	517	776	2.80E-06	2.65E-06	1.82E-06	2.25E-06
M57.2.2	312	354	507	773	4.20E-06	5.05E-06	2.20E-06	4.12E-06
M6.1a	1141	1268	1174	1491	1.81E-05	2.02E-05	1.88E-05	3.49E-05
M6.1b	946	1092	1043	1332	2.00E-05	2.14E-05	2.11E-05	3.97E-05
M6.1c	66	91	68	123	3.26E-05	3.42E-05	2.18E-05	3.42E-05
M6.3	3	2	4	0	2.80E-05	3.05E-05	2.01E-05	3.17E-05
M6.4	7	13	3	9	3.58E-06	4.68E-06	2.41E-06	5.38E-06
M60.2.1	688	970	517	697	2.80E-06	2.65E-06	1.82E-06	2.25E-06
M60.2.2	689	971	517	700	2.80E-06	2.65E-06	1.82E-06	2.25E-06
M60.4a	198	315	220	200	4.28E-05	5.71E-05	2.10E-05	3.49E-05
M60.4b.1	203	366	245	222	4.43E-05	5.90E-05	2.16E-05	3.62E-05
M60.4b.2	195	335	212	213	3.84E-05	5.78E-05	2.78E-05	3.12E-05
M60.4b.3	198	315	220	200	3.68E-05	6.27E-05	2.89E-05	3.23E-05
M60.5	59	92	77	78	3.63E-05	5.89E-05	2.57E-05	3.18E-05
M60.6	22	61	23	27	3.66E-05	5.50E-05	2.65E-05	2.97E-05
M60.7	4	10	7	4	2.94E-06	4.34E-06	2.50E-06	3.13E-06
M7.1	3967	3632	4005	5957	2.80E-06	5.45E-06	1.82E-06	2.25E-06
M7.10	3	9	10	5	2.80E-06	2.65E-06	1.82E-06	2.25E-06
M7.12	17	38	10	28	4.51E-04	3.90E-04	2.96E-04	5.44E-04
M7.13	4	2	8	4	2.80E-06	2.65E-06	1.82E-06	2.25E-06
M7.2	629	671	731	1128	2.80E-06	5.53E-06	1.82E-06	3.46E-06
M7.3	36	59	37	48	2.80E-06	2.65E-06	1.82E-06	2.25E-06
M7.5.1	284	450	416	655	4.07E-05	4.10E-05	3.08E-05	5.87E-05
M7.5.2	270	434	386	618	2.80E-06	2.78E-06	1.82E-06	2.25E-06
M7.7	13	32	7	14	1.52E-05	2.28E-05	1.45E-05	2.82E-05
M7.8	12	13	12	7	1.55E-05	2.36E-05	1.44E-05	2.85E-05
M7.9	3	7	7	7	2.80E-06	3.09E-06	1.82E-06	2.25E-06
M70.1	71	125	50	48	2.80E-06	2.65E-06	1.82E-06	2.25E-06
M70.2	12	6	10	9	2.80E-06	2.65E-06	1.82E-06	2.25E-06
M70.3a	90	171	71	63	2.80E-06	4.66E-06	1.82E-06	2.25E-06
M70.3b	91	168	71	64	2.80E-06	2.65E-06	1.82E-06	2.25E-06
M70.4	206	213	279	413	3.64E-06	6.53E-06	1.86E-06	2.25E-06
M70.5	91	112	159	297	3.75E-06	6.53E-06	1.90E-06	2.25E-06
M79.1a	130	167	143	259	8.90E-06	8.70E-06	7.85E-06	1.43E-05
M79.1b	154	200	171	288	5.88E-06	6.82E-06	6.69E-06	1.54E-05
M79.1c	127	158	138	250	3.92E-06	4.76E-06	2.81E-06	6.27E-06
M79.2	44	120	34	59	4.31E-06	5.29E-06	3.12E-06	6.45E-06
M79.3	28	58	24	38	3.92E-06	4.60E-06	2.77E-06	6.18E-06
M79.4	23	33	47	15	5.80E-06	1.49E-05	2.92E-06	6.23E-06
M88.1.1	409	449	471	637	5.38E-06	1.05E-05	3.01E-06	5.87E-06
M88.1.2	342	364	350	491	4.76E-06	6.45E-06	6.32E-06	2.50E-06
M88.2	187	258	242	217	2.68E-05	2.78E-05	2.01E-05	3.35E-05
M88.3	13	46	21	15	2.40E-05	2.41E-05	1.60E-05	2.76E-05
M88.4	9	18	10	10	2.66E-05	3.47E-05	2.24E-05	2.48E-05
M88.5a	1898	1238	2113	2794	2.80E-06	4.58E-06	1.82E-06	2.25E-06
M88.5b	1297	848	1250	1883	2.80E-06	2.65E-06	1.82E-06	2.25E-06
M88.6a	498	680	488	477	8.40E-05	5.18E-05	6.08E-05	9.93E-05
M88.6b	427	575	400	381	6.87E-05	4.24E-05	4.31E-05	8.01E-05
M88.7	231	269	336	297	2.49E-05	3.21E-05	1.59E-05	1.91E-05
MTCE.1	0	1	0	0	2.66E-05	3.38E-05	1.62E-05	1.90E-05
MTCE.10	15	27	30	22	2.03E-05	2.24E-05	1.92E-05	2.10E-05

MTCE.11	4135	3884	6929	6273	2.80E-06	2.65E-06	1.82E-06	2.25E-06
MTCE.12	7936	10074	14526	7572	3.05E-05	5.19E-05	3.98E-05	3.60E-05
MTCE.13	1	0	0	2	5.31E-04	4.71E-04	5.78E-04	6.46E-04
MTCE.14	0	0	3	0	1.48E-03	1.77E-03	1.76E-03	1.13E-03
MTCE.15	13	44	50	32	2.80E-06	2.65E-06	1.82E-06	2.90E-06
MTCE.16	2190	3125	2257	2226	2.80E-06	2.65E-06	4.05E-06	2.25E-06
MTCE.17	1	0	2	1	2.65E-05	8.46E-05	6.63E-05	5.23E-05
MTCE.18	0	0	1	0	2.78E-04	3.75E-04	1.87E-04	2.27E-04
MTCE.19	1	0	5	2	2.80E-06	2.65E-06	2.42E-06	2.25E-06
MTCE.2	0	0	2	1	2.80E-06	2.65E-06	1.82E-06	2.25E-06
MTCE.20	0	0	0	1	2.80E-06	2.65E-06	6.74E-06	3.33E-06
MTCE.21	17131	15822	14204	15616	2.80E-06	2.65E-06	2.70E-06	2.25E-06
MTCE.22	2	4	3	13	2.80E-06	2.65E-06	1.82E-06	2.25E-06
MTCE.23	14062	9543	16400	10967	1.72E-03	1.50E-03	9.26E-04	1.26E-03
MTCE.24	13	7	26	25	4.00E-06	7.56E-06	3.90E-06	2.09E-05
MTCE.25	3143	3777	3156	6054	2.04E-03	1.31E-03	1.55E-03	1.28E-03
MTCE.26	16207	17101	23716	30151	2.65E-05	1.35E-05	3.45E-05	4.09E-05
MTCE.27	2	0	0	0	2.70E-04	3.07E-04	1.77E-04	4.18E-04
MTCE.29	0	1	3	0	1.13E-03	1.13E-03	1.08E-03	1.69E-03
MTCE.3	2170	2951	3314	1562	4.06E-06	2.65E-06	1.82E-06	2.25E-06
MTCE.30	6	5	8	13	2.80E-06	2.65E-06	4.05E-06	2.25E-06
MTCE.31	9072	10304	11465	9000	5.56E-04	7.14E-04	5.53E-04	3.22E-04
MTCE.32	0	0	0	1	1.22E-05	9.63E-06	1.06E-05	2.13E-05
MTCE.33	245	629	287	616	1.45E-03	1.56E-03	1.19E-03	1.16E-03
MTCE.34	2255	3181	2438	1650	2.80E-06	2.65E-06	1.82E-06	2.25E-06
MTCE.35	3635	5338	2763	6332	2.88E-05	6.99E-05	2.20E-05	5.82E-05
MTCE.36	1	0	0	0	7.54E-04	1.00E-03	5.30E-04	4.43E-04
MTCE.4	3862	1242	11417	3922	2.57E-04	3.57E-04	1.27E-04	3.60E-04
MTCE.5	1	3	2	4	2.80E-06	2.65E-06	1.82E-06	2.25E-06
MTCE.6	1	2	0	2	1.84E-03	5.59E-04	3.54E-03	1.50E-03
MTCE.7	279	383	388	392	2.80E-06	5.66E-06	2.61E-06	6.43E-06
PAR2.1.1	382	336	472	374	2.80E-06	3.84E-06	1.82E-06	3.26E-06
PAR2.1.2	316	274	380	331	4.49E-05	5.82E-05	4.06E-05	5.07E-05
PAR2.3a	284	369	322	553	7.08E-05	5.89E-05	5.70E-05	5.57E-05
PAR2.3b.1	259	318	295	508	6.40E-05	5.24E-05	5.01E-05	5.38E-05
PAR2.3b.2	337	419	380	623	1.76E-05	2.16E-05	1.30E-05	2.75E-05
PAR2.4a.1	990	804	1262	1597	1.66E-05	1.92E-05	1.23E-05	2.61E-05
PAR2.4a.2	997	813	1272	1601	1.85E-05	2.17E-05	1.36E-05	2.75E-05
PAR2.4a.3	885	704	1111	1423	3.91E-05	3.00E-05	3.24E-05	5.06E-05
PAR2.4b	852	674	1052	1363	4.23E-05	3.26E-05	3.51E-05	5.46E-05
PDB1.1a	77	98	68	99	3.15E-05	2.37E-05	2.57E-05	4.07E-05
PDB1.1c	19	28	25	26	3.38E-05	2.52E-05	2.71E-05	4.34E-05
R01B10.1b	501	536	326	182	8.40E-06	1.01E-05	4.83E-06	8.68E-06
R01B10.2	1	2	1	1	6.33E-06	8.81E-06	5.43E-06	6.97E-06
R01B10.3	52	63	172	54	1.52E-04	1.54E-04	6.44E-05	4.44E-05
R01B10.4	64	99	84	67	2.80E-06	2.65E-06	1.82E-06	2.25E-06
R01B10.5.1	369	405	469	652	1.96E-05	2.24E-05	4.22E-05	1.64E-05
R01B10.5.2	211	238	269	396	7.45E-06	1.09E-05	6.36E-06	6.25E-06
R01B10.6	145	170	144	212	2.47E-05	2.56E-05	2.04E-05	3.50E-05
R01E6.1	9	9	12	7	2.34E-05	2.50E-05	1.94E-05	3.53E-05
R01E6.2	13	15	6	4	1.72E-05	1.90E-05	1.11E-05	2.02E-05
R01E6.3a	589	1269	556	1765	2.80E-06	2.65E-06	1.82E-06	2.25E-06
R01E6.3b	841	1868	854	2531	2.91E-06	3.17E-06	1.82E-06	2.25E-06
R01E6.4	16	23	19	8	7.63E-05	1.55E-04	4.68E-05	1.84E-04
R01E6.5	11	8	6	6	9.60E-05	2.01E-04	6.34E-05	2.32E-04
R01E6.6	11	21	15	13	2.80E-06	2.65E-06	1.82E-06	2.25E-06
R01E6.7	7	9	3	6	2.80E-06	2.65E-06	1.82E-06	2.25E-06
R01H10.1	348	360	508	616	2.80E-06	2.65E-06	1.82E-06	2.25E-06
R01H10.3b	95	137	80	148	2.80E-06	2.65E-06	1.82E-06	2.25E-06

R01H10.3c	107	154	86	155	1.75E-05	1.71E-05	1.66E-05	2.49E-05
R01H10.3d	99	139	81	148	6.24E-06	8.49E-06	3.41E-06	7.80E-06
R01H10.4	28	34	22	35	6.24E-06	8.52E-06	3.28E-06	7.29E-06
R01H10.5	9	9	14	4	6.22E-06	8.23E-06	3.30E-06	7.44E-06
R01H10.6	6	9	9	7	4.26E-06	4.87E-06	2.17E-06	4.27E-06
R01H10.7	354	229	620	669	2.80E-06	2.65E-06	1.82E-06	2.25E-06
R01H10.8	356	309	471	654	2.80E-06	2.65E-06	1.82E-06	2.25E-06
R01H2.1	2	4	3	0	1.63E-05	9.95E-06	1.85E-05	2.47E-05
R01H2.2	23	23	8	12	1.31E-05	1.07E-05	1.13E-05	1.93E-05
R01H2.3	799	821	1171	1490	2.80E-06	2.65E-06	1.82E-06	2.25E-06
R01H2.4	13	13	6	8	2.80E-06	2.65E-06	1.82E-06	2.25E-06
R01H2.5	170	214	116	196	5.43E-05	5.27E-05	5.18E-05	8.14E-05
R01H2.6	817	707	1197	1110	2.80E-06	2.65E-06	1.82E-06	2.25E-06
R01H2.7	8	16	9	11	2.01E-05	2.39E-05	8.91E-06	1.86E-05
R01H5.t1	0	0	1	0	1.41E-04	1.15E-04	1.35E-04	1.54E-04
R02C2.1	13	17	10	12	2.80E-06	3.20E-06	1.82E-06	2.25E-06
R02C2.2	3	3	3	6	2.80E-06	2.65E-06	1.82E-06	2.25E-06
R02C2.3	6	6	5	9	2.80E-06	2.65E-06	1.82E-06	2.25E-06
R02C2.4	17	21	27	20	2.80E-06	2.65E-06	1.82E-06	2.25E-06
R02C2.5	5	1	9	8	2.80E-06	2.65E-06	1.82E-06	2.25E-06
R02C2.6	4	3	8	2	2.80E-06	2.65E-06	1.82E-06	2.25E-06
R02C2.7	8	23	11	5	2.80E-06	2.65E-06	1.82E-06	2.25E-06
R02D1.1	28	36	25	23	2.80E-06	2.65E-06	1.82E-06	2.25E-06
R02D3.1	755	1696	1021	1875	2.80E-06	4.50E-06	1.82E-06	2.25E-06
R02D3.2	284	348	365	506	2.80E-06	2.65E-06	1.82E-06	2.25E-06
R02D3.3.1	207	221	301	333	2.74E-05	5.81E-05	2.41E-05	5.47E-05
R02D3.4	334	375	425	643	1.27E-05	1.48E-05	1.07E-05	1.83E-05
R02D3.5.1	374	353	444	540	1.43E-05	1.44E-05	1.35E-05	1.84E-05
R02D3.5.2	327	307	354	461	1.55E-05	1.65E-05	1.29E-05	2.40E-05
R02D3.6	5	10	1	4	3.81E-05	3.40E-05	2.95E-05	4.42E-05
R02D3.7	370	371	430	557	2.67E-05	2.36E-05	1.88E-05	3.02E-05
R02D3.8	107	83	120	166	2.80E-06	2.65E-06	1.82E-06	2.25E-06
R02D5.1	57	49	48	50	2.64E-05	2.50E-05	2.00E-05	3.20E-05
R02D5.3	18	54	26	24	1.38E-05	1.01E-05	1.01E-05	1.72E-05
R02D5.4	0	3	6	0	5.71E-06	4.66E-06	3.13E-06	4.03E-06
R02D5.6	5	12	17	7	2.80E-06	6.51E-06	2.15E-06	2.45E-06
R02D5.7	15	24	17	10	2.80E-06	2.65E-06	1.82E-06	2.25E-06
R02E12.2a	551	625	460	695	2.80E-06	2.65E-06	1.82E-06	2.25E-06
R02E12.2b.1	525	604	433	659	3.67E-06	5.55E-06	2.70E-06	2.25E-06
R02E12.2b.2	520	597	418	651	2.32E-05	2.49E-05	1.26E-05	2.35E-05
R02E12.4	11	13	9	6	3.02E-05	3.29E-05	1.62E-05	3.05E-05
R02E12.5	6	5	8	9	3.00E-05	3.25E-05	1.57E-05	3.02E-05
R02E12.6.1	39	94	38	20	2.80E-06	2.65E-06	1.82E-06	2.25E-06
R02E12.6.2	40	96	39	22	2.80E-06	2.65E-06	2.20E-06	3.06E-06
R02E12.8	3	7	6	2	5.71E-06	1.30E-05	3.61E-06	2.34E-06
R02E12.t1	0	1	0	0	3.89E-06	8.83E-06	2.48E-06	2.25E-06
R02E4.1	5	8	5	10	2.80E-06	2.65E-06	1.82E-06	2.25E-06
R02E4.2	6	22	6	10	2.80E-06	2.65E-06	1.82E-06	2.25E-06
R02E4.3	161	463	46	44	2.80E-06	2.65E-06	1.82E-06	2.25E-06
R02F11.1	156	180	37	59	2.80E-06	2.65E-06	1.82E-06	2.25E-06
R02F11.2	17	16	5	13	3.14E-05	8.53E-05	5.85E-06	6.90E-06
R02F11.3a	99	103	57	106	2.20E-05	2.39E-05	3.39E-06	6.66E-06
R02F11.3b	87	93	51	97	2.80E-06	2.65E-06	1.82E-06	2.25E-06
R02F11.4	235	245	469	618	6.58E-06	6.48E-06	2.48E-06	5.67E-06
R02F2.1a.1	1826	1998	2893	3188	6.58E-06	6.67E-06	2.51E-06	5.91E-06
R02F2.1a.2	960	1100	1615	1550	1.27E-05	1.25E-05	1.65E-05	2.69E-05
R02F2.1b	965	1088	1634	1556	1.21E-04	1.25E-04	1.24E-04	1.69E-04
R02F2.1c	690	734	1036	959	6.38E-05	6.91E-05	6.99E-05	8.28E-05
R02F2.1d.1	980	1129	1662	1564	1.06E-04	1.13E-04	1.17E-04	1.37E-04

R02F2.1d.2	952	1093	1598	1543	8.55E-05	8.59E-05	8.35E-05	9.54E-05
R02F2.1d.3	960	1100	1615	1550	1.20E-04	1.30E-04	1.32E-04	1.53E-04
R02F2.2	17	29	33	36	1.17E-04	1.27E-04	1.28E-04	1.52E-04
R02F2.4	284	277	470	572	1.06E-04	1.15E-04	1.16E-04	1.38E-04
R02F2.5	5	5	6	5	2.80E-06	2.65E-06	1.82E-06	2.25E-06
R02F2.6	3	2	1	0	2.33E-05	2.15E-05	2.51E-05	3.77E-05
R02F2.7.1	262	272	276	457	2.80E-06	2.65E-06	1.82E-06	2.25E-06
R02F2.8	34	76	55	55	2.80E-06	2.65E-06	1.82E-06	2.25E-06
R02F2.9	146	128	160	180	1.35E-05	1.32E-05	9.24E-06	1.89E-05
R03A10.1	15	13	18	15	2.80E-06	5.21E-06	2.59E-06	3.19E-06
R03A10.2	4	11	36	5	2.92E-05	2.42E-05	2.08E-05	2.89E-05
R03A10.3	21	44	23	33	4.40E-06	3.60E-06	3.44E-06	3.53E-06
R03A10.4b.1	145	168	158	184	2.80E-06	2.83E-06	6.38E-06	2.25E-06
R03A10.4b.2	127	153	143	170	2.80E-06	2.65E-06	1.82E-06	2.25E-06
R03A10.5	40	105	42	67	1.27E-05	1.39E-05	9.02E-06	1.30E-05
R03A10.6	4	8	13	4	1.06E-05	1.20E-05	7.74E-06	1.14E-05
R03C1.1	26	15	9	14	3.61E-06	8.94E-06	2.46E-06	4.84E-06
R03C1.3a	21	35	19	24	2.80E-06	2.65E-06	1.82E-06	2.25E-06
R03C1.3b	0	5	2	3	3.02E-06	2.65E-06	1.82E-06	2.25E-06
R03C1.4	2	3	3	1	2.80E-06	3.12E-06	1.82E-06	2.25E-06
R03D7.1	1475	4618	2191	3754	2.80E-06	2.65E-06	1.82E-06	2.25E-06
R03D7.2	130	102	54	119	2.80E-06	2.65E-06	1.82E-06	2.25E-06
R03D7.3	31	28	28	25	4.19E-05	1.24E-04	4.05E-05	8.56E-05
R03D7.4	386	520	453	684	7.84E-06	5.79E-06	2.11E-06	5.76E-06
R03D7.5	11	14	23	10	2.80E-06	2.65E-06	1.82E-06	2.25E-06
R03D7.6	159	164	284	123	2.49E-05	3.18E-05	1.91E-05	3.55E-05
R03D7.7	247	169	340	448	2.80E-06	2.65E-06	1.82E-06	2.25E-06
R03D7.8	12	33	11	6	2.68E-05	2.61E-05	3.12E-05	1.67E-05
R03E1.1.1	231	408	223	417	2.96E-05	1.91E-05	2.65E-05	4.31E-05
R03E1.1.2	203	352	197	380	2.80E-06	2.65E-06	1.82E-06	2.25E-06
R03E1.2.1	891	1372	853	1123	7.08E-06	1.18E-05	4.45E-06	1.03E-05
R03E1.2.2	827	1303	789	1082	7.20E-06	1.18E-05	4.56E-06	1.08E-05
R03E1.3	12	30	23	24	8.13E-05	1.18E-04	5.06E-05	8.23E-05
R03E1.4	2	1	4	0	9.49E-05	1.41E-04	5.89E-05	9.97E-05
R03E9.2	52	94	38	62	2.80E-06	2.65E-06	1.82E-06	2.25E-06
R03E9.3a	117	168	120	170	2.80E-06	2.65E-06	1.82E-06	2.25E-06
R03E9.3b	109	158	106	159	3.16E-06	5.42E-06	1.82E-06	3.04E-06
R03E9.4	7	14	6	10	4.06E-06	5.53E-06	2.71E-06	4.75E-06
R03G5.1a.1	12758	12784	12033	14414	4.00E-06	5.48E-06	2.53E-06	4.68E-06
R03G5.1a.2	12316	12387	11745	14088	2.80E-06	2.65E-06	1.82E-06	2.25E-06
R03G5.1a.3	11107	11132	10967	13095	7.47E-04	7.07E-04	4.58E-04	6.78E-04
R03G5.1c.1	12272	12337	11318	13402	7.97E-04	7.57E-04	4.95E-04	7.32E-04
R03G5.1c.2	12272	12337	11318	13402	7.64E-04	7.23E-04	4.91E-04	7.23E-04
R03G5.1c.3	9041	9017	8739	10100	7.35E-04	6.98E-04	4.41E-04	6.45E-04
R03G5.1d	10322	10338	10132	12246	1.15E-03	1.09E-03	6.89E-04	1.01E-03
R03G5.3	16	23	39	14	1.22E-03	1.15E-03	7.70E-04	1.10E-03
R03G5.5	35	57	30	21	8.81E-04	8.33E-04	5.62E-04	8.39E-04
R03G5.6	5	5	7	4	2.80E-06	2.65E-06	1.82E-06	2.25E-06
R03G5.7	30	22	14	25	6.75E-06	1.04E-05	3.75E-06	3.24E-06
R03G8.1	4	14	7	4	2.80E-06	2.65E-06	2.20E-06	2.25E-06
R03G8.3	48	68	41	56	5.10E-06	3.54E-06	1.82E-06	3.42E-06
R03G8.4	16	11	13	8	2.80E-06	2.65E-06	1.82E-06	2.25E-06
R03G8.5	10	9	8	1	2.80E-06	2.91E-06	1.82E-06	2.25E-06
R03G8.6	168	311	247	489	2.80E-06	2.65E-06	1.82E-06	2.25E-06
R03H10.1	12	29	21	17	2.80E-06	2.65E-06	1.82E-06	2.25E-06
R03H10.2	55	74	34	47	8.01E-06	1.40E-05	7.67E-06	1.87E-05
R03H10.4	0	1	1	1	2.80E-06	2.70E-06	1.82E-06	2.25E-06
R03H10.5	1	0	0	0	4.68E-06	5.92E-06	1.88E-06	3.22E-06
R03H10.6	9	12	24	10	2.80E-06	2.65E-06	1.82E-06	2.25E-06

R03H10.7	13	12	8	5	2.80E-06	2.65E-06	1.82E-06	2.25E-06
R03H4.1	12	19	18	12	2.80E-06	2.65E-06	1.82E-06	2.25E-06
R03H4.2	6	4	5	4	2.80E-06	2.65E-06	1.82E-06	2.25E-06
R03H4.3	7	6	7	2	2.80E-06	2.65E-06	1.82E-06	2.25E-06
R03H4.4	3	6	11	5	2.80E-06	2.65E-06	1.82E-06	2.25E-06
R03H4.5	14	24	46	22	2.80E-06	2.65E-06	1.82E-06	2.25E-06
R03H4.6	13	36	16	19	2.80E-06	2.65E-06	1.82E-06	2.25E-06
R03H4.7	0	3	4	0	2.80E-06	2.65E-06	1.82E-06	2.25E-06
R03H4.8	0	4	0	1	2.80E-06	2.65E-06	1.82E-06	2.25E-06
R03H4.9	7	6	6	3	2.80E-06	2.65E-06	1.82E-06	2.25E-06
R04A9.1	3	3	1	1	2.80E-06	2.65E-06	1.82E-06	2.25E-06
R04A9.2	236	223	355	451	2.80E-06	2.65E-06	1.82E-06	2.25E-06
R04A9.3	19	42	26	21	2.80E-06	2.65E-06	1.82E-06	2.25E-06
R04A9.4	142	195	211	145	7.67E-06	6.85E-06	7.53E-06	1.18E-05
R04A9.5	61	70	72	70	2.80E-06	4.89E-06	2.10E-06	2.25E-06
R04A9.6	8	8	11	5	1.96E-05	2.54E-05	1.89E-05	1.61E-05
R04A9.7	15	19	11	10	4.20E-06	4.55E-06	3.23E-06	3.87E-06
R04B3.1	5	17	7	3	2.80E-06	2.65E-06	1.82E-06	2.25E-06
R04B3.2	29	39	18	53	2.80E-06	2.65E-06	1.82E-06	2.25E-06
R04B3.3	14	27	11	10	2.80E-06	2.65E-06	1.82E-06	2.25E-06
R04B5.1	3	2	4	0	2.80E-06	3.49E-06	1.82E-06	4.03E-06
R04B5.11	4	3	6	2	2.80E-06	2.65E-06	1.82E-06	2.25E-06
R04B5.2	2	3	5	2	2.80E-06	2.65E-06	1.82E-06	2.25E-06
R04B5.3	22	24	13	19	2.80E-06	2.65E-06	1.82E-06	2.25E-06
R04B5.5	50	69	51	27	2.80E-06	2.65E-06	1.82E-06	2.25E-06
R04B5.6	11	7	24	9	2.80E-06	2.65E-06	1.82E-06	2.25E-06
R04B5.7	5	2	2	1	4.98E-06	6.51E-06	3.30E-06	2.25E-06
R04B5.8	2	4	9	3	2.80E-06	2.65E-06	1.82E-06	2.25E-06
R04B5.9	380	357	347	460	2.80E-06	2.65E-06	1.82E-06	2.25E-06
R04D3.1	3	11	8	4	2.80E-06	2.65E-06	1.82E-06	2.25E-06
R04D3.10	4	4	4	3	2.61E-05	2.32E-05	1.55E-05	2.54E-05
R04D3.11	16	12	38	13	2.80E-06	2.65E-06	1.82E-06	2.25E-06
R04D3.12	3	4	2	1	2.80E-06	2.65E-06	1.82E-06	2.25E-06
R04D3.2	117	170	135	187	2.80E-06	2.65E-06	1.82E-06	2.25E-06
R04D3.3.1	540	463	638	729	2.80E-06	2.65E-06	1.82E-06	2.25E-06
R04D3.3.2	536	458	627	718	9.69E-06	1.33E-05	7.29E-06	1.25E-05
R04D3.4	43	60	73	80	4.17E-05	3.38E-05	3.21E-05	4.52E-05
R04D3.6	9	9	2	7	4.15E-05	3.35E-05	3.16E-05	4.46E-05
R04D3.7	5	3	4	3	7.00E-06	9.23E-06	7.73E-06	1.05E-05
R04D3.8	8	9	4	4	2.80E-06	2.65E-06	1.82E-06	2.25E-06
R04D3.9	3	1	4	7	2.80E-06	2.65E-06	1.82E-06	2.25E-06
R04D3.t1	0	1	1	1	2.80E-06	2.65E-06	1.82E-06	2.25E-06
R04D3.t4	0	0	2	0	2.80E-06	2.65E-06	1.82E-06	2.25E-06
R04D3.t5	0	1	1	1	2.80E-06	2.65E-06	1.82E-06	2.25E-06
R04E5.10a	169	233	133	127	2.80E-06	2.65E-06	2.00E-06	2.25E-06
R04E5.10b	163	226	129	126	2.80E-06	2.65E-06	1.82E-06	2.25E-06
R04E5.2	91	107	62	112	9.88E-06	1.29E-05	5.07E-06	5.98E-06
R04E5.7	17	20	23	22	8.82E-06	1.15E-05	4.54E-06	5.47E-06
R04E5.8a	206	224	238	360	4.31E-06	4.81E-06	1.91E-06	4.27E-06
R04E5.8b	16	22	26	44	2.80E-06	2.65E-06	1.82E-06	2.25E-06
R04E5.9	29	36	26	47	6.19E-06	6.35E-06	4.65E-06	8.68E-06
R04E5.t6	0	1	0	1	3.22E-06	4.18E-06	3.39E-06	7.08E-06
R04F11.1	3	9	3	5	2.80E-06	2.94E-06	1.82E-06	3.26E-06
R04F11.2.1	915	1293	1506	801	2.80E-06	2.65E-06	1.82E-06	2.25E-06
R04F11.2.2	1031	1469	1973	852	2.80E-06	2.65E-06	1.82E-06	2.25E-06
R04F11.2.3	901	1290	1488	796	1.82E-04	2.43E-04	1.95E-04	1.28E-04
R04F11.3.1	582	634	662	1141	2.09E-04	2.82E-04	2.61E-04	1.39E-04
R04F11.4	42	66	99	56	1.88E-04	2.55E-04	2.02E-04	1.34E-04
R04F11.5	134	175	172	136	5.10E-05	5.25E-05	3.78E-05	8.04E-05

R05A10.1	9	10	12	12	2.80E-06	3.25E-06	3.35E-06	2.34E-06
R05A10.2	34	42	13	9	2.54E-05	3.14E-05	2.12E-05	2.07E-05
R05A10.3	17	19	18	5	2.80E-06	2.65E-06	1.82E-06	2.25E-06
R05A10.4	2	16	6	5	7.84E-06	9.15E-06	1.95E-06	2.25E-06
R05A10.5	2	3	1	2	2.80E-06	2.65E-06	1.82E-06	2.25E-06
R05A10.6	13	11	7	9	2.80E-06	5.00E-06	1.82E-06	2.25E-06
R05A10.7	7	5	6	1	2.80E-06	2.65E-06	1.82E-06	2.25E-06
R05A10.8	1	6	5	4	2.80E-06	2.65E-06	1.82E-06	2.25E-06
R05C11.1	5	7	2	6	2.80E-06	2.65E-06	1.82E-06	2.25E-06
R05C11.2	5	7	1	3	2.80E-06	2.65E-06	1.82E-06	2.25E-06
R05C11.3	460	557	334	528	2.80E-06	2.65E-06	1.82E-06	2.25E-06
R05C11.4	23	24	23	24	2.80E-06	2.65E-06	1.82E-06	2.25E-06
R05D11.1	13	23	39	43	1.29E-05	1.47E-05	6.09E-06	1.19E-05
R05D11.3.1	803	955	490	695	4.31E-06	4.26E-06	2.81E-06	3.62E-06
R05D11.3.2	714	827	434	633	2.80E-06	2.65E-06	1.82E-06	2.34E-06
R05D11.5	198	324	227	171	1.43E-04	1.60E-04	5.67E-05	9.92E-05
R05D11.6	228	290	211	288	1.30E-04	1.43E-04	5.15E-05	9.27E-05
R05D11.7	183	208	192	217	3.49E-05	5.40E-05	2.61E-05	2.42E-05
R05D11.8	1048	916	1280	1828	2.25E-05	2.70E-05	1.36E-05	2.28E-05
R05D11.9	502	485	714	904	3.53E-05	3.79E-05	2.41E-05	3.36E-05
R05D3.1	228	289	240	426	5.39E-05	4.45E-05	4.28E-05	7.55E-05
R05D3.11	663	761	946	1443	2.71E-05	2.47E-05	2.51E-05	3.92E-05
R05D3.12	36	29	36	57	7.95E-06	9.52E-06	5.45E-06	1.19E-05
R05D3.2	581	634	896	1195	1.78E-05	1.92E-05	1.65E-05	3.10E-05
R05D3.3	63	61	52	56	8.01E-06	6.08E-06	5.21E-06	1.02E-05
R05D3.4a	975	885	839	1524	2.48E-05	2.56E-05	2.49E-05	4.10E-05
R05D3.4b	892	808	800	1442	4.42E-06	4.05E-06	2.37E-06	3.15E-06
R05D3.5	1	8	2	1	3.75E-05	3.22E-05	2.10E-05	4.71E-05
R05D3.6	45	45	75	24	3.97E-05	3.40E-05	2.32E-05	5.16E-05
R05D3.7	2432	2417	2255	3039	2.80E-06	4.63E-06	1.82E-06	2.25E-06
R05D3.8	65	83	85	138	3.05E-05	2.89E-05	3.31E-05	1.31E-05
R05D3.9	229	398	162	330	8.83E-05	8.29E-05	5.33E-05	8.86E-05
R05D7.2	9	10	5	4	1.13E-05	1.36E-05	9.58E-06	1.92E-05
R05D7.3	15	20	27	17	1.19E-05	1.95E-05	5.47E-06	1.37E-05
R05D7.4	122	151	229	181	2.80E-06	2.65E-06	1.82E-06	2.25E-06
R05D7.5	36	48	58	40	2.80E-06	2.65E-06	1.84E-06	2.25E-06
R05D7.6	0	3	1	1	1.45E-05	1.70E-05	1.77E-05	1.73E-05
R05D8.1	6	4	2	5	4.06E-06	5.11E-06	4.25E-06	3.62E-06
R05D8.10	2	5	8	5	2.80E-06	2.65E-06	1.82E-06	2.25E-06
R05D8.11	4	8	3	9	2.80E-06	2.65E-06	1.82E-06	2.25E-06
R05D8.12	4	4	10	3	2.80E-06	2.65E-06	1.82E-06	2.25E-06
R05D8.2	4	3	3	2	2.80E-06	2.65E-06	1.82E-06	2.25E-06
R05D8.3	3	8	16	6	2.80E-06	2.65E-06	1.82E-06	2.25E-06
R05D8.4	2	5	10	4	2.80E-06	2.65E-06	1.82E-06	2.25E-06
R05D8.5	7	7	11	4	2.80E-06	2.65E-06	1.82E-06	2.25E-06
R05D8.6	5	5	6	2	2.80E-06	2.65E-06	1.82E-06	2.25E-06
R05D8.7	1	5	7	7	2.80E-06	2.65E-06	1.82E-06	2.25E-06
R05D8.8	45	61	51	73	2.80E-06	2.65E-06	1.82E-06	2.25E-06
R05D8.9	7	10	8	10	2.80E-06	2.65E-06	1.82E-06	2.25E-06
R05F9.10	1533	1236	1443	1856	5.77E-06	7.38E-06	4.26E-06	7.51E-06
R05F9.11	79	85	142	186	2.80E-06	2.65E-06	1.82E-06	2.25E-06
R05F9.13	1406	1959	2029	598	1.30E-04	9.94E-05	7.99E-05	1.27E-04
R05F9.1a	550	606	661	968	5.66E-06	5.74E-06	6.61E-06	1.07E-05
R05F9.1b	883	934	1046	1419	3.59E-04	4.72E-04	3.37E-04	1.23E-04
R05F9.1c.1	545	604	660	968	3.82E-05	3.98E-05	2.99E-05	5.40E-05
R05F9.1c.2	545	604	661	968	4.43E-05	4.43E-05	3.42E-05	5.72E-05
R05F9.1d	85	96	83	107	3.67E-05	3.84E-05	2.89E-05	5.23E-05
R05F9.3	680	1054	502	272	4.27E-05	4.47E-05	3.37E-05	6.09E-05
R05F9.5	5	14	10	4	2.03E-05	2.17E-05	1.29E-05	2.06E-05

R05F9.6	1097	2051	1492	1623	1.33E-04	1.95E-04	6.38E-05	4.27E-05
R05F9.7	9	5	28	6	2.80E-06	2.65E-06	1.82E-06	2.25E-06
R05F9.8	1178	1712	1833	514	6.67E-05	1.18E-04	5.90E-05	7.93E-05
R05F9.9	231	262	244	350	2.80E-06	2.65E-06	1.90E-06	2.25E-06
R05G6.1	7	17	9	10	2.99E-04	4.11E-04	3.03E-04	1.05E-04
R05G6.10.1	44	98	45	53	2.42E-05	2.59E-05	1.66E-05	2.95E-05
R05G6.10.2	39	90	40	49	2.80E-06	2.65E-06	1.82E-06	2.25E-06
R05G6.11	0	5	1	1	3.02E-06	6.35E-06	2.00E-06	2.92E-06
R05G6.12	4	2	0	1	3.08E-06	6.69E-06	2.06E-06	3.10E-06
R05G6.4	142	168	187	222	2.80E-06	3.47E-06	1.82E-06	2.25E-06
R05G6.5	4	11	5	7	2.80E-06	2.65E-06	1.82E-06	2.25E-06
R05G6.6	35	31	23	26	1.64E-05	1.84E-05	1.41E-05	2.06E-05
R05G6.7.1	4656	5412	6291	5898	2.80E-06	2.65E-06	1.82E-06	2.25E-06
R05G6.7.2	3754	4254	4883	5003	2.80E-06	2.65E-06	1.82E-06	2.25E-06
R05G6.8	363	471	417	624	5.10E-04	5.60E-04	4.49E-04	5.19E-04
R05G6.9	25	44	9	7	4.92E-04	5.26E-04	4.16E-04	5.26E-04
R05G9.1	1	1	1	2	1.65E-05	2.02E-05	1.23E-05	2.28E-05
R05G9.2a	27	48	25	36	2.80E-06	3.70E-06	1.82E-06	2.25E-06
R05G9.2b	416	472	521	532	2.80E-06	2.65E-06	1.82E-06	2.25E-06
R05G9.3	75	71	113	69	3.19E-06	5.37E-06	1.91E-06	3.42E-06
R05G9R.1	32	43	24	36	3.88E-05	4.16E-05	3.17E-05	3.99E-05
R05H10.1	6	40	13	18	1.31E-05	1.17E-05	1.29E-05	9.72E-06
R05H10.2	183	186	148	341	2.80E-06	2.65E-06	1.82E-06	2.25E-06
R05H10.3b	184	180	292	422	2.80E-06	5.13E-06	1.82E-06	2.25E-06
R05H10.5	68	122	345	172	9.86E-06	9.47E-06	5.19E-06	1.48E-05
R05H10.6	114	159	106	58	1.36E-05	1.25E-05	1.40E-05	2.50E-05
R05H10.7	1	1	3	2	1.23E-05	2.08E-05	4.06E-05	2.50E-05
R05H11.1	63	76	53	107	3.00E-06	3.97E-06	1.82E-06	2.25E-06
R05H11.2	12	14	5	11	2.80E-06	2.65E-06	1.82E-06	2.25E-06
R05H5.1	5	6	6	4	4.59E-06	5.24E-06	2.51E-06	6.27E-06
R05H5.2	17	28	16	9	2.80E-06	2.65E-06	1.82E-06	2.25E-06
R05H5.4	48	67	84	80	2.80E-06	2.65E-06	1.82E-06	2.25E-06
R05H5.5	170	171	188	234	2.80E-06	2.65E-06	1.82E-06	2.25E-06
R05H5.6	5	5	2	4	4.31E-06	5.69E-06	4.92E-06	5.78E-06
R05H5.7	4	5	3	2	2.17E-05	2.06E-05	1.56E-05	2.40E-05
R05H5.8	321	409	149	250	2.80E-06	2.65E-06	1.82E-06	2.25E-06
R06A10.1	6	8	1	5	2.80E-06	2.65E-06	1.82E-06	2.25E-06
R06A10.2.1	175	166	185	240	4.08E-05	4.91E-05	1.23E-05	2.55E-05
R06A10.2.2	108	107	73	104	2.80E-06	2.65E-06	1.82E-06	2.25E-06
R06A10.3	0	3	2	0	8.90E-06	7.99E-06	6.12E-06	9.81E-06
R06A10.4	46	58	32	39	8.46E-06	7.91E-06	3.72E-06	6.54E-06
R06A4.10a	5	7	11	11	2.80E-06	2.65E-06	1.82E-06	2.25E-06
R06A4.10b	8	12	18	18	3.25E-06	3.89E-06	1.82E-06	2.25E-06
R06A4.2.1	271	280	529	681	2.80E-06	2.65E-06	1.82E-06	2.25E-06
R06A4.2.2	225	245	463	630	2.80E-06	2.65E-06	1.82E-06	2.25E-06
R06A4.4a	945	794	1366	1928	1.08E-05	1.05E-05	1.36E-05	2.17E-05
R06A4.4b	334	271	484	707	1.00E-05	1.03E-05	1.34E-05	2.25E-05
R06A4.6	9	12	6	7	3.36E-05	2.67E-05	3.16E-05	5.50E-05
R06A4.7	302	276	415	559	2.96E-05	2.27E-05	2.79E-05	5.03E-05
R06A4.8	375	502	494	904	2.80E-06	2.65E-06	1.82E-06	2.25E-06
R06A4.9.1	349	310	331	633	1.37E-05	1.18E-05	1.22E-05	2.03E-05
R06A4.9.2	342	306	325	624	9.24E-06	1.17E-05	7.93E-06	1.79E-05
R06B10.1	46	70	22	25	1.49E-05	1.25E-05	9.22E-06	2.17E-05
R06B10.2	35	50	26	26	1.44E-05	1.22E-05	8.93E-06	2.12E-05
R06B10.3	405	755	693	1597	2.80E-06	2.65E-06	1.82E-06	2.25E-06
R06B10.4a	67	65	59	60	2.80E-06	2.65E-06	1.82E-06	2.25E-06
R06B10.4b	13	20	17	10	4.09E-05	7.20E-05	4.56E-05	1.30E-04
R06B10.5.1	62	53	57	73	2.80E-06	2.65E-06	1.82E-06	2.25E-06
R06B10.7	2	1	2	4	2.80E-06	2.65E-06	1.82E-06	2.25E-06

R06B9.1	28	14	31	24	3.86E-06	3.12E-06	2.31E-06	3.64E-06
R06B9.2	2	2	2	1	2.80E-06	2.65E-06	1.82E-06	3.42E-06
R06B9.3	316	163	445	301	2.80E-06	2.65E-06	1.82E-06	2.25E-06
R06B9.4	54	78	61	57	2.80E-06	2.65E-06	1.82E-06	2.25E-06
R06B9.5	84	66	127	101	2.33E-05	1.14E-05	2.14E-05	1.79E-05
R06B9.6	217	241	354	455	5.18E-06	7.09E-06	3.81E-06	4.41E-06
R06C1.1.1	250	300	295	369	3.39E-05	2.51E-05	3.33E-05	3.27E-05
R06C1.1.2	241	265	314	358	1.47E-05	1.54E-05	1.56E-05	2.48E-05
R06C1.2	279	276	412	548	1.62E-05	1.84E-05	1.25E-05	1.93E-05
R06C1.3	258	204	570	669	1.92E-05	1.99E-05	1.63E-05	2.29E-05
R06C1.4	1216	1279	1111	632	2.61E-05	2.44E-05	2.50E-05	4.11E-05
R06C1.5	1	1	6	1	2.05E-05	1.53E-05	2.95E-05	4.28E-05
R06C1.6	30	56	35	45	2.94E-04	2.92E-04	1.75E-04	1.23E-04
R06C7.1	3270	3542	4414	5772	2.80E-06	2.65E-06	1.82E-06	2.25E-06
R06C7.2	48	51	60	78	2.80E-06	3.76E-06	1.82E-06	2.56E-06
R06C7.3.1	99	192	88	126	1.20E-04	1.23E-04	1.06E-04	1.70E-04
R06C7.3.2	99	201	90	127	7.67E-06	7.70E-06	6.23E-06	9.99E-06
R06C7.5a	500	609	541	758	6.89E-06	1.26E-05	3.99E-06	7.04E-06
R06C7.5b.1	516	617	579	784	7.50E-06	1.44E-05	4.43E-06	7.71E-06
R06C7.5b.2	344	443	381	551	3.62E-05	4.17E-05	2.55E-05	4.41E-05
R06C7.6	20	17	16	22	3.75E-05	4.24E-05	2.74E-05	4.58E-05
R06C7.7.1	323	398	426	532	3.43E-05	4.17E-05	2.47E-05	4.41E-05
R06C7.8	968	971	1698	2149	4.03E-06	3.25E-06	2.10E-06	3.58E-06
R06C7.9	280	364	489	591	1.76E-05	2.04E-05	1.51E-05	2.32E-05
R06F6.1.1	1362	1169	2304	2734	3.54E-05	3.35E-05	4.04E-05	6.31E-05
R06F6.1.2	1320	1138	2253	2707	1.41E-05	1.73E-05	1.60E-05	2.39E-05
R06F6.12	198	202	312	418	9.55E-05	7.74E-05	1.05E-04	1.54E-04
R06F6.2	276	284	508	723	9.27E-05	7.55E-05	1.03E-04	1.53E-04
R06F6.4	220	252	360	369	1.66E-05	1.60E-05	1.70E-05	2.82E-05
R06F6.5a.1	368	350	453	640	9.30E-06	9.02E-06	1.11E-05	1.95E-05
R06F6.5a.2	328	328	435	634	1.58E-05	1.71E-05	1.69E-05	2.13E-05
R06F6.5b	398	379	530	738	3.61E-05	3.25E-05	2.89E-05	5.05E-05
R06F6.6	22	30	22	15	3.22E-05	3.04E-05	2.78E-05	5.00E-05
R06F6.7	14	17	41	11	3.22E-05	2.90E-05	2.79E-05	4.80E-05
R06F6.8a	616	582	939	1233	2.80E-06	2.65E-06	1.82E-06	2.25E-06
R06F6.8b	676	655	1015	1327	3.44E-06	3.97E-06	6.58E-06	2.25E-06
R06F6.9	428	595	584	507	1.55E-05	1.39E-05	1.54E-05	2.50E-05
R07A4.1	25	30	23	40	1.60E-05	1.47E-05	1.56E-05	2.52E-05
R07A4.2	18	23	17	10	3.84E-05	5.04E-05	3.41E-05	3.65E-05
R07A4.3	6	6	8	2	2.80E-06	2.65E-06	1.82E-06	2.25E-06
R07A4.4	11	11	7	7	2.97E-06	3.57E-06	1.82E-06	2.25E-06
R07B1.1	9	5	20	11	2.80E-06	2.65E-06	1.82E-06	2.25E-06
R07B1.10.1	807	684	1004	716	2.80E-06	2.65E-06	1.82E-06	2.25E-06
R07B1.10.2	642	553	854	678	2.80E-06	2.65E-06	1.82E-06	2.25E-06
R07B1.11	15	20	20	7	8.57E-05	6.86E-05	6.94E-05	6.11E-05
R07B1.12	10	29	13	12	1.13E-04	9.21E-05	9.80E-05	9.61E-05
R07B1.2.1	8	5	9	2	3.86E-06	4.87E-06	3.35E-06	2.25E-06
R07B1.2.2	3	5	9	1	2.80E-06	4.81E-06	1.82E-06	2.25E-06
R07B1.3	62	107	57	91	2.80E-06	2.65E-06	1.82E-06	2.25E-06
R07B1.4	172	298	173	229	2.80E-06	2.65E-06	1.82E-06	2.25E-06
R07B1.5	5	6	0	2	4.03E-06	6.56E-06	2.41E-06	4.75E-06
R07B1.6	1	5	0	2	2.67E-05	4.37E-05	1.75E-05	2.85E-05
R07B1.7	16	32	8	4	2.80E-06	2.65E-06	1.82E-06	2.25E-06
R07B1.8	13	14	5	8	2.80E-06	2.65E-06	1.82E-06	2.25E-06
R07B1.9	35	31	12	13	2.80E-06	4.73E-06	1.82E-06	2.25E-06
R07B5.1	11	17	27	18	2.80E-06	2.65E-06	1.82E-06	2.25E-06
R07B5.2	3	3	6	7	2.80E-06	2.65E-06	1.82E-06	2.25E-06
R07B5.3	8	13	18	8	2.80E-06	2.65E-06	1.82E-06	2.25E-06
R07B5.4	4	6	7	5	2.80E-06	2.65E-06	1.82E-06	2.25E-06

R07B5.5	1	3	2	2	2.80E-06	2.65E-06	1.82E-06	2.25E-06
R07B5.6	3	10	13	8	2.80E-06	2.65E-06	1.82E-06	2.25E-06
R07B5.7	6	1	3	4	2.80E-06	2.65E-06	1.82E-06	2.25E-06
R07B5.8b.1	309	313	419	594	2.80E-06	2.65E-06	1.82E-06	2.25E-06
R07B5.8b.2	335	334	439	624	2.80E-06	2.65E-06	1.82E-06	2.25E-06
R07B5.9	174	191	169	274	1.87E-05	1.79E-05	1.65E-05	2.88E-05
R07B7.1	135	158	368	523	1.89E-05	1.78E-05	1.61E-05	2.82E-05
R07B7.10	41	45	23	44	8.01E-06	8.31E-06	5.07E-06	1.01E-05
R07B7.11	105	185	65	79	5.68E-06	6.30E-06	1.01E-05	1.77E-05
R07B7.12	9	11	31	12	5.35E-06	5.55E-06	1.95E-06	4.61E-06
R07B7.13	16	15	13	4	8.15E-06	1.36E-05	3.28E-06	4.93E-06
R07B7.14	7	6	7	5	2.80E-06	2.65E-06	1.82E-06	2.25E-06
R07B7.15	6	11	14	7	2.80E-06	2.65E-06	1.82E-06	2.25E-06
R07B7.16	14	15	12	12	2.80E-06	2.65E-06	1.82E-06	2.25E-06
R07B7.2	344	370	582	784	2.80E-06	2.65E-06	1.82E-06	2.25E-06
R07B7.3	590	646	461	723	2.80E-06	2.65E-06	1.82E-06	2.25E-06
R07B7.4.1	16	30	11	10	1.81E-05	1.84E-05	2.00E-05	3.32E-05
R07B7.4.2	15	30	11	10	5.17E-05	5.35E-05	2.63E-05	5.09E-05
R07B7.5	105	147	54	88	2.80E-06	2.65E-06	1.82E-06	2.25E-06
R07B7.6	45	83	31	42	2.80E-06	2.65E-06	1.82E-06	2.25E-06
R07B7.7	1	3	1	3	8.40E-06	1.11E-05	2.82E-06	5.67E-06
R07B7.8	43	64	50	64	3.22E-06	5.58E-06	1.82E-06	2.41E-06
R07C12.1	9	5	10	8	2.80E-06	2.65E-06	1.82E-06	2.25E-06
R07C12.2	56	73	42	48	3.67E-06	5.16E-06	2.79E-06	4.39E-06
R07C12.3	4	8	4	1	2.80E-06	2.65E-06	1.82E-06	2.25E-06
R07C12.4	7	4	4	6	5.57E-06	6.85E-06	2.71E-06	3.82E-06
R07C3.1	3	8	4	6	2.80E-06	2.65E-06	1.82E-06	2.25E-06
R07C3.10	5	4	7	8	2.80E-06	2.65E-06	1.82E-06	2.25E-06
R07C3.11	5	6	6	6	2.80E-06	2.65E-06	1.82E-06	2.25E-06
R07C3.12	3	5	1	4	2.80E-06	2.65E-06	1.82E-06	2.25E-06
R07C3.13	18	20	9	8	2.80E-06	2.65E-06	1.82E-06	2.25E-06
R07C3.14	4	2	3	0	2.80E-06	2.65E-06	1.82E-06	2.25E-06
R07C3.2	24	40	28	25	2.80E-06	2.65E-06	1.82E-06	2.25E-06
R07C3.3	2	2	5	4	2.80E-06	2.65E-06	1.82E-06	2.25E-06
R07C3.4	25	34	17	14	2.80E-06	4.15E-06	2.00E-06	2.25E-06
R07C3.5	5	4	7	10	2.80E-06	2.65E-06	1.82E-06	2.25E-06
R07C3.6	20	14	9	16	2.80E-06	2.65E-06	1.82E-06	2.25E-06
R07C3.7	5	5	2	10	2.80E-06	2.65E-06	1.82E-06	2.25E-06
R07C3.8	13	31	31	25	2.80E-06	2.65E-06	1.82E-06	2.25E-06
R07C3.9	3	3	8	2	2.80E-06	2.65E-06	1.82E-06	2.25E-06
R07D5.1	36	47	58	45	2.80E-06	2.78E-06	1.91E-06	2.25E-06
R07D5.2	17	17	21	12	2.80E-06	2.65E-06	1.82E-06	2.25E-06
R07E3.1b.1	315	593	278	276	2.80E-06	3.17E-06	2.70E-06	2.59E-06
R07E3.1b.2	305	586	261	266	2.80E-06	2.65E-06	1.82E-06	2.25E-06
R07E3.2	21	18	4	10	2.93E-05	5.21E-05	1.68E-05	2.06E-05
R07E3.3	7	6	2	3	3.02E-05	5.47E-05	1.68E-05	2.11E-05
R07E3.4	61	164	56	85	3.22E-06	2.65E-06	1.82E-06	2.25E-06
R07E3.5a	117	199	84	133	2.80E-06	2.65E-06	1.82E-06	2.25E-06
R07E3.5b.1	113	189	82	130	7.59E-06	1.93E-05	4.54E-06	8.50E-06
R07E3.5b.2	101	170	78	126	7.87E-06	1.26E-05	3.66E-06	7.17E-06
R07E3.6	266	291	78	104	8.23E-06	1.30E-05	3.88E-06	7.60E-06
R07E3.7	12	14	10	3	7.59E-06	1.21E-05	3.81E-06	7.60E-06
R07E4.1a	56	90	54	83	9.58E-06	9.89E-06	1.82E-06	3.01E-06
R07E4.1b	48	76	41	64	2.80E-06	2.65E-06	1.82E-06	2.25E-06
R07E4.3	158	213	279	115	2.80E-06	2.65E-06	1.82E-06	2.25E-06
R07E4.5	156	158	226	324	2.80E-06	2.72E-06	1.82E-06	2.25E-06
R07E4.6a	932	960	729	1191	5.30E-05	6.75E-05	6.09E-05	3.10E-05
R07E4.6b	866	920	683	1121	6.75E-06	6.45E-06	6.36E-06	1.13E-05
R07E5.1	405	397	171	455	4.52E-05	4.40E-05	2.30E-05	4.64E-05

R07E5.11	68	85	124	74	9.65E-05	9.69E-05	4.95E-05	1.00E-04
R07E5.13	265	327	725	315	1.82E-05	1.69E-05	5.01E-06	1.65E-05
R07E5.14.1	452	512	572	494	2.23E-05	2.63E-05	2.64E-05	1.95E-05
R07E5.14.2	402	457	523	433	6.15E-05	7.16E-05	1.09E-04	5.87E-05
R07E5.15	57	128	49	27	7.56E-05	8.09E-05	6.22E-05	6.63E-05
R07E5.16	0	2	4	1	7.97E-05	8.56E-05	6.75E-05	6.89E-05
R07E5.17	14	5	16	9	7.62E-06	1.62E-05	4.26E-06	2.90E-06
R07E5.2.1	700	1079	945	600	2.80E-06	2.65E-06	2.86E-06	2.25E-06
R07E5.3	314	469	424	577	2.80E-06	2.65E-06	1.82E-06	2.25E-06
R07E5.4	159	240	98	149	1.02E-04	1.49E-04	9.00E-05	7.06E-05
R07E5.5	1	3	4	1	1.78E-05	2.51E-05	1.56E-05	2.62E-05
R07E5.6	29	38	15	30	2.76E-05	3.94E-05	1.11E-05	2.08E-05
R07E5.7.1	371	445	473	415	2.80E-06	2.65E-06	1.82E-06	2.25E-06
R07E5.7.2	305	377	345	338	2.80E-06	2.94E-06	1.82E-06	2.25E-06
R07E5.8.1	271	337	318	503	5.65E-05	6.41E-05	4.69E-05	5.08E-05
R07E5.8.2	262	319	291	486	5.40E-05	6.31E-05	3.98E-05	4.81E-05
R07G3.2	35	84	22	28	1.34E-05	1.57E-05	1.02E-05	2.00E-05
R07G3.3a	2119	1869	2127	3556	1.34E-05	1.54E-05	9.69E-06	2.00E-05
R07G3.3b	2094	1837	2107	3528	4.65E-06	1.06E-05	1.90E-06	2.99E-06
R07G3.3c	1307	1119	1288	2119	3.88E-05	3.23E-05	2.53E-05	5.22E-05
R07G3.5	741	804	827	882	3.93E-05	3.26E-05	2.57E-05	5.32E-05
R07G3.6	59	134	29	21	3.57E-05	2.89E-05	2.29E-05	4.65E-05
R07G3.7a	361	399	444	612	8.59E-05	8.81E-05	6.24E-05	8.21E-05
R07G3.7b.1	497	546	620	810	6.19E-06	1.33E-05	1.99E-06	2.25E-06
R07G3.7b.2	307	342	386	557	3.52E-05	3.67E-05	2.81E-05	4.79E-05
R07G3.8	65	141	42	74	3.22E-05	3.35E-05	2.62E-05	4.22E-05
R07G3.9	67	79	64	68	3.52E-05	3.71E-05	2.88E-05	5.13E-05
R07H5.1	339	349	468	509	5.96E-06	1.22E-05	2.50E-06	5.44E-06
R07H5.10	5	7	3	8	1.34E-05	1.49E-05	8.35E-06	1.10E-05
R07H5.11	69	65	73	72	4.42E-05	4.30E-05	3.97E-05	5.33E-05
R07H5.2a.1	671	987	700	1154	2.80E-06	2.65E-06	1.82E-06	2.25E-06
R07H5.2b	617	915	667	1113	1.37E-05	1.22E-05	9.48E-06	1.15E-05
R07H5.3a	87	109	88	131	3.53E-05	4.91E-05	2.40E-05	4.88E-05
R07H5.3b	111	123	117	155	3.56E-05	4.99E-05	2.51E-05	5.16E-05
R07H5.4	248	277	218	228	1.19E-05	1.41E-05	7.84E-06	1.44E-05
R07H5.7	3	0	3	1	1.32E-05	1.39E-05	9.07E-06	1.48E-05
R07H5.8.1	2625	3462	2678	3736	3.93E-05	4.14E-05	2.25E-05	2.90E-05
R07H5.8.2	2471	3229	2549	3658	2.80E-06	2.65E-06	1.82E-06	2.25E-06
R07H5.9	17	20	8	6	2.29E-04	2.86E-04	1.52E-04	2.62E-04
R08A2.1	18	29	14	12	2.62E-04	3.24E-04	1.76E-04	3.12E-04
R08A2.2	23	55	15	24	2.80E-06	2.65E-06	1.82E-06	2.25E-06
R08A2.3	14	19	24	4	2.80E-06	3.62E-06	1.82E-06	2.25E-06
R08A2.4	6	6	8	2	2.80E-06	5.16E-06	1.82E-06	2.25E-06
R08A2.5	8	4	8	5	4.23E-06	5.42E-06	4.72E-06	2.25E-06
R08A2.6	3	5	0	3	2.80E-06	2.65E-06	1.82E-06	2.25E-06
R08A2.7	4	5	5	0	2.80E-06	2.65E-06	1.82E-06	2.25E-06
R08B4.1a	92	140	54	32	2.80E-06	2.65E-06	1.82E-06	2.25E-06
R08B4.1b	101	154	56	35	2.80E-06	2.65E-06	1.82E-06	2.25E-06
R08B4.2	34	38	36	39	2.97E-06	4.26E-06	1.82E-06	2.25E-06
R08B4.3	39	77	47	35	3.14E-06	4.52E-06	1.82E-06	2.25E-06
R08B4.4	4	3	2	2	3.33E-06	3.52E-06	2.30E-06	3.06E-06
R08B4.5	2	9	3	5	5.91E-06	1.10E-05	4.65E-06	4.27E-06
R08C7.1	1	0	1	0	2.80E-06	2.65E-06	1.82E-06	2.25E-06
R08C7.10a	613	609	811	1206	2.80E-06	3.23E-06	1.82E-06	2.25E-06
R08C7.10b	619	595	822	1195	2.80E-06	2.65E-06	1.82E-06	2.25E-06
R08C7.10c	536	499	717	1069	2.79E-05	2.62E-05	2.40E-05	4.41E-05
R08C7.11	0	2	0	0	2.98E-05	2.70E-05	2.57E-05	4.62E-05
R08C7.13	7	9	6	6	2.70E-05	2.38E-05	2.35E-05	4.33E-05
R08C7.2a	335	415	459	584	2.80E-06	2.65E-06	1.82E-06	2.25E-06

R08C7.2b	325	415	449	559	2.80E-06	2.65E-06	1.82E-06	2.25E-06
R08C7.3.1	1892	2002	3834	3826	2.95E-05	3.45E-05	2.63E-05	4.13E-05
R08C7.3.2	1819	1935	3852	3815	2.55E-05	3.08E-05	2.30E-05	3.53E-05
R08C7.3.3	964	998	2280	2124	2.88E-04	2.88E-04	3.80E-04	4.68E-04
R08C7.4	5	4	3	4	2.67E-04	2.69E-04	3.68E-04	4.50E-04
R08C7.5	18	39	19	19	2.44E-04	2.38E-04	3.75E-04	4.31E-04
R08C7.6	14	23	11	11	2.80E-06	2.65E-06	1.82E-06	2.25E-06
R08C7.7	4	2	3	1	2.80E-06	2.67E-06	1.82E-06	2.25E-06
R08C7.8	18	38	11	18	2.80E-06	2.65E-06	1.82E-06	2.25E-06
R08C7.9	3	3	1	6	2.80E-06	2.65E-06	1.82E-06	2.25E-06
R08D7.1	375	334	318	502	2.80E-06	3.84E-06	1.82E-06	2.25E-06
R08D7.3	1388	1488	1797	2356	2.80E-06	2.65E-06	1.82E-06	2.25E-06
R08D7.4.1	256	297	262	386	2.85E-05	2.40E-05	1.57E-05	3.06E-05
R08D7.4.2	243	285	246	374	8.65E-05	8.76E-05	7.28E-05	1.18E-04
R08D7.5	78	103	97	92	5.01E-05	5.49E-05	3.34E-05	6.07E-05
R08D7.6a	1116	1478	1448	2238	4.66E-05	5.16E-05	3.07E-05	5.76E-05
R08D7.6b	1123	1486	1454	2265	1.95E-05	2.44E-05	1.58E-05	1.85E-05
R08D7.6c	903	1206	1148	1753	3.77E-05	4.71E-05	3.18E-05	6.06E-05
R08D7.7	54	78	59	77	4.42E-05	5.52E-05	3.72E-05	7.15E-05
R08E3.1a	1102	1266	2013	1793	3.72E-05	4.69E-05	3.08E-05	5.80E-05
R08E3.1b	1100	1263	2004	1787	3.75E-06	5.11E-06	2.66E-06	4.30E-06
R08E3.2	124	133	192	222	2.66E-05	2.89E-05	3.17E-05	3.48E-05
R08E3.3a	62	92	61	91	2.63E-05	2.85E-05	3.12E-05	3.43E-05
R08E3.3b	89	140	79	130	1.47E-05	1.49E-05	1.48E-05	2.11E-05
R08E3.4a	124	177	119	168	5.01E-06	7.01E-06	3.21E-06	5.89E-06
R08E3.4b	58	99	61	81	5.38E-06	8.01E-06	3.12E-06	6.32E-06
R08E5.2a	96	170	105	156	1.03E-05	1.39E-05	6.45E-06	1.12E-05
R08E5.2b.1	92	150	92	143	4.26E-06	6.85E-06	2.92E-06	4.77E-06
R08E5.2b.2	88	147	86	131	1.01E-05	1.69E-05	7.20E-06	1.32E-05
R08E5.2b.3	88	145	84	129	1.04E-05	1.61E-05	6.78E-06	1.30E-05
R08E5.2b.4	107	181	121	163	9.30E-06	1.47E-05	5.92E-06	1.11E-05
R08E5.2c	91	164	102	145	9.91E-06	1.54E-05	6.16E-06	1.17E-05
R08E5.4	1	1	2	0	1.18E-05	1.89E-05	8.67E-06	1.44E-05
R08F11.1	66	68	85	179	9.63E-06	1.64E-05	7.03E-06	1.23E-05
R08F11.2	5	5	7	4	2.80E-06	2.65E-06	1.82E-06	2.25E-06
R08F11.3	20	41	19	71	2.80E-06	2.70E-06	2.33E-06	6.07E-06
R08F11.4	12	10	25	13	2.80E-06	2.65E-06	1.82E-06	2.25E-06
R08F11.5	7	5	6	2	2.80E-06	2.78E-06	1.82E-06	4.07E-06
R08F11.6	3	8	6	4	2.80E-06	2.65E-06	1.82E-06	2.25E-06
R08F11.7	10	9	15	4	2.80E-06	2.65E-06	1.82E-06	2.25E-06
R08H2.10	1	2	2	2	2.80E-06	2.65E-06	1.82E-06	2.25E-06
R08H2.11	3	4	6	3	2.80E-06	2.65E-06	1.82E-06	2.25E-06
R08H2.12	5	8	11	3	2.80E-06	2.65E-06	1.82E-06	2.25E-06
R08H2.13	4	7	2	0	2.80E-06	2.65E-06	1.82E-06	2.25E-06
R08H2.14	2	11	10	6	2.80E-06	2.65E-06	1.82E-06	2.25E-06
R08H2.2	4	4	5	1	2.80E-06	2.65E-06	1.82E-06	2.25E-06
R08H2.3	2	2	1	1	2.80E-06	2.65E-06	1.82E-06	2.25E-06
R08H2.4	3	5	5	3	2.80E-06	2.65E-06	1.82E-06	2.25E-06
R08H2.5	21	16	25	36	2.80E-06	2.65E-06	1.82E-06	2.25E-06
R08H2.7	2	5	8	2	2.80E-06	2.65E-06	1.82E-06	2.25E-06
R08H2.8	3	5	2	3	2.80E-06	2.65E-06	1.82E-06	3.06E-06
R08H2.9	4	9	16	7	2.80E-06	2.65E-06	1.82E-06	2.25E-06
R09A1.1	1838	1438	1538	2778	2.80E-06	2.65E-06	1.82E-06	2.25E-06
R09A1.2	27	39	44	30	2.80E-06	2.65E-06	1.82E-06	2.25E-06
R09A1.3	18	38	9	12	5.64E-05	4.17E-05	3.07E-05	6.84E-05
R09A1.5	21	26	29	20	2.80E-06	3.31E-06	2.57E-06	2.25E-06
R09A8.1	61	63	128	130	2.80E-06	4.21E-06	1.82E-06	2.25E-06
R09A8.2	128	127	174	261	4.20E-06	4.92E-06	3.77E-06	3.22E-06
R09A8.3.1	510	802	554	954	2.80E-06	2.65E-06	2.84E-06	3.55E-06

R09A8.3.2	477	734	527	873	5.35E-06	5.03E-06	4.74E-06	8.77E-06
R09A8.3.3	383	585	381	636	1.78E-05	2.65E-05	1.26E-05	2.68E-05
R09A8.4	24	28	10	8	1.74E-05	2.53E-05	1.25E-05	2.56E-05
R09A8.5	12	42	23	11	1.70E-05	2.45E-05	1.10E-05	2.27E-05
R09B3.1a	245	236	438	364	5.85E-06	6.45E-06	1.82E-06	2.25E-06
R09B3.1b	222	208	405	325	2.80E-06	5.21E-06	1.97E-06	2.25E-06
R09B3.2	2020	984	4650	1416	2.87E-05	2.61E-05	3.34E-05	3.43E-05
R09B3.4	740	544	844	857	2.79E-05	2.47E-05	3.31E-05	3.28E-05
R09B3.5.1	429	568	484	403	6.11E-04	2.81E-04	9.16E-04	3.44E-04
R09B3.5.2	387	532	389	368	1.22E-04	8.50E-05	9.09E-05	1.14E-04
R09B3.6	19	33	21	38	8.23E-05	1.03E-04	6.04E-05	6.21E-05
R09B5.1	8	11	9	5	7.20E-05	9.35E-05	4.71E-05	5.50E-05
R09B5.10	1	2	0	0	2.80E-06	2.78E-06	1.82E-06	2.72E-06
R09B5.11	28	97	84	83	2.80E-06	2.65E-06	1.82E-06	2.25E-06
R09B5.12	9	5	7	6	2.80E-06	2.65E-06	1.82E-06	2.25E-06
R09B5.2	0	4	0	0	2.80E-06	6.22E-06	3.70E-06	4.52E-06
R09B5.4	42	26	33	12	2.80E-06	2.65E-06	1.82E-06	2.25E-06
R09B5.5	79	98	15	21	2.80E-06	2.65E-06	1.82E-06	2.25E-06
R09B5.6.1	193	396	354	418	2.83E-06	2.65E-06	1.82E-06	2.25E-06
R09B5.6.2	168	364	325	400	7.59E-06	8.89E-06	1.82E-06	2.25E-06
R09B5.7a	1	4	4	1	2.11E-05	4.09E-05	2.52E-05	3.67E-05
R09B5.7b	4	7	8	4	2.05E-05	4.19E-05	2.58E-05	3.92E-05
R09B5.8	18	44	56	24	2.80E-06	2.65E-06	1.82E-06	2.25E-06
R09D1.1	2	10	16	2	2.80E-06	2.65E-06	1.82E-06	2.25E-06
R09D1.10	32	47	31	27	4.87E-06	1.12E-05	9.86E-06	5.22E-06
R09D1.11	20	14	26	9	2.80E-06	2.65E-06	1.82E-06	2.25E-06
R09D1.12	6	10	17	6	2.80E-06	3.52E-06	1.82E-06	2.25E-06
R09D1.13	6	2	2	3	2.80E-06	2.65E-06	1.82E-06	2.25E-06
R09D1.14	2	1	4	3	2.80E-06	2.65E-06	1.82E-06	2.25E-06
R09D1.2	6	9	10	6	2.80E-06	2.65E-06	1.82E-06	2.25E-06
R09D1.3	5	7	6	3	2.80E-06	2.65E-06	1.82E-06	2.25E-06
R09D1.4	4	5	6	2	2.80E-06	2.65E-06	1.82E-06	2.25E-06
R09D1.5	9	9	8	7	2.80E-06	2.65E-06	1.82E-06	2.25E-06
R09D1.6	14	17	9	10	2.80E-06	2.65E-06	1.82E-06	2.25E-06
R09D1.7	2	3	4	3	2.80E-06	2.65E-06	1.82E-06	2.25E-06
R09D1.8	10	14	10	9	2.80E-06	2.65E-06	1.82E-06	2.25E-06
R09D1.9	8	4	10	3	2.80E-06	2.65E-06	1.82E-06	2.25E-06
R09E10.10	0	1	1	0	2.80E-06	2.65E-06	1.82E-06	2.25E-06
R09E10.2	9	35	12	4	2.80E-06	2.65E-06	1.82E-06	2.25E-06
R09E10.3	47	104	33	42	2.80E-06	2.65E-06	1.82E-06	2.25E-06
R09E10.5	37	31	51	97	2.80E-06	5.50E-06	1.82E-06	2.25E-06
R09E10.6	225	537	123	103	2.80E-06	5.18E-06	1.82E-06	2.25E-06
R09E10.7	332	383	348	506	2.80E-06	2.65E-06	1.82E-06	2.25E-06
R09E10.8	2	3	12	6	2.67E-05	6.03E-05	9.51E-06	9.83E-06
R09E10.9	9	14	4	6	6.72E-06	7.33E-06	4.59E-06	8.25E-06
R09E12.1	1	6	9	2	2.80E-06	2.65E-06	2.71E-06	2.25E-06
R09E12.2	4	3	1	6	2.80E-06	2.65E-06	1.82E-06	2.25E-06
R09E12.3	1105	725	899	987	2.80E-06	2.65E-06	1.82E-06	2.25E-06
R09E12.4	8	4	5	3	2.80E-06	2.65E-06	1.82E-06	2.25E-06
R09E12.5	14	38	22	45	1.10E-04	6.85E-05	5.85E-05	7.93E-05
R09E12.6	47	129	47	236	2.80E-06	2.65E-06	1.82E-06	2.25E-06
R09E12.8	7	4	16	10	2.80E-06	3.25E-06	1.82E-06	3.28E-06
R09E12.9	12	21	40	8	2.80E-06	5.63E-06	1.82E-06	8.77E-06
R09F10.1	125	180	108	129	2.80E-06	2.65E-06	1.82E-06	2.25E-06
R09F10.10	5	3	3	2	3.47E-06	5.74E-06	7.53E-06	2.25E-06
R09F10.11	2	3	13	2	1.13E-05	1.53E-05	6.34E-06	9.33E-06
R09F10.2	33	35	10	9	2.80E-06	2.65E-06	1.82E-06	2.25E-06
R09F10.4	206	363	277	279	2.80E-06	2.65E-06	1.82E-06	2.25E-06
R09F10.5	15	23	14	13	3.25E-06	3.25E-06	1.82E-06	2.25E-06

R09F10.6	4	3	7	4	1.40E-05	2.33E-05	1.23E-05	1.52E-05
R09F10.7	32	34	10	9	3.84E-06	5.55E-06	2.33E-06	2.68E-06
R09F10.8.1	296	271	318	427	2.80E-06	2.65E-06	1.82E-06	2.25E-06
R09F10.8.2	232	218	262	358	3.16E-06	3.17E-06	1.82E-06	2.25E-06
R09F10.9	1	4	1	0	2.72E-05	2.35E-05	1.90E-05	3.15E-05
R09G11.1	12	22	8	11	2.55E-05	2.27E-05	1.88E-05	3.17E-05
R09G11.2a.1	165	192	183	287	2.80E-06	2.65E-06	1.82E-06	2.25E-06
R09G11.2a.2	169	199	185	288	2.80E-06	2.65E-06	1.82E-06	2.25E-06
R09G11.2d	73	74	75	83	7.84E-06	8.62E-06	5.67E-06	1.10E-05
R09H10.1	4	9	5	11	7.76E-06	8.62E-06	5.52E-06	1.06E-05
R09H10.2	11	9	8	7	5.46E-06	5.24E-06	3.66E-06	4.99E-06
R09H10.4	157	362	174	275	2.80E-06	2.65E-06	1.82E-06	2.25E-06
R09H10.6	5	7	2	0	2.80E-06	2.65E-06	1.82E-06	2.25E-06
R09H10.7	5	15	5	12	6.13E-06	1.33E-05	4.41E-06	8.61E-06
R09H3.1	14	17	16	9	2.80E-06	2.65E-06	1.82E-06	2.25E-06
R09H3.3	2	2	4	0	2.80E-06	5.58E-06	1.82E-06	3.78E-06
R102.1	1	5	3	2	2.80E-06	2.65E-06	1.82E-06	2.25E-06
R102.10	28	32	26	9	2.80E-06	2.65E-06	1.82E-06	2.25E-06
R102.2	98	111	361	50	2.80E-06	2.65E-06	1.82E-06	2.25E-06
R102.3	12	19	11	8	2.94E-06	3.17E-06	1.82E-06	2.25E-06
R102.4b	221	286	174	252	2.22E-05	2.38E-05	5.33E-05	9.11E-06
R102.5a.1	590	644	921	956	2.80E-06	2.65E-06	1.82E-06	2.25E-06
R102.5a.2	483	552	731	803	1.79E-05	2.19E-05	9.18E-06	1.64E-05
R102.5b	539	587	787	872	5.03E-05	5.19E-05	5.11E-05	6.55E-05
R102.6	20	23	18	9	4.62E-05	4.99E-05	4.55E-05	6.17E-05
R102.7	20	21	9	16	4.77E-05	4.90E-05	4.53E-05	6.19E-05
R102.8	7	20	5	4	2.80E-06	2.65E-06	1.82E-06	2.25E-06
R102.9	3	5	3	0	2.80E-06	2.65E-06	1.82E-06	2.25E-06
R105.1	48	81	28	23	2.80E-06	3.36E-06	1.82E-06	2.25E-06
R106.1	3	4	2	2	2.80E-06	2.65E-06	1.82E-06	2.25E-06
R106.2	11	8	8	16	3.53E-06	5.61E-06	1.82E-06	2.25E-06
R107.1.1	60	61	38	68	2.80E-06	2.65E-06	1.82E-06	2.25E-06
R107.1.2	48	57	35	63	2.80E-06	2.65E-06	1.82E-06	2.25E-06
R107.2	76	100	68	100	3.58E-06	3.47E-06	1.82E-06	3.28E-06
R107.4a	813	857	1273	1694	3.11E-06	3.49E-06	1.82E-06	3.28E-06
R107.4b	753	786	1175	1582	9.16E-06	1.14E-05	5.32E-06	9.67E-06
R107.4c	819	866	1303	1718	3.38E-05	3.36E-05	3.44E-05	5.65E-05
R107.4d	817	864	1290	1704	3.42E-05	3.38E-05	3.47E-05	5.78E-05
R107.5a	457	711	497	488	3.34E-05	3.34E-05	3.46E-05	5.63E-05
R107.6.1	640	623	723	967	3.11E-05	3.11E-05	3.19E-05	5.20E-05
R107.6.2	643	624	731	970	5.30E-05	7.79E-05	3.75E-05	4.55E-05
R107.7.1	1287	1652	1562	1236	2.18E-05	2.01E-05	1.61E-05	2.65E-05
R107.7.2	1145	1476	1315	1144	2.18E-05	2.00E-05	1.61E-05	2.64E-05
R107.8	73	83	53	97	1.91E-04	2.31E-04	1.51E-04	1.47E-04
R10A10.1	54	74	90	67	1.96E-04	2.39E-04	1.47E-04	1.58E-04
R10A10.2	119	143	131	148	2.80E-06	2.65E-06	1.82E-06	2.25E-06
R10D12.1	32	69	31	19	3.73E-05	4.83E-05	4.05E-05	3.72E-05
R10D12.10	33	48	22	23	2.85E-05	3.24E-05	2.04E-05	2.85E-05
R10D12.11	9	6	9	7	2.80E-06	4.39E-06	1.82E-06	2.25E-06
R10D12.12	156	216	277	167	2.88E-06	3.97E-06	1.82E-06	2.25E-06
R10D12.13a	856	931	755	1221	2.80E-06	2.65E-06	1.82E-06	2.25E-06
R10D12.13b	76	98	61	100	3.06E-05	4.00E-05	3.53E-05	2.63E-05
R10D12.13c	73	103	61	88	4.92E-05	5.06E-05	2.82E-05	5.64E-05
R10D12.14a	345	392	377	746	9.46E-06	1.15E-05	4.94E-06	1.00E-05
R10D12.14b	472	483	485	941	9.83E-06	1.31E-05	5.34E-06	9.51E-06
R10D12.15	5	13	7	3	5.72E-05	6.14E-05	4.07E-05	9.94E-05
R10D12.17	17	21	28	16	6.35E-05	6.13E-05	4.24E-05	1.02E-04
R10D12.2	4	3	12	6	2.80E-06	2.65E-06	1.82E-06	2.25E-06
R10D12.3	6	5	11	9	2.80E-06	2.65E-06	1.82E-06	2.25E-06

R10D12.4	6	4	5	6	2.80E-06	2.65E-06	1.82E-06	2.25E-06
R10D12.5	4	2	10	3	2.80E-06	2.65E-06	1.82E-06	2.25E-06
R10D12.6	1	4	0	0	2.80E-06	2.65E-06	1.82E-06	2.25E-06
R10D12.7	3	4	3	1	2.80E-06	2.65E-06	1.82E-06	2.25E-06
R10D12.8	26	44	44	69	2.80E-06	2.65E-06	1.82E-06	2.25E-06
R10E11.1a	1337	1142	1340	2357	2.80E-06	2.65E-06	1.82E-06	2.25E-06
R10E11.1b	1279	1087	1204	2293	2.80E-06	2.65E-06	1.82E-06	3.40E-06
R10E11.2.1	4489	5464	3987	5817	2.28E-05	1.84E-05	1.49E-05	3.23E-05
R10E11.2.2	4065	5121	3478	5402	2.25E-05	1.81E-05	1.38E-05	3.25E-05
R10E11.3a.1	265	277	293	420	5.18E-04	5.96E-04	3.00E-04	5.39E-04
R10E11.3a.2	232	229	256	373	4.73E-04	5.63E-04	2.63E-04	5.05E-04
R10E11.3b	210	212	236	354	1.86E-05	1.83E-05	1.34E-05	2.36E-05
R10E11.4	251	237	325	391	2.08E-05	1.94E-05	1.50E-05	2.69E-05
R10E11.5	31	37	30	28	1.97E-05	1.87E-05	1.44E-05	2.66E-05
R10E11.6a	87	123	94	122	2.11E-05	1.88E-05	1.77E-05	2.64E-05
R10E11.6b	86	119	103	122	2.80E-06	2.70E-06	1.82E-06	2.25E-06
R10E11.7	5	7	5	3	7.22E-06	9.63E-06	5.08E-06	8.12E-06
R10E11.8.1	1259	1230	1210	1219	6.08E-06	7.94E-06	4.72E-06	6.90E-06
R10E11.8.2	1264	1240	1224	1226	2.80E-06	2.65E-06	1.82E-06	2.25E-06
R10E11.8.3	585	712	540	634	1.75E-04	1.61E-04	1.09E-04	1.36E-04
R10E11.9	6	11	6	3	2.30E-04	2.13E-04	1.45E-04	1.79E-04
R10E12.1a	1092	1176	1342	1981	1.04E-04	1.20E-04	6.28E-05	9.10E-05
R10E12.1b	1098	1189	1326	1996	2.80E-06	2.65E-06	1.82E-06	2.25E-06
R10E12.1c	1050	1144	1247	1923	4.44E-05	4.51E-05	3.55E-05	6.46E-05
R10E12.1d	527	562	639	905	4.43E-05	4.53E-05	3.48E-05	6.46E-05
R10E12.2	59	95	73	118	4.36E-05	4.49E-05	3.37E-05	6.41E-05
R10E4.1.1	152	250	188	237	3.94E-05	3.97E-05	3.11E-05	5.44E-05
R10E4.1.2	118	206	149	198	4.65E-06	7.06E-06	3.74E-06	7.44E-06
R10E4.11	53	54	142	154	1.27E-05	1.98E-05	1.02E-05	1.59E-05
R10E4.2a.1	545	539	316	739	1.15E-05	1.90E-05	9.46E-06	1.55E-05
R10E4.2a.2	270	286	164	296	6.52E-06	6.30E-06	1.14E-05	1.52E-05
R10E4.2a.3	244	250	137	277	2.66E-05	2.48E-05	1.00E-05	2.89E-05
R10E4.2a.4	252	261	150	283	2.53E-05	2.53E-05	9.99E-06	2.22E-05
R10E4.2a.5	250	264	149	282	2.34E-05	2.26E-05	8.53E-06	2.13E-05
R10E4.2b.1	252	270	154	278	2.37E-05	2.31E-05	9.17E-06	2.13E-05
R10E4.2b.2	244	258	145	267	2.38E-05	2.38E-05	9.24E-06	2.16E-05
R10E4.2c	121	154	91	168	2.12E-05	2.15E-05	8.44E-06	1.88E-05
R10E4.3	19	17	10	13	2.09E-05	2.09E-05	8.09E-06	1.84E-05
R10E4.4.1	1091	1262	1780	2127	2.19E-05	2.63E-05	1.07E-05	2.44E-05
R10E4.4.2	1051	1192	1691	2079	2.80E-06	2.65E-06	1.82E-06	2.25E-06
R10E4.5	46	42	46	60	5.04E-05	5.51E-05	5.35E-05	7.89E-05
R10E4.6	13	13	18	12	5.12E-05	5.49E-05	5.36E-05	8.14E-05
R10E4.7	39	71	53	36	6.61E-06	5.69E-06	4.30E-06	6.93E-06
R10E4.9	81	71	125	67	2.80E-06	2.65E-06	1.82E-06	2.25E-06
R10E8.1	18	23	31	29	7.36E-06	1.26E-05	6.51E-06	5.44E-06
R10E8.2	2	3	2	1	1.49E-05	1.23E-05	1.50E-05	9.90E-06
R10E8.3	24	46	12	39	2.80E-06	2.65E-06	1.82E-06	2.25E-06
R10E8.4	16	12	16	13	2.80E-06	2.65E-06	1.82E-06	2.25E-06
R10E8.5	3	4	2	1	2.80E-06	2.67E-06	1.82E-06	2.25E-06
R10E8.6	53	63	53	88	2.80E-06	2.65E-06	1.82E-06	2.25E-06
R10E8.7	7	2	3	9	2.80E-06	2.65E-06	1.82E-06	2.25E-06
R10E8.8	17	23	13	17	2.80E-06	2.65E-06	1.82E-06	2.25E-06
R10E9.1.1	447	523	415	707	2.80E-06	2.65E-06	1.82E-06	2.36E-06
R10E9.1.2	223	242	217	354	2.80E-06	2.65E-06	1.82E-06	2.25E-06
R10E9.2	84	192	75	60	2.22E-05	2.45E-05	1.34E-05	2.82E-05
R10F2.1	80	140	102	143	1.11E-05	1.13E-05	7.00E-06	1.41E-05
R10F2.4	3	4	5	1	1.13E-05	2.45E-05	6.60E-06	6.52E-06
R10F2.5	2	1	3	2	2.80E-06	2.65E-06	1.82E-06	2.25E-06
R10F2.6	45	87	40	38	2.80E-06	2.65E-06	1.82E-06	2.25E-06

R10H1.1	51	97	53	48	2.80E-06	2.65E-06	1.82E-06	2.25E-06
R10H1.2a	8	5	5	5	2.80E-06	2.67E-06	1.82E-06	2.25E-06
R10H1.2b	6	5	4	3	9.18E-06	1.65E-05	6.21E-06	6.95E-06
R10H1.4	49	50	15	14	2.80E-06	2.65E-06	1.82E-06	2.25E-06
R10H1.5	18	51	16	11	2.80E-06	2.65E-06	1.82E-06	2.25E-06
R10H10.1.1	291	341	397	257	1.09E-05	1.05E-05	2.17E-06	2.50E-06
R10H10.1.2	242	279	264	202	2.80E-06	3.33E-06	1.82E-06	2.25E-06
R10H10.2	36	84	20	33	4.33E-05	4.80E-05	3.85E-05	3.07E-05
R10H10.3	677	833	517	557	3.94E-05	4.29E-05	2.80E-05	2.64E-05
R10H10.4	5	11	4	2	2.80E-06	5.11E-06	1.82E-06	2.25E-06
R10H10.5	16	32	20	47	6.43E-05	7.47E-05	3.19E-05	4.25E-05
R10H10.6	41	65	56	28	2.80E-06	4.79E-06	1.82E-06	2.25E-06
R10H10.7.1	330	374	434	707	2.80E-06	3.20E-06	1.82E-06	4.00E-06
R11.1	37	55	46	56	1.13E-05	1.68E-05	1.00E-05	6.18E-06
R11.2	32	32	16	10	1.32E-05	1.41E-05	1.13E-05	2.27E-05
R11.3	15	13	17	21	4.28E-06	6.00E-06	3.46E-06	5.20E-06
R11.4	6	6	23	5	5.60E-06	5.29E-06	1.82E-06	2.25E-06
R11.t1	0	0	2	0	2.80E-06	2.65E-06	1.82E-06	2.25E-06
R11.t2	0	0	2	0	2.80E-06	2.65E-06	4.92E-06	2.25E-06
R11.t3	0	0	2	0	2.80E-06	2.65E-06	2.00E-06	2.25E-06
R11.t4	0	0	2	0	2.80E-06	2.65E-06	2.00E-06	2.25E-06
R119.1	72	95	111	143	2.80E-06	2.65E-06	2.00E-06	2.25E-06
R119.2	51	58	128	153	2.80E-06	2.65E-06	2.00E-06	2.25E-06
R119.3	113	126	111	118	3.67E-06	4.58E-06	3.68E-06	5.85E-06
R119.4	5395	4863	6330	8999	3.84E-06	4.10E-06	6.25E-06	9.22E-06
R119.5	53	47	65	79	1.54E-05	1.62E-05	9.86E-06	1.29E-05
R119.6	164	175	219	295	2.01E-04	1.71E-04	1.54E-04	2.70E-04
R119.7	1233	974	1903	2715	2.80E-06	2.65E-06	1.82E-06	2.74E-06
R11A5.1a	497	605	520	859	1.12E-05	1.13E-05	9.73E-06	1.62E-05
R11A5.1b	507	628	542	892	6.79E-05	5.07E-05	6.82E-05	1.20E-04
R11A5.2	674	700	645	978	2.02E-05	2.32E-05	1.37E-05	2.80E-05
R11A5.3	25	54	17	14	2.02E-05	2.36E-05	1.40E-05	2.85E-05
R11A5.4c.1	2377	5478	3809	5294	6.31E-05	6.19E-05	3.93E-05	7.36E-05
R11A5.4c.2	2597	5829	4127	5566	5.26E-06	1.07E-05	2.33E-06	2.36E-06
R11A5.4c.3	2531	5714	4071	5477	1.31E-04	2.86E-04	1.37E-04	2.35E-04
R11A5.4d.1	2618	5870	4160	5614	1.45E-04	3.07E-04	1.50E-04	2.49E-04
R11A5.4d.2	2492	5684	3907	5464	1.30E-04	2.77E-04	1.36E-04	2.26E-04
R11A5.4d.3	2617	5877	4160	5618	1.45E-04	3.07E-04	1.50E-04	2.50E-04
R11A5.4d.4	2616	5871	4160	5616	1.39E-04	2.99E-04	1.42E-04	2.45E-04
R11A5.4d.5	2622	5883	4165	5621	1.46E-04	3.09E-04	1.51E-04	2.51E-04
R11A5.6	6	5	9	4	1.52E-04	3.22E-04	1.57E-04	2.62E-04
R11A5.7.1	287	534	91	145	1.46E-04	3.09E-04	1.51E-04	2.51E-04
R11A5.7.2	271	491	79	135	2.80E-06	2.65E-06	1.91E-06	2.25E-06
R11A8.1	110	160	186	168	1.43E-05	2.52E-05	2.95E-06	5.82E-06
R11A8.2	311	361	273	448	1.49E-05	2.56E-05	2.82E-06	5.98E-06
R11A8.3	9	10	13	12	7.42E-06	1.02E-05	8.16E-06	9.11E-06
R11A8.4.1	416	504	389	661	2.51E-05	2.75E-05	1.43E-05	2.90E-05
R11A8.4.2	377	473	350	625	2.80E-06	2.65E-06	1.82E-06	2.25E-06
R11A8.5	416	532	340	509	2.29E-05	2.62E-05	1.39E-05	2.92E-05
R11A8.6	1584	2054	1743	2683	2.27E-05	2.68E-05	1.37E-05	3.01E-05
R11A8.7a	2556	2132	2371	4366	4.02E-05	4.86E-05	2.14E-05	3.95E-05
R11A8.7b	2865	2356	2763	5009	4.66E-05	5.71E-05	3.34E-05	6.34E-05
R11A8.8	17	26	23	13	3.66E-05	2.88E-05	2.21E-05	5.02E-05
R11B5.1	151	182	117	253	3.86E-05	3.00E-05	2.42E-05	5.42E-05
R11B5.t1	0	1	1	1	2.80E-06	3.12E-06	1.90E-06	2.25E-06
R11B5.t2	0	0	1	0	7.20E-06	8.17E-06	3.63E-06	9.67E-06
R11B5.t3	0	0	1	0	2.80E-06	2.65E-06	1.82E-06	2.25E-06
R11D1.11	142	231	85	96	2.80E-06	2.65E-06	1.82E-06	2.25E-06
R11D1.1a	228	289	241	450	2.80E-06	2.65E-06	1.82E-06	2.25E-06

R11D1.2	4	6	5	7	1.89E-05	2.91E-05	7.38E-06	1.03E-05
R11D1.3	19	10	12	3	1.28E-05	1.53E-05	8.78E-06	2.02E-05
R11D1.4	0	0	1	0	2.80E-06	2.65E-06	1.82E-06	2.25E-06
R11D1.5	4	7	8	0	3.50E-06	2.65E-06	1.82E-06	2.25E-06
R11D1.6	3	3	4	1	2.80E-06	2.65E-06	1.82E-06	2.25E-06
R11D1.7	10	6	6	4	2.80E-06	2.65E-06	1.82E-06	2.25E-06
R11D1.8.1	5427	4806	10596	2895	2.80E-06	2.65E-06	1.82E-06	2.25E-06
R11D1.8.2	4295	3815	8289	2542	2.80E-06	2.65E-06	1.82E-06	2.25E-06
R11D1.8.3	4299	3818	8292	2542	1.40E-03	1.17E-03	1.78E-03	6.00E-04
R11D1.8.4	4291	3810	8288	2540	1.09E-03	9.13E-04	1.37E-03	5.17E-04
R11D1.8.5	4341	3849	8310	2566	1.08E-03	9.08E-04	1.36E-03	5.14E-04
R11D1.9	108	161	309	120	1.09E-03	9.16E-04	1.37E-03	5.19E-04
R11E3.1	56	104	37	46	1.10E-03	9.23E-04	1.37E-03	5.23E-04
R11E3.2	8	16	17	18	1.99E-05	2.80E-05	3.70E-05	1.77E-05
R11E3.4	48	71	66	82	3.67E-06	6.45E-06	1.82E-06	2.43E-06
R11E3.5a	29	51	29	44	2.80E-06	2.65E-06	1.82E-06	2.25E-06
R11E3.5b	14	39	21	30	2.80E-06	3.36E-06	2.15E-06	3.28E-06
R11E3.5c	16	44	23	43	2.80E-06	3.57E-06	1.82E-06	2.61E-06
R11E3.6.1	364	391	386	623	2.80E-06	5.11E-06	1.90E-06	3.35E-06
R11E3.6.2	364	391	386	623	2.80E-06	4.07E-06	1.82E-06	3.37E-06
R11E3.7a	270	349	349	460	1.34E-05	1.36E-05	9.26E-06	1.84E-05
R11E3.7b	112	139	185	246	1.35E-05	1.37E-05	9.33E-06	1.86E-05
R11E3.8.1	922	1056	1391	1758	1.47E-05	1.79E-05	1.23E-05	2.01E-05
R11E3.8.2	875	1014	1279	1674	1.54E-05	1.81E-05	1.66E-05	2.72E-05
R11E3.8.3	858	1001	1272	1667	4.38E-05	4.75E-05	4.31E-05	6.72E-05
R11F4.1.1	145	446	90	122	4.31E-05	4.71E-05	4.10E-05	6.62E-05
R11F4.1.2	121	354	71	105	4.31E-05	4.75E-05	4.15E-05	6.72E-05
R11F4.2a	2	6	6	6	8.32E-06	2.42E-05	3.37E-06	5.62E-06
R11F4.2b	5	10	6	10	8.51E-06	2.36E-05	3.26E-06	5.94E-06
R11F4.3	36	43	22	39	2.80E-06	2.65E-06	1.82E-06	2.25E-06
R11G1.1	27	34	31	14	2.80E-06	2.65E-06	1.82E-06	2.25E-06
R11G1.2	9	10	4	4	2.80E-06	3.04E-06	1.82E-06	2.36E-06
R11G1.3	11	5	5	6	2.80E-06	2.65E-06	1.82E-06	2.25E-06
R11G1.4a.1	96	159	120	171	2.80E-06	2.65E-06	1.82E-06	2.25E-06
R11G1.4a.2	65	127	95	132	2.80E-06	2.65E-06	1.82E-06	2.25E-06
R11G1.4b	93	159	118	168	4.84E-06	7.56E-06	3.94E-06	6.93E-06
R11G1.6a	164	258	155	197	5.04E-06	9.28E-06	4.79E-06	8.21E-06
R11G1.6b	163	255	153	197	5.88E-06	9.50E-06	4.87E-06	8.52E-06
R11G1.6c	137	229	121	171	6.10E-06	9.05E-06	3.75E-06	5.87E-06
R11G1.6d	138	232	124	171	5.96E-06	8.81E-06	3.64E-06	5.78E-06
R11G1.7	7	8	2	1	6.24E-06	9.87E-06	3.59E-06	6.25E-06
R11G10.1a	59	93	60	55	6.30E-06	1.00E-05	3.68E-06	6.27E-06
R11G10.1b.1	62	84	53	66	2.80E-06	2.67E-06	1.82E-06	2.25E-06
R11G10.1b.2	54	77	50	54	3.02E-06	4.50E-06	2.00E-06	2.25E-06
R11G10.2	5	5	14	6	3.81E-06	4.89E-06	2.13E-06	3.26E-06
R11G10.3	14	22	8	3	3.70E-06	4.97E-06	2.22E-06	2.97E-06
R11G11.1	34	21	96	33	2.80E-06	2.65E-06	1.82E-06	2.25E-06
R11G11.10	5	3	12	7	2.80E-06	2.72E-06	1.82E-06	2.25E-06
R11G11.11	7	3	13	10	2.80E-06	2.65E-06	4.45E-06	2.25E-06
R11G11.12	8	9	14	12	2.80E-06	2.65E-06	1.82E-06	2.25E-06
R11G11.13	4	7	6	4	2.80E-06	2.65E-06	1.82E-06	2.25E-06
R11G11.14	8	16	8	7	2.80E-06	2.65E-06	1.82E-06	2.25E-06
R11G11.15	6	9	7	2	2.80E-06	2.65E-06	1.82E-06	2.25E-06
R11G11.2a	11	10	10	10	2.80E-06	2.65E-06	1.82E-06	2.25E-06
R11G11.2b	15	14	12	11	2.80E-06	2.65E-06	1.82E-06	2.25E-06
R11G11.3	7	5	10	4	2.80E-06	2.65E-06	1.82E-06	2.25E-06
R11G11.4	1	7	5	2	2.80E-06	2.65E-06	1.82E-06	2.25E-06
R11G11.5	2	2	0	0	2.80E-06	2.65E-06	1.82E-06	2.25E-06
R11G11.6	16	46	19	19	2.80E-06	2.65E-06	1.82E-06	2.25E-06

R11G11.7	10	31	15	14	2.80E-06	2.65E-06	1.82E-06	2.25E-06
R11G11.8	5	6	10	5	4.06E-06	1.10E-05	3.13E-06	3.87E-06
R11G11.9	5	6	5	2	2.80E-06	7.06E-06	2.35E-06	2.70E-06
R11H6.1.1	1060	1731	1352	1725	2.80E-06	2.65E-06	1.82E-06	2.25E-06
R11H6.1.2	979	1577	1199	1602	2.80E-06	2.65E-06	1.82E-06	2.25E-06
R11H6.2.1	394	526	591	643	7.48E-05	1.15E-04	6.21E-05	9.77E-05
R11H6.2.2	361	462	536	608	7.36E-05	1.12E-04	5.87E-05	9.67E-05
R11H6.3	2	12	5	10	2.72E-05	3.44E-05	2.66E-05	3.57E-05
R11H6.4	27	52	44	46	2.91E-05	3.52E-05	2.81E-05	3.93E-05
R11H6.5	402	426	520	738	2.80E-06	2.65E-06	1.82E-06	2.25E-06
R11H6.6	27	42	31	45	4.37E-06	7.94E-06	4.63E-06	5.98E-06
R12A1.2.1	35	76	33	51	3.35E-05	3.36E-05	2.82E-05	4.95E-05
R12A1.2.2	30	61	22	46	2.88E-06	4.23E-06	2.15E-06	3.85E-06
R12A1.3	34	28	7	11	2.80E-06	4.79E-06	1.82E-06	2.74E-06
R12A1.4.1	147	191	201	221	2.80E-06	4.50E-06	1.82E-06	2.88E-06
R12B2.1a.1	83	191	68	103	4.98E-06	3.89E-06	1.82E-06	2.25E-06
R12B2.1a.2	72	171	53	94	9.44E-06	1.16E-05	8.40E-06	1.14E-05
R12B2.1a.3	74	182	56	96	4.87E-06	1.06E-05	2.61E-06	4.86E-06
R12B2.1b	19	54	27	27	4.73E-06	1.06E-05	2.28E-06	4.97E-06
R12B2.2	10	20	10	19	4.76E-06	1.11E-05	2.35E-06	4.95E-06
R12B2.3	8	10	55	8	2.86E-06	7.70E-06	2.66E-06	3.28E-06
R12B2.4	431	483	521	706	2.80E-06	3.70E-06	1.82E-06	2.99E-06
R12B2.5a	763	710	790	1359	2.80E-06	2.65E-06	5.38E-06	2.25E-06
R12B2.5b.1	1186	1119	1261	2164	3.09E-05	3.27E-05	2.43E-05	4.06E-05
R12B2.5b.2	1163	1097	1241	2148	3.28E-05	2.88E-05	2.21E-05	4.69E-05
R12B2.6	16	23	25	16	3.52E-05	3.13E-05	2.43E-05	5.15E-05
R12B2.7	2	3	3	1	3.48E-05	3.10E-05	2.42E-05	5.17E-05
R12B2.8	4	2	1	3	5.57E-06	7.59E-06	5.69E-06	4.48E-06
R12C12.10	24	54	11	6	2.80E-06	2.65E-06	1.82E-06	2.25E-06
R12C12.1a.1	1066	1207	1331	1687	2.80E-06	2.65E-06	1.82E-06	2.25E-06
R12C12.1a.2	1037	1180	1266	1641	8.06E-06	1.72E-05	2.41E-06	2.25E-06
R12C12.1b	624	710	717	937	3.87E-05	4.14E-05	3.15E-05	4.92E-05
R12C12.2.1	455	486	620	856	3.92E-05	4.22E-05	3.12E-05	4.99E-05
R12C12.2.2	366	384	483	706	3.92E-05	4.22E-05	2.93E-05	4.73E-05
R12C12.3	14	6	12	11	3.98E-05	4.02E-05	3.53E-05	6.02E-05
R12C12.4	2	2	2	1	4.00E-05	3.96E-05	3.43E-05	6.20E-05
R12C12.5	183	156	175	181	2.80E-06	2.65E-06	1.82E-06	2.25E-06
R12C12.6a	229	241	384	246	2.80E-06	2.65E-06	1.82E-06	2.25E-06
R12C12.7	262	262	211	244	2.28E-05	1.83E-05	1.42E-05	1.81E-05
R12C12.8a	157	171	126	293	2.84E-05	2.82E-05	3.10E-05	2.45E-05
R12C12.8b	89	86	55	132	2.82E-05	2.66E-05	1.48E-05	2.11E-05
R12C12.9a.1	113	228	88	98	1.01E-05	1.04E-05	5.27E-06	1.51E-05
R12C12.9a.2	92	188	73	83	1.11E-05	1.02E-05	4.48E-06	1.32E-05
R12C12.9b	93	189	78	85	1.27E-05	2.42E-05	6.43E-06	8.86E-06
R12E2.10	2076	1716	3624	3700	1.36E-05	2.62E-05	7.02E-06	9.85E-06
R12E2.11	522	513	583	620	1.41E-05	2.71E-05	7.71E-06	1.04E-05
R12E2.12	214	285	257	192	9.94E-05	7.76E-05	1.13E-04	1.42E-04
R12E2.13.1	251	313	374	396	5.47E-05	5.08E-05	3.97E-05	5.22E-05
R12E2.13.2	191	243	293	341	3.29E-05	4.14E-05	2.57E-05	2.37E-05
R12E2.14	109	155	253	41	4.13E-05	4.87E-05	4.01E-05	5.24E-05
R12E2.15	167	211	382	81	2.88E-05	3.46E-05	2.87E-05	4.13E-05
R12E2.16	1	7	11	23	4.10E-05	5.50E-05	6.19E-05	1.24E-05
R12E2.3.1	1233	1291	1668	1691	8.31E-05	9.92E-05	1.24E-04	3.24E-05
R12E2.3.2	1040	1127	1292	1469	2.80E-06	6.74E-06	7.29E-06	1.88E-05
R12E2.4a	103	136	82	91	1.14E-04	1.12E-04	1.00E-04	1.25E-04
R12E2.4b	75	97	68	73	1.07E-04	1.09E-04	8.62E-05	1.21E-04
R12E2.5	67	118	116	135	9.74E-06	1.21E-05	5.05E-06	6.90E-06
R12E2.6	10	4	9	3	1.05E-05	1.28E-05	6.20E-06	8.21E-06
R12E2.7	161	267	173	71	5.54E-06	9.23E-06	6.25E-06	8.97E-06

R12E2.8	11	6	5	3	4.98E-06	2.65E-06	2.92E-06	2.25E-06
R12E2.9	195	300	148	158	4.58E-05	7.17E-05	3.20E-05	1.62E-05
R12G8.1	2	2	5	1	2.80E-06	2.65E-06	1.82E-06	2.25E-06
R12G8.2	9	8	6	5	1.77E-05	2.57E-05	8.75E-06	1.15E-05
R12G8.t1	0	0	1	0	2.80E-06	2.65E-06	1.82E-06	2.25E-06
R12G8.t2	1	0	1	0	2.80E-06	2.65E-06	1.82E-06	2.25E-06
R12H7.2	1338	2184	2351	2794	2.80E-06	2.65E-06	1.82E-06	2.25E-06
R12H7.3	61	89	128	76	2.80E-06	2.65E-06	1.82E-06	2.25E-06
R12H7.4	8	7	8	3	1.02E-04	1.57E-04	1.17E-04	1.71E-04
R12H7.5	34	55	31	26	7.67E-06	1.06E-05	1.05E-05	7.69E-06
R13.2	4	5	2	2	2.80E-06	2.65E-06	1.82E-06	2.25E-06
R13.3	48	62	36	60	6.08E-06	9.28E-06	3.61E-06	3.73E-06
R13.4	441	601	470	771	2.80E-06	2.65E-06	1.82E-06	2.25E-06
R13.5	2	4	3	5	3.00E-06	3.65E-06	1.82E-06	3.01E-06
R134.1	10	15	38	13	1.46E-05	1.88E-05	1.01E-05	2.05E-05
R134.2	21	19	10	11	2.80E-06	2.65E-06	1.82E-06	2.25E-06
R13A1.1	5	1	2	0	2.80E-06	2.65E-06	1.82E-06	2.25E-06
R13A1.10	1	3	1	1	2.80E-06	2.65E-06	1.82E-06	2.25E-06
R13A1.2	960	1091	1096	1783	2.80E-06	2.65E-06	1.82E-06	2.25E-06
R13A1.3	39	65	24	39	2.80E-06	2.65E-06	1.82E-06	2.25E-06
R13A1.4	19	16	11	8	2.85E-05	3.06E-05	2.12E-05	4.25E-05
R13A1.5	7	15	16	3	3.39E-06	5.32E-06	1.82E-06	2.72E-06
R13A1.7	22	17	14	13	2.80E-06	2.65E-06	1.82E-06	2.25E-06
R13A1.8	4	12	13	3	2.80E-06	3.15E-06	2.31E-06	2.25E-06
R13A1.9	10	12	10	9	9.55E-06	6.98E-06	3.95E-06	4.54E-06
R13A5.10	24	38	12	27	2.80E-06	2.65E-06	1.82E-06	2.25E-06
R13A5.11	22	44	22	10	2.80E-06	2.65E-06	1.82E-06	2.25E-06
R13A5.12.1	878	1006	1076	1352	5.82E-06	8.70E-06	1.90E-06	5.26E-06
R13A5.12.2	820	896	985	1287	2.80E-06	3.65E-06	1.82E-06	2.25E-06
R13A5.1a.1	499	535	845	995	5.70E-05	6.16E-05	4.54E-05	7.04E-05
R13A5.1b	471	513	801	950	5.74E-05	5.92E-05	4.49E-05	7.24E-05
R13A5.1c	457	490	769	934	2.46E-05	2.50E-05	2.72E-05	3.95E-05
R13A5.1d	481	518	819	976	2.06E-05	2.12E-05	2.28E-05	3.33E-05
R13A5.3	39	123	40	36	2.37E-05	2.40E-05	2.60E-05	3.90E-05
R13A5.4	8	9	11	5	2.46E-05	2.50E-05	2.72E-05	4.00E-05
R13A5.5	26	41	20	35	1.04E-05	3.10E-05	6.94E-06	7.71E-06
R13A5.6	60	103	50	24	2.80E-06	2.65E-06	1.82E-06	2.25E-06
R13A5.7	85	100	132	181	2.80E-06	3.99E-06	1.82E-06	2.90E-06
R13A5.8.1	11623	8913	11128	11864	1.61E-05	2.61E-05	8.75E-06	5.17E-06
R13A5.8.2	10251	7751	9830	11138	6.08E-06	6.74E-06	6.12E-06	1.04E-05
R13A5.9	48	76	41	41	2.07E-03	1.50E-03	1.29E-03	1.70E-03
R13D11.1	1	5	3	5	1.82E-03	1.30E-03	1.14E-03	1.59E-03
R13D11.10	3	1	0	0	2.80E-06	3.86E-06	1.82E-06	2.25E-06
R13D11.3	6	8	6	3	2.80E-06	2.65E-06	1.82E-06	2.25E-06
R13D11.4	15	23	31	22	2.80E-06	2.65E-06	1.82E-06	2.25E-06
R13D11.5	3	6	3	4	2.80E-06	2.65E-06	1.82E-06	2.25E-06
R13D11.6	8	3	6	7	2.80E-06	2.65E-06	1.95E-06	2.25E-06
R13D11.8	15	14	11	7	2.80E-06	2.65E-06	1.82E-06	2.25E-06
R13D11.9	1	1	3	0	2.80E-06	2.65E-06	1.82E-06	2.25E-06
R13D7.1	3	8	13	4	2.80E-06	2.65E-06	1.82E-06	2.25E-06
R13D7.10	3	4	4	5	2.80E-06	2.65E-06	1.82E-06	2.25E-06
R13D7.11	1	2	0	0	2.80E-06	2.65E-06	1.82E-06	2.25E-06
R13D7.2	71	58	54	21	2.80E-06	2.65E-06	1.82E-06	2.25E-06
R13D7.3	2	6	3	5	2.80E-06	2.65E-06	1.82E-06	2.25E-06
R13D7.4	7	4	4	0	7.45E-06	5.77E-06	3.70E-06	2.25E-06
R13D7.5	5	4	4	3	2.80E-06	2.65E-06	1.82E-06	2.25E-06
R13D7.6	3	9	9	5	2.80E-06	2.65E-06	1.82E-06	2.25E-06
R13D7.7	13	18	16	8	2.80E-06	2.65E-06	1.82E-06	2.25E-06
R13D7.9	2	2	1	1	2.80E-06	2.65E-06	1.82E-06	2.25E-06

R13F6.1	124	161	117	126	2.80E-06	2.72E-06	1.82E-06	2.25E-06
R13F6.10	1333	1255	1290	1980	2.80E-06	2.65E-06	1.82E-06	2.25E-06
R13F6.2	0	3	5	0	2.78E-05	3.41E-05	1.71E-05	2.27E-05
R13F6.3	66	122	75	95	4.65E-05	4.14E-05	2.93E-05	5.55E-05
R13F6.4a	386	440	452	808	2.80E-06	2.65E-06	1.82E-06	2.25E-06
R13F6.4d	440	488	515	902	6.58E-06	1.15E-05	4.87E-06	7.60E-06
R13F6.5	4	14	2	3	5.63E-06	6.06E-06	4.28E-06	9.47E-06
R13F6.6a	48	90	51	66	5.68E-06	5.95E-06	4.32E-06	9.36E-06
R13F6.6b	24	36	27	34	2.80E-06	2.65E-06	1.82E-06	2.25E-06
R13F6.8	1	4	2	3	2.80E-06	3.97E-06	1.82E-06	2.47E-06
R13F6.9	41	66	31	47	2.80E-06	2.65E-06	1.82E-06	2.25E-06
R13G10.1	282	354	250	461	2.80E-06	2.65E-06	1.82E-06	2.25E-06
R13G10.2	83	137	142	187	3.50E-06	5.32E-06	1.82E-06	3.22E-06
R13G10.4	9	23	13	8	6.64E-06	7.86E-06	3.83E-06	8.70E-06
R13H4.1	25	35	35	31	3.53E-06	5.48E-06	3.92E-06	6.36E-06
R13H4.3	21	58	35	14	2.80E-06	2.65E-06	1.82E-06	2.25E-06
R13H4.6	6	6	5	0	2.80E-06	2.65E-06	1.82E-06	2.25E-06
R13H4.7	5	11	1	1	2.80E-06	5.40E-06	2.24E-06	2.25E-06
R13H4.8	4	14	7	4	2.80E-06	2.65E-06	1.82E-06	2.25E-06
R13H7.1	7	9	10	7	2.80E-06	2.65E-06	1.82E-06	2.25E-06
R13H7.2a	22	51	29	10	2.80E-06	2.65E-06	1.82E-06	2.25E-06
R13H7.2b	12	27	15	4	2.80E-06	2.65E-06	1.82E-06	2.25E-06
R13H7.2c	12	27	15	4	2.80E-06	2.65E-06	1.82E-06	2.25E-06
R13H7.3	4	3	6	2	2.80E-06	2.65E-06	1.82E-06	2.25E-06
R13H8.1a	719	671	1026	1680	2.80E-06	2.65E-06	1.82E-06	2.25E-06
R13H8.1b	950	892	1320	2005	2.80E-06	2.65E-06	1.82E-06	2.25E-06
R13H8.1c	961	893	1334	2013	3.13E-05	2.76E-05	2.90E-05	5.87E-05
R13H8.1d	533	506	673	1007	3.51E-05	3.11E-05	3.17E-05	5.95E-05
R13H8.1e.1	390	377	536	822	3.55E-05	3.11E-05	3.20E-05	5.97E-05
R13H8.1e.2	365	354	506	780	4.08E-05	3.66E-05	3.35E-05	6.19E-05
R13H8.1f	846	784	1156	1867	3.87E-05	3.54E-05	3.46E-05	6.56E-05
R13H9.1	41	100	44	19	3.98E-05	3.65E-05	3.59E-05	6.83E-05
R13H9.2	1947	2646	2817	747	3.32E-05	2.91E-05	2.95E-05	5.89E-05
R13H9.4	2152	2977	3485	937	5.82E-06	1.34E-05	4.06E-06	2.25E-06
R13H9.5	44	76	48	31	5.08E-04	6.53E-04	4.79E-04	1.57E-04
R13H9.6	57	82	33	19	5.47E-04	7.14E-04	5.76E-04	1.91E-04
R13H9.7	0	2	0	7	5.24E-06	8.54E-06	3.72E-06	2.97E-06
R144.1	40	112	67	44	5.57E-06	7.59E-06	2.10E-06	2.25E-06
R144.10	131	123	190	135	2.80E-06	2.65E-06	1.82E-06	5.74E-06
R144.11	104	118	130	76	2.80E-06	3.36E-06	1.82E-06	2.25E-06
R144.12	154	186	150	138	2.08E-05	1.85E-05	1.96E-05	1.72E-05
R144.13	74	73	62	68	1.15E-05	1.23E-05	9.31E-06	6.72E-06
R144.2a	890	828	2169	2693	1.53E-05	1.75E-05	9.69E-06	1.10E-05
R144.2b	196	210	232	368	1.69E-05	1.57E-05	9.18E-06	1.24E-05
R144.3	81	113	96	118	3.61E-05	3.17E-05	5.72E-05	8.76E-05
R144.4a	415	371	481	578	2.45E-05	2.48E-05	1.89E-05	3.69E-05
R144.4b	289	265	371	426	1.33E-05	1.76E-05	1.03E-05	1.56E-05
R144.5	171	147	245	308	3.31E-05	2.79E-05	2.49E-05	3.70E-05
R144.6	137	135	164	174	2.97E-05	2.57E-05	2.48E-05	3.52E-05
R144.7a	2753	2092	2821	4515	1.22E-05	9.89E-06	1.14E-05	1.76E-05
R144.7b	4580	3171	4802	6365	1.40E-05	1.31E-05	1.09E-05	1.43E-05
R144.9	117	147	117	103	1.18E-04	8.49E-05	7.89E-05	1.56E-04
R148.2	77	97	271	150	1.60E-04	1.05E-04	1.09E-04	1.79E-04
R148.3a	597	483	574	996	2.28E-05	2.71E-05	1.49E-05	1.61E-05
R148.3b	715	561	647	1155	2.18E-05	2.59E-05	4.99E-05	3.41E-05
R148.4	169	115	155	203	1.97E-05	1.51E-05	1.23E-05	2.64E-05
R148.5a	140	161	106	180	1.98E-05	1.47E-05	1.16E-05	2.57E-05
R148.5b	106	130	89	147	4.26E-05	2.74E-05	2.54E-05	4.11E-05
R148.6.1	239	299	481	286	8.48E-06	9.20E-06	4.17E-06	8.77E-06

R148.6.2	201	251	369	231	7.81E-06	9.07E-06	4.28E-06	8.70E-06
R148.7	22	42	20	15	5.21E-05	6.16E-05	6.82E-05	5.01E-05
R148.t1	0	0	1	0	4.26E-05	5.02E-05	5.08E-05	3.93E-05
R151.1	15	36	13	25	3.14E-06	5.69E-06	1.86E-06	2.25E-06
R151.10.1	128	176	137	152	2.80E-06	2.65E-06	1.82E-06	2.25E-06
R151.10.2	146	192	168	172	2.80E-06	3.36E-06	1.82E-06	2.25E-06
R151.11	0	0	3	0	1.72E-05	2.24E-05	1.20E-05	1.65E-05
R151.2a	940	1391	973	1424	2.30E-05	2.85E-05	1.72E-05	2.17E-05
R151.2b.1	912	1334	955	1406	2.80E-06	2.65E-06	1.82E-06	2.25E-06
R151.2b.2	1015	1466	1122	1551	8.63E-05	1.21E-04	5.81E-05	1.05E-04
R151.2b.3	985	1430	1073	1526	9.25E-05	1.28E-04	6.30E-05	1.15E-04
R151.2b.4	1035	1478	1130	1564	9.03E-05	1.23E-04	6.50E-05	1.11E-04
R151.2b.5	911	1328	951	1402	8.89E-05	1.22E-04	6.30E-05	1.11E-04
R151.2c	749	1053	762	1155	8.43E-05	1.14E-04	5.99E-05	1.02E-04
R151.2d.1	1269	1863	1264	1751	9.25E-05	1.27E-04	6.28E-05	1.14E-04
R151.2d.2	1044	1482	1140	1578	8.22E-05	1.09E-04	5.44E-05	1.02E-04
R151.3.1	10303	9606	13932	11339	7.34E-05	1.02E-04	4.76E-05	8.14E-05
R151.3.2	9312	8773	12126	10787	7.89E-05	1.06E-04	5.61E-05	9.58E-05
R151.4a	13	27	13	11	1.47E-03	1.30E-03	1.30E-03	1.30E-03
R151.4b	1	3	2	0	1.58E-03	1.40E-03	1.34E-03	1.47E-03
R151.5a	143	340	119	255	2.80E-06	3.02E-06	1.82E-06	2.25E-06
R151.5b	139	328	114	245	2.80E-06	2.65E-06	1.82E-06	2.25E-06
R151.7a.1	560	678	514	787	8.09E-06	1.81E-05	4.37E-06	1.16E-05
R151.7a.2	555	673	511	779	8.23E-06	1.83E-05	4.39E-06	1.16E-05
R151.7b	364	454	352	548	2.93E-05	3.35E-05	1.75E-05	3.31E-05
R151.8	385	373	376	612	3.08E-05	3.53E-05	1.84E-05	3.47E-05
R151.9	391	447	420	359	2.83E-05	3.34E-05	1.78E-05	3.42E-05
R153.1a	211	264	179	312	1.82E-05	1.67E-05	1.16E-05	2.33E-05
R153.1b	317	424	258	466	7.42E-05	8.02E-05	5.19E-05	5.47E-05
R153.1c	225	273	187	324	1.43E-05	1.69E-05	7.91E-06	1.70E-05
R153.1d	231	283	196	329	1.32E-05	1.67E-05	6.98E-06	1.56E-05
R153.1e	228	275	186	326	1.31E-05	1.50E-05	7.07E-06	1.51E-05
R153.1f	16	20	14	15	1.22E-05	1.42E-05	6.76E-06	1.40E-05
R153.1g	242	286	199	343	1.34E-05	1.52E-05	7.09E-06	1.53E-05
R155.1a.1	953	963	775	981	3.14E-06	3.70E-06	1.82E-06	2.36E-06
R155.1a.2	763	824	665	920	1.18E-05	1.31E-05	6.29E-06	1.34E-05
R155.1b.1	745	807	664	913	6.85E-05	6.54E-05	3.62E-05	5.66E-05
R155.1b.2	338	513	386	612	5.45E-05	5.56E-05	3.09E-05	5.28E-05
R155.1b.3	763	824	665	920	5.75E-05	5.89E-05	3.33E-05	5.66E-05
R155.2	61	118	72	47	3.63E-05	5.20E-05	2.69E-05	5.27E-05
R155.3	28	40	39	24	5.89E-05	6.01E-05	3.34E-05	5.71E-05
R155.4	20	14	20	18	2.80E-06	3.09E-06	1.82E-06	2.25E-06
R160.1a	188	210	177	241	2.80E-06	2.65E-06	1.82E-06	2.25E-06
R160.2	2	0	1	1	2.80E-06	2.65E-06	1.82E-06	2.25E-06
R160.3	13	15	27	13	1.07E-05	1.13E-05	6.54E-06	1.10E-05
R160.4	5	4	0	0	2.80E-06	2.65E-06	1.82E-06	2.25E-06
R160.5	2	2	1	0	3.25E-06	3.54E-06	4.41E-06	2.61E-06
R160.6	1	2	1	0	2.80E-06	2.65E-06	1.82E-06	2.25E-06
R160.7	198	238	176	303	2.80E-06	2.65E-06	1.82E-06	2.25E-06
R166.1	78	88	81	118	2.80E-06	2.65E-06	1.82E-06	2.25E-06
R166.2.1	524	591	699	897	8.46E-06	9.60E-06	4.88E-06	1.04E-05
R166.2.2	454	516	608	820	5.80E-06	6.16E-06	3.92E-06	7.04E-06
R166.3	62	86	104	71	2.41E-05	2.57E-05	2.09E-05	3.31E-05
R166.4	233	254	248	312	2.74E-05	2.94E-05	2.39E-05	3.97E-05
R166.5a	1370	1528	2428	2445	1.04E-05	1.36E-05	1.13E-05	9.56E-06
R166.5b	486	632	972	1471	1.54E-05	1.59E-05	1.07E-05	1.66E-05
R166.6	932	907	1478	978	3.48E-05	3.66E-05	4.01E-05	4.99E-05
R17.1	7	5	10	5	2.22E-05	2.72E-05	2.88E-05	5.39E-05
R17.2	234	229	307	496	5.83E-05	5.36E-05	6.01E-05	4.91E-05

R17.3	22	14	48	67	2.80E-06	2.65E-06	1.82E-06	2.25E-06
R173.1	118	141	137	143	1.35E-05	1.25E-05	1.16E-05	2.30E-05
R173.3	49	53	55	40	2.80E-06	2.65E-06	2.62E-06	4.52E-06
R186.1	11	15	6	16	1.23E-05	1.39E-05	9.27E-06	1.19E-05
R186.2a	9	12	12	3	2.80E-06	2.65E-06	1.82E-06	2.25E-06
R186.2b	7	10	11	5	2.80E-06	2.65E-06	1.82E-06	2.25E-06
R186.3	431	472	433	424	2.80E-06	2.65E-06	1.82E-06	2.25E-06
R186.4	16	24	41	18	2.80E-06	2.65E-06	1.82E-06	2.25E-06
R186.5	9	15	8	13	5.41E-05	5.59E-05	3.53E-05	4.27E-05
R186.6	38	72	33	43	2.80E-06	2.65E-06	2.19E-06	2.25E-06
R186.7	301	277	216	330	2.80E-06	2.65E-06	1.82E-06	2.25E-06
R186.8	197	148	336	100	3.36E-06	6.00E-06	1.90E-06	3.06E-06
R193.1	5	3	2	0	2.48E-05	2.15E-05	1.16E-05	2.18E-05
R193.2	773	669	644	969	6.29E-05	4.46E-05	6.98E-05	2.56E-05
R193.3	16	10	17	5	2.80E-06	2.65E-06	1.82E-06	2.25E-06
R31.1	1318	1547	1160	2241	1.57E-05	1.28E-05	8.51E-06	1.58E-05
R31.2a	725	824	882	1171	2.80E-06	2.65E-06	1.82E-06	2.25E-06
R31.2b.1	531	576	598	863	1.16E-05	1.29E-05	6.67E-06	1.59E-05
R31.2b.2	537	580	603	870	3.67E-05	3.93E-05	2.90E-05	4.75E-05
R31.2b.3	530	574	597	863	3.16E-05	3.24E-05	2.32E-05	4.13E-05
R31.2c	368	411	410	583	3.44E-05	3.51E-05	2.52E-05	4.48E-05
R31.3	11	5	18	11	3.13E-05	3.20E-05	2.29E-05	4.10E-05
R52.1	17	20	22	27	6.12E-05	6.45E-05	4.43E-05	7.78E-05
R52.10	8	6	4	7	2.80E-06	2.65E-06	1.82E-06	2.25E-06
R52.2	457	518	355	612	2.80E-06	2.65E-06	1.82E-06	2.25E-06
R52.3	21	44	9	18	2.80E-06	2.65E-06	1.82E-06	2.25E-06
R52.4	3	0	4	0	1.67E-05	1.79E-05	8.45E-06	1.80E-05
R52.5	3	1	1	1	2.80E-06	4.07E-06	1.82E-06	2.25E-06
R52.6	2	0	2	0	2.80E-06	2.65E-06	1.82E-06	2.25E-06
R52.7	5	4	3	0	2.80E-06	2.65E-06	1.82E-06	2.25E-06
R52.8	15	15	19	8	2.80E-06	2.65E-06	1.82E-06	2.25E-06
R52.9	6	4	11	1	2.80E-06	2.65E-06	1.82E-06	2.25E-06
R53.1a.1	312	326	336	529	2.80E-06	2.65E-06	1.90E-06	2.25E-06
R53.1b.1	289	302	320	507	2.80E-06	2.65E-06	1.82E-06	2.25E-06
R53.1b.2	282	288	308	492	1.93E-05	1.91E-05	1.36E-05	2.64E-05
R53.2	276	326	382	376	2.07E-05	2.05E-05	1.50E-05	2.92E-05
R53.3a	43	36	58	47	2.05E-05	1.98E-05	1.45E-05	2.87E-05
R53.3b	43	36	58	47	3.42E-05	3.81E-05	3.08E-05	3.74E-05
R53.4.1	2441	3282	3849	3292	2.80E-06	2.65E-06	1.82E-06	2.25E-06
R53.4.2	2190	2970	3327	3077	2.80E-06	2.65E-06	1.82E-06	2.25E-06
R53.5	143	148	167	181	4.06E-04	5.16E-04	4.17E-04	4.40E-04
R53.6	225	170	347	170	3.95E-04	5.06E-04	3.90E-04	4.46E-04
R53.7a.1	484	510	803	859	2.22E-05	2.17E-05	1.69E-05	2.26E-05
R53.7b	323	359	538	606	3.93E-05	2.80E-05	3.94E-05	2.38E-05
R53.8	5	7	8	2	2.72E-05	2.71E-05	2.94E-05	3.88E-05
R57.1a	380	458	449	661	2.44E-05	2.57E-05	2.65E-05	3.68E-05
R57.1b.1	312	350	355	556	2.80E-06	2.65E-06	1.82E-06	2.25E-06
R57.1b.2	298	336	338	539	1.78E-05	2.02E-05	1.36E-05	2.48E-05
R57.1c.1	376	452	446	657	1.93E-05	2.05E-05	1.43E-05	2.77E-05
R57.1c.2	377	453	446	660	1.90E-05	2.02E-05	1.40E-05	2.76E-05
R57.1c.3	363	439	429	643	1.84E-05	2.09E-05	1.42E-05	2.59E-05
R57.2	48	52	17	9	1.81E-05	2.06E-05	1.39E-05	2.55E-05
R74.1.1	1701	1771	1941	2926	1.78E-05	2.04E-05	1.37E-05	2.53E-05
R74.1.2	1625	1708	1862	2814	8.26E-06	8.46E-06	1.90E-06	2.25E-06
R74.2	39	84	22	16	5.07E-05	4.99E-05	3.76E-05	7.00E-05
R74.5b.1	287	272	443	492	4.87E-05	4.84E-05	3.64E-05	6.78E-05
R74.5b.2	235	219	339	414	3.64E-06	7.38E-06	1.82E-06	2.25E-06
R74.5b.3	272	247	391	451	1.81E-05	1.62E-05	1.82E-05	2.49E-05
R74.5b.4	322	305	509	535	2.80E-05	2.47E-05	2.63E-05	3.97E-05

R74.5b.5	317	299	494	518	2.49E-05	2.13E-05	2.33E-05	3.32E-05
R74.6	97	148	116	142	3.68E-05	3.29E-05	3.78E-05	4.91E-05
R74.7	274	340	313	405	2.89E-05	2.58E-05	2.93E-05	3.79E-05
R74.8a	975	799	1224	1659	9.49E-06	1.37E-05	7.38E-06	1.12E-05
R74.8b	847	676	1033	1421	2.79E-05	3.26E-05	2.07E-05	3.31E-05
R90.1	128	242	112	180	6.62E-05	5.12E-05	5.41E-05	9.05E-05
R90.2	31	47	22	16	6.90E-05	5.20E-05	5.48E-05	9.30E-05
R90.3	0	14	3	10	5.54E-06	9.87E-06	3.15E-06	6.25E-06
R90.4	8	24	5	4	6.94E-06	9.92E-06	3.21E-06	2.88E-06
R90.5	20	29	18	15	2.80E-06	4.18E-06	1.82E-06	2.54E-06
SSSD1.1	11	11	9	12	2.80E-06	4.87E-06	1.82E-06	2.25E-06
T01A4.1a	467	605	772	1283	2.80E-06	2.65E-06	1.82E-06	2.25E-06
T01A4.1b	283	395	517	765	2.80E-06	2.65E-06	1.82E-06	2.25E-06
T01A4.1c	557	700	853	1443	1.43E-05	1.75E-05	1.54E-05	3.16E-05
T01A4.2	72	61	88	146	1.65E-05	2.18E-05	1.97E-05	3.60E-05
T01A4.3	32	42	16	27	1.55E-05	1.84E-05	1.55E-05	3.23E-05
T01B10.1	7	7	9	12	1.02E-05	8.15E-06	8.09E-06	1.66E-05
T01B10.2	230	314	174	71	2.80E-06	2.65E-06	1.82E-06	2.25E-06
T01B10.4a.1	75	133	78	106	2.80E-06	2.65E-06	1.82E-06	2.25E-06
T01B10.4a.2	66	113	71	94	3.07E-05	3.96E-05	1.51E-05	7.60E-06
T01B10.4b	77	131	79	112	4.59E-06	7.70E-06	3.12E-06	5.22E-06
T01B10.5	15	9	8	4	4.70E-06	7.62E-06	3.30E-06	5.40E-06
T01B11.1	7	6	14	9	4.65E-06	7.49E-06	3.12E-06	5.44E-06
T01B11.2b	96	200	93	124	2.80E-06	2.65E-06	1.82E-06	2.25E-06
T01B11.3	78	89	147	148	2.80E-06	2.65E-06	1.82E-06	2.25E-06
T01B11.4	130	200	59	59	1.80E-05	3.54E-05	1.14E-05	1.87E-05
T01B11.5	83	132	56	77	9.52E-06	1.03E-05	1.17E-05	1.45E-05
T01B4.1	9	11	10	17	1.54E-05	2.24E-05	4.54E-06	5.60E-06
T01B4.3	7	23	32	37	5.74E-06	8.60E-06	2.51E-06	4.27E-06
T01B4.t1	0	0	4	1	2.80E-06	2.65E-06	1.82E-06	2.25E-06
T01B4.t2	0	0	4	1	2.80E-06	5.21E-06	4.99E-06	7.11E-06
T01B4.t3	0	0	4	1	2.80E-06	2.65E-06	3.99E-06	2.25E-06
T01B4.t4	0	0	4	1	2.80E-06	2.65E-06	3.99E-06	2.25E-06
T01B6.1	19	21	10	8	2.80E-06	2.65E-06	3.99E-06	2.25E-06
T01B6.3a	15	31	14	26	2.80E-06	2.65E-06	3.99E-06	2.25E-06
T01B6.3b	27	36	52	48	2.97E-06	3.09E-06	1.82E-06	2.25E-06
T01B6.4	7	8	5	6	2.80E-06	2.65E-06	1.82E-06	2.25E-06
T01B7.1	0	4	0	0	2.80E-06	2.83E-06	2.82E-06	3.22E-06
T01B7.2	4	11	34	9	2.80E-06	2.65E-06	1.82E-06	2.25E-06
T01B7.3	110	123	133	168	2.80E-06	2.65E-06	1.82E-06	2.25E-06
T01B7.4	169	187	404	211	2.80E-06	2.65E-06	2.59E-06	2.25E-06
T01B7.5a	410	521	497	647	1.97E-05	2.08E-05	1.55E-05	2.42E-05
T01B7.5b	137	176	124	191	3.42E-05	3.58E-05	5.32E-05	3.43E-05
T01B7.6	1066	1087	1905	2462	3.26E-05	3.91E-05	2.57E-05	4.13E-05
T01B7.7	350	678	217	138	2.69E-05	3.27E-05	1.59E-05	3.01E-05
T01B7.8	17	8	7	11	4.27E-05	4.12E-05	4.97E-05	7.92E-05
T01B7.9	13	19	3	2	3.37E-05	6.16E-05	1.36E-05	1.07E-05
T01C1.2	14	18	23	19	3.84E-06	2.65E-06	1.82E-06	2.25E-06
T01C1.4	17	21	8	3	3.36E-06	4.66E-06	1.82E-06	2.25E-06
T01C2.1	65	77	28	91	2.80E-06	2.65E-06	1.82E-06	2.25E-06
T01C3.1	617	528	880	1071	6.83E-06	7.96E-06	2.10E-06	2.25E-06
T01C3.10	4	8	15	10	2.80E-06	2.65E-06	1.82E-06	2.68E-06
T01C3.11	3	5	11	1	2.96E-05	2.39E-05	2.74E-05	4.12E-05
T01C3.2	259	330	356	188	2.80E-06	2.65E-06	1.82E-06	2.25E-06
T01C3.3	600	812	1252	720	2.80E-06	2.65E-06	2.24E-06	2.25E-06
T01C3.4	22	26	9	8	7.48E-05	9.00E-05	6.69E-05	4.36E-05
T01C3.5	10	21	3	5	7.97E-05	1.02E-04	1.08E-04	7.68E-05
T01C3.6.1	4457	4692	7345	2668	2.80E-06	2.78E-06	1.82E-06	2.25E-06
T01C3.6.2	3996	4279	5104	2199	2.80E-06	3.04E-06	1.82E-06	2.25E-06

T01C3.7.1	2007	2024	1160	1771	9.38E-04	9.33E-04	1.01E-03	4.51E-04
T01C3.7.2	2027	2043	1253	1799	9.30E-04	9.41E-04	7.73E-04	4.11E-04
T01C3.7.3	1824	1850	1051	1624	1.90E-04	1.81E-04	7.15E-05	1.35E-04
T01C3.8	254	281	288	416	1.98E-04	1.88E-04	7.95E-05	1.41E-04
T01C3.9	360	379	541	548	1.68E-04	1.61E-04	6.29E-05	1.20E-04
T01C4.1	20	22	35	24	1.43E-05	1.49E-05	1.05E-05	1.88E-05
T01C4.2a	25	15	16	5	3.74E-05	3.72E-05	3.66E-05	4.58E-05
T01C4.2b	15	12	11	5	2.80E-06	2.65E-06	1.82E-06	2.25E-06
T01C4.2c	14	14	15	4	2.83E-06	2.65E-06	1.82E-06	2.25E-06
T01C4.3	6	4	10	2	2.80E-06	2.65E-06	1.82E-06	2.25E-06
T01C4.4	5	4	3	4	2.80E-06	2.65E-06	1.82E-06	2.25E-06
T01C4.5	4	8	9	8	2.80E-06	2.65E-06	1.82E-06	2.25E-06
T01C4.6	5	9	21	4	2.80E-06	2.65E-06	1.82E-06	2.25E-06
T01C4.7	4	2	2	0	2.80E-06	2.65E-06	1.82E-06	2.25E-06
T01C4.8	3	0	2	3	2.80E-06	2.65E-06	1.82E-06	2.25E-06
T01C8.1a	297	77	282	81	2.80E-06	2.65E-06	1.82E-06	2.25E-06
T01C8.1b	239	52	213	54	2.80E-06	2.65E-06	1.82E-06	2.25E-06
T01C8.1c.1	242	52	214	52	1.39E-05	3.41E-06	8.62E-06	3.06E-06
T01C8.1c.2	230	50	201	50	1.42E-05	2.91E-06	8.24E-06	2.59E-06
T01C8.2	105	190	119	62	1.44E-05	2.94E-06	8.31E-06	2.50E-06
T01C8.3	7	12	10	6	1.49E-05	3.04E-06	8.45E-06	2.59E-06
T01C8.4	4	3	15	3	2.98E-05	5.09E-05	2.20E-05	1.41E-05
T01C8.5	490	550	481	466	2.80E-06	2.65E-06	1.82E-06	2.25E-06
T01C8.7	17	20	23	11	2.80E-06	2.65E-06	1.82E-06	2.25E-06
T01D1.1	3	1	2	0	3.87E-05	4.10E-05	2.47E-05	2.96E-05
T01D1.2a.1	337	463	453	663	2.80E-06	2.65E-06	1.82E-06	2.25E-06
T01D1.2a.2	380	500	513	776	2.80E-06	2.65E-06	1.82E-06	2.25E-06
T01D1.2a.3	304	414	406	628	1.72E-05	2.24E-05	1.51E-05	2.72E-05
T01D1.2b	294	403	416	630	1.75E-05	2.18E-05	1.54E-05	2.87E-05
T01D1.3	34	77	25	46	1.69E-05	2.17E-05	1.46E-05	2.80E-05
T01D1.4	176	312	148	238	1.90E-05	2.45E-05	1.75E-05	3.26E-05
T01D1.5	6	10	6	8	5.24E-06	1.12E-05	2.51E-06	5.69E-06
T01D1.6	85	98	22	27	2.83E-05	4.74E-05	1.55E-05	3.07E-05
T01D1.7	2	4	5	3	2.80E-06	2.65E-06	1.82E-06	2.25E-06
T01D1.8	2	6	1	1	7.48E-06	8.15E-06	1.82E-06	2.25E-06
T01D3.1	26	34	20	35	2.80E-06	2.65E-06	1.82E-06	2.25E-06
T01D3.2	4	3	5	6	2.80E-06	2.65E-06	1.82E-06	2.25E-06
T01D3.3a	18	29	27	43	2.80E-06	2.65E-06	1.82E-06	2.25E-06
T01D3.3b	24	39	23	45	2.80E-06	2.65E-06	1.82E-06	2.25E-06
T01D3.4	15	23	29	21	2.80E-06	2.65E-06	1.82E-06	2.25E-06
T01D3.5	342	470	468	552	2.80E-06	2.65E-06	1.82E-06	2.25E-06
T01D3.6a	502	627	795	687	2.80E-06	2.65E-06	2.17E-06	2.25E-06
T01D3.6b	486	601	775	682	2.69E-05	3.50E-05	2.40E-05	3.49E-05
T01E8.1.1	62	113	56	76	1.93E-05	2.27E-05	1.99E-05	2.12E-05
T01E8.1.2	57	109	55	75	1.98E-05	2.31E-05	2.06E-05	2.23E-05
T01E8.2	35	33	56	58	4.17E-06	7.17E-06	2.44E-06	4.09E-06
T01E8.3	133	182	113	180	3.84E-06	6.96E-06	2.42E-06	4.07E-06
T01E8.4	464	514	605	659	2.80E-06	2.65E-06	2.93E-06	3.73E-06
T01E8.5	716	852	782	1151	3.50E-06	4.50E-06	1.93E-06	3.80E-06
T01E8.6.1	267	304	456	210	3.86E-05	4.04E-05	3.28E-05	4.40E-05
T01E8.6.2	215	256	302	161	1.98E-05	2.23E-05	1.41E-05	2.56E-05
T01E8.7	3	1	5	5	4.24E-05	4.56E-05	4.71E-05	2.68E-05
T01E8.8	63	81	65	61	3.96E-05	4.45E-05	3.62E-05	2.38E-05
T01E8.9	27	47	16	17	2.80E-06	2.65E-06	1.82E-06	2.25E-06
T01G1.1a	1011	1149	638	1405	6.44E-06	7.80E-06	4.32E-06	4.99E-06
T01G1.1b.1	385	451	231	476	5.77E-06	9.47E-06	2.22E-06	2.92E-06
T01G1.1b.2	475	542	290	583	2.35E-05	2.52E-05	9.66E-06	2.62E-05
T01G1.2	18	34	10	21	2.39E-05	2.64E-05	9.31E-06	2.37E-05
T01G1.3	1162	1168	1408	2216	2.74E-05	2.95E-05	1.09E-05	2.70E-05

T01G1.4	186	260	138	134	4.26E-06	7.59E-06	1.82E-06	3.98E-06
T01G5.1	41	63	39	38	3.62E-05	3.44E-05	2.86E-05	5.55E-05
T01G5.2	32	22	33	26	2.04E-05	2.69E-05	9.82E-06	1.18E-05
T01G5.3	1	8	5	4	4.37E-06	6.35E-06	2.71E-06	3.26E-06
T01G5.4	4	4	8	3	2.80E-06	2.65E-06	1.82E-06	2.25E-06
T01G5.5	4	7	1	5	2.80E-06	2.65E-06	1.82E-06	2.25E-06
T01G5.6	1	1	3	0	2.80E-06	2.65E-06	1.82E-06	2.25E-06
T01G5.7	37	63	51	67	2.80E-06	2.65E-06	1.82E-06	2.25E-06
T01G6.1	3	5	3	1	2.80E-06	2.65E-06	1.82E-06	2.25E-06
T01G6.10	5	4	3	2	4.14E-06	6.67E-06	3.72E-06	6.03E-06
T01G6.2	40	38	35	40	2.80E-06	2.65E-06	1.82E-06	2.25E-06
T01G6.3	3	4	5	9	2.80E-06	2.65E-06	1.82E-06	2.25E-06
T01G6.4	16	25	27	28	3.56E-06	3.20E-06	2.02E-06	2.86E-06
T01G6.5	11	12	14	11	2.80E-06	2.65E-06	1.82E-06	2.25E-06
T01G6.6	8	26	15	11	2.80E-06	2.65E-06	1.82E-06	2.25E-06
T01G6.7	28	39	11	20	2.80E-06	2.65E-06	1.82E-06	2.25E-06
T01G6.8	14	46	19	29	2.80E-06	2.65E-06	1.82E-06	2.25E-06
T01G6.9	9	5	17	4	2.80E-06	3.07E-06	1.82E-06	2.25E-06
T01G9.1	16	21	16	8	2.80E-06	3.54E-06	1.82E-06	2.25E-06
T01G9.2a	348	396	416	670	2.80E-06	2.65E-06	1.82E-06	2.25E-06
T01G9.2b.1	363	417	442	678	2.80E-06	2.65E-06	1.82E-06	2.25E-06
T01G9.2b.2	290	338	333	578	3.03E-05	3.26E-05	2.36E-05	4.69E-05
T01G9.3	29	31	27	40	2.69E-05	2.91E-05	2.13E-05	4.03E-05
T01G9.4	528	704	732	866	2.55E-05	2.80E-05	1.90E-05	4.08E-05
T01G9.5b	442	524	798	921	2.80E-06	2.65E-06	1.82E-06	2.25E-06
T01G9.6a.1	1074	1102	1451	1762	3.12E-05	3.93E-05	2.81E-05	4.11E-05
T01G9.6a.2	864	852	1060	1368	3.09E-05	3.46E-05	3.63E-05	5.17E-05
T01G9.6b.1	1066	1093	1435	1724	1.04E-04	1.01E-04	9.12E-05	1.37E-04
T01G9.6b.2	866	848	1070	1343	1.14E-04	1.06E-04	9.12E-05	1.45E-04
T01H10.1	6	6	6	7	1.09E-04	1.06E-04	9.57E-05	1.42E-04
T01H10.2	6	2	4	2	1.14E-04	1.06E-04	9.17E-05	1.42E-04
T01H10.3	3	5	8	3	2.80E-06	2.65E-06	1.82E-06	2.25E-06
T01H10.4	6	2	3	2	2.80E-06	2.65E-06	1.82E-06	2.25E-06
T01H10.5	16	21	17	3	2.80E-06	2.65E-06	1.82E-06	2.25E-06
T01H10.6	20	19	21	2	2.80E-06	2.65E-06	1.82E-06	2.25E-06
T01H10.7	8	3	7	7	2.80E-06	2.65E-06	1.82E-06	2.25E-06
T01H10.8	106	129	88	127	2.80E-06	2.65E-06	1.82E-06	2.25E-06
T01H3.1.1	1511	2212	1385	2356	2.80E-06	2.65E-06	1.82E-06	2.25E-06
T01H3.1.2	931	1222	756	1401	2.80E-06	2.65E-06	1.82E-06	2.25E-06
T01H3.2	261	408	265	530	1.74E-04	2.40E-04	1.04E-04	2.17E-04
T01H3.3.1	590	657	846	921	1.61E-04	2.00E-04	8.50E-05	1.94E-04
T01H3.3.2	513	584	729	850	9.77E-06	1.44E-05	6.45E-06	1.59E-05
T01H3.4.1	1025	853	1568	1926	3.10E-05	3.26E-05	2.89E-05	3.89E-05
T01H3.4.2	837	693	1256	1533	3.16E-05	3.40E-05	2.92E-05	4.21E-05
T01H3.5	12	30	7	11	6.76E-05	5.31E-05	6.73E-05	1.02E-04
T01H8.1a	1240	1177	1778	2219	6.38E-05	4.99E-05	6.23E-05	9.38E-05
T01H8.1b	1341	1339	1953	2428	2.80E-06	6.37E-06	1.82E-06	2.25E-06
T01H8.1c	1166	1117	1710	2150	5.90E-05	5.29E-05	5.50E-05	8.48E-05
T01H8.2	58	78	40	62	5.35E-05	5.04E-05	5.07E-05	7.78E-05
T02B11.1	7	7	7	2	5.74E-05	5.19E-05	5.48E-05	8.50E-05
T02B11.2	3	5	5	1	6.80E-06	8.65E-06	3.06E-06	5.85E-06
T02B11.3a	274	408	425	151	2.80E-06	2.65E-06	1.82E-06	2.25E-06
T02B11.3b	165	230	261	61	2.80E-06	2.65E-06	1.82E-06	2.25E-06
T02B11.4.1	30	74	60	28	4.43E-05	6.23E-05	4.47E-05	1.96E-05
T02B11.4.2	24	63	51	28	2.37E-05	3.12E-05	2.44E-05	7.02E-06
T02B11.5	2	7	3	3	5.12E-06	1.20E-05	6.67E-06	3.85E-06
T02B11.6	11	8	13	12	4.48E-06	1.11E-05	6.20E-06	4.21E-06
T02B11.7	15	27	13	13	2.80E-06	2.65E-06	1.82E-06	2.25E-06
T02B11.8	14	17	17	12	2.80E-06	2.65E-06	1.82E-06	2.25E-06

T02B11.9	12	8	10	2	2.80E-06	2.65E-06	1.82E-06	2.25E-06
T02B5.1	33	35	33	15	5.49E-06	6.32E-06	4.35E-06	3.78E-06
T02B5.2	2	5	7	0	2.80E-06	2.65E-06	1.82E-06	2.25E-06
T02B5.3	79	51	66	40	2.80E-06	2.65E-06	1.82E-06	2.25E-06
T02B5.4	4	5	3	2	2.80E-06	2.65E-06	1.82E-06	2.25E-06
T02C1.1	8	16	6	5	4.20E-06	2.65E-06	2.28E-06	2.25E-06
T02C1.2	21	24	23	18	2.80E-06	2.65E-06	1.82E-06	2.25E-06
T02C12.1	468	823	411	556	2.80E-06	3.31E-06	1.82E-06	2.25E-06
T02C12.2	190	201	270	339	2.80E-06	2.65E-06	1.82E-06	2.25E-06
T02C12.3	256	258	293	490	1.59E-05	2.64E-05	9.07E-06	1.52E-05
T02C12.4	28	36	7	23	1.69E-05	1.69E-05	1.57E-05	2.43E-05
T02C5.1a	468	863	384	613	2.10E-05	2.00E-05	1.57E-05	3.23E-05
T02C5.1b	450	803	366	593	7.76E-06	9.42E-06	1.82E-06	5.11E-06
T02C5.5a	87	108	78	104	4.00E-05	6.98E-05	2.14E-05	4.21E-05
T02C5.5b	117	146	107	159	4.28E-05	7.21E-05	2.26E-05	4.53E-05
T02C5.5c	113	140	106	154	2.80E-06	2.65E-06	1.82E-06	2.25E-06
T02C5.5d.1	18	26	22	42	2.80E-06	2.65E-06	1.82E-06	2.36E-06
T02C5.5d.2	28	42	31	55	2.80E-06	2.65E-06	1.82E-06	2.41E-06
T02C5.5d.3	130	163	124	179	2.80E-06	2.94E-06	1.82E-06	4.03E-06
T02D1.2	6	8	5	4	2.80E-06	3.15E-06	1.82E-06	3.51E-06
T02D1.3	8	9	10	6	2.80E-06	2.65E-06	1.82E-06	2.36E-06
T02D1.4	9	16	7	2	2.80E-06	2.65E-06	1.82E-06	2.25E-06
T02D1.5	71	160	50	123	2.80E-06	2.65E-06	1.82E-06	2.25E-06
T02D1.6	4	10	15	10	2.80E-06	2.65E-06	1.82E-06	2.25E-06
T02D1.7	5	4	2	3	3.42E-06	7.27E-06	1.82E-06	4.77E-06
T02D1.8	212	276	426	155	2.80E-06	2.65E-06	1.82E-06	2.25E-06
T02E1.1	16	30	8	7	2.80E-06	2.65E-06	1.82E-06	2.25E-06
T02E1.2	356	382	462	704	4.78E-05	5.87E-05	6.25E-05	2.80E-05
T02E1.3a	1180	857	1519	2033	2.80E-06	4.13E-06	1.82E-06	2.25E-06
T02E1.3b	935	682	1246	1723	2.00E-05	2.03E-05	1.69E-05	3.17E-05
T02E1.6	17	43	11	11	5.62E-05	3.85E-05	4.70E-05	7.77E-05
T02E1.7	21	54	19	11	5.13E-05	3.54E-05	4.45E-05	7.60E-05
T02E1.8	14	24	10	17	2.80E-06	6.19E-06	1.82E-06	2.25E-06
T02E9.1	4	14	3	6	2.91E-06	7.06E-06	1.82E-06	2.25E-06
T02E9.2a.1	354	474	138	101	3.14E-06	5.08E-06	1.82E-06	3.06E-06
T02E9.2a.2	303	427	116	96	2.80E-06	2.65E-06	1.82E-06	2.25E-06
T02E9.2b	321	440	119	92	3.52E-05	4.45E-05	8.93E-06	8.07E-06
T02E9.3	22	45	30	19	3.20E-05	4.26E-05	7.98E-06	8.14E-06
T02E9.4	1	4	5	4	3.23E-05	4.17E-05	7.78E-06	7.42E-06
T02E9.5.1	93	81	40	108	2.80E-06	2.65E-06	1.82E-06	2.25E-06
T02E9.5.2	58	63	24	63	2.80E-06	2.65E-06	1.82E-06	2.25E-06
T02E9.6	4	4	5	2	1.24E-05	1.02E-05	3.46E-06	1.15E-05
T02G5.1	7	3	6	8	1.30E-05	1.34E-05	3.52E-06	1.14E-05
T02G5.11	185	189	392	321	2.80E-06	2.65E-06	1.82E-06	2.25E-06
T02G5.12	322	362	502	654	2.80E-06	2.65E-06	1.82E-06	2.25E-06
T02G5.13a	432	838	639	1028	2.79E-05	2.69E-05	3.84E-05	3.89E-05
T02G5.13b.1	352	679	473	809	1.94E-05	2.06E-05	1.97E-05	3.16E-05
T02G5.13b.2	377	741	547	867	3.43E-05	6.28E-05	3.30E-05	6.55E-05
T02G5.13b.3	381	753	551	876	3.43E-05	6.25E-05	3.00E-05	6.33E-05
T02G5.14	3	4	5	6	3.53E-05	6.55E-05	3.33E-05	6.52E-05
T02G5.2	6	10	27	19	3.56E-05	6.64E-05	3.35E-05	6.57E-05
T02G5.3	6	9	11	11	2.80E-06	2.65E-06	1.82E-06	2.25E-06
T02G5.4	243	283	204	261	2.80E-06	2.65E-06	2.64E-06	2.29E-06
T02G5.6	9	13	8	21	2.80E-06	2.65E-06	1.82E-06	2.25E-06
T02G5.7	801	1026	1024	1152	1.63E-05	1.79E-05	8.87E-06	1.40E-05
T02G5.9a	2490	2623	2987	3813	3.11E-06	4.23E-06	1.82E-06	5.82E-06
T02G5.9b	2483	2619	2961	3773	6.85E-05	8.29E-05	5.70E-05	7.91E-05
T02G5.9c.1	2683	2815	3132	3960	1.60E-04	1.59E-04	1.25E-04	1.97E-04
T02G5.9c.2	2690	2819	3158	4000	1.49E-04	1.49E-04	1.16E-04	1.82E-04

T02G5.9c.3	2533	2661	3027	3892	1.45E-04	1.44E-04	1.10E-04	1.72E-04
T02G5.9c.4	2481	2618	2960	3772	1.55E-04	1.53E-04	1.18E-04	1.85E-04
T02G6.1	13	12	7	10	1.45E-04	1.44E-04	1.13E-04	1.79E-04
T02G6.2	10	7	6	11	1.44E-04	1.44E-04	1.12E-04	1.76E-04
T02G6.3	4	3	2	5	2.80E-06	2.65E-06	1.82E-06	2.25E-06
T02G6.4	12	19	24	23	2.80E-06	2.65E-06	1.82E-06	2.25E-06
T02G6.5	58	77	77	116	2.80E-06	2.65E-06	1.82E-06	2.25E-06
T02G6.6	14	5	11	13	2.80E-06	2.65E-06	1.82E-06	2.25E-06
T02G6.7	0	5	3	1	3.75E-06	4.73E-06	3.26E-06	6.05E-06
T02H6.10	0	1	0	1	2.80E-06	2.65E-06	1.82E-06	2.25E-06
T02H6.11.1	1333	1343	744	740	2.80E-06	2.65E-06	1.82E-06	2.25E-06
T02H6.11.2	829	847	515	532	2.80E-06	2.65E-06	1.82E-06	2.25E-06
T02H6.1a	287	199	184	389	2.69E-04	2.56E-04	9.75E-05	1.20E-04
T02H6.1b	357	262	221	452	1.69E-04	1.63E-04	6.82E-05	8.70E-05
T02H6.2	148	142	159	266	1.93E-05	1.27E-05	8.07E-06	2.11E-05
T02H6.3	2	0	2	0	2.12E-05	1.47E-05	8.55E-06	2.16E-05
T02H6.4	5	7	10	1	8.29E-06	7.51E-06	5.79E-06	1.20E-05
T02H6.5	8	8	39	15	2.80E-06	2.65E-06	1.82E-06	2.25E-06
T02H6.6	1	0	0	0	2.80E-06	2.65E-06	1.82E-06	2.25E-06
T02H6.7	6	12	2	0	2.80E-06	2.65E-06	2.35E-06	2.25E-06
T02H6.8	2	1	1	1	2.80E-06	2.65E-06	1.82E-06	2.25E-06
T02H6.9	3	3	1	0	2.80E-06	2.65E-06	1.82E-06	2.25E-06
T03D3.1	40	18	61	61	2.80E-06	2.65E-06	1.82E-06	2.25E-06
T03D3.10	9	9	13	2	2.80E-06	2.65E-06	1.82E-06	2.25E-06
T03D3.11	1	2	3	3	2.86E-06	2.65E-06	2.84E-06	3.51E-06
T03D3.12	5	7	12	7	2.80E-06	2.65E-06	1.82E-06	2.25E-06
T03D3.13	5	3	1	0	2.80E-06	2.65E-06	1.82E-06	2.25E-06
T03D3.14	4	4	5	0	2.80E-06	2.65E-06	1.82E-06	2.25E-06
T03D3.2	8	3	6	3	2.80E-06	2.65E-06	1.82E-06	2.25E-06
T03D3.3	2	11	7	4	2.80E-06	2.65E-06	1.82E-06	2.25E-06
T03D3.4	3	4	4	1	2.80E-06	2.65E-06	1.82E-06	2.25E-06
T03D3.5	48	62	56	45	2.80E-06	2.65E-06	1.82E-06	2.25E-06
T03D3.6	4	5	10	2	2.80E-06	2.65E-06	1.82E-06	2.25E-06
T03D3.7	7	4	8	3	4.09E-06	5.00E-06	3.12E-06	3.08E-06
T03D3.8	4	3	3	1	2.80E-06	2.65E-06	1.82E-06	2.25E-06
T03D3.9	5	5	2	4	2.80E-06	2.65E-06	1.82E-06	2.25E-06
T03D8.1a	580	573	792	1089	2.80E-06	2.65E-06	1.82E-06	2.25E-06
T03D8.1b	173	167	280	372	2.80E-06	2.65E-06	1.82E-06	2.25E-06
T03D8.1c	515	497	726	998	3.08E-05	2.87E-05	2.74E-05	4.64E-05
T03D8.2	194	226	396	198	1.54E-05	1.41E-05	1.63E-05	2.67E-05
T03D8.3	226	304	218	184	3.49E-05	3.19E-05	3.21E-05	5.44E-05
T03D8.4	6	11	10	10	4.01E-05	4.41E-05	5.33E-05	3.29E-05
T03D8.5	16	16	12	13	3.19E-05	4.05E-05	2.00E-05	2.09E-05
T03D8.7	5	11	7	7	2.80E-06	2.65E-06	1.82E-06	2.25E-06
T03E6.1	5	6	4	4	2.80E-06	2.65E-06	1.82E-06	2.25E-06
T03E6.2	10	10	11	6	2.80E-06	3.62E-06	1.82E-06	2.25E-06
T03E6.3	12	6	3	2	2.80E-06	2.65E-06	1.82E-06	2.25E-06
T03E6.4	5	6	3	2	2.80E-06	2.65E-06	1.82E-06	2.25E-06
T03E6.5	3	2	3	1	2.80E-06	2.65E-06	1.82E-06	2.25E-06
T03E6.6	6	4	61	15	2.80E-06	2.65E-06	1.82E-06	2.25E-06
T03E6.7.1	6121	6715	5124	9707	2.80E-06	2.65E-06	1.82E-06	2.25E-06
T03E6.7.2	5172	5809	4171	8425	2.80E-06	2.65E-06	3.97E-06	2.25E-06
T03E6.8	23	19	30	22	5.75E-04	5.96E-04	3.13E-04	7.33E-04
T03F1.10	38	50	64	51	5.71E-04	6.06E-04	3.00E-04	7.47E-04
T03F1.11	66	116	62	25	2.80E-06	2.65E-06	1.82E-06	2.25E-06
T03F1.12	86	118	230	101	4.45E-06	5.53E-06	4.88E-06	4.79E-06
T03F1.2	52	60	52	60	2.13E-05	3.54E-05	1.30E-05	6.48E-06
T03F1.3	906	1214	1660	1760	1.70E-05	2.20E-05	2.95E-05	1.60E-05
T03F1.5	159	205	130	78	8.37E-06	9.13E-06	5.45E-06	7.76E-06

T03F1.6a	10	21	26	25	7.14E-05	9.04E-05	8.51E-05	1.11E-04
T03F1.6b	10	19	25	23	1.57E-05	1.91E-05	8.36E-06	6.18E-06
T03F1.7	91	104	129	156	2.80E-06	2.65E-06	1.82E-06	2.25E-06
T03F1.9	684	732	1061	1406	2.80E-06	2.65E-06	1.82E-06	2.25E-06
T03F6.1	344	627	386	533	8.74E-06	9.42E-06	8.05E-06	1.20E-05
T03F6.2	290	285	271	402	2.83E-05	2.86E-05	2.86E-05	4.67E-05
T03F6.3	339	403	468	497	4.67E-05	8.04E-05	3.41E-05	5.81E-05
T03F6.4	17	28	8	10	2.12E-05	1.97E-05	1.29E-05	2.36E-05
T03F6.5	618	487	958	1115	4.05E-05	4.55E-05	3.64E-05	4.77E-05
T03F6.6	67	82	76	36	2.80E-06	2.65E-06	1.82E-06	2.25E-06
T03F6.7	7	4	8	2	3.91E-05	2.91E-05	3.94E-05	5.66E-05
T03F6.8	11	9	9	9	8.26E-06	9.52E-06	6.09E-06	3.55E-06
T03F7.1.1	80	112	73	86	2.80E-06	2.65E-06	1.82E-06	2.25E-06
T03F7.1.2	73	84	62	76	2.80E-06	2.65E-06	1.82E-06	2.25E-06
T03F7.2	2	5	8	5	4.12E-06	5.42E-06	2.44E-06	3.55E-06
T03F7.3	5	12	5	5	3.78E-06	4.10E-06	2.08E-06	3.15E-06
T03F7.4	12	15	12	10	2.80E-06	2.65E-06	1.82E-06	2.25E-06
T03F7.5	5	4	12	6	2.80E-06	2.65E-06	1.82E-06	2.25E-06
T03F7.6	5	8	5	5	2.80E-06	2.65E-06	1.82E-06	2.25E-06
T03F7.7a	52	125	56	81	2.80E-06	2.65E-06	1.82E-06	2.25E-06
T03F7.7b	55	122	50	78	2.80E-06	2.65E-06	1.82E-06	2.25E-06
T03G11.1	55	103	58	101	4.62E-06	1.05E-05	3.23E-06	5.76E-06
T03G11.10	2	6	12	4	5.10E-06	1.07E-05	3.02E-06	5.80E-06
T03G11.2	4	3	4	0	2.80E-06	2.65E-06	1.82E-06	2.25E-06
T03G11.3	9	4	8	4	2.80E-06	2.65E-06	1.82E-06	2.25E-06
T03G11.4	113	113	94	103	2.80E-06	2.65E-06	1.82E-06	2.25E-06
T03G11.5	2	9	2	7	2.80E-06	2.65E-06	1.82E-06	2.25E-06
T03G11.6	81	124	130	175	6.24E-06	5.90E-06	3.39E-06	4.57E-06
T03G11.8	29	74	15	26	2.80E-06	2.65E-06	1.82E-06	2.25E-06
T03G11.9	3	3	3	7	9.49E-06	1.37E-05	9.91E-06	1.65E-05
T03G6.1	80	180	52	42	4.76E-06	1.15E-05	1.82E-06	3.42E-06
T03G6.2a	55	91	47	69	2.80E-06	2.65E-06	1.82E-06	2.25E-06
T03G6.2b	89	116	81	98	7.87E-06	1.67E-05	3.33E-06	3.31E-06
T03G6.2c	50	90	49	72	3.30E-06	5.13E-06	1.82E-06	3.31E-06
T03G6.3.1	219	373	252	378	5.35E-06	6.59E-06	3.17E-06	4.75E-06
T03G6.t1	0	1	0	1	4.03E-06	6.85E-06	2.57E-06	4.66E-06
T04A11.1	65	93	68	85	1.37E-05	2.21E-05	1.03E-05	1.91E-05
T04A11.10	10	6	6	0	2.80E-06	2.65E-06	1.82E-06	2.25E-06
T04A11.11	14	11	15	11	7.53E-06	1.02E-05	5.14E-06	7.92E-06
T04A11.12	6	6	3	5	2.80E-06	2.65E-06	1.82E-06	2.25E-06
T04A11.2	20	26	18	33	2.80E-06	2.65E-06	1.82E-06	2.25E-06
T04A11.3	199	277	271	459	2.80E-06	2.65E-06	1.82E-06	2.25E-06
T04A11.4	65	93	68	85	2.80E-06	2.88E-06	1.82E-06	3.13E-06
T04A11.5	20	26	18	33	7.31E-06	9.63E-06	6.49E-06	1.36E-05
T04A11.6	440	414	625	1026	7.53E-06	1.02E-05	5.14E-06	7.92E-06
T04A11.7a	2	4	7	1	2.80E-06	2.88E-06	1.82E-06	3.13E-06
T04A11.7b	3	5	6	2	1.58E-05	1.40E-05	1.46E-05	2.96E-05
T04A11.8	9	8	12	5	2.80E-06	2.65E-06	1.82E-06	2.25E-06
T04A11.9	5	5	6	0	2.80E-06	2.65E-06	1.82E-06	2.25E-06
T04A6.1a	39	87	96	72	2.80E-06	2.65E-06	1.82E-06	2.25E-06
T04A6.1b	11	34	41	24	2.80E-06	2.65E-06	1.82E-06	2.25E-06
T04A6.2	1	3	7	7	7.11E-06	1.50E-05	1.14E-05	1.06E-05
T04A6.3	13	11	6	6	2.80E-06	6.67E-06	5.54E-06	4.00E-06
T04A8.1	5	4	11	1	2.80E-06	2.65E-06	1.82E-06	2.25E-06
T04A8.10	158	172	235	143	2.80E-06	2.65E-06	1.82E-06	2.25E-06
T04A8.11	234	262	217	216	2.80E-06	2.65E-06	1.82E-06	2.25E-06
T04A8.12	163	222	163	169	2.34E-05	2.40E-05	2.26E-05	1.70E-05
T04A8.13	92	122	69	52	3.30E-05	3.49E-05	1.99E-05	2.44E-05
T04A8.14	2237	2199	2514	3909	2.18E-05	2.81E-05	1.42E-05	1.82E-05

T04A8.15	293	362	313	498	4.34E-06	5.42E-06	2.11E-06	2.25E-06
T04A8.16	332	339	438	560	5.15E-05	4.79E-05	3.77E-05	7.24E-05
T04A8.2	4	4	2	3	1.48E-05	1.73E-05	1.03E-05	2.02E-05
T04A8.3	17	37	14	6	1.45E-05	1.40E-05	1.24E-05	1.96E-05
T04A8.4	43	31	42	41	2.80E-06	2.65E-06	1.82E-06	2.25E-06
T04A8.5	73	153	65	160	2.80E-06	2.83E-06	1.82E-06	2.25E-06
T04A8.6	544	518	825	1002	6.33E-06	4.31E-06	4.03E-06	4.86E-06
T04A8.7a	1029	995	1161	1621	5.35E-06	1.06E-05	3.10E-06	9.45E-06
T04A8.7b	814	820	928	1313	6.09E-05	5.48E-05	6.01E-05	9.01E-05
T04A8.8	554	610	768	775	5.00E-05	4.57E-05	3.67E-05	6.33E-05
T04A8.9.1	428	475	518	843	5.01E-05	4.76E-05	3.71E-05	6.49E-05
T04A8.9.2	113	155	101	238	5.61E-05	5.83E-05	5.06E-05	6.30E-05
T04B2.1	2	3	8	6	2.78E-05	2.92E-05	2.19E-05	4.40E-05
T04B2.2	27	42	25	9	1.67E-05	2.17E-05	9.73E-06	2.83E-05
T04B2.3a	41	46	28	32	2.80E-06	2.65E-06	1.82E-06	2.25E-06
T04B2.3b	35	37	16	25	2.80E-06	2.94E-06	1.82E-06	2.25E-06
T04B2.4	7	12	3	3	3.70E-06	3.94E-06	1.82E-06	2.32E-06
T04B2.6	8	13	12	8	4.84E-06	4.84E-06	1.82E-06	2.77E-06
T04B2.7	26	26	23	21	2.80E-06	2.65E-06	1.82E-06	2.25E-06
T04B2.8	5	8	4	0	2.80E-06	2.65E-06	1.82E-06	2.25E-06
T04B8.1	11	14	18	16	2.80E-06	2.65E-06	1.82E-06	2.25E-06
T04B8.2	5	8	8	11	2.80E-06	2.65E-06	1.82E-06	2.25E-06
T04B8.3	20	16	8	28	2.80E-06	2.65E-06	1.82E-06	2.25E-06
T04B8.5a	286	364	226	462	2.80E-06	2.65E-06	1.82E-06	2.25E-06
T04B8.5b	294	386	237	475	2.80E-06	2.65E-06	1.82E-06	2.45E-06
T04B8.5c.1	266	342	211	442	1.12E-05	1.35E-05	5.76E-06	1.45E-05
T04B8.5c.2	286	364	226	462	1.07E-05	1.33E-05	5.63E-06	1.39E-05
T04B8.5c.3	274	352	232	451	1.14E-05	1.39E-05	5.89E-06	1.52E-05
T04C10.1	147	242	159	277	1.12E-05	1.35E-05	5.76E-06	1.45E-05
T04C10.2a	457	476	389	530	1.11E-05	1.35E-05	6.12E-06	1.47E-05
T04C10.2b	712	817	587	882	5.94E-06	9.26E-06	4.19E-06	9.00E-06
T04C10.3	1	3	1	6	3.61E-05	3.55E-05	2.00E-05	3.36E-05
T04C12.1	69	117	61	37	3.55E-05	3.85E-05	1.90E-05	3.53E-05
T04C12.2a	3	4	6	4	2.80E-06	2.65E-06	1.82E-06	2.25E-06
T04C12.2b	3	3	4	4	1.10E-05	1.76E-05	6.34E-06	4.75E-06
T04C12.3.1	42	58	109	30	2.80E-06	2.65E-06	1.82E-06	2.25E-06
T04C12.3.2	24	37	41	15	2.80E-06	2.65E-06	1.82E-06	2.25E-06
T04C12.4.1	32179	30060	35115	47678	8.93E-06	1.16E-05	1.51E-05	5.13E-06
T04C12.4.2	32518	30418	35478	47850	4.96E-06	7.25E-06	5.52E-06	2.50E-06
T04C12.4.3	32059	29936	34894	47567	2.31E-03	2.04E-03	1.64E-03	2.75E-03
T04C12.4.4	28634	27385	30705	41229	2.57E-03	2.27E-03	1.82E-03	3.04E-03
T04C12.5	21611	20270	24412	31618	2.31E-03	2.04E-03	1.64E-03	2.76E-03
T04C12.6.1	32422	30490	34017	44532	2.78E-03	2.52E-03	1.94E-03	3.22E-03
T04C12.6.2	31623	29747	33194	44182	1.83E-03	1.62E-03	1.35E-03	2.15E-03
T04C12.6.3	30600	29048	31883	42754	2.67E-03	2.37E-03	1.82E-03	2.95E-03
T04C12.7	17	23	15	15	2.83E-03	2.52E-03	1.94E-03	3.18E-03
T04C12.8	14	40	39	10	2.73E-03	2.45E-03	1.85E-03	3.07E-03
T04C4.1a	231	232	483	736	3.14E-06	3.99E-06	1.82E-06	2.25E-06
T04C4.1b	220	222	465	700	4.17E-06	1.13E-05	7.58E-06	2.41E-06
T04C4.1c	34	30	51	50	8.57E-06	8.15E-06	1.17E-05	2.20E-05
T04C9.1a	810	826	1034	1468	8.34E-06	7.96E-06	1.15E-05	2.13E-05
T04C9.1b	599	641	774	1072	6.47E-06	5.40E-06	6.32E-06	7.65E-06
T04C9.1c	316	355	473	615	2.74E-05	2.64E-05	2.28E-05	3.99E-05
T04C9.2	1	2	2	1	2.59E-05	2.62E-05	2.18E-05	3.72E-05
T04C9.3	23	21	52	71	2.30E-05	2.44E-05	2.24E-05	3.59E-05
T04C9.4a	214	287	321	197	2.80E-06	2.65E-06	1.82E-06	2.25E-06
T04C9.4b	235	309	401	226	1.18E-05	1.02E-05	1.73E-05	2.92E-05
T04C9.4c	130	197	284	141	4.56E-05	5.78E-05	4.46E-05	3.38E-05
T04C9.6a.1	117	217	124	217	5.58E-05	6.93E-05	6.19E-05	4.31E-05

T04C9.6a.2	123	221	115	210	2.67E-05	3.82E-05	3.79E-05	2.32E-05
T04C9.6b	118	225	121	215	7.22E-06	1.27E-05	4.99E-06	1.08E-05
T04D1.2	16	14	14	6	7.76E-06	1.32E-05	4.72E-06	1.07E-05
T04D1.3a	598	583	768	1012	6.83E-06	1.23E-05	4.56E-06	1.00E-05
T04D1.3b	138	106	156	269	2.80E-06	2.65E-06	1.82E-06	2.25E-06
T04D1.4	800	840	660	1075	4.00E-05	3.68E-05	3.34E-05	5.43E-05
T04D3.1	70	103	96	132	5.00E-05	3.63E-05	3.68E-05	7.83E-05
T04D3.2	35	19	14	14	9.55E-06	9.47E-06	5.12E-06	1.03E-05
T04D3.3	31	53	24	23	5.35E-06	7.41E-06	4.76E-06	8.07E-06
T04D3.4	9	12	21	7	2.80E-06	2.65E-06	1.82E-06	2.25E-06
T04D3.8	59	39	101	46	2.80E-06	2.65E-06	1.82E-06	2.25E-06
T04D3.t4	0	0	1	0	2.80E-06	2.65E-06	1.82E-06	2.25E-06
T04F3.1	1763	2280	996	2722	1.81E-05	1.13E-05	2.02E-05	1.14E-05
T04F3.2	8	15	12	12	2.80E-06	2.65E-06	1.82E-06	2.25E-06
T04F3.3	26	68	30	20	1.87E-05	2.29E-05	6.87E-06	2.32E-05
T04F3.4	55	52	60	36	2.80E-06	2.65E-06	1.82E-06	2.25E-06
T04F8.1	143	165	137	201	2.80E-06	4.26E-06	1.82E-06	2.25E-06
T04F8.3	9	14	9	17	1.60E-05	1.43E-05	1.14E-05	8.43E-06
T04F8.4	15	17	26	13	1.48E-05	1.62E-05	9.26E-06	1.68E-05
T04F8.6	117	166	95	186	2.80E-06	2.78E-06	1.82E-06	2.86E-06
T04F8.8	440	528	839	703	2.80E-06	2.65E-06	1.82E-06	2.25E-06
T04F8.9	13	10	10	14	4.90E-06	6.56E-06	2.59E-06	6.25E-06
T04G9.1	101	105	77	137	7.91E-05	8.97E-05	9.82E-05	1.02E-04
T04G9.2	94	125	40	27	2.80E-06	2.65E-06	1.82E-06	2.25E-06
T04G9.4	60	114	69	84	2.80E-06	2.65E-06	1.82E-06	2.41E-06
T04G9.5.1	1472	1838	1067	1078	5.35E-06	6.72E-06	1.82E-06	2.25E-06
T04G9.5.2	1049	1340	745	879	6.78E-06	1.22E-05	5.08E-06	7.62E-06
T04G9.6	4	12	5	8	2.06E-04	2.43E-04	9.72E-05	1.21E-04
T04G9.7	315	389	690	351	1.83E-04	2.21E-04	8.47E-05	1.23E-04
T04H1.1	11	8	9	4	2.80E-06	2.65E-06	1.82E-06	2.25E-06
T04H1.10	6	4	4	2	8.73E-05	1.02E-04	1.24E-04	7.82E-05
T04H1.2.1	1176	1005	1125	1502	2.80E-06	2.65E-06	1.82E-06	2.25E-06
T04H1.2.2	1128	977	1075	1475	2.80E-06	2.65E-06	1.82E-06	2.25E-06
T04H1.3	6	3	18	1	7.01E-05	5.66E-05	4.37E-05	7.19E-05
T04H1.5	142	155	155	172	6.78E-05	5.55E-05	4.21E-05	7.12E-05
T04H1.6	21	46	8	6	2.80E-06	2.65E-06	3.02E-06	2.25E-06
T04H1.7	17	31	28	25	1.83E-05	1.89E-05	1.30E-05	1.78E-05
T04H1.8	15	24	33	19	2.80E-06	4.39E-06	1.82E-06	2.25E-06
T04H1.9	10	97	32	90	2.80E-06	2.65E-06	1.82E-06	2.25E-06
T05A1.1a	15	30	13	15	2.80E-06	2.65E-06	1.82E-06	2.25E-06
T05A1.1b.1	9	22	7	11	2.80E-06	7.41E-06	1.82E-06	5.82E-06
T05A1.2	5086	6001	11817	11686	2.80E-06	2.65E-06	1.82E-06	2.25E-06
T05A1.3	36	39	40	22	2.80E-06	2.65E-06	1.82E-06	2.25E-06
T05A1.5a	9	12	7	13	5.87E-04	6.54E-04	8.87E-04	1.08E-03
T05A1.5b	13	21	3	12	2.80E-06	2.65E-06	1.82E-06	2.25E-06
T05A10.1a	398	611	372	748	2.80E-06	2.65E-06	1.82E-06	2.25E-06
T05A10.1b	125	145	122	165	2.80E-06	2.65E-06	1.82E-06	2.25E-06
T05A10.1c	319	521	303	642	6.61E-06	9.58E-06	4.03E-06	9.99E-06
T05A10.1d	434	670	397	801	6.41E-06	7.04E-06	4.06E-06	6.79E-06
T05A10.1e	391	605	374	743	5.10E-06	7.86E-06	3.15E-06	8.23E-06
T05A10.1f	381	591	369	720	7.00E-06	1.02E-05	4.17E-06	1.04E-05
T05A10.1g	426	655	386	781	6.36E-06	9.28E-06	3.95E-06	9.69E-06
T05A10.1i	353	529	336	670	6.94E-06	1.02E-05	4.37E-06	1.05E-05
T05A10.1j	277	414	264	499	6.89E-06	1.00E-05	4.06E-06	1.01E-05
T05A10.2	5	6	5	0	5.57E-06	7.91E-06	3.46E-06	8.50E-06
T05A10.3	337	558	141	242	4.51E-06	6.35E-06	2.79E-06	6.50E-06
T05A10.4	14	25	12	11	2.80E-06	2.65E-06	1.82E-06	2.25E-06
T05A10.5	21	44	7	9	2.86E-05	4.46E-05	7.78E-06	1.65E-05
T05A10.6	7	13	5	2	2.80E-06	3.97E-06	1.82E-06	2.25E-06

T05A12.1	6	6	5	3	3.16E-06	6.24E-06	1.82E-06	2.25E-06
T05A12.2.1	368	612	451	654	2.80E-06	4.36E-06	1.82E-06	2.25E-06
T05A12.2.2	327	555	404	619	2.80E-06	2.65E-06	1.82E-06	2.25E-06
T05A12.3	312	420	296	474	1.96E-05	3.08E-05	1.56E-05	2.80E-05
T05A12.4a	282	173	302	301	2.02E-05	3.24E-05	1.62E-05	3.07E-05
T05A12.4b	140	64	146	133	1.50E-05	1.91E-05	9.27E-06	1.83E-05
T05A6.1	36	47	33	34	6.16E-06	3.57E-06	4.30E-06	5.29E-06
T05A6.2b	342	334	393	723	5.35E-06	2.65E-06	3.63E-06	4.09E-06
T05A6.4	3	5	3	6	5.01E-06	6.19E-06	2.99E-06	3.80E-06
T05A6.5	5	2	6	2	3.13E-05	2.89E-05	2.34E-05	5.32E-05
T05A6.6	5	7	13	5	2.80E-06	2.65E-06	1.82E-06	2.25E-06
T05A7.1	77	124	75	96	2.80E-06	2.65E-06	1.82E-06	2.25E-06
T05A7.10	6	11	7	3	2.80E-06	2.65E-06	1.82E-06	2.25E-06
T05A7.11	15	26	37	22	1.18E-05	1.80E-05	7.51E-06	1.19E-05
T05A7.2	4	4	0	2	2.80E-06	2.65E-06	1.82E-06	2.25E-06
T05A7.3	1	0	0	1	2.80E-06	2.65E-06	1.82E-06	2.25E-06
T05A7.4.1	66	118	61	76	2.80E-06	2.65E-06	1.82E-06	2.25E-06
T05A7.4.2	60	108	54	69	2.80E-06	2.65E-06	1.82E-06	2.25E-06
T05A7.5	38	51	31	23	1.28E-05	2.17E-05	7.73E-06	1.19E-05
T05A7.6	48	130	12	35	1.20E-05	2.03E-05	7.00E-06	1.10E-05
T05A7.7	9	12	7	9	3.11E-06	3.94E-06	1.82E-06	2.25E-06
T05A7.8	6	7	10	3	2.80E-06	5.90E-06	1.82E-06	2.25E-06
T05A7.9	13	2	22	15	2.80E-06	2.65E-06	1.82E-06	2.25E-06
T05A8.1	0	3	0	3	2.80E-06	2.65E-06	1.82E-06	2.25E-06
T05A8.2	0	5	3	2	3.92E-06	2.65E-06	4.32E-06	3.62E-06
T05A8.3	97	167	123	242	2.80E-06	2.65E-06	1.82E-06	2.25E-06
T05A8.4	100	129	101	225	2.80E-06	2.65E-06	1.82E-06	2.25E-06
T05A8.5	2	5	9	5	1.60E-05	2.61E-05	1.32E-05	3.21E-05
T05A8.6	2	3	1	1	5.07E-06	6.16E-06	3.33E-06	9.15E-06
T05A8.7	14	15	11	8	2.80E-06	2.65E-06	1.82E-06	2.25E-06
T05A8.8	2	0	5	2	2.80E-06	2.65E-06	1.82E-06	2.25E-06
T05B11.1	49	50	37	46	2.80E-06	2.65E-06	1.82E-06	2.25E-06
T05B11.2	9	3	6	3	2.80E-06	2.65E-06	1.82E-06	2.25E-06
T05B11.3	1208	1347	928	1330	5.43E-06	5.24E-06	2.68E-06	4.12E-06
T05B11.4	71	94	88	96	2.80E-06	2.65E-06	1.82E-06	2.25E-06
T05B11.5	3	3	1	2	1.33E-04	1.40E-04	6.63E-05	1.17E-04
T05B11.6	5	9	6	12	5.54E-06	6.96E-06	4.48E-06	6.03E-06
T05B11.7	6	8	6	4	2.80E-06	2.65E-06	1.82E-06	2.25E-06
T05B4.1	4	7	7	3	2.80E-06	2.65E-06	1.82E-06	2.25E-06
T05B4.10	2	4	1	1	2.80E-06	2.65E-06	1.82E-06	2.25E-06
T05B4.11	14	22	19	17	2.80E-06	2.65E-06	1.82E-06	2.25E-06
T05B4.12	11	16	5	0	2.80E-06	2.65E-06	1.82E-06	2.25E-06
T05B4.13	2	2	0	1	2.80E-06	2.65E-06	1.82E-06	2.25E-06
T05B4.2	14	32	16	23	2.80E-06	2.65E-06	1.82E-06	2.25E-06
T05B4.4	3	3	11	5	2.80E-06	2.65E-06	1.82E-06	2.25E-06
T05B4.5	13	7	10	4	2.80E-06	2.65E-06	1.82E-06	2.25E-06
T05B4.6	6	6	7	5	2.80E-06	2.65E-06	1.82E-06	2.25E-06
T05B4.7	6	7	4	1	2.80E-06	2.65E-06	1.82E-06	2.25E-06
T05B4.8	3	5	3	2	2.80E-06	2.65E-06	1.82E-06	2.25E-06
T05B4.9	4	2	0	1	2.80E-06	2.65E-06	1.82E-06	2.25E-06
T05B9.1	1268	1071	1828	2099	2.80E-06	2.65E-06	1.82E-06	2.25E-06
T05B9.2	3	4	2	1	2.80E-06	2.65E-06	1.82E-06	2.25E-06
T05C1.1	13	18	11	10	1.33E-04	1.06E-04	1.25E-04	1.77E-04
T05C1.2	10	5	22	4	2.80E-06	2.65E-06	1.82E-06	2.25E-06
T05C1.3	13	25	4	4	2.80E-06	2.65E-06	1.82E-06	2.25E-06
T05C1.4a	207	277	189	292	2.80E-06	2.65E-06	3.66E-06	2.25E-06
T05C1.4b	193	261	177	272	2.80E-06	3.76E-06	1.82E-06	2.25E-06
T05C1.4c	62	87	69	92	6.16E-06	7.80E-06	3.66E-06	6.99E-06
T05C1.5	1	0	5	2	6.19E-06	7.91E-06	3.70E-06	7.02E-06

T05C12.1	30	74	14	27	7.17E-06	9.50E-06	5.19E-06	8.55E-06
T05C12.10	546	883	109	145	2.80E-06	2.65E-06	1.82E-06	2.25E-06
T05C12.11	11	20	16	9	3.44E-06	8.01E-06	1.82E-06	2.47E-06
T05C12.2	10	5	8	8	1.59E-05	2.44E-05	2.08E-06	3.40E-06
T05C12.3	22	45	12	9	2.80E-06	2.65E-06	1.82E-06	2.25E-06
T05C12.4	23	32	28	16	2.80E-06	2.65E-06	1.82E-06	2.25E-06
T05C12.5	6	10	7	6	2.80E-06	5.13E-06	1.82E-06	2.25E-06
T05C12.6a	423	512	814	942	2.80E-06	2.65E-06	1.82E-06	2.25E-06
T05C12.6b	445	529	852	994	2.80E-06	2.65E-06	1.82E-06	2.25E-06
T05C12.6c	423	513	836	942	2.18E-05	2.50E-05	2.73E-05	3.90E-05
T05C12.7.1	2673	2453	2960	4037	2.27E-05	2.54E-05	2.82E-05	4.06E-05
T05C12.7.2	2387	2132	2616	3710	2.28E-05	2.61E-05	2.93E-05	4.08E-05
T05C12.8	5	10	16	18	1.60E-04	1.39E-04	1.15E-04	1.94E-04
T05C12.9	3	10	5	6	1.62E-04	1.36E-04	1.15E-04	2.02E-04
T05C3.2	20	35	50	18	2.80E-06	2.65E-06	1.82E-06	2.25E-06
T05C3.4	9	14	8	4	2.80E-06	2.65E-06	1.82E-06	2.25E-06
T05C3.5.1	1812	1815	2186	2543	2.80E-06	2.65E-06	1.82E-06	2.25E-06
T05C3.5.2	1548	1608	1772	2175	2.80E-06	2.65E-06	1.82E-06	2.25E-06
T05C3.7	9	6	24	11	1.32E-04	1.25E-04	1.04E-04	1.49E-04
T05C3.8	5	0	5	0	1.31E-04	1.28E-04	9.75E-05	1.48E-04
T05C7.1	22	31	14	30	2.80E-06	2.65E-06	1.82E-06	2.25E-06
T05D4.1.1	736	1183	1175	1120	2.80E-06	2.65E-06	1.82E-06	2.25E-06
T05D4.1.2	612	950	978	1001	4.54E-06	6.03E-06	1.88E-06	4.97E-06
T05D4.2	11	13	11	7	6.29E-05	9.55E-05	6.53E-05	7.68E-05
T05D4.3	21	42	23	19	5.47E-05	8.02E-05	5.69E-05	7.19E-05
T05D4.4	101	172	53	128	2.80E-06	2.65E-06	1.82E-06	2.25E-06
T05D4.5	42	71	26	25	2.80E-06	2.78E-06	1.82E-06	2.25E-06
T05E11.1.1	6566	6992	16652	8312	5.57E-06	8.94E-06	1.90E-06	5.67E-06
T05E11.1.2	5926	6276	13953	7706	3.16E-06	5.08E-06	1.82E-06	2.25E-06
T05E11.2	16	23	6	8	9.74E-04	9.80E-04	1.61E-03	9.90E-04
T05E11.3.1	4602	4464	5905	6872	1.03E-03	1.03E-03	1.58E-03	1.08E-03
T05E11.3.2	4297	4176	5540	6555	3.08E-06	4.18E-06	1.82E-06	2.25E-06
T05E11.4	299	307	273	397	1.99E-04	1.82E-04	1.66E-04	2.39E-04
T05E11.5	1557	1453	2375	3068	1.96E-04	1.80E-04	1.64E-04	2.40E-04
T05E11.6	50	63	140	191	2.62E-05	2.54E-05	1.56E-05	2.80E-05
T05E11.7	9	9	12	7	1.00E-04	8.85E-05	9.97E-05	1.59E-04
T05E11.8	15	19	20	10	5.77E-06	6.88E-06	1.05E-05	1.77E-05
T05E11.9	625	837	575	701	2.80E-06	2.65E-06	1.82E-06	2.25E-06
T05E12.1	8	4	7	1	2.80E-06	3.31E-06	2.39E-06	2.25E-06
T05E12.2	4	5	5	1	6.02E-05	7.62E-05	3.61E-05	5.43E-05
T05E12.3	10	23	17	27	2.80E-06	2.65E-06	1.82E-06	2.25E-06
T05E12.4	5	5	4	1	2.80E-06	2.65E-06	1.82E-06	2.25E-06
T05E12.7	22	75	29	44	2.80E-06	3.09E-06	1.82E-06	3.08E-06
T05E7.1	136	96	106	85	2.80E-06	2.65E-06	1.82E-06	2.25E-06
T05E7.2	23	42	12	12	2.83E-06	9.15E-06	2.44E-06	4.57E-06
T05E7.3	464	504	645	1183	1.04E-05	6.98E-06	5.30E-06	5.24E-06
T05E7.4	15	26	7	15	4.12E-06	7.09E-06	1.82E-06	2.25E-06
T05E7.5	43	44	25	35	1.64E-05	1.68E-05	1.48E-05	3.36E-05
T05E8.1	14	28	22	13	2.80E-06	3.73E-06	1.82E-06	2.25E-06
T05E8.2	2	7	7	4	3.70E-06	3.57E-06	1.82E-06	2.41E-06
T05E8.3	591	654	784	1151	2.80E-06	2.65E-06	1.82E-06	2.25E-06
T05F1.10	37	48	35	19	2.80E-06	2.65E-06	1.82E-06	2.25E-06
T05F1.11	96	88	77	75	2.29E-05	2.40E-05	1.98E-05	3.59E-05
T05F1.13	30	54	23	31	4.12E-06	5.05E-06	2.53E-06	2.25E-06
T05F1.2	876	1011	1408	2039	4.59E-06	3.99E-06	2.41E-06	2.88E-06
T05F1.3.1	5808	6493	15424	5619	3.19E-06	5.45E-06	1.82E-06	2.65E-06
T05F1.3.2	4883	5474	12055	5106	4.68E-05	5.10E-05	4.89E-05	8.74E-05
T05F1.4	145	165	227	198	1.28E-03	1.35E-03	2.22E-03	9.97E-04
T05F1.5	31	82	37	25	1.10E-03	1.16E-03	1.76E-03	9.21E-04

T05F1.6	2384	2309	3053	4516	1.97E-05	2.12E-05	2.01E-05	2.16E-05
T05F1.7	11	32	24	13	3.02E-06	7.56E-06	2.35E-06	2.25E-06
T05F1.8	79	121	53	30	6.35E-05	5.81E-05	5.29E-05	9.66E-05
T05F1.9	12	9	8	8	2.80E-06	2.65E-06	1.82E-06	2.25E-06
T05G11.1	78	67	155	173	6.89E-06	9.95E-06	3.01E-06	2.25E-06
T05G11.2	5	8	7	7	2.80E-06	2.65E-06	1.82E-06	2.25E-06
T05G11.3	3	4	17	7	5.91E-06	4.79E-06	7.63E-06	1.05E-05
T05G11.4	4	3	1	6	2.80E-06	2.65E-06	1.82E-06	2.25E-06
T05G11.5	5	4	2	0	2.80E-06	2.65E-06	1.82E-06	2.25E-06
T05G11.6	6	8	9	3	2.80E-06	2.65E-06	1.82E-06	2.25E-06
T05G11.7	8	5	5	4	2.80E-06	2.65E-06	1.82E-06	2.25E-06
T05G11.8	6	12	2	6	2.80E-06	2.65E-06	1.82E-06	2.25E-06
T05G5.1	169	232	91	133	2.80E-06	2.65E-06	1.82E-06	2.25E-06
T05G5.10	5221	3648	14337	3414	2.80E-06	2.65E-06	1.82E-06	2.25E-06
T05G5.11	14	18	7	9	1.27E-05	1.65E-05	4.45E-06	8.03E-06
T05G5.12	14	18	6	9	9.28E-04	6.13E-04	1.66E-03	4.87E-04
T05G5.2	4	7	9	3	4.51E-06	5.48E-06	1.82E-06	2.32E-06
T05G5.3.1	685	820	1075	1319	5.63E-06	6.82E-06	1.82E-06	2.90E-06
T05G5.3.2	621	757	964	1221	2.80E-06	2.65E-06	1.82E-06	2.25E-06
T05G5.4	4	11	13	4	6.41E-05	7.25E-05	6.55E-05	9.91E-05
T05G5.5	98	166	125	149	6.07E-05	6.99E-05	6.13E-05	9.59E-05
T05G5.6.1	1311	3422	1978	3075	2.80E-06	4.92E-06	3.99E-06	2.25E-06
T05G5.6.2	1042	2755	1595	2661	1.16E-05	1.86E-05	9.66E-06	1.42E-05
T05G5.7	1834	1337	2360	2220	1.45E-04	3.57E-04	1.42E-04	2.73E-04
T05G5.9a	390	516	280	558	1.33E-04	3.33E-04	1.33E-04	2.74E-04
T05G5.9b	370	494	262	539	2.07E-04	1.42E-04	1.73E-04	2.01E-04
T05H10.1	1493	1430	1464	2768	2.04E-05	2.55E-05	9.51E-06	2.34E-05
T05H10.2	253	332	156	260	2.09E-05	2.64E-05	9.66E-06	2.45E-05
T05H10.3	25	17	8	22	3.81E-05	3.44E-05	2.43E-05	5.67E-05
T05H10.4	199	243	251	395	2.21E-05	2.75E-05	8.89E-06	1.83E-05
T05H10.5a	955	1140	1460	1931	5.01E-06	3.20E-06	1.82E-06	3.53E-06
T05H10.5b	923	1104	1403	1889	1.41E-05	1.63E-05	1.16E-05	2.25E-05
T05H10.5c	2906	3641	3537	4570	3.62E-05	4.09E-05	3.60E-05	5.89E-05
T05H10.6a.1	1678	2133	1780	2329	3.50E-05	3.95E-05	3.46E-05	5.75E-05
T05H10.6a.2	1679	2137	1781	2330	6.53E-05	7.73E-05	5.17E-05	8.25E-05
T05H10.6a.3	1638	2105	1774	2325	1.53E-04	1.83E-04	1.05E-04	1.70E-04
T05H10.7a	806	905	963	1279	1.53E-04	1.84E-04	1.05E-04	1.70E-04
T05H10.7b.1	828	942	981	1317	1.46E-04	1.77E-04	1.03E-04	1.66E-04
T05H10.7b.2	800	901	964	1275	3.77E-05	4.00E-05	2.94E-05	4.81E-05
T05H10.7b.3	795	892	947	1269	3.55E-05	3.81E-05	2.74E-05	4.53E-05
T05H10.8	15	14	13	11	4.14E-05	4.40E-05	3.25E-05	5.30E-05
T05H4.1	310	265	258	432	4.12E-05	4.36E-05	3.19E-05	5.28E-05
T05H4.10	166	200	187	271	2.80E-06	2.65E-06	1.82E-06	2.25E-06
T05H4.11	569	622	700	832	3.35E-05	2.71E-05	1.82E-05	3.76E-05
T05H4.12.1	1347	1328	3500	1298	1.23E-05	1.40E-05	9.04E-06	1.61E-05
T05H4.12.2	1226	1172	2778	1189	5.57E-05	5.75E-05	4.46E-05	6.54E-05
T05H4.13a	710	845	780	1033	2.53E-04	2.35E-04	4.27E-04	1.96E-04
T05H4.13b	827	967	931	1170	2.36E-04	2.13E-04	3.49E-04	1.84E-04
T05H4.13c.1	732	865	794	1030	5.06E-05	5.69E-05	3.62E-05	5.91E-05
T05H4.13c.2	689	811	735	973	4.85E-05	5.36E-05	3.55E-05	5.51E-05
T05H4.14	436	495	860	1113	4.91E-05	5.48E-05	3.46E-05	5.55E-05
T05H4.15a	9	13	8	10	5.19E-05	5.77E-05	3.60E-05	5.89E-05
T05H4.15b.1	9	11	6	9	2.48E-05	2.66E-05	3.18E-05	5.08E-05
T05H4.15b.2	10	10	22	11	2.80E-06	2.65E-06	1.82E-06	2.25E-06
T05H4.15b.3	9	8	7	8	2.80E-06	2.65E-06	1.82E-06	2.25E-06
T05H4.15c	10	11	22	12	2.80E-06	2.65E-06	1.82E-06	2.25E-06
T05H4.2	14	32	17	7	2.80E-06	2.65E-06	1.82E-06	2.25E-06
T05H4.3	15	19	28	12	2.80E-06	2.65E-06	1.82E-06	2.25E-06
T05H4.4	24	23	29	25	2.80E-06	2.67E-06	1.82E-06	2.25E-06

T05H4.5.1	799	1252	1350	1793	2.80E-06	2.65E-06	1.82E-06	2.25E-06
T05H4.5.2	738	1136	1140	1593	2.94E-06	2.67E-06	2.31E-06	2.47E-06
T05H4.6.1	1628	1676	1312	1964	8.76E-05	1.30E-04	9.63E-05	1.58E-04
T05H4.6.2	1607	1662	1306	1948	8.87E-05	1.29E-04	8.92E-05	1.54E-04
T05H4.6.3	1183	1259	1000	1581	9.20E-05	8.95E-05	4.83E-05	8.92E-05
T05H4.7	12	13	4	6	8.51E-05	8.31E-05	4.50E-05	8.28E-05
T05H4.8	3	3	4	2	9.73E-05	9.78E-05	5.35E-05	1.04E-04
T06A1.1	7	14	8	6	2.80E-06	2.65E-06	1.82E-06	2.25E-06
T06A1.2	5	4	2	1	2.80E-06	2.65E-06	1.82E-06	2.25E-06
T06A1.4	8	6	7	7	2.80E-06	2.65E-06	1.82E-06	2.25E-06
T06A1.5	72	119	86	98	2.80E-06	2.65E-06	1.82E-06	2.25E-06
T06A1.6	6	21	20	31	2.80E-06	2.65E-06	1.82E-06	2.25E-06
T06A1.7	2	1	2	3	5.18E-06	8.09E-06	4.03E-06	5.67E-06
T06A10.1	210	201	171	304	2.80E-06	4.10E-06	2.68E-06	5.13E-06
T06A10.2	30	43	63	25	2.80E-06	2.65E-06	1.82E-06	2.25E-06
T06A10.3	73	96	105	83	7.95E-06	7.19E-06	4.21E-06	9.24E-06
T06A10.4	280	303	387	385	7.95E-06	1.08E-05	1.09E-05	5.31E-06
T06A4.1a	25	22	11	9	1.60E-05	1.99E-05	1.50E-05	1.46E-05
T06A4.1b	28	24	13	11	3.32E-05	3.39E-05	2.98E-05	3.66E-05
T06A4.2	25	39	15	6	2.80E-06	2.65E-06	1.82E-06	2.25E-06
T06A4.3a	90	147	32	53	2.80E-06	2.65E-06	1.82E-06	2.25E-06
T06A4.3b	73	113	25	48	5.24E-06	7.70E-06	2.04E-06	2.25E-06
T06C10.2	7	3	8	3	6.10E-06	9.39E-06	1.82E-06	2.88E-06
T06C10.3	41	67	23	23	5.54E-06	8.12E-06	1.82E-06	2.92E-06
T06C10.4.1	110	142	83	111	2.80E-06	2.65E-06	1.82E-06	2.25E-06
T06C10.4.2	85	105	52	87	2.80E-06	4.23E-06	1.82E-06	2.25E-06
T06C10.6	18	28	13	9	1.82E-05	2.22E-05	8.93E-06	1.48E-05
T06C12.1	6	7	6	8	1.86E-05	2.17E-05	7.40E-06	1.53E-05
T06C12.10.1	37	44	19	26	2.80E-06	2.65E-06	1.82E-06	2.25E-06
T06C12.10.2	26	33	13	15	2.80E-06	2.65E-06	1.82E-06	2.25E-06
T06C12.11	2	5	5	3	2.80E-06	3.02E-06	1.82E-06	2.25E-06
T06C12.12	1	0	0	3	2.80E-06	2.65E-06	1.82E-06	2.25E-06
T06C12.13	8	21	19	11	2.80E-06	2.65E-06	1.82E-06	2.25E-06
T06C12.14	1	4	1	2	2.80E-06	2.65E-06	1.82E-06	2.25E-06
T06C12.15	5	5	9	4	2.80E-06	2.65E-06	1.82E-06	2.25E-06
T06C12.2	4	3	16	4	2.80E-06	2.65E-06	1.82E-06	2.25E-06
T06C12.3	5	5	7	3	2.80E-06	2.65E-06	1.82E-06	2.25E-06
T06C12.4	59	69	75	89	2.80E-06	2.65E-06	1.82E-06	2.25E-06
T06C12.5	15	24	13	24	2.80E-06	2.65E-06	1.82E-06	2.25E-06
T06C12.6	18	21	17	19	5.91E-06	6.53E-06	4.88E-06	7.15E-06
T06C12.7	23	61	24	32	2.80E-06	2.65E-06	1.82E-06	2.25E-06
T06C12.8	9	11	17	13	2.80E-06	2.65E-06	1.82E-06	2.25E-06
T06C12.9	0	1	1	0	2.80E-06	5.11E-06	1.82E-06	2.27E-06
T06D10.2.1	447	572	382	580	2.80E-06	2.65E-06	1.82E-06	2.25E-06
T06D10.2.2	418	513	357	540	2.80E-06	2.65E-06	1.82E-06	2.25E-06
T06D4.2	3	7	8	5	2.87E-05	3.47E-05	1.60E-05	3.00E-05
T06D4.3	8	16	14	9	3.22E-05	3.73E-05	1.79E-05	3.34E-05
T06D4.4	57	68	54	53	2.80E-06	2.65E-06	1.82E-06	2.25E-06
T06D8.10	266	655	72	200	2.80E-06	2.65E-06	1.82E-06	2.25E-06
T06D8.1a	2354	2327	895	1418	3.11E-06	3.49E-06	1.91E-06	2.32E-06
T06D8.1b	2353	2324	895	1416	6.50E-06	1.51E-05	1.82E-06	3.91E-06
T06D8.2	10	19	10	17	4.48E-05	4.18E-05	1.11E-05	2.17E-05
T06D8.3	48	72	34	42	4.48E-05	4.18E-05	1.11E-05	2.17E-05
T06D8.5.1	281	334	263	331	2.80E-06	2.65E-06	1.82E-06	2.25E-06
T06D8.5.3	244	294	211	285	3.56E-06	5.05E-06	1.82E-06	2.50E-06
T06D8.6.1	858	765	1446	1306	2.36E-05	2.65E-05	1.44E-05	2.23E-05
T06D8.6.2	630	560	1011	973	2.29E-05	2.61E-05	1.29E-05	2.15E-05
T06D8.7.1	155	180	279	210	8.06E-05	6.78E-05	8.83E-05	9.85E-05
T06D8.7.2	138	167	221	184	8.24E-05	6.92E-05	8.61E-05	1.02E-04

T06D8.8.1	1283	1266	1494	1699	2.98E-05	3.27E-05	3.49E-05	3.25E-05
T06D8.8.2	1082	1083	1235	1487	2.66E-05	3.04E-05	2.77E-05	2.85E-05
T06D8.9.1	273	430	615	550	9.70E-05	9.04E-05	7.35E-05	1.03E-04
T06E4.1	1004	1108	1417	1869	1.04E-04	9.83E-05	7.73E-05	1.15E-04
T06E4.10	34	22	5	7	2.18E-05	3.24E-05	3.19E-05	3.52E-05
T06E4.11	35	38	16	16	2.73E-05	2.84E-05	2.51E-05	4.08E-05
T06E4.12	62	58	2	18	5.54E-06	3.39E-06	1.82E-06	2.25E-06
T06E4.13	0	2	1	1	4.73E-06	4.87E-06	1.82E-06	2.25E-06
T06E4.3a	931	914	1214	1902	9.72E-06	8.57E-06	1.82E-06	2.27E-06
T06E4.3b	942	906	1226	1898	2.80E-06	2.65E-06	1.82E-06	2.25E-06
T06E4.4	868	1065	1493	620	1.37E-05	1.27E-05	1.16E-05	2.25E-05
T06E4.5	15	22	10	7	1.37E-05	1.24E-05	1.16E-05	2.21E-05
T06E4.6	826	1027	1244	598	1.04E-04	1.21E-04	1.17E-04	5.98E-05
T06E4.7	5	12	6	6	2.80E-06	2.67E-06	1.82E-06	2.25E-06
T06E4.8	53	43	11	10	1.04E-04	1.22E-04	1.02E-04	6.05E-05
T06E4.9	54	41	13	18	2.80E-06	2.65E-06	1.82E-06	2.25E-06
T06E6.1	230	201	182	289	9.21E-06	7.06E-06	1.82E-06	2.25E-06
T06E6.10	39	65	39	46	8.62E-06	6.19E-06	1.82E-06	2.32E-06
T06E6.11	4	2	4	1	1.87E-05	1.54E-05	9.62E-06	1.89E-05
T06E6.12	6	5	9	1	5.82E-06	9.18E-06	3.79E-06	5.51E-06
T06E6.13	2	4	3	5	2.80E-06	2.65E-06	1.82E-06	2.25E-06
T06E6.14	7	6	12	5	2.80E-06	2.65E-06	1.82E-06	2.25E-06
T06E6.15	9	4	7	3	2.80E-06	2.65E-06	1.82E-06	2.25E-06
T06E6.2a.1	4369	3381	7324	8351	2.80E-06	2.65E-06	1.82E-06	2.25E-06
T06E6.2a.2	3332	2473	5204	6418	2.80E-06	2.65E-06	1.82E-06	2.25E-06
T06E6.2b	4357	3372	7318	8335	3.36E-04	2.45E-04	3.66E-04	5.15E-04
T06E6.3	2	2	6	1	3.20E-04	2.24E-04	3.25E-04	4.95E-04
T06E6.4	1	3	1	0	3.36E-04	2.46E-04	3.67E-04	5.16E-04
T06E6.5	12	3	12	4	2.80E-06	2.65E-06	1.82E-06	2.25E-06
T06E6.6	0	6	4	4	2.80E-06	2.65E-06	1.82E-06	2.25E-06
T06E6.7	6	3	7	4	2.80E-06	2.65E-06	1.82E-06	2.25E-06
T06E6.8	4	4	19	5	2.80E-06	2.65E-06	1.82E-06	2.25E-06
T06E6.9	4	3	6	1	2.80E-06	2.65E-06	1.82E-06	2.25E-06
T06E8.1	253	358	325	457	2.80E-06	2.65E-06	1.82E-06	2.25E-06
T06E8.2	1	0	0	1	2.80E-06	2.65E-06	1.82E-06	2.25E-06
T06F4.1a	36	60	38	49	2.82E-05	3.76E-05	2.35E-05	4.09E-05
T06F4.1b	19	26	16	17	2.80E-06	2.65E-06	1.82E-06	2.25E-06
T06F4.2a	36	37	36	25	3.11E-06	4.89E-06	2.13E-06	3.40E-06
T06F4.2b	36	38	39	26	3.84E-06	4.95E-06	2.10E-06	2.77E-06
T06F4.3	2	2	2	4	2.80E-06	2.65E-06	1.82E-06	2.25E-06
T06G6.1	5	3	2	2	2.80E-06	2.65E-06	1.82E-06	2.25E-06
T06G6.11	17	29	13	15	2.80E-06	2.65E-06	1.82E-06	2.25E-06
T06G6.12	2	3	10	1	2.80E-06	2.65E-06	1.82E-06	2.25E-06
T06G6.2	3	5	8	3	2.80E-06	3.12E-06	1.82E-06	2.25E-06
T06G6.3a	6	12	10	4	2.80E-06	2.65E-06	2.20E-06	2.25E-06
T06G6.3b	8	16	18	4	2.80E-06	2.65E-06	1.82E-06	2.25E-06
T06G6.4	9	14	8	7	2.80E-06	2.65E-06	1.82E-06	2.25E-06
T06G6.5	0	3	1	0	2.80E-06	2.65E-06	1.82E-06	2.25E-06
T06G6.7	2	8	4	3	2.80E-06	2.65E-06	1.82E-06	2.25E-06
T06G6.8	3	8	3	1	2.80E-06	2.65E-06	1.82E-06	2.25E-06
T06G6.9	542	386	407	554	2.80E-06	2.65E-06	1.82E-06	2.25E-06
T06H11.1a	48	54	24	39	2.80E-06	2.65E-06	1.82E-06	2.25E-06
T06H11.1b	64	74	34	44	8.01E-05	5.39E-05	3.91E-05	6.57E-05
T06H11.2	2	5	2	3	2.91E-06	3.09E-06	1.82E-06	2.25E-06
T06H11.4	45	82	29	69	3.25E-06	3.57E-06	1.82E-06	2.25E-06
T06H11.5	0	3	1	1	2.80E-06	2.65E-06	1.82E-06	2.25E-06
T07A5.1	20	69	40	60	3.72E-06	6.40E-06	1.82E-06	4.59E-06
T07A5.2	344	271	610	555	2.80E-06	2.65E-06	1.82E-06	2.25E-06
T07A5.3	37	40	13	41	2.80E-06	3.91E-06	1.82E-06	2.88E-06

T07A5.4	5	6	4	0	3.13E-05	2.33E-05	3.61E-05	4.05E-05
T07A5.5	102	97	173	42	2.80E-06	2.65E-06	1.82E-06	2.25E-06
T07A5.6a	187	166	191	222	2.80E-06	2.65E-06	1.82E-06	2.25E-06
T07A5.6b	150	145	155	164	1.06E-04	9.50E-05	1.17E-04	3.50E-05
T07A9.1	57	74	55	82	4.30E-05	3.61E-05	2.86E-05	4.10E-05
T07A9.10	292	290	355	495	2.56E-05	2.34E-05	1.73E-05	2.25E-05
T07A9.11.1	7051	7567	27815	7513	1.08E-05	1.33E-05	6.78E-06	1.25E-05
T07A9.11.2	6822	7241	27702	7354	1.73E-05	1.62E-05	1.37E-05	2.36E-05
T07A9.12a	12	31	12	13	1.65E-03	1.67E-03	4.24E-03	1.41E-03
T07A9.12b	9	21	10	8	1.62E-03	1.63E-03	4.29E-03	1.40E-03
T07A9.13a	214	243	396	160	2.83E-06	6.90E-06	1.84E-06	2.45E-06
T07A9.13b	171	223	294	139	2.80E-06	5.29E-06	1.82E-06	2.25E-06
T07A9.14	455	352	554	516	4.41E-05	4.73E-05	5.31E-05	2.64E-05
T07A9.2	78	136	120	126	3.42E-05	4.21E-05	3.83E-05	2.23E-05
T07A9.3	157	275	158	223	5.65E-05	4.13E-05	4.48E-05	5.15E-05
T07A9.5a	208	228	252	348	5.85E-06	9.63E-06	5.85E-06	7.58E-06
T07A9.5b	224	270	274	375	1.16E-05	1.92E-05	7.58E-06	1.32E-05
T07A9.6	2138	2118	2907	4109	1.49E-05	1.54E-05	1.17E-05	2.00E-05
T07A9.7	20	23	36	28	1.34E-05	1.52E-05	1.06E-05	1.79E-05
T07A9.8	208	277	190	263	7.20E-05	6.73E-05	6.37E-05	1.11E-04
T07A9.9a.1	1367	1610	1783	2487	2.80E-06	2.65E-06	1.82E-06	2.25E-06
T07A9.9a.2	1364	1604	1780	2477	2.04E-05	2.57E-05	1.22E-05	2.08E-05
T07A9.9a.3	1327	1539	1688	2343	6.72E-05	7.48E-05	5.71E-05	9.83E-05
T07A9.9b.1	1306	1512	1717	2371	6.76E-05	7.51E-05	5.74E-05	9.86E-05
T07A9.9b.2	1265	1441	1620	2227	6.94E-05	7.60E-05	5.74E-05	9.83E-05
T07A9.9c.1	475	538	472	678	6.88E-05	7.53E-05	5.89E-05	1.00E-04
T07A9.9c.2	412	473	432	615	7.30E-05	7.86E-05	6.09E-05	1.03E-04
T07A9.9c.3	392	447	428	606	7.20E-05	7.70E-05	4.66E-05	8.25E-05
T07A9.9c.4	445	505	441	627	6.43E-05	6.98E-05	4.39E-05	7.72E-05
T07A9.9c.5	383	425	322	511	6.89E-05	7.42E-05	4.90E-05	8.56E-05
T07A9.9c.6	436	471	378	537	9.03E-05	9.68E-05	5.82E-05	1.02E-04
T07A9.9c.7	360	401	318	504	7.84E-05	8.22E-05	4.29E-05	8.40E-05
T07C12.1	1	4	11	4	8.86E-05	9.04E-05	5.00E-05	8.77E-05
T07C12.10	4	4	2	1	6.61E-05	6.96E-05	3.80E-05	7.43E-05
T07C12.11	14	18	11	9	2.80E-06	2.65E-06	1.82E-06	2.25E-06
T07C12.12	162	213	197	357	2.80E-06	2.65E-06	1.82E-06	2.25E-06
T07C12.13	7	3	6	3	2.80E-06	2.65E-06	1.82E-06	2.25E-06
T07C12.14	261	217	345	264	9.21E-06	1.15E-05	7.29E-06	1.63E-05
T07C12.3	68	93	114	99	3.47E-06	2.65E-06	1.95E-06	2.25E-06
T07C12.4	7	14	6	9	4.51E-05	3.54E-05	3.88E-05	3.67E-05
T07C12.5	16	14	28	26	2.80E-06	3.04E-06	2.57E-06	2.77E-06
T07C12.6	15	15	19	6	2.80E-06	2.65E-06	1.82E-06	2.25E-06
T07C12.8	14	30	38	23	2.80E-06	2.65E-06	1.82E-06	2.25E-06
T07C12.9	175	269	374	174	2.80E-06	2.65E-06	1.82E-06	2.25E-06
T07C4.1.1	396	423	641	761	2.80E-06	2.65E-06	1.82E-06	2.25E-06
T07C4.1.2	335	368	542	675	1.83E-05	2.66E-05	2.54E-05	1.46E-05
T07C4.10	219	237	137	376	2.66E-05	2.68E-05	2.80E-05	4.10E-05
T07C4.11	80	106	221	124	2.50E-05	2.60E-05	2.63E-05	4.05E-05
T07C4.12	51	69	161	87	7.73E-06	7.91E-06	3.15E-06	1.07E-05
T07C4.2	7	9	6	10	5.57E-06	6.98E-06	1.00E-05	6.95E-06
T07C4.3a	1229	1019	1997	2437	1.31E-05	1.68E-05	2.70E-05	1.80E-05
T07C4.3b	1350	1115	2226	2768	2.80E-06	2.65E-06	1.82E-06	2.25E-06
T07C4.7.1	776	775	725	658	6.83E-05	5.35E-05	7.22E-05	1.09E-04
T07C4.7.2	675	652	599	576	6.51E-05	5.08E-05	6.99E-05	1.07E-04
T07C4.9a	102	178	225	256	7.06E-05	6.66E-05	4.29E-05	4.81E-05
T07C4.9b.1	61	128	128	195	1.22E-04	1.11E-04	7.05E-05	8.37E-05
T07C4.9b.2	69	141	139	204	5.77E-06	9.50E-06	8.27E-06	1.16E-05
T07C5.1b	30	58	36	32	4.70E-06	9.34E-06	6.43E-06	1.21E-05
T07C5.1c	77	139	94	95	5.15E-06	9.92E-06	6.74E-06	1.22E-05

T07C5.2	3	5	4	5	4.40E-06	8.04E-06	3.44E-06	3.78E-06
T07C5.3	1	2	7	5	5.29E-06	8.99E-06	4.19E-06	5.24E-06
T07C5.4	5	4	5	5	2.80E-06	2.65E-06	1.82E-06	2.25E-06
T07C5.5	3	6	1	2	2.80E-06	2.65E-06	1.82E-06	2.25E-06
T07D1.1a	17	18	11	16	2.80E-06	2.65E-06	1.82E-06	2.25E-06
T07D1.1b	20	21	17	17	2.80E-06	2.65E-06	1.82E-06	2.25E-06
T07D1.1c	13	20	16	16	2.80E-06	2.65E-06	1.82E-06	2.25E-06
T07D1.2.1	82	132	58	112	2.80E-06	2.65E-06	1.82E-06	2.25E-06
T07D1.2.2	77	120	47	99	2.80E-06	2.65E-06	1.82E-06	2.25E-06
T07D1.2.3	66	104	46	97	5.40E-06	8.20E-06	2.48E-06	5.91E-06
T07D1.3	3	3	0	1	5.46E-06	8.04E-06	2.17E-06	5.62E-06
T07D1.4.1	99	133	134	178	5.07E-06	7.56E-06	2.30E-06	5.98E-06
T07D1.4.2	95	130	132	175	2.80E-06	2.65E-06	1.82E-06	2.25E-06
T07D1.5	1	2	7	1	6.05E-06	7.67E-06	5.32E-06	8.73E-06
T07D10.1	6	5	7	3	5.80E-06	7.51E-06	5.25E-06	8.59E-06
T07D10.2	2	5	5	5	2.80E-06	2.65E-06	1.82E-06	2.25E-06
T07D10.3	10	28	12	28	2.80E-06	2.65E-06	1.82E-06	2.25E-06
T07D10.4	15	6	26	3	2.80E-06	2.65E-06	1.82E-06	2.25E-06
T07D10.5	3	5	4	2	2.80E-06	2.65E-06	1.82E-06	2.25E-06
T07D10.t1	0	0	1	0	2.80E-06	2.65E-06	1.82E-06	2.25E-06
T07D3.1	17	25	15	18	2.80E-06	2.65E-06	1.82E-06	2.25E-06
T07D3.2	4	6	1	3	2.80E-06	2.65E-06	1.82E-06	2.25E-06
T07D3.3	3	4	3	4	2.80E-06	2.65E-06	1.82E-06	2.25E-06
T07D3.4	30	55	22	36	2.80E-06	2.65E-06	1.82E-06	2.25E-06
T07D3.5	1	2	3	4	2.80E-06	2.65E-06	1.82E-06	2.25E-06
T07D3.6	2	7	14	5	2.80E-06	4.31E-06	1.82E-06	2.41E-06
T07D3.7a	392	341	424	564	2.80E-06	2.65E-06	1.82E-06	2.25E-06
T07D3.9a.1	111	118	132	117	2.80E-06	2.65E-06	1.82E-06	2.25E-06
T07D3.9a.2	98	107	120	110	1.36E-05	1.11E-05	9.55E-06	1.57E-05
T07D3.9b.1	107	114	124	114	8.48E-06	8.54E-06	6.58E-06	7.20E-06
T07D3.9b.2	100	108	121	108	8.23E-06	8.49E-06	6.56E-06	7.42E-06
T07D4.1	12	20	9	11	7.84E-06	7.88E-06	5.92E-06	6.70E-06
T07D4.2a	45	55	26	54	7.87E-06	8.04E-06	6.21E-06	6.84E-06
T07D4.2b	62	78	53	73	2.80E-06	2.65E-06	1.82E-06	2.25E-06
T07D4.3	706	754	628	1102	6.58E-06	7.62E-06	2.48E-06	6.34E-06
T07D4.4a	839	840	1115	1601	4.37E-06	5.18E-06	2.42E-06	4.14E-06
T07D4.4b.1	705	679	791	1135	1.97E-05	1.98E-05	1.14E-05	2.47E-05
T07D4.4b.2	586	575	710	1010	3.06E-05	2.90E-05	2.65E-05	4.69E-05
T07D4.4c	567	564	685	965	3.47E-05	3.15E-05	2.53E-05	4.48E-05
T07E3.2	2	17	16	2	3.41E-05	3.16E-05	2.69E-05	4.72E-05
T07E3.3	347	309	429	533	3.45E-05	3.24E-05	2.71E-05	4.71E-05
T07E3.4a.1	120	364	112	131	2.80E-06	6.67E-06	4.32E-06	2.25E-06
T07E3.4a.2	102	316	84	118	5.00E-05	4.21E-05	4.02E-05	6.17E-05
T07E3.4b.1	102	316	84	118	8.96E-06	2.57E-05	5.43E-06	7.85E-06
T07E3.4b.2	100	312	84	117	8.74E-06	2.56E-05	4.68E-06	8.12E-06
T07E3.5	192	249	246	315	8.74E-06	2.56E-05	4.68E-06	8.12E-06
T07E3.6a	144	177	92	53	8.26E-06	2.43E-05	4.52E-06	7.76E-06
T07F10.1a	286	408	244	450	1.53E-05	1.87E-05	1.27E-05	2.02E-05
T07F10.1b	259	380	228	422	1.40E-05	1.63E-05	5.83E-06	4.14E-06
T07F10.3	247	329	357	427	9.63E-06	1.30E-05	5.34E-06	1.22E-05
T07F10.4a	136	206	79	80	9.88E-06	1.37E-05	5.67E-06	1.30E-05
T07F10.5	21	25	26	28	2.00E-05	2.51E-05	1.88E-05	2.77E-05
T07F10.6	5	14	14	15	9.63E-06	1.38E-05	3.64E-06	4.54E-06
T07F12.1	20	21	14	25	6.47E-06	7.27E-06	5.23E-06	6.95E-06
T07F12.2	79	116	98	105	2.80E-06	2.65E-06	1.82E-06	2.25E-06
T07F12.3	14	10	10	5	2.80E-06	2.65E-06	1.82E-06	2.63E-06
T07F12.4	11	51	12	20	7.56E-06	1.05E-05	6.09E-06	8.05E-06
T07F8.1	13	12	18	6	2.80E-06	2.65E-06	1.82E-06	2.25E-06
T07F8.2	9	12	10	5	2.80E-06	5.42E-06	1.82E-06	2.25E-06

T07F8.3a	1366	1200	2294	3044	2.80E-06	2.65E-06	1.82E-06	2.25E-06
T07F8.4	485	376	623	717	2.80E-06	2.65E-06	1.82E-06	2.25E-06
T07G12.1	15	39	9	22	5.21E-05	4.33E-05	5.70E-05	9.33E-05
T07G12.11	336	344	404	617	4.68E-05	3.42E-05	3.91E-05	5.55E-05
T07G12.12	230	253	194	352	2.80E-06	5.11E-06	1.82E-06	2.45E-06
T07G12.14	0	1	3	3	2.18E-05	2.11E-05	1.70E-05	3.21E-05
T07G12.2	18	24	7	11	2.11E-05	2.20E-05	1.16E-05	2.60E-05
T07G12.3	31	44	31	14	2.80E-06	2.65E-06	1.82E-06	2.25E-06
T07G12.4	15	13	6	10	2.80E-06	2.65E-06	1.82E-06	2.25E-06
T07G12.5	13	11	12	4	2.80E-06	2.65E-06	1.82E-06	2.25E-06
T07G12.6	321	371	390	600	2.80E-06	2.65E-06	1.82E-06	2.25E-06
T07G12.8	48	51	59	51	2.80E-06	2.65E-06	1.82E-06	2.25E-06
T07H3.1	13	21	16	17	1.93E-05	2.11E-05	1.53E-05	2.90E-05
T07H3.2	4	6	8	5	8.40E-06	8.44E-06	6.72E-06	7.17E-06
T07H3.3a	4	11	3	6	2.80E-06	2.65E-06	1.82E-06	2.25E-06
T07H3.3b	5	10	3	6	2.80E-06	2.65E-06	1.82E-06	2.25E-06
T07H3.4	6	4	1	4	2.80E-06	2.65E-06	1.82E-06	2.25E-06
T07H3.5	2	5	0	1	2.80E-06	2.65E-06	1.82E-06	2.25E-06
T07H3.6	8	12	13	4	2.80E-06	2.65E-06	1.82E-06	2.25E-06
T07H3.7	1	0	0	1	2.80E-06	2.65E-06	1.82E-06	2.25E-06
T07H6.1a	14	37	21	20	2.80E-06	2.65E-06	1.82E-06	2.25E-06
T07H6.1b.1	5	15	4	5	2.80E-06	2.65E-06	1.82E-06	2.25E-06
T07H6.1b.2	4	15	3	5	2.80E-06	2.65E-06	1.82E-06	2.25E-06
T07H6.2	34	29	58	80	2.80E-06	2.65E-06	1.82E-06	2.25E-06
T07H6.3a	3568	3100	2701	1764	2.80E-06	2.65E-06	1.82E-06	2.25E-06
T07H6.3b.1	3246	2777	2375	1614	2.80E-06	2.65E-06	2.77E-06	4.70E-06
T07H6.3b.2	3184	2709	2313	1597	3.37E-04	2.77E-04	1.66E-04	1.34E-04
T07H6.3b.3	3184	2709	2313	1597	4.01E-04	3.24E-04	1.91E-04	1.60E-04
T07H6.4	17	14	25	13	3.82E-04	3.07E-04	1.81E-04	1.54E-04
T07H6.5	14	17	14	27	3.94E-04	3.16E-04	1.86E-04	1.59E-04
T07H6.t1	0	0	1	0	2.80E-06	2.65E-06	1.82E-06	2.25E-06
T07H6.t2	0	0	1	0	2.80E-06	2.65E-06	1.82E-06	2.25E-06
T07H8.1	4	6	5	2	2.80E-06	2.65E-06	1.82E-06	2.25E-06
T07H8.3	3	3	4	1	2.80E-06	2.65E-06	1.82E-06	2.25E-06
T07H8.4a	74	107	106	77	2.80E-06	2.65E-06	1.82E-06	2.25E-06
T07H8.4b	71	108	98	79	2.80E-06	2.65E-06	1.82E-06	2.25E-06
T07H8.4c	58	81	86	59	2.80E-06	2.65E-06	1.82E-06	2.25E-06
T07H8.4e	74	104	103	76	2.80E-06	2.65E-06	1.82E-06	2.25E-06
T07H8.4f.1	74	106	105	76	2.80E-06	2.65E-06	1.82E-06	2.25E-06
T07H8.4f.2	74	107	104	76	2.80E-06	2.65E-06	1.82E-06	2.25E-06
T07H8.4g	49	88	48	63	2.80E-06	2.65E-06	1.82E-06	2.25E-06
T07H8.4h	75	106	111	80	2.80E-06	2.65E-06	1.82E-06	2.25E-06
T07H8.5	11	8	20	6	2.80E-06	2.65E-06	1.82E-06	2.25E-06
T07H8.6	36	42	28	26	2.80E-06	2.65E-06	1.82E-06	2.25E-06
T07H8.7	5	6	3	2	2.80E-06	2.65E-06	1.82E-06	2.25E-06
T08A11.1	774	720	871	1288	2.80E-06	2.65E-06	1.82E-06	2.25E-06
T08A11.2	1433	1455	2106	2781	2.80E-06	2.65E-06	1.82E-06	2.25E-06
T08A9.1	214	302	193	361	1.41E-05	1.24E-05	1.04E-05	1.89E-05
T08A9.10	1	3	3	0	3.77E-05	3.62E-05	3.61E-05	5.88E-05
T08A9.11	92	157	145	90	5.54E-06	7.38E-06	3.24E-06	7.51E-06
T08A9.13	3	2	11	3	2.80E-06	2.65E-06	1.82E-06	2.25E-06
T08A9.2	44	83	54	47	1.62E-05	2.62E-05	1.67E-05	1.28E-05
T08A9.3	12	21	28	14	2.80E-06	2.65E-06	2.20E-06	2.25E-06
T08A9.4	8	7	6	4	9.27E-06	1.65E-05	7.42E-06	7.96E-06
T08A9.5	5	7	2	2	2.80E-06	2.65E-06	2.06E-06	2.25E-06
T08A9.6	16	6	10	10	2.80E-06	2.65E-06	1.82E-06	2.25E-06
T08A9.7.1	1192	1386	4555	1001	2.80E-06	2.65E-06	1.82E-06	2.25E-06
T08A9.7.2	953	1136	3446	891	5.21E-06	2.65E-06	2.11E-06	2.61E-06
T08A9.8	356	180	837	181	3.94E-04	4.33E-04	9.79E-04	2.66E-04

T08A9.9.1	2990	3295	11951	2364	3.08E-04	3.47E-04	7.26E-04	2.32E-04
T08A9.9.2	2959	3260	11918	2348	1.28E-04	6.10E-05	1.96E-04	5.22E-05
T08A9.9.3	2533	2771	9654	2094	8.29E-04	8.63E-04	2.16E-03	5.26E-04
T08A9.t1	1	0	3	0	8.50E-04	8.84E-04	2.23E-03	5.42E-04
T08B1.1	291	410	382	568	7.47E-04	7.72E-04	1.85E-03	4.96E-04
T08B1.2a	100	151	45	103	2.80E-06	2.65E-06	3.04E-06	2.25E-06
T08B1.2b	98	126	42	92	1.74E-05	2.32E-05	1.49E-05	2.73E-05
T08B1.3	46	35	40	32	1.07E-05	1.52E-05	3.13E-06	8.84E-06
T08B1.4	11	37	20	14	1.18E-05	1.43E-05	3.30E-06	8.91E-06
T08B1.5	9	7	4	6	3.75E-06	2.70E-06	2.11E-06	2.25E-06
T08B1.6	24	49	26	29	2.80E-06	6.69E-06	2.50E-06	2.25E-06
T08B2.10.1	7745	8322	13407	4278	2.80E-06	2.65E-06	1.82E-06	2.25E-06
T08B2.10.2	6165	6785	10383	3732	2.80E-06	2.65E-06	1.82E-06	2.25E-06
T08B2.11	77	93	67	55	1.87E-03	1.89E-03	2.10E-03	8.28E-04
T08B2.12	320	607	291	126	1.46E-03	1.52E-03	1.60E-03	7.10E-04
T08B2.2	6	7	8	6	6.89E-06	7.86E-06	3.90E-06	3.96E-06
T08B2.3	10	3	17	20	7.81E-05	1.40E-04	4.62E-05	2.47E-05
T08B2.4	37	29	40	38	2.80E-06	2.65E-06	1.82E-06	2.25E-06
T08B2.5a	505	484	601	906	2.80E-06	2.65E-06	2.30E-06	3.33E-06
T08B2.5b	501	470	594	897	1.15E-05	8.52E-06	8.09E-06	9.49E-06
T08B2.5c	537	504	632	914	1.99E-05	1.80E-05	1.54E-05	2.87E-05
T08B2.5d	67	58	84	97	2.02E-05	1.79E-05	1.56E-05	2.90E-05
T08B2.5e	71	74	97	97	2.02E-05	1.80E-05	1.55E-05	2.77E-05
T08B2.5f	453	434	532	842	1.52E-05	1.24E-05	1.24E-05	1.77E-05
T08B2.5g	457	445	551	851	1.33E-05	1.31E-05	1.18E-05	1.46E-05
T08B2.7b.1	1577	2246	1442	2219	2.02E-05	1.83E-05	1.54E-05	3.01E-05
T08B2.7b.2	1552	2215	1388	2183	1.88E-05	1.73E-05	1.47E-05	2.81E-05
T08B2.7c	1688	2355	1410	2265	7.55E-05	1.02E-04	4.49E-05	8.53E-05
T08B2.8	197	200	205	149	7.45E-05	1.00E-04	4.34E-05	8.42E-05
T08B2.9a	929	982	998	1297	7.17E-05	9.45E-05	3.90E-05	7.73E-05
T08B2.9b.1	916	969	998	1299	3.93E-05	3.76E-05	2.66E-05	2.38E-05
T08B2.9b.2	929	969	1027	1304	5.71E-05	5.71E-05	3.99E-05	6.41E-05
T08B2.9b.3	907	954	979	1277	6.87E-05	6.86E-05	4.87E-05	7.82E-05
T08B2.9b.4	881	931	965	1258	6.27E-05	6.18E-05	4.51E-05	7.07E-05
T08B2.t1	0	0	1	0	6.81E-05	6.77E-05	4.78E-05	7.70E-05
T08B6.1	3	5	9	3	5.91E-05	5.90E-05	4.21E-05	6.78E-05
T08B6.2	16	5	3	10	2.80E-06	2.65E-06	1.82E-06	2.25E-06
T08B6.3	4	2	4	2	2.80E-06	2.65E-06	1.82E-06	2.25E-06
T08B6.4	112	175	49	59	2.80E-06	2.65E-06	1.82E-06	2.25E-06
T08B6.5	5	8	4	4	2.80E-06	2.65E-06	1.82E-06	2.25E-06
T08B6.6	9	5	13	10	6.22E-06	9.18E-06	1.82E-06	2.63E-06
T08B6.7	4	5	8	4	2.80E-06	2.65E-06	1.82E-06	2.25E-06
T08B6.9	22	68	17	21	2.80E-06	2.65E-06	1.82E-06	2.25E-06
T08D10.1	93	72	123	93	2.80E-06	2.65E-06	1.82E-06	2.25E-06
T08D10.2	73	92	171	202	2.80E-06	4.50E-06	1.82E-06	2.25E-06
T08D10.3	9	10	11	9	6.30E-06	4.60E-06	5.41E-06	5.06E-06
T08D10.4	5	2	4	8	3.42E-06	4.05E-06	5.19E-06	7.56E-06
T08D2.1	207	221	328	185	2.80E-06	2.65E-06	1.82E-06	2.25E-06
T08D2.2	30	31	55	57	2.80E-06	2.65E-06	1.82E-06	2.25E-06
T08D2.3	376	450	351	671	4.60E-05	4.64E-05	4.74E-05	3.30E-05
T08D2.4	10	6	18	13	3.53E-06	3.44E-06	4.21E-06	5.38E-06
T08D2.5	82	121	87	133	1.38E-05	1.56E-05	8.38E-06	1.98E-05
T08D2.6	6	15	11	20	3.86E-06	2.65E-06	4.52E-06	4.03E-06
T08D2.7	24	28	28	25	9.38E-06	1.31E-05	6.49E-06	1.22E-05
T08D2.8	23	19	28	44	3.19E-06	7.56E-06	3.83E-06	8.57E-06
T08D2.9	31	30	58	64	2.80E-06	2.65E-06	1.82E-06	2.25E-06
T08E11.1	204	12	190	12	2.80E-06	2.65E-06	1.82E-06	3.40E-06
T08E11.2	32	33	24	23	5.49E-06	5.03E-06	6.69E-06	9.09E-06
T08E11.3	15	24	14	21	1.13E-05	2.65E-06	6.81E-06	2.25E-06

T08E11.4	41	64	49	58	3.70E-06	3.62E-06	1.82E-06	2.25E-06
T08E11.5	8	5	21	4	2.80E-06	2.70E-06	1.82E-06	2.25E-06
T08E11.6	6	4	4	4	2.80E-06	2.94E-06	1.82E-06	2.27E-06
T08E11.7	7	12	21	6	2.80E-06	2.65E-06	1.82E-06	2.25E-06
T08E11.8	4	7	8	5	2.80E-06	2.65E-06	1.82E-06	2.25E-06
T08G11.1a	2945	3474	2867	5361	2.80E-06	2.65E-06	1.82E-06	2.25E-06
T08G11.2	15	37	12	12	2.80E-06	2.65E-06	1.82E-06	2.25E-06
T08G11.3	7	12	21	11	3.42E-05	3.81E-05	2.17E-05	5.00E-05
T08G11.4.1	346	385	397	585	2.80E-06	4.60E-06	1.82E-06	2.25E-06
T08G11.4.2	333	374	367	551	2.80E-06	2.65E-06	1.82E-06	2.25E-06
T08G11.5	26	53	31	21	2.06E-05	2.17E-05	1.54E-05	2.80E-05
T08G2.2	5	11	10	8	2.17E-05	2.30E-05	1.55E-05	2.88E-05
T08G2.3.1	423	604	412	467	2.80E-06	3.47E-06	1.82E-06	2.25E-06
T08G2.3.2	384	556	350	426	2.80E-06	2.65E-06	1.82E-06	2.25E-06
T08G3.1	3	2	4	2	3.45E-05	4.66E-05	2.19E-05	3.06E-05
T08G3.10	7	2	10	6	3.41E-05	4.67E-05	2.02E-05	3.04E-05
T08G3.11	2	5	2	1	2.80E-06	2.65E-06	1.82E-06	2.25E-06
T08G3.12	2	1	7	3	2.80E-06	2.65E-06	1.82E-06	2.25E-06
T08G3.13	2	4	1	0	2.80E-06	2.65E-06	1.82E-06	2.25E-06
T08G3.2	4	1	3	8	2.80E-06	2.65E-06	1.82E-06	2.25E-06
T08G3.3	4	5	6	3	2.80E-06	2.65E-06	1.82E-06	2.25E-06
T08G3.4	15	28	4	6	2.80E-06	2.65E-06	1.82E-06	2.25E-06
T08G3.5	4	3	5	1	2.80E-06	2.65E-06	1.82E-06	2.25E-06
T08G3.6	1	5	4	1	2.80E-06	2.65E-06	1.82E-06	2.25E-06
T08G3.7	24	50	17	23	2.80E-06	2.65E-06	1.82E-06	2.25E-06
T08G3.8	12	8	9	6	2.80E-06	2.65E-06	1.82E-06	2.25E-06
T08G3.9	9	13	9	4	2.80E-06	2.65E-06	1.82E-06	2.25E-06
T08G5.1	13	6	12	6	2.80E-06	2.65E-06	1.82E-06	2.25E-06
T08G5.10	102	132	367	109	2.80E-06	2.65E-06	1.82E-06	2.25E-06
T08G5.11	1	0	5	1	2.80E-06	2.65E-06	1.82E-06	2.25E-06
T08G5.12	4	3	4	0	3.86E-05	4.72E-05	9.04E-05	3.31E-05
T08G5.2	11	7	18	8	2.80E-06	2.65E-06	2.20E-06	2.25E-06
T08G5.3	15	25	11	6	2.80E-06	2.65E-06	1.82E-06	2.25E-06
T08G5.4	39	44	58	41	2.80E-06	2.65E-06	1.82E-06	2.25E-06
T08G5.5a	724	661	1287	1611	2.86E-06	4.47E-06	1.82E-06	2.25E-06
T08G5.5b	778	709	1377	1679	2.80E-06	2.65E-06	1.82E-06	2.25E-06
T08G5.7	9	10	8	10	2.90E-05	2.50E-05	3.36E-05	5.19E-05
T08G5.8	6	3	10	3	2.85E-05	2.45E-05	3.29E-05	4.94E-05
T08G5.9	5	1	4	1	2.80E-06	2.65E-06	1.82E-06	2.25E-06
T08H10.1	771	987	967	1087	2.80E-06	2.65E-06	1.82E-06	2.25E-06
T08H10.2	2	3	2	3	2.80E-06	2.65E-06	1.82E-06	2.25E-06
T08H10.3	21	15	10	6	8.21E-05	9.93E-05	6.70E-05	9.30E-05
T08H10.4	9	13	10	5	2.80E-06	2.65E-06	1.82E-06	2.25E-06
T08H10.5	2	1	2	3	4.14E-06	2.80E-06	1.82E-06	2.25E-06
T08H4.1	94	107	68	155	2.80E-06	2.65E-06	1.82E-06	2.25E-06
T08H4.2	9	9	6	24	2.80E-06	2.65E-06	1.82E-06	2.25E-06
T08H4.3	11	19	16	16	3.61E-06	3.89E-06	1.82E-06	4.79E-06
T09A12.1	18	32	16	11	2.80E-06	2.65E-06	1.82E-06	3.33E-06
T09A12.2a	13	24	15	8	2.80E-06	2.65E-06	1.82E-06	2.25E-06
T09A12.2b	12	27	13	13	2.80E-06	3.31E-06	1.82E-06	2.25E-06
T09A12.3	35	45	24	30	2.80E-06	3.68E-06	1.82E-06	2.25E-06
T09A12.4a.1	357	454	549	643	2.80E-06	5.03E-06	1.82E-06	2.25E-06
T09A12.4a.2	453	547	641	744	2.80E-06	2.65E-06	1.82E-06	2.25E-06
T09A12.4a.3	414	509	607	718	1.76E-05	2.11E-05	1.76E-05	2.54E-05
T09A12.4b	329	406	461	547	2.23E-05	2.54E-05	2.05E-05	2.94E-05
T09A12.4c	449	538	639	739	2.09E-05	2.43E-05	1.99E-05	2.91E-05
T09A12.4d.1	449	538	639	739	2.13E-05	2.48E-05	1.94E-05	2.85E-05
T09A12.4d.2	410	500	605	713	2.26E-05	2.56E-05	2.09E-05	2.98E-05
T09A5.10.1	602	739	975	1146	1.91E-05	2.16E-05	1.77E-05	2.52E-05

T09A5.10.2	560	691	935	1112	2.15E-05	2.48E-05	2.07E-05	3.01E-05
T09A5.11.1	1471	1681	1805	2392	2.53E-05	2.93E-05	2.66E-05	3.86E-05
T09A5.11.2	1423	1634	1699	2318	2.54E-05	2.96E-05	2.76E-05	4.05E-05
T09A5.11.3	1255	1452	1462	2084	1.04E-04	1.12E-04	8.28E-05	1.35E-04
T09A5.12.1	410	528	572	739	9.84E-05	1.07E-04	7.64E-05	1.29E-04
T09A5.12.2	330	417	441	583	1.05E-04	1.15E-04	7.96E-05	1.40E-04
T09A5.14	1	4	1	0	1.67E-05	2.04E-05	1.52E-05	2.42E-05
T09A5.15	96	110	194	91	1.73E-05	2.07E-05	1.51E-05	2.46E-05
T09A5.1a	46	84	23	35	2.80E-06	2.65E-06	1.82E-06	2.25E-06
T09A5.1b	30	44	16	28	2.87E-05	3.10E-05	3.77E-05	2.18E-05
T09A5.2a	113	155	89	150	6.41E-06	1.10E-05	2.08E-06	3.91E-06
T09A5.2b	21	25	19	29	5.18E-06	7.17E-06	1.82E-06	3.87E-06
T09A5.2c	17	22	11	20	6.16E-06	7.96E-06	3.15E-06	6.57E-06
T09A5.3	16	13	14	10	4.84E-06	5.45E-06	2.84E-06	5.38E-06
T09A5.4	20	30	18	17	3.42E-06	4.18E-06	1.82E-06	3.24E-06
T09A5.5.1	162	189	249	167	2.80E-06	2.65E-06	1.82E-06	2.25E-06
T09A5.5.2	156	185	180	141	2.80E-06	3.54E-06	1.82E-06	2.25E-06
T09A5.6	98	119	81	100	3.22E-05	3.54E-05	3.22E-05	2.66E-05
T09A5.7.1	233	252	273	143	2.94E-05	3.29E-05	2.20E-05	2.13E-05
T09A5.7.2	207	224	184	120	2.10E-05	2.41E-05	1.13E-05	1.72E-05
T09A5.8	448	505	659	745	5.35E-05	5.46E-05	4.08E-05	2.64E-05
T09A5.9.1	356	356	407	506	4.77E-05	4.88E-05	2.76E-05	2.22E-05
T09A5.9.2	378	370	435	533	3.81E-05	4.05E-05	3.64E-05	5.09E-05
T09B4.1	961	963	1927	2154	3.82E-05	3.61E-05	2.84E-05	4.36E-05
T09B4.10.1	227	346	235	336	4.24E-05	3.92E-05	3.17E-05	4.80E-05
T09B4.10.2	170	269	201	275	4.76E-05	4.50E-05	6.21E-05	8.56E-05
T09B4.2	214	272	240	348	2.20E-05	3.17E-05	1.48E-05	2.62E-05
T09B4.3	16	12	12	4	2.35E-05	3.51E-05	1.81E-05	3.05E-05
T09B4.4	28	45	11	15	2.37E-05	2.85E-05	1.73E-05	3.10E-05
T09B4.5b	21	27	23	62	4.73E-06	3.36E-06	2.31E-06	2.25E-06
T09B4.6	9	18	6	5	5.91E-06	8.94E-06	1.82E-06	2.54E-06
T09B4.7	17	34	19	11	1.01E-05	1.22E-05	7.16E-06	2.38E-05
T09B4.8	457	543	381	430	2.80E-06	3.28E-06	1.82E-06	2.25E-06
T09B4.9	479	495	377	586	2.80E-06	3.49E-06	1.82E-06	2.25E-06
T09B4.t1	0	0	1	0	3.55E-05	3.98E-05	1.93E-05	2.68E-05
T09B4.t2	0	0	1	0	3.49E-05	3.41E-05	1.79E-05	3.43E-05
T09B9.1	116	108	184	193	2.80E-06	2.65E-06	1.82E-06	2.25E-06
T09B9.2	20	33	19	9	2.80E-06	2.65E-06	1.82E-06	2.25E-06
T09B9.3	6	4	18	6	8.43E-06	7.41E-06	8.69E-06	1.13E-05
T09B9.4.1	262	238	348	449	2.80E-06	2.65E-06	1.82E-06	2.25E-06
T09B9.4.2	181	175	224	288	2.80E-06	2.65E-06	1.82E-06	2.25E-06
T09B9.5	0	7	7	1	1.43E-05	1.23E-05	1.24E-05	1.97E-05
T09D3.1	4	7	6	2	1.27E-05	1.16E-05	1.02E-05	1.62E-05
T09D3.2	3	3	0	2	2.80E-06	2.65E-06	1.82E-06	2.25E-06
T09D3.3	13	8	64	8	2.80E-06	2.65E-06	1.82E-06	2.25E-06
T09D3.4	5	8	2	7	2.80E-06	2.65E-06	1.82E-06	2.25E-06
T09D3.5	2	6	8	5	2.80E-06	2.65E-06	2.62E-06	2.25E-06
T09D3.6	6	8	11	9	2.80E-06	2.65E-06	1.82E-06	2.25E-06
T09D3.7	1	3	4	4	2.80E-06	2.65E-06	1.82E-06	2.25E-06
T09D3.8	5	3	3	0	2.80E-06	2.65E-06	1.82E-06	2.25E-06
T09E11.1	3	3	5	5	2.80E-06	2.65E-06	1.82E-06	2.25E-06
T09E11.10	1	4	1	3	2.80E-06	2.65E-06	1.82E-06	2.25E-06
T09E11.11	6	12	16	11	2.80E-06	2.65E-06	1.82E-06	2.25E-06
T09E11.2	3	5	0	1	2.80E-06	2.65E-06	1.82E-06	2.25E-06
T09E11.3	1	3	5	2	2.80E-06	2.65E-06	1.82E-06	2.25E-06
T09E11.4	5	5	5	2	2.80E-06	2.65E-06	1.82E-06	2.25E-06
T09E11.5	3	11	8	3	2.80E-06	2.65E-06	1.82E-06	2.25E-06
T09E11.6	2	4	5	1	2.80E-06	2.65E-06	1.82E-06	2.25E-06
T09E11.7	3	10	4	5	2.80E-06	2.65E-06	1.82E-06	2.25E-06

T09E11.8	3	5	4	1	2.80E-06	2.65E-06	1.82E-06	2.25E-06
T09E11.9	0	5	3	5	2.80E-06	2.65E-06	1.82E-06	2.25E-06
T09E8.1a	761	902	1280	1653	2.80E-06	2.65E-06	1.82E-06	2.25E-06
T09E8.1b	740	873	1252	1629	2.80E-06	2.65E-06	1.82E-06	2.25E-06
T09E8.1c	901	1084	1508	1978	3.39E-05	3.80E-05	3.71E-05	5.92E-05
T09E8.1d	558	673	837	1167	3.72E-05	4.14E-05	4.10E-05	6.58E-05
T09E8.1e	579	677	884	1225	3.55E-05	4.03E-05	3.86E-05	6.26E-05
T09E8.1f	741	874	1261	1633	3.41E-05	3.89E-05	3.33E-05	5.73E-05
T09E8.2	626	694	926	1346	4.26E-05	4.70E-05	4.23E-05	7.23E-05
T09E8.3	444	439	359	387	3.78E-05	4.21E-05	4.19E-05	6.70E-05
T09E8.4	8	6	9	7	2.27E-05	2.38E-05	2.19E-05	3.93E-05
T09E8.5	1	6	13	2	8.23E-05	7.69E-05	4.33E-05	5.76E-05
T09F3.1	71	117	73	66	2.80E-06	2.65E-06	1.82E-06	2.25E-06
T09F3.3.1	2394	2222	4008	3689	2.80E-06	2.65E-06	1.82E-06	2.25E-06
T09F3.3.2	2239	2050	3681	3502	5.54E-06	8.65E-06	3.72E-06	4.14E-06
T09F3.4	48	42	30	37	2.40E-04	2.10E-04	2.61E-04	2.97E-04
T09F3.5	113	137	199	230	2.26E-04	1.96E-04	2.42E-04	2.84E-04
T09F5.1	20	47	16	11	8.01E-06	6.61E-06	3.26E-06	4.95E-06
T09F5.10	12	20	18	3	7.50E-06	8.60E-06	8.60E-06	1.23E-05
T09F5.11	20	30	20	13	2.80E-06	5.08E-06	1.82E-06	2.25E-06
T09F5.12	15	48	12	9	2.80E-06	2.65E-06	1.82E-06	2.25E-06
T09F5.13	4	7	5	8	2.80E-06	2.65E-06	1.82E-06	2.25E-06
T09F5.14	3	2	4	2	2.80E-06	3.31E-06	1.82E-06	2.25E-06
T09F5.15	11	5	12	3	2.80E-06	2.65E-06	1.82E-06	2.25E-06
T09F5.16	4	5	11	3	2.80E-06	2.65E-06	1.82E-06	2.25E-06
T09F5.2	1	1	3	1	2.80E-06	2.65E-06	1.82E-06	2.25E-06
T09F5.3	4	5	22	3	2.80E-06	2.65E-06	1.82E-06	2.25E-06
T09F5.4	11	16	18	4	2.80E-06	2.65E-06	1.82E-06	2.25E-06
T09F5.5	2	3	5	3	2.80E-06	2.65E-06	1.82E-06	2.25E-06
T09F5.6	4	8	4	3	2.80E-06	2.65E-06	1.82E-06	2.25E-06
T09F5.8	3	4	4	2	2.80E-06	2.65E-06	1.82E-06	2.25E-06
T09F5.9	74	22	142	23	2.80E-06	2.65E-06	1.82E-06	2.25E-06
T09H2.1	18	25	30	11	2.80E-06	2.65E-06	1.82E-06	2.25E-06
T10A3.1a	55	57	37	44	1.72E-05	4.81E-06	2.14E-05	4.27E-06
T10A3.1b	68	65	41	51	2.80E-06	2.65E-06	1.82E-06	2.25E-06
T10B10.1	756	625	454	157	2.80E-06	2.65E-06	1.82E-06	2.25E-06
T10B10.2	424	550	708	593	2.80E-06	2.65E-06	1.82E-06	2.25E-06
T10B10.3.1	535	270	785	422	6.21E-05	4.85E-05	2.43E-05	1.04E-05
T10B10.3.2	420	203	575	325	3.35E-05	4.10E-05	3.64E-05	3.76E-05
T10B10.4a	157	173	112	146	2.10E-05	1.00E-05	2.01E-05	1.33E-05
T10B10.5	8	12	10	10	2.10E-05	9.60E-06	1.87E-05	1.31E-05
T10B10.6	13	39	8	10	1.30E-05	1.35E-05	6.01E-06	9.69E-06
T10B10.7	9	7	17	6	2.80E-06	2.65E-06	1.82E-06	2.25E-06
T10B10.8	52	70	44	44	2.80E-06	6.40E-06	1.82E-06	2.25E-06
T10B11.1	150	183	234	65	2.80E-06	2.65E-06	1.82E-06	2.25E-06
T10B11.2	409	570	451	757	6.92E-06	8.78E-06	3.81E-06	4.70E-06
T10B11.3	341	367	501	546	2.86E-05	3.29E-05	2.90E-05	9.94E-06
T10B11.4	7	7	3	3	2.60E-05	3.42E-05	1.86E-05	3.86E-05
T10B11.5	24	36	13	18	3.18E-05	3.23E-05	3.04E-05	4.08E-05
T10B11.6	209	262	196	280	2.80E-06	2.65E-06	1.82E-06	2.25E-06
T10B11.7a	154	151	254	360	3.19E-06	4.52E-06	1.82E-06	2.25E-06
T10B11.7b	138	139	231	334	2.08E-05	2.47E-05	1.27E-05	2.24E-05
T10B11.8	240	256	300	436	9.55E-06	8.83E-06	1.02E-05	1.79E-05
T10B5.10	18	29	32	15	9.80E-06	9.31E-06	1.07E-05	1.90E-05
T10B5.2	13	23	7	4	1.54E-05	1.55E-05	1.25E-05	2.24E-05
T10B5.3	401	395	504	618	2.80E-06	2.96E-06	2.26E-06	2.25E-06
T10B5.4	16	17	19	15	2.80E-06	2.65E-06	1.82E-06	2.25E-06
T10B5.5a	2686	1923	1881	2833	3.76E-05	3.50E-05	3.07E-05	4.65E-05
T10B5.5b.1	1981	1456	1321	1910	2.80E-06	2.65E-06	1.86E-06	2.25E-06

T10B5.5b.2	1893	1400	1260	1871	1.53E-04	1.04E-04	6.97E-05	1.30E-04
T10B5.6	220	255	522	413	1.27E-04	8.84E-05	5.53E-05	9.86E-05
T10B5.7	194	270	309	238	1.54E-04	1.08E-04	6.68E-05	1.23E-04
T10B5.8	16	18	26	12	2.12E-05	2.32E-05	3.27E-05	3.19E-05
T10B9.1	8	23	7	13	2.27E-05	2.99E-05	2.36E-05	2.24E-05
T10B9.10	6	9	7	9	2.80E-06	2.65E-06	1.82E-06	2.25E-06
T10B9.2	19	54	19	68	2.80E-06	2.65E-06	1.82E-06	2.25E-06
T10B9.3	9	14	12	12	2.80E-06	2.65E-06	1.82E-06	2.25E-06
T10B9.4	6	9	12	6	2.80E-06	3.65E-06	1.82E-06	3.91E-06
T10B9.5	10	13	7	8	2.80E-06	2.65E-06	1.82E-06	2.25E-06
T10B9.6	5	14	12	7	2.80E-06	2.65E-06	1.82E-06	2.25E-06
T10B9.7	30	91	38	45	2.80E-06	2.65E-06	1.82E-06	2.25E-06
T10B9.8	10	9	8	8	2.80E-06	2.65E-06	1.82E-06	2.25E-06
T10B9.9	3	4	3	5	2.80E-06	6.22E-06	1.82E-06	2.61E-06
T10C6.1	1	10	2	2	2.80E-06	2.65E-06	1.82E-06	2.25E-06
T10C6.11	22	53	40	11	2.80E-06	2.65E-06	1.82E-06	2.25E-06
T10C6.12	36	51	59	8	2.80E-06	2.65E-06	1.82E-06	2.25E-06
T10C6.13	21	27	26	14	5.15E-06	1.17E-05	6.09E-06	2.25E-06
T10C6.14	5	16	9	3	1.01E-05	1.36E-05	1.08E-05	2.25E-06
T10C6.2	3	4	11	4	5.71E-06	6.96E-06	4.61E-06	3.06E-06
T10C6.3	1	2	0	2	2.80E-06	5.42E-06	2.10E-06	2.25E-06
T10C6.4	7	5	9	5	2.80E-06	2.65E-06	1.82E-06	2.25E-06
T10C6.5	372	421	497	477	2.80E-06	2.65E-06	1.82E-06	2.25E-06
T10C6.6a.1	333	320	475	594	2.80E-06	2.65E-06	1.82E-06	2.25E-06
T10C6.6b.1	249	250	347	469	5.28E-05	5.64E-05	4.58E-05	5.43E-05
T10C6.6b.2	251	253	352	471	2.32E-05	2.11E-05	2.16E-05	3.33E-05
T10C6.6b.3	267	264	372	486	2.11E-05	2.00E-05	1.92E-05	3.20E-05
T10C6.6b.4	254	255	351	472	2.04E-05	1.94E-05	1.86E-05	3.07E-05
T10C6.6b.5	259	263	358	481	2.20E-05	2.05E-05	1.99E-05	3.21E-05
T10C6.7	106	100	140	221	2.07E-05	1.97E-05	1.87E-05	3.10E-05
T10C6.8	128	178	224	305	2.10E-05	2.01E-05	1.89E-05	3.13E-05
T10C6.9	10	12	10	13	1.07E-05	9.52E-06	9.20E-06	1.79E-05
T10D4.1	4	5	4	6	1.24E-05	1.63E-05	1.42E-05	2.38E-05
T10D4.10	5	5	3	1	2.80E-06	2.65E-06	1.82E-06	2.25E-06
T10D4.11	1	2	2	0	2.80E-06	2.65E-06	1.82E-06	2.25E-06
T10D4.12	6	3	0	0	2.80E-06	2.65E-06	1.82E-06	2.25E-06
T10D4.13	3	37	7	19	2.80E-06	2.65E-06	1.82E-06	2.25E-06
T10D4.14	1	2	2	1	2.80E-06	2.65E-06	1.82E-06	2.25E-06
T10D4.2	6	4	5	4	2.80E-06	1.21E-05	1.82E-06	5.29E-06
T10D4.3	20	8	12	23	2.80E-06	2.65E-06	1.82E-06	2.25E-06
T10D4.4	2	1	3	0	2.80E-06	2.65E-06	1.82E-06	2.25E-06
T10D4.5	4	1	9	5	2.80E-06	2.65E-06	1.82E-06	2.25E-06
T10D4.6	262	105	426	154	2.80E-06	2.65E-06	1.82E-06	2.25E-06
T10D4.7	2	0	0	1	2.80E-06	2.65E-06	1.82E-06	2.25E-06
T10D4.8	2	6	13	6	1.18E-05	4.50E-06	1.25E-05	5.60E-06
T10D4.9	3	3	5	3	2.80E-06	2.65E-06	1.82E-06	2.25E-06
T10E10.1	3279	2755	2518	1512	2.80E-06	2.65E-06	1.82E-06	2.25E-06
T10E10.2.1	4080	3406	3092	1725	2.80E-06	2.65E-06	1.82E-06	2.25E-06
T10E10.2.2	3499	2979	2605	1633	3.62E-04	2.87E-04	1.81E-04	1.34E-04
T10E10.3	5	4	9	6	4.29E-04	3.39E-04	2.12E-04	1.46E-04
T10E10.4	21	36	6	13	3.80E-04	3.05E-04	1.84E-04	1.42E-04
T10E10.5	2757	2329	1955	1233	2.80E-06	2.65E-06	1.82E-06	2.25E-06
T10E10.6	2341	1847	1807	1166	2.80E-06	2.65E-06	1.82E-06	2.25E-06
T10E10.7	124	112	34	38	3.15E-04	2.51E-04	1.45E-04	1.13E-04
T10E9.1	197	232	272	278	2.90E-04	2.16E-04	1.46E-04	1.16E-04
T10E9.2	371	385	459	681	1.50E-05	1.28E-05	2.68E-06	3.71E-06
T10E9.3	171	207	88	87	1.89E-05	2.10E-05	1.69E-05	2.14E-05
T10E9.5	3	6	3	2	1.80E-05	1.77E-05	1.45E-05	2.66E-05
T10E9.6	8	16	8	3	1.07E-05	1.22E-05	3.57E-06	4.36E-06

T10E9.7a	694	902	1130	775	2.80E-06	2.65E-06	1.82E-06	2.25E-06
T10E9.7b	587	821	845	671	2.80E-06	3.94E-06	1.82E-06	2.25E-06
T10E9.8	33	29	18	16	5.54E-05	6.81E-05	5.87E-05	4.97E-05
T10E9.9	47	73	36	65	7.26E-05	9.60E-05	6.81E-05	6.67E-05
T10F2.1a	1550	1512	1653	2133	6.83E-06	5.69E-06	2.42E-06	2.68E-06
T10F2.1b.1	1564	1528	1624	2151	4.51E-06	6.61E-06	2.24E-06	5.02E-06
T10F2.1b.2	1550	1512	1653	2133	7.78E-05	7.17E-05	5.40E-05	8.60E-05
T10F2.2	262	373	257	481	7.69E-05	7.10E-05	5.20E-05	8.49E-05
T10F2.3	687	662	1090	1471	7.59E-05	6.99E-05	5.27E-05	8.39E-05
T10F2.4.1	1076	1162	1491	1889	3.24E-05	4.36E-05	2.07E-05	4.78E-05
T10F2.4.2	863	889	1210	1547	2.78E-05	2.53E-05	2.87E-05	4.79E-05
T10F2.5	92	155	281	125	6.33E-05	6.45E-05	5.71E-05	8.92E-05
T10G3.1	10	37	19	26	6.52E-05	6.35E-05	5.95E-05	9.39E-05
T10G3.2	4	8	6	5	2.49E-05	3.96E-05	4.95E-05	2.72E-05
T10G3.4	5	6	9	2	2.80E-06	3.20E-06	1.82E-06	2.25E-06
T10G3.5a	621	657	483	940	2.80E-06	2.65E-06	1.82E-06	2.25E-06
T10G3.5b	580	620	449	885	2.80E-06	2.65E-06	1.82E-06	2.25E-06
T10G3.6	242	196	1083	275	1.78E-05	1.77E-05	8.98E-06	2.16E-05
T10H10.1	338	406	223	322	1.79E-05	1.81E-05	9.06E-06	2.20E-05
T10H10.2	97	129	80	119	6.33E-05	4.85E-05	1.84E-04	5.78E-05
T10H10.3	34	63	57	49	5.77E-06	6.56E-06	2.48E-06	4.41E-06
T10H4.1	6	7	11	3	5.68E-06	7.14E-06	3.06E-06	5.60E-06
T10H4.10	4	3	7	3	2.97E-06	5.18E-06	3.24E-06	3.44E-06
T10H4.11	15	10	2	8	2.80E-06	2.65E-06	1.82E-06	2.25E-06
T10H4.12	39	68	43	34	2.80E-06	2.65E-06	1.82E-06	2.25E-06
T10H4.2	4	6	4	2	2.80E-06	2.65E-06	1.82E-06	2.25E-06
T10H4.3	3	5	11	2	3.28E-06	5.42E-06	2.35E-06	2.29E-06
T10H4.4	5	5	22	4	2.80E-06	2.65E-06	1.82E-06	2.25E-06
T10H4.5	6	7	10	1	2.80E-06	2.65E-06	1.82E-06	2.25E-06
T10H4.6	4	6	22	5	2.80E-06	2.65E-06	1.82E-06	2.25E-06
T10H4.7	6	4	7	4	2.80E-06	2.65E-06	1.82E-06	2.25E-06
T10H4.8	3	19	8	11	2.80E-06	2.65E-06	1.82E-06	2.25E-06
T10H4.9	4	2	9	4	2.80E-06	2.65E-06	1.82E-06	2.25E-06
T10H9.1	8	9	8	25	2.80E-06	2.65E-06	1.82E-06	2.25E-06
T10H9.2	43	59	24	43	2.80E-06	2.65E-06	1.82E-06	2.25E-06
T10H9.3	351	330	430	451	2.80E-06	2.65E-06	1.82E-06	2.25E-06
T10H9.4	888	893	897	1273	2.80E-06	2.65E-06	1.82E-06	2.25E-06
T10H9.5a	352	1431	404	1953	3.04E-05	2.70E-05	2.42E-05	3.14E-05
T10H9.5b	334	1346	385	1850	1.23E-04	1.17E-04	8.10E-05	1.42E-04
T10H9.5c.1	351	1425	414	1961	2.11E-05	8.10E-05	1.58E-05	9.40E-05
T10H9.5c.2	333	1335	381	1889	1.98E-05	7.55E-05	1.49E-05	8.83E-05
T10H9.6a	5	14	7	10	1.70E-05	6.52E-05	1.30E-05	7.63E-05
T10H9.6b	5	16	7	10	2.19E-05	8.29E-05	1.63E-05	9.98E-05
T10H9.8	19	22	22	22	2.80E-06	2.65E-06	1.82E-06	2.25E-06
T11A5.1	9	10	10	8	2.80E-06	2.65E-06	1.82E-06	2.25E-06
T11A5.2	8	6	2	2	5.40E-06	5.92E-06	4.08E-06	5.04E-06
T11A5.3	3	5	3	2	2.80E-06	2.65E-06	1.82E-06	2.25E-06
T11A5.4	4	4	3	2	2.80E-06	2.65E-06	1.82E-06	2.25E-06
T11A5.5	6	10	1	2	2.80E-06	2.65E-06	1.82E-06	2.25E-06
T11A5.6	7	28	3	18	2.80E-06	2.65E-06	1.82E-06	2.25E-06
T11A5.7	4	2	5	2	2.80E-06	2.65E-06	1.82E-06	2.25E-06
T11B7.1	46	46	36	28	2.80E-06	2.65E-06	1.82E-06	2.25E-06
T11B7.2	17	18	31	7	2.80E-06	2.65E-06	1.82E-06	2.25E-06
T11B7.4a	703	835	546	691	3.28E-06	3.09E-06	1.82E-06	2.25E-06
T11B7.4b	685	781	549	681	6.69E-06	6.69E-06	7.93E-06	2.25E-06
T11B7.4c	554	627	468	604	5.82E-05	6.53E-05	2.94E-05	4.59E-05
T11B7.4d	737	889	585	729	3.93E-05	4.23E-05	2.05E-05	3.14E-05
T11B7.4e	683	780	538	675	2.67E-05	2.85E-05	1.47E-05	2.34E-05
T11B7.5.1	30	42	46	11	1.84E-05	2.10E-05	9.51E-06	1.46E-05

T11B7.5.2	24	31	37	6	3.70E-05	3.99E-05	1.90E-05	2.93E-05
T11F1.1	6	5	5	4	5.15E-06	6.82E-06	5.14E-06	2.25E-06
T11F1.2	8	9	24	8	4.45E-06	5.45E-06	4.46E-06	2.25E-06
T11F1.3	1	0	0	1	2.80E-06	2.65E-06	1.82E-06	2.25E-06
T11F1.5	5	2	7	5	2.80E-06	2.65E-06	1.82E-06	2.25E-06
T11F1.6	4	2	21	2	2.80E-06	2.65E-06	1.82E-06	2.25E-06
T11F1.7a	4	8	13	10	2.80E-06	2.65E-06	1.82E-06	2.25E-06
T11F1.7b	2	6	6	9	2.80E-06	2.65E-06	1.82E-06	2.25E-06
T11F1.8	13	14	12	11	2.80E-06	2.65E-06	1.82E-06	2.25E-06
T11F8.1	85	85	117	147	2.80E-06	2.65E-06	1.82E-06	2.25E-06
T11F8.2	34	25	21	32	2.80E-06	2.65E-06	1.82E-06	2.25E-06
T11F8.4	20	38	11	19	1.21E-05	1.15E-05	1.08E-05	1.68E-05
T11F8.5	21	28	120	47	3.00E-06	2.65E-06	1.82E-06	2.25E-06
T11F9.1	6	9	21	13	2.80E-06	2.65E-06	1.82E-06	2.25E-06
T11F9.10	7	6	12	3	7.34E-06	9.23E-06	2.72E-05	1.32E-05
T11F9.11	116	235	98	151	2.80E-06	2.65E-06	1.82E-06	2.25E-06
T11F9.12	281	392	233	520	2.80E-06	2.65E-06	1.82E-06	2.25E-06
T11F9.13	1	0	0	0	1.25E-05	2.39E-05	6.87E-06	1.30E-05
T11F9.15	1	0	1	1	1.34E-05	1.76E-05	7.22E-06	1.99E-05
T11F9.16	1	0	1	1	2.80E-06	2.65E-06	1.82E-06	2.25E-06
T11F9.17	4	4	13	3	2.80E-06	2.65E-06	1.82E-06	2.25E-06
T11F9.18	4	3	5	5	2.80E-06	2.65E-06	1.82E-06	2.25E-06
T11F9.19	6	4	5	3	2.80E-06	2.65E-06	1.82E-06	2.25E-06
T11F9.2a	13	34	22	29	2.80E-06	2.65E-06	1.82E-06	2.25E-06
T11F9.2b	15	34	24	31	2.80E-06	2.65E-06	1.82E-06	2.25E-06
T11F9.3	182	202	185	443	2.80E-06	2.65E-06	1.82E-06	2.25E-06
T11F9.4	81	107	81	122	2.80E-06	2.65E-06	1.82E-06	2.25E-06
T11F9.5	5	11	5	10	1.79E-05	1.88E-05	1.18E-05	3.49E-05
T11F9.6	11	13	8	17	5.18E-06	6.48E-06	3.37E-06	6.27E-06
T11F9.7	2	2	3	2	2.80E-06	2.65E-06	1.82E-06	2.25E-06
T11F9.8	8	14	4	7	2.80E-06	2.65E-06	1.82E-06	2.54E-06
T11F9.9	943	827	413	340	2.80E-06	2.65E-06	1.82E-06	2.25E-06
T11G6.1b	499	665	572	838	2.80E-06	3.17E-06	1.82E-06	2.25E-06
T11G6.3	54	95	40	32	1.17E-04	9.72E-05	3.35E-05	3.40E-05
T11G6.4	62	84	54	80	3.56E-05	4.48E-05	2.65E-05	4.80E-05
T11G6.5	998	927	1236	1968	3.84E-06	6.40E-06	1.86E-06	2.25E-06
T11G6.6	1	2	1	0	5.26E-06	6.72E-06	2.99E-06	5.44E-06
T11G6.7	0	1	0	0	2.48E-05	2.18E-05	2.00E-05	3.93E-05
T11G6.8.1	661	782	858	1096	2.80E-06	2.65E-06	1.82E-06	2.25E-06
T11G6.8.2	556	659	682	941	2.80E-06	2.65E-06	1.82E-06	2.25E-06
T12A2.1	32	79	56	70	5.22E-05	5.83E-05	4.41E-05	6.95E-05
T12A2.10	4	5	5	12	5.03E-05	5.63E-05	4.02E-05	6.84E-05
T12A2.11	2	8	4	7	2.80E-06	6.24E-06	3.04E-06	4.70E-06
T12A2.12	0	4	7	1	2.80E-06	2.65E-06	1.82E-06	2.25E-06
T12A2.13	12	9	63	16	2.80E-06	2.65E-06	1.82E-06	2.25E-06
T12A2.15a	85	178	98	146	2.80E-06	2.65E-06	1.82E-06	2.25E-06
T12A2.15b	34	65	24	49	2.80E-06	2.65E-06	4.90E-06	2.25E-06
T12A2.16a	10	8	10	3	4.40E-06	8.70E-06	3.30E-06	6.07E-06
T12A2.16b	17	12	11	5	3.67E-06	6.61E-06	1.82E-06	4.23E-06
T12A2.2.1	2442	2539	3170	4297	2.80E-06	2.65E-06	1.82E-06	2.25E-06
T12A2.2.2	2292	2385	3012	4056	2.80E-06	2.65E-06	1.82E-06	2.25E-06
T12A2.2.3	2065	2138	2745	3773	9.22E-05	9.05E-05	7.79E-05	1.30E-04
T12A2.3	8	19	15	4	9.01E-05	8.85E-05	7.70E-05	1.28E-04
T12A2.5	9	11	16	6	1.02E-04	9.93E-05	8.79E-05	1.49E-04
T12A2.6	5	6	15	8	2.80E-06	2.67E-06	1.82E-06	2.25E-06
T12A2.7	298	332	210	253	2.80E-06	2.65E-06	1.82E-06	2.25E-06
T12A2.8.1	898	987	1090	1369	2.80E-06	2.65E-06	1.82E-06	2.25E-06
T12A2.8.2	484	506	604	858	3.88E-05	4.08E-05	1.78E-05	2.65E-05
T12A2.9	5	5	7	1	3.95E-05	4.11E-05	3.12E-05	4.84E-05

T12A7.1.1	40	56	59	105	4.12E-05	4.07E-05	3.35E-05	5.87E-05
T12A7.1.2	37	49	43	92	2.80E-06	2.65E-06	1.82E-06	2.25E-06
T12A7.3	7	3	5	1	2.80E-06	3.04E-06	2.20E-06	4.84E-06
T12A7.5	49	73	39	67	2.80E-06	2.72E-06	1.82E-06	4.34E-06
T12A7.6	32	38	17	11	2.80E-06	2.65E-06	1.82E-06	2.25E-06
T12A7.7	2	4	8	4	2.80E-06	2.65E-06	1.82E-06	2.25E-06
T12B3.1	4	8	7	3	3.22E-06	3.62E-06	1.82E-06	2.25E-06
T12B3.2	82	126	92	134	2.80E-06	2.65E-06	1.82E-06	2.25E-06
T12B3.3	270	514	263	214	2.80E-06	2.65E-06	1.82E-06	2.25E-06
T12B3.4	191	185	277	370	6.16E-06	8.94E-06	4.50E-06	8.10E-06
T12B5.1	25	17	19	24	2.61E-05	4.69E-05	1.65E-05	1.66E-05
T12B5.10	81	139	74	116	2.40E-05	2.20E-05	2.27E-05	3.74E-05
T12B5.11	45	61	40	35	2.97E-06	2.65E-06	1.82E-06	2.29E-06
T12B5.12	9	8	12	7	8.54E-06	1.39E-05	5.08E-06	9.83E-06
T12B5.13	6	10	30	14	5.04E-06	6.45E-06	2.92E-06	3.15E-06
T12B5.2	24	24	18	23	2.80E-06	2.65E-06	1.82E-06	2.25E-06
T12B5.3	7	6	7	12	2.80E-06	2.65E-06	2.90E-06	2.25E-06
T12B5.4	13	20	70	25	2.80E-06	2.65E-06	1.82E-06	2.25E-06
T12B5.5	2	1	2	3	2.80E-06	2.65E-06	1.82E-06	2.25E-06
T12B5.6a	9	20	13	17	2.80E-06	2.65E-06	5.74E-06	2.54E-06
T12B5.6b	30	44	21	35	2.80E-06	2.65E-06	1.82E-06	2.25E-06
T12B5.7	5	5	5	6	4.06E-06	8.54E-06	3.83E-06	6.16E-06
T12B5.8	49	55	27	31	3.53E-06	4.87E-06	1.82E-06	3.31E-06
T12B5.9	1	3	1	3	2.80E-06	2.65E-06	1.82E-06	2.25E-06
T12C9.1	8	17	20	17	5.77E-06	6.11E-06	2.08E-06	2.92E-06
T12C9.3	40	39	23	61	2.80E-06	2.65E-06	1.82E-06	2.25E-06
T12C9.5a.1	11	37	22	45	2.80E-06	2.65E-06	1.82E-06	2.25E-06
T12C9.5a.2	10	35	18	45	2.80E-06	2.65E-06	1.82E-06	2.25E-06
T12C9.5b	9	30	17	39	2.80E-06	3.54E-06	1.82E-06	3.67E-06
T12C9.6	9	12	21	7	2.80E-06	3.52E-06	1.82E-06	3.85E-06
T12C9.7	259	213	329	442	2.80E-06	3.02E-06	1.82E-06	3.33E-06
T12D8.1	435	475	459	851	2.80E-06	2.65E-06	1.82E-06	2.25E-06
T12D8.2.1	609	559	521	745	2.12E-05	1.64E-05	1.75E-05	2.90E-05
T12D8.2.2	290	297	287	371	6.10E-06	6.30E-06	4.19E-06	9.60E-06
T12D8.3	150	148	236	298	6.83E-05	5.92E-05	3.80E-05	6.72E-05
T12D8.4	58	75	40	50	4.95E-05	4.79E-05	3.19E-05	5.09E-05
T12D8.7	157	168	253	283	1.54E-05	1.44E-05	1.58E-05	2.46E-05
T12D8.8.1	963	900	1138	1245	4.34E-06	5.29E-06	1.95E-06	2.99E-06
T12D8.8.2	974	895	1169	1251	2.98E-05	3.02E-05	3.13E-05	4.32E-05
T12D8.8.3	784	768	927	1061	7.49E-05	6.61E-05	5.76E-05	7.78E-05
T12D8.9a	532	601	315	576	7.30E-05	6.33E-05	5.70E-05	7.53E-05
T12D8.9b	540	610	324	585	6.90E-05	6.39E-05	5.31E-05	7.50E-05
T12E12.3	222	235	357	555	1.58E-05	1.68E-05	6.09E-06	1.37E-05
T12E12.4a.1	793	888	1262	1553	1.53E-05	1.63E-05	5.96E-06	1.33E-05
T12E12.4a.2	721	813	1199	1478	8.79E-06	8.78E-06	9.20E-06	1.77E-05
T12E12.4b	736	831	1212	1502	3.67E-05	3.88E-05	3.80E-05	5.77E-05
T12E12.6	31	42	27	19	3.67E-05	3.91E-05	3.97E-05	6.04E-05
T12F5.1	114	143	108	142	3.65E-05	3.90E-05	3.92E-05	5.99E-05
T12F5.2	185	197	179	268	2.80E-06	2.65E-06	1.82E-06	2.25E-06
T12F5.3	544	657	509	986	1.70E-05	2.02E-05	1.05E-05	1.70E-05
T12F5.4	463	491	432	661	1.89E-05	1.90E-05	1.19E-05	2.20E-05
T12F5.5a	2006	1888	2346	3494	1.59E-05	1.82E-05	9.69E-06	2.32E-05
T12F5.5b	1865	1760	2213	3294	1.11E-05	1.11E-05	6.72E-06	1.27E-05
T12G3.1.1	302	673	261	456	6.73E-05	5.98E-05	5.12E-05	9.41E-05
T12G3.2a.1	328	472	332	620	9.86E-05	8.79E-05	7.61E-05	1.40E-04
T12G3.2a.2	331	477	330	621	1.38E-05	2.91E-05	7.78E-06	1.68E-05
T12G3.2b.1	337	488	336	626	1.20E-05	1.63E-05	7.91E-06	1.82E-05
T12G3.2b.2	309	449	318	604	1.21E-05	1.64E-05	7.82E-06	1.82E-05
T12G3.2b.3	309	448	318	603	1.21E-05	1.66E-05	7.85E-06	1.81E-05

T12G3.2c	329	475	326	618	1.21E-05	1.66E-05	8.13E-06	1.90E-05
T12G3.4	167	185	216	216	1.24E-05	1.70E-05	8.29E-06	1.94E-05
T12G3.6	359	411	521	483	1.26E-05	1.72E-05	8.14E-06	1.91E-05
T12G3.8	101	128	87	116	1.37E-05	1.43E-05	1.15E-05	1.42E-05
T13A10.1	22	20	8	10	5.38E-05	5.82E-05	5.08E-05	5.82E-05
T13A10.11a.:	137	303	143	236	1.17E-05	1.40E-05	6.54E-06	1.08E-05
T13A10.11a.:	133	291	136	226	5.26E-06	4.52E-06	1.82E-06	2.25E-06
T13A10.12	9	8	10	8	1.18E-05	2.46E-05	8.00E-06	1.63E-05
T13A10.13	3	5	5	5	1.16E-05	2.40E-05	7.74E-06	1.59E-05
T13A10.14	8	6	6	3	2.80E-06	2.65E-06	1.82E-06	2.25E-06
T13A10.2	1	0	2	1	2.80E-06	2.65E-06	1.82E-06	2.25E-06
T13A10.6	6	3	3	3	2.80E-06	2.65E-06	1.82E-06	2.25E-06
T13A10.7	6	5	4	3	2.80E-06	2.65E-06	1.82E-06	2.25E-06
T13A10.9	9	8	16	4	2.80E-06	2.65E-06	1.82E-06	2.25E-06
T13B5.1.1	176	246	122	279	2.80E-06	2.65E-06	1.82E-06	2.25E-06
T13B5.1.2	139	195	89	215	2.80E-06	2.65E-06	1.82E-06	2.25E-06
T13B5.3	171	342	292	418	9.13E-06	1.20E-05	4.12E-06	1.16E-05
T13B5.4	72	85	42	46	8.62E-06	1.14E-05	3.59E-06	1.07E-05
T13B5.5	4	7	8	1	1.45E-05	2.74E-05	1.61E-05	2.84E-05
T13B5.6	7	5	8	2	6.44E-06	7.19E-06	2.44E-06	3.31E-06
T13B5.7	4	7	8	4	2.80E-06	2.65E-06	1.82E-06	2.25E-06
T13B5.8	82	71	105	128	2.80E-06	2.65E-06	1.82E-06	2.25E-06
T13B5.9	16	18	12	26	2.80E-06	2.65E-06	1.82E-06	2.25E-06
T13C2.2	36	39	23	30	1.30E-05	1.06E-05	1.08E-05	1.63E-05
T13C2.3a	40	82	49	40	2.80E-06	2.65E-06	1.82E-06	2.25E-06
T13C2.3b	54	90	66	47	2.80E-06	2.65E-06	1.82E-06	2.25E-06
T13C2.4	281	190	386	274	3.44E-06	6.69E-06	2.75E-06	2.77E-06
T13C2.6a.1	2106	2060	2944	3916	3.78E-06	5.95E-06	3.01E-06	2.65E-06
T13C2.6a.2	1687	1696	2409	3157	5.30E-05	3.38E-05	4.74E-05	4.15E-05
T13C2.6b	1693	1700	2410	3154	7.05E-05	6.51E-05	6.41E-05	1.05E-04
T13C2.7	15	20	15	14	5.68E-05	5.40E-05	5.28E-05	8.54E-05
T13C5.1a	17	28	19	22	6.83E-05	6.48E-05	6.32E-05	1.02E-04
T13C5.1b	13	20	16	21	2.80E-06	2.65E-06	1.82E-06	2.25E-06
T13C5.2	6	17	11	7	2.80E-06	2.65E-06	1.82E-06	2.25E-06
T13C5.3	12	9	7	2	2.80E-06	2.65E-06	1.82E-06	2.25E-06
T13C5.4	3	5	4	3	2.80E-06	2.65E-06	1.82E-06	2.25E-06
T13C5.5a	83	98	72	100	2.80E-06	2.65E-06	1.82E-06	2.25E-06
T13C5.5b.1	144	184	169	190	2.80E-06	2.65E-06	1.82E-06	2.25E-06
T13C5.6	23	37	32	43	1.14E-05	1.27E-05	6.43E-06	1.10E-05
T13C5.7	7	11	6	2	1.01E-05	1.22E-05	7.73E-06	1.07E-05
T13F2.1.1	1244	2071	1365	1531	4.31E-06	6.56E-06	3.92E-06	6.50E-06
T13F2.1.2	1286	2118	1494	1548	2.80E-06	2.65E-06	1.82E-06	2.25E-06
T13F2.1.3	1155	1886	1251	1462	9.09E-05	1.43E-04	6.49E-05	8.98E-05
T13F2.10	2040	2789	2945	760	9.43E-05	1.47E-04	7.13E-05	9.11E-05
T13F2.11	1975	2581	2531	706	9.34E-05	1.44E-04	6.58E-05	9.50E-05
T13F2.12	12	12	12	5	5.64E-04	7.29E-04	5.30E-04	1.69E-04
T13F2.2.1	516	519	413	501	5.14E-04	6.35E-04	4.29E-04	1.48E-04
T13F2.2.2	415	438	349	466	3.64E-06	3.44E-06	2.37E-06	2.25E-06
T13F2.3a	535	447	593	862	1.17E-04	1.11E-04	6.09E-05	9.12E-05
T13F2.3b	524	432	585	856	8.87E-05	8.85E-05	4.85E-05	8.00E-05
T13F2.4	17	18	11	4	1.78E-05	1.40E-05	1.28E-05	2.31E-05
T13F2.5	19	24	36	25	1.82E-05	1.42E-05	1.32E-05	2.38E-05
T13F2.6	803	921	1429	1612	2.80E-06	2.72E-06	1.82E-06	2.25E-06
T13F2.7	615	757	632	960	2.80E-06	2.65E-06	2.37E-06	2.25E-06
T13F2.9	199	439	167	85	3.70E-05	4.01E-05	4.29E-05	5.97E-05
T13F3.1	3	1	12	9	3.14E-05	3.64E-05	2.10E-05	3.93E-05
T13F3.2	4	5	12	3	2.79E-05	5.81E-05	1.52E-05	9.56E-06
T13F3.3	7	5	3	8	2.80E-06	2.65E-06	1.82E-06	2.25E-06
T13F3.4	2	4	8	4	2.80E-06	2.65E-06	1.82E-06	2.25E-06

T13F3.5	10	19	14	8	2.80E-06	2.65E-06	1.82E-06	2.25E-06
T13F3.6	292	410	626	431	2.80E-06	2.65E-06	1.82E-06	2.25E-06
T13F3.7	4	15	8	3	2.80E-06	2.65E-06	1.82E-06	2.25E-06
T13F3.8	58	78	31	33	5.23E-05	6.94E-05	7.30E-05	6.20E-05
T13F3.9	4	3	5	4	2.80E-06	2.65E-06	1.82E-06	2.25E-06
T13G4.1	1	3	3	5	1.02E-05	1.29E-05	3.53E-06	4.66E-06
T13G4.2	3	7	5	7	2.80E-06	2.65E-06	1.82E-06	2.25E-06
T13G4.3	57	83	45	70	2.80E-06	2.65E-06	1.82E-06	2.25E-06
T13G4.4	33	45	16	29	2.80E-06	2.65E-06	1.82E-06	2.25E-06
T13G4.5	5	1	6	2	2.80E-06	2.65E-06	1.82E-06	2.25E-06
T13G4.6	2	1	4	4	3.67E-06	4.73E-06	1.82E-06	2.59E-06
T13G4.7	0	1	0	1	2.80E-06	2.65E-06	1.82E-06	2.25E-06
T13H10.1	24	46	25	16	2.80E-06	2.65E-06	1.82E-06	2.25E-06
T13H10.2	13	32	18	22	2.80E-06	2.65E-06	1.82E-06	2.25E-06
T13H2.4a	689	572	697	1207	2.80E-06	2.91E-06	1.82E-06	2.25E-06
T13H2.4b	18	36	18	25	2.80E-06	2.65E-06	1.82E-06	2.25E-06
T13H2.5a	629	459	619	816	1.48E-05	1.16E-05	9.73E-06	2.08E-05
T13H2.5b	269	201	292	405	2.80E-06	2.65E-06	1.82E-06	2.25E-06
T13H5.1a	55	121	50	82	9.35E-06	6.45E-06	5.98E-06	9.74E-06
T13H5.1b	75	146	58	90	9.18E-06	6.48E-06	6.49E-06	1.11E-05
T13H5.2	75	109	37	72	2.88E-06	5.98E-06	1.82E-06	3.44E-06
T13H5.3	23	42	11	7	3.16E-06	5.82E-06	1.82E-06	3.06E-06
T13H5.5	282	260	415	201	6.80E-06	9.31E-06	2.19E-06	5.24E-06
T13H5.6	53	82	45	73	2.80E-06	2.65E-06	1.82E-06	2.25E-06
T13H5.7	215	429	198	272	4.40E-05	3.84E-05	4.22E-05	2.52E-05
T14A8.1	78	87	51	78	3.75E-06	5.48E-06	2.08E-06	4.14E-06
T14B1.1.1	73	84	140	122	2.17E-05	4.10E-05	1.30E-05	2.21E-05
T14B1.1.2	71	84	137	123	5.57E-06	5.90E-06	2.37E-06	4.48E-06
T14B1.1.3	25	47	57	51	3.08E-06	3.36E-06	3.84E-06	4.14E-06
T14B1.2	12	10	8	8	3.11E-06	3.49E-06	3.92E-06	4.34E-06
T14B4.1	555	505	728	1148	2.80E-06	4.39E-06	3.68E-06	4.05E-06
T14B4.2	163	133	496	211	2.80E-06	2.65E-06	1.82E-06	2.25E-06
T14B4.3	200	272	215	240	1.77E-05	1.52E-05	1.51E-05	2.94E-05
T14B4.4b.1	53	46	41	24	3.70E-05	2.85E-05	7.33E-05	3.85E-05
T14B4.4b.2	55	51	48	22	2.16E-05	2.77E-05	1.51E-05	2.08E-05
T14B4.5	26	25	22	19	8.54E-06	7.01E-06	4.30E-06	3.10E-06
T14B4.6	192	326	155	104	8.01E-06	7.01E-06	4.56E-06	2.56E-06
T14B4.7a.1	258	405	189	141	4.98E-06	4.52E-06	2.73E-06	2.92E-06
T14B4.7a.2	189	338	115	124	1.62E-05	2.61E-05	8.53E-06	7.06E-06
T14B4.7b.1	192	328	122	125	1.82E-05	2.70E-05	8.67E-06	7.98E-06
T14B4.7b.2	187	324	120	121	1.34E-05	2.26E-05	5.30E-06	7.04E-06
T14B4.8	7	11	3	13	1.46E-05	2.36E-05	6.05E-06	7.65E-06
T14B4.9	7	7	11	7	1.47E-05	2.40E-05	6.14E-06	7.62E-06
T14B4.t1	0	0	2	1	2.80E-06	2.65E-06	1.82E-06	2.25E-06
T14C1.1	14	21	9	10	2.80E-06	2.65E-06	1.82E-06	2.25E-06
T14C1.2	2	1	3	1	2.80E-06	2.65E-06	1.82E-06	2.25E-06
T14D7.1	134	196	124	113	2.80E-06	2.65E-06	1.82E-06	2.25E-06
T14D7.2	328	694	296	503	2.80E-06	2.65E-06	1.82E-06	2.25E-06
T14D7.3	9	23	4	3	1.11E-05	1.53E-05	6.67E-06	7.51E-06
T14E8.1a	297	514	281	441	1.43E-05	2.85E-05	8.36E-06	1.75E-05
T14E8.1b.1	298	513	282	444	3.28E-06	7.88E-06	1.82E-06	2.25E-06
T14E8.1b.2	265	471	274	418	9.04E-06	1.48E-05	5.56E-06	1.08E-05
T14E8.1c	294	512	279	438	9.18E-06	1.49E-05	5.65E-06	1.10E-05
T14E8.2	5	10	5	5	8.51E-06	1.43E-05	5.72E-06	1.08E-05
T14E8.3a	12	17	17	14	9.13E-06	1.50E-05	5.65E-06	1.09E-05
T14E8.3b	12	17	17	14	2.80E-06	2.65E-06	1.82E-06	2.25E-06
T14E8.3c	11	17	17	13	2.80E-06	2.65E-06	1.82E-06	2.25E-06
T14E8.3d	3	9	7	4	2.80E-06	2.65E-06	1.82E-06	2.25E-06
T14E8.4	8	14	10	6	2.80E-06	2.65E-06	1.82E-06	2.25E-06

T14F9.1.1	1880	2571	1722	2823	2.80E-06	2.65E-06	1.82E-06	2.25E-06
T14F9.1.2	1724	2342	1552	2612	2.80E-06	2.65E-06	1.82E-06	2.25E-06
T14F9.2	57	68	62	84	1.19E-04	1.54E-04	7.10E-05	1.44E-04
T14F9.3	269	470	402	782	1.12E-04	1.43E-04	6.55E-05	1.36E-04
T14F9.4b	6	13	6	7	4.54E-06	5.11E-06	3.21E-06	5.35E-06
T14F9.5	4	3	6	4	1.71E-05	2.82E-05	1.67E-05	4.00E-05
T14G10.2a.1	1102	951	1411	2142	2.80E-06	2.70E-06	1.82E-06	2.25E-06
T14G10.2a.2	1083	942	1408	2133	2.80E-06	2.65E-06	1.82E-06	2.25E-06
T14G10.2b.1	1137	988	1462	2242	2.66E-05	2.17E-05	2.22E-05	4.15E-05
T14G10.2b.2	1063	908	1400	2176	2.66E-05	2.18E-05	2.25E-05	4.21E-05
T14G10.3	197	252	254	232	2.64E-05	2.17E-05	2.21E-05	4.18E-05
T14G10.4	29	42	39	25	2.76E-05	2.22E-05	2.36E-05	4.53E-05
T14G10.5a.1	1302	1474	1877	2471	4.94E-05	5.96E-05	4.14E-05	4.67E-05
T14G10.5a.2	1266	1434	1840	2436	7.78E-06	1.07E-05	6.81E-06	5.40E-06
T14G10.5b	1347	1517	1948	2542	5.33E-05	5.70E-05	5.00E-05	8.12E-05
T14G10.6	455	558	678	904	5.40E-05	5.77E-05	5.11E-05	8.34E-05
T14G10.7.1	357	440	421	540	5.13E-05	5.45E-05	4.83E-05	7.77E-05
T14G10.7.2	336	407	374	487	4.19E-05	4.86E-05	4.06E-05	6.69E-05
T14G10.8	54	60	47	23	2.19E-05	2.55E-05	1.68E-05	2.66E-05
T14G11.1	51	92	53	63	2.28E-05	2.60E-05	1.65E-05	2.65E-05
T14G11.3	615	629	564	728	9.32E-06	9.79E-06	5.28E-06	3.19E-06
T14G12.2	33	37	23	30	4.37E-06	7.46E-06	2.95E-06	4.34E-06
T14G12.3	538	541	442	587	2.91E-05	2.82E-05	1.74E-05	2.77E-05
T14G12.4a	14	9	5	16	5.24E-06	5.55E-06	2.37E-06	3.82E-06
T14G12.4b.1	8	2	2	10	9.10E-05	8.65E-05	4.87E-05	7.98E-05
T14G12.4b.2	8	2	2	10	2.80E-06	2.65E-06	1.82E-06	2.25E-06
T14G12.6	4	4	2	4	2.80E-06	2.65E-06	1.82E-06	2.25E-06
T14G8.1	293	381	238	479	2.80E-06	2.65E-06	1.82E-06	2.25E-06
T14G8.2	8	5	5	5	2.80E-06	2.65E-06	1.82E-06	2.25E-06
T14G8.3a	531	645	421	598	5.91E-06	7.25E-06	3.12E-06	7.74E-06
T14G8.3b	456	567	379	537	2.80E-06	2.65E-06	1.82E-06	2.25E-06
T14G8.4	13	25	9	11	1.81E-05	2.07E-05	9.33E-06	1.64E-05
T15B12.1a	49	78	44	79	1.85E-05	2.17E-05	1.00E-05	1.75E-05
T15B12.1b	128	181	168	176	2.80E-06	2.65E-06	1.82E-06	2.25E-06
T15B12.2	30	30	15	11	7.78E-06	1.17E-05	4.56E-06	1.01E-05
T15B12.t1	0	0	0	1	7.84E-06	1.05E-05	6.71E-06	8.68E-06
T15B7.10	3	5	6	5	2.80E-06	2.65E-06	1.82E-06	2.25E-06
T15B7.11	6	10	10	14	2.80E-06	2.65E-06	1.82E-06	2.25E-06
T15B7.12	8	6	9	5	2.80E-06	2.65E-06	1.82E-06	2.25E-06
T15B7.13	6	8	13	7	2.80E-06	2.65E-06	1.82E-06	2.25E-06
T15B7.14	19	22	13	5	2.80E-06	2.65E-06	1.82E-06	2.25E-06
T15B7.15	10	14	11	8	2.80E-06	2.65E-06	1.82E-06	2.25E-06
T15B7.16	12	16	8	14	2.80E-06	2.65E-06	1.82E-06	2.25E-06
T15B7.17	3	9	4	5	2.80E-06	2.65E-06	1.82E-06	2.25E-06
T15B7.2.1	597	992	907	881	2.80E-06	2.65E-06	1.82E-06	2.25E-06
T15B7.2.2	509	867	699	702	2.80E-06	2.65E-06	1.82E-06	2.25E-06
T15B7.3	2069	3494	3739	6526	8.59E-05	1.35E-04	8.50E-05	1.02E-04
T15B7.4	1333	1349	1476	1591	8.59E-05	1.38E-04	7.67E-05	9.51E-05
T15B7.5	40	77	49	45	2.33E-04	3.71E-04	2.74E-04	5.89E-04
T15B7.6	4	1	1	0	1.47E-04	1.40E-04	1.06E-04	1.41E-04
T15B7.7	11	6	7	7	4.73E-06	8.62E-06	3.77E-06	4.27E-06
T15B7.8	28	9	31	16	2.80E-06	2.65E-06	1.82E-06	2.25E-06
T15D6.1	2	3	3	2	2.80E-06	2.65E-06	1.82E-06	2.25E-06
T15D6.10	4	5	4	10	3.47E-06	2.65E-06	2.51E-06	2.25E-06
T15D6.11	6	3	6	3	2.80E-06	2.65E-06	1.82E-06	2.25E-06
T15D6.12	10	9	8	3	2.80E-06	2.65E-06	1.82E-06	2.25E-06
T15D6.2	5	0	9	1	2.80E-06	2.65E-06	1.82E-06	2.25E-06
T15D6.3	2	7	8	5	2.80E-06	2.65E-06	1.82E-06	2.25E-06
T15D6.4	17	15	10	10	2.80E-06	2.65E-06	1.82E-06	2.25E-06

T15D6.5	4	3	3	3	2.80E-06	2.65E-06	1.82E-06	2.25E-06
T15D6.6	3	3	12	7	2.80E-06	2.65E-06	1.82E-06	2.25E-06
T15D6.7	3	0	6	3	2.80E-06	2.65E-06	1.82E-06	2.25E-06
T15D6.8	3	5	5	5	2.80E-06	2.65E-06	1.82E-06	2.25E-06
T15D6.9	8	8	8	4	2.80E-06	2.65E-06	1.82E-06	2.25E-06
T15H9.1.1	379	437	481	695	2.80E-06	2.65E-06	1.82E-06	2.25E-06
T15H9.1.2	1140	1266	1213	2085	2.80E-06	2.65E-06	1.82E-06	2.25E-06
T15H9.2	82	88	96	120	3.95E-05	4.31E-05	3.26E-05	5.82E-05
T15H9.3	10	7	12	5	8.56E-05	8.98E-05	5.93E-05	1.26E-04
T15H9.4	11	16	8	7	1.21E-05	1.23E-05	9.22E-06	1.42E-05
T15H9.5	9	9	6	6	2.80E-06	2.65E-06	1.82E-06	2.25E-06
T15H9.6	9	13	49	17	2.80E-06	2.65E-06	1.82E-06	2.25E-06
T15H9.7	108	140	234	268	2.80E-06	2.65E-06	1.82E-06	2.25E-06
T16A1.1a	11	23	19	18	2.80E-06	2.65E-06	2.15E-06	2.25E-06
T16A1.1b	11	17	10	14	1.60E-05	1.96E-05	2.26E-05	3.19E-05
T16A1.2	15	20	12	13	2.80E-06	2.65E-06	1.82E-06	2.25E-06
T16A1.3	4	12	3	4	2.80E-06	2.65E-06	1.82E-06	2.25E-06
T16A1.4	1	1	1	1	2.80E-06	2.65E-06	1.82E-06	2.25E-06
T16A1.5	2	0	0	1	2.80E-06	2.65E-06	1.82E-06	2.25E-06
T16A1.7	16	18	22	10	2.80E-06	2.65E-06	1.82E-06	2.25E-06
T16A1.8	12	6	10	9	2.80E-06	2.65E-06	1.82E-06	2.25E-06
T16A9.2	12	6	4	6	2.80E-06	2.65E-06	1.82E-06	2.25E-06
T16A9.3	7	15	2	5	2.80E-06	2.65E-06	1.82E-06	2.25E-06
T16A9.4	65	93	47	96	2.80E-06	2.65E-06	1.82E-06	2.25E-06
T16D1.1	1	3	1	1	2.80E-06	2.65E-06	1.82E-06	2.25E-06
T16D1.2	32	80	40	44	2.80E-06	3.47E-06	1.82E-06	3.04E-06
T16D1.t1	0	0	1	0	2.80E-06	2.65E-06	1.82E-06	2.25E-06
T16G1.1	32	32	19	10	2.80E-06	5.90E-06	2.02E-06	2.77E-06
T16G1.10a	50	51	49	45	2.80E-06	2.65E-06	1.82E-06	2.25E-06
T16G1.11	711	703	718	748	2.80E-06	2.65E-06	1.82E-06	2.25E-06
T16G1.2	65	85	22	22	1.24E-05	1.19E-05	7.89E-06	8.93E-06
T16G1.3	3	4	15	5	9.64E-05	9.00E-05	6.34E-05	8.15E-05
T16G1.4	18	24	14	24	5.94E-06	7.35E-06	1.82E-06	2.25E-06
T16G1.5	13	39	35	13	2.80E-06	2.65E-06	1.82E-06	2.25E-06
T16G1.6	41	84	29	57	2.80E-06	2.65E-06	1.82E-06	2.25E-06
T16G1.7	27	70	30	67	2.80E-06	2.72E-06	1.82E-06	2.25E-06
T16G1.8	15	18	9	6	3.56E-06	6.85E-06	1.82E-06	3.96E-06
T16G1.9	375	467	334	541	2.80E-06	5.26E-06	1.82E-06	4.27E-06
T16G12.1	989	745	1034	1181	2.80E-06	2.65E-06	1.82E-06	2.25E-06
T16G12.10	2	3	3	3	1.35E-05	1.58E-05	7.80E-06	1.56E-05
T16G12.3	237	171	388	324	1.92E-05	1.36E-05	1.31E-05	1.84E-05
T16G12.4	63	96	62	74	2.80E-06	2.65E-06	1.82E-06	2.25E-06
T16G12.5	854	615	933	1340	1.59E-05	1.08E-05	1.69E-05	1.75E-05
T16G12.6	411	449	625	933	1.10E-05	1.58E-05	7.03E-06	1.04E-05
T16G12.7	10	35	5	9	2.57E-05	1.75E-05	1.82E-05	3.23E-05
T16G12.8	55	92	95	78	1.56E-05	1.61E-05	1.55E-05	2.85E-05
T16G12.9	9	6	7	7	2.80E-06	3.86E-06	1.82E-06	2.25E-06
T16H12.1	9	13	12	18	5.12E-06	8.09E-06	5.76E-06	5.82E-06
T16H12.10	6	9	7	2	2.80E-06	2.65E-06	1.82E-06	2.25E-06
T16H12.11	139	163	199	213	2.80E-06	2.65E-06	1.82E-06	2.25E-06
T16H12.2	18	23	9	11	2.80E-06	2.65E-06	1.82E-06	2.25E-06
T16H12.3a	102	135	108	146	1.56E-05	1.73E-05	1.45E-05	1.92E-05
T16H12.3b	88	122	96	125	3.70E-06	4.44E-06	1.82E-06	2.25E-06
T16H12.4.1	225	280	382	473	8.88E-06	1.11E-05	6.12E-06	1.02E-05
T16H12.4.2	198	254	328	421	9.52E-06	1.25E-05	6.76E-06	1.09E-05
T16H12.5a	340	340	405	716	2.04E-05	2.40E-05	2.25E-05	3.44E-05
T16H12.5b.1	431	430	520	860	1.95E-05	2.36E-05	2.10E-05	3.33E-05
T16H12.5b.2	440	440	535	868	2.81E-05	2.65E-05	2.18E-05	4.75E-05
T16H12.5b.3	334	335	401	711	2.81E-05	2.65E-05	2.21E-05	4.50E-05

T16H12.6	74	154	52	52	3.02E-05	2.85E-05	2.39E-05	4.78E-05
T16H12.8	6	6	5	3	2.91E-05	2.76E-05	2.27E-05	4.97E-05
T16H12.9	15	28	24	10	4.31E-06	8.46E-06	1.97E-06	2.43E-06
T16H5.1a	8	13	6	10	2.80E-06	2.65E-06	1.82E-06	2.25E-06
T16H5.1b.1	9	9	5	10	2.80E-06	3.41E-06	2.00E-06	2.25E-06
T16H5.1b.2	8	11	5	10	2.80E-06	2.65E-06	1.82E-06	2.25E-06
T17A3.1	9	13	17	8	2.80E-06	2.65E-06	1.82E-06	2.25E-06
T17A3.10	1	2	4	5	2.80E-06	2.65E-06	1.82E-06	2.25E-06
T17A3.11	4	2	2	3	2.80E-06	2.65E-06	1.82E-06	2.25E-06
T17A3.12	6	58	24	55	2.80E-06	2.65E-06	1.82E-06	2.25E-06
T17A3.2	18	23	17	17	2.80E-06	2.65E-06	1.82E-06	2.25E-06
T17A3.3	5	6	8	8	2.80E-06	1.97E-05	5.61E-06	1.59E-05
T17A3.4	3	3	8	6	2.80E-06	2.65E-06	1.82E-06	2.25E-06
T17A3.6	4	5	6	2	2.80E-06	2.65E-06	1.82E-06	2.25E-06
T17A3.7	1	7	7	5	2.80E-06	2.65E-06	1.82E-06	2.25E-06
T17A3.8	13	24	24	18	2.80E-06	2.65E-06	1.82E-06	2.25E-06
T17A3.9	0	5	11	1	2.80E-06	2.65E-06	1.82E-06	2.25E-06
T17E9.1a	991	1127	1363	2099	2.80E-06	2.65E-06	1.82E-06	2.25E-06
T17E9.1b	267	323	417	648	2.80E-06	2.65E-06	1.82E-06	2.25E-06
T17E9.2a	993	1045	1172	1327	2.95E-05	3.17E-05	2.64E-05	5.02E-05
T17E9.2b	992	1106	1210	1401	2.27E-05	2.59E-05	2.31E-05	4.42E-05
T17E9.2c.1	893	941	1079	1269	8.12E-05	8.08E-05	6.24E-05	8.72E-05
T17E9.2c.2	993	1045	1172	1327	6.64E-05	7.00E-05	5.28E-05	7.54E-05
T17H7.1	73	95	22	16	7.08E-05	7.05E-05	5.56E-05	8.08E-05
T17H7.4a.1	493	673	521	835	8.12E-05	8.08E-05	6.24E-05	8.72E-05
T17H7.4a.2	493	668	516	823	3.72E-06	4.58E-06	1.82E-06	2.25E-06
T17H7.4a.3	508	697	547	857	2.64E-05	3.40E-05	1.81E-05	3.59E-05
T17H7.4b.1	453	612	479	765	2.64E-05	3.38E-05	1.80E-05	3.54E-05
T17H7.4b.2	453	607	474	753	2.61E-05	3.39E-05	1.83E-05	3.54E-05
T17H7.4b.3	468	636	505	787	2.65E-05	3.39E-05	1.83E-05	3.60E-05
T17H7.4c.1	499	674	533	853	2.65E-05	3.36E-05	1.81E-05	3.54E-05
T17H7.4c.2	499	669	528	841	2.62E-05	3.37E-05	1.84E-05	3.54E-05
T17H7.4c.3	514	698	559	875	2.51E-05	3.21E-05	1.75E-05	3.45E-05
T17H7.4d	157	220	136	210	2.51E-05	3.18E-05	1.73E-05	3.41E-05
T17H7.4e	328	430	324	470	2.50E-05	3.20E-05	1.77E-05	3.41E-05
T17H7.4f.1	348	460	349	518	4.68E-06	6.16E-06	2.62E-06	5.02E-06
T17H7.4f.2	300	387	285	409	2.57E-05	3.18E-05	1.65E-05	2.96E-05
T17H7.4f.3	305	393	294	433	2.21E-05	2.75E-05	1.44E-05	2.64E-05
T17H7.4f.4	323	426	322	461	2.45E-05	2.98E-05	1.51E-05	2.68E-05
T17H7.4f.5	341	459	338	500	2.64E-05	3.21E-05	1.66E-05	3.01E-05
T17H7.4g.1	425	601	410	611	2.48E-05	3.09E-05	1.61E-05	2.85E-05
T17H7.4g.2	591	828	616	972	2.36E-05	3.00E-05	1.52E-05	2.78E-05
T17H7.4h	294	383	281	406	2.61E-05	3.49E-05	1.64E-05	3.02E-05
T17H7.4i	309	406	300	437	2.74E-05	3.62E-05	1.86E-05	3.62E-05
T17H7.4j	508	697	547	857	2.71E-05	3.34E-05	1.69E-05	3.01E-05
T17H7.4k.1	470	626	493	767	2.58E-05	3.20E-05	1.63E-05	2.93E-05
T17H7.4k.2	469	625	491	765	2.61E-05	3.39E-05	1.83E-05	3.54E-05
T17H7.4l	287	383	277	405	2.63E-05	3.31E-05	1.79E-05	3.45E-05
T17H7.7	149	274	59	65	2.66E-05	3.35E-05	1.81E-05	3.48E-05
T18D3.1	92	81	65	107	2.50E-05	3.15E-05	1.57E-05	2.84E-05
T18D3.2	6	4	3	4	1.05E-05	1.82E-05	2.70E-06	3.67E-06
T18D3.3	44	68	38	24	5.43E-06	4.52E-06	2.50E-06	5.06E-06
T18D3.4	1200	1443	867	1327	2.80E-06	2.65E-06	1.82E-06	2.25E-06
T18D3.5	4	4	2	3	4.28E-06	6.27E-06	2.41E-06	2.25E-06
T18D3.6	21	20	34	40	2.13E-05	2.41E-05	9.99E-06	1.89E-05
T18D3.7.1	20	37	12	10	2.80E-06	2.65E-06	1.82E-06	2.25E-06
T18D3.7.2	10	22	7	6	2.88E-06	2.65E-06	3.04E-06	4.41E-06
T18D3.8	8	8	10	12	3.78E-06	6.59E-06	1.82E-06	2.25E-06
T18D3.9	12	21	11	7	3.50E-06	7.27E-06	1.82E-06	2.25E-06

T18D3.t1	1	0	3	0	2.80E-06	2.65E-06	1.82E-06	2.68E-06
T18H9.1	200	146	55	72	2.80E-06	4.07E-06	1.82E-06	2.25E-06
T18H9.2b	4029	5211	4829	7559	2.80E-06	2.65E-06	3.04E-06	2.25E-06
T18H9.4	10	4	5	1	1.33E-05	9.20E-06	2.39E-06	3.85E-06
T18H9.5a	63	70	58	92	3.49E-04	4.27E-04	2.72E-04	5.26E-04
T18H9.5b	47	48	44	73	2.80E-06	2.65E-06	1.82E-06	2.25E-06
T18H9.6	367	344	392	725	3.58E-06	3.76E-06	2.15E-06	4.21E-06
T18H9.7a	707	745	901	1278	3.39E-06	3.25E-06	2.06E-06	4.23E-06
T18H9.7c	372	430	530	767	2.84E-05	2.51E-05	1.97E-05	4.50E-05
T19A5.2a	459	463	511	802	2.79E-05	2.77E-05	2.31E-05	4.05E-05
T19A5.2b	454	462	502	797	2.40E-05	2.62E-05	2.23E-05	3.98E-05
T19A5.2c	425	449	461	753	2.62E-05	2.50E-05	1.90E-05	3.68E-05
T19A5.2d	470	472	522	816	2.60E-05	2.50E-05	1.87E-05	3.67E-05
T19A5.3a	182	307	114	115	2.29E-05	2.28E-05	1.61E-05	3.25E-05
T19A5.3b	159	259	80	101	2.60E-05	2.47E-05	1.88E-05	3.63E-05
T19A5.4	18	30	18	22	8.12E-06	1.29E-05	3.32E-06	4.12E-06
T19A5.5	7	2	11	6	9.44E-06	1.45E-05	3.08E-06	4.81E-06
T19A6.1b	515	567	548	835	2.80E-06	2.65E-06	1.82E-06	2.25E-06
T19A6.2a	623	739	618	845	2.80E-06	2.65E-06	1.82E-06	2.25E-06
T19A6.2b.1	518	628	547	768	6.28E-05	6.54E-05	4.35E-05	8.18E-05
T19A6.2b.2	504	619	528	755	3.25E-05	3.64E-05	2.10E-05	3.54E-05
T19A6.2b.3	574	686	582	808	3.12E-05	3.57E-05	2.14E-05	3.72E-05
T19A6.2b.4	564	677	577	799	2.83E-05	3.28E-05	1.93E-05	3.40E-05
T19A6.4	114	122	132	174	3.25E-05	3.67E-05	2.15E-05	3.68E-05
T19B10.1	80	98	62	93	3.20E-05	3.62E-05	2.13E-05	3.64E-05
T19B10.10	5	4	2	3	9.32E-06	9.44E-06	7.03E-06	1.14E-05
T19B10.11	160	163	568	192	5.26E-06	6.08E-06	2.66E-06	4.93E-06
T19B10.3	263	638	159	253	2.80E-06	2.65E-06	1.82E-06	2.25E-06
T19B10.4a	503	666	460	579	4.17E-05	4.01E-05	9.63E-05	4.02E-05
T19B10.5	148	150	81	137	1.36E-05	3.12E-05	5.36E-06	1.05E-05
T19B10.6.1	439	447	700	698	6.69E-05	8.37E-05	3.98E-05	6.19E-05
T19B10.6.2	397	416	662	673	7.14E-06	6.82E-06	2.53E-06	5.31E-06
T19B10.7.1	600	754	800	1121	4.05E-05	3.89E-05	4.20E-05	5.17E-05
T19B10.7.2	533	666	769	1044	3.82E-05	3.79E-05	4.15E-05	5.21E-05
T19B10.8	694	801	805	1145	3.56E-05	4.23E-05	3.09E-05	5.34E-05
T19B10.9	38	33	17	25	3.72E-05	4.39E-05	3.49E-05	5.85E-05
T19B4.1	106	171	92	157	3.07E-05	3.35E-05	2.32E-05	4.07E-05
T19B4.2	1965	1645	3183	4607	2.80E-06	2.65E-06	1.82E-06	2.25E-06
T19B4.3.1	162	292	329	206	5.94E-06	9.07E-06	3.35E-06	7.08E-06
T19B4.3.2	136	231	247	169	5.39E-05	4.26E-05	5.68E-05	1.02E-04
T19B4.5	614	452	729	994	2.77E-05	4.71E-05	3.66E-05	2.82E-05
T19B4.7	1281	1114	1740	2619	2.67E-05	4.29E-05	3.16E-05	2.67E-05
T19C3.1	38	54	70	56	5.72E-05	3.98E-05	4.42E-05	7.43E-05
T19C3.2	173	230	49	51	2.98E-05	2.45E-05	2.63E-05	4.89E-05
T19C3.3	60	85	177	29	2.83E-06	3.78E-06	3.39E-06	3.35E-06
T19C3.4	119	161	195	196	2.39E-05	3.00E-05	4.41E-06	5.67E-06
T19C3.5	32	71	40	49	1.60E-05	2.14E-05	3.06E-05	6.21E-06
T19C3.6	3	4	1	1	1.09E-05	1.39E-05	1.16E-05	1.44E-05
T19C3.7	1	2	10	7	2.80E-06	4.68E-06	1.82E-06	2.74E-06
T19C3.8	370	361	536	704	2.80E-06	2.65E-06	1.82E-06	2.25E-06
T19C3.9	16	38	24	23	2.80E-06	2.65E-06	1.82E-06	2.25E-06
T19C4.1	52	70	43	35	2.32E-05	2.14E-05	2.19E-05	3.55E-05
T19C4.10	1	6	5	4	4.17E-06	9.36E-06	4.08E-06	4.81E-06
T19C4.2	6	6	5	8	4.31E-06	5.48E-06	2.31E-06	2.32E-06
T19C4.3	4	2	6	4	2.80E-06	2.65E-06	1.82E-06	2.25E-06
T19C4.4	3	8	3	2	2.80E-06	2.65E-06	1.82E-06	2.25E-06
T19C4.5	5	38	9	12	2.80E-06	2.65E-06	1.82E-06	2.25E-06
T19C4.6	8	15	6	11	2.80E-06	2.65E-06	1.82E-06	2.25E-06
T19C4.7	396	545	190	64	2.80E-06	3.68E-06	1.82E-06	2.25E-06

T19C4.8	3	2	7	6	2.80E-06	2.65E-06	1.82E-06	2.25E-06
T19C4.9	3	4	7	3	1.29E-04	1.67E-04	4.01E-05	1.67E-05
T19C9.1	3	5	4	1	2.80E-06	2.65E-06	1.82E-06	2.25E-06
T19C9.2	1	1	3	3	2.80E-06	2.65E-06	1.82E-06	2.25E-06
T19C9.3	5	4	3	5	2.80E-06	2.65E-06	1.82E-06	2.25E-06
T19C9.4	12	1	8	7	2.80E-06	2.65E-06	1.82E-06	2.25E-06
T19C9.5	5	5	4	2	2.80E-06	2.65E-06	1.82E-06	2.25E-06
T19C9.6	3	1	2	1	2.80E-06	2.65E-06	1.82E-06	2.25E-06
T19C9.8	10	29	15	24	2.80E-06	2.65E-06	1.82E-06	2.25E-06
T19D12.1	794	735	671	995	2.80E-06	2.65E-06	1.82E-06	2.25E-06
T19D12.10	13	16	11	5	2.80E-06	2.65E-06	1.82E-06	2.25E-06
T19D12.2a.1	230	268	170	325	1.50E-05	1.31E-05	8.25E-06	1.51E-05
T19D12.2a.2	222	256	156	311	2.80E-06	2.65E-06	1.82E-06	2.25E-06
T19D12.2b	203	236	146	285	1.02E-05	1.12E-05	4.88E-06	1.15E-05
T19D12.2c.1	229	264	156	316	1.27E-05	1.39E-05	5.83E-06	1.43E-05
T19D12.2c.2	214	250	154	305	1.45E-05	1.59E-05	6.78E-06	1.64E-05
T19D12.3	1	1	2	2	1.19E-05	1.30E-05	5.28E-06	1.32E-05
T19D12.4a	607	457	558	619	1.32E-05	1.46E-05	6.20E-06	1.52E-05
T19D12.4b	579	428	519	586	2.80E-06	2.65E-06	1.82E-06	2.25E-06
T19D12.5	17	23	10	5	2.12E-05	1.51E-05	1.27E-05	1.74E-05
T19D12.6	263	494	324	505	2.12E-05	1.48E-05	1.24E-05	1.72E-05
T19D12.7	25	35	43	30	2.80E-06	2.65E-06	1.82E-06	2.25E-06
T19D12.8	10	4	8	4	1.14E-05	2.03E-05	9.17E-06	1.76E-05
T19D12.9	31	45	29	37	2.80E-06	2.65E-06	1.91E-06	2.25E-06
T19D2.1	60	124	44	61	2.80E-06	2.65E-06	1.82E-06	2.25E-06
T19D2.2	240	314	209	246	2.80E-06	3.20E-06	1.82E-06	2.25E-06
T19D2.3	6	11	2	4	2.80E-06	4.52E-06	1.82E-06	2.25E-06
T19D7.1	69	97	97	87	1.74E-05	2.15E-05	9.84E-06	1.43E-05
T19D7.2	35	70	10	15	2.80E-06	3.57E-06	1.82E-06	2.25E-06
T19D7.3	20	31	4	5	5.18E-06	6.88E-06	4.74E-06	5.24E-06
T19D7.4	27	64	39	56	9.07E-06	1.71E-05	1.82E-06	3.13E-06
T19D7.5	5	4	0	3	5.15E-06	7.56E-06	1.82E-06	2.25E-06
T19D7.6	13	23	12	11	2.80E-06	3.33E-06	1.82E-06	2.47E-06
T19D7.7	14	15	9	10	2.80E-06	2.65E-06	1.82E-06	2.25E-06
T19E10.1a	1092	858	1249	1583	2.80E-06	4.13E-06	1.82E-06	2.25E-06
T19E10.1b	1352	1081	1683	2070	2.80E-06	2.65E-06	1.82E-06	2.25E-06
T19E7.1	18	19	24	21	4.41E-05	3.27E-05	3.28E-05	5.13E-05
T19E7.2a	1168	1257	1433	1781	4.53E-05	3.43E-05	3.67E-05	5.58E-05
T19E7.2b	831	925	1036	1439	3.42E-06	3.41E-06	2.97E-06	3.22E-06
T19E7.2c.1	1073	1177	1349	1675	6.50E-05	6.61E-05	5.19E-05	7.97E-05
T19E7.2c.2	1089	1188	1360	1688	5.77E-05	6.06E-05	4.68E-05	8.02E-05
T19E7.2c.3	1181	1264	1463	1803	6.61E-05	6.85E-05	5.41E-05	8.29E-05
T19E7.2c.4	1168	1257	1433	1781	6.99E-05	7.20E-05	5.68E-05	8.70E-05
T19E7.2c.5	1145	1235	1402	1740	6.57E-05	6.64E-05	5.30E-05	8.06E-05
T19E7.2c.6	1072	1176	1347	1674	6.50E-05	6.61E-05	5.19E-05	7.97E-05
T19E7.3.1	176	270	222	267	6.52E-05	6.65E-05	5.20E-05	7.96E-05
T19E7.3.2	144	232	169	234	6.89E-05	7.14E-05	5.63E-05	8.64E-05
T19E7.5	4	5	10	5	1.55E-05	2.24E-05	1.27E-05	1.88E-05
T19E7.6	29	44	17	32	1.42E-05	2.16E-05	1.08E-05	1.85E-05
T19F4.1a	16	23	11	6	2.80E-06	2.65E-06	1.82E-06	2.25E-06
T19F4.1b	15	24	11	4	6.75E-06	9.68E-06	2.57E-06	5.98E-06
T19H12.10	23	30	19	19	2.80E-06	2.65E-06	1.82E-06	2.25E-06
T19H12.11	17	18	9	6	2.80E-06	2.65E-06	1.82E-06	2.25E-06
T19H12.12	14	25	12	12	2.80E-06	2.65E-06	1.82E-06	2.25E-06
T19H12.3	32	58	36	14	2.80E-06	2.65E-06	1.82E-06	2.25E-06
T19H12.4	2	3	3	4	3.89E-06	6.59E-06	2.17E-06	2.68E-06
T19H12.5	10	6	9	7	8.06E-06	1.38E-05	5.90E-06	2.83E-06
T19H12.6	11	15	18	7	2.80E-06	2.65E-06	1.82E-06	2.25E-06
T19H12.7	3	10	5	2	2.80E-06	2.65E-06	1.82E-06	2.25E-06

T19H12.8	12	17	13	6	2.80E-06	2.65E-06	1.82E-06	2.25E-06
T19H5.1	9	10	8	7	2.80E-06	2.65E-06	1.82E-06	2.25E-06
T19H5.2	3	6	12	7	2.80E-06	2.65E-06	1.82E-06	2.25E-06
T19H5.3	10	6	24	9	2.80E-06	2.65E-06	1.82E-06	2.25E-06
T19H5.4	182	248	215	219	2.80E-06	2.65E-06	1.82E-06	2.25E-06
T19H5.5	7	4	12	3	2.80E-06	2.65E-06	1.82E-06	2.25E-06
T19H5.6	9	5	6	4	1.26E-05	1.63E-05	9.71E-06	1.22E-05
T20B12.1	378	477	397	625	2.80E-06	2.65E-06	1.82E-06	2.25E-06
T20B12.2.1	502	421	737	812	2.80E-06	2.65E-06	1.82E-06	2.25E-06
T20B12.2.2	363	311	507	609	1.65E-05	1.97E-05	1.13E-05	2.20E-05
T20B12.4	6	5	5	5	4.07E-05	3.22E-05	3.89E-05	5.29E-05
T20B12.5	3	0	11	3	3.95E-05	3.20E-05	3.59E-05	5.32E-05
T20B12.6a	209	455	209	337	2.80E-06	2.65E-06	1.82E-06	2.25E-06
T20B12.6b	209	455	210	333	2.80E-06	2.65E-06	2.02E-06	2.25E-06
T20B12.7.1	615	767	1069	942	7.03E-06	1.44E-05	4.57E-06	9.11E-06
T20B12.8	769	923	949	1306	6.66E-06	1.37E-05	4.35E-06	8.52E-06
T20B12.9	9	28	31	21	7.31E-05	8.60E-05	8.26E-05	8.99E-05
T20B3.1	27	75	29	38	3.88E-05	4.40E-05	3.11E-05	5.29E-05
T20B3.11	1	5	8	5	2.80E-06	2.65E-06	1.82E-06	2.25E-06
T20B3.12	9	23	8	7	2.80E-06	4.21E-06	1.82E-06	2.25E-06
T20B3.13	10	8	12	5	2.80E-06	2.65E-06	1.82E-06	2.25E-06
T20B3.14	26	17	52	6	2.80E-06	2.65E-06	1.82E-06	2.25E-06
T20B3.15	0	3	4	3	2.80E-06	2.65E-06	1.82E-06	2.25E-06
T20B3.16	8	14	10	13	1.11E-05	6.88E-06	1.45E-05	2.25E-06
T20B3.3	4	3	5	5	2.80E-06	2.65E-06	1.82E-06	2.25E-06
T20B3.4	4	5	5	0	2.80E-06	2.65E-06	1.82E-06	2.25E-06
T20B3.5	7	3	4	2	2.80E-06	2.65E-06	1.82E-06	2.25E-06
T20B3.7	14	14	13	14	2.80E-06	2.65E-06	1.82E-06	2.25E-06
T20B3.8	6	5	9	3	2.80E-06	2.65E-06	1.82E-06	2.25E-06
T20B5.1.1	402	483	387	569	2.80E-06	2.65E-06	1.82E-06	2.25E-06
T20B5.1.2	338	420	333	513	2.80E-06	2.65E-06	1.82E-06	2.25E-06
T20B5.2	12	19	17	12	1.34E-05	1.52E-05	8.36E-06	1.52E-05
T20B5.3	147	333	195	260	1.36E-05	1.60E-05	8.73E-06	1.66E-05
T20B6.1	1	1	3	1	2.80E-06	2.65E-06	1.82E-06	2.25E-06
T20B6.2	15	19	14	11	5.32E-06	1.14E-05	4.59E-06	7.58E-06
T20B6.3	16	23	8	12	2.80E-06	2.65E-06	1.82E-06	2.25E-06
T20C4.1	6	4	2	2	2.80E-06	2.65E-06	1.82E-06	2.25E-06
T20C7.1	7	6	3	6	2.80E-06	3.12E-06	1.82E-06	2.25E-06
T20C7.2	2	7	3	5	2.80E-06	2.65E-06	1.82E-06	2.25E-06
T20D3.1	4	0	1	2	2.80E-06	2.65E-06	1.82E-06	2.25E-06
T20D3.11	950	1114	1066	1667	2.80E-06	2.65E-06	1.82E-06	2.25E-06
T20D3.12	0	1	1	1	2.80E-06	2.65E-06	1.82E-06	2.25E-06
T20D3.13	0	1	4	2	4.79E-05	5.31E-05	3.50E-05	6.76E-05
T20D3.2.1	1378	1997	2090	1426	2.80E-06	2.65E-06	1.82E-06	2.25E-06
T20D3.2.2	1138	1652	1557	1275	2.80E-06	2.65E-06	3.01E-06	2.25E-06
T20D3.3a.1	355	381	238	459	2.01E-04	2.76E-04	1.99E-04	1.67E-04
T20D3.3a.2	317	341	206	419	1.80E-04	2.47E-04	1.60E-04	1.62E-04
T20D3.3b	314	332	194	395	2.96E-05	3.00E-05	1.29E-05	3.07E-05
T20D3.6.1	100	125	282	80	3.10E-05	3.15E-05	1.31E-05	3.29E-05
T20D3.6.2	93	121	198	55	3.12E-05	3.12E-05	1.26E-05	3.16E-05
T20D3.7	530	514	496	906	1.97E-05	2.32E-05	3.61E-05	1.26E-05
T20D3.8	190	283	229	319	1.78E-05	2.19E-05	2.47E-05	8.46E-06
T20D4.1	8	7	9	2	3.70E-05	3.39E-05	2.25E-05	5.08E-05
T20D4.10	23	23	3	6	2.51E-05	3.53E-05	1.97E-05	3.38E-05
T20D4.11	28	28	13	9	2.80E-06	2.65E-06	1.82E-06	2.25E-06
T20D4.12	12	9	2	7	4.26E-06	4.02E-06	1.82E-06	2.25E-06
T20D4.13	6	7	17	8	5.24E-06	4.95E-06	1.82E-06	2.25E-06
T20D4.15	3	6	7	3	2.80E-06	2.65E-06	1.82E-06	2.25E-06
T20D4.16	1	0	2	0	2.80E-06	2.65E-06	2.46E-06	2.25E-06

T20D4.17	0	1	1	1	2.80E-06	2.65E-06	1.82E-06	2.25E-06
T20D4.18	0	4	8	3	2.80E-06	2.65E-06	1.82E-06	2.25E-06
T20D4.19	1	3	0	1	2.80E-06	2.65E-06	1.82E-06	2.25E-06
T20D4.2	4	7	6	4	2.80E-06	2.65E-06	1.82E-06	2.25E-06
T20D4.20	3	1	1	0	2.80E-06	2.65E-06	1.82E-06	2.25E-06
T20D4.3	29	17	18	10	2.80E-06	2.65E-06	1.82E-06	2.25E-06
T20D4.4	14	6	7	8	2.80E-06	2.65E-06	1.82E-06	2.25E-06
T20D4.5	18	19	22	3	2.80E-06	2.65E-06	1.82E-06	2.25E-06
T20D4.6	42	39	28	35	2.80E-06	2.65E-06	1.82E-06	2.25E-06
T20D4.7	4	17	12	2	2.80E-06	2.65E-06	1.82E-06	2.25E-06
T20D4.8	10	11	6	4	3.14E-06	2.75E-06	1.82E-06	2.25E-06
T20D4.9	16	17	19	12	2.80E-06	3.84E-06	1.88E-06	2.25E-06
T20F10.1	138	129	129	272	2.80E-06	2.65E-06	1.82E-06	2.25E-06
T20F10.2a	118	174	79	123	2.80E-06	2.65E-06	1.82E-06	2.25E-06
T20F10.2b	95	150	65	108	5.07E-06	4.47E-06	3.08E-06	8.01E-06
T20F10.2c	58	91	25	56	1.20E-05	1.67E-05	5.23E-06	1.01E-05
T20F10.4	10	12	13	14	1.30E-05	1.93E-05	5.78E-06	1.18E-05
T20F10.5	11	19	13	21	1.41E-05	2.08E-05	3.94E-06	1.09E-05
T20F10.6	54	52	52	44	2.80E-06	2.65E-06	1.82E-06	2.25E-06
T20F5.1	5	8	35	13	2.80E-06	2.65E-06	1.82E-06	2.25E-06
T20F5.2	588	774	756	596	2.80E-06	2.65E-06	1.82E-06	2.25E-06
T20F5.3	81	102	88	99	2.80E-06	2.65E-06	7.78E-06	3.58E-06
T20F5.4	59	52	38	52	9.08E-05	1.13E-04	7.60E-05	7.39E-05
T20F5.5	33	45	15	17	7.90E-06	9.39E-06	5.58E-06	7.74E-06
T20F5.6.1	703	618	852	1111	4.62E-06	3.84E-06	1.93E-06	3.26E-06
T20F5.6.2	733	643	890	1155	5.43E-06	6.98E-06	1.82E-06	2.25E-06
T20F5.7	262	258	233	348	2.79E-05	2.31E-05	2.20E-05	3.54E-05
T20F7.1	147	137	132	133	2.95E-05	2.45E-05	2.33E-05	3.74E-05
T20F7.3	5	7	8	5	3.29E-05	3.06E-05	1.91E-05	3.51E-05
T20F7.5	27	45	47	42	6.41E-06	5.63E-06	3.75E-06	4.66E-06
T20F7.7	11	15	9	17	2.80E-06	2.65E-06	1.82E-06	2.25E-06
T20G5.1	5689	4772	6428	8826	2.80E-06	2.65E-06	1.82E-06	2.25E-06
T20G5.10	99	119	111	104	2.80E-06	2.65E-06	1.82E-06	2.25E-06
T20G5.11.1	570	508	922	838	1.19E-04	9.46E-05	8.78E-05	1.49E-04
T20G5.11.2	564	503	916	834	2.69E-05	3.05E-05	1.96E-05	2.26E-05
T20G5.12	1	3	5	2	3.69E-05	3.11E-05	3.89E-05	4.36E-05
T20G5.13	2	3	2	1	5.25E-05	4.42E-05	5.54E-05	6.23E-05
T20G5.14	429	456	486	459	2.80E-06	2.65E-06	1.82E-06	2.25E-06
T20G5.2.1	6885	6233	8353	8274	2.80E-06	2.65E-06	1.82E-06	2.25E-06
T20G5.2.2	6222	5611	7305	7414	7.41E-05	7.45E-05	5.47E-05	6.37E-05
T20G5.4	15	40	33	34	4.60E-04	3.93E-04	3.63E-04	4.44E-04
T20G5.5	43	47	99	57	4.89E-04	4.17E-04	3.74E-04	4.68E-04
T20G5.7	479	641	330	313	2.80E-06	2.88E-06	1.82E-06	2.25E-06
T20G5.8	92	198	150	51	2.80E-06	2.65E-06	1.86E-06	2.25E-06
T20G5.9	114	107	227	131	9.31E-05	1.18E-04	4.18E-05	4.89E-05
T20H12.1	6	3	7	4	5.63E-05	1.14E-04	5.97E-05	2.51E-05
T20H4.1	17	39	28	13	2.58E-05	2.29E-05	3.34E-05	2.38E-05
T20H4.2	23	15	27	11	2.80E-06	2.65E-06	1.82E-06	2.25E-06
T20H4.3b	1052	1544	1129	1403	2.80E-06	2.65E-06	1.82E-06	2.25E-06
T20H4.4.1	468	448	541	925	3.64E-06	2.65E-06	2.79E-06	2.25E-06
T20H4.4.2	451	422	511	886	6.52E-05	9.04E-05	4.55E-05	6.98E-05
T20H9.1	2	3	1	0	3.09E-05	2.79E-05	2.32E-05	4.90E-05
T20H9.2	4	8	7	6	3.11E-05	2.75E-05	2.30E-05	4.91E-05
T20H9.3	11	12	18	6	2.80E-06	2.65E-06	1.82E-06	2.25E-06
T20H9.4	0	2	11	2	2.80E-06	2.65E-06	1.82E-06	2.25E-06
T20H9.5	2	2	3	2	2.80E-06	2.65E-06	1.82E-06	2.25E-06
T20H9.6	11	20	16	8	2.80E-06	2.65E-06	1.82E-06	2.25E-06
T21B10.1	269	297	374	220	2.80E-06	2.65E-06	1.82E-06	2.25E-06
T21B10.2a.1	3651	4823	6055	6046	2.80E-06	2.65E-06	1.82E-06	2.25E-06

T21B10.2a.2	3579	4732	5871	5991	3.02E-05	3.15E-05	2.73E-05	1.98E-05
T21B10.2b	2744	3549	4587	4407	3.11E-04	3.88E-04	3.35E-04	4.13E-04
T21B10.3	1359	1263	1931	2737	3.07E-04	3.83E-04	3.27E-04	4.12E-04
T21B10.4	689	644	755	924	3.03E-04	3.70E-04	3.29E-04	3.91E-04
T21B10.5	439	475	676	613	4.11E-05	3.61E-05	3.80E-05	6.65E-05
T21B10.6	53	98	35	55	5.07E-05	4.48E-05	3.62E-05	5.46E-05
T21B10.7.1	2951	2507	2976	3827	6.00E-05	6.14E-05	6.02E-05	6.73E-05
T21B10.7.2	2949	2497	2959	3810	4.00E-06	6.98E-06	1.82E-06	3.33E-06
T21B4.1	1	3	5	4	1.71E-04	1.37E-04	1.12E-04	1.78E-04
T21B4.10	8	12	17	10	1.80E-04	1.44E-04	1.18E-04	1.87E-04
T21B4.12	6	5	10	4	2.80E-06	2.65E-06	1.82E-06	2.25E-06
T21B4.14	3	5	11	2	2.80E-06	2.65E-06	1.82E-06	2.25E-06
T21B4.15	1	5	9	3	2.80E-06	2.65E-06	1.82E-06	2.25E-06
T21B4.17	19	25	24	31	2.80E-06	2.65E-06	1.82E-06	2.25E-06
T21B4.2	89	104	61	77	2.80E-06	2.65E-06	1.82E-06	2.25E-06
T21B4.3	2	2	0	0	2.80E-06	2.65E-06	1.82E-06	2.25E-06
T21B4.4a	10	6	3	4	1.06E-05	1.17E-05	4.74E-06	7.38E-06
T21B4.4b	12	8	3	5	2.80E-06	2.65E-06	1.82E-06	2.25E-06
T21B4.5	3	6	7	2	2.80E-06	2.65E-06	1.82E-06	2.25E-06
T21B4.6	5	4	10	1	2.80E-06	2.65E-06	1.82E-06	2.25E-06
T21B4.7	1	3	7	3	2.80E-06	2.65E-06	1.82E-06	2.25E-06
T21B4.8	3	7	28	6	2.80E-06	2.65E-06	1.82E-06	2.25E-06
T21B4.9	3	7	5	6	2.80E-06	2.65E-06	1.82E-06	2.25E-06
T21B4.t1	0	0	1	0	2.80E-06	2.65E-06	2.02E-06	2.25E-06
T21B6.1.1	472	449	471	650	2.80E-06	2.65E-06	1.82E-06	2.25E-06
T21B6.1.2	462	444	457	645	2.80E-06	2.65E-06	1.82E-06	2.25E-06
T21B6.2	50	129	46	124	2.07E-05	1.86E-05	1.34E-05	2.29E-05
T21B6.3	294	333	269	340	2.10E-05	1.90E-05	1.35E-05	2.35E-05
T21B6.4	4	4	7	3	4.40E-06	1.07E-05	2.64E-06	8.77E-06
T21B6.5	5	4	7	1	1.16E-05	1.24E-05	6.89E-06	1.08E-05
T21C12.1a	53	131	44	87	2.80E-06	2.65E-06	1.82E-06	2.25E-06
T21C12.1b	24	53	19	31	2.80E-06	2.65E-06	1.82E-06	2.25E-06
T21C12.1d	21	61	17	48	2.80E-06	3.81E-06	1.82E-06	2.25E-06
T21C12.1e	21	62	16	49	2.80E-06	2.94E-06	1.82E-06	2.25E-06
T21C12.1f	15	61	18	39	2.80E-06	2.65E-06	1.82E-06	2.25E-06
T21C12.1g	4	2	3	3	2.80E-06	2.65E-06	1.82E-06	2.25E-06
T21C12.2	714	1345	820	1272	2.80E-06	4.02E-06	1.82E-06	2.25E-06
T21C12.3	10	19	11	8	2.80E-06	2.65E-06	1.82E-06	2.25E-06
T21C12.8	6	9	5	6	5.82E-05	1.04E-04	4.35E-05	8.32E-05
T21C9.1	264	328	501	607	2.80E-06	2.91E-06	1.82E-06	2.25E-06
T21C9.11	3	7	4	2	2.80E-06	2.65E-06	1.82E-06	2.25E-06
T21C9.12	832	696	994	1240	3.65E-05	4.29E-05	4.51E-05	6.74E-05
T21C9.13	557	587	988	898	2.80E-06	2.65E-06	1.82E-06	2.25E-06
T21C9.2	456	546	550	914	5.40E-05	4.27E-05	4.20E-05	6.46E-05
T21C9.3a.1	412	678	486	511	6.72E-05	6.69E-05	7.76E-05	8.71E-05
T21C9.3a.2	415	681	496	520	1.48E-05	1.68E-05	1.16E-05	2.39E-05
T21C9.3b	464	755	550	587	2.56E-05	3.98E-05	1.96E-05	2.55E-05
T21C9.4	599	443	432	400	2.55E-05	3.95E-05	1.98E-05	2.57E-05
T21C9.6	165	236	221	234	2.65E-05	4.07E-05	2.04E-05	2.69E-05
T21C9.7	4	6	7	4	1.39E-04	9.68E-05	6.51E-05	7.44E-05
T21C9.8	7	35	34	6	4.96E-06	6.69E-06	4.32E-06	5.62E-06
T21C9.9	32	56	31	25	2.80E-06	2.65E-06	1.82E-06	2.25E-06
T21D11.1	17	22	8	1	2.80E-06	6.43E-06	4.30E-06	2.25E-06
T21D12.11	7	12	12	9	2.80E-06	4.21E-06	1.82E-06	2.25E-06
T21D12.12	126	143	57	64	2.80E-06	2.65E-06	1.82E-06	2.25E-06
T21D12.3.1	218	285	307	280	2.80E-06	2.65E-06	1.82E-06	2.25E-06
T21D12.3.2	153	201	218	225	2.33E-05	2.50E-05	6.87E-06	9.51E-06
T21D12.4	198	290	176	279	2.30E-05	2.83E-05	2.10E-05	2.37E-05
T21D12.5	3	0	2	1	2.02E-05	2.51E-05	1.88E-05	2.39E-05

T21D12.7	6	8	8	9	1.78E-05	2.46E-05	1.03E-05	2.02E-05
T21D12.9a	76	169	62	158	2.80E-06	2.65E-06	1.82E-06	2.25E-06
T21D12.9b	42	71	34	42	2.80E-06	2.65E-06	1.82E-06	2.25E-06
T21D12.9c.1	63	138	58	139	3.05E-06	6.40E-06	1.82E-06	5.08E-06
T21D12.9c.2	60	135	55	135	2.80E-06	3.99E-06	1.82E-06	2.25E-06
T21D9.1	14	16	10	10	3.11E-06	6.43E-06	1.86E-06	5.51E-06
T21D9.2	2	2	0	2	3.00E-06	6.37E-06	1.82E-06	5.42E-06
T21E12.2	18	26	6	17	2.80E-06	2.65E-06	1.82E-06	2.25E-06
T21E12.3	1	2	2	2	2.80E-06	2.65E-06	1.82E-06	2.25E-06
T21E12.4	4021	4296	6224	7956	2.80E-06	2.65E-06	1.82E-06	2.25E-06
T21E12.5	7	16	6	5	2.80E-06	2.65E-06	1.82E-06	2.25E-06
T21E3.1	1893	1636	3544	3568	3.23E-05	3.26E-05	3.26E-05	5.14E-05
T21E3.2	6	12	6	4	2.80E-06	3.02E-06	1.82E-06	2.25E-06
T21E3.3	355	481	386	686	9.06E-05	7.40E-05	1.10E-04	1.37E-04
T21E8.1a	471	293	245	289	2.80E-06	2.65E-06	1.82E-06	2.25E-06
T21E8.1b	474	303	251	294	5.85E-06	7.49E-06	4.14E-06	9.09E-06
T21E8.2	316	207	176	192	1.43E-05	8.44E-06	4.85E-06	7.06E-06
T21E8.3	34	34	34	38	1.40E-05	8.46E-06	4.83E-06	6.99E-06
T21E8.4	3	12	3	2	9.32E-06	5.77E-06	3.39E-06	4.57E-06
T21F2.1a	11	17	7	6	2.80E-06	2.65E-06	1.82E-06	2.25E-06
T21F2.1b	6	16	6	3	2.80E-06	2.65E-06	1.82E-06	2.25E-06
T21F4.1	139	535	133	250	2.80E-06	2.65E-06	1.82E-06	2.25E-06
T21G5.1	29	59	26	34	2.80E-06	2.65E-06	1.82E-06	2.25E-06
T21G5.2	15	20	20	25	1.50E-05	5.44E-05	9.33E-06	2.16E-05
T21G5.3	2004	2105	1489	2919	2.80E-06	3.44E-06	1.82E-06	2.25E-06
T21G5.4	97	149	69	43	3.05E-06	3.86E-06	2.66E-06	4.09E-06
T21G5.5a	159	201	142	221	8.78E-05	8.71E-05	4.25E-05	1.03E-04
T21G5.5b	177	234	161	243	1.15E-05	1.67E-05	5.34E-06	4.09E-06
T21G5.5c	171	240	163	248	1.19E-05	1.42E-05	6.92E-06	1.33E-05
T21H3.1a	1764	2631	3066	3669	1.46E-05	1.83E-05	8.66E-06	1.61E-05
T21H3.1b	514	874	1029	1231	1.34E-05	1.78E-05	8.33E-06	1.57E-05
T21H3.2	57	135	34	36	2.13E-04	3.00E-04	2.41E-04	3.56E-04
T21H3.3.1	3298	2972	3187	3868	1.47E-04	2.35E-04	1.91E-04	2.82E-04
T21H3.3.2	1769	1685	1655	2636	2.80E-06	5.40E-06	1.82E-06	2.25E-06
T21H3.4	9	17	13	6	2.53E-04	2.16E-04	1.59E-04	2.39E-04
T21H3.5	394	314	346	135	2.12E-04	1.91E-04	1.29E-04	2.54E-04
T21H8.2	2	6	3	7	2.80E-06	2.65E-06	1.82E-06	2.25E-06
T21H8.3	6	9	7	4	3.83E-05	2.88E-05	2.19E-05	1.05E-05
T21H8.4	6	6	5	2	2.80E-06	2.65E-06	1.82E-06	2.25E-06
T21H8.5	21	29	12	23	2.80E-06	2.65E-06	1.82E-06	2.25E-06
T22A3.3a	669	547	1058	1290	2.80E-06	2.65E-06	1.82E-06	2.25E-06
T22A3.3b.1	449	336	648	819	4.42E-06	5.77E-06	1.82E-06	3.89E-06
T22A3.3b.2	659	541	1068	1282	4.44E-05	3.43E-05	4.57E-05	6.87E-05
T22A3.3b.3	450	337	648	819	5.56E-05	3.93E-05	5.22E-05	8.15E-05
T22A3.3b.4	486	360	687	853	7.45E-05	5.78E-05	7.86E-05	1.16E-04
T22A3.3b.5	449	336	648	819	5.66E-05	4.01E-05	5.31E-05	8.28E-05
T22A3.3b.6	490	367	696	865	5.75E-05	4.03E-05	5.29E-05	8.11E-05
T22A3.4a	151	202	94	173	5.47E-05	3.87E-05	5.14E-05	8.02E-05
T22A3.4b	150	200	97	178	5.73E-05	4.06E-05	5.30E-05	8.13E-05
T22A3.5	464	433	499	725	1.04E-05	1.31E-05	4.21E-06	9.56E-06
T22A3.6	15	24	7	7	9.74E-06	1.23E-05	4.10E-06	9.29E-06
T22A3.8	255	293	203	533	1.98E-05	1.74E-05	1.38E-05	2.48E-05
T22A3.t1	0	1	0	1	2.80E-06	2.65E-06	1.82E-06	2.25E-06
T22A3.t2	0	0	0	1	3.02E-06	3.28E-06	1.82E-06	5.08E-06
T22A3.t3	0	0	0	1	2.80E-06	2.65E-06	1.82E-06	2.25E-06
T22A3.t4	0	0	0	1	2.80E-06	2.65E-06	1.82E-06	2.25E-06
T22B11.1	6	6	8	6	2.80E-06	2.65E-06	1.82E-06	2.25E-06
T22B11.2	40	40	22	22	2.80E-06	2.65E-06	1.82E-06	2.25E-06
T22B11.3	7	11	4	7	2.80E-06	2.65E-06	1.82E-06	2.25E-06

T22B11.4a	90	170	81	122	4.06E-06	3.84E-06	1.82E-06	2.25E-06
T22B11.4b	105	167	107	144	2.80E-06	2.65E-06	1.82E-06	2.25E-06
T22B11.5	3231	3121	3345	4988	5.15E-06	9.18E-06	3.01E-06	5.60E-06
T22B2.1	18	25	20	14	6.08E-06	9.13E-06	4.03E-06	6.70E-06
T22B2.2	6	4	11	2	1.03E-04	9.38E-05	6.92E-05	1.27E-04
T22B2.3	10	16	26	13	2.80E-06	2.65E-06	1.82E-06	2.25E-06
T22B2.4	64	116	57	82	2.80E-06	2.65E-06	1.82E-06	2.25E-06
T22B2.5	6	2	4	2	2.80E-06	2.65E-06	1.82E-06	2.25E-06
T22B2.6	5	11	0	4	5.63E-06	9.63E-06	3.26E-06	5.78E-06
T22B2.7	1	2	1	0	2.80E-06	2.65E-06	1.82E-06	2.25E-06
T22B3.1	17	31	10	10	2.80E-06	2.65E-06	1.82E-06	2.25E-06
T22B3.2a	119	261	107	96	2.80E-06	2.65E-06	1.82E-06	2.25E-06
T22B3.2b	119	256	93	94	2.80E-06	2.86E-06	1.82E-06	2.25E-06
T22B3.3	50	91	28	21	4.20E-06	8.73E-06	2.46E-06	2.72E-06
T22B7.1a.1	105	138	104	127	4.28E-06	8.70E-06	2.19E-06	2.72E-06
T22B7.1a.2	55	98	53	82	1.00E-05	1.72E-05	3.66E-06	3.40E-06
T22B7.1b.1	105	138	104	127	5.63E-06	6.98E-06	3.63E-06	5.47E-06
T22B7.1b.2	48	85	50	80	4.14E-06	6.96E-06	2.59E-06	4.95E-06
T22B7.1b.3	55	98	53	82	5.63E-06	6.98E-06	3.63E-06	5.47E-06
T22B7.1b.4	50	89	50	81	4.54E-06	7.59E-06	3.08E-06	6.07E-06
T22B7.1c.1	105	138	104	127	4.14E-06	6.96E-06	2.59E-06	4.95E-06
T22B7.1c.2	55	98	53	82	4.42E-06	7.43E-06	2.88E-06	5.74E-06
T22B7.1d	42	67	41	69	5.63E-06	6.98E-06	3.63E-06	5.47E-06
T22B7.3	23	15	15	5	4.14E-06	6.96E-06	2.59E-06	4.95E-06
T22B7.4	37	62	23	33	4.62E-06	6.96E-06	2.93E-06	6.09E-06
T22B7.5	6	9	17	5	2.80E-06	2.65E-06	1.82E-06	2.25E-06
T22B7.7	193	222	161	303	2.80E-06	3.86E-06	1.82E-06	2.25E-06
T22B7.8	6	3	2	1	2.80E-06	2.65E-06	1.82E-06	2.25E-06
T22C1.10a	524	548	654	1023	1.66E-05	1.80E-05	9.00E-06	2.09E-05
T22C1.10b	520	546	665	1028	3.56E-06	2.65E-06	1.82E-06	2.25E-06
T22C1.11	70	80	59	88	1.44E-05	1.43E-05	1.17E-05	2.27E-05
T22C1.12	2	2	1	1	1.48E-05	1.47E-05	1.23E-05	2.35E-05
T22C1.2	90	86	137	196	5.63E-06	6.06E-06	3.08E-06	5.67E-06
T22C1.3	314	372	354	517	2.80E-06	2.65E-06	1.82E-06	2.25E-06
T22C1.4	91	91	198	99	1.81E-05	1.64E-05	1.80E-05	3.17E-05
T22C1.5	72	74	146	192	2.67E-05	2.98E-05	1.96E-05	3.52E-05
T22C1.6	348	401	334	488	3.50E-05	3.31E-05	4.96E-05	3.06E-05
T22C1.7	123	154	127	171	1.60E-05	1.55E-05	2.11E-05	3.43E-05
T22C1.8	51	96	37	40	2.56E-05	2.79E-05	1.60E-05	2.89E-05
T22C1.9	28	53	15	7	4.51E-06	5.34E-06	3.02E-06	5.04E-06
T22C1.t1	0	0	1	0	2.91E-06	5.16E-06	1.82E-06	2.25E-06
T22C8.1	7	6	0	1	5.01E-06	8.97E-06	1.82E-06	2.25E-06
T22C8.2	60	73	54	122	2.80E-06	2.65E-06	1.82E-06	2.25E-06
T22C8.3	21	31	26	25	2.80E-06	2.65E-06	1.82E-06	2.25E-06
T22C8.4	9	17	25	15	4.17E-06	4.79E-06	2.44E-06	6.81E-06
T22C8.5	4	8	6	6	2.80E-06	3.41E-06	1.97E-06	2.34E-06
T22C8.6	28	29	8	17	2.80E-06	2.65E-06	2.08E-06	2.25E-06
T22C8.7	66	74	33	16	2.80E-06	2.65E-06	1.82E-06	2.25E-06
T22C8.8	50	71	42	63	3.47E-06	3.39E-06	1.82E-06	2.25E-06
T22D1.1	7	7	6	5	4.03E-06	4.29E-06	1.82E-06	2.25E-06
T22D1.10	738	813	859	959	6.02E-06	8.09E-06	3.30E-06	6.09E-06
T22D1.11	23	19	25	25	2.80E-06	2.65E-06	1.82E-06	2.25E-06
T22D1.12	13	36	15	21	5.52E-05	5.75E-05	4.19E-05	5.77E-05
T22D1.2	2	9	24	14	2.80E-06	2.65E-06	1.82E-06	2.25E-06
T22D1.3a	957	911	1019	1302	2.80E-06	3.23E-06	1.82E-06	2.25E-06
T22D1.3b.1	981	930	1053	1318	2.80E-06	2.65E-06	1.93E-06	2.25E-06
T22D1.3b.2	944	900	996	1273	5.81E-05	5.22E-05	4.02E-05	6.34E-05
T22D1.4	3412	2966	3388	4389	5.78E-05	5.18E-05	4.04E-05	6.24E-05
T22D1.5.1	729	863	1440	1406	7.59E-05	6.83E-05	5.21E-05	8.22E-05

T22D1.5.2	737	873	1549	1415	1.96E-04	1.61E-04	1.27E-04	2.02E-04
T22D1.6	1	3	1	3	6.37E-05	7.13E-05	8.19E-05	9.87E-05
T22D1.8	9	14	8	6	6.86E-05	7.67E-05	9.38E-05	1.06E-04
T22D1.9.1	3045	2858	4401	5822	2.80E-06	2.65E-06	1.82E-06	2.25E-06
T22D1.9.2	2783	2638	4058	5513	2.80E-06	2.65E-06	1.82E-06	2.25E-06
T22D2.1	135	186	93	126	1.08E-04	9.55E-05	1.01E-04	1.65E-04
T22E5.1a	10	9	10	8	1.05E-04	9.45E-05	1.00E-04	1.68E-04
T22E5.1b	8	8	10	7	4.84E-06	6.30E-06	2.17E-06	3.62E-06
T22E5.2	23	24	38	44	5.40E-06	4.60E-06	3.52E-06	3.49E-06
T22E5.3	14	5	5	3	4.28E-06	4.05E-06	3.48E-06	3.01E-06
T22E5.5.1	2330	2973	2439	2813	2.80E-06	2.65E-06	1.82E-06	2.45E-06
T22E5.5.2	1926	2345	1989	2409	2.80E-06	2.65E-06	1.82E-06	2.25E-06
T22E5.6	9	8	9	15	1.63E-04	1.96E-04	1.11E-04	1.58E-04
T22E5.7	0	0	0	1	1.74E-04	2.01E-04	1.17E-04	1.75E-04
T22E7.1a	116	163	69	137	2.80E-06	2.65E-06	1.82E-06	2.25E-06
T22E7.1b	110	154	81	142	2.80E-06	2.65E-06	1.82E-06	2.25E-06
T22E7.2	6	2	2	2	1.07E-05	1.42E-05	4.14E-06	1.01E-05
T22F3.1	4	7	7	4	7.50E-06	9.95E-06	3.61E-06	7.78E-06
T22F3.10	4	7	3	6	2.80E-06	2.65E-06	1.82E-06	2.25E-06
T22F3.11a	9	4	6	5	2.80E-06	2.65E-06	1.82E-06	2.25E-06
T22F3.11b	8	1	6	2	2.80E-06	2.65E-06	1.82E-06	2.25E-06
T22F3.12	4	2	0	2	2.80E-06	2.65E-06	1.82E-06	2.25E-06
T22F3.2a	378	333	470	573	2.80E-06	2.65E-06	1.82E-06	2.25E-06
T22F3.2b	283	260	363	465	2.80E-06	2.65E-06	1.82E-06	2.25E-06
T22F3.3a	6029	4867	6882	9427	3.08E-05	2.56E-05	2.49E-05	3.75E-05
T22F3.3b.1	5009	4152	5986	8271	2.86E-05	2.48E-05	2.39E-05	3.78E-05
T22F3.3b.2	4980	4129	5962	8255	2.24E-04	1.71E-04	1.66E-04	2.81E-04
T22F3.4.1	5072	5182	12326	5326	2.16E-04	1.69E-04	1.68E-04	2.87E-04
T22F3.4.2	4341	4456	9474	4779	2.15E-04	1.68E-04	1.67E-04	2.86E-04
T22F3.5	3	3	3	1	8.63E-04	8.33E-04	1.37E-03	7.28E-04
T22F3.6	2	3	5	2	8.14E-04	7.90E-04	1.16E-03	7.20E-04
T22F3.7	6	15	7	6	2.80E-06	2.65E-06	1.82E-06	2.25E-06
T22F3.8	3	11	6	9	2.80E-06	2.65E-06	1.82E-06	2.25E-06
T22F7.1	57	79	61	97	2.80E-06	2.65E-06	1.82E-06	2.25E-06
T22F7.3	25	45	17	13	2.80E-06	2.65E-06	1.82E-06	2.25E-06
T22F7.4	19	21	14	5	3.70E-06	4.84E-06	2.57E-06	5.04E-06
T22F7.5	14	20	5	10	2.80E-06	2.65E-06	1.82E-06	2.25E-06
T22F7.t1	0	0	2	0	3.53E-06	3.68E-06	1.82E-06	2.25E-06
T22G5.1	5	11	7	0	2.80E-06	2.65E-06	1.82E-06	2.25E-06
T22G5.2	125	202	122	52	2.80E-06	2.65E-06	2.00E-06	2.25E-06
T22G5.3	31	43	34	49	2.80E-06	2.65E-06	1.82E-06	2.25E-06
T22G5.4	4	7	9	4	3.38E-05	5.16E-05	2.15E-05	1.13E-05
T22G5.5.1	261	563	274	642	3.72E-06	4.87E-06	2.66E-06	4.72E-06
T22G5.5.2	215	448	207	511	2.80E-06	2.65E-06	1.82E-06	2.25E-06
T22G5.6	12	23	32	14	1.50E-05	3.05E-05	1.02E-05	2.96E-05
T22G5.7	0	2	1	0	1.55E-05	3.06E-05	9.75E-06	2.97E-05
T22H2.1	1	4	1	4	5.82E-06	1.05E-05	1.01E-05	5.44E-06
T22H2.2	9	6	2	3	2.80E-06	2.65E-06	1.82E-06	2.25E-06
T22H2.3	2	1	3	6	2.80E-06	2.65E-06	1.82E-06	2.25E-06
T22H2.4	8	8	1	2	2.80E-06	2.65E-06	1.82E-06	2.25E-06
T22H2.5a.1	65	84	55	92	2.80E-06	2.65E-06	1.82E-06	2.25E-06
T22H2.5a.2	48	56	32	70	3.25E-06	3.07E-06	1.82E-06	2.25E-06
T22H2.5b	58	74	39	85	5.85E-06	7.14E-06	3.23E-06	6.66E-06
T22H2.6a	157	191	182	159	6.10E-06	6.74E-06	2.66E-06	7.17E-06
T22H2.6b	148	173	170	144	5.80E-06	6.98E-06	2.53E-06	6.84E-06
T22H6.1	2	0	3	0	1.51E-05	1.73E-05	1.14E-05	1.23E-05
T22H6.2b	356	564	180	207	1.49E-05	1.64E-05	1.11E-05	1.16E-05
T22H6.3	8	12	8	5	2.80E-06	2.65E-06	1.82E-06	2.25E-06
T22H6.4	7	5	14	5	1.64E-05	2.46E-05	5.41E-06	7.69E-06

T22H6.5	15	25	22	3	2.80E-06	2.65E-06	1.82E-06	2.25E-06
T22H6.7	25	30	23	12	2.80E-06	2.65E-06	1.82E-06	2.25E-06
T22H9.1	157	194	145	202	3.44E-06	5.45E-06	3.30E-06	2.25E-06
T22H9.2a	286	262	426	604	4.28E-06	4.87E-06	2.57E-06	2.25E-06
T22H9.2b	266	244	345	542	1.22E-05	1.42E-05	7.31E-06	1.25E-05
T22H9.3	58	93	42	27	1.21E-05	1.05E-05	1.18E-05	2.06E-05
T22H9.4	18	15	30	15	1.38E-05	1.20E-05	1.16E-05	2.26E-05
T23B12.1	196	193	206	247	2.80E-06	3.09E-06	1.82E-06	2.25E-06
T23B12.11	22	39	15	19	2.80E-06	2.65E-06	1.82E-06	2.25E-06
T23B12.2	177	219	231	225	2.74E-05	2.55E-05	1.87E-05	2.77E-05
T23B12.4	1072	789	1093	1626	6.27E-06	1.05E-05	2.79E-06	4.34E-06
T23B12.5	5	12	1	4	1.95E-05	2.29E-05	1.66E-05	2.00E-05
T23B12.6	454	475	809	986	4.34E-05	3.02E-05	2.88E-05	5.29E-05
T23B12.7	314	406	270	331	2.80E-06	2.65E-06	1.82E-06	2.25E-06
T23B3.1	377	504	516	520	2.38E-05	2.35E-05	2.76E-05	4.15E-05
T23B3.2	137	210	150	94	3.64E-05	4.44E-05	2.04E-05	3.08E-05
T23B3.3	5	9	12	7	3.02E-05	3.82E-05	2.69E-05	3.35E-05
T23B3.4	10	15	2	4	2.95E-05	4.26E-05	2.10E-05	1.62E-05
T23B3.5	127	199	72	54	2.80E-06	2.65E-06	1.82E-06	2.25E-06
T23B3.6	14	13	10	6	2.80E-06	2.65E-06	1.82E-06	2.25E-06
T23B5.1a.1	897	883	1172	1576	1.97E-05	2.92E-05	7.27E-06	6.75E-06
T23B5.1b	456	422	595	858	2.80E-06	2.65E-06	1.82E-06	2.25E-06
T23B5.1c	303	257	391	539	4.12E-05	3.83E-05	3.51E-05	5.82E-05
T23B5.3a	740	553	997	1253	4.89E-05	4.28E-05	4.15E-05	7.39E-05
T23B5.3b	275	226	319	450	5.14E-05	4.12E-05	4.32E-05	7.35E-05
T23B5.3c.1	379	269	456	599	2.93E-05	2.07E-05	2.57E-05	3.98E-05
T23B5.3c.2	312	215	356	494	1.83E-05	1.42E-05	1.38E-05	2.41E-05
T23B5.4	37	34	47	27	3.73E-05	2.50E-05	2.92E-05	4.74E-05
T23B7.1	51	92	109	35	4.35E-05	2.83E-05	3.23E-05	5.53E-05
T23B7.2	14	19	18	6	1.10E-05	9.52E-06	9.06E-06	6.43E-06
T23B7.3	12	10	12	6	1.72E-05	2.93E-05	2.39E-05	9.49E-06
T23C6.1	43	51	47	60	3.08E-06	3.97E-06	2.59E-06	2.25E-06
T23C6.3.1	92	213	113	138	3.64E-06	2.86E-06	2.37E-06	2.25E-06
T23C6.3.2	74	192	105	128	2.94E-06	3.28E-06	2.08E-06	3.28E-06
T23C6.4	35	23	18	31	5.46E-06	1.20E-05	4.37E-06	6.59E-06
T23C6.5	5	14	8	7	5.15E-06	1.27E-05	4.77E-06	7.17E-06
T23D5.1	4	5	5	4	3.22E-06	2.65E-06	1.82E-06	2.29E-06
T23D5.10	3	7	2	0	2.80E-06	2.65E-06	1.82E-06	2.25E-06
T23D5.11	2	3	3	1	2.80E-06	2.65E-06	1.82E-06	2.25E-06
T23D5.12	3	7	2	0	2.80E-06	2.65E-06	1.82E-06	2.25E-06
T23D5.2	15	29	10	12	2.80E-06	2.65E-06	1.82E-06	2.25E-06
T23D5.3	1	1	0	1	2.80E-06	2.65E-06	1.82E-06	2.25E-06
T23D5.5	1	1	1	1	2.80E-06	2.65E-06	1.82E-06	2.25E-06
T23D5.6	5	1	3	3	2.80E-06	2.65E-06	1.82E-06	2.25E-06
T23D5.7	4	2	3	0	2.80E-06	2.65E-06	1.82E-06	2.25E-06
T23D5.8	0	0	1	0	2.80E-06	2.65E-06	1.82E-06	2.25E-06
T23D5.9	1	4	2	4	2.80E-06	2.65E-06	1.82E-06	2.25E-06
T23D8.1	356	548	865	1326	2.80E-06	2.65E-06	1.82E-06	2.25E-06
T23D8.2	6	17	12	6	2.80E-06	2.65E-06	1.82E-06	2.25E-06
T23D8.3	525	622	588	817	2.17E-05	3.15E-05	3.43E-05	6.49E-05
T23D8.4.1	3143	3009	3502	5171	2.80E-06	2.65E-06	1.82E-06	2.25E-06
T23D8.4.2	2844	2744	3224	4792	4.03E-05	4.51E-05	2.94E-05	5.04E-05
T23D8.5	22	31	173	62	1.17E-04	1.06E-04	8.47E-05	1.54E-04
T23D8.6	84	61	410	115	1.18E-04	1.08E-04	8.71E-05	1.60E-04
T23D8.7	693	642	1135	1445	7.90E-06	1.05E-05	4.04E-05	1.79E-05
T23D8.8	15	20	31	18	1.93E-05	1.33E-05	6.14E-05	2.13E-05
T23D8.9a	313	233	332	519	2.88E-05	2.52E-05	3.06E-05	4.82E-05
T23D8.9b	92	63	85	163	2.80E-06	2.65E-06	1.82E-06	2.25E-06
T23E1.1	8	13	16	9	1.44E-05	1.01E-05	9.93E-06	1.92E-05

T23E1.2	180	182	156	258	1.18E-05	7.62E-06	7.07E-06	1.67E-05
T23E1.3	1	12	10	5	2.80E-06	2.65E-06	1.82E-06	2.25E-06
T23E7.2a	1010	881	1022	1444	1.32E-05	1.26E-05	7.45E-06	1.52E-05
T23E7.2b	1276	1149	1244	1769	2.80E-06	2.65E-06	1.82E-06	2.25E-06
T23E7.2c	862	771	901	1291	4.03E-05	3.32E-05	2.65E-05	4.63E-05
T23E7.2d	418	433	359	604	4.70E-05	4.00E-05	2.98E-05	5.23E-05
T23E7.2e	1307	1185	1257	1834	3.61E-05	3.06E-05	2.46E-05	4.35E-05
T23E7.4	84	80	78	67	5.99E-05	5.87E-05	3.35E-05	6.96E-05
T23E7.5	1	2	0	2	4.52E-05	3.88E-05	2.83E-05	5.10E-05
T23E7.6	5	12	3	2	1.64E-05	1.48E-05	9.93E-06	1.05E-05
T23F1.1	4	3	6	2	2.80E-06	2.65E-06	1.82E-06	2.25E-06
T23F1.2	27	27	20	11	2.80E-06	6.06E-06	1.82E-06	2.25E-06
T23F1.3	3	5	2	1	2.80E-06	2.65E-06	1.82E-06	2.25E-06
T23F1.4	6	2	1	2	4.68E-06	4.42E-06	2.26E-06	2.25E-06
T23F1.5	21	24	15	24	2.80E-06	2.65E-06	1.82E-06	2.25E-06
T23F1.6	14	16	7	2	2.80E-06	2.65E-06	1.82E-06	2.25E-06
T23F1.7a	34	62	34	44	2.80E-06	2.65E-06	1.82E-06	2.25E-06
T23F1.7b	34	58	34	40	2.80E-06	2.65E-06	1.82E-06	2.25E-06
T23F1.t1	1	0	3	0	2.80E-06	2.65E-06	1.82E-06	2.25E-06
T23F11.1.1	655	1086	635	1187	2.80E-06	2.65E-06	1.82E-06	2.25E-06
T23F11.1.2	388	617	396	699	2.80E-06	2.65E-06	2.99E-06	2.25E-06
T23F11.2	58	123	63	27	3.94E-05	6.16E-05	2.48E-05	5.73E-05
T23F11.4	41	69	35	57	4.04E-05	6.07E-05	2.68E-05	5.85E-05
T23F11.5	6	5	7	9	7.76E-06	1.55E-05	5.47E-06	2.90E-06
T23F11.6	5	18	16	14	3.67E-06	5.82E-06	2.04E-06	4.09E-06
T23F2.1.1	259	321	117	115	2.80E-06	2.65E-06	1.82E-06	2.25E-06
T23F2.2a	64	102	38	86	2.80E-06	3.44E-06	2.11E-06	2.27E-06
T23F2.2b	81	111	58	93	1.36E-05	1.59E-05	3.99E-06	4.86E-06
T23F2.3	76	92	196	49	2.80E-06	2.65E-06	1.82E-06	2.25E-06
T23F2.4	1	1	1	2	2.80E-06	2.65E-06	1.82E-06	2.25E-06
T23F2.5	385	560	1047	364	2.89E-05	3.30E-05	4.84E-05	1.49E-05
T23F4.1	2	7	1	1	2.80E-06	2.65E-06	1.82E-06	2.25E-06
T23F4.2	38	63	33	62	1.09E-04	1.49E-04	1.92E-04	8.25E-05
T23F4.3	5	7	4	1	2.80E-06	2.65E-06	1.82E-06	2.25E-06
T23F4.4	18	16	22	6	5.01E-06	7.88E-06	2.84E-06	6.59E-06
T23F4.5	1	2	0	0	2.80E-06	2.65E-06	1.82E-06	2.25E-06
T23F6.1	15	21	5	6	2.80E-06	2.65E-06	1.82E-06	2.25E-06
T23F6.2	1	0	2	0	2.80E-06	2.65E-06	1.82E-06	2.25E-06
T23F6.3	17	30	11	10	3.47E-06	4.60E-06	1.82E-06	2.25E-06
T23F6.4.1	496	575	453	752	2.80E-06	2.65E-06	1.82E-06	2.25E-06
T23F6.4.2	459	534	428	733	2.80E-06	3.54E-06	1.82E-06	2.25E-06
T23F6.5	13	23	6	8	2.00E-05	2.18E-05	1.19E-05	2.43E-05
T23G11.1	117	186	78	64	1.96E-05	2.15E-05	1.19E-05	2.51E-05
T23G11.10	11	2	2	2	2.80E-06	2.65E-06	1.82E-06	2.25E-06
T23G11.3	7199	5449	11451	14285	2.58E-05	3.88E-05	1.12E-05	1.14E-05
T23G11.4	154	180	150	197	2.80E-06	2.65E-06	1.82E-06	2.25E-06
T23G11.5	426	404	602	731	5.73E-04	4.10E-04	5.93E-04	9.13E-04
T23G11.6b	231	417	144	218	2.23E-05	2.46E-05	1.41E-05	2.29E-05
T23G11.7a	103	105	136	181	2.34E-05	2.09E-05	2.15E-05	3.22E-05
T23G11.7b	448	477	583	766	1.13E-05	1.94E-05	4.61E-06	8.61E-06
T23G4.1.1	99	63	94	104	9.97E-06	9.60E-06	8.58E-06	1.41E-05
T23G4.1.2	93	59	89	101	4.94E-05	4.97E-05	4.18E-05	6.78E-05
T23G4.2	1	1	0	1	6.64E-06	3.99E-06	4.10E-06	5.60E-06
T23G4.3	100	110	218	239	6.41E-06	3.84E-06	3.99E-06	5.58E-06
T23G4.4	14	16	18	11	2.80E-06	2.65E-06	1.82E-06	2.25E-06
T23G4.5	2	6	4	4	7.39E-06	7.67E-06	1.05E-05	1.42E-05
T23G5.1.1	1905	1953	3007	3756	2.80E-06	2.65E-06	1.82E-06	2.25E-06
T23G5.1.2	1948	1995	3081	3794	2.80E-06	2.65E-06	1.82E-06	2.25E-06
T23G5.1.3	1849	1877	2900	3663	8.26E-05	8.00E-05	8.48E-05	1.31E-04

T23G5.2a.1	759	727	1185	1322	8.67E-05	8.39E-05	8.92E-05	1.36E-04
T23G5.2a.2	714	680	1101	1253	8.74E-05	8.38E-05	8.92E-05	1.39E-04
T23G5.2b.1	266	257	337	356	3.43E-05	3.11E-05	3.49E-05	4.80E-05
T23G5.2b.2	233	222	282	333	3.40E-05	3.06E-05	3.41E-05	4.79E-05
T23G5.3	3	5	5	4	3.14E-05	2.86E-05	2.59E-05	3.37E-05
T23G5.5	32	43	31	25	3.62E-05	3.26E-05	2.85E-05	4.15E-05
T23G5.6	65	89	51	79	2.80E-06	2.65E-06	1.82E-06	2.25E-06
T23G7.1	814	693	1091	1375	2.80E-06	2.65E-06	1.82E-06	2.25E-06
T23G7.2a	149	204	186	259	5.74E-06	7.41E-06	2.93E-06	5.60E-06
T23G7.3	396	510	213	483	4.15E-05	3.34E-05	3.62E-05	5.63E-05
T23G7.4	467	513	523	870	1.16E-05	1.51E-05	9.46E-06	1.63E-05
T23H2.1	2723	2460	3356	4637	3.73E-05	4.53E-05	1.30E-05	3.65E-05
T23H2.2	147	264	155	147	1.86E-05	1.93E-05	1.36E-05	2.79E-05
T23H2.3	790	576	829	1215	5.18E-05	4.42E-05	4.15E-05	7.08E-05
T23H2.4	13	16	14	12	1.44E-05	2.44E-05	9.86E-06	1.15E-05
T23H4.1	2	14	4	6	2.92E-05	2.01E-05	1.99E-05	3.61E-05
T23H4.2.1	59	121	56	112	2.80E-06	2.65E-06	1.82E-06	2.25E-06
T23H4.2.2	50	104	46	98	2.80E-06	2.65E-06	1.82E-06	2.25E-06
T23H4.3	12	28	10	22	4.96E-06	9.60E-06	3.06E-06	7.56E-06
T24A11.1a	1740	1683	2341	3190	4.73E-06	9.28E-06	2.82E-06	7.44E-06
T24A11.1b	1874	1755	2503	3540	2.80E-06	2.72E-06	1.82E-06	2.25E-06
T24A11.2	14	15	24	21	5.48E-05	5.01E-05	4.80E-05	8.07E-05
T24A11.3	160	231	91	172	5.33E-05	4.71E-05	4.63E-05	8.09E-05
T24A6.1	35	36	16	23	2.80E-06	2.65E-06	1.82E-06	2.25E-06
T24A6.10	4	3	2	4	1.12E-05	1.53E-05	4.15E-06	9.69E-06
T24A6.11	7	8	12	3	1.35E-05	1.31E-05	4.01E-06	7.11E-06
T24A6.12	13	13	8	5	2.80E-06	2.65E-06	1.82E-06	2.25E-06
T24A6.13	6	9	10	3	2.80E-06	2.65E-06	1.82E-06	2.25E-06
T24A6.14	8	9	6	4	2.80E-06	2.65E-06	1.82E-06	2.25E-06
T24A6.15	4	4	5	1	2.80E-06	2.65E-06	1.82E-06	2.25E-06
T24A6.16	2	2	2	2	2.80E-06	2.65E-06	1.82E-06	2.25E-06
T24A6.17	41	27	22	26	2.80E-06	2.65E-06	1.82E-06	2.25E-06
T24A6.18	6	5	3	4	2.80E-06	2.65E-06	1.82E-06	2.25E-06
T24A6.19	3	9	6	4	9.32E-06	5.82E-06	3.26E-06	4.75E-06
T24A6.2	6	2	11	2	2.80E-06	2.65E-06	1.82E-06	2.25E-06
T24A6.20	44	64	60	73	2.80E-06	2.65E-06	1.82E-06	2.25E-06
T24A6.21	7	4	13	1	2.80E-06	2.65E-06	1.82E-06	2.25E-06
T24A6.3	0	1	3	2	4.93E-06	6.80E-06	4.37E-06	6.59E-06
T24A6.4	5	7	9	3	4.37E-06	2.65E-06	5.27E-06	2.25E-06
T24A6.5	3	1	0	0	2.80E-06	2.65E-06	1.82E-06	2.25E-06
T24A6.6	5	7	5	5	2.80E-06	2.65E-06	1.82E-06	2.25E-06
T24A6.7	4	1	8	0	2.80E-06	2.65E-06	1.82E-06	2.25E-06
T24A6.8	3	7	5	2	2.80E-06	2.65E-06	1.82E-06	2.25E-06
T24A6.9	128	250	1213	766	2.80E-06	2.65E-06	1.82E-06	2.25E-06
T24B1.1	383	531	333	567	2.80E-06	2.65E-06	1.82E-06	2.25E-06
T24B8.1.1	6487	7025	15437	4212	1.73E-05	3.20E-05	1.07E-04	8.32E-05
T24B8.1.2	5176	5677	11519	3696	2.21E-05	2.90E-05	1.25E-05	2.63E-05
T24B8.1.3	5265	5773	12053	3815	1.54E-03	1.58E-03	2.39E-03	8.04E-04
T24B8.2	231	361	290	522	1.30E-03	1.34E-03	1.88E-03	7.44E-04
T24B8.3a.1	243	297	224	185	1.33E-03	1.37E-03	1.97E-03	7.71E-04
T24B8.3a.2	174	222	166	130	2.09E-05	3.09E-05	1.71E-05	3.80E-05
T24B8.3b.1	184	220	154	142	3.12E-05	3.60E-05	1.87E-05	1.91E-05
T24B8.3b.2	163	197	146	119	3.07E-05	3.70E-05	1.91E-05	1.84E-05
T24B8.4	251	311	183	363	2.41E-05	2.72E-05	1.31E-05	1.49E-05
T24B8.5	227	204	1743	100	2.17E-05	2.48E-05	1.27E-05	1.28E-05
T24B8.6	17	14	19	14	7.00E-06	8.20E-06	3.33E-06	8.14E-06
T24B8.7a	1626	1332	3292	4247	1.06E-04	8.99E-05	5.29E-04	3.75E-05
T24B8.7b	1636	1330	3301	4257	2.91E-06	2.65E-06	2.13E-06	2.25E-06
T24C12.1	10	10	6	4	2.08E-05	1.61E-05	2.74E-05	4.36E-05

T24C12.2	15	19	25	19	2.09E-05	1.60E-05	2.74E-05	4.37E-05
T24C12.3	100	174	96	159	2.80E-06	2.65E-06	1.82E-06	2.25E-06
T24C12.4	17	27	17	11	2.80E-06	2.65E-06	1.82E-06	2.25E-06
T24C2.1	18	23	17	5	5.35E-06	8.81E-06	3.35E-06	6.84E-06
T24C2.2	14	18	24	6	2.91E-06	4.34E-06	1.90E-06	2.25E-06
T24C2.3	2	3	1	2	2.80E-06	2.65E-06	1.82E-06	2.25E-06
T24C2.4	7	5	0	3	2.80E-06	2.91E-06	2.66E-06	2.25E-06
T24C2.5	13	22	13	15	2.80E-06	2.65E-06	1.82E-06	2.25E-06
T24C4.1	670	737	744	970	2.80E-06	2.65E-06	1.82E-06	2.25E-06
T24C4.2	13	17	37	44	2.80E-06	4.36E-06	1.82E-06	2.52E-06
T24C4.3	1	1	0	0	5.56E-05	5.78E-05	4.02E-05	6.46E-05
T24C4.4	18	19	18	19	2.80E-06	2.65E-06	2.11E-06	3.10E-06
T24C4.5	198	162	281	311	2.80E-06	2.65E-06	1.82E-06	2.25E-06
T24C4.6a	1535	1491	2088	2879	4.31E-06	4.29E-06	2.81E-06	3.64E-06
T24C4.6b	1521	1466	2063	2864	1.83E-05	1.42E-05	1.69E-05	2.31E-05
T24C4.7	304	328	459	558	5.28E-05	4.85E-05	4.68E-05	7.96E-05
T24C4.8	1	11	7	4	5.43E-05	4.94E-05	4.79E-05	8.21E-05
T24C4.9	6	23	2	4	1.38E-05	1.40E-05	1.35E-05	2.03E-05
T24D1.1a	1141	1035	1385	1918	2.80E-06	2.65E-06	1.82E-06	2.25E-06
T24D1.1b	1667	1565	1979	2691	2.80E-06	7.64E-06	1.82E-06	2.25E-06
T24D1.2.1	389	433	612	603	5.95E-05	5.10E-05	4.70E-05	8.04E-05
T24D1.2.2	354	403	515	560	5.82E-05	5.16E-05	4.49E-05	7.54E-05
T24D1.3	762	630	1310	1110	2.77E-05	2.91E-05	2.84E-05	3.45E-05
T24D1.4	237	197	276	349	2.76E-05	2.97E-05	2.62E-05	3.51E-05
T24D1.5	255	277	306	328	6.80E-05	5.31E-05	7.60E-05	7.95E-05
T24D11.1	86	96	55	100	2.02E-05	1.58E-05	1.53E-05	2.38E-05
T24D3.1	3	1	3	3	2.21E-05	2.26E-05	1.72E-05	2.28E-05
T24D5.1	3	9	4	0	7.00E-06	7.38E-06	2.92E-06	6.54E-06
T24D5.2	25	37	47	47	2.80E-06	2.65E-06	1.82E-06	2.25E-06
T24D5.3	3	3	6	5	2.80E-06	2.65E-06	1.82E-06	2.25E-06
T24D5.4	1	0	1	0	2.80E-06	2.65E-06	1.86E-06	2.29E-06
T24D5.5	17	25	20	47	2.80E-06	2.65E-06	1.82E-06	2.25E-06
T24D5.6	9	2	11	9	2.80E-06	2.65E-06	1.82E-06	2.25E-06
T24D8.1	9	15	5	12	2.80E-06	2.65E-06	1.82E-06	3.44E-06
T24D8.2	20	17	5	4	2.80E-06	2.65E-06	1.82E-06	2.25E-06
T24D8.3	1	1	2	1	2.80E-06	2.65E-06	1.82E-06	2.25E-06
T24D8.4	0	1	2	0	9.30E-06	7.46E-06	1.82E-06	2.25E-06
T24D8.5	5	12	8	6	2.80E-06	2.65E-06	1.82E-06	2.25E-06
T24D8.6	0	1	1	2	2.80E-06	2.65E-06	1.82E-06	2.25E-06
T24E12.1	18	31	42	31	2.80E-06	2.65E-06	1.82E-06	2.25E-06
T24E12.10	8	16	24	21	2.80E-06	2.65E-06	1.82E-06	2.25E-06
T24E12.11	30	25	38	36	2.80E-06	2.65E-06	2.00E-06	2.25E-06
T24E12.12	1	0	0	1	2.80E-06	2.65E-06	1.82E-06	2.25E-06
T24E12.2	2	2	6	5	4.37E-06	3.44E-06	3.61E-06	4.23E-06
T24E12.3	6	9	11	2	2.80E-06	2.65E-06	1.82E-06	2.25E-06
T24E12.4	2	3	1	2	2.80E-06	2.65E-06	1.82E-06	2.25E-06
T24E12.5	20	29	39	16	2.80E-06	2.65E-06	1.82E-06	2.25E-06
T24E12.6	2	3	7	2	2.80E-06	2.65E-06	1.82E-06	2.25E-06
T24E12.7	3	3	2	1	2.80E-06	2.65E-06	1.82E-06	2.25E-06
T24E12.8	2	8	6	4	2.80E-06	2.65E-06	1.82E-06	2.25E-06
T24E12.9	55	58	70	78	2.80E-06	2.65E-06	1.82E-06	2.25E-06
T24F1.2	703	587	1243	1424	2.80E-06	2.65E-06	1.82E-06	2.25E-06
T24F1.3a	62	99	79	92	2.80E-06	2.72E-06	2.26E-06	3.10E-06
T24F1.3b.1	59	90	72	84	3.81E-05	3.01E-05	4.39E-05	6.21E-05
T24F1.3b.2	56	88	72	83	3.50E-06	5.26E-06	2.90E-06	4.16E-06
T24F1.3b.3	60	91	76	87	3.78E-06	5.45E-06	3.01E-06	4.34E-06
T24F1.4	2	4	6	4	3.64E-06	5.42E-06	3.04E-06	4.34E-06
T24F1.5	28	35	80	19	3.61E-06	5.16E-06	2.97E-06	4.18E-06
T24F1.6a	38	38	37	38	2.80E-06	2.65E-06	1.82E-06	2.25E-06

T24F1.6b	40	41	39	41	7.76E-06	9.18E-06	1.44E-05	4.23E-06
T24F1.7	14	16	13	1	2.80E-06	2.65E-06	1.82E-06	2.25E-06
T24G10.2	507	614	674	1051	2.80E-06	2.65E-06	1.82E-06	2.25E-06
T24H10.1	605	446	601	625	6.61E-06	7.14E-06	3.99E-06	2.25E-06
T24H10.3	305	392	386	324	3.93E-05	4.50E-05	3.40E-05	6.54E-05
T24H10.4	280	279	478	600	7.31E-05	5.09E-05	4.72E-05	6.07E-05
T24H10.5	3	20	20	9	3.62E-05	4.39E-05	2.98E-05	3.09E-05
T24H10.6	143	166	641	240	2.66E-05	2.50E-05	2.95E-05	4.57E-05
T24H10.7a	156	297	128	156	2.80E-06	2.65E-06	1.82E-06	2.25E-06
T24H10.7b	110	162	87	114	4.02E-05	4.41E-05	1.17E-04	5.42E-05
T24H10.7c	84	195	44	81	1.28E-05	2.30E-05	6.83E-06	1.03E-05
T24H7.1	1591	1553	1419	2061	1.30E-05	1.80E-05	6.67E-06	1.08E-05
T24H7.2.1	1016	1432	1074	1715	1.46E-05	3.20E-05	4.97E-06	1.13E-05
T24H7.2.2	950	1368	1001	1636	1.47E-04	1.36E-04	8.55E-05	1.53E-04
T24H7.3.1	301	337	365	338	3.78E-05	5.04E-05	2.60E-05	5.13E-05
T24H7.3.2	224	254	238	241	3.82E-05	5.20E-05	2.62E-05	5.29E-05
T24H7.4	96	95	220	92	4.76E-05	5.04E-05	3.76E-05	4.30E-05
T24H7.5a	86	108	62	128	4.39E-05	4.71E-05	3.04E-05	3.80E-05
T24H7.5b	408	641	480	693	3.35E-05	3.13E-05	4.99E-05	2.58E-05
T24H7.5c.1	332	517	396	536	2.80E-06	3.09E-06	1.82E-06	3.13E-06
T24H7.5c.2	302	485	357	521	1.02E-05	1.52E-05	7.84E-06	1.40E-05
T25B2.1	1	0	0	2	4.84E-05	7.11E-05	3.75E-05	6.27E-05
T25B2.2a	45	84	59	61	4.65E-05	7.05E-05	3.58E-05	6.44E-05
T25B2.2b	47	87	61	64	2.80E-06	2.65E-06	1.82E-06	2.25E-06
T25B6.1	2	3	3	2	2.80E-06	4.87E-06	2.37E-06	3.01E-06
T25B6.2	655	634	399	548	3.33E-06	5.82E-06	2.82E-06	3.64E-06
T25B6.3	2	1	3	1	2.80E-06	2.65E-06	1.82E-06	2.25E-06
T25B6.4	3	1	0	0	2.85E-05	2.60E-05	1.13E-05	1.91E-05
T25B6.5	0	2	0	0	2.80E-06	2.65E-06	1.82E-06	2.25E-06
T25B6.6	1	6	2	1	2.80E-06	2.65E-06	1.82E-06	2.25E-06
T25B6.7	28	29	21	37	2.80E-06	2.65E-06	1.82E-06	2.25E-06
T25B6.t1	0	0	2	0	2.80E-06	2.65E-06	1.82E-06	2.25E-06
T25B6.t2	0	0	1	0	2.80E-06	2.65E-06	1.82E-06	2.25E-06
T25B9.1	124	157	122	190	2.80E-06	2.65E-06	2.02E-06	2.25E-06
T25B9.2	6	29	12	5	2.80E-06	2.65E-06	1.82E-06	2.25E-06
T25B9.3a	8	6	5	10	1.10E-05	1.31E-05	7.03E-06	1.35E-05
T25B9.3b	5	8	10	8	2.80E-06	2.78E-06	1.82E-06	2.25E-06
T25B9.4	31	57	19	17	2.80E-06	2.65E-06	1.82E-06	2.25E-06
T25B9.5	21	32	17	13	2.80E-06	2.65E-06	1.82E-06	2.25E-06
T25B9.6	63	80	59	41	2.80E-06	3.57E-06	1.82E-06	2.25E-06
T25B9.7	22	47	24	34	2.80E-06	2.65E-06	1.82E-06	2.25E-06
T25B9.8	83	100	68	124	4.00E-06	4.79E-06	2.44E-06	2.25E-06
T25B9.9.1	1247	1793	1530	2269	2.80E-06	2.99E-06	1.82E-06	2.25E-06
T25B9.9.2	1089	1527	1320	2022	6.27E-06	7.14E-06	3.35E-06	7.53E-06
T25C12.1a	29	45	53	78	8.43E-05	1.15E-04	6.73E-05	1.23E-04
T25C12.1b	29	48	54	85	8.34E-05	1.10E-04	6.58E-05	1.24E-04
T25C12.2	1	1	0	2	2.80E-06	3.07E-06	2.50E-06	4.54E-06
T25C12.4	0	2	0	1	2.80E-06	2.94E-06	2.28E-06	4.43E-06
T25C8.1	5	11	11	10	2.80E-06	2.65E-06	1.82E-06	2.25E-06
T25C8.2.1	3566	3437	4508	4934	2.80E-06	2.65E-06	1.82E-06	2.25E-06
T25C8.2.3	3332	3186	4078	4633	2.80E-06	2.65E-06	1.82E-06	2.25E-06
T25C8.3	11	21	4	5	2.97E-04	2.70E-04	2.44E-04	3.30E-04
T25D1.1	15	6	8	14	3.31E-04	2.99E-04	2.63E-04	3.69E-04
T25D1.2	4	6	7	4	2.80E-06	2.65E-06	1.82E-06	2.25E-06
T25D1.3	9	12	6	8	2.80E-06	2.65E-06	1.82E-06	2.25E-06
T25D10.1	55	138	43	52	2.80E-06	2.65E-06	1.82E-06	2.25E-06
T25D10.2	7	12	4	9	2.80E-06	2.65E-06	1.82E-06	2.25E-06
T25D10.3	5	6	11	6	5.24E-06	1.24E-05	2.66E-06	3.96E-06
T25D10.4	36	39	12	23	2.80E-06	2.65E-06	1.82E-06	2.25E-06

T25D10.5	35	56	15	24	2.80E-06	2.65E-06	1.82E-06	2.25E-06
T25D3.2	365	371	346	416	5.43E-06	5.58E-06	1.82E-06	2.79E-06
T25D3.3	23	45	27	46	4.37E-06	6.59E-06	1.82E-06	2.41E-06
T25D3.4	381	348	722	794	4.38E-05	4.21E-05	2.70E-05	4.01E-05
T25E12.10	6	5	8	5	2.80E-06	3.70E-06	1.82E-06	3.22E-06
T25E12.11	7	3	8	7	1.85E-05	1.60E-05	2.28E-05	3.10E-05
T25E12.12	3	10	6	5	2.80E-06	2.65E-06	1.82E-06	2.25E-06
T25E12.13	5	7	5	2	2.80E-06	2.65E-06	1.82E-06	2.25E-06
T25E12.4a	415	367	417	561	2.80E-06	2.65E-06	1.82E-06	2.25E-06
T25E12.4b	17	28	18	33	2.80E-06	2.65E-06	1.82E-06	2.25E-06
T25E12.5.1	1101	905	1600	1850	1.27E-05	1.06E-05	8.31E-06	1.38E-05
T25E12.5.2	940	747	1365	1669	2.83E-06	4.42E-06	1.95E-06	4.41E-06
T25E12.6	27	24	9	25	1.21E-04	9.39E-05	1.14E-04	1.63E-04
T25E12.7	5	1	2	3	1.15E-04	8.67E-05	1.09E-04	1.65E-04
T25E12.8	5	4	3	4	2.94E-06	2.65E-06	1.82E-06	2.25E-06
T25E12.9	1	5	6	4	2.80E-06	2.65E-06	1.82E-06	2.25E-06
T25E4.1	31	60	17	10	2.80E-06	2.65E-06	1.82E-06	2.25E-06
T25E4.2	10	6	1	5	2.80E-06	2.65E-06	1.82E-06	2.25E-06
T25F10.1	430	445	437	259	3.56E-06	6.48E-06	1.82E-06	2.25E-06
T25F10.2.1	38	80	42	51	2.80E-06	2.65E-06	1.82E-06	2.25E-06
T25F10.2.2	38	84	43	53	1.34E-04	1.31E-04	8.85E-05	6.47E-05
T25F10.4	8	8	4	5	2.80E-06	4.92E-06	1.82E-06	2.68E-06
T25F10.5	9	14	3	5	2.80E-06	5.13E-06	1.82E-06	2.74E-06
T25F10.6a	4683	4955	5017	5942	2.80E-06	2.65E-06	1.82E-06	2.25E-06
T25F10.6b.1	3556	3758	3838	4200	2.80E-06	2.65E-06	1.82E-06	2.25E-06
T25F10.6b.2	3491	3638	3673	4046	3.56E-04	3.56E-04	2.48E-04	3.63E-04
T25G12.1	7	5	4	1	2.03E-04	2.03E-04	1.43E-04	1.93E-04
T25G12.11	9	19	12	6	1.98E-04	1.95E-04	1.36E-04	1.84E-04
T25G12.2	23	52	17	22	2.80E-06	2.65E-06	1.82E-06	2.25E-06
T25G12.3	20	16	6	16	2.80E-06	3.17E-06	1.82E-06	2.25E-06
T25G12.4	201	237	186	166	2.80E-06	3.41E-06	1.82E-06	2.25E-06
T25G12.5.1	263	381	324	393	9.21E-06	6.96E-06	1.82E-06	5.91E-06
T25G12.5.2	226	327	279	370	2.35E-05	2.61E-05	1.41E-05	1.56E-05
T25G12.6	59	82	91	110	2.19E-05	2.99E-05	1.75E-05	2.63E-05
T25G12.7	95	148	86	144	2.04E-05	2.79E-05	1.64E-05	2.68E-05
T25G3.1	144	140	220	86	2.80E-06	3.28E-06	2.51E-06	3.73E-06
T25G3.2.1	2526	2555	5262	6146	1.14E-05	1.67E-05	6.71E-06	1.38E-05
T25G3.2.2	2452	2470	5112	6017	2.52E-05	2.31E-05	2.50E-05	1.21E-05
T25G3.3.1	810	891	922	1291	6.52E-05	6.23E-05	8.84E-05	1.27E-04
T25G3.3.2	739	795	797	1135	6.78E-05	6.45E-05	9.19E-05	1.34E-04
T25G3.4	835	988	1156	1415	5.23E-05	5.44E-05	3.88E-05	6.70E-05
T26A5.1	31	68	32	30	5.35E-05	5.44E-05	3.76E-05	6.60E-05
T26A5.2a	379	387	502	738	3.74E-05	4.18E-05	3.37E-05	5.09E-05
T26A5.2b	284	297	392	562	2.80E-06	3.91E-06	1.82E-06	2.25E-06
T26A5.3	484	525	549	656	1.58E-05	1.53E-05	1.37E-05	2.48E-05
T26A5.4	268	358	510	614	1.60E-05	1.59E-05	1.44E-05	2.55E-05
T26A5.5a	967	835	1105	1762	3.65E-05	3.73E-05	2.69E-05	3.97E-05
T26A5.6.1	208	277	248	300	2.01E-05	2.54E-05	2.49E-05	3.70E-05
T26A5.6.2	190	251	229	274	2.67E-05	2.18E-05	1.99E-05	3.91E-05
T26A5.7a	303	365	359	479	1.28E-05	1.61E-05	9.91E-06	1.48E-05
T26A5.7b.1	468	562	549	799	1.36E-05	1.70E-05	1.07E-05	1.58E-05
T26A5.7b.2	461	549	550	786	4.56E-05	5.18E-05	3.51E-05	5.78E-05
T26A5.8	164	231	220	219	4.00E-05	4.54E-05	3.06E-05	5.49E-05
T26A5.9.1	2574	2852	1764	1650	6.93E-05	7.80E-05	5.38E-05	9.49E-05
T26A5.9.2	2537	2822	1729	1608	3.93E-05	5.22E-05	3.43E-05	4.21E-05
T26A8.1.1	765	823	798	1209	4.69E-04	4.91E-04	2.09E-04	2.41E-04
T26A8.1.2	707	756	729	1158	4.78E-04	5.02E-04	2.12E-04	2.43E-04
T26A8.2	5	3	13	9	3.25E-05	3.30E-05	2.20E-05	4.12E-05
T26A8.3	4	3	4	1	3.25E-05	3.28E-05	2.18E-05	4.27E-05

T26A8.5	6	9	11	5	2.80E-06	2.65E-06	1.82E-06	2.25E-06
T26C11.1	0	4	3	1	2.80E-06	2.65E-06	1.82E-06	2.25E-06
T26C11.2	2	5	14	12	2.80E-06	2.65E-06	1.82E-06	2.25E-06
T26C11.3	2	2	1	0	2.80E-06	2.65E-06	1.82E-06	2.25E-06
T26C11.4	48	60	73	110	2.80E-06	2.65E-06	1.82E-06	2.25E-06
T26C11.5	43	54	62	47	2.80E-06	2.65E-06	1.82E-06	2.25E-06
T26C11.7	104	100	149	186	2.80E-06	3.20E-06	2.68E-06	4.97E-06
T26C11.8	0	4	3	3	3.67E-06	4.34E-06	3.43E-06	3.22E-06
T26C12.1.1	428	562	607	787	6.55E-06	5.95E-06	6.12E-06	9.42E-06
T26C12.1.2	368	490	552	735	2.80E-06	2.65E-06	1.82E-06	2.25E-06
T26C12.2	7	3	7	4	2.26E-05	2.80E-05	2.08E-05	3.33E-05
T26C12.3	7	16	18	17	2.13E-05	2.68E-05	2.08E-05	3.42E-05
T26C12.4	10	9	11	7	2.80E-06	2.65E-06	1.82E-06	2.25E-06
T26C12.6	148	143	205	181	2.80E-06	2.65E-06	1.82E-06	2.25E-06
T26C5.1	243	329	230	152	2.80E-06	2.65E-06	1.82E-06	2.25E-06
T26C5.2	183	217	77	56	2.94E-05	2.68E-05	2.65E-05	2.89E-05
T26C5.3a	607	636	554	981	3.77E-05	4.82E-05	2.32E-05	1.89E-05
T26C5.3b	555	576	501	907	1.15E-05	1.29E-05	3.13E-06	2.81E-06
T26C5.3c	672	698	599	1060	3.58E-05	3.54E-05	2.13E-05	4.65E-05
T26C5.4	28	28	16	13	3.79E-05	3.72E-05	2.23E-05	4.98E-05
T26C5.5	16	20	19	14	3.39E-05	3.33E-05	1.97E-05	4.30E-05
T26E3.1	0	2	1	0	7.70E-06	7.27E-06	2.86E-06	2.86E-06
T26E3.10	3	3	5	1	3.98E-06	4.68E-06	3.06E-06	2.79E-06
T26E3.2	17	29	15	20	2.80E-06	2.65E-06	1.82E-06	2.25E-06
T26E3.3a	318	357	399	567	2.80E-06	2.65E-06	1.82E-06	2.25E-06
T26E3.3b	318	357	399	567	2.80E-06	2.80E-06	1.82E-06	2.25E-06
T26E3.4	300	286	198	287	2.53E-05	2.68E-05	2.06E-05	3.62E-05
T26E3.5	3	4	4	0	3.53E-05	3.75E-05	2.88E-05	5.06E-05
T26E3.6	0	0	0	1	4.09E-05	3.68E-05	1.76E-05	3.14E-05
T26E3.7	21	17	22	14	2.80E-06	2.65E-06	1.82E-06	2.25E-06
T26E3.8	4	7	5	5	2.80E-06	2.65E-06	1.82E-06	2.25E-06
T26E3.9	1	3	2	4	7.34E-06	5.61E-06	4.99E-06	3.91E-06
T26E4.1	0	6	3	5	2.80E-06	2.65E-06	1.82E-06	2.25E-06
T26E4.10	3	1	0	0	2.80E-06	2.65E-06	1.82E-06	2.25E-06
T26E4.11	2	2	10	4	2.80E-06	2.65E-06	1.82E-06	2.25E-06
T26E4.12	7	6	2	4	2.80E-06	2.65E-06	1.82E-06	2.25E-06
T26E4.13	5	5	15	6	2.80E-06	2.65E-06	1.82E-06	2.25E-06
T26E4.14	1	2	3	0	2.80E-06	2.65E-06	1.82E-06	2.25E-06
T26E4.15	5	2	4	0	2.80E-06	2.65E-06	1.82E-06	2.25E-06
T26E4.16	3	3	5	6	2.80E-06	2.65E-06	1.82E-06	2.25E-06
T26E4.2	1	2	8	1	2.80E-06	2.65E-06	1.82E-06	2.25E-06
T26E4.3	6	5	20	8	2.80E-06	2.65E-06	1.82E-06	2.25E-06
T26E4.4	7	24	15	10	2.80E-06	2.65E-06	1.82E-06	2.25E-06
T26E4.5	2	3	8	4	2.80E-06	2.65E-06	1.82E-06	2.25E-06
T26E4.6	5	2	9	5	2.80E-06	2.65E-06	1.82E-06	2.25E-06
T26E4.7	6	7	4	8	2.80E-06	2.65E-06	1.82E-06	2.25E-06
T26E4.8	3	1	1	1	2.80E-06	2.65E-06	1.82E-06	2.25E-06
T26E4.9	3	0	1	1	2.80E-06	2.65E-06	1.82E-06	2.25E-06
T26F2.1	5	13	7	3	2.80E-06	2.65E-06	1.82E-06	2.25E-06
T26F2.2	12	21	14	17	2.80E-06	2.65E-06	1.82E-06	2.25E-06
T26G10.1	382	421	248	366	2.80E-06	2.65E-06	1.82E-06	2.25E-06
T26G10.3	5	2	6	0	2.80E-06	2.65E-06	1.82E-06	2.25E-06
T26G10.4	3	2	1	1	2.72E-05	2.84E-05	1.15E-05	2.10E-05
T26G10.5	7	10	11	6	2.80E-06	2.65E-06	1.82E-06	2.25E-06
T26G10.6	29	30	33	43	2.80E-06	2.65E-06	1.82E-06	2.25E-06
T26H10.1	51	78	27	47	2.80E-06	2.80E-06	2.11E-06	2.25E-06
T26H2.1	5	3	6	2	9.49E-06	9.28E-06	7.03E-06	1.13E-05
T26H2.2	2	8	8	2	2.80E-06	3.81E-06	1.82E-06	2.25E-06
T26H2.3	7	8	29	9	2.80E-06	2.65E-06	1.82E-06	2.25E-06

T26H2.4	2	4	1	3	2.80E-06	2.65E-06	1.82E-06	2.25E-06
T26H2.5	3	1	1	1	2.80E-06	2.65E-06	1.82E-06	2.25E-06
T26H2.6	5	7	13	8	2.80E-06	2.65E-06	1.82E-06	2.25E-06
T26H2.7	11	14	19	19	2.80E-06	2.65E-06	1.82E-06	2.25E-06
T26H2.8	8	4	5	1	2.80E-06	2.65E-06	1.82E-06	2.25E-06
T26H2.9	21	31	11	29	2.80E-06	2.65E-06	1.82E-06	2.25E-06
T26H5.1	3	0	13	6	2.80E-06	2.65E-06	1.82E-06	2.25E-06
T26H5.10	2	6	5	4	2.80E-06	2.65E-06	1.82E-06	2.25E-06
T26H5.3	5	8	5	4	2.80E-06	2.65E-06	1.82E-06	2.25E-06
T26H5.4	1	1	3	2	2.80E-06	2.65E-06	1.82E-06	2.25E-06
T26H5.5	5	7	10	9	2.80E-06	2.65E-06	1.82E-06	2.25E-06
T26H5.6	4	3	12	2	2.80E-06	2.65E-06	1.82E-06	2.25E-06
T26H5.7	5	4	6	1	2.80E-06	2.65E-06	1.82E-06	2.25E-06
T26H5.8	9	21	12	12	2.80E-06	2.65E-06	1.82E-06	2.25E-06
T26H5.9	23	74	181	56	2.80E-06	2.65E-06	1.82E-06	2.25E-06
T26H8.2	7	10	14	9	2.80E-06	2.65E-06	1.82E-06	2.25E-06
T26H8.3	0	3	3	0	1.15E-05	3.48E-05	5.86E-05	2.24E-05
T26H8.4	8	7	25	11	2.80E-06	2.65E-06	1.82E-06	2.25E-06
T27A1.1	2969	2793	6123	1617	2.80E-06	2.65E-06	1.82E-06	2.25E-06
T27A1.2	8	16	8	9	2.80E-06	2.65E-06	1.82E-06	2.25E-06
T27A1.3	3	10	10	1	6.33E-04	5.63E-04	8.50E-04	2.77E-04
T27A1.4	90	153	106	129	2.80E-06	2.65E-06	1.82E-06	2.25E-06
T27A1.5a.1	106	160	92	85	2.80E-06	2.65E-06	1.82E-06	2.25E-06
T27A1.5a.2	91	132	74	71	7.87E-06	1.26E-05	6.01E-06	9.04E-06
T27A1.5b.1	99	138	85	74	8.01E-06	1.14E-05	4.52E-06	5.15E-06
T27A1.5b.2	91	132	74	71	7.50E-06	1.03E-05	3.97E-06	4.70E-06
T27A1.6	6	20	35	31	8.09E-06	1.07E-05	4.54E-06	4.86E-06
T27A1.7	3	3	3	2	7.50E-06	1.03E-05	3.97E-06	4.70E-06
T27A10.2	4	10	10	6	2.80E-06	2.65E-06	1.91E-06	2.25E-06
T27A10.3a.1	129	192	135	149	2.80E-06	2.65E-06	1.82E-06	2.25E-06
T27A10.3a.2	114	172	116	134	2.80E-06	2.65E-06	1.82E-06	2.25E-06
T27A10.3b	108	167	111	130	1.29E-05	1.81E-05	8.75E-06	1.19E-05
T27A10.5	1	0	0	2	1.23E-05	1.76E-05	8.16E-06	1.16E-05
T27A10.6.1	271	423	177	155	1.23E-05	1.79E-05	8.20E-06	1.19E-05
T27A10.6.2	247	393	165	146	2.80E-06	2.65E-06	1.82E-06	2.25E-06
T27A10.7	79	126	45	51	1.05E-05	1.54E-05	4.45E-06	4.81E-06
T27A3.1a	448	555	257	523	1.06E-05	1.59E-05	4.59E-06	5.02E-06
T27A3.1b	430	533	252	507	6.94E-06	1.05E-05	2.57E-06	3.60E-06
T27A3.1c	374	453	227	476	2.34E-05	2.74E-05	8.75E-06	2.20E-05
T27A3.1d.1	288	363	202	416	2.32E-05	2.72E-05	8.86E-06	2.20E-05
T27A3.1d.2	274	351	190	407	2.42E-05	2.77E-05	9.57E-06	2.48E-05
T27A3.1e	286	368	196	413	2.21E-05	2.63E-05	1.01E-05	2.56E-05
T27A3.2	1240	996	1497	1839	2.10E-05	2.54E-05	9.49E-06	2.51E-05
T27A3.3	57	93	123	29	2.00E-05	2.44E-05	8.93E-06	2.32E-05
T27A3.4	266	354	241	91	5.36E-05	4.07E-05	4.21E-05	6.39E-05
T27A3.5	39	80	15	22	1.62E-05	2.50E-05	2.28E-05	6.63E-06
T27A3.6	131	187	164	178	4.28E-05	5.37E-05	2.52E-05	1.17E-05
T27A3.7	317	309	377	509	4.17E-06	8.09E-06	1.82E-06	2.25E-06
T27A3.8	1	6	6	2	1.43E-05	1.93E-05	1.17E-05	1.57E-05
T27A8.1	72	65	49	74	2.23E-05	2.05E-05	1.73E-05	2.87E-05
T27A8.2	16	18	31	25	2.80E-06	2.65E-06	1.82E-06	2.25E-06
T27A8.3	17	10	10	19	5.07E-06	4.31E-06	2.24E-06	4.18E-06
T27A8.4	5	8	0	2	4.20E-06	4.47E-06	5.30E-06	5.29E-06
T27A8.5	8	3	7	6	3.47E-06	2.65E-06	1.82E-06	3.10E-06
T27B1.1	57	60	53	66	2.80E-06	2.88E-06	1.82E-06	2.25E-06
T27B1.2.1	110	136	48	138	2.80E-06	2.65E-06	1.82E-06	2.25E-06
T27B1.2.2	57	76	29	78	2.80E-06	2.65E-06	1.82E-06	2.25E-06
T27B7.1	15	35	17	10	5.82E-06	6.80E-06	1.82E-06	5.87E-06
T27B7.2	9	6	43	16	4.37E-06	5.48E-06	1.82E-06	4.79E-06

T27B7.3	25	22	24	28	2.80E-06	2.65E-06	1.82E-06	2.25E-06
T27B7.5	6	11	7	1	2.80E-06	2.65E-06	2.19E-06	2.25E-06
T27B7.6a	25	36	32	22	2.80E-06	2.65E-06	1.82E-06	2.25E-06
T27B7.6b	16	30	30	16	2.80E-06	2.65E-06	1.82E-06	2.25E-06
T27B7.7	3	5	2	3	2.80E-06	3.04E-06	1.86E-06	2.25E-06
T27C10.1	6	4	9	1	2.80E-06	3.25E-06	2.24E-06	2.25E-06
T27C10.2	8	6	12	8	2.80E-06	2.65E-06	1.82E-06	2.25E-06
T27C10.3	136	128	243	322	2.80E-06	2.65E-06	1.82E-06	2.25E-06
T27C10.4	6	8	22	3	2.80E-06	2.65E-06	1.82E-06	2.25E-06
T27C10.6	226	308	249	388	1.49E-05	1.33E-05	1.74E-05	2.84E-05
T27C10.7	30	102	20	12	2.80E-06	2.65E-06	1.82E-06	2.25E-06
T27C4.1.1	203	295	287	249	3.33E-06	4.31E-06	2.41E-06	4.61E-06
T27C4.1.2	187	274	266	234	1.51E-05	4.86E-05	6.56E-06	4.86E-06
T27C4.2	34	34	34	34	1.82E-05	2.50E-05	1.68E-05	1.79E-05
T27C4.3	3	3	5	4	1.95E-05	2.69E-05	1.80E-05	1.95E-05
T27C4.4a	264	318	590	664	2.80E-06	2.65E-06	1.82E-06	2.25E-06
T27C4.4b	1227	2332	6166	7046	2.80E-06	2.65E-06	1.82E-06	2.25E-06
T27C4.4c	222	260	519	548	9.55E-06	1.08E-05	1.39E-05	1.93E-05
T27C4.4d	249	297	564	617	4.52E-05	8.11E-05	1.48E-04	2.08E-04
T27C4.5	2	4	2	3	1.09E-05	1.20E-05	1.65E-05	2.15E-05
T27C5.1	8	3	8	8	1.06E-05	1.20E-05	1.56E-05	2.11E-05
T27C5.10	3	3	0	1	2.80E-06	2.65E-06	1.82E-06	2.25E-06
T27C5.11	5	2	5	2	2.80E-06	2.65E-06	1.82E-06	2.25E-06
T27C5.12	2	7	51	8	2.80E-06	2.65E-06	1.82E-06	2.25E-06
T27C5.13	3	1	1	0	2.80E-06	2.65E-06	1.82E-06	2.25E-06
T27C5.14	6	5	3	1	2.80E-06	2.65E-06	3.99E-06	2.25E-06
T27C5.2	1	2	0	0	2.80E-06	2.65E-06	1.82E-06	2.25E-06
T27C5.3	5	2	10	4	2.80E-06	2.65E-06	1.82E-06	2.25E-06
T27C5.4	7	8	17	9	2.80E-06	2.65E-06	1.82E-06	2.25E-06
T27C5.5	4	3	3	0	2.80E-06	2.65E-06	1.82E-06	2.25E-06
T27C5.6	0	3	2	0	2.80E-06	2.65E-06	1.82E-06	2.25E-06
T27C5.7	6	3	0	1	2.80E-06	2.65E-06	1.82E-06	2.25E-06
T27C5.8	1	1	7	1	2.80E-06	2.65E-06	1.82E-06	2.25E-06
T27D1.1	186	198	192	254	2.80E-06	2.65E-06	1.82E-06	2.25E-06
T27D1.3	21	27	84	27	2.80E-06	2.65E-06	1.82E-06	2.25E-06
T27D12.1.1	185	460	171	189	2.22E-05	2.24E-05	1.49E-05	2.44E-05
T27D12.1.2	183	456	168	187	2.80E-06	2.65E-06	5.14E-06	2.25E-06
T27D12.1.3	137	357	112	143	1.11E-05	2.62E-05	6.71E-06	9.15E-06
T27D12.2a	317	614	344	833	1.19E-05	2.80E-05	7.12E-06	9.78E-06
T27D12.2b	331	641	358	848	1.03E-05	2.54E-05	5.48E-06	8.66E-06
T27D12.3	2	8	1	7	1.23E-05	2.25E-05	8.66E-06	2.59E-05
T27E4.1	2	13	9	7	1.21E-05	2.22E-05	8.53E-06	2.49E-05
T27E4.2	399	1990	102	217	2.80E-06	2.65E-06	1.82E-06	2.25E-06
T27E4.3	591	2371	157	242	2.80E-06	2.65E-06	1.82E-06	2.25E-06
T27E4.5	9	7	3	4	1.02E-04	4.81E-04	1.70E-05	4.46E-05
T27E4.6	24	36	20	16	1.53E-04	5.81E-04	2.65E-05	5.04E-05
T27E4.7.1	35	109	40	40	2.80E-06	2.65E-06	1.82E-06	2.25E-06
T27E4.7.2	24	85	31	32	2.80E-06	2.65E-06	1.82E-06	2.25E-06
T27E4.8	509	2448	139	294	4.70E-06	1.39E-05	3.52E-06	4.34E-06
T27E4.9	591	2371	157	242	4.70E-06	1.57E-05	3.95E-06	5.04E-06
T27E7.1	81	125	105	34	1.30E-04	5.91E-04	2.31E-05	6.04E-05
T27E7.10	11	11	11	8	1.53E-04	5.81E-04	2.65E-05	5.04E-05
T27E7.2	6	10	2	8	1.27E-05	1.85E-05	1.07E-05	4.27E-06
T27E7.3	5	10	12	6	2.80E-06	2.65E-06	1.82E-06	2.25E-06
T27E7.4	4	8	8	3	2.80E-06	2.65E-06	1.82E-06	2.25E-06
T27E7.5	6	1	5	1	2.80E-06	2.65E-06	1.82E-06	2.25E-06
T27E7.6	8	16	18	11	2.80E-06	2.65E-06	1.82E-06	2.25E-06
T27E7.8	6	3	7	7	2.80E-06	2.65E-06	1.82E-06	2.25E-06
T27E7.9	6	7	4	1	2.80E-06	2.65E-06	1.82E-06	2.25E-06

T27E9.1a.1	15799	17712	19960	24019	2.80E-06	2.65E-06	1.82E-06	2.25E-06
T27E9.1a.2	14411	16283	17777	22250	2.80E-06	2.65E-06	1.82E-06	2.25E-06
T27E9.1c	13918	15572	17585	20932	1.59E-03	1.68E-03	1.30E-03	1.94E-03
T27E9.2.1	2754	2767	2863	2999	1.46E-03	1.56E-03	1.17E-03	1.81E-03
T27E9.2.2	502	599	534	200	1.73E-03	1.82E-03	1.42E-03	2.09E-03
T27E9.3	312	244	486	465	1.90E-04	1.81E-04	1.29E-04	1.66E-04
T27E9.4a.1	213	293	246	367	1.62E-04	1.83E-04	1.12E-04	5.18E-05
T27E9.4a.2	224	302	252	383	3.51E-05	2.59E-05	3.56E-05	4.20E-05
T27E9.4b	212	289	242	364	1.32E-05	1.72E-05	9.97E-06	1.84E-05
T27E9.5	256	275	420	432	1.34E-05	1.71E-05	9.84E-06	1.84E-05
T27E9.6	0	3	1	0	1.34E-05	1.72E-05	9.93E-06	1.84E-05
T27E9.7.1	3212	3044	3074	4403	1.64E-05	1.66E-05	1.75E-05	2.22E-05
T27E9.7.2	2992	2875	2906	4146	2.80E-06	2.65E-06	1.82E-06	2.25E-06
T27E9.8	3	1	6	2	1.49E-04	1.34E-04	9.31E-05	1.65E-04
T27E9.9	7	15	10	10	1.47E-04	1.33E-04	9.27E-05	1.63E-04
T27F2.1.1	515	567	590	758	2.80E-06	2.65E-06	1.82E-06	2.25E-06
T27F2.1.2	476	535	512	716	2.80E-06	2.65E-06	1.82E-06	2.25E-06
T27F2.2	110	207	141	217	3.32E-05	3.46E-05	2.48E-05	3.93E-05
T27F2.3.1	242	215	186	196	3.20E-05	3.40E-05	2.24E-05	3.87E-05
T27F2.3.2	285	250	244	215	3.84E-06	6.85E-06	3.21E-06	6.09E-06
T27F2.4	16	80	23	26	4.80E-05	4.03E-05	2.40E-05	3.12E-05
T27F6.1	7	15	7	2	5.47E-05	4.54E-05	3.05E-05	3.32E-05
T27F6.2	10	15	6	9	3.22E-06	1.52E-05	3.01E-06	4.21E-06
T27F6.4	598	510	741	948	2.80E-06	2.65E-06	1.82E-06	2.25E-06
T27F6.5a.1	110	136	126	139	2.80E-06	2.65E-06	1.82E-06	2.25E-06
T27F6.5a.2	98	122	113	127	1.00E-04	8.05E-05	8.06E-05	1.27E-04
T27F6.5b	18	23	20	17	8.01E-06	9.36E-06	5.98E-06	8.14E-06
T27F6.5c	30	36	29	37	7.90E-06	9.28E-06	5.92E-06	8.23E-06
T27F6.8	30	120	29	33	5.85E-06	7.06E-06	4.23E-06	4.43E-06
T27F6.9	3	6	4	3	6.72E-06	7.59E-06	4.23E-06	6.63E-06
T27F7.1.1	522	530	511	593	2.80E-06	8.49E-06	1.82E-06	2.25E-06
T27F7.1.2	393	402	336	434	2.80E-06	2.65E-06	1.82E-06	2.25E-06
T27F7.2a	58	70	62	74	8.03E-05	7.70E-05	5.12E-05	7.33E-05
T27F7.2b.1	51	53	57	68	6.99E-05	6.75E-05	3.89E-05	6.20E-05
T27F7.2b.2	52	54	58	70	2.80E-06	2.80E-06	1.82E-06	2.52E-06
T27F7.2c	34	40	37	43	2.80E-06	2.65E-06	1.91E-06	2.81E-06
T27F7.3a	898	1026	799	1115	2.80E-06	2.65E-06	1.90E-06	2.81E-06
T27F7.3b.1	1040	1248	872	1047	3.50E-06	3.89E-06	2.48E-06	3.55E-06
T27F7.3b.2	845	1020	735	969	4.53E-05	4.89E-05	2.62E-05	4.51E-05
T28A11.1	6	5	1	2	2.06E-04	2.33E-04	1.12E-04	1.66E-04
T28A11.10	1	2	4	0	1.63E-04	1.85E-04	9.21E-05	1.50E-04
T28A11.11	4	5	5	3	2.80E-06	2.65E-06	1.82E-06	2.25E-06
T28A11.12	5	2	11	3	2.80E-06	2.65E-06	1.82E-06	2.25E-06
T28A11.13	1	2	3	0	2.80E-06	2.65E-06	1.82E-06	2.25E-06
T28A11.15	3	9	8	7	2.80E-06	2.65E-06	1.82E-06	2.25E-06
T28A11.16	15	17	5	3	2.80E-06	2.65E-06	1.82E-06	2.25E-06
T28A11.17	15	16	9	11	2.80E-06	2.65E-06	1.82E-06	2.25E-06
T28A11.18	4	6	2	7	2.80E-06	2.99E-06	1.82E-06	2.25E-06
T28A11.19	2	6	6	6	2.80E-06	2.65E-06	1.82E-06	2.25E-06
T28A11.20	3	0	7	7	2.80E-06	2.65E-06	1.82E-06	2.25E-06
T28A11.21	74	84	44	63	2.80E-06	2.65E-06	1.82E-06	2.25E-06
T28A11.22	6	9	14	13	2.80E-06	2.65E-06	1.82E-06	2.25E-06
T28A11.3	2	7	1	2	8.79E-06	9.44E-06	3.41E-06	6.03E-06
T28A11.4	0	1	1	1	2.80E-06	2.65E-06	1.99E-06	2.27E-06
T28A11.5	2	0	3	2	2.80E-06	2.65E-06	1.82E-06	2.25E-06
T28A11.6	10	12	12	2	2.80E-06	2.65E-06	1.82E-06	2.25E-06
T28A11.7	4	2	7	9	2.80E-06	2.65E-06	1.82E-06	2.25E-06
T28A11.8	4	2	6	4	3.50E-06	3.97E-06	2.73E-06	2.25E-06
T28A11.9	3	3	4	1	2.80E-06	2.65E-06	1.82E-06	2.25E-06

T28A8.1	3	11	16	1	2.80E-06	2.65E-06	1.82E-06	2.25E-06
T28A8.2	30	21	28	20	2.80E-06	2.65E-06	1.82E-06	2.25E-06
T28A8.3	208	241	316	391	2.80E-06	2.65E-06	1.82E-06	2.25E-06
T28A8.4	178	211	274	315	3.16E-06	2.65E-06	1.91E-06	2.25E-06
T28A8.5	77	127	100	114	1.21E-05	1.32E-05	1.19E-05	1.82E-05
T28A8.6	199	233	254	399	1.41E-05	1.58E-05	1.42E-05	2.01E-05
T28A8.7.1	203	199	371	567	6.75E-06	1.05E-05	5.70E-06	8.03E-06
T28A8.7.2	192	195	357	547	1.11E-05	1.22E-05	9.20E-06	1.79E-05
T28B11.1.1	1339	1162	1842	2266	9.83E-06	9.10E-06	1.17E-05	2.21E-05
T28B11.1.2	899	796	1201	1397	9.41E-06	9.02E-06	1.14E-05	2.15E-05
T28B4.1a	51	110	45	60	5.47E-05	4.49E-05	4.90E-05	7.44E-05
T28B4.1b	48	106	45	58	5.43E-05	4.54E-05	4.72E-05	6.78E-05
T28B4.1c	49	117	48	64	3.56E-06	7.25E-06	2.04E-06	3.35E-06
T28B4.2	3	6	2	7	3.25E-06	6.74E-06	1.97E-06	3.15E-06
T28B4.3	157	393	422	228	3.00E-06	6.77E-06	1.91E-06	3.15E-06
T28B4.4.1	15	20	17	6	2.80E-06	2.65E-06	1.82E-06	2.25E-06
T28B4.4.2	12	15	14	5	3.69E-05	8.74E-05	6.46E-05	4.31E-05
T28B4.t2	1	0	0	0	2.80E-06	2.65E-06	1.82E-06	2.25E-06
T28B8.1.1	83	241	92	212	2.80E-06	2.75E-06	1.82E-06	2.25E-06
T28B8.1.2	46	140	46	118	2.80E-06	2.65E-06	1.82E-06	2.25E-06
T28B8.2	6	24	23	6	1.10E-05	3.01E-05	7.91E-06	2.25E-05
T28B8.3a	32	35	26	28	9.49E-06	2.73E-05	6.18E-06	1.96E-05
T28B8.3b	6	3	2	2	2.80E-06	5.87E-06	3.88E-06	2.25E-06
T28B8.4	62	75	44	41	2.80E-06	2.65E-06	1.82E-06	2.25E-06
T28B8.5	12	6	8	4	2.80E-06	2.65E-06	1.82E-06	2.25E-06
T28B8.6	20	42	13	12	2.80E-06	2.65E-06	1.82E-06	2.25E-06
T28C12.1	22	17	16	8	2.80E-06	2.65E-06	1.82E-06	2.25E-06
T28C12.2	14	23	23	18	3.72E-06	7.41E-06	1.82E-06	2.25E-06
T28C12.3	21	32	8	14	2.80E-06	2.65E-06	1.82E-06	2.25E-06
T28C12.4a	155	576	89	195	2.80E-06	2.65E-06	1.82E-06	2.25E-06
T28C12.4b	139	490	73	170	2.80E-06	2.65E-06	1.82E-06	2.25E-06
T28C12.5	26	116	21	56	7.81E-06	2.75E-05	2.92E-06	7.92E-06
T28C12.6	22	21	10	20	8.09E-06	2.70E-05	2.77E-06	7.94E-06
T28C6.1	60	64	40	26	2.80E-06	7.56E-06	1.82E-06	3.10E-06
T28C6.3	11	10	10	9	2.80E-06	2.65E-06	1.82E-06	2.25E-06
T28C6.4	5350	4993	5679	2546	6.64E-06	6.69E-06	2.88E-06	2.32E-06
T28C6.5	12	40	23	7	2.80E-06	2.65E-06	1.82E-06	2.25E-06
T28C6.6	5354	5006	5673	2551	6.25E-04	5.51E-04	4.32E-04	2.39E-04
T28C6.7	100	132	55	147	2.80E-06	4.21E-06	1.82E-06	2.25E-06
T28C6.8	62	92	45	66	6.27E-04	5.54E-04	4.33E-04	2.40E-04
T28C6.9	678	765	358	840	8.12E-06	1.01E-05	2.90E-06	9.58E-06
T28D6.2	25	33	38	41	8.82E-06	1.24E-05	4.15E-06	7.53E-06
T28D6.3	63	91	136	38	1.25E-05	1.33E-05	4.30E-06	1.25E-05
T28D6.5a	79	91	106	113	2.80E-06	2.65E-06	2.08E-06	2.74E-06
T28D6.5b	89	90	102	119	1.66E-05	2.27E-05	2.34E-05	8.05E-06
T28D6.6	597	527	710	906	3.00E-06	3.28E-06	2.62E-06	3.44E-06
T28D6.7	67	87	54	49	3.36E-06	3.20E-06	2.51E-06	3.62E-06
T28D6.9	100	89	217	110	5.39E-05	4.49E-05	4.17E-05	6.57E-05
T28D9.1.1	1217	1213	2122	1272	6.61E-06	8.09E-06	3.46E-06	3.87E-06
T28D9.1.2	924	924	1479	1066	2.86E-05	2.41E-05	4.05E-05	2.53E-05
T28D9.10.1	690	721	1138	548	1.87E-04	1.76E-04	2.12E-04	1.57E-04
T28D9.10.2	570	598	897	440	1.59E-04	1.50E-04	1.66E-04	1.47E-04
T28D9.11	1	2	0	1	1.24E-04	1.23E-04	1.34E-04	7.94E-05
T28D9.12	1	3	1	1	1.14E-04	1.13E-04	1.17E-04	7.08E-05
T28D9.2a	471	511	735	826	2.80E-06	2.65E-06	1.82E-06	2.25E-06
T28D9.2b	455	507	689	784	2.80E-06	2.65E-06	1.82E-06	2.25E-06
T28D9.2c	335	367	492	555	7.36E-05	7.54E-05	7.47E-05	1.04E-04
T28D9.2d	490	545	689	697	4.99E-05	5.25E-05	4.91E-05	6.90E-05
T28D9.3a	52	150	68	89	6.04E-05	6.25E-05	5.77E-05	8.04E-05

T28D9.3b	67	195	91	147	7.14E-05	7.50E-05	6.53E-05	8.15E-05
T28D9.3c	46	139	60	92	5.68E-06	1.55E-05	4.83E-06	7.80E-06
T28D9.4a	526	458	529	700	6.27E-06	1.72E-05	5.54E-06	1.10E-05
T28D9.4b	429	369	420	613	6.24E-06	1.78E-05	5.30E-06	1.00E-05
T28D9.7	79	112	74	99	3.93E-05	3.24E-05	2.57E-05	4.21E-05
T28D9.9	4	2	4	5	7.55E-05	6.14E-05	4.81E-05	8.67E-05
T28F12.1	10	12	13	6	2.91E-06	3.91E-06	1.82E-06	2.95E-06
T28F12.2a.1	181	234	166	269	2.80E-06	2.65E-06	1.82E-06	2.25E-06
T28F12.2a.2	141	171	131	214	2.80E-06	2.65E-06	1.82E-06	2.25E-06
T28F12.2c	36	41	32	61	9.07E-06	1.11E-05	5.41E-06	1.08E-05
T28F12.2d.1	49	59	48	92	7.03E-06	8.04E-06	4.25E-06	8.55E-06
T28F12.2d.2	80	90	76	134	8.74E-06	9.39E-06	5.05E-06	1.19E-05
T28F12.2d.3	174	229	158	260	9.49E-06	1.08E-05	6.05E-06	1.43E-05
T28F12.2e	184	236	165	272	4.00E-06	4.26E-06	2.48E-06	5.38E-06
T28F12.2f	174	229	158	260	8.65E-06	1.08E-05	5.12E-06	1.04E-05
T28F12.2g.1	141	169	134	216	7.95E-06	9.63E-06	4.65E-06	9.45E-06
T28F12.2g.2	181	234	166	269	8.71E-06	1.08E-05	5.14E-06	1.04E-05
T28F12.2g.3	110	133	101	168	8.51E-06	9.65E-06	5.27E-06	1.05E-05
T28F12.3	572	500	441	879	1.29E-05	1.57E-05	7.67E-06	1.54E-05
T28F2.1	10	12	20	14	7.28E-06	8.31E-06	4.34E-06	8.93E-06
T28F2.2	42	43	73	82	1.43E-05	1.18E-05	7.16E-06	1.76E-05
T28F2.3	11	13	12	23	2.80E-06	2.65E-06	1.82E-06	2.25E-06
T28F2.4a	91	95	62	89	6.47E-06	6.27E-06	7.32E-06	1.02E-05
T28F2.4b	26	30	24	29	2.80E-06	2.65E-06	1.82E-06	2.25E-06
T28F2.5	87	114	80	75	4.17E-06	4.10E-06	1.84E-06	3.28E-06
T28F2.6	55	62	23	32	4.37E-06	4.76E-06	2.62E-06	3.91E-06
T28F2.7	42	59	28	41	4.90E-06	6.06E-06	2.92E-06	3.37E-06
T28F2.8	58	67	45	37	4.90E-06	5.21E-06	1.82E-06	2.29E-06
T28F2.t1	1	0	0	0	2.80E-06	3.04E-06	1.82E-06	2.25E-06
T28F3.1a	441	445	763	930	4.96E-06	5.42E-06	2.51E-06	2.54E-06
T28F3.1b	409	402	718	889	2.80E-06	2.65E-06	1.82E-06	2.25E-06
T28F3.3	229	284	294	425	2.32E-05	2.21E-05	2.61E-05	3.92E-05
T28F3.4a.1	42	77	73	78	2.44E-05	2.26E-05	2.78E-05	4.25E-05
T28F3.4a.2	37	68	72	74	2.12E-05	2.48E-05	1.77E-05	3.16E-05
T28F3.4b.1	34	72	71	72	2.80E-06	4.13E-06	2.70E-06	3.55E-06
T28F3.4b.2	24	48	62	56	2.80E-06	3.97E-06	2.90E-06	3.69E-06
T28F3.5	36	42	59	46	2.80E-06	4.13E-06	2.81E-06	3.51E-06
T28F3.6	3	7	13	6	2.80E-06	4.02E-06	3.57E-06	3.98E-06
T28F3.7	4	5	5	2	2.80E-06	2.65E-06	1.82E-06	2.25E-06
T28F3.8	190	201	315	297	2.80E-06	2.65E-06	1.82E-06	2.25E-06
T28F3.9	14	10	27	20	2.80E-06	2.65E-06	1.82E-06	2.25E-06
T28F4.1.1	147	194	185	220	1.26E-05	1.26E-05	1.36E-05	1.58E-05
T28F4.1.2	94	140	125	161	2.80E-06	2.65E-06	1.82E-06	2.25E-06
T28F4.1.3	85	124	116	150	1.26E-05	1.57E-05	1.03E-05	1.51E-05
T28F4.2	12	15	8	3	1.10E-05	1.55E-05	9.53E-06	1.52E-05
T28F4.3	39	46	28	25	1.10E-05	1.52E-05	9.77E-06	1.56E-05
T28F4.4	75	124	68	126	2.80E-06	2.65E-06	1.82E-06	2.25E-06
T28F4.5.1	476	1408	725	503	2.97E-06	3.31E-06	1.82E-06	2.25E-06
T28F4.5.2	312	847	460	337	3.70E-06	5.77E-06	2.17E-06	4.97E-06
T28F4.6	43	186	28	51	7.78E-05	2.17E-04	7.71E-05	6.61E-05
T28H10.1	27	34	15	17	6.29E-05	1.61E-04	6.03E-05	5.45E-05
T28H10.2	46	78	39	51	5.60E-06	2.28E-05	2.37E-06	5.33E-06
T28H10.3	201	757	150	323	3.36E-06	3.99E-06	1.82E-06	2.25E-06
T28H10.4	4	2	6	0	8.62E-06	1.38E-05	4.76E-06	7.69E-06
T28H11.1	148	285	98	71	1.48E-05	5.26E-05	7.18E-06	1.91E-05
T28H11.2	10	9	9	4	2.80E-06	2.65E-06	2.17E-06	2.25E-06
T28H11.3	12	14	6	3	1.42E-05	2.58E-05	6.12E-06	5.47E-06
T28H11.4	19	17	17	16	2.80E-06	2.65E-06	1.82E-06	2.25E-06
T28H11.5	135	192	96	78	2.80E-06	2.65E-06	1.82E-06	2.25E-06

T28H11.7	30	104	35	21	2.80E-06	2.65E-06	1.82E-06	2.25E-06
T28H11.8.1	35	31	19	23	1.17E-05	1.57E-05	5.41E-06	5.44E-06
T28H11.8.2	33	31	19	22	5.29E-06	1.73E-05	4.01E-06	2.97E-06
VB0393L.2	111	109	238	108	2.80E-06	2.65E-06	1.82E-06	2.25E-06
VB0395L.1	1	16	3	26	2.80E-06	2.65E-06	1.82E-06	2.25E-06
VC27A7L.1	12	8	10	5	3.09E-05	2.87E-05	4.31E-05	2.42E-05
VC5.1	3	3	9	4	2.80E-06	2.65E-06	1.82E-06	2.29E-06
VC5.2	65	141	60	99	2.80E-06	2.65E-06	1.82E-06	2.25E-06
VC5.3a	2706	4671	3220	4211	2.80E-06	2.65E-06	2.30E-06	2.25E-06
VC5.3c	2167	3751	2509	3315	3.78E-06	7.75E-06	2.28E-06	4.63E-06
VC5.4	362	383	564	679	6.81E-05	1.11E-04	5.27E-05	8.51E-05
VC5.5	21	50	28	36	6.75E-05	1.10E-04	5.08E-05	8.29E-05
VC5.6	16	17	16	24	2.53E-05	2.53E-05	2.57E-05	3.82E-05
VF11C1L.1	256	318	320	482	2.80E-06	4.39E-06	1.82E-06	2.70E-06
VF13D12L.1.1	392	534	900	1012	2.80E-06	2.65E-06	1.82E-06	2.25E-06
VF13D12L.1.2	358	494	810	949	6.47E-06	7.59E-06	5.27E-06	9.78E-06
VF13D12L.3	270	439	356	471	2.56E-05	3.30E-05	3.83E-05	5.31E-05
VF36H2L.1	196	216	244	245	2.48E-05	3.23E-05	3.65E-05	5.28E-05
VF39H2L.1	120	142	117	110	2.26E-05	3.47E-05	1.94E-05	3.16E-05
VH15N14R.1	12	12	9	20	2.00E-05	2.08E-05	1.62E-05	2.01E-05
VK10D6R.1	29	60	21	20	1.59E-05	1.78E-05	1.01E-05	1.17E-05
VM106R.1	38	46	33	29	2.80E-06	2.65E-06	1.82E-06	2.70E-06
VT23B5.1	85	104	115	147	1.20E-05	2.35E-05	5.67E-06	6.66E-06
VT23B5.2	131	181	136	198	4.59E-06	5.24E-06	2.59E-06	2.81E-06
VW02B12L.1	2306	2460	3569	4157	3.30E-06	3.81E-06	2.90E-06	4.57E-06
VW02B12L.1	2256	2407	3496	4122	4.17E-06	5.45E-06	2.82E-06	5.08E-06
VW02B12L.2	0	1	3	2	9.57E-05	9.64E-05	9.64E-05	1.39E-04
VW02B12L.3	407	442	680	697	9.41E-05	9.48E-05	9.49E-05	1.38E-04
VW02B12L.3	298	314	469	567	2.80E-06	2.65E-06	1.82E-06	2.25E-06
VW02B12L.4	184	155	267	203	4.04E-05	4.14E-05	4.39E-05	5.56E-05
VW06B3R.1b	501	673	700	779	3.66E-05	3.64E-05	3.74E-05	5.59E-05
VY10G11R.1	8	11	14	9	2.96E-05	2.36E-05	2.80E-05	2.62E-05
VY35H6BL.1	43	31	61	97	4.37E-05	5.54E-05	3.97E-05	5.45E-05
VY35H6BL.2	15	8	17	17	2.80E-06	2.65E-06	1.82E-06	2.25E-06
VZC374L.1	8	18	3	13	5.38E-06	3.68E-06	4.97E-06	9.76E-06
VZK822L.2	8	7	14	6	4.59E-06	2.65E-06	3.39E-06	4.18E-06
W01A11.2	215	234	291	330	2.80E-06	2.65E-06	1.82E-06	2.25E-06
W01A11.4	1356	1066	1333	1097	2.80E-06	2.65E-06	2.57E-06	2.25E-06
W01A11.5	126	90	108	96	2.23E-05	2.29E-05	1.96E-05	2.75E-05
W01A11.7.1	13	25	18	10	1.98E-04	1.47E-04	1.27E-04	1.29E-04
W01A11.7.2	3	11	12	2	6.02E-06	4.05E-06	3.35E-06	3.67E-06
W01A8.1a	1425	1627	1286	1993	2.80E-06	3.23E-06	1.82E-06	2.25E-06
W01A8.1b.1	2220	2478	1999	3149	2.80E-06	2.65E-06	1.82E-06	2.25E-06
W01A8.1b.2	2070	2313	1818	2925	1.27E-04	1.37E-04	7.46E-05	1.43E-04
W01A8.1b.3	1432	1643	1297	2024	1.42E-04	1.49E-04	8.31E-05	1.62E-04
W01A8.2	62	79	43	54	1.34E-04	1.41E-04	7.65E-05	1.52E-04
W01A8.3	13	13	8	6	1.38E-04	1.49E-04	8.11E-05	1.56E-04
W01A8.5	339	346	582	650	9.94E-06	1.20E-05	4.48E-06	6.95E-06
W01A8.6	36	51	16	25	2.80E-06	2.65E-06	1.82E-06	2.25E-06
W01A8.7	6	12	11	9	3.76E-05	3.62E-05	4.20E-05	5.79E-05
W01B11.1	4	7	3	0	3.58E-06	4.81E-06	1.82E-06	2.25E-06
W01B11.2	33	32	21	22	2.80E-06	2.65E-06	1.82E-06	2.25E-06
W01B11.3	2702	2478	2414	3275	2.80E-06	2.65E-06	1.82E-06	2.25E-06
W01B11.5	53	66	28	35	2.80E-06	2.65E-06	1.82E-06	2.25E-06
W01B11.6a.1	66	272	70	99	1.69E-04	1.47E-04	9.84E-05	1.65E-04
W01B11.6a.2	53	206	54	87	4.51E-06	5.29E-06	1.82E-06	2.38E-06
W01B6.1	40	41	19	32	1.79E-05	6.99E-05	1.24E-05	2.16E-05
W01B6.2	39	101	42	25	1.43E-05	5.24E-05	9.46E-06	1.88E-05
W01B6.3	14	17	8	12	3.16E-06	3.07E-06	1.82E-06	2.25E-06

W01B6.4	2	4	2	1	3.89E-06	9.50E-06	2.71E-06	2.25E-06
W01B6.5	23	31	13	12	2.80E-06	2.65E-06	1.82E-06	2.25E-06
W01B6.6	21	34	13	13	2.80E-06	2.65E-06	1.82E-06	2.25E-06
W01B6.7	68	92	39	60	2.80E-06	2.65E-06	1.82E-06	2.25E-06
W01B6.8	5	4	7	2	2.80E-06	2.91E-06	1.82E-06	2.25E-06
W01B6.9.1	499	670	531	855	8.12E-06	1.04E-05	3.02E-06	5.76E-06
W01B6.9.2	458	623	492	811	2.80E-06	2.65E-06	1.82E-06	2.25E-06
W01C8.3	87	94	90	147	2.94E-05	3.73E-05	2.04E-05	4.05E-05
W01C8.4a	20	25	17	37	2.88E-05	3.70E-05	2.01E-05	4.09E-05
W01C8.4b	33	41	32	55	3.42E-06	3.47E-06	2.30E-06	4.61E-06
W01C8.6a	16	23	21	17	2.80E-06	2.65E-06	1.82E-06	2.63E-06
W01C8.6b	16	23	32	22	2.80E-06	2.65E-06	1.82E-06	2.65E-06
W01C9.1	10	18	4	7	2.80E-06	2.65E-06	1.82E-06	2.25E-06
W01C9.2	13	25	22	15	2.80E-06	2.65E-06	1.82E-06	2.25E-06
W01C9.3	62	43	79	77	2.80E-06	2.72E-06	1.82E-06	2.25E-06
W01C9.4	22	45	7	14	2.80E-06	2.65E-06	1.82E-06	2.25E-06
W01C9.5	5	10	7	5	2.80E-06	2.65E-06	2.10E-06	2.52E-06
W01D2.1.1	6482	4861	20435	4080	2.80E-06	5.13E-06	1.82E-06	2.25E-06
W01D2.1.2	6101	4569	19428	3944	2.80E-06	2.65E-06	1.82E-06	2.25E-06
W01D2.1.3	6052	4491	19092	3890	1.97E-03	1.39E-03	4.04E-03	9.95E-04
W01D2.2b	26	44	41	51	2.05E-03	1.45E-03	4.25E-03	1.07E-03
W01D2.3	14	29	22	12	1.99E-03	1.39E-03	4.08E-03	1.03E-03
W01D2.4	6	5	3	3	2.80E-06	3.39E-06	2.17E-06	3.33E-06
W01D2.5	179	225	348	356	2.80E-06	4.71E-06	2.46E-06	2.25E-06
W01D2.6	1	1	3	0	2.80E-06	2.65E-06	1.82E-06	2.25E-06
W01F3.1a	107	238	115	203	1.59E-05	1.89E-05	2.01E-05	2.53E-05
W01F3.1b	78	176	78	157	2.80E-06	2.65E-06	1.82E-06	2.25E-06
W01F3.2	112	243	206	280	6.52E-06	1.37E-05	4.57E-06	9.96E-06
W01F3.3	1001	1119	374	475	6.02E-06	1.29E-05	3.92E-06	9.74E-06
W01G7.1	197	294	254	386	1.29E-05	2.64E-05	1.54E-05	2.59E-05
W01G7.2	1	4	2	5	1.62E-05	1.71E-05	3.95E-06	6.18E-06
W01G7.3	279	190	947	245	8.79E-06	1.24E-05	7.36E-06	1.38E-05
W01G7.4	114	131	253	240	2.80E-06	2.65E-06	1.82E-06	2.25E-06
W01G7.5	139	186	533	692	7.19E-05	4.62E-05	1.59E-04	5.07E-05
W01H2.2	0	0	1	0	1.51E-05	1.64E-05	2.18E-05	2.56E-05
W01H2.3a	16	12	20	23	1.04E-05	1.31E-05	2.58E-05	4.14E-05
W01H2.3b	4	5	2	5	2.80E-06	2.65E-06	1.82E-06	2.25E-06
W01H2.t1	0	0	1	0	2.80E-06	2.65E-06	1.82E-06	2.25E-06
W02A11.1	106	88	180	187	2.80E-06	2.65E-06	1.82E-06	2.25E-06
W02A11.4	179	231	523	737	2.80E-06	2.65E-06	1.82E-06	2.25E-06
W02A11.5	17	25	19	29	1.18E-05	9.23E-06	1.30E-05	1.67E-05
W02A11.6	54	57	50	59	1.57E-05	1.91E-05	2.98E-05	5.19E-05
W02A11.8	6	19	8	13	2.80E-06	2.65E-06	1.82E-06	2.25E-06
W02A2.1	2161	4275	5210	5079	5.82E-06	5.82E-06	3.52E-06	5.13E-06
W02A2.2	449	829	591	800	2.80E-06	2.65E-06	1.82E-06	2.25E-06
W02A2.3	214	213	47	71	1.90E-04	3.54E-04	2.97E-04	3.58E-04
W02A2.4	19	33	26	16	5.60E-05	9.77E-05	4.80E-05	8.01E-05
W02A2.5	6	7	4	2	1.65E-05	1.55E-05	2.35E-06	4.39E-06
W02A2.6	548	465	982	1304	2.80E-06	2.65E-06	1.82E-06	2.25E-06
W02A2.7	4968	3885	6249	7978	2.80E-06	2.65E-06	1.82E-06	2.25E-06
W02A2.8	11	13	6	6	2.52E-05	2.02E-05	2.93E-05	4.81E-05
W02A2.9	7	8	4	5	2.82E-04	2.08E-04	2.31E-04	3.63E-04
W02B12.1	136	140	136	151	3.14E-06	3.49E-06	1.82E-06	2.25E-06
W02B12.10	344	283	512	643	4.51E-06	4.87E-06	1.82E-06	2.59E-06
W02B12.11.1	392	364	566	686	1.31E-05	1.27E-05	8.51E-06	1.16E-05
W02B12.11.2	318	287	455	595	4.64E-05	3.60E-05	4.49E-05	6.96E-05
W02B12.12a	76	145	32	34	2.62E-05	2.30E-05	2.46E-05	3.68E-05
W02B12.12b	80	151	33	35	2.96E-05	2.52E-05	2.76E-05	4.45E-05
W02B12.3a	500	609	607	630	7.70E-06	1.39E-05	2.11E-06	2.77E-06

W02B12.3c	185	215	306	248	8.09E-06	1.44E-05	2.17E-06	2.83E-06
W02B12.4	49	56	37	35	5.03E-05	5.78E-05	3.97E-05	5.09E-05
W02B12.7	34	39	10	8	3.98E-05	4.37E-05	4.29E-05	4.29E-05
W02B12.8a	324	377	538	693	2.91E-06	3.12E-06	1.82E-06	2.25E-06
W02B12.8b.1	159	156	240	342	2.80E-06	2.83E-06	1.82E-06	2.25E-06
W02B12.8b.2	299	328	468	627	2.44E-05	2.68E-05	2.64E-05	4.20E-05
W02B12.9	225	294	211	318	2.35E-05	2.18E-05	2.31E-05	4.07E-05
W02B3.2	31	34	65	86	2.50E-05	2.59E-05	2.55E-05	4.21E-05
W02B3.3	3	2	3	1	1.92E-05	2.37E-05	1.17E-05	2.18E-05
W02B3.4	6	11	16	12	2.80E-06	2.65E-06	2.22E-06	3.62E-06
W02B3.5	3	4	5	3	2.80E-06	2.65E-06	1.82E-06	2.25E-06
W02B3.6	1	2	1	3	2.80E-06	2.65E-06	1.82E-06	2.25E-06
W02B3.7	3	0	1	0	2.80E-06	2.65E-06	1.82E-06	2.25E-06
W02B8.1	24	40	19	27	2.80E-06	2.65E-06	1.82E-06	2.25E-06
W02B8.2	146	106	179	213	2.80E-06	2.65E-06	1.82E-06	2.25E-06
W02B8.3	34	26	17	23	4.23E-06	6.67E-06	2.19E-06	3.82E-06
W02B8.4	19	17	20	8	4.26E-06	2.91E-06	3.39E-06	4.99E-06
W02B8.5	4	5	5	0	2.80E-06	2.65E-06	1.82E-06	2.25E-06
W02B8.6	15	13	11	7	2.80E-06	2.65E-06	1.82E-06	2.25E-06
W02B9.1a	1316	1140	1792	2534	2.80E-06	2.65E-06	1.82E-06	2.25E-06
W02B9.2	8	2	2	6	2.80E-06	2.65E-06	1.82E-06	2.25E-06
W02C12.1	30	42	30	48	3.28E-05	2.68E-05	2.90E-05	5.07E-05
W02C12.2	14	20	20	14	2.80E-06	2.65E-06	1.82E-06	2.25E-06
W02C12.3a.1	105	133	129	166	2.80E-06	2.65E-06	1.82E-06	2.25E-06
W02C12.3a.2	104	131	126	166	2.80E-06	3.09E-06	2.13E-06	2.25E-06
W02C12.3b	115	139	128	168	7.56E-06	9.02E-06	6.03E-06	9.58E-06
W02C12.3c	121	149	132	177	7.50E-06	8.91E-06	5.90E-06	9.60E-06
W02C12.3d.1	241	322	257	370	8.37E-06	9.55E-06	6.05E-06	9.81E-06
W02C12.3d.2	121	149	132	177	8.40E-06	9.76E-06	5.96E-06	9.85E-06
W02C12.3e.1	115	139	128	168	1.05E-05	1.32E-05	7.27E-06	1.29E-05
W02C12.3f	96	119	119	156	8.40E-06	9.76E-06	5.96E-06	9.85E-06
W02C12.3g.1	223	307	253	357	8.37E-06	9.55E-06	6.05E-06	9.81E-06
W02C12.3g.2	90	108	112	130	7.56E-06	8.86E-06	6.10E-06	9.87E-06
W02C12.3h.1	101	125	123	162	9.60E-06	1.25E-05	7.09E-06	1.23E-05
W02C12.3h.2	105	133	129	166	7.34E-06	8.33E-06	5.96E-06	8.52E-06
W02C12.3h.3	106	141	123	169	7.67E-06	8.97E-06	6.07E-06	9.87E-06
W02C12.3h.4	121	149	132	177	7.56E-06	9.02E-06	6.03E-06	9.58E-06
W02C12.3h.5	104	131	126	166	8.51E-06	1.07E-05	6.43E-06	1.09E-05
W02C12.3h.6	92	117	109	148	8.40E-06	9.76E-06	5.96E-06	9.85E-06
W02D3.1.1	812	714	388	482	7.50E-06	8.91E-06	5.90E-06	9.60E-06
W02D3.1.2	698	640	320	402	9.27E-06	1.11E-05	7.14E-06	1.20E-05
W02D3.10a	389	379	503	781	1.65E-04	1.37E-04	5.12E-05	7.86E-05
W02D3.10b	245	250	348	531	1.41E-04	1.22E-04	4.21E-05	6.53E-05
W02D3.11a	301	335	398	556	1.01E-05	9.26E-06	8.47E-06	1.62E-05
W02D3.11b	281	322	362	515	9.60E-06	9.26E-06	8.87E-06	1.67E-05
W02D3.12	69	103	110	76	1.92E-05	2.02E-05	1.65E-05	2.85E-05
W02D3.2	307	483	323	471	2.02E-05	2.19E-05	1.69E-05	2.98E-05
W02D3.3	23	47	33	28	2.18E-05	3.08E-05	2.26E-05	1.93E-05
W02D3.4	398	482	532	591	2.12E-05	3.15E-05	1.45E-05	2.61E-05
W02D3.5.1	1273	2416	4261	1359	2.80E-06	3.49E-06	1.82E-06	2.25E-06
W02D3.5.2	1026	1985	3408	1214	4.17E-05	4.77E-05	3.63E-05	4.98E-05
W02D3.6	8	14	4	8	2.70E-04	4.83E-04	5.87E-04	2.31E-04
W02D3.8	163	158	187	214	2.14E-04	3.92E-04	4.63E-04	2.04E-04
W02D3.9.1	405	502	552	765	2.80E-06	2.65E-06	1.82E-06	2.25E-06
W02D3.9.2	392	492	545	763	1.02E-05	9.34E-06	7.62E-06	1.08E-05
W02D3.9.3	415	510	561	784	2.19E-05	2.56E-05	1.94E-05	3.32E-05
W02D7.1	0	1	5	2	2.11E-05	2.50E-05	1.91E-05	3.30E-05
W02D7.10	5	6	8	3	2.52E-05	2.93E-05	2.22E-05	3.83E-05
W02D7.11	4	1	1	0	2.80E-06	2.65E-06	1.82E-06	2.25E-06

W02D7.12	0	4	0	1	2.80E-06	2.65E-06	1.82E-06	2.25E-06
W02D7.2	84	204	70	227	2.80E-06	2.65E-06	1.82E-06	2.25E-06
W02D7.3	7	13	11	8	2.80E-06	2.65E-06	1.82E-06	2.25E-06
W02D7.4	42	43	77	28	8.79E-06	2.02E-05	4.76E-06	1.91E-05
W02D7.5	18	27	17	24	2.80E-06	3.17E-06	1.86E-06	2.25E-06
W02D7.6	118	147	158	177	6.72E-06	6.51E-06	8.04E-06	3.60E-06
W02D7.7.1	564	1017	626	475	2.88E-06	4.10E-06	1.82E-06	3.08E-06
W02D7.7.2	460	845	523	399	1.19E-05	1.40E-05	1.04E-05	1.44E-05
W02D7.8	3	4	8	3	8.26E-05	1.41E-04	5.96E-05	5.59E-05
W02D7.9	1	0	2	0	8.22E-05	1.43E-04	6.08E-05	5.72E-05
W02D9.1.1	428	429	841	1032	2.80E-06	2.65E-06	1.82E-06	2.25E-06
W02D9.1.2	391	378	772	972	2.80E-06	2.65E-06	1.82E-06	2.25E-06
W02D9.10	38	99	36	39	2.92E-05	2.77E-05	3.74E-05	5.66E-05
W02D9.2	331	429	494	673	2.88E-05	2.63E-05	3.70E-05	5.75E-05
W02D9.3	185	109	208	254	6.13E-06	1.51E-05	3.77E-06	5.06E-06
W02D9.4	73	73	94	78	3.78E-05	4.63E-05	3.67E-05	6.17E-05
W02D9.5	61	57	151	40	1.46E-05	8.09E-06	1.07E-05	1.61E-05
W02D9.6	59	34	252	72	8.04E-06	7.59E-06	6.74E-06	6.90E-06
W02D9.7	304	392	1196	269	1.74E-05	1.53E-05	2.80E-05	9.15E-06
W02D9.8	1	3	0	1	2.53E-05	1.38E-05	7.04E-05	2.48E-05
W02D9.9	0	3	1	0	8.87E-05	1.08E-04	2.27E-04	6.30E-05
W02F12.1	61	83	54	78	2.80E-06	2.65E-06	1.82E-06	2.25E-06
W02F12.2	51	104	29	29	2.80E-06	2.65E-06	1.82E-06	2.25E-06
W02F12.3	866	926	1544	1644	6.83E-06	8.78E-06	3.94E-06	7.02E-06
W02F12.4a	229	278	325	385	5.52E-06	1.06E-05	2.04E-06	2.52E-06
W02F12.4b	76	71	108	106	7.57E-05	7.64E-05	8.78E-05	1.15E-04
W02F12.4c	121	128	192	199	2.05E-05	2.35E-05	1.90E-05	2.77E-05
W02F12.5.1	2244	2258	2202	2882	2.07E-05	1.82E-05	1.91E-05	2.31E-05
W02F12.5.2	1924	1897	1783	2451	2.31E-05	2.31E-05	2.38E-05	3.05E-05
W02F12.6	272	301	451	395	1.53E-04	1.46E-04	9.78E-05	1.58E-04
W02F12.7	4	4	4	1	1.33E-04	1.24E-04	8.01E-05	1.36E-04
W02G9.1	21	26	21	14	4.96E-05	5.19E-05	5.35E-05	5.79E-05
W02G9.2.1	60	159	84	150	2.80E-06	2.65E-06	1.82E-06	2.25E-06
W02G9.2.2	59	163	83	153	3.30E-06	3.86E-06	2.15E-06	2.25E-06
W02G9.2.3	49	153	49	135	2.86E-06	7.17E-06	2.61E-06	5.76E-06
W02G9.3	90	81	185	165	2.94E-06	7.64E-06	2.68E-06	6.09E-06
W02G9.4	76	54	60	47	2.80E-06	7.62E-06	1.82E-06	5.71E-06
W02G9.5	79	75	156	182	9.72E-06	8.25E-06	1.30E-05	1.43E-05
W02G9.t1	0	1	1	1	9.49E-06	6.37E-06	4.88E-06	4.72E-06
W02G9.t2	0	1	1	1	1.01E-05	9.02E-06	1.29E-05	1.86E-05
W02H3.1	20	19	12	19	2.80E-06	2.65E-06	1.82E-06	2.25E-06
W02H3.2	3	1	3	7	2.80E-06	2.65E-06	1.82E-06	2.25E-06
W02H3.t1	0	1	6	0	3.70E-06	3.31E-06	1.82E-06	2.81E-06
W02H5.1	13	9	38	47	2.80E-06	2.65E-06	1.82E-06	2.25E-06
W02H5.10	0	2	2	2	2.80E-06	2.65E-06	5.34E-06	2.25E-06
W02H5.2	59	51	95	120	5.15E-06	3.39E-06	9.82E-06	1.50E-05
W02H5.3	7	18	10	11	2.80E-06	2.65E-06	1.82E-06	2.25E-06
W02H5.4	7	18	10	11	1.09E-05	8.91E-06	1.14E-05	1.78E-05
W02H5.5	5	10	4	9	2.80E-06	2.65E-06	1.82E-06	2.25E-06
W02H5.6	3	6	4	1	2.80E-06	2.65E-06	1.82E-06	2.25E-06
W02H5.7	38	33	39	40	2.80E-06	2.65E-06	1.82E-06	2.25E-06
W02H5.8	24	63	37	47	2.80E-06	2.65E-06	1.82E-06	2.25E-06
W02H5.9	43	46	159	202	3.11E-06	2.65E-06	2.08E-06	2.63E-06
W03A3.1	8	9	7	6	2.80E-06	3.60E-06	1.82E-06	2.29E-06
W03A3.2	430	501	568	898	5.68E-06	5.74E-06	1.37E-05	2.15E-05
W03A5.1	27	53	28	13	2.80E-06	2.65E-06	1.82E-06	2.25E-06
W03A5.2	28	24	16	17	9.46E-06	1.04E-05	8.13E-06	1.59E-05
W03A5.3	14	29	17	17	2.80E-06	2.65E-06	1.82E-06	2.25E-06
W03A5.4.1	249	190	502	607	4.31E-06	3.49E-06	1.82E-06	2.25E-06

W03A5.4.2	131	121	273	400	3.22E-06	6.27E-06	2.53E-06	3.13E-06
W03A5.5	58	53	103	152	1.57E-05	1.13E-05	2.06E-05	3.07E-05
W03A5.6	20	13	31	49	1.48E-05	1.29E-05	2.01E-05	3.63E-05
W03A5.7	119	136	79	77	1.14E-05	9.84E-06	1.32E-05	2.40E-05
W03B1.1	1	0	1	2	6.22E-06	3.81E-06	6.27E-06	1.22E-05
W03B1.2	41	34	35	28	1.78E-05	1.92E-05	7.67E-06	9.24E-06
W03B1.3.1	5	14	6	6	2.80E-06	2.65E-06	1.82E-06	2.25E-06
W03B1.3.2	6	13	7	6	2.80E-06	2.65E-06	1.82E-06	2.25E-06
W03B1.4	165	211	194	326	2.80E-06	2.65E-06	1.82E-06	2.25E-06
W03B1.5	15	32	28	27	2.80E-06	2.65E-06	1.82E-06	2.25E-06
W03B1.6	8	13	4	14	1.33E-05	1.61E-05	1.02E-05	2.11E-05
W03B1.7	4	10	9	8	2.80E-06	2.65E-06	1.82E-06	2.25E-06
W03B1.8	10	16	20	14	2.80E-06	2.65E-06	1.82E-06	2.25E-06
W03B1.9	17	39	28	28	2.80E-06	2.65E-06	1.82E-06	2.25E-06
W03C9.1	13	17	11	9	2.80E-06	2.65E-06	1.82E-06	2.25E-06
W03C9.2	300	257	491	601	2.80E-06	2.65E-06	1.82E-06	2.25E-06
W03C9.3.1	852	730	946	1121	2.80E-06	2.65E-06	1.82E-06	2.25E-06
W03C9.3.2	632	526	627	798	2.42E-05	1.95E-05	2.57E-05	3.89E-05
W03C9.5	91	116	240	98	8.72E-05	7.06E-05	6.30E-05	9.22E-05
W03C9.6.1	25	30	20	30	1.07E-04	8.45E-05	6.94E-05	1.09E-04
W03C9.6.2	24	22	10	27	2.59E-05	3.12E-05	4.45E-05	2.24E-05
W03C9.7.1	1826	1378	2264	2833	2.80E-06	2.65E-06	1.82E-06	2.25E-06
W03C9.7.2	1914	1468	2426	3054	2.80E-06	2.65E-06	1.82E-06	2.25E-06
W03C9.8	13	14	5	2	1.18E-04	8.38E-05	9.49E-05	1.47E-04
W03D2.10	4	7	2	1	1.14E-04	8.26E-05	9.41E-05	1.46E-04
W03D2.1a	23	39	43	37	5.32E-06	5.42E-06	1.82E-06	2.25E-06
W03D2.1b	22	38	41	36	2.80E-06	2.65E-06	1.82E-06	2.25E-06
W03D2.1c	23	38	41	36	2.80E-06	2.65E-06	1.90E-06	2.25E-06
W03D2.2	3	4	5	3	2.80E-06	2.65E-06	1.90E-06	2.25E-06
W03D2.4.1	1812	1543	2112	2293	2.80E-06	2.65E-06	1.82E-06	2.25E-06
W03D2.4.2	1274	1120	1392	1692	2.80E-06	2.65E-06	1.82E-06	2.25E-06
W03D2.5a	18	40	31	23	2.07E-04	1.67E-04	1.57E-04	2.10E-04
W03D2.5b	14	23	17	17	1.82E-04	1.51E-04	1.30E-04	1.94E-04
W03D2.6	6	39	4	20	3.19E-06	6.69E-06	3.57E-06	3.26E-06
W03D2.7	2	1	0	0	2.80E-06	4.23E-06	2.15E-06	2.65E-06
W03D2.9	5	5	2	1	2.80E-06	4.07E-06	1.82E-06	2.25E-06
W03D8.1	13	16	9	10	2.80E-06	2.65E-06	1.82E-06	2.25E-06
W03D8.10	58	69	48	39	2.80E-06	2.65E-06	1.82E-06	2.25E-06
W03D8.11	11	13	7	5	2.80E-06	2.65E-06	1.82E-06	2.25E-06
W03D8.2	22	35	20	14	8.37E-06	9.39E-06	4.50E-06	4.52E-06
W03D8.3	9	5	7	3	2.80E-06	2.65E-06	1.82E-06	2.25E-06
W03D8.5	21	16	11	12	2.80E-06	3.39E-06	1.82E-06	2.25E-06
W03D8.7	12	4	9	1	2.80E-06	2.65E-06	1.82E-06	2.25E-06
W03D8.8	21	40	13	19	4.42E-06	3.20E-06	1.82E-06	2.25E-06
W03D8.9	151	215	188	91	2.80E-06	2.65E-06	1.82E-06	2.25E-06
W03F11.1	796	1044	929	1389	2.80E-06	3.28E-06	1.82E-06	2.25E-06
W03F11.2	7	18	13	10	1.57E-05	2.11E-05	1.27E-05	7.58E-06
W03F11.3	14	21	4	7	1.14E-04	1.41E-04	8.64E-05	1.59E-04
W03F11.4	89	188	58	59	2.80E-06	2.65E-06	1.82E-06	2.25E-06
W03F11.5	8	13	7	6	2.80E-06	3.62E-06	1.82E-06	2.25E-06
W03F11.6a	505	543	566	940	2.80E-06	4.71E-06	1.82E-06	2.25E-06
W03F11.6b	199	214	232	392	2.80E-06	2.65E-06	1.82E-06	2.25E-06
W03F11.6c	400	432	487	797	1.07E-05	1.08E-05	7.78E-06	1.59E-05
W03F11.6d	378	388	440	683	1.09E-05	1.11E-05	8.27E-06	1.72E-05
W03F8.10	256	406	574	669	1.04E-05	1.07E-05	8.27E-06	1.67E-05
W03F8.2	33	39	23	12	1.05E-05	1.02E-05	7.93E-06	1.52E-05
W03F8.3	181	162	199	217	1.41E-05	2.12E-05	2.06E-05	2.96E-05
W03F8.4	628	659	1069	1105	2.80E-06	2.65E-06	1.82E-06	2.25E-06
W03F8.5	320	489	556	1009	1.62E-05	1.37E-05	1.16E-05	1.56E-05

W03F8.9	6	5	5	3	7.84E-05	7.77E-05	8.69E-05	1.11E-04
W03F9.1	252	251	569	673	1.12E-05	1.61E-05	1.26E-05	2.83E-05
W03F9.11	10	10	11	11	2.80E-06	2.65E-06	1.82E-06	2.25E-06
W03F9.2a	60	48	78	89	1.57E-05	1.48E-05	2.31E-05	3.37E-05
W03F9.2b	73	50	90	91	2.80E-06	2.65E-06	1.82E-06	2.25E-06
W03F9.3	1	4	0	1	8.96E-06	6.77E-06	7.58E-06	1.07E-05
W03F9.4	14	23	12	32	1.12E-05	7.27E-06	9.02E-06	1.12E-05
W03F9.5.1	255	283	492	595	2.80E-06	2.65E-06	1.82E-06	2.25E-06
W03F9.5.2	240	265	444	547	2.80E-06	2.65E-06	1.82E-06	2.25E-06
W03F9.5.3	184	212	316	380	2.31E-05	2.42E-05	2.90E-05	4.33E-05
W03F9.6	4	6	10	4	2.21E-05	2.31E-05	2.66E-05	4.05E-05
W03F9.7	2	5	4	0	2.23E-05	2.43E-05	2.49E-05	3.70E-05
W03F9.8	2	0	2	2	2.80E-06	2.65E-06	1.82E-06	2.25E-06
W03F9.9	1	4	6	2	2.80E-06	2.65E-06	1.82E-06	2.25E-06
W03G1.1	22	17	5	8	2.80E-06	2.65E-06	1.82E-06	2.25E-06
W03G1.2	29	33	17	19	2.80E-06	2.65E-06	1.82E-06	2.25E-06
W03G1.3	14	37	24	43	2.80E-06	2.65E-06	1.82E-06	2.25E-06
W03G1.4	47	130	48	235	3.08E-06	3.31E-06	1.82E-06	2.25E-06
W03G1.5	7	15	17	13	2.80E-06	3.15E-06	1.82E-06	3.10E-06
W03G1.6a	455	401	594	813	2.80E-06	5.69E-06	1.82E-06	8.75E-06
W03G1.6b	374	343	492	698	2.80E-06	2.65E-06	1.82E-06	2.25E-06
W03G1.7a	108	43	210	59	2.05E-05	1.70E-05	1.74E-05	2.94E-05
W03G1.7b	115	46	217	61	1.93E-05	1.67E-05	1.65E-05	2.89E-05
W03G1.8	1	2	5	3	6.83E-06	2.65E-06	8.66E-06	2.99E-06
W03G11.1a	5930	7442	8568	12126	7.11E-06	2.70E-06	8.75E-06	3.04E-06
W03G11.2	20	30	20	17	2.80E-06	2.65E-06	1.82E-06	2.25E-06
W03G11.3	50	88	64	85	6.07E-04	7.19E-04	5.70E-04	9.96E-04
W03G11.4.1	105	101	70	125	2.80E-06	2.99E-06	1.82E-06	2.25E-06
W03G11.4.2	97	99	63	121	3.47E-06	5.79E-06	2.90E-06	4.75E-06
W03G11.t1	0	0	3	0	6.08E-06	5.53E-06	2.64E-06	5.80E-06
W03G9.1.1	233	338	275	431	6.19E-06	5.95E-06	2.61E-06	6.18E-06
W03G9.1.2	197	270	214	365	2.80E-06	2.65E-06	2.99E-06	2.25E-06
W03G9.2	200	204	257	385	1.13E-05	1.55E-05	8.67E-06	1.68E-05
W03G9.3	403	286	519	475	1.18E-05	1.52E-05	8.31E-06	1.75E-05
W03G9.4.1	1214	1241	1745	2165	1.45E-05	1.39E-05	1.21E-05	2.24E-05
W03G9.4.2	1081	1143	1626	2034	2.91E-05	1.95E-05	2.44E-05	2.76E-05
W03G9.5	13	20	15	6	6.56E-05	6.33E-05	6.14E-05	9.40E-05
W03G9.6	7	17	16	3	6.47E-05	6.47E-05	6.34E-05	9.79E-05
W03G9.7	13	11	14	3	2.80E-06	2.65E-06	1.82E-06	2.25E-06
W03G9.8	65	75	247	97	2.80E-06	2.65E-06	1.82E-06	2.25E-06
W03G9.9	3	5	18	9	2.80E-06	2.65E-06	1.82E-06	2.25E-06
W03H1.2	1	2	0	1	2.92E-05	3.19E-05	7.23E-05	3.50E-05
W03H9.1	15	20	13	10	2.80E-06	2.65E-06	2.02E-06	2.25E-06
W03H9.2	5	6	11	7	2.80E-06	2.65E-06	1.82E-06	2.25E-06
W03H9.3	1	2	2	6	2.80E-06	2.65E-06	1.82E-06	2.25E-06
W03H9.4	283	289	432	650	2.80E-06	2.65E-06	1.82E-06	2.25E-06
W04A4.2	26	39	26	31	2.80E-06	2.65E-06	1.82E-06	2.25E-06
W04A4.3	3	2	0	0	1.41E-05	1.36E-05	1.40E-05	2.60E-05
W04A4.4	3	3	5	6	4.42E-06	6.27E-06	2.88E-06	4.25E-06
W04A4.5	355	414	467	805	2.80E-06	2.65E-06	1.82E-06	2.25E-06
W04A4.6	20	21	18	8	2.80E-06	2.65E-06	1.82E-06	2.25E-06
W04A8.1	122	104	188	256	1.19E-05	1.31E-05	1.02E-05	2.17E-05
W04A8.2	3	6	3	4	4.00E-06	3.99E-06	2.35E-06	2.25E-06
W04A8.3	11	12	14	24	6.50E-06	5.24E-06	6.52E-06	1.10E-05
W04A8.4	54	92	64	124	2.80E-06	4.15E-06	1.82E-06	2.36E-06
W04A8.5	10	13	11	17	2.80E-06	2.65E-06	1.82E-06	2.25E-06
W04A8.6	339	278	339	512	4.84E-06	7.78E-06	3.72E-06	8.91E-06
W04A8.7	595	537	416	861	2.80E-06	2.65E-06	1.82E-06	2.25E-06
W04B5.1	24	67	41	46	1.71E-05	1.32E-05	1.11E-05	2.07E-05

W04B5.2	16	18	14	14	1.19E-05	1.01E-05	5.41E-06	1.38E-05
W04B5.3a	146	135	165	175	4.26E-06	1.12E-05	4.74E-06	6.57E-06
W04B5.3b	144	133	174	178	2.80E-06	2.65E-06	1.82E-06	2.25E-06
W04B5.4	85	115	115	119	1.28E-05	1.12E-05	9.38E-06	1.23E-05
W04B5.5	94	101	112	177	1.11E-05	9.65E-06	8.71E-06	1.10E-05
W04B5.6	3	9	8	2	1.31E-05	1.68E-05	1.16E-05	1.48E-05
W04C9.1	278	378	251	294	1.08E-05	1.10E-05	8.36E-06	1.63E-05
W04C9.2	258	360	612	266	2.80E-06	2.65E-06	1.82E-06	2.25E-06
W04C9.3	7	13	22	12	1.24E-05	1.59E-05	7.27E-06	1.05E-05
W04C9.4	222	171	120	181	6.52E-05	8.60E-05	1.01E-04	5.40E-05
W04C9.5	3	9	14	5	2.80E-06	2.65E-06	1.82E-06	2.25E-06
W04C9.6	15	13	20	13	4.52E-05	3.29E-05	1.59E-05	2.96E-05
W04D12.1	14	17	1	5	2.80E-06	2.65E-06	1.82E-06	2.25E-06
W04D2.1a	1438	1733	1115	1785	2.80E-06	2.65E-06	1.82E-06	2.25E-06
W04D2.1b	1215	1449	933	1479	6.08E-06	6.98E-06	1.82E-06	2.25E-06
W04D2.2a	5	3	3	0	5.08E-05	5.79E-05	2.57E-05	5.07E-05
W04D2.3	60	104	80	94	5.06E-05	5.70E-05	2.53E-05	4.95E-05
W04D2.4	545	529	723	1001	2.80E-06	2.65E-06	1.82E-06	2.25E-06
W04D2.5.1	210	275	367	264	4.45E-06	7.30E-06	3.86E-06	5.62E-06
W04D2.6a	919	864	923	1360	3.10E-05	2.84E-05	2.67E-05	4.57E-05
W04D2.6b	614	570	640	989	3.12E-05	3.85E-05	3.54E-05	3.15E-05
W04D2.7	1	2	1	1	4.35E-05	3.86E-05	2.84E-05	5.17E-05
W04E12.1	3	1	11	3	3.64E-05	3.19E-05	2.47E-05	4.71E-05
W04E12.2	13	7	14	12	2.80E-06	3.15E-06	1.82E-06	2.25E-06
W04E12.3	1	4	4	2	2.80E-06	2.65E-06	1.82E-06	2.25E-06
W04E12.4	16	13	4	5	2.80E-06	2.65E-06	1.82E-06	2.25E-06
W04E12.5	6	20	10	1	2.80E-06	2.65E-06	1.82E-06	2.25E-06
W04E12.6	371	499	571	643	2.80E-06	2.65E-06	1.82E-06	2.25E-06
W04E12.7	51	51	41	25	2.80E-06	2.99E-06	1.82E-06	2.25E-06
W04E12.8	1634	2063	2785	2390	4.09E-05	5.19E-05	4.09E-05	5.69E-05
W04E12.9	5	4	8	3	9.02E-06	8.52E-06	4.72E-06	3.55E-06
W04G3.1	224	399	152	97	1.77E-04	2.11E-04	1.96E-04	2.08E-04
W04G3.10	2	1	1	0	2.80E-06	2.65E-06	1.82E-06	2.25E-06
W04G3.11	6	3	1	4	2.04E-05	3.43E-05	9.00E-06	7.08E-06
W04G3.12	3	1	1	3	2.80E-06	2.65E-06	1.82E-06	2.25E-06
W04G3.2	473	463	286	161	2.80E-06	2.65E-06	1.82E-06	2.25E-06
W04G3.3	276	413	178	99	2.80E-06	2.65E-06	1.82E-06	2.25E-06
W04G3.4	32	20	22	29	2.46E-05	2.27E-05	9.66E-06	6.70E-06
W04G3.5	251	385	307	367	2.29E-05	3.24E-05	9.62E-06	6.61E-06
W04G3.6a.1	26	36	31	37	2.80E-06	2.65E-06	1.82E-06	2.25E-06
W04G3.6a.2	31	54	54	61	1.97E-05	2.85E-05	1.57E-05	2.31E-05
W04G3.6a.3	28	39	31	42	3.47E-06	4.55E-06	2.70E-06	3.96E-06
W04G3.6b.1	31	54	54	61	3.02E-06	5.00E-06	3.44E-06	4.79E-06
W04G3.6b.2	28	39	31	42	3.50E-06	4.60E-06	2.51E-06	4.23E-06
W04G3.6c	18	47	42	50	3.02E-06	5.00E-06	3.44E-06	4.79E-06
W04G3.6d	61	101	84	111	3.50E-06	4.60E-06	2.51E-06	4.23E-06
W04G3.7	9	6	5	7	2.80E-06	4.81E-06	2.95E-06	4.34E-06
W04G5.10	16	21	7	16	3.44E-06	5.37E-06	3.08E-06	5.02E-06
W04G5.11	0	3	0	1	2.80E-06	2.65E-06	1.82E-06	2.25E-06
W04G5.2	15	12	3	3	2.80E-06	2.65E-06	1.82E-06	2.25E-06
W04G5.3	5	5	8	2	2.80E-06	2.65E-06	1.82E-06	2.25E-06
W04G5.4	8	12	14	10	2.83E-06	2.65E-06	1.82E-06	2.25E-06
W04G5.5	2	4	3	3	2.80E-06	2.65E-06	2.66E-06	2.25E-06
W04G5.6	5	5	10	7	2.80E-06	2.65E-06	1.82E-06	2.25E-06
W04G5.7	0	3	1	0	2.80E-06	2.65E-06	1.82E-06	2.25E-06
W04G5.8	3	5	1	2	2.80E-06	2.65E-06	1.82E-06	2.25E-06
W04G5.9	6	6	5	6	2.80E-06	2.65E-06	1.82E-06	2.25E-06
W04H10.1	2	4	1	2	2.80E-06	2.65E-06	1.82E-06	2.25E-06
W04H10.2	9	6	6	3	2.80E-06	2.65E-06	1.82E-06	2.25E-06

W04H10.3a	40	70	50	78	2.80E-06	2.65E-06	1.82E-06	2.25E-06
W04H10.3b.	42	74	77	87	2.80E-06	2.65E-06	1.82E-06	2.25E-06
W04H10.3b.	40	65	49	82	2.80E-06	3.57E-06	1.82E-06	3.37E-06
W04H10.3b.	38	58	43	74	2.80E-06	3.57E-06	2.55E-06	3.55E-06
W04H10.4	3	11	0	12	2.80E-06	3.17E-06	1.82E-06	3.42E-06
W05B10.1	793	665	1150	1156	2.80E-06	3.25E-06	1.82E-06	3.53E-06
W05B10.2	156	156	184	200	2.80E-06	2.65E-06	1.82E-06	2.25E-06
W05B10.3	4	2	1	5	1.54E-04	1.22E-04	1.45E-04	1.80E-04
W05B10.4	11	25	10	13	2.60E-05	2.45E-05	1.99E-05	2.67E-05
W05B10.5	4	4	17	6	2.80E-06	2.65E-06	1.82E-06	2.25E-06
W05B2.1	6630	7068	9583	9398	2.80E-06	3.41E-06	1.82E-06	2.25E-06
W05B2.2	9	19	10	7	2.80E-06	2.65E-06	1.82E-06	2.25E-06
W05B2.3	6	13	4	3	7.66E-04	7.72E-04	7.21E-04	8.73E-04
W05B2.4	20	27	26	25	2.80E-06	2.65E-06	1.82E-06	2.25E-06
W05B2.5	8303	8411	12163	11886	2.80E-06	2.65E-06	1.82E-06	2.25E-06
W05B2.6	6330	6387	9074	8575	2.80E-06	2.65E-06	1.82E-06	2.25E-06
W05B2.7	8	21	11	15	9.19E-04	8.79E-04	8.76E-04	1.06E-03
W05B2.8	0	2	4	1	7.26E-04	6.92E-04	6.78E-04	7.90E-04
W05B5.1	121	97	89	100	2.80E-06	2.65E-06	1.82E-06	2.25E-06
W05B5.2	8	9	13	12	2.80E-06	2.65E-06	2.86E-06	2.25E-06
W05B5.3a	21	45	14	26	2.15E-05	1.63E-05	1.03E-05	1.43E-05
W05B5.3b.1	13	33	10	22	2.80E-06	2.65E-06	1.82E-06	2.25E-06
W05B5.3b.2	21	45	14	26	2.80E-06	2.94E-06	1.82E-06	2.25E-06
W05B5.4	6	15	12	10	2.80E-06	2.65E-06	1.82E-06	2.25E-06
W05E10.1.1	43	55	37	49	2.80E-06	2.94E-06	1.82E-06	2.25E-06
W05E10.1.2	37	54	35	49	3.30E-06	7.78E-06	4.28E-06	4.41E-06
W05E10.2	0	3	1	2	2.94E-06	3.57E-06	1.82E-06	2.70E-06
W05E10.3.1	54	82	38	80	2.80E-06	3.76E-06	1.82E-06	2.88E-06
W05E10.3.2	40	59	22	66	2.80E-06	2.65E-06	1.82E-06	2.25E-06
W05E10.4	186	273	238	262	3.25E-06	4.66E-06	1.82E-06	3.85E-06
W05E10.5	9	15	3	3	3.33E-06	4.63E-06	1.82E-06	4.41E-06
W05E7.1	267	393	655	427	1.09E-05	1.52E-05	9.13E-06	1.24E-05
W05E7.2	2	3	2	5	2.80E-06	2.65E-06	1.82E-06	2.25E-06
W05E7.3	120	133	143	66	4.42E-05	6.14E-05	7.05E-05	5.67E-05
W05F2.2	247	178	321	317	2.80E-06	2.65E-06	1.82E-06	2.25E-06
W05F2.3	2178	1877	2695	2690	2.51E-05	2.63E-05	1.94E-05	1.11E-05
W05F2.4a	361	472	220	355	1.75E-05	1.19E-05	1.48E-05	1.80E-05
W05F2.4b	201	261	95	202	3.03E-04	2.47E-04	2.44E-04	3.01E-04
W05F2.5	25	35	22	30	6.02E-06	7.41E-06	2.39E-06	4.75E-06
W05F2.6	402	346	685	797	8.23E-06	1.01E-05	2.53E-06	6.63E-06
W05F2.7	60	66	77	54	2.86E-06	3.76E-06	1.82E-06	2.74E-06
W05G11.2	25	33	18	24	3.07E-05	2.49E-05	3.40E-05	4.88E-05
W05G11.3.1	231	241	152	114	2.80E-06	2.65E-06	1.82E-06	2.25E-06
W05G11.3.2	203	218	138	107	4.84E-06	6.03E-06	2.26E-06	3.73E-06
W05G11.4	9	8	7	10	2.84E-05	2.80E-05	1.22E-05	1.13E-05
W05G11.5	1	6	5	5	2.50E-05	2.53E-05	1.11E-05	1.06E-05
W05G11.6a.	403	666	574	933	2.80E-06	2.65E-06	1.82E-06	2.25E-06
W05G11.6a.	404	674	576	935	2.80E-06	2.65E-06	1.82E-06	2.25E-06
W05G11.6b.	403	666	574	933	2.30E-05	3.59E-05	2.13E-05	4.28E-05
W05G11.6b.	379	614	538	893	2.31E-05	3.64E-05	2.14E-05	4.29E-05
W05G11.6b.	392	653	556	927	2.30E-05	3.59E-05	2.13E-05	4.28E-05
W05G11.6c	269	441	398	632	2.26E-05	3.46E-05	2.09E-05	4.28E-05
W05G11.6d.	427	724	620	960	2.24E-05	3.53E-05	2.07E-05	4.26E-05
W05G11.6d.	362	603	518	842	2.07E-05	3.21E-05	1.99E-05	3.91E-05
W05G11.6d.	363	611	520	844	2.18E-05	3.49E-05	2.06E-05	3.93E-05
W05H12.1	79	92	78	63	2.27E-05	3.57E-05	2.11E-05	4.23E-05
W05H12.2	11	16	8	10	2.27E-05	3.61E-05	2.12E-05	4.24E-05
W05H5.1	0	1	3	0	5.54E-06	6.08E-06	3.55E-06	3.55E-06
W05H5.2	1	1	3	1	2.80E-06	2.65E-06	1.82E-06	2.25E-06

W05H5.3	7	7	12	3	2.80E-06	2.65E-06	1.82E-06	2.25E-06
W05H5.4	3	8	4	3	2.80E-06	2.65E-06	1.82E-06	2.25E-06
W05H5.5	7	1	3	3	2.80E-06	2.65E-06	1.82E-06	2.25E-06
W05H5.6	4	4	5	2	2.80E-06	2.65E-06	1.82E-06	2.25E-06
W05H5.7	3	3	6	3	2.80E-06	2.65E-06	1.82E-06	2.25E-06
W05H5.8	0	5	5	0	2.80E-06	2.65E-06	1.82E-06	2.25E-06
W05H7.1	21	29	20	21	2.80E-06	2.65E-06	1.82E-06	2.25E-06
W05H7.2	36	46	68	32	2.80E-06	2.65E-06	1.82E-06	2.25E-06
W05H7.3	20	12	44	21	3.84E-06	5.00E-06	2.39E-06	3.08E-06
W05H7.4a	92	85	126	147	1.22E-05	1.48E-05	1.50E-05	8.73E-06
W05H7.4b	130	119	184	178	4.28E-06	2.65E-06	6.12E-06	3.60E-06
W05H7.4c	25	28	34	25	5.66E-06	4.95E-06	5.05E-06	7.26E-06
W05H7.4d	16	14	18	13	6.38E-06	5.53E-06	5.89E-06	7.02E-06
W05H7.4e	118	108	170	173	3.47E-06	3.68E-06	3.08E-06	2.79E-06
W05H9.1.1	321	763	425	426	5.57E-06	4.60E-06	4.08E-06	3.62E-06
W05H9.1.2	300	717	387	411	5.85E-06	5.05E-06	5.48E-06	6.88E-06
W05H9.2	116	143	190	262	2.70E-05	6.06E-05	2.33E-05	2.88E-05
W05H9.4	177	172	243	357	2.70E-05	6.09E-05	2.26E-05	2.97E-05
W06A11.1	12	18	15	14	4.42E-06	5.13E-06	4.70E-06	8.01E-06
W06A11.2	23	12	29	23	7.73E-06	7.09E-06	6.91E-06	1.25E-05
W06A11.3	8	17	8	9	2.80E-06	3.33E-06	1.91E-06	2.25E-06
W06A11.4	31	30	30	38	2.80E-06	2.65E-06	1.82E-06	2.25E-06
W06A7.2	118	148	145	110	2.80E-06	2.65E-06	1.82E-06	2.25E-06
W06A7.3a	3874	3643	3713	6175	3.33E-06	3.04E-06	2.10E-06	3.28E-06
W06A7.3c	3772	3574	3663	6109	2.49E-05	2.95E-05	1.99E-05	1.86E-05
W06A7.3d	3618	3372	3481	5910	5.55E-05	4.93E-05	3.46E-05	7.10E-05
W06A7.3e	2726	2399	2572	4347	5.42E-05	4.85E-05	3.42E-05	7.04E-05
W06A7.4	868	813	804	1385	2.28E-04	2.01E-04	1.43E-04	2.99E-04
W06A7.5	89	179	92	57	4.48E-04	3.72E-04	2.75E-04	5.73E-04
W06B11.1	17	29	18	20	1.26E-04	1.11E-04	7.57E-05	1.61E-04
W06B11.2	399	493	349	582	6.39E-05	1.21E-04	4.30E-05	3.29E-05
W06B11.3	123	138	121	135	2.80E-06	3.41E-06	1.82E-06	2.25E-06
W06B11.4	25	45	5	5	1.35E-05	1.58E-05	7.69E-06	1.58E-05
W06B3.1	14	24	11	15	1.69E-05	1.80E-05	1.08E-05	1.49E-05
W06B3.2a	87	160	58	101	4.76E-06	8.09E-06	1.82E-06	2.25E-06
W06B3.2b	108	208	70	116	2.80E-06	3.84E-06	1.82E-06	2.25E-06
W06B3.2c	91	181	61	106	6.50E-06	1.13E-05	2.82E-06	6.07E-06
W06B3.2d	116	234	72	126	6.41E-06	1.17E-05	2.71E-06	5.53E-06
W06B4.1	567	495	902	1058	6.66E-06	1.25E-05	2.90E-06	6.23E-06
W06B4.2	76	92	60	82	7.87E-06	1.50E-05	3.17E-06	6.86E-06
W06B4.3	217	215	307	462	4.24E-05	3.50E-05	4.39E-05	6.36E-05
W06D11.1	32	48	81	74	5.15E-06	5.90E-06	2.64E-06	4.48E-06
W06D11.2	12	7	4	9	8.04E-06	7.54E-06	7.40E-06	1.38E-05
W06D11.3	77	105	112	84	5.04E-06	7.14E-06	8.31E-06	9.36E-06
W06D11.4	5	19	7	7	3.14E-06	2.65E-06	1.82E-06	2.25E-06
W06D11.5	3	1	2	0	2.05E-05	2.65E-05	1.94E-05	1.80E-05
W06D12.1	3	4	23	3	2.80E-06	2.65E-06	1.82E-06	2.25E-06
W06D12.2	31	26	36	42	2.80E-06	2.65E-06	1.82E-06	2.25E-06
W06D12.3	38	194	105	220	2.80E-06	2.65E-06	1.82E-06	2.25E-06
W06D12.4	2	4	9	7	2.80E-06	2.65E-06	1.95E-06	2.81E-06
W06D12.5	20	49	19	48	4.06E-06	1.95E-05	7.29E-06	1.88E-05
W06D12.6	1	1	3	0	2.80E-06	2.65E-06	1.82E-06	2.25E-06
W06D12.7	5	2	13	15	2.80E-06	2.72E-06	1.82E-06	2.27E-06
W06D12.t1	0	0	1	0	2.80E-06	2.65E-06	1.82E-06	2.25E-06
W06D12.t2	1	0	0	0	2.80E-06	2.65E-06	1.82E-06	2.25E-06
W06D4.1	403	530	391	477	2.80E-06	2.65E-06	1.82E-06	2.25E-06
W06D4.2	60	115	30	37	2.80E-06	2.65E-06	1.82E-06	2.25E-06
W06D4.3	12	29	15	9	3.01E-05	3.74E-05	1.90E-05	2.86E-05
W06D4.4.1	220	311	194	305	8.93E-06	1.62E-05	2.90E-06	4.43E-06

W06D4.4.2	214	303	178	275	2.80E-06	3.76E-06	1.82E-06	2.25E-06
W06D4.5	88	77	258	242	1.26E-05	1.68E-05	7.23E-06	1.40E-05
W06D4.6	394	314	452	615	1.26E-05	1.68E-05	6.81E-06	1.30E-05
W06D4.7	0	3	8	14	2.02E-05	1.67E-05	3.84E-05	4.45E-05
W06D4.8	0	1	6	12	1.67E-05	1.25E-05	1.24E-05	2.09E-05
W06E11.1	107	123	272	402	2.80E-06	2.78E-06	5.12E-06	1.10E-05
W06E11.2	108	96	85	91	2.80E-06	2.65E-06	3.97E-06	9.81E-06
W06E11.4	274	307	222	307	8.26E-06	8.97E-06	1.36E-05	2.49E-05
W06E11.5a	86	68	101	189	2.05E-05	1.72E-05	1.05E-05	1.39E-05
W06E11.5b	69	65	59	113	3.16E-05	3.35E-05	1.67E-05	2.85E-05
W06E11.7	37	27	79	37	1.58E-05	1.18E-05	1.21E-05	2.79E-05
W06F12.1a	420	430	493	603	1.85E-05	1.64E-05	1.03E-05	2.43E-05
W06F12.1b.1	297	323	377	490	8.68E-06	5.98E-06	1.21E-05	6.97E-06
W06F12.1b.2	508	564	595	903	2.34E-05	2.26E-05	1.78E-05	2.69E-05
W06F12.1c	265	294	343	457	2.15E-05	2.21E-05	1.77E-05	2.85E-05
W06F12.1d	685	734	737	1057	2.78E-05	2.92E-05	2.12E-05	3.97E-05
W06F12.1e.1	362	381	431	544	1.85E-05	1.94E-05	1.56E-05	2.56E-05
W06F12.1e.2	508	564	595	903	2.67E-05	2.71E-05	1.87E-05	3.32E-05
W06F12.2a	50	102	34	89	2.32E-05	2.30E-05	1.79E-05	2.80E-05
W06F12.2b	69	121	57	101	2.78E-05	2.92E-05	2.12E-05	3.97E-05
W06F12.2c	62	106	43	84	3.19E-06	6.14E-06	1.82E-06	4.54E-06
W06F12.3	4	8	3	7	3.92E-06	6.48E-06	2.10E-06	4.59E-06
W06G6.1	11	14	13	6	4.06E-06	6.59E-06	1.84E-06	4.43E-06
W06G6.10	2	2	2	1	2.80E-06	2.65E-06	1.82E-06	2.25E-06
W06G6.11	7	6	19	8	2.80E-06	2.65E-06	1.82E-06	2.25E-06
W06G6.12	3	2	7	3	2.80E-06	2.65E-06	1.82E-06	2.25E-06
W06G6.13	4	7	14	4	2.80E-06	2.65E-06	3.32E-06	2.25E-06
W06G6.2	0	2	1	0	2.80E-06	2.65E-06	1.82E-06	2.25E-06
W06G6.3	3	4	1	4	2.80E-06	2.65E-06	1.82E-06	2.25E-06
W06G6.4	4	3	4	2	2.80E-06	2.65E-06	1.82E-06	2.25E-06
W06G6.6	5	9	2	5	2.80E-06	2.65E-06	1.82E-06	2.25E-06
W06G6.7	2	6	6	3	2.80E-06	2.65E-06	1.82E-06	2.25E-06
W06G6.8	5	4	6	4	2.80E-06	2.65E-06	1.82E-06	2.25E-06
W06G6.9	3	2	1	0	2.80E-06	2.65E-06	1.82E-06	2.25E-06
W06H3.1	498	492	581	772	2.80E-06	2.65E-06	1.82E-06	2.25E-06
W06H3.2	249	256	326	355	2.80E-06	2.65E-06	1.82E-06	2.25E-06
W06H3.3	254	345	860	1197	2.69E-05	2.51E-05	2.04E-05	3.35E-05
W06H8.1a	2107	2114	2239	3201	2.02E-05	1.96E-05	1.72E-05	2.31E-05
W06H8.1b	2479	2547	2619	3973	1.41E-05	1.81E-05	3.10E-05	5.33E-05
W06H8.1c	2442	2448	2589	3862	9.48E-05	8.98E-05	6.55E-05	1.16E-04
W06H8.1d	2403	2410	2558	3810	9.78E-05	9.49E-05	6.72E-05	1.26E-04
W06H8.1e	1938	1920	2039	2906	1.24E-04	1.18E-04	8.56E-05	1.58E-04
W06H8.1f.1	2026	2047	2148	3091	1.28E-04	1.21E-04	8.84E-05	1.63E-04
W06H8.1f.2	1945	1923	2038	2911	1.17E-04	1.09E-04	8.00E-05	1.41E-04
W06H8.1f.3	1937	1911	2030	2890	1.33E-04	1.27E-04	9.20E-05	1.63E-04
W06H8.2	9	10	10	8	1.28E-04	1.20E-04	8.74E-05	1.54E-04
W06H8.4	6	13	5	5	1.17E-04	1.09E-04	7.96E-05	1.40E-04
W06H8.5	871	1104	870	909	2.80E-06	2.65E-06	1.82E-06	2.25E-06
W06H8.6	132	192	132	190	2.80E-06	2.65E-06	1.82E-06	2.25E-06
W06H8.7	3	2	4	3	3.86E-05	4.63E-05	2.51E-05	3.24E-05
W06H8.8a	26	28	28	31	1.46E-05	2.00E-05	9.49E-06	1.69E-05
W06H8.8b	25	27	28	30	2.80E-06	2.65E-06	1.82E-06	2.25E-06
W06H8.8c	180	188	142	194	2.80E-06	2.65E-06	1.82E-06	2.25E-06
W06H8.8d	13347	9826	675	1968	2.80E-06	2.65E-06	1.82E-06	2.25E-06
W06H8.8e	14368	11132	1412	3108	2.80E-06	2.65E-06	1.82E-06	2.25E-06
W06H8.8f	14369	11137	1413	3109	4.71E-05	3.27E-05	1.82E-06	5.58E-06
W06H8.8g	14370	11140	1410	3109	2.88E-05	2.11E-05	1.84E-06	4.99E-06
W07A12.4	17	26	17	9	2.90E-05	2.12E-05	1.86E-06	5.04E-06
W07A12.5	10	14	3	2	2.88E-05	2.11E-05	1.84E-06	4.99E-06

W07A12.6	10	22	12	28	2.80E-06	2.65E-06	1.82E-06	2.25E-06
W07A12.7	82	146	95	214	2.80E-06	2.65E-06	1.82E-06	2.25E-06
W07A12.8	10	13	19	17	2.80E-06	2.65E-06	1.82E-06	2.25E-06
W07A8.1	4	2	5	1	5.54E-06	9.31E-06	4.17E-06	1.16E-05
W07A8.2a	464	535	715	1032	2.80E-06	3.02E-06	3.04E-06	3.35E-06
W07A8.2b	447	534	687	1005	2.80E-06	2.65E-06	1.82E-06	2.25E-06
W07A8.3.1	509	444	505	769	1.56E-05	1.70E-05	1.57E-05	2.79E-05
W07A8.3.2	494	438	502	760	1.59E-05	1.80E-05	1.59E-05	2.87E-05
W07A8.4	5	8	3	2	2.12E-05	1.75E-05	1.37E-05	2.58E-05
W07A8.5	5	0	4	0	2.05E-05	1.72E-05	1.36E-05	2.54E-05
W07B3.2a.1	695	740	594	885	2.80E-06	2.65E-06	1.82E-06	2.25E-06
W07B3.2a.2	414	421	323	426	2.80E-06	2.65E-06	1.82E-06	2.25E-06
W07B3.2b	451	461	345	469	3.40E-05	3.41E-05	1.89E-05	3.47E-05
W07B3.2c.1	556	561	425	625	2.02E-05	1.94E-05	1.02E-05	1.67E-05
W07B3.2c.2	461	452	348	482	2.91E-05	2.81E-05	1.45E-05	2.43E-05
W07B3.2d.1	259	268	208	329	3.18E-05	3.03E-05	1.58E-05	2.87E-05
W07B3.2d.2	293	281	239	324	3.12E-05	2.89E-05	1.53E-05	2.62E-05
W07B3.2e.1	159	168	121	142	2.91E-05	2.84E-05	1.52E-05	2.97E-05
W07B3.2e.2	158	164	119	140	2.62E-05	2.38E-05	1.39E-05	2.33E-05
W07B3.2f	431	443	340	450	3.20E-05	3.20E-05	1.59E-05	2.30E-05
W07B8.1	30	27	24	28	3.21E-05	3.14E-05	1.57E-05	2.28E-05
W07B8.3	33	24	14	19	2.80E-05	2.72E-05	1.44E-05	2.35E-05
W07B8.4	2	6	3	5	3.25E-06	2.75E-06	1.82E-06	2.43E-06
W07E11.1	816	984	845	1239	3.89E-06	2.67E-06	1.82E-06	2.25E-06
W07E11.2	38	64	38	29	2.80E-06	2.65E-06	1.82E-06	2.25E-06
W07E11.3a	14	25	34	14	1.34E-05	1.53E-05	9.07E-06	1.64E-05
W07E11.3b	20	28	35	15	7.08E-06	1.13E-05	4.61E-06	4.34E-06
W07E11.4	64	84	64	54	2.80E-06	4.50E-06	4.23E-06	2.25E-06
W07E6.2	160	183	178	239	4.51E-06	5.98E-06	5.14E-06	2.72E-06
W07E6.3	8	12	13	12	3.32E-05	4.12E-05	2.16E-05	2.25E-05
W07E6.5	24	27	33	34	1.17E-05	1.27E-05	8.49E-06	1.41E-05
W07G1.1	13	23	16	12	2.80E-06	2.65E-06	1.82E-06	2.25E-06
W07G1.10	1	1	0	2	3.70E-06	3.94E-06	3.32E-06	4.21E-06
W07G1.2	5	3	1	2	2.80E-06	2.78E-06	1.82E-06	2.25E-06
W07G1.3	158	183	194	271	2.80E-06	2.65E-06	1.82E-06	2.25E-06
W07G1.5a	18	20	18	11	2.80E-06	2.65E-06	1.82E-06	2.25E-06
W07G1.5b	18	20	19	10	1.45E-05	1.59E-05	1.16E-05	2.00E-05
W07G1.6	4	9	8	3	2.80E-06	2.65E-06	1.82E-06	2.25E-06
W07G1.7	2	0	4	0	2.80E-06	2.65E-06	1.82E-06	2.25E-06
W07G1.8	0	3	1	1	2.80E-06	2.65E-06	1.82E-06	2.25E-06
W07G1.9	0	3	1	1	2.80E-06	2.65E-06	1.82E-06	2.25E-06
W07G4.1	1	3	23	7	2.80E-06	2.65E-06	1.82E-06	2.25E-06
W07G4.2	6	3	6	7	2.80E-06	2.65E-06	1.82E-06	2.25E-06
W07G4.3.1	559	524	932	1300	2.80E-06	2.65E-06	4.39E-06	2.25E-06
W07G4.3.2	558	521	932	1292	2.80E-06	2.65E-06	1.82E-06	2.25E-06
W07G4.3.3	554	512	932	1289	2.38E-05	2.11E-05	2.58E-05	4.44E-05
W07G4.4	2624	2376	2991	4208	2.38E-05	2.09E-05	2.58E-05	4.42E-05
W07G4.5a	149	315	150	176	2.42E-05	2.12E-05	2.65E-05	4.53E-05
W07G4.5b	162	330	154	180	1.44E-04	1.23E-04	1.07E-04	1.85E-04
W07G4.6	28	37	17	15	1.32E-05	2.65E-05	8.69E-06	1.26E-05
W07G4.t1	1	0	3	0	1.71E-05	3.29E-05	1.06E-05	1.52E-05
W07G9.2	17	50	15	31	2.80E-06	2.67E-06	1.82E-06	2.25E-06
W08A12.1a	415	305	339	562	2.80E-06	2.65E-06	2.99E-06	2.25E-06
W08A12.1c	621	458	547	858	2.80E-06	5.79E-06	1.82E-06	3.06E-06
W08A12.1d	415	305	339	562	3.00E-05	2.08E-05	1.59E-05	3.26E-05
W08A12.3	13	10	14	12	3.86E-05	2.69E-05	2.21E-05	4.28E-05
W08A12.4	6	9	12	4	3.00E-05	2.08E-05	1.59E-05	3.26E-05
W08D2.1	36	63	73	45	2.80E-06	2.65E-06	1.82E-06	2.25E-06
W08D2.3a	10	14	17	12	2.80E-06	2.65E-06	1.82E-06	2.25E-06

W08D2.3b	13	14	19	12	2.80E-06	3.60E-06	2.88E-06	2.25E-06
W08D2.5	1099	1083	1302	2033	2.80E-06	2.65E-06	1.82E-06	2.25E-06
W08D2.6	9	16	11	14	2.80E-06	2.65E-06	1.82E-06	2.25E-06
W08D2.7	763	853	984	1541	2.97E-05	2.76E-05	2.29E-05	4.41E-05
W08D2.8	13	28	11	7	2.80E-06	2.65E-06	1.82E-06	2.25E-06
W08D2.9	156	245	723	106	2.67E-05	2.82E-05	2.24E-05	4.32E-05
W08E12.1	62	66	58	55	2.80E-06	2.65E-06	1.82E-06	2.25E-06
W08E12.2	11	8	2	0	1.21E-04	1.80E-04	3.66E-04	6.62E-05
W08E12.3	19	17	2	2	5.77E-06	5.79E-06	3.52E-06	4.12E-06
W08E12.4	31	28	6	4	3.39E-06	2.65E-06	1.82E-06	2.25E-06
W08E12.5	22	23	5	3	4.56E-06	3.86E-06	1.82E-06	2.25E-06
W08E12.6	4	3	4	0	8.15E-06	6.96E-06	1.82E-06	2.25E-06
W08E12.7.1	5401	5089	6102	8319	5.80E-06	5.71E-06	1.82E-06	2.25E-06
W08E12.7.2	3401	3153	3653	5199	2.80E-06	2.65E-06	1.82E-06	2.25E-06
W08E12.9	56	63	168	215	3.56E-04	3.17E-04	2.62E-04	4.41E-04
W08E3.2	280	306	325	399	3.21E-04	2.81E-04	2.24E-04	3.94E-04
W08E3.3	2160	2013	1201	1490	1.87E-05	1.98E-05	3.64E-05	5.76E-05
W08E3.4	17	31	5	7	1.57E-05	1.62E-05	1.19E-05	1.80E-05
W08F4.1	1	0	0	0	1.73E-04	1.52E-04	6.25E-05	9.57E-05
W08F4.10	1	0	1	0	4.96E-06	8.54E-06	1.82E-06	2.25E-06
W08F4.11	5	9	5	4	2.80E-06	2.65E-06	1.82E-06	2.25E-06
W08F4.12	3	4	4	3	2.80E-06	2.65E-06	1.82E-06	2.25E-06
W08F4.13	9	9	14	2	2.80E-06	2.80E-06	1.82E-06	2.25E-06
W08F4.2	7	5	8	10	2.80E-06	2.65E-06	1.82E-06	2.25E-06
W08F4.3.1	481	525	428	464	2.80E-06	2.65E-06	1.82E-06	2.25E-06
W08F4.3.2	367	387	296	354	2.80E-06	2.65E-06	1.82E-06	2.25E-06
W08F4.5	2	6	4	1	6.17E-05	6.36E-05	3.57E-05	4.78E-05
W08F4.6	592	747	132	167	5.82E-05	5.80E-05	3.06E-05	4.51E-05
W08F4.7	1	1	2	2	2.80E-06	2.65E-06	1.82E-06	2.25E-06
W08F4.8a	638	513	582	723	6.58E-05	7.84E-05	9.55E-06	1.49E-05
W08F4.9	0	3	3	2	2.80E-06	2.65E-06	1.82E-06	2.25E-06
W08G11.1	8	19	7	6	6.38E-05	4.85E-05	3.79E-05	5.81E-05
W08G11.3a	167	104	225	304	2.80E-06	2.65E-06	1.82E-06	2.25E-06
W08G11.3b	127	86	142	262	2.80E-06	2.65E-06	1.82E-06	2.25E-06
W08G11.4	986	854	1013	1557	9.80E-06	5.77E-06	8.60E-06	1.43E-05
W08G11.5	3	2	6	2	9.16E-06	5.85E-06	6.67E-06	1.52E-05
W08G11.6	6	2	3	6	4.10E-05	3.35E-05	2.74E-05	5.20E-05
W09B12.1.1	36	69	36	56	2.80E-06	2.65E-06	1.82E-06	2.25E-06
W09B12.1.2	15	48	24	37	4.90E-06	2.65E-06	1.82E-06	3.94E-06
W09B6.1a.1	2703	3367	2855	4462	2.80E-06	2.88E-06	1.82E-06	2.25E-06
W09B6.1a.2	2638	3286	2765	4297	2.80E-06	2.65E-06	1.82E-06	2.25E-06
W09B6.1b	755	1113	882	1406	4.65E-05	5.47E-05	3.19E-05	6.16E-05
W09B6.3	155	163	159	245	4.58E-05	5.38E-05	3.12E-05	5.98E-05
W09B6.4b	12	7	18	25	3.42E-05	4.77E-05	2.60E-05	5.12E-05
W09B6.5	20	34	40	42	9.80E-06	9.73E-06	6.54E-06	1.25E-05
W09B7.1	6	3	5	8	4.82E-06	2.65E-06	4.70E-06	8.05E-06
W09B7.2	8	54	7	30	2.80E-06	4.42E-06	3.59E-06	4.66E-06
W09B7.3	2	6	8	4	2.80E-06	2.65E-06	1.82E-06	2.25E-06
W09C2.1b	53	76	79	104	2.80E-06	6.00E-06	1.82E-06	2.83E-06
W09C2.3a	794	943	680	1210	2.80E-06	2.65E-06	1.82E-06	2.25E-06
W09C2.3b	971	1111	844	1536	4.70E-06	6.37E-06	4.57E-06	7.42E-06
W09C2.3c	967	1114	843	1534	1.99E-05	2.24E-05	1.11E-05	2.44E-05
W09C3.1	63	94	61	59	2.49E-05	2.70E-05	1.41E-05	3.17E-05
W09C3.2	16	33	15	7	2.44E-05	2.66E-05	1.39E-05	3.11E-05
W09C3.3	3	4	4	5	5.35E-06	7.54E-06	3.37E-06	4.03E-06
W09C3.4	129	158	132	179	2.80E-06	2.65E-06	1.82E-06	2.25E-06
W09C3.6	169	255	120	85	2.80E-06	2.65E-06	1.82E-06	2.25E-06
W09C3.7	116	193	114	80	1.48E-05	1.71E-05	9.82E-06	1.64E-05
W09C3.8	10	24	17	9	1.80E-05	2.57E-05	8.35E-06	7.29E-06

W09C5.1.1	516	710	636	774	2.37E-05	3.73E-05	1.52E-05	1.31E-05
W09C5.1.2	430	576	506	670	2.80E-06	2.70E-06	1.82E-06	2.25E-06
W09C5.3	7	8	5	7	6.00E-05	7.80E-05	4.81E-05	7.23E-05
W09C5.4	14	19	11	2	5.88E-05	7.44E-05	4.50E-05	7.36E-05
W09C5.5	78	117	62	117	2.80E-06	2.65E-06	1.82E-06	2.25E-06
W09C5.6a.1	8153	5569	18977	5520	4.17E-06	5.37E-06	2.13E-06	2.25E-06
W09C5.6a.2	6657	4677	14977	5028	3.81E-06	5.40E-06	1.97E-06	4.59E-06
W09C5.6b.1	4131	2907	9965	3688	2.07E-03	1.33E-03	3.13E-03	1.12E-03
W09C5.6b.2	4594	3280	10619	3832	1.69E-03	1.12E-03	2.48E-03	1.03E-03
W09C5.6b.3	4088	2863	9931	3640	1.21E-03	8.07E-04	1.91E-03	8.71E-04
W09C5.6b.4	5222	3698	11393	3959	1.13E-03	7.63E-04	1.70E-03	7.58E-04
W09C5.6b.5	4326	3033	10105	3728	1.29E-03	8.51E-04	2.03E-03	9.20E-04
W09C5.6b.6	6657	4677	14977	5028	1.50E-03	1.00E-03	2.13E-03	9.13E-04
W09C5.7	405	410	530	711	1.57E-03	1.04E-03	2.39E-03	1.09E-03
W09C5.8.1	2017	2061	2462	2265	1.69E-03	1.12E-03	2.48E-03	1.03E-03
W09C5.8.2	1569	1644	1883	1866	1.80E-05	1.72E-05	1.53E-05	2.54E-05
W09C5.9	0	0	0	3	2.63E-04	2.54E-04	2.09E-04	2.37E-04
W09D10.1.1	548	560	792	1063	2.78E-04	2.75E-04	2.17E-04	2.66E-04
W09D10.1.2	404	392	598	810	2.80E-06	2.65E-06	1.82E-06	2.25E-06
W09D10.2	1142	1099	1524	2056	3.30E-05	3.18E-05	3.10E-05	5.14E-05
W09D10.3	255	323	346	204	3.02E-05	2.77E-05	2.91E-05	4.87E-05
W09D10.4	364	341	596	567	2.90E-05	2.63E-05	2.51E-05	4.19E-05
W09D10.5	47	70	60	45	2.70E-05	3.23E-05	2.38E-05	1.73E-05
W09D12.1.1	173	263	179	221	2.12E-05	1.88E-05	2.26E-05	2.65E-05
W09D12.1.2	181	263	181	217	5.46E-06	7.67E-06	4.52E-06	4.18E-06
W09D12.2	6	4	12	4	1.31E-05	1.88E-05	8.82E-06	1.34E-05
W09D12.3	18	12	62	41	1.37E-05	1.88E-05	8.91E-06	1.32E-05
W09D6.1a	56	63	75	113	2.80E-06	2.65E-06	1.82E-06	2.25E-06
W09D6.1b	58	64	75	114	2.80E-06	2.65E-06	4.87E-06	3.96E-06
W09D6.2	5	5	3	1	2.80E-06	2.65E-06	2.08E-06	3.85E-06
W09D6.3	1	8	12	1	2.80E-06	2.65E-06	2.02E-06	3.80E-06
W09D6.4	31	83	14	15	2.80E-06	2.65E-06	1.82E-06	2.25E-06
W09D6.5	256	335	219	268	2.80E-06	2.65E-06	1.82E-06	2.25E-06
W09D6.6	376	378	360	591	7.76E-06	1.97E-05	2.28E-06	3.01E-06
W09D6.7	0	1	6	11	2.85E-05	3.52E-05	1.59E-05	2.40E-05
W09D6.8	2	1	3	1	1.48E-05	1.41E-05	9.24E-06	1.87E-05
W09E7.1	3	7	3	2	2.80E-06	2.65E-06	3.90E-06	8.84E-06
W09G10.1	6	5	7	4	2.80E-06	2.65E-06	1.95E-06	2.25E-06
W09G10.3	9	24	14	7	2.80E-06	2.65E-06	1.82E-06	2.25E-06
W09G10.4a	410	396	361	596	2.80E-06	2.65E-06	1.82E-06	2.25E-06
W09G10.4b	322	278	266	453	2.80E-06	2.65E-06	1.82E-06	2.25E-06
W09G10.5	4	6	10	5	1.13E-05	1.03E-05	6.49E-06	1.32E-05
W09G10.6	5	5	14	3	1.18E-05	9.63E-06	6.36E-06	1.34E-05
W09G10.t2	0	0	3	0	2.80E-06	2.65E-06	1.82E-06	2.25E-06
W09G10.t3	0	1	0	1	2.80E-06	2.65E-06	1.82E-06	2.25E-06
W09G12.1	14	28	13	14	2.80E-06	2.65E-06	2.99E-06	2.25E-06
W09G12.4	3	5	2	4	2.80E-06	2.65E-06	1.82E-06	2.25E-06
W09G12.5	71	62	196	107	2.80E-06	2.94E-06	1.82E-06	2.25E-06
W09G12.6	4	6	8	2	2.80E-06	2.65E-06	1.82E-06	2.25E-06
W09G12.7	37	54	24	29	8.15E-06	6.72E-06	1.46E-05	9.87E-06
W09G12.8	5	9	10	6	2.80E-06	2.65E-06	1.82E-06	2.25E-06
W09G12.9	23	24	31	40	3.75E-06	5.16E-06	1.82E-06	2.36E-06
W09G3.1a	45	57	44	51	2.80E-06	2.65E-06	1.82E-06	2.25E-06
W09G3.1b	16	9	31	20	6.22E-06	6.14E-06	5.47E-06	8.68E-06
W09G3.3	101	124	166	187	5.15E-06	6.19E-06	3.28E-06	4.70E-06
W09G3.5	19	27	32	25	4.14E-06	2.65E-06	5.25E-06	4.18E-06
W09G3.6	393	346	607	823	1.83E-05	2.12E-05	1.96E-05	2.72E-05
W09G3.7a	75	83	116	171	2.80E-06	2.65E-06	1.82E-06	2.25E-06
W09G3.7b	18	27	34	48	1.24E-05	1.03E-05	1.24E-05	2.08E-05

W09G3.8.1	147	187	194	135	7.36E-06	7.70E-06	7.42E-06	1.35E-05
W09G3.8.2	139	174	180	114	5.38E-06	7.62E-06	6.61E-06	1.15E-05
W09H1.1a	5	9	5	0	1.51E-05	1.81E-05	1.30E-05	1.11E-05
W09H1.1b	4	7	8	1	1.62E-05	1.92E-05	1.37E-05	1.07E-05
W09H1.2	10	13	12	20	2.80E-06	2.65E-06	1.82E-06	2.25E-06
W09H1.3	24	31	37	51	2.80E-06	2.65E-06	1.82E-06	2.25E-06
W09H1.4	3	7	8	5	2.83E-06	3.47E-06	2.20E-06	4.54E-06
W09H1.5	232	254	323	325	8.46E-06	1.03E-05	8.47E-06	1.44E-05
W09H1.6a	2640	2718	2854	3502	2.80E-06	2.65E-06	1.82E-06	2.25E-06
W09H1.6b	2433	2501	2521	3295	2.24E-05	2.32E-05	2.03E-05	2.52E-05
W10C4.1	8	7	11	6	2.68E-04	2.61E-04	1.89E-04	2.86E-04
W10C6.1	747	834	1225	1695	2.91E-04	2.83E-04	1.96E-04	3.17E-04
W10C6.2	0	1	1	0	2.80E-06	2.65E-06	1.82E-06	2.25E-06
W10C8.1	3	1	5	2	1.88E-05	1.98E-05	2.00E-05	3.42E-05
W10C8.2	75	79	144	243	2.80E-06	2.65E-06	1.82E-06	2.25E-06
W10C8.3	1	3	2	1	2.80E-06	2.65E-06	1.82E-06	2.25E-06
W10C8.4a	14	18	14	15	6.38E-06	6.35E-06	7.96E-06	1.66E-05
W10C8.4b	4	11	17	4	2.80E-06	2.65E-06	1.82E-06	2.25E-06
W10C8.5.1	788	1290	1098	729	2.80E-06	2.65E-06	1.82E-06	2.25E-06
W10C8.5.2	722	1209	1015	695	2.80E-06	2.65E-06	1.93E-06	2.25E-06
W10C8.5.3	684	1162	927	671	6.60E-05	1.02E-04	5.99E-05	4.91E-05
W10C8.6	9	14	9	0	6.41E-05	1.01E-04	5.86E-05	4.95E-05
W10D5.1	35	88	50	78	6.15E-05	9.87E-05	5.43E-05	4.85E-05
W10D5.2.1	922	934	929	1038	3.95E-06	5.82E-06	2.57E-06	2.25E-06
W10D5.2.2	625	614	558	739	3.64E-06	8.65E-06	3.39E-06	6.50E-06
W10D5.3a	725	730	979	1305	9.47E-05	9.07E-05	6.21E-05	8.57E-05
W10D5.3c	803	843	1097	1461	1.15E-04	1.07E-04	6.70E-05	1.10E-04
W10D5.3d	709	719	952	1278	3.92E-05	3.73E-05	3.45E-05	5.67E-05
W10D5.3e	743	776	994	1329	3.34E-05	3.31E-05	2.97E-05	4.88E-05
W10D5.3f	920	948	1258	1600	3.85E-05	3.68E-05	3.36E-05	5.57E-05
W10D5.3g	749	763	1067	1384	3.55E-05	3.50E-05	3.09E-05	5.10E-05
W10D9.1	3	5	9	6	3.06E-05	2.98E-05	2.72E-05	4.27E-05
W10D9.2	10	4	10	6	3.70E-05	3.56E-05	3.43E-05	5.50E-05
W10D9.3	74	111	169	100	2.80E-06	2.65E-06	1.82E-06	2.25E-06
W10D9.4	249	232	351	462	2.80E-06	2.65E-06	1.82E-06	2.25E-06
W10D9.5.1	297	342	251	208	6.89E-06	9.76E-06	1.02E-05	7.49E-06
W10D9.5.2	242	283	191	176	1.78E-05	1.56E-05	1.63E-05	2.65E-05
W10G11.1	4	5	0	2	6.96E-05	7.57E-05	3.83E-05	3.92E-05
W10G11.10	4	3	2	2	5.63E-05	6.22E-05	2.89E-05	3.29E-05
W10G11.11	4	4	2	2	2.80E-06	2.65E-06	1.82E-06	2.25E-06
W10G11.12	3	1	1	1	2.80E-06	2.65E-06	1.82E-06	2.25E-06
W10G11.13	1	2	0	1	2.80E-06	2.65E-06	1.82E-06	2.25E-06
W10G11.14	2	3	1	0	2.80E-06	2.65E-06	1.82E-06	2.25E-06
W10G11.15	1	2	2	0	2.80E-06	2.65E-06	1.82E-06	2.25E-06
W10G11.16	18	21	22	41	2.80E-06	2.65E-06	1.82E-06	2.25E-06
W10G11.17	32	34	30	60	2.80E-06	2.65E-06	1.82E-06	2.25E-06
W10G11.19	39	36	40	63	2.80E-06	2.65E-06	1.82E-06	3.76E-06
W10G11.2	2	7	11	5	2.80E-06	2.72E-06	1.82E-06	4.09E-06
W10G11.3	26	29	13	8	2.80E-06	2.65E-06	1.84E-06	3.58E-06
W10G11.4	3	0	3	4	2.80E-06	2.65E-06	1.82E-06	2.25E-06
W10G11.5	5	3	2	1	4.62E-06	4.87E-06	1.82E-06	2.25E-06
W10G11.6	2	4	1	0	2.80E-06	2.65E-06	1.82E-06	2.25E-06
W10G11.7	3	0	0	1	2.80E-06	2.65E-06	1.82E-06	2.25E-06
W10G11.8	8	3	1	9	2.80E-06	2.65E-06	1.82E-06	2.25E-06
W10G11.9	7	4	2	3	2.80E-06	2.65E-06	1.82E-06	2.25E-06
W10G11.t1	0	0	3	0	2.80E-06	2.65E-06	1.82E-06	2.25E-06
W10G11.t2	0	1	1	1	2.80E-06	2.65E-06	1.82E-06	2.25E-06
W10G6.1	1	1	1	0	2.80E-06	2.65E-06	2.99E-06	2.25E-06
W10G6.2a.1	118	199	82	109	2.80E-06	2.65E-06	1.82E-06	2.25E-06

W10G6.2a.2	105	166	69	96	2.80E-06	2.65E-06	1.82E-06	2.25E-06
W10G6.2b	112	182	70	97	8.26E-06	1.31E-05	3.74E-06	6.12E-06
W10G6.3.1	820	1309	975	1697	8.60E-06	1.28E-05	3.66E-06	6.30E-06
W10G6.3.2	747	1171	870	1579	9.02E-06	1.38E-05	3.66E-06	6.27E-06
Y102A11A.1	15	20	13	17	4.72E-05	7.12E-05	3.66E-05	7.85E-05
Y102A11A.2a	40	72	39	56	4.78E-05	7.08E-05	3.62E-05	8.11E-05
Y102A11A.2b	35	62	37	54	2.80E-06	2.65E-06	1.82E-06	2.25E-06
Y102A11A.3	363	401	316	636	2.80E-06	3.28E-06	1.82E-06	2.25E-06
Y102A11A.6	16	17	15	19	2.80E-06	2.88E-06	1.82E-06	2.25E-06
Y102A11A.7	13	11	8	3	7.90E-06	8.23E-06	4.46E-06	1.11E-05
Y102A11A.8	37	47	41	50	2.80E-06	2.65E-06	1.82E-06	2.25E-06
Y102A5A.1	1470	1187	2276	3264	2.80E-06	2.65E-06	1.82E-06	2.25E-06
Y102A5B.1	7	7	4	7	2.80E-06	2.65E-06	1.82E-06	2.25E-06
Y102A5B.2	2	2	4	0	3.82E-05	2.92E-05	3.86E-05	6.82E-05
Y102A5B.3	5	11	12	4	2.80E-06	2.65E-06	1.82E-06	2.25E-06
Y102A5C.1	148	122	125	203	2.80E-06	2.65E-06	1.82E-06	2.25E-06
Y102A5C.10	2	0	2	4	2.80E-06	2.65E-06	1.82E-06	2.25E-06
Y102A5C.11	4	2	6	9	1.49E-05	1.16E-05	8.18E-06	1.64E-05
Y102A5C.12	2	0	2	4	2.80E-06	2.65E-06	1.82E-06	2.25E-06
Y102A5C.13	2	1	1	2	2.80E-06	2.65E-06	1.82E-06	2.25E-06
Y102A5C.14	3	6	4	6	2.80E-06	2.65E-06	1.82E-06	2.25E-06
Y102A5C.15	8	3	4	3	2.80E-06	2.65E-06	1.82E-06	2.25E-06
Y102A5C.16	3	9	2	1	2.80E-06	2.65E-06	1.82E-06	2.25E-06
Y102A5C.17	2	9	4	5	2.80E-06	2.65E-06	1.82E-06	2.25E-06
Y102A5C.19	16	17	12	15	2.80E-06	2.65E-06	1.82E-06	2.25E-06
Y102A5C.2	46	33	35	44	2.80E-06	2.65E-06	1.82E-06	2.25E-06
Y102A5C.20	10	21	29	29	2.80E-06	2.65E-06	1.82E-06	2.25E-06
Y102A5C.21	6	10	2	6	8.71E-06	5.90E-06	4.32E-06	6.70E-06
Y102A5C.22	2	3	2	1	2.80E-06	2.65E-06	1.82E-06	2.25E-06
Y102A5C.23	6	6	10	5	2.80E-06	2.65E-06	1.82E-06	2.25E-06
Y102A5C.24	1	1	3	2	2.80E-06	2.65E-06	1.82E-06	2.25E-06
Y102A5C.25	1	3	9	2	2.80E-06	2.65E-06	1.82E-06	2.25E-06
Y102A5C.26	15	8	12	6	2.80E-06	2.65E-06	1.82E-06	2.25E-06
Y102A5C.27	1	1	0	1	2.80E-06	2.65E-06	1.82E-06	2.25E-06
Y102A5C.28	5	6	5	3	2.80E-06	2.65E-06	1.82E-06	2.25E-06
Y102A5C.29	3	8	3	5	2.80E-06	2.65E-06	1.82E-06	2.25E-06
Y102A5C.3	15	26	12	26	2.80E-06	2.65E-06	1.82E-06	2.25E-06
Y102A5C.30	5	5	5	1	2.80E-06	2.65E-06	1.82E-06	2.25E-06
Y102A5C.31	3	1	3	1	2.80E-06	2.70E-06	1.82E-06	2.29E-06
Y102A5C.32	2	2	6	3	2.80E-06	2.65E-06	1.82E-06	2.25E-06
Y102A5C.33	6	6	12	5	2.80E-06	2.65E-06	1.82E-06	2.25E-06
Y102A5C.34	0	2	1	0	2.80E-06	2.65E-06	1.82E-06	2.25E-06
Y102A5C.35	3	4	9	10	2.80E-06	2.65E-06	1.82E-06	2.25E-06
Y102A5C.36	7	45	32	55	2.80E-06	2.65E-06	1.82E-06	2.25E-06
Y102A5C.4	8	6	10	3	2.80E-06	2.65E-06	1.82E-06	2.25E-06
Y102A5C.5	26	104	43	146	2.80E-06	7.41E-06	3.63E-06	7.71E-06
Y102A5C.6	153	340	74	362	2.80E-06	2.65E-06	1.82E-06	2.25E-06
Y102A5C.7	2	4	2	3	5.96E-06	2.25E-05	6.41E-06	2.69E-05
Y102A5C.8	4	2	5	6	1.91E-05	4.01E-05	6.01E-06	3.63E-05
Y102A5C.9	1	1	1	2	2.80E-06	2.65E-06	1.82E-06	2.25E-06
Y102A5D.1	3	6	1	1	2.80E-06	2.65E-06	1.82E-06	2.25E-06
Y102E9.1a.1	376	394	404	511	2.80E-06	2.65E-06	1.82E-06	2.25E-06
Y102E9.1a.2	352	371	376	490	2.80E-06	2.65E-06	1.82E-06	2.25E-06
Y102E9.1a.3	364	383	386	499	2.70E-05	2.67E-05	1.89E-05	2.94E-05
Y102E9.1a.4	363	380	383	498	2.93E-05	2.92E-05	2.04E-05	3.28E-05
Y102E9.1b	367	387	379	496	2.82E-05	2.81E-05	1.95E-05	3.11E-05
Y102E9.1c	392	394	410	520	3.01E-05	2.98E-05	2.07E-05	3.32E-05
Y102E9.3	1	2	2	0	2.58E-05	2.57E-05	1.74E-05	2.80E-05
Y102E9.5	14	31	32	34	2.93E-05	2.78E-05	1.99E-05	3.12E-05

Y102E9.6	0	3	0	0	2.80E-06	2.65E-06	1.82E-06	2.25E-06
Y102E9.t1	0	0	1	0	2.80E-06	5.66E-06	4.03E-06	5.29E-06
Y102F5A.1	3	6	3	6	2.80E-06	2.65E-06	1.82E-06	2.25E-06
Y104H12A.1	72	91	58	76	2.80E-06	2.65E-06	1.82E-06	2.25E-06
Y104H12BL.1	139	117	246	365	2.80E-06	2.65E-06	1.82E-06	2.25E-06
Y104H12BR.1	173	135	385	538	3.81E-06	4.55E-06	1.99E-06	3.22E-06
Y104H12D.1	62	81	117	94	1.32E-05	1.05E-05	1.52E-05	2.78E-05
Y104H12D.2	158	167	101	132	3.63E-05	2.67E-05	5.26E-05	9.06E-05
Y104H12D.3	95	138	315	314	1.16E-05	1.44E-05	1.43E-05	1.42E-05
Y104H12D.4	85	97	90	60	1.69E-05	1.68E-05	7.02E-06	1.13E-05
Y105C5A.1	91	74	113	142	4.79E-06	6.59E-06	1.03E-05	1.27E-05
Y105C5A.10	0	1	1	0	1.01E-05	1.09E-05	6.98E-06	5.74E-06
Y105C5A.11	11	5	2	1	3.86E-06	2.99E-06	3.13E-06	4.86E-06
Y105C5A.12	35	11	176	6	2.80E-06	2.65E-06	1.82E-06	2.25E-06
Y105C5A.13a	81	29	540	10	2.80E-06	2.65E-06	1.82E-06	2.25E-06
Y105C5A.13b	35	11	176	6	1.70E-05	5.03E-06	5.55E-05	2.34E-06
Y105C5A.14	20	40	20	24	2.36E-05	7.99E-06	1.02E-04	2.34E-06
Y105C5A.15	74	96	66	102	1.70E-05	5.03E-06	5.55E-05	2.34E-06
Y105C5A.17	7	6	15	4	2.80E-06	4.42E-06	1.82E-06	2.25E-06
Y105C5A.18	14	13	8	10	3.11E-06	3.78E-06	1.82E-06	3.42E-06
Y105C5A.19	3	8	2	4	2.80E-06	2.65E-06	1.82E-06	2.25E-06
Y105C5A.2	12	6	14	12	2.80E-06	2.65E-06	1.82E-06	2.25E-06
Y105C5A.20	3	8	2	4	2.80E-06	2.65E-06	1.82E-06	2.25E-06
Y105C5A.21	8	6	6	3	2.80E-06	2.65E-06	1.82E-06	2.25E-06
Y105C5A.22	1	3	0	3	2.80E-06	2.65E-06	1.82E-06	2.25E-06
Y105C5A.23	35	32	41	45	2.80E-06	2.65E-06	1.82E-06	2.25E-06
Y105C5A.24	50	79	41	74	2.80E-06	2.65E-06	1.82E-06	2.25E-06
Y105C5A.25	29	44	31	25	2.80E-06	2.65E-06	2.08E-06	2.81E-06
Y105C5A.26	3	2	0	1	3.42E-06	5.11E-06	1.82E-06	4.07E-06
Y105C5A.27	4	1	3	1	3.11E-06	4.44E-06	2.15E-06	2.25E-06
Y105C5A.3	13	20	3	6	2.80E-06	2.65E-06	1.82E-06	2.25E-06
Y105C5A.4	13	19	5	4	2.80E-06	2.65E-06	1.82E-06	2.25E-06
Y105C5A.5	13	20	4	5	2.80E-06	2.65E-06	1.82E-06	2.25E-06
Y105C5A.6	13	19	3	6	2.80E-06	2.65E-06	1.82E-06	2.25E-06
Y105C5A.7	32	33	28	40	2.80E-06	2.65E-06	1.82E-06	2.25E-06
Y105C5A.8a	50	132	48	84	2.80E-06	2.65E-06	1.82E-06	2.25E-06
Y105C5A.9a	1	1	0	2	2.80E-06	2.65E-06	1.82E-06	2.25E-06
Y105C5A.9b	0	0	0	1	7.67E-06	1.92E-05	4.79E-06	1.04E-05
Y105C5A.t1	0	0	3	0	2.80E-06	2.65E-06	1.82E-06	2.25E-06
Y105C5A.t2	0	0	1	0	2.80E-06	2.65E-06	1.82E-06	2.25E-06
Y105C5B.1	6	7	21	7	2.80E-06	2.65E-06	2.99E-06	2.25E-06
Y105C5B.10	0	3	2	5	2.80E-06	2.65E-06	1.82E-06	2.25E-06
Y105C5B.11	10	15	12	8	2.80E-06	2.65E-06	1.82E-06	2.25E-06
Y105C5B.12a	413	543	507	886	2.80E-06	2.65E-06	1.82E-06	2.25E-06
Y105C5B.12b	443	597	873	1008	2.80E-06	2.65E-06	1.82E-06	2.25E-06
Y105C5B.13	26	40	30	21	3.46E-05	4.29E-05	2.76E-05	5.95E-05
Y105C5B.14	3	2	3	4	3.57E-05	4.54E-05	4.58E-05	6.52E-05
Y105C5B.15	242	375	381	673	4.48E-06	6.48E-06	3.35E-06	2.90E-06
Y105C5B.16	3	4	3	1	2.80E-06	2.65E-06	1.82E-06	2.25E-06
Y105C5B.17	5	11	6	4	2.09E-05	3.06E-05	2.14E-05	4.66E-05
Y105C5B.18	15	37	19	11	2.80E-06	2.65E-06	1.82E-06	2.25E-06
Y105C5B.19	71	78	102	95	2.80E-06	2.65E-06	1.82E-06	2.25E-06
Y105C5B.2	5	7	18	11	3.14E-06	7.33E-06	2.59E-06	2.25E-06
Y105C5B.20	24	19	6	23	5.10E-06	5.29E-06	4.77E-06	5.49E-06
Y105C5B.21a	144	158	135	212	2.80E-06	2.65E-06	1.82E-06	2.25E-06
Y105C5B.21b	169	187	152	243	4.00E-06	3.02E-06	1.82E-06	3.10E-06
Y105C5B.21b	137	153	125	204	4.28E-06	4.44E-06	2.61E-06	5.06E-06
Y105C5B.23	2	7	7	8	4.68E-06	4.89E-06	2.75E-06	5.42E-06
Y105C5B.24	0	3	1	1	4.54E-06	4.76E-06	2.68E-06	5.40E-06

Y105C5B.25	3	10	5	6	2.80E-06	2.65E-06	1.82E-06	2.25E-06
Y105C5B.28a	610	899	729	564	2.80E-06	2.65E-06	1.82E-06	2.25E-06
Y105C5B.28b	579	801	694	519	2.80E-06	2.65E-06	1.82E-06	2.25E-06
Y105C5B.29	1	4	0	1	4.79E-05	6.67E-05	3.73E-05	3.56E-05
Y105C5B.3	11	10	9	9	5.00E-05	6.53E-05	3.90E-05	3.60E-05
Y105C5B.30	5	2	5	4	2.80E-06	2.65E-06	1.82E-06	2.25E-06
Y105C5B.4	10	8	18	10	2.80E-06	2.65E-06	1.82E-06	2.25E-06
Y105C5B.5	213	250	501	184	2.80E-06	2.65E-06	1.82E-06	2.25E-06
Y105C5B.6a	11	10	4	6	2.80E-06	2.65E-06	1.82E-06	2.25E-06
Y105C5B.6b	7	4	2	4	4.89E-05	5.42E-05	7.48E-05	3.39E-05
Y105C5B.7	5	9	5	6	2.80E-06	2.65E-06	1.82E-06	2.25E-06
Y105C5B.8	10	15	16	5	2.80E-06	2.65E-06	1.82E-06	2.25E-06
Y105C5B.9	35	55	30	27	2.80E-06	2.65E-06	1.82E-06	2.25E-06
Y105E8A.1	19	21	14	11	2.80E-06	2.65E-06	1.82E-06	2.25E-06
Y105E8A.10a	287	392	315	529	4.51E-06	6.72E-06	2.51E-06	2.81E-06
Y105E8A.10b	266	364	295	504	3.08E-06	3.23E-06	1.82E-06	2.25E-06
Y105E8A.10c	262	360	288	503	1.07E-05	1.38E-05	7.65E-06	1.59E-05
Y105E8A.10c	271	364	295	507	1.04E-05	1.35E-05	7.53E-06	1.59E-05
Y105E8A.11	99	79	378	90	1.06E-05	1.37E-05	7.58E-06	1.63E-05
Y105E8A.13	23	33	15	19	1.07E-05	1.36E-05	7.58E-06	1.61E-05
Y105E8A.14	219	146	465	403	2.76E-05	2.08E-05	6.85E-05	2.02E-05
Y105E8A.15	2	4	0	0	2.80E-06	3.57E-06	1.82E-06	2.25E-06
Y105E8A.16.[illegible]	7341	8109	10185	5040	3.76E-05	2.36E-05	5.19E-05	5.55E-05
Y105E8A.16.[illegible]	5758	6650	7663	4578	2.80E-06	2.65E-06	1.82E-06	2.25E-06
Y105E8A.17	409	364	459	759	9.76E-04	1.02E-03	8.82E-04	5.38E-04
Y105E8A.19	595	682	489	862	7.69E-04	8.39E-04	6.66E-04	4.91E-04
Y105E8A.2	248	351	348	509	2.70E-05	2.27E-05	1.97E-05	4.02E-05
Y105E8A.20.[illegible]	111	120	95	158	2.75E-05	2.97E-05	1.47E-05	3.20E-05
Y105E8A.20.[illegible]	103	110	91	150	1.50E-05	2.01E-05	1.37E-05	2.48E-05
Y105E8A.21.[illegible]	360	429	591	868	7.95E-06	8.12E-06	4.43E-06	9.09E-06
Y105E8A.21.[illegible]	350	418	562	845	7.67E-06	7.75E-06	4.41E-06	8.97E-06
Y105E8A.22	111	103	186	324	1.38E-05	1.56E-05	1.48E-05	2.68E-05
Y105E8A.24a	497	480	564	1086	1.39E-05	1.57E-05	1.45E-05	2.70E-05
Y105E8A.24b	470	473	554	1060	1.43E-05	1.25E-05	1.55E-05	3.34E-05
Y105E8A.25	242	238	255	461	1.59E-05	1.45E-05	1.17E-05	2.79E-05
Y105E8A.26a	131	154	402	480	1.39E-05	1.33E-05	1.07E-05	2.52E-05
Y105E8A.26b	127	146	395	467	1.72E-05	1.59E-05	1.18E-05	2.63E-05
Y105E8A.27	32	56	13	13	4.17E-06	4.63E-06	8.33E-06	1.23E-05
Y105E8A.28	1	8	3	3	4.17E-06	4.55E-06	8.45E-06	1.23E-05
Y105E8A.29	285	275	341	571	4.56E-06	7.56E-06	1.82E-06	2.25E-06
Y105E8A.3	862	808	934	1312	2.80E-06	2.65E-06	1.82E-06	2.25E-06
Y105E8A.32	0	0	1	1	1.54E-05	1.40E-05	1.20E-05	2.48E-05
Y105E8A.4	158	270	250	387	3.80E-05	3.36E-05	2.68E-05	4.64E-05
Y105E8A.5	5	8	8	13	2.80E-06	2.65E-06	1.82E-06	2.25E-06
Y105E8A.6	102	97	103	114	2.26E-05	3.65E-05	2.33E-05	4.45E-05
Y105E8A.7a	78	145	75	119	2.80E-06	2.65E-06	1.82E-06	2.25E-06
Y105E8A.7b	78	142	75	120	1.01E-05	9.10E-06	6.65E-06	9.09E-06
Y105E8A.7c	2	3	0	0	3.08E-06	5.42E-06	1.93E-06	3.78E-06
Y105E8A.7d	23	31	23	28	2.97E-06	5.11E-06	1.86E-06	3.67E-06
Y105E8A.8	177	187	269	367	2.80E-06	2.65E-06	1.82E-06	2.25E-06
Y105E8A.9	852	772	992	1523	2.97E-06	3.81E-06	1.93E-06	2.92E-06
Y105E8B.10	25	21	14	7	1.33E-05	1.33E-05	1.32E-05	2.22E-05
Y105E8B.11	5881	5809	5126	6987	3.17E-05	2.71E-05	2.40E-05	4.55E-05
Y105E8B.1b.[illegible]	3980	3676	3668	4846	6.05E-06	4.81E-06	2.20E-06	2.25E-06
Y105E8B.1b.[illegible]	2183	2108	2173	3142	3.28E-03	3.06E-03	1.86E-03	3.13E-03
Y105E8B.1b.[illegible]	3845	3519	3612	4775	4.72E-04	4.12E-04	2.83E-04	4.62E-04
Y105E8B.1b.[illegible]	2157	2068	2144	3134	1.61E-04	1.47E-04	1.04E-04	1.86E-04
Y105E8B.1c	3916	4050	3034	4876	6.37E-04	5.51E-04	3.89E-04	6.35E-04
Y105E8B.1d	3134	2921	2869	4195	2.56E-04	2.32E-04	1.66E-04	2.99E-04

Y105E8B.1e.1	3997	4115	3062	4901	3.32E-04	3.24E-04	1.67E-04	3.32E-04
Y105E8B.1e.2	3846	3960	2966	4705	4.11E-04	3.61E-04	2.45E-04	4.41E-04
Y105E8B.1e.3	1439	1416	1176	1872	3.15E-04	3.07E-04	1.57E-04	3.10E-04
Y105E8B.1f	5131	5008	4031	5835	3.55E-04	3.46E-04	1.78E-04	3.49E-04
Y105E8B.2a	441	511	692	949	2.09E-04	1.94E-04	1.11E-04	2.18E-04
Y105E8B.2b.1	259	279	402	542	4.35E-04	4.01E-04	2.23E-04	3.98E-04
Y105E8B.2b.2	207	215	326	447	2.21E-05	2.42E-05	2.26E-05	3.83E-05
Y105E8B.4	920	769	1269	1705	2.08E-05	2.11E-05	2.10E-05	3.49E-05
Y105E8B.5	359	490	632	379	2.26E-05	2.21E-05	2.31E-05	3.92E-05
Y105E8B.6	41	63	49	39	8.45E-05	6.67E-05	7.58E-05	1.26E-04
Y105E8B.7	26	29	19	27	4.93E-05	6.35E-05	5.64E-05	4.18E-05
Y105E8B.8a	395	470	486	631	1.87E-05	2.71E-05	1.45E-05	1.43E-05
Y105E8B.8b	369	435	451	583	3.64E-06	3.84E-06	1.82E-06	3.04E-06
Y105E8B.9	63	59	44	60	2.73E-05	3.07E-05	2.18E-05	3.50E-05
Y106G6A.1	118	173	114	181	2.68E-05	2.98E-05	2.13E-05	3.40E-05
Y106G6A.2a	319	430	379	515	3.67E-06	3.23E-06	1.82E-06	2.79E-06
Y106G6A.4	81	126	45	41	8.51E-06	1.18E-05	5.34E-06	1.05E-05
Y106G6D.1	7	15	7	5	2.47E-05	3.15E-05	1.91E-05	3.20E-05
Y106G6D.2	26	33	21	7	1.63E-05	2.39E-05	5.89E-06	6.61E-06
Y106G6D.3	67	112	43	36	2.80E-06	2.65E-06	1.82E-06	2.25E-06
Y106G6D.4	15	26	17	8	3.89E-06	4.68E-06	2.04E-06	2.25E-06
Y106G6D.5a	130	144	117	259	4.26E-06	6.72E-06	1.82E-06	2.25E-06
Y106G6D.5b	127	143	114	243	2.80E-06	2.65E-06	1.82E-06	2.25E-06
Y106G6D.6	200	226	185	339	7.50E-06	7.86E-06	4.39E-06	1.20E-05
Y106G6D.7	451	488	568	947	7.56E-06	8.04E-06	4.41E-06	1.16E-05
Y106G6D.8.1	157	219	209	154	8.40E-06	8.97E-06	5.05E-06	1.14E-05
Y106G6D.8.2	130	188	178	140	1.57E-05	1.61E-05	1.29E-05	2.65E-05
Y106G6E.1	10	4	10	5	3.24E-05	4.27E-05	2.81E-05	2.55E-05
Y106G6E.2	18	39	8	14	3.80E-05	5.19E-05	3.39E-05	3.29E-05
Y106G6E.3	20	33	10	16	2.80E-06	2.65E-06	1.82E-06	2.25E-06
Y106G6E.4	37	68	57	65	2.80E-06	3.97E-06	1.82E-06	2.25E-06
Y106G6E.5.1	331	453	383	595	3.33E-06	5.18E-06	1.82E-06	2.25E-06
Y106G6E.6.1	981	924	961	1523	5.35E-06	9.28E-06	5.36E-06	7.53E-06
Y106G6E.6.2	464	461	494	789	1.59E-05	2.06E-05	1.20E-05	2.30E-05
Y106G6G.1	18	28	24	13	4.80E-05	4.27E-05	3.06E-05	5.98E-05
Y106G6G.2	21	30	21	35	4.02E-05	3.77E-05	2.78E-05	5.49E-05
Y106G6G.3	3	9	2	4	2.80E-06	2.65E-06	1.82E-06	2.25E-06
Y106G6G.4	47	96	25	29	2.80E-06	2.65E-06	1.82E-06	2.25E-06
Y106G6G.5	75	88	306	87	2.80E-06	2.65E-06	1.82E-06	2.25E-06
Y106G6G.6	11	17	12	10	5.29E-06	1.02E-05	1.82E-06	2.61E-06
Y106G6H.1	204	212	200	226	2.80E-06	2.65E-06	5.50E-06	2.25E-06
Y106G6H.10	5	9	12	10	2.80E-06	2.65E-06	1.82E-06	2.25E-06
Y106G6H.12	499	607	774	1024	1.57E-05	1.53E-05	9.99E-06	1.39E-05
Y106G6H.13	57	123	96	46	2.80E-06	2.65E-06	1.82E-06	2.25E-06
Y106G6H.14	156	166	314	239	1.72E-05	1.97E-05	1.73E-05	2.83E-05
Y106G6H.15	151	180	146	227	1.53E-05	3.12E-05	1.68E-05	9.92E-06
Y106G6H.16	91	81	119	70	2.34E-05	2.35E-05	3.06E-05	2.87E-05
Y106G6H.2a.	15577	13226	15694	19311	2.31E-05	2.60E-05	1.45E-05	2.79E-05
Y106G6H.2a.	14205	12151	14026	18063	2.20E-05	1.85E-05	1.87E-05	1.36E-05
Y106G6H.2a.	15169	12970	15521	19070	8.11E-04	6.51E-04	5.32E-04	8.08E-04
Y106G6H.2a.	14707	12449	15016	18606	7.59E-04	6.13E-04	4.87E-04	7.75E-04
Y106G6H.2a.	14185	12127	14019	18054	8.08E-04	6.53E-04	5.38E-04	8.16E-04
Y106G6H.2c	13938	11843	14069	17178	7.84E-04	6.27E-04	5.21E-04	7.97E-04
Y106G6H.3	7164	5733	17685	4431	7.56E-04	6.11E-04	4.86E-04	7.73E-04
Y106G6H.4	3	6	6	3	8.15E-04	6.54E-04	5.35E-04	8.07E-04
Y106G6H.5.1	593	750	855	1169	2.13E-03	1.61E-03	3.42E-03	1.06E-03
Y106G6H.5.2	583	738	815	1146	2.80E-06	2.65E-06	1.82E-06	2.25E-06
Y106G6H.6.1	271	345	413	481	2.37E-05	2.83E-05	2.22E-05	3.75E-05
Y106G6H.6.2	257	330	395	472	2.53E-05	3.02E-05	2.30E-05	3.99E-05

Y106G6H.7	445	498	572	893	2.06E-05	2.48E-05	2.04E-05	2.94E-05
Y106G6H.8	234	149	277	150	2.02E-05	2.45E-05	2.02E-05	2.98E-05
Y106G6H.9	5	9	12	10	1.77E-05	1.87E-05	1.48E-05	2.86E-05
Y108F1.1	33	46	35	62	4.92E-05	2.96E-05	3.79E-05	2.53E-05
Y108F1.3	56	45	53	66	2.80E-06	2.65E-06	1.82E-06	2.25E-06
Y108F1.4	28	36	31	47	5.15E-06	6.80E-06	3.55E-06	7.78E-06
Y108F1.5	36	43	77	60	7.95E-06	6.03E-06	4.90E-06	7.53E-06
Y108G3AL.2	179	195	127	189	4.09E-06	4.97E-06	2.95E-06	5.53E-06
Y108G3AL.3	16	26	30	21	4.06E-06	4.58E-06	5.65E-06	5.44E-06
Y108G3AL.7	519	409	849	1359	1.87E-05	1.92E-05	8.62E-06	1.59E-05
Y10G11A.1	480	358	481	640	2.80E-06	2.65E-06	1.82E-06	2.25E-06
Y10G11A.3	8	6	6	7	1.10E-05	8.20E-06	1.17E-05	2.32E-05
Y110A2AL.1	2	0	3	1	9.05E-05	6.38E-05	5.90E-05	9.69E-05
Y110A2AL.10	2	0	1	1	2.80E-06	2.65E-06	1.82E-06	2.25E-06
Y110A2AL.12	47	57	85	121	2.80E-06	2.65E-06	1.82E-06	2.25E-06
Y110A2AL.12	19	27	53	71	2.80E-06	2.65E-06	1.82E-06	2.25E-06
Y110A2AL.13	29	30	252	103	3.64E-06	4.18E-06	4.30E-06	7.53E-06
Y110A2AL.2	2	1	5	0	2.80E-06	2.75E-06	3.70E-06	6.14E-06
Y110A2AL.3	0	7	13	2	6.41E-06	6.27E-06	3.62E-05	1.83E-05
Y110A2AL.4a	12	19	24	16	2.80E-06	2.65E-06	1.82E-06	2.25E-06
Y110A2AL.4b	6	7	16	7	2.80E-06	2.65E-06	2.33E-06	2.25E-06
Y110A2AL.5	1	5	0	2	2.80E-06	2.65E-06	1.91E-06	2.25E-06
Y110A2AL.6	1	0	0	0	2.80E-06	2.65E-06	2.17E-06	2.25E-06
Y110A2AL.7	1	1	2	1	2.80E-06	2.65E-06	1.82E-06	2.25E-06
Y110A2AL.8a	545	793	271	402	2.80E-06	2.65E-06	1.82E-06	2.25E-06
Y110A2AL.8b	512	755	255	384	2.80E-06	2.65E-06	1.82E-06	2.25E-06
Y110A2AL.9	14	20	26	3	1.39E-05	1.92E-05	4.50E-06	8.25E-06
Y110A2AM.1	45	43	103	131	1.37E-05	1.92E-05	4.46E-06	8.30E-06
Y110A2AM.3	74	73	149	175	4.98E-06	6.72E-06	6.01E-06	2.25E-06
Y110A2AM.4	58	37	221	75	4.84E-06	4.36E-06	7.22E-06	1.13E-05
Y110A2AR.1	322	347	710	695	1.31E-05	1.22E-05	1.72E-05	2.49E-05
Y110A2AR.2	85	83	202	251	1.79E-05	1.08E-05	4.45E-05	1.86E-05
Y110A2AR.3b	30	44	38	46	3.95E-05	4.02E-05	5.67E-05	6.85E-05
Y110A7A.10	518	475	694	850	1.39E-05	1.28E-05	2.15E-05	3.30E-05
Y110A7A.11	125	134	211	172	9.27E-06	1.29E-05	7.65E-06	1.14E-05
Y110A7A.12	74	96	106	75	3.25E-05	2.81E-05	2.83E-05	4.28E-05
Y110A7A.13	378	403	462	456	1.69E-05	1.71E-05	1.86E-05	1.87E-05
Y110A7A.14.	1669	1410	1505	1491	5.52E-06	6.74E-06	5.14E-06	4.48E-06
Y110A7A.14.	1296	1131	1023	1192	3.70E-05	3.73E-05	2.95E-05	3.59E-05
Y110A7A.15a	581	549	685	899	1.78E-04	1.42E-04	1.05E-04	1.28E-04
Y110A7A.15b	159	152	194	253	1.92E-04	1.58E-04	9.86E-05	1.42E-04
Y110A7A.16	491	561	741	1126	3.39E-05	3.03E-05	2.60E-05	4.21E-05
Y110A7A.17a	785	823	1271	1798	3.02E-05	2.73E-05	2.40E-05	3.86E-05
Y110A7A.17b	356	318	580	797	1.44E-05	1.55E-05	1.41E-05	2.64E-05
Y110A7A.17b	296	255	470	685	3.31E-05	3.28E-05	3.49E-05	6.09E-05
Y110A7A.18	1366	1329	1437	1934	3.86E-05	3.26E-05	4.10E-05	6.95E-05
Y110A7A.19.	661	612	959	1222	3.98E-05	3.23E-05	4.11E-05	7.39E-05
Y110A7A.19.	630	579	898	1173	4.76E-05	4.37E-05	3.26E-05	5.41E-05
Y110A7A.1a	746	718	944	1520	3.53E-05	3.08E-05	3.33E-05	5.24E-05
Y110A7A.1b	744	714	948	1516	3.53E-05	3.07E-05	3.28E-05	5.28E-05
Y110A7A.2	5	7	3	3	1.58E-05	1.44E-05	1.31E-05	2.60E-05
Y110A7A.20	1	3	3	2	1.58E-05	1.43E-05	1.31E-05	2.58E-05
Y110A7A.3	41	40	54	59	2.80E-06	2.65E-06	1.82E-06	2.25E-06
Y110A7A.4	240	260	335	320	2.80E-06	2.65E-06	1.82E-06	2.25E-06
Y110A7A.5	168	231	224	361	2.83E-06	2.65E-06	2.42E-06	3.26E-06
Y110A7A.6a.	639	839	558	892	2.65E-05	2.71E-05	2.41E-05	2.84E-05
Y110A7A.6a.	583	762	497	812	9.97E-06	1.30E-05	8.66E-06	1.72E-05
Y110A7A.6a.	595	775	513	821	4.62E-05	5.73E-05	2.63E-05	5.18E-05
Y110A7A.6b.	618	792	561	863	4.75E-05	5.87E-05	2.64E-05	5.32E-05

Y110A7A.6b.	573	732	492	796	4.89E-05	6.02E-05	2.75E-05	5.42E-05
Y110A7A.7	49	66	63	58	4.77E-05	5.78E-05	2.82E-05	5.35E-05
Y110A7A.9a	528	695	845	1155	4.90E-05	5.91E-05	2.74E-05	5.47E-05
Y110A7A.9b.	442	605	714	997	5.74E-06	7.30E-06	4.79E-06	5.44E-06
Y110A7A.9b.	528	695	845	1155	1.83E-05	2.27E-05	1.91E-05	3.22E-05
Y111B2A.1	135	96	149	185	1.79E-05	2.32E-05	1.88E-05	3.25E-05
Y111B2A.10a	229	221	311	411	1.91E-05	2.37E-05	1.99E-05	3.35E-05
Y111B2A.10b	278	258	373	480	1.30E-05	8.78E-06	9.38E-06	1.44E-05
Y111B2A.10b	267	257	356	463	1.92E-05	1.75E-05	1.70E-05	2.77E-05
Y111B2A.10b	221	211	300	396	1.81E-05	1.58E-05	1.58E-05	2.51E-05
Y111B2A.11	855	866	977	1492	2.06E-05	1.88E-05	1.79E-05	2.87E-05
Y111B2A.12	108	97	187	203	2.08E-05	1.88E-05	1.84E-05	2.99E-05
Y111B2A.13	47	89	58	47	2.77E-05	2.65E-05	2.06E-05	3.88E-05
Y111B2A.14.	205	240	273	481	1.88E-05	1.60E-05	2.12E-05	2.85E-05
Y111B2A.14.:	181	228	254	467	8.09E-06	1.45E-05	6.49E-06	6.50E-06
Y111B2A.15	268	346	235	394	4.87E-06	5.40E-06	4.23E-06	9.18E-06
Y111B2A.16	38	69	199	122	4.42E-06	5.24E-06	4.03E-06	9.15E-06
Y111B2A.17	319	288	400	530	2.11E-05	2.57E-05	1.20E-05	2.49E-05
Y111B2A.18.	1927	1332	1426	2195	4.93E-06	8.46E-06	1.68E-05	1.27E-05
Y111B2A.18.:	1133	789	864	1260	1.52E-05	1.30E-05	1.24E-05	2.03E-05
Y111B2A.18.:	991	693	732	1117	1.39E-04	9.05E-05	6.67E-05	1.27E-04
Y111B2A.19	70	81	137	262	1.25E-04	8.22E-05	6.20E-05	1.12E-04
Y111B2A.2	457	313	901	296	1.41E-04	9.32E-05	6.78E-05	1.28E-04
Y111B2A.20	292	303	337	398	4.62E-06	5.05E-06	5.89E-06	1.39E-05
Y111B2A.21	19	16	8	15	1.01E-04	6.51E-05	1.29E-04	5.23E-05
Y111B2A.22	1085	945	1076	1929	2.66E-05	2.61E-05	2.00E-05	2.91E-05
Y111B2A.24	64	71	240	289	2.94E-06	2.65E-06	1.82E-06	2.25E-06
Y111B2A.25	48	57	39	61	1.69E-05	1.39E-05	1.09E-05	2.41E-05
Y111B2A.26	69	100	34	84	5.21E-06	5.48E-06	1.27E-05	1.89E-05
Y111B2A.27	8	11	6	1	2.80E-06	2.65E-06	1.82E-06	2.25E-06
Y111B2A.28	15	32	55	82	6.92E-06	9.47E-06	2.22E-06	6.77E-06
Y111B2A.3.1	478	494	741	913	2.80E-06	2.65E-06	1.82E-06	2.25E-06
Y111B2A.3.2	864	732	1572	1123	4.17E-06	8.41E-06	9.97E-06	1.84E-05
Y111B2A.4	76	92	29	70	2.15E-05	2.10E-05	2.17E-05	3.31E-05
Y111B2A.5a	74	93	49	86	3.77E-05	3.01E-05	4.46E-05	3.93E-05
Y111B2A.8	165	155	236	335	6.16E-06	7.04E-06	1.82E-06	4.54E-06
Y111B2A.9a	70	52	82	131	6.36E-06	7.54E-06	2.73E-06	5.94E-06
Y111B2A.9b	41	26	55	77	9.69E-06	8.60E-06	9.02E-06	1.58E-05
Y113G7A.1	3	3	1	1	6.41E-06	4.50E-06	4.90E-06	9.65E-06
Y113G7A.10	8	11	9	5	3.70E-06	2.65E-06	3.23E-06	5.58E-06
Y113G7A.11	6	3	8	8	2.80E-06	2.65E-06	1.82E-06	2.25E-06
Y113G7A.12	2	4	1	2	2.80E-06	2.65E-06	1.82E-06	2.25E-06
Y113G7A.13	0	4	2	2	2.80E-06	2.65E-06	1.82E-06	2.25E-06
Y113G7A.14	8	9	5	2	2.80E-06	2.65E-06	1.82E-06	2.25E-06
Y113G7A.15	4	10	11	2	2.80E-06	2.65E-06	1.82E-06	2.25E-06
Y113G7A.16	9	15	29	18	2.80E-06	2.65E-06	1.82E-06	2.25E-06
Y113G7A.2	6	7	25	10	2.80E-06	2.65E-06	1.82E-06	2.25E-06
Y113G7A.3.1	1342	1065	1568	2057	2.80E-06	2.65E-06	3.19E-06	2.45E-06
Y113G7A.3.2	1189	968	1343	1848	2.80E-06	2.65E-06	1.82E-06	2.25E-06
Y113G7A.4a	370	437	463	526	5.84E-05	4.37E-05	4.44E-05	7.18E-05
Y113G7A.4b	239	337	308	334	5.39E-05	4.14E-05	3.96E-05	6.73E-05
Y113G7A.5	31	47	44	34	1.47E-05	1.64E-05	1.20E-05	1.68E-05
Y113G7A.6a	29	31	15	28	9.60E-06	1.28E-05	8.05E-06	1.08E-05
Y113G7A.6b	26	26	15	27	2.80E-06	2.94E-06	1.90E-06	2.25E-06
Y113G7A.7	4	5	3	4	2.80E-06	2.65E-06	1.82E-06	2.25E-06
Y113G7A.8.1	114	174	230	341	2.80E-06	2.65E-06	1.82E-06	2.25E-06
Y113G7A.8.2	120	177	233	349	2.80E-06	2.65E-06	1.82E-06	2.25E-06
Y113G7A.9	205	201	326	414	6.97E-06	1.01E-05	9.15E-06	1.68E-05
Y113G7A.t1	0	0	0	1	7.59E-06	1.06E-05	9.60E-06	1.78E-05

Y113G7A.t3	1	1	0	0	2.32E-05	2.15E-05	2.40E-05	3.76E-05
Y113G7B.11	5	12	3	4	2.80E-06	2.65E-06	1.82E-06	2.25E-06
Y113G7B.12	86	72	91	83	2.80E-06	2.65E-06	1.82E-06	2.25E-06
Y113G7B.14	22	33	14	29	2.80E-06	2.65E-06	1.82E-06	2.25E-06
Y113G7B.15	2	2	3	6	3.08E-06	2.65E-06	2.11E-06	2.38E-06
Y113G7B.16	132	164	216	288	2.80E-06	2.65E-06	1.82E-06	2.25E-06
Y113G7B.17.	3601	2709	3443	4380	2.80E-06	2.65E-06	1.82E-06	2.25E-06
Y113G7B.17.	3040	2288	2741	3656	9.10E-06	1.07E-05	9.69E-06	1.59E-05
Y113G7B.1a	4	6	6	1	3.12E-04	2.22E-04	1.94E-04	3.05E-04
Y113G7B.1b	7	6	7	1	3.23E-04	2.30E-04	1.90E-04	3.12E-04
Y113G7B.2	5	10	2	0	2.80E-06	2.65E-06	1.82E-06	2.25E-06
Y113G7B.23	651	495	879	1279	2.80E-06	2.65E-06	1.82E-06	2.25E-06
Y113G7B.24	92	110	148	194	2.80E-06	2.65E-06	1.82E-06	2.25E-06
Y113G7B.26	1	0	0	1	2.42E-05	1.74E-05	2.13E-05	3.82E-05
Y113G7B.3	20	29	19	20	1.21E-05	1.36E-05	1.27E-05	2.05E-05
Y113G7B.4	88	88	114	119	2.80E-06	2.65E-06	1.82E-06	2.25E-06
Y113G7B.5a	150	165	250	293	2.80E-06	3.20E-06	1.82E-06	2.25E-06
Y113G7B.5b	139	138	202	262	1.01E-05	9.50E-06	8.47E-06	1.09E-05
Y113G7B.6	6	4	6	2	1.61E-05	1.68E-05	1.75E-05	2.53E-05
Y113G7B.7	76	99	91	80	1.58E-05	1.48E-05	1.50E-05	2.40E-05
Y113G7B.8	0	7	9	6	2.80E-06	2.65E-06	1.82E-06	2.25E-06
Y113G7B.9	1	4	6	5	9.02E-06	1.11E-05	7.02E-06	7.62E-06
Y113G7C.1	160	236	79	54	2.80E-06	2.65E-06	1.82E-06	2.25E-06
Y116A8A.1	2	2	1	1	2.80E-06	2.65E-06	1.82E-06	2.25E-06
Y116A8A.10	100	90	183	61	3.95E-06	5.50E-06	1.82E-06	2.25E-06
Y116A8A.2	6	7	12	2	2.80E-06	2.65E-06	1.82E-06	2.25E-06
Y116A8A.3	2	1	2	1	1.74E-05	1.48E-05	2.07E-05	8.52E-06
Y116A8A.4	18	14	9	7	2.80E-06	2.65E-06	1.82E-06	2.25E-06
Y116A8A.6	11	24	12	12	2.80E-06	2.65E-06	1.82E-06	2.25E-06
Y116A8A.7	7	9	2	3	2.80E-06	2.65E-06	1.82E-06	2.25E-06
Y116A8A.8	5	10	7	4	2.80E-06	2.65E-06	1.82E-06	2.25E-06
Y116A8A.9.1	1065	773	1065	1376	2.80E-06	2.65E-06	1.82E-06	2.25E-06
Y116A8A.9.2	1060	763	1057	1369	2.80E-06	2.65E-06	1.82E-06	2.25E-06
Y116A8B.1	7	5	11	8	7.50E-05	5.14E-05	4.88E-05	7.79E-05
Y116A8B.4	7	19	15	6	8.50E-05	5.78E-05	5.52E-05	8.82E-05
Y116A8B.5	9	6	6	3	2.80E-06	2.65E-06	1.82E-06	2.25E-06
Y116A8C.1	5	6	6	1	2.80E-06	7.19E-06	3.92E-06	2.25E-06
Y116A8C.10b	29	55	82	81	2.80E-06	2.65E-06	1.82E-06	2.25E-06
Y116A8C.10b	80	88	128	140	2.80E-06	2.65E-06	1.82E-06	2.25E-06
Y116A8C.11	4	10	23	10	3.78E-06	6.77E-06	6.96E-06	8.48E-06
Y116A8C.13	83	115	163	302	7.53E-06	7.83E-06	7.84E-06	1.06E-05
Y116A8C.14	8	8	15	7	2.80E-06	2.65E-06	1.99E-06	2.25E-06
Y116A8C.15	68	53	74	89	3.72E-06	4.87E-06	4.76E-06	1.09E-05
Y116A8C.16a	175	144	217	302	2.80E-06	2.65E-06	1.82E-06	2.25E-06
Y116A8C.16b	24	27	39	48	1.36E-05	1.00E-05	9.64E-06	1.43E-05
Y116A8C.17	11	19	8	8	7.90E-06	6.14E-06	6.38E-06	1.10E-05
Y116A8C.18	15	3	14	12	3.67E-06	3.89E-06	3.88E-06	5.89E-06
Y116A8C.19	11	13	11	9	2.80E-06	2.65E-06	1.82E-06	2.25E-06
Y116A8C.20	5	6	8	3	2.80E-06	2.65E-06	1.82E-06	2.25E-06
Y116A8C.21	4	6	11	8	2.80E-06	2.65E-06	1.82E-06	2.25E-06
Y116A8C.22	13	14	18	14	2.80E-06	2.65E-06	1.82E-06	2.25E-06
Y116A8C.23	13	20	10	7	2.80E-06	2.65E-06	1.82E-06	2.25E-06
Y116A8C.24	33	52	19	19	2.80E-06	2.65E-06	1.82E-06	2.25E-06
Y116A8C.25	13	20	17	12	2.80E-06	2.65E-06	1.82E-06	2.25E-06
Y116A8C.28a	91	120	92	176	2.80E-06	3.52E-06	1.82E-06	2.25E-06
Y116A8C.28a	120	149	120	211	2.80E-06	2.65E-06	1.82E-06	2.25E-06
Y116A8C.28b	73	83	78	113	1.33E-05	1.65E-05	8.73E-06	2.06E-05
Y116A8C.28c	120	149	120	211	1.71E-05	2.01E-05	1.11E-05	2.42E-05
Y116A8C.29	13	27	19	7	1.47E-05	1.58E-05	1.02E-05	1.83E-05

Y116A8C.3	18	32	30	28	1.71E-05	2.01E-05	1.11E-05	2.42E-05
Y116A8C.30a	75	74	193	93	5.57E-06	1.10E-05	5.30E-06	2.41E-06
Y116A8C.32	221	246	576	782	2.80E-06	4.13E-06	2.68E-06	3.08E-06
Y116A8C.33	30	35	30	33	1.59E-05	1.48E-05	2.66E-05	1.59E-05
Y116A8C.34.:	214	237	346	378	1.01E-05	1.07E-05	1.72E-05	2.88E-05
Y116A8C.35	254	354	396	241	2.80E-06	2.65E-06	1.82E-06	2.25E-06
Y116A8C.36	682	546	1049	1408	2.30E-05	2.40E-05	2.42E-05	3.26E-05
Y116A8C.37	15	19	10	3	2.71E-05	3.57E-05	2.75E-05	2.07E-05
Y116A8C.38	31	49	17	21	2.12E-05	1.61E-05	2.13E-05	3.52E-05
Y116A8C.4.1	23	49	12	11	2.80E-06	2.65E-06	1.82E-06	2.25E-06
Y116A8C.4.2	23	46	11	10	2.80E-06	3.20E-06	1.82E-06	2.25E-06
Y116A8C.40	4	5	3	1	2.80E-06	2.65E-06	1.82E-06	2.25E-06
Y116A8C.43	7	9	12	3	2.80E-06	2.65E-06	1.82E-06	2.25E-06
Y116A8C.44	5	1	4	4	2.80E-06	2.65E-06	1.82E-06	2.25E-06
Y116A8C.5	10	11	12	6	2.80E-06	2.65E-06	1.82E-06	2.25E-06
Y116A8C.7	0	5	2	1	2.80E-06	2.65E-06	1.82E-06	2.25E-06
Y116A8C.8	2	0	0	0	2.80E-06	2.65E-06	1.82E-06	2.25E-06
Y116A8C.9	58	53	112	171	2.80E-06	2.65E-06	1.82E-06	2.25E-06
Y116F11A.1	5	6	3	3	2.80E-06	2.65E-06	1.82E-06	2.25E-06
Y116F11A.3	2	1	0	1	5.12E-06	4.42E-06	6.43E-06	1.21E-05
Y116F11A.4	0	3	5	1	2.80E-06	2.65E-06	1.82E-06	2.25E-06
Y116F11A.6	14	12	21	6	2.80E-06	2.65E-06	1.82E-06	2.25E-06
Y116F11B.1	8	11	9	6	2.80E-06	2.65E-06	1.82E-06	2.25E-06
Y116F11B.10	41	71	58	115	4.06E-06	3.28E-06	3.95E-06	2.25E-06
Y116F11B.11	5	12	12	4	3.05E-06	3.97E-06	2.22E-06	2.25E-06
Y116F11B.12	244	278	360	449	2.80E-06	2.65E-06	1.82E-06	3.01E-06
Y116F11B.12	227	248	326	398	2.80E-06	2.65E-06	1.82E-06	2.25E-06
Y116F11B.12	195	225	283	367	1.25E-05	1.34E-05	1.20E-05	1.84E-05
Y116F11B.13	8	7	4	8	1.31E-05	1.35E-05	1.22E-05	1.85E-05
Y116F11B.14	11	11	10	5	1.21E-05	1.32E-05	1.14E-05	1.83E-05
Y116F11B.2	3	1	9	5	2.80E-06	2.65E-06	1.82E-06	2.25E-06
Y116F11B.3.:	193	144	217	139	2.80E-06	2.65E-06	1.82E-06	2.25E-06
Y116F11B.3.:	186	138	212	137	2.80E-06	2.65E-06	1.82E-06	2.25E-06
Y116F11B.4	7	1	10	4	6.86E-06	4.84E-06	5.01E-06	3.96E-06
Y116F11B.5	3	5	12	3	6.61E-06	4.63E-06	4.90E-06	3.91E-06
Y116F11B.6	11	12	7	11	2.80E-06	2.65E-06	1.82E-06	2.25E-06
Y116F11B.7	6	22	10	4	2.80E-06	2.65E-06	1.82E-06	2.25E-06
Y116F11B.8	10	18	24	18	2.80E-06	2.65E-06	1.82E-06	2.25E-06
Y116F11B.9a	12	9	15	5	2.80E-06	2.65E-06	1.82E-06	2.25E-06
Y119C1A.1	111	175	266	359	2.80E-06	2.65E-06	1.82E-06	2.25E-06
Y119C1B.1	13	21	25	9	2.80E-06	2.65E-06	1.82E-06	2.25E-06
Y119C1B.10	26	54	20	27	1.61E-05	2.40E-05	2.51E-05	4.19E-05
Y119C1B.11	0	1	2	1	2.80E-06	2.70E-06	2.20E-06	2.25E-06
Y119C1B.12	2	3	1	2	3.14E-06	6.16E-06	1.82E-06	2.63E-06
Y119C1B.3	18	25	30	8	2.80E-06	2.65E-06	1.82E-06	2.25E-06
Y119C1B.4	341	399	569	409	2.80E-06	2.65E-06	1.82E-06	2.25E-06
Y119C1B.5	115	336	138	278	2.80E-06	2.65E-06	1.82E-06	2.25E-06
Y119C1B.6	9	13	11	2	4.33E-05	4.78E-05	4.70E-05	4.17E-05
Y119C1B.8a	584	481	772	1074	5.29E-06	1.46E-05	4.14E-06	1.03E-05
Y119C1B.8b	554	455	716	1015	4.14E-06	5.66E-06	3.30E-06	2.25E-06
Y119C1B.9	71	104	58	61	2.56E-05	1.99E-05	2.20E-05	3.78E-05
Y119D3A.1	3	3	5	3	2.70E-05	2.09E-05	2.27E-05	3.97E-05
Y119D3A.2	3	4	9	4	1.00E-05	1.38E-05	5.32E-06	6.90E-06
Y119D3A.3	6	14	5	9	2.80E-06	2.65E-06	1.82E-06	2.25E-06
Y119D3A.4	6	8	3	9	2.80E-06	2.65E-06	1.82E-06	2.25E-06
Y119D3B.10	4	5	14	12	2.80E-06	2.65E-06	1.82E-06	2.25E-06
Y119D3B.11.	173	185	260	468	2.80E-06	2.65E-06	1.82E-06	2.25E-06
Y119D3B.11.	164	177	249	459	2.80E-06	2.65E-06	1.82E-06	2.25E-06
Y119D3B.12a	187	154	193	286	1.02E-05	1.03E-05	9.99E-06	2.22E-05

Y119D3B.12a	174	147	177	275	9.94E-06	1.02E-05	9.84E-06	2.24E-05
Y119D3B.12a	185	152	195	289	1.74E-05	1.36E-05	1.17E-05	2.14E-05
Y119D3B.13	64	64	78	80	1.64E-05	1.31E-05	1.09E-05	2.09E-05
Y119D3B.14	110	74	168	253	1.42E-05	1.10E-05	9.73E-06	1.78E-05
Y119D3B.15.	225	313	442	261	9.52E-06	8.99E-06	7.54E-06	9.56E-06
Y119D3B.15.	194	278	321	217	5.74E-06	3.65E-06	5.69E-06	1.06E-05
Y119D3B.16	53	60	228	187	4.82E-05	6.33E-05	6.16E-05	4.49E-05
Y119D3B.17	109	138	136	151	6.22E-05	8.43E-05	6.70E-05	5.59E-05
Y119D3B.18	14	19	11	16	5.49E-06	5.87E-06	1.53E-05	1.55E-05
Y119D3B.19	16	17	16	12	6.27E-06	7.49E-06	5.08E-06	6.97E-06
Y119D3B.20	14	17	7	14	2.80E-06	2.67E-06	1.82E-06	2.25E-06
Y119D3B.21	2517	1192	6706	1038	2.80E-06	2.65E-06	1.82E-06	2.25E-06
Y119D3B.22	8	24	15	17	2.80E-06	2.75E-06	1.82E-06	2.25E-06
Y119D3B.3	5	7	4	1	8.37E-04	3.74E-04	1.45E-03	2.77E-04
Y119D3B.4	7	0	2	8	2.80E-06	3.15E-06	1.82E-06	2.25E-06
Y119D3B.5	5	7	4	1	2.80E-06	2.65E-06	1.82E-06	2.25E-06
Y119D3B.6	15	11	19	23	2.88E-06	2.65E-06	1.82E-06	2.63E-06
Y119D3B.7	11	4	16	9	2.80E-06	2.65E-06	1.82E-06	2.25E-06
Y119D3B.8	15	25	14	13	2.80E-06	2.65E-06	1.82E-06	2.61E-06
Y119D3B.9	2	17	3	7	2.80E-06	2.65E-06	1.82E-06	2.25E-06
Y119D3B.t1	0	0	1	0	2.80E-06	2.88E-06	1.82E-06	2.25E-06
Y11D7A.1	29	21	29	35	2.80E-06	2.65E-06	1.82E-06	2.25E-06
Y11D7A.11	275	226	126	95	2.80E-06	2.65E-06	1.82E-06	2.25E-06
Y11D7A.13	150	195	201	260	6.08E-06	4.15E-06	3.95E-06	5.89E-06
Y11D7A.14	112	155	79	81	3.11E-05	2.41E-05	9.27E-06	8.64E-06
Y11D7A.16	3	3	3	1	1.33E-05	1.63E-05	1.16E-05	1.85E-05
Y11D7A.17	22	16	53	25	2.80E-06	3.65E-06	1.82E-06	2.25E-06
Y11D7A.3a	215	205	278	249	2.80E-06	2.65E-06	1.82E-06	2.25E-06
Y11D7A.3b	215	209	266	267	2.80E-06	2.65E-06	3.50E-06	2.25E-06
Y11D7A.4	5	6	3	6	1.64E-05	1.48E-05	1.38E-05	1.53E-05
Y11D7A.5	64	129	37	11	1.53E-05	1.40E-05	1.23E-05	1.53E-05
Y11D7A.7	108	124	112	132	2.80E-06	2.65E-06	1.82E-06	2.25E-06
Y11D7A.8	9	14	6	4	1.48E-05	2.81E-05	5.56E-06	2.25E-06
Y11D7A.9	74	181	21	28	2.02E-05	2.19E-05	1.36E-05	1.98E-05
Y12A6A.1	100	124	91	73	2.80E-06	2.65E-06	1.82E-06	2.25E-06
Y12A6A.2	17	26	24	19	9.27E-06	2.14E-05	1.82E-06	2.81E-06
Y13C8A.1	13	19	5	9	1.29E-05	1.51E-05	7.60E-06	7.53E-06
Y13C8A.2	10	9	7	12	2.80E-06	3.99E-06	2.55E-06	2.47E-06
Y14H12A.1	58	49	83	38	4.48E-06	6.22E-06	1.82E-06	2.50E-06
Y14H12A.2	1	8	5	2	3.19E-06	2.72E-06	1.82E-06	3.08E-06
Y14H12B.1a	310	288	435	508	1.43E-05	1.14E-05	1.33E-05	7.53E-06
Y14H12B.1b	279	241	350	479	2.80E-06	3.15E-06	1.82E-06	2.25E-06
Y14H12B.1c	310	288	435	508	4.41E-05	3.87E-05	4.03E-05	5.81E-05
Y14H12B.2	336	370	583	704	4.04E-05	3.30E-05	3.30E-05	5.58E-05
Y15E3A.1a.1	56	71	40	40	4.41E-05	3.87E-05	4.03E-05	5.81E-05
Y15E3A.1a.2	57	71	41	40	2.69E-05	2.80E-05	3.04E-05	4.53E-05
Y15E3A.1a.3	57	71	41	40	3.36E-06	4.02E-06	1.82E-06	2.25E-06
Y15E3A.1b.1	56	71	40	40	3.64E-06	4.29E-06	1.82E-06	2.25E-06
Y15E3A.1b.2	57	71	41	40	3.44E-06	4.05E-06	1.82E-06	2.25E-06
Y15E3A.1b.3	57	71	41	40	3.58E-06	4.29E-06	1.82E-06	2.25E-06
Y15E3A.2	2	7	3	5	3.44E-06	4.05E-06	1.82E-06	2.25E-06
Y15E3A.3	25	19	24	12	4.56E-06	5.40E-06	2.15E-06	2.59E-06
Y15E3A.4	136	271	190	264	2.80E-06	2.65E-06	1.82E-06	2.25E-06
Y15E3A.5	49	109	112	52	2.80E-06	2.65E-06	1.82E-06	2.25E-06
Y16B4A.2	2222	1477	2606	2749	8.96E-06	1.69E-05	8.16E-06	1.40E-05
Y16E11A.1	18	30	27	16	6.52E-06	1.37E-05	9.68E-06	5.56E-06
Y16E11A.2	15	31	24	14	3.88E-05	2.44E-05	2.96E-05	3.85E-05
Y17D7A.1	6	7	9	13	2.80E-06	2.65E-06	1.82E-06	2.25E-06
Y17D7A.3a	11	34	15	23	2.80E-06	2.65E-06	1.82E-06	2.25E-06

Y17D7A.3b	12	40	18	24	2.80E-06	2.65E-06	1.82E-06	2.25E-06
Y17D7A.4	4	13	10	7	2.80E-06	2.65E-06	1.82E-06	2.25E-06
Y17D7B.1	6	16	11	5	2.80E-06	2.94E-06	1.82E-06	2.25E-06
Y17D7B.2	7	6	12	10	2.80E-06	2.65E-06	1.82E-06	2.25E-06
Y17D7B.3	5	2	5	5	2.80E-06	2.65E-06	1.82E-06	2.25E-06
Y17D7B.5	2	4	3	1	2.80E-06	2.65E-06	1.82E-06	2.25E-06
Y17D7B.6	2	4	0	1	2.80E-06	2.65E-06	1.82E-06	2.25E-06
Y17D7B.7	119	258	247	60	2.80E-06	2.65E-06	1.82E-06	2.25E-06
Y17D7B.8	1	7	3	0	2.80E-06	2.65E-06	1.82E-06	2.25E-06
Y17D7C.1	5	7	16	8	3.70E-05	7.58E-05	5.00E-05	1.50E-05
Y17D7C.2	1	6	3	2	2.80E-06	2.65E-06	1.82E-06	2.25E-06
Y17G7A.1.1	1212	1105	2525	2361	2.80E-06	2.65E-06	1.82E-06	2.25E-06
Y17G7A.1.2	1086	1000	2275	2253	2.80E-06	2.65E-06	1.82E-06	2.25E-06
Y17G7B.1	22	30	47	27	1.27E-04	1.09E-04	1.72E-04	1.98E-04
Y17G7B.10a.	157	269	218	290	1.13E-04	9.79E-05	1.53E-04	1.88E-04
Y17G7B.10a.	166	277	228	300	7.08E-06	9.13E-06	9.84E-06	6.97E-06
Y17G7B.10a.	158	289	218	304	1.17E-05	1.89E-05	1.06E-05	1.73E-05
Y17G7B.10a.	162	292	224	306	1.27E-05	2.00E-05	1.14E-05	1.85E-05
Y17G7B.10a.	159	288	218	304	1.09E-05	1.88E-05	9.75E-06	1.68E-05
Y17G7B.10a.	158	263	215	287	1.21E-05	2.06E-05	1.09E-05	1.83E-05
Y17G7B.10b	175	307	243	319	1.09E-05	1.87E-05	9.73E-06	1.68E-05
Y17G7B.11	50	50	37	40	1.08E-05	1.71E-05	9.60E-06	1.58E-05
Y17G7B.12	91	89	78	77	1.14E-05	1.89E-05	1.03E-05	1.67E-05
Y17G7B.13	79	100	266	361	4.45E-06	4.21E-06	2.13E-06	2.86E-06
Y17G7B.14	2	9	9	4	1.20E-05	1.11E-05	6.71E-06	8.16E-06
Y17G7B.15a	396	404	478	634	7.17E-06	8.57E-06	1.57E-05	2.63E-05
Y17G7B.15b	289	306	341	488	2.80E-06	2.65E-06	1.82E-06	2.25E-06
Y17G7B.17	247	253	230	323	1.79E-05	1.72E-05	1.40E-05	2.30E-05
Y17G7B.18a	118	105	190	294	1.32E-05	1.31E-05	1.01E-05	1.78E-05
Y17G7B.18b	121	101	172	293	2.29E-05	2.21E-05	1.38E-05	2.40E-05
Y17G7B.19	4	2	1	5	1.15E-05	9.68E-06	1.21E-05	2.31E-05
Y17G7B.20	1304	1053	1424	1281	1.17E-05	9.20E-06	1.08E-05	2.27E-05
Y17G7B.21	164	166	261	274	2.80E-06	2.65E-06	1.82E-06	2.25E-06
Y17G7B.22	8	7	0	4	8.70E-05	6.64E-05	6.19E-05	6.87E-05
Y17G7B.23	1	2	0	1	3.03E-05	2.90E-05	3.14E-05	4.07E-05
Y17G7B.24	3	2	3	3	2.80E-06	2.65E-06	1.82E-06	2.25E-06
Y17G7B.2a	403	416	558	720	2.80E-06	2.65E-06	1.82E-06	2.25E-06
Y17G7B.2b	514	532	718	979	2.80E-06	2.65E-06	1.82E-06	2.25E-06
Y17G7B.2c	258	255	373	467	2.63E-05	2.57E-05	2.37E-05	3.78E-05
Y17G7B.3	151	171	250	251	2.64E-05	2.58E-05	2.40E-05	4.04E-05
Y17G7B.4	126	123	161	242	2.08E-05	1.94E-05	1.96E-05	3.02E-05
Y17G7B.5a	1860	1487	2623	3234	9.49E-06	1.02E-05	1.02E-05	1.27E-05
Y17G7B.6	1	9	8	4	1.15E-05	1.07E-05	9.60E-06	1.78E-05
Y17G7B.7.1	900	1138	1170	1465	7.42E-05	5.60E-05	6.81E-05	1.04E-04
Y17G7B.7.2	587	725	734	893	2.80E-06	2.65E-06	1.82E-06	2.25E-06
Y17G7B.8	20	45	24	37	8.62E-05	1.03E-04	7.29E-05	1.13E-04
Y17G7B.9	121	96	181	241	8.75E-05	1.02E-04	7.12E-05	1.07E-04
Y17G9A.1	3	1	7	0	2.80E-06	4.60E-06	1.82E-06	3.22E-06
Y17G9A.2	9	21	10	15	8.48E-06	6.35E-06	8.25E-06	1.36E-05
Y17G9A.3	2	8	12	4	2.80E-06	2.65E-06	1.82E-06	2.25E-06
Y17G9A.4	8	15	17	10	2.80E-06	3.07E-06	1.82E-06	2.25E-06
Y17G9A.5	7	6	7	5	2.80E-06	2.65E-06	1.82E-06	2.25E-06
Y17G9A.6	10	4	5	3	2.80E-06	2.65E-06	1.82E-06	2.25E-06
Y17G9A.7a	8	3	4	5	2.80E-06	2.65E-06	1.82E-06	2.25E-06
Y17G9A.7b	2	2	2	3	2.80E-06	2.65E-06	1.82E-06	2.25E-06
Y17G9B.1	3	13	16	9	2.80E-06	2.65E-06	1.82E-06	2.25E-06
Y17G9B.2	7	1	3	2	2.80E-06	2.65E-06	1.82E-06	2.25E-06
Y17G9B.3	318	393	544	624	2.80E-06	2.65E-06	1.82E-06	2.25E-06
Y17G9B.4	37	35	18	29	3.16E-06	2.65E-06	1.82E-06	2.25E-06

Y17G9B.5	204	276	250	293	2.32E-05	2.71E-05	2.58E-05	3.65E-05
Y17G9B.6	4	8	6	3	5.04E-06	4.52E-06	1.82E-06	3.17E-06
Y17G9B.7	5	5	5	5	2.16E-05	2.76E-05	1.72E-05	2.49E-05
Y17G9B.8	34	43	39	64	2.80E-06	2.65E-06	1.82E-06	2.25E-06
Y17G9B.9	511	422	698	695	2.80E-06	2.65E-06	1.82E-06	2.25E-06
Y18D10A.1	665	532	544	1136	3.98E-06	4.76E-06	2.97E-06	6.03E-06
Y18D10A.10	4	3	4	0	4.46E-05	3.48E-05	3.96E-05	4.87E-05
Y18D10A.11	873	611	1061	1150	1.52E-05	1.15E-05	8.09E-06	2.08E-05
Y18D10A.12	0	6	2	0	2.80E-06	2.65E-06	1.82E-06	2.25E-06
Y18D10A.13	1100	919	1289	2146	1.05E-04	6.93E-05	8.29E-05	1.11E-04
Y18D10A.16	118	131	307	103	2.80E-06	2.65E-06	1.82E-06	2.25E-06
Y18D10A.18	13	14	12	3	1.59E-05	1.25E-05	1.21E-05	2.49E-05
Y18D10A.19.	2639	2185	2885	1485	2.81E-05	2.95E-05	4.76E-05	1.97E-05
Y18D10A.19.	2298	1814	2301	1297	2.80E-06	2.65E-06	1.82E-06	2.25E-06
Y18D10A.2	3	2	14	6	5.85E-04	4.58E-04	4.16E-04	2.65E-04
Y18D10A.21	14	20	5	9	5.12E-04	3.82E-04	3.33E-04	2.32E-04
Y18D10A.22	11	12	0	10	2.80E-06	2.65E-06	1.82E-06	2.25E-06
Y18D10A.23	69	71	70	93	2.80E-06	2.65E-06	1.82E-06	2.25E-06
Y18D10A.24	10	10	56	59	2.80E-06	2.65E-06	1.82E-06	2.25E-06
Y18D10A.25	242	231	164	183	5.04E-06	4.89E-06	3.32E-06	5.44E-06
Y18D10A.26	4	3	10	5	2.80E-06	2.65E-06	6.80E-06	8.84E-06
Y18D10A.3	13	21	48	30	2.62E-05	2.36E-05	1.15E-05	1.59E-05
Y18D10A.4	4	1	2	5	2.80E-06	2.65E-06	1.82E-06	2.25E-06
Y18D10A.5	649	656	516	942	2.80E-06	2.65E-06	3.33E-06	2.59E-06
Y18D10A.6a	131	144	235	193	2.80E-06	2.65E-06	1.82E-06	2.25E-06
Y18D10A.6b.	85	110	154	150	5.29E-05	5.05E-05	2.74E-05	6.16E-05
Y18D10A.6b.	112	121	194	171	4.96E-06	5.16E-06	5.79E-06	5.87E-06
Y18D10A.7a	29	43	27	24	4.65E-06	5.69E-06	5.48E-06	6.61E-06
Y18D10A.7b.	32	40	33	26	5.07E-06	5.18E-06	5.72E-06	6.23E-06
Y18D10A.7b.	29	34	24	22	2.80E-06	2.65E-06	1.82E-06	2.25E-06
Y18D10A.7c	29	43	27	24	2.80E-06	2.65E-06	1.82E-06	2.25E-06
Y18D10A.8	126	168	109	148	2.80E-06	2.65E-06	1.82E-06	2.25E-06
Y18D10A.9	89	109	90	99	2.80E-06	2.65E-06	1.82E-06	2.25E-06
Y18H1A.1	14	35	18	16	7.08E-06	8.94E-06	3.99E-06	6.68E-06
Y18H1A.10	16	32	10	12	9.83E-06	1.14E-05	6.47E-06	8.79E-06
Y18H1A.11	9	22	13	12	2.80E-06	2.65E-06	1.82E-06	2.25E-06
Y18H1A.12	13	26	28	24	2.80E-06	2.65E-06	1.82E-06	2.25E-06
Y18H1A.13	17	26	14	5	2.80E-06	2.83E-06	1.82E-06	2.25E-06
Y18H1A.14	3	3	3	2	2.80E-06	3.04E-06	2.26E-06	2.38E-06
Y18H1A.15	18	27	19	9	2.80E-06	3.02E-06	1.82E-06	2.25E-06
Y18H1A.2	88	86	123	131	2.80E-06	2.65E-06	1.82E-06	2.25E-06
Y18H1A.3	431	386	530	736	2.80E-06	2.65E-06	1.82E-06	2.25E-06
Y18H1A.6	247	181	286	381	1.04E-05	9.65E-06	9.51E-06	1.25E-05
Y18H1A.7	105	83	119	220	1.17E-05	9.92E-06	9.37E-06	1.61E-05
Y18H1A.8	4	4	0	3	1.23E-05	8.57E-06	9.31E-06	1.53E-05
Y18H1A.9	50	96	22	20	8.79E-06	6.56E-06	6.47E-06	1.48E-05
Y19D10A.1	5	1	7	3	2.80E-06	2.65E-06	1.82E-06	2.25E-06
Y19D10A.10	7	14	6	17	3.11E-06	5.61E-06	1.82E-06	2.25E-06
Y19D10A.11	16	104	24	92	2.80E-06	2.65E-06	1.82E-06	2.25E-06
Y19D10A.12	510	2161	887	5575	2.80E-06	2.65E-06	1.82E-06	2.25E-06
Y19D10A.13	0	5	3	5	2.80E-06	7.51E-06	1.82E-06	5.65E-06
Y19D10A.15	3	7	5	7	3.49E-05	1.40E-04	3.95E-05	3.07E-04
Y19D10A.16	39	220	57	278	2.80E-06	2.65E-06	1.82E-06	2.25E-06
Y19D10A.17	8	2	5	0	2.80E-06	2.65E-06	1.82E-06	2.25E-06
Y19D10A.2	2	4	3	4	4.40E-06	2.34E-05	4.19E-06	2.52E-05
Y19D10A.3	4	5	2	4	2.80E-06	2.65E-06	1.82E-06	2.25E-06
Y19D10A.4	32	240	33	332	2.80E-06	2.65E-06	1.82E-06	2.25E-06
Y19D10A.5	12	37	11	40	2.80E-06	2.65E-06	1.82E-06	2.25E-06
Y19D10A.6	3	0	1	0	2.80E-06	1.78E-05	1.82E-06	2.09E-05

Y19D10A.7	9	276	7	179	2.80E-06	2.72E-06	1.82E-06	2.50E-06
Y19D10A.8	8	91	19	75	2.80E-06	2.65E-06	1.82E-06	2.25E-06
Y19D10A.9	2156	18158	5271	15876	2.80E-06	2.42E-05	1.82E-06	1.34E-05
Y19D10B.1	4	5	13	8	2.80E-06	7.94E-06	1.82E-06	5.56E-06
Y19D10B.2	3	6	36	6	2.71E-04	2.16E-03	4.31E-04	1.60E-03
Y19D10B.3	3	2	1	0	2.80E-06	2.65E-06	1.82E-06	2.25E-06
Y19D10B.4	13	18	13	14	2.80E-06	2.65E-06	1.82E-06	2.25E-06
Y19D10B.5	18	21	47	40	2.80E-06	2.65E-06	1.82E-06	2.25E-06
Y19D10B.6	25	58	28	45	2.80E-06	2.65E-06	1.82E-06	2.25E-06
Y19D10B.7	5188	224	11504	83	2.80E-06	2.65E-06	1.99E-06	2.25E-06
Y19D2B.1	42	49	55	45	2.80E-06	3.73E-06	1.82E-06	2.45E-06
Y19D2B.2	5	8	6	2	1.02E-03	4.15E-05	1.47E-03	1.31E-05
Y1A5A.1	38	62	70	14	1.82E-05	2.01E-05	1.55E-05	1.57E-05
Y1A5A.2	2	1	0	0	2.80E-06	2.65E-06	1.82E-06	2.25E-06
Y1B5A.1	1	2	1	0	6.66E-06	1.03E-05	8.00E-06	2.25E-06
Y1E3A.1	0	1	2	0	2.80E-06	2.65E-06	1.82E-06	2.25E-06
Y1H11.1	3	6	6	1	2.80E-06	2.65E-06	1.82E-06	2.25E-06
Y1H11.2	11	28	17	6	2.80E-06	2.65E-06	1.82E-06	2.25E-06
Y20C6A.1	18	32	24	27	2.80E-06	2.65E-06	1.82E-06	2.25E-06
Y20C6A.2	21	29	30	18	2.80E-06	3.94E-06	1.82E-06	2.25E-06
Y20C6A.3	1	1	7	5	2.80E-06	2.65E-06	1.82E-06	2.25E-06
Y20F4.2	83	132	138	146	2.80E-06	2.65E-06	1.82E-06	2.25E-06
Y20F4.3	40	45	75	77	2.80E-06	2.65E-06	1.82E-06	2.25E-06
Y20F4.4	289	431	440	457	4.51E-06	6.74E-06	4.87E-06	6.36E-06
Y20F4.5	226	263	379	578	4.31E-06	4.58E-06	5.25E-06	6.66E-06
Y20F4.8	7	11	5	4	7.81E-06	1.10E-05	7.74E-06	9.94E-06
Y22D7AL.1	5	4	1	1	1.16E-05	1.28E-05	1.27E-05	2.39E-05
Y22D7AL.10.[illegible]	861	898	1327	886	2.80E-06	2.65E-06	1.82E-06	2.25E-06
Y22D7AL.10.[illegible]	802	841	1145	836	2.80E-06	2.65E-06	1.82E-06	2.25E-06
Y22D7AL.11	27	34	18	35	1.77E-04	1.74E-04	1.77E-04	1.46E-04
Y22D7AL.12	0	0	1	0	1.61E-04	1.59E-04	1.50E-04	1.35E-04
Y22D7AL.13	1	0	0	0	2.80E-06	2.65E-06	1.82E-06	2.25E-06
Y22D7AL.14	33	35	27	19	2.80E-06	2.65E-06	1.82E-06	2.25E-06
Y22D7AL.16	0	2	1	1	2.80E-06	2.65E-06	1.82E-06	2.25E-06
Y22D7AL.2	0	3	3	6	4.65E-06	4.66E-06	2.48E-06	2.25E-06
Y22D7AL.3	3	1	6	1	2.80E-06	2.65E-06	1.82E-06	2.25E-06
Y22D7AL.4	12	16	6	12	2.80E-06	2.65E-06	1.82E-06	2.25E-06
Y22D7AL.5.1	3038	2488	1281	2427	2.80E-06	2.65E-06	1.82E-06	2.25E-06
Y22D7AL.5.2	2588	2013	1105	2005	2.80E-06	2.65E-06	1.82E-06	2.25E-06
Y22D7AL.6	2	5	5	3	1.61E-04	1.25E-04	4.42E-05	1.03E-04
Y22D7AL.7	46	54	81	83	1.69E-04	1.24E-04	4.69E-05	1.05E-04
Y22D7AL.8	40	66	52	68	2.80E-06	2.65E-06	1.82E-06	2.25E-06
Y22D7AL.9	59	56	77	89	4.34E-06	4.81E-06	4.97E-06	6.30E-06
Y22D7AL.t1	0	0	0	1	3.39E-06	5.29E-06	2.86E-06	4.63E-06
Y22D7AL.t2	0	0	0	1	2.80E-06	2.65E-06	2.15E-06	3.06E-06
Y22D7AL.t3	0	0	0	1	2.80E-06	2.65E-06	1.82E-06	2.25E-06
Y22D7AL.t5	0	0	1	0	2.80E-06	2.65E-06	1.82E-06	2.25E-06
Y22D7AR.1	30	23	43	28	2.80E-06	2.65E-06	1.82E-06	2.25E-06
Y22D7AR.10	245	411	1467	408	2.80E-06	2.65E-06	1.82E-06	2.25E-06
Y22D7AR.11	18	17	18	13	3.19E-06	2.65E-06	2.97E-06	2.38E-06
Y22D7AR.12	35	45	20	14	1.19E-04	1.89E-04	4.65E-04	1.60E-04
Y22D7AR.13.	7	18	22	21	2.80E-06	2.65E-06	1.82E-06	2.25E-06
Y22D7AR.13.	7	13	17	19	2.80E-06	2.65E-06	1.82E-06	2.25E-06
Y22D7AR.14	6	4	7	3	2.80E-06	2.65E-06	1.82E-06	2.25E-06
Y22D7AR.2	21	34	19	23	2.80E-06	2.65E-06	1.82E-06	2.25E-06
Y22D7AR.3	0	1	0	0	2.80E-06	2.65E-06	1.82E-06	2.25E-06
Y22D7AR.5	1	5	2	4	2.80E-06	2.65E-06	1.82E-06	2.25E-06
Y22D7AR.6	33	62	59	72	2.80E-06	2.65E-06	1.82E-06	2.25E-06
Y22D7AR.7	4	12	17	15	2.80E-06	2.65E-06	1.82E-06	2.25E-06

Y22D7AR.8	2	4	2	5	2.88E-06	5.13E-06	3.37E-06	5.08E-06
Y22D7AR.9	28	37	43	35	2.80E-06	2.65E-06	1.82E-06	2.25E-06
Y22F5A.1	19	63	12	33	2.80E-06	2.65E-06	1.82E-06	2.25E-06
Y22F5A.2	5	12	15	7	2.80E-06	2.65E-06	2.10E-06	2.25E-06
Y22F5A.4.1	2050	1973	3129	1428	2.80E-06	5.29E-06	1.82E-06	2.36E-06
Y22F5A.4.2	1838	1774	2722	1355	2.80E-06	2.65E-06	1.82E-06	2.25E-06
Y22F5A.5	647	688	1123	347	2.07E-04	1.89E-04	2.06E-04	1.16E-04
Y22F5A.6	5	10	7	3	2.28E-04	2.08E-04	2.20E-04	1.35E-04
Y23B4A.1	1	1	3	0	7.86E-05	7.90E-05	8.88E-05	3.38E-05
Y23B4A.2	13	17	32	24	2.80E-06	2.65E-06	1.82E-06	2.25E-06
Y23H5A.1a	52	47	26	75	2.80E-06	2.65E-06	1.82E-06	2.25E-06
Y23H5A.1b	131	134	132	208	2.80E-06	2.94E-06	3.81E-06	3.53E-06
Y23H5A.2	47	36	82	76	6.36E-06	5.42E-06	2.06E-06	7.35E-06
Y23H5A.3	359	373	537	591	6.75E-06	6.53E-06	4.43E-06	8.61E-06
Y23H5A.4	20	21	21	8	7.06E-06	5.11E-06	8.02E-06	9.18E-06
Y23H5A.5a	58	99	68	72	3.88E-05	3.81E-05	3.78E-05	5.13E-05
Y23H5A.5b	56	94	65	66	2.80E-06	2.65E-06	1.82E-06	2.25E-06
Y23H5A.5c	61	90	66	74	2.80E-06	4.21E-06	1.99E-06	2.61E-06
Y23H5A.5d.1	58	100	79	80	2.80E-06	4.21E-06	2.00E-06	2.52E-06
Y23H5A.5d.2	59	102	79	80	3.28E-06	4.58E-06	2.31E-06	3.19E-06
Y23H5A.7a	1020	955	999	1590	2.80E-06	4.10E-06	2.24E-06	2.79E-06
Y23H5A.7b	1135	1056	1123	1729	2.80E-06	4.02E-06	2.15E-06	2.68E-06
Y23H5A.8	55	54	49	88	4.19E-05	3.70E-05	2.67E-05	5.24E-05
Y23H5B.1	9	27	20	20	4.28E-05	3.76E-05	2.75E-05	5.23E-05
Y23H5B.2	3	6	9	3	9.88E-06	9.15E-06	5.72E-06	1.27E-05
Y23H5B.3	9	6	4	2	2.80E-06	2.65E-06	1.82E-06	2.25E-06
Y23H5B.4	7	13	14	10	2.80E-06	2.65E-06	1.82E-06	2.25E-06
Y23H5B.5	443	258	531	708	2.80E-06	2.65E-06	1.82E-06	2.25E-06
Y23H5B.6	369	321	319	544	2.80E-06	2.65E-06	1.82E-06	2.25E-06
Y23H5B.7a	8	28	64	26	2.26E-05	1.24E-05	1.76E-05	2.90E-05
Y23H5B.7b	5	2	5	6	1.78E-05	1.47E-05	1.00E-05	2.11E-05
Y23H5B.7c	4	3	6	4	2.80E-06	3.94E-06	6.20E-06	3.10E-06
Y23H5B.8	7	13	3	13	2.80E-06	2.65E-06	1.82E-06	2.25E-06
Y23H5B.9	1	0	0	0	2.80E-06	2.65E-06	1.82E-06	2.25E-06
Y24D9A.1a	637	554	474	878	2.80E-06	2.65E-06	1.82E-06	2.25E-06
Y24D9A.1b	415	348	307	551	2.80E-06	2.65E-06	1.82E-06	2.25E-06
Y24D9A.2	138	121	232	265	3.03E-05	2.49E-05	1.46E-05	3.35E-05
Y24D9A.4c	10002	8117	9226	9515	2.49E-05	1.98E-05	1.20E-05	2.66E-05
Y24D9A.5	28	32	45	54	8.99E-06	7.43E-06	9.82E-06	1.39E-05
Y24D9A.6	7	10	13	5	1.47E-03	1.12E-03	8.80E-04	1.12E-03
Y24D9A.7	12	0	5	5	3.70E-06	3.99E-06	3.86E-06	5.71E-06
Y24D9A.8a.1	994	1431	1517	1414	2.80E-06	2.65E-06	2.00E-06	2.25E-06
Y24D9A.8a.2	1002	1446	1521	1420	5.74E-06	2.65E-06	1.82E-06	2.25E-06
Y24D9A.8a.3	862	1255	1241	1259	1.03E-04	1.40E-04	1.02E-04	1.17E-04
Y24D9A.8b.1	840	1205	1216	1220	1.10E-04	1.51E-04	1.09E-04	1.26E-04
Y24D9A.8b.2	862	1255	1241	1259	9.99E-05	1.37E-04	9.35E-05	1.17E-04
Y24D9B.1	33	64	17	16	6.96E-05	9.43E-05	6.56E-05	8.12E-05
Y24F12A.1	338	332	342	570	9.99E-05	1.37E-04	9.35E-05	1.17E-04
Y24F12A.2	846	777	1102	1047	2.80E-06	3.78E-06	1.82E-06	2.25E-06
Y24F12A.3	15	18	16	21	2.64E-05	2.45E-05	1.74E-05	3.58E-05
Y24F12A.4	27	36	26	46	9.19E-05	7.97E-05	7.79E-05	9.14E-05
Y25C1A.1	53	93	31	35	2.80E-06	2.65E-06	1.82E-06	2.56E-06
Y25C1A.10	5	5	2	5	4.06E-06	5.11E-06	2.53E-06	5.53E-06
Y25C1A.11	5	5	9	10	3.28E-06	5.45E-06	1.82E-06	2.25E-06
Y25C1A.12	2	5	3	3	2.80E-06	2.65E-06	1.82E-06	2.25E-06
Y25C1A.13	84	101	139	102	2.80E-06	2.65E-06	1.82E-06	2.25E-06
Y25C1A.2	2	10	3	1	2.80E-06	2.65E-06	1.82E-06	2.25E-06
Y25C1A.3	1	4	5	2	9.21E-06	1.05E-05	9.93E-06	9.00E-06
Y25C1A.4	10	4	4	1	2.80E-06	2.65E-06	1.82E-06	2.25E-06

Y25C1A.6.1	8	6	12	11	2.80E-06	2.65E-06	1.82E-06	2.25E-06
Y25C1A.6.2	15	12	24	22	2.80E-06	2.65E-06	1.82E-06	2.25E-06
Y25C1A.7a.1	223	217	300	339	2.80E-06	2.65E-06	1.82E-06	2.25E-06
Y25C1A.7a.2	209	200	256	315	2.80E-06	2.65E-06	2.51E-06	2.86E-06
Y25C1A.7b	199	188	248	303	2.90E-05	2.67E-05	2.54E-05	3.54E-05
Y25C1A.7c	274	311	363	471	2.72E-05	2.46E-05	2.17E-05	3.30E-05
Y25C1A.8a	123	135	159	165	2.58E-05	2.30E-05	2.09E-05	3.16E-05
Y25C1A.8b	119	144	161	159	2.07E-05	2.22E-05	1.78E-05	2.86E-05
Y25C1A.9	5	0	6	4	1.33E-05	1.38E-05	1.12E-05	1.43E-05
Y25C1A.t1	0	1	0	1	1.01E-05	1.15E-05	8.87E-06	1.08E-05
Y25C1A.t2	0	1	0	1	2.80E-06	2.65E-06	1.82E-06	2.25E-06
Y26D4A.10	13	18	22	22	2.80E-06	2.65E-06	1.82E-06	2.25E-06
Y26D4A.12	5	2	2	0	2.80E-06	2.65E-06	1.82E-06	2.25E-06
Y26D4A.13	30	34	56	35	2.80E-06	2.65E-06	1.82E-06	2.25E-06
Y26D4A.14	47	71	57	44	2.80E-06	2.65E-06	1.82E-06	2.25E-06
Y26D4A.15	3	3	0	0	2.80E-06	2.65E-06	1.82E-06	2.25E-06
Y26D4A.16	0	1	3	0	2.80E-06	2.65E-06	1.82E-06	2.25E-06
Y26D4A.2	5	4	10	1	2.80E-06	2.65E-06	1.82E-06	2.25E-06
Y26D4A.3	3	5	5	1	2.80E-06	2.65E-06	1.82E-06	2.25E-06
Y26D4A.4.1	1	3	3	0	2.80E-06	2.65E-06	1.82E-06	2.25E-06
Y26D4A.4.2	1	3	3	0	2.80E-06	2.65E-06	1.82E-06	2.25E-06
Y26D4A.5	8	5	10	4	2.80E-06	2.65E-06	1.82E-06	2.25E-06
Y26D4A.6	0	3	2	1	2.80E-06	2.65E-06	1.82E-06	2.25E-06
Y26D4A.8	24	27	14	13	2.80E-06	2.65E-06	2.13E-06	2.25E-06
Y26D4A.9	67	94	111	52	2.80E-06	2.65E-06	1.82E-06	2.25E-06
Y26E6A.1	150	224	295	287	2.80E-06	2.65E-06	1.82E-06	2.25E-06
Y26E6A.2	16	29	22	17	2.80E-06	2.65E-06	1.88E-06	2.25E-06
Y26E6A.3	63	94	76	51	1.24E-05	1.76E-05	1.59E-05	1.91E-05
Y26G10.1	4	6	13	5	2.80E-06	2.65E-06	1.82E-06	2.25E-06
Y26G10.2	6	8	3	5	1.15E-05	1.62E-05	9.00E-06	7.47E-06
Y27F2A.1	7	0	2	3	2.80E-06	2.65E-06	1.82E-06	2.25E-06
Y27F2A.2	3	6	6	1	2.80E-06	2.65E-06	1.82E-06	2.25E-06
Y27F2A.3a	82	91	158	150	2.80E-06	2.65E-06	1.82E-06	2.25E-06
Y27F2A.3b	60	71	112	108	2.80E-06	2.65E-06	1.82E-06	2.25E-06
Y27F2A.4	8	2	4	3	7.92E-06	8.31E-06	9.95E-06	1.16E-05
Y27F2A.5	4	2	1	3	6.89E-06	7.70E-06	8.36E-06	9.96E-06
Y27F2A.6	22	17	19	21	2.80E-06	2.65E-06	1.82E-06	2.25E-06
Y27F2A.7	4	2	8	3	2.80E-06	2.65E-06	1.82E-06	2.25E-06
Y27F2A.8	10	7	8	4	2.80E-06	2.65E-06	1.82E-06	2.25E-06
Y27F2A.9	1	1	1	0	2.80E-06	2.65E-06	1.82E-06	2.25E-06
Y2C2A.1	85	134	109	95	2.80E-06	2.65E-06	1.82E-06	2.25E-06
Y2H9A.1	503	497	813	1028	2.80E-06	2.65E-06	1.82E-06	2.25E-06
Y2H9A.2	3	3	5	4	2.80E-06	2.65E-06	1.82E-06	2.25E-06
Y2H9A.3	880	927	388	304	1.81E-05	1.69E-05	1.90E-05	2.97E-05
Y2H9A.4	18	23	20	18	2.80E-06	2.65E-06	1.82E-06	2.25E-06
Y32B12A.1	1	2	0	0	1.09E-04	1.08E-04	3.12E-05	3.02E-05
Y32B12A.2	2	1	8	2	2.80E-06	2.65E-06	1.82E-06	2.25E-06
Y32B12A.3	6	1	2	1	2.80E-06	2.65E-06	1.82E-06	2.25E-06
Y32B12A.4	0	2	2	0	2.80E-06	2.65E-06	1.82E-06	2.25E-06
Y32B12A.5	1	2	0	1	2.80E-06	2.65E-06	1.82E-06	2.25E-06
Y32B12A.6	7	5	15	4	2.80E-06	2.65E-06	1.82E-06	2.25E-06
Y32B12A.7	4	5	5	1	2.80E-06	2.65E-06	1.82E-06	2.25E-06
Y32B12B.1	5	5	9	8	2.80E-06	2.65E-06	1.82E-06	2.25E-06
Y32B12B.2	334	364	403	628	2.80E-06	2.65E-06	1.82E-06	2.25E-06
Y32B12B.3	5	2	9	3	2.80E-06	2.65E-06	1.82E-06	2.25E-06
Y32B12B.4	439	363	560	833	1.50E-05	1.55E-05	1.18E-05	2.27E-05
Y32B12B.5	8	4	6	2	2.80E-06	2.65E-06	1.82E-06	2.25E-06
Y32B12B.6	6	4	3	1	1.03E-05	8.07E-06	8.58E-06	1.58E-05
Y32B12B.7	5	6	9	5	2.80E-06	2.65E-06	1.82E-06	2.25E-06

Y32B12C.1	4	6	8	9	2.80E-06	2.65E-06	1.82E-06	2.25E-06
Y32B12C.2	3	4	7	4	2.80E-06	2.65E-06	1.82E-06	2.25E-06
Y32B12C.3	4	9	11	8	2.80E-06	2.65E-06	1.82E-06	2.25E-06
Y32F6A.2	25	39	14	17	2.80E-06	2.65E-06	1.82E-06	2.25E-06
Y32F6A.3	992	1092	1400	2153	2.80E-06	2.65E-06	1.82E-06	2.25E-06
Y32F6A.4	244	378	246	436	2.80E-06	2.65E-06	1.82E-06	2.25E-06
Y32F6A.5.1	542	923	772	802	3.76E-05	3.91E-05	3.45E-05	6.56E-05
Y32F6A.6	19	37	11	31	1.50E-05	2.20E-05	9.86E-06	2.16E-05
Y32F6B.1	186	280	152	170	3.86E-05	6.21E-05	3.58E-05	4.59E-05
Y32F6B.2	13	20	13	7	2.80E-06	4.97E-06	1.82E-06	3.55E-06
Y32F6B.3	42	47	26	42	8.01E-06	1.14E-05	4.26E-06	5.87E-06
Y32G9A.1	1	1	2	3	2.80E-06	2.65E-06	1.82E-06	2.25E-06
Y32G9A.10	22	34	66	38	5.04E-06	5.32E-06	2.02E-06	4.05E-06
Y32G9A.11	1	5	1	2	2.80E-06	2.65E-06	1.82E-06	2.25E-06
Y32G9A.12	3	3	3	10	3.81E-06	5.58E-06	7.45E-06	5.31E-06
Y32G9A.13	1	9	1	2	2.80E-06	2.65E-06	1.82E-06	2.25E-06
Y32G9A.2	3	2	1	1	2.80E-06	2.65E-06	1.82E-06	2.27E-06
Y32G9A.3	1	7	1	2	2.80E-06	3.33E-06	1.82E-06	2.25E-06
Y32G9A.4	11	21	13	11	2.80E-06	2.65E-06	1.82E-06	2.25E-06
Y32G9A.5	0	0	1	1	2.80E-06	2.65E-06	1.82E-06	2.25E-06
Y32G9A.6	18	22	18	10	2.80E-06	2.65E-06	1.82E-06	2.25E-06
Y32G9A.8	802	382	974	746	2.80E-06	2.65E-06	1.82E-06	2.25E-06
Y32G9B.1	29	53	21	15	2.80E-06	2.65E-06	1.82E-06	2.25E-06
Y32H12A.1	5	5	7	3	6.33E-05	2.85E-05	5.01E-05	4.73E-05
Y32H12A.2.1	349	326	327	601	2.80E-06	4.23E-06	1.82E-06	2.25E-06
Y32H12A.2.2	322	307	300	584	2.80E-06	2.65E-06	1.82E-06	2.25E-06
Y32H12A.3.1	70	90	81	75	1.92E-05	1.70E-05	1.17E-05	2.66E-05
Y32H12A.3.2	59	78	65	70	1.96E-05	1.76E-05	1.19E-05	2.86E-05
Y32H12A.4	282	349	375	312	7.64E-06	9.28E-06	5.76E-06	6.59E-06
Y32H12A.5.1	380	443	424	690	6.86E-06	8.54E-06	4.90E-06	6.52E-06
Y32H12A.5.2	300	332	336	541	5.38E-05	6.29E-05	4.66E-05	4.78E-05
Y32H12A.6	23	48	29	20	1.92E-05	2.11E-05	1.39E-05	2.80E-05
Y32H12A.7.1	255	362	280	504	1.91E-05	2.00E-05	1.39E-05	2.77E-05
Y32H12A.7.2	254	349	294	509	2.80E-06	5.53E-06	2.30E-06	2.25E-06
Y32H12A.8	470	536	334	744	1.48E-05	1.99E-05	1.06E-05	2.35E-05
Y32H12A.9	16	16	9	20	1.46E-05	1.90E-05	1.10E-05	2.36E-05
Y34B4A.10.1	112	146	59	36	4.37E-06	4.71E-06	2.02E-06	5.58E-06
Y34B4A.10.2	101	131	48	34	1.36E-05	1.28E-05	4.97E-06	1.36E-05
Y34B4A.2	23	50	54	49	7.25E-06	8.94E-06	2.50E-06	2.25E-06
Y34B4A.3	45	50	43	61	7.36E-06	9.05E-06	2.28E-06	2.25E-06
Y34B4A.4a	37	88	50	88	2.80E-06	5.11E-06	3.81E-06	4.25E-06
Y34B4A.4b	38	93	56	89	4.26E-06	4.44E-06	2.64E-06	4.61E-06
Y34B4A.5	45	53	42	37	2.80E-06	3.54E-06	1.82E-06	3.01E-06
Y34B4A.6	704	1000	997	675	2.80E-06	3.41E-06	1.82E-06	2.79E-06
Y34B4A.7.1	57	114	42	98	1.07E-05	1.19E-05	6.49E-06	7.06E-06
Y34B4A.7.2	49	102	32	82	9.70E-05	1.30E-04	8.94E-05	7.47E-05
Y34B4A.8	129	140	135	214	4.56E-06	8.62E-06	2.19E-06	6.30E-06
Y34D9A.1	238	324	276	355	4.82E-06	9.50E-06	2.06E-06	6.48E-06
Y34D9A.10	338	336	451	688	6.30E-06	6.45E-06	4.28E-06	8.39E-06
Y34D9A.11	397	381	1446	236	2.09E-05	2.69E-05	1.58E-05	2.51E-05
Y34D9A.3	126	159	290	299	2.14E-05	2.02E-05	1.86E-05	3.51E-05
Y34D9A.4	459	368	786	816	1.16E-04	1.05E-04	2.74E-04	5.51E-05
Y34D9A.7	55	56	110	167	6.97E-06	8.31E-06	1.04E-05	1.33E-05
Y34D9B.1a	44	64	48	74	2.88E-05	2.18E-05	3.21E-05	4.11E-05
Y34D9B.1b	43	61	41	70	4.96E-06	4.76E-06	6.43E-06	1.21E-05
Y34F4.1	4	15	5	5	2.86E-06	3.91E-06	2.02E-06	3.85E-06
Y34F4.2	6	42	18	22	3.02E-06	4.05E-06	1.88E-06	3.96E-06
Y34F4.3	32	31	18	42	2.80E-06	6.08E-06	1.82E-06	2.25E-06
Y34F4.4	6	8	10	9	2.80E-06	7.35E-06	2.17E-06	3.28E-06

Y34F4.5	78	80	89	65	9.80E-06	8.97E-06	3.59E-06	1.03E-05
Y35H6.1	32	29	20	35	2.80E-06	2.65E-06	1.82E-06	2.25E-06
Y35H6.3	32	22	24	46	1.37E-05	1.33E-05	1.02E-05	9.18E-06
Y36E3A.1	13	12	16	9	7.36E-06	6.32E-06	3.01E-06	6.48E-06
Y37A1A.2	39	93	43	51	5.57E-06	3.62E-06	2.71E-06	6.43E-06
Y37A1A.3	30	62	34	37	2.80E-06	2.65E-06	1.82E-06	2.25E-06
Y37A1B.10	18	10	19	9	2.86E-06	6.40E-06	2.04E-06	2.99E-06
Y37A1B.11	33	55	45	56	2.80E-06	4.02E-06	1.82E-06	2.25E-06
Y37A1B.12	2	4	21	5	2.80E-06	2.65E-06	1.82E-06	2.25E-06
Y37A1B.13	7	11	18	5	2.80E-06	2.80E-06	1.82E-06	2.43E-06
Y37A1B.15	9	10	14	5	2.80E-06	2.65E-06	1.82E-06	2.25E-06
Y37A1B.1a	1440	1367	1678	2883	2.80E-06	2.65E-06	1.82E-06	2.25E-06
Y37A1B.2a	134	134	206	174	2.80E-06	2.65E-06	1.82E-06	2.25E-06
Y37A1B.2b	620	695	937	1263	4.23E-05	3.80E-05	3.21E-05	6.81E-05
Y37A1B.2c	599	610	794	1077	1.66E-05	1.57E-05	1.66E-05	1.73E-05
Y37A1B.2d	590	608	776	1065	3.17E-05	3.36E-05	3.12E-05	5.19E-05
Y37A1B.4	4	1	1	2	3.93E-05	3.79E-05	3.39E-05	5.68E-05
Y37A1B.5	145	558	260	410	3.89E-05	3.79E-05	3.33E-05	5.64E-05
Y37A1B.6	2	3	3	3	2.80E-06	2.65E-06	1.82E-06	2.25E-06
Y37A1B.7	39	83	31	15	1.10E-05	4.01E-05	1.29E-05	2.50E-05
Y37A1B.8	8	11	14	9	2.80E-06	2.65E-06	1.82E-06	2.25E-06
Y37A1B.9	5	10	10	5	6.80E-06	1.36E-05	3.52E-06	2.25E-06
Y37A1B.t1	0	0	1	0	2.80E-06	2.65E-06	1.82E-06	2.25E-06
Y37A1C.1a	529	421	601	858	2.80E-06	2.65E-06	1.82E-06	2.25E-06
Y37A1C.1b	82	59	81	131	2.80E-06	2.65E-06	1.82E-06	2.25E-06
Y37B11A.1	17	23	21	22	2.03E-05	1.53E-05	1.50E-05	2.64E-05
Y37B11A.2	85	126	140	178	1.16E-05	7.91E-06	7.49E-06	1.49E-05
Y37B11A.3	190	170	278	296	2.80E-06	2.65E-06	1.82E-06	2.25E-06
Y37B11A.t2	0	1	0	1	2.80E-06	3.84E-06	2.95E-06	4.61E-06
Y37D8A.1	226	231	271	234	2.09E-05	1.76E-05	1.99E-05	2.61E-05
Y37D8A.10	353	431	525	442	2.80E-06	2.65E-06	1.82E-06	2.25E-06
Y37D8A.11a	269	293	456	465	3.92E-05	3.78E-05	3.06E-05	3.26E-05
Y37D8A.11b	217	233	374	344	5.66E-05	6.52E-05	5.47E-05	5.69E-05
Y37D8A.12b	76	59	73	76	1.92E-05	1.98E-05	2.12E-05	2.67E-05
Y37D8A.13	516	520	535	1002	1.99E-05	2.02E-05	2.23E-05	2.53E-05
Y37D8A.14.1	1678	1942	2838	1834	2.01E-05	1.48E-05	1.26E-05	1.62E-05
Y37D8A.14.2	1434	1654	2245	1631	1.69E-05	1.61E-05	1.14E-05	2.64E-05
Y37D8A.15	47	93	77	35	2.53E-04	2.76E-04	2.78E-04	2.22E-04
Y37D8A.16	88	191	78	88	2.52E-04	2.75E-04	2.57E-04	2.30E-04
Y37D8A.17	139	115	182	250	8.43E-06	1.58E-05	9.00E-06	5.04E-06
Y37D8A.18	152	129	257	167	1.57E-05	3.21E-05	9.04E-06	1.26E-05
Y37D8A.19	555	934	1927	945	1.91E-05	1.49E-05	1.63E-05	2.76E-05
Y37D8A.21.1	96	106	94	150	2.98E-05	2.39E-05	3.27E-05	2.63E-05
Y37D8A.21.2	119	123	123	166	1.73E-04	2.75E-04	3.90E-04	2.36E-04
Y37D8A.22	135	136	351	453	1.26E-05	1.31E-05	8.04E-06	1.58E-05
Y37D8A.23a	85	130	80	108	1.56E-05	1.52E-05	1.05E-05	1.75E-05
Y37D8A.23b	79	119	70	102	9.02E-06	8.60E-06	1.53E-05	2.43E-05
Y37D8A.23c	72	106	67	92	5.52E-06	7.96E-06	3.37E-06	5.62E-06
Y37D8A.25	31	52	83	58	5.35E-06	7.62E-06	3.08E-06	5.56E-06
Y37D8A.26	0	3	0	0	6.02E-06	8.38E-06	3.64E-06	6.18E-06
Y37D8A.3	113	265	81	80	8.15E-06	1.29E-05	1.42E-05	1.23E-05
Y37D8A.4	100	195	207	201	2.80E-06	2.65E-06	1.82E-06	2.25E-06
Y37D8A.5	20	49	11	14	1.20E-05	2.65E-05	5.58E-06	6.79E-06
Y37D8A.6	52	43	106	60	1.30E-05	2.39E-05	1.75E-05	2.09E-05
Y37D8A.8	3	10	3	4	2.80E-06	4.71E-06	1.82E-06	2.25E-06
Y37E11AL.1	4	7	6	5	1.46E-05	1.14E-05	1.94E-05	1.35E-05
Y37E11AL.2	17	23	9	6	2.80E-06	4.42E-06	1.82E-06	2.25E-06
Y37E11AL.3b	172	155	258	423	2.80E-06	2.65E-06	1.82E-06	2.25E-06
Y37E11AL.4	0	1	1	0	2.94E-06	3.76E-06	1.82E-06	2.25E-06

Y37E11AL.5	18	26	12	20	2.69E-05	2.29E-05	2.62E-05	5.31E-05
Y37E11AL.6	5	6	5	6	2.80E-06	2.65E-06	1.82E-06	2.25E-06
Y37E11AL.7	194	223	291	307	2.80E-06	2.65E-06	1.82E-06	2.25E-06
Y37E11AL.8	191	150	336	506	2.80E-06	2.65E-06	1.82E-06	2.25E-06
Y37E11AL.9	4	3	2	2	1.84E-05	2.00E-05	1.80E-05	2.34E-05
Y37E11AM.1	227	255	239	499	8.65E-06	6.40E-06	9.89E-06	1.84E-05
Y37E11AM.2	87	57	245	168	2.80E-06	2.65E-06	1.82E-06	2.25E-06
Y37E11AM.3	218	258	242	413	8.09E-06	8.57E-06	5.54E-06	1.43E-05
Y37E11AM.3	84	96	104	164	1.44E-05	8.89E-06	2.63E-05	2.23E-05
Y37E11AR.1	31	28	28	57	1.28E-05	1.43E-05	9.26E-06	1.95E-05
Y37E11AR.2	114	138	64	120	1.48E-05	1.59E-05	1.19E-05	2.32E-05
Y37E11AR.3a	51	68	52	52	2.80E-06	2.65E-06	1.82E-06	3.10E-06
Y37E11AR.3b	48	66	56	50	8.90E-06	1.02E-05	3.26E-06	7.53E-06
Y37E11AR.3c	45	63	53	50	4.73E-06	5.95E-06	3.13E-06	3.87E-06
Y37E11AR.4	22	19	7	24	4.54E-06	5.90E-06	3.44E-06	3.80E-06
Y37E11AR.5	46	63	72	77	4.28E-06	5.66E-06	3.28E-06	3.82E-06
Y37E11AR.6	33	36	33	35	2.80E-06	2.65E-06	1.82E-06	2.25E-06
Y37E11AR.7	4	7	2	3	3.19E-06	4.10E-06	3.24E-06	4.27E-06
Y37E11B.10a	46	78	33	49	3.42E-06	3.52E-06	2.22E-06	2.90E-06
Y37E11B.10b	51	76	45	51	2.80E-06	2.65E-06	1.82E-06	2.25E-06
Y37E11B.10c	20	27	10	9	3.28E-06	5.26E-06	1.82E-06	2.81E-06
Y37E11B.1a	13	14	17	10	3.19E-06	4.50E-06	1.84E-06	2.56E-06
Y37E11B.1b	12	6	6	5	3.89E-06	4.97E-06	1.82E-06	2.25E-06
Y37E11B.2	20	53	32	32	2.80E-06	2.65E-06	1.82E-06	2.25E-06
Y37E11B.3	58	51	120	55	2.80E-06	2.65E-06	1.82E-06	2.25E-06
Y37E11B.4	142	170	393	459	2.83E-06	7.09E-06	2.95E-06	3.64E-06
Y37E11B.5	100	143	178	228	1.34E-05	1.11E-05	1.80E-05	1.02E-05
Y37E11B.6	44	48	42	39	4.31E-06	4.89E-06	7.78E-06	1.12E-05
Y37E11B.7	7	7	20	6	5.71E-06	7.72E-06	6.61E-06	1.05E-05
Y37E11B.9	4	1	4	3	1.20E-05	1.24E-05	7.45E-06	8.55E-06
Y37E11C.1	151	120	257	256	2.80E-06	2.65E-06	3.63E-06	2.25E-06
Y37E3.1	131	137	365	320	2.80E-06	2.65E-06	1.82E-06	2.25E-06
Y37E3.10.1	632	899	1358	1561	4.62E-06	3.47E-06	5.12E-06	6.30E-06
Y37E3.10.2	479	639	944	1201	1.09E-05	1.08E-05	1.98E-05	2.14E-05
Y37E3.11	226	471	360	401	4.57E-05	6.14E-05	6.39E-05	9.07E-05
Y37E3.13	22	21	10	15	5.17E-05	6.51E-05	6.63E-05	1.04E-04
Y37E3.15b	264	245	345	485	2.04E-05	4.00E-05	2.11E-05	2.90E-05
Y37E3.16.1	107	99	93	110	2.80E-06	2.65E-06	1.82E-06	2.25E-06
Y37E3.16.2	97	89	85	104	2.51E-05	2.20E-05	2.13E-05	3.70E-05
Y37E3.17a	1121	1313	1081	1567	7.06E-06	6.16E-06	3.99E-06	5.82E-06
Y37E3.17b	1114	1305	1070	1562	7.06E-06	6.11E-06	4.01E-06	6.07E-06
Y37E3.19	7	3	4	2	5.04E-05	5.57E-05	3.16E-05	5.65E-05
Y37E3.3	247	269	785	182	4.58E-05	5.07E-05	2.86E-05	5.16E-05
Y37E3.5a	8	5	10	9	2.80E-06	2.65E-06	1.82E-06	2.25E-06
Y37E3.5b	7	5	9	7	8.18E-05	8.42E-05	1.69E-04	4.84E-05
Y37E3.7.1	7228	6762	12570	4983	2.80E-06	2.65E-06	1.82E-06	2.25E-06
Y37E3.7.2	6270	6014	9083	4043	2.80E-06	2.65E-06	1.82E-06	2.25E-06
Y37E3.8a.1	6998	7889	18856	5761	1.79E-03	1.58E-03	2.02E-03	9.90E-04
Y37E3.8a.2	6322	7042	15342	5365	1.58E-03	1.43E-03	1.49E-03	8.17E-04
Y37E3.8b.1	4124	4504	10576	4009	1.62E-03	1.73E-03	2.85E-03	1.07E-03
Y37E3.8b.2	6322	7042	15342	5365	1.47E-03	1.54E-03	2.32E-03	9.99E-04
Y37E3.9	1389	1466	1409	1729	1.09E-03	1.13E-03	1.83E-03	8.55E-04
Y37F4.1	2	15	1	2	1.47E-03	1.54E-03	2.32E-03	9.99E-04
Y37F4.2	0	0	2	1	1.44E-04	1.44E-04	9.51E-05	1.44E-04
Y37F4.3	1	1	0	0	2.80E-06	2.65E-06	1.82E-06	2.25E-06
Y37F4.4	0	3	2	4	2.80E-06	2.65E-06	1.82E-06	2.25E-06
Y37F4.5	12	21	3	5	2.80E-06	2.65E-06	1.82E-06	2.25E-06
Y37F4.6	41	48	32	53	2.80E-06	2.65E-06	1.82E-06	2.25E-06
Y37F4.7	18	13	12	9	2.80E-06	2.65E-06	1.82E-06	2.25E-06

Y37F4.8	1	7	3	1	3.30E-06	3.68E-06	1.82E-06	3.44E-06
Y37H2A.1	198	214	341	381	3.33E-06	2.65E-06	1.82E-06	2.25E-06
Y37H2A.10	2	2	2	1	2.80E-06	2.65E-06	1.82E-06	2.25E-06
Y37H2A.11	1	0	1	0	1.41E-05	1.44E-05	1.59E-05	2.19E-05
Y37H2A.12	55	56	32	69	2.80E-06	2.65E-06	1.82E-06	2.25E-06
Y37H2A.4	26	49	26	44	2.80E-06	2.65E-06	1.82E-06	2.25E-06
Y37H2A.5	223	229	319	418	8.85E-06	8.52E-06	3.35E-06	8.93E-06
Y37H2A.6	36	49	41	50	3.05E-06	5.42E-06	1.99E-06	4.14E-06
Y37H2A.7	22	24	18	18	1.91E-05	1.85E-05	1.78E-05	2.87E-05
Y37H2A.8	26	16	17	17	3.28E-06	4.21E-06	2.42E-06	3.64E-06
Y37H2A.9	21	44	23	30	2.80E-06	2.65E-06	1.82E-06	2.25E-06
Y37H2B.1	1	1	0	3	3.11E-06	2.65E-06	1.82E-06	2.25E-06
Y37H2C.1	13	16	21	10	2.83E-06	5.63E-06	2.02E-06	3.26E-06
Y37H2C.2	4	1	1	2	2.80E-06	2.65E-06	1.82E-06	2.25E-06
Y37H2C.3	3	6	8	5	2.80E-06	2.65E-06	1.82E-06	2.25E-06
Y37H2C.4	3	0	1	4	2.80E-06	2.65E-06	1.82E-06	2.25E-06
Y37H9A.1a	71	69	81	130	2.80E-06	2.65E-06	1.82E-06	2.25E-06
Y37H9A.1b	66	65	81	130	2.80E-06	2.65E-06	1.82E-06	2.25E-06
Y37H9A.2	3	6	1	4	4.98E-06	4.58E-06	3.70E-06	7.33E-06
Y37H9A.3	157	183	236	238	4.73E-06	4.42E-06	3.79E-06	7.49E-06
Y37H9A.4	39	38	55	66	2.80E-06	2.65E-06	1.82E-06	2.25E-06
Y37H9A.5	33	42	65	27	7.39E-06	8.15E-06	7.23E-06	9.00E-06
Y37H9A.6.1	80	93	85	62	7.39E-06	6.80E-06	6.78E-06	1.01E-05
Y37H9A.6.2	72	92	75	59	9.27E-06	1.11E-05	1.19E-05	6.09E-06
Y38A10A.1	5	2	0	4	1.75E-05	1.92E-05	1.21E-05	1.09E-05
Y38A10A.2	50	71	20	16	1.54E-05	1.86E-05	1.05E-05	1.01E-05
Y38A10A.3	7	5	14	3	2.80E-06	2.65E-06	1.82E-06	2.25E-06
Y38A10A.4	6	8	4	1	3.58E-06	4.81E-06	1.82E-06	2.25E-06
Y38A10A.5.1	4875	5335	4882	5606	2.80E-06	2.65E-06	1.82E-06	2.25E-06
Y38A10A.5.2	4229	4599	4158	5093	2.80E-06	2.65E-06	1.82E-06	2.25E-06
Y38A10A.6	1338	1518	1383	1678	3.87E-04	4.00E-04	2.52E-04	3.57E-04
Y38A10A.7	178	203	190	268	3.88E-04	3.99E-04	2.48E-04	3.75E-04
Y38A8.1	43	73	33	38	8.72E-05	9.35E-05	5.87E-05	8.79E-05
Y38A8.2.1	1132	1126	1482	1501	2.90E-05	3.13E-05	2.02E-05	3.51E-05
Y38A8.2.2	847	867	1037	1122	8.15E-06	1.30E-05	4.06E-06	5.78E-06
Y38A8.3	649	621	996	1305	1.53E-04	1.44E-04	1.30E-04	1.63E-04
Y38C1AA.11	175	197	270	171	1.45E-04	1.40E-04	1.15E-04	1.54E-04
Y38C1AA.12	109	86	264	185	2.36E-05	2.14E-05	2.36E-05	3.82E-05
Y38C1AA.13	77	107	102	95	2.64E-05	2.81E-05	2.65E-05	2.07E-05
Y38C1AA.1a	153	143	392	343	5.12E-06	3.84E-06	8.09E-06	6.99E-06
Y38C1AA.1b	21	30	67	61	1.13E-05	1.49E-05	9.75E-06	1.12E-05
Y38C1AA.1c	52	79	182	217	1.09E-05	9.63E-06	1.82E-05	1.96E-05
Y38C1AA.2	439	466	548	744	3.50E-06	4.73E-06	7.29E-06	8.19E-06
Y38C1AA.3	68	133	108	126	5.38E-06	7.72E-06	1.23E-05	1.81E-05
Y38C1AA.4	100	81	128	108	2.98E-05	2.99E-05	2.43E-05	4.06E-05
Y38C1AA.5a	22	28	14	19	7.56E-06	1.40E-05	7.82E-06	1.12E-05
Y38C1AA.5b	24	27	14	20	6.47E-06	4.95E-06	5.39E-06	5.62E-06
Y38C1AA.6	31	33	23	34	2.80E-06	2.65E-06	1.82E-06	2.25E-06
Y38C1AA.7	138	331	187	64	2.80E-06	2.65E-06	1.82E-06	2.25E-06
Y38C1AA.8	2	9	5	10	2.80E-06	2.80E-06	1.82E-06	2.45E-06
Y38C1AA.9	7	10	7	2	3.22E-05	7.30E-05	2.84E-05	1.20E-05
Y38C1AB.1	27	30	22	18	2.80E-06	3.84E-06	1.82E-06	3.62E-06
Y38C1AB.2	9	18	14	6	2.80E-06	2.65E-06	1.82E-06	2.25E-06
Y38C1AB.3	0	4	5	3	3.84E-06	4.05E-06	2.04E-06	2.25E-06
Y38C1AB.4	172	215	152	330	2.80E-06	2.65E-06	1.82E-06	2.25E-06
Y38C1AB.5	27	30	22	18	2.80E-06	2.65E-06	1.82E-06	2.25E-06
Y38C1AB.6	9	18	14	6	4.62E-06	5.45E-06	2.64E-06	7.11E-06
Y38C1AB.7	0	4	5	3	3.84E-06	4.05E-06	2.04E-06	2.25E-06
Y38C1AB.8	172	218	158	332	2.80E-06	2.65E-06	1.82E-06	2.25E-06

Y38C1BA.1	14	10	12	30	2.80E-06	2.65E-06	1.82E-06	2.25E-06
Y38C1BA.2a	17	30	40	21	4.48E-06	5.37E-06	2.68E-06	6.93E-06
Y38C1BA.2b	12	23	30	14	2.80E-06	2.65E-06	1.82E-06	3.51E-06
Y38C1BA.3	608	591	159	185	2.80E-06	2.65E-06	1.86E-06	2.25E-06
Y38C9A.1	386	360	489	792	2.80E-06	2.65E-06	1.82E-06	2.25E-06
Y38C9A.2.1	680	618	766	1109	5.56E-05	5.10E-05	9.46E-06	1.36E-05
Y38C9A.2.2	572	506	582	847	9.55E-06	8.41E-06	7.87E-06	1.57E-05
Y38C9B.1	38	52	40	34	3.30E-05	2.84E-05	2.42E-05	4.33E-05
Y38C9B.2	1	2	4	0	3.41E-05	2.85E-05	2.26E-05	4.05E-05
Y38C9B.3	2	1	5	1	2.83E-06	3.65E-06	1.93E-06	2.25E-06
Y38E10A.1	0	2	1	0	2.80E-06	2.65E-06	1.82E-06	2.25E-06
Y38E10A.10	7	9	5	6	2.80E-06	2.65E-06	1.82E-06	2.25E-06
Y38E10A.11	0	3	0	3	2.80E-06	2.65E-06	1.82E-06	2.25E-06
Y38E10A.12	3	5	0	4	2.80E-06	2.65E-06	1.82E-06	2.25E-06
Y38E10A.13	44	171	110	131	2.80E-06	2.65E-06	1.82E-06	2.25E-06
Y38E10A.14	230	4605	489	2218	2.80E-06	2.65E-06	1.82E-06	2.25E-06
Y38E10A.15	4	380	7	213	1.46E-05	5.35E-05	2.37E-05	3.49E-05
Y38E10A.16	1	32	9	35	1.90E-05	3.59E-04	2.63E-05	1.47E-04
Y38E10A.17	82	134	39	34	2.80E-06	1.24E-04	1.82E-06	5.90E-05
Y38E10A.18	9	7	2	4	2.80E-06	1.51E-05	2.92E-06	1.40E-05
Y38E10A.19	6	2	1	0	5.07E-06	7.80E-06	1.82E-06	2.25E-06
Y38E10A.2	0	4	4	1	2.80E-06	2.65E-06	1.82E-06	2.25E-06
Y38E10A.20	6	13	5	9	2.80E-06	2.65E-06	1.82E-06	2.25E-06
Y38E10A.21	8	15	22	31	2.80E-06	2.65E-06	1.82E-06	2.25E-06
Y38E10A.22	127	68	235	356	2.80E-06	2.65E-06	1.82E-06	2.25E-06
Y38E10A.23	51	56	115	126	2.80E-06	2.65E-06	2.28E-06	3.96E-06
Y38E10A.25	2	3	1	0	1.55E-05	7.83E-06	1.87E-05	3.49E-05
Y38E10A.26	18	32	30	31	1.24E-05	1.28E-05	1.81E-05	2.45E-05
Y38E10A.28	7	32	13	18	2.80E-06	2.65E-06	1.82E-06	2.25E-06
Y38E10A.3	3	11	3	3	9.32E-06	1.57E-05	1.01E-05	1.29E-05
Y38E10A.4	19	16	28	22	2.80E-06	2.83E-06	1.82E-06	2.25E-06
Y38E10A.5	51	26	42	25	2.80E-06	2.65E-06	1.82E-06	2.25E-06
Y38E10A.6a	655	487	626	1039	2.80E-06	2.65E-06	1.82E-06	2.25E-06
Y38E10A.6b	698	516	654	1098	4.48E-06	2.65E-06	2.39E-06	2.25E-06
Y38E10A.7	51	100	36	30	1.82E-05	1.28E-05	1.13E-05	2.32E-05
Y38E10A.8	4	7	1	3	1.86E-05	1.30E-05	1.13E-05	2.35E-05
Y38E10A.9	2	1	1	0	6.30E-06	1.17E-05	2.90E-06	2.99E-06
Y38E10A.t1	0	0	1	0	2.80E-06	2.65E-06	1.82E-06	2.25E-06
Y38F1A.1	29	61	18	18	2.80E-06	2.65E-06	1.82E-06	2.25E-06
Y38F1A.2	23	38	54	67	2.80E-06	2.65E-06	1.82E-06	2.25E-06
Y38F1A.3	222	351	89	134	3.84E-06	7.62E-06	1.82E-06	2.25E-06
Y38F1A.4	9	8	6	6	2.80E-06	4.13E-06	4.05E-06	6.18E-06
Y38F1A.5.1	90	62	60	114	8.76E-06	1.31E-05	2.28E-06	4.25E-06
Y38F1A.5.2	74	58	48	92	2.80E-06	2.65E-06	1.82E-06	2.25E-06
Y38F1A.6.1	1067	2767	1247	3743	5.66E-06	3.68E-06	2.46E-06	5.76E-06
Y38F1A.6.2	1043	2722	1233	3697	4.12E-06	3.04E-06	1.82E-06	4.12E-06
Y38F1A.6.3	1046	2728	1241	3703	7.16E-05	1.75E-04	5.45E-05	2.02E-04
Y38F1A.7	5	10	6	6	7.26E-05	1.79E-04	5.58E-05	2.07E-04
Y38F1A.8	23	57	23	20	7.36E-05	1.81E-04	5.68E-05	2.09E-04
Y38F1A.9	151	192	469	163	2.80E-06	2.65E-06	1.82E-06	2.25E-06
Y38F2AL.1	54	58	53	83	2.80E-06	5.66E-06	1.82E-06	2.25E-06
Y38F2AL.2	4	9	6	2	5.17E-05	6.21E-05	1.05E-04	4.48E-05
Y38F2AL.3a	1789	2151	1221	2631	7.00E-06	7.12E-06	4.46E-06	8.64E-06
Y38F2AL.3b	743	872	447	904	2.80E-06	2.65E-06	1.82E-06	2.25E-06
Y38F2AL.4	714	765	2653	1386	1.24E-04	1.41E-04	5.51E-05	1.46E-04
Y38F2AL.5	4	3	6	5	1.20E-04	1.34E-04	4.72E-05	1.18E-04
Y38F2AL.6	16	18	37	29	5.71E-05	5.78E-05	1.38E-04	8.91E-05
Y38F2AR.1	85	104	232	189	2.80E-06	2.65E-06	1.82E-06	2.25E-06
Y38F2AR.10	9	18	4	5	5.35E-06	5.69E-06	8.05E-06	7.78E-06

Y38F2AR.13	35	26	60	80	5.26E-06	6.08E-06	9.35E-06	9.40E-06
Y38F2AR.14	0	4	0	2	2.80E-06	3.44E-06	1.82E-06	2.25E-06
Y38F2AR.2.1	1970	1899	1389	1401	4.34E-06	3.04E-06	4.83E-06	7.94E-06
Y38F2AR.2.2	1125	1098	871	1040	2.80E-06	3.31E-06	1.82E-06	2.25E-06
Y38F2AR.3	110	116	115	133	2.70E-04	2.46E-04	1.24E-04	1.54E-04
Y38F2AR.5	161	126	243	364	2.16E-04	1.99E-04	1.09E-04	1.60E-04
Y38F2AR.6	118	93	148	253	1.44E-05	1.43E-05	9.78E-06	1.40E-05
Y38F2AR.7	225	285	189	350	1.12E-05	8.31E-06	1.10E-05	2.04E-05
Y38F2AR.9.1	907	789	2109	729	7.78E-06	5.79E-06	6.36E-06	1.34E-05
Y38F2AR.9.2	650	556	1168	565	1.07E-05	1.28E-05	5.85E-06	1.34E-05
Y38H6A.1	15	11	21	23	2.38E-04	1.95E-04	3.60E-04	1.54E-04
Y38H6A.2	4	3	7	2	1.93E-04	1.56E-04	2.25E-04	1.34E-04
Y38H6A.3	1	6	4	2	2.80E-06	2.65E-06	2.33E-06	3.15E-06
Y38H6A.4	5	2	7	2	2.80E-06	2.65E-06	1.82E-06	2.25E-06
Y38H6C.10	5	17	7	14	2.80E-06	2.65E-06	1.82E-06	2.25E-06
Y38H6C.11	20	34	22	44	2.80E-06	2.65E-06	1.82E-06	2.25E-06
Y38H6C.12	3	7	2	2	2.80E-06	2.65E-06	1.82E-06	2.25E-06
Y38H6C.13	2	6	7	1	2.80E-06	3.31E-06	1.82E-06	3.62E-06
Y38H6C.14	12	16	15	17	2.80E-06	2.65E-06	1.82E-06	2.25E-06
Y38H6C.15	12	14	12	1	2.80E-06	2.65E-06	1.82E-06	2.25E-06
Y38H6C.16	38	49	24	26	2.80E-06	2.65E-06	1.82E-06	2.25E-06
Y38H6C.17	5	16	8	17	2.80E-06	2.65E-06	1.82E-06	2.25E-06
Y38H6C.18	4	3	7	3	5.63E-06	6.85E-06	2.31E-06	3.08E-06
Y38H6C.19	3	4	4	5	2.80E-06	2.65E-06	1.82E-06	2.25E-06
Y38H6C.2	3	4	9	2	2.80E-06	2.65E-06	1.82E-06	2.25E-06
Y38H6C.20	14	5	22	8	2.80E-06	2.65E-06	1.82E-06	2.25E-06
Y38H6C.21	5	27	2	20	2.80E-06	2.65E-06	1.82E-06	2.25E-06
Y38H6C.22	1	3	1	0	2.80E-06	2.65E-06	1.82E-06	2.25E-06
Y38H6C.3	3	0	0	0	2.80E-06	3.41E-06	1.82E-06	2.25E-06
Y38H6C.4	1	0	0	0	2.80E-06	2.65E-06	1.82E-06	2.25E-06
Y38H6C.5	7	12	10	12	2.80E-06	2.65E-06	1.82E-06	2.25E-06
Y38H6C.7	3	2	2	2	2.80E-06	2.65E-06	1.82E-06	2.25E-06
Y38H6C.8	2	2	0	2	2.80E-06	2.65E-06	1.82E-06	2.25E-06
Y38H6C.9	2	9	4	7	2.80E-06	2.65E-06	1.82E-06	2.25E-06
Y38H8A.1	30	56	20	20	2.80E-06	2.65E-06	1.82E-06	2.25E-06
Y38H8A.2a	6	9	7	3	2.80E-06	2.65E-06	1.82E-06	2.25E-06
Y38H8A.2b	3	3	4	2	3.92E-06	6.90E-06	1.82E-06	2.25E-06
Y38H8A.3	64	112	51	24	2.80E-06	2.65E-06	1.82E-06	2.25E-06
Y38H8A.4	33	54	29	20	2.80E-06	2.65E-06	1.82E-06	2.25E-06
Y38H8A.5	2	4	0	2	7.39E-06	1.22E-05	3.83E-06	2.25E-06
Y38H8A.7	17	23	56	48	3.70E-06	5.74E-06	2.11E-06	2.25E-06
Y39A1A.10	3	3	1	0	2.80E-06	2.65E-06	1.82E-06	2.25E-06
Y39A1A.11	81	83	147	101	4.40E-06	5.61E-06	9.40E-06	9.94E-06
Y39A1A.12.1	580	531	955	1215	2.80E-06	2.65E-06	1.82E-06	2.25E-06
Y39A1A.12.2	702	648	1100	1379	1.02E-05	9.87E-06	1.20E-05	1.02E-05
Y39A1A.13	168	172	240	242	3.14E-05	2.71E-05	3.36E-05	5.28E-05
Y39A1A.14	162	222	295	186	3.23E-05	2.82E-05	3.30E-05	5.10E-05
Y39A1A.15a	236	294	312	512	1.62E-05	1.56E-05	1.50E-05	1.87E-05
Y39A1A.15b	244	292	338	539	2.39E-05	3.09E-05	2.83E-05	2.20E-05
Y39A1A.15c	215	280	294	483	7.45E-06	8.78E-06	6.41E-06	1.30E-05
Y39A1A.15d	51	75	65	85	8.62E-06	9.76E-06	7.78E-06	1.53E-05
Y39A1A.16	3	5	11	5	8.65E-06	1.07E-05	7.71E-06	1.56E-05
Y39A1A.17	4	4	8	2	7.28E-06	1.01E-05	6.03E-06	9.74E-06
Y39A1A.18	1	1	2	0	2.80E-06	2.65E-06	1.82E-06	2.25E-06
Y39A1A.19	82	152	50	80	2.80E-06	2.65E-06	1.82E-06	2.25E-06
Y39A1A.1a	241	308	490	611	2.80E-06	2.65E-06	1.82E-06	2.25E-06
Y39A1A.1b	242	307	496	614	5.60E-06	9.79E-06	2.22E-06	4.39E-06
Y39A1A.1c	175	215	351	497	1.79E-05	2.17E-05	2.37E-05	3.65E-05
Y39A1A.2	11	25	6	1	2.32E-05	2.78E-05	3.10E-05	4.73E-05

Y39A1A.20	31	49	59	40	1.83E-05	2.13E-05	2.39E-05	4.18E-05
Y39A1A.21a	87	131	164	108	2.80E-06	4.97E-06	1.82E-06	2.25E-06
Y39A1A.22	121	128	491	587	1.02E-05	1.52E-05	1.26E-05	1.05E-05
Y39A1A.23.1	192	154	198	255	1.32E-05	1.88E-05	1.62E-05	1.32E-05
Y39A1A.23.2	178	143	173	239	6.36E-06	6.35E-06	1.68E-05	2.48E-05
Y39A1A.24	143	99	241	108	1.24E-05	9.42E-06	8.35E-06	1.32E-05
Y39A1A.25	25	41	34	39	1.30E-05	9.84E-06	8.20E-06	1.40E-05
Y39A1A.3	181	156	227	226	2.07E-05	1.35E-05	2.27E-05	1.26E-05
Y39A1A.5	306	248	412	665	5.66E-06	8.76E-06	5.01E-06	7.08E-06
Y39A1A.6	171	158	233	192	2.85E-05	2.32E-05	2.33E-05	2.86E-05
Y39A1A.7	259	395	172	217	1.74E-05	1.33E-05	1.52E-05	3.03E-05
Y39A1A.8	49	75	78	58	2.35E-05	2.05E-05	2.08E-05	2.12E-05
Y39A1A.9	93	134	80	36	5.14E-05	7.41E-05	2.22E-05	3.46E-05
Y39A1A.t1	0	1	1	1	8.09E-06	1.17E-05	8.38E-06	7.69E-06
Y39A1B.1	2	5	4	2	5.35E-06	7.30E-06	3.01E-06	2.25E-06
Y39A1B.2	20	28	13	22	2.80E-06	2.65E-06	1.82E-06	2.25E-06
Y39A1B.3	960	788	1040	1847	2.80E-06	2.65E-06	1.82E-06	2.25E-06
Y39A1C.1	13	15	13	9	2.80E-06	2.65E-06	1.82E-06	2.25E-06
Y39A1C.2	331	357	322	573	2.39E-05	1.85E-05	1.68E-05	3.69E-05
Y39A1C.3.1	3537	2885	3022	3394	4.12E-06	4.47E-06	2.68E-06	2.29E-06
Y39A1C.3.2	2879	2295	2482	2862	1.12E-05	1.14E-05	7.09E-06	1.56E-05
Y39A1C.4	151	159	324	406	3.35E-04	2.58E-04	1.86E-04	2.58E-04
Y39A3A.2	2	3	2	4	3.33E-04	2.51E-04	1.87E-04	2.66E-04
Y39A3A.3	5	5	3	5	9.16E-06	9.10E-06	1.28E-05	1.98E-05
Y39A3A.4	16	12	3	10	2.80E-06	2.65E-06	1.82E-06	2.25E-06
Y39A3A.5	0	0	1	1	2.80E-06	2.65E-06	1.82E-06	2.25E-06
Y39A3A.6	1	3	6	3	2.80E-06	2.65E-06	1.82E-06	2.25E-06
Y39A3A.7	1	4	5	5	2.80E-06	2.65E-06	1.82E-06	2.25E-06
Y39A3B.1	6	14	1	3	2.80E-06	2.65E-06	1.82E-06	2.25E-06
Y39A3B.2	3	6	4	4	2.80E-06	2.65E-06	1.82E-06	2.25E-06
Y39A3B.3	23	24	33	34	2.80E-06	2.65E-06	1.82E-06	2.25E-06
Y39A3B.4	3	6	4	2	2.80E-06	2.65E-06	1.82E-06	2.25E-06
Y39A3B.5a	16	23	16	27	3.98E-06	3.91E-06	3.72E-06	4.72E-06
Y39A3B.5b	13	26	16	24	2.80E-06	2.65E-06	1.82E-06	2.25E-06
Y39A3B.5c	16	21	15	27	2.80E-06	2.65E-06	1.82E-06	2.25E-06
Y39A3B.5d	12	24	17	24	2.80E-06	2.65E-06	1.82E-06	2.25E-06
Y39A3CL.2	59	82	57	67	2.80E-06	2.65E-06	1.82E-06	2.25E-06
Y39A3CL.3	60	93	189	60	2.80E-06	2.65E-06	1.82E-06	2.25E-06
Y39A3CL.4a	138	158	211	251	2.80E-06	2.65E-06	1.82E-06	2.25E-06
Y39A3CL.4b	122	132	178	213	4.39E-05	6.43E-05	9.00E-05	3.53E-05
Y39A3CL.4c	130	146	193	229	1.10E-05	1.19E-05	1.10E-05	1.61E-05
Y39A3CL.5a	128	206	143	195	1.11E-05	1.13E-05	1.05E-05	1.55E-05
Y39A3CL.5b.1	126	200	143	195	1.17E-05	1.24E-05	1.13E-05	1.65E-05
Y39A3CL.5b.2	104	153	110	158	6.24E-06	9.47E-06	4.54E-06	7.62E-06
Y39A3CL.6	21	67	23	63	6.13E-06	9.18E-06	4.52E-06	7.62E-06
Y39A3CL.7a	143	133	232	413	6.41E-06	8.91E-06	4.41E-06	7.83E-06
Y39A3CL.7b	200	178	328	490	2.80E-06	7.30E-06	1.82E-06	5.85E-06
Y39A3CR.1a	84	77	123	152	1.11E-05	9.76E-06	1.17E-05	2.58E-05
Y39A3CR.1b	83	92	145	174	1.83E-05	1.54E-05	1.96E-05	3.61E-05
Y39A3CR.3	159	178	387	560	1.09E-05	9.42E-06	1.04E-05	1.58E-05
Y39A3CR.4	303	314	1653	295	8.23E-06	8.60E-06	9.35E-06	1.38E-05
Y39A3CR.5	6	18	30	17	5.82E-06	6.16E-06	9.24E-06	1.65E-05
Y39A3CR.6	25	44	14	23	9.81E-05	9.60E-05	3.48E-04	7.67E-05
Y39A3CR.7	148	128	180	224	2.80E-06	3.68E-06	4.23E-06	2.97E-06
Y39A3CR.8	18	16	7	8	2.80E-06	4.50E-06	1.82E-06	2.25E-06
Y39B6A.1.1	2526	2424	2450	3136	1.62E-05	1.33E-05	1.28E-05	1.97E-05
Y39B6A.1.2	2350	2256	2301	3041	4.06E-06	3.41E-06	1.82E-06	2.25E-06
Y39B6A.10	79	62	93	84	1.19E-04	1.08E-04	7.51E-05	1.19E-04
Y39B6A.11	27	12	42	25	1.19E-04	1.08E-04	7.56E-05	1.23E-04

Y39B6A.12a	60	63	75	88	1.13E-05	8.36E-06	8.64E-06	9.63E-06
Y39B6A.12b	58	60	71	87	2.80E-06	2.65E-06	2.79E-06	2.25E-06
Y39B6A.13	77	69	125	118	5.74E-06	5.69E-06	4.66E-06	6.77E-06
Y39B6A.14	211	216	385	548	5.68E-06	5.55E-06	4.52E-06	6.84E-06
Y39B6A.16	66	45	71	65	1.37E-05	1.16E-05	1.45E-05	1.69E-05
Y39B6A.17a	33	60	44	45	1.43E-05	1.38E-05	1.70E-05	2.98E-05
Y39B6A.17b	33	66	67	52	8.62E-06	5.55E-06	6.03E-06	6.81E-06
Y39B6A.18	66	58	58	30	2.80E-06	3.94E-06	1.99E-06	2.52E-06
Y39B6A.19	16	22	14	16	2.80E-06	4.42E-06	3.08E-06	2.95E-06
Y39B6A.2	453	341	453	548	2.80E-06	2.65E-06	1.82E-06	2.25E-06
Y39B6A.20.1	14200	9222	18778	15516	2.80E-06	2.65E-06	1.82E-06	2.25E-06
Y39B6A.20.2	12974	8585	17763	15198	3.39E-05	2.41E-05	2.20E-05	3.29E-05
Y39B6A.21	21	22	19	24	1.16E-03	7.13E-04	1.00E-03	1.02E-03
Y39B6A.22	1	2	11	2	1.12E-03	7.01E-04	1.00E-03	1.06E-03
Y39B6A.23	7	6	9	4	2.80E-06	2.65E-06	1.82E-06	2.25E-06
Y39B6A.24.1	19	7	11	8	2.80E-06	2.65E-06	1.82E-06	2.25E-06
Y39B6A.24.2	19	7	9	8	2.80E-06	2.65E-06	1.82E-06	2.25E-06
Y39B6A.25	24	29	26	13	2.80E-06	2.65E-06	1.82E-06	2.25E-06
Y39B6A.27	22	15	21	10	2.80E-06	2.65E-06	1.82E-06	2.25E-06
Y39B6A.29	3	6	6	3	2.80E-06	2.65E-06	1.82E-06	2.25E-06
Y39B6A.30	105	132	54	54	3.16E-06	2.65E-06	1.97E-06	2.25E-06
Y39B6A.31	10	26	8	8	2.80E-06	2.65E-06	1.82E-06	2.25E-06
Y39B6A.32	15	21	23	25	2.80E-06	3.25E-06	1.82E-06	2.25E-06
Y39B6A.34	38	56	94	59	2.80E-06	2.65E-06	1.82E-06	2.25E-06
Y39B6A.35	210	174	263	258	4.12E-06	5.45E-06	4.10E-06	5.51E-06
Y39B6A.36	273	296	390	418	8.88E-06	1.24E-05	1.43E-05	1.11E-05
Y39B6A.37	103	111	185	221	1.97E-05	1.54E-05	1.60E-05	1.94E-05
Y39B6A.38	42	32	86	96	3.46E-05	3.55E-05	3.22E-05	4.26E-05
Y39B6A.39	226	270	355	361	9.04E-06	9.20E-06	1.06E-05	1.56E-05
Y39B6A.4	2	8	7	4	3.81E-06	2.75E-06	5.08E-06	6.99E-06
Y39B6A.40	31	41	31	30	2.30E-05	2.60E-05	2.36E-05	2.96E-05
Y39B6A.41	18	25	25	14	2.80E-06	2.65E-06	1.82E-06	2.25E-06
Y39B6A.42	168	146	175	151	6.02E-06	7.54E-06	3.92E-06	4.68E-06
Y39B6A.43a	111	86	128	204	2.80E-06	2.65E-06	1.82E-06	2.25E-06
Y39B6A.43b	107	85	126	203	5.00E-05	4.11E-05	3.39E-05	3.61E-05
Y39B6A.46	10	9	35	26	5.43E-06	3.97E-06	4.08E-06	8.01E-06
Y39B6A.47	456	421	565	786	5.29E-06	3.97E-06	4.06E-06	8.07E-06
Y39B6A.48	13	27	26	34	2.80E-06	2.65E-06	2.61E-06	2.38E-06
Y39B6A.5	110	72	302	98	1.92E-05	1.67E-05	1.55E-05	2.65E-05
Y39B6A.6	5	11	12	6	2.80E-06	3.49E-06	2.31E-06	3.76E-06
Y39B6A.7	21	43	59	64	2.99E-05	1.85E-05	5.34E-05	2.14E-05
Y39B6A.8	17	11	12	17	2.80E-06	2.65E-06	1.82E-06	2.25E-06
Y39B6A.9	10	13	14	7	2.80E-06	3.84E-06	3.63E-06	4.84E-06
Y39B6A.t2	0	0	0	1	2.80E-06	2.65E-06	1.82E-06	2.25E-06
Y39B6A.t5	0	1	0	0	2.80E-06	2.65E-06	1.82E-06	2.25E-06
Y39B6A.t6	1	0	0	0	2.80E-06	2.65E-06	1.82E-06	2.25E-06
Y39B6A.t7	0	0	1	0	2.80E-06	2.65E-06	1.82E-06	2.25E-06
Y39B6A.t8	0	1	0	1	2.80E-06	2.65E-06	1.82E-06	2.25E-06
Y39C12A.1	27	49	14	24	2.80E-06	2.65E-06	1.82E-06	2.25E-06
Y39C12A.2	69	84	50	63	2.80E-06	2.65E-06	1.82E-06	2.25E-06
Y39C12A.3	10	5	13	4	3.64E-06	6.24E-06	1.82E-06	2.61E-06
Y39C12A.4	8	8	11	6	8.18E-06	9.42E-06	3.86E-06	6.00E-06
Y39C12A.5	8	8	9	4	2.80E-06	2.65E-06	1.82E-06	2.25E-06
Y39C12A.6	8	9	8	0	2.80E-06	2.65E-06	1.82E-06	2.25E-06
Y39C12A.7	3	4	6	5	2.80E-06	2.65E-06	1.82E-06	2.25E-06
Y39C12A.8	116	150	79	143	2.80E-06	2.65E-06	1.82E-06	2.25E-06
Y39C12A.9	9	14	17	8	2.80E-06	2.65E-06	1.82E-06	2.25E-06
Y39D8A.1a	25	53	17	35	1.18E-05	1.44E-05	5.25E-06	1.17E-05
Y39D8A.1b	2	16	2	10	2.80E-06	3.20E-06	2.68E-06	2.25E-06

Y39D8A.1c	1	11	8	11	2.80E-06	3.73E-06	1.82E-06	2.25E-06
Y39D8A.1d	21	51	5	29	2.80E-06	2.91E-06	1.82E-06	2.25E-06
Y39D8B.1	26	23	26	16	2.80E-06	2.65E-06	1.82E-06	2.25E-06
Y39D8B.3	12	16	9	13	2.80E-06	3.84E-06	1.82E-06	2.25E-06
Y39D8C.1	246	309	206	356	2.80E-06	2.65E-06	1.82E-06	2.25E-06
Y39E4A.1	7	12	6	4	2.80E-06	2.65E-06	1.82E-06	2.25E-06
Y39E4A.2a	94	94	82	130	4.98E-06	5.92E-06	2.71E-06	5.80E-06
Y39E4A.2b	95	108	106	140	2.80E-06	2.86E-06	1.82E-06	2.25E-06
Y39E4A.3a.1	695	620	716	858	8.93E-06	8.44E-06	5.07E-06	9.92E-06
Y39E4A.3a.3	658	601	686	842	7.31E-06	7.83E-06	5.30E-06	8.64E-06
Y39E4A.3b	851	715	891	1107	6.00E-05	5.05E-05	4.02E-05	5.95E-05
Y39E4B.1	888	834	1318	1735	5.26E-05	4.54E-05	3.57E-05	5.41E-05
Y39E4B.10	63	86	201	306	5.68E-05	4.51E-05	3.87E-05	5.94E-05
Y39E4B.11	16	31	4	5	4.53E-05	4.02E-05	4.37E-05	7.10E-05
Y39E4B.12a.1	478	533	489	791	4.03E-06	5.21E-06	8.38E-06	1.58E-05
Y39E4B.12a.2	492	544	512	810	2.80E-06	4.44E-06	1.82E-06	2.25E-06
Y39E4B.12b	481	538	502	790	2.28E-05	2.40E-05	1.52E-05	3.02E-05
Y39E4B.12c	490	556	512	814	2.29E-05	2.39E-05	1.55E-05	3.02E-05
Y39E4B.13	9	12	7	6	2.25E-05	2.38E-05	1.53E-05	2.97E-05
Y39E4B.14	12	11	8	14	2.28E-05	2.45E-05	1.55E-05	3.05E-05
Y39E4B.2	415	413	700	906	2.80E-06	2.83E-06	1.82E-06	2.25E-06
Y39E4B.3a	362	532	632	738	2.80E-06	2.65E-06	1.82E-06	2.56E-06
Y39E4B.3b	347	515	617	718	3.03E-05	2.85E-05	3.32E-05	5.31E-05
Y39E4B.3c	218	290	366	416	2.82E-05	3.91E-05	3.20E-05	4.61E-05
Y39E4B.4	7	9	4	4	2.61E-05	3.66E-05	3.02E-05	4.34E-05
Y39E4B.5	566	467	913	1114	2.50E-05	3.15E-05	2.73E-05	3.83E-05
Y39E4B.6	112	59	159	160	2.80E-06	2.65E-06	1.82E-06	2.25E-06
Y39E4B.7	172	147	290	407	3.82E-05	2.98E-05	4.01E-05	6.03E-05
Y39E4B.8	12	14	8	7	1.25E-05	6.22E-06	1.15E-05	1.43E-05
Y39E4B.9	9	24	7	14	1.36E-05	1.10E-05	1.49E-05	2.58E-05
Y39F10A.1	5	10	12	6	2.80E-06	2.65E-06	1.82E-06	2.25E-06
Y39F10A.2	11	6	5	6	2.80E-06	2.75E-06	1.82E-06	2.25E-06
Y39F10A.3	8	17	9	4	2.80E-06	2.65E-06	1.82E-06	2.25E-06
Y39F10B.1a	387	355	373	493	2.80E-06	2.65E-06	1.82E-06	2.25E-06
Y39F10C.1	4	13	10	4	2.80E-06	2.65E-06	1.82E-06	2.25E-06
Y39G10AL.1	10	10	8	6	2.40E-05	2.08E-05	1.51E-05	2.46E-05
Y39G10AL.3.1	271	379	487	536	2.80E-06	2.65E-06	1.82E-06	2.25E-06
Y39G10AL.3.2	260	365	413	527	2.80E-06	2.65E-06	1.82E-06	2.25E-06
Y39G10AL.t1	0	1	0	1	2.49E-05	3.30E-05	2.92E-05	3.97E-05
Y39G10AR.1	199	208	239	325	2.38E-05	3.16E-05	2.46E-05	3.88E-05
Y39G10AR.1	156	175	176	286	2.80E-06	2.65E-06	1.82E-06	2.25E-06
Y39G10AR.1	1526	1291	1829	2428	8.04E-06	7.94E-06	6.29E-06	1.05E-05
Y39G10AR.1	1177	977	1508	1708	6.41E-06	6.80E-06	4.72E-06	9.45E-06
Y39G10AR.1	1112	933	1385	1627	8.29E-05	6.62E-05	6.46E-05	1.06E-04
Y39G10AR.1	703	616	1011	1114	8.71E-05	6.83E-05	7.26E-05	1.02E-04
Y39G10AR.1	580	532	845	965	8.59E-05	6.81E-05	6.96E-05	1.01E-04
Y39G10AR.1	816	910	1566	1896	3.85E-05	3.18E-05	3.60E-05	4.90E-05
Y39G10AR.1	768	851	1482	1848	3.47E-05	3.00E-05	3.29E-05	4.64E-05
Y39G10AR.1	150	159	313	115	3.49E-05	3.68E-05	4.36E-05	6.52E-05
Y39G10AR.1	30	35	12	16	3.47E-05	3.63E-05	4.36E-05	6.71E-05
Y39G10AR.1	84	82	102	83	7.95E-06	7.96E-06	1.08E-05	4.88E-06
Y39G10AR.1	487	366	890	1033	4.79E-06	5.29E-06	1.82E-06	2.25E-06
Y39G10AR.1	226	165	380	481	5.71E-06	5.26E-06	4.50E-06	4.52E-06
Y39G10AR.2	417	492	622	879	1.64E-05	1.16E-05	1.95E-05	2.79E-05
Y39G10AR.2	122	104	152	201	1.39E-05	9.58E-06	1.52E-05	2.38E-05
Y39G10AR.2	114	96	132	187	2.34E-05	2.60E-05	2.27E-05	3.95E-05
Y39G10AR.2	4	5	3	5	9.46E-06	7.62E-06	7.67E-06	1.25E-05
Y39G10AR.2	6	26	4	2	9.13E-06	7.25E-06	6.87E-06	1.20E-05
Y39G10AR.3	12	18	22	10	2.80E-06	2.65E-06	1.82E-06	2.25E-06

Y39G10AR.5	23	19	13	12	6.41E-06	2.62E-05	2.77E-06	2.25E-06
Y39G10AR.6	34	22	28	14	2.80E-06	2.65E-06	1.82E-06	2.25E-06
Y39G10AR.7	289	269	526	669	2.80E-06	2.65E-06	1.82E-06	2.25E-06
Y39G10AR.8	1301	1502	1285	1878	2.80E-06	2.65E-06	1.82E-06	2.25E-06
Y39G10AR.9	302	349	411	544	1.83E-05	1.61E-05	2.16E-05	3.40E-05
Y39G8B.10	5	2	6	0	8.70E-05	9.49E-05	5.59E-05	1.01E-04
Y39G8B.1a	206	268	205	242	2.43E-05	2.66E-05	2.16E-05	3.52E-05
Y39G8B.1b	157	196	156	182	2.80E-06	2.65E-06	1.82E-06	2.25E-06
Y39G8B.2	31	49	60	58	2.15E-05	2.64E-05	1.39E-05	2.03E-05
Y39G8B.3	4	8	12	5	1.85E-05	2.18E-05	1.20E-05	1.72E-05
Y39G8B.4	11	10	11	11	2.80E-06	4.07E-06	3.43E-06	4.09E-06
Y39G8B.5	21	19	18	14	2.80E-06	2.65E-06	1.82E-06	2.25E-06
Y39G8B.7	11	30	23	6	2.80E-06	2.65E-06	1.82E-06	2.25E-06
Y39G8B.8	1	3	2	0	2.80E-06	2.65E-06	1.82E-06	2.25E-06
Y39G8B.9	116	119	217	19	2.80E-06	5.61E-06	2.97E-06	2.25E-06
Y39G8C.1	240	272	373	622	2.80E-06	2.65E-06	1.82E-06	2.25E-06
Y39G8C.2	10	13	6	4	4.05E-05	3.92E-05	4.93E-05	5.33E-06
Y39G8C.3a	14	14	17	11	7.34E-06	7.83E-06	7.42E-06	1.52E-05
Y39G8C.3b	1	4	5	2	2.80E-06	2.65E-06	1.82E-06	2.25E-06
Y39G8C.4	2	12	5	3	2.80E-06	2.65E-06	1.82E-06	2.25E-06
Y39G8C.5	85	74	125	187	2.80E-06	2.65E-06	1.82E-06	2.25E-06
Y39H10A.1	2	2	2	2	2.80E-06	2.65E-06	1.82E-06	2.25E-06
Y39H10A.2	5	8	8	6	9.46E-06	7.80E-06	9.07E-06	1.67E-05
Y39H10A.3b	220	222	399	503	2.80E-06	2.65E-06	1.82E-06	2.25E-06
Y39H10A.4	6	5	2	8	2.80E-06	2.65E-06	1.82E-06	2.25E-06
Y39H10A.5	3	2	8	5	1.45E-05	1.38E-05	1.71E-05	2.67E-05
Y39H10A.6	41	73	88	131	2.80E-06	2.65E-06	1.82E-06	2.25E-06
Y39H10A.7a	309	302	469	548	2.80E-06	2.65E-06	1.82E-06	2.25E-06
Y39H10A.7b.	301	294	454	544	2.80E-06	3.39E-06	2.81E-06	5.17E-06
Y39H10A.7b.	216	232	373	439	1.90E-05	1.75E-05	1.87E-05	2.70E-05
Y39H10B.1	2	3	2	1	2.00E-05	1.85E-05	1.96E-05	2.90E-05
Y39H10B.2	33	53	40	30	1.64E-05	1.67E-05	1.84E-05	2.68E-05
Y40A1A.1	5	8	9	4	2.80E-06	2.65E-06	1.82E-06	2.25E-06
Y40A1A.2	8	4	0	7	5.29E-06	8.01E-06	4.17E-06	3.87E-06
Y40A1A.3	13	17	1	9	2.80E-06	2.65E-06	1.82E-06	2.25E-06
Y40B10A.1a	1755	1922	3161	1620	2.80E-06	2.65E-06	1.82E-06	2.25E-06
Y40B10A.2	48	47	100	76	2.80E-06	2.65E-06	1.82E-06	2.25E-06
Y40B10A.3	3	8	8	3	3.57E-04	3.69E-04	4.18E-04	2.64E-04
Y40B10A.4	9	11	4	3	7.90E-06	7.30E-06	1.07E-05	1.00E-05
Y40B10A.5	2	1	4	3	2.80E-06	2.65E-06	1.82E-06	2.25E-06
Y40B10A.6	5	7	7	4	2.80E-06	2.65E-06	1.82E-06	2.25E-06
Y40B10A.7	9	10	3	9	2.80E-06	2.65E-06	1.82E-06	2.25E-06
Y40B10A.8	144	208	203	337	2.80E-06	2.65E-06	1.82E-06	2.25E-06
Y40B10A.9	28	42	32	59	2.80E-06	2.65E-06	1.82E-06	2.25E-06
Y40B10B.1	1	1	3	2	1.15E-05	1.57E-05	1.05E-05	2.16E-05
Y40B10B.2	4	8	5	4	2.80E-06	3.28E-06	1.82E-06	3.94E-06
Y40B1A.1	6	5	7	11	2.80E-06	2.65E-06	1.82E-06	2.25E-06
Y40B1A.2	28	24	33	28	2.80E-06	2.65E-06	1.82E-06	2.25E-06
Y40B1A.3	31	31	74	55	2.94E-06	2.65E-06	2.24E-06	4.34E-06
Y40B1A.4	57	99	115	153	8.65E-06	6.98E-06	6.63E-06	6.95E-06
Y40B1A.5	31	23	47	58	4.96E-06	4.68E-06	7.69E-06	7.04E-06
Y40B1B.3	7	10	6	10	1.04E-05	1.71E-05	1.37E-05	2.25E-05
Y40B1B.5	564	466	553	674	8.57E-06	6.00E-06	8.45E-06	1.29E-05
Y40B1B.6.1	480	462	595	873	2.80E-06	2.65E-06	1.82E-06	2.25E-06
Y40B1B.6.2	409	391	502	761	9.63E-05	7.51E-05	6.14E-05	9.24E-05
Y40B1B.7	293	283	315	157	2.07E-05	1.89E-05	1.67E-05	3.03E-05
Y40C5A.2	19	11	15	4	1.98E-05	1.79E-05	1.58E-05	2.96E-05
Y40C5A.3	46	61	35	36	7.61E-05	6.95E-05	5.33E-05	3.28E-05
Y40C5A.4a	89	136	51	76	2.80E-06	2.65E-06	1.82E-06	2.25E-06

Y40C5A.4b	21	38	15	34	2.80E-06	2.65E-06	1.82E-06	2.25E-06
Y40C7B.1	66	70	52	49	6.19E-06	8.91E-06	2.31E-06	4.25E-06
Y40C7B.3	12	11	14	11	2.80E-06	2.99E-06	1.82E-06	2.27E-06
Y40C7B.4	4	5	15	3	2.80E-06	2.70E-06	1.82E-06	2.25E-06
Y40C7B.5	24	30	47	25	2.80E-06	2.65E-06	1.82E-06	2.25E-06
Y40D12A.1a	212	211	327	433	2.80E-06	2.65E-06	2.22E-06	2.25E-06
Y40D12A.1b	87	89	120	161	2.80E-06	2.65E-06	1.88E-06	2.25E-06
Y40D12A.2	192	312	215	208	1.12E-05	1.06E-05	1.13E-05	1.84E-05
Y40D12A.3	6	4	3	5	1.04E-05	1.01E-05	9.37E-06	1.55E-05
Y40G12A.1	233	256	254	217	1.31E-05	2.02E-05	9.57E-06	1.14E-05
Y40G12A.2	86	103	124	160	2.80E-06	2.65E-06	1.82E-06	2.25E-06
Y40H4A.1a.1	34	45	32	31	3.29E-05	3.41E-05	2.33E-05	2.46E-05
Y40H4A.1a.2	29	40	19	25	1.29E-05	1.45E-05	1.20E-05	1.92E-05
Y40H4A.1b.1	41	53	37	33	2.80E-06	2.65E-06	1.82E-06	2.25E-06
Y40H4A.1b.2	30	43	24	27	2.80E-06	2.65E-06	1.82E-06	2.25E-06
Y40H4A.2	20	25	13	12	2.80E-06	2.65E-06	1.82E-06	2.25E-06
Y40H7A.1	5	3	1	2	2.80E-06	2.65E-06	1.82E-06	2.25E-06
Y40H7A.10	202	316	231	288	2.80E-06	2.65E-06	1.82E-06	2.25E-06
Y40H7A.11	4	7	14	4	2.80E-06	2.65E-06	1.82E-06	2.25E-06
Y40H7A.2	6	18	16	16	2.17E-05	3.21E-05	1.61E-05	2.49E-05
Y40H7A.3	6	13	8	5	2.80E-06	2.65E-06	2.71E-06	2.25E-06
Y40H7A.5	6	5	2	2	2.80E-06	2.65E-06	1.82E-06	2.25E-06
Y40H7A.6	7	8	13	4	2.80E-06	2.65E-06	1.82E-06	2.25E-06
Y40H7A.7	22	27	32	14	2.80E-06	2.65E-06	1.82E-06	2.25E-06
Y40H7A.8	7	2	3	3	2.80E-06	2.65E-06	1.82E-06	2.25E-06
Y40H7A.9	20	23	14	15	3.75E-06	4.34E-06	3.55E-06	2.25E-06
Y41C4A.1	0	9	1	2	2.80E-06	2.65E-06	1.82E-06	2.25E-06
Y41C4A.10	154	153	474	168	3.11E-06	3.39E-06	1.82E-06	2.25E-06
Y41C4A.11	31	30	42	23	2.80E-06	2.65E-06	1.82E-06	2.25E-06
Y41C4A.12a	20	27	24	15	3.32E-05	3.11E-05	6.64E-05	2.91E-05
Y41C4A.12b	19	26	25	16	3.00E-06	2.72E-06	2.64E-06	2.25E-06
Y41C4A.13	163	237	125	139	2.80E-06	3.12E-06	1.90E-06	2.25E-06
Y41C4A.14	77	76	95	141	2.80E-06	2.99E-06	1.99E-06	2.25E-06
Y41C4A.16	532	1350	1644	3275	2.93E-05	4.03E-05	1.46E-05	2.01E-05
Y41C4A.17	11	15	4	5	8.57E-06	7.99E-06	6.89E-06	1.26E-05
Y41C4A.18	9	10	8	7	6.20E-05	1.49E-04	1.25E-04	3.07E-04
Y41C4A.19	134	208	371	407	4.28E-06	5.53E-06	1.82E-06	2.25E-06
Y41C4A.2	11	16	20	10	3.00E-06	3.15E-06	1.82E-06	2.25E-06
Y41C4A.4a	59	110	89	160	1.72E-05	2.53E-05	3.11E-05	4.21E-05
Y41C4A.4b	39	77	63	108	2.80E-06	2.65E-06	1.82E-06	2.25E-06
Y41C4A.4c	70	122	96	156	4.87E-06	8.60E-06	4.79E-06	1.06E-05
Y41C4A.4d	45	83	71	114	4.45E-06	8.28E-06	4.66E-06	9.87E-06
Y41C4A.4e	58	107	85	156	6.55E-06	1.08E-05	5.85E-06	1.17E-05
Y41C4A.5	0	12	1	4	4.59E-06	8.01E-06	4.72E-06	9.36E-06
Y41C4A.6	6	4	13	5	5.15E-06	8.97E-06	4.92E-06	1.11E-05
Y41C4A.7	10	16	4	6	2.80E-06	2.65E-06	1.82E-06	2.25E-06
Y41C4A.8	28	47	31	24	2.80E-06	2.65E-06	2.66E-06	2.25E-06
Y41C4A.9.1	205	183	299	388	2.80E-06	2.65E-06	1.82E-06	2.25E-06
Y41C4A.9.2	185	160	263	355	4.23E-06	6.72E-06	3.06E-06	2.92E-06
Y41D4A.1	2	4	4	0	1.23E-05	1.03E-05	1.16E-05	1.86E-05
Y41D4A.2	112	107	133	64	1.20E-05	9.76E-06	1.11E-05	1.84E-05
Y41D4A.3	3	1	4	1	2.80E-06	2.65E-06	1.82E-06	2.25E-06
Y41D4A.4.1	1141	998	1106	1546	1.37E-05	1.24E-05	1.06E-05	6.30E-06
Y41D4A.4.2	1120	974	1094	1531	2.80E-06	2.65E-06	1.82E-06	2.25E-06
Y41D4A.5	856	787	766	1322	5.87E-05	4.85E-05	3.70E-05	6.39E-05
Y41D4A.6	94	96	154	200	5.89E-05	4.84E-05	3.74E-05	6.47E-05
Y41D4A.7	5	11	6	13	5.91E-05	5.14E-05	3.44E-05	7.34E-05
Y41D4A.8	6	12	15	21	7.64E-06	7.38E-06	8.16E-06	1.31E-05
Y41D4B.1	11	21	15	5	2.80E-06	2.65E-06	1.82E-06	2.25E-06

Y41D4B.10	12	17	12	11	2.80E-06	2.65E-06	1.82E-06	2.25E-06
Y41D4B.11	262	247	162	285	2.80E-06	3.15E-06	1.82E-06	2.25E-06
Y41D4B.12a	25	50	55	55	2.80E-06	2.65E-06	1.82E-06	2.25E-06
Y41D4B.12b	22	38	57	60	2.61E-05	2.32E-05	1.05E-05	2.28E-05
Y41D4B.13	274	230	393	506	3.81E-06	7.19E-06	5.45E-06	6.72E-06
Y41D4B.14	17	21	15	11	3.58E-06	5.87E-06	6.05E-06	7.87E-06
Y41D4B.15	1	1	2	0	2.80E-05	2.22E-05	2.61E-05	4.15E-05
Y41D4B.16	217	249	382	168	3.75E-06	4.36E-06	2.15E-06	2.25E-06
Y41D4B.17	5	27	21	18	2.80E-06	2.65E-06	1.82E-06	2.25E-06
Y41D4B.18	13	14	12	7	1.65E-05	1.79E-05	1.89E-05	1.03E-05
Y41D4B.19a	261	196	331	528	2.80E-06	2.65E-06	1.82E-06	2.25E-06
Y41D4B.19b	1166	946	1418	2306	2.80E-06	2.65E-06	1.82E-06	2.25E-06
Y41D4B.20	5	6	4	4	2.28E-05	1.62E-05	1.88E-05	3.71E-05
Y41D4B.21	31	64	98	133	3.32E-05	2.54E-05	2.62E-05	5.27E-05
Y41D4B.24	5	8	2	4	2.80E-06	2.65E-06	1.82E-06	2.25E-06
Y41D4B.26	3	4	3	14	2.80E-06	4.89E-06	5.16E-06	8.66E-06
Y41D4B.4	304	278	427	653	2.80E-06	2.65E-06	1.82E-06	2.25E-06
Y41D4B.5.1	6548	5772	18720	3476	2.80E-06	2.65E-06	1.82E-06	2.25E-06
Y41D4B.5.2	5030	4737	14293	2981	1.47E-05	1.27E-05	1.35E-05	2.54E-05
Y41D4B.6	13	7	5	15	2.80E-03	2.33E-03	5.21E-03	1.19E-03
Y41D4B.7	20	19	17	13	2.12E-03	1.88E-03	3.92E-03	1.01E-03
Y41D4B.8	10	42	29	16	2.80E-06	2.65E-06	1.82E-06	2.25E-06
Y41D4B.9	42	52	42	45	2.80E-06	2.65E-06	1.82E-06	2.25E-06
Y41E3.11	1093	888	1356	1985	2.80E-06	4.05E-06	1.93E-06	2.25E-06
Y41E3.12	2	4	5	1	4.12E-06	4.81E-06	2.68E-06	3.53E-06
Y41E3.13	1	2	3	1	3.28E-05	2.52E-05	2.65E-05	4.78E-05
Y41E3.14	2	5	2	5	2.80E-06	2.65E-06	1.82E-06	2.25E-06
Y41E3.15	7	4	6	3	2.80E-06	2.65E-06	1.82E-06	2.25E-06
Y41E3.16	271	259	363	564	2.80E-06	2.65E-06	1.82E-06	2.25E-06
Y41E3.17	4	2	3	3	2.80E-06	2.65E-06	1.82E-06	2.25E-06
Y41E3.18	17	20	25	13	1.40E-05	1.27E-05	1.22E-05	2.35E-05
Y41E3.1a	505	552	580	1004	2.80E-06	2.65E-06	1.82E-06	2.25E-06
Y41E3.2	2010	2023	1066	705	2.80E-06	2.65E-06	1.82E-06	2.25E-06
Y41E3.3	10	23	15	14	2.47E-05	2.55E-05	1.84E-05	3.94E-05
Y41E3.4	969	991	1082	1503	2.25E-04	2.14E-04	7.75E-05	6.33E-05
Y41E3.5	27	37	121	17	2.80E-06	2.65E-06	1.82E-06	2.25E-06
Y41E3.6	6	7	6	6	4.37E-05	4.22E-05	3.18E-05	5.44E-05
Y41E3.7a	329	364	405	523	8.15E-06	1.06E-05	2.38E-05	4.12E-06
Y41E3.7b	383	425	447	574	2.80E-06	2.65E-06	1.82E-06	2.25E-06
Y41E3.7c.1	329	364	405	523	2.81E-05	2.94E-05	2.25E-05	3.59E-05
Y41E3.7c.2	239	256	351	428	2.84E-05	2.98E-05	2.16E-05	3.42E-05
Y41E3.8	81	105	111	51	2.81E-05	2.94E-05	2.25E-05	3.59E-05
Y41E3.9	282	256	468	648	2.53E-05	2.56E-05	2.41E-05	3.63E-05
Y41G9A.1	11	16	10	6	2.03E-05	2.49E-05	1.81E-05	1.03E-05
Y41G9A.2	5	9	4	2	7.90E-06	6.77E-06	8.53E-06	1.46E-05
Y41G9A.3	16	12	8	18	2.80E-06	2.65E-06	1.82E-06	2.25E-06
Y41G9A.4a	18	18	30	23	2.80E-06	2.65E-06	1.82E-06	2.25E-06
Y41G9A.4b	65	72	102	84	2.80E-06	2.65E-06	1.82E-06	2.25E-06
Y41G9A.5	15	42	18	18	2.80E-06	2.65E-06	1.82E-06	2.25E-06
Y41G9A.6	5	3	4	4	2.80E-06	2.65E-06	2.44E-06	2.47E-06
Y42A5A.1	27	40	39	30	2.80E-06	3.68E-06	1.82E-06	2.25E-06
Y42A5A.2	41	76	14	22	2.80E-06	2.65E-06	1.82E-06	2.25E-06
Y42A5A.3	15	16	7	4	2.80E-06	2.65E-06	1.82E-06	2.25E-06
Y42A5A.4	4	15	9	1	2.80E-06	4.18E-06	1.82E-06	2.25E-06
Y42A5A.5	14	17	10	13	3.86E-06	3.89E-06	1.82E-06	2.25E-06
Y42G9A.1	14	40	21	26	2.80E-06	2.65E-06	1.82E-06	2.25E-06
Y42G9A.2	23	43	34	10	6.22E-06	7.14E-06	2.90E-06	4.63E-06
Y42G9A.3a	107	174	57	103	2.80E-06	3.09E-06	1.82E-06	2.25E-06
Y42G9A.3b	101	160	54	104	6.24E-06	1.10E-05	6.01E-06	2.25E-06

Y42G9A.4a	791	753	869	1179	9.32E-06	1.43E-05	3.24E-06	7.22E-06
Y42G9A.4c.1	693	674	764	1038	8.65E-06	1.29E-05	3.01E-06	7.15E-06
Y42G9A.4c.2	669	676	747	1041	4.52E-05	4.06E-05	3.23E-05	5.41E-05
Y42G9A.4c.3	646	643	728	1001	4.61E-05	4.24E-05	3.31E-05	5.55E-05
Y42G9A.4d	693	674	764	1038	4.06E-05	3.87E-05	2.95E-05	5.07E-05
Y42G9A.6a	323	371	474	660	4.76E-05	4.48E-05	3.49E-05	5.92E-05
Y42G9A.6b.1	347	387	499	672	4.61E-05	4.24E-05	3.31E-05	5.55E-05
Y42G9A.6b.2	275	306	377	560	1.76E-05	1.91E-05	1.68E-05	2.89E-05
Y42H9AR.1.1	666	684	725	1005	1.89E-05	1.99E-05	1.77E-05	2.93E-05
Y42H9AR.1.2	649	668	705	996	1.70E-05	1.79E-05	1.52E-05	2.78E-05
Y42H9AR.1.3	596	601	632	894	4.07E-05	3.95E-05	2.89E-05	4.94E-05
Y42H9AR.2	7	14	8	8	3.87E-05	3.76E-05	2.73E-05	4.77E-05
Y42H9AR.3	133	164	118	195	4.36E-05	4.15E-05	3.01E-05	5.25E-05
Y42H9AR.4	593	508	666	1038	2.80E-06	2.65E-06	1.82E-06	2.25E-06
Y42H9AR.5	16	25	10	4	8.20E-06	9.55E-06	4.74E-06	9.65E-06
Y42H9B.1	51	92	17	30	2.58E-05	2.09E-05	1.89E-05	3.63E-05
Y42H9B.2	979	979	1233	2126	3.64E-06	5.37E-06	1.82E-06	2.25E-06
Y42H9B.3	250	354	289	386	4.84E-06	8.23E-06	1.82E-06	2.29E-06
Y43B11AL.1	18	8	13	9	1.48E-05	1.39E-05	1.21E-05	2.58E-05
Y43B11AL.2a	2	3	3	5	3.15E-05	4.22E-05	2.37E-05	3.91E-05
Y43B11AL.2a	2	3	2	5	2.80E-06	2.65E-06	1.82E-06	2.25E-06
Y43B11AL.2b	2	4	4	5	2.80E-06	2.65E-06	1.82E-06	2.25E-06
Y43B11AL.3	5	3	14	1	2.80E-06	2.65E-06	1.82E-06	2.25E-06
Y43B11AR.1	176	256	209	300	2.80E-06	2.65E-06	1.82E-06	2.25E-06
Y43B11AR.2	3	6	7	2	2.80E-06	2.65E-06	1.82E-06	2.25E-06
Y43B11AR.3	35	65	39	59	3.81E-05	5.24E-05	2.95E-05	5.22E-05
Y43B11AR.4.	10243	9688	11550	13420	2.80E-06	2.65E-06	1.82E-06	2.25E-06
Y43B11AR.4.	9175	8759	10233	12666	3.42E-06	6.00E-06	2.48E-06	4.63E-06
Y43B11AR.5	3	7	1	2	1.36E-03	1.21E-03	9.96E-04	1.43E-03
Y43B11AR.6	5	4	5	1	1.28E-03	1.16E-03	9.31E-04	1.42E-03
Y43C5A.1	125	125	213	52	2.80E-06	2.65E-06	1.82E-06	2.25E-06
Y43C5A.2	505	796	593	543	2.80E-06	2.65E-06	1.82E-06	2.25E-06
Y43C5A.3	25	83	46	43	4.28E-05	4.04E-05	4.75E-05	1.43E-05
Y43C5A.4	19	32	8	9	3.94E-05	5.86E-05	3.01E-05	3.40E-05
Y43C5A.5	217	308	295	284	4.12E-06	1.30E-05	4.94E-06	5.71E-06
Y43C5A.6a	337	371	415	469	3.25E-06	5.16E-06	1.82E-06	2.25E-06
Y43C5A.6b	338	373	414	468	2.37E-05	3.18E-05	2.10E-05	2.49E-05
Y43C5B.2	6	16	5	4	2.61E-05	2.71E-05	2.09E-05	2.92E-05
Y43C5B.3	40	60	17	29	2.76E-05	2.88E-05	2.20E-05	3.07E-05
Y43D4A.1	3	8	8	12	2.80E-06	2.65E-06	1.82E-06	2.25E-06
Y43D4A.2	110	197	111	152	4.37E-06	6.19E-06	1.82E-06	2.54E-06
Y43D4A.3a	170	183	254	322	2.80E-06	2.75E-06	1.90E-06	3.49E-06
Y43D4A.3b	58	65	109	113	2.35E-05	3.97E-05	1.54E-05	2.60E-05
Y43D4A.4a	136	151	220	279	1.68E-05	1.71E-05	1.63E-05	2.55E-05
Y43D4A.4b	58	65	109	113	1.20E-05	1.27E-05	1.46E-05	1.87E-05
Y43D4A.5	67	84	29	28	1.90E-05	1.99E-05	2.00E-05	3.13E-05
Y43D4A.6	5	6	15	6	1.41E-05	1.49E-05	1.72E-05	2.20E-05
Y43D4A.7	16	20	4	8	2.80E-06	2.65E-06	1.82E-06	2.25E-06
Y43E12A.1	1184	1290	1711	1841	2.80E-06	2.65E-06	1.82E-06	2.25E-06
Y43E12A.2	125	200	67	141	4.23E-06	5.00E-06	1.82E-06	2.25E-06
Y43E12A.3	175	202	301	322	1.04E-04	1.08E-04	9.83E-05	1.31E-04
Y43F11A.1	13	19	12	12	1.14E-05	1.73E-05	3.99E-06	1.03E-05
Y43F11A.4	13	19	12	12	2.02E-05	2.21E-05	2.27E-05	2.99E-05
Y43F11A.5	235	238	327	537	2.80E-06	3.52E-06	1.82E-06	2.25E-06
Y43F11A.6	42	64	38	67	2.80E-06	3.52E-06	1.82E-06	2.25E-06
Y43F4A.1a	72	137	55	60	1.44E-05	1.38E-05	1.30E-05	2.64E-05
Y43F4A.1b	25	27	12	18	4.23E-06	6.06E-06	2.48E-06	5.40E-06
Y43F4A.3	0	2	0	0	3.64E-06	6.56E-06	1.82E-06	2.45E-06
Y43F4A.4	26	18	9	23	6.13E-06	6.24E-06	1.91E-06	3.55E-06

Y43F4A.t1	0	0	2	0	2.80E-06	2.65E-06	1.82E-06	2.25E-06
Y43F4B.10	148	170	235	113	9.16E-06	5.98E-06	2.06E-06	6.50E-06
Y43F4B.2	323	447	454	396	2.80E-06	2.65E-06	2.00E-06	2.25E-06
Y43F4B.3	336	310	405	569	3.42E-05	3.71E-05	3.53E-05	2.10E-05
Y43F4B.4	346	373	554	640	1.74E-05	2.27E-05	1.59E-05	1.71E-05
Y43F4B.5a.1	628	835	920	1151	5.78E-05	5.04E-05	4.53E-05	7.86E-05
Y43F4B.5a.2	568	773	849	1087	3.11E-05	3.16E-05	3.24E-05	4.61E-05
Y43F4B.5b	512	705	754	951	3.66E-05	4.60E-05	3.49E-05	5.39E-05
Y43F4B.7	191	314	225	349	3.56E-05	4.57E-05	3.46E-05	5.47E-05
Y43F4B.9.1	35	41	105	92	3.44E-05	4.48E-05	3.30E-05	5.13E-05
Y43F4B.9.2	32	40	75	85	1.35E-05	2.09E-05	1.03E-05	1.98E-05
Y43F8A.1	4	3	4	3	9.91E-06	1.10E-05	1.94E-05	2.10E-05
Y43F8A.2	81	140	71	68	8.85E-06	1.04E-05	1.35E-05	1.89E-05
Y43F8A.3	33	18	13	13	2.80E-06	2.65E-06	1.82E-06	2.25E-06
Y43F8A.4	11	13	38	12	5.57E-06	9.13E-06	3.19E-06	3.76E-06
Y43F8A.5	31	37	36	33	2.80E-06	2.65E-06	1.82E-06	2.25E-06
Y43F8B.10	8	10	2	4	2.80E-06	2.65E-06	2.50E-06	2.25E-06
Y43F8B.11	7	14	8	3	2.80E-06	2.65E-06	1.82E-06	2.25E-06
Y43F8B.12	3	3	3	6	2.80E-06	2.65E-06	1.82E-06	2.25E-06
Y43F8B.13	15	6	20	8	2.80E-06	2.75E-06	1.82E-06	2.25E-06
Y43F8B.14	14	18	28	17	2.80E-06	2.65E-06	1.82E-06	2.25E-06
Y43F8B.1b	147	170	149	254	2.80E-06	2.65E-06	1.82E-06	2.25E-06
Y43F8B.1c	121	151	129	219	2.80E-06	2.65E-06	1.82E-06	2.25E-06
Y43F8B.2a	129	361	99	143	1.68E-05	1.84E-05	1.11E-05	2.34E-05
Y43F8B.2b	220	529	176	225	1.73E-05	2.04E-05	1.20E-05	2.52E-05
Y43F8B.4	7	3	1	2	1.51E-05	4.00E-05	7.56E-06	1.35E-05
Y43F8B.5	2	2	1	1	2.63E-05	5.97E-05	1.37E-05	2.16E-05
Y43F8B.6	0	0	0	1	2.80E-06	2.65E-06	1.82E-06	2.25E-06
Y43F8B.7	1	2	4	6	2.80E-06	2.65E-06	1.82E-06	2.25E-06
Y43F8B.8	1	2	0	1	2.80E-06	2.65E-06	1.82E-06	2.25E-06
Y43F8B.9	18	15	4	8	2.80E-06	2.65E-06	1.82E-06	2.25E-06
Y43F8C.1	4	17	3	1	2.80E-06	2.65E-06	1.82E-06	2.25E-06
Y43F8C.10	3	4	1	3	3.28E-06	2.65E-06	1.82E-06	2.25E-06
Y43F8C.11	7	15	20	3	2.80E-06	6.82E-06	1.82E-06	2.25E-06
Y43F8C.12	602	522	600	1210	2.80E-06	2.65E-06	1.82E-06	2.25E-06
Y43F8C.13	116	203	140	112	2.80E-06	5.45E-06	5.01E-06	2.25E-06
Y43F8C.14	331	272	462	723	1.85E-05	1.52E-05	1.20E-05	2.99E-05
Y43F8C.15	4	2	0	2	1.18E-05	1.96E-05	9.29E-06	9.18E-06
Y43F8C.16	2	6	4	6	1.48E-05	1.15E-05	1.34E-05	2.60E-05
Y43F8C.17	0	1	0	1	2.80E-06	2.65E-06	1.82E-06	2.25E-06
Y43F8C.18	7	11	5	4	2.80E-06	2.65E-06	1.82E-06	2.25E-06
Y43F8C.19	5	11	2	5	2.80E-06	2.65E-06	1.82E-06	2.25E-06
Y43F8C.20	28	29	64	16	2.80E-06	2.65E-06	1.82E-06	2.25E-06
Y43F8C.3	46	63	83	80	2.80E-06	2.65E-06	1.82E-06	2.25E-06
Y43F8C.4	10	16	18	6	5.10E-06	4.97E-06	7.56E-06	2.34E-06
Y43F8C.5	44	61	24	22	2.80E-06	2.70E-06	2.46E-06	2.92E-06
Y43F8C.6	56	56	113	111	2.80E-06	2.65E-06	1.82E-06	2.25E-06
Y43F8C.8	127	141	156	179	6.41E-06	8.38E-06	2.28E-06	2.56E-06
Y43F8C.9	56	56	31	16	4.28E-06	4.05E-06	5.63E-06	6.84E-06
Y43F8C.t1	1	0	0	0	2.24E-05	2.35E-05	1.79E-05	2.54E-05
Y43F8C.t6	0	1	0	0	1.72E-05	1.62E-05	6.20E-06	3.94E-06
Y43F8C.t7	0	0	2	0	2.80E-06	2.65E-06	1.82E-06	2.25E-06
Y43H11AL.1.	545	520	561	769	2.80E-06	2.65E-06	1.82E-06	2.25E-06
Y43H11AL.1.	308	278	311	409	2.80E-06	2.65E-06	2.00E-06	2.25E-06
Y43H11AL.1.	328	300	322	415	3.49E-05	3.15E-05	2.34E-05	3.96E-05
Y43H11AL.2	254	202	328	439	3.12E-05	2.66E-05	2.05E-05	3.33E-05
Y43H11AL.3	1271	1062	1117	2152	3.26E-05	2.82E-05	2.09E-05	3.32E-05
Y44A6B.1	6	2	8	2	2.99E-05	2.25E-05	2.51E-05	4.15E-05
Y44A6B.2	4	5	6	1	2.04E-05	1.61E-05	1.16E-05	2.77E-05

Y44A6B.3	10	7	8	5	2.80E-06	2.65E-06	1.82E-06	2.25E-06
Y44A6B.4	3	9	5	5	2.80E-06	2.65E-06	1.82E-06	2.25E-06
Y44A6C.1	38	200	39	75	2.80E-06	2.65E-06	1.82E-06	2.25E-06
Y44A6C.2	7	18	10	3	2.80E-06	2.65E-06	1.82E-06	2.25E-06
Y44A6D.1	0	2	0	0	3.47E-06	1.73E-05	2.33E-06	5.51E-06
Y44A6D.2	112	125	138	96	2.80E-06	2.65E-06	1.82E-06	2.25E-06
Y44A6D.3	61	101	46	55	2.80E-06	2.65E-06	1.82E-06	2.25E-06
Y44A6D.4	11	7	26	11	2.05E-05	2.16E-05	1.64E-05	1.41E-05
Y44A6D.5	20	46	25	12	7.17E-06	1.12E-05	3.52E-06	5.20E-06
Y44A6D.6	2	1	3	1	2.80E-06	2.65E-06	1.82E-06	2.25E-06
Y44A6E.1a	24	68	17	56	2.80E-06	3.91E-06	1.82E-06	2.25E-06
Y44A6E.1b	26	73	17	58	2.80E-06	2.65E-06	1.82E-06	2.25E-06
Y44E3A.1a	23	34	44	18	2.80E-06	4.73E-06	1.82E-06	3.31E-06
Y44E3A.1b	23	35	52	17	2.80E-06	4.55E-06	1.82E-06	3.06E-06
Y44E3A.2	45	73	67	78	5.54E-06	7.72E-06	6.89E-06	3.49E-06
Y44E3A.3	97	146	315	118	2.80E-06	3.78E-06	3.88E-06	2.25E-06
Y44E3A.4	218	210	298	444	2.80E-06	3.02E-06	1.90E-06	2.72E-06
Y44E3A.6a	549	555	835	1141	2.64E-05	3.75E-05	5.57E-05	2.58E-05
Y44E3A.6b	544	555	830	1148	1.53E-05	1.39E-05	1.36E-05	2.50E-05
Y44E3B.1a	137	220	263	251	2.14E-05	2.05E-05	2.12E-05	3.58E-05
Y44E3B.1b.1	164	241	330	292	2.16E-05	2.08E-05	2.14E-05	3.66E-05
Y44E3B.1b.2	153	235	316	276	1.60E-05	2.43E-05	2.00E-05	2.36E-05
Y44E3B.1c	127	214	247	234	1.47E-05	2.03E-05	1.92E-05	2.10E-05
Y44E3B.2	12	7	19	8	1.58E-05	2.29E-05	2.12E-05	2.29E-05
Y44F5A.1	187	193	141	164	1.60E-05	2.55E-05	2.03E-05	2.37E-05
Y45F10A.2.1	2727	2206	4043	3740	2.80E-06	2.65E-06	1.82E-06	2.25E-06
Y45F10A.2.2	2298	1804	3370	3316	1.56E-05	1.52E-05	7.65E-06	1.10E-05
Y45F10A.3	20	42	27	24	1.77E-04	1.35E-04	1.71E-04	1.95E-04
Y45F10A.4	59	66	100	109	1.69E-04	1.25E-04	1.61E-04	1.95E-04
Y45F10A.5	28	44	52	9	3.08E-06	6.11E-06	2.71E-06	2.97E-06
Y45F10A.6a	452	606	809	1066	5.82E-06	6.14E-06	6.41E-06	8.61E-06
Y45F10A.6b	477	638	841	1083	7.25E-06	1.07E-05	8.75E-06	2.25E-06
Y45F10B.1	3	4	3	1	1.28E-05	1.62E-05	1.49E-05	2.42E-05
Y45F10B.10	35	48	48	39	1.34E-05	1.70E-05	1.54E-05	2.44E-05
Y45F10B.11	4	8	9	4	2.80E-06	2.65E-06	1.82E-06	2.25E-06
Y45F10B.12	3	5	7	2	2.80E-06	2.65E-06	1.82E-06	2.25E-06
Y45F10B.13a	194	275	134	225	2.80E-06	2.65E-06	1.82E-06	2.25E-06
Y45F10B.13b	210	284	145	237	2.80E-06	2.65E-06	1.82E-06	2.25E-06
Y45F10B.13c	52	104	48	60	1.14E-05	1.53E-05	5.14E-06	1.06E-05
Y45F10B.14	5	8	10	10	1.16E-05	1.49E-05	5.23E-06	1.05E-05
Y45F10B.15	1	0	2	0	1.04E-05	1.97E-05	6.27E-06	9.67E-06
Y45F10B.2	13	7	4	6	2.80E-06	2.65E-06	1.82E-06	2.25E-06
Y45F10B.3	11	9	11	4	2.80E-06	2.65E-06	1.82E-06	2.25E-06
Y45F10B.4	6	5	5	4	2.80E-06	2.65E-06	1.82E-06	2.25E-06
Y45F10B.5	6	6	32	4	5.40E-06	4.18E-06	3.52E-06	2.25E-06
Y45F10B.6	9	4	7	4	2.80E-06	2.65E-06	1.82E-06	2.25E-06
Y45F10B.7	11	11	9	17	2.80E-06	2.65E-06	2.33E-06	2.25E-06
Y45F10B.8	9	9	6	3	2.80E-06	2.65E-06	1.82E-06	2.25E-06
Y45F10B.9	8	9	5	3	2.80E-06	2.65E-06	1.82E-06	2.29E-06
Y45F10C.1	7	22	15	12	2.80E-06	2.65E-06	1.82E-06	2.25E-06
Y45F10C.2	327	730	352	601	2.80E-06	2.65E-06	1.82E-06	2.25E-06
Y45F10C.3	784	821	1102	1466	2.80E-06	2.65E-06	1.82E-06	2.25E-06
Y45F10C.4	329	495	1100	488	9.44E-05	1.99E-04	6.61E-05	1.39E-04
Y45F10C.6	8	6	4	3	6.90E-05	6.83E-05	6.31E-05	1.04E-04
Y45F10D.10	16	30	21	7	9.19E-05	1.31E-04	2.00E-04	1.09E-04
Y45F10D.11a	67	103	119	124	2.80E-06	2.65E-06	1.82E-06	2.25E-06
Y45F10D.11b	17	37	28	36	3.84E-06	6.77E-06	3.26E-06	2.25E-06
Y45F10D.12.1	7621	6003	8767	9267	6.13E-06	8.91E-06	7.09E-06	9.11E-06
Y45F10D.12.2	6244	4934	7976	8821	3.78E-06	7.78E-06	4.05E-06	6.43E-06

Y45F10D.13a	65	81	49	38	1.28E-03	9.54E-04	9.59E-04	1.25E-03
Y45F10D.14	16	7	13	9	1.18E-03	8.80E-04	9.80E-04	1.34E-03
Y45F10D.15	3	4	5	5	4.26E-06	5.00E-06	2.10E-06	2.25E-06
Y45F10D.16	4	12	6	7	2.80E-06	2.65E-06	1.82E-06	2.25E-06
Y45F10D.2	17	11	14	10	2.80E-06	2.65E-06	1.82E-06	2.25E-06
Y45F10D.3a	430	480	447	744	2.80E-06	2.65E-06	1.82E-06	2.25E-06
Y45F10D.3b	404	439	421	715	3.53E-06	2.65E-06	1.90E-06	2.25E-06
Y45F10D.4	246	362	252	211	1.98E-05	2.09E-05	1.34E-05	2.76E-05
Y45F10D.6	8	5	11	0	2.42E-05	2.48E-05	1.64E-05	3.44E-05
Y45F10D.7	322	366	362	595	5.05E-05	7.01E-05	3.36E-05	3.48E-05
Y45F10D.9	425	463	593	736	2.80E-06	2.65E-06	1.82E-06	2.25E-06
Y45F3A.1	14	45	15	11	1.30E-05	1.40E-05	9.53E-06	1.93E-05
Y45F3A.2	69	102	71	95	3.07E-05	3.16E-05	2.79E-05	4.28E-05
Y45F3A.3a.1	252	378	309	343	2.80E-06	4.73E-06	1.82E-06	2.25E-06
Y45F3A.3a.2	232	356	273	322	1.16E-05	1.62E-05	7.76E-06	1.28E-05
Y45F3A.3a.3	233	356	273	322	1.42E-05	2.01E-05	1.13E-05	1.55E-05
Y45F3A.3b.1	228	354	268	319	1.39E-05	2.01E-05	1.06E-05	1.55E-05
Y45F3A.3b.2	232	356	273	322	1.39E-05	2.00E-05	1.06E-05	1.54E-05
Y45F3A.3b.3	233	356	273	322	1.39E-05	2.04E-05	1.07E-05	1.57E-05
Y45F3A.3b.4	228	354	268	318	1.39E-05	2.01E-05	1.06E-05	1.55E-05
Y45F3A.4	9	40	51	32	1.39E-05	2.00E-05	1.06E-05	1.54E-05
Y45F3A.5	9	7	3	3	1.40E-05	2.05E-05	1.07E-05	1.57E-05
Y45F3A.6	10	7	7	7	2.80E-06	5.24E-06	4.61E-06	3.58E-06
Y45F3A.7	2	4	1	1	4.09E-06	3.02E-06	1.82E-06	2.25E-06
Y45F3A.8	13	17	3	7	2.80E-06	2.65E-06	1.82E-06	2.25E-06
Y45F3A.9	58	50	65	125	2.80E-06	2.65E-06	1.82E-06	2.25E-06
Y45G12A.1	4	4	2	2	2.80E-06	2.65E-06	1.82E-06	2.25E-06
Y45G12B.1a	752	835	836	1081	3.92E-06	3.20E-06	2.86E-06	6.81E-06
Y45G12B.1b	217	260	259	347	2.80E-06	2.65E-06	1.82E-06	2.25E-06
Y45G12B.1c.[illegible]	502	576	635	784	3.39E-05	3.56E-05	2.45E-05	3.92E-05
Y45G12B.1c.[illegible]	494	564	603	769	1.88E-05	2.13E-05	1.46E-05	2.42E-05
Y45G12B.2a	153	160	176	204	2.94E-05	3.18E-05	2.42E-05	3.69E-05
Y45G12B.2b	78	71	100	128	2.90E-05	3.12E-05	2.30E-05	3.62E-05
Y45G12B.3	112	88	134	186	1.44E-05	1.42E-05	1.08E-05	1.54E-05
Y45G12C.1	21	35	22	13	9.27E-06	7.96E-06	7.73E-06	1.22E-05
Y45G12C.10	4	5	4	3	9.58E-06	7.12E-06	7.45E-06	1.28E-05
Y45G12C.11	3	8	11	9	2.80E-06	3.23E-06	1.82E-06	2.25E-06
Y45G12C.12	4	8	5	4	2.80E-06	2.65E-06	1.82E-06	2.25E-06
Y45G12C.14	2	2	2	1	2.80E-06	2.65E-06	1.82E-06	2.25E-06
Y45G12C.15	1	5	1	3	2.80E-06	2.65E-06	1.82E-06	2.25E-06
Y45G12C.16	98	176	235	527	2.80E-06	2.65E-06	1.82E-06	2.25E-06
Y45G12C.3	10	42	0	6	2.80E-06	2.65E-06	1.82E-06	2.25E-06
Y45G12C.4	4	10	10	4	1.86E-05	3.15E-05	2.90E-05	8.02E-05
Y45G12C.5	4	1	2	6	2.80E-06	1.01E-05	1.82E-06	2.25E-06
Y45G12C.6	3	1	5	5	2.80E-06	2.65E-06	1.82E-06	2.25E-06
Y45G12C.7	5	6	9	0	2.80E-06	2.65E-06	1.82E-06	2.25E-06
Y45G12C.8	8	2	5	0	2.80E-06	2.65E-06	1.82E-06	2.25E-06
Y45G12C.9	1	5	8	6	2.80E-06	2.65E-06	1.82E-06	2.25E-06
Y45G5AL.1b	367	308	401	602	2.80E-06	2.65E-06	1.82E-06	2.25E-06
Y45G5AL.2	2	8	9	9	2.80E-06	2.65E-06	1.82E-06	2.25E-06
Y45G5AM.1a	110	379	108	355	4.89E-05	3.88E-05	3.47E-05	6.44E-05
Y45G5AM.1a	76	297	60	298	2.80E-06	2.65E-06	1.82E-06	2.25E-06
Y45G5AM.2	368	387	426	640	8.65E-06	2.82E-05	5.54E-06	2.24E-05
Y45G5AM.3	43	44	46	66	6.75E-06	2.49E-05	3.46E-06	2.12E-05
Y45G5AM.4	2	3	2	1	1.21E-05	1.20E-05	9.11E-06	1.69E-05
Y45G5AM.5	8	8	11	4	3.89E-06	3.78E-06	2.71E-06	4.81E-06
Y45G5AM.6	51	73	50	53	2.80E-06	2.65E-06	1.82E-06	2.25E-06
Y45G5AM.7	264	240	217	425	2.80E-06	2.65E-06	1.82E-06	2.25E-06
Y45G5AM.8	147	147	151	236	4.76E-06	6.43E-06	3.02E-06	3.96E-06

Y45G5AM.9a	398	354	538	812	2.11E-05	1.81E-05	1.13E-05	2.73E-05
Y45G5AM.9b	268	208	367	564	8.99E-06	8.49E-06	6.01E-06	1.16E-05
Y45G5AM.9c	335	286	462	734	2.55E-05	2.14E-05	2.24E-05	4.17E-05
Y46B2A.1	50	37	27	80	2.35E-05	1.72E-05	2.09E-05	3.97E-05
Y46B2A.2	14	28	27	9	2.49E-05	2.01E-05	2.23E-05	4.38E-05
Y46B2A.3	5	19	1	0	3.42E-06	2.65E-06	1.82E-06	4.41E-06
Y46C8AL.1	63	137	36	43	2.80E-06	2.65E-06	1.82E-06	2.25E-06
Y46C8AL.2	91	307	146	94	2.80E-06	2.65E-06	1.82E-06	2.25E-06
Y46C8AL.3	5	6	9	7	3.72E-06	7.64E-06	1.82E-06	2.25E-06
Y46C8AL.4	21	12	20	11	7.42E-06	2.37E-05	7.76E-06	6.16E-06
Y46C8AL.5.1	118	139	114	76	2.80E-06	2.65E-06	1.82E-06	2.25E-06
Y46C8AL.8	5	7	5	6	2.80E-06	2.65E-06	1.82E-06	2.25E-06
Y46C8AL.9a	7	12	15	11	8.88E-06	9.87E-06	5.58E-06	4.59E-06
Y46C8AL.9b	5	11	14	5	2.80E-06	2.65E-06	1.82E-06	2.25E-06
Y46C8AR.1.1	8	17	11	4	2.80E-06	2.65E-06	1.82E-06	2.25E-06
Y46C8AR.3	3	3	2	0	2.80E-06	2.65E-06	1.82E-06	2.25E-06
Y46D2A.1	58	52	63	29	2.80E-06	2.65E-06	1.82E-06	2.25E-06
Y46D2A.2	104	96	86	71	2.80E-06	2.65E-06	1.82E-06	2.25E-06
Y46D2A.3	1	0	0	0	4.56E-06	3.86E-06	3.23E-06	2.25E-06
Y46E12A.1	3	7	12	2	1.19E-05	1.03E-05	6.40E-06	6.52E-06
Y46E12A.2	0	0	0	3	2.80E-06	2.65E-06	1.82E-06	2.25E-06
Y46E12A.3	2	5	5	1	2.80E-06	2.65E-06	2.22E-06	2.25E-06
Y46E12A.4	6	5	7	3	2.80E-06	2.65E-06	1.82E-06	2.25E-06
Y46E12BL.1	14	16	17	18	2.80E-06	2.65E-06	1.82E-06	2.25E-06
Y46E12BL.2	375	436	517	805	2.80E-06	2.65E-06	1.82E-06	2.25E-06
Y46E12BL.3	303	289	648	578	2.80E-06	2.65E-06	1.82E-06	2.25E-06
Y46E12BL.4	53	80	215	212	1.00E-05	1.10E-05	9.00E-06	1.73E-05
Y46E12BR.1	9	23	14	16	3.50E-05	3.15E-05	4.87E-05	5.36E-05
Y46G5A.10	137	183	88	122	3.30E-06	4.71E-06	8.73E-06	1.06E-05
Y46G5A.12.1	193	196	303	231	2.80E-06	2.65E-06	1.82E-06	2.25E-06
Y46G5A.12.2	173	180	246	212	6.69E-06	8.44E-06	2.81E-06	4.79E-06
Y46G5A.13	62	98	85	150	2.63E-05	2.53E-05	2.69E-05	2.53E-05
Y46G5A.14	8	14	15	4	2.71E-05	2.67E-05	2.51E-05	2.67E-05
Y46G5A.15	15	20	24	19	5.32E-06	7.94E-06	4.76E-06	1.03E-05
Y46G5A.16	1	3	2	2	2.80E-06	3.97E-06	2.92E-06	2.25E-06
Y46G5A.17.1	1179	1599	1874	2089	2.80E-06	2.65E-06	1.90E-06	2.25E-06
Y46G5A.17.2	1012	1366	1598	1800	2.80E-06	2.65E-06	1.82E-06	2.25E-06
Y46G5A.19.1	58	111	81	90	4.72E-05	6.04E-05	4.88E-05	6.71E-05
Y46G5A.19.2	95	126	123	127	4.62E-05	5.89E-05	4.75E-05	6.60E-05
Y46G5A.1a	302	335	480	606	6.08E-06	1.10E-05	5.52E-06	7.58E-06
Y46G5A.2	58	50	62	80	7.84E-06	9.81E-06	6.61E-06	8.41E-06
Y46G5A.20	16	20	15	11	9.32E-06	9.76E-06	9.64E-06	1.50E-05
Y46G5A.21	136	216	120	226	5.35E-06	4.36E-06	3.72E-06	5.91E-06
Y46G5A.22a	16	29	15	6	3.56E-06	4.21E-06	2.17E-06	2.25E-06
Y46G5A.22b	22	32	20	7	7.48E-06	1.12E-05	4.30E-06	9.99E-06
Y46G5A.23	4	5	1	4	2.80E-06	3.23E-06	1.82E-06	2.25E-06
Y46G5A.24	3	6	5	4	2.80E-06	3.36E-06	1.82E-06	2.25E-06
Y46G5A.25	10	15	13	7	2.80E-06	2.65E-06	1.82E-06	2.25E-06
Y46G5A.26a	11	22	6	16	2.80E-06	2.65E-06	1.82E-06	2.25E-06
Y46G5A.26b	1	7	1	7	2.80E-06	2.65E-06	1.82E-06	2.25E-06
Y46G5A.27	12	19	11	12	2.80E-06	2.65E-06	1.82E-06	2.25E-06
Y46G5A.28	18	13	13	10	2.80E-06	2.65E-06	1.82E-06	2.25E-06
Y46G5A.29	143	213	44	44	2.80E-06	2.65E-06	1.82E-06	2.25E-06
Y46G5A.30	106	165	76	63	2.80E-06	2.65E-06	1.82E-06	2.25E-06
Y46G5A.31	1193	1039	1479	1943	6.41E-06	9.02E-06	1.82E-06	2.25E-06
Y46G5A.34	7	12	7	3	5.04E-06	7.38E-06	2.35E-06	2.41E-06
Y46G5A.35	34	49	72	27	5.53E-05	4.55E-05	4.46E-05	7.23E-05
Y46G5A.36	10	7	1	1	2.80E-06	4.18E-06	1.82E-06	2.25E-06
Y46G5A.37	2	2	4	2	5.57E-06	7.59E-06	7.67E-06	3.55E-06

Y46G5A.38	17	14	6	1	3.02E-06	2.65E-06	1.82E-06	2.25E-06
Y46G5A.39	4	8	2	4	2.80E-06	2.65E-06	1.82E-06	2.25E-06
Y46G5A.4	1725	1593	1737	2810	5.29E-06	4.13E-06	1.82E-06	2.25E-06
Y46G5A.5	309	333	533	555	2.80E-06	2.65E-06	1.82E-06	2.25E-06
Y46G5A.6	91	95	99	121	2.85E-05	2.48E-05	1.87E-05	3.72E-05
Y46G5A.7	13	18	7	9	3.82E-05	3.89E-05	4.29E-05	5.52E-05
Y46G5A.8	17	19	37	24	3.39E-06	3.33E-06	2.41E-06	3.62E-06
Y46H3A.1a	5	3	7	3	2.80E-06	2.65E-06	1.82E-06	2.25E-06
Y46H3A.1b	6	5	8	3	3.28E-06	3.47E-06	4.63E-06	3.71E-06
Y46H3A.2	270	1078	83	81	2.80E-06	2.65E-06	1.82E-06	2.25E-06
Y46H3A.3	173	751	54	60	2.80E-06	2.65E-06	1.82E-06	2.25E-06
Y46H3A.4	8	24	8	3	7.00E-05	2.64E-04	1.40E-05	1.69E-05
Y46H3A.5	2	3	7	6	3.55E-05	1.46E-04	7.22E-06	9.90E-06
Y46H3A.6	473	545	627	799	2.80E-06	2.65E-06	1.82E-06	2.25E-06
Y46H3A.7	159	203	269	227	2.80E-06	2.65E-06	1.82E-06	2.25E-06
Y46H3B.1	24	42	44	55	2.45E-05	2.66E-05	2.11E-05	3.32E-05
Y46H3B.2	22	32	30	45	2.00E-05	2.42E-05	2.21E-05	2.30E-05
Y46H3C.1	6	7	6	0	2.80E-06	2.65E-06	1.91E-06	2.95E-06
Y46H3C.2	8	4	13	4	2.80E-06	2.65E-06	1.82E-06	2.45E-06
Y46H3C.3	1	7	3	4	2.80E-06	2.65E-06	1.82E-06	2.25E-06
Y46H3C.4	106	88	111	144	2.80E-06	2.65E-06	1.82E-06	2.25E-06
Y46H3C.5	24	22	14	20	2.80E-06	2.65E-06	1.82E-06	2.25E-06
Y46H3C.6	7	8	8	8	6.41E-06	5.03E-06	4.37E-06	6.99E-06
Y46H3C.7	32	48	20	42	5.18E-06	4.50E-06	1.97E-06	3.46E-06
Y46H3D.1	22	12	33	3	2.80E-06	2.65E-06	1.82E-06	2.25E-06
Y46H3D.2	7	2	9	4	6.78E-06	9.60E-06	2.75E-06	7.15E-06
Y46H3D.3	7	8	5	4	7.56E-06	3.89E-06	7.38E-06	2.25E-06
Y46H3D.4	24	19	17	16	2.80E-06	2.65E-06	1.82E-06	2.25E-06
Y46H3D.5a.1	22	46	21	20	2.80E-06	2.65E-06	1.82E-06	2.25E-06
Y46H3D.5a.2	17	31	10	15	2.80E-06	2.65E-06	1.82E-06	2.25E-06
Y46H3D.5b	16	33	12	14	2.80E-06	3.33E-06	1.82E-06	2.25E-06
Y46H3D.6	7	18	4	7	2.80E-06	2.65E-06	1.82E-06	2.25E-06
Y46H3D.7	9	22	15	6	2.80E-06	2.65E-06	1.82E-06	2.25E-06
Y46H3D.8	3	3	0	1	2.80E-06	2.65E-06	1.82E-06	2.25E-06
Y47A7.1	325	288	361	588	2.80E-06	2.65E-06	1.82E-06	2.25E-06
Y47A7.2	22	33	25	41	2.80E-06	2.65E-06	1.82E-06	2.25E-06
Y47C4A.1	33	46	35	62	1.06E-05	8.83E-06	7.63E-06	1.54E-05
Y47D3A.1	18	17	13	26	3.19E-06	4.52E-06	2.35E-06	4.77E-06
Y47D3A.10	23	28	25	17	5.15E-06	6.80E-06	3.55E-06	7.78E-06
Y47D3A.11	15	25	18	8	2.80E-06	2.65E-06	1.82E-06	2.25E-06
Y47D3A.12	5	5	7	6	2.83E-06	3.25E-06	2.00E-06	2.25E-06
Y47D3A.13	91	195	53	31	2.80E-06	2.65E-06	1.82E-06	2.25E-06
Y47D3A.14	39	41	92	79	2.80E-06	2.65E-06	1.82E-06	2.25E-06
Y47D3A.15	50	46	163	173	2.48E-05	5.02E-05	9.40E-06	6.79E-06
Y47D3A.16	313	446	794	1246	5.04E-06	5.00E-06	7.71E-06	8.19E-06
Y47D3A.17a	203	232	193	315	6.78E-06	5.90E-06	1.44E-05	1.89E-05
Y47D3A.2	12	20	30	15	1.88E-05	2.53E-05	3.10E-05	6.01E-05
Y47D3A.20	90	76	196	41	9.10E-06	9.84E-06	5.63E-06	1.14E-05
Y47D3A.21	210	174	548	276	2.80E-06	2.65E-06	2.41E-06	2.25E-06
Y47D3A.22	69	126	108	105	2.76E-05	2.20E-05	3.90E-05	1.01E-05
Y47D3A.23a	162	140	378	353	4.06E-05	3.18E-05	6.90E-05	4.29E-05
Y47D3A.23b	87	78	192	241	3.08E-06	5.29E-06	3.13E-06	3.76E-06
Y47D3A.25.1	132	184	159	185	7.70E-06	6.27E-06	1.17E-05	1.35E-05
Y47D3A.25.2	121	169	135	160	7.08E-06	6.00E-06	1.02E-05	1.58E-05
Y47D3A.27	145	145	294	274	2.26E-05	2.98E-05	1.77E-05	2.54E-05
Y47D3A.28	148	210	247	362	2.09E-05	2.75E-05	1.51E-05	2.22E-05
Y47D3A.29	867	830	701	1466	1.45E-05	1.37E-05	1.91E-05	2.20E-05
Y47D3A.30	26	31	17	13	7.64E-06	1.03E-05	8.31E-06	1.50E-05
Y47D3A.31	26	35	13	14	2.16E-05	1.95E-05	1.14E-05	2.93E-05

Y47D3A.32	16	24	29	33	2.80E-06	2.65E-06	1.82E-06	2.25E-06
Y47D3A.4	261	294	341	389	4.70E-06	5.98E-06	1.82E-06	2.25E-06
Y47D3A.5	21	22	52	29	6.02E-06	8.54E-06	7.12E-06	9.99E-06
Y47D3A.6a	290	208	363	581	1.37E-05	1.45E-05	1.16E-05	1.64E-05
Y47D3A.6b	123	89	126	172	2.80E-06	2.65E-06	3.33E-06	2.29E-06
Y47D3B.1	36	45	41	20	9.74E-06	6.61E-06	7.94E-06	1.57E-05
Y47D3B.10.1	456	543	319	333	1.08E-05	7.38E-06	7.18E-06	1.21E-05
Y47D3B.10.2	473	569	329	337	2.80E-06	3.25E-06	2.04E-06	2.25E-06
Y47D3B.11	89	117	104	113	2.80E-05	3.15E-05	1.27E-05	1.64E-05
Y47D3B.2a	20	27	19	13	2.93E-05	3.33E-05	1.32E-05	1.68E-05
Y47D3B.2b	38	61	35	26	5.32E-06	6.61E-06	4.05E-06	5.42E-06
Y47D3B.3	16	19	34	14	4.40E-06	5.61E-06	2.71E-06	2.29E-06
Y47D3B.4	16	23	20	28	5.96E-06	9.05E-06	3.57E-06	3.28E-06
Y47D3B.5a	35	59	30	47	2.80E-06	2.65E-06	1.84E-06	2.25E-06
Y47D3B.5b	34	60	30	48	2.80E-06	2.65E-06	1.82E-06	2.25E-06
Y47D3B.6	146	139	31	51	2.80E-06	2.65E-06	1.82E-06	2.25E-06
Y47D3B.7	172	211	229	285	2.80E-06	2.65E-06	1.82E-06	2.25E-06
Y47D3B.9	139	158	193	228	1.53E-05	1.37E-05	2.11E-06	4.27E-06
Y47D7A.10	3	0	0	0	5.68E-06	6.59E-06	4.92E-06	7.56E-06
Y47D7A.11	8	10	13	5	6.22E-06	6.67E-06	5.61E-06	8.19E-06
Y47D7A.12	13	5	10	6	2.80E-06	2.65E-06	1.82E-06	2.25E-06
Y47D7A.14	262	218	321	449	2.80E-06	2.72E-06	2.42E-06	2.25E-06
Y47D7A.15	170	130	51	42	2.80E-06	2.65E-06	1.82E-06	2.25E-06
Y47D7A.16	27	46	34	32	1.62E-05	1.27E-05	1.29E-05	2.23E-05
Y47D7A.2	4	1	7	2	3.86E-05	2.79E-05	7.54E-06	7.67E-06
Y47D7A.3	10	14	9	11	2.80E-06	3.89E-06	1.97E-06	2.29E-06
Y47D7A.5	178	274	94	38	2.80E-06	2.65E-06	1.82E-06	2.25E-06
Y47D7A.6	17	22	49	10	2.80E-06	2.65E-06	1.82E-06	2.25E-06
Y47D7A.7	7	17	18	9	2.46E-05	3.58E-05	8.47E-06	4.23E-06
Y47D7A.8	28	35	37	39	2.80E-06	2.65E-06	2.66E-06	2.25E-06
Y47D7A.9	5	3	4	1	2.80E-06	4.92E-06	3.59E-06	2.25E-06
Y47D9A.1b	331	446	452	527	4.70E-06	5.55E-06	4.05E-06	5.26E-06
Y47D9A.2a	75	86	110	187	2.80E-06	2.65E-06	1.82E-06	2.25E-06
Y47D9A.2b	97	110	128	224	2.56E-05	3.26E-05	2.27E-05	3.27E-05
Y47D9A.3	9	6	7	5	6.38E-06	6.90E-06	6.09E-06	1.28E-05
Y47D9A.4	2	4	1	0	6.16E-06	6.59E-06	5.28E-06	1.14E-05
Y47D9A.5.1	16	16	12	13	2.80E-06	2.65E-06	1.82E-06	2.25E-06
Y47D9A.5.2	11	16	7	12	2.80E-06	2.65E-06	1.82E-06	2.25E-06
Y47G6A.1.1	216	229	247	333	2.80E-06	2.65E-06	1.82E-06	2.25E-06
Y47G6A.10	182	206	337	568	2.80E-06	2.65E-06	1.82E-06	2.25E-06
Y47G6A.11	861	895	1417	1959	1.44E-05	1.44E-05	1.07E-05	1.79E-05
Y47G6A.12	900	946	1883	2133	8.60E-06	9.18E-06	1.03E-05	2.15E-05
Y47G6A.13	34	34	19	12	2.64E-05	2.59E-05	2.82E-05	4.82E-05
Y47G6A.14	119	152	171	202	2.56E-05	2.54E-05	3.48E-05	4.87E-05
Y47G6A.17	26	45	18	23	2.80E-06	2.65E-06	1.82E-06	2.25E-06
Y47G6A.18.1	295	367	303	353	9.46E-06	1.14E-05	8.86E-06	1.29E-05
Y47G6A.18.2	164	173	142	182	2.80E-06	2.65E-06	1.82E-06	2.25E-06
Y47G6A.19a	198	332	142	218	2.30E-05	2.71E-05	1.54E-05	2.22E-05
Y47G6A.19b	197	336	143	221	1.87E-05	1.87E-05	1.06E-05	1.67E-05
Y47G6A.2.1	448	497	619	719	1.38E-05	2.19E-05	6.45E-06	1.22E-05
Y47G6A.2.2	415	444	563	682	1.28E-05	2.07E-05	6.07E-06	1.16E-05
Y47G6A.20a	260	264	467	491	2.99E-05	3.14E-05	2.69E-05	3.86E-05
Y47G6A.20b	384	448	639	723	3.23E-05	3.26E-05	2.85E-05	4.26E-05
Y47G6A.20c	241	248	449	469	2.74E-05	2.63E-05	3.21E-05	4.16E-05
Y47G6A.21.1	142	209	194	174	1.88E-05	2.07E-05	2.03E-05	2.84E-05
Y47G6A.21.2	127	202	164	164	2.57E-05	2.50E-05	3.11E-05	4.01E-05
Y47G6A.22	170	264	252	221	1.94E-05	2.70E-05	1.72E-05	1.91E-05
Y47G6A.23	518	482	422	821	1.83E-05	2.74E-05	1.53E-05	1.89E-05
Y47G6A.24	243	221	264	257	2.27E-05	3.34E-05	2.19E-05	2.37E-05

Y47G6A.25	194	192	223	329	1.11E-05	9.79E-06	5.90E-06	1.41E-05
Y47G6A.26	18	31	37	10	4.37E-05	3.75E-05	3.09E-05	3.71E-05
Y47G6A.27	107	101	146	221	1.58E-05	1.48E-05	1.18E-05	2.16E-05
Y47G6A.28	201	234	431	534	3.42E-06	5.55E-06	4.56E-06	2.25E-06
Y47G6A.29	400	335	375	718	9.46E-06	8.44E-06	8.40E-06	1.57E-05
Y47G6A.3	21	37	16	3	1.24E-05	1.36E-05	1.73E-05	2.65E-05
Y47G6A.30	1	0	2	1	6.08E-06	4.79E-06	3.70E-06	8.75E-06
Y47G6A.31	9	12	11	10	4.31E-06	7.17E-06	2.13E-06	2.25E-06
Y47G6A.32	4	5	8	3	2.80E-06	2.65E-06	1.82E-06	2.25E-06
Y47G6A.4	253	250	447	455	2.80E-06	2.65E-06	1.82E-06	2.25E-06
Y47G6A.5a	59	71	94	82	2.80E-06	2.65E-06	1.82E-06	2.25E-06
Y47G6A.5b	39	46	61	62	1.38E-05	1.29E-05	1.58E-05	1.99E-05
Y47G6A.6	273	307	411	534	2.94E-06	3.36E-06	3.06E-06	3.28E-06
Y47G6A.7a.1	82	129	88	133	2.80E-06	2.72E-06	2.50E-06	3.13E-06
Y47G6A.7a.2	77	119	75	122	1.23E-05	1.31E-05	1.21E-05	1.94E-05
Y47G6A.7b.1	81	126	86	129	6.22E-06	9.23E-06	4.34E-06	8.10E-06
Y47G6A.7b.2	73	118	73	117	6.08E-06	8.86E-06	3.84E-06	7.71E-06
Y47G6A.8	551	536	771	947	5.91E-06	8.65E-06	4.08E-06	7.53E-06
Y47G6A.9	168	150	136	143	5.85E-06	8.97E-06	3.81E-06	7.56E-06
Y47G7B.1	2	3	3	3	4.71E-05	4.33E-05	4.29E-05	6.50E-05
Y47G7B.2	12	16	23	18	2.62E-05	2.21E-05	1.38E-05	1.79E-05
Y47G7B.3	4	5	1	3	2.80E-06	2.65E-06	1.82E-06	2.25E-06
Y47H10A.1	17	20	15	15	2.80E-06	2.65E-06	1.82E-06	2.25E-06
Y47H10A.2	13	6	22	14	2.80E-06	2.65E-06	1.82E-06	2.25E-06
Y47H10A.3	0	6	2	4	2.80E-06	2.65E-06	1.82E-06	2.25E-06
Y47H10A.4	16	27	20	19	2.80E-06	2.65E-06	1.82E-06	2.25E-06
Y47H10A.5	19	36	35	15	2.80E-06	2.65E-06	1.82E-06	2.25E-06
Y47H9A.1	33	32	24	21	2.80E-06	2.86E-06	1.82E-06	2.25E-06
Y47H9A.2	0	3	0	2	2.80E-06	3.78E-06	2.53E-06	2.25E-06
Y47H9B.2	11	12	14	7	2.94E-06	2.70E-06	1.82E-06	2.25E-06
Y47H9C.1	44	51	56	36	2.80E-06	2.65E-06	1.82E-06	2.25E-06
Y47H9C.10	13	16	8	14	2.80E-06	2.65E-06	1.82E-06	2.25E-06
Y47H9C.11	19	32	31	30	4.34E-06	4.76E-06	3.61E-06	2.86E-06
Y47H9C.12	4	2	3	1	2.80E-06	2.65E-06	1.82E-06	2.25E-06
Y47H9C.14	109	93	83	20	2.80E-06	3.02E-06	2.02E-06	2.41E-06
Y47H9C.15	3	2	1	3	2.80E-06	2.65E-06	1.82E-06	2.25E-06
Y47H9C.2	27	38	26	22	4.63E-05	3.73E-05	2.29E-05	6.81E-06
Y47H9C.4a	411	450	539	829	2.80E-06	2.65E-06	1.82E-06	2.25E-06
Y47H9C.4b	404	446	542	820	2.80E-06	3.52E-06	1.82E-06	2.25E-06
Y47H9C.4c	359	371	479	734	1.23E-05	1.28E-05	1.05E-05	2.00E-05
Y47H9C.5b	46	78	66	73	1.26E-05	1.31E-05	1.10E-05	2.05E-05
Y47H9C.6	2	4	3	3	1.28E-05	1.25E-05	1.11E-05	2.10E-05
Y47H9C.7	302	256	360	510	5.38E-06	8.62E-06	5.03E-06	6.86E-06
Y47H9C.8	126	126	181	230	2.80E-06	2.65E-06	1.82E-06	2.25E-06
Y47H9C.9	10	19	25	12	2.96E-05	2.37E-05	2.29E-05	4.01E-05
Y48A5A.1	216	152	218	315	1.15E-05	1.08E-05	1.07E-05	1.68E-05
Y48A5A.2	17	35	44	41	2.80E-06	2.65E-06	1.82E-06	2.25E-06
Y48A5A.3	41	50	160	51	1.67E-05	1.11E-05	1.10E-05	1.96E-05
Y48A6A.1	12	27	18	15	3.00E-06	5.85E-06	5.07E-06	5.82E-06
Y48A6B.1	11	15	7	3	1.28E-05	1.47E-05	3.24E-05	1.28E-05
Y48A6B.10	210	127	207	325	2.80E-06	3.23E-06	1.82E-06	2.25E-06
Y48A6B.11a	506	470	646	1024	2.80E-06	2.65E-06	1.82E-06	2.25E-06
Y48A6B.11b	478	452	620	998	1.75E-05	1.00E-05	1.12E-05	2.18E-05
Y48A6B.13.1	1117	704	1289	1697	1.91E-05	1.67E-05	1.58E-05	3.10E-05
Y48A6B.13.2	1103	698	1269	1664	1.88E-05	1.68E-05	1.59E-05	3.15E-05
Y48A6B.3.1	440	435	663	608	4.75E-05	2.83E-05	3.57E-05	5.80E-05
Y48A6B.3.2	325	328	511	471	4.76E-05	2.85E-05	3.56E-05	5.77E-05
Y48A6B.4	0	5	3	0	8.00E-05	7.47E-05	7.84E-05	8.88E-05
Y48A6B.5	111	131	144	151	6.80E-05	6.49E-05	6.96E-05	7.92E-05

Y48A6B.6a	8	18	6	11	2.80E-06	2.65E-06	1.82E-06	2.25E-06
Y48A6B.6b	9	18	7	12	1.58E-05	1.76E-05	1.33E-05	1.72E-05
Y48A6B.7	78	150	126	150	2.80E-06	2.65E-06	1.82E-06	2.25E-06
Y48A6B.8	15	21	24	13	2.80E-06	2.65E-06	1.82E-06	2.25E-06
Y48A6C.1	136	124	223	423	1.50E-05	2.73E-05	1.58E-05	2.32E-05
Y48A6C.2	30	17	48	24	2.80E-06	2.65E-06	1.82E-06	2.25E-06
Y48A6C.3	124	102	196	355	1.10E-05	9.47E-06	1.17E-05	2.75E-05
Y48A6C.4	107	110	200	325	7.00E-06	3.76E-06	7.29E-06	4.50E-06
Y48A6C.5a	33	33	53	68	1.39E-05	1.08E-05	1.43E-05	3.20E-05
Y48A6C.5b	33	33	52	70	1.18E-05	1.15E-05	1.44E-05	2.89E-05
Y48B6A.10	25	32	60	34	2.80E-06	2.65E-06	1.82E-06	2.77E-06
Y48B6A.11	267	240	306	501	2.80E-06	2.65E-06	2.79E-06	4.63E-06
Y48B6A.12.1	727	792	925	1120	2.80E-06	2.65E-06	3.39E-06	2.36E-06
Y48B6A.12.2	733	795	934	1122	1.11E-05	9.47E-06	8.31E-06	1.68E-05
Y48B6A.12.3	657	705	811	1035	3.82E-05	3.93E-05	3.16E-05	4.73E-05
Y48B6A.13a	49	67	72	93	3.68E-05	3.77E-05	3.05E-05	4.52E-05
Y48B6A.13b.	65	83	96	105	3.84E-05	3.90E-05	3.09E-05	4.86E-05
Y48B6A.13b.[illegible]	63	82	81	99	5.24E-06	6.77E-06	5.01E-06	7.98E-06
Y48B6A.14.1	3457	2661	4058	5555	6.33E-06	7.62E-06	6.07E-06	8.21E-06
Y48B6A.14.2	1249	979	1264	1857	6.13E-06	7.54E-06	5.14E-06	7.74E-06
Y48B6A.2.1	4732	4411	12475	2673	5.60E-04	4.07E-04	4.28E-04	7.23E-04
Y48B6A.2.2	3688	3487	9069	2225	2.36E-04	1.75E-04	1.55E-04	2.82E-04
Y48B6A.3	286	333	253	521	1.29E-03	1.14E-03	2.22E-03	5.86E-04
Y48B6A.4	9	5	6	6	1.19E-03	1.06E-03	1.90E-03	5.75E-04
Y48B6A.5	31	68	34	24	1.15E-05	1.26E-05	6.58E-06	1.67E-05
Y48B6A.6a.1	136	183	102	182	2.80E-06	2.65E-06	1.82E-06	2.25E-06
Y48B6A.6a.2	114	160	90	158	2.80E-06	5.63E-06	1.95E-06	2.25E-06
Y48B6A.6b	93	150	75	137	6.24E-06	7.96E-06	3.06E-06	6.72E-06
Y48B6A.7	26	16	19	7	6.16E-06	8.17E-06	3.17E-06	6.86E-06
Y48B6A.8	57	39	35	36	5.35E-06	8.15E-06	2.81E-06	6.32E-06
Y48B6A.9	4	6	6	1	2.80E-06	2.65E-06	1.82E-06	2.25E-06
Y48C3A.1	1	4	11	6	2.94E-06	2.65E-06	1.82E-06	2.25E-06
Y48C3A.10	132	137	296	140	2.80E-06	2.65E-06	1.82E-06	2.25E-06
Y48C3A.11	7	9	2	6	2.80E-06	2.65E-06	2.30E-06	2.25E-06
Y48C3A.12	224	201	528	679	2.56E-05	2.51E-05	3.74E-05	2.18E-05
Y48C3A.14	230	267	357	431	2.80E-06	2.65E-06	1.82E-06	2.25E-06
Y48C3A.16	129	202	233	130	1.19E-05	1.01E-05	1.83E-05	2.91E-05
Y48C3A.20	131	78	174	222	9.38E-06	1.03E-05	9.48E-06	1.41E-05
Y48C3A.3	61	82	77	87	3.16E-05	4.68E-05	3.72E-05	2.56E-05
Y48C3A.4	118	195	114	127	1.00E-05	5.63E-06	8.66E-06	1.37E-05
Y48C3A.5a	17	16	13	22	7.08E-06	8.99E-06	5.81E-06	8.10E-06
Y48C3A.5b	15	13	11	20	7.56E-06	1.18E-05	4.76E-06	6.52E-06
Y48C3A.5c	12	12	10	20	2.80E-06	2.65E-06	1.82E-06	2.25E-06
Y48C3A.7	308	271	343	487	2.80E-06	2.65E-06	1.82E-06	2.25E-06
Y48C3A.8	170	96	343	205	2.80E-06	2.65E-06	1.82E-06	2.25E-06
Y48C3A.9	119	84	227	274	1.35E-05	1.12E-05	9.77E-06	1.71E-05
Y48D7A.1	3	1	3	3	2.17E-05	1.16E-05	2.85E-05	2.11E-05
Y48E1A.1a	435	373	333	611	1.39E-05	9.23E-06	1.72E-05	2.56E-05
Y48E1A.1b	65	58	60	88	2.80E-06	2.65E-06	1.82E-06	2.25E-06
Y48E1B.1	43	34	93	113	9.35E-06	7.56E-06	4.65E-06	1.05E-05
Y48E1B.10	115	87	189	64	9.21E-06	7.75E-06	5.52E-06	1.00E-05
Y48E1B.11	9	11	56	12	3.22E-06	2.65E-06	4.54E-06	6.81E-06
Y48E1B.13a	16	12	19	10	1.75E-05	1.25E-05	1.87E-05	7.83E-06
Y48E1B.13b	4	4	5	4	2.80E-06	2.65E-06	7.12E-06	2.25E-06
Y48E1B.13c	2	3	1	2	2.80E-06	2.65E-06	1.82E-06	2.25E-06
Y48E1B.14a.[illegible]	313	298	451	562	2.80E-06	2.65E-06	1.82E-06	2.25E-06
Y48E1B.14a.[illegible]	281	270	407	518	2.80E-06	2.65E-06	1.82E-06	2.25E-06
Y48E1B.14b	76	78	114	127	1.13E-05	1.01E-05	1.06E-05	1.63E-05
Y48E1B.15	0	4	1	2	1.05E-05	9.52E-06	9.88E-06	1.55E-05

Y48E1B.16	10	17	13	22	9.44E-06	9.15E-06	9.20E-06	1.27E-05
Y48E1B.2a	169	158	202	299	2.80E-06	2.65E-06	1.82E-06	2.25E-06
Y48E1B.2b	94	72	103	158	2.80E-06	2.65E-06	1.82E-06	2.25E-06
Y48E1B.3	196	170	220	306	6.89E-06	6.08E-06	5.36E-06	9.81E-06
Y48E1B.4	104	74	122	169	7.28E-06	5.26E-06	5.19E-06	9.81E-06
Y48E1B.5	100	116	183	179	1.74E-05	1.43E-05	1.27E-05	2.19E-05
Y48E1B.7	104	104	171	254	5.18E-06	3.49E-06	3.95E-06	6.75E-06
Y48E1B.8	163	112	218	196	7.03E-06	7.70E-06	8.38E-06	1.01E-05
Y48E1B.9	31	55	24	49	5.15E-06	4.87E-06	5.52E-06	1.01E-05
Y48E1C.1a	106	107	101	156	1.58E-05	1.03E-05	1.38E-05	1.53E-05
Y48E1C.1b	126	130	133	202	5.04E-06	8.46E-06	2.55E-06	6.41E-06
Y48E1C.1c	32	40	33	58	8.82E-06	8.41E-06	5.48E-06	1.04E-05
Y48E1C.2	138	159	198	236	5.91E-06	5.77E-06	4.06E-06	7.62E-06
Y48E1C.3	51	67	142	223	6.08E-06	7.17E-06	4.06E-06	8.84E-06
Y48E1C.4a	95	122	110	111	1.34E-05	1.46E-05	1.25E-05	1.84E-05
Y48E1C.4b	92	121	112	109	6.22E-06	7.70E-06	1.12E-05	2.18E-05
Y48G10A.2	190	188	244	301	1.39E-05	1.68E-05	1.05E-05	1.30E-05
Y48G10A.3	37	62	251	250	1.23E-05	1.53E-05	9.78E-06	1.17E-05
Y48G10A.4	307	255	427	574	1.72E-05	1.61E-05	1.44E-05	2.20E-05
Y48G10A.6	124	133	150	118	3.89E-06	6.16E-06	1.72E-05	2.12E-05
Y48G1A.1	95	87	102	179	1.47E-05	1.15E-05	1.33E-05	2.21E-05
Y48G1A.2	190	174	357	354	5.03E-05	5.10E-05	3.96E-05	3.85E-05
Y48G1A.3	119	136	199	248	3.92E-06	3.41E-06	2.75E-06	5.96E-06
Y48G1A.4	255	233	262	415	3.14E-05	2.72E-05	3.84E-05	4.70E-05
Y48G1A.5	1197	962	2156	2756	1.06E-05	1.14E-05	1.15E-05	1.77E-05
Y48G1A.6	152	88	248	350	1.17E-05	1.01E-05	7.82E-06	1.53E-05
Y48G1BL.1	79	101	135	107	4.34E-05	3.29E-05	5.08E-05	8.02E-05
Y48G1BL.2	132	143	150	268	8.26E-06	4.52E-06	8.76E-06	1.53E-05
Y48G1BL.3	1750	1412	2972	3225	9.24E-06	1.12E-05	1.03E-05	1.01E-05
Y48G1BL.4	26	27	19	22	6.16E-06	6.32E-06	4.57E-06	1.01E-05
Y48G1BL.5	33	37	201	70	1.34E-04	1.02E-04	1.48E-04	1.98E-04
Y48G1BL.6	46	23	67	37	1.21E-05	1.19E-05	5.78E-06	8.25E-06
Y48G1BL.7	6	8	18	8	7.56E-06	8.01E-06	3.00E-05	1.29E-05
Y48G1BM.1	55	81	77	86	1.87E-05	8.81E-06	1.77E-05	1.21E-05
Y48G1BM.2	4	10	20	9	2.80E-06	2.65E-06	1.82E-06	2.25E-06
Y48G1BM.5	62	69	75	102	4.82E-06	6.69E-06	4.39E-06	6.05E-06
Y48G1BM.6	14	35	16	63	2.80E-06	2.65E-06	1.82E-06	2.25E-06
Y48G1BM.7	6	8	98	59	2.80E-06	2.65E-06	1.84E-06	3.08E-06
Y48G1BM.8	113	79	151	109	2.80E-06	2.65E-06	1.82E-06	3.06E-06
Y48G1BM.9	57	63	190	98	2.80E-06	2.65E-06	2.13E-05	1.58E-05
Y48G1BR.1	43	62	33	38	1.31E-05	8.68E-06	1.14E-05	1.02E-05
Y48G1C.1	132	117	197	222	7.59E-06	7.94E-06	1.65E-05	1.05E-05
Y48G1C.10	77	87	120	155	6.24E-06	8.49E-06	3.12E-06	4.43E-06
Y48G1C.11	132	92	197	295	9.97E-06	8.33E-06	9.68E-06	1.34E-05
Y48G1C.12	62	79	229	190	8.12E-06	8.68E-06	8.24E-06	1.31E-05
Y48G1C.2.1	545	317	760	805	1.19E-05	7.83E-06	1.15E-05	2.13E-05
Y48G1C.2.2	468	298	650	742	1.07E-05	1.28E-05	2.56E-05	2.62E-05
Y48G1C.2.3	331	205	463	623	2.44E-05	1.34E-05	2.21E-05	2.89E-05
Y48G1C.4	157	165	247	290	2.36E-05	1.42E-05	2.13E-05	3.00E-05
Y48G1C.5	34	53	39	30	2.18E-05	1.27E-05	1.98E-05	3.29E-05
Y48G1C.6	6	18	16	12	1.31E-05	1.30E-05	1.34E-05	1.94E-05
Y48G1C.7	539	427	972	1390	2.80E-06	2.65E-06	1.82E-06	2.25E-06
Y48G1C.8	493	388	770	1175	2.80E-06	2.80E-06	1.82E-06	2.25E-06
Y48G1C.9.1	78	75	250	92	2.72E-05	2.03E-05	3.19E-05	5.62E-05
Y48G1C.9.2	67	76	203	79	2.22E-05	1.65E-05	2.26E-05	4.25E-05
Y48G8AL.1	396	430	449	757	2.52E-05	2.29E-05	5.25E-05	2.39E-05
Y48G8AL.10	195	207	339	459	1.37E-05	1.47E-05	2.71E-05	1.30E-05
Y48G8AL.11	167	172	252	355	1.37E-05	1.41E-05	1.01E-05	2.11E-05
Y48G8AL.12	255	132	347	36	8.79E-06	8.81E-06	9.93E-06	1.66E-05

Y48G8AL.13.:	140	352	182	203	8.57E-06	8.33E-06	8.40E-06	1.46E-05
Y48G8AL.13.:	133	332	162	200	8.79E-05	4.30E-05	7.78E-05	9.96E-06
Y48G8AL.13.:	93	249	121	162	1.44E-05	3.43E-05	1.22E-05	1.68E-05
Y48G8AL.14	137	207	542	423	1.48E-05	3.48E-05	1.17E-05	1.78E-05
Y48G8AL.15	24	27	36	36	1.22E-05	3.09E-05	1.04E-05	1.71E-05
Y48G8AL.5	448	294	634	951	2.63E-05	3.76E-05	6.78E-05	6.53E-05
Y48G8AL.6	553	438	770	1251	5.57E-06	5.92E-06	5.43E-06	6.70E-06
Y48G8AL.7	32	53	111	63	2.56E-05	1.59E-05	2.36E-05	4.37E-05
Y48G8AL.8a.:	9362	7465	11411	8917	1.77E-05	1.33E-05	1.61E-05	3.22E-05
Y48G8AL.8a.:	7795	6213	9611	8211	4.96E-06	7.75E-06	1.12E-05	7.85E-06
Y48G8AL.8b	7271	5704	8566	6607	1.69E-03	1.27E-03	1.34E-03	1.29E-03
Y48G8AL.9	84	74	106	177	1.41E-03	1.06E-03	1.13E-03	1.19E-03
Y48G8AR.1	29	67	54	56	1.50E-03	1.11E-03	1.15E-03	1.09E-03
Y48G8AR.2	14	17	11	10	9.18E-06	7.64E-06	7.54E-06	1.55E-05
Y48G8AR.3	0	0	0	1	2.80E-06	4.18E-06	2.33E-06	2.97E-06
Y48G9A.1	1236	1030	943	1778	2.80E-06	2.65E-06	1.82E-06	2.25E-06
Y48G9A.10	12	22	8	13	2.80E-06	2.65E-06	1.82E-06	2.25E-06
Y48G9A.11	41	43	52	93	2.57E-05	2.03E-05	1.28E-05	2.97E-05
Y48G9A.12	4	3	5	1	2.80E-06	2.65E-06	1.82E-06	2.25E-06
Y48G9A.2	130	110	135	219	4.28E-06	4.26E-06	3.53E-06	7.80E-06
Y48G9A.3	186	201	163	359	2.80E-06	2.65E-06	1.82E-06	2.25E-06
Y48G9A.4	225	196	199	378	1.62E-05	1.29E-05	1.09E-05	2.19E-05
Y48G9A.6	2	4	2	1	1.08E-05	1.10E-05	6.12E-06	1.67E-05
Y48G9A.7	3	3	5	6	6.55E-06	5.40E-06	3.77E-06	8.84E-06
Y48G9A.9a	128	175	143	189	2.80E-06	2.65E-06	1.82E-06	2.25E-06
Y48G9A.9b	67	81	41	88	2.80E-06	2.65E-06	1.82E-06	2.34E-06
Y49A10A.1	7	7	9	4	1.35E-05	1.75E-05	9.82E-06	1.60E-05
Y49A3A.1	253	153	341	478	6.92E-06	7.88E-06	2.75E-06	7.29E-06
Y49A3A.2.1	4650	5638	5500	9033	2.80E-06	2.65E-06	1.82E-06	2.25E-06
Y49A3A.2.2	4721	5583	5524	8973	2.16E-05	1.24E-05	1.90E-05	3.28E-05
Y49A3A.2.3	3603	4346	4076	6859	2.23E-04	2.56E-04	1.72E-04	3.48E-04
Y49A3A.3	139	136	148	323	1.89E-04	2.11E-04	1.44E-04	2.88E-04
Y49A3A.4	5	7	6	6	2.21E-04	2.52E-04	1.63E-04	3.38E-04
Y49A3A.5.1	604	770	758	502	1.27E-05	1.17E-05	8.82E-06	2.37E-05
Y49A3A.5.2	601	769	758	502	2.80E-06	2.65E-06	1.82E-06	2.25E-06
Y49C4A.1	2	1	2	2	9.85E-05	1.19E-04	8.04E-05	6.57E-05
Y49C4A.2	8	5	8	7	1.09E-04	1.31E-04	8.91E-05	7.28E-05
Y49C4A.3	6	4	4	1	2.80E-06	2.65E-06	1.82E-06	2.25E-06
Y49C4A.4	2	4	4	4	2.80E-06	2.65E-06	1.82E-06	2.25E-06
Y49C4A.5	4	3	2	0	2.80E-06	2.65E-06	1.82E-06	2.25E-06
Y49C4A.6	2	2	6	2	2.80E-06	2.65E-06	1.82E-06	2.25E-06
Y49C4A.7	3	3	6	2	2.80E-06	2.65E-06	1.82E-06	2.25E-06
Y49C4A.8a	78	104	54	114	2.80E-06	2.65E-06	1.82E-06	2.25E-06
Y49C4A.8b.1	96	120	72	128	2.80E-06	2.65E-06	1.82E-06	2.25E-06
Y49C4A.8b.2	76	111	58	110	5.10E-06	6.43E-06	2.30E-06	5.98E-06
Y49C4A.8b.3	67	97	48	103	4.98E-06	5.90E-06	2.44E-06	5.35E-06
Y49C4A.9	1	11	5	5	4.70E-06	6.48E-06	2.33E-06	5.47E-06
Y49E10.1	1355	1046	1514	1728	5.21E-06	7.12E-06	2.42E-06	6.43E-06
Y49E10.10.1	50	54	43	29	2.80E-06	2.65E-06	1.82E-06	2.25E-06
Y49E10.10.2	44	51	43	28	1.07E-04	7.82E-05	7.79E-05	1.10E-04
Y49E10.11a	696	843	614	1095	4.20E-06	4.29E-06	2.35E-06	2.25E-06
Y49E10.11b	623	670	496	905	3.75E-06	4.10E-06	2.39E-06	2.25E-06
Y49E10.15	1334	936	1262	417	2.02E-05	2.31E-05	1.16E-05	2.55E-05
Y49E10.16	243	283	409	253	2.08E-05	2.11E-05	1.08E-05	2.42E-05
Y49E10.17	13	18	7	9	5.47E-04	3.63E-04	3.37E-04	1.37E-04
Y49E10.18	178	149	135	148	2.59E-05	2.85E-05	2.83E-05	2.16E-05
Y49E10.19	1473	1422	2318	3232	2.80E-06	2.65E-06	1.82E-06	2.25E-06
Y49E10.2	186	204	376	161	1.88E-05	1.49E-05	9.27E-06	1.25E-05
Y49E10.20.1	309	460	308	539	4.33E-05	3.95E-05	4.44E-05	7.64E-05

Y49E10.20.2	269	421	287	508	4.07E-05	4.22E-05	5.35E-05	2.83E-05
Y49E10.21	48	76	134	162	2.00E-05	2.81E-05	1.30E-05	2.80E-05
Y49E10.22	17	24	77	48	1.87E-05	2.77E-05	1.30E-05	2.84E-05
Y49E10.23a	65	76	40	64	4.62E-06	6.90E-06	8.38E-06	1.25E-05
Y49E10.23b	64	76	40	63	2.80E-06	3.25E-06	7.20E-06	5.53E-06
Y49E10.24	5	4	11	3	3.08E-06	3.39E-06	1.82E-06	2.43E-06
Y49E10.25	2	3	1	5	3.00E-06	3.36E-06	1.82E-06	2.36E-06
Y49E10.26	8	6	4	15	2.80E-06	2.65E-06	1.82E-06	2.25E-06
Y49E10.27	2	1	1	1	2.80E-06	2.65E-06	1.82E-06	2.25E-06
Y49E10.3a	322	249	411	470	2.80E-06	2.65E-06	1.82E-06	2.70E-06
Y49E10.3b	309	242	437	461	2.80E-06	2.65E-06	1.82E-06	2.25E-06
Y49E10.4	167	193	238	311	2.97E-05	2.17E-05	2.47E-05	3.48E-05
Y49E10.5	7	10	3	9	4.16E-05	3.07E-05	3.82E-05	4.98E-05
Y49E10.6.1	733	823	1415	895	1.40E-05	1.53E-05	1.30E-05	2.09E-05
Y49E10.6.2	654	737	1147	812	2.80E-06	2.65E-06	1.82E-06	2.25E-06
Y49E10.7	2	6	5	4	1.30E-04	1.38E-04	1.63E-04	1.27E-04
Y49E10.8	1	4	1	1	1.75E-04	1.87E-04	2.00E-04	1.75E-04
Y49E10.9	6	14	9	5	2.80E-06	2.65E-06	1.82E-06	2.25E-06
Y49F6A.1	12	16	22	10	2.80E-06	2.65E-06	1.82E-06	2.25E-06
Y49F6A.2	7	3	2	3	2.80E-06	2.65E-06	1.82E-06	2.25E-06
Y49F6A.3	7	3	2	3	2.80E-06	2.65E-06	1.82E-06	2.25E-06
Y49F6A.5	5	11	33	19	2.80E-06	2.65E-06	1.82E-06	2.25E-06
Y49F6A.6	8	3	3	9	2.80E-06	2.65E-06	1.82E-06	2.25E-06
Y49F6B.1	54	45	160	165	2.80E-06	2.65E-06	1.93E-06	2.25E-06
Y49F6B.10	844	1179	359	329	2.80E-06	2.65E-06	1.82E-06	2.25E-06
Y49F6B.11	1	10	6	3	5.18E-06	4.07E-06	1.00E-05	1.27E-05
Y49F6B.12	1	19	0	9	7.99E-05	1.05E-04	2.21E-05	2.50E-05
Y49F6B.13	6	2	2	3	2.80E-06	2.65E-06	1.82E-06	2.25E-06
Y49F6B.14	2	3	2	3	2.80E-06	5.87E-06	1.82E-06	2.36E-06
Y49F6B.2	113	119	141	124	2.80E-06	2.65E-06	1.82E-06	2.25E-06
Y49F6B.3	3	6	14	6	2.80E-06	2.65E-06	1.82E-06	2.25E-06
Y49F6B.4	291	291	283	409	1.55E-05	1.54E-05	1.26E-05	1.37E-05
Y49F6B.5	1	3	4	2	2.80E-06	2.65E-06	1.82E-06	2.25E-06
Y49F6B.6	1	1	0	3	1.84E-05	1.74E-05	1.16E-05	2.08E-05
Y49F6B.7	10	6	6	7	2.80E-06	2.65E-06	1.82E-06	2.25E-06
Y49F6B.8	20	37	17	12	2.80E-06	2.65E-06	1.82E-06	2.25E-06
Y49F6B.9a	64	61	102	115	2.80E-06	2.65E-06	1.82E-06	2.25E-06
Y49F6B.9b.1	74	71	126	128	2.80E-06	4.34E-06	1.82E-06	2.25E-06
Y49F6B.9b.2	64	61	102	115	6.19E-06	5.58E-06	6.43E-06	8.95E-06
Y49F6C.1	1	10	5	0	6.52E-06	5.92E-06	7.23E-06	9.06E-06
Y49F6C.2	2	5	9	7	6.19E-06	5.58E-06	6.43E-06	8.95E-06
Y49F6C.3	15	14	15	38	2.80E-06	2.65E-06	1.82E-06	2.25E-06
Y49F6C.4	15	8	12	8	2.80E-06	2.65E-06	1.82E-06	2.25E-06
Y49F6C.5	3	5	3	4	2.80E-06	2.65E-06	1.82E-06	3.71E-06
Y49F6C.6	35	44	20	36	2.80E-06	2.65E-06	1.82E-06	2.25E-06
Y49F6C.7	3	1	3	3	2.80E-06	2.65E-06	1.82E-06	2.25E-06
Y49F6C.8	39	52	117	50	4.96E-06	5.87E-06	1.84E-06	4.09E-06
Y49G5A.1	12	58	12	25	2.80E-06	2.65E-06	1.82E-06	2.25E-06
Y49G5B.1	85	73	208	181	8.88E-06	1.12E-05	1.73E-05	9.15E-06
Y4C6A.1	6	16	5	8	2.80E-06	1.12E-05	1.82E-06	4.09E-06
Y4C6A.2a	21	35	32	15	1.15E-05	9.36E-06	1.84E-05	1.97E-05
Y4C6A.2b	10	22	12	8	2.80E-06	2.65E-06	1.82E-06	2.25E-06
Y4C6A.3	334	297	440	543	2.80E-06	2.65E-06	1.82E-06	2.25E-06
Y4C6A.4	12	10	1	4	2.80E-06	2.65E-06	1.82E-06	2.25E-06
Y4C6B.1	402	401	523	747	3.77E-05	3.16E-05	3.23E-05	4.92E-05
Y4C6B.2a	22	51	15	17	4.12E-06	3.25E-06	1.82E-06	2.25E-06
Y4C6B.2b	10	17	4	5	2.90E-05	2.74E-05	2.46E-05	4.33E-05
Y4C6B.3	14	22	13	8	2.80E-06	3.44E-06	1.82E-06	2.25E-06
Y4C6B.4a	24	24	23	16	2.80E-06	3.39E-06	1.82E-06	2.25E-06

Y4C6B.4b	6	4	5	3	2.80E-06	2.65E-06	1.82E-06	2.25E-06
Y4C6B.5	99	128	104	117	2.80E-06	2.65E-06	1.82E-06	2.25E-06
Y4C6B.6	396	383	452	457	2.80E-06	2.65E-06	1.82E-06	2.25E-06
Y50C1A.1	269	223	318	491	7.20E-06	8.81E-06	4.92E-06	6.84E-06
Y50C1A.2	12	10	6	3	2.63E-05	2.40E-05	1.96E-05	2.44E-05
Y50D4A.1a	286	296	401	579	1.52E-05	1.19E-05	1.17E-05	2.22E-05
Y50D4A.1b	280	287	391	575	2.80E-06	2.65E-06	1.82E-06	2.25E-06
Y50D4A.2.1	203	178	182	208	1.27E-05	1.24E-05	1.16E-05	2.06E-05
Y50D4A.3	10	12	4	2	1.27E-05	1.23E-05	1.15E-05	2.09E-05
Y50D4A.4	127	152	159	200	3.55E-05	2.94E-05	2.07E-05	2.92E-05
Y50D4A.5	31	39	38	28	2.80E-06	2.65E-06	1.82E-06	2.25E-06
Y50D4B.1	0	4	1	3	6.64E-06	7.51E-06	5.41E-06	8.41E-06
Y50D4B.2	4	7	2	3	7.92E-06	9.42E-06	6.32E-06	5.76E-06
Y50D4B.3	133	190	154	291	2.80E-06	2.94E-06	1.82E-06	2.25E-06
Y50D4B.4	198	203	200	355	2.80E-06	2.65E-06	1.82E-06	2.25E-06
Y50D4B.5	4	6	5	4	1.24E-05	1.67E-05	9.35E-06	2.18E-05
Y50D4B.6	16	35	22	18	1.72E-05	1.66E-05	1.13E-05	2.47E-05
Y50D4B.7	3	2	24	5	2.80E-06	2.65E-06	1.82E-06	2.25E-06
Y50D4C.1a	239	196	367	527	2.80E-06	2.72E-06	1.82E-06	2.25E-06
Y50D4C.1b	153	135	376	308	2.80E-06	2.65E-06	1.82E-06	2.25E-06
Y50D4C.2	135	145	113	184	1.69E-05	1.31E-05	1.69E-05	3.00E-05
Y50D4C.3	109	82	239	323	1.52E-05	1.26E-05	2.43E-05	2.45E-05
Y50D4C.4	147	164	415	609	8.37E-06	8.52E-06	4.57E-06	9.18E-06
Y50D4C.5	119	93	147	297	6.22E-06	4.42E-06	8.87E-06	1.48E-05
Y50D4C.6	46	55	65	74	6.75E-06	7.09E-06	1.24E-05	2.24E-05
Y50D7A.1	119	68	196	239	8.37E-06	6.19E-06	6.72E-06	1.68E-05
Y50D7A.10.1	80	115	140	114	9.30E-06	1.05E-05	8.53E-06	1.20E-05
Y50D7A.10.2	80	115	140	113	5.26E-06	2.86E-06	5.65E-06	8.50E-06
Y50D7A.11	46	37	91	110	1.76E-05	2.40E-05	2.01E-05	2.02E-05
Y50D7A.2	86	82	208	264	1.68E-05	2.28E-05	1.92E-05	1.91E-05
Y50D7A.3a	230	149	673	691	8.68E-06	6.59E-06	1.12E-05	1.67E-05
Y50D7A.3b	68	53	154	149	5.29E-06	4.76E-06	8.33E-06	1.30E-05
Y50D7A.4.1	618	602	953	1360	1.74E-05	1.06E-05	3.31E-05	4.19E-05
Y50D7A.4.2	606	593	929	1349	1.52E-05	1.12E-05	2.24E-05	2.67E-05
Y50D7A.5	9	9	1	7	2.70E-05	2.48E-05	2.71E-05	4.77E-05
Y50D7A.6	32	51	22	49	2.65E-05	2.45E-05	2.64E-05	4.73E-05
Y50D7A.7.1	451	811	469	694	2.80E-06	2.65E-06	1.82E-06	2.25E-06
Y50D7A.7.2	466	828	504	723	2.80E-06	2.65E-06	1.82E-06	2.25E-06
Y50D7A.8	260	204	253	402	2.71E-05	4.60E-05	1.83E-05	3.35E-05
Y50D7A.9	187	179	204	234	2.87E-05	4.82E-05	2.02E-05	3.58E-05
Y50E8A.1	15	19	17	3	1.63E-05	1.21E-05	1.03E-05	2.02E-05
Y50E8A.10	16	20	10	4	1.93E-05	1.75E-05	1.37E-05	1.94E-05
Y50E8A.11	6	3	10	2	6.44E-06	7.70E-06	4.76E-06	2.25E-06
Y50E8A.12	17	18	15	3	2.80E-06	3.23E-06	1.82E-06	2.25E-06
Y50E8A.14	18	13	15	8	2.80E-06	2.65E-06	1.82E-06	2.25E-06
Y50E8A.16	48	65	58	80	3.44E-06	3.44E-06	1.99E-06	2.25E-06
Y50E8A.17	0	3	1	0	2.80E-06	2.65E-06	1.82E-06	2.25E-06
Y50E8A.2	21	25	11	2	2.80E-06	2.80E-06	1.82E-06	2.95E-06
Y50E8A.3	35	35	71	18	2.80E-06	3.02E-06	1.82E-06	2.25E-06
Y50E8A.4a	782	710	992	1208	7.84E-06	8.81E-06	2.68E-06	2.25E-06
Y50E8A.4b	916	801	1075	1454	8.60E-06	8.12E-06	1.14E-05	3.55E-06
Y50E8A.4c	902	795	1061	1412	5.48E-05	4.70E-05	4.52E-05	6.80E-05
Y50E8A.5	7	15	5	4	5.90E-05	4.87E-05	4.51E-05	7.53E-05
Y50E8A.6	8	12	16	8	5.81E-05	4.84E-05	4.45E-05	7.31E-05
Y50E8A.7	5	8	8	7	2.80E-06	2.65E-06	1.82E-06	2.25E-06
Y50E8A.8	4	3	6	4	2.80E-06	2.65E-06	1.82E-06	2.25E-06
Y50E8A.9	10	21	7	4	2.80E-06	2.65E-06	1.82E-06	2.25E-06
Y50F7A.2	7	14	11	7	2.80E-06	2.65E-06	1.82E-06	2.25E-06
Y51A2A.1	9	6	10	13	2.80E-06	2.65E-06	1.82E-06	2.25E-06

Y51A2A.11	6	2	2	1	2.80E-06	4.52E-06	2.46E-06	2.25E-06
Y51A2A.2	2	3	2	2	2.80E-06	2.65E-06	1.82E-06	2.25E-06
Y51A2A.3	1	2	1	1	2.80E-06	2.65E-06	1.82E-06	2.25E-06
Y51A2A.4	0	2	1	1	2.80E-06	2.65E-06	1.82E-06	2.25E-06
Y51A2A.5	1	7	5	6	2.80E-06	2.65E-06	1.82E-06	2.25E-06
Y51A2A.6	0	2	3	4	2.80E-06	2.65E-06	1.82E-06	2.25E-06
Y51A2A.7	6	4	2	1	2.80E-06	2.65E-06	1.82E-06	2.25E-06
Y51A2A.8	2	4	2	1	2.80E-06	2.65E-06	1.82E-06	2.25E-06
Y51A2A.9	3	5	2	1	2.80E-06	2.65E-06	1.82E-06	2.25E-06
Y51A2B.1	3	6	1	1	2.80E-06	2.65E-06	1.82E-06	2.25E-06
Y51A2B.2	9	6	12	10	2.80E-06	2.65E-06	1.82E-06	2.25E-06
Y51A2B.3	3	2	2	3	2.80E-06	2.65E-06	1.82E-06	2.25E-06
Y51A2B.4	3	11	3	3	2.80E-06	2.65E-06	1.82E-06	2.25E-06
Y51A2B.5	4	12	6	2	2.80E-06	2.65E-06	1.82E-06	2.25E-06
Y51A2B.6	11	19	27	9	2.80E-06	2.65E-06	1.82E-06	2.25E-06
Y51A2B.7	3	4	5	1	2.80E-06	2.65E-06	1.82E-06	2.25E-06
Y51A2B.8	3	1	0	3	2.80E-06	2.65E-06	1.82E-06	2.25E-06
Y51A2D.1	17	19	28	15	2.80E-06	2.65E-06	1.82E-06	2.25E-06
Y51A2D.11	47	119	71	32	2.80E-06	2.65E-06	1.82E-06	2.25E-06
Y51A2D.12	10	11	14	6	2.80E-06	2.65E-06	1.82E-06	2.25E-06
Y51A2D.13a	114	203	134	242	9.18E-06	2.19E-05	9.02E-06	5.02E-06
Y51A2D.13b	59	144	60	167	2.80E-06	2.65E-06	1.82E-06	2.25E-06
Y51A2D.14	140	262	188	100	7.67E-06	1.29E-05	5.87E-06	1.31E-05
Y51A2D.15	74	82	38	93	4.00E-06	9.23E-06	2.64E-06	9.11E-06
Y51A2D.17	23	68	33	57	6.13E-05	1.08E-04	5.35E-05	3.52E-05
Y51A2D.18	140	152	134	186	2.80E-06	2.65E-06	1.82E-06	2.27E-06
Y51A2D.19a	49	54	32	54	2.80E-06	5.24E-06	1.82E-06	3.73E-06
Y51A2D.19b	48	55	33	53	9.74E-06	1.00E-05	6.07E-06	1.04E-05
Y51A2D.19c	49	60	36	54	2.80E-06	2.65E-06	1.82E-06	2.25E-06
Y51A2D.20	4	5	6	3	2.80E-06	2.65E-06	1.82E-06	2.25E-06
Y51A2D.21	19	41	23	12	2.80E-06	2.65E-06	1.82E-06	2.25E-06
Y51A2D.5	63	104	76	95	2.80E-06	2.65E-06	1.82E-06	2.25E-06
Y51A2D.7a	186	166	250	354	5.68E-06	1.16E-05	4.46E-06	2.88E-06
Y51A2D.7b	197	177	277	385	3.16E-06	4.95E-06	2.50E-06	3.85E-06
Y51A2D.8	5	13	5	6	6.94E-06	5.85E-06	6.09E-06	1.06E-05
Y51A2D.9	190	436	843	333	6.92E-06	5.87E-06	6.34E-06	1.09E-05
Y51B11A.1	20	18	2	2	2.80E-06	2.65E-06	1.82E-06	2.25E-06
Y51B9A.10	4	0	2	5	3.86E-05	8.37E-05	1.12E-04	5.44E-05
Y51B9A.2	6	3	3	0	2.80E-06	2.65E-06	1.82E-06	2.25E-06
Y51B9A.3	18	28	19	14	2.80E-06	2.65E-06	1.82E-06	2.25E-06
Y51B9A.4	21	24	41	31	2.80E-06	2.65E-06	1.82E-06	2.25E-06
Y51B9A.5	51	37	18	10	2.80E-06	3.15E-06	1.82E-06	2.25E-06
Y51B9A.6	9	11	7	10	2.80E-06	2.65E-06	2.93E-06	2.74E-06
Y51B9A.7	8	12	1	3	2.09E-05	1.43E-05	4.81E-06	3.31E-06
Y51B9A.8	2	2	1	3	2.80E-06	2.65E-06	1.82E-06	2.25E-06
Y51B9A.9	9	12	9	10	2.80E-06	3.78E-06	1.82E-06	2.25E-06
Y51F10.10	218	243	262	374	2.80E-06	2.65E-06	1.82E-06	2.25E-06
Y51F10.11	15	9	30	32	2.80E-06	2.65E-06	1.82E-06	2.25E-06
Y51F10.2	1279	847	1988	2198	2.08E-05	2.19E-05	1.63E-05	2.87E-05
Y51F10.3	101	81	315	346	3.11E-06	2.65E-06	4.05E-06	5.33E-06
Y51F10.4a	178	209	245	306	1.35E-04	8.44E-05	1.36E-04	1.86E-04
Y51F10.4b	124	145	165	212	9.41E-06	7.12E-06	1.91E-05	2.59E-05
Y51F10.5	193	180	374	444	1.01E-05	1.12E-05	9.06E-06	1.39E-05
Y51F10.6	31	50	45	72	1.09E-05	1.20E-05	9.40E-06	1.49E-05
Y51F10.7	533	839	1293	624	1.39E-05	1.23E-05	1.76E-05	2.57E-05
Y51F10.8	5	4	15	10	5.24E-06	7.99E-06	4.94E-06	9.76E-06
Y51F10.9	103	111	191	334	7.90E-05	1.17E-04	1.25E-04	7.43E-05
Y51H1A.1a	60	67	139	173	2.80E-06	2.65E-06	1.82E-06	2.25E-06
Y51H1A.1b	85	88	163	206	6.44E-06	6.53E-06	7.76E-06	1.68E-05

Y51H1A.2	159	149	290	366	5.74E-06	6.06E-06	8.66E-06	1.33E-05
Y51H1A.3b	394	480	702	513	5.74E-06	5.61E-06	7.16E-06	1.12E-05
Y51H1A.5.1	100	136	195	223	9.52E-06	8.44E-06	1.13E-05	1.76E-05
Y51H1A.5.2	90	123	169	207	6.48E-05	7.46E-05	7.51E-05	6.78E-05
Y51H1A.6	194	176	343	496	6.83E-06	8.78E-06	8.67E-06	1.22E-05
Y51H1A.7	84	94	133	193	6.44E-06	8.33E-06	7.89E-06	1.19E-05
Y51H4A.1	18	6	8	15	8.99E-06	7.72E-06	1.03E-05	1.85E-05
Y51H4A.10	0	0	1	0	7.50E-06	7.94E-06	7.73E-06	1.38E-05
Y51H4A.12	865	686	1232	1566	3.14E-06	2.65E-06	1.82E-06	2.25E-06
Y51H4A.13	32	55	11	12	2.80E-06	2.65E-06	1.82E-06	2.25E-06
Y51H4A.15a	110	140	170	132	1.96E-05	1.47E-05	1.82E-05	2.85E-05
Y51H4A.15b	90	111	118	109	2.80E-06	3.17E-06	1.82E-06	2.25E-06
Y51H4A.16	6	6	4	2	1.84E-05	2.21E-05	1.85E-05	1.77E-05
Y51H4A.18	5	14	9	9	1.92E-05	2.24E-05	1.64E-05	1.87E-05
Y51H4A.19	13	19	22	16	2.80E-06	2.65E-06	1.82E-06	2.25E-06
Y51H4A.2	5	4	3	9	2.80E-06	3.86E-06	1.82E-06	2.25E-06
Y51H4A.20	4	14	4	4	2.80E-06	2.99E-06	2.37E-06	2.25E-06
Y51H4A.21	3	4	7	6	2.80E-06	2.65E-06	1.82E-06	2.25E-06
Y51H4A.22	18	20	15	8	2.80E-06	5.95E-06	1.82E-06	2.25E-06
Y51H4A.23	1	2	3	2	2.80E-06	2.65E-06	1.82E-06	2.25E-06
Y51H4A.24	114	133	184	197	2.80E-06	2.65E-06	1.82E-06	2.25E-06
Y51H4A.25a	32	34	30	33	2.80E-06	2.65E-06	1.82E-06	2.25E-06
Y51H4A.25b	26	27	17	19	7.87E-06	8.68E-06	8.27E-06	1.09E-05
Y51H4A.26	3	1	0	0	2.80E-06	2.65E-06	1.82E-06	2.25E-06
Y51H4A.28	49	57	32	27	4.51E-06	4.44E-06	1.93E-06	2.65E-06
Y51H4A.4	18	9	13	11	2.80E-06	2.65E-06	1.82E-06	2.25E-06
Y51H4A.5	25	28	26	32	1.74E-05	1.92E-05	7.40E-06	7.71E-06
Y51H4A.6	2	5	8	2	3.02E-06	2.65E-06	1.82E-06	2.25E-06
Y51H4A.7.1	122	227	167	195	2.80E-06	2.65E-06	1.82E-06	2.25E-06
Y51H4A.7.2	116	216	152	191	2.80E-06	2.65E-06	2.53E-06	2.25E-06
Y51H4A.8	32	35	50	31	6.08E-06	1.07E-05	5.39E-06	7.78E-06
Y51H4A.9	178	177	97	87	5.71E-06	1.00E-05	4.87E-06	7.53E-06
Y51H4A.t1	0	1	0	0	5.88E-06	6.08E-06	5.98E-06	4.59E-06
Y51H4A.t7	0	1	0	0	1.77E-05	1.66E-05	6.27E-06	6.95E-06
Y51H7BM.1	15	22	29	25	2.80E-06	2.65E-06	1.82E-06	2.25E-06
Y51H7BR.1	3	1	4	4	2.80E-06	2.65E-06	1.82E-06	2.25E-06
Y51H7BR.2	5	6	6	6	2.80E-06	2.65E-06	1.82E-06	2.25E-06
Y51H7BR.3	31	20	51	32	2.80E-06	2.65E-06	1.82E-06	2.25E-06
Y51H7BR.4	5	11	3	2	2.80E-06	2.65E-06	1.82E-06	2.25E-06
Y51H7BR.5	6	2	6	11	7.11E-06	4.34E-06	7.60E-06	5.89E-06
Y51H7BR.6	4	3	4	2	2.80E-06	2.70E-06	1.82E-06	2.25E-06
Y51H7BR.7	8	5	0	3	2.80E-06	2.65E-06	1.82E-06	2.32E-06
Y51H7BR.8	1	1	1	1	2.80E-06	2.65E-06	1.82E-06	2.25E-06
Y51H7C.1	411	700	546	926	2.80E-06	2.65E-06	1.82E-06	2.25E-06
Y51H7C.10	8	1	9	5	2.80E-06	2.65E-06	1.82E-06	2.25E-06
Y51H7C.11	344	250	280	491	3.27E-05	5.26E-05	2.83E-05	5.92E-05
Y51H7C.12	11	6	5	9	2.80E-06	2.65E-06	1.82E-06	2.25E-06
Y51H7C.13	361	550	207	230	1.17E-05	8.01E-06	6.18E-06	1.34E-05
Y51H7C.15	16	17	8	16	2.80E-06	2.65E-06	1.82E-06	2.25E-06
Y51H7C.2	1	1	0	0	3.91E-05	5.62E-05	1.46E-05	2.00E-05
Y51H7C.3	89	97	215	141	3.81E-06	3.81E-06	1.82E-06	3.06E-06
Y51H7C.4	31	51	38	40	2.80E-06	2.65E-06	1.82E-06	2.25E-06
Y51H7C.5	102	113	139	218	2.11E-05	2.17E-05	3.31E-05	2.68E-05
Y51H7C.6a.1	318	448	335	577	6.80E-06	1.06E-05	5.43E-06	7.06E-06
Y51H7C.6a.2	328	465	349	585	1.32E-05	1.38E-05	1.17E-05	2.26E-05
Y51H7C.6b	193	302	261	395	1.29E-05	1.71E-05	8.80E-06	1.87E-05
Y51H7C.6c	116	210	158	235	1.31E-05	1.76E-05	9.09E-06	1.88E-05
Y51H7C.7	137	114	183	166	1.22E-05	1.80E-05	1.07E-05	2.00E-05
Y51H7C.8	1	4	10	1	1.08E-05	1.85E-05	9.58E-06	1.76E-05

Y51H7C.9	13	16	5	9	2.93E-05	2.31E-05	2.55E-05	2.86E-05
Y52B11A.1	4	7	11	1	2.80E-06	2.65E-06	3.33E-06	2.25E-06
Y52B11A.10	191	189	158	288	2.80E-06	2.65E-06	1.82E-06	2.25E-06
Y52B11A.11	187	141	231	268	2.80E-06	2.65E-06	1.82E-06	2.25E-06
Y52B11A.2a	90	120	170	156	8.43E-06	7.88E-06	4.54E-06	1.02E-05
Y52B11A.2b	42	49	80	76	1.16E-05	8.28E-06	9.35E-06	1.34E-05
Y52B11A.3a	214	281	271	338	1.16E-05	1.46E-05	1.42E-05	1.61E-05
Y52B11A.3b	160	216	210	284	1.51E-05	1.66E-05	1.87E-05	2.19E-05
Y52B11A.4	44	48	59	58	1.36E-05	1.69E-05	1.12E-05	1.73E-05
Y52B11A.5	1	1	1	1	1.18E-05	1.51E-05	1.01E-05	1.68E-05
Y52B11A.6	5	7	9	2	2.97E-06	3.04E-06	2.59E-06	3.13E-06
Y52B11A.7	6	7	10	13	2.80E-06	2.65E-06	1.82E-06	2.25E-06
Y52B11A.8	229	168	258	234	2.80E-06	2.65E-06	1.82E-06	2.25E-06
Y52B11A.9	154	193	146	255	2.80E-06	2.65E-06	1.82E-06	2.25E-06
Y52B11B.1	7	7	0	0	4.10E-05	2.84E-05	3.01E-05	3.37E-05
Y52D3.1a.1	159	174	242	299	1.41E-05	1.67E-05	8.71E-06	1.88E-05
Y52D3.1a.2	138	161	210	279	2.80E-06	2.65E-06	1.82E-06	2.25E-06
Y52D5A.1	34	54	189	32	1.30E-05	1.35E-05	1.29E-05	1.97E-05
Y52D5A.2	22	22	10	17	1.31E-05	1.44E-05	1.30E-05	2.13E-05
Y52E8A.1	13	10	46	31	2.80E-06	2.65E-06	5.01E-06	2.25E-06
Y52E8A.2	80	138	149	96	2.80E-06	2.65E-06	1.82E-06	2.25E-06
Y52E8A.3	6	5	13	4	2.80E-06	2.65E-06	5.34E-06	4.45E-06
Y52E8A.4	12	9	19	17	1.49E-05	2.43E-05	1.80E-05	1.43E-05
Y52E8A.5	6	10	10	6	2.80E-06	2.65E-06	2.11E-06	2.25E-06
Y53C10A.1	73	75	99	108	2.80E-06	2.65E-06	1.82E-06	2.25E-06
Y53C10A.10	49	68	21	19	2.80E-06	2.65E-06	1.82E-06	2.25E-06
Y53C10A.12.[illegible]	568	545	687	1044	7.62E-06	7.41E-06	6.72E-06	9.06E-06
Y53C10A.12.[illegible]	511	475	620	954	2.91E-06	3.81E-06	1.82E-06	2.25E-06
Y53C10A.13	15	24	22	27	2.74E-05	2.48E-05	2.15E-05	4.04E-05
Y53C10A.14	29	43	32	39	2.81E-05	2.47E-05	2.22E-05	4.21E-05
Y53C10A.15	4	5	5	4	2.80E-06	2.65E-06	1.82E-06	2.50E-06
Y53C10A.2	2	4	7	4	3.78E-06	5.32E-06	2.71E-06	4.09E-06
Y53C10A.3	4	7	5	2	2.80E-06	2.65E-06	1.82E-06	2.25E-06
Y53C10A.4	64	64	47	98	2.80E-06	2.65E-06	1.82E-06	2.25E-06
Y53C10A.5	109	205	139	199	2.80E-06	2.65E-06	1.82E-06	2.25E-06
Y53C10A.7	30	41	27	49	2.80E-06	2.65E-06	1.82E-06	3.04E-06
Y53C10A.9	39	103	31	27	9.86E-06	1.75E-05	8.16E-06	1.44E-05
Y53C12A.1	1506	1296	2425	2910	3.72E-06	4.81E-06	2.19E-06	4.88E-06
Y53C12A.2.1	28	57	14	27	2.80E-06	2.65E-06	1.82E-06	2.25E-06
Y53C12A.2.2	28	53	13	27	7.02E-05	5.70E-05	7.35E-05	1.09E-04
Y53C12A.3	302	362	409	430	2.80E-06	3.39E-06	1.82E-06	2.25E-06
Y53C12A.4.1	382	482	382	579	2.80E-06	3.78E-06	1.82E-06	2.25E-06
Y53C12A.4.2	246	280	218	357	1.80E-05	2.03E-05	1.58E-05	2.05E-05
Y53C12A.5	13	13	6	11	2.68E-05	3.20E-05	1.74E-05	3.26E-05
Y53C12A.6	26	42	65	69	2.70E-05	2.91E-05	1.56E-05	3.15E-05
Y53C12A.7	11	10	9	7	2.80E-06	2.65E-06	1.82E-06	2.25E-06
Y53C12A.8	14	19	13	8	7.42E-06	1.13E-05	1.21E-05	1.58E-05
Y53C12B.1	329	383	357	571	3.05E-06	2.65E-06	1.82E-06	2.25E-06
Y53C12B.2	495	573	625	648	2.80E-06	2.65E-06	1.82E-06	2.25E-06
Y53C12B.3a	1514	1111	1700	2722	1.55E-05	1.70E-05	1.09E-05	2.16E-05
Y53C12B.3b	1368	1013	1600	2559	4.46E-05	4.88E-05	3.67E-05	4.69E-05
Y53C12B.5a	20	11	5	11	6.21E-05	4.30E-05	4.54E-05	8.96E-05
Y53C12B.5b	29	32	35	30	5.77E-05	4.04E-05	4.39E-05	8.67E-05
Y53C12B.6	8	12	14	18	2.80E-06	2.65E-06	1.82E-06	2.25E-06
Y53C12B.7	5	15	6	9	2.80E-06	2.72E-06	2.06E-06	2.25E-06
Y53C12C.1	2	3	4	8	2.80E-06	2.65E-06	1.82E-06	2.25E-06
Y53F4A.2	5	2	4	4	2.80E-06	3.94E-06	1.82E-06	2.25E-06
Y53F4B.1	4	10	12	11	2.80E-06	2.65E-06	1.82E-06	2.25E-06
Y53F4B.10	4	6	4	2	2.80E-06	2.65E-06	1.82E-06	2.25E-06

Y53F4B.11	25	34	26	7	2.80E-06	2.65E-06	1.82E-06	2.25E-06
Y53F4B.12	14	16	18	13	2.80E-06	2.65E-06	1.82E-06	2.25E-06
Y53F4B.13	262	249	328	599	3.67E-06	4.71E-06	2.48E-06	2.25E-06
Y53F4B.14	59	68	240	67	2.80E-06	2.65E-06	1.82E-06	2.25E-06
Y53F4B.15	153	179	186	231	1.05E-05	9.42E-06	8.55E-06	1.93E-05
Y53F4B.16	2	3	2	1	1.77E-05	1.92E-05	4.68E-05	1.61E-05
Y53F4B.17	10	4	6	5	1.28E-05	1.41E-05	1.01E-05	1.55E-05
Y53F4B.18	159	157	348	431	2.80E-06	2.65E-06	1.82E-06	2.25E-06
Y53F4B.19	12	55	8	10	2.80E-06	2.65E-06	1.82E-06	2.25E-06
Y53F4B.2	26	42	34	45	1.11E-05	1.03E-05	1.58E-05	2.41E-05
Y53F4B.20	24	12	16	7	2.80E-06	5.71E-06	1.82E-06	2.25E-06
Y53F4B.21	105	92	94	210	3.39E-06	5.16E-06	2.88E-06	4.70E-06
Y53F4B.22	592	509	594	833	2.80E-06	2.65E-06	1.82E-06	2.25E-06
Y53F4B.23	10	15	19	12	3.56E-06	2.96E-06	2.08E-06	5.74E-06
Y53F4B.24	23	36	9	10	4.68E-05	3.81E-05	3.06E-05	5.30E-05
Y53F4B.25	37	39	18	24	2.80E-06	3.17E-06	2.79E-06	2.25E-06
Y53F4B.26	9	12	6	4	2.80E-06	2.65E-06	1.82E-06	2.25E-06
Y53F4B.27b.[illegible]	52	121	23	23	2.80E-06	2.65E-06	1.82E-06	2.25E-06
Y53F4B.27b.[illegible]	52	120	23	23	2.80E-06	2.78E-06	1.82E-06	2.25E-06
Y53F4B.28	50	83	29	55	3.47E-06	7.62E-06	1.82E-06	2.25E-06
Y53F4B.3	88	99	151	129	3.47E-06	7.56E-06	1.82E-06	2.25E-06
Y53F4B.30	325	601	1159	446	2.80E-06	3.23E-06	1.82E-06	2.25E-06
Y53F4B.31.1	108	92	131	64	1.64E-05	1.74E-05	1.83E-05	1.93E-05
Y53F4B.31.2	86	72	93	54	5.01E-05	8.76E-05	1.16E-04	5.53E-05
Y53F4B.32	8	10	9	4	1.71E-05	1.38E-05	1.35E-05	8.16E-06
Y53F4B.33	49	68	118	43	1.50E-05	1.18E-05	1.05E-05	7.53E-06
Y53F4B.35	2	3	2	2	2.80E-06	2.65E-06	1.82E-06	2.25E-06
Y53F4B.36	4	14	3	3	7.87E-06	1.03E-05	1.23E-05	5.56E-06
Y53F4B.37	11	3	18	3	2.80E-06	2.65E-06	1.82E-06	2.25E-06
Y53F4B.38	4	5	5	7	2.80E-06	2.65E-06	1.82E-06	2.25E-06
Y53F4B.40	6	4	11	13	2.80E-06	2.65E-06	2.08E-06	2.25E-06
Y53F4B.41	22	20	24	47	2.80E-06	2.65E-06	1.82E-06	2.25E-06
Y53F4B.42	17	18	48	54	3.72E-06	2.65E-06	4.45E-06	6.50E-06
Y53F4B.43	3	2	1	3	3.75E-06	3.23E-06	2.66E-06	6.43E-06
Y53F4B.44	1	0	0	0	2.80E-06	2.65E-06	3.70E-06	5.15E-06
Y53F4B.45	199	396	461	297	2.80E-06	2.65E-06	1.82E-06	2.25E-06
Y53F4B.46	0	2	1	0	2.80E-06	2.65E-06	1.82E-06	2.25E-06
Y53F4B.4a	80	63	71	115	1.85E-05	3.47E-05	2.78E-05	2.21E-05
Y53F4B.4b	89	66	80	120	2.80E-06	2.65E-06	1.82E-06	2.25E-06
Y53F4B.5	1	1	1	2	6.78E-06	5.05E-06	3.92E-06	7.83E-06
Y53F4B.6	2	3	3	4	7.06E-06	4.95E-06	4.14E-06	7.65E-06
Y53F4B.7	9	12	6	4	2.80E-06	2.65E-06	1.82E-06	2.25E-06
Y53F4B.8	5	10	4	5	2.80E-06	2.65E-06	1.82E-06	2.25E-06
Y53F4B.9	447	390	731	1118	2.80E-06	2.78E-06	1.82E-06	2.25E-06
Y53G8AL.1	20	27	34	19	2.80E-06	2.65E-06	1.82E-06	2.25E-06
Y53G8AL.2.1	660	900	690	860	1.37E-05	1.13E-05	1.46E-05	2.76E-05
Y53G8AL.2.2	596	828	609	800	2.80E-06	2.65E-06	1.82E-06	2.25E-06
Y53G8AL.3	5	4	5	1	5.12E-05	6.60E-05	3.49E-05	5.36E-05
Y53G8AM.2	38	30	48	63	5.14E-05	6.75E-05	3.42E-05	5.54E-05
Y53G8AM.4	3	4	43	10	2.80E-06	2.65E-06	1.82E-06	2.25E-06
Y53G8AM.5	4	1	3	0	1.92E-05	1.43E-05	1.58E-05	2.55E-05
Y53G8AM.6	4	3	5	4	2.80E-06	2.65E-06	3.37E-06	2.25E-06
Y53G8AM.7	6	4	4	0	2.80E-06	2.65E-06	1.82E-06	2.25E-06
Y53G8AM.8.[illegible]	66	97	53	108	2.80E-06	2.65E-06	1.82E-06	2.25E-06
Y53G8AM.8.[illegible]	66	96	53	105	2.80E-06	2.65E-06	1.82E-06	2.25E-06
Y53G8AR.1	7	3	5	4	2.80E-06	3.84E-06	1.82E-06	3.64E-06
Y53G8AR.2a	328	295	1013	1174	2.83E-06	3.89E-06	1.82E-06	3.62E-06
Y53G8AR.2b	176	135	211	430	2.80E-06	2.65E-06	1.82E-06	2.25E-06
Y53G8AR.3	210	216	201	299	1.36E-05	1.16E-05	2.74E-05	3.92E-05

Y53G8AR.5	54	73	53	97	8.85E-06	6.40E-06	6.91E-06	1.74E-05
Y53G8AR.6	137	163	403	364	2.61E-05	2.54E-05	1.63E-05	2.98E-05
Y53G8AR.7a	71	80	55	78	7.00E-06	8.94E-06	4.46E-06	1.01E-05
Y53G8AR.7b	7	6	5	7	1.15E-05	1.29E-05	2.20E-05	2.45E-05
Y53G8AR.8	62	96	72	108	4.84E-06	5.16E-06	2.44E-06	4.30E-06
Y53G8AR.9	159	141	406	285	3.53E-06	2.86E-06	1.82E-06	2.83E-06
Y53G8B.1	51	50	52	42	5.07E-06	7.41E-06	3.83E-06	7.11E-06
Y53G8B.2	46	95	64	68	8.96E-06	7.51E-06	1.49E-05	1.29E-05
Y53G8B.3	12	11	14	10	8.90E-06	8.25E-06	5.90E-06	5.89E-06
Y53G8B.4	139	158	131	187	4.48E-06	8.76E-06	4.06E-06	5.33E-06
Y53G8B.4b	212	244	215	286	2.80E-06	2.65E-06	1.82E-06	2.25E-06
Y53H1A.1	100	119	126	215	1.33E-05	1.43E-05	8.18E-06	1.44E-05
Y53H1A.2	136	126	186	150	1.92E-05	2.09E-05	1.27E-05	2.08E-05
Y53H1A.3	0	1	1	3	5.66E-06	6.37E-06	4.65E-06	9.78E-06
Y53H1A.4	3	3	5	3	2.11E-05	1.84E-05	1.87E-05	1.87E-05
Y53H1A.5	167	148	255	263	2.80E-06	2.65E-06	1.82E-06	2.25E-06
Y53H1A.6	2	2	0	0	2.80E-06	2.65E-06	1.82E-06	2.25E-06
Y53H1B.1	11	22	13	11	1.95E-05	1.63E-05	1.94E-05	2.47E-05
Y53H1B.2	156	317	265	306	2.80E-06	2.65E-06	1.82E-06	2.25E-06
Y53H1B.4	2	1	1	0	2.80E-06	2.65E-06	1.82E-06	2.25E-06
Y53H1B.6	1	2	6	0	2.38E-05	4.58E-05	2.63E-05	3.76E-05
Y53H1C.1a	393	358	618	679	2.80E-06	2.65E-06	1.82E-06	2.25E-06
Y53H1C.1b	370	339	547	637	2.80E-06	2.65E-06	1.82E-06	2.25E-06
Y53H1C.3	11	26	18	7	2.18E-05	1.87E-05	2.23E-05	3.02E-05
Y54B9A.1	8	8	13	11	2.12E-05	1.83E-05	2.04E-05	2.93E-05
Y54C5A.1	0	3	3	0	2.80E-06	2.65E-06	1.82E-06	2.25E-06
Y54E10A.11	558	549	674	1189	2.80E-06	2.65E-06	2.42E-06	2.54E-06
Y54E10A.12	307	251	293	547	2.80E-06	2.65E-06	1.82E-06	2.25E-06
Y54E10A.13	2	1	1	1	1.40E-05	1.30E-05	1.10E-05	2.40E-05
Y54E10A.14	120	118	133	179	2.02E-05	1.56E-05	1.25E-05	2.89E-05
Y54E10A.15	344	299	620	780	2.80E-06	2.65E-06	1.82E-06	2.25E-06
Y54E10A.16b	49	56	49	51	1.40E-05	1.30E-05	1.01E-05	1.68E-05
Y54E10A.17.1	195	327	193	169	1.83E-05	1.50E-05	2.14E-05	3.33E-05
Y54E10A.17.2	165	255	147	136	8.90E-06	9.60E-06	5.79E-06	7.44E-06
Y54E10A.2	251	247	506	624	3.81E-05	6.03E-05	2.45E-05	2.65E-05
Y54E10A.3	416	539	546	630	3.17E-05	4.63E-05	1.84E-05	2.10E-05
Y54E10A.4a.1	205	193	198	254	1.15E-05	1.07E-05	1.52E-05	2.31E-05
Y54E10A.4a.2	195	178	192	245	4.31E-05	5.28E-05	3.68E-05	5.25E-05
Y54E10A.4b	205	193	198	254	1.04E-05	9.20E-06	6.51E-06	1.03E-05
Y54E10A.5.1	259	256	392	360	1.29E-05	1.11E-05	8.27E-06	1.30E-05
Y54E10A.5.2	157	183	206	233	1.04E-05	9.20E-06	6.51E-06	1.03E-05
Y54E10A.6	386	254	880	1249	4.52E-05	4.23E-05	4.46E-05	5.05E-05
Y54E10A.7	125	156	197	164	3.13E-05	3.44E-05	2.67E-05	3.73E-05
Y54E10A.9a	1575	1400	1667	2745	2.73E-05	1.70E-05	4.05E-05	7.09E-05
Y54E10A.9b	1447	1264	1615	2430	1.93E-05	2.28E-05	1.98E-05	2.04E-05
Y54E10A.9c	1144	1049	1335	2086	7.62E-05	6.40E-05	5.25E-05	1.07E-04
Y54E10BL.1	4	6	1	0	8.00E-05	6.60E-05	5.81E-05	1.08E-04
Y54E10BL.2	324	453	152	174	6.46E-05	5.60E-05	4.91E-05	9.46E-05
Y54E10BL.3	19	10	62	27	2.80E-06	2.65E-06	1.82E-06	2.25E-06
Y54E10BL.4	136	155	123	161	4.04E-05	5.33E-05	1.23E-05	1.74E-05
Y54E10BL.5	450	648	2189	592	3.39E-06	2.65E-06	7.18E-06	3.85E-06
Y54E10BL.6	168	175	397	362	9.41E-06	1.01E-05	5.54E-06	8.93E-06
Y54E10BR.1	227	245	429	433	1.21E-04	1.65E-04	3.84E-04	1.28E-04
Y54E10BR.3	243	311	315	284	1.06E-05	1.05E-05	1.64E-05	1.84E-05
Y54E10BR.4	294	383	350	409	8.62E-06	8.81E-06	1.06E-05	1.32E-05
Y54E10BR.5	538	543	622	676	2.36E-05	2.86E-05	2.00E-05	2.22E-05
Y54E10BR.6	260	318	473	350	2.76E-05	3.39E-05	2.14E-05	3.08E-05
Y54E10BR.7	14	11	18	15	8.60E-05	8.19E-05	6.47E-05	8.67E-05
Y54E10BR.8	144	133	215	226	4.45E-05	5.14E-05	5.26E-05	4.81E-05

Y54E2A.1	7	16	8	7	2.80E-06	2.65E-06	1.82E-06	2.25E-06
Y54E2A.10	3	5	10	5	1.15E-05	1.01E-05	1.12E-05	1.46E-05
Y54E2A.11a.1	1207	1225	1568	2335	2.80E-06	2.65E-06	1.82E-06	2.25E-06
Y54E2A.11a.2	1135	1146	1511	2163	2.80E-06	2.65E-06	1.82E-06	2.25E-06
Y54E2A.11b.1	1053	1067	1379	2054	5.24E-05	5.03E-05	4.44E-05	8.15E-05
Y54E2A.11b.2	270	310	370	497	5.77E-05	5.50E-05	5.00E-05	8.83E-05
Y54E2A.12	205	157	298	337	5.71E-05	5.47E-05	4.87E-05	8.95E-05
Y54E2A.13	0	1	0	1	5.03E-05	5.46E-05	4.49E-05	7.44E-05
Y54E2A.3	382	240	490	402	1.53E-05	1.10E-05	1.44E-05	2.02E-05
Y54E2A.4	39	28	34	68	2.80E-06	2.65E-06	1.82E-06	2.25E-06
Y54E2A.5	6	13	6	2	4.88E-05	2.90E-05	4.07E-05	4.12E-05
Y54E2A.6	738	743	632	1268	8.18E-06	5.55E-06	4.65E-06	1.14E-05
Y54E2A.7	11	26	9	14	2.80E-06	2.65E-06	1.82E-06	2.25E-06
Y54E2A.8	187	169	170	220	1.43E-05	1.36E-05	7.94E-06	1.97E-05
Y54E2A.9	4	13	7	5	2.80E-06	3.70E-06	1.82E-06	2.25E-06
Y54E5A.1	42	83	43	53	1.33E-05	1.13E-05	7.87E-06	1.26E-05
Y54E5A.2	8	6	9	2	2.80E-06	2.65E-06	1.82E-06	2.25E-06
Y54E5A.3	14	25	10	14	3.86E-06	7.22E-06	2.59E-06	3.94E-06
Y54E5A.4.1	475	376	534	823	2.80E-06	2.65E-06	1.82E-06	2.25E-06
Y54E5A.4.2	423	326	464	764	2.80E-06	2.65E-06	1.82E-06	2.25E-06
Y54E5A.5.1	191	257	306	240	2.96E-05	2.21E-05	2.16E-05	4.12E-05
Y54E5A.5.2	163	230	263	224	2.93E-05	2.13E-05	2.09E-05	4.25E-05
Y54E5A.6	70	77	84	128	3.68E-05	4.67E-05	3.83E-05	3.71E-05
Y54E5A.7	234	254	338	447	3.12E-05	4.17E-05	3.28E-05	3.45E-05
Y54E5A.8a	43	54	57	54	5.99E-06	6.22E-06	4.66E-06	8.79E-06
Y54E5A.8b	41	51	56	54	1.16E-05	1.19E-05	1.10E-05	1.79E-05
Y54E5B.1a	379	345	458	536	5.54E-06	6.59E-06	4.79E-06	5.60E-06
Y54E5B.1b.1	238	208	280	315	4.17E-06	4.92E-06	3.72E-06	4.43E-06
Y54E5B.1b.2	110	94	110	135	1.67E-05	1.44E-05	1.32E-05	1.90E-05
Y54E5B.2	154	213	201	281	1.68E-05	1.39E-05	1.29E-05	1.79E-05
Y54E5B.3a	149	166	291	180	1.53E-05	1.23E-05	9.93E-06	1.50E-05
Y54E5B.3b	158	183	343	202	6.52E-06	8.54E-06	5.54E-06	9.58E-06
Y54E5B.5	2	4	19	9	2.14E-05	2.25E-05	2.72E-05	2.08E-05
Y54F10AL.1a	356	330	265	425	2.19E-05	2.39E-05	3.09E-05	2.25E-05
Y54F10AL.1b	197	183	142	203	2.80E-06	2.65E-06	1.82E-06	2.25E-06
Y54F10AL.2b	193	201	388	363	3.31E-05	2.90E-05	1.60E-05	3.17E-05
Y54F10AL.2c	388	316	506	599	2.85E-05	2.50E-05	1.34E-05	2.36E-05
Y54F10AM.1	5	4	10	10	1.33E-05	1.31E-05	1.74E-05	2.01E-05
Y54F10AM.1(	307	279	621	1020	1.32E-05	1.02E-05	1.12E-05	1.64E-05
Y54F10AM.1	39	54	86	62	2.80E-06	2.65E-06	1.82E-06	2.25E-06
Y54F10AM.2a	64	64	40	47	8.57E-06	7.35E-06	1.13E-05	2.29E-05
Y54F10AM.2b	73	78	63	53	4.56E-06	5.95E-06	6.52E-06	5.80E-06
Y54F10AM.2c	71	74	60	53	3.44E-06	3.25E-06	1.82E-06	2.25E-06
Y54F10AM.2c	73	77	62	53	3.61E-06	3.65E-06	2.02E-06	2.25E-06
Y54F10AM.4a	291	404	371	518	3.78E-06	3.73E-06	2.08E-06	2.27E-06
Y54F10AM.4b	276	405	296	393	4.09E-06	4.07E-06	2.26E-06	2.38E-06
Y54F10AM.4c	279	402	294	389	7.81E-06	1.02E-05	6.47E-06	1.12E-05
Y54F10AM.6	48	68	12	14	1.51E-05	2.09E-05	1.05E-05	1.72E-05
Y54F10AM.7	3	9	11	15	1.53E-05	2.08E-05	1.05E-05	1.71E-05
Y54F10AM.8	719	977	770	706	7.03E-06	9.42E-06	1.82E-06	2.25E-06
Y54F10AM.8	665	904	699	665	2.80E-06	2.65E-06	1.82E-06	2.43E-06
Y54F10AR.1	107	72	182	286	4.22E-05	5.41E-05	2.94E-05	3.33E-05
Y54F10AR.2	82	87	141	189	4.04E-05	5.19E-05	2.76E-05	3.25E-05
Y54F10BL.1	13	14	7	3	1.54E-05	9.76E-06	1.70E-05	3.30E-05
Y54F10BM.1	408	306	533	789	1.03E-05	1.04E-05	1.16E-05	1.91E-05
Y54F10BM.1(	9	7	16	2	2.80E-06	2.65E-06	1.82E-06	2.25E-06
Y54F10BM.1:	4	9	2	2	1.99E-05	1.41E-05	1.69E-05	3.09E-05
Y54F10BM.1:	42	44	80	43	2.80E-06	2.65E-06	1.82E-06	2.25E-06
Y54F10BM.1:	36	42	117	37	2.80E-06	2.65E-06	1.82E-06	2.25E-06

Y54F10BM.14	52	46	194	65	4.98E-06	4.95E-06	6.18E-06	4.09E-06
Y54F10BM.15	17	21	18	13	5.91E-06	6.51E-06	1.25E-05	4.86E-06
Y54F10BM.2	1271	890	1373	2116	1.75E-05	1.46E-05	4.25E-05	1.76E-05
Y54F10BM.3	39	66	36	24	2.80E-06	2.65E-06	1.82E-06	2.25E-06
Y54F10BM.4	11	10	15	6	4.00E-05	2.65E-05	2.81E-05	5.35E-05
Y54F10BM.5	9	6	14	3	2.80E-06	2.99E-06	1.82E-06	2.25E-06
Y54F10BM.6	8	4	4	8	2.80E-06	2.65E-06	1.82E-06	2.25E-06
Y54F10BM.7	24	16	24	13	2.80E-06	2.65E-06	1.82E-06	2.25E-06
Y54F10BM.9	106	105	121	139	2.80E-06	2.65E-06	1.82E-06	2.25E-06
Y54G11A.1	118	107	355	382	2.80E-06	2.65E-06	1.82E-06	2.25E-06
Y54G11A.12	2	4	5	5	3.33E-06	3.12E-06	2.48E-06	3.51E-06
Y54G11A.13	278	459	402	523	7.03E-06	6.03E-06	1.38E-05	1.83E-05
Y54G11A.14	34	43	25	18	2.80E-06	2.65E-06	1.82E-06	2.25E-06
Y54G11A.15	3	5	5	1	1.98E-05	3.08E-05	1.86E-05	2.99E-05
Y54G11A.16	4	3	11	3	2.83E-06	3.36E-06	1.82E-06	2.25E-06
Y54G11A.2	96	129	404	171	2.80E-06	2.65E-06	1.82E-06	2.25E-06
Y54G11A.4	12	48	12	21	2.80E-06	2.65E-06	1.82E-06	2.25E-06
Y54G11A.5.1	303	582	447	580	1.17E-05	1.48E-05	3.20E-05	1.67E-05
Y54G11A.5.2	279	536	398	555	2.80E-06	3.41E-06	1.82E-06	2.25E-06
Y54G11A.6	319	547	458	579	2.09E-05	3.79E-05	2.01E-05	3.21E-05
Y54G11A.7	53	153	62	86	1.96E-05	3.55E-05	1.82E-05	3.13E-05
Y54G11A.8a	168	193	135	176	2.24E-05	3.63E-05	2.09E-05	3.27E-05
Y54G11A.8b	147	181	110	149	3.98E-06	1.09E-05	3.02E-06	5.20E-06
Y54G11A.9	135	146	285	160	1.43E-05	1.55E-05	7.47E-06	1.20E-05
Y54G11B.1	9	23	8	15	1.39E-05	1.61E-05	6.74E-06	1.13E-05
Y54G2A.1	49	35	116	24	1.81E-05	1.85E-05	2.48E-05	1.72E-05
Y54G2A.10a	20	13	37	7	2.80E-06	2.65E-06	1.82E-06	2.25E-06
Y54G2A.10b	18	8	24	4	1.05E-05	7.09E-06	1.62E-05	4.14E-06
Y54G2A.11a	157	362	142	304	3.95E-06	2.65E-06	4.76E-06	2.25E-06
Y54G2A.11b	80	129	95	132	2.80E-06	2.65E-06	2.41E-06	2.25E-06
Y54G2A.12	107	176	144	156	9.97E-06	2.17E-05	5.87E-06	1.55E-05
Y54G2A.13	49	63	39	14	1.35E-05	2.05E-05	1.04E-05	1.79E-05
Y54G2A.14.1	434	435	574	344	1.37E-05	2.13E-05	1.20E-05	1.61E-05
Y54G2A.14.2	383	393	544	336	3.00E-06	3.62E-06	1.82E-06	2.25E-06
Y54G2A.15	18	33	22	8	6.13E-05	5.80E-05	5.28E-05	3.90E-05
Y54G2A.16	5	9	7	9	6.01E-05	5.82E-05	5.55E-05	4.23E-05
Y54G2A.17a.	482	487	455	779	4.26E-06	7.35E-06	3.39E-06	2.25E-06
Y54G2A.17a.	493	502	477	785	2.80E-06	2.65E-06	1.82E-06	2.25E-06
Y54G2A.17b	482	487	455	779	3.12E-05	2.98E-05	1.92E-05	4.05E-05
Y54G2A.17c.	138	127	114	187	3.21E-05	3.09E-05	2.02E-05	4.11E-05
Y54G2A.17c.:	153	151	144	199	3.12E-05	2.98E-05	1.92E-05	4.05E-05
Y54G2A.17c.:	142	136	122	193	1.95E-05	1.70E-05	1.05E-05	2.12E-05
Y54G2A.18.1	702	698	630	659	2.05E-05	1.91E-05	1.25E-05	2.14E-05
Y54G2A.18.2	524	532	482	518	1.88E-05	1.70E-05	1.05E-05	2.06E-05
Y54G2A.19	18	16	28	29	1.01E-04	9.48E-05	5.89E-05	7.61E-05
Y54G2A.20	7	4	11	6	9.04E-05	8.67E-05	5.41E-05	7.18E-05
Y54G2A.21	43	78	32	118	6.22E-06	5.24E-06	6.30E-06	8.05E-06
Y54G2A.22	64	73	85	134	2.80E-06	2.65E-06	1.82E-06	2.25E-06
Y54G2A.23.1	583	586	408	515	2.80E-06	2.65E-06	1.82E-06	2.61E-06
Y54G2A.23.2	532	516	315	471	7.00E-06	7.54E-06	6.05E-06	1.18E-05
Y54G2A.24a	4	12	3	4	9.44E-05	8.96E-05	4.30E-05	6.70E-05
Y54G2A.24b	3	10	2	3	9.36E-05	8.57E-05	3.60E-05	6.65E-05
Y54G2A.25a	43	38	24	35	2.80E-06	2.65E-06	1.82E-06	2.25E-06
Y54G2A.25b	14	15	6	21	2.80E-06	2.65E-06	1.82E-06	2.25E-06
Y54G2A.25c	43	38	24	35	2.80E-06	2.65E-06	1.82E-06	2.25E-06
Y54G2A.26b.	283	274	326	448	2.80E-06	2.65E-06	1.82E-06	2.25E-06
Y54G2A.26b.	323	314	377	511	2.80E-06	2.65E-06	1.82E-06	2.25E-06
Y54G2A.27	22	27	26	37	1.87E-05	1.71E-05	1.40E-05	2.38E-05
Y54G2A.28	77	80	84	143	1.87E-05	1.71E-05	1.42E-05	2.37E-05

Y54G2A.29	30	47	23	36	3.58E-06	4.15E-06	2.75E-06	4.81E-06
Y54G2A.2a	719	750	829	1325	7.84E-06	7.70E-06	5.56E-06	1.17E-05
Y54G2A.2b	709	729	807	1296	2.80E-06	2.70E-06	1.82E-06	2.25E-06
Y54G2A.3	173	298	183	193	4.16E-05	4.10E-05	3.12E-05	6.15E-05
Y54G2A.31	690	640	596	873	4.09E-05	3.97E-05	3.03E-05	6.00E-05
Y54G2A.32.1	30	34	18	15	3.06E-05	4.98E-05	2.11E-05	2.74E-05
Y54G2A.32.2	27	31	17	13	9.19E-05	8.05E-05	5.17E-05	9.34E-05
Y54G2A.33	112	114	138	111	5.74E-06	6.14E-06	2.24E-06	2.32E-06
Y54G2A.35	27	33	60	60	5.12E-06	5.55E-06	2.10E-06	2.25E-06
Y54G2A.36	221	263	247	290	1.54E-05	1.48E-05	1.23E-05	1.22E-05
Y54G2A.37	45	78	40	11	2.80E-06	2.65E-06	2.92E-06	3.60E-06
Y54G2A.38	11	6	6	7	6.17E-05	6.94E-05	4.49E-05	6.51E-05
Y54G2A.39	72	101	64	82	3.98E-06	6.51E-06	2.30E-06	2.25E-06
Y54G2A.4	133	179	100	76	2.80E-06	2.65E-06	1.82E-06	2.25E-06
Y54G2A.40	8	17	12	16	5.26E-06	6.98E-06	3.04E-06	4.84E-06
Y54G2A.41	8	16	6	6	9.80E-06	1.25E-05	4.81E-06	4.50E-06
Y54G2A.42	5	3	3	2	2.80E-06	2.65E-06	1.82E-06	2.25E-06
Y54G2A.44	0	4	11	5	2.80E-06	5.00E-06	1.82E-06	2.25E-06
Y54G2A.45	127	87	192	75	2.80E-06	2.65E-06	1.82E-06	2.25E-06
Y54G2A.46	4	10	30	5	2.80E-06	2.65E-06	1.82E-06	2.25E-06
Y54G2A.47	5	6	7	2	1.34E-05	8.65E-06	1.31E-05	6.34E-06
Y54G2A.48	1	5	5	2	2.80E-06	2.65E-06	4.61E-06	2.25E-06
Y54G2A.49	11	13	15	3	2.80E-06	2.65E-06	1.82E-06	2.25E-06
Y54G2A.50	16	35	9	15	2.80E-06	2.65E-06	1.82E-06	2.25E-06
Y54G2A.5a.1	214	258	301	229	3.95E-06	4.42E-06	3.50E-06	2.25E-06
Y54G2A.5a.2	132	133	198	109	3.89E-06	8.07E-06	1.82E-06	2.95E-06
Y54G2A.5b	4	12	5	3	2.15E-05	2.45E-05	1.97E-05	1.85E-05
Y54G2A.6	191	291	759	357	1.69E-05	1.61E-05	1.65E-05	1.12E-05
Y54G2A.8a	10	22	11	11	2.80E-06	2.65E-06	1.82E-06	2.25E-06
Y54G2A.8b	10	20	11	11	2.46E-05	3.54E-05	6.36E-05	3.69E-05
Y54G2A.9	60	92	50	69	2.80E-06	2.65E-06	1.82E-06	2.25E-06
Y54G9A.1	2	1	4	1	2.80E-06	2.65E-06	1.82E-06	2.25E-06
Y54G9A.10	3	4	11	4	6.52E-06	9.47E-06	3.53E-06	6.03E-06
Y54G9A.3	14	29	27	27	2.80E-06	2.65E-06	1.82E-06	2.25E-06
Y54G9A.4	21	15	6	15	2.80E-06	2.65E-06	1.82E-06	2.25E-06
Y54G9A.5	144	117	290	236	2.80E-06	2.65E-06	1.82E-06	2.25E-06
Y54G9A.6	364	404	605	726	2.80E-06	2.65E-06	1.82E-06	2.25E-06
Y54G9A.7	226	231	338	309	1.91E-05	1.46E-05	2.50E-05	2.51E-05
Y54G9A.8	0	3	1	0	3.45E-05	3.62E-05	3.73E-05	5.53E-05
Y54G9A.9	26	31	35	13	3.07E-05	2.96E-05	2.99E-05	3.37E-05
Y54H5A.1.1	603	645	690	761	2.80E-06	2.65E-06	1.82E-06	2.25E-06
Y54H5A.1.2	521	575	578	695	7.87E-06	8.86E-06	6.89E-06	3.17E-06
Y54H5A.2.1	271	263	268	243	4.35E-05	4.40E-05	3.24E-05	4.41E-05
Y54H5A.3	325	414	427	405	4.28E-05	4.46E-05	3.09E-05	4.58E-05
Y54H5A.4.1	142	157	126	186	2.41E-05	2.21E-05	1.55E-05	1.73E-05
Y54H5A.4.2	135	151	117	175	3.89E-05	4.68E-05	3.33E-05	3.89E-05
Y54H5A.4.3	135	151	117	175	1.13E-05	1.18E-05	6.51E-06	1.19E-05
Y55B1AL.1	71	93	49	100	1.09E-05	1.16E-05	6.18E-06	1.14E-05
Y55B1AL.2	139	140	406	145	1.09E-05	1.16E-05	6.18E-06	1.14E-05
Y55B1AL.3a	149	134	169	242	1.11E-05	1.38E-05	5.01E-06	1.26E-05
Y55B1AL.3b.:	94	82	102	123	7.21E-05	6.86E-05	1.37E-04	6.04E-05
Y55B1AL.3b.:	100	91	106	168	5.99E-06	5.11E-06	4.43E-06	7.83E-06
Y55B1AR.1.1	969	1491	2813	949	6.10E-06	5.03E-06	4.32E-06	6.43E-06
Y55B1AR.2a	338	310	397	533	5.57E-06	4.79E-06	3.84E-06	7.51E-06
Y55B1AR.2b	296	268	349	437	2.01E-04	2.93E-04	3.80E-04	1.58E-04
Y55B1AR.3	5	5	5	5	1.93E-05	1.68E-05	1.48E-05	2.45E-05
Y55B1AR.4	12	34	4	9	1.86E-05	1.59E-05	1.43E-05	2.21E-05
Y55B1BL.1	66	118	44	67	2.80E-06	2.65E-06	1.82E-06	2.25E-06
Y55B1BM.1b	245	327	233	359	2.80E-06	3.54E-06	1.82E-06	2.25E-06

Y55B1BM.1c	202	248	186	289	9.55E-06	1.61E-05	4.14E-06	7.78E-06
Y55B1BR.1	6	1	6	1	1.47E-05	1.86E-05	9.13E-06	1.73E-05
Y55B1BR.2	96	98	117	125	1.81E-05	2.11E-05	1.09E-05	2.08E-05
Y55B1BR.3	39	47	35	52	2.80E-06	2.65E-06	1.82E-06	2.25E-06
Y55B1BR.4	23	33	42	80	4.26E-06	4.10E-06	3.37E-06	4.45E-06
Y55B1BR.5	10	19	12	27	2.80E-06	2.65E-06	1.82E-06	2.25E-06
Y55B1BR.6	6	5	1	5	2.80E-06	2.65E-06	1.82E-06	4.23E-06
Y55D5A.1a	19	22	10	19	2.80E-06	4.79E-06	2.08E-06	5.78E-06
Y55D5A.1b	7	13	4	6	4.68E-06	3.68E-06	1.82E-06	3.13E-06
Y55D5A.2	4	3	4	4	2.80E-06	2.65E-06	1.82E-06	2.25E-06
Y55D5A.3	8	9	12	14	2.80E-06	2.65E-06	1.82E-06	2.25E-06
Y55D5A.4	3	24	14	9	2.80E-06	2.65E-06	1.82E-06	2.25E-06
Y55D5A.5	642	570	720	1241	2.80E-06	2.65E-06	1.82E-06	2.25E-06
Y55D5A.6	3	6	4	6	2.80E-06	4.23E-06	1.82E-06	2.25E-06
Y55D9A.1a	706	651	1113	1294	1.24E-05	1.04E-05	9.06E-06	1.93E-05
Y55D9A.1b	807	803	1331	1499	2.80E-06	2.65E-06	1.82E-06	2.25E-06
Y55D9A.2a	217	218	283	418	3.22E-05	2.81E-05	3.30E-05	4.74E-05
Y55D9A.2b	195	202	254	385	3.16E-05	2.97E-05	3.39E-05	4.71E-05
Y55F3AL.1	366	358	621	947	1.40E-05	1.33E-05	1.19E-05	2.16E-05
Y55F3AM.1.1	130	148	177	161	1.29E-05	1.26E-05	1.09E-05	2.05E-05
Y55F3AM.1.2	125	137	159	159	6.22E-06	5.74E-06	6.87E-06	1.29E-05
Y55F3AM.10	21	31	26	10	2.33E-05	2.50E-05	2.06E-05	2.32E-05
Y55F3AM.11	26	33	45	21	2.21E-05	2.29E-05	1.83E-05	2.26E-05
Y55F3AM.12	333	299	641	788	2.80E-06	3.86E-06	2.22E-06	2.25E-06
Y55F3AM.12	182	199	415	496	3.00E-06	3.60E-06	3.37E-06	2.25E-06
Y55F3AM.13	368	346	448	562	2.68E-05	2.27E-05	3.36E-05	5.09E-05
Y55F3AM.14	25	40	20	13	2.02E-05	2.09E-05	3.00E-05	4.43E-05
Y55F3AM.15	444	335	442	666	3.62E-05	3.21E-05	2.87E-05	4.44E-05
Y55F3AM.2	4	7	7	4	2.80E-06	2.65E-06	1.82E-06	2.25E-06
Y55F3AM.3a	691	561	862	1087	2.95E-05	2.10E-05	1.91E-05	3.55E-05
Y55F3AM.3b	97	66	48	87	2.80E-06	2.65E-06	1.82E-06	2.25E-06
Y55F3AM.3c.	523	409	613	862	3.59E-05	2.75E-05	2.91E-05	4.53E-05
Y55F3AM.3c.	472	379	606	823	3.02E-05	1.94E-05	9.71E-06	2.17E-05
Y55F3AM.4	151	128	213	213	3.81E-05	2.81E-05	2.91E-05	5.04E-05
Y55F3AM.5	60	72	100	118	3.68E-05	2.79E-05	3.07E-05	5.15E-05
Y55F3AM.6a	140	106	196	209	1.78E-05	1.42E-05	1.63E-05	2.02E-05
Y55F3AM.6b	129	97	171	182	7.22E-06	8.20E-06	7.84E-06	1.14E-05
Y55F3AM.7	39	30	41	49	1.26E-05	9.02E-06	1.15E-05	1.51E-05
Y55F3AM.8	54	66	157	62	1.39E-05	9.84E-06	1.20E-05	1.57E-05
Y55F3AM.9	203	218	228	334	6.55E-06	4.76E-06	4.48E-06	6.61E-06
Y55F3AR.2	56	52	152	128	1.32E-05	1.52E-05	2.49E-05	1.21E-05
Y55F3AR.3	1546	869	2612	2599	1.60E-05	1.63E-05	1.17E-05	2.12E-05
Y55F3BL.1	61	69	126	87	7.62E-06	6.67E-06	1.34E-05	1.40E-05
Y55F3BL.2	93	100	307	322	7.27E-05	3.86E-05	8.00E-05	9.82E-05
Y55F3BR.1	266	255	310	468	5.43E-06	5.79E-06	7.29E-06	6.21E-06
Y55F3BR.10	18	14	19	14	7.50E-06	7.62E-06	1.61E-05	2.08E-05
Y55F3BR.11	6	10	9	7	1.28E-05	1.16E-05	9.73E-06	1.81E-05
Y55F3BR.4	9	21	10	14	5.12E-06	3.76E-06	3.52E-06	3.19E-06
Y55F3BR.5	25	20	32	21	2.80E-06	2.65E-06	1.82E-06	2.25E-06
Y55F3BR.6	296	173	559	348	2.80E-06	2.65E-06	1.82E-06	2.25E-06
Y55F3BR.7	26	25	72	54	3.64E-06	2.75E-06	3.02E-06	2.45E-06
Y55F3BR.8a	129	134	205	306	2.48E-05	1.37E-05	3.04E-05	2.34E-05
Y55F3BR.8b	103	112	172	274	4.59E-06	4.15E-06	8.25E-06	7.65E-06
Y55F3C.10	4	7	2	3	7.20E-06	7.06E-06	7.43E-06	1.37E-05
Y55F3C.11	10	32	20	39	6.61E-06	6.77E-06	7.18E-06	1.41E-05
Y55F3C.2	3	3	2	1	2.80E-06	2.65E-06	1.82E-06	2.25E-06
Y55F3C.4	1	0	0	0	2.80E-06	3.17E-06	1.82E-06	3.28E-06
Y55F3C.5	0	1	1	1	2.80E-06	2.65E-06	1.82E-06	2.25E-06
Y55F3C.6	47	130	48	235	2.80E-06	2.65E-06	1.82E-06	2.25E-06

Y55F3C.7a	56	48	27	47	2.80E-06	2.65E-06	1.82E-06	2.25E-06
Y55F3C.7b	55	45	27	46	2.80E-06	5.69E-06	1.82E-06	8.75E-06
Y55F3C.8	3	6	4	2	4.03E-06	3.25E-06	1.82E-06	2.72E-06
Y55F3C.9	41	45	37	47	4.00E-06	3.09E-06	1.82E-06	2.68E-06
Y55H10A.2	4	5	6	4	2.80E-06	2.65E-06	1.82E-06	2.25E-06
Y56A3A.10	7	7	13	4	2.80E-06	2.65E-06	1.82E-06	2.25E-06
Y56A3A.11	84	95	105	89	2.80E-06	2.65E-06	1.82E-06	2.25E-06
Y56A3A.12a.	130	229	314	294	2.80E-06	2.65E-06	1.82E-06	2.25E-06
Y56A3A.12a.	129	229	313	294	1.03E-05	1.11E-05	8.42E-06	8.79E-06
Y56A3A.12b.	144	250	341	302	6.94E-06	1.16E-05	1.09E-05	1.26E-05
Y56A3A.12b.	129	228	316	288	7.31E-06	1.23E-05	1.16E-05	1.34E-05
Y56A3A.13	125	172	144	181	7.73E-06	1.27E-05	1.19E-05	1.30E-05
Y56A3A.14	8	8	9	10	7.67E-06	1.28E-05	1.22E-05	1.38E-05
Y56A3A.15	8	7	14	10	1.02E-05	1.33E-05	7.65E-06	1.19E-05
Y56A3A.16	128	117	561	472	2.80E-06	2.65E-06	1.82E-06	2.25E-06
Y56A3A.17a	346	360	679	867	2.80E-06	2.65E-06	1.82E-06	2.25E-06
Y56A3A.17b	328	321	593	807	7.70E-06	6.67E-06	2.20E-05	2.28E-05
Y56A3A.19	534	542	1114	425	2.32E-05	2.27E-05	2.96E-05	4.66E-05
Y56A3A.2	259	208	431	620	2.46E-05	2.27E-05	2.89E-05	4.86E-05
Y56A3A.20.1	927	791	1119	1700	1.18E-04	1.13E-04	1.60E-04	7.53E-05
Y56A3A.20.2	714	610	830	1326	1.51E-05	1.15E-05	1.64E-05	2.91E-05
Y56A3A.21.1	1784	1756	1834	1723	8.00E-05	6.45E-05	6.28E-05	1.18E-04
Y56A3A.21.2	1648	1600	1593	1644	7.87E-05	6.35E-05	5.95E-05	1.17E-04
Y56A3A.22	230	252	254	300	2.78E-04	2.58E-04	1.86E-04	2.16E-04
Y56A3A.27.1	116	150	147	229	2.99E-04	2.74E-04	1.88E-04	2.39E-04
Y56A3A.27.2	106	128	131	216	3.00E-05	3.10E-05	2.16E-05	3.14E-05
Y56A3A.28	111	70	182	199	5.40E-06	6.59E-06	4.45E-06	8.57E-06
Y56A3A.29a	182	172	293	278	5.18E-06	5.90E-06	4.17E-06	8.48E-06
Y56A3A.29b	93	87	136	106	1.07E-05	6.37E-06	1.14E-05	1.54E-05
Y56A3A.30	39	81	43	71	2.13E-05	1.90E-05	2.23E-05	2.61E-05
Y56A3A.31	43	66	128	173	2.55E-05	2.25E-05	2.42E-05	2.33E-05
Y56A3A.32.1	559	640	1113	1312	2.80E-06	3.60E-06	1.82E-06	2.68E-06
Y56A3A.32.2	392	450	252	423	2.97E-06	4.29E-06	5.72E-06	9.56E-06
Y56A3A.33	26	28	37	40	2.74E-05	2.97E-05	3.55E-05	5.17E-05
Y56A3A.36	36	22	52	52	2.06E-05	2.23E-05	8.60E-06	1.78E-05
Y56A3A.4	206	189	284	293	2.80E-06	2.67E-06	2.44E-06	3.24E-06
Y56A3A.5	9	10	15	10	4.76E-06	2.75E-06	4.48E-06	5.53E-06
Y56A3A.6.1	138	115	115	168	2.09E-05	1.81E-05	1.87E-05	2.38E-05
Y56A3A.6.2	130	104	101	158	2.80E-06	2.65E-06	1.82E-06	2.25E-06
Y56A3A.9	9	6	5	2	6.05E-06	4.76E-06	3.28E-06	5.91E-06
Y57A10A.1	157	166	175	298	6.05E-06	4.58E-06	3.06E-06	5.89E-06
Y57A10A.10	41	116	101	114	2.80E-06	2.65E-06	1.82E-06	2.25E-06
Y57A10A.11	1384	1093	290	387	5.43E-06	5.42E-06	3.94E-06	8.30E-06
Y57A10A.13	599	665	829	1099	5.91E-06	1.58E-05	9.48E-06	1.32E-05
Y57A10A.14	30	37	17	22	1.07E-04	7.96E-05	1.45E-05	2.40E-05
Y57A10A.15	62	90	99	185	2.81E-05	2.94E-05	2.53E-05	4.14E-05
Y57A10A.16	112	134	99	170	4.90E-06	5.69E-06	1.82E-06	2.88E-06
Y57A10A.19	120	124	284	300	2.80E-06	2.96E-06	2.24E-06	5.17E-06
Y57A10A.2	43	49	42	59	2.25E-05	2.54E-05	1.29E-05	2.74E-05
Y57A10A.20	33	35	88	110	1.05E-05	1.03E-05	1.62E-05	2.11E-05
Y57A10A.21	7	22	23	33	4.34E-06	4.68E-06	2.77E-06	4.79E-06
Y57A10A.22	3	4	4	6	2.80E-06	2.70E-06	4.68E-06	7.24E-06
Y57A10A.24	12	16	21	13	2.80E-06	5.50E-06	3.95E-06	7.02E-06
Y57A10A.25	207	194	276	372	2.80E-06	2.65E-06	1.82E-06	2.43E-06
Y57A10A.26.	53	106	61	87	2.80E-06	2.65E-06	1.82E-06	2.25E-06
Y57A10A.26.	40	82	42	73	1.37E-05	1.21E-05	1.19E-05	1.98E-05
Y57A10A.27	140	189	204	237	6.41E-06	1.21E-05	4.81E-06	8.48E-06
Y57A10A.28	110	83	174	196	5.49E-06	1.06E-05	3.75E-06	8.05E-06
Y57A10A.29	68	87	267	107	1.16E-05	1.49E-05	1.10E-05	1.58E-05

Y57A10A.3	55	71	70	94	1.31E-05	9.31E-06	1.35E-05	1.87E-05
Y57A10A.30a	32	39	42	33	1.62E-05	1.96E-05	4.14E-05	2.05E-05
Y57A10A.30b	41	41	59	43	8.06E-06	9.84E-06	6.67E-06	1.11E-05
Y57A10A.30b	39	48	54	42	4.96E-06	5.71E-06	4.23E-06	4.12E-06
Y57A10A.31	609	489	1318	1854	4.23E-06	3.99E-06	3.95E-06	3.55E-06
Y57A10A.32	53	32	160	182	5.29E-06	6.14E-06	4.77E-06	4.57E-06
Y57A10A.35	0	1	1	0	2.17E-05	1.65E-05	3.05E-05	5.30E-05
Y57A10A.36	28	34	45	15	1.29E-05	7.33E-06	2.52E-05	3.54E-05
Y57A10A.37	3	2	2	3	2.80E-06	2.65E-06	1.82E-06	2.25E-06
Y57A10A.4	30	44	29	61	2.34E-05	2.68E-05	2.45E-05	1.01E-05
Y57A10A.5	65	74	68	118	2.80E-06	2.65E-06	1.82E-06	2.25E-06
Y57A10A.6	42	47	40	72	2.80E-06	2.65E-06	1.82E-06	2.90E-06
Y57A10A.7	40	46	45	66	4.26E-06	4.55E-06	2.90E-06	6.18E-06
Y57A10A.8	165	159	221	284	2.80E-06	2.75E-06	1.82E-06	3.58E-06
Y57A10A.9	16	14	12	21	2.80E-06	2.65E-06	1.82E-06	2.70E-06
Y57A10B.1	29	43	23	20	1.15E-05	1.05E-05	1.01E-05	1.60E-05
Y57A10B.2	10	9	9	6	3.02E-06	2.65E-06	1.82E-06	3.17E-06
Y57A10B.3	80	114	54	101	2.80E-06	2.75E-06	1.82E-06	2.25E-06
Y57A10B.4	6	2	1	2	2.80E-06	2.65E-06	1.82E-06	2.25E-06
Y57A10B.5	6	4	4	4	9.35E-06	1.26E-05	4.12E-06	9.49E-06
Y57A10B.6	7	7	5	12	2.80E-06	2.65E-06	1.82E-06	2.25E-06
Y57A10B.7	8	16	7	5	2.80E-06	2.65E-06	1.82E-06	2.25E-06
Y57A10B.t1	0	0	1	0	2.80E-06	2.65E-06	1.82E-06	2.25E-06
Y57A10C.1	9	10	7	9	2.80E-06	2.65E-06	1.82E-06	2.25E-06
Y57A10C.10	0	6	3	1	2.80E-06	2.65E-06	1.82E-06	2.25E-06
Y57A10C.11	2	6	8	5	2.80E-06	2.65E-06	1.82E-06	2.25E-06
Y57A10C.3	7	6	6	3	2.80E-06	2.65E-06	1.82E-06	2.25E-06
Y57A10C.4	7	9	4	8	2.80E-06	2.65E-06	1.82E-06	2.25E-06
Y57A10C.5	5	10	6	6	2.80E-06	2.65E-06	1.82E-06	2.25E-06
Y57A10C.6	258	406	216	349	2.80E-06	2.65E-06	1.82E-06	2.25E-06
Y57A10C.7	1	2	10	2	2.80E-06	2.65E-06	1.82E-06	2.25E-06
Y57A10C.8	6	7	5	5	2.16E-05	3.21E-05	1.18E-05	2.35E-05
Y57A10C.9	4	3	1	1	2.80E-06	2.65E-06	1.82E-06	2.25E-06
Y57A10C.t1	0	0	0	1	2.80E-06	2.65E-06	1.82E-06	2.25E-06
Y57E12AL.1b	596	695	1056	1224	2.80E-06	2.65E-06	1.82E-06	2.25E-06
Y57E12AL.2	2	3	4	0	2.80E-06	2.65E-06	1.82E-06	2.25E-06
Y57E12AL.3	22	56	50	76	4.97E-05	5.48E-05	5.73E-05	8.20E-05
Y57E12AL.4	5	7	7	7	2.80E-06	2.65E-06	1.82E-06	2.25E-06
Y57E12AL.5	247	248	379	366	2.80E-06	4.95E-06	3.04E-06	5.69E-06
Y57E12AL.6.1	496	579	1026	502	2.80E-06	2.65E-06	1.82E-06	2.25E-06
Y57E12AL.6.2	424	495	852	462	3.32E-05	3.15E-05	3.32E-05	3.95E-05
Y57E12AR.1	23	31	27	11	1.16E-04	1.28E-04	1.57E-04	9.47E-05
Y57E12B.1	0	0	0	1	9.15E-05	1.01E-04	1.20E-04	8.01E-05
Y57E12B.2	1	1	0	0	6.22E-06	7.94E-06	4.76E-06	2.38E-06
Y57E12B.3	28	44	49	18	2.80E-06	2.65E-06	1.82E-06	2.25E-06
Y57E12B.4	3	6	6	2	2.80E-06	2.65E-06	1.82E-06	2.25E-06
Y57G11A.1a	128	198	67	127	2.80E-06	2.65E-06	1.82E-06	2.25E-06
Y57G11A.1b	130	195	62	127	2.80E-06	2.65E-06	1.82E-06	2.25E-06
Y57G11A.2	161	289	137	95	7.53E-06	1.10E-05	2.57E-06	6.00E-06
Y57G11A.3	62	94	37	71	8.37E-06	1.19E-05	2.61E-06	6.57E-06
Y57G11A.4	72	145	51	75	8.79E-06	1.49E-05	4.87E-06	4.16E-06
Y57G11A.5	1	2	3	5	7.50E-06	1.08E-05	2.92E-06	6.90E-06
Y57G11A.6	5	4	6	1	1.01E-05	1.93E-05	4.66E-06	8.48E-06
Y57G11B.1	9	5	6	7	2.80E-06	2.65E-06	1.82E-06	2.25E-06
Y57G11B.2	25	39	14	17	2.80E-06	2.65E-06	1.82E-06	2.25E-06
Y57G11B.3	20	29	8	19	2.80E-06	2.65E-06	1.82E-06	2.25E-06
Y57G11B.4	7	6	5	6	5.71E-06	8.44E-06	2.10E-06	3.13E-06
Y57G11B.5	531	663	705	696	2.80E-06	3.09E-06	1.82E-06	2.25E-06
Y57G11B.6	2	2	1	2	2.80E-06	2.65E-06	1.82E-06	2.25E-06

Y57G11B.7	19	43	27	9	8.77E-05	1.03E-04	7.58E-05	9.23E-05
Y57G11C.1	6	2	19	5	2.80E-06	2.65E-06	1.82E-06	2.25E-06
Y57G11C.10a	2055	2830	2290	2838	3.28E-06	7.01E-06	3.04E-06	2.25E-06
Y57G11C.10a	2096	2880	2395	2907	2.80E-06	2.65E-06	1.82E-06	2.25E-06
Y57G11C.10b	1966	2720	2202	2751	1.36E-04	1.77E-04	9.87E-05	1.51E-04
Y57G11C.11a	60	55	71	86	1.39E-04	1.80E-04	1.03E-04	1.55E-04
Y57G11C.14	30	58	35	22	1.28E-04	1.68E-04	9.36E-05	1.44E-04
Y57G11C.16.	7049	7419	14915	5930	7.08E-06	6.14E-06	5.45E-06	8.14E-06
Y57G11C.16.	6230	6424	12514	5581	4.62E-06	8.41E-06	3.50E-06	2.72E-06
Y57G11C.16.	6285	6504	12540	5635	1.40E-03	1.39E-03	1.93E-03	9.48E-04
Y57G11C.17	15	42	25	31	1.24E-03	1.21E-03	1.62E-03	8.93E-04
Y57G11C.18	23	11	26	24	1.25E-03	1.22E-03	1.63E-03	9.02E-04
Y57G11C.2	4	7	9	3	2.80E-06	2.72E-06	1.82E-06	2.25E-06
Y57G11C.20	22	37	35	16	2.80E-06	2.65E-06	1.84E-06	2.25E-06
Y57G11C.21	1	1	2	1	2.80E-06	2.65E-06	1.82E-06	2.25E-06
Y57G11C.22a	33	37	27	45	2.80E-06	2.65E-06	1.82E-06	2.25E-06
Y57G11C.23	16	39	13	15	2.80E-06	2.65E-06	1.82E-06	2.25E-06
Y57G11C.24a	407	480	329	508	2.80E-06	2.78E-06	1.82E-06	2.88E-06
Y57G11C.24a	407	481	332	508	2.80E-06	2.65E-06	1.82E-06	2.25E-06
Y57G11C.24a	424	493	341	518	1.66E-05	1.85E-05	8.75E-06	1.67E-05
Y57G11C.24a	407	480	330	508	1.66E-05	1.85E-05	8.80E-06	1.66E-05
Y57G11C.24c	401	480	333	508	1.68E-05	1.84E-05	8.78E-06	1.65E-05
Y57G11C.24c	407	480	330	508	1.69E-05	1.88E-05	8.89E-06	1.69E-05
Y57G11C.24c	400	479	332	508	1.63E-05	1.85E-05	8.82E-06	1.66E-05
Y57G11C.24c	398	476	329	505	1.69E-05	1.88E-05	8.89E-06	1.69E-05
Y57G11C.24c	400	479	335	509	1.64E-05	1.85E-05	8.82E-06	1.67E-05
Y57G11C.24c	397	475	331	502	1.65E-05	1.87E-05	8.89E-06	1.68E-05
Y57G11C.24c	400	479	332	509	1.63E-05	1.85E-05	8.89E-06	1.67E-05
Y57G11C.24c	398	479	332	509	1.65E-05	1.86E-05	8.95E-06	1.68E-05
Y57G11C.24c	387	466	322	499	1.63E-05	1.85E-05	8.82E-06	1.67E-05
Y57G11C.24c	424	493	341	518	1.62E-05	1.84E-05	8.80E-06	1.66E-05
Y57G11C.24e	309	366	279	407	1.69E-05	1.92E-05	9.13E-06	1.75E-05
Y57G11C.24e	308	364	278	407	1.68E-05	1.84E-05	8.78E-06	1.65E-05
Y57G11C.24g	441	525	367	548	1.73E-05	1.94E-05	1.02E-05	1.83E-05
Y57G11C.24h	420	500	332	503	1.72E-05	1.93E-05	1.01E-05	1.83E-05
Y57G11C.25	39	39	50	54	1.60E-05	1.80E-05	8.66E-06	1.60E-05
Y57G11C.26	5	6	15	3	1.69E-05	1.90E-05	8.69E-06	1.63E-05
Y57G11C.27	2	2	2	1	5.85E-06	5.53E-06	4.88E-06	6.52E-06
Y57G11C.28	14	12	21	13	2.80E-06	2.65E-06	1.82E-06	2.25E-06
Y57G11C.29	13	19	16	13	2.80E-06	2.65E-06	1.82E-06	2.25E-06
Y57G11C.30	13	41	60	27	2.80E-06	2.65E-06	1.82E-06	2.25E-06
Y57G11C.31	122	172	105	139	2.80E-06	2.65E-06	1.82E-06	2.25E-06
Y57G11C.32	18	28	25	24	2.80E-06	2.65E-06	1.82E-06	2.25E-06
Y57G11C.33	138	198	244	257	1.01E-05	1.34E-05	5.65E-06	9.22E-06
Y57G11C.34	125	181	180	146	2.80E-06	2.65E-06	1.82E-06	2.25E-06
Y57G11C.35	0	5	0	0	2.86E-05	3.87E-05	3.29E-05	4.27E-05
Y57G11C.36.	215	200	262	278	2.09E-05	2.86E-05	1.96E-05	1.96E-05
Y57G11C.36.	200	194	248	270	2.80E-06	2.83E-06	1.82E-06	2.25E-06
Y57G11C.36.	168	163	193	217	1.36E-05	1.20E-05	1.08E-05	1.41E-05
Y57G11C.37	38	43	14	37	1.39E-05	1.27E-05	1.12E-05	1.50E-05
Y57G11C.38	17	27	31	12	1.46E-05	1.34E-05	1.09E-05	1.51E-05
Y57G11C.39	4	6	2	3	2.80E-06	2.65E-06	1.82E-06	2.25E-06
Y57G11C.3a	301	358	462	541	2.80E-06	2.65E-06	2.10E-06	2.25E-06
Y57G11C.3b	231	278	363	457	2.80E-06	2.65E-06	1.82E-06	2.25E-06
Y57G11C.4	89	124	110	109	3.31E-05	3.72E-05	3.31E-05	4.78E-05
Y57G11C.40	18	10	22	17	3.34E-05	3.80E-05	3.42E-05	5.31E-05
Y57G11C.41	11	5	8	4	1.14E-05	1.50E-05	9.17E-06	1.12E-05
Y57G11C.42	29	86	35	15	3.70E-06	2.65E-06	2.93E-06	2.81E-06
Y57G11C.43	143	196	268	277	2.80E-06	2.65E-06	1.82E-06	2.25E-06

Y57G11C.44	5	10	7	6	2.80E-06	4.58E-06	1.82E-06	2.25E-06
Y57G11C.45	32	36	30	42	1.76E-05	2.28E-05	2.15E-05	2.74E-05
Y57G11C.46	15	23	26	30	2.80E-06	2.65E-06	1.82E-06	2.25E-06
Y57G11C.47	3	6	5	5	7.48E-06	7.94E-06	4.56E-06	7.87E-06
Y57G11C.48	9	15	7	4	2.80E-06	2.65E-06	1.82E-06	2.38E-06
Y57G11C.49	6	8	6	4	2.80E-06	2.65E-06	1.82E-06	2.25E-06
Y57G11C.5	32	77	38	38	2.80E-06	3.94E-06	1.82E-06	2.25E-06
Y57G11C.50	20	20	17	13	2.80E-06	2.65E-06	1.82E-06	2.25E-06
Y57G11C.51	158	112	271	178	2.80E-06	5.74E-06	1.95E-06	2.41E-06
Y57G11C.52	3	8	4	3	2.80E-06	2.65E-06	1.82E-06	2.25E-06
Y57G11C.6	24	38	20	21	3.66E-05	2.45E-05	4.09E-05	3.32E-05
Y57G11C.7	6	11	21	6	2.80E-06	2.91E-06	1.82E-06	2.25E-06
Y57G11C.8	25	32	37	37	2.80E-06	2.78E-06	1.82E-06	2.25E-06
Y57G11C.9a	998	850	1304	1747	2.80E-06	2.65E-06	1.82E-06	2.25E-06
Y57G11C.9c.[illegible]	319	254	345	574	2.80E-06	3.04E-06	2.42E-06	2.99E-06
Y57G11C.9c.[illegible]	199	138	176	234	6.18E-05	4.97E-05	5.25E-05	8.69E-05
Y57G7A.1	18	21	40	16	2.40E-05	1.80E-05	1.69E-05	3.47E-05
Y57G7A.10a	228	304	260	336	8.92E-05	5.84E-05	5.13E-05	8.42E-05
Y57G7A.11	9	16	12	15	4.65E-06	5.13E-06	6.74E-06	3.33E-06
Y57G7A.12	4	7	2	3	2.42E-05	3.05E-05	1.80E-05	2.87E-05
Y57G7A.2	8	16	12	8	2.80E-06	3.07E-06	1.82E-06	2.45E-06
Y57G7A.3a	4	1	2	1	2.80E-06	2.65E-06	1.82E-06	2.25E-06
Y57G7A.3b	2	2	0	0	2.80E-06	3.12E-06	1.82E-06	2.25E-06
Y57G7A.4	5	10	9	6	2.80E-06	2.65E-06	1.82E-06	2.25E-06
Y57G7A.5	29	33	13	18	2.80E-06	2.65E-06	1.82E-06	2.25E-06
Y57G7A.6	62	155	54	71	2.80E-06	2.65E-06	1.82E-06	2.25E-06
Y57G7A.7	5	2	9	2	4.42E-06	4.76E-06	1.82E-06	2.25E-06
Y57G7A.8	12	18	30	17	2.80E-06	6.06E-06	1.82E-06	2.36E-06
Y57G7A.9	16	9	18	8	2.80E-06	2.65E-06	1.82E-06	2.25E-06
Y58A7A.1	54	87	40	64	2.80E-06	2.65E-06	2.06E-06	2.25E-06
Y58A7A.2	15	18	4	6	2.80E-06	2.65E-06	2.00E-06	2.25E-06
Y58A7A.3	178	276	135	416	1.54E-05	2.34E-05	7.42E-06	1.46E-05
Y58A7A.4	128	182	100	306	5.10E-06	5.77E-06	1.82E-06	2.25E-06
Y58A7A.5	31	46	21	36	7.17E-06	1.05E-05	3.53E-06	1.34E-05
Y58A7A.6	8	6	8	5	5.21E-06	6.98E-06	2.64E-06	9.99E-06
Y58G8A.1	30	45	42	44	3.30E-06	4.63E-06	1.82E-06	3.08E-06
Y58G8A.4a	61	76	50	62	2.80E-06	2.65E-06	1.82E-06	2.25E-06
Y58G8A.4b	66	77	51	62	2.80E-06	3.04E-06	1.97E-06	2.54E-06
Y58G8A.5	4	2	6	1	4.59E-06	5.42E-06	2.46E-06	3.76E-06
Y59A8A.1	448	511	535	772	4.82E-06	5.29E-06	2.42E-06	3.62E-06
Y59A8A.2	173	170	258	366	2.80E-06	2.65E-06	1.82E-06	2.25E-06
Y59A8A.3.1	2868	2184	1964	2970	2.67E-05	2.88E-05	2.08E-05	3.70E-05
Y59A8A.3.3	2319	1782	1548	2385	9.97E-06	9.26E-06	9.68E-06	1.69E-05
Y59A8A.4	6	6	0	2	1.30E-04	9.32E-05	5.77E-05	1.08E-04
Y59A8A.5	1	3	2	2	1.28E-04	9.27E-05	5.55E-05	1.05E-04
Y59A8A.6	19	18	30	12	2.80E-06	2.65E-06	1.82E-06	2.25E-06
Y59A8A.7	3	3	7	2	2.80E-06	2.65E-06	1.82E-06	2.25E-06
Y59A8B.10	301	368	213	429	2.80E-06	2.65E-06	1.82E-06	2.25E-06
Y59A8B.11	29	50	19	22	2.80E-06	2.65E-06	1.82E-06	2.25E-06
Y59A8B.12	175	149	531	133	2.27E-05	2.62E-05	1.05E-05	2.60E-05
Y59A8B.13	135	149	175	201	3.53E-06	5.77E-06	1.82E-06	2.25E-06
Y59A8B.14	217	160	445	625	5.49E-05	4.41E-05	1.08E-04	3.35E-05
Y59A8B.19	4	2	8	5	1.38E-05	1.44E-05	1.16E-05	1.65E-05
Y59A8B.1a	437	371	454	669	1.04E-05	7.27E-06	1.39E-05	2.42E-05
Y59A8B.2	73	89	104	145	2.80E-06	2.65E-06	1.82E-06	2.25E-06
Y59A8B.20	185	326	77	43	9.74E-06	7.80E-06	6.60E-06	1.20E-05
Y59A8B.21	41	40	119	159	7.62E-06	8.76E-06	7.05E-06	1.21E-05
Y59A8B.22.1	840	619	870	1208	2.41E-05	4.01E-05	6.52E-06	4.50E-06
Y59A8B.22.2	854	632	894	1252	4.42E-06	4.07E-06	8.33E-06	1.37E-05

Y59A8B.23	651	650	501	889	4.20E-05	2.92E-05	2.83E-05	4.85E-05
Y59A8B.24	4	3	3	1	3.88E-05	2.72E-05	2.65E-05	4.57E-05
Y59A8B.25	52	60	49	82	3.22E-05	3.04E-05	1.61E-05	3.53E-05
Y59A8B.3	5	8	11	1	2.80E-06	2.65E-06	1.82E-06	2.25E-06
Y59A8B.4	0	4	4	3	1.33E-05	1.45E-05	8.14E-06	1.68E-05
Y59A8B.5	3	21	24	7	2.80E-06	2.65E-06	1.82E-06	2.25E-06
Y59A8B.6.1	516	621	448	799	2.80E-06	2.65E-06	1.82E-06	2.25E-06
Y59A8B.6.2	486	575	423	777	2.80E-06	2.65E-06	1.82E-06	2.25E-06
Y59A8B.7	276	272	381	462	1.88E-05	2.14E-05	1.06E-05	2.34E-05
Y59A8B.8	142	167	141	180	1.87E-05	2.09E-05	1.06E-05	2.40E-05
Y59A8B.9	128	139	184	244	3.18E-05	2.96E-05	2.86E-05	4.28E-05
Y59A8B.t2	1	0	0	0	1.54E-05	1.72E-05	9.99E-06	1.57E-05
Y59C2A.1	54	93	62	112	2.54E-05	2.61E-05	2.38E-05	3.89E-05
Y59C2A.2	98	81	125	204	2.80E-06	2.65E-06	1.82E-06	2.25E-06
Y59C2A.3	155	99	67	104	4.00E-06	6.53E-06	2.99E-06	6.68E-06
Y59E1A.1	17	17	11	7	8.20E-06	6.40E-06	6.80E-06	1.37E-05
Y59E1A.2	99	140	193	146	7.22E-06	4.36E-06	2.02E-06	3.89E-06
Y59E1B.1	3	0	6	3	2.80E-06	2.65E-06	1.82E-06	2.25E-06
Y59E1B.2	1	0	1	2	1.61E-05	2.15E-05	2.04E-05	1.90E-05
Y59E9AL.1	1	2	2	1	2.80E-06	2.65E-06	1.82E-06	2.25E-06
Y59E9AL.2	219	368	253	162	2.80E-06	2.65E-06	1.82E-06	2.25E-06
Y59E9AL.3	222	367	256	164	2.80E-06	2.65E-06	1.82E-06	2.25E-06
Y59E9AL.4.1	269	352	246	451	2.09E-05	3.31E-05	1.57E-05	1.24E-05
Y59E9AL.4.2	246	316	221	405	2.17E-05	3.39E-05	1.63E-05	1.29E-05
Y59E9AL.5	6	5	4	3	8.54E-06	1.06E-05	5.08E-06	1.15E-05
Y59E9AL.6	131	199	113	77	8.32E-06	1.01E-05	4.87E-06	1.10E-05
Y59E9AL.7	175	222	215	147	2.80E-06	2.65E-06	1.82E-06	2.25E-06
Y59E9AL.8	7	5	10	6	2.11E-05	3.03E-05	1.18E-05	9.96E-06
Y59E9AR.1	1930	2620	2407	754	2.79E-05	3.35E-05	2.23E-05	1.88E-05
Y59E9AR.10	1	3	1	0	2.80E-06	2.65E-06	1.82E-06	2.25E-06
Y59E9AR.2	17	17	16	8	5.50E-04	7.05E-04	4.46E-04	1.73E-04
Y59E9AR.3	6	6	5	3	2.80E-06	2.65E-06	1.82E-06	2.25E-06
Y59E9AR.4	0	2	5	7	2.80E-06	2.65E-06	1.82E-06	2.25E-06
Y59E9AR.5	6	6	5	3	2.80E-06	2.65E-06	1.82E-06	2.25E-06
Y59E9AR.6	0	5	5	6	2.80E-06	2.65E-06	1.82E-06	2.25E-06
Y59E9AR.7	1951	2659	2474	785	2.80E-06	2.65E-06	1.82E-06	2.25E-06
Y59E9AR.8	17	17	16	8	2.80E-06	2.65E-06	1.82E-06	2.25E-06
Y59E9AR.9	1	3	1	0	5.56E-04	7.16E-04	4.59E-04	1.80E-04
Y59H11AL.1a	10	28	15	9	2.80E-06	2.65E-06	1.82E-06	2.25E-06
Y59H11AL.1b	5	23	6	5	2.80E-06	2.65E-06	1.82E-06	2.25E-06
Y59H11AM.1	464	740	718	188	2.80E-06	2.65E-06	1.82E-06	2.25E-06
Y59H11AM.2	40	84	32	35	2.80E-06	2.65E-06	1.82E-06	2.25E-06
Y59H11AM.3	40	84	32	35	1.35E-04	2.04E-04	1.36E-04	4.40E-05
Y59H11AM.4	14	23	6	7	4.96E-06	9.84E-06	2.59E-06	3.49E-06
Y59H11AR.2a	464	823	430	712	4.96E-06	9.84E-06	2.59E-06	3.49E-06
Y59H11AR.2a	465	826	432	710	2.80E-06	4.36E-06	1.82E-06	2.25E-06
Y59H11AR.2b	412	746	367	644	1.34E-05	2.24E-05	8.05E-06	1.65E-05
Y59H11AR.2c	270	489	258	427	1.34E-05	2.24E-05	8.07E-06	1.64E-05
Y59H11AR.2c	237	438	226	377	1.38E-05	2.35E-05	7.98E-06	1.73E-05
Y59H11AR.3	1	2	5	1	1.43E-05	2.45E-05	8.91E-06	1.82E-05
Y59H11AR.4	46	67	75	60	1.46E-05	2.55E-05	9.07E-06	1.87E-05
Y59H11AR.5	0	1	1	0	2.80E-06	2.65E-06	1.82E-06	2.25E-06
Y5F2A.1.1	1050	1914	2231	789	3.75E-06	5.16E-06	3.97E-06	3.94E-06
Y5F2A.1.2	1039	1886	2219	774	2.80E-06	2.65E-06	1.82E-06	2.25E-06
Y5F2A.3	12	6	11	4	2.19E-04	3.78E-04	3.03E-04	1.32E-04
Y5F2A.4	240	271	304	403	2.25E-04	3.85E-04	3.12E-04	1.34E-04
Y5H2A.1	4	1	0	0	2.80E-06	2.65E-06	1.82E-06	2.25E-06
Y5H2A.2	6	10	13	4	1.85E-05	1.98E-05	1.53E-05	2.50E-05
Y5H2A.3	4	6	1	3	2.80E-06	2.65E-06	1.82E-06	2.25E-06

Y5H2A.4	15	15	3	3	2.80E-06	2.65E-06	1.82E-06	2.25E-06
Y5H2B.1	5	7	11	8	2.80E-06	2.65E-06	1.82E-06	2.25E-06
Y5H2B.2a	42	64	54	71	2.80E-06	2.65E-06	1.82E-06	2.25E-06
Y5H2B.2b	43	65	55	68	2.80E-06	2.65E-06	1.82E-06	2.25E-06
Y5H2B.3	6	8	9	1	3.61E-06	5.18E-06	3.01E-06	4.88E-06
Y5H2B.4	6	6	4	5	3.50E-06	5.00E-06	2.92E-06	4.43E-06
Y5H2B.5	20	26	19	16	2.80E-06	2.65E-06	1.82E-06	2.25E-06
Y5H2B.6	2	4	2	1	2.80E-06	2.65E-06	1.82E-06	2.25E-06
Y5H2B.7	4	3	1	3	2.80E-06	2.65E-06	1.82E-06	2.25E-06
Y60A3A.1.1	226	246	215	367	2.80E-06	2.65E-06	1.82E-06	2.25E-06
Y60A3A.1.2	197	218	192	333	2.80E-06	2.65E-06	1.82E-06	2.25E-06
Y60A3A.10.1	164	159	320	410	8.76E-06	9.02E-06	5.43E-06	1.14E-05
Y60A3A.10.2	101	105	197	340	8.37E-06	8.76E-06	5.30E-06	1.14E-05
Y60A3A.11	34	22	43	29	9.77E-06	8.94E-06	1.24E-05	1.96E-05
Y60A3A.12	42	30	40	68	9.74E-06	9.58E-06	1.24E-05	2.64E-05
Y60A3A.13.1	272	318	319	354	2.80E-06	2.65E-06	1.82E-06	2.25E-06
Y60A3A.13.2	238	291	268	315	3.22E-06	2.65E-06	2.00E-06	4.21E-06
Y60A3A.14	46	70	137	153	2.08E-05	2.30E-05	1.59E-05	2.18E-05
Y60A3A.15	4	7	10	6	1.93E-05	2.24E-05	1.42E-05	2.06E-05
Y60A3A.16	10	10	10	7	4.73E-06	6.80E-06	9.17E-06	1.26E-05
Y60A3A.17	10	11	14	9	2.80E-06	2.65E-06	1.82E-06	2.25E-06
Y60A3A.18	69	94	67	61	2.80E-06	2.65E-06	1.82E-06	2.25E-06
Y60A3A.2	1	3	0	2	2.80E-06	2.65E-06	1.82E-06	2.25E-06
Y60A3A.21	34	109	158	51	1.14E-05	1.46E-05	7.18E-06	8.07E-06
Y60A3A.22	5	4	3	0	2.80E-06	2.65E-06	1.82E-06	2.25E-06
Y60A3A.23	3	4	6	0	1.16E-05	3.52E-05	3.51E-05	1.40E-05
Y60A3A.24	17	23	6	9	2.80E-06	2.65E-06	1.82E-06	2.25E-06
Y60A3A.25	10	18	8	7	2.80E-06	2.65E-06	1.82E-06	2.25E-06
Y60A3A.3	4	0	1	0	2.80E-06	2.65E-06	1.82E-06	2.25E-06
Y60A3A.4	4	6	1	2	2.80E-06	2.65E-06	1.82E-06	2.25E-06
Y60A3A.5	3	2	0	0	2.80E-06	2.65E-06	1.82E-06	2.25E-06
Y60A3A.6	4	2	2	2	2.80E-06	2.65E-06	1.82E-06	2.25E-06
Y60A3A.7	164	258	228	267	2.80E-06	2.65E-06	1.82E-06	2.25E-06
Y60A3A.8	66	83	59	109	2.80E-06	2.65E-06	1.82E-06	2.25E-06
Y60A3A.9	478	628	560	499	1.59E-05	2.36E-05	1.44E-05	2.08E-05
Y60A3A.t2	0	0	1	0	6.52E-06	7.75E-06	3.79E-06	8.64E-06
Y60A9.1	6	4	15	5	6.56E-05	8.14E-05	5.00E-05	5.50E-05
Y60A9.2	3	2	3	2	2.80E-06	2.65E-06	1.82E-06	2.25E-06
Y60A9.3	0	9	4	7	2.80E-06	2.65E-06	1.82E-06	2.25E-06
Y60A9A.1	12	9	13	28	2.80E-06	2.65E-06	1.82E-06	2.25E-06
Y60C6A.1	3	6	4	6	2.80E-06	2.65E-06	1.82E-06	2.25E-06
Y60C6A.t1	1	0	0	0	4.06E-06	2.88E-06	2.88E-06	7.62E-06
Y61A9LA.1	152	140	180	264	2.80E-06	2.65E-06	1.82E-06	2.25E-06
Y61A9LA.10	564	593	529	794	2.80E-06	2.65E-06	1.82E-06	2.25E-06
Y61A9LA.11	64	68	187	93	8.15E-06	7.09E-06	6.29E-06	1.14E-05
Y61A9LA.12	5	6	4	3	1.93E-05	1.92E-05	1.18E-05	2.19E-05
Y61A9LA.3a	152	153	109	199	9.60E-06	9.65E-06	1.83E-05	1.12E-05
Y61A9LA.3b	97	111	69	116	2.80E-06	2.65E-06	1.82E-06	2.25E-06
Y61A9LA.3c	145	151	107	190	7.31E-06	6.96E-06	3.41E-06	7.67E-06
Y61A9LA.4	1	4	0	4	6.58E-06	7.12E-06	3.04E-06	6.32E-06
Y61A9LA.5	6	12	7	6	7.20E-06	7.06E-06	3.46E-06	7.58E-06
Y61A9LA.7	11	12	0	3	2.80E-06	2.65E-06	1.82E-06	2.25E-06
Y61A9LA.8	383	377	479	977	2.80E-06	2.65E-06	1.82E-06	2.25E-06
Y61A9LA.9	5	3	11	6	3.70E-06	3.81E-06	1.82E-06	2.25E-06
Y61B8A.1	3	6	2	2	1.92E-05	1.78E-05	1.56E-05	3.92E-05
Y61B8A.2	5	6	9	3	2.80E-06	2.65E-06	1.82E-06	2.25E-06
Y61B8A.3	20	8	22	10	2.80E-06	2.65E-06	1.82E-06	2.25E-06
Y61B8A.4	5	15	9	8	2.80E-06	2.65E-06	1.82E-06	2.25E-06
Y61B8B.1	3	2	6	4	2.80E-06	2.65E-06	1.82E-06	2.25E-06

Y61B8B.2	1	0	0	2	2.80E-06	2.65E-06	1.82E-06	2.25E-06
Y62E10A.1.1	3184	2794	4384	1442	2.80E-06	2.65E-06	1.82E-06	2.25E-06
Y62E10A.1.2	2319	2077	2420	944	2.80E-06	2.65E-06	1.82E-06	2.25E-06
Y62E10A.10	290	361	473	307	7.80E-04	6.47E-04	6.99E-04	2.84E-04
Y62E10A.12.:	200	238	494	201	5.68E-04	4.81E-04	3.86E-04	1.86E-04
Y62E10A.12.:	152	195	168	126	4.05E-05	4.76E-05	4.30E-05	3.44E-05
Y62E10A.13a	225	424	276	250	5.52E-05	6.20E-05	8.87E-05	4.45E-05
Y62E10A.13b	235	440	303	270	3.99E-05	4.83E-05	2.87E-05	2.65E-05
Y62E10A.13d	256	524	339	304	2.89E-05	5.14E-05	2.31E-05	2.58E-05
Y62E10A.14	541	523	932	790	3.13E-05	5.54E-05	2.63E-05	2.89E-05
Y62E10A.15	241	273	363	369	2.72E-05	5.26E-05	2.34E-05	2.59E-05
Y62E10A.16	28	46	59	73	5.82E-05	5.32E-05	6.53E-05	6.83E-05
Y62E10A.17	68	81	144	127	3.01E-05	3.22E-05	2.95E-05	3.70E-05
Y62E10A.18	14	38	25	21	2.80E-06	3.25E-06	2.88E-06	4.39E-06
Y62E10A.19	24	55	8	15	6.97E-06	7.86E-06	9.60E-06	1.05E-05
Y62E10A.2.1	365	479	495	406	4.12E-06	1.06E-05	4.77E-06	4.95E-06
Y62E10A.2.2	243	339	299	302	4.54E-06	9.79E-06	1.82E-06	2.27E-06
Y62E10A.2.3	283	368	327	331	5.96E-05	7.39E-05	5.26E-05	5.32E-05
Y62E10A.2.4	312	401	400	358	4.09E-05	5.39E-05	3.27E-05	4.08E-05
Y62E10A.20.:	128	232	110	164	6.33E-05	7.77E-05	4.76E-05	5.94E-05
Y62E10A.20.:	47	54	51	62	5.39E-05	6.55E-05	4.50E-05	4.97E-05
Y62E10A.3	1	9	7	0	1.02E-05	1.74E-05	5.69E-06	1.05E-05
Y62E10A.4	9	9	29	7	7.90E-06	8.57E-06	5.58E-06	8.37E-06
Y62E10A.5a.:	64	76	91	91	2.80E-06	2.65E-06	1.82E-06	2.25E-06
Y62E10A.5a.:	72	84	127	102	2.80E-06	2.65E-06	2.26E-06	2.25E-06
Y62E10A.5b.:	58	64	87	88	1.20E-05	1.35E-05	1.12E-05	1.38E-05
Y62E10A.5b.:	63	73	90	91	1.38E-05	1.52E-05	1.59E-05	1.57E-05
Y62E10A.5b.:	72	84	127	102	1.13E-05	1.18E-05	1.10E-05	1.38E-05
Y62E10A.6	198	240	140	220	1.22E-05	1.33E-05	1.13E-05	1.41E-05
Y62E10A.8	123	158	107	127	1.38E-05	1.52E-05	1.59E-05	1.57E-05
Y62E10A.9.1	74	151	53	86	1.50E-05	1.72E-05	6.91E-06	1.34E-05
Y62E10A.9.2	75	155	54	86	1.77E-05	2.15E-05	1.00E-05	1.47E-05
Y62E10A.9.3	70	145	44	83	1.11E-05	2.14E-05	5.17E-06	1.04E-05
Y62F5A.10	33	21	20	12	1.16E-05	2.26E-05	5.41E-06	1.06E-05
Y62F5A.1a	232	201	252	345	1.20E-05	2.35E-05	4.90E-06	1.14E-05
Y62F5A.1b	232	194	246	344	2.80E-06	2.65E-06	1.82E-06	2.25E-06
Y62F5A.9	12	18	10	8	2.59E-05	2.12E-05	1.83E-05	3.10E-05
Y62H9A.1	6	3	7	2	2.62E-05	2.07E-05	1.81E-05	3.12E-05
Y62H9A.10	9	2	8	4	2.80E-06	2.65E-06	1.82E-06	2.25E-06
Y62H9A.11	6	1	9	3	2.80E-06	2.65E-06	1.82E-06	2.25E-06
Y62H9A.12	10	13	5	6	2.80E-06	2.65E-06	1.82E-06	2.25E-06
Y62H9A.13	7	9	4	9	2.80E-06	2.65E-06	1.82E-06	2.25E-06
Y62H9A.14	6	2	3	3	2.80E-06	2.65E-06	1.82E-06	2.25E-06
Y62H9A.2	4	2	5	0	2.80E-06	2.65E-06	1.82E-06	2.25E-06
Y62H9A.3	473	624	612	748	2.80E-06	2.65E-06	1.82E-06	2.25E-06
Y62H9A.4	702	723	1330	1221	2.80E-06	2.65E-06	1.82E-06	2.25E-06
Y62H9A.5	1519	2053	2266	1534	7.96E-05	9.91E-05	6.70E-05	1.01E-04
Y62H9A.6	1590	2297	3606	2457	1.23E-04	1.20E-04	1.52E-04	1.72E-04
Y62H9A.7	2	2	3	0	2.94E-04	3.75E-04	2.85E-04	2.38E-04
Y62H9A.8	3	1	4	2	2.97E-04	4.06E-04	4.39E-04	3.69E-04
Y62H9A.9	3	9	9	4	2.80E-06	2.65E-06	1.82E-06	2.25E-06
Y63D3A.10	13	17	21	7	2.80E-06	2.65E-06	1.82E-06	2.25E-06
Y63D3A.11	4	5	8	3	2.80E-06	2.65E-06	1.82E-06	2.25E-06
Y63D3A.2	5	3	8	4	2.80E-06	2.65E-06	1.84E-06	2.25E-06
Y63D3A.3	3	3	2	7	2.80E-06	2.65E-06	1.82E-06	2.25E-06
Y63D3A.4	108	123	168	222	2.80E-06	2.65E-06	1.82E-06	2.25E-06
Y63D3A.5.1	769	687	1225	1297	2.80E-06	2.65E-06	1.82E-06	2.25E-06
Y63D3A.6a.1	611	726	641	956	9.77E-06	1.05E-05	9.89E-06	1.61E-05
Y63D3A.6a.2	584	712	609	933	4.03E-05	3.40E-05	4.18E-05	5.46E-05

Y63D3A.6b	869	1053	858	1367	3.65E-05	4.10E-05	2.49E-05	4.59E-05
Y63D3A.7.1	165	197	694	159	3.72E-05	4.29E-05	2.53E-05	4.78E-05
Y63D3A.7.2	130	154	514	138	3.68E-05	4.22E-05	2.37E-05	4.66E-05
Y63D3A.7.3	130	157	514	139	5.02E-05	5.66E-05	1.37E-04	3.89E-05
Y63D3A.8	293	212	407	392	3.91E-05	4.38E-05	1.01E-04	3.34E-05
Y63D3A.9	6	2	11	2	3.97E-05	4.53E-05	1.02E-04	3.41E-05
Y64G10A.1	3	6	2	5	2.17E-05	1.48E-05	1.96E-05	2.33E-05
Y64G10A.2	2	2	3	1	2.80E-06	2.65E-06	1.82E-06	2.25E-06
Y64G10A.3	4	0	2	1	2.80E-06	2.65E-06	1.82E-06	2.25E-06
Y64G10A.6	48	71	45	49	2.80E-06	2.65E-06	1.82E-06	2.25E-06
Y64G10A.7	42	76	68	73	2.80E-06	2.65E-06	1.82E-06	2.25E-06
Y64G10A.8	7	10	9	6	2.80E-06	2.65E-06	1.82E-06	2.25E-06
Y64H9A.1	24	22	32	11	2.80E-06	2.65E-06	1.82E-06	2.25E-06
Y64H9A.2	27	50	34	8	2.80E-06	2.65E-06	1.82E-06	2.25E-06
Y65A5A.1	15	24	29	18	1.57E-05	1.36E-05	1.36E-05	5.78E-06
Y65A5A.2	185	121	260	323	5.94E-06	1.04E-05	4.87E-06	2.25E-06
Y65A5A.3	22	15	53	38	3.11E-06	4.71E-06	3.92E-06	2.99E-06
Y65A5A.t1	1	0	3	0	3.04E-05	1.88E-05	2.78E-05	4.27E-05
Y65A5A.t2	1	0	3	0	1.68E-05	1.08E-05	2.63E-05	2.33E-05
Y65B4A.1	167	201	88	161	2.80E-06	2.65E-06	3.04E-06	2.25E-06
Y65B4A.2	30	63	30	23	2.80E-06	2.65E-06	3.04E-06	2.25E-06
Y65B4A.3	257	239	314	209	1.34E-05	1.53E-05	4.61E-06	1.04E-05
Y65B4A.4	55	71	83	99	2.80E-06	4.44E-06	1.82E-06	2.25E-06
Y65B4A.6	219	290	944	918	3.90E-05	3.43E-05	3.10E-05	2.55E-05
Y65B4A.7	7	6	7	3	1.64E-05	2.00E-05	1.61E-05	2.37E-05
Y65B4A.8.1	101	76	156	154	2.02E-05	2.52E-05	5.66E-05	6.79E-05
Y65B4A.8.2	124	92	159	170	2.80E-06	2.65E-06	1.82E-06	2.25E-06
Y65B4A.9	32	30	10	12	6.44E-06	4.58E-06	6.49E-06	7.89E-06
Y65B4BL.1	290	381	117	102	8.51E-06	5.95E-06	7.09E-06	9.36E-06
Y65B4BL.3	74	88	147	113	2.80E-06	2.65E-06	1.82E-06	2.25E-06
Y65B4BL.4	271	188	380	467	2.56E-05	3.18E-05	6.72E-06	7.24E-06
Y65B4BL.5	3082	2739	3074	4821	7.67E-06	8.60E-06	9.89E-06	9.40E-06
Y65B4BL.6	14	22	5	3	3.11E-05	2.04E-05	2.84E-05	4.31E-05
Y65B4BL.7	16	15	38	39	1.29E-04	1.08E-04	8.35E-05	1.62E-04
Y65B4BR.1	20	10	46	9	8.18E-06	1.21E-05	1.90E-06	2.25E-06
Y65B4BR.2	25	47	19	14	2.80E-06	2.65E-06	4.30E-06	5.44E-06
Y65B4BR.3	185	235	319	409	2.80E-06	2.65E-06	2.95E-06	2.25E-06
Y65B4BR.4a	1578	1539	1709	2695	3.25E-06	5.77E-06	1.82E-06	2.25E-06
Y65B4BR.4b	1206	1142	1293	1985	6.27E-06	7.51E-06	7.03E-06	1.11E-05
Y65B4BR.5a.:	4104	3559	5311	4607	5.97E-05	5.50E-05	4.21E-05	8.19E-05
Y65B4BR.5a.:	3631	3130	4689	4308	5.64E-05	5.04E-05	3.93E-05	7.46E-05
Y65B4BR.6a	736	725	248	269	6.64E-04	5.44E-04	5.59E-04	5.99E-04
Y65B4BR.6b	657	682	251	302	5.78E-04	4.71E-04	4.86E-04	5.51E-04
Y65B4BR.8	167	175	255	280	5.51E-05	5.13E-05	1.21E-05	1.62E-05
Y65B4BR.9	2	8	3	3	5.40E-05	5.30E-05	1.34E-05	1.99E-05
Y66A7A.1	0	2	16	2	2.80E-05	2.77E-05	2.78E-05	3.76E-05
Y66A7A.2	48	68	51	67	2.80E-06	2.65E-06	1.82E-06	2.25E-06
Y66A7A.3	2	3	5	9	2.80E-06	2.65E-06	1.82E-06	2.25E-06
Y66A7A.4	10	18	8	11	1.13E-05	1.51E-05	7.80E-06	1.26E-05
Y66A7A.5	111	139	190	186	2.80E-06	2.65E-06	1.82E-06	2.81E-06
Y66A7A.6.1	342	524	254	168	2.80E-06	2.65E-06	1.82E-06	2.25E-06
Y66A7A.6.2	329	494	222	161	5.82E-06	6.88E-06	6.49E-06	7.83E-06
Y66A7A.6.3	318	471	215	161	2.78E-05	4.03E-05	1.34E-05	1.10E-05
Y66A7A.7	16	27	54	11	2.87E-05	4.07E-05	1.26E-05	1.13E-05
Y66A7A.8	47	48	63	58	2.81E-05	3.93E-05	1.24E-05	1.14E-05
Y66C5A.1	12	14	6	4	5.07E-06	8.07E-06	1.11E-05	2.79E-06
Y66C5A.2	0	2	0	0	3.44E-06	3.33E-06	3.01E-06	3.42E-06
Y66D12A.1	4	2	6	4	6.05E-06	6.67E-06	1.97E-06	2.25E-06
Y66D12A.10	111	120	265	186	2.80E-06	2.65E-06	1.82E-06	2.25E-06

Y66D12A.11	10	24	7	10	2.80E-06	2.65E-06	1.82E-06	2.25E-06
Y66D12A.12	44	49	125	81	1.46E-05	1.49E-05	2.27E-05	1.97E-05
Y66D12A.13	32	41	21	30	2.80E-06	2.65E-06	1.82E-06	2.25E-06
Y66D12A.14	34	37	41	31	4.68E-06	4.92E-06	8.66E-06	6.93E-06
Y66D12A.15	427	406	505	774	2.80E-06	2.67E-06	1.82E-06	2.25E-06
Y66D12A.17	309	363	501	687	2.80E-06	2.65E-06	1.82E-06	2.25E-06
Y66D12A.18	29	30	56	73	1.85E-05	1.66E-05	1.42E-05	2.69E-05
Y66D12A.19	15	25	43	79	1.35E-05	1.49E-05	1.42E-05	2.40E-05
Y66D12A.20	19	57	22	21	3.78E-06	3.68E-06	4.74E-06	7.62E-06
Y66D12A.21	118	207	172	167	3.30E-06	5.18E-06	6.14E-06	1.39E-05
Y66D12A.22.	177	210	218	99	2.80E-06	5.08E-06	1.82E-06	2.25E-06
Y66D12A.22.	124	169	131	69	1.32E-05	2.19E-05	1.25E-05	1.50E-05
Y66D12A.23	73	105	73	141	5.08E-05	5.70E-05	4.07E-05	2.28E-05
Y66D12A.24	22	59	52	38	3.33E-05	4.29E-05	2.29E-05	1.49E-05
Y66D12A.25	3	2	4	0	5.96E-06	8.09E-06	3.88E-06	9.22E-06
Y66D12A.3	8	27	9	8	2.80E-06	6.64E-06	4.03E-06	3.64E-06
Y66D12A.5	289	221	366	534	2.80E-06	2.65E-06	1.82E-06	2.25E-06
Y66D12A.6	38	45	122	68	2.80E-06	2.65E-06	1.82E-06	2.25E-06
Y66D12A.7	22	30	41	31	1.47E-05	1.06E-05	1.21E-05	2.19E-05
Y66D12A.8	224	190	241	235	1.04E-05	1.16E-05	2.16E-05	1.49E-05
Y66D12A.9	262	305	317	262	4.62E-06	5.95E-06	5.61E-06	5.24E-06
Y66H1A.5	58	113	103	100	3.12E-05	2.50E-05	2.18E-05	2.63E-05
Y66H1A.6a	190	318	142	286	3.42E-05	3.76E-05	2.69E-05	2.74E-05
Y66H1A.6b	184	299	143	278	9.35E-06	1.72E-05	1.08E-05	1.30E-05
Y66H1B.2a	3759	3741	2827	5089	5.71E-06	9.05E-06	2.79E-06	6.90E-06
Y66H1B.2c	3880	3847	2926	5254	5.52E-06	8.46E-06	2.79E-06	6.70E-06
Y66H1B.3.1	1231	1105	1274	2272	1.56E-04	1.47E-04	7.65E-05	1.70E-04
Y66H1B.3.2	1208	1078	1242	2247	1.52E-04	1.43E-04	7.47E-05	1.66E-04
Y66H1B.4	452	634	532	661	4.08E-05	3.46E-05	2.75E-05	6.05E-05
Y66H1B.5	827	790	770	1413	4.09E-05	3.45E-05	2.74E-05	6.12E-05
Y67A10A.1	10	24	16	18	2.84E-05	3.76E-05	2.17E-05	3.34E-05
Y67A10A.10	23	30	29	20	7.03E-05	6.35E-05	4.26E-05	9.65E-05
Y67A10A.2	4	3	2	5	2.80E-06	2.65E-06	1.82E-06	2.25E-06
Y67A10A.3	34	45	16	21	4.51E-06	5.55E-06	3.70E-06	3.15E-06
Y67A10A.4	22	35	34	41	2.80E-06	2.65E-06	1.82E-06	2.25E-06
Y67A10A.5	3	3	5	2	2.80E-06	2.65E-06	1.82E-06	2.25E-06
Y67A10A.6	9	15	5	9	2.80E-06	3.97E-06	2.66E-06	3.96E-06
Y67A10A.7	5	14	9	14	2.80E-06	2.65E-06	1.82E-06	2.25E-06
Y67A10A.8	39	51	33	26	2.80E-06	2.65E-06	1.82E-06	2.25E-06
Y67A10A.9	56	115	51	110	2.80E-06	2.65E-06	1.82E-06	2.25E-06
Y67A6A.1	32	34	11	3	4.42E-06	5.48E-06	2.44E-06	2.36E-06
Y67A6A.2	25	34	34	42	2.80E-06	5.03E-06	1.82E-06	4.09E-06
Y67D2.1a.1	122	143	156	224	9.63E-06	9.68E-06	2.15E-06	2.25E-06
Y67D2.1a.2	106	134	134	201	2.80E-06	2.65E-06	1.82E-06	2.27E-06
Y67D2.1b.1	94	110	114	170	8.29E-06	9.18E-06	6.89E-06	1.22E-05
Y67D2.1b.2	80	95	95	137	7.70E-06	9.20E-06	6.34E-06	1.17E-05
Y67D2.2	339	290	303	406	8.06E-06	8.91E-06	6.36E-06	1.17E-05
Y67D2.3	314	378	606	352	8.37E-06	9.39E-06	6.47E-06	1.15E-05
Y67D2.4	82	83	79	110	2.14E-05	1.73E-05	1.25E-05	2.06E-05
Y67D2.5	44	61	40	43	5.75E-05	6.54E-05	7.22E-05	5.17E-05
Y67D2.6	134	153	229	285	9.74E-06	9.31E-06	6.10E-06	1.05E-05
Y67D2.7	228	207	397	378	8.09E-06	1.06E-05	4.79E-06	6.34E-06
Y67D2.t3	0	1	0	0	6.52E-06	7.06E-06	7.27E-06	1.12E-05
Y67D8A.1.1	458	453	530	903	1.91E-05	1.64E-05	2.17E-05	2.55E-05
Y67D8A.1.2	462	451	554	903	2.80E-06	2.65E-06	1.82E-06	2.25E-06
Y67D8A.2a.2	63	70	76	147	1.57E-05	1.46E-05	1.18E-05	2.48E-05
Y67D8A.2b.1	57	70	66	126	1.41E-05	1.30E-05	1.10E-05	2.22E-05
Y67D8A.2b.2	62	70	75	142	9.04E-06	9.50E-06	7.11E-06	1.70E-05
Y67D8A.2c.2	82	87	98	189	8.82E-06	1.02E-05	6.65E-06	1.57E-05

Y67D8A.3	65	53	98	81	9.55E-06	1.02E-05	7.51E-06	1.75E-05
Y67D8B.1	13	10	14	5	1.24E-05	1.24E-05	9.66E-06	2.30E-05
Y67D8B.2	4	4	4	3	9.52E-06	7.33E-06	9.33E-06	9.54E-06
Y67D8B.3	11	5	5	1	2.80E-06	2.65E-06	1.82E-06	2.25E-06
Y67D8B.4	24	19	68	26	2.80E-06	2.65E-06	1.82E-06	2.25E-06
Y67D8B.5	12	11	5	4	2.88E-06	2.65E-06	1.82E-06	2.25E-06
Y67D8C.1	2	7	7	3	3.81E-06	2.86E-06	7.03E-06	3.33E-06
Y67D8C.10a	2362	2271	1969	3274	2.80E-06	2.65E-06	1.82E-06	2.25E-06
Y67D8C.10b	2615	2468	2157	3528	2.80E-06	3.17E-06	2.19E-06	2.25E-06
Y67D8C.10c	2623	2570	2214	3873	7.51E-05	6.82E-05	4.07E-05	8.36E-05
Y67D8C.12	67	63	389	408	6.74E-05	6.01E-05	3.62E-05	7.30E-05
Y67D8C.2	7	5	4	2	7.15E-05	6.62E-05	3.93E-05	8.48E-05
Y67D8C.3a	282	261	266	413	1.43E-05	1.27E-05	5.40E-05	6.99E-05
Y67D8C.3b.1	259	239	233	388	2.80E-06	2.65E-06	1.82E-06	2.25E-06
Y67D8C.3b.2	272	249	247	395	1.52E-05	1.33E-05	9.33E-06	1.79E-05
Y67D8C.4	12	7	5	2	1.57E-05	1.37E-05	9.20E-06	1.89E-05
Y67D8C.5	4552	3960	2251	6084	1.53E-05	1.33E-05	9.04E-06	1.79E-05
Y67D8C.6	5	7	18	9	2.80E-06	2.65E-06	1.82E-06	2.25E-06
Y67D8C.7	2	1	1	1	3.92E-05	3.22E-05	1.26E-05	4.21E-05
Y67D8C.8	756	603	2343	577	2.80E-06	2.65E-06	1.82E-06	2.25E-06
Y67D8C.9	34	44	38	55	2.80E-06	2.65E-06	1.82E-06	2.25E-06
Y67H2A.1	373	393	494	728	2.38E-04	1.79E-04	4.80E-04	1.46E-04
Y67H2A.10	687	638	846	1128	2.80E-06	2.65E-06	1.82E-06	2.25E-06
Y67H2A.2a	295	249	472	415	1.79E-05	1.79E-05	1.55E-05	2.81E-05
Y67H2A.2b	160	137	321	182	3.92E-05	3.44E-05	3.14E-05	5.17E-05
Y67H2A.3	6	7	18	7	1.94E-05	1.55E-05	2.02E-05	2.19E-05
Y67H2A.4a	249	305	313	394	2.55E-05	2.07E-05	3.33E-05	2.33E-05
Y67H2A.4b	128	113	225	207	2.80E-06	2.65E-06	1.82E-06	2.25E-06
Y67H2A.5.1	1700	1623	3799	1469	1.60E-05	1.86E-05	1.31E-05	2.04E-05
Y67H2A.5.2	1623	1601	3164	1310	1.44E-05	1.20E-05	1.65E-05	1.87E-05
Y67H2A.6	331	348	492	593	3.48E-04	3.14E-04	5.06E-04	2.42E-04
Y67H2A.7	163	214	195	300	3.33E-04	3.10E-04	4.22E-04	2.16E-04
Y67H2A.8.1	1920	2975	3862	3565	2.64E-05	2.63E-05	2.56E-05	3.81E-05
Y67H2A.8.2	1766	2665	3463	3335	8.37E-06	1.04E-05	6.52E-06	1.24E-05
Y67H2A.9	30	79	36	35	1.55E-04	2.26E-04	2.03E-04	2.31E-04
Y68A4A.1	5	4	9	2	1.43E-04	2.04E-04	1.83E-04	2.17E-04
Y68A4A.10a	2	3	1	3	2.86E-06	7.09E-06	2.22E-06	2.68E-06
Y68A4A.10b	1	2	1	3	2.80E-06	2.65E-06	1.82E-06	2.25E-06
Y68A4A.11	4	10	9	9	2.80E-06	2.65E-06	1.82E-06	2.25E-06
Y68A4A.12	21	27	30	18	2.80E-06	2.65E-06	1.82E-06	2.25E-06
Y68A4A.13	16	21	61	10	2.80E-06	2.65E-06	1.82E-06	2.25E-06
Y68A4A.2	9	10	20	6	2.80E-06	3.20E-06	2.46E-06	2.25E-06
Y68A4A.3	2	2	8	5	1.05E-05	1.30E-05	2.60E-05	5.26E-06
Y68A4A.4	16	50	38	65	2.80E-06	2.65E-06	1.82E-06	2.25E-06
Y68A4A.5	5	7	3	1	2.80E-06	2.65E-06	1.82E-06	2.25E-06
Y68A4A.6	11	13	12	13	2.80E-06	5.18E-06	2.71E-06	5.74E-06
Y68A4A.7	4	4	8	8	2.80E-06	2.65E-06	1.82E-06	2.25E-06
Y68A4A.8	10	4	15	6	2.80E-06	2.65E-06	1.82E-06	2.25E-06
Y68A4A.9	4	2	2	1	2.80E-06	2.65E-06	1.82E-06	2.25E-06
Y68A4B.1	2	0	0	0	2.80E-06	2.65E-06	1.82E-06	2.25E-06
Y68A4B.2	6	6	15	2	2.80E-06	2.65E-06	1.82E-06	2.25E-06
Y68A4B.3	1	1	4	1	2.80E-06	2.65E-06	1.82E-06	2.25E-06
Y69A2AL.1	8	8	7	1	2.80E-06	2.65E-06	1.82E-06	2.25E-06
Y69A2AL.2	63	90	116	83	2.80E-06	2.65E-06	1.82E-06	2.25E-06
Y69A2AR.11	6	5	3	4	2.80E-06	2.65E-06	1.82E-06	2.25E-06
Y69A2AR.12	18	7	6	4	1.49E-05	2.01E-05	1.78E-05	1.57E-05
Y69A2AR.13	22	10	14	10	2.80E-06	2.65E-06	1.82E-06	2.25E-06
Y69A2AR.14	29	27	15	14	3.53E-06	2.65E-06	1.82E-06	2.25E-06
Y69A2AR.15	3	14	11	8	3.95E-06	2.65E-06	1.82E-06	2.25E-06

Y69A2AR.16	422	448	640	991	2.80E-06	2.65E-06	1.82E-06	2.25E-06
Y69A2AR.17	64	66	60	87	2.80E-06	2.65E-06	1.82E-06	2.25E-06
Y69A2AR.18a	4045	4079	3992	4311	1.58E-05	1.59E-05	1.56E-05	2.99E-05
Y69A2AR.18a	4094	4148	4096	4428	1.25E-05	1.22E-05	7.63E-06	1.37E-05
Y69A2AR.18a	3191	3216	3032	3581	3.86E-04	3.68E-04	2.48E-04	3.31E-04
Y69A2AR.18b	3135	3160	3178	3440	5.07E-04	4.85E-04	3.30E-04	4.41E-04
Y69A2AR.18b	3167	3194	3215	3470	3.94E-04	3.75E-04	2.44E-04	3.55E-04
Y69A2AR.18c	3401	3414	3323	3654	3.71E-04	3.53E-04	2.45E-04	3.27E-04
Y69A2AR.18c	1805	1782	1718	1946	3.74E-04	3.56E-04	2.47E-04	3.29E-04
Y69A2AR.19	127	203	94	78	3.88E-04	3.68E-04	2.47E-04	3.35E-04
Y69A2AR.1a	267	265	506	662	3.31E-04	3.09E-04	2.05E-04	2.87E-04
Y69A2AR.1c	263	261	481	643	3.70E-06	5.58E-06	1.82E-06	2.25E-06
Y69A2AR.1d	296	281	575	693	2.04E-05	1.91E-05	2.51E-05	4.05E-05
Y69A2AR.21	206	215	255	215	2.01E-05	1.88E-05	2.39E-05	3.94E-05
Y69A2AR.22	36	41	46	26	1.83E-05	1.64E-05	2.31E-05	3.44E-05
Y69A2AR.23	0	6	0	3	4.44E-05	4.37E-05	3.58E-05	3.72E-05
Y69A2AR.24	2	5	1	3	6.61E-06	7.12E-06	5.50E-06	3.85E-06
Y69A2AR.25	30	12	22	13	2.80E-06	2.65E-06	1.82E-06	2.25E-06
Y69A2AR.26	3	1	5	1	2.80E-06	2.65E-06	1.82E-06	2.25E-06
Y69A2AR.27	14	17	4	6	3.30E-06	2.65E-06	1.82E-06	2.25E-06
Y69A2AR.28	119	114	397	130	2.80E-06	2.65E-06	1.82E-06	2.25E-06
Y69A2AR.29	4	3	6	6	3.98E-06	4.58E-06	1.82E-06	2.25E-06
Y69A2AR.2a	207	196	423	483	3.09E-05	2.79E-05	6.70E-05	2.71E-05
Y69A2AR.2b	38	31	61	70	2.80E-06	2.65E-06	1.82E-06	2.25E-06
Y69A2AR.3.1	207	230	608	167	1.00E-05	8.97E-06	1.33E-05	1.88E-05
Y69A2AR.3.2	166	198	442	131	8.04E-06	6.19E-06	8.38E-06	1.19E-05
Y69A2AR.30a	277	322	399	410	6.62E-05	6.95E-05	1.27E-04	4.29E-05
Y69A2AR.30a	249	267	358	348	5.30E-05	5.97E-05	9.18E-05	3.36E-05
Y69A2AR.31	66	103	84	91	3.33E-05	3.66E-05	3.13E-05	3.97E-05
Y69A2AR.32a	101	82	114	120	3.54E-05	3.58E-05	3.31E-05	3.97E-05
Y69A2AR.32b	90	69	107	110	2.80E-06	2.88E-06	1.82E-06	2.25E-06
Y69A2AR.32b	79	63	93	100	9.18E-06	7.04E-06	6.74E-06	8.77E-06
Y69A2AR.4	42	48	57	43	1.25E-05	9.02E-06	9.64E-06	1.22E-05
Y69A2AR.5	17	31	22	13	1.12E-05	8.44E-06	8.58E-06	1.14E-05
Y69A2AR.6	101	118	125	113	2.80E-06	2.65E-06	2.00E-06	2.25E-06
Y69A2AR.7a	88	188	111	188	2.80E-06	3.07E-06	1.82E-06	2.25E-06
Y69A2AR.7b.	85	179	113	208	1.38E-05	1.53E-05	1.12E-05	1.24E-05
Y69A2AR.7b.	85	179	113	205	8.54E-06	1.72E-05	7.00E-06	1.46E-05
Y69A2AR.7b.	57	116	70	103	7.56E-06	1.50E-05	6.52E-06	1.48E-05
Y69A2AR.7c	57	116	70	103	7.62E-06	1.52E-05	6.60E-06	1.48E-05
Y69A2AR.8	16	15	2	3	8.06E-06	1.55E-05	6.45E-06	1.17E-05
Y69A2AR.9	8	27	7	5	8.06E-06	1.55E-05	6.45E-06	1.17E-05
Y69E1A.1	142	286	137	91	4.93E-06	4.36E-06	1.82E-06	2.25E-06
Y69E1A.2	282	428	243	122	2.80E-06	3.17E-06	1.82E-06	2.25E-06
Y69E1A.3	21	39	26	21	1.49E-05	2.83E-05	9.35E-06	7.67E-06
Y69E1A.4	6	31	16	6	2.59E-05	3.72E-05	1.45E-05	9.02E-06
Y69E1A.5	125	174	186	116	2.80E-06	2.65E-06	1.82E-06	2.25E-06
Y69E1A.6	11	7	11	9	2.80E-06	2.91E-06	1.82E-06	2.25E-06
Y69E1A.7	3	12	10	7	2.70E-05	3.55E-05	2.61E-05	2.01E-05
Y69E1A.8	23	33	35	14	2.80E-06	2.65E-06	1.82E-06	2.25E-06
Y69F12A.1	17	27	16	10	2.80E-06	2.65E-06	1.82E-06	2.25E-06
Y69F12A.2a.1	491	462	543	350	2.80E-06	3.20E-06	2.33E-06	2.25E-06
Y69F12A.2a.2	455	436	488	334	2.80E-06	2.65E-06	1.82E-06	2.25E-06
Y69F12A.2b	461	438	490	340	3.34E-05	2.97E-05	2.41E-05	1.91E-05
Y69F12A.3	0	1	0	0	3.38E-05	3.06E-05	2.36E-05	1.99E-05
Y69H2.1	21	38	27	24	3.13E-05	2.81E-05	2.16E-05	1.85E-05
Y69H2.10a	7	11	6	7	2.80E-06	2.65E-06	1.82E-06	2.25E-06
Y69H2.10b	8	12	4	6	2.80E-06	3.81E-06	1.86E-06	2.25E-06
Y69H2.11	6	8	8	6	2.80E-06	2.65E-06	1.82E-06	2.25E-06

Y69H2.12	4	3	5	2	2.80E-06	2.65E-06	1.82E-06	2.25E-06
Y69H2.13	4	5	3	2	2.80E-06	2.65E-06	1.82E-06	2.25E-06
Y69H2.14.1	1117	1837	2788	2389	2.80E-06	2.65E-06	1.82E-06	2.25E-06
Y69H2.14.2	1056	1742	2468	2264	2.80E-06	2.65E-06	1.82E-06	2.25E-06
Y69H2.2	19	19	29	37	1.19E-04	1.85E-04	1.93E-04	2.04E-04
Y69H2.3a.1	458	659	473	1540	1.13E-04	1.76E-04	1.72E-04	1.95E-04
Y69H2.3a.2	455	653	470	1538	2.80E-06	2.65E-06	1.82E-06	2.25E-06
Y69H2.3b	270	472	307	857	3.14E-05	4.26E-05	2.11E-05	8.47E-05
Y69H2.3c	249	423	273	751	3.12E-05	4.23E-05	2.10E-05	8.47E-05
Y69H2.3d.1	431	573	433	1442	1.21E-05	2.00E-05	8.96E-06	3.09E-05
Y69H2.3d.2	388	527	392	1291	1.26E-05	2.02E-05	8.98E-06	3.05E-05
Y69H2.6	30	35	26	18	3.38E-05	4.25E-05	2.21E-05	9.08E-05
Y69H2.7	178	168	271	323	3.30E-05	4.23E-05	2.17E-05	8.80E-05
Y69H2.8	1	5	3	4	3.84E-06	4.21E-06	2.15E-06	2.25E-06
Y69H2.9	16	16	36	16	1.50E-05	1.33E-05	1.48E-05	2.18E-05
Y6B3A.1a	808	808	911	1246	2.80E-06	2.65E-06	1.82E-06	2.25E-06
Y6B3A.1b	209	185	221	347	2.80E-06	2.65E-06	2.77E-06	2.25E-06
Y6B3B.1	31	50	29	28	1.72E-05	1.63E-05	1.26E-05	2.14E-05
Y6B3B.10	15	18	16	9	1.39E-05	1.16E-05	9.57E-06	1.86E-05
Y6B3B.11	5	3	2	4	2.80E-06	2.65E-06	1.82E-06	2.25E-06
Y6B3B.12	58	69	101	153	2.80E-06	2.65E-06	1.82E-06	2.25E-06
Y6B3B.2	8	8	16	19	2.80E-06	2.65E-06	1.82E-06	2.25E-06
Y6B3B.3	23	12	13	19	6.55E-06	7.38E-06	7.43E-06	1.39E-05
Y6B3B.4	102	95	209	337	2.80E-06	2.65E-06	1.82E-06	2.25E-06
Y6B3B.5a	25	43	23	40	2.80E-06	2.65E-06	1.82E-06	2.25E-06
Y6B3B.5b	39	52	39	64	6.78E-06	5.95E-06	9.04E-06	1.80E-05
Y6B3B.7	2	4	5	4	2.80E-06	4.15E-06	1.82E-06	3.28E-06
Y6B3B.9	110	136	106	144	3.84E-06	4.81E-06	2.50E-06	5.04E-06
Y6D11A.2	282	431	345	379	2.80E-06	2.65E-06	1.82E-06	2.25E-06
Y6D1A.1	226	223	262	360	7.20E-06	8.41E-06	4.52E-06	7.58E-06
Y6D1A.2	10	22	15	9	3.13E-05	4.52E-05	2.49E-05	3.38E-05
Y6E2A.1	16	16	57	22	1.23E-05	1.14E-05	9.26E-06	1.57E-05
Y6E2A.2	2	10	8	1	2.80E-06	2.65E-06	1.82E-06	2.25E-06
Y6E2A.4	8	4	5	6	2.80E-06	2.65E-06	4.50E-06	2.25E-06
Y6E2A.5	9	13	7	8	2.80E-06	2.65E-06	1.82E-06	2.25E-06
Y6E2A.6	5	5	6	5	2.80E-06	2.65E-06	1.82E-06	2.25E-06
Y6E2A.7	6	8	2	4	2.80E-06	2.65E-06	1.82E-06	2.25E-06
Y6E2A.8	13	19	8	4	2.80E-06	2.65E-06	1.82E-06	2.25E-06
Y6E2A.9a	4	10	4	3	2.80E-06	2.65E-06	1.82E-06	2.25E-06
Y6E2A.9b	4	8	2	2	2.80E-06	3.04E-06	1.82E-06	2.25E-06
Y6G8.1	1	3	2	7	2.80E-06	2.65E-06	1.82E-06	2.25E-06
Y6G8.2	24	32	16	37	2.80E-06	2.65E-06	1.82E-06	2.25E-06
Y6G8.3	41	79	85	89	2.80E-06	2.65E-06	1.82E-06	2.25E-06
Y6G8.4	10	5	7	1	2.80E-06	2.65E-06	1.82E-06	2.25E-06
Y6G8.5	5	4	9	7	5.15E-06	9.39E-06	6.96E-06	9.00E-06
Y6G8.6	2	2	5	0	2.80E-06	2.65E-06	1.82E-06	2.25E-06
Y70C5A.2	29	38	38	34	2.80E-06	2.65E-06	1.82E-06	2.25E-06
Y70C5A.t1	1	0	3	0	2.80E-06	2.65E-06	1.82E-06	2.25E-06
Y70C5B.1	1	2	7	2	2.80E-06	2.65E-06	1.82E-06	2.25E-06
Y70C5C.1	31	21	23	19	2.80E-06	2.65E-06	2.99E-06	2.25E-06
Y70C5C.2	3	5	2	0	2.80E-06	2.65E-06	1.82E-06	2.25E-06
Y70C5C.3	11	8	6	4	2.80E-06	2.65E-06	1.82E-06	2.25E-06
Y70C5C.4	3	4	7	3	2.80E-06	2.65E-06	1.82E-06	2.25E-06
Y70C5C.5	9	31	9	4	2.80E-06	2.65E-06	1.82E-06	2.25E-06
Y70C5C.6a	4	13	8	2	2.80E-06	2.65E-06	1.82E-06	2.25E-06
Y70C5C.6b	5	13	7	2	2.80E-06	2.65E-06	1.82E-06	2.25E-06
Y70C5C.7	1	6	2	4	2.80E-06	2.65E-06	1.82E-06	2.25E-06
Y70D2A.1	14	22	9	7	2.80E-06	2.65E-06	1.82E-06	2.25E-06
Y70D2A.2	35	93	28	61	2.80E-06	2.65E-06	1.82E-06	2.25E-06

Y70G10A.1	6	6	3	2	2.80E-06	2.65E-06	1.82E-06	2.25E-06
Y70G10A.2	13	13	9	7	2.80E-06	5.87E-06	1.82E-06	3.26E-06
Y70G10A.3	18	71	33	52	2.80E-06	2.65E-06	1.82E-06	2.25E-06
Y71A12B.10	20	34	31	49	2.80E-06	2.65E-06	1.82E-06	2.25E-06
Y71A12B.11	29	27	20	21	2.80E-06	3.62E-06	1.82E-06	2.25E-06
Y71A12B.12a	49	60	81	118	2.80E-06	2.65E-06	1.82E-06	3.22E-06
Y71A12B.12a	45	60	78	116	3.42E-06	2.99E-06	1.82E-06	2.25E-06
Y71A12B.12b	36	41	70	98	2.80E-06	2.75E-06	2.55E-06	4.59E-06
Y71A12B.13a	7	7	11	16	2.80E-06	2.78E-06	2.48E-06	4.57E-06
Y71A12B.13b	4	4	5	9	2.80E-06	2.65E-06	2.90E-06	5.02E-06
Y71A12B.14	23	21	47	46	2.80E-06	2.65E-06	1.82E-06	2.34E-06
Y71A12B.15	6	6	7	7	2.80E-06	2.65E-06	1.82E-06	3.51E-06
Y71A12B.2	11	6	5	7	3.33E-06	2.86E-06	4.41E-06	5.33E-06
Y71A12B.3	15	9	9	12	2.80E-06	2.65E-06	1.82E-06	2.25E-06
Y71A12B.4	24	30	170	36	2.80E-06	2.65E-06	1.82E-06	2.25E-06
Y71A12B.5	2	1	2	0	2.80E-06	2.65E-06	1.82E-06	2.25E-06
Y71A12B.6	1	0	6	0	2.80E-06	2.65E-06	2.15E-06	2.25E-06
Y71A12B.8	14	7	30	8	2.80E-06	2.65E-06	1.82E-06	2.25E-06
Y71A12B.9	79	85	141	178	2.80E-06	2.65E-06	1.82E-06	2.25E-06
Y71A12C.1	3	6	2	2	2.80E-06	2.65E-06	1.82E-06	2.25E-06
Y71A12C.2	9	14	3	5	4.42E-06	4.50E-06	5.16E-06	8.03E-06
Y71D11A.1	371	421	176	124	2.80E-06	2.65E-06	1.82E-06	2.25E-06
Y71D11A.2b	70	72	97	73	2.80E-06	2.65E-06	1.82E-06	2.25E-06
Y71D11A.3a	23	54	35	32	4.73E-06	5.08E-06	1.82E-06	2.25E-06
Y71D11A.3b	19	34	15	28	2.06E-05	2.00E-05	1.85E-05	1.72E-05
Y71D11A.5	14	31	32	20	2.80E-06	5.00E-06	2.22E-06	2.52E-06
Y71F9AL.1	159	167	204	251	2.80E-06	2.99E-06	1.82E-06	2.25E-06
Y71F9AL.10	133	142	176	224	2.80E-06	2.65E-06	1.82E-06	2.25E-06
Y71F9AL.11	29	23	58	68	1.24E-05	1.23E-05	1.04E-05	1.57E-05
Y71F9AL.12	195	179	304	385	2.07E-05	2.08E-05	1.78E-05	2.80E-05
Y71F9AL.13a	9310	8191	16149	9101	5.49E-06	4.13E-06	7.16E-06	1.03E-05
Y71F9AL.13a	8140	7174	13466	8435	2.12E-05	1.84E-05	2.15E-05	3.37E-05
Y71F9AL.13b	6991	6557	11059	7821	1.49E-03	1.24E-03	1.68E-03	1.17E-03
Y71F9AL.13b	6893	6466	10592	7602	1.29E-03	1.07E-03	1.38E-03	1.07E-03
Y71F9AL.13b	6883	6462	10228	7543	1.24E-03	1.10E-03	1.28E-03	1.12E-03
Y71F9AL.13b	5134	5028	7382	6438	9.79E-04	8.67E-04	9.78E-04	8.67E-04
Y71F9AL.13b	4156	4312	6884	5874	1.45E-03	1.29E-03	1.40E-03	1.28E-03
Y71F9AL.13b	5040	4935	7248	6334	7.43E-04	6.87E-04	6.95E-04	7.48E-04
Y71F9AL.13b	8140	7174	13466	8435	9.38E-04	9.20E-04	1.01E-03	1.07E-03
Y71F9AL.13b	6916	6470	10680	7658	8.59E-04	7.95E-04	8.04E-04	8.67E-04
Y71F9AL.14.1	99	129	97	109	1.39E-03	1.15E-03	1.49E-03	1.15E-03
Y71F9AL.14.2	105	145	115	115	1.18E-03	1.04E-03	1.19E-03	1.05E-03
Y71F9AL.16	593	557	599	888	1.99E-05	2.45E-05	1.27E-05	1.76E-05
Y71F9AL.17	2036	2128	1870	3011	2.07E-05	2.70E-05	1.47E-05	1.82E-05
Y71F9AL.18.1	1110	843	1136	1766	4.15E-05	3.68E-05	2.73E-05	4.99E-05
Y71F9AL.18.2	1098	835	1102	1749	5.71E-05	5.64E-05	3.41E-05	6.79E-05
Y71F9AL.2	19	39	14	14	4.15E-05	2.98E-05	2.77E-05	5.31E-05
Y71F9AL.4	29	80	39	41	4.12E-05	2.96E-05	2.69E-05	5.27E-05
Y71F9AL.5	71	61	86	48	2.80E-06	2.70E-06	1.82E-06	2.25E-06
Y71F9AL.6	4	5	2	5	2.80E-06	4.92E-06	1.82E-06	2.25E-06
Y71F9AL.7	7	8	19	6	1.23E-05	1.00E-05	9.71E-06	6.70E-06
Y71F9AL.8	115	111	123	171	2.80E-06	2.65E-06	1.82E-06	2.25E-06
Y71F9AL.9.1	2944	2475	2725	4276	2.80E-06	2.65E-06	1.82E-06	2.25E-06
Y71F9AL.9.2	1581	1338	1342	2000	1.28E-05	1.17E-05	8.91E-06	1.53E-05
Y71F9AM.4a	265	350	429	509	2.31E-04	1.84E-04	1.39E-04	2.70E-04
Y71F9AM.4b	175	240	288	341	1.87E-04	1.49E-04	1.03E-04	1.90E-04
Y71F9AM.6.1	2710	2528	1615	2216	1.14E-05	1.42E-05	1.20E-05	1.76E-05
Y71F9AM.6.2	2834	2631	1751	2291	1.21E-05	1.57E-05	1.30E-05	1.89E-05
Y71F9AM.6.3	2081	1903	1137	1745	2.80E-04	2.47E-04	1.09E-04	1.84E-04

Y71F9AR.1	151	213	132	201	2.22E-04	1.95E-04	8.93E-05	1.44E-04
Y71F9AR.2	10	10	7	9	3.00E-04	2.59E-04	1.07E-04	2.02E-04
Y71F9AR.3	177	186	261	447	4.98E-06	6.64E-06	2.82E-06	5.33E-06
Y71F9B.1	19	52	148	40	2.80E-06	2.65E-06	1.82E-06	2.25E-06
Y71F9B.10a.1	582	614	907	1102	8.85E-06	8.78E-06	8.49E-06	1.79E-05
Y71F9B.10a.2	562	586	871	1080	5.07E-06	1.31E-05	2.57E-05	8.57E-06
Y71F9B.10b	308	337	408	523	1.42E-05	1.41E-05	1.44E-05	2.16E-05
Y71F9B.13a	42	65	69	77	1.42E-05	1.40E-05	1.43E-05	2.19E-05
Y71F9B.13b	31	55	57	65	1.94E-05	2.00E-05	1.67E-05	2.65E-05
Y71F9B.14	7	9	12	6	7.08E-06	1.04E-05	7.58E-06	1.05E-05
Y71F9B.15	2	7	3	2	5.80E-06	9.71E-06	6.92E-06	9.74E-06
Y71F9B.16	150	178	213	186	2.80E-06	2.65E-06	1.82E-06	2.25E-06
Y71F9B.2	126	174	177	164	2.80E-06	2.65E-06	1.82E-06	2.25E-06
Y71F9B.3.1	1270	1049	1517	1916	2.23E-05	2.49E-05	2.06E-05	2.22E-05
Y71F9B.3.2	927	756	1052	1424	1.46E-05	1.90E-05	1.34E-05	1.53E-05
Y71F9B.4	477	470	680	219	1.85E-04	1.45E-04	1.44E-04	2.24E-04
Y71F9B.5a	73	91	59	83	1.75E-04	1.34E-04	1.29E-04	2.15E-04
Y71F9B.5b.1	75	91	57	83	1.11E-04	1.03E-04	1.03E-04	4.10E-05
Y71F9B.5b.2	58	80	48	78	4.31E-06	5.05E-06	2.26E-06	3.94E-06
Y71F9B.6	65	67	126	206	4.40E-06	5.03E-06	2.17E-06	3.89E-06
Y71F9B.8	70	82	66	97	3.81E-06	4.97E-06	2.06E-06	4.12E-06
Y71G10AL.1a	285	238	579	844	4.54E-06	4.42E-06	5.72E-06	1.15E-05
Y71G10AL.1b	253	204	506	794	4.26E-06	4.71E-06	2.61E-06	4.75E-06
Y71G10AR.1	64	37	124	230	2.21E-05	1.75E-05	2.93E-05	5.26E-05
Y71G10AR.4	101	91	212	331	1.17E-05	8.89E-06	1.52E-05	2.94E-05
Y71G12A.2	142	120	214	307	1.06E-05	5.79E-06	1.34E-05	3.07E-05
Y71G12A.3	86	111	94	178	1.13E-05	9.58E-06	1.54E-05	2.96E-05
Y71G12A.4	13	24	21	16	8.32E-06	6.64E-06	8.16E-06	1.45E-05
Y71G12B.10	227	239	250	290	3.64E-06	4.44E-06	2.59E-06	6.07E-06
Y71G12B.11a	758	886	584	1039	2.80E-06	2.65E-06	1.82E-06	2.25E-06
Y71G12B.11b	367	465	269	478	2.41E-05	2.40E-05	1.73E-05	2.48E-05
Y71G12B.12a	196	212	226	288	1.11E-05	1.22E-05	5.56E-06	1.22E-05
Y71G12B.12b	104	99	114	170	1.31E-05	1.57E-05	6.23E-06	1.37E-05
Y71G12B.13	117	96	218	360	2.22E-05	2.27E-05	1.67E-05	2.62E-05
Y71G12B.14	141	154	450	136	2.28E-05	2.05E-05	1.63E-05	2.99E-05
Y71G12B.15.	390	454	538	694	7.90E-06	6.11E-06	9.57E-06	1.95E-05
Y71G12B.15.	235	269	310	393	5.48E-05	5.66E-05	1.14E-04	4.25E-05
Y71G12B.16	11	22	6	10	3.28E-05	3.61E-05	2.95E-05	4.69E-05
Y71G12B.17	53	68	31	24	3.59E-05	3.88E-05	3.08E-05	4.82E-05
Y71G12B.18	34	5	10	2	2.80E-06	2.65E-06	1.82E-06	2.25E-06
Y71G12B.1a	385	289	574	664	5.60E-06	6.80E-06	2.13E-06	2.25E-06
Y71G12B.2	1	10	3	1	3.75E-06	2.65E-06	1.82E-06	2.25E-06
Y71G12B.20a	105	138	150	173	2.92E-05	2.07E-05	2.83E-05	4.04E-05
Y71G12B.20b	32	31	31	37	2.80E-06	2.65E-06	1.82E-06	2.25E-06
Y71G12B.21	26	19	26	31	4.93E-06	6.14E-06	4.59E-06	6.52E-06
Y71G12B.22	11	17	11	7	4.28E-06	3.91E-06	2.70E-06	3.98E-06
Y71G12B.23a	34	40	53	36	4.68E-06	3.23E-06	3.04E-06	4.48E-06
Y71G12B.23b	31	33	39	26	2.80E-06	2.65E-06	1.82E-06	2.25E-06
Y71G12B.24	247	268	271	334	2.80E-06	2.65E-06	2.04E-06	2.25E-06
Y71G12B.25	276	309	128	140	2.80E-06	2.65E-06	1.82E-06	2.25E-06
Y71G12B.26	8	10	8	5	1.67E-05	1.71E-05	1.19E-05	1.81E-05
Y71G12B.27	15	25	9	8	2.04E-05	2.16E-05	6.16E-06	8.32E-06
Y71G12B.28	27	26	44	49	2.80E-06	2.65E-06	1.82E-06	2.25E-06
Y71G12B.3	12	11	6	6	4.65E-06	7.33E-06	1.82E-06	2.25E-06
Y71G12B.30	5	22	11	7	5.91E-06	5.40E-06	6.27E-06	8.64E-06
Y71G12B.31	1	2	1	1	2.80E-06	2.65E-06	1.82E-06	2.25E-06
Y71G12B.32	1	2	5	3	2.80E-06	2.65E-06	1.82E-06	2.25E-06
Y71G12B.33	3	5	2	1	2.80E-06	2.65E-06	1.82E-06	2.25E-06
Y71G12B.4	102	147	128	104	2.80E-06	2.65E-06	1.82E-06	2.25E-06

Y71G12B.5	9	12	12	9	2.80E-06	2.65E-06	1.82E-06	2.25E-06
Y71G12B.6.1	99	180	102	112	1.04E-05	1.42E-05	8.51E-06	8.52E-06
Y71G12B.6.2	90	171	91	96	2.80E-06	2.65E-06	1.82E-06	2.25E-06
Y71G12B.7	6	3	9	4	8.96E-06	1.54E-05	5.99E-06	8.14E-06
Y71G12B.8	310	389	427	625	8.71E-06	1.56E-05	5.72E-06	7.47E-06
Y71G12B.9a	424	359	555	778	2.80E-06	2.65E-06	1.82E-06	2.25E-06
Y71G12B.9b	221	193	299	424	1.39E-05	1.65E-05	1.25E-05	2.25E-05
Y71H10A.1a	559	737	605	968	1.86E-05	1.48E-05	1.58E-05	2.73E-05
Y71H10A.1b.	475	622	518	854	1.61E-05	1.33E-05	1.42E-05	2.48E-05
Y71H10A.1b.	556	721	606	965	2.30E-05	2.86E-05	1.62E-05	3.19E-05
Y71H10A.1b.	512	650	564	883	2.31E-05	2.86E-05	1.64E-05	3.34E-05
Y71H10A.2.1	111	262	200	351	2.63E-05	3.23E-05	1.87E-05	3.67E-05
Y71H10A.2.2	122	267	206	353	2.67E-05	3.20E-05	1.91E-05	3.70E-05
Y71H10A.2.3	120	271	205	355	6.64E-06	1.48E-05	7.78E-06	1.68E-05
Y71H10A.2.4	120	266	205	353	6.92E-06	1.43E-05	7.60E-06	1.61E-05
Y71H10A.2.5	124	269	206	353	6.69E-06	1.43E-05	7.43E-06	1.59E-05
Y71H10B.1a.	584	733	541	984	6.80E-06	1.43E-05	7.58E-06	1.61E-05
Y71H10B.1a.	558	706	523	967	6.94E-06	1.42E-05	7.51E-06	1.59E-05
Y71H10B.1b	686	828	610	1149	3.64E-05	4.31E-05	2.19E-05	4.92E-05
Y71H10B.1c	536	679	507	946	3.58E-05	4.28E-05	2.19E-05	4.99E-05
Y71H2AL.1	114	170	115	95	3.69E-05	4.21E-05	2.14E-05	4.97E-05
Y71H2AL.2	5	12	7	0	3.17E-05	3.79E-05	1.95E-05	4.49E-05
Y71H2AM.1.:	198	214	184	247	2.07E-05	2.92E-05	1.36E-05	1.39E-05
Y71H2AM.1.:	192	210	181	246	2.80E-06	3.49E-06	1.82E-06	2.25E-06
Y71H2AM.10	38	46	67	43	1.25E-05	1.27E-05	7.54E-06	1.25E-05
Y71H2AM.11	307	422	301	423	1.21E-05	1.25E-05	7.43E-06	1.25E-05
Y71H2AM.12	29	38	41	47	2.80E-06	2.65E-06	1.82E-06	2.25E-06
Y71H2AM.15	75	94	36	39	2.21E-05	2.86E-05	1.41E-05	2.44E-05
Y71H2AM.15	66	86	32	36	3.81E-06	4.71E-06	3.50E-06	4.95E-06
Y71H2AM.16	38	65	28	33	2.88E-06	3.41E-06	1.82E-06	2.25E-06
Y71H2AM.17	208	155	270	379	2.97E-06	3.65E-06	1.82E-06	2.25E-06
Y71H2AM.19	421	445	438	928	2.97E-06	4.79E-06	1.82E-06	2.25E-06
Y71H2AM.2	154	214	364	334	1.88E-05	1.33E-05	1.59E-05	2.76E-05
Y71H2AM.20	352	324	405	618	2.40E-05	2.39E-05	1.62E-05	4.24E-05
Y71H2AM.20	320	285	372	595	1.91E-05	2.51E-05	2.94E-05	3.33E-05
Y71H2AM.20	168	167	214	336	3.26E-05	2.83E-05	2.44E-05	4.59E-05
Y71H2AM.23	1225	1352	1164	1694	3.17E-05	2.66E-05	2.39E-05	4.73E-05
Y71H2AM.23	1152	1273	1056	1624	3.82E-05	3.59E-05	3.17E-05	6.14E-05
Y71H2AM.24	75	87	82	99	6.36E-05	6.63E-05	3.93E-05	7.06E-05
Y71H2AM.25	1	2	3	3	7.20E-05	7.52E-05	4.29E-05	8.15E-05
Y71H2AM.3	148	149	240	284	1.00E-05	1.10E-05	7.11E-06	1.06E-05
Y71H2AM.4.:	1706	2242	3580	1516	2.80E-06	2.65E-06	1.82E-06	2.25E-06
Y71H2AM.4.:	377	406	684	372	1.14E-05	1.08E-05	1.20E-05	1.76E-05
Y71H2AM.5.:	1522	2088	3752	1247	1.85E-04	2.29E-04	2.52E-04	1.32E-04
Y71H2AM.5.:	1205	1724	2562	1009	8.57E-05	8.71E-05	1.01E-04	6.79E-05
Y71H2AM.5.:	1631	2129	3324	1403	2.77E-04	3.59E-04	4.44E-04	1.82E-04
Y71H2AM.6	40	55	63	64	2.43E-04	3.29E-04	3.36E-04	1.64E-04
Y71H2AM.7	121	115	191	256	3.32E-04	4.10E-04	4.41E-04	2.29E-04
Y71H2AM.8	134	127	166	288	5.38E-06	6.98E-06	5.50E-06	6.90E-06
Y71H2AM.9	335	296	832	682	1.13E-05	1.02E-05	1.16E-05	1.92E-05
Y71H2AR.1.1	167	185	149	147	9.94E-06	8.91E-06	8.02E-06	1.72E-05
Y71H2AR.1.2	168	186	149	148	1.99E-05	1.66E-05	3.22E-05	3.25E-05
Y71H2AR.2	3	3	1	4	3.44E-05	3.61E-05	2.00E-05	2.44E-05
Y71H2AR.3	37	44	40	46	3.47E-05	3.63E-05	2.00E-05	2.46E-05
Y71H2B.1	30	44	52	41	2.80E-06	2.65E-06	1.82E-06	2.25E-06
Y71H2B.10a	1011	981	913	1514	6.36E-06	7.14E-06	4.46E-06	6.34E-06
Y71H2B.10b	237	245	160	334	6.83E-06	9.47E-06	7.73E-06	7.51E-06
Y71H2B.10c	742	730	719	1146	3.65E-05	3.35E-05	2.14E-05	4.39E-05
Y71H2B.11	30	24	29	25	3.90E-05	3.81E-05	1.71E-05	4.41E-05

Y71H2B.2	375	359	497	588	3.33E-05	3.09E-05	2.10E-05	4.13E-05
Y71H2B.3	113	132	388	362	4.68E-06	3.54E-06	2.95E-06	3.13E-06
Y71H2B.4	115	250	263	107	3.26E-05	2.95E-05	2.81E-05	4.11E-05
Y71H2B.5	144	176	177	308	1.28E-05	1.42E-05	2.86E-05	3.30E-05
Y71H2B.6	146	173	189	224	3.74E-05	7.69E-05	5.57E-05	2.80E-05
Y71H2B.7.1	42	59	38	36	6.13E-06	7.09E-06	4.90E-06	1.05E-05
Y71H2B.7.2	37	51	30	31	2.25E-05	2.52E-05	1.89E-05	2.77E-05
Y71H2B.8	19	20	29	23	3.36E-06	4.47E-06	1.99E-06	2.32E-06
Y71H9A.1	4	11	12	8	3.30E-06	4.29E-06	1.82E-06	2.25E-06
Y71H9A.2	9	14	12	5	2.80E-06	2.65E-06	2.46E-06	2.41E-06
Y71H9A.3.1	105	153	105	128	2.80E-06	2.65E-06	1.82E-06	2.25E-06
Y71H9A.3.2	98	144	102	125	2.80E-06	2.65E-06	1.82E-06	2.25E-06
Y72A10A.1	19	44	27	27	9.38E-06	1.29E-05	6.10E-06	9.18E-06
Y73B3A.1	564	456	965	1124	8.93E-06	1.24E-05	6.05E-06	9.15E-06
Y73B3A.10	62	49	49	90	2.80E-06	4.84E-06	2.04E-06	2.52E-06
Y73B3A.11	8	17	10	11	3.05E-05	2.33E-05	3.39E-05	4.88E-05
Y73B3A.12	138	108	102	138	9.83E-06	7.33E-06	5.05E-06	1.14E-05
Y73B3A.13	17	12	26	41	2.80E-06	4.55E-06	1.84E-06	2.50E-06
Y73B3A.14	2	4	2	0	4.40E-05	3.26E-05	2.12E-05	3.54E-05
Y73B3A.15	2	5	6	5	2.80E-06	2.65E-06	1.82E-06	2.92E-06
Y73B3A.16	23	28	35	45	2.80E-06	2.65E-06	1.82E-06	2.25E-06
Y73B3A.18a	7421	5796	6095	5818	2.80E-06	2.65E-06	1.82E-06	2.25E-06
Y73B3A.18b	7359	5707	6004	5729	3.08E-06	3.54E-06	3.06E-06	4.86E-06
Y73B3A.2	336	540	477	490	7.49E-04	5.53E-04	4.01E-04	4.72E-04
Y73B3A.20	23	13	17	30	6.78E-04	4.97E-04	3.60E-04	4.24E-04
Y73B3A.21	49	38	52	65	5.17E-05	7.85E-05	4.78E-05	6.05E-05
Y73B3A.3	75	80	108	140	5.15E-06	2.75E-06	2.48E-06	5.40E-06
Y73B3A.4	99	104	183	313	8.18E-06	5.98E-06	5.65E-06	8.70E-06
Y73B3A.5	13	16	12	24	5.10E-06	5.13E-06	4.77E-06	7.62E-06
Y73B3A.6	10	8	13	14	6.50E-06	6.43E-06	7.80E-06	1.65E-05
Y73B3A.7	4	3	4	4	2.80E-06	2.65E-06	1.82E-06	2.32E-06
Y73B3A.8	0	1	1	1	2.80E-06	2.65E-06	1.82E-06	2.25E-06
Y73B3A.9	89	91	101	165	2.80E-06	2.65E-06	1.82E-06	2.25E-06
Y73B3A.t1	0	0	1	1	2.80E-06	2.65E-06	1.82E-06	2.25E-06
Y73B3B.1	121	112	254	381	1.05E-05	1.02E-05	7.76E-06	1.57E-05
Y73B3B.2	22	18	16	30	2.80E-06	2.65E-06	1.82E-06	2.25E-06
Y73B3B.3	3	8	3	7	1.20E-05	1.05E-05	1.64E-05	3.03E-05
Y73B3B.5	141	180	98	192	3.42E-06	2.65E-06	1.82E-06	3.73E-06
Y73B6A.1	12	18	12	15	2.80E-06	4.55E-06	1.82E-06	3.40E-06
Y73B6A.2	3	11	6	1	9.16E-06	1.10E-05	4.14E-06	1.00E-05
Y73B6A.3	15	12	8	4	2.80E-06	2.65E-06	1.82E-06	2.25E-06
Y73B6A.4	158	196	198	238	2.80E-06	2.65E-06	1.82E-06	2.25E-06
Y73B6A.5a.1	1174	1041	1700	2634	4.40E-06	3.33E-06	1.82E-06	2.25E-06
Y73B6A.5a.2	1185	1044	1712	2642	1.18E-05	1.38E-05	9.62E-06	1.43E-05
Y73B6A.5b	557	507	831	1225	4.11E-05	3.45E-05	3.88E-05	7.42E-05
Y73B6A.6	4	4	1	1	4.14E-05	3.45E-05	3.90E-05	7.42E-05
Y73B6BL.1	51	189	59	140	3.38E-05	2.91E-05	3.28E-05	5.97E-05
Y73B6BL.10	4	4	7	1	2.80E-06	2.65E-06	1.82E-06	2.25E-06
Y73B6BL.11	8	7	15	3	2.80E-06	8.52E-06	1.82E-06	5.35E-06
Y73B6BL.12	15	28	41	28	2.80E-06	2.65E-06	1.82E-06	2.25E-06
Y73B6BL.13	4	9	15	4	2.80E-06	2.65E-06	1.82E-06	2.25E-06
Y73B6BL.14	9	14	16	10	2.80E-06	4.31E-06	4.35E-06	3.67E-06
Y73B6BL.16	2	3	23	7	2.80E-06	2.65E-06	2.99E-06	2.25E-06
Y73B6BL.18.:	496	604	608	1017	3.36E-06	4.95E-06	3.88E-06	2.99E-06
Y73B6BL.18.:	475	586	590	995	2.80E-06	2.65E-06	2.06E-06	2.25E-06
Y73B6BL.18.:	475	585	590	995	1.34E-05	1.54E-05	1.07E-05	2.20E-05
Y73B6BL.19	33	58	34	46	1.28E-05	1.49E-05	1.04E-05	2.16E-05
Y73B6BL.2	223	288	327	385	1.28E-05	1.49E-05	1.03E-05	2.15E-05
Y73B6BL.20	5	10	2	1	2.80E-06	3.28E-06	1.82E-06	2.25E-06

Y73B6BL.21	20	21	31	21	2.15E-05	2.62E-05	2.05E-05	2.98E-05
Y73B6BL.22	1	2	3	0	2.80E-06	2.91E-06	1.82E-06	2.25E-06
Y73B6BL.23	309	300	484	464	2.80E-06	2.65E-06	2.39E-06	2.25E-06
Y73B6BL.24.1	1128	1465	1756	1518	2.80E-06	2.65E-06	1.82E-06	2.25E-06
Y73B6BL.24.3	1034	1337	1643	1466	5.83E-05	5.34E-05	5.94E-05	7.03E-05
Y73B6BL.25	31	61	37	64	1.03E-04	1.26E-04	1.04E-04	1.11E-04
Y73B6BL.26	18	20	19	13	1.01E-04	1.23E-04	1.04E-04	1.15E-04
Y73B6BL.27	58	78	50	59	2.80E-06	4.84E-06	2.02E-06	4.32E-06
Y73B6BL.28	10	12	5	8	2.80E-06	2.65E-06	1.82E-06	2.25E-06
Y73B6BL.29	54	68	76	116	1.30E-05	1.65E-05	7.31E-06	1.06E-05
Y73B6BL.3	228	209	332	338	3.11E-06	3.54E-06	1.82E-06	2.25E-06
Y73B6BL.30	176	199	253	232	6.24E-06	7.43E-06	5.72E-06	1.08E-05
Y73B6BL.31a	85	193	85	149	2.46E-05	2.13E-05	2.33E-05	2.93E-05
Y73B6BL.31b	67	144	59	115	3.21E-05	3.42E-05	3.00E-05	3.39E-05
Y73B6BL.32.1	520	404	933	439	6.36E-06	1.36E-05	4.14E-06	8.93E-06
Y73B6BL.32.2	482	383	731	379	6.61E-06	1.34E-05	3.79E-06	9.11E-06
Y73B6BL.33	519	491	582	776	1.02E-04	7.46E-05	1.19E-04	6.89E-05
Y73B6BL.34	854	994	453	295	1.18E-04	8.89E-05	1.17E-04	7.48E-05
Y73B6BL.35	40	51	21	18	2.57E-05	2.30E-05	1.88E-05	3.09E-05
Y73B6BL.36	1	6	7	8	9.90E-05	1.09E-04	3.42E-05	2.75E-05
Y73B6BL.37	5	9	2	5	8.76E-06	1.06E-05	2.99E-06	3.17E-06
Y73B6BL.38	2209	1829	3327	3008	2.80E-06	2.65E-06	1.82E-06	2.25E-06
Y73B6BL.39	7	15	10	8	2.80E-06	2.65E-06	1.82E-06	2.25E-06
Y73B6BL.4	332	552	471	651	1.49E-04	1.16E-04	1.46E-04	1.63E-04
Y73B6BL.40	9	10	11	17	2.80E-06	2.65E-06	1.82E-06	2.25E-06
Y73B6BL.41	11	10	14	30	1.92E-05	3.02E-05	1.77E-05	3.03E-05
Y73B6BL.42	16	26	11	11	2.80E-06	2.65E-06	1.82E-06	2.25E-06
Y73B6BL.43	6	8	4	3	2.80E-06	2.65E-06	1.82E-06	2.68E-06
Y73B6BL.44	31	73	19	38	2.80E-06	2.65E-06	1.82E-06	2.25E-06
Y73B6BL.45	1	1	0	0	2.80E-06	2.65E-06	1.82E-06	2.25E-06
Y73B6BL.5a.1	473	480	619	855	4.62E-06	1.03E-05	1.84E-06	4.54E-06
Y73B6BL.5a.2	450	468	566	824	2.80E-06	2.65E-06	1.82E-06	2.25E-06
Y73B6BL.5b	447	463	578	816	2.98E-05	2.86E-05	2.54E-05	4.33E-05
Y73B6BL.5c.1	418	413	523	788	2.85E-05	2.80E-05	2.33E-05	4.19E-05
Y73B6BL.5c.2	450	468	566	824	2.74E-05	2.68E-05	2.31E-05	4.02E-05
Y73B6BL.6a.1	7205	5447	8257	7782	2.76E-05	2.58E-05	2.25E-05	4.18E-05
Y73B6BL.6a.2	7457	5726	8688	8009	2.85E-05	2.80E-05	2.33E-05	4.19E-05
Y73B6BL.6a.3	7240	5495	8287	7792	4.11E-04	2.93E-04	3.06E-04	3.56E-04
Y73B6BL.6a.4	7515	5806	8729	8079	4.17E-04	3.02E-04	3.16E-04	3.60E-04
Y73B6BL.6a.5	7206	5442	8257	7777	4.08E-04	2.92E-04	3.04E-04	3.53E-04
Y73B6BL.6a.6	2228	1887	2263	3250	4.20E-04	3.07E-04	3.17E-04	3.63E-04
Y73B6BL.6a.7	2485	2195	2713	3493	4.08E-04	2.91E-04	3.04E-04	3.54E-04
Y73B6BL.6b.1	7530	5811	8743	8144	2.52E-04	2.01E-04	1.66E-04	2.95E-04
Y73B6BL.6b.2	2501	2193	2725	3558	2.69E-04	2.25E-04	1.91E-04	3.04E-04
Y73B6BL.6b.3	2500	2200	2727	3558	4.19E-04	3.05E-04	3.17E-04	3.64E-04
Y73B6BL.7	16	33	18	27	2.69E-04	2.22E-04	1.90E-04	3.07E-04
Y73B6BL.9a	254	267	239	275	2.68E-04	2.23E-04	1.91E-04	3.07E-04
Y73B6BL.9b	177	180	157	198	2.80E-06	2.65E-06	1.82E-06	2.25E-06
Y73B6BR.1a	161	327	138	173	3.30E-05	3.28E-05	2.02E-05	2.87E-05
Y73C8A.1	4	6	6	3	2.97E-05	2.85E-05	1.71E-05	2.67E-05
Y73C8B.1	18	48	11	24	1.04E-05	2.00E-05	5.81E-06	9.00E-06
Y73C8B.2	26	67	30	39	2.80E-06	2.65E-06	1.82E-06	2.25E-06
Y73C8B.3	75	136	72	74	2.80E-06	5.42E-06	1.82E-06	2.32E-06
Y73C8B.4	7	6	11	7	2.91E-06	7.06E-06	2.19E-06	3.51E-06
Y73C8B.5	1	0	0	0	9.07E-06	1.55E-05	5.67E-06	7.17E-06
Y73C8C.1	9	2	5	1	2.80E-06	2.65E-06	1.82E-06	2.25E-06
Y73C8C.10	1	6	6	4	2.80E-06	2.65E-06	1.82E-06	2.25E-06
Y73C8C.11	3	5	5	1	2.80E-06	2.65E-06	1.82E-06	2.25E-06
Y73C8C.12	0	0	3	1	2.80E-06	2.65E-06	1.82E-06	2.25E-06

Y73C8C.2	5	8	26	11	2.80E-06	2.65E-06	1.82E-06	2.25E-06
Y73C8C.3	17	15	28	19	2.80E-06	2.65E-06	1.82E-06	2.25E-06
Y73C8C.4	9	4	14	6	2.80E-06	2.65E-06	1.84E-06	2.25E-06
Y73C8C.5	2	6	6	3	2.80E-06	2.65E-06	1.82E-06	2.25E-06
Y73C8C.6	5	5	6	4	2.80E-06	2.65E-06	1.82E-06	2.25E-06
Y73C8C.7	9	14	16	10	2.80E-06	2.65E-06	1.82E-06	2.25E-06
Y73C8C.8	22	29	32	29	2.80E-06	2.65E-06	1.82E-06	2.25E-06
Y73C8C.9	4	4	4	3	2.80E-06	2.65E-06	1.82E-06	2.25E-06
Y73E7A.1b	120	129	134	141	2.80E-06	2.65E-06	1.82E-06	2.25E-06
Y73E7A.2	98	95	90	164	2.80E-06	2.65E-06	1.82E-06	2.25E-06
Y73E7A.3	33	61	27	44	1.96E-05	1.99E-05	1.42E-05	1.85E-05
Y73E7A.4	18	30	25	12	7.90E-06	7.22E-06	4.72E-06	1.06E-05
Y73E7A.5	2	3	5	1	2.80E-06	4.31E-06	1.82E-06	2.65E-06
Y73E7A.6	140	144	237	241	2.80E-06	4.29E-06	2.46E-06	2.25E-06
Y73E7A.7	60	101	97	108	2.80E-06	2.65E-06	1.82E-06	2.25E-06
Y73E7A.8	77	115	38	30	3.25E-05	3.16E-05	3.58E-05	4.49E-05
Y73E7A.9	66	54	127	203	5.82E-06	9.28E-06	6.14E-06	8.43E-06
Y73F4A.1	75	107	67	102	3.72E-06	5.26E-06	1.82E-06	2.25E-06
Y73F4A.2	60	94	43	37	4.40E-06	3.41E-06	5.52E-06	1.09E-05
Y73F4A.3	10	17	11	15	1.28E-05	1.72E-05	7.42E-06	1.39E-05
Y73F8A.1	58	44	79	42	8.20E-06	1.22E-05	3.83E-06	4.07E-06
Y73F8A.10	5	3	3	0	2.80E-06	3.02E-06	1.82E-06	2.27E-06
Y73F8A.11	41	83	41	118	2.94E-06	2.65E-06	2.61E-06	2.25E-06
Y73F8A.12	8	17	8	2	2.80E-06	2.65E-06	1.82E-06	2.25E-06
Y73F8A.13	22	33	25	23	2.80E-06	5.29E-06	1.82E-06	6.41E-06
Y73F8A.14	9	13	9	3	2.80E-06	4.60E-06	1.82E-06	2.25E-06
Y73F8A.15	6	16	5	2	2.88E-06	4.07E-06	2.13E-06	2.43E-06
Y73F8A.16	3	7	20	6	2.80E-06	3.52E-06	1.82E-06	2.25E-06
Y73F8A.17	5	6	14	4	2.80E-06	4.58E-06	1.82E-06	2.25E-06
Y73F8A.18	1	1	7	6	2.80E-06	2.65E-06	1.82E-06	2.25E-06
Y73F8A.19	17	22	26	18	2.80E-06	2.65E-06	1.82E-06	2.25E-06
Y73F8A.2	4	3	4	2	2.80E-06	2.65E-06	1.82E-06	2.25E-06
Y73F8A.20	36	44	23	26	2.80E-06	2.65E-06	1.82E-06	2.25E-06
Y73F8A.21	24	69	25	46	2.80E-06	2.65E-06	1.82E-06	2.25E-06
Y73F8A.22	1	4	7	3	2.80E-06	2.80E-06	1.82E-06	2.25E-06
Y73F8A.23	5	2	2	1	2.80E-06	3.47E-06	1.82E-06	2.25E-06
Y73F8A.24	165	159	154	221	2.80E-06	2.65E-06	1.82E-06	2.25E-06
Y73F8A.25a	395	333	443	624	2.80E-06	2.65E-06	1.82E-06	2.25E-06
Y73F8A.25b	323	240	322	544	1.20E-05	1.10E-05	7.31E-06	1.29E-05
Y73F8A.26	54	94	69	86	2.29E-05	1.83E-05	1.67E-05	2.91E-05
Y73F8A.3	2	5	9	0	2.50E-05	1.75E-05	1.62E-05	3.38E-05
Y73F8A.30	16	11	5	6	3.11E-06	5.11E-06	2.59E-06	3.98E-06
Y73F8A.31	54	41	50	72	2.80E-06	2.65E-06	1.82E-06	2.25E-06
Y73F8A.32	4	5	2	3	2.80E-06	2.65E-06	1.82E-06	2.25E-06
Y73F8A.33	7	10	6	11	1.16E-05	8.31E-06	6.98E-06	1.24E-05
Y73F8A.34a.[illegible]	612	552	932	996	2.80E-06	2.88E-06	1.82E-06	2.25E-06
Y73F8A.34a.[illegible]	537	499	825	886	2.80E-06	2.65E-06	1.82E-06	2.25E-06
Y73F8A.34b.[illegible]	366	289	422	464	4.10E-05	3.49E-05	4.06E-05	5.35E-05
Y73F8A.34b.[illegible]	280	234	325	369	4.12E-05	3.62E-05	4.12E-05	5.46E-05
Y73F8A.35	5	15	12	11	5.72E-05	4.26E-05	4.29E-05	5.82E-05
Y73F8A.36	2	5	0	0	6.36E-05	5.02E-05	4.80E-05	6.73E-05
Y73F8A.37	44	60	50	81	2.80E-06	2.65E-06	1.82E-06	2.25E-06
Y73F8A.4	14	16	16	13	2.80E-06	2.65E-06	1.82E-06	2.25E-06
Y73F8A.5	23	40	30	29	9.38E-06	1.21E-05	6.94E-06	1.39E-05
Y73F8A.6	240	528	284	407	2.80E-06	2.65E-06	1.82E-06	2.25E-06
Y73F8A.7	3	1	3	0	2.80E-06	2.65E-06	1.82E-06	2.25E-06
Y73F8A.8	14	18	6	6	2.52E-05	5.24E-05	1.94E-05	3.43E-05
Y73F8A.9	14	17	6	7	2.80E-06	2.65E-06	1.82E-06	2.25E-06
Y74C10AR.1	230	298	372	709	2.80E-06	2.65E-06	1.82E-06	2.25E-06

Y74C10AR.2	4	2	3	3	2.80E-06	2.65E-06	1.82E-06	2.25E-06
Y74C10AR.3	143	128	196	279	2.62E-05	3.20E-05	2.76E-05	6.48E-05
Y74C9A.1	1	8	3	2	2.80E-06	2.65E-06	1.82E-06	2.25E-06
Y74C9A.2.1	509	728	708	774	7.56E-06	6.40E-06	6.76E-06	1.19E-05
Y74C9A.2.2	358	504	481	500	2.80E-06	2.65E-06	1.82E-06	2.25E-06
Y74C9A.2.3	481	670	614	701	8.13E-05	1.10E-04	7.36E-05	9.93E-05
Y74C9A.2.4	541	751	753	752	6.24E-05	8.29E-05	5.45E-05	6.99E-05
Y74C9A.2.5	488	686	679	714	9.72E-05	1.28E-04	8.08E-05	1.14E-04
Y74C9A.3.1	118	152	172	179	9.20E-05	1.21E-04	8.33E-05	1.03E-04
Y74C9A.3.2	108	127	148	157	8.90E-05	1.18E-04	8.06E-05	1.05E-04
Y74C9A.4a	449	391	601	799	1.51E-05	1.83E-05	1.43E-05	1.83E-05
Y74C9A.4b	488	430	668	984	1.54E-05	1.71E-05	1.37E-05	1.79E-05
Y74C9A.5	143	199	233	293	2.98E-05	2.45E-05	2.59E-05	4.26E-05
Y74E4A.1a	62	131	20	40	2.62E-05	2.18E-05	2.33E-05	4.24E-05
Y74E4A.1b	63	138	20	42	7.59E-06	9.97E-06	8.05E-06	1.25E-05
Y75B12A.1	42	41	50	87	5.07E-06	1.01E-05	1.82E-06	2.63E-06
Y75B12A.2	5	8	6	2	4.79E-06	9.89E-06	1.82E-06	2.56E-06
Y75B12B.1	919	772	1013	1276	4.42E-06	4.10E-06	3.44E-06	7.38E-06
Y75B12B.10	4	8	4	8	2.80E-06	2.65E-06	1.82E-06	2.25E-06
Y75B12B.11	5	1	7	2	1.07E-04	8.46E-05	7.64E-05	1.19E-04
Y75B12B.2.1	6406	5869	7116	5909	2.80E-06	2.65E-06	1.82E-06	2.25E-06
Y75B12B.2.2	5737	5282	6048	5513	2.80E-06	2.65E-06	1.82E-06	2.25E-06
Y75B12B.2.3	5707	5225	6012	5462	1.12E-03	9.73E-04	8.13E-04	8.33E-04
Y75B12B.3	5	6	27	10	9.98E-04	8.68E-04	6.84E-04	7.70E-04
Y75B12B.4	1451	1632	1821	1297	1.00E-03	8.68E-04	6.88E-04	7.71E-04
Y75B12B.5.1	2206	2396	2739	2210	2.80E-06	2.65E-06	6.91E-06	3.15E-06
Y75B12B.5.2	2002	2194	2472	2123	1.69E-04	1.79E-04	1.38E-04	1.21E-04
Y75B12B.6	85	120	89	135	3.85E-04	3.95E-04	3.11E-04	3.10E-04
Y75B12B.7	4	5	6	2	3.45E-04	3.57E-04	2.77E-04	2.94E-04
Y75B12B.8	4	2	1	2	3.36E-06	4.47E-06	2.28E-06	4.27E-06
Y75B12B.9	1	1	2	0	2.80E-06	2.65E-06	1.82E-06	2.25E-06
Y75B7AL.1	30	40	15	22	2.80E-06	2.65E-06	1.82E-06	2.25E-06
Y75B7AL.2	5	10	2	10	2.80E-06	2.65E-06	1.82E-06	2.25E-06
Y75B7AL.3	26	39	24	21	2.80E-06	2.65E-06	1.82E-06	2.25E-06
Y75B7AL.4a	484	371	854	1257	2.80E-06	2.65E-06	1.82E-06	2.25E-06
Y75B7AL.4b	126	103	212	308	2.80E-06	3.04E-06	1.82E-06	2.25E-06
Y75B7AR.1	91	103	33	14	1.60E-05	1.16E-05	1.84E-05	3.34E-05
Y75B7B.1	18	35	12	14	1.64E-05	1.27E-05	1.80E-05	3.23E-05
Y75B7B.2	12	19	19	9	1.90E-05	2.04E-05	4.50E-06	2.36E-06
Y75B8A.1	43	57	121	94	2.80E-06	3.23E-06	1.82E-06	2.25E-06
Y75B8A.10	4	3	2	7	2.80E-06	2.65E-06	1.82E-06	2.25E-06
Y75B8A.11	9	15	9	6	4.03E-06	5.03E-06	7.36E-06	7.06E-06
Y75B8A.12	3	15	8	10	2.80E-06	2.65E-06	1.82E-06	2.25E-06
Y75B8A.13	424	357	413	852	2.80E-06	2.65E-06	1.82E-06	2.25E-06
Y75B8A.14	175	155	244	250	2.80E-06	2.65E-06	1.82E-06	2.25E-06
Y75B8A.16	122	130	219	219	2.93E-05	2.33E-05	1.85E-05	4.72E-05
Y75B8A.17	173	123	506	235	2.09E-05	1.75E-05	1.89E-05	2.39E-05
Y75B8A.18	89	89	125	106	9.04E-06	9.10E-06	1.06E-05	1.30E-05
Y75B8A.19	26	29	40	50	3.57E-05	2.40E-05	6.79E-05	3.89E-05
Y75B8A.20	189	282	129	26	1.94E-05	1.84E-05	1.78E-05	1.86E-05
Y75B8A.21	3	3	3	6	2.80E-06	2.65E-06	1.82E-06	2.25E-06
Y75B8A.22	517	590	549	985	3.31E-05	4.66E-05	1.47E-05	3.67E-06
Y75B8A.23	12	20	6	2	2.80E-06	2.65E-06	1.82E-06	2.25E-06
Y75B8A.24	983	770	804	1509	1.40E-05	1.51E-05	9.68E-06	2.14E-05
Y75B8A.25	145	165	192	307	5.15E-06	8.12E-06	1.82E-06	2.25E-06
Y75B8A.26	53	52	46	64	1.67E-05	1.24E-05	8.91E-06	2.07E-05
Y75B8A.27	89	140	66	116	8.71E-06	9.36E-06	7.51E-06	1.48E-05
Y75B8A.28	19	26	18	22	2.80E-06	2.65E-06	1.82E-06	2.25E-06
Y75B8A.2a	50	57	98	51	6.78E-06	1.01E-05	3.26E-06	7.08E-06

Y75B8A.2b	27	40	39	32	2.80E-06	2.65E-06	1.82E-06	2.25E-06
Y75B8A.3	35	52	28	26	3.61E-06	3.89E-06	4.61E-06	2.97E-06
Y75B8A.30	331	308	315	445	2.94E-06	4.13E-06	2.77E-06	2.81E-06
Y75B8A.31	124	132	107	188	2.80E-06	2.65E-06	1.82E-06	2.25E-06
Y75B8A.32	12	18	22	8	3.67E-05	3.23E-05	2.27E-05	3.96E-05
Y75B8A.33	7	7	3	6	1.46E-05	1.47E-05	8.22E-06	1.78E-05
Y75B8A.34	6	3	20	6	2.80E-06	2.65E-06	1.82E-06	2.25E-06
Y75B8A.35a.:	67	81	88	79	2.80E-06	2.65E-06	1.82E-06	2.25E-06
Y75B8A.35a.:	46	60	39	53	2.80E-06	2.65E-06	1.82E-06	2.25E-06
Y75B8A.35a.:	46	59	38	52	4.00E-06	4.55E-06	3.41E-06	3.78E-06
Y75B8A.35b	65	81	87	79	3.53E-06	4.34E-06	1.95E-06	3.26E-06
Y75B8A.37	1	1	2	0	3.53E-06	4.29E-06	1.90E-06	3.19E-06
Y75B8A.38	1	7	16	10	4.14E-06	4.87E-06	3.61E-06	4.03E-06
Y75B8A.39	9	12	10	1	2.80E-06	2.65E-06	1.82E-06	2.25E-06
Y75B8A.4.1	64	146	61	90	2.80E-06	6.67E-06	1.05E-05	8.10E-06
Y75B8A.4.2	61	140	55	88	2.80E-06	2.65E-06	1.82E-06	2.25E-06
Y75B8A.40	8	10	4	3	3.02E-06	6.51E-06	1.88E-06	3.42E-06
Y75B8A.41	5	5	8	2	2.94E-06	6.35E-06	1.82E-06	3.40E-06
Y75B8A.5	31	37	23	29	2.80E-06	2.65E-06	1.82E-06	2.25E-06
Y75B8A.6	24	30	14	28	2.80E-06	2.65E-06	1.82E-06	2.25E-06
Y75B8A.7	152	121	155	213	2.80E-06	2.91E-06	1.82E-06	2.25E-06
Y75B8A.8	143	214	176	404	2.80E-06	2.83E-06	1.82E-06	2.25E-06
Y75B8A.9a	62	87	60	60	8.51E-06	6.40E-06	5.65E-06	9.58E-06
Y75B8A.9b	50	63	42	40	7.03E-06	9.95E-06	5.63E-06	1.59E-05
Y75D11A.1	2	1	4	4	3.56E-06	4.71E-06	2.24E-06	2.77E-06
Y75D11A.2	22	21	23	40	4.12E-06	4.89E-06	2.24E-06	2.63E-06
Y75D11A.3	54	50	84	95	2.80E-06	2.65E-06	1.82E-06	2.25E-06
Y75D11A.6	1	0	0	0	4.54E-06	4.10E-06	3.08E-06	6.63E-06
Y75D11A.t2	0	0	2	0	7.90E-06	6.90E-06	7.98E-06	1.11E-05
Y76A2A.1	32	50	23	9	2.80E-06	2.65E-06	1.82E-06	2.25E-06
Y76A2A.2	584	663	762	1204	2.80E-06	2.65E-06	1.97E-06	2.25E-06
Y76A2B.1	2282	1935	3237	4190	4.06E-06	5.98E-06	1.90E-06	2.25E-06
Y76A2B.2	13	19	31	16	1.65E-05	1.77E-05	1.40E-05	2.74E-05
Y76A2B.3	385	614	356	547	6.96E-05	5.57E-05	6.42E-05	1.03E-04
Y76A2B.4	101	96	74	72	2.80E-06	2.65E-06	1.82E-06	2.25E-06
Y76A2B.5	429	574	486	597	2.02E-05	3.04E-05	1.22E-05	2.31E-05
Y76A2B.6	244	246	669	549	2.46E-05	2.21E-05	1.18E-05	1.41E-05
Y76B12C.1	11	6	7	3	4.40E-05	5.56E-05	3.24E-05	4.91E-05
Y76B12C.2	436	324	683	1219	1.32E-05	1.26E-05	2.36E-05	2.39E-05
Y76B12C.3	144	206	192	164	2.80E-06	2.65E-06	1.82E-06	2.25E-06
Y76B12C.4	38	52	25	29	1.30E-05	9.13E-06	1.32E-05	2.92E-05
Y76B12C.6	250	195	517	621	3.04E-05	4.11E-05	2.63E-05	2.78E-05
Y76B12C.7	395	395	339	671	5.60E-06	7.25E-06	2.41E-06	3.44E-06
Y76B12C.9	8	9	7	4	1.53E-05	1.13E-05	2.06E-05	3.05E-05
Y76G2A.1	27	28	21	33	9.88E-06	9.34E-06	5.52E-06	1.35E-05
Y76G2A.2	20	9	40	18	2.80E-06	2.65E-06	1.82E-06	2.25E-06
Y77E11A.1.1	310	370	354	519	7.31E-06	7.17E-06	3.70E-06	7.17E-06
Y77E11A.1.2	250	280	285	425	9.32E-06	3.97E-06	1.22E-05	6.75E-06
Y77E11A.10	38	65	31	28	2.09E-05	2.35E-05	1.55E-05	2.81E-05
Y77E11A.11.:	271	367	199	317	1.65E-05	1.75E-05	1.22E-05	2.25E-05
Y77E11A.11.:	251	346	194	303	2.80E-06	2.91E-06	1.82E-06	2.25E-06
Y77E11A.12a	18	23	12	26	1.19E-05	1.52E-05	5.69E-06	1.12E-05
Y77E11A.12b	19	26	15	28	1.19E-05	1.55E-05	5.98E-06	1.15E-05
Y77E11A.13a	223	241	202	321	2.80E-06	2.65E-06	1.82E-06	2.25E-06
Y77E11A.13b	194	190	198	248	2.80E-06	2.65E-06	1.82E-06	2.25E-06
Y77E11A.14	4	5	6	2	2.37E-05	2.41E-05	1.39E-05	2.73E-05
Y77E11A.15	2075	2941	3610	5724	2.73E-05	2.53E-05	1.81E-05	2.81E-05
Y77E11A.16	1	4	2	1	2.80E-06	2.65E-06	1.82E-06	2.25E-06
Y77E11A.2	42	104	39	64	2.52E-04	3.37E-04	2.85E-04	5.58E-04

Y77E11A.3	1	1	1	2	2.80E-06	2.65E-06	1.82E-06	2.25E-06
Y77E11A.4	116	100	96	129	3.70E-06	8.65E-06	2.24E-06	4.52E-06
Y77E11A.6	57	43	148	55	2.80E-06	2.65E-06	1.82E-06	2.25E-06
Y77E11A.7a	491	422	675	886	2.05E-05	1.67E-05	1.10E-05	1.83E-05
Y77E11A.7b	491	416	683	894	1.53E-05	1.09E-05	2.59E-05	1.19E-05
Y77E11A.7c	412	366	574	740	3.02E-05	2.45E-05	2.70E-05	4.38E-05
Y77E11A.8	1	2	1	2	2.59E-05	2.07E-05	2.34E-05	3.79E-05
Y77E11A.9	1	4	4	2	2.66E-05	2.23E-05	2.41E-05	3.84E-05
Y79H2A.11	442	321	471	699	2.80E-06	2.65E-06	1.82E-06	2.25E-06
Y79H2A.12	2	4	6	3	2.80E-06	2.65E-06	1.82E-06	2.25E-06
Y79H2A.1a.1	1609	2349	2093	3256	2.06E-05	1.41E-05	1.42E-05	2.61E-05
Y79H2A.1a.2	1573	2294	2025	3228	2.80E-06	2.65E-06	1.82E-06	2.25E-06
Y79H2A.1b.1	1360	1989	1783	2650	1.08E-04	1.49E-04	9.17E-05	1.76E-04
Y79H2A.1b.2	1319	1926	1710	2614	9.74E-05	1.34E-04	8.16E-05	1.61E-04
Y79H2A.2	53	90	63	62	9.83E-05	1.36E-04	8.38E-05	1.54E-04
Y79H2A.3a	694	555	645	981	1.05E-04	1.45E-04	8.90E-05	1.68E-04
Y79H2A.3b	293	225	293	435	1.11E-05	1.79E-05	8.64E-06	1.05E-05
Y79H2A.4	1	2	2	4	1.18E-05	8.94E-06	7.14E-06	1.34E-05
Y79H2A.6	619	565	615	812	8.60E-06	6.24E-06	5.59E-06	1.03E-05
Y7A5A.1.1	355	601	393	565	2.80E-06	2.65E-06	1.82E-06	2.25E-06
Y7A5A.1.2	334	569	373	553	4.73E-05	4.08E-05	3.06E-05	4.98E-05
Y7A5A.10	3	1	0	0	2.34E-05	3.73E-05	1.68E-05	2.99E-05
Y7A5A.11	6	3	6	2	2.31E-05	3.72E-05	1.68E-05	3.07E-05
Y7A5A.2	8	10	5	5	2.80E-06	2.65E-06	1.82E-06	2.25E-06
Y7A5A.3	3	7	2	4	2.80E-06	2.65E-06	1.82E-06	2.25E-06
Y7A5A.5	0	1	1	1	2.80E-06	2.65E-06	1.82E-06	2.25E-06
Y7A5A.6	2	6	0	0	2.80E-06	2.65E-06	1.82E-06	2.25E-06
Y7A5A.7	7	7	4	1	2.80E-06	2.65E-06	1.82E-06	2.25E-06
Y7A5A.8	8	9	5	4	2.80E-06	2.65E-06	1.82E-06	2.25E-06
Y7A5A.9	5	6	10	8	2.80E-06	2.65E-06	1.82E-06	2.25E-06
Y7A5A.t4	0	0	0	1	2.80E-06	2.65E-06	1.82E-06	2.25E-06
Y7A9A.1	37	43	36	34	2.80E-06	2.65E-06	1.82E-06	2.25E-06
Y7A9C.1	11	24	13	21	2.80E-06	2.65E-06	1.82E-06	2.25E-06
Y7A9C.2	28	35	50	25	2.80E-06	2.65E-06	1.82E-06	2.25E-06
Y7A9C.3	1	2	2	1	2.80E-06	2.65E-06	1.82E-06	2.25E-06
Y7A9C.4	0	1	1	2	2.80E-06	2.65E-06	1.82E-06	2.25E-06
Y7A9C.5	8	4	5	5	2.80E-06	2.65E-06	1.82E-06	2.25E-06
Y7A9C.6	7	3	4	1	2.80E-06	2.65E-06	1.82E-06	2.25E-06
Y7A9C.7	10	5	7	6	2.80E-06	2.65E-06	1.82E-06	2.25E-06
Y7A9C.8	10	7	8	6	2.80E-06	2.65E-06	1.82E-06	2.25E-06
Y7A9C.9	7	5	18	4	2.80E-06	2.65E-06	1.82E-06	2.25E-06
Y7A9D.1	12	14	24	15	2.80E-06	2.65E-06	1.82E-06	2.25E-06
Y7A9D.t2	1	0	3	0	2.80E-06	2.65E-06	1.82E-06	2.25E-06
Y80D3A.1	472	297	871	889	2.80E-06	2.72E-06	3.21E-06	2.47E-06
Y80D3A.10	19	30	48	28	2.80E-06	2.65E-06	3.04E-06	2.25E-06
Y80D3A.11	11	13	3	14	2.66E-05	1.58E-05	3.19E-05	4.03E-05
Y80D3A.2.1	1363	1018	1351	1988	4.82E-06	7.17E-06	7.89E-06	5.69E-06
Y80D3A.2.2	1177	890	1224	1788	3.39E-06	3.78E-06	1.82E-06	3.46E-06
Y80D3A.3	15	9	26	13	3.43E-05	2.42E-05	2.21E-05	4.02E-05
Y80D3A.4	23	12	18	13	2.96E-05	2.12E-05	2.00E-05	3.61E-05
Y80D3A.5	51	92	39	44	2.80E-06	2.65E-06	1.97E-06	2.25E-06
Y80D3A.7	13	79	34	48	2.80E-06	2.65E-06	1.82E-06	2.25E-06
Y80D3A.8	35	52	40	22	3.72E-06	6.32E-06	1.84E-06	2.56E-06
Y80D3A.9	75	78	74	77	2.80E-06	3.23E-06	1.82E-06	2.25E-06
Y81B9A.1	3	5	2	1	2.80E-06	2.65E-06	1.82E-06	2.25E-06
Y81B9A.2	4	4	3	6	1.00E-05	9.81E-06	6.41E-06	8.25E-06
Y81B9A.3	3	2	2	1	2.80E-06	2.65E-06	1.82E-06	2.25E-06
Y81B9A.4	2	2	3	1	2.80E-06	2.65E-06	1.82E-06	2.25E-06
Y81G3A.1	22	73	18	18	2.80E-06	2.65E-06	1.82E-06	2.25E-06

Y81G3A.2	13	15	26	8	2.80E-06	2.65E-06	1.82E-06	2.25E-06
Y81G3A.3	358	430	338	585	3.36E-06	1.06E-05	1.82E-06	2.25E-06
Y81G3A.4	12	18	4	4	2.80E-06	2.65E-06	1.82E-06	2.25E-06
Y81G3A.5	5	12	9	8	7.28E-06	8.25E-06	4.46E-06	9.54E-06
Y82E9BL.1	0	2	2	1	2.80E-06	2.65E-06	1.82E-06	2.25E-06
Y82E9BL.10	35	53	30	27	2.80E-06	2.65E-06	1.82E-06	2.25E-06
Y82E9BL.11	14	18	10	10	2.80E-06	2.65E-06	1.82E-06	2.25E-06
Y82E9BL.12	5	2	4	1	4.31E-06	6.14E-06	2.41E-06	2.65E-06
Y82E9BL.13	22	30	13	10	2.80E-06	2.65E-06	1.82E-06	2.25E-06
Y82E9BL.14	13	22	13	4	2.80E-06	2.65E-06	1.82E-06	2.25E-06
Y82E9BL.15	9	25	10	8	2.80E-06	3.52E-06	1.82E-06	2.25E-06
Y82E9BL.16	2	5	8	2	2.80E-06	2.65E-06	1.82E-06	2.25E-06
Y82E9BL.17	10	12	7	9	2.80E-06	3.23E-06	1.82E-06	2.25E-06
Y82E9BL.18	13	9	8	9	2.80E-06	2.65E-06	1.82E-06	2.25E-06
Y82E9BL.2	1	1	6	1	2.80E-06	2.65E-06	1.82E-06	2.25E-06
Y82E9BL.3	2	3	7	0	2.80E-06	2.65E-06	1.82E-06	2.25E-06
Y82E9BL.4	2	6	6	1	2.80E-06	2.65E-06	1.82E-06	2.25E-06
Y82E9BL.5	0	4	2	2	2.80E-06	2.65E-06	1.82E-06	2.25E-06
Y82E9BL.6	2	2	4	3	2.80E-06	2.65E-06	1.82E-06	2.25E-06
Y82E9BL.7	7	15	13	18	2.80E-06	2.65E-06	1.82E-06	2.25E-06
Y82E9BL.8	12	8	15	11	2.80E-06	2.65E-06	1.82E-06	2.25E-06
Y82E9BL.9	1	9	1	1	2.80E-06	2.65E-06	1.82E-06	2.25E-06
Y82E9BR.1	9	24	24	20	2.80E-06	2.65E-06	1.82E-06	2.25E-06
Y82E9BR.10	1	3	7	4	2.80E-06	2.65E-06	1.82E-06	2.25E-06
Y82E9BR.11	2	6	2	2	2.80E-06	2.65E-06	1.82E-06	2.25E-06
Y82E9BR.12	8	9	9	8	2.80E-06	2.65E-06	1.82E-06	2.25E-06
Y82E9BR.13	24	33	22	15	2.80E-06	2.65E-06	1.82E-06	2.25E-06
Y82E9BR.14a	251	325	336	333	2.80E-06	2.65E-06	1.82E-06	2.25E-06
Y82E9BR.14b	195	261	290	291	3.47E-06	4.52E-06	2.08E-06	2.25E-06
Y82E9BR.15	69	79	310	152	3.07E-05	3.75E-05	2.67E-05	3.27E-05
Y82E9BR.16a	361	295	538	541	2.03E-05	2.57E-05	1.97E-05	2.44E-05
Y82E9BR.16b	51	55	109	129	1.52E-05	1.65E-05	4.45E-05	2.69E-05
Y82E9BR.17a	32	16	17	39	2.19E-05	1.69E-05	2.12E-05	2.64E-05
Y82E9BR.17b	14	3	5	11	9.58E-06	9.76E-06	1.33E-05	1.94E-05
Y82E9BR.18	231	311	228	416	2.80E-06	2.65E-06	1.82E-06	2.43E-06
Y82E9BR.19	289	223	315	530	2.80E-06	2.65E-06	1.82E-06	2.25E-06
Y82E9BR.2	103	86	85	199	8.65E-06	1.10E-05	5.56E-06	1.25E-05
Y82E9BR.20	7	3	10	5	2.58E-05	1.88E-05	1.83E-05	3.80E-05
Y82E9BR.21	21	15	6	9	7.25E-06	5.71E-06	3.88E-06	1.12E-05
Y82E9BR.22	45	40	138	60	2.80E-06	2.65E-06	1.88E-06	2.25E-06
Y82E9BR.23	15	10	10	3	2.80E-06	2.65E-06	1.82E-06	2.25E-06
Y82E9BR.3.1	6619	6252	16862	5005	2.40E-05	2.02E-05	4.79E-05	2.57E-05
Y82E9BR.3.2	5589	5411	14085	4624	2.80E-06	2.65E-06	1.82E-06	2.25E-06
Y82E9BR.3.3	4397	4528	9907	3977	1.21E-03	1.08E-03	2.01E-03	7.37E-04
Y82E9BR.3.4	5490	5351	13712	4589	1.16E-03	1.06E-03	1.91E-03	7.73E-04
Y82E9BR.4	14	13	16	13	1.32E-03	1.29E-03	1.94E-03	9.62E-04
Y82E9BR.5	3	1	2	2	1.02E-03	9.39E-04	1.66E-03	6.85E-04
Y82E9BR.6	0	3	1	1	2.80E-06	2.65E-06	1.82E-06	2.25E-06
Y82E9BR.7	5	2	5	3	2.80E-06	2.65E-06	1.82E-06	2.25E-06
Y82E9BR.8	0	2	0	2	2.80E-06	2.65E-06	1.82E-06	2.25E-06
Y82E9BR.9	9	11	19	8	2.80E-06	2.65E-06	1.82E-06	2.25E-06
Y87G2A.1	265	304	455	649	2.80E-06	2.65E-06	1.82E-06	2.25E-06
Y87G2A.10.1	213	314	231	268	2.80E-06	2.78E-06	3.30E-06	2.25E-06
Y87G2A.10.2	187	273	164	210	1.23E-05	1.34E-05	1.38E-05	2.43E-05
Y87G2A.11	111	138	125	161	3.45E-05	4.81E-05	2.44E-05	3.49E-05
Y87G2A.12	18	20	19	30	3.27E-05	4.51E-05	1.87E-05	2.95E-05
Y87G2A.13.1	157	234	390	450	1.14E-05	1.34E-05	8.36E-06	1.33E-05
Y87G2A.14	6	12	6	15	2.80E-06	2.65E-06	1.82E-06	2.25E-06
Y87G2A.15	5	7	3	1	9.83E-06	1.38E-05	1.59E-05	2.26E-05

Y87G2A.16	18	9	12	18	2.80E-06	2.65E-06	1.82E-06	2.25E-06
Y87G2A.17	37	56	56	53	2.80E-06	2.65E-06	1.82E-06	2.25E-06
Y87G2A.18	73	58	127	102	2.80E-06	2.65E-06	1.82E-06	2.25E-06
Y87G2A.19	35	74	52	19	7.22E-06	1.03E-05	7.12E-06	8.32E-06
Y87G2A.2	42	65	37	47	2.29E-05	1.72E-05	2.59E-05	2.57E-05
Y87G2A.20	35	26	24	16	7.78E-06	1.55E-05	7.53E-06	3.40E-06
Y87G2A.3	207	234	217	302	5.57E-06	8.12E-06	3.19E-06	4.99E-06
Y87G2A.4	44	46	32	38	6.61E-06	4.63E-06	2.95E-06	2.43E-06
Y87G2A.5	2199	2151	2097	3134	1.33E-05	1.42E-05	9.09E-06	1.56E-05
Y87G2A.6.1	325	333	349	486	5.21E-06	5.16E-06	2.46E-06	3.62E-06
Y87G2A.6.2	298	299	331	468	6.90E-05	6.38E-05	4.28E-05	7.90E-05
Y87G2A.7	216	229	458	500	1.70E-05	1.64E-05	1.19E-05	2.04E-05
Y87G2A.8a.1	911	1169	982	1577	1.76E-05	1.67E-05	1.27E-05	2.22E-05
Y87G2A.8a.2	914	1175	983	1580	1.53E-05	1.53E-05	2.11E-05	2.84E-05
Y87G2A.8a.3	914	1169	984	1579	5.97E-05	7.24E-05	4.19E-05	8.31E-05
Y8A9A.1	1	6	4	2	6.11E-05	7.42E-05	4.27E-05	8.48E-05
Y8A9A.2	204	303	96	133	6.17E-05	7.46E-05	4.33E-05	8.57E-05
Y8A9A.3	9	5	3	4	2.80E-06	2.65E-06	1.82E-06	2.25E-06
Y8A9A.4	2	2	1	3	5.49E-06	7.70E-06	1.82E-06	2.88E-06
Y8A9A.5	3	5	2	4	2.80E-06	2.65E-06	1.82E-06	2.25E-06
Y8A9A.6	0	2	2	0	2.80E-06	2.65E-06	1.82E-06	2.25E-06
Y8G1A.1a	11	7	2	4	2.80E-06	2.65E-06	1.82E-06	2.25E-06
Y8G1A.1b	5	3	3	2	2.80E-06	2.65E-06	1.82E-06	2.25E-06
Y8G1A.2	359	542	385	488	2.80E-06	2.65E-06	1.82E-06	2.25E-06
Y92C3A.3	1	1	1	1	2.80E-06	2.65E-06	1.82E-06	2.25E-06
Y92C3A.t2	0	0	1	0	2.82E-05	4.02E-05	1.97E-05	3.08E-05
Y92C3A.t3	0	0	1	0	2.80E-06	2.65E-06	1.82E-06	2.25E-06
Y92C3B.1	131	160	690	209	2.80E-06	2.65E-06	1.82E-06	2.25E-06
Y92C3B.2a.1	798	736	631	1055	2.80E-06	2.65E-06	1.82E-06	2.25E-06
Y92C3B.2a.2	781	719	599	1017	4.19E-05	4.84E-05	1.44E-04	5.37E-05
Y92C3B.2b.1	320	281	242	417	5.21E-05	4.54E-05	2.68E-05	5.54E-05
Y92C3B.2b.2	320	282	246	422	5.22E-05	4.54E-05	2.60E-05	5.46E-05
Y92C3B.2b.3	285	237	166	368	5.22E-05	4.33E-05	2.57E-05	5.47E-05
Y92C3B.2c	659	585	483	864	4.91E-05	4.09E-05	2.46E-05	5.20E-05
Y92C3B.2d	691	618	489	881	5.53E-05	4.35E-05	2.10E-05	5.74E-05
Y92C3B.3a	40	60	129	135	5.18E-05	4.34E-05	2.47E-05	5.45E-05
Y92C3B.3b	218	177	1045	429	5.47E-05	4.62E-05	2.52E-05	5.60E-05
Y92H12A.1	289	281	396	624	7.64E-06	1.08E-05	1.61E-05	2.07E-05
Y92H12A.2	132	200	107	173	2.99E-05	2.30E-05	9.33E-05	4.73E-05
Y92H12A.3	68	48	61	105	1.23E-05	1.13E-05	1.10E-05	2.14E-05
Y92H12A.4	157	137	181	284	5.74E-06	8.20E-06	3.02E-06	6.05E-06
Y92H12A.5	315	300	463	812	9.63E-06	6.43E-06	5.61E-06	1.19E-05
Y92H12A.t1	0	0	1	1	7.48E-06	6.16E-06	5.61E-06	1.09E-05
Y92H12BL.1	67	130	310	383	8.54E-06	7.70E-06	8.18E-06	1.77E-05
Y92H12BL.2	4	5	15	8	2.80E-06	2.65E-06	1.82E-06	2.25E-06
Y92H12BL.4	88	99	138	194	4.48E-06	8.20E-06	1.35E-05	2.06E-05
Y92H12BL.5	40	54	257	122	2.80E-06	2.72E-06	5.61E-06	3.69E-06
Y92H12BM.1	58	52	58	84	1.22E-05	1.29E-05	1.24E-05	2.15E-05
Y92H12BR.2	246	226	214	276	6.75E-06	8.60E-06	2.82E-05	1.65E-05
Y92H12BR.3a	223	316	547	791	3.01E-05	2.55E-05	1.96E-05	3.50E-05
Y92H12BR.3b	84	120	211	263	4.11E-05	3.57E-05	2.33E-05	3.71E-05
Y92H12BR.4	1	1	2	1	1.30E-05	1.75E-05	2.08E-05	3.72E-05
Y92H12BR.6	82	100	102	104	1.15E-05	1.56E-05	1.88E-05	2.90E-05
Y92H12BR.7	53	48	132	183	2.80E-06	2.65E-06	1.82E-06	2.25E-06
Y92H12BR.8	163	204	290	289	7.31E-06	8.41E-06	5.92E-06	7.44E-06
Y94A7B.1	2	5	2	3	3.44E-06	2.94E-06	5.58E-06	9.54E-06
Y94A7B.3	2	4	1	0	2.00E-05	2.37E-05	2.32E-05	2.86E-05
Y94A7B.4	3	1	5	3	2.80E-06	2.65E-06	1.82E-06	2.25E-06
Y94A7B.5	2	3	3	5	2.80E-06	2.65E-06	1.82E-06	2.25E-06

Y94A7B.6	1	2	5	1	2.80E-06	2.65E-06	1.82E-06	2.25E-06
Y94A7B.7	1	7	2	5	2.80E-06	2.65E-06	1.82E-06	2.25E-06
Y94A7B.8	2	5	4	0	2.80E-06	2.65E-06	1.82E-06	2.25E-06
Y94A7B.9	4	3	2	3	2.80E-06	2.65E-06	1.82E-06	2.25E-06
Y94H6A.1	7	14	4	5	2.80E-06	2.65E-06	1.82E-06	2.25E-06
Y94H6A.10	590	942	218	258	2.80E-06	2.65E-06	1.82E-06	2.25E-06
Y94H6A.11	77	40	176	194	2.80E-06	2.65E-06	1.82E-06	2.25E-06
Y94H6A.2	6	2	2	8	1.02E-04	1.54E-04	2.45E-05	3.58E-05
Y94H6A.3	127	117	264	209	9.97E-06	4.89E-06	1.49E-05	2.02E-05
Y94H6A.4	7	4	28	4	2.80E-06	2.65E-06	1.82E-06	2.25E-06
Y94H6A.5a	259	217	151	273	2.10E-05	1.83E-05	2.84E-05	2.77E-05
Y94H6A.5b	261	217	153	274	2.80E-06	2.65E-06	3.01E-06	2.25E-06
Y94H6A.6	10	21	26	40	1.13E-05	8.94E-06	4.28E-06	9.56E-06
Y94H6A.7	24	23	41	20	1.11E-05	8.76E-06	4.25E-06	9.40E-06
Y94H6A.8.1	452	527	682	515	2.80E-06	3.33E-06	2.84E-06	5.40E-06
Y94H6A.8.2	391	476	541	452	4.17E-06	3.76E-06	4.63E-06	2.79E-06
Y94H6A.9a	424	319	514	619	8.93E-05	9.83E-05	8.77E-05	8.17E-05
Y94H6A.9b	511	415	636	785	8.13E-05	9.34E-05	7.32E-05	7.54E-05
Y94H6A.t1	1	0	1	2	5.03E-05	3.57E-05	3.96E-05	5.89E-05
Y95B8A.1	31	60	19	29	4.82E-05	3.70E-05	3.90E-05	5.95E-05
Y95B8A.11	53	75	103	135	2.80E-06	2.65E-06	1.82E-06	2.47E-06
Y95B8A.12a	16	29	16	18	2.80E-06	2.99E-06	1.82E-06	2.25E-06
Y95B8A.12b	17	29	16	17	4.54E-06	6.06E-06	5.74E-06	9.29E-06
Y95B8A.2	42	95	38	24	2.80E-06	2.65E-06	1.82E-06	2.25E-06
Y95B8A.4	13	19	4	4	2.80E-06	2.65E-06	1.82E-06	2.25E-06
Y95B8A.5	200	146	321	479	5.68E-06	1.21E-05	3.35E-06	2.61E-06
Y95B8A.6a.1	35	45	45	40	2.80E-06	3.12E-06	1.82E-06	2.25E-06
Y95B8A.6a.2	32	42	46	37	2.09E-05	1.44E-05	2.18E-05	4.01E-05
Y95B8A.6b	15	15	19	17	2.80E-06	3.39E-06	2.33E-06	2.56E-06
Y95B8A.7	135	164	428	602	3.33E-06	4.13E-06	3.12E-06	3.08E-06
Y95B8A.8	67	27	111	74	2.80E-06	2.65E-06	1.97E-06	2.25E-06
Y95D11A.2	16	18	25	22	7.90E-06	9.05E-06	1.63E-05	2.82E-05
Y95D11A.t1	0	0	1	0	1.80E-05	6.85E-06	1.94E-05	1.60E-05
Y97E10AL.1	4	16	17	4	3.42E-06	3.62E-06	3.46E-06	3.78E-06
Y97E10AL.2.1	504	621	712	761	2.80E-06	2.65E-06	1.82E-06	2.25E-06
Y97E10AL.2.2	459	574	653	737	2.80E-06	2.65E-06	1.82E-06	2.25E-06
Y97E10AL.3	257	264	536	207	4.81E-05	5.60E-05	4.42E-05	5.83E-05
Y97E10AM.1	1	3	1	0	4.73E-05	5.58E-05	4.37E-05	6.09E-05
Y97E10AR.1	32	51	49	78	4.70E-05	4.56E-05	6.38E-05	3.04E-05
Y97E10AR.2a	39	37	35	51	2.80E-06	2.65E-06	1.82E-06	2.25E-06
Y97E10AR.2b	39	32	30	45	2.80E-06	2.65E-06	1.82E-06	2.86E-06
Y97E10AR.3	189	194	181	252	2.80E-06	2.65E-06	1.82E-06	2.27E-06
Y97E10AR.4	264	289	405	418	2.80E-06	2.65E-06	1.82E-06	2.25E-06
Y97E10AR.5	165	212	333	182	2.26E-05	2.19E-05	1.41E-05	2.42E-05
Y97E10AR.6	442	661	489	724	1.75E-05	1.81E-05	1.74E-05	2.22E-05
Y97E10AR.7.[illegible]	166	227	350	200	2.86E-05	3.48E-05	3.76E-05	2.54E-05
Y97E10AR.7.[illegible]	152	213	267	173	3.14E-05	4.43E-05	2.26E-05	4.12E-05
Y97E10AR.8	3	2	1	0	3.67E-05	4.75E-05	5.04E-05	3.56E-05
Y97E10B.1	8	13	15	7	3.23E-05	4.27E-05	3.69E-05	2.95E-05
Y97E10B.10	4	2	5	2	2.80E-06	2.65E-06	1.82E-06	2.25E-06
Y97E10B.2	9	1	8	5	2.80E-06	2.65E-06	1.82E-06	2.25E-06
Y97E10B.3	5	7	7	2	2.80E-06	2.65E-06	1.82E-06	2.25E-06
Y97E10B.4	3	5	11	2	2.80E-06	2.65E-06	1.82E-06	2.25E-06
Y97E10B.6	16	12	20	17	2.80E-06	2.65E-06	1.82E-06	2.25E-06
Y97E10B.7	9	20	8	5	2.80E-06	2.65E-06	1.82E-06	2.25E-06
Y97E10B.8	3	1	2	0	2.80E-06	2.65E-06	1.82E-06	2.25E-06
Y97E10B.9	5	7	4	5	2.80E-06	2.65E-06	1.82E-06	2.25E-06
Y97E10C.1.1	445	506	288	447	2.80E-06	2.65E-06	1.82E-06	2.25E-06
Y97E10C.1.2	254	297	144	266	2.80E-06	2.65E-06	1.82E-06	2.25E-06

Y9C12A.1	6	7	9	7	4.27E-05	4.58E-05	1.80E-05	3.44E-05
Y9C2UA.1a	19	65	37	34	4.54E-05	5.01E-05	1.67E-05	3.82E-05
Y9C2UA.1b	23	69	41	40	2.80E-06	2.65E-06	1.82E-06	2.25E-06
Y9C2UA.2	0	8	3	2	2.80E-06	6.43E-06	2.51E-06	2.86E-06
Y9C9A.1	9	6	1	3	2.80E-06	5.63E-06	2.31E-06	2.77E-06
Y9C9A.10	2	5	4	3	2.80E-06	2.72E-06	1.82E-06	2.25E-06
Y9C9A.11	5	7	7	2	2.80E-06	2.65E-06	1.82E-06	2.25E-06
Y9C9A.12	3	7	9	13	2.80E-06	2.65E-06	1.82E-06	2.25E-06
Y9C9A.13	16	12	27	10	2.80E-06	2.65E-06	1.82E-06	2.25E-06
Y9C9A.14	19	17	21	13	2.80E-06	2.65E-06	1.82E-06	2.25E-06
Y9C9A.15	8	8	12	3	2.80E-06	2.65E-06	1.82E-06	2.25E-06
Y9C9A.16	7	3	4	5	2.80E-06	2.65E-06	1.82E-06	2.25E-06
Y9C9A.17	12	5	11	4	2.80E-06	2.65E-06	1.82E-06	2.25E-06
Y9C9A.18	9	6	5	2	2.80E-06	2.65E-06	1.82E-06	2.25E-06
Y9C9A.2	2	4	5	2	2.80E-06	2.65E-06	1.82E-06	2.25E-06
Y9C9A.3	9	13	5	3	2.80E-06	2.65E-06	1.82E-06	2.25E-06
Y9C9A.4a	3	5	5	4	2.80E-06	2.65E-06	1.82E-06	2.25E-06
Y9C9A.4b	3	6	4	3	2.80E-06	2.65E-06	1.82E-06	2.25E-06
Y9C9A.5	6	7	11	1	2.80E-06	2.65E-06	1.82E-06	2.25E-06
Y9C9A.6	17	7	8	7	2.80E-06	2.65E-06	1.82E-06	2.25E-06
Y9C9A.7	4	2	4	7	2.80E-06	2.65E-06	1.82E-06	2.25E-06
Y9C9A.8	19	22	13	12	2.80E-06	2.65E-06	1.82E-06	2.25E-06
Y9C9A.9	5	10	8	8	2.80E-06	2.65E-06	1.82E-06	2.25E-06
Y9D1A.1	26	61	35	48	2.80E-06	2.65E-06	1.82E-06	2.25E-06
Y9D1A.2	15	24	14	38	2.80E-06	2.65E-06	1.82E-06	2.25E-06
ZC101.1	106	117	79	79	2.80E-06	4.71E-06	1.86E-06	3.15E-06
ZC101.2a	2828	2832	2207	4326	3.70E-06	5.58E-06	2.24E-06	7.49E-06
ZC101.2b	1116	1173	868	1561	4.37E-06	4.55E-06	2.11E-06	2.61E-06
ZC101.2c	2595	2598	2045	3972	4.20E-05	3.97E-05	2.13E-05	5.16E-05
ZC101.2e	2878	2878	2241	4337	3.16E-05	3.14E-05	1.60E-05	3.56E-05
ZC101.2f	3086	3132	2316	4606	4.16E-05	3.94E-05	2.14E-05	5.12E-05
ZC101.3	73	127	41	31	3.11E-05	2.94E-05	1.57E-05	3.76E-05
ZC116.1	94	98	89	57	4.51E-05	4.33E-05	2.20E-05	5.41E-05
ZC116.2	59	141	19	20	4.96E-06	8.15E-06	1.82E-06	2.25E-06
ZC116.3	170	190	188	270	1.55E-05	1.52E-05	9.53E-06	7.53E-06
ZC116.4	1	4	7	2	1.69E-05	3.82E-05	3.55E-06	4.61E-06
ZC123.1	139	181	69	69	2.80E-06	2.65E-06	1.82E-06	2.25E-06
ZC123.3	156	208	172	228	2.80E-06	2.65E-06	1.82E-06	2.25E-06
ZC123.4a	36	41	30	44	6.52E-06	8.01E-06	2.10E-06	2.59E-06
ZC123.4b	37	40	37	48	3.58E-06	4.52E-06	2.57E-06	4.21E-06
ZC123.4c	36	42	33	48	2.80E-06	2.65E-06	1.82E-06	2.38E-06
ZC13.10	1	5	5	3	2.80E-06	2.65E-06	1.82E-06	2.61E-06
ZC13.1a	46	73	66	102	2.80E-06	2.65E-06	1.82E-06	2.47E-06
ZC13.1b	40	63	59	94	2.80E-06	2.65E-06	1.82E-06	2.25E-06
ZC13.2	13	19	18	19	3.64E-06	5.45E-06	3.39E-06	6.45E-06
ZC13.3	57	68	34	26	3.72E-06	5.53E-06	3.57E-06	7.02E-06
ZC13.4	103	145	29	26	2.80E-06	2.65E-06	1.82E-06	2.25E-06
ZC132.2	2	4	2	1	2.80E-06	2.94E-06	1.82E-06	2.25E-06
ZC132.3a	9	13	26	10	1.06E-05	1.42E-05	1.95E-06	2.25E-06
ZC132.3b	16	18	40	12	2.80E-06	2.65E-06	1.82E-06	2.25E-06
ZC132.4	23	27	21	30	2.80E-06	2.65E-06	1.82E-06	2.25E-06
ZC132.5	21	16	18	15	2.80E-06	2.65E-06	1.82E-06	2.25E-06
ZC132.6	6	6	5	6	2.80E-06	2.65E-06	1.82E-06	2.25E-06
ZC132.7	3	3	8	6	2.80E-06	2.65E-06	1.82E-06	2.25E-06
ZC132.8	2	2	5	2	2.80E-06	2.65E-06	1.82E-06	2.25E-06
ZC132.9	2	3	3	1	2.80E-06	2.65E-06	1.82E-06	2.25E-06
ZC132.t1	0	0	1	1	2.80E-06	2.65E-06	1.82E-06	2.25E-06
ZC142.1	2	4	9	2	2.80E-06	2.65E-06	1.82E-06	2.25E-06
ZC142.2	9	10	5	2	2.80E-06	2.65E-06	1.82E-06	2.25E-06

ZC15.1	4	11	1	9	2.80E-06	2.65E-06	1.82E-06	2.25E-06
ZC15.2	1	2	2	4	2.80E-06	2.65E-06	1.82E-06	2.25E-06
ZC15.3	2	3	5	7	2.80E-06	2.65E-06	1.82E-06	2.25E-06
ZC15.4	3	5	9	4	2.80E-06	2.65E-06	1.82E-06	2.25E-06
ZC15.5	30	14	38	21	2.80E-06	2.65E-06	1.82E-06	2.25E-06
ZC15.6	2	3	1	0	2.80E-06	2.65E-06	1.82E-06	2.25E-06
ZC15.7	32	34	57	44	2.80E-06	2.65E-06	1.82E-06	2.25E-06
ZC15.8.1	178	121	207	57	2.80E-06	2.65E-06	1.82E-06	2.25E-06
ZC15.8.2	142	90	175	51	2.80E-06	2.65E-06	2.10E-06	2.25E-06
ZC155.1.1	1314	1541	1773	2128	5.42E-05	3.48E-05	4.10E-05	1.39E-05
ZC155.1.2	1090	1282	1429	1863	4.21E-05	2.52E-05	3.37E-05	1.21E-05
ZC155.2	13	17	6	4	1.31E-04	1.45E-04	1.15E-04	1.70E-04
ZC155.3	581	558	653	1089	1.15E-04	1.28E-04	9.81E-05	1.58E-04
ZC155.4	412	478	427	447	2.80E-06	2.80E-06	1.82E-06	2.25E-06
ZC155.5a	3	2	5	7	2.55E-05	2.31E-05	1.86E-05	3.83E-05
ZC155.5b	3	3	5	4	3.53E-05	3.87E-05	2.38E-05	3.08E-05
ZC155.7	249	210	131	260	2.80E-06	2.65E-06	1.82E-06	2.25E-06
ZC168.1	98	88	76	157	2.80E-06	2.65E-06	1.82E-06	2.25E-06
ZC168.2	0	4	3	1	2.21E-05	1.76E-05	7.56E-06	1.85E-05
ZC168.3	370	302	502	712	4.12E-06	3.49E-06	2.08E-06	5.31E-06
ZC168.4.1	1801	1878	2926	3558	2.80E-06	2.65E-06	1.82E-06	2.25E-06
ZC168.4.2	1899	1970	3118	3670	3.48E-05	2.68E-05	3.07E-05	5.38E-05
ZC168.4.3	1890	1955	3111	3664	1.30E-04	1.28E-04	1.38E-04	2.07E-04
ZC168.4.4	1822	1883	2975	3594	1.36E-04	1.33E-04	1.45E-04	2.11E-04
ZC168.5	138	227	100	58	1.39E-04	1.36E-04	1.49E-04	2.16E-04
ZC168.6	68	72	69	21	1.38E-04	1.34E-04	1.46E-04	2.18E-04
ZC178.1	30	0	4	0	2.31E-05	3.58E-05	1.09E-05	7.78E-06
ZC178.2	15	0	50	24	2.06E-05	2.06E-05	1.36E-05	5.11E-06
ZC190.10	4	3	8	5	2.80E-06	2.65E-06	1.82E-06	2.25E-06
ZC190.2	2	3	5	6	2.80E-06	2.65E-06	1.82E-06	2.25E-06
ZC190.4	219	187	184	304	2.80E-06	2.65E-06	1.82E-06	2.25E-06
ZC190.5	6	10	6	10	2.80E-06	2.65E-06	1.82E-06	2.25E-06
ZC190.6	20	13	86	19	8.29E-06	6.69E-06	4.54E-06	9.24E-06
ZC190.7	13	11	15	6	2.80E-06	2.65E-06	1.82E-06	2.25E-06
ZC190.8	9	8	10	10	2.80E-06	2.65E-06	3.81E-06	2.25E-06
ZC190.9	2	1	4	4	2.80E-06	2.65E-06	1.90E-06	2.25E-06
ZC196.1	9	6	14	2	2.80E-06	2.65E-06	1.82E-06	2.25E-06
ZC196.2	9	9	9	6	2.80E-06	2.65E-06	1.82E-06	2.25E-06
ZC196.3	17	26	15	18	2.80E-06	2.65E-06	1.82E-06	2.25E-06
ZC196.4	16	16	19	17	2.80E-06	2.65E-06	1.82E-06	2.25E-06
ZC196.5	33	56	14	19	2.80E-06	2.65E-06	1.82E-06	2.25E-06
ZC196.6	10	15	19	12	2.80E-06	2.65E-06	1.82E-06	2.25E-06
ZC196.7	27	43	47	33	2.80E-06	3.49E-06	1.82E-06	2.25E-06
ZC196.8	8	6	10	10	2.80E-06	2.65E-06	1.82E-06	2.25E-06
ZC196.9	5	4	10	8	2.80E-06	2.65E-06	1.82E-06	2.25E-06
ZC204.1	3	0	0	0	2.80E-06	2.65E-06	1.82E-06	2.25E-06
ZC204.10	8	3	3	2	2.80E-06	2.65E-06	1.82E-06	2.25E-06
ZC204.11	1	3	4	3	2.80E-06	2.65E-06	1.82E-06	2.25E-06
ZC204.12	116	156	130	64	2.80E-06	2.65E-06	1.82E-06	2.25E-06
ZC204.13	4	3	4	2	2.80E-06	2.65E-06	1.82E-06	2.25E-06
ZC204.14	24	25	23	17	1.44E-05	1.84E-05	1.05E-05	6.41E-06
ZC204.15	8	4	11	3	2.80E-06	2.65E-06	1.82E-06	2.25E-06
ZC204.16	2	5	4	1	5.91E-06	5.79E-06	3.68E-06	3.35E-06
ZC204.2	45	35	46	75	2.80E-06	2.65E-06	1.82E-06	2.25E-06
ZC204.3	2	4	5	5	2.80E-06	2.65E-06	1.82E-06	2.25E-06
ZC204.4a	4	3	4	5	3.64E-06	2.67E-06	2.42E-06	4.88E-06
ZC204.4b	4	3	3	4	2.80E-06	2.65E-06	1.82E-06	2.25E-06
ZC204.5	1	2	3	1	2.80E-06	2.65E-06	1.82E-06	2.25E-06
ZC204.6	1	2	3	0	2.80E-06	2.65E-06	1.82E-06	2.25E-06

ZC204.7	8	9	4	7	2.80E-06	2.65E-06	1.82E-06	2.25E-06
ZC204.8	1	1	3	0	2.80E-06	2.65E-06	1.82E-06	2.25E-06
ZC204.9	3	2	7	3	2.80E-06	2.65E-06	1.82E-06	2.25E-06
ZC21.10	5	2	4	2	2.80E-06	2.65E-06	1.82E-06	2.25E-06
ZC21.2a	26	29	23	27	2.80E-06	2.65E-06	1.82E-06	2.25E-06
ZC21.2b	25	28	28	27	2.80E-06	2.65E-06	1.82E-06	2.25E-06
ZC21.3	184	207	105	98	2.80E-06	2.65E-06	1.82E-06	2.25E-06
ZC21.6a	9	12	9	8	2.80E-06	2.65E-06	1.82E-06	2.25E-06
ZC21.6b	9	15	11	11	2.02E-05	2.15E-05	7.51E-06	8.64E-06
ZC21.8	4	14	5	3	2.80E-06	2.65E-06	1.82E-06	2.25E-06
ZC21.9	12	16	4	2	2.80E-06	2.65E-06	1.82E-06	2.25E-06
ZC239.1	15	12	13	11	2.80E-06	3.39E-06	1.82E-06	2.25E-06
ZC239.10	3	4	4	1	2.80E-06	2.65E-06	1.82E-06	2.25E-06
ZC239.12	7	3	4	3	2.80E-06	2.65E-06	1.82E-06	2.25E-06
ZC239.13	6	5	16	5	2.80E-06	2.65E-06	1.82E-06	2.25E-06
ZC239.14	2	4	5	3	2.80E-06	2.65E-06	1.82E-06	2.25E-06
ZC239.15	9	14	14	7	2.80E-06	2.65E-06	2.77E-06	2.25E-06
ZC239.16	17	20	29	9	2.80E-06	2.65E-06	1.82E-06	2.25E-06
ZC239.17	5	13	13	12	2.80E-06	2.65E-06	1.82E-06	2.25E-06
ZC239.19	2	2	2	1	4.06E-06	4.52E-06	4.52E-06	2.25E-06
ZC239.2	1	3	5	4	2.80E-06	2.72E-06	1.88E-06	2.25E-06
ZC239.20	1	7	4	0	2.80E-06	2.65E-06	1.82E-06	2.25E-06
ZC239.21	2	5	5	4	2.80E-06	2.65E-06	1.82E-06	2.25E-06
ZC239.3	2	0	2	2	2.80E-06	2.65E-06	1.82E-06	2.25E-06
ZC239.4	4	5	4	4	2.80E-06	2.65E-06	1.82E-06	2.25E-06
ZC239.5	6	5	9	2	2.80E-06	2.65E-06	1.82E-06	2.25E-06
ZC239.6.1	48	60	78	57	2.80E-06	2.65E-06	1.82E-06	2.25E-06
ZC239.6.2	35	52	69	55	2.80E-06	2.65E-06	1.82E-06	2.25E-06
ZC239.6.3	33	51	67	54	3.08E-06	3.62E-06	3.26E-06	2.95E-06
ZC239.7	13	20	15	6	2.80E-06	3.44E-06	3.15E-06	3.10E-06
ZC239.8	4	4	2	2	2.80E-06	3.44E-06	3.12E-06	3.10E-06
ZC239.9	2	7	5	2	2.80E-06	2.65E-06	1.82E-06	2.25E-06
ZC247.1	12304	18242	9813	15824	2.80E-06	2.65E-06	1.82E-06	2.25E-06
ZC247.2	66	75	34	45	2.80E-06	2.65E-06	1.82E-06	2.25E-06
ZC247.3	43	46	38	38	1.03E-04	1.44E-04	5.35E-05	1.07E-04
ZC250.1	68	95	29	26	3.16E-06	3.41E-06	1.82E-06	2.25E-06
ZC250.2	14	22	12	18	3.16E-06	3.20E-06	1.82E-06	2.25E-06
ZC250.3	21	33	15	12	4.00E-06	5.29E-06	1.82E-06	2.25E-06
ZC262.1	1	8	2	1	2.80E-06	2.65E-06	1.82E-06	2.25E-06
ZC262.2a	187	191	164	199	2.80E-06	3.39E-06	1.82E-06	2.25E-06
ZC262.2b	138	152	125	140	2.80E-06	4.63E-06	1.82E-06	2.25E-06
ZC262.3a	1012	1119	1019	1831	2.98E-05	2.88E-05	1.70E-05	2.55E-05
ZC262.3b	690	742	688	1285	2.61E-05	2.71E-05	1.54E-05	2.12E-05
ZC262.4	2	5	2	3	4.22E-05	4.41E-05	2.76E-05	6.13E-05
ZC262.5	45	45	75	24	3.72E-05	3.77E-05	2.41E-05	5.56E-05
ZC262.8.1	343	375	293	225	2.80E-06	2.65E-06	1.82E-06	2.25E-06
ZC262.8.2	993	1040	951	1445	3.05E-05	2.89E-05	3.31E-05	1.31E-05
ZC266.1	11	10	8	8	6.33E-05	6.54E-05	3.52E-05	3.34E-05
ZC266.2	18	17	11	29	4.62E-05	4.57E-05	2.88E-05	5.40E-05
ZC302.1.1	618	605	756	1106	3.05E-06	2.65E-06	1.82E-06	2.25E-06
ZC302.1.2	547	550	698	1042	2.80E-06	2.65E-06	1.82E-06	3.15E-06
ZC302.2a	547	577	914	1038	2.87E-05	2.66E-05	2.29E-05	4.13E-05
ZC302.3	6	18	12	8	2.79E-05	2.65E-05	2.32E-05	4.28E-05
ZC308.1a	2035	1606	1817	2955	3.52E-05	3.51E-05	3.83E-05	5.36E-05
ZC308.1b	1042	809	880	1505	2.80E-06	2.67E-06	1.82E-06	2.25E-06
ZC308.1c	1179	937	1007	1698	5.10E-05	3.81E-05	2.97E-05	5.95E-05
ZC308.1d	1150	899	994	1675	4.46E-05	3.27E-05	2.45E-05	5.17E-05
ZC308.4	382	430	761	575	4.24E-05	3.19E-05	2.36E-05	4.91E-05
ZC317.1	57	90	99	115	5.31E-05	3.92E-05	2.99E-05	6.22E-05

ZC317.2	2	3	2	4	4.32E-05	4.59E-05	5.60E-05	5.22E-05
ZC317.3	7	16	6	8	6.24E-06	9.31E-06	7.05E-06	1.01E-05
ZC317.4	0	5	1	0	2.80E-06	2.65E-06	1.82E-06	2.25E-06
ZC317.5	2	8	5	1	2.80E-06	2.65E-06	1.82E-06	2.25E-06
ZC317.6	35	110	25	26	2.80E-06	2.65E-06	1.82E-06	2.25E-06
ZC317.7	251	265	347	350	2.80E-06	2.65E-06	1.82E-06	2.25E-06
ZC328.2	40	70	52	35	3.75E-06	1.12E-05	1.82E-06	2.25E-06
ZC328.3a	136	299	141	203	1.49E-05	1.49E-05	1.34E-05	1.67E-05
ZC328.3b	75	181	75	115	2.86E-06	4.73E-06	2.42E-06	2.25E-06
ZC328.4	276	380	248	296	6.30E-06	1.31E-05	4.25E-06	7.53E-06
ZC328.5	1	4	3	5	7.25E-06	1.65E-05	4.72E-06	8.93E-06
ZC334.1	5	6	9	4	2.32E-05	3.01E-05	1.36E-05	2.00E-05
ZC334.10	2	3	2	0	2.80E-06	2.65E-06	1.82E-06	2.25E-06
ZC334.11	30	31	203	25	2.80E-06	2.65E-06	1.82E-06	2.25E-06
ZC334.2	6	8	8	2	2.80E-06	2.65E-06	1.82E-06	2.25E-06
ZC334.3	9	5	12	7	1.33E-05	1.30E-05	5.87E-05	8.93E-06
ZC334.7	5	3	2	2	2.80E-06	2.65E-06	1.82E-06	2.25E-06
ZC334.8	2	3	1	0	2.80E-06	2.65E-06	2.20E-06	2.25E-06
ZC334.9	1	3	12	0	2.80E-06	2.65E-06	1.82E-06	2.25E-06
ZC373.2	730	958	2420	1252	2.80E-06	2.65E-06	1.82E-06	2.25E-06
ZC373.3	4	6	6	3	2.80E-06	2.65E-06	4.28E-06	2.25E-06
ZC373.4	145	202	79	178	2.15E-04	2.67E-04	4.64E-04	2.96E-04
ZC373.5	12	21	20	27	2.80E-06	2.65E-06	1.82E-06	2.25E-06
ZC373.6	176	177	35	33	4.09E-06	5.37E-06	1.82E-06	4.03E-06
ZC373.7	280	176	148	77	2.80E-06	2.65E-06	1.82E-06	2.25E-06
ZC374.1	2	10	2	6	3.01E-05	2.86E-05	3.90E-06	4.54E-06
ZC374.2	32	49	33	53	3.34E-05	1.98E-05	1.15E-05	7.38E-06
ZC376.1	6	19	17	13	2.80E-06	2.65E-06	1.82E-06	2.25E-06
ZC376.2	153	144	230	191	2.80E-06	2.80E-06	1.82E-06	2.59E-06
ZC376.3	60	56	66	55	2.80E-06	2.65E-06	1.82E-06	2.25E-06
ZC376.4	21	31	32	32	8.46E-06	7.51E-06	8.27E-06	8.48E-06
ZC376.5	436	437	626	797	3.08E-06	2.70E-06	2.20E-06	2.27E-06
ZC376.6	496	532	628	1019	3.11E-06	4.31E-06	3.08E-06	3.80E-06
ZC376.7a.1	431	611	584	783	2.72E-05	2.58E-05	2.55E-05	4.00E-05
ZC376.7a.2	414	589	569	772	1.52E-05	1.54E-05	1.26E-05	2.51E-05
ZC376.7b.1	495	733	728	971	2.98E-05	3.99E-05	2.63E-05	4.35E-05
ZC376.7b.2	415	583	576	775	3.08E-05	4.14E-05	2.76E-05	4.62E-05
ZC376.7c	423	583	584	794	3.07E-05	4.30E-05	2.94E-05	4.84E-05
ZC376.8	22	41	22	12	3.19E-05	4.24E-05	2.88E-05	4.79E-05
ZC395.10.1	1424	1745	1278	1800	3.24E-05	4.22E-05	2.91E-05	4.88E-05
ZC395.10.2	1247	1524	990	1567	2.88E-06	5.08E-06	1.88E-06	2.25E-06
ZC395.11	1	0	1	1	2.19E-04	2.53E-04	1.28E-04	2.22E-04
ZC395.2	97	162	139	147	1.96E-04	2.26E-04	1.01E-04	1.97E-04
ZC395.3a.1	617	529	584	732	2.80E-06	2.65E-06	1.82E-06	2.25E-06
ZC395.3b	689	606	630	805	1.49E-05	2.35E-05	1.39E-05	1.81E-05
ZC395.4	6	12	4	3	4.89E-05	3.96E-05	3.01E-05	4.66E-05
ZC395.5	21	100	22	19	4.45E-05	3.70E-05	2.65E-05	4.18E-05
ZC395.6	115	137	110	143	2.80E-06	2.65E-06	1.82E-06	2.25E-06
ZC395.7	81	129	161	87	7.76E-06	3.49E-05	5.28E-06	5.65E-06
ZC395.8	517	604	670	772	9.24E-06	1.04E-05	5.74E-06	9.22E-06
ZC404.1	5	4	3	2	1.39E-05	2.08E-05	1.79E-05	1.19E-05
ZC404.10	3	2	2	3	2.43E-05	2.68E-05	2.05E-05	2.91E-05
ZC404.11	3	5	15	4	2.80E-06	2.65E-06	1.82E-06	2.25E-06
ZC404.12	4	15	4	3	2.80E-06	2.65E-06	1.82E-06	2.25E-06
ZC404.13	6	5	5	4	2.80E-06	2.65E-06	1.82E-06	2.25E-06
ZC404.3a	496	539	692	839	2.80E-06	2.65E-06	1.82E-06	2.25E-06
ZC404.3b	228	235	322	380	2.80E-06	2.65E-06	1.82E-06	2.25E-06
ZC404.5	5	6	8	4	2.94E-05	3.02E-05	2.67E-05	4.00E-05
ZC404.6	5	2	1	2	4.37E-05	4.25E-05	4.01E-05	5.84E-05

ZC404.7	2	1	1	0	2.80E-06	2.65E-06	1.82E-06	2.25E-06
ZC404.9	747	689	898	1337	2.80E-06	2.65E-06	1.82E-06	2.25E-06
ZC410.1a	17	70	9	32	2.80E-06	2.65E-06	1.82E-06	2.25E-06
ZC410.1b	17	58	5	23	2.85E-05	2.48E-05	2.23E-05	4.10E-05
ZC410.2.1	391	439	468	588	2.80E-06	5.08E-06	1.82E-06	2.25E-06
ZC410.2.2	361	396	408	542	2.80E-06	5.18E-06	1.82E-06	2.25E-06
ZC410.3	170	244	279	381	3.00E-05	3.18E-05	2.33E-05	3.62E-05
ZC410.4a	76	135	52	107	2.93E-05	3.03E-05	2.15E-05	3.53E-05
ZC410.4b	76	133	49	103	1.07E-05	1.45E-05	1.14E-05	1.92E-05
ZC410.5a	108	236	102	100	3.44E-06	5.77E-06	1.82E-06	3.89E-06
ZC410.5b	97	201	73	85	3.44E-06	5.69E-06	1.82E-06	3.76E-06
ZC410.7a.1	296	447	323	405	1.75E-05	3.61E-05	1.08E-05	1.30E-05
ZC410.7a.2	267	416	266	379	1.74E-05	3.41E-05	8.55E-06	1.23E-05
ZC410.7b	208	302	198	272	9.37E-05	1.34E-04	6.65E-05	1.03E-04
ZC410.7c	226	338	220	299	2.35E-05	3.46E-05	1.52E-05	2.68E-05
ZC411.1	1	3	3	1	2.47E-05	3.39E-05	1.53E-05	2.60E-05
ZC412.1	12	19	14	7	2.14E-05	3.03E-05	1.36E-05	2.28E-05
ZC412.2	10	10	17	9	2.80E-06	2.65E-06	1.82E-06	2.25E-06
ZC412.3.1	92	181	164	129	2.80E-06	2.65E-06	1.82E-06	2.25E-06
ZC412.3.2	75	148	104	108	2.80E-06	2.65E-06	1.82E-06	2.25E-06
ZC412.3.3	75	153	109	113	1.19E-05	2.21E-05	1.38E-05	1.34E-05
ZC412.4	12	33	9	32	1.11E-05	2.07E-05	1.00E-05	1.28E-05
ZC412.5	7	13	4	1	1.10E-05	2.13E-05	1.04E-05	1.34E-05
ZC412.6	100	237	272	91	2.80E-06	5.18E-06	1.82E-06	4.27E-06
ZC412.7	19	63	16	8	2.80E-06	2.72E-06	1.82E-06	2.25E-06
ZC412.8	8	14	5	2	5.71E-05	1.28E-04	1.01E-04	4.18E-05
ZC412.9	5	8	5	4	2.03E-05	6.35E-05	1.11E-05	6.86E-06
ZC416.1	4	14	15	11	3.56E-06	5.87E-06	1.82E-06	2.25E-06
ZC416.2	9	24	32	3	2.80E-06	2.65E-06	1.82E-06	2.25E-06
ZC416.4	10	12	6	13	2.80E-06	3.52E-06	2.61E-06	2.36E-06
ZC416.6	506	594	419	705	3.19E-06	8.07E-06	7.40E-06	2.25E-06
ZC416.8a	32	40	28	42	2.80E-06	2.65E-06	1.82E-06	2.25E-06
ZC416.8b	25	32	13	24	2.86E-05	3.17E-05	1.54E-05	3.20E-05
ZC434.2.1	16794	15682	30616	11580	2.80E-06	2.65E-06	1.82E-06	2.25E-06
ZC434.2.2	14809	14088	25125	10784	2.80E-06	2.65E-06	1.82E-06	2.25E-06
ZC434.3	101	141	60	31	2.89E-03	2.55E-03	3.43E-03	1.60E-03
ZC434.4	304	367	348	444	2.62E-03	2.36E-03	2.90E-03	1.54E-03
ZC434.9a	195	350	139	227	7.95E-06	1.05E-05	3.06E-06	2.25E-06
ZC434.9b	167	297	122	197	3.84E-05	4.38E-05	2.86E-05	4.51E-05
ZC443.1	30	34	15	22	1.11E-05	1.88E-05	5.14E-06	1.03E-05
ZC443.2	6	10	2	4	1.17E-05	1.97E-05	5.56E-06	1.11E-05
ZC443.3.1	36	127	23	47	3.42E-06	3.65E-06	1.82E-06	2.25E-06
ZC443.3.2	32	103	18	41	2.80E-06	2.65E-06	1.82E-06	2.25E-06
ZC443.4	34	71	25	53	2.80E-06	8.94E-06	1.82E-06	2.81E-06
ZC443.5	11	12	14	8	2.91E-06	8.89E-06	1.82E-06	3.01E-06
ZC443.6	108	195	78	109	2.80E-06	3.97E-06	1.82E-06	2.52E-06
ZC449.1	18	58	15	13	2.80E-06	2.65E-06	1.82E-06	2.25E-06
ZC449.2	21	19	13	13	6.80E-06	1.16E-05	3.19E-06	5.51E-06
ZC449.3a.1	96	108	101	116	2.80E-06	3.76E-06	1.82E-06	2.25E-06
ZC449.3a.2	92	103	91	113	2.80E-06	2.65E-06	1.82E-06	2.25E-06
ZC449.3b	126	166	133	153	8.88E-06	9.42E-06	6.07E-06	8.61E-06
ZC449.4	25	33	34	11	8.93E-06	9.44E-06	5.76E-06	8.82E-06
ZC449.5.1	182	210	89	137	8.60E-06	1.07E-05	5.90E-06	8.37E-06
ZC449.5.2	144	166	55	106	4.23E-06	5.26E-06	3.74E-06	2.25E-06
ZC449.6	7	6	0	2	1.00E-05	1.09E-05	3.19E-06	6.05E-06
ZC455.1	33	55	25	29	1.04E-05	1.13E-05	2.59E-06	6.16E-06
ZC455.10	69	75	26	29	2.80E-06	2.65E-06	1.82E-06	2.25E-06
ZC455.11	5	3	5	0	2.80E-06	2.65E-06	1.82E-06	2.25E-06
ZC455.12	3	2	8	2	7.98E-06	8.17E-06	1.95E-06	2.70E-06

ZC455.3	10	8	17	7	2.80E-06	2.65E-06	1.82E-06	2.25E-06
ZC455.4	67	111	65	62	2.80E-06	2.65E-06	1.82E-06	2.25E-06
ZC455.5a	24	53	37	37	2.80E-06	2.65E-06	1.82E-06	2.25E-06
ZC455.6a	114	186	102	90	4.48E-06	6.98E-06	2.82E-06	3.33E-06
ZC455.6b	29	36	25	24	2.80E-06	3.36E-06	1.82E-06	2.25E-06
ZC455.7	4	5	5	7	7.87E-06	1.21E-05	4.59E-06	4.99E-06
ZC455.8a	5	5	4	4	8.88E-06	1.04E-05	4.97E-06	5.89E-06
ZC455.9	4	5	15	11	2.80E-06	2.65E-06	1.82E-06	2.25E-06
ZC47.10	6	8	7	4	2.80E-06	2.65E-06	1.82E-06	2.25E-06
ZC47.11	1	5	9	3	2.80E-06	2.65E-06	1.82E-06	2.25E-06
ZC47.12	8	6	6	5	2.80E-06	2.65E-06	1.82E-06	2.25E-06
ZC47.14	10	25	13	19	2.80E-06	2.65E-06	1.82E-06	2.25E-06
ZC47.3	7	8	10	3	2.80E-06	2.65E-06	1.82E-06	2.25E-06
ZC47.4	7	11	8	7	2.80E-06	2.91E-06	1.82E-06	2.25E-06
ZC47.5	15	22	14	11	2.80E-06	2.65E-06	1.82E-06	2.25E-06
ZC47.6	9	9	5	3	2.80E-06	2.65E-06	1.82E-06	2.25E-06
ZC47.7	15	17	16	11	2.80E-06	2.65E-06	1.82E-06	2.25E-06
ZC47.8	2	4	2	3	2.80E-06	2.65E-06	1.82E-06	2.25E-06
ZC47.9	6	7	14	7	2.80E-06	2.65E-06	1.82E-06	2.25E-06
ZC477.1	100	195	48	43	2.80E-06	2.65E-06	1.82E-06	2.25E-06
ZC477.10	12	14	19	9	2.80E-06	2.65E-06	1.82E-06	2.25E-06
ZC477.2	12	28	13	17	9.88E-06	1.82E-05	3.08E-06	3.42E-06
ZC477.3a	252	374	226	380	2.80E-06	2.65E-06	1.82E-06	2.25E-06
ZC477.3b	161	247	151	254	2.80E-06	2.65E-06	1.82E-06	2.25E-06
ZC477.4	3	2	3	1	2.27E-05	3.18E-05	1.32E-05	2.75E-05
ZC477.5	263	326	293	363	2.32E-05	3.36E-05	1.42E-05	2.94E-05
ZC477.7	30	72	24	17	2.80E-06	2.65E-06	1.82E-06	2.25E-06
ZC477.9a	945	1129	778	1169	2.79E-05	3.26E-05	2.02E-05	3.09E-05
ZC477.9b	394	541	345	520	5.74E-06	1.30E-05	2.99E-06	2.61E-06
ZC477.9c	1034	1307	847	1300	3.38E-05	3.81E-05	1.81E-05	3.36E-05
ZC482.1	7	15	21	7	2.78E-05	3.61E-05	1.59E-05	2.95E-05
ZC482.2	3	8	15	4	3.25E-05	3.88E-05	1.73E-05	3.28E-05
ZC482.3	9	6	23	4	2.80E-06	2.65E-06	1.82E-06	2.25E-06
ZC482.4	11	10	16	7	2.80E-06	2.65E-06	1.82E-06	2.25E-06
ZC482.5	2	11	6	7	2.80E-06	2.65E-06	1.82E-06	2.25E-06
ZC482.6	2	5	7	7	2.80E-06	2.65E-06	1.82E-06	2.25E-06
ZC482.7	9	4	18	15	2.80E-06	2.65E-06	1.82E-06	2.25E-06
ZC482.8	12	12	7	1	2.80E-06	2.65E-06	1.82E-06	2.25E-06
ZC482.9	8	11	13	4	2.80E-06	2.65E-06	1.82E-06	2.25E-06
ZC487.1a	9	17	8	10	2.80E-06	2.65E-06	1.82E-06	2.25E-06
ZC487.1b	12	20	7	13	2.80E-06	2.65E-06	1.82E-06	2.25E-06
ZC487.2	6	4	6	1	2.80E-06	2.65E-06	1.82E-06	2.25E-06
ZC487.3	6	3	5	1	2.80E-06	2.65E-06	1.82E-06	2.25E-06
ZC487.4	10	17	28	6	2.80E-06	2.65E-06	1.82E-06	2.25E-06
ZC487.5	6	9	13	4	2.80E-06	2.65E-06	1.82E-06	2.25E-06
ZC504.1	5	7	3	2	2.80E-06	2.65E-06	2.04E-06	2.25E-06
ZC504.2	25	35	13	17	2.80E-06	2.65E-06	1.82E-06	2.25E-06
ZC504.3	109	103	147	220	2.80E-06	2.65E-06	1.82E-06	2.25E-06
ZC504.4a	218	373	162	346	2.80E-06	2.65E-06	1.82E-06	2.25E-06
ZC504.4b	177	304	144	308	5.29E-06	4.73E-06	4.65E-06	8.59E-06
ZC504.4c	217	368	162	342	6.30E-06	1.02E-05	3.04E-06	8.01E-06
ZC504.4d	176	297	139	306	5.96E-06	9.68E-06	3.15E-06	8.34E-06
ZC504.5	11	13	16	7	6.22E-06	9.95E-06	3.02E-06	7.87E-06
ZC506.1	332	424	242	490	5.99E-06	9.55E-06	3.08E-06	8.34E-06
ZC506.3.1	174	265	198	270	2.80E-06	2.65E-06	1.82E-06	2.25E-06
ZC506.3.3	151	223	175	244	1.13E-05	1.36E-05	5.36E-06	1.34E-05
ZC513.1	14	22	9	3	9.77E-06	1.40E-05	7.23E-06	1.22E-05
ZC513.10	10	10	7	9	1.11E-05	1.54E-05	8.33E-06	1.43E-05
ZC513.11	10	6	5	4	2.80E-06	2.72E-06	1.82E-06	2.25E-06

ZC513.12	54	65	32	19	2.80E-06	2.65E-06	1.82E-06	2.25E-06
ZC513.2	2	11	4	3	2.80E-06	2.65E-06	1.82E-06	2.25E-06
ZC513.3	28	31	19	9	1.68E-05	1.91E-05	6.49E-06	4.75E-06
ZC513.4	298	461	326	481	2.80E-06	2.65E-06	1.82E-06	2.25E-06
ZC513.5	314	389	587	681	2.80E-06	2.65E-06	1.82E-06	2.25E-06
ZC513.6	2785	2175	4422	4953	1.17E-05	1.71E-05	8.31E-06	1.51E-05
ZC513.8	176	254	130	160	2.03E-05	2.38E-05	2.47E-05	3.54E-05
ZC513.9	1	6	14	5	2.03E-04	1.50E-04	2.10E-04	2.91E-04
ZC518.1a	92	144	80	152	1.93E-05	2.63E-05	9.29E-06	1.41E-05
ZC518.1b	90	140	80	151	2.80E-06	2.65E-06	1.82E-06	2.25E-06
ZC518.2	1153	982	1114	1904	5.82E-06	8.60E-06	3.28E-06	7.71E-06
ZC518.3a	1241	1028	2025	2894	5.40E-06	7.94E-06	3.13E-06	7.29E-06
ZC518.3b	1740	1501	3184	3956	3.55E-05	2.85E-05	2.23E-05	4.71E-05
ZC518.4	2	0	5	0	7.49E-05	5.86E-05	7.96E-05	1.40E-04
ZC53.1	32	32	73	109	6.19E-05	5.04E-05	7.37E-05	1.13E-04
ZC53.2	2	3	10	3	2.80E-06	2.65E-06	1.82E-06	2.25E-06
ZC53.4	38	34	89	43	2.80E-06	2.65E-06	3.95E-06	7.29E-06
ZC53.6	5	3	5	13	2.80E-06	2.65E-06	1.82E-06	2.25E-06
ZC53.7	46	45	97	89	2.80E-06	2.65E-06	1.82E-06	2.25E-06
ZC581.1	46	124	42	81	2.80E-06	2.65E-06	1.82E-06	2.25E-06
ZC581.2	20	33	14	9	5.96E-06	5.50E-06	8.18E-06	9.27E-06
ZC581.3	132	191	105	139	4.79E-06	1.22E-05	2.84E-06	6.79E-06
ZC581.6	34	37	32	23	2.80E-06	3.70E-06	1.82E-06	2.25E-06
ZC581.7	17	46	4	7	6.36E-06	8.68E-06	3.30E-06	5.38E-06
ZC581.9	53	57	54	56	2.80E-06	2.65E-06	1.82E-06	2.25E-06
ZC64.2.1	264	348	493	206	2.80E-06	4.10E-06	1.82E-06	2.25E-06
ZC64.2.2	244	298	393	184	2.80E-06	2.65E-06	1.82E-06	2.25E-06
ZC64.3a	101	154	82	117	5.18E-05	6.45E-05	6.29E-05	3.25E-05
ZC64.3b	100	153	80	117	4.89E-05	5.64E-05	5.12E-05	2.96E-05
ZC64.3c	46	81	28	47	4.48E-06	6.45E-06	2.37E-06	4.16E-06
ZC64.4	6	2	4	3	4.45E-06	6.43E-06	2.31E-06	4.18E-06
ZC8.1	9	6	8	6	3.95E-06	6.56E-06	1.82E-06	3.24E-06
ZC8.3	130	209	87	121	2.80E-06	2.65E-06	1.82E-06	2.25E-06
ZC8.4a	1172	1365	1150	2612	2.80E-06	2.65E-06	1.82E-06	2.25E-06
ZC8.4b	933	1093	916	2158	8.06E-06	1.22E-05	3.52E-06	6.03E-06
ZC8.4d	1063	1237	1055	2471	1.74E-05	1.91E-05	1.11E-05	3.11E-05
ZC8.4e	153	156	143	243	1.64E-05	1.82E-05	1.05E-05	3.06E-05
ZC8.6	79	78	138	132	1.69E-05	1.86E-05	1.09E-05	3.15E-05
ZC84.1	51	57	28	20	1.24E-05	1.19E-05	7.53E-06	1.58E-05
ZC84.2	7	9	10	5	4.17E-06	3.89E-06	4.76E-06	5.62E-06
ZC84.3	57	121	95	208	2.80E-06	2.65E-06	1.82E-06	2.25E-06
ZC84.4	11	18	12	8	2.80E-06	2.65E-06	1.82E-06	2.25E-06
ZC84.5	1	2	1	0	2.80E-06	4.84E-06	2.62E-06	7.08E-06
ZC84.6	40	42	31	24	2.80E-06	2.65E-06	1.82E-06	2.25E-06
ZC84.7	1	4	1	2	2.80E-06	2.65E-06	1.82E-06	2.25E-06
ZC97.1b	198	294	200	175	2.80E-06	2.65E-06	1.82E-06	2.25E-06
ZK1005.1a	485	470	526	678	2.80E-06	2.65E-06	1.82E-06	2.25E-06
ZK1005.1b	493	486	531	686	2.61E-05	3.66E-05	1.71E-05	1.85E-05
ZK1010.1.1	10069	9724	23925	5578	7.92E-06	7.27E-06	5.59E-06	8.91E-06
ZK1010.1.2	7454	7354	17467	4611	8.06E-06	7.51E-06	5.65E-06	9.02E-06
ZK1010.2	207	266	411	437	2.28E-03	2.08E-03	3.53E-03	1.02E-03
ZK1010.4	4	6	1	2	1.88E-03	1.76E-03	2.87E-03	9.36E-04
ZK1010.5	169	233	94	79	1.79E-05	2.18E-05	2.32E-05	3.05E-05
ZK1010.6	11	9	7	4	2.80E-06	2.65E-06	1.82E-06	2.25E-06
ZK1010.8.1	9	35	26	34	8.01E-06	1.04E-05	2.90E-06	3.01E-06
ZK1010.8.2	7	21	11	25	2.80E-06	2.65E-06	1.82E-06	2.25E-06
ZK1010.9	17	19	11	6	2.80E-06	2.65E-06	1.82E-06	2.25E-06
ZK1010.t1	3	2	3	2	2.80E-06	2.65E-06	1.82E-06	2.25E-06
ZK1025.1	148	116	201	241	2.80E-06	2.65E-06	1.82E-06	2.25E-06

ZK1025.10	23	34	13	7	4.37E-06	2.75E-06	2.84E-06	2.34E-06
ZK1025.2	96	214	44	23	5.18E-06	3.84E-06	4.59E-06	6.79E-06
ZK1025.4a	9	32	7	9	2.80E-06	3.68E-06	1.82E-06	2.25E-06
ZK1025.4b	10	33	7	8	1.19E-05	2.50E-05	3.53E-06	2.29E-06
ZK1025.5	9	19	19	5	2.80E-06	2.65E-06	1.82E-06	2.25E-06
ZK1025.6	8	5	0	9	2.80E-06	2.65E-06	1.82E-06	2.25E-06
ZK1025.7	55	98	56	23	2.80E-06	4.92E-06	3.39E-06	2.25E-06
ZK1025.8	96	214	44	23	2.80E-06	2.65E-06	1.82E-06	2.25E-06
ZK1025.9	12	31	20	14	4.17E-06	7.04E-06	2.77E-06	2.25E-06
ZK1037.1	3	4	0	3	1.19E-05	2.50E-05	3.53E-06	2.29E-06
ZK1037.10	7	10	6	2	2.80E-06	3.20E-06	1.82E-06	2.25E-06
ZK1037.11	8	5	16	11	2.80E-06	2.65E-06	1.82E-06	2.25E-06
ZK1037.2	11	5	4	7	2.80E-06	2.65E-06	1.82E-06	2.25E-06
ZK1037.3	5	6	3	1	2.80E-06	2.65E-06	1.82E-06	2.25E-06
ZK1037.4	6	5	8	4	2.80E-06	2.65E-06	1.82E-06	2.25E-06
ZK1037.5	4	2	11	4	2.80E-06	2.65E-06	1.82E-06	2.25E-06
ZK1037.6	37	47	53	92	2.80E-06	2.65E-06	1.82E-06	2.25E-06
ZK1037.7	3	5	25	1	2.80E-06	2.65E-06	1.82E-06	2.25E-06
ZK1037.8	1	4	5	1	2.80E-06	3.17E-06	2.46E-06	5.29E-06
ZK1037.9	4	3	6	3	2.80E-06	2.65E-06	1.82E-06	2.25E-06
ZK105.1	347	557	220	401	2.80E-06	2.65E-06	1.82E-06	2.25E-06
ZK105.2	11	4	7	7	2.80E-06	2.65E-06	1.82E-06	2.25E-06
ZK105.3	20	40	14	7	6.93E-05	1.05E-04	2.86E-05	6.43E-05
ZK105.4	2	10	6	9	2.80E-06	2.65E-06	1.82E-06	2.25E-06
ZK105.5	9	14	7	7	2.80E-06	2.65E-06	1.82E-06	2.25E-06
ZK105.6	2	9	8	9	2.80E-06	2.65E-06	1.82E-06	2.25E-06
ZK105.7	2	4	4	3	2.80E-06	2.65E-06	1.82E-06	2.25E-06
ZK105.8	8	10	11	4	2.80E-06	2.65E-06	1.82E-06	2.25E-06
ZK1053.1	4	3	15	1	2.80E-06	2.65E-06	1.82E-06	2.25E-06
ZK1053.2	56	90	32	25	2.80E-06	2.65E-06	1.82E-06	2.25E-06
ZK1053.3	8	12	13	7	2.80E-06	2.65E-06	1.82E-06	2.25E-06
ZK1053.4	81	107	107	155	3.67E-06	5.58E-06	1.82E-06	2.25E-06
ZK1053.5	3	4	5	7	2.80E-06	2.65E-06	1.82E-06	2.25E-06
ZK1053.6	13	18	2	6	4.17E-06	5.21E-06	3.59E-06	6.41E-06
ZK1053.7	4	1	1	2	2.80E-06	2.65E-06	1.82E-06	2.25E-06
ZK1055.1	1968	1757	4165	4315	2.80E-06	2.65E-06	1.82E-06	2.25E-06
ZK1055.2	5	5	7	2	2.80E-06	2.65E-06	1.82E-06	2.25E-06
ZK1055.3	5	4	3	2	4.83E-05	4.07E-05	6.65E-05	8.50E-05
ZK1055.4	11	13	10	9	2.80E-06	2.65E-06	1.82E-06	2.25E-06
ZK1055.5	7	2	18	6	2.80E-06	2.65E-06	1.82E-06	2.25E-06
ZK1055.6a	31	69	24	54	2.80E-06	2.65E-06	1.82E-06	2.25E-06
ZK1055.6b	36	71	29	55	2.80E-06	2.65E-06	1.82E-06	2.25E-06
ZK1055.6c	10	19	12	18	2.80E-06	4.02E-06	1.82E-06	2.68E-06
ZK1055.7.1	137	258	131	190	2.80E-06	4.31E-06	1.82E-06	2.83E-06
ZK1055.7.2	128	244	121	181	2.80E-06	3.28E-06	1.82E-06	2.65E-06
ZK1058.1.1	933	1163	1381	1641	8.74E-06	1.56E-05	5.43E-06	9.74E-06
ZK1058.1.2	904	1139	1327	1603	8.37E-06	1.51E-05	5.14E-06	9.49E-06
ZK1058.3	61	59	62	74	4.10E-05	4.83E-05	3.95E-05	5.79E-05
ZK1058.4.1	1085	1272	914	1311	4.47E-05	5.32E-05	4.27E-05	6.37E-05
ZK1058.4.2	886	1023	727	1121	6.08E-06	5.55E-06	4.03E-06	5.94E-06
ZK1058.5	117	104	109	132	7.26E-05	8.04E-05	3.98E-05	7.04E-05
ZK1058.6	24	129	28	49	7.31E-05	7.97E-05	3.90E-05	7.43E-05
ZK1058.9	378	537	484	641	1.62E-05	1.36E-05	9.80E-06	1.47E-05
ZK1067.1	93	151	77	168	2.94E-06	1.49E-05	2.22E-06	4.81E-06
ZK1067.2	1210	762	1147	1494	3.77E-05	5.06E-05	3.14E-05	5.14E-05
ZK1067.3	297	354	462	500	2.80E-06	3.70E-06	1.82E-06	3.51E-06
ZK1067.4	18	23	12	13	2.04E-05	1.21E-05	1.26E-05	2.02E-05
ZK1067.5	11	7	12	5	2.64E-05	2.98E-05	2.67E-05	3.57E-05
ZK1067.6	163	207	128	161	2.80E-06	2.65E-06	1.82E-06	2.25E-06

ZK1067.7	491	576	98	159	2.80E-06	2.65E-06	1.82E-06	2.25E-06
ZK1067.8	41	35	38	76	7.70E-06	9.23E-06	3.94E-06	6.09E-06
ZK1067.t1	0	0	2	0	3.91E-05	4.34E-05	5.08E-06	1.02E-05
ZK1073.1.1	213	230	137	254	7.92E-06	6.40E-06	4.79E-06	1.18E-05
ZK1073.1.2	112	127	76	129	2.80E-06	2.65E-06	2.00E-06	2.25E-06
ZK1073.2	17	24	17	23	1.61E-05	1.64E-05	6.74E-06	1.54E-05
ZK1086.1a	107	149	81	224	1.28E-05	1.37E-05	5.65E-06	1.19E-05
ZK1086.1b	86	123	70	197	3.00E-06	3.97E-06	1.95E-06	3.24E-06
ZK1086.2	7	6	6	4	4.42E-06	5.82E-06	2.17E-06	7.42E-06
ZK1086.3	5	5	3	3	4.17E-06	5.63E-06	2.20E-06	7.67E-06
ZK1098.1	657	742	424	816	2.80E-06	2.65E-06	1.82E-06	2.25E-06
ZK1098.10a	589	673	878	1113	2.80E-06	2.65E-06	1.82E-06	2.25E-06
ZK1098.11	61	63	145	65	3.31E-05	3.53E-05	1.39E-05	3.30E-05
ZK1098.2	278	361	435	662	1.85E-05	2.00E-05	1.79E-05	2.81E-05
ZK1098.3	46	62	78	74	2.25E-05	2.20E-05	3.49E-05	1.93E-05
ZK1098.4	278	290	304	369	1.65E-05	2.03E-05	1.69E-05	3.17E-05
ZK1098.5	214	201	312	195	2.80E-06	2.78E-06	2.41E-06	2.83E-06
ZK1098.6	22	43	22	9	2.90E-05	2.86E-05	2.06E-05	3.09E-05
ZK1098.8	498	457	734	1037	3.96E-05	3.51E-05	3.75E-05	2.89E-05
ZK1098.9	16	24	11	7	2.94E-06	5.42E-06	1.91E-06	2.25E-06
ZK112.1	324	509	302	530	1.88E-05	1.63E-05	1.81E-05	3.15E-05
ZK112.2	1037	1030	753	1515	2.80E-06	3.84E-06	1.82E-06	2.25E-06
ZK112.3	8	10	4	10	1.86E-05	2.76E-05	1.13E-05	2.44E-05
ZK112.4	2	1	0	2	2.63E-05	2.47E-05	1.24E-05	3.09E-05
ZK112.5	5	4	6	2	2.80E-06	2.65E-06	1.82E-06	2.25E-06
ZK112.6	4	8	3	6	2.80E-06	2.65E-06	1.82E-06	2.25E-06
ZK112.7	129	189	207	289	2.80E-06	2.65E-06	1.82E-06	2.25E-06
ZK1127.1	681	567	1417	904	2.80E-06	2.65E-06	1.82E-06	2.25E-06
ZK1127.10.1	1904	2197	2653	2797	2.80E-06	2.65E-06	1.82E-06	2.56E-06
ZK1127.10.2	1850	2118	2509	2737	7.36E-05	5.79E-05	9.97E-05	7.85E-05
ZK1127.11	242	266	347	468	1.56E-04	1.70E-04	1.41E-04	1.84E-04
ZK1127.12	218	246	371	215	1.54E-04	1.66E-04	1.36E-04	1.83E-04
ZK1127.2	56	98	33	28	1.04E-05	1.08E-05	9.75E-06	1.62E-05
ZK1127.3	216	259	301	304	1.77E-05	1.89E-05	1.96E-05	1.41E-05
ZK1127.4	291	336	224	268	3.42E-06	5.66E-06	1.82E-06	2.25E-06
ZK1127.5	158	212	164	191	3.54E-05	4.01E-05	3.21E-05	4.00E-05
ZK1127.6.1	537	525	413	791	3.01E-05	3.28E-05	1.51E-05	2.23E-05
ZK1127.6.2	463	432	371	715	1.44E-05	1.83E-05	9.77E-06	1.40E-05
ZK1127.7	595	631	864	1133	3.91E-05	3.61E-05	1.96E-05	4.62E-05
ZK1127.9a	933	974	788	1472	3.72E-05	3.28E-05	1.94E-05	4.62E-05
ZK1127.9b	908	953	769	1455	2.57E-05	2.57E-05	2.43E-05	3.93E-05
ZK1127.9c	762	789	649	1268	3.81E-05	3.75E-05	2.09E-05	4.82E-05
ZK1127.9d	244	285	207	419	3.74E-05	3.71E-05	2.06E-05	4.82E-05
ZK1127.9e.1	653	685	575	1135	4.10E-05	4.01E-05	2.27E-05	5.48E-05
ZK1127.9e.2	779	832	684	1302	1.88E-05	2.08E-05	1.04E-05	2.59E-05
ZK1128.1	167	178	178	213	3.67E-05	3.64E-05	2.10E-05	5.12E-05
ZK1128.2a	110	151	183	206	3.56E-05	3.59E-05	2.04E-05	4.78E-05
ZK1128.2b	107	146	168	200	1.60E-05	1.61E-05	1.11E-05	1.64E-05
ZK1128.3	22	42	40	27	8.54E-06	1.11E-05	9.26E-06	1.29E-05
ZK1128.4	265	231	301	367	8.34E-06	1.08E-05	8.55E-06	1.25E-05
ZK1128.5	244	310	389	432	2.80E-06	4.10E-06	2.70E-06	2.25E-06
ZK1128.6a	450	477	678	804	2.83E-05	2.33E-05	2.09E-05	3.15E-05
ZK1128.6b.1	412	452	611	749	1.77E-05	2.12E-05	1.83E-05	2.51E-05
ZK1128.6b.2	371	427	572	721	2.18E-05	2.19E-05	2.14E-05	3.14E-05
ZK1128.7	51	70	27	37	2.56E-05	2.65E-05	2.47E-05	3.73E-05
ZK1128.8a.1	217	249	305	317	2.44E-05	2.65E-05	2.45E-05	3.81E-05
ZK1128.8a.2	173	207	233	267	9.24E-06	1.20E-05	3.19E-06	5.38E-06
ZK1128.8b	178	219	240	291	3.52E-05	3.81E-05	3.22E-05	4.13E-05
ZK1151.1a	2511	3388	1658	4145	3.15E-05	3.56E-05	2.76E-05	3.91E-05

ZK1151.1b	1553	1816	784	2361	2.84E-05	3.31E-05	2.49E-05	3.73E-05
ZK1151.1c	1545	1801	780	2354	2.58E-05	3.29E-05	1.11E-05	3.42E-05
ZK1151.1d	2298	3138	1534	3790	1.14E-05	1.26E-05	3.75E-06	1.39E-05
ZK1151.1e	2495	3353	1640	4124	1.14E-05	1.25E-05	3.74E-06	1.39E-05
ZK1151.1f	2266	3093	1514	3721	2.52E-05	3.25E-05	1.10E-05	3.34E-05
ZK1151.1g	1425	1656	721	2164	2.66E-05	3.38E-05	1.14E-05	3.54E-05
ZK1151.1h	2306	3148	1541	3805	2.55E-05	3.28E-05	1.11E-05	3.36E-05
ZK1193.1	4190	5922	5835	7112	1.21E-05	1.33E-05	3.97E-06	1.47E-05
ZK1193.2	160	213	254	256	2.51E-05	3.24E-05	1.09E-05	3.33E-05
ZK1193.3	48	33	109	94	4.63E-04	6.18E-04	4.19E-04	6.31E-04
ZK1193.4	16	11	19	23	4.79E-06	6.00E-06	4.94E-06	6.14E-06
ZK1193.5a	98	130	66	113	3.75E-06	2.65E-06	5.56E-06	5.91E-06
ZK1193.5b	31	41	15	34	3.16E-06	2.65E-06	2.44E-06	3.64E-06
ZK1193.5c	102	151	72	132	7.76E-06	9.73E-06	3.41E-06	7.20E-06
ZK121.1a	142	153	356	141	3.14E-06	3.91E-06	1.82E-06	2.74E-06
ZK121.1b	142	153	356	141	7.50E-06	1.05E-05	3.44E-06	7.80E-06
ZK121.2	140	178	75	109	3.20E-05	3.26E-05	5.22E-05	2.55E-05
ZK1225.1	6	3	3	4	3.20E-05	3.26E-05	5.22E-05	2.55E-05
ZK1225.10	2	1	0	2	9.35E-06	1.12E-05	3.26E-06	5.85E-06
ZK1225.11	1	1	1	0	2.80E-06	2.65E-06	1.82E-06	2.25E-06
ZK1225.13	0	1	6	3	2.80E-06	2.65E-06	1.82E-06	2.25E-06
ZK1225.2	4	5	4	1	2.80E-06	2.65E-06	1.82E-06	2.25E-06
ZK1225.4	36	64	13	17	2.80E-06	2.65E-06	1.82E-06	2.25E-06
ZK1225.5	11	26	15	11	2.80E-06	2.65E-06	1.82E-06	2.25E-06
ZK1225.6	45	50	76	17	2.80E-06	4.34E-06	1.82E-06	2.25E-06
ZK1225.7	54	82	48	51	2.80E-06	2.65E-06	1.82E-06	2.25E-06
ZK1225.8	12	12	13	16	1.40E-05	1.47E-05	1.54E-05	4.25E-06
ZK1225.9	2	6	2	3	7.17E-06	1.03E-05	4.14E-06	5.44E-06
ZK1236.1	170	182	190	261	2.80E-06	2.65E-06	1.82E-06	2.25E-06
ZK1236.2	182	279	151	219	2.80E-06	2.65E-06	1.82E-06	2.25E-06
ZK1236.3a	258	288	215	399	9.69E-06	9.79E-06	7.05E-06	1.19E-05
ZK1236.3b	225	257	182	363	1.65E-05	2.39E-05	8.91E-06	1.60E-05
ZK1236.5	177	165	264	225	8.96E-06	9.44E-06	4.85E-06	1.11E-05
ZK1236.6	346	315	376	361	1.04E-05	1.12E-05	5.45E-06	1.34E-05
ZK1236.7.1	262	396	241	380	3.84E-05	3.38E-05	3.73E-05	3.92E-05
ZK1236.7.3	227	345	209	329	4.04E-05	3.47E-05	2.86E-05	3.38E-05
ZK1236.9	7	11	12	6	2.37E-05	3.39E-05	1.42E-05	2.76E-05
ZK1240.1	11	5	8	4	2.60E-05	3.73E-05	1.56E-05	3.03E-05
ZK1240.2	10	37	121	39	2.80E-06	2.65E-06	1.82E-06	2.25E-06
ZK1240.3	40	76	36	47	2.80E-06	2.65E-06	1.82E-06	2.25E-06
ZK1240.4	4	5	10	1	2.80E-06	3.73E-06	8.40E-06	3.33E-06
ZK1240.5	8	9	12	9	3.72E-06	6.67E-06	2.17E-06	3.51E-06
ZK1240.6	0	2	2	1	2.80E-06	2.65E-06	1.82E-06	2.25E-06
ZK1240.8	4	6	9	2	2.80E-06	2.65E-06	1.82E-06	2.25E-06
ZK1240.9	3	11	5	8	2.80E-06	2.65E-06	1.82E-06	2.25E-06
ZK1248.1	21	14	10	13	2.80E-06	2.65E-06	1.82E-06	2.25E-06
ZK1248.10	898	800	1396	1855	2.80E-06	2.65E-06	1.82E-06	2.25E-06
ZK1248.11.1	158	207	239	203	2.80E-06	2.65E-06	1.82E-06	2.25E-06
ZK1248.13	278	335	343	510	2.95E-05	2.48E-05	2.99E-05	4.90E-05
ZK1248.14	375	551	524	688	2.44E-05	3.03E-05	2.41E-05	2.52E-05
ZK1248.15	358	347	355	522	1.65E-05	1.88E-05	1.33E-05	2.44E-05
ZK1248.17	157	159	81	28	1.66E-05	2.30E-05	1.51E-05	2.45E-05
ZK1248.18	1	6	11	23	2.67E-05	2.44E-05	1.72E-05	3.13E-05
ZK1248.19	37	50	62	28	6.11E-05	5.84E-05	2.05E-05	8.75E-06
ZK1248.2	63	36	74	68	2.80E-06	5.77E-06	7.29E-06	1.88E-05
ZK1248.20	3	15	6	2	7.98E-06	1.02E-05	8.71E-06	4.86E-06
ZK1248.3a	637	603	706	1043	6.61E-06	3.57E-06	5.05E-06	5.71E-06
ZK1248.3b	549	525	598	886	2.80E-06	4.05E-06	1.82E-06	2.25E-06
ZK1248.4	633	653	1081	214	2.68E-05	2.39E-05	1.93E-05	3.52E-05

ZK1248.5	42	102	40	10	2.81E-05	2.54E-05	1.99E-05	3.64E-05
ZK1248.6	993	1094	1659	326	3.03E-04	2.95E-04	3.37E-04	8.23E-05
ZK1248.7	99	87	119	123	4.68E-06	1.07E-05	2.90E-06	2.25E-06
ZK1248.8	2	1	1	1	2.65E-04	2.76E-04	2.89E-04	7.00E-05
ZK1248.9	12	17	8	5	1.02E-05	8.44E-06	7.94E-06	1.01E-05
ZK1251.1	43	66	39	17	2.80E-06	2.65E-06	1.82E-06	2.25E-06
ZK1251.11	8	3	0	0	2.80E-06	2.65E-06	1.82E-06	2.25E-06
ZK1251.2	8	20	32	7	1.10E-05	1.59E-05	6.49E-06	3.49E-06
ZK1251.3	10	17	5	2	2.86E-06	2.65E-06	1.82E-06	2.25E-06
ZK1251.5	16	31	4	4	2.83E-06	6.67E-06	7.34E-06	2.25E-06
ZK1251.6	1749	2113	3406	609	2.80E-06	2.65E-06	1.82E-06	2.25E-06
ZK1251.7	4	1	5	6	2.80E-06	4.52E-06	1.82E-06	2.25E-06
ZK1251.8	21	26	22	13	4.36E-04	4.98E-04	5.53E-04	1.22E-04
ZK1251.9	920	905	1454	1948	2.80E-06	2.65E-06	1.82E-06	2.25E-06
ZK1290.1	5	2	1	0	2.80E-06	2.65E-06	1.82E-06	2.25E-06
ZK1290.10	18	5	10	11	1.92E-05	1.78E-05	1.97E-05	3.26E-05
ZK1290.11	2	2	3	0	2.80E-06	2.65E-06	1.82E-06	2.25E-06
ZK1290.12	200	374	83	76	2.80E-06	2.65E-06	1.82E-06	2.25E-06
ZK1290.13	14	25	16	19	2.80E-06	2.65E-06	1.82E-06	2.25E-06
ZK1290.14	21	74	31	27	1.41E-05	2.50E-05	3.83E-06	4.32E-06
ZK1290.15	1	4	12	3	2.80E-06	2.72E-06	1.82E-06	2.25E-06
ZK1290.2a	15	19	16	25	7.06E-06	2.35E-05	6.78E-06	7.29E-06
ZK1290.2b.1	7	6	13	12	2.80E-06	2.65E-06	3.32E-06	2.25E-06
ZK1290.2b.2	5	5	8	12	2.80E-06	2.65E-06	1.82E-06	2.25E-06
ZK1290.3a	755	1060	440	231	2.80E-06	2.65E-06	1.82E-06	2.25E-06
ZK1290.3b	501	718	285	148	2.80E-06	2.65E-06	1.82E-06	2.25E-06
ZK1290.4a	378	391	495	791	7.74E-05	1.03E-04	2.93E-05	1.90E-05
ZK1290.4b.1	393	406	518	804	6.77E-05	9.16E-05	2.51E-05	1.61E-05
ZK1290.4b.2	378	392	488	786	1.72E-05	1.68E-05	1.46E-05	2.89E-05
ZK1290.4c	460	485	615	938	1.72E-05	1.68E-05	1.48E-05	2.83E-05
ZK1290.5	55	175	49	53	1.73E-05	1.69E-05	1.45E-05	2.89E-05
ZK1290.6	22	60	10	9	1.70E-05	1.70E-05	1.48E-05	2.79E-05
ZK1290.7	7	11	6	2	6.38E-06	1.92E-05	3.70E-06	4.93E-06
ZK1290.8	331	329	151	107	2.80E-06	4.10E-06	1.82E-06	2.25E-06
ZK1290.9	5	16	3	3	2.80E-06	2.65E-06	1.82E-06	2.25E-06
ZK1307.1a	330	470	252	213	3.56E-05	3.34E-05	1.06E-05	9.24E-06
ZK1307.1b.1	309	442	239	203	2.80E-06	2.65E-06	1.82E-06	2.25E-06
ZK1307.1b.2	172	247	134	115	3.81E-05	5.13E-05	1.89E-05	1.97E-05
ZK1307.2	20	26	18	4	3.58E-05	4.84E-05	1.80E-05	1.89E-05
ZK1307.3	33	43	22	11	3.76E-05	5.11E-05	1.91E-05	2.02E-05
ZK1307.4	16	30	11	12	5.82E-06	7.17E-06	3.43E-06	2.25E-06
ZK1307.5	231	280	262	285	1.06E-05	1.31E-05	4.61E-06	2.83E-06
ZK1307.6	507	468	629	958	5.15E-06	9.13E-06	2.30E-06	3.10E-06
ZK1307.7	6	6	8	4	2.05E-05	2.35E-05	1.51E-05	2.03E-05
ZK1307.8.1	904	1092	688	976	2.64E-05	2.30E-05	2.13E-05	4.01E-05
ZK1307.8.2	816	999	608	857	2.80E-06	2.65E-06	1.82E-06	2.25E-06
ZK1307.9	247	254	381	381	5.75E-05	6.57E-05	2.85E-05	4.99E-05
ZK131.1	7	19	11	1	5.99E-05	6.93E-05	2.90E-05	5.05E-05
ZK131.10	17	23	44	15	2.48E-05	2.41E-05	2.49E-05	3.07E-05
ZK131.11a	64	80	34	70	2.80E-06	6.45E-06	2.57E-06	2.25E-06
ZK131.11b	128	159	113	174	4.96E-06	6.32E-06	8.33E-06	3.51E-06
ZK131.2	21	18	15	11	6.52E-06	7.70E-06	2.26E-06	5.74E-06
ZK131.3	21	18	15	11	7.53E-06	8.86E-06	4.34E-06	8.23E-06
ZK131.4	7	19	11	1	5.71E-06	4.63E-06	2.66E-06	2.41E-06
ZK131.5	53	58	132	22	5.71E-06	4.63E-06	2.66E-06	2.41E-06
ZK131.6	17	23	47	17	2.80E-06	6.45E-06	2.57E-06	2.25E-06
ZK131.7	21	18	15	11	1.61E-05	1.66E-05	2.61E-05	5.35E-06
ZK131.8	7	19	11	1	4.96E-06	6.35E-06	8.93E-06	3.98E-06
ZK131.9	35	39	62	17	5.71E-06	4.63E-06	2.66E-06	2.41E-06

ZK1320.1	122	113	99	57	2.80E-06	6.45E-06	2.57E-06	2.25E-06
ZK1320.10.1	57	81	43	48	1.06E-05	1.12E-05	1.22E-05	4.14E-06
ZK1320.10.2	46	63	40	45	2.01E-05	1.76E-05	1.06E-05	7.53E-06
ZK1320.11	41	41	37	35	9.94E-06	1.33E-05	4.87E-06	6.72E-06
ZK1320.12a	286	341	368	468	8.29E-06	1.07E-05	4.70E-06	6.52E-06
ZK1320.12b	292	347	374	468	7.22E-06	6.82E-06	4.25E-06	4.95E-06
ZK1320.13	41	25	36	15	2.00E-05	2.25E-05	1.67E-05	2.63E-05
ZK1320.2.1	482	646	681	445	2.06E-05	2.31E-05	1.71E-05	2.65E-05
ZK1320.2.2	390	522	538	413	2.22E-05	1.28E-05	1.27E-05	6.52E-06
ZK1320.3.1	638	926	1450	751	7.57E-05	9.59E-05	6.96E-05	5.61E-05
ZK1320.3.2	506	724	1195	709	6.92E-05	8.75E-05	6.21E-05	5.89E-05
ZK1320.4	17	22	23	24	1.05E-04	1.44E-04	1.55E-04	9.94E-05
ZK1320.5	5	8	7	3	8.52E-05	1.15E-04	1.31E-04	9.59E-05
ZK1320.6	58	111	51	69	2.80E-06	2.65E-06	1.82E-06	2.25E-06
ZK1320.7	81	87	58	106	2.80E-06	2.65E-06	1.82E-06	2.25E-06
ZK1320.9.1	505	619	672	970	3.81E-06	6.90E-06	2.19E-06	3.64E-06
ZK1320.9.2	451	558	600	912	8.12E-06	8.25E-06	3.79E-06	8.55E-06
ZK1321.1	1	5	10	0	3.60E-05	4.17E-05	3.12E-05	5.55E-05
ZK1321.2a	18	48	22	31	3.56E-05	4.16E-05	3.08E-05	5.77E-05
ZK1321.2b	19	49	23	32	2.80E-06	2.65E-06	1.82E-06	2.25E-06
ZK1321.2c	27	55	30	35	2.80E-06	3.47E-06	1.82E-06	2.25E-06
ZK1321.2d.1	20	49	22	27	2.80E-06	3.25E-06	1.82E-06	2.25E-06
ZK1321.2d.2	19	49	22	27	2.80E-06	3.04E-06	1.82E-06	2.25E-06
ZK1321.2e	20	50	21	29	2.80E-06	3.09E-06	1.82E-06	2.25E-06
ZK1321.2f.1	19	49	22	30	2.80E-06	3.17E-06	1.82E-06	2.25E-06
ZK1321.2f.2	18	49	22	29	2.80E-06	3.33E-06	1.82E-06	2.25E-06
ZK1321.2f.3	18	48	22	31	2.80E-06	3.31E-06	1.82E-06	2.25E-06
ZK1321.2f.4	19	49	23	32	2.80E-06	3.25E-06	1.82E-06	2.25E-06
ZK1321.3.1	317	413	288	286	2.80E-06	3.47E-06	1.82E-06	2.25E-06
ZK1321.3.2	269	357	236	261	2.80E-06	3.25E-06	1.82E-06	2.25E-06
ZK1321.4	340	463	358	375	3.56E-05	4.39E-05	2.11E-05	2.58E-05
ZK154.1	77	163	34	30	3.55E-05	4.45E-05	2.03E-05	2.77E-05
ZK154.3	224	281	323	361	2.59E-05	3.33E-05	1.77E-05	2.29E-05
ZK154.4	62	97	51	60	1.32E-05	2.64E-05	3.79E-06	4.14E-06
ZK154.5	139	178	211	283	1.62E-05	1.91E-05	1.51E-05	2.09E-05
ZK154.6a	47	73	40	53	8.26E-06	1.22E-05	4.41E-06	6.41E-06
ZK154.6b	12	15	17	9	4.45E-06	5.37E-06	4.39E-06	7.26E-06
ZK154.7	81	140	103	136	6.61E-06	9.71E-06	3.66E-06	6.00E-06
ZK177.1	137	136	173	198	2.97E-06	3.49E-06	2.73E-06	2.25E-06
ZK177.10	7	5	6	5	3.95E-06	6.43E-06	3.26E-06	5.31E-06
ZK177.11	2	1	4	2	1.11E-05	1.04E-05	9.11E-06	1.29E-05
ZK177.2	40	67	33	76	2.80E-06	2.65E-06	1.82E-06	2.25E-06
ZK177.3	10	8	1	3	2.80E-06	2.65E-06	1.82E-06	2.25E-06
ZK177.4.1	384	425	550	671	5.04E-06	7.96E-06	2.70E-06	7.67E-06
ZK177.4.2	370	407	531	654	2.80E-06	2.65E-06	1.82E-06	2.25E-06
ZK177.5.1	192	242	220	347	2.07E-05	2.17E-05	1.93E-05	2.91E-05
ZK177.5.2	176	230	195	320	2.07E-05	2.15E-05	1.93E-05	2.94E-05
ZK177.6	906	891	1400	1530	1.22E-05	1.45E-05	9.06E-06	1.77E-05
ZK177.8a	462	502	651	893	1.34E-05	1.65E-05	9.66E-06	1.95E-05
ZK177.8b.1	429	473	636	839	5.58E-05	5.19E-05	5.62E-05	7.57E-05
ZK177.8b.2	417	462	600	828	2.61E-05	2.68E-05	2.39E-05	4.05E-05
ZK177.9	2	2	3	0	2.88E-05	2.99E-05	2.77E-05	4.52E-05
ZK180.1	20	30	12	17	2.80E-05	2.93E-05	2.62E-05	4.46E-05
ZK180.2	13	25	1	15	2.80E-06	2.65E-06	1.82E-06	2.25E-06
ZK180.3a	178	176	231	296	2.80E-06	2.75E-06	1.82E-06	2.25E-06
ZK180.3b.1	131	137	186	257	2.80E-06	3.78E-06	1.82E-06	2.25E-06
ZK180.3b.2	131	137	187	257	1.19E-05	1.11E-05	1.00E-05	1.59E-05
ZK180.3b.3	116	130	164	237	1.11E-05	1.10E-05	1.03E-05	1.75E-05
ZK180.4	1191	1387	1271	1614	1.11E-05	1.10E-05	1.03E-05	1.75E-05

ZK180.5a	879	902	301	432	1.11E-05	1.17E-05	1.02E-05	1.81E-05
ZK180.5c	636	627	238	306	1.24E-04	1.37E-04	8.63E-05	1.35E-04
ZK180.6	836	1046	222	318	5.26E-05	5.10E-05	1.17E-05	2.08E-05
ZK185.1	29	33	48	80	4.78E-05	4.45E-05	1.16E-05	1.85E-05
ZK185.2	17	21	14	24	3.98E-05	4.71E-05	6.89E-06	1.22E-05
ZK185.3	44	80	53	63	3.78E-06	4.05E-06	4.06E-06	8.37E-06
ZK185.4	1	3	4	1	2.80E-06	2.65E-06	1.82E-06	2.25E-06
ZK185.5	38	68	76	61	5.60E-06	9.60E-06	4.37E-06	6.43E-06
ZK20.1	27	34	20	28	2.80E-06	2.65E-06	1.82E-06	2.25E-06
ZK20.2	5	3	2	0	4.03E-06	6.80E-06	5.23E-06	5.17E-06
ZK20.3	1464	1622	2236	2601	2.80E-06	2.65E-06	1.82E-06	2.25E-06
ZK20.6.1	216	365	198	226	2.80E-06	2.65E-06	1.82E-06	2.25E-06
ZK20.6.2	203	330	183	210	1.12E-04	1.18E-04	1.12E-04	1.60E-04
ZK218.1	2	0	0	3	9.60E-06	1.53E-05	5.72E-06	8.07E-06
ZK218.10	1	1	0	2	1.00E-05	1.54E-05	5.87E-06	8.32E-06
ZK218.11	1	5	2	3	2.80E-06	2.65E-06	1.82E-06	2.25E-06
ZK218.12	1	5	2	3	2.80E-06	2.65E-06	1.82E-06	2.25E-06
ZK218.13	19	86	430	552	2.80E-06	2.65E-06	1.82E-06	2.25E-06
ZK218.3	2	4	7	7	2.80E-06	4.44E-06	1.82E-06	2.27E-06
ZK218.4	1	1	2	3	2.17E-05	9.28E-05	3.20E-04	5.07E-04
ZK218.5	0	11	5	1	2.80E-06	2.65E-06	1.82E-06	2.25E-06
ZK218.6	3	5	4	3	2.80E-06	2.65E-06	1.82E-06	2.25E-06
ZK218.7	1	2	0	0	2.80E-06	2.65E-06	1.82E-06	2.25E-06
ZK218.8	3	1	1	2	2.80E-06	2.65E-06	1.82E-06	2.25E-06
ZK218.9	0	3	3	0	2.80E-06	2.65E-06	1.82E-06	2.25E-06
ZK228.1	3	5	1	1	2.80E-06	2.65E-06	1.82E-06	2.25E-06
ZK228.10	3	2	0	2	2.80E-06	2.65E-06	1.82E-06	2.25E-06
ZK228.3	36	43	55	35	2.80E-06	2.65E-06	1.82E-06	2.25E-06
ZK228.5	4	1	4	2	2.80E-06	2.65E-06	1.82E-06	2.25E-06
ZK228.6	4	1	4	1	4.28E-06	4.84E-06	4.26E-06	3.35E-06
ZK228.7	4	2	3	1	2.80E-06	2.65E-06	1.82E-06	2.25E-06
ZK228.8	2	0	4	2	2.80E-06	2.65E-06	1.82E-06	2.25E-06
ZK250.10	6	17	19	9	2.80E-06	2.65E-06	1.82E-06	2.25E-06
ZK250.2	4	3	3	3	2.80E-06	2.65E-06	1.82E-06	2.25E-06
ZK250.5a.1	29	46	40	29	2.80E-06	2.65E-06	1.82E-06	2.25E-06
ZK250.5a.2	22	33	26	24	2.80E-06	2.65E-06	1.82E-06	2.25E-06
ZK250.5b	30	39	29	28	2.80E-06	2.65E-06	1.82E-06	2.25E-06
ZK250.6	14	18	17	14	2.80E-06	2.65E-06	1.82E-06	2.25E-06
ZK250.7	10	16	24	3	2.80E-06	2.65E-06	1.82E-06	2.25E-06
ZK250.8	25	21	21	17	2.80E-06	2.65E-06	1.82E-06	2.25E-06
ZK250.9	15	31	24	14	2.80E-06	2.65E-06	1.82E-06	2.25E-06
ZK256.1b	292	269	459	548	2.80E-06	2.65E-06	1.82E-06	2.25E-06
ZK256.1c	462	457	673	833	2.80E-06	2.65E-06	1.82E-06	2.25E-06
ZK262.1	2	8	26	8	1.49E-05	1.30E-05	1.53E-05	2.25E-05
ZK262.10	4	5	15	4	1.57E-05	1.47E-05	1.49E-05	2.27E-05
ZK262.11	2	5	3	1	2.80E-06	2.65E-06	1.86E-06	2.25E-06
ZK262.12	11	4	17	11	2.80E-06	2.65E-06	1.82E-06	2.25E-06
ZK262.13	52	45	98	26	2.80E-06	2.65E-06	1.82E-06	2.25E-06
ZK262.2	7	12	9	9	2.80E-06	2.65E-06	1.82E-06	2.25E-06
ZK262.3	6	12	11	7	2.80E-06	2.65E-06	3.33E-06	2.25E-06
ZK262.4	9	9	6	1	2.80E-06	2.65E-06	1.82E-06	2.25E-06
ZK262.6	2	2	2	1	2.80E-06	2.65E-06	1.82E-06	2.25E-06
ZK262.7	3	3	2	2	4.37E-06	4.13E-06	1.90E-06	2.25E-06
ZK262.8	6	2	2	1	2.80E-06	2.65E-06	1.82E-06	2.25E-06
ZK262.9	9	5	6	2	2.80E-06	2.65E-06	1.82E-06	2.25E-06
ZK262.t1	0	0	1	0	2.80E-06	2.65E-06	1.82E-06	2.25E-06
ZK265.1a	22	40	16	30	2.80E-06	2.65E-06	1.82E-06	2.25E-06
ZK265.1b	22	36	17	31	2.80E-06	2.65E-06	1.82E-06	2.25E-06
ZK265.2	539	873	227	230	2.80E-06	2.65E-06	1.82E-06	2.25E-06

ZK265.3	70	138	24	33	2.80E-06	2.65E-06	1.82E-06	2.25E-06
ZK265.4	3	3	2	8	5.03E-05	7.70E-05	1.38E-05	1.72E-05
ZK265.5	7	10	6	5	7.67E-06	1.43E-05	1.82E-06	2.90E-06
ZK265.6	259	286	297	261	2.80E-06	2.65E-06	1.82E-06	2.25E-06
ZK265.8	22	32	19	9	2.80E-06	2.65E-06	1.82E-06	2.25E-06
ZK265.9	357	376	465	537	4.76E-05	4.97E-05	3.55E-05	3.85E-05
ZK270.1	296	412	197	378	2.80E-06	2.65E-06	1.82E-06	2.25E-06
ZK270.2a.1	8066	8643	3444	8937	4.18E-05	4.16E-05	3.55E-05	5.05E-05
ZK270.2a.2	8074	8651	3446	8939	9.91E-06	1.30E-05	4.30E-06	1.02E-05
ZK270.2a.3	7875	8392	3273	8607	6.35E-05	6.42E-05	1.76E-05	5.65E-05
ZK270.2b	609	580	539	974	6.36E-05	6.44E-05	1.77E-05	5.66E-05
ZK270.2d	7898	8425	3289	8652	6.49E-05	6.53E-05	1.75E-05	5.69E-05
ZK270.2e	7876	8394	3275	8609	3.57E-05	3.21E-05	2.06E-05	4.59E-05
ZK285.1	4	5	5	3	6.34E-05	6.39E-05	1.72E-05	5.58E-05
ZK285.2	14	11	7	5	6.47E-05	6.52E-05	1.75E-05	5.68E-05
ZK287.2.1	72	75	82	67	2.80E-06	2.65E-06	1.82E-06	2.25E-06
ZK287.2.2	72	75	83	68	3.28E-06	2.65E-06	1.82E-06	2.25E-06
ZK287.3	39	60	93	39	3.70E-06	3.62E-06	2.73E-06	2.77E-06
ZK287.4	69	61	36	57	3.61E-06	3.54E-06	2.70E-06	2.72E-06
ZK287.5.1	380	443	680	469	1.41E-05	2.06E-05	2.19E-05	1.14E-05
ZK287.5.2	231	254	374	248	2.80E-06	2.65E-06	1.82E-06	2.25E-06
ZK287.6	65	77	106	104	6.41E-05	7.06E-05	7.46E-05	6.35E-05
ZK287.7	139	168	137	193	7.31E-05	7.59E-05	7.70E-05	6.30E-05
ZK287.8a	3	4	3	10	8.74E-06	9.76E-06	9.26E-06	1.12E-05
ZK287.8b	2	2	3	4	1.18E-05	1.34E-05	7.54E-06	1.31E-05
ZK287.9	72	67	78	38	2.80E-06	2.65E-06	1.82E-06	2.25E-06
ZK328.1a	646	696	808	998	2.80E-06	2.65E-06	1.82E-06	2.25E-06
ZK328.1b	606	653	761	953	3.24E-05	2.85E-05	2.28E-05	1.37E-05
ZK328.2	1503	1547	2210	2588	1.91E-05	1.94E-05	1.55E-05	2.37E-05
ZK328.4	353	386	469	617	1.89E-05	1.93E-05	1.55E-05	2.39E-05
ZK328.5a	840	822	938	1634	5.53E-05	5.37E-05	5.29E-05	7.64E-05
ZK328.5b	1558	1406	1497	2626	2.15E-05	2.22E-05	1.86E-05	3.02E-05
ZK328.6	25	42	26	22	4.20E-05	3.88E-05	3.05E-05	6.56E-05
ZK328.7a	18	29	24	12	4.58E-05	3.91E-05	2.87E-05	6.21E-05
ZK328.7b	10	26	22	12	2.80E-06	3.31E-06	1.82E-06	2.25E-06
ZK328.8	9	7	7	6	2.80E-06	2.65E-06	1.82E-06	2.25E-06
ZK337.1a.1	478	770	355	753	2.80E-06	2.65E-06	1.82E-06	2.25E-06
ZK337.1a.2	465	753	345	752	2.80E-06	2.65E-06	1.82E-06	2.25E-06
ZK337.1b.1	475	766	358	759	1.18E-05	1.80E-05	5.72E-06	1.50E-05
ZK337.1b.2	459	746	343	753	1.13E-05	1.73E-05	5.45E-06	1.47E-05
ZK337.1c.1	490	787	362	764	1.16E-05	1.76E-05	5.69E-06	1.49E-05
ZK337.1c.2	463	756	346	753	1.12E-05	1.72E-05	5.45E-06	1.47E-05
ZK337.2	21	46	23	33	1.15E-05	1.75E-05	5.54E-06	1.44E-05
ZK337.4	8	6	5	2	1.12E-05	1.72E-05	5.43E-06	1.46E-05
ZK337.5	7	39	15	14	2.80E-06	2.75E-06	1.82E-06	2.25E-06
ZK353.10	0	2	3	0	2.80E-06	2.65E-06	1.82E-06	2.25E-06
ZK353.1a	499	522	785	994	2.80E-06	4.76E-06	1.82E-06	2.25E-06
ZK353.1b	285	299	477	654	2.80E-06	2.65E-06	1.82E-06	2.25E-06
ZK353.2	6	8	4	2	3.28E-05	3.25E-05	3.36E-05	5.25E-05
ZK353.3	2	7	9	3	2.86E-05	2.84E-05	3.12E-05	5.28E-05
ZK353.4	10	18	10	8	2.80E-06	2.83E-06	1.82E-06	2.25E-06
ZK353.5	6	4	3	3	2.80E-06	2.65E-06	1.82E-06	2.25E-06
ZK353.6.1	729	931	948	996	2.80E-06	2.65E-06	1.82E-06	2.25E-06
ZK353.6.2	667	838	808	920	2.80E-06	2.65E-06	1.82E-06	2.25E-06
ZK353.7	354	383	505	558	5.13E-05	6.19E-05	4.34E-05	5.63E-05
ZK353.8.1	745	668	864	1134	5.00E-05	5.93E-05	3.94E-05	5.54E-05
ZK353.8.2	711	626	765	1075	3.38E-05	3.45E-05	3.14E-05	4.28E-05
ZK353.9	146	198	192	177	5.38E-05	4.56E-05	4.06E-05	6.58E-05
ZK354.1	1928	2562	3096	757	5.64E-05	4.70E-05	3.95E-05	6.85E-05

ZK354.10	1613	2250	2295	639	2.11E-05	2.71E-05	1.81E-05	2.06E-05
ZK354.11	2376	3303	3539	901	5.02E-04	6.30E-04	5.25E-04	1.58E-04
ZK354.12	1	0	0	1	4.99E-04	6.58E-04	4.62E-04	1.59E-04
ZK354.2a	57	91	20	20	6.20E-04	8.15E-04	6.01E-04	1.89E-04
ZK354.2b	41	82	18	19	2.80E-06	2.65E-06	1.82E-06	2.25E-06
ZK354.3	115	196	45	66	5.43E-06	8.20E-06	1.82E-06	2.25E-06
ZK354.5	2350	3207	4018	954	3.47E-06	6.56E-06	1.82E-06	2.25E-06
ZK354.6	53	53	15	21	1.37E-05	2.21E-05	3.50E-06	6.32E-06
ZK354.7	106	124	33	43	6.05E-04	7.80E-04	6.73E-04	1.97E-04
ZK354.8	36	48	32	23	5.38E-06	5.08E-06	1.82E-06	2.25E-06
ZK354.9	10	15	3	6	1.76E-05	1.94E-05	3.57E-06	5.74E-06
ZK355.1	4	6	13	4	2.80E-06	3.33E-06	1.82E-06	2.25E-06
ZK355.2b	61	80	88	115	2.80E-06	2.65E-06	1.82E-06	2.25E-06
ZK355.3	2	3	1	1	2.80E-06	2.65E-06	1.82E-06	2.25E-06
ZK355.4	0	5	22	16	5.94E-06	7.35E-06	5.58E-06	9.00E-06
ZK355.5	7	7	18	7	2.80E-06	2.65E-06	1.82E-06	2.25E-06
ZK355.6	6	9	8	6	2.80E-06	2.65E-06	1.82E-06	2.25E-06
ZK355.7	1	3	0	1	2.80E-06	2.65E-06	1.82E-06	2.25E-06
ZK370.2	32	53	28	33	2.80E-06	2.65E-06	1.82E-06	2.25E-06
ZK370.3a	744	959	866	1348	2.80E-06	2.65E-06	1.82E-06	2.25E-06
ZK370.3b	738	952	861	1336	2.80E-06	3.81E-06	1.82E-06	2.25E-06
ZK370.4a	1172	1435	1731	2502	2.68E-05	3.26E-05	2.03E-05	3.90E-05
ZK370.4b	1232	1478	1816	2596	2.63E-05	3.20E-05	2.00E-05	3.82E-05
ZK370.5.1	487	630	663	919	3.26E-05	3.77E-05	3.13E-05	5.59E-05
ZK370.6	11	9	10	14	3.19E-05	3.62E-05	3.06E-05	5.40E-05
ZK370.7	202	210	214	227	3.48E-05	4.26E-05	3.08E-05	5.28E-05
ZK370.8	530	622	769	863	2.80E-06	2.65E-06	1.82E-06	2.25E-06
ZK377.1	291	218	124	57	1.81E-05	1.78E-05	1.25E-05	1.63E-05
ZK377.2a	222	269	285	481	3.10E-05	3.44E-05	2.93E-05	4.05E-05
ZK377.2b	256	330	355	555	1.66E-05	1.17E-05	4.59E-06	2.61E-06
ZK380.1	2	6	6	4	6.52E-06	7.46E-06	5.45E-06	1.14E-05
ZK380.2	9	7	16	4	6.55E-06	7.99E-06	5.92E-06	1.14E-05
ZK380.3	18	14	18	6	2.80E-06	2.65E-06	1.82E-06	2.25E-06
ZK380.4	2	7	1	1	2.80E-06	2.65E-06	1.82E-06	2.25E-06
ZK380.5	6	9	4	6	2.80E-06	2.65E-06	1.82E-06	2.25E-06
ZK380.t2	20	11	9	12	2.80E-06	2.65E-06	1.82E-06	2.25E-06
ZK381.1	451	521	347	516	2.80E-06	2.65E-06	1.82E-06	2.25E-06
ZK381.2	6	17	13	9	3.07E-05	1.59E-05	8.98E-06	1.48E-05
ZK381.3.1	2	1	1	3	4.57E-05	4.99E-05	2.29E-05	4.20E-05
ZK381.3.2	2	1	1	3	2.80E-06	2.65E-06	1.82E-06	2.25E-06
ZK381.4a.1	2167	2047	2043	3242	2.80E-06	2.65E-06	1.82E-06	2.25E-06
ZK381.4a.2	1952	1869	1878	3087	2.80E-06	2.65E-06	1.82E-06	2.25E-06
ZK381.4b	2190	2064	2079	3278	1.10E-04	9.80E-05	6.74E-05	1.32E-04
ZK381.5a	25	47	27	51	9.18E-05	8.30E-05	5.74E-05	1.17E-04
ZK381.5b	31	46	33	48	9.89E-05	8.80E-05	6.11E-05	1.19E-04
ZK381.8	9	18	19	13	2.80E-06	2.99E-06	1.82E-06	2.74E-06
ZK384.1	0	2	0	1	2.80E-06	2.78E-06	1.82E-06	2.45E-06
ZK384.2	1	0	1	3	2.80E-06	2.65E-06	1.82E-06	2.25E-06
ZK384.3	6	8	6	0	2.80E-06	2.65E-06	1.82E-06	2.25E-06
ZK384.4	21	51	99	21	2.80E-06	2.65E-06	1.82E-06	2.25E-06
ZK39.2	0	2	3	0	2.80E-06	2.65E-06	1.82E-06	2.25E-06
ZK39.3	1	2	1	3	3.22E-06	7.41E-06	9.89E-06	2.59E-06
ZK39.4	2	1	1	0	2.80E-06	2.65E-06	1.82E-06	2.25E-06
ZK39.5	1	1	0	0	2.80E-06	2.65E-06	1.82E-06	2.25E-06
ZK39.6	49	30	83	38	2.80E-06	2.65E-06	1.82E-06	2.25E-06
ZK39.7	2	3	4	1	2.80E-06	2.65E-06	1.82E-06	2.25E-06
ZK39.8	1	1	1	4	9.49E-06	5.48E-06	1.04E-05	5.91E-06
ZK39.9	3	2	1	1	2.80E-06	2.65E-06	1.82E-06	2.25E-06
ZK39.t1	0	0	1	1	2.80E-06	2.65E-06	1.82E-06	2.25E-06

ZK402.1	26	22	37	27	2.80E-06	2.65E-06	1.82E-06	2.25E-06
ZK402.2	8	18	7	16	2.80E-06	2.65E-06	1.82E-06	2.25E-06
ZK402.3	13	44	21	28	3.70E-06	2.96E-06	3.43E-06	3.08E-06
ZK402.5	47	70	76	77	2.80E-06	5.77E-06	1.82E-06	4.36E-06
ZK418.1	3	7	3	2	3.67E-06	1.17E-05	3.86E-06	6.36E-06
ZK418.10	4	11	3	5	3.39E-06	4.79E-06	3.57E-06	4.48E-06
ZK418.11	8	5	7	3	2.80E-06	2.65E-06	1.82E-06	2.25E-06
ZK418.2a	44	78	25	15	2.80E-06	2.65E-06	1.82E-06	2.25E-06
ZK418.2b	40	76	24	14	2.80E-06	2.65E-06	1.82E-06	2.25E-06
ZK418.3	0	1	1	3	3.33E-06	5.55E-06	1.82E-06	2.25E-06
ZK418.4.1	217	255	474	332	3.11E-06	5.61E-06	1.82E-06	2.25E-06
ZK418.5	472	569	365	413	2.80E-06	2.65E-06	1.82E-06	2.25E-06
ZK418.6	43	63	39	42	2.56E-05	2.85E-05	3.64E-05	3.15E-05
ZK418.7	21	38	22	33	6.65E-05	7.57E-05	3.35E-05	4.67E-05
ZK418.8	151	136	217	156	2.80E-06	2.70E-06	1.82E-06	2.25E-06
ZK418.9a	828	835	835	1203	2.80E-06	3.36E-06	1.82E-06	2.47E-06
ZK418.9b.1	887	908	905	1267	1.76E-05	1.50E-05	1.65E-05	1.46E-05
ZK418.9b.2	847	871	865	1229	5.37E-05	5.12E-05	3.53E-05	6.27E-05
ZK418.9b.3	754	771	768	1144	5.19E-05	5.02E-05	3.44E-05	5.95E-05
ZK418.9b.4	784	796	793	1162	5.17E-05	5.03E-05	3.44E-05	6.03E-05
ZK430.1	665	835	884	1517	5.35E-05	5.16E-05	3.54E-05	6.51E-05
ZK430.2	128	168	161	172	5.34E-05	5.12E-05	3.51E-05	6.36E-05
ZK430.3	3	3	0	0	1.48E-05	1.75E-05	1.28E-05	2.71E-05
ZK430.5	22	34	15	16	1.38E-05	1.71E-05	1.13E-05	1.49E-05
ZK430.7.1	294	323	404	580	2.80E-06	2.65E-06	1.82E-06	2.25E-06
ZK430.7.2	250	277	343	555	2.80E-06	2.65E-06	1.82E-06	2.25E-06
ZK430.8	307	467	176	323	2.00E-05	2.07E-05	1.79E-05	3.17E-05
ZK455.1.1	1532	2038	2054	2506	2.09E-05	2.18E-05	1.86E-05	3.72E-05
ZK455.1.2	1459	1932	1992	2443	1.34E-05	1.93E-05	5.01E-06	1.13E-05
ZK455.2	22	29	25	22	5.58E-05	7.01E-05	4.87E-05	7.33E-05
ZK455.3	3	3	8	2	6.13E-05	7.67E-05	5.45E-05	8.24E-05
ZK455.4	85	92	77	97	2.80E-06	2.65E-06	1.82E-06	2.25E-06
ZK455.5	6	15	23	19	2.80E-06	2.65E-06	1.82E-06	2.25E-06
ZK455.6	15	33	15	22	4.96E-06	5.08E-06	2.92E-06	4.54E-06
ZK455.7	100	127	118	137	2.80E-06	2.65E-06	1.82E-06	2.25E-06
ZK455.8a	76	98	35	64	2.80E-06	2.88E-06	1.82E-06	2.25E-06
ZK455.8b	46	54	12	31	2.88E-06	3.47E-06	2.22E-06	3.17E-06
ZK470.1	20	24	23	18	3.58E-06	4.36E-06	1.82E-06	2.43E-06
ZK470.2a	115	213	93	138	2.80E-06	2.70E-06	1.82E-06	2.25E-06
ZK470.2b.1	91	163	81	130	2.80E-06	2.65E-06	1.82E-06	2.25E-06
ZK470.2b.2	84	159	85	114	1.06E-05	1.85E-05	5.56E-06	1.02E-05
ZK470.2b.3	94	157	76	118	9.38E-06	1.59E-05	5.45E-06	1.08E-05
ZK470.2c	288	434	292	374	6.22E-06	1.11E-05	4.10E-06	6.79E-06
ZK470.4	5	4	0	2	8.01E-06	1.26E-05	4.21E-06	8.07E-06
ZK470.5a	130	219	120	175	3.98E-05	5.67E-05	2.63E-05	4.15E-05
ZK470.5b.1	166	256	141	194	2.80E-06	2.65E-06	1.82E-06	2.25E-06
ZK470.5b.2	156	240	135	179	1.08E-05	1.72E-05	6.49E-06	1.17E-05
ZK470.6	17	15	5	8	1.14E-05	1.66E-05	6.30E-06	1.07E-05
ZK484.1.1	2574	3056	2877	4386	1.23E-05	1.79E-05	6.91E-06	1.13E-05
ZK484.1.2	2329	2678	2448	4073	2.80E-06	2.65E-06	1.82E-06	2.25E-06
ZK484.1.3	1506	1790	1630	2452	3.02E-04	3.39E-04	2.20E-04	4.14E-04
ZK484.1.4	995	1190	1228	1678	3.10E-04	3.37E-04	2.12E-04	4.35E-04
ZK484.1.5	1564	1867	1725	2646	2.71E-04	3.04E-04	1.91E-04	3.55E-04
ZK484.2b	442	603	306	350	2.15E-04	2.43E-04	1.72E-04	2.91E-04
ZK484.3.1	498	470	591	693	2.72E-04	3.07E-04	1.95E-04	3.70E-04
ZK484.4a	375	386	763	1197	2.44E-05	3.15E-05	1.10E-05	1.55E-05
ZK484.4b	380	394	774	1217	4.32E-05	3.85E-05	3.34E-05	4.83E-05
ZK484.5	173	468	130	42	1.52E-05	1.48E-05	2.02E-05	3.90E-05
ZK484.6	12	14	13	5	1.62E-05	1.59E-05	2.15E-05	4.18E-05

ZK484.7	19	34	22	11	4.89E-05	1.25E-04	2.39E-05	9.54E-06
ZK488.1	14	7	11	12	2.80E-06	2.65E-06	1.82E-06	2.25E-06
ZK488.10	1	1	1	0	2.80E-06	3.47E-06	1.82E-06	2.25E-06
ZK488.2	24	39	31	23	2.80E-06	2.65E-06	1.82E-06	2.25E-06
ZK488.3	3	1	2	0	2.80E-06	2.65E-06	1.82E-06	2.25E-06
ZK488.4	2	4	4	0	2.80E-06	3.20E-06	1.82E-06	2.25E-06
ZK488.5	9	9	13	7	2.80E-06	2.65E-06	1.82E-06	2.25E-06
ZK488.6	2	5	7	2	2.80E-06	2.65E-06	1.82E-06	2.25E-06
ZK488.7	2	2	6	2	2.80E-06	2.65E-06	1.82E-06	2.25E-06
ZK488.8	8	2	3	3	2.80E-06	2.65E-06	1.82E-06	2.25E-06
ZK488.9	6	2	9	3	2.80E-06	2.65E-06	1.82E-06	2.25E-06
ZK507.1	17	36	21	12	2.80E-06	2.65E-06	1.82E-06	2.25E-06
ZK507.3	21	33	21	9	2.80E-06	2.65E-06	1.82E-06	2.25E-06
ZK507.4	18	72	30	40	2.80E-06	3.84E-06	1.82E-06	2.25E-06
ZK507.6	605	600	905	1284	2.80E-06	3.09E-06	1.82E-06	2.25E-06
ZK512.1	19	9	13	7	3.25E-06	1.23E-05	3.52E-06	5.80E-06
ZK512.10	14	18	7	6	3.26E-05	3.05E-05	3.17E-05	5.55E-05
ZK512.11	8	22	30	9	2.80E-06	2.65E-06	1.82E-06	2.25E-06
ZK512.2	450	467	534	578	5.80E-06	7.06E-06	1.90E-06	2.25E-06
ZK512.3	62	75	53	111	2.80E-06	6.98E-06	6.56E-06	2.43E-06
ZK512.4	116	115	247	68	2.74E-05	2.69E-05	2.12E-05	2.83E-05
ZK512.6	16	26	28	25	2.80E-06	2.65E-06	1.82E-06	2.29E-06
ZK512.7	399	342	417	417	5.63E-05	5.27E-05	7.79E-05	2.65E-05
ZK512.8	32	84	31	15	2.80E-06	2.65E-06	1.82E-06	2.25E-06
ZK512.9	2	2	2	0	9.27E-05	7.51E-05	6.31E-05	7.78E-05
ZK520.2	68	60	55	67	6.41E-06	1.59E-05	4.05E-06	2.43E-06
ZK520.4a	1138	1039	1703	2161	2.80E-06	2.65E-06	1.82E-06	2.25E-06
ZK520.4b.1	896	823	1281	1703	7.42E-06	6.19E-06	3.90E-06	5.87E-06
ZK520.4b.2	896	817	1279	1703	4.19E-05	3.61E-05	4.08E-05	6.39E-05
ZK520.4c	899	828	1283	1704	4.27E-05	3.71E-05	3.98E-05	6.52E-05
ZK520.4d	1036	959	1578	2015	4.28E-05	3.68E-05	3.97E-05	6.53E-05
ZK520.5.1	77	106	56	32	4.24E-05	3.69E-05	3.94E-05	6.45E-05
ZK520.5.2	56	83	34	20	4.23E-05	3.70E-05	4.19E-05	6.60E-05
ZK524.2a	153	229	114	194	1.32E-05	1.71E-05	6.23E-06	4.39E-06
ZK524.2c	131	200	88	180	1.09E-05	1.52E-05	4.28E-06	3.10E-06
ZK524.2d	162	245	121	204	3.14E-06	4.44E-06	1.82E-06	3.22E-06
ZK524.2e	175	260	118	203	3.30E-06	4.79E-06	1.82E-06	3.67E-06
ZK524.3a	311	296	442	454	2.80E-06	4.02E-06	1.82E-06	2.83E-06
ZK524.3b	311	296	442	454	3.36E-06	4.73E-06	1.82E-06	3.15E-06
ZK524.4	574	532	921	1248	1.35E-05	1.21E-05	1.25E-05	1.58E-05
ZK525.2	194	255	307	309	1.27E-05	1.15E-05	1.18E-05	1.49E-05
ZK54.1b	31	45	36	59	2.11E-05	1.84E-05	2.20E-05	3.68E-05
[truncated: 40,502 more chars]
